# Increased circulating resistin levels in early-onset breast cancer patients of normal body mass index correlate with lymph node negative involvement and longer disease free survival: a multi-center POSH cohort serum proteomics study

**DOI:** 10.1186/s13058-018-0938-6

**Published:** 2018-03-22

**Authors:** Bashar Zeidan, Antigoni Manousopoulou, Diana J. Garay-Baquero, Cory H. White, Samantha E. T. Larkin, Kathleen N. Potter, Theodoros I. Roumeliotis, Evangelia K. Papachristou, Ellen Copson, Ramsey I. Cutress, Stephen A. Beers, Diana Eccles, Paul A. Townsend, Spiros D. Garbis

**Affiliations:** 1 Cancer Sciences Unit, Faculty of Medicine, University of Southampton, Southampton, UK; 2 Institute for Life Sciences, University of Southampton, Southampton, UK; 3 Clinical and Experimental Sciences Unit, Faculty of Medicine, University of Southampton, Southampton, UK; 4 Division of Cancer Sciences, University of Manchester, Manchester Cancer Research Centre, Manchester Academic Health Science, Manchester Biomedical Research Centre, Health Innovation Manchester, Manchester, UK; 5 Present address: The Institute for Cancer Research, London, UK; 6 Present address: Cancer Research Cambridge Institute, University of Cambridge, Cambridge, UK; 7 Present address: Merck Exploratory Science Center, Cambridge, MA USA; 8 Faculty of Biology, Medicine and Health, University of Manchester, Manchester, UK

**Keywords:** Quantitative serum proteomics, Resistin, Insulin resistance, Glycolysis/gluconeogenesis, lymph-node involvement, Early-onset breast cancer

## Abstract

**Background:**

Early-onset breast cancer (EOBC) affects about one in 300 women aged 40 years or younger and is associated with worse outcomes than later onset breast cancer. This study explored novel serum proteins as surrogate markers of prognosis in patients with EOBC.

**Methods:**

Serum samples from EOBC patients (stages 1–3) were analysed using agnostic high-precision quantitative proteomics. Patients received anthracycline-based chemotherapy. The discovery cohort (*n* = 399) either had more than 5-year disease-free survival (DFS) (good outcome group, *n* = 203) or DFS of less than 2 years (poor outcome group, *n* = 196). Expressed proteins were assessed for differential expression between the two groups. Bioinformatics pathway and network analysis in combination with literature research were used to determine clinically relevant proteins. ELISA analysis against an independent sample set from the Prospective study of Outcomes in Sporadic versus Hereditary breast cancer (POSH) cohort (*n* = 181) was used to validate expression levels of the selected target. Linear and generalized linear modelling was applied to determine the effect of target markers, body mass index (BMI), lymph node involvement (LN), oestrogen receptor (ER), progesterone receptor and human epidermal growth factor receptor 2 status on patients’ outcome.

**Results:**

A total of 5346 unique proteins were analysed (peptide FDR *p ≤ 0.05*). Of these, 812 were differentially expressed in the good vs poor outcome groups and showed significant enrichment for the insulin signalling (*p = 0.01*) and the glycolysis/gluconeogenesis (*p = 0.01*) pathways. These proteins further correlated with interaction networks involving glucose and fatty acid metabolism. A consistent nodal protein to these metabolic networks was resistin (upregulated in the good outcome group, *p = 0.009).* ELISA validation demonstrated resistin to be upregulated in the good outcome group (*p = 0.04*), irrespective of BMI and ER status. LN involvement was the only covariate with a significant association with resistin measurements (*p = 0.004*). An ancillary *in-silico* observation was the induction of the inflammatory response, leucocyte infiltration, lymphocyte migration and recruitment of phagocytes (*p < 0.0001*, *z-score* > 2). Survival analysis showed that resistin overexpression was associated with improved DFS.

**Conclusions:**

Higher circulating resistin correlated with node-negative patients and longer DFS independent of BMI and ER status in women with EOBC. Overexpression of serum resistin in EOBC may be a surrogate indicator of improved prognosis.

**Electronic supplementary material:**

The online version of this article (10.1186/s13058-018-0938-6) contains supplementary material, which is available to authorized users.

## Background

Approximately one in 300 women aged 40 years or younger is diagnosed with breast cancer in the UK and young age at diagnosis is associated with worse clinical outcomes and greater likelihood of genetic susceptibility (http://www.cancerresearchuk.org/health-professional/cancer-statistics/statistics-by-cancer-type/breast-cancer) [1, 2]. Current prognostic biomarkers are based on tumour characteristics, tumour grade and stage, and receptor status. Host factors that may influence prognosis are not currently included in commonly used models [3]. Identifying novel host markers associated with EOBC prognosis may improve our understanding and management of this subgroup of patients.

As a quantitative proteomics approach, the use of chemical labelling with isobaric stable isotope reagents, such as isobaric tags for relative and absolute quantitation (iTRAQ) and tandem mass tags (TMT), has been applied in combination with liquid chromatography–mass spectrometry (LC-MS) techniques for the discovery of candidate cancer biomarkers in serum or plasma [4, 5]. Such methodological approaches provide the distinct advantage of simultaneously measuring protein expression under the same instrumental analysis conditions, thereby reducing experimental bias and improving relative quantitative accuracy and precision [6]. An iTRAQ LC-MS approach that also used a peptide-based affinity enrichment pre-treatment step was applied to plasma samples derived from stage I–III breast cancer patients relative to healthy volunteers [7]. Another iTRAQ LC-MS study that used affinity depletion of the high-abundant proteins was applied to serum samples derived from post-menopausal breast cancer patients relative to healthy controls [8]. In this study, however, we utilized quantitative LC-MS proteomic methods that do not depend on prior affinity enrichment or depletion of plasma/serum which may compromise their analysis for clinically relevant protein markers [5, 9]. In this capacity, the entire serum protein content was subjected to quantitative proteomic analysis. Using serum from a cohort study of early-onset breast cancer cases, we explored the potential for quantitative discovery proteomics to reveal novel markers of poor outcome in young women with EOBC [2].

## Methods

### Patient inclusion criteria

**Fig. 1:**
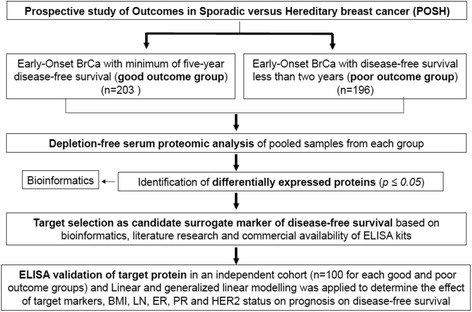
Experimental design for the high-precision LC-MS proteomic discovery analysis, data reduction and subsequent targeted validation pipeline. BrCA breast cancer, ELISA enzyme-linked immunosorbent assay, BMI body mass index, LN lymph node, ER oestrogen receptor, PR progesterone receptor, HER2 human epidermal growth factor receptor 2

The present study included patients with early-stage (T1–T3) invasive breast carcinoma, diagnosed between January 2000 and December 2007 from the Prospective study of Outcomes in Sporadic versus Hereditary breast cancer (POSH) cohort, a UK-wide multi-centre prospective observational study of EOBC patients, aged 40 years or younger and treated with standard therapies according to local protocols (Additional file 1: Section 1) [1, 2, 10]. Patients included in this study received anthracycline-based chemotherapy. For the discovery phase, patients were selected based on the period of disease-free follow up to provide a discovery cohort enriched for poor and for good outcomes. The good outcome group comprised 203 randomly selected patients with disease-free survival (DFS) of at least 5 years following treatment. The poor outcome group included 196 patients who experienced local recurrence, new primary contralateral and/or distant metastasis and/or death within 2 years of initial diagnosis. The full patient clinico-pathological characteristics are presented in Table 1. The study design is summarized in Fig. 1.

**Table 1:** Clinical characteristics of the discovery cohort

Clinical characteristics	Good outcome group	Poor outcome group	*p* value
*n*	203	196	
Age (years)			
Median	37	36	0.89
Range	25–40	18–41	
Relapse (years)			
Median	9.3	1.3	< 0.0001
Range	5.0–10.2	0.4–2.0	
BMI (kg/m^2^)			
Mean	25.2	26.3	0.13*
SD	5.1	5.4	
Histology			
Invasive ductal carcinoma	203	190	
Invasive lobular carcinoma	0	0	
Unknown	0	6	
Grade 1	10	6	
Grade 2	75	47	
Grade 3	114	137	
Unknown	4	6	
Lymph node status			
Negative	104	61	< 0.0001
Positive	95	127	
Undetermined	4	8	
ER status			
Positive	138	108	< 0.0001
Negative	43	88	
Unknown	22	0	
PR status			
Positive	87	75	0.43
Negative	79	86	
Unknown	42	35	
HER2 receptor status			
Positive	53	82	0.77
Negative	59	92	
Unknown	91	22	
Triple-negative tumours	32	35	
Resection margin			
R0 resection	142	141	
R1 resection	24	22	
Unknown	37	33	
Chemotherapy			
FEC	69	71	
ECMF	28	31	
FEC + docetaxel	22	14	
AC	16	16	
EC + paclitaxel	15	12	
EC + paclitaxel + gemcitabine	8	8	
EC	8	6	
Null	22	8	
Other	15	30	

*A* adriamycin, *BI* body mass index, *C* cyclophosphamide, *E* epirubicin, *ER* oestrogen receptor, *F* 5-fluorouracil, *HER2* human epidermal growth factor receptor 2, *M* methotrexate, *PR* progesterone receptor, *SD* standard deviation

*Unpaired T-test between groups

### Serum procurement and processing

Peripheral blood samples were drawn from patients in the POSH cohort at their local cancer unit and processed and stored in accordance with the POSH SOPs (Additional file 1: Sections 1A and 1B) [1, 2]. For the good outcome group, using the randomization function of Microsoft Excel (2011), individual 20-μl aliquots from 102 and 101 specimens respectively were pooled together to create two biological replicate pools (good outcome groups 1 and 2). Identical procedures were undertaken for the poor outcome group, with 98 samples being pooled in each biological replicate (poor outcome groups 1 and 2). An aliquot of 100 μl from each sample pool was mixed with 400 μl 6 M guanidine in 9:1 water:methanol and subjected to high-performance size-exclusion chromatography (HP SEC) and dialysis exchange for the serum protein pre-fractionation and purification steps [9, 11–14].

### Quantitative LC-MS proteomics

For each sample pool, 100 μg protein content derived from the respective SEC segments was prepared. Briefly, the segmented protein fractions were subjected to dialysis purification and lyophilized to dryness. The purified proteins were re-solubilized in 200 μl dissolution buffer (0.5 M triethylammonium bicarbonate, 0.05% SDS), quantified and subjected to proteolysis with trypsin using a standardized protocol. The tryptic peptide mixtures generated from each of the four segments were then isobaric stable isotope labelled with the iTRAQ reagents (per manufacturer specifications) for each of the good and poor outcome groups (and their biological replicates), and were pooled. The resulting iTRAQ peptides were initially fractionated with alkaline C_8_ reverse phase (RP) liquid chromatography [13, 15]. Each peptide fraction was further separated with on-line nano-capillary C_18_ reverse phase liquid chromatography under acidic conditions, subjected to nano-spray ionization and measured with ultra-high-resolution mass spectrometry using the hybrid ion-trap/FT-Orbitrap Elite platform [12–14, 16]. Reporter ion ratios derived from unique peptides were used for the relative quantitation of each respective protein. Raw reporter ion intensity values were median normalized and log_2_ transformed. Proteins identified with a minimum of two unique peptides and a one-sample *t*-test *p ≤ 0.05* were considered as differentially expressed between the good and poor outcome groups and were further subjected to bioinformatics analysis [12, 15, 17, 18]*.* A detailed description of the quantitative proteomics approach used can be found in Additional file 2: Section 2: Supplementary Methods - Serum Proteomics.

### Bioinformatics analysis

Hierarchical clustering of the differentiated proteins was performed using Cluster 3.0 (C Clustering Library 1.52) and Java Treeview (version 1.1.6r4) such that distances were calculated using the Euclidean-based metric and then clustered using the complete linkage method. MetaCore (Clarivate Analytics, Boston, MA, USA), Ingenuity Pathway Analysis including its Diseases & Functions module (Qiagen, Silicon Valley, CA, USA) and DAVID Bioinformatics Resources 6.8 (National Institute of Allergy and Infectious Diseases (NIAID), NIH) (https://david.ncifcrf.gov/) were applied to differentially expressed proteins analysed with at least two unique peptides to identify significantly over-represented networks and gene ontology (GO) terms. Fisher exact and FDR-corrected *p ≤ 0.05* was considered significant.

### Single-blinded ELISA measurements in the validation cohort

To replicate the accuracy of relative quantitation of a target protein, ELISA was performed against individual sera derived from an independent validation sample set within the POSH cohort and sharing analogous inclusion criteria with the discovery sample set. As high BMI levels may constitute a confounding factor for resistin expression, normal BMI status was used as an additional inclusion criterion. For the ELISA validation a single-blinded design was used, wherein assignment of patient IDs to a good or poor outcome group was unavailable to the analyst performing the measurements and uncovered by an independent clinician after the measurements were completed. In particular, the validation cohort was comprised of 200 samples (*n* = 100 good outcome patients and *n* = 100 poor outcome patients) randomly selected from the POSH cohort using the randomization function of Microsoft Excel (2011). Of the randomly selected patients, sufficient serum volume was only available for 90 and 91 samples from the good and poor outcome groups respectively (Table 2). The size of the validation cohort was based on the logistic models requiring a minimum of 10 events per predictor variable [19–21], which in our study included ER, PR, HER2, LN and BMI status. The ELISA measurements were performed using a resistin sandwich ELISA kit (USCN Life Sciences Inc., Wuhan, P.R. China) according to the manufacturer’s protocols. Absorbance was measured with the GloMax^®^ Discover, Promega plate reader (Thermo Fisher Scientific). Data were analysed in Prism (version 7.0a). Statistical analyses of the ELISA measurements were based on Welch’s two-sample *t* test for unequal variances to assess significant differences between groups at *p ≤ 0.05*. This test was deemed appropriate as there is a balance of samples in groups and each group is well above the suggested level of 15 per group which allows control of the type I error rate even in non-normal distributions [22–24].

**Table 2:** Clinical characteristics of the validation cohort

Clinical characteristics	Good outcome group	Poor outcome group	*p* value
*n*	90	91	
Age (years)			
Median	37	35	0.35
Range	26–40	18–40	
Relapse (years)			
Median	9.2	1.0	< 0.0001
Range	5.0–11.2	0.3–2.0	
BMI (kg/m^2^)*			
Mean	23.3	23.2	0.84
SD	2.1	2.3	
Histology			
Invasive ductal carcinoma	83	83	
Invasive lobular carcinoma	6	7	
Unknown	1	1	
Grade 1	2	1	
Grade 2	30	16	
Grade 3	57	73	
Unknown	1	1	
Lymph node status			
Negative	45	26	0.001
Positive	45	65	
Undetermined	0	0	
ER status			
Positive	59	41	0.003
Negative	31	50	
Unknown	0	0	
PR status			
Positive	42	24	0.001
Negative	32	52	
Unknown	16	15	
HER2 receptor status			
Positive	24	35	0.47
Negative	49	49	
Unknown	17	7	
Triple-negative tumours	17	22	
Resection margin			
R0 resection	67	67	
R1 resection	7	12	
Unknown	16	12	
Chemotherapy			
FEC	27	28	
ECMF	22	18	
FEC + docetaxel	5	14	
AC	5	5	
EC + paclitaxel	5	4	
EC + paclitaxel + gemcitabine	2	4	
EC	5	1	
Null	10	2	
Other	9	15	

*A* adriamycin, *BI* body mass index, *C* cyclophosphamide, *E* epirubicin, *ER* oestrogen receptor, *F* 5-fluorouracil, *HER2* human epidermal growth factor receptor 2, *M* methotrexate, *PR* progesterone receptor, *SD* standard deviation

*p = 0.13 between groups (unpaired *t* test)

### Linear and generalized linear modelling

Modelling patient outcome in the validation cohort as a function of resistin and other variables was performed using generalized linear modelling and the function *glm* within the R statistical computing environment (https://www.R-project.org/) and using the logit link function appropriate for the binomial family. For linear modelling of resistin as a function of BMI, lymph node (LN) involvement (N0 = negative; N1–N3 = positive), ER (Allred score: 0–2 = negative; 3–8 = positive), PR (0–2 = negative; 3–8 = positive) and HER2 status (0, 1+ = negative; 2+ = equivocal; 3+ = positive), the linear modelling function *lm* was utilized (https://www.R-project.org/). The reference for each categorical variable was as follows: LN = negative; ER = negative, PR = negative, HER2 = negative. All coefficients were tested with the function *coeftest* available within R (https://www.R-project.org/).

### ROC and AUC analysis

A prediction vector was generated with the predict function in R and then merged with a vector of true outcome results. To determine a threshold by which a prediction would be considered positive (good outcome result), a receiver operating characteristic (ROC) curve was generated by selecting 101 potential threshold values between 0 and 1 with a 0.01 step size and calculating the true positive and false positive rates for each threshold value. The cost function for these threshold values was the sum of the false positives and false negatives given the threshold setting. These results indicated that a threshold of 0.5 was reasonable, above which a prediction was determined to be positive (good outcome) and below which a prediction was determined to be negative (poor outcome). The area under the curve (AUC) measure was calculated using the *auc* function in the pROC package available within R.

### In-silico survival analysis in breast cancer tissue samples

A meta-analysis-based biomarker assessment of resistin in breast cancer tissue samples was performed using the online software tool Kaplan–Meier Plotter (http://kmplot.com). Kaplan–Meier Plotter assesses the effects of 54,675 genes on patient DFS using 5143 breast cancer samples with a mean follow-up of 200 months [25].

## Results

### Quantitative proteomic analysis and in-silico bioinformatics interpretation

Quantitative proteomics yielded a total of 5346 unique proteins (peptide FDR-corrected *p* ≤ 0.05) from all four HP-SEC-derived segments (Additional file 3: Section 3). Of these, 812 proteins were differentially expressed between the good and poor outcome groups (*p ≤ 0.05, ≥ 2 unique peptides*) (Additional file 4: Section 4) and were subjected to further bioinformatics analysis. The mass spectrometry proteomics data have been deposited with the ProteomeXchange Consortium via the PRIDE partner repository with the dataset identifier PXD008443.

### Pathway and network analysis

**Fig. 2:**
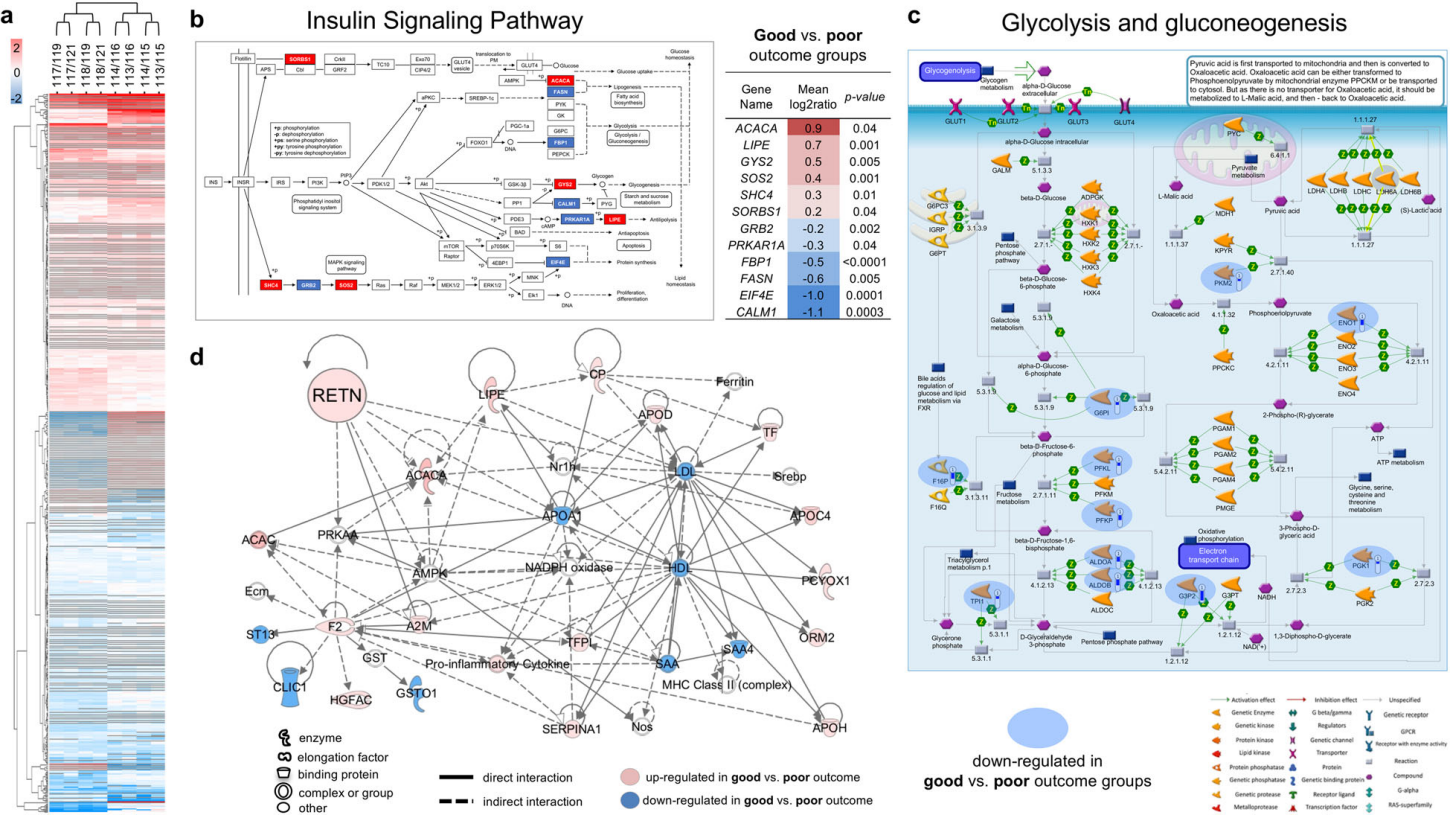
**a** Hierarchical clustering analysis of all differentially expressed proteins (DEPs) (812 proteins at *p ≤ 0.05* with ≥ 2 unique peptides). **b** Insulin signalling pathway significantly over-represented in DEPs between good and poor outcome groups (Fisher exact *p* = 0.015) using KEGG Pathway analysis with DAVID. Tabulation of gene names of the observed differentially expressed proteins constituent to the pathway presented. **c** MetaCore showed that glycolysis/gluconeogenesis was a significantly enriched process in the DEPs between good and poor outcome groups (FDR corrected *p = 0.011*). **d** Network analysis of differentially expressed proteins using Ingenuity Pathway Analysis showed participation of resistin in the small molecule biochemistry network (score = 23; focus molecules = 20)

Significant enrichment was observed for the insulin pathway in the differentially expressed proteins between the good and poor outcome groups (*p = 0.015,* KEGG Pathway analysis using DAVID) (Fig. 2a). MetaCore pathway analysis identified glycolysis/gluconeogenesis as a significantly enriched process in the differentially expressed proteins between the good and poor outcome groups (*p < 0.011*, FDR corrected) (Fig. 2b). Ingenuity Pathway Analysis identified small molecule biochemistry, in particular glucose and fatty acid metabolism, as a significantly over-represented network (score = 23, focus molecules = 20) in the differentially expressed proteins between the good and poor outcome groups. Resistin was a key molecular participant in this network (Fig. 2c), and was chosen for targeted validation based on its previously reported role in breast cancer biology and insulin resistance risk [26–36].

### Resistin ELISA validation measurements

**Fig. 3:**
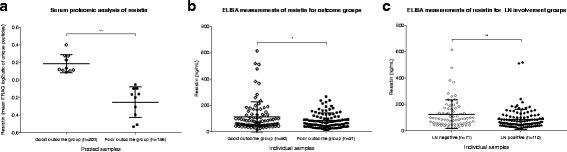
**a** Serum proteomic analysis of resistin showed higher circulating levels in good compared to poor outcome groups. Each point represents log_2_ ratio of reporter ion intensity of each clinical group (good or poor outcome respectively) over the mean of all four reporter ion intensities from both clinical groups produced from a given unique peptide (good vs poor outcome iTRAQ mean log_2_ ratio = 0.2, SD = 0.13 between biological replicates, *p* = 0.009). **b** Resistin ELISA measurements across individual samples from the validation cohort in good outcome group (*n* = 90, mean (SD) = 114.2 (114.5) ng/ml) compared to poor outcome group (*n* = 91, mean (SD) = 86.8 (57.7) ng/ml) (*p = 0.04*). **c** Resistin expression higher in LN-negative vs LN-positive patients, irrespective of outcome group (LN-negative group, *n* = 71, mean (SD) = 124.8 (107.5) ng/ml; LN-positive group, *n* = 110, mean (SD) = 84.7 (75.6) ng/ml; *p = 0.0037,* Welch’s two-sample *t* test)*.* iTRAQ isobaric tags for relative and absolute quantitation, *ELISA* enzyme-linked immunosorbent assay, *LN* lymph node. * denotes *p*<0.05, and ** denotes *p*<0.01.

Resistin was measured to be upregulated in the good outcome group from the proteomic discovery stage using pooled serum samples (*p = 0.009*) (Figure 3a). The upregulation of serum resistin in the good outcome group relative to the poor outcome group was confirmed with ELISA against the validation cohort (good outcome group, *n* = 90, mean (SD) = 114.2 (114.5) ng/ml; poor outcome group, *n* = 91, mean (SD) = 86.8 (57.7) ng/ml; *p = 0.04*) (Fig. 3b) (Additional file 5: Section 5).

### ROC/AUC and KM survival analysis

**Fig. 4:**
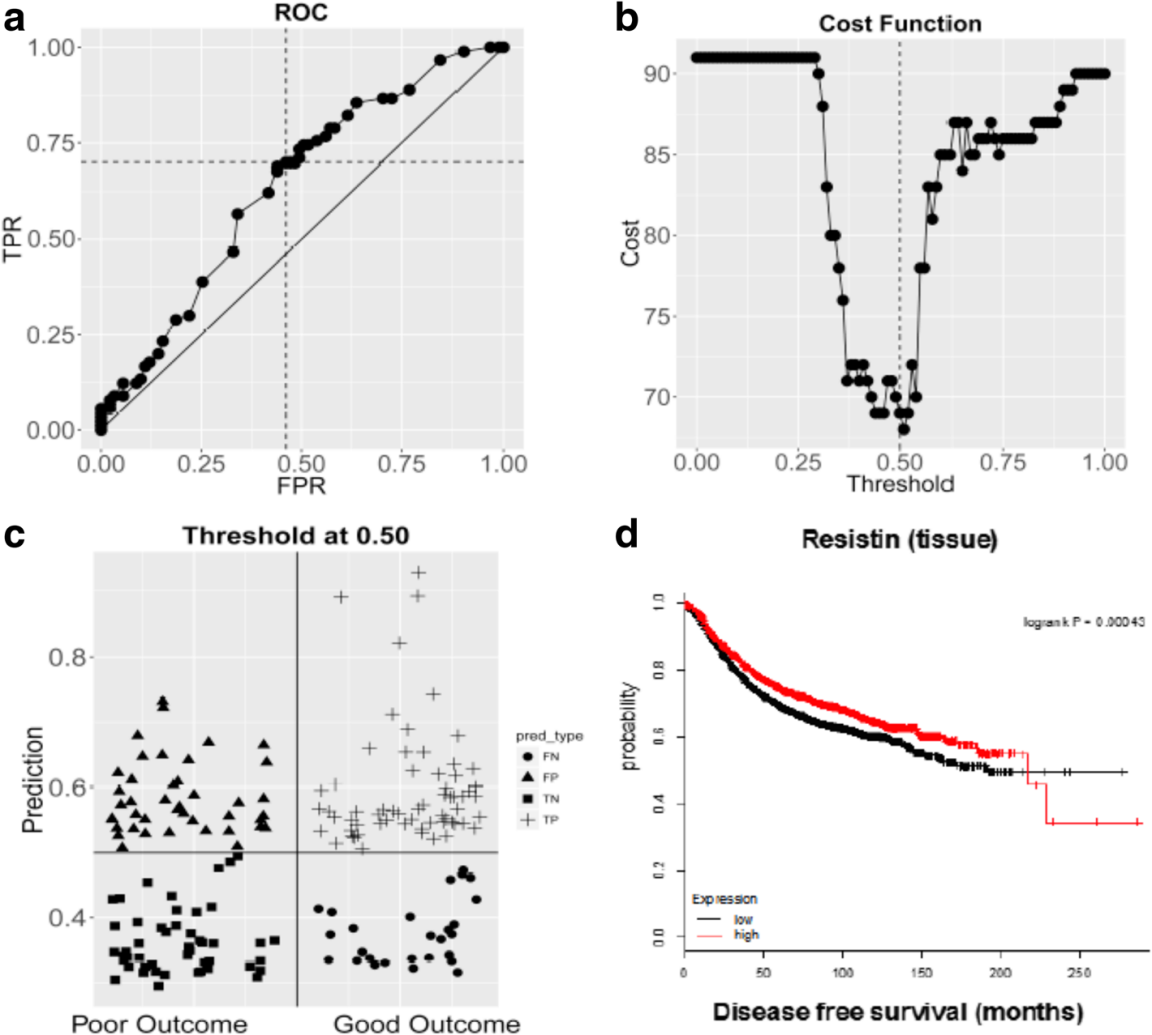
**a** Receiver operating characteristic (ROC) curve of the true positive rate (TPR) versus the false positive rate (FPR) with AUC = 0.6352. **b** Cost function with equivalent penalties for false negatives and false positives. **c** Distribution plot of 30 false negatives (FN), 33 false positives (FP), 58 true negatives (TN) and 60 true positives (TP). **d** *In-silico* Kaplan–Meier survival analysis of resistin expression at the tissue level

To determine the predictive power of resistin for outcome, a receiver-operating characteristic curve (ROC) was generated (Fig. 4a) along with a cost function with equivalent penalties for false negatives and false positives (Fig. 4b, c). The AUC measure of the ROC curve indicated a moderate level of success for utilizing resistin measures to predict outcome. Using the measure of true positives, true negatives, false positives and false negatives, serum resistin provided an accuracy of 0.652, a sensitivity of 0.667 and a specificity of 0.637.

Finally, using publically available data, *in-silico* Kaplan–Meier survival analysis showed a longer disease-free survival in patients with higher resistin levels at the tissue level for up to 200 months (Fig. 4d).

### Prediction of biological function directionality (induction or inhibition)

**Fig. 5:**
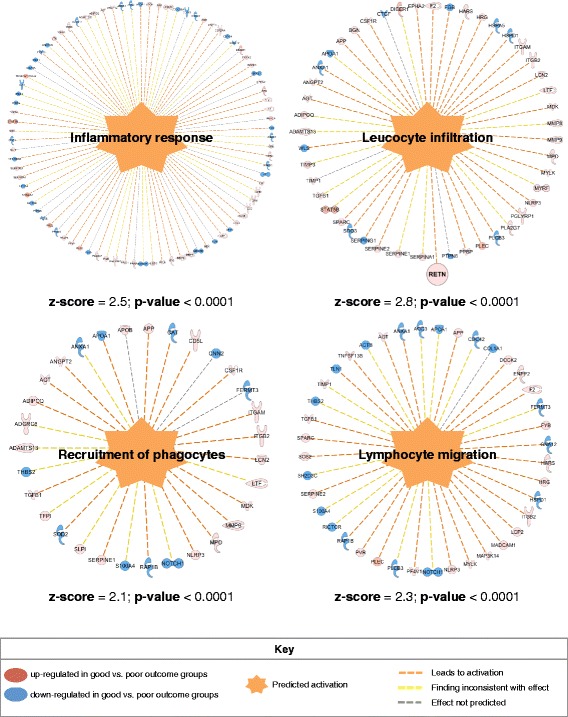
Significant induction (*p < 0.0001*) of inflammatory response, leucocyte infiltration, lymphocyte migration and recruitment of phagocytes in the good vs poor outcome groups based on downstream differentiated proteins. *z*-score > 2 signifies a positive induction effect

The Diseases & Functions module of Ingenuity Pathway Analysis demonstrated that inflammatory response, leucocyte infiltration, lymphocyte migration and recruitment of phagocytes were significantly induced biological processes based on the downstream differentially expressed proteins of the good vs poor outcome groups. Resistin was specifically found to participate in the activation of leucocyte infiltration (Fig. 5).

### Linear and generalized linear modelling

Both linear and generalized linear modelling techniques were utilized to determine which covariates would relate to DFS and resistin expression (Additional file 6: Section 6). LN involvement was found to correlate with worse patient outcome (*p = 0.004*) and demonstrated a significant difference in mean value of resistin between LN groups. More specifically, LN-negative patients had significantly higher resistin levels compared to those with LN involvement (LN-negative group, *n* = 71, mean (SD) = 124.8 (107.5) ng/ml; LN-positive group, *n* = 110, mean (SD) = 84.7 (75.6) ng/ml; *p = 0.0037,* Welch’s two-sample *t* test) (Fig. 3c, Additional file 6: Section 6).

## Discussion

Improvements made in breast cancer survival have been associated with the wider use of neo/adjuvant chemotherapy such as anthracycline/taxane-based treatment [37]. Routine immunohistochemical analysis is used for both prognosis and predictive markers of response to hormonal therapy and trastuzumab (ER/PR and HER2 respectively). Young age [38, 39] and obesity [2] at breast cancer diagnosis have been reported to be independent prognostic markers of adverse disease outcome. The aim of this study was to find serum proteomic markers of additional prognostic relevance to EOBC outcomes.

This study implemented a high-precision quantitative serum proteomics discovery analysis followed by targeted serum ELISA-based validation in an independent sample set of non-obese EOBC patient samples (Fig. 1). The applied proteomics method achieved the highest degree of proteome coverage in breast cancer serum to date (5346 unique proteins with peptide FDR *p ≤ 0.05*). The methodological feature that led to this comprehensive proteome result was its ability to analyse non-depleted serum that also contains exosome-enriched and other extracellular vessicle- derived proteins in addition to directly secreted proteins, as reported previously [9, 12, 14]. Such an in-depth analysis was deemed essential for the unbiased interrogation of expected systemic effects and their affiliated biological pathways and networks induced by treatment.

Hierarchical clustering analysis of all 812 differentially expressed proteins (DEPs) is presented in heatmap format in Fig. 2a. The DEPs were then subjected to canonical pathway analysis, which achieved significant enrichment for the insulin signalling (*p = 0.015*) (Fig. 2b) and glycolysis/gluconeogenesis (*p = 0.011)* pathways (Fig. 2c)*.* Interestingly, the majority of observed proteins that encoded for both of these pathways were of exosomal origin, as listed in the manually curated ExoCarta Web-based compendium (http://www.exocarta.org) [40–42]. Of relevance, all enzymes mapping to the glycolysis/gluconeogenesis pathway were upregulated in the poor outcome group, suggesting that poor-prognosis patients catabolize glucose more actively compared to patients with longer survival (Fig. 2c). One noteworthy enzyme found to be upregulated in the poor outcome group was the pyruvate kinase M2 isoform (PKM2) known to play an important role in tumorigenesis. As observed in different types of cancers, including breast cancer, pyruvate kinase expression shifts to the PKM2 isoform in order to utilize glucose more efficiently to generate biomass under anaerobic conditions [43]. The functional involvement of the insulin signalling and the glycolysis/gluconeogenesis pathways were further verified with Ingenuity Pathway Analysis that showed significant enrichment for glucose and fatty acid metabolism (Fig. 2d) and included resistin, a secreted protein, as one of its key nodal components. We focused on serum resistin given its association with the insulin signalling and glycolysis/gluconeogenesis pathways as a candidate marker of EOBC prognosis.

In agreement with the discovery cohort (Fig. 3a), resistin was found to be upregulated in the good outcome group in the normal weight validation cohort (Fig. 3b). To address accurate protein inference, ELISA was used as the measurement approach for the validation cohort because it allowed the analysis of the intact form of resistin, whereas bottom-up proteomics, as used in this study, allows the assessment of protein expression at the derived peptide level resulting from the trypsin proteolysis step.

In this work, both linear and generalized linear regression analysis confirmed that ER, PR and HER2 exhibited a significant degree of interdependence (*p < 0.05*) (Additional file 6: Section 6). A receiver operating characteristic (ROC) curve (Fig. 4a) and associated cost curve (Fig. 4b) were used to assess the value of resistin in outcome prediction between the two groups in this study, The AUC measure of the ROC curve indicated a moderate level of success for utilizing resistin measures to predict outcome. Using the measure of true positives, true negatives, false positives and false negatives (Fig. 4c), serum resistin provided an accuracy of 0.652, a sensitivity of 0.667 and a specificity of 0.637. We explored resistin expression at the tissue level using an *in-silico* meta-analysis micro-array database, the Kaplan–Meier Plotter software tool (http://kmplot.com/analysis/). Consistent with the serum observations in our current study, this analysis showed that high tissue levels of resistin were associated with longer disease-free survival (*p* < 0.001) (Fig. 4d).

Resistin is a pro-inflammatory molecular that has been implicated in obesity-mediated type 2 diabetes. Obesity is a host factor that adversely influences breast cancer prognosis [2, 42]. There is evidence that insulin resistance may develop after breast cancer adjuvant therapy [41], and a recent prospective study reported that increased resistin levels coincided with the concurrent increase in serum insulin and insulin resistance following treatment (surgery followed by chemotherapy and radiotherapy) among stage II–III breast cancer patients in an adiposity-independent way [35]. It is therefore possible that derangement of glucose metabolism through insulin resistance may be a result of late toxic effects of chemotherapy possibly due to impaired pancreatic beta-cell function. However, in our present study all patients received chemotherapy and so any differential effect cannot be due to the chemotherapy alone. Recent reports strongly suggest that resistin production in humans is largely from macrophages rather than adipose tissue alone (also known to contain macrophages) [30, 33, 44]. Insulin pathophysiology has been associated with inflammatory markers independent of BMI in subjects at risk of type 2 diabetes [45]. Additionally, in transgenic mice, production of human resistin from macrophages was associated with increased inflammation and contributed to the acquisition of insulin resistance [33]. Our current proteomic findings add to the evidence suggesting that resistin is a potential surrogate marker of disturbed insulin pathophysiology and inflammation that could provide an explanation for the observed association between higher resistin level and improved DFS.

As an ancillary finding, resistin levels were significantly higher in LN-positive vs LN-negative patients, irrespective of outcome group (*p = 0.0037*) (Fig. 3c). A regression model further examined this trend where the LN status demonstrated a significant association with resistin measurements. Resistin overexpression was found to correlate with node-negative status (*p* = 0.0428). This trend, in combination with the results from the association testing, provide further evidence that resistin and nodal status could be linked (Additional file 6: Section 6). During inflammation, macrophages can be both a major source of resistin and themselves able to respond to resistin in an autocrine loop, leading to an increase in pro-inflammatory ‘M1-like’ macrophages and a reduction in anti-inflammatory ‘M2-like’ macrophages [33, 46]. Given that the lymph node status existed at presentation and all patients received chemotherapy, we considered whether the overexpression of resistin *per se* may have influenced the tumour micro-environment to exert a suppressive effect on tumour cell motility or extravasation. The association of anti-inflammatory ‘M2-like’ monocytes and macrophages with metastases in preclinical models [47] provides a possible mechanism whereby increased resistin levels could lead to a lower potential for metastatic spread by promoting a pre-existing pro-inflammatory tumour microenvironment. To further explore this hypothesis, the *post-priori* examination of the downstream differentially expressed proteins between the good vs poor outcome groups using the Diseases & Functions module of Ingenuity Pathway Analysis identified the inflammatory response, leucocyte infiltration (also implicating resistin), lymphocyte migration and recruitment of phagocytes to be significantly induced biological processes (*p < 0.0001, z-score > 2*) (Fig. 5). Overall, improved prognosis associated with increased resistin levels may indicate an immunomodulatory role of this protein during early breast tumour development limiting the ability of the tumour primary cells to spread to distant sites. Further examining the mechanistic link between circulating resistin levels and patient LN status was beyond the scope of the present study; future studies will be required to explore this hypothesis. This is a relatively small study, and a larger follow-up study is warranted, ideally with pre-treatment serum samples to determine whether the observed specific correlation with metastasis to axillary lymph nodes holds true at all ages. A potential technical limitation was the sample pooling strategy used in the discovery phase, which did not permit the assessment of anticipated inter-individual heterogeneity in protein expression levels. However, extensive sample pooling is more likely to find larger, more consistent, protein differences that are therefore more likely to replicate. In addition, the accuracy of relative protein quantitation for resistin was validated with ELISA measurements against individual serum specimens from a separate validation cohort, and from the *in-silico* analysis of an independent cohort at the tissue level.

## Conclusions

A high-precision serum proteomics-based pipeline identified increased serum resistin to positively correlate with disease-free survival independent of BMI in women with EOBC. High resistin levels were associated with less axillary lymph node involvement at presentation and better survival. We hypothesize that individuals with early breast cancer who have relatively higher resistin levels may provide an environment from which tumours are less likely to metastasize. Further prospective studies are needed to confirm these findings and elucidate the mechanistic role of resistin in EOBC patients.

## Additional files

Additional file 1: Sections 1A and 1B presenting POSH Serum Procurement SOPs. (ZIP 244 kb)

Additional file 2: Section 2 presenting the serum proteomics method. (PDF 82 kb)

Additional file 3: Section 3 presenting the total serum proteome. (PDF 1522 kb)

Additional file 4: Section 4 presenting differentially expressed proteins in good vs poor outcome groups. (PDF 489 kb)

Additional file 5: Section 5 presenting ELISA measurements for resistin. (PDF 356 kb)

Additional file 6: Section 6 presenting linear and generalized linear modelling of resistin, ER, PR, LN and HER-2 clinical parameters. (DOCX 108 kb)

## Abbreviations

AUC: Area under the curve
EOBC: Early-onset breast cancer
HPLC: High-performance liquid chromatography
iTRAQ: Isobaric tags for relative and absolute quantitation
LC-MS: Liquid chromatography–mass spectrometry
POSH: Prospective study of Outcomes in Sporadic versus Hereditary breast cancer
ROC: Receiver operating characteristic
