# Supplementary material for: Increased circulating resistin levels in early-onset breast cancer patients of normal body mass index correlate with lymph node negative involvement and longer disease free survival: a multi-center POSH cohort serum proteomics study
Source: Breast Cancer Res. 2018 Mar 22;20:19. doi: 10.1186/s13058-018-0938-6 (PMC5863447; doi:10.1186/s13058-018-0938-6)
Supplement: Supplementary file 2 — Section 2 presenting the serum proteomics method. (PDF 82 kb) [file 13058_2018_938_MOESM2_ESM.pdf]

Circulating resistin in early-onset breast cancer patients with normal body mass index correlates with disease-free survival and lymph node involvement: A serum proteomics study from the multi-center POSH cohort

Zeidan *et al. Breast Cancer Research, 2018*

SERUM PROTEOMICS

Size exclusion chromatography

The serum specimens were thawed and vortexed for 2 minutes. Individual 20 µL aliquots from the 203 specimens of the good outcome group were pooled together. Identical procedures were undertaken for the 196 specimens comprising the poor outcome group. An aliquot of 100 µL from each sample pool was mixed with 400 µL 6 M Guanidine in 9:1 Water: methanol and subjected to High Performance Size-Exclusion Chromatography (HP SEC) on the Dionex HPLC P680 pump equipped with the PDA-100 photodiode array detector. SEC was applied with two serially connected Shodex SEC KW-804 (8.0×300 mm) columns under isocratic elution of 6M Guanidine Hydrochloride at 200 µL/min. Four SEC protein fractions (assigned as Segments 1-4) were generated.

Dialysis Purification

The protein fractions were purified using 3 KDa MWCO Slide-A-Lyzer dialysis cassettes (Thermo Fisher, Waltham, MA, US) dialysed into ultrapure water, renewed three times after overnight equilibrations. After dialysis exchange, the samples were transferred into 15 mL tubes and dried with a speedvac concentrator. The residual protein content was dissolved into 200 µL dissolution buffer (0.5M triethylammonium bicarbonate, 0.05% SDS) with probe sonication. Protein concentration was

measured with the Bradford assay according to manufacturer's instructions (Bio-Rad Protein Assay).

Trypsin Proteolysis and iTRAQ Labeling

For each one of the two biological states 2×100 μg protein content derived from the respective SEC segments were diluted up to 20 μL with dissolution buffer and then reduced by the addition of 2 μL 50 mM tris-2-carboxymethyl phosphine (TCEP) for 60 minutes at 60 °C. The cysteine residues were blocked by the addition of 1 μL 200 mM methyl methanethiosulfonate (MMTS) for 10 minutes at room temperature. The samples were diluted with 14 μL ultra pure water prior to the addition of 6 μL proteomics grade trypsin (500 ng/μL; Roche Diagnostics, Basel, Switzerland) solution and were left for overnight digestion in dark. For the iTRAQ 8-plex peptide labeling each reagent was diluted with 50 μL isopropanol and then mixed with each one of the tryptic digests for 2 hours. The iTRAQ labelling scheme was as follows: Segments 1 and 3: 117 and 118 = good outcome groups, 119 and 121 = poor outcome groups; Segments 2 and 4: 113 and 114 = good outcome groups, 115 and 116 = poor outcome groups. The four different samples of each segment separately were then combined and the mixture was lyophilized to dryness with a speedvac concentrator.

High-pH Reverse Phase (RP) Peptide Fractionation

High-PH RP C_8 fractionation of the iTRAQ labelled peptides was performed on the Ultimate (LC Packings, USA) HPLC system using the Waters, XBridge C8 column (150 × 3 mm, 3.5 μm particle). The composition of mobile phase (A) was 2% acetonitrile, 0.1% ammonium hydroxide, whereas the composition of mobile phase (B) was 100% acetonitrile, 0.1% ammonium hydroxide. The dried-up peptide pellet was dissolved in 100 μL of 95% mobile phase (A) and 5% mobile phase (B) with extensive vortex mixing. The sample was centrifuged at 13K rpm for 10 min, and the

supernatant was injected in a 100 μL sample loop. The gradient used was the following: for 10 min isocratic 5% (B), for 60 min gradient up to 30% (B), for 20 min gradient up to 85% (B), for 10 min isocratic 85% (B), for 10 min down at 2% (B) at a flow rate 0.2 mL/min. Signal response was monitored at 215 and the column temperature was set at 30 °C. Fraction collection was performed on a peak-dependent manner. The peptide fractions were dried-up using a speedvac concentrator for 4−5 h at 30°C and stored at −20 °C until the LC−MS analysis.

LC-FT-Orbitrap MS Analysis

The LC−MS experiments were performed on the Dionex Ultimate 3000 UHPLC system coupled with the high resolution nano-ESI LTQ-Velos Pro Orbitrap-Elite mass spectrometer (Thermo Scientific). Individual peptide fractions were reconstituted in 30 μL of loading solution (2% acetonitrile, 0.1% formic acid), and a 10 μL volume was loaded on the Acclaim PepMap 100, 100 μm × 2 cm C18, 5 μm particle trapping column with the ulPickUp Injection mode using the loading pump at 5 μL/min flow rate for 5 min. Two separate analyses for HCD and CID fragmentation for each one of the collected fractions were performed. For the analytical separation the Acclaim PepMap RSLC, 75 μm × 25 cm, nanoViper, C18, 2 μm particle column retrofitted to a PicoTip emitter (FS360-20-10-D-20-C7) was used for multistep gradient elution. Mobile phase (A) was composed of 2% acetonitrile, 0.1% formic acid, and mobile phase (B) was composed of 100% acetonitrile, 0.1% formic acid. The gradient elution method at flow rate 300 nL/min was as follows: for 10 min isocratic gradient 5% (B), for 90 min gradient up to 40% (B), for 10 min gradient up to 85% (B), for 10 min isocratic 85% (B), for 5 min down to 2% (B), for 8 min isocratic equilibration 3% (B) at 35 °C. Separated peptides were transferred to the gaseous phase with positive ion electrospray ionization applying a voltage of 2.5 kV. Top 10

multiply charged precursor isotopic clusters with m/z value larger than 350 or smaller than 1900 and intensity threshold 500 counts were selected with FT mass resolution of 120000 and isolated for HCD fragmentation within a mass window of 1.2 Da. Tandem mass spectra were acquired with FT resolution of 15 000 with in m/z range of 100−1900. For the CID experiments, top 20 precursors were selected with FT mass resolution of 240 000 within a mass window of 2 Da. Normalized collision energy was set to 35, and already targeted precursors were dynamically excluded for further isolation and activation for 30 s with 5 ppm mass tolerance for both types of analysis.

Database searching

The unprocessed raw files were submitted to Proteome Discoverer 1.4 for target decoy searching with SequestHT searching against the UniProtKB homo sapiens database comprised of 20,159 entries (release date January 2015), allowing for up to two missed cleavages, a tolerance of 10 ppm, a minimum peptide length of six, and a maximum of two variable (one equal) modifications of; oxidation (M), deamidation (N, Q), or phosphorylation (S,T, Y), iTRAQ 8plex (Y). Methythio (C) and iTRAQ (K and N-terminus) were set as fixed modifications. Fragment ion mass tolerances of 0.02 Da for the FT-acquired HCD spectra and 0.5 Da for the IT-acquired CID spectra. FDR was estimated with the Percolator and set to ≤0.05 and validation was based on q value at <0.05. Reporter ions were extracted with a tolerance of 20 ppm and were rejected if any channels were absent. Percent co-isolation excluding peptides from quantitation was set at 50. Reporter ion ratios from unique peptides only were taken into consideration for the quantitation of the respective protein. Raw iTRAQ intensity values of unique peptides were median-normalized and log2transformed. The multi-consensus protocol was used to process

the iTRAQ-labelled proteotypic peptides observed for all 4 SEC derived segments. In adherence to the Paris Publication Guidelines for the analysis and documentation of peptide and protein identifications (http://www.mcponline.org/site/misc/ParisReport_Final.xhtml), only proteins identified with at least two unique peptides were considered in the bioinformatics analysis. A one-sample T-Test was performed to identify proteins that were differentially expressed between good and poor outcome groups. Significance was set at $p \leq 0.05$. All mass spectrometry proteomics data have been deposited to the ProteomeXchange Consortium via the PRIDE partner repository with the dataset identifier PXD008443.
