# Supplementary material for: Increased circulating resistin levels in early-onset breast cancer patients of normal body mass index correlate with lymph node negative involvement and longer disease free survival: a multi-center POSH cohort serum proteomics study
Source: Breast Cancer Res. 2018 Mar 22;20:19. doi: 10.1186/s13058-018-0938-6 (PMC5863447; doi:10.1186/s13058-018-0938-6)
Supplement: Supplementary file 3 — Section 3 presenting the total serum proteome. (PDF 1522 kb) [file 13058_2018_938_MOESM3_ESM.pdf]

Supplementary Section 3. Analysed proteins

Accession	Description	ΣCoverage	Σ# Proteins	Σ# Unique Peptides	Σ# Peptides	Σ# PSMs	117/119	118/119	117/121	118/121	114/116	114/115	113/116	113/115	# AAs	MW [kDa]	calc. pI
Q8NDY3	[Protein ADP-ribosylarginine] hydrolase-like protein 1 OS=Homo sapiens GN=ADPRHL1 PE=2 SV=1 - [ARHL1_HUMAN]	2.26	1	1	1	1	1.20	1.29	1.30	1.42					354	40.1	5.85
Q96QU6	1-aminocyclopropane-1-carboxylate synthase-like protein 1 OS=Homo sapiens GN=ACCS PE=2 SV=1 - [1A1L1_HUMAN]	2.99	1	1	1	1	1.13	1.25	1.05	1.17					501	57.3	6.43
Q9Y2I7	1-phosphatidylinositol 3-phosphate 5-kinase OS=Homo sapiens GN=PIKFYVE PE=1 SV=3 - [FYV1_HUMAN]	1.62	9	1	3	4					0.83	0.84	0.66	0.67	2098	237.0	6.70
Q01970	1-phosphatidylinositol 4,5-bisphosphate phosphodiesterase beta-3 OS=Homo sapiens GN=PLCB3 PE=1 SV=2 - [PLCB3_HUMAN]	3.16	2	2	3	5	0.37	0.34	0.43	0.40	0.90	0.94	0.87	0.92	1234	138.7	5.90
Q15147	1-phosphatidylinositol 4,5-bisphosphate phosphodiesterase beta-4 OS=Homo sapiens GN=PLCB4 PE=1 SV=3 - [PLCB4_HUMAN]	2.21	1	2	3	3	0.92	0.92	0.94	0.94	0.79	0.92	0.85	1.00	1175	134.4	6.90
P51178	1-phosphatidylinositol 4,5-bisphosphate phosphodiesterase delta-1 OS=Homo sapiens GN=PLCD1 PE=1 SV=2 - [PLCD1_HUMAN]	8.20	1	3	3	4	1.17	1.16	1.16	1.15					756	85.6	6.70
Q8N3E9	1-phosphatidylinositol 4,5-bisphosphate phosphodiesterase delta-3 OS=Homo sapiens GN=PLCD3 PE=1 SV=3 - [PLCD3_HUMAN]	3.68	1	1	1	1					1.28	1.28	0.97	0.97	789	89.2	6.98
Q07973	1,25-dihydroxyvitamin D(3) 24-hydroxylase, mitochondrial OS=Homo sapiens GN=CYP24A1 PE=1 SV=2 - [CP24A_HUMAN]	5.84	1	1	1	1					1.66	1.62	1.08	1.06	514	58.8	8.75
P31946	14-3-3 protein beta/alpha OS=Homo sapiens GN=YWHAB PE=1 SV=3 - [1433B_HUMAN]	12.60	6	1	3	13	0.59	0.08	0.69	0.09					246	28.1	4.83
P63104	14-3-3 protein zeta/delta OS=Homo sapiens GN=YWHAZ PE=1 SV=1 - [1433Z_HUMAN]	35.92	6	5	8	33	0.57	0.58	0.62	0.63	0.83	0.79	0.86	0.82	245	27.7	4.79
Q9BPX1	17-beta-hydroxysteroid dehydrogenase 14 OS=Homo sapiens GN=HSD17B14 PE=1 SV=1 - [DHB14_HUMAN]	9.26	1	1	1	4	0.91	0.85	0.89	0.84	0.86	0.88	0.94	0.96	270	28.3	6.06
Q9C0C2	182 kDa tankyrase-1-binding protein OS=Homo sapiens GN=TNKS1BP1 PE=1 SV=4 - [TB182_HUMAN]	4.22	1	3	3	4	0.91	0.61	0.85	0.60	0.56	0.57	0.57	0.58	1729	181.7	4.86
O75600	2-amino-3-ketobutyrate coenzyme A ligase, mitochondrial OS=Homo sapiens GN=GCAT PE=2 SV=1 - [KBL_HUMAN]	6.21	1	1	1	6	1.24	1.25	1.33	1.36	1.56	1.64	1.40	1.49	419	45.3	8.05
Q96SZ5	2-aminoethanethiol dioxygenase OS=Homo sapiens GN=ADO PE=1 SV=2 - [AEDO_HUMAN]	7.41	1	1	1	1					0.98	1.18	0.96	1.16	270	29.7	6.04
Q9UJ83	2-hydroxyacyl-CoA lyase 1 OS=Homo sapiens GN=HACL1 PE=1 SV=2 - [HACL1_HUMAN]	7.09	1	2	2	8	1.01	1.15	1.09	1.24	0.96	0.93	1.06	1.03	578	63.7	7.36
Q8N543	2-oxoglutarate and iron-dependent oxygenase domain-containing protein 1 OS=Homo sapiens GN=OGFOD1 PE=1 SV=1 - [OGFD1_HUMAN]	4.06	1	1	1	1	1.34	1.63	1.19	1.44					542	63.2	5.11
P21953	2-oxoisovalerate dehydrogenase subunit beta, mitochondrial OS=Homo sapiens GN=BCKDHB PE=1 SV=2 - [ODBB_HUMAN]	4.34	1	1	1	1							0.67	0.74	392	43.1	6.29
Q9NYL5	24-hydroxycholesterol 7-alpha-hydroxylase OS=Homo sapiens GN=CYP39A1 PE=2 SV=2 - [CP39A_HUMAN]	5.33	2	1	2	2					0.80	1.02	0.87	1.11	469	54.1	8.65
P62333	26S protease regulatory subunit 10B OS=Homo sapiens GN=PSMC6 PE=1 SV=1 - [PRS10_HUMAN]	5.40	1	1	1	2	0.92	1.16	0.83	1.05					389	44.1	7.49
Q99460	26S proteasome non-ATPase regulatory subunit 1 OS=Homo sapiens GN=PSMD1 PE=1 SV=2 - [PSMD1_HUMAN]	1.26	1	1	1	1	1.17	1.16	1.25	1.25					953	105.8	5.39
O00231	26S proteasome non-ATPase regulatory subunit 11 OS=Homo sapiens GN=PSMD11 PE=1 SV=3 - [PSD11_HUMAN]	2.61	1	1	1	1					0.95	0.81	1.12	0.95	422	47.4	6.48
Q9UNM6	26S proteasome non-ATPase regulatory subunit 13 OS=Homo sapiens GN=PSMD13 PE=1 SV=2 - [PSD13_HUMAN]	3.72	1	1	1	1	0.89	0.96	0.79	0.84					376	42.9	5.81
Q13200	26S proteasome non-ATPase regulatory subunit 2 OS=Homo sapiens GN=PSMD2 PE=1 SV=3 - [PSMD2_HUMAN]	2.75	1	1	1	1	1.21	1.09	1.19	1.09					908	100.1	5.20
P55036	26S proteasome non-ATPase regulatory subunit 4 OS=Homo sapiens GN=PSMD4 PE=1 SV=1 - [PSMD4_HUMAN]	6.63	1	1	1	1	0.87	0.85	1.00	0.99					377	40.7	4.79
Q15008	26S proteasome non-ATPase regulatory subunit 6 OS=Homo sapiens GN=PSMD6 PE=1 SV=1 - [PSMD6_HUMAN]	2.31	1	1	1	2	0.72	0.78	0.71	0.77					389	45.5	5.62

P48556	26S proteasome non-ATPase regulatory subunit 8 OS=Homo sapiens GN=PSMD8 PE=1 SV=2 - [PSMD8_HUMAN]	10.29	1	1	1	1	0.97	0.67	1.37	0.96					350	39.6	9.70
Q9Y2Q9	28S ribosomal protein S28, mitochondrial OS=Homo sapiens GN=MRPS28 PE=1 SV=1 - [RT28_HUMAN]	6.95	1	1	1	1	1.20	1.26	1.17	1.24					187	20.8	9.10
P82675	28S ribosomal protein S5, mitochondrial OS=Homo sapiens GN=MRPS5 PE=1 SV=2 - [RT05_HUMAN]	2.56	1	1	1	1					0.73	1.00	0.67	0.92	430	48.0	9.92
P04035	3-hydroxy-3-methylglutaryl-coenzyme A reductase OS=Homo sapiens GN=HMGCR PE=1 SV=1 - [HMDH_HUMAN]	1.35	1	1	1	1	0.41	0.51	0.44	0.55					888	97.4	6.74
Q6NVY1	3-hydroxyisobutyryl-CoA hydrolase, mitochondrial OS=Homo sapiens GN=HIBCH PE=1 SV=2 - [HIBCH_HUMAN]	3.11	1	1	1	1	1.40	1.31	1.40	1.33					386	43.5	8.19
P42765	3-ketoacyl-CoA thiolase, mitochondrial OS=Homo sapiens GN=ACAA2 PE=1 SV=2 - [THIM_HUMAN]	1.76	1	1	1	1					1.16	1.35	1.01	1.17	397	41.9	8.09
P09110	3-ketoacyl-CoA thiolase, peroxisomal OS=Homo sapiens GN=ACAA1 PE=1 SV=2 - [THIK_HUMAN]	2.59	1	1	1	1					0.89	0.89	1.08	1.07	424	44.3	8.44
Q7Z7H8	39S ribosomal protein L10, mitochondrial OS=Homo sapiens GN=MRPL10 PE=1 SV=3 - [RM10_HUMAN]	8.05	1	1	1	1	1.14	1.04	1.23	1.12					261	29.3	9.58
Q9H2W6	39S ribosomal protein L46, mitochondrial OS=Homo sapiens GN=MRPL46 PE=1 SV=1 - [RM46_HUMAN]	4.30	1	1	1	1					1.01	0.44	0.64	0.28	279	31.7	7.05
P49189	4-trimethylaminobutyraldehyde dehydrogenase OS=Homo sapiens GN=ALDH9A1 PE=1 SV=3 - [AL9A1_HUMAN]	2.43	1	1	1	2	1.00	0.95	0.93	0.89					494	53.8	5.87
P63220	40S ribosomal protein S21 OS=Homo sapiens GN=RPS21 PE=1 SV=1 - [RS21_HUMAN]	21.69	1	1	1	1					1.19	1.12	1.06	1.00	83	9.1	8.50
P61247	40S ribosomal protein S3a OS=Homo sapiens GN=RPS3A PE=1 SV=2 - [RS3A_HUMAN]	4.55	1	1	1	3					0.27	0.34	0.86	0.69	264	29.9	9.73
P62081	40S ribosomal protein S7 OS=Homo sapiens GN=RPS7 PE=1 SV=1 - [RS7_HUMAN]	7.73	1	1	1	1	0.96	0.69	0.72	0.52					194	22.1	10.10
Q13702	43 kDa receptor-associated protein of the synapse OS=Homo sapiens GN=RAPSN PE=1 SV=4 - [RAPSN_HUMAN]	10.44	1	2	2	4	0.20	0.15	0.36	0.27	0.73	1.19	0.74	1.21	412	46.3	8.12
P08195	4F2 cell-surface antigen heavy chain OS=Homo sapiens GN=SLC3A2 PE=1 SV=3 - [4F2_HUMAN]	15.56	1	7	8	12	1.14	1.04	1.23	1.10	0.95	1.04	0.88	0.97	630	68.0	5.01
P13196	5-aminolevulinate synthase, nonspecific, mitochondrial OS=Homo sapiens GN=ALAS1 PE=1 SV=2 - [HEM1_HUMAN]	2.34	1	1	1	2					1.59	1.87	1.58	1.86	640	70.5	8.46
P49914	5-formyltetrahydrofolate cyclo-ligase OS=Homo sapiens GN=MTHFS PE=1 SV=2 - [MTHFS_HUMAN]	9.36	1	1	1	1	0.98	0.95	1.09	1.07					203	23.2	7.88
P41595	5-hydroxytryptamine receptor 2B OS=Homo sapiens GN=HTR2B PE=1 SV=1 - [5HT2B_HUMAN]	1.66	1	1	1	1					0.95	0.63	1.06	0.70	481	54.3	9.10
P28335	5-hydroxytryptamine receptor 2C OS=Homo sapiens GN=HTR2C PE=1 SV=1 - [5HT2C_HUMAN]	6.11	1	1	1	1	0.86	0.14	0.53	0.09					458	51.8	8.91
P46098	5-hydroxytryptamine receptor 3A OS=Homo sapiens GN=HTR3A PE=1 SV=1 - [5HT3A_HUMAN]	2.93	1	1	1	1					1.58	1.38	1.41	1.22	478	55.2	7.39
O14841	5-oxoprolinase OS=Homo sapiens GN=OPLAH PE=1 SV=3 - [OPLA_HUMAN]	3.03	2	2	3	6					1.50	1.79	0.91	1.08	1288	137.4	6.58
P17643	5,6-dihydroxyindole-2-carboxylic acid oxidase OS=Homo sapiens GN=TYRP1 PE=1 SV=2 - [TYRP1_HUMAN]	1.68	1	1	1	2					0.81	0.35	0.74	0.32	537	60.7	5.99
Q9H816	5' exonuclease Apollo OS=Homo sapiens GN=DCLRE1B PE=1 SV=1 - [DCR1B_HUMAN]	3.57	1	1	1	1	2.95	1.89	1.22	0.79					532	60.0	8.41
Q13131	5'-AMP-activated protein kinase catalytic subunit alpha-1 OS=Homo sapiens GN=PRKAA1 PE=1 SV=4 - [AAPK1_HUMAN]	1.79	1	1	1	5	1.09	1.17	0.92	1.00	0.79	0.93	0.79	0.93	559	64.0	8.12
Q9UGJ0	5'-AMP-activated protein kinase subunit gamma-2 OS=Homo sapiens GN=PRKAG2 PE=1 SV=1 - [AAKG2_HUMAN]	2.81	1	1	1	1	0.62	0.37	0.45	0.26					569	63.0	9.35
P21589	5'-nucleotidase OS=Homo sapiens GN=NT5E PE=1 SV=1 - [5NTD_HUMAN]	4.53	1	1	1	1					1.47				574	63.3	7.03
O43422	52 kDa repressor of the inhibitor of the protein kinase OS=Homo sapiens GN=PRKRIR PE=2 SV=2 - [P52K_HUMAN]	4.47	1	2	2	2	0.71	0.60	0.89	0.73					761	87.6	5.86

Q15646	59 kDa 2'-5'-oligoadenylate synthase-like protein OS=Homo sapiens GN=OASL PE=1 SV=2 - [OASL_HUMAN]	3.70	1	1	1	2					1.35	1.42	1.18	1.23	514	59.2	7.87
Q16877	6-phosphofructo-2-kinase/fructose-2,6-bisphosphatase 4 OS=Homo sapiens GN=PFKFB4 PE=2 SV=6 - [F264_HUMAN]	1.71	1	1	1	1	0.92	1.22	0.77	1.04					469	54.0	6.62
Q01813	6-phosphofructokinase type C OS=Homo sapiens GN=PFKP PE=1 SV=2 - [K6PP_HUMAN]	4.85	1	3	3	5	0.71	0.80	0.75	0.77					784	85.5	7.55
P17858	6-phosphofructokinase, liver type OS=Homo sapiens GN=PFKL PE=1 SV=6 - [K6PL_HUMAN]	8.59	2	5	5	10	0.81	0.88	0.78	0.88					780	85.0	7.50
P52209	6-phosphogluconate dehydrogenase, decarboxylating OS=Homo sapiens GN=PGD PE=1 SV=3 - [6PGD_HUMAN]	22.57	1	10	10	22	0.91	0.94	0.99	1.05	1.02	1.19	0.86	1.00	483	53.1	7.23
O95336	6-phosphogluconolactonase OS=Homo sapiens GN=PGLS PE=1 SV=2 - [6PGL_HUMAN]	8.14	1	1	1	2					0.88	0.52	0.82	0.48	258	27.5	6.05
P10809	60 kDa heat shock protein, mitochondrial OS=Homo sapiens GN=HSPD1 PE=1 SV=2 - [CH60_HUMAN]	5.93	1	2	2	4					0.60	0.68	0.66	0.75	573	61.0	5.87
P05387	60S acidic ribosomal protein P2 OS=Homo sapiens GN=RPLP2 PE=1 SV=1 - [RLA2_HUMAN]	11.30	1	1	1	3					1.43	1.17	1.33	1.08	115	11.7	4.54
Q96D46	60S ribosomal export protein NMD3 OS=Homo sapiens GN=NMD3 PE=1 SV=1 - [NMD3_HUMAN]	8.35	1	2	2	2	0.90	1.04	0.87	1.02					503	57.6	7.14
Q96L21	60S ribosomal protein L10-like OS=Homo sapiens GN=RPL10L PE=1 SV=3 - [RL10L_HUMAN]	13.08	1	2	2	2	0.99	0.87	1.09	0.96					214	24.5	10.01
P62910	60S ribosomal protein L32 OS=Homo sapiens GN=RPL32 PE=1 SV=2 - [RL32_HUMAN]	5.19	1	1	1	3					0.76	0.49	0.92	0.59	135	15.8	11.33
P49207	60S ribosomal protein L34 OS=Homo sapiens GN=RPL34 PE=1 SV=3 - [RL34_HUMAN]	5.98	1	1	1	1	0.74	0.73	0.94	0.92					117	13.3	11.47
P42766	60S ribosomal protein L35 OS=Homo sapiens GN=RPL35 PE=1 SV=2 - [RL35_HUMAN]	4.88	2	1	1	1	1.66	1.64	1.75	1.75					123	14.5	11.05
P83881	60S ribosomal protein L36a OS=Homo sapiens GN=RPL36A PE=1 SV=2 - [RL36A_HUMAN]	5.66	2	1	1	2					0.82	1.20	0.37	0.54	106	12.4	10.58
P46777	60S ribosomal protein L5 OS=Homo sapiens GN=RPL5 PE=1 SV=3 - [RL5_HUMAN]	2.02	1	1	1	2	1.07	1.34	0.99	1.26					297	34.3	9.72
P62424	60S ribosomal protein L7a OS=Homo sapiens GN=RPL7A PE=1 SV=2 - [RL7A_HUMAN]	3.76	1	1	1	1					1.13	1.23	1.99	2.16	266	30.0	10.61
P62917	60S ribosomal protein L8 OS=Homo sapiens GN=RPL8 PE=1 SV=2 - [RL8_HUMAN]	10.51	1	2	2	4	1.23	1.24	1.31	1.33	1.04	0.73	0.98	0.69	257	28.0	11.03
Q9UNU6	7-alpha-hydroxycholest-4-en-3-one 12-alpha-hydroxylase OS=Homo sapiens GN=CYP8B1 PE=2 SV=2 - [CP8B1_HUMAN]	5.59	1	2	2	2	0.08	0.08	0.06	0.06	0.85	0.79	0.96	0.89	501	58.0	8.66
P08253	72 kDa type IV collagenase OS=Homo sapiens GN=MMP2 PE=1 SV=2 - [MMP2_HUMAN]	9.85	1	5	5	12	1.08	0.87	1.06	0.84	0.87	1.33	0.78	1.18	660	73.8	5.47
P11021	78 kDa glucose-regulated protein OS=Homo sapiens GN=HSPA5 PE=1 SV=2 - [GRP78_HUMAN]	36.85	2	19	21	83	0.95	0.87	0.96	0.92	0.75	0.72	0.78	0.77	654	72.3	5.16
O60318	80 kDa MCM3-associated protein OS=Homo sapiens GN=MCM3AP PE=1 SV=2 - [MCM3A_HUMAN]	0.86	1	1	1	1	1.26	1.15	1.17	1.07					1980	218.3	6.39
O60733	85 kDa calcium-independent phospholipase A2 OS=Homo sapiens GN=PLA2G6 PE=1 SV=2 - [PA2G6_HUMAN]	0.99	1	1	1	1					0.24	0.63	0.26	0.68	806	89.8	7.27
Q9H324	A disintegrin and metalloproteinase with thrombospondin motifs 10 OS=Homo sapiens GN=ADAMTS10 PE=1 SV=2 - [ATS10_HUMAN]	1.45	1	1	1	1	1.92	1.21	1.91	1.21					1103	120.8	7.94
P58397	A disintegrin and metalloproteinase with thrombospondin motifs 12 OS=Homo sapiens GN=ADAMTS12 PE=1 SV=2 - [ATS12_HUMAN]	1.57	1	1	1	2	0.99	0.98	0.96	0.95					1594	177.6	7.87
Q76LX8	A disintegrin and metalloproteinase with thrombospondin motifs 13 OS=Homo sapiens GN=ADAMTS13 PE=1 SV=1 - [ATS13_HUMAN]	19.41	1	21	21	59	1.20	1.24	1.13	1.16	1.22	1.01	1.18	1.03	1427	153.5	7.17
Q8WXS8	A disintegrin and metalloproteinase with thrombospondin motifs 14 OS=Homo sapiens GN=ADAMTS14 PE=2 SV=2 - [ATS14_HUMAN]	0.49	1	1	1	1	0.90	0.45	1.25	0.63					1223	133.8	7.18
Q8TE56	A disintegrin and metalloproteinase with thrombospondin motifs 17 OS=Homo sapiens GN=ADAMTS17 PE=2 SV=2 - [ATS17_HUMAN]	4.38	1	2	2	6	0.70	0.84	0.67	0.80	0.90	0.84	0.81	0.78	1095	121.0	8.06

O95450	A disintegrin and metalloproteinase with thrombospondin motifs 2 OS=Homo sapiens GN=ADAMTS2 PE=2 SV=2 - [ATS2_HUMAN]	5.45	1	2	2	2					0.92	0.89	0.98	0.95	1211	134.7	7.15
P59510	A disintegrin and metalloproteinase with thrombospondin motifs 20 OS=Homo sapiens GN=ADAMTS20 PE=2 SV=2 - [ATS20_HUMAN]	1.47	1	2	2	4	1.02	0.70	0.85	0.72					1910	214.6	7.21
Q9UNA0	A disintegrin and metalloproteinase with thrombospondin motifs 5 OS=Homo sapiens GN=ADAMTS5 PE=1 SV=2 - [ATS5_HUMAN]	1.08	1	1	1	1	0.97	0.95	1.19	1.16					930	101.7	8.85
Q9UKP5	A disintegrin and metalloproteinase with thrombospondin motifs 6 OS=Homo sapiens GN=ADAMTS6 PE=2 SV=2 - [ATS6_HUMAN]	5.73	1	2	2	4					1.57	2.30	0.88	1.29	1117	125.2	6.68
Q9UKP4	A disintegrin and metalloproteinase with thrombospondin motifs 7 OS=Homo sapiens GN=ADAMTS7 PE=1 SV=2 - [ATS7_HUMAN]	1.54	1	1	1	3	0.78	0.84	0.67	0.73					1686	184.0	6.20
Q02952	A-kinase anchor protein 12 OS=Homo sapiens GN=AKAP12 PE=1 SV=4 - [AKA12_HUMAN]	1.40	1	1	1	1	1.31	0.99	1.29	0.97					1782	191.4	4.41
Q12802	A-kinase anchor protein 13 OS=Homo sapiens GN=AKAP13 PE=1 SV=2 - [AKP13_HUMAN]	3.63	1	5	5	8	0.21	0.12	0.27	0.15	1.13	0.85	1.24	1.02	2813	307.4	5.24
Q86UN6	A-kinase anchor protein 14 OS=Homo sapiens GN=AKAP14 PE=1 SV=1 - [AKA28_HUMAN]	5.58	1	1	1	2					1.18	1.50	0.85	1.08	197	22.8	6.80
Q13023	A-kinase anchor protein 6 OS=Homo sapiens GN=AKAP6 PE=1 SV=3 - [AKAP6_HUMAN]	1.03	1	2	2	6	1.40	1.66	1.09	1.30	1.10	1.10	1.09	1.09	2319	256.6	5.01
Q99996	A-kinase anchor protein 9 OS=Homo sapiens GN=AKAP9 PE=1 SV=3 - [AKAP9_HUMAN]	1.59	3	5	6	7	0.97	1.09	1.07	1.21	0.99	0.83	0.72	0.77	3911	453.4	4.98
Q2M3C7	A-kinase anchor protein SPHKAP OS=Homo sapiens GN=SPHKAP PE=1 SV=1 - [SPKAP_HUMAN]	0.71	1	1	1	3	1.41	1.25	1.51	1.36	1.18	1.02	1.61	1.39	1700	186.3	5.14
P42684	Abelson tyrosine-protein kinase 2 OS=Homo sapiens GN=ABL2 PE=1 SV=1 - [ABL2_HUMAN]	5.33	1	2	2	3	0.86	0.80	0.74	0.68					1182	128.3	8.07
Q96IU4	Abhydrolase domain-containing protein 14B OS=Homo sapiens GN=ABHD14B PE=1 SV=1 - [ABHEB_HUMAN]	10.00	1	2	2	3					0.90	0.94	0.94	0.93	210	22.3	6.40
P08910	Abhydrolase domain-containing protein 2 OS=Homo sapiens GN=ABHD2 PE=2 SV=1 - [ABHD2_HUMAN]	6.35	1	1	2	4	0.67	0.17	0.86	0.22					425	48.3	6.70
Q8WU67	Abhydrolase domain-containing protein 3 OS=Homo sapiens GN=ABHD3 PE=2 SV=2 - [ABHD3_HUMAN]	3.42	1	1	1	1					0.91	1.22	2.37	3.18	409	46.0	7.17
Q8TB40	Abhydrolase domain-containing protein 4 OS=Homo sapiens GN=ABHD4 PE=2 SV=1 - [ABHD4_HUMAN]	8.48	1	1	1	1	0.45	0.51	0.61	0.69					342	38.8	7.59
Q8IZT6	Abnormal spindle-like microcephaly-associated protein OS=Homo sapiens GN=ASPM PE=1 SV=2 - [ASPM_HUMAN]	0.75	1	2	2	2	1.09	1.12	1.11	1.15	0.88	0.81	0.87	0.80	3477	409.5	10.45
Q9Y4K1	Absent in melanoma 1 protein OS=Homo sapiens GN=AIM1 PE=1 SV=3 - [AIM1_HUMAN]	5.40	1	4	4	11	1.53	1.14	1.32	1.00	0.45	0.27	0.59	0.54	1723	188.6	5.86
Q9BWD1	Acetyl-CoA acetyltransferase, cytosolic OS=Homo sapiens GN=ACAT2 PE=1 SV=2 - [THIC_HUMAN]	8.82	1	2	2	5	1.21	1.27	1.27	1.35					397	41.3	6.92
Q13085	Acetyl-CoA carboxylase 1 OS=Homo sapiens GN=ACACA PE=1 SV=2 - [ACACA_HUMAN]	1.88	1	4	4	8	1.06	0.91	1.04	0.90	3.97	3.26	4.00	3.23	2346	265.4	6.37
O00763	Acetyl-CoA carboxylase 2 OS=Homo sapiens GN=ACACB PE=1 SV=3 - [ACACB_HUMAN]	0.65	1	1	1	1	1.17	0.87	1.46	1.08					2458	276.4	6.49
Q9NR19	Acetyl-coenzyme A synthetase, cytoplasmic OS=Homo sapiens GN=ACSS2 PE=1 SV=1 - [ACSA_HUMAN]	0.86	1	1	1	1	1.49	1.93	1.63	2.13					701	78.5	6.46
Q07001	Acetylcholine receptor subunit delta OS=Homo sapiens GN=CHRND PE=1 SV=1 - [ACHD_HUMAN]	1.93	1	1	1	1	0.36	0.48	0.46	0.61					517	58.9	6.55
Q04844	Acetylcholine receptor subunit epsilon OS=Homo sapiens GN=CHRNE PE=1 SV=2 - [ACHE_HUMAN]	3.04	1	1	1	1	0.72	0.85	0.68	0.80					493	54.7	5.26
Q9Y215	Acetylcholinesterase collagenic tail peptide OS=Homo sapiens GN=COLQ PE=1 SV=2 - [COLQ_HUMAN]	0.66	1	1	1	1					1.36	1.31	1.43	1.38	455	47.7	8.09
P22303	Acetylcholinesterase OS=Homo sapiens GN=ACHE PE=1 SV=1 - [ACES_HUMAN]	10.26	1	2	2	3	0.72	0.87	1.40	0.95					614	67.8	6.29
P46597	Acetylserotonin O-methyltransferase OS=Homo sapiens GN=ASMT PE=1 SV=1 - [ASMT_HUMAN]	10.43	1	2	2	2							0.63	0.07	345	38.4	5.19

Q13510	Acid ceramidase OS=Homo sapiens GN=ASAH1 PE=1 SV=5 - [ASAH1_HUMAN]	2.28	1	1	1	1	0.80	0.71	1.02	0.90					395	44.6	7.62
Q92485	Acid sphingomyelinase-like phosphodiesterase 3b OS=Homo sapiens GN=SMPDL3B PE=2 SV=2 - [ASM3B_HUMAN]	2.64	1	1	1	1	0.71	0.74	0.70	0.73					455	50.8	5.64
Q9UHC3	Acid-sensing ion channel 3 OS=Homo sapiens GN=ASIC3 PE=1 SV=2 - [ASIC3_HUMAN]	4.33	1	1	1	2	1.25	1.04	1.35	1.13					531	58.9	7.14
Q96QF7	Acidic repeat-containing protein OS=Homo sapiens GN=ACRC PE=2 SV=1 - [ACRC_HUMAN]	0.72	1	1	1	2					0.74	0.95	0.72	0.90	691	76.1	4.60
Q8NEB7	Acrosin-binding protein OS=Homo sapiens GN=ACRBP PE=2 SV=1 - [ACRBP_HUMAN]	3.13	1	1	1	1					1.06	1.17	1.02	1.12	543	61.3	5.16
Q8N4X5	Actin filament-associated protein 1-like 2 OS=Homo sapiens GN=AFAP1L2 PE=1 SV=1 - [AF1L2_HUMAN]	3.06	1	2	2	3	0.95	0.99	1.04	1.09					818	91.2	5.31
O14639	Actin-binding LIM protein 1 OS=Homo sapiens GN=ABLIM1 PE=1 SV=3 - [ABLM1_HUMAN]	1.41	1	1	1	2	0.98	1.13	0.95	1.10					778	87.6	8.59
Q9NQW6	Actin-binding protein anillin OS=Homo sapiens GN=ANLN PE=1 SV=2 - [ANLN_HUMAN]	1.87	1	1	1	1					0.87	0.72	1.60	1.33	1124	124.1	8.07
P61160	Actin-related protein 2 OS=Homo sapiens GN=ACTR2 PE=1 SV=1 - [ARP2_HUMAN]	5.84	9	1	3	5	0.75	0.74	0.76	0.75					394	44.7	6.74
O15143	Actin-related protein 2/3 complex subunit 1B OS=Homo sapiens GN=ARPC1B PE=1 SV=3 - [ARC1B_HUMAN]	6.72	1	2	2	3	0.71	0.58	0.70	0.58					372	40.9	8.35
O15144	Actin-related protein 2/3 complex subunit 2 OS=Homo sapiens GN=ARPC2 PE=1 SV=1 - [ARPC2_HUMAN]	4.33	1	1	1	10	0.74	0.76	0.79	0.82	0.62	0.69	0.67	0.74	300	34.3	7.36
P59998	Actin-related protein 2/3 complex subunit 4 OS=Homo sapiens GN=ARPC4 PE=1 SV=3 - [ARPC4_HUMAN]	6.55	1	1	1	2	0.85	0.94	0.83	0.92					168	19.7	8.43
O15511	Actin-related protein 2/3 complex subunit 5 OS=Homo sapiens GN=ARPC5 PE=1 SV=3 - [ARPC5_HUMAN]	24.50	1	3	3	9					1.07	0.89	0.89	0.82	151	16.3	5.67
P61158	Actin-related protein 3 OS=Homo sapiens GN=ACTR3 PE=1 SV=3 - [ARP3_HUMAN]	12.92	3	4	5	13	0.79	0.73	0.95	0.92	1.08	0.77	1.07	0.76	418	47.3	5.88
Q8TDG2	Actin-related protein T1 OS=Homo sapiens GN=ACTRT1 PE=2 SV=2 - [ACTT1_HUMAN]	6.65	1	1	1	5	1.48	0.93	1.72	0.96					376	41.7	6.79
P68032	Actin, alpha cardiac muscle 1 OS=Homo sapiens GN=ACTC1 PE=1 SV=1 - [ACTC_HUMAN]	28.12	5	1	11	179	0.61	0.55	0.55	0.50	0.63	0.77	0.79	0.97	377	42.0	5.39
P60709	Actin, cytoplasmic 1 OS=Homo sapiens GN=ACTB PE=1 SV=1 - [ACTB_HUMAN]	50.67	3	4	16	241	0.66	0.67	0.69	0.70	0.77	0.81	0.78	0.85	375	41.7	5.48
Q9C0C7	Activating molecule in BECN1-regulated autophagy protein 1 OS=Homo sapiens GN=AMBRA1 PE=1 SV=2 - [AMRA1_HUMAN]	0.92	1	1	1	1	1.45	1.38	1.51	1.45					1298	142.4	7.18
Q8N3C0	Activating signal cointegrator 1 complex subunit 3 OS=Homo sapiens GN=ASCC3 PE=1 SV=3 - [ASCC3_HUMAN]	2.27	1	2	3	8	0.98	0.80	1.09	0.89					2202	251.3	7.09
Q5U623	Activating transcription factor 7-interacting protein 2 OS=Homo sapiens GN=ATF7IP2 PE=1 SV=2 - [MCAF2_HUMAN]	5.43	1	2	2	2					5.54	4.10	5.12	3.79	682	75.7	7.75
Q9ULW3	Activator of basal transcription 1 OS=Homo sapiens GN=ABT1 PE=1 SV=1 - [ABT1_HUMAN]	2.57	1	1	1	1	0.49	0.12	0.97	0.23					272	31.1	9.88
Q9H2P0	Activity-dependent neuroprotector homeobox protein OS=Homo sapiens GN=ADNP PE=1 SV=1 - [ADNP_HUMAN]	1.09	1	1	1	1	0.12	0.14	0.11	0.13					1102	123.5	7.34
Q6JQN1	Acyl-CoA dehydrogenase family member 10 OS=Homo sapiens GN=ACAD10 PE=2 SV=1 - [ACD10_HUMAN]	1.51	1	1	1	1					1.03	1.04	1.09	1.10	1059	118.8	8.06
Q9H845	Acyl-CoA dehydrogenase family member 9, mitochondrial OS=Homo sapiens GN=ACAD9 PE=1 SV=1 - [ACAD9_HUMAN]	1.45	1	1	1	4					3.94	3.91	3.57	3.56	621	68.7	7.96
Q4L235	Acyl-CoA synthetase family member 4 OS=Homo sapiens GN=AASDH PE=2 SV=3 - [ACSF4_HUMAN]	2.55	1	1	1	2					1.19	1.16	1.16	1.13	1098	122.5	7.24
Q9BR61	Acyl-CoA-binding domain-containing protein 6 OS=Homo sapiens GN=ACBD6 PE=1 SV=1 - [ACBD6_HUMAN]	3.55	1	1	1	1				0.85					282	31.1	5.11
P07108	Acyl-CoA-binding protein OS=Homo sapiens GN=DBI PE=1 SV=2 - [ACBP_HUMAN]	32.18	1	2	2	11					1.51	1.39	1.38	1.32	87	10.0	6.57

P0C7M7	Acyl-coenzyme A synthetase ACSM4, mitochondrial OS=Homo sapiens GN=ACSM4 PE=1 SV=1 - [ACSM4_HUMAN]	1.03	1	1	1	1	1.36	1.24	1.43	1.32					580	65.7	8.59
Q6NUN0	Acyl-coenzyme A synthetase ACSM5, mitochondrial OS=Homo sapiens GN=ACSM5 PE=2 SV=2 - [ACSM5_HUMAN]	1.38	1	1	1	1					1.12	1.46	1.11	1.45	579	64.7	8.40
Q6P461	Acyl-coenzyme A synthetase ACSM6, mitochondrial OS=Homo sapiens GN=ACSM6 PE=2 SV=3 - [ACSM6_HUMAN]	5.21	1	1	1	1					1.04	1.05	1.00	1.01	480	53.5	8.41
Q8WXI4	Acyl-coenzyme A thioesterase 11 OS=Homo sapiens GN=ACOT11 PE=1 SV=1 - [ACO11_HUMAN]	1.81	1	2	2	2					0.66	0.84	0.70	0.88	607	68.4	8.35
Q9Y305	Acyl-coenzyme A thioesterase 9, mitochondrial OS=Homo sapiens GN=ACOT9 PE=1 SV=2 - [ACOT9_HUMAN]	3.64	1	1	1	1					1.44	0.83	1.27	0.73	439	49.9	8.60
O75608	Acyl-protein thioesterase 1 OS=Homo sapiens GN=LYPLA1 PE=1 SV=1 - [LYPA1_HUMAN]	5.65	1	1	1	4	0.99	0.98	0.86	0.87	1.10	1.17	1.11	1.18	230	24.7	6.77
O95372	Acyl-protein thioesterase 2 OS=Homo sapiens GN=LYPLA2 PE=1 SV=1 - [LYPA2_HUMAN]	3.46	1	1	1	2					1.03	1.10	0.97	1.04	231	24.7	7.23
O15204	ADAM DEC1 OS=Homo sapiens GN=ADAMDEC1 PE=1 SV=2 - [ADEC1_HUMAN]	2.77	1	1	1	4	1.00	0.98	0.99	0.97	1.00	0.95	1.06	1.00	470	52.7	7.34
Q86TH1	ADAMTS-like protein 2 OS=Homo sapiens GN=ADAMTSL2 PE=1 SV=1 - [ATL2_HUMAN]	3.15	1	3	3	6	1.03	1.03	1.18	1.19	0.97	0.87	0.90	0.89	951	104.6	6.42
Q6UY14	ADAMTS-like protein 4 OS=Homo sapiens GN=ADAMTSL4 PE=1 SV=2 - [ATL4_HUMAN]	7.36	1	6	6	30	0.96	0.85	0.94	0.92	0.98	0.88	0.98	0.93	1074	116.5	8.34
P46108	Adapter molecule crk OS=Homo sapiens GN=CRK PE=1 SV=2 - [CRK_HUMAN]	2.30	1	1	1	3					1.13	1.29	1.15	1.31	304	33.8	5.55
O43734	Adapter protein CIKS OS=Homo sapiens GN=TRAF3IP2 PE=1 SV=3 - [CIKS_HUMAN]	3.83	1	1	1	1	0.87	0.80	0.91	0.85					574	64.6	6.76
P07741	Adenine phosphoribosyltransferase OS=Homo sapiens GN=APRT PE=1 SV=2 - [APT_HUMAN]	21.67	1	3	3	7					1.00	0.96	0.91	0.90	180	19.6	6.02
O95996	Adenomatous polyposis coli protein 2 OS=Homo sapiens GN=APC2 PE=1 SV=1 - [APC2_HUMAN]	1.13	1	1	1	1	1.12	1.12	1.25	1.25					2303	243.8	8.82
P25054	Adenomatous polyposis coli protein OS=Homo sapiens GN=APC PE=1 SV=2 - [APC_HUMAN]	0.98	1	2	2	2					0.84	0.96	0.76	0.87	2843	311.5	7.80
Q9NZK5	Adenosine deaminase CECR1 OS=Homo sapiens GN=CECR1 PE=1 SV=2 - [CECR1_HUMAN]	4.70	1	2	2	4	1.28	1.75	1.35	1.85	1.43	0.79	1.28	0.71	511	58.9	7.91
Q8NCV1	Adenosine deaminase domain-containing protein 2 OS=Homo sapiens GN=ADAD2 PE=2 SV=1 - [ADAD2_HUMAN]	5.66	1	1	1	2					0.71	0.72	0.74	0.75	583	61.7	8.84
P55263	Adenosine kinase OS=Homo sapiens GN=ADK PE=1 SV=2 - [ADK_HUMAN]	2.76	1	1	1	1					1.71	1.63	1.06	1.01	362	40.5	6.70
Q08462	Adenylate cyclase type 2 OS=Homo sapiens GN=ADCY2 PE=1 SV=5 - [ADCY2_HUMAN]	1.28	1	1	1	1					0.93	1.01	0.86	0.94	1091	123.5	8.09
O95622	Adenylate cyclase type 5 OS=Homo sapiens GN=ADCY5 PE=1 SV=3 - [ADCY5_HUMAN]	0.63	1	1	1	2	0.85	0.93	0.77	0.84					1261	138.8	7.24
O43306	Adenylate cyclase type 6 OS=Homo sapiens GN=ADCY6 PE=1 SV=2 - [ADCY6_HUMAN]	1.20	1	1	1	3					1.77	2.01	1.64	1.86	1168	130.5	8.22
P40145	Adenylate cyclase type 8 OS=Homo sapiens GN=ADCY8 PE=1 SV=1 - [ADCY8_HUMAN]	1.92	1	2	2	2	0.97	1.00	0.98	1.01					1251	140.0	6.99
P54819	Adenylate kinase 2, mitochondrial OS=Homo sapiens GN=AK2 PE=1 SV=2 - [KAD2_HUMAN]	10.46	1	1	1	1							1.42		239	26.5	7.81
P00568	Adenylate kinase isoenzyme 1 OS=Homo sapiens GN=AK1 PE=1 SV=3 - [KAD1_HUMAN]	13.40	1	2	2	7	0.82	0.68	0.84	0.70	1.01	1.06	0.93	0.97	194	21.6	8.63
P30566	Adenylosuccinate lyase OS=Homo sapiens GN=ADSL PE=1 SV=2 - [PUR8_HUMAN]	4.96	1	1	1	1	1.11	1.16	1.72	1.81					484	54.9	7.11
Q01518	Adenylyl cyclase-associated protein 1 OS=Homo sapiens GN=CAP1 PE=1 SV=5 - [CAP1_HUMAN]	31.58	1	11	11	45	0.69	0.82	0.75	0.85	0.89	0.91	0.84	0.86	475	51.9	8.06
O95396	Adenylyltransferase and sulfurtransferase MOCS3 OS=Homo sapiens GN=MOCS3 PE=1 SV=1 - [MOCS3_HUMAN]	2.17	1	1	1	1					1.05	1.01	1.05	1.01	460	49.6	6.21

Q9HDC9	Adipocyte plasma membrane-associated protein OS=Homo sapiens GN=APMAP PE=1 SV=2 - [APMAP_HUMAN]	31.73	1	11	11	54	1.07	1.02	1.01	1.00	0.95	0.98	0.89	1.02	416	46.5	6.16
Q15848	Adiponectin OS=Homo sapiens GN=ADIPOQ PE=1 SV=1 - [ADIPO_HUMAN]	27.05	1	5	5	31	1.21	1.27	1.26	1.30	1.06	1.13	0.99	1.05	244	26.4	5.74
Q86V24	Adiponectin receptor protein 2 OS=Homo sapiens GN=ADIPOR2 PE=1 SV=1 - [ADR2_HUMAN]	4.40	1	1	1	1					0.37	1.14	0.55	1.67	386	43.9	6.60
Q6IQ32	ADNP homeobox protein 2 OS=Homo sapiens GN=ADNP2 PE=1 SV=1 - [ADNP2_HUMAN]	1.50	1	2	2	3	1.29	1.64	1.19	1.52	0.81	0.90	0.73	0.81	1131	122.8	9.16
Q9BW91	ADP-ribose pyrophosphatase, mitochondrial OS=Homo sapiens GN=NUDT9 PE=1 SV=1 - [NUDT9_HUMAN]	10.86	1	2	2	2	1.15	1.34	1.32	1.57					350	39.1	8.22
Q10588	ADP-ribosyl cyclase 2 OS=Homo sapiens GN=BST1 PE=1 SV=2 - [BST1_HUMAN]	15.41	1	5	5	12	1.28	1.26	1.25	1.25	0.89	0.99	0.91	0.95	318	35.7	7.80
P61204	ADP-ribosylation factor 3 OS=Homo sapiens GN=ARF3 PE=1 SV=2 - [ARF3_HUMAN]	22.10	2	3	4	13	0.93	1.00	0.90	0.97	0.93	0.91	0.98	0.96	181	20.6	7.43
P18085	ADP-ribosylation factor 4 OS=Homo sapiens GN=ARF4 PE=1 SV=3 - [ARF4_HUMAN]	9.44	1	2	2	4	0.99	1.00	0.90	0.94					180	20.5	7.14
P84085	ADP-ribosylation factor 5 OS=Homo sapiens GN=ARF5 PE=1 SV=2 - [ARF5_HUMAN]	23.33	1	3	4	7	0.90	0.95	0.93	0.95	1.32	1.32	1.18	1.18	180	20.5	6.79
Q8N6H7	ADP-ribosylation factor GTPase-activating protein 2 OS=Homo sapiens GN=ARFGAP2 PE=1 SV=1 - [ARFG2_HUMAN]	1.54	1	1	1	2	1.77	1.74	1.72	1.69					521	56.7	7.99
Q9UJY5	ADP-ribosylation factor-binding protein GGA1 OS=Homo sapiens GN=GGA1 PE=1 SV=1 - [GGA1_HUMAN]	3.29	1	1	1	33	0.91	1.00	0.89	1.00	0.94	0.98	0.90	1.17	639	70.3	5.29
Q9UJY4	ADP-ribosylation factor-binding protein GGA2 OS=Homo sapiens GN=GGA2 PE=1 SV=3 - [GGA2_HUMAN]	1.79	1	1	1	3	1.98	2.11	1.85	1.97	0.35	0.78	0.77	1.73	613	67.1	6.55
P36404	ADP-ribosylation factor-like protein 2 OS=Homo sapiens GN=ARL2 PE=1 SV=4 - [ARL2_HUMAN]	9.78	1	1	1	1		0.58		0.56					184	20.9	6.34
P56559	ADP-ribosylation factor-like protein 4C OS=Homo sapiens GN=ARL4C PE=1 SV=1 - [ARL4C_HUMAN]	7.29	1	1	1	1	0.94	1.36	0.96	1.39					192	21.5	9.09
Q96BM9	ADP-ribosylation factor-like protein 8A OS=Homo sapiens GN=ARL8A PE=1 SV=1 - [ARL8A_HUMAN]	10.22	2	2	2	2					2.74	2.09	2.67	2.04	186	21.4	7.77
Q6T311	ADP-ribosylation factor-like protein 9 OS=Homo sapiens GN=ARL9 PE=2 SV=1 - [ARL9_HUMAN]	6.95	1	1	1	1					1.01	0.71	1.22	0.86	187	20.7	6.52
Q13795	ADP-ribosylation factor-related protein 1 OS=Homo sapiens GN=ARFRP1 PE=1 SV=1 - [ARFRP_HUMAN]	17.91	1	1	1	5	0.53		1.15		0.90	0.80	0.81	0.89	201	22.6	7.56
P05141	ADP/ATP translocase 2 OS=Homo sapiens GN=SLC25A5 PE=1 SV=7 - [ADT2_HUMAN]	7.05	4	2	2	3	0.28	0.34	0.29	0.35					298	32.8	9.69
O75366	Advillin OS=Homo sapiens GN=AVIL PE=1 SV=3 - [AVIL_HUMAN]	1.71	1	1	1	1	0.90	0.92	0.85	0.89					819	92.0	5.71
P51816	AF4/FMR2 family member 2 OS=Homo sapiens GN=AFF2 PE=1 SV=4 - [AFF2_HUMAN]	1.22	1	2	2	5	0.96	1.35	0.95	1.34	1.20	1.14	1.09	1.21	1311	144.7	8.05
P51826	AF4/FMR2 family member 3 OS=Homo sapiens GN=AFF3 PE=1 SV=2 - [AFF3_HUMAN]	5.46	1	2	2	5					0.65	0.63	0.71	0.67	1226	133.4	8.10
Q9UHB7	AF4/FMR2 family member 4 OS=Homo sapiens GN=AFF4 PE=1 SV=1 - [AFF4_HUMAN]	1.55	1	1	1	1	1.34	0.89	1.31	0.88					1163	127.4	9.31
P55196	Afadin OS=Homo sapiens GN=MLLT4 PE=1 SV=3 - [AFAD_HUMAN]	0.88	1	1	1	7	0.75	0.72	0.83	0.78	0.87	0.78	0.91	0.91	1824	206.7	6.47
P43652	Afamin OS=Homo sapiens GN=AFM PE=1 SV=1 - [AFAM_HUMAN]	69.28	1	47	47	1476	1.09	1.09	1.09	1.09	1.12	1.14	1.11	1.13	599	69.0	5.90
Q9Y4W6	AFG3-like protein 2 OS=Homo sapiens GN=AFG3L2 PE=1 SV=2 - [AFG32_HUMAN]	3.64	1	2	2	2	0.73	0.64	0.78	0.69	0.96	0.98	0.86	0.87	797	88.5	8.66
O95154	Aflatoxin B1 aldehyde reductase member 3 OS=Homo sapiens GN=AKR7A3 PE=1 SV=2 - [ARK73_HUMAN]	4.53	1	1	1	1					0.91	0.96	0.72	0.76	331	37.2	7.15
P42127	Agouti-signaling protein OS=Homo sapiens GN=ASIP PE=1 SV=1 - [ASIP_HUMAN]	9.09	1	1	1	1					0.87	0.75	0.82	0.70	132	14.5	9.64

P24298	Alanine aminotransferase 1 OS=Homo sapiens GN=GPT PE=1 SV=3 - [ALAT1_HUMAN]	1.81	1	1	1	3	0.75	0.76	0.78	0.79	0.93	1.19	1.10	1.41	496	54.6	7.18
Q8IUZ5	Alanine--glyoxylate aminotransferase 2-like 2 OS=Homo sapiens GN=AGXT2L2 PE=2 SV=1 - [AT2L2_HUMAN]	5.78	1	2	2	2					0.76	1.01	0.73	1.20	450	49.7	6.76
Q5JTZ9	Alanine--tRNA ligase, mitochondrial OS=Homo sapiens GN=AARS2 PE=1 SV=1 - [SYAM_HUMAN]	2.64	1	1	1	1					0.74				985	107.3	6.27
P47895	Aldehyde dehydrogenase family 1 member A3 OS=Homo sapiens GN=ALDH1A3 PE=1 SV=2 - [AL1A3_HUMAN]	4.49	1	2	2	3	0.87	0.78	0.80	0.73	1.05	0.88	0.97	0.81	512	56.1	7.25
P30837	Aldehyde dehydrogenase X, mitochondrial OS=Homo sapiens GN=ALDH1B1 PE=1 SV=3 - [AL1B1_HUMAN]	3.29	1	1	1	2	0.10	0.12	0.12	0.15					517	57.2	6.80
P05091	Aldehyde dehydrogenase, mitochondrial OS=Homo sapiens GN=ALDH2 PE=1 SV=2 - [ALDH2_HUMAN]	3.09	1	1	2	3	0.82	0.88	0.78	0.84					517	56.3	7.05
Q06278	Aldehyde oxidase OS=Homo sapiens GN=AOX1 PE=2 SV=2 - [ADO_HUMAN]	0.90	1	1	1	2	0.99	0.80	1.29	1.05					1338	147.8	7.17
O60218	Aldo-keto reductase family 1 member B10 OS=Homo sapiens GN=AKR1B10 PE=1 SV=2 - [AK1BA_HUMAN]	2.53	2	1	1	1	1.53	1.11	1.43	1.04					316	36.0	7.84
Q9UM73	ALK tyrosine kinase receptor OS=Homo sapiens GN=ALK PE=1 SV=3 - [ALK_HUMAN]	0.49	1	1	1	4					0.91	1.23	0.82	1.10	1620	176.3	7.08
P05186	Alkaline phosphatase, tissue-nonspecific isozyme OS=Homo sapiens GN=ALPL PE=1 SV=4 - [PPBT_HUMAN]	1.72	1	1	1	2					0.84	0.77	0.93	0.86	524	57.3	6.67
Q6NUM9	All-trans-retinol 13,14-reductase OS=Homo sapiens GN=RETSAT PE=2 SV=2 - [RETST_HUMAN]	5.90	1	2	2	2	1.06	1.00	1.17	1.11	1.06	0.99	1.13	1.06	610	66.8	8.28
P55008	Allograft inflammatory factor 1 OS=Homo sapiens GN=AIF1 PE=1 SV=1 - [AIF1_HUMAN]	11.56	1	2	2	3					1.17	0.93	1.13	0.90	147	16.7	6.24
Q9BQI0	Allograft inflammatory factor 1-like OS=Homo sapiens GN=AIF1L PE=1 SV=1 - [AIF1L_HUMAN]	4.00	1	1	1	2					1.58	1.21	1.50	1.15	150	17.1	7.20
Q11128	Alpha-(1,3)-fucosyltransferase OS=Homo sapiens GN=FUT5 PE=2 SV=1 - [FUT5_HUMAN]	8.82	2	2	2	2					1.02	0.46	1.23	0.56	374	43.0	8.28
P51993	Alpha-(1,3)-fucosyltransferase OS=Homo sapiens GN=FUT6 PE=1 SV=1 - [FUT6_HUMAN]	1.95	1	1	1	3					0.86	0.69	0.85	0.90	359	41.8	8.68
P02763	Alpha-1-acid glycoprotein 1 OS=Homo sapiens GN=ORM1 PE=1 SV=1 - [A1AG1_HUMAN]	64.18	1	9	17	1658	1.07	1.13	1.18	1.15	1.12	0.98	1.15	0.99	201	23.5	5.02
P19652	Alpha-1-acid glycoprotein 2 OS=Homo sapiens GN=ORM2 PE=1 SV=2 - [A1AG2_HUMAN]	65.67	1	8	16	1888	1.14	1.09	1.17	1.10	1.24	1.11	1.25	1.13	201	23.6	5.11
P01011	Alpha-1-antichymotrypsin OS=Homo sapiens GN=SERPINA3 PE=1 SV=2 - [AACT_HUMAN]	65.01	1	29	29	1203	1.09	1.16	1.08	1.12	1.03	1.07	0.99	1.04	423	47.6	5.52
P01009	Alpha-1-antitrypsin OS=Homo sapiens GN=SERPINA1 PE=1 SV=3 - [A1AT_HUMAN]	84.69	1	55	57	13969	1.22	1.19	1.22	1.19	1.06	1.05	1.04	1.06	418	46.7	5.59
P26572	Alpha-1,3-mannosyl-glycoprotein 2-beta-N-acetylglucosaminyltransferase OS=Homo sapiens GN=MGAT1 PE=2 SV=2 - [MGAT1_HUMAN]	4.94	1	2	2	3	0.97	1.02	0.96	1.02					445	50.8	9.16
Q09328	Alpha-1,6-mannosylglycoprotein 6-beta-N-acetylglucosaminyltransferase A OS=Homo sapiens GN=MGAT5 PE=1 SV=1 - [MGT5A_HUMAN]	1.62	1	1	1	24	1.11	1.14	1.14	1.20	1.14	1.09	1.25	1.14	741	84.5	8.12
P35348	Alpha-1A adrenergic receptor OS=Homo sapiens GN=ADRA1A PE=2 SV=2 - [ADA1A_HUMAN]	8.15	1	2	2	2					0.63	0.60	0.80	0.76	466	51.5	9.03
P04217	Alpha-1B-glycoprotein OS=Homo sapiens GN=A1BG PE=1 SV=4 - [A1BG_HUMAN]	58.18	1	29	29	2052	1.05	1.04	1.06	1.05	0.79	0.75	0.81	0.81	495	54.2	5.86
P08697	Alpha-2-antiplasmin OS=Homo sapiens GN=SERPINF2 PE=1 SV=3 - [A2AP_HUMAN]	51.32	1	23	23	526	1.13	1.10	1.11	1.09	1.06	1.06	1.00	1.01	491	54.5	6.29
P02765	Alpha-2-HS-glycoprotein OS=Homo sapiens GN=AHSG PE=1 SV=1 - [FETUA_HUMAN]	67.57	1	21	21	2624	1.20	1.15	1.16	1.13	0.87	0.84	0.93	0.95	367	39.3	5.72
P01023	Alpha-2-macroglobulin OS=Homo sapiens GN=A2M PE=1 SV=3 - [A2MG_HUMAN]	75.37	7	106	122	15003	1.19	1.25	1.21	1.26	1.15	1.19	1.12	1.17	1474	163.2	6.46
Q92186	Alpha-2,8-sialyltransferase 8B OS=Homo sapiens GN=ST8SIA2 PE=2 SV=1 - [SIA8B_HUMAN]	5.07	1	2	2	2					1.01	0.54	1.36	0.72	375	42.4	9.44

O15466	Alpha-2,8-sialyltransferase 8E OS=Homo sapiens GN=ST8SIA5 PE=2 SV=2 - [SIA8E_HUMAN]	3.19	1	1	1	31	1.15	1.00	1.02	0.92	0.90	0.84	0.98	0.89	376	43.9	9.01
P12814	Alpha-actinin-1 OS=Homo sapiens GN=ACTN1 PE=1 SV=2 - [ACTN1_HUMAN]	39.13	1	16	31	81	0.66	0.67	0.65	0.67	0.89	1.00	1.86	2.09	892	103.0	5.41
P35609	Alpha-actinin-2 OS=Homo sapiens GN=ACTN2 PE=1 SV=1 - [ACTN2_HUMAN]	15.77	1	3	14	29	0.72	0.68	0.75	0.70					894	103.8	5.45
O43707	Alpha-actinin-4 OS=Homo sapiens GN=ACTN4 PE=1 SV=2 - [ACTN4_HUMAN]	20.97	1	4	17	43	0.86	0.74	0.85	0.74					911	104.8	5.44
P04745	Alpha-amylase 1 OS=Homo sapiens GN=AMY1A PE=1 SV=2 - [AMY1_HUMAN]	4.70	1	1	2	8	1.01	1.22	1.16	1.41	1.59	2.00	1.31	1.64	511	57.7	6.93
P19961	Alpha-amylase 2B OS=Homo sapiens GN=AMY2B PE=1 SV=1 - [AMY2B_HUMAN]	4.70	1	1	2	4	1.55	1.56	1.79	1.83					511	57.7	7.09
P06733	Alpha-enolase OS=Homo sapiens GN=ENO1 PE=1 SV=2 - [ENOA_HUMAN]	32.49	3	11	11	25	0.71	0.61	0.80	0.67	0.79	0.82	0.71	0.74	434	47.1	7.39
P02771	Alpha-fetoprotein OS=Homo sapiens GN=AFP PE=1 SV=1 - [FETA_HUMAN]	2.30	1	1	1	4				0.03					609	68.6	5.68
Q16352	Alpha-internexin OS=Homo sapiens GN=INA PE=1 SV=2 - [AINX_HUMAN]	5.21	1	2	2	2	1.74	1.10	1.80	1.16					499	55.4	5.40
P00709	Alpha-lactalbumin OS=Homo sapiens GN=LALBA PE=1 SV=1 [LALBA_HUMAN]	15.49	1	2	2	4					8.19	4.91	5.97	3.57	142	16.2	5.00
Q16706	Alpha-mannosidase 2 OS=Homo sapiens GN=MAN2A1 PE=1 SV=2 - [MA2A1_HUMAN]	10.93	1	9	9	16	1.08	1.05	1.02	1.03					1144	131.1	7.58
P49641	Alpha-mannosidase 2x OS=Homo sapiens GN=MAN2A2 PE=2 SV=3 - [MA2A2_HUMAN]	3.91	1	3	3	4	1.05	1.03	1.02	1.06					1150	130.5	6.84
Q9NSC7	Alpha-N-acetylgalactosaminide alpha-2,6-sialyltransferase 1 OS=Homo sapiens GN=ST6GALNAC1 PE=1 SV=1 - [SIA7A_HUMAN]	2.00	1	1	1	1	1.34	1.11	1.58	1.31					600	68.5	9.92
Q9UJ37	Alpha-N-acetylgalactosaminide alpha-2,6-sialyltransferase 2 OS=Homo sapiens GN=ST6GALNAC2 PE=1 SV=1 - [SIA7B_HUMAN]	2.41	1	1	1	1					0.98	0.99	0.92	0.93	374	41.9	9.32
P54802	Alpha-N-acetylglucosaminidase OS=Homo sapiens GN=NAGLU PE=1 SV=2 - [ANAG_HUMAN]	17.09	1	9	9	28	1.09	1.08	1.12	1.12	1.19	1.17	1.35	1.19	743	82.2	6.65
Q96QP1	Alpha-protein kinase 1 OS=Homo sapiens GN=ALPK1 PE=2 SV=3 - [ALPK1_HUMAN]	3.54	1	2	2	3	2.03	1.89	2.09	1.95	1.39	0.79	1.20	0.68	1244	138.8	6.24
Q86TB3	Alpha-protein kinase 2 OS=Homo sapiens GN=ALPK2 PE=1 SV=3 - [ALPK2_HUMAN]	2.76	1	3	3	3	1.15	1.16	1.06	1.10	0.93	1.05	0.92	1.04	2170	236.9	5.24
Q96L96	Alpha-protein kinase 3 OS=Homo sapiens GN=ALPK3 PE=2 SV=2 - [ALPK3_HUMAN]	1.31	1	1	1	1					0.39	0.22	1.13	0.65	1907	201.1	7.58
P54920	Alpha-soluble NSF attachment protein OS=Homo sapiens GN=NAPA PE=1 SV=3 - [SNAA_HUMAN]	5.08	1	1	1	3					0.27	0.23	0.90	0.66	295	33.2	5.36
P37840	Alpha-synuclein OS=Homo sapiens GN=SNCA PE=1 SV=1 - [SYUA_HUMAN]	41.43	2	4	4	16					0.93	0.93	0.79	0.94	140	14.5	4.70
O75443	Alpha-tectorin OS=Homo sapiens GN=TECTA PE=1 SV=3 - [TECTA_HUMAN]	1.16	1	2	2	4	1.07	1.13	1.24	1.36	1.32	1.41	1.26	1.34	2155	239.4	5.40
Q60I27	ALS2 C-terminal-like protein OS=Homo sapiens GN=ALS2CL PE=1 SV=1 - [AL2CL_HUMAN]	3.46	1	2	2	2					0.73	0.66	0.92	0.83	953	107.7	6.15
Q8TCU4	Alstrom syndrome protein 1 OS=Homo sapiens GN=ALMS1 PE=1 SV=3 - [ALMS1_HUMAN]	0.48	1	2	2	2	0.94	0.97	0.77	0.80					4167	460.7	6.28
Q06203	Amidophosphoribosyltransferase OS=Homo sapiens GN=PPAT PE=1 SV=1 - [PUR1_HUMAN]	4.45	1	1	1	1	0.66	0.19							517	57.4	6.76
P51172	Amiloride-sensitive sodium channel subunit delta OS=Homo sapiens GN=SCNN1D PE=1 SV=2 - [SCNND_HUMAN]	3.13	1	1	1	2	1.34	1.29	1.16	1.13					638	70.2	7.87
P51170	Amiloride-sensitive sodium channel subunit gamma OS=Homo sapiens GN=SCNN1G PE=1 SV=4 - [SCNNG_HUMAN]	2.31	1	1	1	1					0.09	0.11	0.09	0.12	649	74.2	7.50
Q03154	Aminoacylase-1 OS=Homo sapiens GN=ACY1 PE=1 SV=1 - [ACY1_HUMAN]	3.68	1	1	1	1	0.79	0.92	0.97	1.13					408	45.9	6.18

A2RU49	Aminoglycoside phosphotransferase domain-containing protein 1 OS=Homo sapiens GN=AGPHD1 PE=2 SV=2 - [AGPD1_HUMAN]	6.43	1	1	1	1					1.69	1.44	1.56	1.33	373	41.9	6.84
P15144	Aminopeptidase N OS=Homo sapiens GN=ANPEP PE=1 SV=4 - [AMPN_HUMAN]	11.58	1	12	12	33	0.93	0.95	0.98	1.00	0.91	0.97	0.91	0.98	967	109.5	5.48
Q8N6M6	Aminopeptidase O OS=Homo sapiens GN=AOPEP PE=1 SV=2 - [AMPO_HUMAN]	1.83	1	1	1	2	1.16	1.24	1.27	1.36					819	93.5	6.19
Q6Q4G3	Aminopeptidase Q OS=Homo sapiens GN=AQPEP PE=1 SV=4 - [AMPQ_HUMAN]	0.91	1	1	1	2					0.68	0.81	0.63	0.74	990	113.2	5.63
Q86SJ2	Amphoterin-induced protein 2 OS=Homo sapiens GN=AMIGO2 PE=1 SV=1 - [AMGO2_HUMAN]	11.49	1	3	4	5	1.10	0.98	1.11	0.99	0.32	0.07	0.34	0.08	522	57.9	8.40
Q86WK7	Amphoterin-induced protein 3 OS=Homo sapiens GN=AMIGO3 PE=2 SV=1 - [AMGO3_HUMAN]	7.34	1	3	3	5					1.13	1.10	1.04	0.96	504	55.2	7.87
Q99767	Amyloid beta A4 precursor protein-binding family A member 2 OS=Homo sapiens GN=APBA2 PE=1 SV=3 - [APBA2_HUMAN]	2.67	1	1	1	1	1.61	1.03	1.53	0.98					749	82.5	4.86
Q7Z5R6	Amyloid beta A4 precursor protein-binding family B member 1-interacting protein OS=Homo sapiens GN=APBB1IP PE=1 SV=1 - [AB1IP_HUMAN]	3.45	1	1	1	1	0.74	0.64	0.77	0.67					666	73.1	5.59
Q92870	Amyloid beta A4 precursor protein-binding family B member 2 OS=Homo sapiens GN=APBB2 PE=1 SV=3 - [APBB2_HUMAN]	2.77	1	1	1	1	0.34	0.34	0.32	0.32					758	83.3	6.10
O95704	Amyloid beta A4 precursor protein-binding family B member 3 OS=Homo sapiens GN=APBB3 PE=1 SV=2 - [APBB3_HUMAN]	3.50	1	1	1	1	1.40	1.29	1.38	1.28					486	52.6	6.40
P05067	Amyloid beta A4 protein OS=Homo sapiens GN=APP PE=1 SV=3 - [A4_HUMAN]	15.19	2	11	11	31	1.29	1.33	1.27	1.35	1.13	1.07	1.18	1.06	770	86.9	4.82
Q9H8U3	AN1-type zinc finger protein 3 OS=Homo sapiens GN=ZFAND3 PE=1 SV=1 - [ZFAN3_HUMAN]	3.96	1	1	1	1					1.14	1.13	1.29	1.28	227	25.2	7.43
Q9H1A4	Anaphase-promoting complex subunit 1 OS=Homo sapiens GN=ANAPC1 PE=1 SV=1 - [APC1_HUMAN]	2.42	1	3	3	4	0.67	0.79	0.62	0.77	1.06	1.23	0.74	0.85	1944	216.4	6.30
Q9UJX4	Anaphase-promoting complex subunit 5 OS=Homo sapiens GN=ANAPC5 PE=1 SV=2 - [APC5_HUMAN]	1.19	1	1	1	1	1.08	0.82	0.96	0.74					755	85.0	6.87
P10275	Androgen receptor OS=Homo sapiens GN=AR PE=1 SV=2 - [ANDR_HUMAN]	1.85	1	1	1	1	0.08	0.23	0.43	1.22					919	98.9	6.42
Q8N7X0	Androglobin OS=Homo sapiens GN=ADGB PE=2 SV=3 - [ADGB_HUMAN]	1.68	1	2	2	5	0.80	0.37	0.81	0.38	1.27	1.27	1.09	1.17	1667	189.6	8.31
P03950	Angiogenin OS=Homo sapiens GN=ANG PE=1 SV=1 - [ANGI_HUMAN]	62.59	1	9	9	42	1.11	1.11	1.06	1.07	1.00	1.03	1.06	1.12	147	16.5	9.64
Q9Y2J4	Angiomotin-like protein 2 OS=Homo sapiens GN=AMOTL2 PE=1 SV=3 - [AMOL2_HUMAN]	1.80	1	1	1	1	0.82	0.82	0.83	0.83					779	85.7	7.30
Q15389	Angiopoietin-1 OS=Homo sapiens GN=ANGPT1 PE=1 SV=2 - [ANGP1_HUMAN]	7.23	2	3	3	3	2.87	0.77	1.35	0.36	1.33	1.00	1.43	0.99	498	57.5	6.76
Q02763	Angiopoietin-1 receptor OS=Homo sapiens GN=TEK PE=1 SV=2 - [TIE2_HUMAN]	2.14	1	2	2	3					0.71	0.89	1.25	1.57	1124	125.7	6.89
O15123	Angiopoietin-2 OS=Homo sapiens GN=ANGPT2 PE=1 SV=1 - [ANGP2_HUMAN]	6.05	1	2	2	5	1.18	1.18	1.19	1.20					496	56.9	5.58
Q9Y264	Angiopoietin-4 OS=Homo sapiens GN=ANGPT4 PE=1 SV=1 - [ANGP4_HUMAN]	2.19	1	1	1	1	0.98	1.36	0.88	1.22					503	56.8	8.91
Q9Y5C1	Angiopoietin-related protein 3 OS=Homo sapiens GN=ANGPTL3 PE=1 SV=1 - [ANGL3_HUMAN]	8.48	1	4	4	13	1.32	1.48	1.28	1.32	1.04	1.01	1.08	1.04	460	53.6	6.70
Q9BY76	Angiopoietin-related protein 4 OS=Homo sapiens GN=ANGPTL4 PE=1 SV=2 - [ANGL4_HUMAN]	4.93	1	1	1	1					0.86	0.80	0.92	0.86	406	45.2	8.85
Q8NI99	Angiopoietin-related protein 6 OS=Homo sapiens GN=ANGPTL6 PE=1 SV=1 - [ANGL6_HUMAN]	9.79	1	2	2	2	0.64		0.57						470	51.7	8.53
Q9BYF1	Angiotensin-converting enzyme 2 OS=Homo sapiens GN=ACE2 PE=1 SV=2 - [ACE2_HUMAN]	1.99	1	1	1	2					0.47	0.85	0.59	1.05	805	92.4	5.54
P12821	Angiotensin-converting enzyme OS=Homo sapiens GN=ACE PE=1 SV=1 - [ACE_HUMAN]	1.76	2	3	3	4	0.98	0.92	1.01	0.95					1306	149.6	6.39

P01019	Angiotensinogen OS=Homo sapiens GN=AGT PE=1 SV=1 - [ANGT_HUMAN]	45.36	1	20	20	614	1.26	1.37	1.28	1.38	1.20	1.31	1.13	1.24	485	53.1	6.32
Q96Q91	Anion exchange protein 4 OS=Homo sapiens GN=SLC4A9 PE=1 SV=2 - [B3A4_HUMAN]	1.63	1	1	1	1	1.80	1.75	1.85	1.80					983	108.2	7.09
Q7Z5J8	Ankyrin and armadillo repeat-containing protein OS=Homo sapiens GN=ANKAR PE=2 SV=3 - [ANKAR_HUMAN]	3.42	1	2	3	3	0.93	0.94	0.94	0.96					1434	161.9	8.10
Q8N961	Ankyrin repeat and BTB/POZ domain-containing protein 2 OS=Homo sapiens GN=ABTB2 PE=2 SV=1 - [ABTB2_HUMAN]	1.31	1	1	1	2	0.69	0.74	0.63	0.67					839	93.2	5.90
A6QL63	Ankyrin repeat and BTB/POZ domain-containing protein BTBD11 OS=Homo sapiens GN=BTBD11 PE=2 SV=3 - [BTBDB_HUMAN]	2.45	1	2	2	4	1.19	1.09	1.09	1.01	1.00	1.21	2.51	3.02	1104	120.8	6.83
Q9P2G1	Ankyrin repeat and IBR domain-containing protein 1 OS=Homo sapiens GN=ANKIB1 PE=1 SV=3 - [AKIB1_HUMAN]	1.84	1	1	1	1	1.45	1.81	0.88	1.11					1089	121.9	5.11
Q8IWZ3	Ankyrin repeat and KH domain-containing protein 1 OS=Homo sapiens GN=ANKHD1 PE=1 SV=1 - [ANKH1_HUMAN]	2.71	1	3	3	6	1.52	0.99	1.62	1.07	1.12	1.13	0.99	1.00	2542	269.3	5.73
Q9P2S6	Ankyrin repeat and MYND domain-containing protein 1 OS=Homo sapiens GN=ANKMY1 PE=2 SV=2 - [ANKY1_HUMAN]	1.06	1	1	1	1	1.04	1.16	1.13	1.27					941	105.4	6.73
Q8IV38	Ankyrin repeat and MYND domain-containing protein 2 OS=Homo sapiens GN=ANKMY2 PE=1 SV=1 - [ANKY2_HUMAN]	10.43	1	2	3	18	1.33	1.27	1.22	1.32					441	49.3	6.25
Q8NFD2	Ankyrin repeat and protein kinase domain-containing protein 1 OS=Homo sapiens GN=ANKK1 PE=1 SV=1 - [ANKK1_HUMAN]	6.01	1	2	2	3	0.80	0.97	0.72	0.85					765	84.6	7.08
Q92625	Ankyrin repeat and SAM domain-containing protein 1A OS=Homo sapiens GN=ANKS1A PE=1 SV=4 - [ANS1A_HUMAN]	2.20	1	2	2	3					1.11	1.21	1.20	1.28	1134	123.0	6.38
Q6ZW76	Ankyrin repeat and SAM domain-containing protein 3 OS=Homo sapiens GN=ANKS3 PE=1 SV=1 - [ANKS3_HUMAN]	3.20	1	2	2	2	1.17	1.19	1.07	1.10	0.48	0.65	0.56	0.76	656	72.0	5.45
Q8WXI3	Ankyrin repeat and SOCS box protein 10 OS=Homo sapiens GN=ASB10 PE=2 SV=2 - [ASB10_HUMAN]	2.78	1	1	1	2	0.77	0.82	0.90	0.97					467	50.9	7.01
Q8WXK3	Ankyrin repeat and SOCS box protein 13 OS=Homo sapiens GN=ASB13 PE=1 SV=2 - [ASB13_HUMAN]	3.96	1	1	1	3					0.72	0.86	0.62	0.73	278	30.0	6.81
A6NK59	Ankyrin repeat and SOCS box protein 14 OS=Homo sapiens GN=ASB14 PE=2 SV=2 - [ASB14_HUMAN]	3.75	1	1	1	2	0.86	1.05	1.02	1.25					587	65.3	6.71
Q6ZVZ8	Ankyrin repeat and SOCS box protein 18 OS=Homo sapiens GN=ASB18 PE=2 SV=2 - [ASB18_HUMAN]	8.15	1	2	2	2	0.71	0.67	0.83	0.80					466	50.8	6.96
Q96Q27	Ankyrin repeat and SOCS box protein 2 OS=Homo sapiens GN=ASB2 PE=1 SV=1 - [ASB2_HUMAN]	1.87	1	1	1	1	1.03	0.79	1.09	0.84					587	65.0	7.62
Q9H672	Ankyrin repeat and SOCS box protein 7 OS=Homo sapiens GN=ASB7 PE=1 SV=2 - [ASB7_HUMAN]	1.89	1	1	1	1	1.30	1.12	1.22	1.06					318	36.0	8.29
Q9H765	Ankyrin repeat and SOCS box protein 8 OS=Homo sapiens GN=ASB8 PE=2 SV=1 - [ASB8_HUMAN]	5.90	1	1	1	1	1.32	1.43	1.02	1.11					288	31.6	5.83
Q7Z6G8	Ankyrin repeat and sterile alpha motif domain-containing protein 1B OS=Homo sapiens GN=ANKS1B PE=1 SV=2 - [ANS1B_HUMAN]	0.80	1	1	1	2					1.16	1.31	1.21	1.37	1248	138.0	6.37
Q9H8Y5	Ankyrin repeat and zinc finger domain-containing protein 1 OS=Homo sapiens GN=ANKZF1 PE=1 SV=1 - [ANKZ1_HUMAN]	6.89	1	2	2	9	0.20	0.23	0.27	0.30					726	80.9	8.41
Q6UB98	Ankyrin repeat domain-containing protein 12 OS=Homo sapiens GN=ANKRD12 PE=1 SV=3 - [ANR12_HUMAN]	1.21	1	1	1	2	1.07	0.55	1.15	0.59					2062	235.5	7.01
Q8IZ07	Ankyrin repeat domain-containing protein 13A OS=Homo sapiens GN=ANKRD13A PE=1 SV=3 - [AN13A_HUMAN]	4.58	1	1	2	3					0.43	0.66	1.02	1.57	590	67.6	5.02
Q86YJ7	Ankyrin repeat domain-containing protein 13B OS=Homo sapiens GN=ANKRD13B PE=2 SV=4 - [AN13B_HUMAN]	2.72	1	1	1	2					1.01	1.12	1.00	1.11	626	70.2	6.93
Q6ZTN6	Ankyrin repeat domain-containing protein 13D OS=Homo sapiens GN=ANKRD13D PE=1 SV=2 - [AN13D_HUMAN]	5.02	1	1	2	2					0.92	0.90	0.92	0.90	518	58.4	5.38
Q6P6B7	Ankyrin repeat domain-containing protein 16 OS=Homo sapiens GN=ANKRD16 PE=2 SV=1 - [ANR16_HUMAN]	15.51	1	4	4	18	0.87	0.80	0.89	0.83	0.55	0.49	0.54	0.65	361	39.3	7.25
O75179	Ankyrin repeat domain-containing protein 17 OS=Homo sapiens GN=ANKRD17 PE=1 SV=3 - [ANR17_HUMAN]	0.50	1	1	1	3	0.66	0.76	0.69	0.81					2603	274.1	6.52

Q8IVF6	Ankyrin repeat domain-containing protein 18A OS=Homo sapiens GN=ANKRD18A PE=1 SV=3 - [AN18A_HUMAN]	0.71	1	1	1	1	1.36	1.64	1.38	1.68					992	115.5	7.83
Q9GZV1	Ankyrin repeat domain-containing protein 2 OS=Homo sapiens GN=ANKRD2 PE=1 SV=3 - [ANKR2_HUMAN]	3.33	1	1	1	2	0.61	0.51	0.62	0.52					360	39.8	6.09
Q5SQ80	Ankyrin repeat domain-containing protein 20A2 OS=Homo sapiens GN=ANKRD20A2 PE=2 SV=1 - [A20A2_HUMAN]	4.01	5	2	3	4					2.16	1.94	1.91	1.71	823	94.0	7.94
Q5CZ79	Ankyrin repeat domain-containing protein 20B OS=Homo sapiens GN=ANKRD20A8P PE=2 SV=2 - [AN20B_HUMAN]	2.31	2	1	2	3	2.14	1.87	1.77	1.55					823	93.9	8.32
Q8TF21	Ankyrin repeat domain-containing protein 24 OS=Homo sapiens GN=ANKRD24 PE=2 SV=2 - [ANR24_HUMAN]	2.79	1	2	2	2	0.97	0.92	0.96	0.92					1146	124.1	5.01
Q9UPS8	Ankyrin repeat domain-containing protein 26 OS=Homo sapiens GN=ANKRD26 PE=1 SV=3 - [ANR26_HUMAN]	2.87	7	4	5	5	0.61	0.33	0.98	0.54	0.67	0.54	0.89	0.71	1709	196.2	5.72
Q9BXX3	Ankyrin repeat domain-containing protein 30A OS=Homo sapiens GN=ANKRD30A PE=1 SV=3 - [AN30A_HUMAN]	0.64	1	1	1	2					0.92	1.04	0.91	1.02	1397	158.7	6.48
Q9BXX2	Ankyrin repeat domain-containing protein 30B OS=Homo sapiens GN=ANKRD30B PE=2 SV=3 - [AN30B_HUMAN]	1.51	1	1	2	2	1.66	1.57	1.38	1.33					1392	157.9	6.35
A5PLL1	Ankyrin repeat domain-containing protein 34B OS=Homo sapiens GN=ANKRD34B PE=2 SV=3 - [AN34B_HUMAN]	0.97	1	1	1	2					0.79	0.98	0.77	0.94	514	56.4	7.71
Q8N2N9	Ankyrin repeat domain-containing protein 36B OS=Homo sapiens GN=ANKRD36B PE=2 SV=4 - [AN36B_HUMAN]	1.63	3	2	2	4	6.27	5.65	5.94	5.44	1.63				1353	153.5	8.85
Q6AI12	Ankyrin repeat domain-containing protein 40 OS=Homo sapiens GN=ANKRD40 PE=1 SV=2 - [ANR40_HUMAN]	4.08	1	1	1	1	1.15	1.20	1.24	1.31					368	41.1	4.97
Q8N9B4	Ankyrin repeat domain-containing protein 42 OS=Homo sapiens GN=ANKRD42 PE=2 SV=2 - [ANR42_HUMAN]	3.60	1	1	1	1					1.76	1.36	0.96	0.74	389	43.0	6.48
Q9NU02	Ankyrin repeat domain-containing protein 5 OS=Homo sapiens GN=ANKRD5 PE=2 SV=2 - [ANKR5_HUMAN]	1.42	1	1	1	2	1.02	1.02	1.18	1.18					776	86.6	8.28
A6NEL2	Ankyrin repeat domain-containing protein SOWAHB OS=Homo sapiens GN=SOWAHB PE=2 SV=1 - [SWAHB_HUMAN]	2.90	1	1	1	1					0.66	0.79	0.65	0.77	793	85.7	9.60
Q9H9E1	Ankyrin repeat family A protein 2 OS=Homo sapiens GN=ANKRA2 PE=1 SV=1 - [ANRA2_HUMAN]	7.03	1	1	1	1	0.78	0.49	0.51	0.33					313	34.2	5.11
Q8WWH4	Ankyrin repeat, SAM and basic leucine zipper domain-containing protein 1 OS=Homo sapiens GN=ASZ1 PE=2 SV=1 - [ASZ1_HUMAN]	6.32	1	2	2	2	1.08	0.48	0.87	0.39					475	53.4	5.87
P16157	Ankyrin-1 OS=Homo sapiens GN=ANK1 PE=1 SV=3 - [ANK1_HUMAN]	1.06	1	2	2	2					1.30	0.83	0.97	0.36	1881	206.1	6.01
Q01484	Ankyrin-2 OS=Homo sapiens GN=ANK2 PE=1 SV=4 - [ANK2_HUMAN]	2.68	13	6	7	17	1.34	1.19	1.14	1.05	1.02	1.15	0.99	0.92	3957	433.4	5.14
Q12955	Ankyrin-3 OS=Homo sapiens GN=ANK3 PE=1 SV=3 - [ANK3_HUMAN]	1.14	1	3	3	4	1.37	1.32	1.19	1.15	1.41	1.41	1.31	1.32	4377	480.1	6.49
P04083	Annexin A1 OS=Homo sapiens GN=ANXA1 PE=1 SV=2 - [ANXA1_HUMAN]	13.01	1	3	3	6					0.83	0.83	0.83	0.83	346	38.7	7.02
Q9UJ72	Annexin A10 OS=Homo sapiens GN=ANXA10 PE=1 SV=3 - [ANX10_HUMAN]	4.32	1	1	1	4	0.91	0.92	0.83	0.84					324	37.3	5.33
P50995	Annexin A11 OS=Homo sapiens GN=ANXA11 PE=1 SV=1 - [ANX11_HUMAN]	1.78	1	1	1	2	1.29	1.13	1.26	1.11					505	54.4	7.65
P12429	Annexin A3 OS=Homo sapiens GN=ANXA3 PE=1 SV=3 - [ANXA3_HUMAN]	4.95	1	1	1	2	1.42	1.56	0.91	1.01					323	36.4	5.92
P08133	Annexin A6 OS=Homo sapiens GN=ANXA6 PE=1 SV=3 - [ANXA6_HUMAN]	2.38	1	1	1	2	1.08	0.85	1.06	0.84					673	75.8	5.60
P20073	Annexin A7 OS=Homo sapiens GN=ANXA7 PE=1 SV=3 - [ANXA7_HUMAN]	5.33	1	2	2	2	0.86	0.87	0.89	0.91					488	52.7	5.68
P13928	Annexin A8 OS=Homo sapiens GN=ANXA8 PE=1 SV=3 - [ANXA8_HUMAN]	3.36	3	1	1	1					1.17	0.83	1.14	0.80	327	36.9	5.78
Q3ZCQ2	Annexin-2 receptor OS=Homo sapiens GN=ANXA2R PE=2 SV=2 - [AX2R_HUMAN]	15.03	1	2	2	3	1.10	1.04	1.18	1.12	0.47	0.51	0.45	0.49	193	21.7	4.97

Q5XXA6	Anoctamin-1 OS=Homo sapiens GN=ANO1 PE=1 SV=1 - [ANO1_HUMAN]	1.12	1	1	1	1	0.85	0.91	1.34	1.43					986	114.0	8.54
Q9NW15	Anoctamin-10 OS=Homo sapiens GN=ANO10 PE=1 SV=2 - [ANO10_HUMAN]	1.82	1	1	1	1					0.95	0.95	0.93	0.93	660	76.3	7.40
Q9NQ90	Anoctamin-2 OS=Homo sapiens GN=ANO2 PE=1 SV=2 - [ANO2_HUMAN]	3.19	1	2	2	2	0.93	1.18	1.00	1.27	0.20	0.11	0.67	0.35	1003	113.9	6.55
Q4KMQ2	Anoctamin-6 OS=Homo sapiens GN=ANO6 PE=1 SV=2 - [ANO6_HUMAN]	2.20	1	1	1	2	1.03	0.78	0.88	0.67					910	106.1	7.77
Q6IWH7	Anoctamin-7 OS=Homo sapiens GN=ANO7 PE=1 SV=2 - [ANO7_HUMAN]	1.39	1	1	1	1					0.86	0.69	0.87	0.69	933	105.5	7.87
Q9H6X2	Anthrax toxin receptor 1 OS=Homo sapiens GN=ANTXR1 PE=1 SV=2 - [ANTR1_HUMAN]	2.30	1	1	1	3	0.85	0.79	0.95	0.93					564	62.7	7.61
P58335	Anthrax toxin receptor 2 OS=Homo sapiens GN=ANTXR2 PE=1 SV=5 - [ANTR2_HUMAN]	4.70	1	3	3	4	0.70	1.14	0.84	1.21					489	53.6	7.46
P46013	Antigen KI-67 OS=Homo sapiens GN=MKI67 PE=1 SV=2 - [KI67_HUMAN]	1.63	1	3	3	3	0.22	0.29	0.30	0.38	0.82	0.71	1.25	1.08	3256	358.5	9.45
Q03518	Antigen peptide transporter 1 OS=Homo sapiens GN=TAP1 PE=1 SV=2 - [TAP1_HUMAN]	2.10	1	1	1	1					0.32	0.24	0.94	0.69	808	87.2	8.02
P03973	Antileukoproteinase OS=Homo sapiens GN=SLPI PE=1 SV=2 - [SLPI_HUMAN]	27.27	1	4	4	7					1.17	1.17	1.27	1.26	132	14.3	8.75
P01008	Antithrombin-III OS=Homo sapiens GN=SERPINC1 PE=1 SV=1 - [ANT3_HUMAN]	65.95	1	44	44	3122	1.14	1.05	1.15	1.06	0.96	1.00	0.92	0.99	464	52.6	6.71
P56377	AP-1 complex subunit sigma-2 OS=Homo sapiens GN=AP1S2 PE=1 SV=1 - [AP1S2_HUMAN]	15.92	1	2	2	2					0.33	0.37	0.26	0.29	157	18.6	5.47
O00203	AP-3 complex subunit beta-1 OS=Homo sapiens GN=AP3B1 PE=1 SV=3 - [AP3B1_HUMAN]	0.64	1	1	1	1					1.11	1.59	1.18	1.69	1094	121.2	6.04
Q13367	AP-3 complex subunit beta-2 OS=Homo sapiens GN=AP3B2 PE=1 SV=2 - [AP3B2_HUMAN]	0.83	1	1	1	1	0.71	0.13	0.60	0.11					1082	119.0	5.59
O14617	AP-3 complex subunit delta-1 OS=Homo sapiens GN=AP3D1 PE=1 SV=1 - [AP3D1_HUMAN]	0.95	1	1	1	1					1.31	1.27	0.95	0.93	1153	130.1	8.48
Q96N21	AP-4 complex accessory subunit tepsin OS=Homo sapiens GN=C17orf56 PE=1 SV=1 - [AP4AT_HUMAN]	6.48	1	1	1	1	1.57	1.49	1.49	1.41					525	55.1	7.03
Q9Y6B7	AP-4 complex subunit beta-1 OS=Homo sapiens GN=AP4B1 PE=2 SV=2 - [AP4B1_HUMAN]	2.57	1	1	1	1						0.83		0.46	739	83.2	5.86
Q9UPM8	AP-4 complex subunit epsilon-1 OS=Homo sapiens GN=AP4E1 PE=1 SV=2 - [AP4E1_HUMAN]	0.53	1	1	1	1	0.67	0.64	0.76	0.72					1137	127.2	5.99
Q2M2I8	AP2-associated protein kinase 1 OS=Homo sapiens GN=AAK1 PE=1 SV=3 - [AAK1_HUMAN]	1.35	1	1	1	1	0.77	1.00	0.94	1.25					961	103.8	6.60
Q8N7J2	APC membrane recruitment protein 2 OS=Homo sapiens GN=FAM123A PE=1 SV=3 - [AMER2_HUMAN]	2.38	1	1	1	1	1.20	1.09	1.07	0.98					671	69.5	6.99
Q8N944	APC membrane recruitment protein 3 OS=Homo sapiens GN=FAM123C PE=2 SV=2 - [AMER3_HUMAN]	6.27	1	2	2	4					1.58	1.39	0.93	1.10	861	90.4	5.69
P02647	Apolipoprotein A-I OS=Homo sapiens GN=APOA1 PE=1 SV=1 - [APOA1_HUMAN]	90.64	9	47	50	7470	0.84	0.78	0.85	0.79	0.98	0.95	0.98	0.96	267	30.8	5.76
P02652	Apolipoprotein A-II OS=Homo sapiens GN=APOA2 PE=1 SV=1 - [APOA2_HUMAN]	77.00	2	11	12	1459	0.81	0.80	0.83	0.80	1.67	1.72	1.62	1.76	100	11.2	6.62
P06727	Apolipoprotein A-IV OS=Homo sapiens GN=APOA4 PE=1 SV=3 - [APOA4_HUMAN]	83.08	2	32	32	761	0.85	0.81	0.88	0.84	0.79	0.73	0.80	0.75	396	45.4	5.38
P04114	Apolipoprotein B-100 OS=Homo sapiens GN=APOB PE=1 SV=2 - [APOB_HUMAN]	70.46	7	341	343	11580	1.18	1.21	1.17	1.22	1.05	1.10	0.99	1.03	4563	515.3	7.05
P02654	Apolipoprotein C-I OS=Homo sapiens GN=APOC1 PE=1 SV=1 - [APOC1_HUMAN]	48.19	1	10	10	939	0.46	0.48	0.43	0.47	3.31	3.35	2.87	2.91	83	9.3	8.47
P02655	Apolipoprotein C-II OS=Homo sapiens GN=APOC2 PE=1 SV=1 - [APOC2_HUMAN]	56.44	1	5	5	191	0.79	0.91	0.77	0.95	2.82	2.81	2.74	2.69	101	11.3	4.72

P02656	Apolipoprotein C-III OS=Homo sapiens GN=APOC3 PE=1 SV=1 - [APOC3_HUMAN]	76.77	1	9	9	1425	0.82	0.72	0.82	0.69	1.66	1.58	1.67	1.49	99	10.8	5.41
P55056	Apolipoprotein C-IV OS=Homo sapiens GN=APOC4 PE=1 SV=1 - [APOC4_HUMAN]	45.67	1	7	7	52	1.10	1.12	1.20	1.22	2.16	2.12	1.89	2.03	127	14.5	8.92
P05090	Apolipoprotein D OS=Homo sapiens GN=APOD PE=1 SV=1 - [APOD_HUMAN]	51.85	1	12	12	699	1.16	1.08	1.15	1.08	1.19	1.24	1.15	1.19	189	21.3	5.15
P02649	Apolipoprotein E OS=Homo sapiens GN=APOE PE=1 SV=1 - [APOE_HUMAN]	80.13	1	31	31	887	0.94	0.90	0.94	0.93	0.95	0.92	0.96	0.99	317	36.1	5.73
Q13790	Apolipoprotein F OS=Homo sapiens GN=APOF PE=1 SV=2 - [APOF_HUMAN]	22.70	1	7	7	23	0.77	1.14	0.70	1.06	1.18	1.13	1.16	1.15	326	35.4	5.64
O14791	Apolipoprotein L1 OS=Homo sapiens GN=APOL1 PE=1 SV=5 [APOL1_HUMAN]	45.23	1	22	22	289	0.90	0.82	0.86	0.83	0.97	0.96	1.04	0.94	398	43.9	5.81
Q9BWW9	Apolipoprotein L5 OS=Homo sapiens GN=APOL5 PE=1 SV=1 [APOL5_HUMAN]	3.00	1	1	1	1	1.29	1.33	1.34	1.40					433	47.0	9.31
O95445	Apolipoprotein M OS=Homo sapiens GN=APOM PE=1 SV=2 - [APOM_HUMAN]	78.19	1	15	15	251	1.03	1.00	1.05	1.05	1.26	1.23	1.21	1.22	188	21.2	6.01
P08519	Apolipoprotein(a) OS=Homo sapiens GN=LPA PE=1 SV=1 - [APOA_HUMAN]	25.15	2	29	29	191	0.96	0.95	1.01	0.99	0.89	0.94	0.90	0.95	4548	501.0	5.88
O95831	Apoptosis-inducing factor 1, mitochondrial OS=Homo sapiens GN=AIFM1 PE=1 SV=1 - [AIFM1_HUMAN]	2.61	1	1	1	1	1.53	2.32	2.00	3.08					613	66.9	8.95
Q9BRQ8	Apoptosis-inducing factor 2 OS=Homo sapiens GN=AIFM2 PE=1 SV=1 - [AIFM2_HUMAN]	1.88	1	1	1	1	0.73	0.72	0.80	0.79					373	40.5	9.11
Q96KQ4	Apoptosis-stimulating of p53 protein 1 OS=Homo sapiens GN=PPP1R13B PE=1 SV=3 - [ASPP1_HUMAN]	1.38	1	1	1	1	2.13	1.62	2.51	1.91					1090	119.5	6.76
O14727	Apoptotic protease-activating factor 1 OS=Homo sapiens GN=APAF1 PE=1 SV=2 - [APAF_HUMAN]	1.92	8	1	3	3					0.85	1.30	0.91	1.38	1248	141.7	6.40
Q8IW19	Aprataxin and PNK-like factor OS=Homo sapiens GN=APLF PE=1 SV=1 - [APLF_HUMAN]	8.41	1	2	2	8	0.95	1.16	0.79	0.97	2.05	1.76	1.94	1.66	511	56.9	5.08
P55087	Aquaporin-4 OS=Homo sapiens GN=AQP4 PE=1 SV=2 - [AQP4_HUMAN]	3.10	1	1	1	1					0.13	0.10	7.00	5.40	323	34.8	7.66
Q13520	Aquaporin-6 OS=Homo sapiens GN=AQP6 PE=2 SV=2 - [AQP6_HUMAN]	5.32	1	1	1	1					0.82	0.58	0.64	0.45	282	29.4	8.66
O75342	Arachidonate 12-lipoxygenase, 12R-type OS=Homo sapiens GN=ALOX12B PE=1 SV=1 - [LX12B_HUMAN]	2.43	1	1	1	18	1.24	1.11	1.30	1.12					701	80.3	7.64
O15296	Arachidonate 15-lipoxygenase B OS=Homo sapiens GN=ALOX15B PE=2 SV=3 - [LX15B_HUMAN]	2.37	1	1	1	1					1.39	1.05	1.34	1.02	676	75.8	6.10
P09917	Arachidonate 5-lipoxygenase OS=Homo sapiens GN=ALOX5 PE=1 SV=2 - [LOX5_HUMAN]	2.23	1	1	1	1	0.37	0.74	0.42	0.85					674	77.9	5.77
Q15057	Arf-GAP with coiled-coil, ANK repeat and PH domain-containing protein 2 OS=Homo sapiens GN=ACAP2 PE=1 SV=3 - [ACAP2_HUMAN]	2.70	2	2	2	6	0.77	0.74	0.66	0.69	0.77	0.89	0.77	0.89	778	88.0	6.80
Q9UPQ3	Arf-GAP with GTPase, ANK repeat and PH domain-containing protein 1 OS=Homo sapiens GN=AGAP1 PE=1 SV=4 - [AGAP1_HUMAN]	1.52	1	1	1	2	0.88	0.88	0.92	0.94	1.19	1.32	1.13	1.25	857	94.4	7.94
Q8TF27	Arf-GAP with GTPase, ANK repeat and PH domain-containing protein 11 OS=Homo sapiens GN=AGAP11 PE=2 SV=2 - [AGA11_HUMAN]	2.36	1	1	1	1					1.45	1.50	1.44	1.49	550	60.5	7.71
Q99490	Arf-GAP with GTPase, ANK repeat and PH domain-containing protein 2 OS=Homo sapiens GN=AGAP2 PE=1 SV=2 - [AGAP2_HUMAN]	1.68	1	1	1	2					0.88	0.89	0.87	0.88	1192	124.6	9.89
Q5SRD3	Arf-GAP with GTPase, ANK repeat and PH domain-containing protein 8 OS=Homo sapiens GN=AGAP8 PE=2 SV=1 - [AGAP8_HUMAN]	4.68	5	1	1	1	0.47	0.62	0.49	0.64					663	73.0	6.79
Q96P48	Arf-GAP with Rho-GAP domain, ANK repeat and PH domain-containing protein 1 OS=Homo sapiens GN=ARAP1 PE=1 SV=3 - [ARAP1_HUMAN]	2.69	1	3	3	10	0.91	0.89	1.01	0.98	0.56	0.55	0.86	0.83	1450	162.1	6.23
Q8TDY4	Arf-GAP with SH3 domain, ANK repeat and PH domain-containing protein 3 OS=Homo sapiens GN=ASAP3 PE=1 SV=1 - [ASAP3_HUMAN]	5.20	1	2	2	3	0.80	0.69	1.03	0.88					903	99.1	6.40
P53365	Arfaptin-2 OS=Homo sapiens GN=ARFIP2 PE=1 SV=1 - [ARFP2_HUMAN]	5.28	1	1	2	187	1.07	1.36	1.44	1.86					341	37.8	6.04

Q7L4I2	Arginine/serine-rich coiled-coil protein 2 OS=Homo sapiens GN=RSRC2 PE=1 SV=1 - [RSRC2_HUMAN]	5.99	1	2	2	7	1.97	2.18	2.12	2.35	0.71	0.27	0.82	0.75	434	50.5	11.33
Q9NVT9	Armadillo repeat-containing protein 1 OS=Homo sapiens GN=ARMC1 PE=1 SV=1 - [ARMC1_HUMAN]	13.48	1	1	1	1	0.98	1.46	0.66	1.00					282	31.3	5.74
Q6NXE6	Armadillo repeat-containing protein 6 OS=Homo sapiens GN=ARMC6 PE=1 SV=2 - [ARMC6_HUMAN]	8.18	1	2	2	2	1.03	0.89	1.02	0.89					501	54.1	6.24
Q9P291	Armadillo repeat-containing X-linked protein 1 OS=Homo sapiens GN=ARMCX1 PE=1 SV=1 - [ARMX1_HUMAN]	6.84	1	2	2	4	1.00	0.95	0.93	0.89					453	49.1	9.22
Q5H9R4	Armadillo repeat-containing X-linked protein 4 OS=Homo sapiens GN=ARMCX4 PE=2 SV=2 - [ARMX4_HUMAN]	5.56	1	1	1	1					1.34	1.37	1.45	1.49	360	39.4	9.19
P20711	Aromatic-L-amino-acid decarboxylase OS=Homo sapiens GN=DDC PE=1 SV=2 - [DDC_HUMAN]	3.33	1	1	1	1	1.25	1.73	1.04	1.43					480	53.9	7.20
Q8TBH0	Arrestin domain-containing protein 2 OS=Homo sapiens GN=ARRDC2 PE=2 SV=2 - [ARRD2_HUMAN]	8.85	1	1	1	1	0.62	0.62	0.72	0.74					407	44.4	9.20
P36575	Arrestin-C OS=Homo sapiens GN=ARR3 PE=1 SV=2 - [ARRC_HUMAN]	4.64	1	1	1	1	0.81	0.85	0.79	0.84					388	42.8	5.77
Q9NZN9	Aryl-hydrocarbon-interacting protein-like 1 OS=Homo sapiens GN=AIPL1 PE=1 SV=2 - [AIPL1_HUMAN]	5.99	2	1	2	4	0.76	0.71	0.73	0.69					384	43.9	5.95
P51690	Arylsulfatase E OS=Homo sapiens GN=ARSE PE=1 SV=2 - [ARSE_HUMAN]	2.55	1	1	1	1					1.26	1.22	1.37	1.32	589	65.6	6.96
Q5FYB1	Arylsulfatase I OS=Homo sapiens GN=ARSI PE=1 SV=1 - [ARSI_HUMAN]	1.05	1	1	1	3	0.69	0.65	0.73	0.67					569	64.0	8.62
P07307	Asialoglycoprotein receptor 2 OS=Homo sapiens GN=ASGR2 PE=1 SV=2 - [ASGR2_HUMAN]	15.11	1	3	3	5	1.54	1.48	1.57	1.53	0.53	0.97	0.84	1.53	311	35.1	6.25
P17174	Aspartate aminotransferase, cytoplasmic OS=Homo sapiens GN=GOT1 PE=1 SV=3 - [AATC_HUMAN]	9.69	7	3	4	11	0.83	0.80	0.84	0.76					413	46.2	7.01
P00505	Aspartate aminotransferase, mitochondrial OS=Homo sapiens GN=GOT2 PE=1 SV=3 - [AATM_HUMAN]	4.19	1	1	1	1					1.99	1.84	1.66	1.53	430	47.5	9.01
Q5U4P2	Aspartate beta-hydroxylase domain-containing protein 1 OS=Homo sapiens GN=ASPHD1 PE=1 SV=3 - [ASPH1_HUMAN]	17.95	1	2	2	16	1.05	0.95	0.96	0.88	0.73	0.68	1.06	0.98	390	41.1	8.56
P14868	Aspartate--tRNA ligase, cytoplasmic OS=Homo sapiens GN=DARS PE=1 SV=2 - [SYDC_HUMAN]	6.19	1	2	2	4	1.44	1.42	1.41	1.39	1.30	1.40	1.32	1.42	501	57.1	6.55
Q9ULA0	Aspartyl aminopeptidase OS=Homo sapiens GN=DNPEP PE=1 SV=1 - [DNPEP_HUMAN]	3.37	1	1	1	1	0.85	0.96	0.90	1.03					475	52.4	7.42
Q9BXN1	Asporin OS=Homo sapiens GN=ASPN PE=1 SV=2 - [ASPN_HUMAN]	2.89	1	1	1	1	0.80	0.97	0.94	1.15					380	43.4	7.08
O75129	Astrotactin-2 OS=Homo sapiens GN=ASTN2 PE=2 SV=2 - [ASTN2_HUMAN]	1.19	1	1	1	1					0.52	1.69	0.20	0.67	1339	148.1	5.97
Q7Z591	AT-hook-containing transcription factor OS=Homo sapiens GN=AKNA PE=1 SV=2 - [AKNA_HUMAN]	2.78	1	2	2	5	1.29	1.26	1.29	1.25					1439	155.0	6.32
O14497	AT-rich interactive domain-containing protein 1A OS=Homo sapiens GN=ARID1A PE=1 SV=3 - [ARI1A_HUMAN]	0.44	1	1	1	2					0.73	0.87	0.67	0.80	2285	241.9	6.70
Q8NFD5	AT-rich interactive domain-containing protein 1B OS=Homo sapiens GN=ARID1B PE=1 SV=2 - [ARI1B_HUMAN]	3.09	1	2	2	3	0.81	0.85	0.73	0.76					2236	236.0	6.73
Q68CP9	AT-rich interactive domain-containing protein 2 OS=Homo sapiens GN=ARID2 PE=1 SV=2 - [ARID2_HUMAN]	1.14	1	1	1	1					1.18	1.34	0.99	1.12	1835	197.3	7.42
Q99856	AT-rich interactive domain-containing protein 3A OS=Homo sapiens GN=ARID3A PE=1 SV=2 - [ARI3A_HUMAN]	3.88	2	2	2	2	0.94	1.01	1.26	1.35	1.04	1.24	0.80	0.95	593	62.9	4.91
P29374	AT-rich interactive domain-containing protein 4A OS=Homo sapiens GN=ARID4A PE=1 SV=3 - [ARI4A_HUMAN]	2.47	1	2	2	11	1.00	1.20	1.11	1.30					1257	142.7	5.10
Q14865	AT-rich interactive domain-containing protein 5B OS=Homo sapiens GN=ARID5B PE=1 SV=3 - [ARI5B_HUMAN]	1.77	1	2	2	2	1.09	1.08	1.05	1.05	1.15	0.87	1.13	0.85	1188	132.3	8.72
P0C7T5	Ataxin-1-like OS=Homo sapiens GN=ATXN1L PE=1 SV=1 - [ATX1L_HUMAN]	7.69	1	2	2	2	1.15	1.15	1.17	1.16	1.05	0.94	1.01	0.90	689	73.3	6.60

Q9UBB4	Ataxin-10 OS=Homo sapiens GN=ATXN10 PE=1 SV=1 - [ATX10_HUMAN]	3.16	1	1	1	5					1.07	1.20	1.09	1.22	475	53.5	5.25
O15265	Ataxin-7 OS=Homo sapiens GN=ATXN7 PE=1 SV=1 - [ATX7_HUMAN]	5.94	1	2	2	3	0.92	0.83	1.00	0.90	1.28	1.23	1.39	1.34	892	95.4	9.85
Q9ULK2	Ataxin-7-like protein 1 OS=Homo sapiens GN=ATXN7L1 PE=2 SV=3 - [AT7L1_HUMAN]	1.86	1	2	2	2	0.85	0.75	1.15	1.02	1.02	1.86	0.87	1.59	861	91.5	9.79
O95477	ATP-binding cassette sub-family A member 1 OS=Homo sapiens GN=ABCA1 PE=1 SV=3 - [ABCA1_HUMAN]	1.06	2	2	4	47					4.24	3.40	0.98	0.75	2261	254.1	6.86
Q86UK0	ATP-binding cassette sub-family A member 12 OS=Homo sapiens GN=ABCA12 PE=1 SV=3 - [ABCAC_HUMAN]	1.43	1	1	2	4					0.87	0.55	0.87	0.78	2595	293.0	7.75
Q86UQ4	ATP-binding cassette sub-family A member 13 OS=Homo sapiens GN=ABCA13 PE=2 SV=3 - [ABCAD_HUMAN]	0.69	1	2	2	2					1.52	2.02	1.33	1.78	5058	575.8	6.46
Q8IZY2	ATP-binding cassette sub-family A member 7 OS=Homo sapiens GN=ABCA7 PE=1 SV=3 - [ABCA7_HUMAN]	0.79	1	1	1	3	0.55	0.57	0.41	0.44	0.60	0.60	0.74	0.74	2146	234.2	7.24
O94911	ATP-binding cassette sub-family A member 8 OS=Homo sapiens GN=ABCA8 PE=1 SV=3 - [ABCA8_HUMAN]	0.82	7	1	2	6					0.16	3.82	0.35	8.41	1581	179.1	7.18
Q8IUA7	ATP-binding cassette sub-family A member 9 OS=Homo sapiens GN=ABCA9 PE=1 SV=1 - [ABCA9_HUMAN]	0.80	1	1	1	1					0.88	0.12	0.99	0.13	1624	184.2	6.93
Q2M3G0	ATP-binding cassette sub-family B member 5 OS=Homo sapiens GN=ABCB5 PE=1 SV=3 - [ABCB5_HUMAN]	5.54	2	2	3	4					0.99	1.04	0.93	0.98	812	89.8	7.94
O75027	ATP-binding cassette sub-family B member 7, mitochondrial OS=Homo sapiens GN=ABCB7 PE=1 SV=2 - [ABCB7_HUMAN]	2.66	1	1	1	2	1.08	1.44	1.17	1.57	1.47	1.06	0.85	0.61	752	82.6	9.33
Q9NP78	ATP-binding cassette sub-family B member 9 OS=Homo sapiens GN=ABCB9 PE=1 SV=1 - [ABCB9_HUMAN]	3.13	2	1	3	9	1.45	1.34	1.41	1.32	1.34	1.99	1.17	1.73	766	84.4	7.93
Q96J66	ATP-binding cassette sub-family C member 11 OS=Homo sapiens GN=ABCC11 PE=1 SV=1 - [ABCCB_HUMAN]	1.01	1	1	1	1	1.02	1.43	0.68	0.96					1382	154.2	8.09
O60706	ATP-binding cassette sub-family C member 9 OS=Homo sapiens GN=ABCC9 PE=1 SV=2 - [ABCC9_HUMAN]	3.03	4	3	3	6	1.73	1.26	1.29	1.02					1549	174.1	7.37
P33897	ATP-binding cassette sub-family D member 1 OS=Homo sapiens GN=ABCD1 PE=1 SV=2 - [ABCD1_HUMAN]	2.68	1	1	1	1	0.18	0.56	0.27	0.83					745	82.9	8.95
P28288	ATP-binding cassette sub-family D member 3 OS=Homo sapiens GN=ABCD3 PE=1 SV=1 - [ABCD3_HUMAN]	4.70	1	2	2	5	0.97	1.03	0.96	1.03	0.94	0.47	0.78	0.39	659	75.4	9.36
P53396	ATP-citrate synthase OS=Homo sapiens GN=ACLY PE=1 SV=3 - [ACLY_HUMAN]	1.27	1	1	1	1	0.71	0.67	0.76	0.72					1101	120.8	7.33
O76031	ATP-dependent Clp protease ATP-binding subunit clpX-like, mitochondrial OS=Homo sapiens GN=CLPX PE=1 SV=2 - [CLPX_HUMAN]	4.74	1	2	2	2	0.85	0.91	0.86	0.93					633	69.2	7.58
Q9H611	ATP-dependent DNA helicase PIF1 OS=Homo sapiens GN=PIF1 PE=1 SV=2 - [PIF1_HUMAN]	3.90	1	1	2	2					1.27	1.76	1.99	2.77	641	69.8	9.72
O94761	ATP-dependent DNA helicase Q4 OS=Homo sapiens GN=RECQL4 PE=1 SV=1 - [RECQ4_HUMAN]	0.99	1	1	1	1					1.55		2.34		1208	133.0	8.09
O94762	ATP-dependent DNA helicase Q5 OS=Homo sapiens GN=RECQL5 PE=1 SV=2 - [RECQ5_HUMAN]	1.21	1	1	1	1							0.97	0.43	991	108.8	8.56
Q9NUU7	ATP-dependent RNA helicase DDX19A OS=Homo sapiens GN=DDX19A PE=1 SV=1 - [DD19A_HUMAN]	5.02	2	1	1	1	1.11	1.18	1.04	1.10					478	53.9	6.58
O00571	ATP-dependent RNA helicase DDX3X OS=Homo sapiens GN=DDX3X PE=1 SV=3 - [DDX3X_HUMAN]	5.89	2	3	3	4	0.72	0.72	0.75	0.74					662	73.2	7.18
Q8N8A6	ATP-dependent RNA helicase DDX51 OS=Homo sapiens GN=DDX51 PE=1 SV=3 - [DDX51_HUMAN]	1.20	1	1	1	1					0.85	0.98	0.68	0.78	666	72.4	8.16
Q8TDD1	ATP-dependent RNA helicase DDX54 OS=Homo sapiens GN=DDX54 PE=1 SV=2 - [DDX54_HUMAN]	1.82	1	1	1	1					1.06	1.11	1.04	1.09	881	98.5	10.02
Q7Z478	ATP-dependent RNA helicase DHX29 OS=Homo sapiens GN=DHX29 PE=1 SV=2 - [DHX29_HUMAN]	0.66	1	1	1	5					0.85	0.70	1.08	0.89	1369	155.1	8.09
Q14562	ATP-dependent RNA helicase DHX8 OS=Homo sapiens GN=DHX8 PE=1 SV=1 - [DHX8_HUMAN]	1.56	1	1	1	1	0.93	0.74	0.86	0.69					1220	139.2	8.32

Q8IYB8	ATP-dependent RNA helicase SUPV3L1, mitochondrial OS=Homo sapiens GN=SUPV3L1 PE=1 SV=1 - [SUV3_HUMAN]	2.04	1	1	1	1	1.69	1.86	1.77	1.97					786	87.9	7.99
Q96TA2	ATP-dependent zinc metalloprotease YME1L1 OS=Homo sapiens GN=YME1L1 PE=1 SV=2 - [YMEL1_HUMAN]	5.69	1	2	2	2	0.92	0.91	0.86	0.85					773	86.4	8.76
P78508	ATP-sensitive inward rectifier potassium channel 10 OS=Homo sapiens GN=KCNJ10 PE=1 SV=1 - [IRK10_HUMAN]	3.17	1	1	1	2					0.28	0.13	0.22	0.11	379	42.5	8.06
Q99712	ATP-sensitive inward rectifier potassium channel 15 OS=Homo sapiens GN=KCNJ15 PE=1 SV=2 - [IRK15_HUMAN]	3.73	1	1	1	1					0.76	0.75	0.90	0.89	375	42.6	7.69
Q15842	ATP-sensitive inward rectifier potassium channel 8 OS=Homo sapiens GN=KCNJ8 PE=1 SV=1 - [IRK8_HUMAN]	3.54	1	1	1	1					1.34	1.35	1.30	1.31	424	47.9	9.26
Q6PL18	ATPase family AAA domain-containing protein 2 OS=Homo sapiens GN=ATAD2 PE=1 SV=1 - [ATAD2_HUMAN]	1.73	1	1	1	1					0.87	0.85	1.04	1.01	1390	158.5	6.32
Q9ULI0	ATPase family AAA domain-containing protein 2B OS=Homo sapiens GN=ATAD2B PE=1 SV=3 - [ATD2B_HUMAN]	5.14	1	3	3	6					0.80	0.65	1.14	0.91	1458	164.8	6.80
Q96QE3	ATPase family AAA domain-containing protein 5 OS=Homo sapiens GN=ATAD5 PE=1 SV=4 - [ATAD5_HUMAN]	1.74	1	2	2	3	0.23	0.23	0.25	0.25	1.41	1.80	1.35	1.85	1844	207.4	9.19
P20594	Atrial natriuretic peptide receptor 2 OS=Homo sapiens GN=NPR2 PE=1 SV=1 - [ANPRB_HUMAN]	0.96	1	1	1	1	0.91	0.66	0.87	0.63					1047	116.9	6.87
O75882	Attractin OS=Homo sapiens GN=ATRN PE=1 SV=2 - [ATRN_HUMAN]	23.65	1	26	27	227	0.99	0.96	1.01	1.00	0.90	0.87	0.96	0.95	1429	158.4	7.31
O75143	Autophagy-related protein 13 OS=Homo sapiens GN=ATG13 PE=1 SV=1 - [ATG13_HUMAN]	1.74	1	1	1	1					0.78	0.66	0.81	0.68	517	56.5	5.12
Q96BY7	Autophagy-related protein 2 homolog B OS=Homo sapiens GN=ATG2B PE=1 SV=5 - [ATG2B_HUMAN]	1.30	1	1	1	1					0.94	0.80	0.85	0.72	2078	232.6	5.76
Q674R7	Autophagy-related protein 9B OS=Homo sapiens GN=ATG9B PE=2 SV=1 - [ATG9B_HUMAN]	2.49	1	1	2	2	1.35	1.07	1.69	1.34					924	101.0	8.28
Q9Y2T1	Axin-2 OS=Homo sapiens GN=AXIN2 PE=1 SV=1 - [AXIN2_HUMAN]	4.15	1	2	2	2	0.36	0.33	0.57	0.52					843	93.5	7.78
O14645	Axonemal dynein light intermediate polypeptide 1 OS=Homo sapiens GN=DNALI1 PE=2 SV=2 - [IDLC_HUMAN]	16.28	1	3	3	4	1.59	1.44	1.62	1.48	0.96	0.79	0.84	0.69	258	29.6	8.50
P20160	Azurocidin OS=Homo sapiens GN=AZU1 PE=1 SV=3 - [CAP7_HUMAN]	13.55	1	3	3	17	0.91	0.90	0.90	0.90	0.94	0.84	0.98	0.95	251	26.9	9.50
P11912	B-cell antigen receptor complex-associated protein alpha chain OS=Homo sapiens GN=CD79A PE=1 SV=2 - [CD79A_HUMAN]	6.64	1	1	1	1	0.75	0.59	1.67	1.32					226	25.0	5.00
Q8WUZ0	B-cell CLL/lymphoma 7 protein family member C OS=Homo sapiens GN=BCL7C PE=1 SV=3 - [BCL7C_HUMAN]	3.69	1	1	1	1					0.71	0.90	0.67	0.85	217	23.5	5.20
O00512	B-cell CLL/lymphoma 9 protein OS=Homo sapiens GN=BCL9 PE=1 SV=4 - [BCL9_HUMAN]	2.66	1	2	2	6	0.75	0.76	0.96	0.97	0.92	1.32	0.76	1.10	1426	149.2	8.91
P20273	B-cell receptor CD22 OS=Homo sapiens GN=CD22 PE=1 SV=2 - [CD22_HUMAN]	1.42	1	1	1	3	0.87	1.03	0.63	0.75					847	95.3	6.65
P17213	Bactericidal permeability-increasing protein OS=Homo sapiens GN=BPI PE=1 SV=4 - [BPI_HUMAN]	2.26	1	1	1	1		0.95							487	53.9	9.38
Q13075	Baculoviral IAP repeat-containing protein 1 OS=Homo sapiens GN=NAIP PE=1 SV=3 - [BIRC1_HUMAN]	0.93	1	1	1	1	1.03	0.92	1.12	1.01					1403	159.5	5.99
Q13490	Baculoviral IAP repeat-containing protein 2 OS=Homo sapiens GN=BIRC2 PE=1 SV=2 - [BIRC2_HUMAN]	3.07	1	1	1	1					0.40	0.60	0.59	0.89	618	69.9	6.70
Q9NR09	Baculoviral IAP repeat-containing protein 6 OS=Homo sapiens GN=BIRC6 PE=1 SV=2 - [BIRC6_HUMAN]	0.31	1	1	1	1	1.50		1.05						4857	529.9	6.05
O94812	BAI1-associated protein 3 OS=Homo sapiens GN=BAIAP3 PE=1 SV=2 - [BAIP3_HUMAN]	1.10	1	1	2	6	0.83	0.88	0.83	0.89					1187	131.8	6.37
P02730	Band 3 anion transport protein OS=Homo sapiens GN=SLC4A1 PE=1 SV=3 - [B3AT_HUMAN]	8.67	1	5	5	8	1.60	1.66	1.40	1.50					911	101.7	5.19
Q9H329	Band 4.1-like protein 4B OS=Homo sapiens GN=EPB41L4B PE=2 SV=2 - [E41LB_HUMAN]	0.67	1	1	1	1					1.05	1.10	1.06	1.11	900	99.7	9.04

Q9HCM4	Band 4.1-like protein 5 OS=Homo sapiens GN=EPB41L5 PE=1 SV=3 - [E41L5_HUMAN]	3.82	10	2	2	2					1.35	2.58	1.14	2.18	733	81.8	6.58
Q8TAM1	Bardet-Biedl syndrome 10 protein OS=Homo sapiens GN=BBS10 PE=1 SV=2 - [BBS10_HUMAN]	2.49	1	1	1	10	1.30	0.99	1.39	1.05	1.10	1.16	1.08	1.32	723	80.8	7.77
Q8N3I7	Bardet-Biedl syndrome 5 protein OS=Homo sapiens GN=BBS5 PE=1 SV=1 - [BBS5_HUMAN]	4.11	1	1	1	1	1.29	1.42	1.37	1.52					341	38.7	5.60
Q8IWZ6	Bardet-Biedl syndrome 7 protein OS=Homo sapiens GN=BBS7 PE=1 SV=2 - [BBS7_HUMAN]	1.82	1	1	1	1					1.06	1.18	0.86	0.96	715	80.3	6.05
O75531	Barrier-to-autointegration factor OS=Homo sapiens GN=BANF1 PE=1 SV=1 - [BAF_HUMAN]	7.87	1	1	1	2					1.92	1.75	1.77	1.61	89	10.1	6.09
P98160	Basement membrane-specific heparan sulfate proteoglycan core protein OS=Homo sapiens GN=HSPG2 PE=1 SV=4 - [PGBM_HUMAN]	6.01	1	20	20	63	0.96	0.95	0.97	1.04	0.95	0.98	0.91	1.00	4391	468.5	6.51
Q68DE3	Basic helix-loop-helix domain-containing protein KIAA2018 OS=Homo sapiens GN=KIAA2018 PE=1 SV=3 - [K2018_HUMAN]	0.45	1	1	1	2	1.16	1.30	1.15	1.30					2245	241.5	7.61
Q7L1Q6	Basic leucine zipper and W2 domain-containing protein 1 OS=Homo sapiens GN=BZW1 PE=1 SV=1 - [BZW1_HUMAN]	6.21	1	1	1	2	0.64	0.67	0.82	0.88					419	48.0	5.92
P35613	Basigin OS=Homo sapiens GN=BSG PE=1 SV=2 - [BASI_HUMAN]	2.34	1	1	1	1	0.96	1.06	0.92	1.02					385	42.2	5.66
Q5TBC7	Bcl-2-like protein 15 OS=Homo sapiens GN=BCL2L15 PE=2 SV=1 - [B2L15_HUMAN]	9.20	1	1	1	1	0.99	1.01	0.96	1.00					163	17.7	4.41
Q6W2J9	BCL-6 corepressor OS=Homo sapiens GN=BCOR PE=1 SV=1 - [BCOR_HUMAN]	1.94	1	2	2	3	0.70	0.96	1.21	1.67					1755	192.1	6.48
Q5H9F3	BCL-6 corepressor-like protein 1 OS=Homo sapiens GN=BCORL1 PE=1 SV=1 - [BCORL_HUMAN]	1.81	1	1	1	1					0.97	0.86	1.05	0.93	1711	182.4	7.24
Q16620	BDNF/NT-3 growth factors receptor OS=Homo sapiens GN=NTRK2 PE=1 SV=1 - [NTRK2_HUMAN]	1.82	1	1	1	1	0.87	0.89	0.91	0.94					822	91.9	6.47
Q14457	Beclin-1 OS=Homo sapiens GN=BECN1 PE=1 SV=2 - [BECN1_HUMAN]	11.33	1	2	2	2	1.30	1.44	1.26	1.39	0.44	0.64	1.21	1.78	450	51.9	4.89
A8MW95	Beclin-1-like protein 1 OS=Homo sapiens GN=BECN1L1 PE=3 SV=1 - [BCN1L_HUMAN]	3.40	1	1	1	3	0.65	0.74	0.68	0.79					441	49.0	4.88
Q7L4P6	BEN domain-containing protein 5 OS=Homo sapiens GN=BEND5 PE=1 SV=1 - [BEND5_HUMAN]	3.09	1	1	1	1	1.26	1.08	1.27	1.09					421	48.2	6.20
Q5SZJ8	BEN domain-containing protein 6 OS=Homo sapiens GN=BEND6 PE=2 SV=2 - [BEND6_HUMAN]	6.81	1	1	1	1	1.28	1.67	1.49	1.96					279	31.2	8.35
O76090	Bestrophin-1 OS=Homo sapiens GN=BEST1 PE=1 SV=1 - [BEST1_HUMAN]	2.56	1	2	2	2					1.13	1.29	0.99	1.13	585	67.6	6.90
Q8N1M1	Bestrophin-3 OS=Homo sapiens GN=BEST3 PE=2 SV=1 - [BEST3_HUMAN]	1.35	1	1	1	1	0.37	0.39	0.34	0.37					668	76.1	6.58
Q8NFU0	Bestrophin-4 OS=Homo sapiens GN=BEST4 PE=2 SV=1 - [BEST4_HUMAN]	3.81	1	1	1	1	0.98	1.11	1.13	1.29					473	53.5	6.07
Q13884	Beta-1-syntrophin OS=Homo sapiens GN=SNTB1 PE=1 SV=3 - [SNTB1_HUMAN]	2.04	1	1	1	2	1.13	0.61	1.96	1.07	1.26	1.38	1.29	1.41	538	58.0	8.63
Q96L58	Beta-1,3-galactosyltransferase 6 OS=Homo sapiens GN=B3GALT6 PE=2 SV=2 - [B3GT6_HUMAN]	8.21	1	2	2	2	1.23	0.97	1.34	1.06	0.59	0.74	0.75	0.93	329	37.1	9.66
Q6Y288	Beta-1,3-glucosyltransferase OS=Homo sapiens GN=B3GALTL PE=1 SV=2 - [B3GLT_HUMAN]	1.61	1	1	1	1	0.97	0.33	0.91	0.31					498	56.5	7.50
P15291	Beta-1,4-galactosyltransferase 1 OS=Homo sapiens GN=B4GALT1 PE=1 SV=5 - [B4GT1_HUMAN]	2.76	1	1	1	2	1.33	1.32	1.25	1.25					398	43.9	8.65
O43286	Beta-1,4-galactosyltransferase 5 OS=Homo sapiens GN=B4GALT5 PE=2 SV=1 - [B4GT5_HUMAN]	2.58	1	1	1	2	0.67	0.67	0.66	0.67					388	45.1	8.15
Q09327	Beta-1,4-mannosyl-glycoprotein 4-beta-N-acetylglucosaminyltransferase OS=Homo sapiens GN=MGAT3 PE=2 SV=3 - [MGAT3_HUMAN]	3.56	1	1	1	1					2.45				533	61.3	8.28
P02749	Beta-2-glycoprotein 1 OS=Homo sapiens GN=APOH PE=1 SV=3 - [APOH_HUMAN]	72.17	1	24	24	1085	1.16	1.09	1.15	1.10	1.08	1.11	1.11	1.09	345	38.3	7.97

P61769	Beta-2-microglobulin OS=Homo sapiens GN=B2M PE=1 SV=1 - [B2MG_HUMAN]	48.74	1	6	6	67	0.85	0.86	0.80	0.80	1.14	1.19	1.18	1.29	119	13.7	6.52
Q13425	Beta-2-syntrophin OS=Homo sapiens GN=SNTB2 PE=1 SV=1 - [SNTB2_HUMAN]	0.93	1	1	1	1					0.17	1.87	0.53	5.70	540	57.9	8.82
Q562R1	Beta-actin-like protein 2 OS=Homo sapiens GN=ACTBL2 PE=1 SV=2 - [ACTBL_HUMAN]	21.01	2	2	7	96	0.71	0.86	0.74	0.89					376	42.0	5.59
P35612	Beta-adducin OS=Homo sapiens GN=ADD2 PE=1 SV=3 - [ADDB_HUMAN]	2.34	1	1	1	1						1.25			726	80.8	5.92
P25098	Beta-adrenergic receptor kinase 1 OS=Homo sapiens GN=ADRBK1 PE=1 SV=2 - [ARBK1_HUMAN]	0.87	1	1	1	1	0.56	0.53	0.66	0.62					689	79.5	7.28
Q96KN2	Beta-Ala-His dipeptidase OS=Homo sapiens GN=CNDP1 PE=1 SV=4 - [CNDP1_HUMAN]	49.31	1	21	21	200	1.21	1.19	1.17	1.20	1.26	1.43	1.25	1.37	507	56.7	5.30
Q6UWU2	Beta-galactosidase-1-like protein OS=Homo sapiens GN=GLB1L PE=2 SV=1 - [GLB1L_HUMAN]	3.52	2	2	2	4	0.95	1.03	1.05	1.14	1.19	1.16	1.14	1.12	654	74.1	8.92
P15907	Beta-galactoside alpha-2,6-sialyltransferase 1 OS=Homo sapiens GN=ST6GAL1 PE=1 SV=1 - [SIAT1_HUMAN]	2.46	1	1	1	2	1.35	1.10	1.33	1.10					406	46.6	9.01
P08236	Beta-glucuronidase OS=Homo sapiens GN=GUSB PE=1 SV=2 - [BGLR_HUMAN]	2.61	1	2	2	2	0.99	1.07	1.36	1.48	0.81	1.15	0.85	1.20	651	74.7	7.02
P07686	Beta-hexosaminidase subunit beta OS=Homo sapiens GN=HEXB PE=1 SV=3 - [HEXB_HUMAN]	1.80	1	1	1	2	1.17	1.16	1.30	1.30					556	63.1	6.76
O00462	Beta-mannosidase OS=Homo sapiens GN=MANBA PE=1 SV=3 - [MANBA_HUMAN]	2.73	1	2	2	4	1.45	1.55	1.54	1.65	2.06	2.09	2.01	2.04	879	100.8	5.52
Q9HBI1	Beta-parvin OS=Homo sapiens GN=PARVB PE=1 SV=1 - [PARVB_HUMAN]	15.66	2	5	5	9	0.66	0.83	0.75	0.83					364	41.7	6.73
Q9H115	Beta-soluble NSF attachment protein OS=Homo sapiens GN=NAPB PE=1 SV=2 - [SNAB_HUMAN]	9.73	1	2	2	2	0.78	0.92	0.83	0.99	0.88	1.03	0.86	1.01	298	33.5	5.47
Q8N3L3	Beta-taxilin OS=Homo sapiens GN=TXLNB PE=1 SV=3 - [TXLNB_HUMAN]	2.78	1	1	1	1					0.93	1.23	0.99	1.30	684	76.5	4.93
Q96PL2	Beta-tectorin OS=Homo sapiens GN=TECTB PE=2 SV=1 - [TECTB_HUMAN]	7.60	1	1	1	2					1.33	2.99	0.40	0.90	329	36.9	8.05
Q9HAY6	Beta,beta-carotene 15,15'-monooxygenase OS=Homo sapiens GN=BCMO1 PE=1 SV=1 - [BCDO1_HUMAN]	2.01	1	1	1	1					0.85	0.81	0.86	0.82	547	62.6	6.67
Q9BYV7	Beta,beta-carotene 9',10'-oxygenase OS=Homo sapiens GN=BCO2 PE=1 SV=5 - [BCDO2_HUMAN]	2.42	1	1	1	1	1.30	0.85	1.31	0.86					579	65.6	8.41
Q93088	Betaine--homocysteine S-methyltransferase 1 OS=Homo sapiens GN=BHMT PE=1 SV=2 - [BHMT1_HUMAN]	2.22	1	1	1	2	1.09	0.85	1.11	0.87					406	45.0	7.03
A1A5D9	Bicaudal D-related protein 2 OS=Homo sapiens GN=CCDC64B PE=2 SV=2 - [BICR2_HUMAN]	2.36	1	1	1	3					0.76	0.72	0.54	0.51	508	56.8	5.02
O43252	Bifunctional 3'-phosphoadenosine 5'-phosphosulfate synthase 1 OS=Homo sapiens GN=PAPSS1 PE=1 SV=2 - [PAPS1_HUMAN]	2.56	1	1	1	14	1.15	1.18	1.23	1.17	0.67	0.88	0.92	1.21	624	70.8	6.86
Q3LXA3	Bifunctional ATP-dependent dihydroxyacetone kinase/FAD-AMP lyase (cyclizing) OS=Homo sapiens GN=DAK PE=1 SV=2 - [DHAK_HUMAN]	2.78	1	1	1	1	1.85	1.25	1.73	1.19					575	58.9	7.49
Q13057	Bifunctional coenzyme A synthase OS=Homo sapiens GN=COASY PE=1 SV=4 - [COASY_HUMAN]	3.01	1	1	1	5	2.02	1.36	2.63	1.77					564	62.3	6.99
P52848	Bifunctional heparan sulfate N-deacetylase/N-sulfotransferase 1 OS=Homo sapiens GN=NDST1 PE=1 SV=1 - [NDST1_HUMAN]	0.79	1	1	1	12	1.04	1.18	0.94	1.06	1.38	1.01	1.27	1.00	882	100.8	7.97
O95803	Bifunctional heparan sulfate N-deacetylase/N-sulfotransferase 3 OS=Homo sapiens GN=NDST3 PE=2 SV=1 - [NDST3_HUMAN]	1.60	1	1	1	1					1.03	0.78	1.15	0.87	873	100.8	8.06
P13995	Bifunctional methylenetetrahydrofolate dehydrogenase/cyclohydrolase, mitochondrial OS=Homo sapiens GN=MTHFD2 PE=1 SV=2 - [MTDC_HUMAN]	3.43	1	1	1	1					0.82	0.86	0.90	0.94	350	37.9	8.73
O60502	Bifunctional protein NCOAT OS=Homo sapiens GN=MGEA5 PE=1 SV=2 - [NCOAT_HUMAN]	1.42	1	1	1	1					1.17	1.01	0.85	0.73	916	102.8	4.91
P31939	Bifunctional purine biosynthesis protein PURH OS=Homo sapiens GN=ATIC PE=1 SV=3 - [PUR9_HUMAN]	2.20	1	1	1	13	0.83	0.95	0.83	0.95	1.13	0.86	1.15	0.87	592	64.6	6.71

P21810	Biglycan OS=Homo sapiens GN=BGN PE=1 SV=2 - [PGS1_HUMAN]	5.43	1	2	2	3	1.18	1.11	1.13	1.07	1.74	1.63	1.70	1.60	368	41.6	7.52
Q9Y2P5	Bile acyl-CoA synthetase OS=Homo sapiens GN=SLC27A5 PE=1 SV=1 - [S27A5_HUMAN]	2.61	1	1	1	1					0.94	0.86	0.96	0.88	690	75.3	7.66
O95342	Bile salt export pump OS=Homo sapiens GN=ABCB11 PE=1 SV=2 - [ABCBB_HUMAN]	0.91	1	1	1	1					0.99	0.94	0.90	0.85	1321	146.3	6.57
Q8NFC6	Biorientation of chromosomes in cell division protein 1-like OS=Homo sapiens GN=BOD1L PE=1 SV=2 - [BOD1L_HUMAN]	0.46	1	1	1	1					1.21	1.55	1.03	1.32	3051	330.3	5.08
P43251	Biotinidase OS=Homo sapiens GN=BTD PE=1 SV=2 - [BTD_HUMAN]	17.13	1	8	8	56	0.95	1.03	0.92	0.97	1.10	1.19	1.07	1.11	543	61.1	6.25
Q13867	Bleomycin hydrolase OS=Homo sapiens GN=BLMH PE=1 SV=1 - [BLMH_HUMAN]	4.40	1	1	1	2					0.98	1.12	0.99	1.13	455	52.5	6.27
Q8NE79	Blood vessel epicardial substance OS=Homo sapiens GN=BVES PE=1 SV=1 - [POPD1_HUMAN]	1.39	1	1	1	1					0.60	0.80	0.75	0.99	360	41.4	7.90
P54132	Bloom syndrome protein OS=Homo sapiens GN=BLM PE=1 SV=1 - [BLM_HUMAN]	1.48	1	1	1	2	1.06	1.48	1.23	1.71					1417	158.9	7.49
Q6UWU4	Bombesin receptor-activated protein C6orf89 OS=Homo sapiens GN=C6orf89 PE=1 SV=1 - [CF089_HUMAN]	3.17	1	1	1	1	1.46	1.43	1.51	1.50					347	39.8	6.83
P13727	Bone marrow proteoglycan OS=Homo sapiens GN=PRG2 PE=1 SV=2 - [PRG2_HUMAN]	17.12	1	5	5	20	2.29	2.50	2.31	2.55	1.50	1.88	1.66	1.99	222	25.2	6.76
P13497	Bone morphogenetic protein 1 OS=Homo sapiens GN=BMP1 PE=1 SV=2 - [BMP1_HUMAN]	2.84	1	2	2	3					1.02	1.06	1.04	1.10	986	111.2	6.90
P55107	Bone morphogenetic protein 3B OS=Homo sapiens GN=GDF10 PE=1 SV=1 - [BMP3B_HUMAN]	3.14	1	1	1	1	1.04	0.89	0.97	0.84					478	53.1	9.51
P36894	Bone morphogenetic protein receptor type-1A OS=Homo sapiens GN=BMPR1A PE=1 SV=2 - [BMR1A_HUMAN]	2.82	1	2	2	2	1.32	1.10	1.05	0.88					532	60.2	7.58
Q9BQP9	BPI fold-containing family A member 3 OS=Homo sapiens GN=BPIFA3 PE=2 SV=3 - [BPIA3_HUMAN]	11.42	1	1	1	3			1.37						254	28.4	6.65
Q8TDL5	BPI fold-containing family B member 1 OS=Homo sapiens GN=BPIFB1 PE=2 SV=1 - [BPIB1_HUMAN]	10.54	2	5	5	14	1.10	0.96	1.27	1.16					484	52.4	7.23
Q8NFQ6	BPI fold-containing family C protein OS=Homo sapiens GN=BPIFC PE=1 SV=1 - [BPIFC_HUMAN]	7.10	1	1	3	5	0.89	0.88	0.91	0.90					507	56.4	5.82
P80723	Brain acid soluble protein 1 OS=Homo sapiens GN=BASP1 PE=1 SV=2 - [BASP1_HUMAN]	31.72	1	4	4	20	0.89	0.95	1.13	1.12	1.19	1.14	0.98	0.99	227	22.7	4.63
P23560	Brain-derived neurotrophic factor OS=Homo sapiens GN=BDNF PE=1 SV=1 - [BDNF_HUMAN]	13.77	1	2	2	4					0.99	1.52	1.12	1.71	247	27.8	8.79
Q9BUH8	Brain-enriched guanylate kinase-associated protein OS=Homo sapiens GN=BEGAIN PE=1 SV=1 - [BEGIN_HUMAN]	1.69	1	1	1	1	1.05	1.05	1.20	1.21					593	64.8	5.57
Q6UXY1	Brain-specific angiogenesis inhibitor 1-associated protein 2-like protein 2 OS=Homo sapiens GN=BAIAP2L2 PE=1 SV=1 - [BI2L2_HUMAN]	3.59	1	1	1	1	1.00	1.08	1.05	1.14					529	58.9	9.51
O60241	Brain-specific angiogenesis inhibitor 2 OS=Homo sapiens GN=BAI2 PE=2 SV=2 - [BAI2_HUMAN]	1.14	1	1	1	2					0.19	0.10	0.63	0.34	1585	172.5	7.42
Q9NXR7	BRCA1-A complex subunit BRE OS=Homo sapiens GN=BRE PE=1 SV=2 - [BRE_HUMAN]	3.39	1	1	1	1					1.04	0.77	1.03	0.76	383	43.5	5.81
Q96RL1	BRCA1-A complex subunit RAP80 OS=Homo sapiens GN=UIMC1 PE=1 SV=2 - [UIMC1_HUMAN]	1.95	1	1	1	2					0.94	0.78	0.73	0.60	719	79.7	5.45
Q6PJG6	BRCA1-associated ATM activator 1 OS=Homo sapiens GN=BRAT1 PE=1 SV=2 - [BRAT1_HUMAN]	4.99	1	1	1	1	1.21	1.09	1.08	1.00					821	88.1	5.27
Q99728	BRCA1-associated RING domain protein 1 OS=Homo sapiens GN=BARD1 PE=1 SV=2 - [BARD1_HUMAN]	2.06	1	2	2	3	0.81	0.86	0.77	0.83	1.28	1.20	1.21	1.13	777	86.6	8.72
P56945	Breast cancer anti-estrogen resistance protein 1 OS=Homo sapiens GN=BCAR1 PE=1 SV=2 - [BCAR1_HUMAN]	0.92	1	1	1	34	1.01	0.94	0.90	0.84	0.90	0.67	1.62	0.71	870	93.3	5.67
Q9HCU9	Breast cancer metastasis-suppressor 1 OS=Homo sapiens GN=BRMS1 PE=1 SV=1 - [BRMS1_HUMAN]	7.72	1	1	1	1					0.99	1.00	0.98	0.99	246	28.4	4.74

Q5PSV4	Breast cancer metastasis-suppressor 1-like protein OS=Homo sapiens GN=BRMS1L PE=1 SV=2 - [BRM1L_HUMAN]	3.10	1	1	1	5	1.05	1.30	0.90	1.21					323	37.6	5.15
P51587	Breast cancer type 2 susceptibility protein OS=Homo sapiens GN=BRCA2 PE=1 SV=2 - [BRCA2_HUMAN]	1.43	1	2	2	2	0.94	1.29	0.99	1.37					3418	384.0	6.73
Q9Y6D6	Brefeldin A-inhibited guanine nucleotide-exchange protein 1 OS=Homo sapiens GN=ARFGEF1 PE=1 SV=2 - [BIG1_HUMAN]	0.59	1	1	1	52					0.82	1.00	0.92	1.13	1849	208.6	5.85
Q9Y6D5	Brefeldin A-inhibited guanine nucleotide-exchange protein 2 OS=Homo sapiens GN=ARFGEF2 PE=1 SV=3 - [BIG2_HUMAN]	4.71	1	4	4	5	0.80	0.88	0.80	0.76					1785	201.9	6.33
Q96GW7	Brevican core protein OS=Homo sapiens GN=BCAN PE=1 SV=2 - [PGCB_HUMAN]	4.94	1	2	3	4					1.60	1.38	1.43	1.23	911	99.1	4.64
Q9UBW5	Bridging integrator 2 OS=Homo sapiens GN=BIN2 PE=1 SV=3 - [BIN2_HUMAN]	9.91	1	5	5	78	0.37	0.25	0.48	0.30	1.87	1.96	1.41	1.42	565	61.8	5.15
Q9UIF9	Bromodomain adjacent to zinc finger domain protein 2A OS=Homo sapiens GN=BAZ2A PE=1 SV=4 - [BAZ2A_HUMAN]	2.52	7	2	3	5	1.20	1.36	1.30	1.46	0.86	0.77	0.90	0.81	1905	211.1	6.64
Q9UIF8	Bromodomain adjacent to zinc finger domain protein 2B OS=Homo sapiens GN=BAZ2B PE=1 SV=3 - [BAZ2B_HUMAN]	1.29	1	1	1	1	0.92	0.84	0.69	0.64					2168	240.3	6.54
Q9ULD4	Bromodomain and PHD finger-containing protein 3 OS=Homo sapiens GN=BRPF3 PE=1 SV=2 - [BRPF3_HUMAN]	1.33	1	1	1	2	0.86	0.73	0.92	0.79					1205	135.7	6.58
Q9NSI6	Bromodomain and WD repeat-containing protein 1 OS=Homo sapiens GN=BRWD1 PE=1 SV=4 - [BRWD1_HUMAN]	1.03	1	1	2	3					1.06	0.97	0.95	0.86	2320	262.8	8.46
Q6RI45	Bromodomain and WD repeat-containing protein 3 OS=Homo sapiens GN=BRWD3 PE=1 SV=2 - [BRWD3_HUMAN]	2.33	1	3	3	21	0.76	0.97	0.75	0.95	2.24	1.99	2.25	1.99	1802	203.5	7.84
Q58F21	Bromodomain testis-specific protein OS=Homo sapiens GN=BRDT PE=1 SV=4 - [BRDT_HUMAN]	2.75	1	2	2	2	1.32	1.34	1.17	1.19					947	107.9	8.95
O60885	Bromodomain-containing protein 4 OS=Homo sapiens GN=BRD4 PE=1 SV=2 - [BRD4_HUMAN]	1.25	1	1	1	1					0.71	0.41	0.81	0.47	1362	152.1	9.19
Q9H0E9	Bromodomain-containing protein 8 OS=Homo sapiens GN=BRD8 PE=1 SV=2 - [BRD8_HUMAN]	1.46	1	1	1	1					0.94	1.12	0.98	1.16	1235	135.3	4.60
Q9H8M2	Bromodomain-containing protein 9 OS=Homo sapiens GN=BRD9 PE=1 SV=2 - [BRD9_HUMAN]	3.02	1	1	1	2	0.50	0.42	0.49	0.41	0.33	0.40	0.36	0.43	597	67.0	6.19
Q9BSF8	BTB/POZ domain-containing protein 10 OS=Homo sapiens GN=BTBD10 PE=1 SV=2 - [BTBDA_HUMAN]	3.16	1	1	1	1					1.06	1.12	0.89	0.94	475	53.7	7.77
Q9Y2F9	BTB/POZ domain-containing protein 3 OS=Homo sapiens GN=BTBD3 PE=2 SV=1 - [BTBD3_HUMAN]	2.30	1	1	1	1					0.73	0.83	0.80	0.91	522	58.4	7.46
Q5XKL5	BTB/POZ domain-containing protein 8 OS=Homo sapiens GN=BTBD8 PE=2 SV=2 - [BTBD8_HUMAN]	3.70	1	1	1	1					0.51	0.60	0.62	0.73	378	42.8	6.00
Q68DU8	BTB/POZ domain-containing protein KCTD16 OS=Homo sapiens GN=KCTD16 PE=2 SV=1 - [KCD16_HUMAN]	3.27	1	1	1	1					0.64	0.75	0.67	0.79	428	49.1	8.09
Q8N5Z5	BTB/POZ domain-containing protein KCTD17 OS=Homo sapiens GN=KCTD17 PE=1 SV=3 - [KCD17_HUMAN]	4.98	1	1	1	1					0.84		1.48		321	35.6	4.86
Q8WVF5	BTB/POZ domain-containing protein KCTD4 OS=Homo sapiens GN=KCTD4 PE=2 SV=2 - [KCTD4_HUMAN]	3.47	1	1	1	1					1.06	0.85	1.40	1.12	259	29.9	7.06
Q6ZWB6	BTB/POZ domain-containing protein KCTD8 OS=Homo sapiens GN=KCTD8 PE=2 SV=1 - [KCTD8_HUMAN]	8.03	1	2	2	4	1.28	1.29	1.24	1.25	1.06	0.85	1.30	1.04	473	52.4	8.37
Q9BRD0	BUD13 homolog OS=Homo sapiens GN=BUD13 PE=1 SV=1 - [BUD13_HUMAN]	1.45	1	1	1	5	1.01	0.86	0.97	0.83	1.02	1.46	1.03	1.49	619	70.5	9.86
Q8WVV5	Butyrophilin subfamily 2 member A2 OS=Homo sapiens GN=BTN2A2 PE=2 SV=2 - [BT2A2_HUMAN]	4.78	1	2	2	2	1.48	1.06	1.30	0.94	0.15	1.55	0.39	4.02	523	59.0	6.01
P78410	Butyrophilin subfamily 3 member A2 OS=Homo sapiens GN=BTN3A2 PE=1 SV=2 - [BT3A2_HUMAN]	4.19	1	1	1	1					1.44	1.32	1.40	1.28	334	36.4	5.33
P41238	C->U-editing enzyme APOBEC-1 OS=Homo sapiens GN=APOBEC1 PE=1 SV=3 - [ABEC1_HUMAN]	7.20	1	1	1	2	1.01	0.89	1.04	0.93	0.68	0.89	0.73	0.96	236	28.2	8.78
P11586	C-1-tetrahydrofolate synthase, cytoplasmic OS=Homo sapiens GN=MTHFD1 PE=1 SV=3 - [C1TC_HUMAN]	4.06	1	2	2	2	0.97	1.14	0.75	1.05					935	101.5	7.30

O00421	C-C chemokine receptor-like 2 OS=Homo sapiens GN=CCRL2 PE=1 SV=2 - [CCRL2_HUMAN]	5.23	1	1	1	1					0.55	0.10	0.66	0.12	344	39.5	7.77
O15467	C-C motif chemokine 16 OS=Homo sapiens GN=CCL16 PE=1 SV=1 - [CCL16_HUMAN]	13.33	1	2	2	5					1.03	0.99	1.03	0.99	120	13.6	9.54
P55774	C-C motif chemokine 18 OS=Homo sapiens GN=CCL18 PE=1 SV=1 - [CCL18_HUMAN]	10.11	1	1	1	1					1.30	1.28	1.09	1.07	89	9.8	8.75
O00585	C-C motif chemokine 21 OS=Homo sapiens GN=CCL21 PE=1 SV=1 - [CCL21_HUMAN]	8.96	1	1	1	1					1.09	0.99	1.08	0.98	134	14.6	10.10
O15444	C-C motif chemokine 25 OS=Homo sapiens GN=CCL25 PE=1 SV=2 - [CCL25_HUMAN]	12.67	1	1	1	1					1.05	1.36	1.26	1.63	150	16.6	10.17
P13501	C-C motif chemokine 5 OS=Homo sapiens GN=CCL5 PE=1 SV=3 - [CCL5_HUMAN]	12.09	1	1	1	2					0.99	0.86	1.22	1.06	91	10.0	9.07
Q9UQF2	C-Jun-amino-terminal kinase-interacting protein 1 OS=Homo sapiens GN=MAPK8IP1 PE=1 SV=1 - [JIP1_HUMAN]	4.08	1	1	1	1	0.87	1.17	0.50	0.68					711	77.5	4.98
Q13387	C-Jun-amino-terminal kinase-interacting protein 2 OS=Homo sapiens GN=MAPK8IP2 PE=2 SV=2 - [JIP2_HUMAN]	2.67	1	1	1	1					0.95	1.03	0.69	0.75	824	87.9	4.46
Q7Z401	C-myc promoter-binding protein OS=Homo sapiens GN=DENND4A PE=1 SV=2 - [MYCPP_HUMAN]	1.23	1	1	1	1	1.50	1.54	1.28	1.32					1863	209.1	7.31
P02741	C-reactive protein OS=Homo sapiens GN=CRP PE=1 SV=1 - [CRP_HUMAN]	23.21	1	7	7	34	1.25	1.38	1.27	1.53	1.31	1.42	1.33	1.45	224	25.0	5.63
Q9P126	C-type lectin domain family 1 member B OS=Homo sapiens GN=CLEC1B PE=1 SV=2 - [CLC1B_HUMAN]	5.24	1	1	1	1					1.05	1.22	1.16	1.34	229	26.6	8.50
Q9Y240	C-type lectin domain family 11 member A OS=Homo sapiens GN=CLEC11A PE=1 SV=1 - [CLC11_HUMAN]	4.33	1	1	1	1	1.29	1.12	1.08	0.94					323	35.7	5.16
P0C7M8	C-type lectin domain family 2 member L OS=Homo sapiens GN=CLEC2L PE=2 SV=1 - [CLC2L_HUMAN]	1.87	1	1	1	1					0.61	0.59	0.53	0.51	214	23.9	7.20
Q8N1N0	C-type lectin domain family 4 member F OS=Homo sapiens GN=CLEC4F PE=2 SV=2 - [CLC4F_HUMAN]	2.38	1	1	1	1	0.32	0.54	0.25	0.42					589	65.5	6.70
Q9BXN2	C-type lectin domain family 7 member A OS=Homo sapiens GN=CLEC7A PE=1 SV=1 - [CLC7A_HUMAN]	7.69	1	1	1	6	0.98	0.76	0.96	0.75					247	27.6	5.36
Q9UBG0	C-type mannose receptor 2 OS=Homo sapiens GN=MRC2 PE=1 SV=2 - [MRC2_HUMAN]	1.76	1	2	2	4	0.94	0.97	0.89	0.92					1479	166.6	5.83
P42830	C-X-C motif chemokine 5 OS=Homo sapiens GN=CXCL5 PE=1 SV=1 - [CXCL5_HUMAN]	10.53	1	1	1	1					0.61	0.25	0.92	0.37	114	12.0	8.88
Q8IZJ3	C3 and PZP-like alpha-2-macroglobulin domain-containing protein 8 OS=Homo sapiens GN=CPAMD8 PE=1 SV=2 - [CPMD8_HUMAN]	0.95	1	1	1	1					1.01	1.03	1.04	1.06	1885	206.6	6.42
P04003	C4b-binding protein alpha chain OS=Homo sapiens GN=C4BPA PE=1 SV=2 - [C4BPA_HUMAN]	52.26	1	32	32	889	0.91	0.87	0.91	0.90	1.06	0.96	1.08	1.00	597	67.0	7.30
P20851	C4b-binding protein beta chain OS=Homo sapiens GN=C4BPB PE=1 SV=1 - [C4BPB_HUMAN]	47.22	1	11	11	241	0.98	1.01	0.99	1.07	1.22	1.17	1.21	1.10	252	28.3	5.14
Q9P296	C5a anaphylatoxin chemotactic receptor C5L2 OS=Homo sapiens GN=GPR77 PE=2 SV=1 - [C5ARL_HUMAN]	6.23	1	1	1	5	0.69	0.57	0.56	0.47					337	36.1	7.87
P21730	C5a anaphylatoxin chemotactic receptor OS=Homo sapiens GN=C5AR1 PE=1 SV=2 - [C5AR_HUMAN]	3.71	1	1	1	1					0.48	0.81	0.55	0.92	350	39.3	9.01
O75844	CAAX prenyl protease 1 homolog OS=Homo sapiens GN=ZMPSTE24 PE=1 SV=2 - [FACE1_HUMAN]	3.37	4	1	2	6					1.64	0.92	2.07	1.17	475	54.8	7.49
Q9NYQ6	Cadherin EGF LAG seven-pass G-type receptor 1 OS=Homo sapiens GN=CELSR1 PE=1 SV=1 - [CELR1_HUMAN]	2.55	1	5	5	7	0.97	1.00	1.01	1.05	1.13	1.08	1.09	1.04	3014	329.3	5.92
Q9NYQ7	Cadherin EGF LAG seven-pass G-type receptor 3 OS=Homo sapiens GN=CELSR3 PE=1 SV=2 - [CELR3_HUMAN]	1.21	1	2	2	2	0.95	1.06	1.07	1.20					3312	358.0	6.68
P12830	Cadherin-1 OS=Homo sapiens GN=CDH1 PE=1 SV=3 - [CADH1_HUMAN]	10.32	1	7	8	21	0.96	1.04	0.98	1.07	1.02	1.13	1.02	1.10	882	97.4	4.73
P55290	Cadherin-13 OS=Homo sapiens GN=CDH13 PE=1 SV=1 - [CAD13_HUMAN]	14.73	1	8	8	41	0.93	0.82	0.90	0.79	0.87	0.82	0.97	0.91	713	78.2	4.98

P55291	Cadherin-15 OS=Homo sapiens GN=CDH15 PE=1 SV=1 - [CAD15_HUMAN]	1.84	1	1	1	1					1.02	1.13	0.86	0.95	814	88.9	4.98
Q9H159	Cadherin-19 OS=Homo sapiens GN=CDH19 PE=2 SV=1 - [CAD19_HUMAN]	1.42	1	1	1	1					5.04	2.88	1.19	0.68	772	86.9	4.73
P19022	Cadherin-2 OS=Homo sapiens GN=CDH2 PE=1 SV=4 - [CADH2_HUMAN]	7.28	1	4	4	10	1.03	0.97	1.05	1.00	0.89	0.92	0.98	1.02	906	99.7	4.81
Q9H251	Cadherin-23 OS=Homo sapiens GN=CDH23 PE=1 SV=1 - [CAD23_HUMAN]	0.75	1	1	1	4	0.73	0.51	0.67	0.47					3354	369.3	4.67
P22223	Cadherin-3 OS=Homo sapiens GN=CDH3 PE=1 SV=2 - [CADH3_HUMAN]	2.77	1	1	2	2	0.76	0.94	0.73	0.90					829	91.4	4.75
P33151	Cadherin-5 OS=Homo sapiens GN=CDH5 PE=1 SV=5 - [CADH5_HUMAN]	14.29	1	9	9	76	0.98	1.02	0.93	0.97	0.88	0.86	0.91	0.88	784	87.5	5.43
P55285	Cadherin-6 OS=Homo sapiens GN=CDH6 PE=1 SV=1 - [CADH6_HUMAN]	3.42	1	2	2	5					0.99	1.04	0.99	1.03	790	88.3	4.93
Q9ULB5	Cadherin-7 OS=Homo sapiens GN=CDH7 PE=2 SV=2 - [CADH7_HUMAN]	1.78	1	1	1	1	0.64		0.67						785	87.0	4.81
P55286	Cadherin-8 OS=Homo sapiens GN=CDH8 PE=1 SV=2 - [CADH8_HUMAN]	8.01	1	3	3	3					0.85	1.06	1.01	1.26	799	88.2	4.70
Q9BYE9	Cadherin-related family member 2 OS=Homo sapiens GN=CDHR2 PE=1 SV=2 - [CDHR2_HUMAN]	3.05	1	3	3	5	0.55	0.53	0.54	0.52					1310	141.5	4.50
Q9Y6J0	Calcineurin-binding protein cabin-1 OS=Homo sapiens GN=CABIN1 PE=1 SV=1 - [CABIN_HUMAN]	1.53	1	2	2	3	1.96	1.05							2220	246.2	6.02
P30988	Calcitonin receptor OS=Homo sapiens GN=CALCR PE=1 SV=1 - [CALCR_HUMAN]	1.22	1	1	1	1					0.71	1.04	0.91	1.32	490	57.3	8.60
Q86XJ0	Calcium homeostasis modulator protein 3 OS=Homo sapiens GN=CALHM3 PE=2 SV=1 - [CAHM3_HUMAN]	4.57	1	1	1	1					0.85	1.25	0.88	1.30	350	39.1	8.53
P49069	Calcium signal-modulating cyclophilin ligand OS=Homo sapiens GN=CAMLG PE=1 SV=1 - [CAMLG_HUMAN]	3.72	1	1	1	1					0.86	0.85	0.91	0.89	296	32.9	8.05
Q9NPA1	Calcium-activated potassium channel subunit beta-3 OS=Homo sapiens GN=KCNMB3 PE=1 SV=2 - [KCMB3_HUMAN]	11.47	1	1	1	1	0.97		1.06						279	31.6	7.28
Q9P1Z2	Calcium-binding and coiled-coil domain-containing protein 1 OS=Homo sapiens GN=CALCOCO1 PE=1 SV=2 - [CACO1_HUMAN]	7.24	1	3	3	3	1.36	1.50	0.85	0.94	0.92	0.83	0.91	0.82	691	77.3	4.82
Q96KC9	Calcium-binding and spermatid-specific protein 1 OS=Homo sapiens GN=CABS1 PE=2 SV=3 - [CABS1_HUMAN]	2.03	1	1	1	1	1.22	1.50	1.25	1.54					395	43.0	4.21
O75746	Calcium-binding mitochondrial carrier protein Aralar1 OS=Homo sapiens GN=SLC25A12 PE=1 SV=2 - [CMC1_HUMAN]	1.77	1	1	1	1					1.07	1.04	0.98	0.95	678	74.7	8.38
Q9UJS0	Calcium-binding mitochondrial carrier protein Aralar2 OS=Homo sapiens GN=SLC25A13 PE=1 SV=2 - [CMC2_HUMAN]	4.59	1	1	1	1					1.70	2.09	1.71	2.10	675	74.1	8.62
Q6KCM7	Calcium-binding mitochondrial carrier protein SCaMC-2 OS=Homo sapiens GN=SLC25A25 PE=1 SV=1 - [SCMC2_HUMAN]	3.20	1	1	1	4	1.00	1.44	0.52	0.75					469	52.6	8.35
Q9Y376	Calcium-binding protein 39 OS=Homo sapiens GN=CAB39 PE=1 SV=1 - [CAB39_HUMAN]	3.23	1	1	1	1					0.30	0.33	0.29	0.33	341	39.8	6.89
Q9H9S4	Calcium-binding protein 39-like OS=Homo sapiens GN=CAB39L PE=1 SV=3 - [CB39L_HUMAN]	2.67	1	1	1	3	0.66	0.85	0.59	0.77	0.77	0.66	0.79	0.69	337	39.1	8.40
Q9BXU9	Calcium-binding protein 8 OS=Homo sapiens GN=CALN1 PE=2 SV=1 - [CABP8_HUMAN]	9.59	1	1	1	1	1.64	1.23	1.78	1.35					219	24.8	4.84
Q86UW7	Calcium-dependent secretion activator 2 OS=Homo sapiens GN=CADPS2 PE=1 SV=2 - [CAPS2_HUMAN]	1.62	1	2	2	162	1.10	0.87	1.12	0.91	1.00	0.91	0.97	0.88	1296	147.6	6.19
Q9NP80	Calcium-independent phospholipase A2-gamma OS=Homo sapiens GN=PNPLA8 PE=1 SV=1 - [PLPL8_HUMAN]	1.28	1	1	1	3	0.97	0.90	0.95	0.89	0.91	0.75	0.96	0.80	782	88.4	9.23
Q9Y2V2	Calcium-regulated heat stable protein 1 OS=Homo sapiens GN=CARHSP1 PE=1 SV=2 - [CHSP1_HUMAN]	7.48	1	1	1	2					1.09	1.14	1.00	1.03	147	15.9	8.21
Q01064	Calcium/calmodulin-dependent 3',5'-cyclic nucleotide phosphodiesterase 1B OS=Homo sapiens GN=PDE1B PE=1 SV=2 - [PDE1B_HUMAN]	4.29	1	1	1	3	1.09	1.08	0.99	1.00					536	61.3	5.52

Q14123	Calcium/calmodulin-dependent 3',5'-cyclic nucleotide phosphodiesterase 1C OS=Homo sapiens GN=PDE1C PE=1 SV=1 - [PDE1C_HUMAN]	3.81	1	2	2	2					0.63	0.96	0.88	1.33	709	80.7	8.82
Q6P2M8	Calcium/calmodulin-dependent protein kinase type 1B OS=Homo sapiens GN=PNCK PE=2 SV=2 - [KCC1B_HUMAN]	1.75	1	1	1	7					1.21	1.37	1.04	1.16	343	38.5	6.76
Q8IU85	Calcium/calmodulin-dependent protein kinase type 1D OS=Homo sapiens GN=CAMK1D PE=1 SV=1 - [KCC1D_HUMAN]	3.90	3	1	2	21					1.36	1.33	1.36	1.34	385	42.9	7.21
Q13557	Calcium/calmodulin-dependent protein kinase type II subunit delta OS=Homo sapiens GN=CAMK2D PE=1 SV=3 - [KCC2D_HUMAN]	2.61	1	1	1	3	1.18	0.89	0.95	0.71					499	56.3	7.25
Q13938	Calcyphosin OS=Homo sapiens GN=CAPS PE=1 SV=1 - [CAYP1_HUMAN]	7.94	4	1	2	5	0.92	1.99	0.61	1.32					189	21.0	4.89
Q05682	Caldesmon OS=Homo sapiens GN=CALD1 PE=1 SV=3 - [CALD1_HUMAN]	16.39	1	12	12	34					1.81	1.73	1.80	1.75	793	93.2	5.66
Q13939	Calicin OS=Homo sapiens GN=CCIN PE=2 SV=3 - [CALI_HUMAN]	1.36	1	1	1	1					1.21	0.96	1.10	0.87	588	66.5	8.18
P62158	Calmodulin OS=Homo sapiens GN=CALM1 PE=1 SV=2 - [CALM_HUMAN]	11.41	2	2	2	3	0.57	0.49	0.57	0.50	0.26	0.57	0.29	0.64	149	16.8	4.22
Q96L46	Calpain small subunit 2 OS=Homo sapiens GN=CAPNS2 PE=2 SV=2 - [CPNS2_HUMAN]	4.44	1	1	1	1					2.14	1.64	2.25	1.72	248	27.6	5.73
P07384	Calpain-1 catalytic subunit OS=Homo sapiens GN=CAPN1 PE=1 SV=1 - [CAN1_HUMAN]	2.52	1	1	1	2	0.76	0.80	0.75	0.78					714	81.8	5.67
Q6ZSI9	Calpain-12 OS=Homo sapiens GN=CAPN12 PE=2 SV=1 - [CAN12_HUMAN]	3.62	1	1	1	1	2.93	3.91	2.93	3.92					719	81.0	6.38
P20807	Calpain-3 OS=Homo sapiens GN=CAPN3 PE=1 SV=2 - [CAN3_HUMAN]	5.97	1	2	2	2	1.05	1.14	1.26	1.38					821	94.2	6.18
O15484	Calpain-5 OS=Homo sapiens GN=CAPN5 PE=1 SV=2 - [CAN5_HUMAN]	3.44	1	1	1	2					0.21	0.15	1.14	0.79	640	73.1	7.64
A6NHC0	Calpain-8 OS=Homo sapiens GN=CAPN8 PE=2 SV=3 - [CAN8_HUMAN]	4.69	1	2	2	8	0.75	0.59	0.84	0.67	1.11	0.94	1.12	0.96	703	79.1	5.22
P20810	Calpastatin OS=Homo sapiens GN=CAST PE=1 SV=4 - [ICAL_HUMAN]	4.80	1	1	1	1	1.24	0.96	1.21	0.95					708	76.5	5.07
P51911	Calponin-1 OS=Homo sapiens GN=CNN1 PE=1 SV=2 - [CNN1_HUMAN]	15.15	1	2	2	3	1.40	1.62	1.08	1.26	1.06	0.52	1.32	0.64	297	33.1	9.07
Q99439	Calponin-2 OS=Homo sapiens GN=CNN2 PE=1 SV=4 - [CNN2_HUMAN]	27.18	1	7	7	27	0.80	0.78	0.79	0.79	1.07	1.05	0.99	0.92	309	33.7	7.33
P27797	Calreticulin OS=Homo sapiens GN=CALR PE=1 SV=1 - [CALR_HUMAN]	5.04	1	1	1	1	0.73	0.66	0.61	0.55					417	48.1	4.44
P22676	Calretinin OS=Homo sapiens GN=CALB2 PE=1 SV=2 - [CALB2_HUMAN]	7.01	1	1	1	2					1.10	1.18	0.97	1.04	271	31.5	5.15
O94985	Calsyntenin-1 OS=Homo sapiens GN=CLSTN1 PE=1 SV=1 - [CSTN1_HUMAN]	5.20	1	4	4	13	1.10	1.06	1.09	1.07	1.05	1.07	1.00	1.01	981	109.7	4.91
O43852	Calumenin OS=Homo sapiens GN=CALU PE=1 SV=2 - [CALU_HUMAN]	6.35	1	2	2	4	0.87	0.68	0.83	0.66	0.96	1.11	1.02	1.18	315	37.1	4.64
Q8NCB2	CaM kinase-like vesicle-associated protein OS=Homo sapiens GN=CAMKV PE=2 SV=2 - [CAMKV_HUMAN]	2.79	1	1	1	1					0.47	0.56	0.92	1.10	501	54.3	5.55
Q9Y233	cAMP and cAMP-inhibited cGMP 3',5'-cyclic phosphodiesterase 10A OS=Homo sapiens GN=PDE10A PE=1 SV=1 - [PDE10_HUMAN]	1.80	1	1	1	1	0.82	1.02	0.71	0.88					779	88.4	6.60
P10644	cAMP-dependent protein kinase type I-alpha regulatory subunit OS=Homo sapiens GN=PRKAR1A PE=1 SV=1 - [KAP0_HUMAN]	5.51	1	2	2	3					0.90	0.75	0.91	0.75	381	43.0	5.35
P31321	cAMP-dependent protein kinase type I-beta regulatory subunit OS=Homo sapiens GN=PRKAR1B PE=1 SV=4 - [KAP1_HUMAN]	2.36	1	1	1	2					0.46	0.45	0.80	0.64	381	43.0	5.71
Q07343	cAMP-specific 3',5'-cyclic phosphodiesterase 4B OS=Homo sapiens GN=PDE4B PE=1 SV=1 - [PDE4B_HUMAN]	3.13	1	1	1	2					1.29	1.76	1.02	1.39	736	83.3	5.25
Q08499	cAMP-specific 3',5'-cyclic phosphodiesterase 4D OS=Homo sapiens GN=PDE4D PE=1 SV=2 - [PDE4D_HUMAN]	1.11	1	1	1	2					2.15	1.83	1.82	1.56	809	91.1	5.54

Q92887	Canalicular multispecific organic anion transporter 1 OS=Homo sapiens GN=ABCC2 PE=1 SV=3 - [MRP2_HUMAN]	1.94	1	2	2	12	0.73	0.97	0.70	0.80	1.17	1.16	1.15	1.14	1545	174.1	8.32
Q6PK30	Cancer/testis antigen 75 OS=Homo sapiens GN=CT75 PE=2 SV=2 - [CT75_HUMAN]	3.97	1	1	1	1	0.91	0.99	0.93	1.03					378	40.6	10.98
P30622	CAP-Gly domain-containing linker protein 1 OS=Homo sapiens GN=CLIP1 PE=1 SV=2 - [CLIP1_HUMAN]	2.64	1	3	3	3	0.71	0.82	0.75	0.88	1.75	1.64	1.75	1.63	1438	162.1	5.36
Q9UDT6	CAP-Gly domain-containing linker protein 2 OS=Homo sapiens GN=CLIP2 PE=1 SV=1 - [CLIP2_HUMAN]	1.63	1	1	1	3					1.47	1.59	1.18	1.28	1046	115.8	6.73
Q8N3C7	CAP-Gly domain-containing linker protein 4 OS=Homo sapiens GN=CLIP4 PE=1 SV=1 - [CLIP4_HUMAN]	1.13	1	1	1	1		3.34		0.89					705	76.3	8.70
Q6IMN6	Caprin-2 OS=Homo sapiens GN=CAPRIN2 PE=1 SV=1 - [CAPR2_HUMAN]	2.31	1	1	1	2	0.90	0.86	0.91	0.88					1127	125.8	6.71
Q8IV04	Carabin OS=Homo sapiens GN=TBC1D10C PE=1 SV=1 - [TB10C_HUMAN]	2.24	1	1	1	2					0.74	0.74	0.85	0.84	446	49.7	8.76
P31327	Carbamoyl-phosphate synthase [ammonia], mitochondrial OS=Homo sapiens GN=CPS1 PE=1 SV=2 - [CPSM_HUMAN]	1.47	1	2	2	7	0.77	0.73	0.77	0.77	1.06	1.04	1.04	1.03	1500	164.8	6.74
Q7LGC8	Carbohydrate sulfotransferase 3 OS=Homo sapiens GN=CHST3 PE=1 SV=3 - [CHST3_HUMAN]	3.34	1	1	1	1					1.09	0.74	1.09	0.74	479	54.7	8.59
Q7L1S5	Carbohydrate sulfotransferase 9 OS=Homo sapiens GN=CHST9 PE=2 SV=2 - [CHST9_HUMAN]	2.03	1	1	1	1	1.03	0.99	1.07	1.03					443	52.0	9.38
P00915	Carbonic anhydrase 1 OS=Homo sapiens GN=CA1 PE=1 SV=2 - [CAH1_HUMAN]	32.57	1	6	6	74	0.78	0.81	0.78	0.80	0.67	0.70	0.62	0.66	261	28.9	7.12
P00918	Carbonic anhydrase 2 OS=Homo sapiens GN=CA2 PE=1 SV=2 - [CAH2_HUMAN]	25.00	1	5	5	34	0.95	0.81	0.86	0.75	0.76	0.77	0.73	0.74	260	29.2	7.40
P07451	Carbonic anhydrase 3 OS=Homo sapiens GN=CA3 PE=1 SV=3 - [CAH3_HUMAN]	6.54	1	1	1	1	0.85	0.78	0.98	0.90					260	29.5	7.34
P23280	Carbonic anhydrase 6 OS=Homo sapiens GN=CA6 PE=1 SV=3 - [CAH6_HUMAN]	1.95	1	1	1	5	0.96	0.93	0.99	0.98	0.87	1.05	0.99	1.23	308	35.3	7.02
P43166	Carbonic anhydrase 7 OS=Homo sapiens GN=CA7 PE=1 SV=1 - [CAH7_HUMAN]	3.41	1	1	1	1	0.96	1.07	0.96	1.07					264	29.6	7.42
O75493	Carbonic anhydrase-related protein 11 OS=Homo sapiens GN=CA11 PE=1 SV=2 - [CAH11_HUMAN]	8.84	1	1	1	1	0.95	1.02	0.95	1.02					328	36.2	9.74
P16152	Carbonyl reductase [NADPH] 1 OS=Homo sapiens GN=CBR1 PE=1 SV=3 - [CBR1_HUMAN]	11.19	1	2	3	8	0.99	0.97	1.04	1.02	1.96	2.01	1.69	1.74	277	30.4	8.32
O75828	Carbonyl reductase [NADPH] 3 OS=Homo sapiens GN=CBR3 PE=1 SV=3 - [CBR3_HUMAN]	7.22	1	1	2	4					1.01	1.34	1.05	1.39	277	30.8	6.18
Q6UWW8	Carboxylesterase 3 OS=Homo sapiens GN=CES3 PE=1 SV=1 - [EST3_HUMAN]	3.33	1	1	1	1					6.19	6.59	5.90	6.29	571	62.2	5.62
Q5XG92	Carboxylesterase 4A OS=Homo sapiens GN=CES4A PE=2 SV=2 - [EST4A_HUMAN]	2.32	1	1	1	1	0.61	0.23	0.83	0.31					561	63.5	9.31
Q96IY4	Carboxypeptidase B2 OS=Homo sapiens GN=CPB2 PE=1 SV=2 - [CBPB2_HUMAN]	25.30	1	11	12	138	1.01	1.05	1.03	1.07	1.18	1.10	1.16	1.15	423	48.4	7.71
P14384	Carboxypeptidase M OS=Homo sapiens GN=CPM PE=1 SV=2 - [CBPM_HUMAN]	1.35	1	1	1	1	1.34	1.49	1.39	1.56					443	50.5	7.36
P15169	Carboxypeptidase N catalytic chain OS=Homo sapiens GN=CPN1 PE=1 SV=1 - [CBPN_HUMAN]	39.52	1	14	14	112	1.03	1.03	1.02	1.04	0.87	0.86	0.88	0.86	458	52.3	7.34
P22792	Carboxypeptidase N subunit 2 OS=Homo sapiens GN=CPN2 PE=1 SV=3 - [CPN2_HUMAN]	40.73	1	16	16	323	1.08	1.08	1.08	1.09	1.02	1.09	0.98	1.01	545	60.5	5.99
Q9Y646	Carboxypeptidase Q OS=Homo sapiens GN=CPQ PE=1 SV=1 - [CBPQ_HUMAN]	9.11	1	3	4	9	1.03	1.14	0.85	0.99					472	51.9	6.18
Q66K79	Carboxypeptidase Z OS=Homo sapiens GN=CPZ PE=1 SV=2 - [CBPZ_HUMAN]	2.91	1	1	1	3	2.28	2.17	2.39	2.29					652	73.6	7.97
Q8N3K9	Cardiomyopathy-associated protein 5 OS=Homo sapiens GN=CMYA5 PE=1 SV=3 - [CMYA5_HUMAN]	1.92	3	4	5	5	0.87	1.01	0.76	0.89					4069	448.9	4.78

Q9UBD9	Cardiotrophin-like cytokine factor 1 OS=Homo sapiens GN=CLCF1 PE=1 SV=1 - [CLCF1_HUMAN]	7.56	1	1	1	1					1.90	2.06	1.66	1.80	225	25.2	8.46
P43155	Carnitine O-acetyltransferase OS=Homo sapiens GN=CRAT PE=1 SV=5 - [CACP_HUMAN]	2.08	9	1	2	2	1.01	1.03	1.03	1.05					626	70.8	8.44
Q8TCG5	Carnitine O-palmitoyltransferase 1, brain isoform OS=Homo sapiens GN=CPT1C PE=2 SV=1 - [CPT1C_HUMAN]	1.00	1	1	1	1					1.01	0.61	1.31	0.80	803	90.9	8.12
P23786	Carnitine O-palmitoyltransferase 2, mitochondrial OS=Homo sapiens GN=CPT2 PE=1 SV=2 - [CPT2_HUMAN]	3.80	1	1	1	1	2.18	1.11	2.84	1.45					658	73.7	8.18
Q9NQ79	Cartilage acidic protein 1 OS=Homo sapiens GN=CRTAC1 PE=1 SV=2 - [CRAC1_HUMAN]	32.68	1	17	17	94	1.15	1.18	1.15	1.18	1.04	1.02	1.04	1.09	661	71.4	5.12
O75339	Cartilage intermediate layer protein 1 OS=Homo sapiens GN=CILP PE=1 SV=4 - [CILP1_HUMAN]	1.60	3	1	3	6	0.90	1.00	0.86	0.97	1.27	0.83	1.19	0.78	1184	132.5	8.41
Q8IUL8	Cartilage intermediate layer protein 2 OS=Homo sapiens GN=CILP2 PE=2 SV=2 - [CILP2_HUMAN]	3.37	1	3	4	6	1.07	1.10	1.07	1.11					1156	126.2	8.22
P21941	Cartilage matrix protein OS=Homo sapiens GN=MATN1 PE=1 SV=1 - [MATN1_HUMAN]	2.82	1	1	1	3	0.86	1.18	0.89	1.23					496	53.7	7.69
P49747	Cartilage oligomeric matrix protein OS=Homo sapiens GN=COMP PE=1 SV=2 - [COMP_HUMAN]	28.93	1	14	16	70	0.81	0.77	0.83	0.79	0.75	0.84	0.80	0.90	757	82.8	4.60
Q9NQ75	Cas scaffolding protein family member 4 OS=Homo sapiens GN=CASS4 PE=1 SV=2 - [CASS4_HUMAN]	2.42	1	1	1	2					1.49	1.44	1.42	1.38	786	87.1	7.08
Q96PB1	CAS1 domain-containing protein 1 OS=Homo sapiens GN=CASD1 PE=2 SV=1 - [CASD1_HUMAN]	4.89	1	2	2	3	1.67	1.20	1.62	1.17	0.77	0.83	0.99	1.07	797	91.6	8.82
P48729	Casein kinase I isoform alpha OS=Homo sapiens GN=CSNK1A1 PE=1 SV=2 - [KC1A_HUMAN]	10.68	1	2	2	4	1.51	1.18	1.52	1.21	1.26	1.03	1.21	0.99	337	38.9	9.57
P48730	Casein kinase I isoform delta OS=Homo sapiens GN=CSNK1D PE=1 SV=2 - [KC1D_HUMAN]	5.06	1	1	1	2							0.41		415	47.3	9.74
Q9Y6M4	Casein kinase I isoform gamma-3 OS=Homo sapiens GN=CSNK1G3 PE=1 SV=2 - [KC1G3_HUMAN]	4.25	1	1	1	1	1.23	1.15	1.22	1.14					447	51.4	9.22
Q9BWT7	Caspase recruitment domain-containing protein 10 OS=Homo sapiens GN=CARD10 PE=2 SV=2 - [CAR10_HUMAN]	3.10	1	2	2	3	0.65	1.44	0.59	1.32	0.69	0.71	3.70	3.99	1032	115.9	5.95
Q9BXL7	Caspase recruitment domain-containing protein 11 OS=Homo sapiens GN=CARD11 PE=1 SV=3 - [CAR11_HUMAN]	0.61	1	1	1	1	0.76	0.63	0.76	0.64					1154	133.2	6.09
Q9BXL6	Caspase recruitment domain-containing protein 14 OS=Homo sapiens GN=CARD14 PE=1 SV=2 - [CAR14_HUMAN]	1.10	1	1	1	2	1.13	1.16	0.96	0.98	1.52	1.43	1.70	1.59	1004	113.2	5.92
Q9BX69	Caspase recruitment domain-containing protein 6 OS=Homo sapiens GN=CARD6 PE=1 SV=2 - [CARD6_HUMAN]	1.25	1	1	1	2	1.49	1.02	1.38	0.95					1037	116.4	6.37
P31944	Caspase-14 OS=Homo sapiens GN=CASP14 PE=1 SV=2 - [CASPE_HUMAN]	19.01	1	5	5	11	0.79	0.73	0.67	0.62	0.81	0.75	0.92	0.84	242	27.7	5.58
P51878	Caspase-5 OS=Homo sapiens GN=CASP5 PE=2 SV=3 - [CASP5_HUMAN]	1.84	1	1	1	1	0.82	0.71	0.84	0.74					434	49.7	9.16
Q14790	Caspase-8 OS=Homo sapiens GN=CASP8 PE=1 SV=1 - [CASP8_HUMAN]	3.34	2	1	2	2	1.28	1.38	1.22	1.33					479	55.4	5.10
Q9BXF3	Cat eye syndrome critical region protein 2 OS=Homo sapiens GN=CECR2 PE=1 SV=2 - [CECR2_HUMAN]	2.56	7	1	2	2	1.57	1.46	1.69	1.59					1484	164.1	6.98
P04040	Catalase OS=Homo sapiens GN=CAT PE=1 SV=3 - [CATA_HUMAN]	16.70	1	8	8	20	0.80	0.78	0.69	0.67	0.92	0.88	0.90	0.82	527	59.7	7.39
P35221	Catenin alpha-1 OS=Homo sapiens GN=CTNNA1 PE=1 SV=1 - [CTNA1_HUMAN]	3.86	1	2	2	3	0.64	0.62	0.77	0.80					906	100.0	6.29
P26232	Catenin alpha-2 OS=Homo sapiens GN=CTNNA2 PE=1 SV=5 - [CTNA2_HUMAN]	1.89	1	1	1	1	1.04	1.13	1.08	1.19					953	105.2	5.71
Q9UI47	Catenin alpha-3 OS=Homo sapiens GN=CTNNA3 PE=1 SV=2 - [CTNA3_HUMAN]	2.46	1	2	2	2	0.95	1.00	0.96	1.02					895	99.7	6.16
P49913	Cathelicidin antimicrobial peptide OS=Homo sapiens GN=CAMP PE=1 SV=1 - [CAMP_HUMAN]	30.59	1	6	6	31	0.91	0.97	0.90	0.97	1.31	1.03	1.36	1.26	170	19.3	9.41

P07858	Cathepsin B OS=Homo sapiens GN=CTSB PE=1 SV=3 - [CATB_HUMAN]	7.96	1	2	2	3	0.95	0.83	0.84	0.74	0.89	1.32	1.31	1.95	339	37.8	6.30
P07339	Cathepsin D OS=Homo sapiens GN=CTSD PE=1 SV=1 - [CATD_HUMAN]	23.06	1	8	8	22	1.25	1.20	1.21	1.20	1.19	1.31	1.04	1.14	412	44.5	6.54
Q9UBX1	Cathepsin F OS=Homo sapiens GN=CTSF PE=1 SV=1 - [CATF_HUMAN]	9.09	1	3	3	10	1.16	1.11	1.13	1.09	1.01	0.85	1.02	0.85	484	53.3	8.22
P08311	Cathepsin G OS=Homo sapiens GN=CTSG PE=1 SV=2 - [CATG_HUMAN]	23.14	1	4	4	15	1.08	1.07	1.07	1.06	0.87	0.90	0.92	0.90	255	28.8	11.19
P07711	Cathepsin L1 OS=Homo sapiens GN=CTSL1 PE=1 SV=2 - [CATL1_HUMAN]	4.20	1	1	1	1					1.48	0.02			333	37.5	5.45
O60911	Cathepsin L2 OS=Homo sapiens GN=CTSL2 PE=1 SV=2 - [CATL2_HUMAN]	6.89	1	1	1	1	0.58	0.61	0.52	0.55					334	37.3	8.76
P43234	Cathepsin O OS=Homo sapiens GN=CTSO PE=2 SV=1 - [CATO_HUMAN]	5.30	1	1	1	1					0.58	0.47	0.68	0.54	321	35.9	7.42
P25774	Cathepsin S OS=Homo sapiens GN=CTSS PE=1 SV=3 - [CATS_HUMAN]	6.65	1	1	1	1					0.84	0.74	0.77	0.68	331	37.5	8.34
P56202	Cathepsin W OS=Homo sapiens GN=CTSW PE=1 SV=2 - [CATW_HUMAN]	2.39	1	1	1	3					1.29	1.45	1.26	1.41	376	42.1	7.42
Q9UBR2	Cathepsin Z OS=Homo sapiens GN=CTSZ PE=1 SV=1 - [CATZ_HUMAN]	5.28	1	2	2	5					1.35	1.47	1.24	1.35	303	33.8	7.11
Q86XQ3	Cation channel sperm-associated protein 3 OS=Homo sapiens GN=CATSPER3 PE=1 SV=1 - [CTSR3_HUMAN]	5.78	1	1	1	1	0.98		0.85						398	46.4	6.30
Q9H7T0	Cation channel sperm-associated protein subunit beta OS=Homo sapiens GN=CATSPERB PE=1 SV=2 - [CTSRB_HUMAN]	1.25	1	1	1	1					0.90	0.67	0.86	0.64	1116	126.8	7.17
Q6ZRH7	Cation channel sperm-associated protein subunit gamma OS=Homo sapiens GN=CATSPERG PE=2 SV=3 - [CTSRG_HUMAN]	0.78	1	1	1	1					0.65	0.86	0.55	0.72	1159	132.9	6.37
Q8WUX2	Cation transport regulator-like protein 2 OS=Homo sapiens GN=CHAC2 PE=2 SV=1 - [CHAC2_HUMAN]	3.26	1	1	1	1	1.49	1.05	1.59	1.14					184	20.9	5.43
P11717	Cation-independent mannose-6-phosphate receptor OS=Homo sapiens GN=IGF2R PE=1 SV=3 - [MPRI_HUMAN]	5.14	1	9	9	17	1.02	1.00	1.01	1.07					2491	274.2	5.94
P51636	Caveolin-2 OS=Homo sapiens GN=CAV2 PE=1 SV=2 - [CAV2_HUMAN]	15.43	1	1	1	2					1.00	1.18	1.06	1.26	162	18.3	5.27
P49715	CCAAT/enhancer-binding protein alpha OS=Homo sapiens GN=CEBPA PE=1 SV=3 - [CEBPA_HUMAN]	5.31	1	1	1	1	0.90	0.85	0.93	0.89					358	37.5	7.68
A5YKK6	CCR4-NOT transcription complex subunit 1 OS=Homo sapiens GN=CNOT1 PE=1 SV=2 - [CNOT1_HUMAN]	2.23	1	3	3	3	0.83	0.75	0.88	0.80	1.35	1.34	1.21	1.20	2376	266.8	7.11
Q6YHK3	CD109 antigen OS=Homo sapiens GN=CD109 PE=1 SV=2 - [CD109_HUMAN]	9.20	1	9	9	24	1.04	1.03	1.07	1.07					1445	161.6	5.85
Q13740	CD166 antigen OS=Homo sapiens GN=ALCAM PE=1 SV=2 - [CD166_HUMAN]	8.75	1	4	4	11	1.27	1.21	1.23	1.25	1.25	1.11	1.35	1.20	583	65.1	6.25
Q8N6Q3	CD177 antigen OS=Homo sapiens GN=CD177 PE=1 SV=2 - [CD177_HUMAN]	3.89	1	2	2	4	1.36	1.38	1.43	1.46	0.96	1.02	1.05	1.11	437	46.3	6.29
Q9Y5K6	CD2-associated protein OS=Homo sapiens GN=CD2AP PE=1 SV=1 - [CD2AP_HUMAN]	3.76	1	2	2	3					1.14	1.32	1.13	1.31	639	71.4	6.40
Q15762	CD226 antigen OS=Homo sapiens GN=CD226 PE=1 SV=2 - [CD226_HUMAN]	4.17	1	1	1	1					0.83	0.83	0.80	0.80	336	38.6	7.94
P26842	CD27 antigen OS=Homo sapiens GN=CD27 PE=1 SV=2 - [CD27_HUMAN]	5.38	1	1	1	1	1.30	1.50	1.22	1.42					260	29.1	7.64
P29965	CD40 ligand OS=Homo sapiens GN=CD40LG PE=1 SV=1 - [CD40L_HUMAN]	9.96	1	1	1	2					0.79	0.90	0.70	0.80	261	29.3	8.31
P16070	CD44 antigen OS=Homo sapiens GN=CD44 PE=1 SV=3 - [CD44_HUMAN]	4.58	1	4	4	38	1.12	1.02	1.14	1.03	0.93	0.80	0.92	0.80	742	81.5	5.33
O43866	CD5 antigen-like OS=Homo sapiens GN=CD5L PE=1 SV=1 - [CD5L_HUMAN]	68.88	1	25	26	661	1.08	1.06	1.12	1.09	1.15	1.18	1.15	1.10	347	38.1	5.47

P13987	CD59 glycoprotein OS=Homo sapiens GN=CD59 PE=1 SV=1 - [CD59_HUMAN]	15.63	1	2	2	8					0.97	1.02	0.93	1.06	128	14.2	6.48
P27701	CD82 antigen OS=Homo sapiens GN=CD82 PE=1 SV=1 - [CD82_HUMAN]	5.62	1	1	1	1	0.89	0.83	0.93	0.87					267	29.6	5.24
P48960	CD97 antigen OS=Homo sapiens GN=CD97 PE=1 SV=4 - [CD97_HUMAN]	3.59	1	2	2	2	1.27	0.96	1.03	0.79					835	91.8	6.87
Q9H3Q1	Cdc42 effector protein 4 OS=Homo sapiens GN=CDC42EP4 PE=1 SV=1 - [BORG4_HUMAN]	4.21	1	1	1	2					0.44	0.22	0.74	0.38	356	38.0	5.19
Q15642	Cdc42-interacting protein 4 OS=Homo sapiens GN=TRIP10 PE=1 SV=3 - [CIP4_HUMAN]	4.16	1	1	1	3					1.07	1.12	1.06	1.10	601	68.3	5.73
Q96SN8	CDK5 regulatory subunit-associated protein 2 OS=Homo sapiens GN=CDK5RAP2 PE=1 SV=5 - [CK5P2_HUMAN]	3.12	1	4	5	17	1.46	1.04	1.60	1.20	0.86	0.93	0.85	0.90	1893	214.9	5.58
Q9BY67	Cell adhesion molecule 1 OS=Homo sapiens GN=CADM1 PE=1 SV=2 - [CADM1_HUMAN]	9.73	1	3	3	9	1.18	1.07	1.10	1.08	0.67	0.56	0.76	0.63	442	48.5	5.07
Q8N3J6	Cell adhesion molecule 2 OS=Homo sapiens GN=CADM2 PE=2 SV=1 - [CADM2_HUMAN]	3.45	1	1	1	1					0.93	0.78	0.97	0.82	435	47.5	5.33
O75943	Cell cycle checkpoint protein RAD17 OS=Homo sapiens GN=RAD17 PE=1 SV=2 - [RAD17_HUMAN]	1.17	1	1	1	1	0.85	0.85	0.85	0.86					681	77.0	7.06
Q9NQS1	Cell death regulator Aven OS=Homo sapiens GN=AVEN PE=1 SV=1 - [AVEN_HUMAN]	5.80	1	1	1	1	0.28	0.22	0.34	0.27					362	38.5	4.98
P60953	Cell division control protein 42 homolog OS=Homo sapiens GN=CDC42 PE=1 SV=2 - [CDC42_HUMAN]	27.23	4	3	4	9	0.75	0.77	0.76	0.78	1.07	1.01	1.02	0.96	191	21.2	6.55
Q99741	Cell division control protein 6 homolog OS=Homo sapiens GN=CDC6 PE=1 SV=1 - [CDC6_HUMAN]	1.96	1	1	1	1	0.92	0.94	0.87	0.90					560	62.7	9.58
Q99459	Cell division cycle 5-like protein OS=Homo sapiens GN=CDC5L PE=1 SV=2 - [CDC5L_HUMAN]	1.12	1	1	1	3	0.83	0.87	0.81	0.86					802	92.2	8.18
Q8IX12	Cell division cycle and apoptosis regulator protein 1 OS=Homo sapiens GN=CCAR1 PE=1 SV=2 - [CCAR1_HUMAN]	2.52	1	2	2	2	0.93	1.02	0.94	1.04					1150	132.7	5.76
Q12834	Cell division cycle protein 20 homolog OS=Homo sapiens GN=CDC20 PE=1 SV=2 - [CDC20_HUMAN]	4.61	1	1	1	1					1.17	0.81	1.10	0.76	499	54.7	9.23
P30260	Cell division cycle protein 27 homolog OS=Homo sapiens GN=CDC27 PE=1 SV=2 - [CDC27_HUMAN]	4.00	1	2	2	3	0.92	1.02	0.86	0.96					824	91.8	7.02
Q96GN5	Cell division cycle-associated 7-like protein OS=Homo sapiens GN=CDCA7L PE=1 SV=2 - [CDA7L_HUMAN]	3.74	1	1	1	1	1.46	1.06	1.31	0.96					454	52.2	5.74
Q69YH5	Cell division cycle-associated protein 2 OS=Homo sapiens GN=CDCA2 PE=1 SV=2 - [CDCA2_HUMAN]	3.71	1	2	2	3	0.84	0.78	0.76	0.72					1023	112.6	8.40
Q9BXL8	Cell division cycle-associated protein 4 OS=Homo sapiens GN=CDCA4 PE=2 SV=1 - [CDCA4_HUMAN]	10.79	1	2	2	2					0.66	0.76	0.99	1.14	241	26.1	4.97
Q99675	Cell growth regulator with RING finger domain protein 1 OS=Homo sapiens GN=CGRRF1 PE=1 SV=1 - [CGRF1_HUMAN]	3.92	1	1	1	1	1.03	0.81	0.88	0.69					332	38.2	5.16
Q9NX58	Cell growth-regulating nucleolar protein OS=Homo sapiens GN=LYAR PE=1 SV=2 - [LYAR_HUMAN]	5.28	1	2	2	3	0.61	0.82	0.70	0.95	1.42	1.51	1.24	1.32	379	43.6	9.54
Q8TD46	Cell surface glycoprotein CD200 receptor 1 OS=Homo sapiens GN=CD200R1 PE=1 SV=2 - [MO2R1_HUMAN]	7.38	1	1	1	1					0.83	0.79	0.76	0.72	325	36.6	7.93
P43121	Cell surface glycoprotein MUC18 OS=Homo sapiens GN=MCAM PE=1 SV=2 - [MUC18_HUMAN]	8.51	1	5	5	31	0.98	0.94	1.01	0.96	0.94	1.08	1.00	1.06	646	71.6	5.76
P04637	Cellular tumor antigen p53 OS=Homo sapiens GN=TP53 PE=1 SV=4 - [P53_HUMAN]	3.56	1	1	1	1	1.30	1.24	1.21	1.17					393	43.6	6.79
Q9NXG0	Centlein OS=Homo sapiens GN=CNTLN PE=2 SV=5 - [CNTLN_HUMAN]	3.35	1	3	3	4	0.97	0.93	1.06	0.93					1405	161.5	8.15
Q7Z7A1	Centriolin OS=Homo sapiens GN=CNTRL PE=1 SV=2 - [CNTRL_HUMAN]	3.23	1	6	6	12	1.10	1.12	0.99	0.96	0.76	0.72	0.63	0.59	2325	268.7	5.55
P49454	Centromere protein F OS=Homo sapiens GN=CENPF PE=1 SV=2 - [CENPF_HUMAN]	1.21	1	3	4	15	1.50	1.17	1.06	0.92					3210	367.5	5.07

Q9BS16	Centromere protein K OS=Homo sapiens GN=CENPK PE=1 SV=1 - [CENPK_HUMAN]	8.18	1	1	1	1	0.98	1.20	1.11	1.38					269	31.6	4.89
Q9NSP4	Centromere protein M OS=Homo sapiens GN=CENPM PE=1 SV=1 - [CENPM_HUMAN]	7.22	1	1	1	1					0.58	0.62	0.54	0.58	180	19.7	7.20
Q96H22	Centromere protein N OS=Homo sapiens GN=CENPN PE=1 SV=2 - [CENPN_HUMAN]	3.24	1	1	1	1					0.68	0.71	1.02	1.07	339	39.5	9.13
Q7L2Z9	Centromere protein Q OS=Homo sapiens GN=CENPQ PE=1 SV=1 - [CENPQ_HUMAN]	7.46	1	2	2	2	1.14	1.19	1.00	1.05	1.34	1.43	1.37	1.47	268	30.6	9.42
Q13352	Centromere protein R OS=Homo sapiens GN=ITGB3BP PE=1 SV=2 - [CENPR_HUMAN]	12.43	1	1	1	1	0.12	0.08	0.22	0.17					177	20.2	9.07
Q96BT3	Centromere protein T OS=Homo sapiens GN=CENPT PE=1 SV=2 - [CENPT_HUMAN]	3.21	1	1	1	1	1.04	1.08	0.96	1.01					561	60.4	6.58
Q7Z7K6	Centromere protein V OS=Homo sapiens GN=CENPV PE=1 SV=1 - [CENPV_HUMAN]	4.73	1	1	1	1	1.34	1.09	1.18	0.97					275	29.9	9.73
O43264	Centromere/kinetochore protein zw10 homolog OS=Homo sapiens GN=ZW10 PE=1 SV=3 - [ZW10_HUMAN]	3.59	2	1	2	2					1.17	1.23	1.02	1.07	779	88.8	6.27
Q9C0D2	Centrosomal protein KIAA1731 OS=Homo sapiens GN=KIAA1731 PE=2 SV=4 - [K1731_HUMAN]	3.00	1	2	3	3	0.74		0.58						2601	295.0	6.00
Q8N8E3	Centrosomal protein of 112 kDa OS=Homo sapiens GN=CEP112 PE=1 SV=2 - [CE112_HUMAN]	2.93	1	2	2	3	0.42	0.25	0.77	0.45	0.96	0.70	1.08	0.79	955	112.7	6.68
Q66GS9	Centrosomal protein of 135 kDa OS=Homo sapiens GN=CEP135 PE=1 SV=2 - [CP135_HUMAN]	0.96	1	1	1	1					0.97	1.68	1.14	1.97	1140	133.4	6.21
O94986	Centrosomal protein of 152 kDa OS=Homo sapiens GN=CEP152 PE=1 SV=3 - [CE152_HUMAN]	1.21	1	1	1	1					1.00	0.86	0.85	0.72	1654	189.0	5.44
Q9UPV0	Centrosomal protein of 164 kDa OS=Homo sapiens GN=CEP164 PE=1 SV=3 - [CE164_HUMAN]	2.19	1	2	2	2	0.96	0.71	1.26	0.94					1460	164.2	5.36
Q5SW79	Centrosomal protein of 170 kDa OS=Homo sapiens GN=CEP170 PE=1 SV=1 - [CE170_HUMAN]	2.97	1	2	2	3	0.78	0.96	0.66	0.81					1584	175.2	7.11
Q8NHQ1	Centrosomal protein of 70 kDa OS=Homo sapiens GN=CEP70 PE=1 SV=2 - [CEP70_HUMAN]	1.51	1	1	1	2					0.80	0.70	0.98	0.86	597	69.7	5.71
Q6P2H3	Centrosomal protein of 85 kDa OS=Homo sapiens GN=CEP85 PE=1 SV=1 - [CEP85_HUMAN]	6.82	1	2	2	4	0.94	0.92	1.06	1.04					762	85.6	6.00
Q96ST8	Centrosomal protein of 89 kDa OS=Homo sapiens GN=CEP89 PE=1 SV=3 - [CEP89_HUMAN]	1.40	1	1	1	2	1.16	1.17	1.13	1.14					783	89.5	6.80
Q96GE4	Centrosomal protein of 95 kDa OS=Homo sapiens GN=CEP95 PE=1 SV=1 - [CEP95_HUMAN]	2.80	1	2	2	2					1.24	1.22	1.13	1.12	821	95.2	8.59
Q8IW35	Centrosomal protein of 97 kDa OS=Homo sapiens GN=CEP97 PE=1 SV=1 - [CEP97_HUMAN]	2.89	1	1	1	1					0.56	0.43	1.05	0.79	865	96.9	5.02
Q8NA72	Centrosomal protein POC5 OS=Homo sapiens GN=POC5 PE=1 SV=2 - [POC5_HUMAN]	8.17	1	2	2	2	0.63		0.93		1.13	1.16	1.27	1.30	575	63.3	7.42
Q5VT06	Centrosome-associated protein 350 OS=Homo sapiens GN=CEP350 PE=1 SV=1 - [CE350_HUMAN]	4.04	14	5	8	59	0.65	0.84	0.75	0.96	1.40	1.21	1.14	0.99	3117	350.7	6.33
Q9BV73	Centrosome-associated protein CEP250 OS=Homo sapiens GN=CEP250 PE=1 SV=2 - [CP250_HUMAN]	3.03	1	6	6	7	0.90	0.88	1.02	1.01	3.07	1.30	1.49	0.91	2442	281.0	5.02
Q16739	Ceramide glucosyltransferase OS=Homo sapiens GN=UGCG PE=1 SV=1 - [CEGT_HUMAN]	3.05	1	1	1	4					0.78	0.90	0.86	0.99	394	44.8	7.78
Q49MI3	Ceramide kinase-like protein OS=Homo sapiens GN=CERKL PE=1 SV=1 - [CERKL_HUMAN]	1.61	1	1	1	2	0.78	0.93	0.89	1.08					558	62.6	8.27
Q9HA82	Ceramide synthase 4 OS=Homo sapiens GN=CERS4 PE=1 SV=2 - [CERS4_HUMAN]	3.30	1	1	1	1					2.53	2.74	2.34	2.53	394	46.4	9.07
Q8IUK8	Cerebellin-2 OS=Homo sapiens GN=CBLN2 PE=2 SV=1 - [CBLN2_HUMAN]	5.80	1	1	1	1	1.11	1.35	1.01	1.23					224	24.1	8.48
Q9NTU7	Cerebellin-4 OS=Homo sapiens GN=CBLN4 PE=1 SV=1 - [CBLN4_HUMAN]	16.42	1	3	3	7	0.93	0.91	0.87	0.90					201	21.8	8.73

P00450	Ceruloplasmin OS=Homo sapiens GN=CP PE=1 SV=1 - [CERU_HUMAN]	60.56	1	63	63	3655	1.12	1.16	1.12	1.17	1.20	1.21	1.11	1.18	1065	122.1	5.72
P29973	cGMP-gated cation channel alpha-1 OS=Homo sapiens GN=CNGA1 PE=1 SV=3 - [CNGA1_HUMAN]	8.70	1	2	2	10	1.64	1.73	1.77	1.82					690	79.5	7.81
Q14432	cGMP-inhibited 3',5'-cyclic phosphodiesterase A OS=Homo sapiens GN=PDE3A PE=1 SV=3 - [PDE3A_HUMAN]	0.96	1	1	1	3					2.75	2.65	2.36	2.28	1141	124.9	6.00
O76074	cGMP-specific 3',5'-cyclic phosphodiesterase OS=Homo sapiens GN=PDE5A PE=1 SV=2 - [PDE5A_HUMAN]	1.71	4	1	2	10					1.42	1.51	1.41	1.49	875	99.9	6.09
Q7LBR1	Charged multivesicular body protein 1b OS=Homo sapiens GN=CHMP1B PE=1 SV=1 - [CHM1B_HUMAN]	2.51	1	1	1	1					0.86	0.66	0.91	0.69	199	22.1	8.10
Q8WUX9	Charged multivesicular body protein 7 OS=Homo sapiens GN=CHMP7 PE=1 SV=1 - [CHMP7_HUMAN]	1.99	1	1	1	1					1.19	1.35	1.24	1.41	453	50.9	5.35
P36222	Chitinase-3-like protein 1 OS=Homo sapiens GN=CHI3L1 PE=1 SV=2 - [CH3L1_HUMAN]	1.83	1	1	1	1					1.00	0.82	0.94	0.77	383	42.6	8.46
Q15782	Chitinase-3-like protein 2 OS=Homo sapiens GN=CHI3L2 PE=1 SV=1 - [CH3L2_HUMAN]	6.15	1	2	2	2	1.82	1.93	1.80	1.91	0.58	0.47	0.76	0.61	390	43.5	7.53
Q13231	Chitotriosidase-1 OS=Homo sapiens GN=CHIT1 PE=1 SV=1 - [CHIT1_HUMAN]	6.87	1	2	2	6	1.27	1.27	1.26	1.27		0.81		1.33	466	51.6	6.96
O00299	Chloride intracellular channel protein 1 OS=Homo sapiens GN=CLIC1 PE=1 SV=4 - [CLIC1_HUMAN]	32.37	1	7	7	24	0.84	0.85	0.79	0.81	0.87	0.90	0.89	0.88	241	26.9	5.17
Q96NY7	Chloride intracellular channel protein 6 OS=Homo sapiens GN=CLIC6 PE=2 SV=3 - [CLIC6_HUMAN]	9.23	1	2	2	2	0.77	1.57	0.77	1.12					704	73.0	4.37
P51797	Chloride transport protein 6 OS=Homo sapiens GN=CLCN6 PE=1 SV=2 - [CLCN6_HUMAN]	1.15	1	1	1	1	1.17	1.11	1.16	1.11					869	97.2	6.81
P32238	Cholecystokinin receptor type A OS=Homo sapiens GN=CCKAR PE=1 SV=1 - [CCKAR_HUMAN]	7.94	1	1	1	1					0.50	0.51	0.46	0.47	428	47.8	9.20
P11597	Cholesteryl ester transfer protein OS=Homo sapiens GN=CETP PE=1 SV=2 - [CETP_HUMAN]	23.73	1	11	11	73	1.10	1.22	1.06	1.20	1.08	0.87	1.00	0.87	493	54.7	6.09
P28329	Choline O-acetyltransferase OS=Homo sapiens GN=CHAT PE=1 SV=4 - [CLAT_HUMAN]	2.01	9	1	2	4					0.81	0.71	0.77	0.68	748	82.5	8.59
P06276	Cholinesterase OS=Homo sapiens GN=BCHE PE=1 SV=1 - [CHLE_HUMAN]	27.57	1	14	16	75	1.11	1.13	1.11	1.14	1.26	1.33	1.19	1.24	602	68.4	7.42
O15335	Chondroadherin OS=Homo sapiens GN=CHAD PE=2 SV=2 - [CHAD_HUMAN]	3.62	1	1	1	1					1.29	1.35	1.23	1.28	359	40.5	9.39
Q8TDX6	Chondroitin sulfate N-acetylgalactosaminyltransferase 1 OS=Homo sapiens GN=CSGALNACT1 PE=1 SV=2 - [CGAT1_HUMAN]	1.13	1	1	1	1					4.83	5.07	4.12	4.31	532	61.3	8.41
Q6UVK1	Chondroitin sulfate proteoglycan 4 OS=Homo sapiens GN=CSPG4 PE=1 SV=2 - [CSPG4_HUMAN]	3.45	1	5	5	9	0.91	0.95	0.88	0.88	0.71	0.61	0.73	0.63	2322	250.4	5.47
O95196	Chondroitin sulfate proteoglycan 5 OS=Homo sapiens GN=CSPG5 PE=1 SV=3 - [CSPG5_HUMAN]	5.30	1	2	2	2	1.63	1.70	1.83	1.91	0.74	0.56	0.77	0.59	566	60.0	4.50
Q9H9P2	Chondrolectin OS=Homo sapiens GN=CHODL PE=2 SV=2 - [CHODL_HUMAN]	2.56	1	1	1	1					1.21	0.93	1.26	0.96	273	30.4	6.83
Q9Y5P2	Chondrosarcoma-associated gene 2/3 protein OS=Homo sapiens GN=CSAG2 PE=2 SV=2 - [CSAG2_HUMAN]	17.32	2	2	2	23	0.95	1.03	1.02	0.84	1.20	1.45	1.12	1.27	127	14.4	10.32
Q9BU40	Chordin-like protein 1 OS=Homo sapiens GN=CHRDL1 PE=2 SV=1 - [CRDL1_HUMAN]	2.22	1	1	1	2	0.94	1.09	1.12	1.30					450	51.1	8.13
Q9NP62	Chorion-specific transcription factor GCMa OS=Homo sapiens GN=GCM1 PE=2 SV=1 - [GCM1_HUMAN]	2.52	1	1	1	4	0.91	1.03	1.07	1.22	1.23	1.03	1.26	1.05	436	49.2	7.02
P01243	Chorionic somatomammotropin hormone OS=Homo sapiens GN=CSH1 PE=1 SV=2 - [CSH_HUMAN]	23.96	2	2	5	31					2.16	3.44	2.33	3.61	217	25.0	5.59
Q13111	Chromatin assembly factor 1 subunit A OS=Homo sapiens GN=CHAF1A PE=1 SV=2 - [CAF1A_HUMAN]	5.02	1	2	2	3	1.49	1.76	1.47	1.74					956	106.9	5.94
Q14781	Chromobox protein homolog 2 OS=Homo sapiens GN=CBX2 PE=1 SV=2 - [CBX2_HUMAN]	4.32	1	1	1	1					0.87	0.77	0.98	0.87	532	56.0	10.01

O14646	Chromodomain-helicase-DNA-binding protein 1 OS=Homo sapiens GN=CHD1 PE=1 SV=2 - [CHD1_HUMAN]	1.46	1	1	2	7					0.49	0.65	1.14	1.30	1710	196.6	7.14
Q86WJ1	Chromodomain-helicase-DNA-binding protein 1-like OS=Homo sapiens GN=CHD1L PE=1 SV=2 - [CHD1L_HUMAN]	1.23	1	1	1	3	2.24	2.28	2.59	2.64					897	100.9	6.90
O14647	Chromodomain-helicase-DNA-binding protein 2 OS=Homo sapiens GN=CHD2 PE=1 SV=2 - [CHD2_HUMAN]	2.30	1	2	3	7	1.38	1.30	1.35	1.29	1.30	1.32	1.11	1.13	1828	211.2	8.10
Q8TD26	Chromodomain-helicase-DNA-binding protein 6 OS=Homo sapiens GN=CHD6 PE=1 SV=4 - [CHD6_HUMAN]	2.80	9	3	4	5	1.16	1.04	0.93	0.87	1.11	1.01	0.78	0.71	2715	305.2	6.27
Q9P2D1	Chromodomain-helicase-DNA-binding protein 7 OS=Homo sapiens GN=CHD7 PE=1 SV=3 - [CHD7_HUMAN]	0.57	9	1	2	3	1.72	1.74	1.60	1.63	0.34	0.40	0.43	0.51	2997	335.7	6.34
Q9HCK8	Chromodomain-helicase-DNA-binding protein 8 OS=Homo sapiens GN=CHD8 PE=1 SV=5 - [CHD8_HUMAN]	0.74	9	1	2	7					1.35	1.54	1.10	1.27	2581	290.3	6.47
Q3L8U1	Chromodomain-helicase-DNA-binding protein 9 OS=Homo sapiens GN=CHD9 PE=1 SV=2 - [CHD9_HUMAN]	0.90	9	1	2	3	1.05	0.83	0.80	0.64	1.08	1.20	1.20	1.33	2897	325.8	7.01
P10645	Chromogranin-A OS=Homo sapiens GN=CHGA PE=1 SV=7 - [CMGA_HUMAN]	4.16	1	1	1	6					2.05	2.07	1.96	1.97	457	50.7	4.60
Q8WVB6	Chromosome transmission fidelity protein 18 homolog OS=Homo sapiens GN=CHTF18 PE=1 SV=1 - [CTF18_HUMAN]	1.74	1	1	1	1					0.87	0.90	1.07	1.11	975	107.3	7.21
O95239	Chromosome-associated kinesin KIF4A OS=Homo sapiens GN=KIF4A PE=1 SV=3 - [KIF4A_HUMAN]	1.95	1	1	2	3					0.73	0.82	0.63	0.71	1232	139.8	6.27
P08861	Chymotrypsin-like elastase family member 3B OS=Homo sapiens GN=CELA3B PE=1 SV=3 - [CEL3B_HUMAN]	8.15	1	1	1	1					0.87	1.02	1.08	1.27	270	29.2	6.28
P40313	Chymotrypsin-like protease CTRL-1 OS=Homo sapiens GN=CTRL PE=2 SV=1 - [CTRL_HUMAN]	7.58	1	1	1	1	1.85	1.89	1.17	1.20					264	28.0	8.19
Q9P2M7	Cingulin OS=Homo sapiens GN=CGN PE=1 SV=2 - [CING_HUMAN]	2.09	2	2	3	13	0.74	0.53	0.73	0.53	0.91	1.00	0.97	0.90	1197	136.3	5.54
O14578	Citron Rho-interacting kinase OS=Homo sapiens GN=CIT PE=1 SV=2 - [CTRO_HUMAN]	1.43	1	2	3	9	1.73	2.27	1.25	1.64	0.92	0.81	1.03	0.90	2027	231.3	6.57
Q8IZ96	CKLF-like MARVEL transmembrane domain-containing protein 1 OS=Homo sapiens GN=CMTM1 PE=2 SV=1 - [CKLF1_HUMAN]	17.16	1	1	1	1	1.35	1.03	1.57	1.20					169	18.6	6.99
O14503	Class E basic helix-loop-helix protein 40 OS=Homo sapiens GN=BHLHE40 PE=1 SV=1 - [BHE40_HUMAN]	3.88	1	1	1	1	0.67	0.63	0.68	0.64					412	45.5	8.10
O60641	Clathrin coat assembly protein AP180 OS=Homo sapiens GN=SNAP91 PE=1 SV=2 - [AP180_HUMAN]	2.87	1	2	2	2					1.17	1.44	0.99	1.21	907	92.4	4.86
P53675	Clathrin heavy chain 2 OS=Homo sapiens GN=CLTCL1 PE=1 SV=2 - [CLH2_HUMAN]	0.85	2	1	1	1	0.40	0.38	0.40	0.38					1640	186.9	5.85
P78369	Claudin-10 OS=Homo sapiens GN=CLDN10 PE=1 SV=2 - [CLD10_HUMAN]	6.14	1	2	2	2					1.08	0.81	0.91	0.68	228	24.5	8.03
P56749	Claudin-12 OS=Homo sapiens GN=CLDN12 PE=1 SV=1 - [CLD12_HUMAN]	6.15	1	1	1	1					0.98	1.18	2.77	3.31	244	27.1	8.46
Q9UKF6	Cleavage and polyadenylation specificity factor subunit 3 OS=Homo sapiens GN=CPSF3 PE=1 SV=1 - [CPSF3_HUMAN]	2.49	1	1	1	1	0.72	0.78	0.98	1.07					684	77.4	5.60
O96005	Cleft lip and palate transmembrane protein 1 OS=Homo sapiens GN=CLPTM1 PE=1 SV=1 - [CLPT1_HUMAN]	5.83	1	2	2	2	1.22	1.31	1.13	1.22					669	76.0	6.30
Q7Z460	CLIP-associating protein 1 OS=Homo sapiens GN=CLASP1 PE=1 SV=1 - [CLAP1_HUMAN]	2.54	1	2	2	4	1.88	1.38	1.61	1.20					1538	169.3	9.03
O75122	CLIP-associating protein 2 OS=Homo sapiens GN=CLASP2 PE=1 SV=2 - [CLAP2_HUMAN]	0.93	2	2	2	6	1.17	1.17	1.20	1.21					1294	141.0	8.47
P10909	Clusterin OS=Homo sapiens GN=CLU PE=1 SV=1 - [CLUS_HUMAN]	46.77	1	23	23	1479	1.03	1.01	1.01	0.98	0.81	0.81	0.81	0.83	449	52.5	6.27
Q96AJ1	Clusterin-associated protein 1 OS=Homo sapiens GN=CLUAP1 PE=1 SV=4 - [CLUA1_HUMAN]	2.91	1	1	1	1					0.63	0.90	0.51	0.73	413	48.1	4.77
Q11201	CMP-N-acetylneuraminate-beta-galactosamide-alpha-2,3-sialyltransferase 1 OS=Homo sapiens GN=ST3GAL1 PE=2 SV=1 - [SIA4A_HUMAN]	4.41	1	1	1	1	0.71	0.76	1.06	1.14					340	39.1	9.09

P78382	CMP-sialic acid transporter OS=Homo sapiens GN=SLC35A1 PE=2 SV=1 - [S35A1_HUMAN]	1.78	1	1	1	1					1.68	2.37	1.16	1.63	337	36.8	8.95
Q14019	Coactosin-like protein OS=Homo sapiens GN=COTL1 PE=1 SV=3 - [COTL1_HUMAN]	38.03	1	7	7	31	0.97	1.10	0.95	1.10	1.56	1.48	1.31	1.33	142	15.9	5.67
P00740	Coagulation factor IX OS=Homo sapiens GN=F9 PE=1 SV=2 - [FA9_HUMAN]	37.96	1	14	15	101	1.15	1.23	1.14	1.28	0.84	0.80	0.87	0.85	461	51.7	5.47
P12259	Coagulation factor V OS=Homo sapiens GN=F5 PE=1 SV=4 - [FA5_HUMAN]	21.58	1	42	42	307	1.04	1.04	1.05	1.08	1.08	1.13	1.08	1.14	2224	251.5	6.05
P08709	Coagulation factor VII OS=Homo sapiens GN=F7 PE=1 SV=1 - [FA7_HUMAN]	25.11	1	8	8	77	1.11	1.20	1.02	1.11	0.89	0.87	0.94	0.91	466	51.6	7.23
P00451	Coagulation factor VIII OS=Homo sapiens GN=F8 PE=1 SV=1 - [FA8_HUMAN]	3.15	1	5	5	7	0.94	0.87	0.98	0.91	0.73	0.64	1.00	0.88	2351	266.8	7.36
P00742	Coagulation factor X OS=Homo sapiens GN=F10 PE=1 SV=2 - [FA10_HUMAN]	37.70	1	18	18	133	1.17	1.20	1.09	1.16	0.87	0.86	0.86	0.91	488	54.7	5.94
P03951	Coagulation factor XI OS=Homo sapiens GN=F11 PE=1 SV=1 [FA11_HUMAN]	43.04	2	23	23	99	1.03	1.02	1.09	1.09	1.07	1.06	1.05	0.97	625	70.1	8.10
P00748	Coagulation factor XII OS=Homo sapiens GN=F12 PE=1 SV=3 - [FA12_HUMAN]	40.33	1	26	26	451	1.32	1.27	1.33	1.32	1.05	0.98	1.08	1.02	615	67.7	7.74
P00488	Coagulation factor XIII A chain OS=Homo sapiens GN=F13A1 PE=1 SV=4 - [F13A_HUMAN]	33.88	2	19	19	70	0.74	0.72	0.79	0.78	3.06	1.77	2.76	1.56	732	83.2	6.09
P05160	Coagulation factor XIII B chain OS=Homo sapiens GN=F13B PE=1 SV=3 - [F13B_HUMAN]	43.72	1	24	24	187	1.17	1.13	1.16	1.13	0.91	0.92	0.92	0.94	661	75.5	6.39
P53621	Coatomer subunit alpha OS=Homo sapiens GN=COPA PE=1 SV=2 - [COPA_HUMAN]	2.04	2	1	3	5					1.06	0.84	1.01	0.80	1224	138.3	7.66
P53618	Coatomer subunit beta OS=Homo sapiens GN=COPB1 PE=1 SV=3 - [COPB_HUMAN]	3.46	7	2	3	7	0.85	0.79	0.84	0.79	0.77	0.97	0.95	1.20	953	107.1	6.05
P35606	Coatomer subunit beta' OS=Homo sapiens GN=COPB2 PE=1 SV=2 - [COPB2_HUMAN]	4.75	1	2	2	2	1.05	1.44	1.03	1.41	1.11	1.03	0.81	0.74	906	102.4	5.27
P48444	Coatomer subunit delta OS=Homo sapiens GN=ARCN1 PE=1 SV=1 - [COPD_HUMAN]	7.05	1	3	3	8					1.17	1.13	1.21	1.17	511	57.2	6.21
Q9P299	Coatomer subunit zeta-2 OS=Homo sapiens GN=COPZ2 PE=2 SV=1 - [COPZ2_HUMAN]	9.05	1	1	1	1	0.75	0.65	0.76	0.66					210	23.5	5.17
Q8IWY9	Codanin-1 OS=Homo sapiens GN=CDAN1 PE=1 SV=4 - [CDAN1_HUMAN]	4.24	1	2	2	2				0.29	0.79	0.95	0.82	0.99	1227	134.0	6.77
Q96MF6	Coenzyme Q-binding protein COQ10 homolog A, mitochondrial OS=Homo sapiens GN=COQ10A PE=2 SV=2 - [CQ10A_HUMAN]	4.05	1	1	1	1					1.92	1.63	1.65	1.40	247	27.7	9.76
P23528	Cofilin-1 OS=Homo sapiens GN=CFL1 PE=1 SV=3 - [COF1_HUMAN]	55.42	2	9	10	33	0.75	0.76	0.77	0.75	0.72	0.75	0.71	0.70	166	18.5	8.09
Q8WVM7	Cohesin subunit SA-1 OS=Homo sapiens GN=STAG1 PE=1 SV=3 - [STAG1_HUMAN]	1.11	1	2	2	2	1.34	1.01	1.16	0.88					1258	144.3	5.59
Q9UJ98	Cohesin subunit SA-3 OS=Homo sapiens GN=STAG3 PE=1 SV=2 - [STAG3_HUMAN]	2.04	1	1	1	2					1.45	1.15	1.60	1.27	1225	138.9	5.39
Q8TD31	Coiled-coil alpha-helical rod protein 1 OS=Homo sapiens GN=CCHCR1 PE=1 SV=2 - [CCHCR_HUMAN]	1.41	1	1	1	1	2.59	2.35	1.13	1.02					782	88.6	5.95
Q6P1N0	Coiled-coil and C2 domain-containing protein 1A OS=Homo sapiens GN=CC2D1A PE=1 SV=1 - [C2D1A_HUMAN]	3.05	1	2	2	2	0.89	0.90	0.96	0.97	0.76	0.77	0.78	0.78	951	104.0	8.09
Q9P2K1	Coiled-coil and C2 domain-containing protein 2A OS=Homo sapiens GN=CC2D2A PE=1 SV=3 - [C2D2A_HUMAN]	0.74	1	1	1	2					1.38	1.08	1.40	1.09	1620	186.1	6.74
Q8IYK2	Coiled-coil domain-containing protein 105 OS=Homo sapiens GN=CCDC105 PE=2 SV=3 - [CC105_HUMAN]	1.80	1	1	1	1					1.09	1.16	1.08	1.15	499	56.9	9.86
Q6ZU64	Coiled-coil domain-containing protein 108 OS=Homo sapiens GN=CCDC108 PE=2 SV=2 - [CC108_HUMAN]	0.62	1	1	1	1					0.71	0.78	0.93	1.02	1925	217.1	6.51
Q96M91	Coiled-coil domain-containing protein 11 OS=Homo sapiens GN=CCDC11 PE=2 SV=2 - [CCD11_HUMAN]	1.56	1	1	1	1					0.75	1.23	0.58	0.95	514	61.8	8.90

Q8TBZ0	Coiled-coil domain-containing protein 110 OS=Homo sapiens GN=CCDC110 PE=2 SV=1 - [CC110_HUMAN]	3.36	1	3	3	10	0.97	0.90	0.99	0.94					833	96.7	6.24
Q8NEF3	Coiled-coil domain-containing protein 112 OS=Homo sapiens GN=CCDC112 PE=2 SV=2 - [CC112_HUMAN]	2.91	1	2	2	3		1.36		1.79	0.32	0.30	0.88	0.84	446	53.5	9.48
Q96NT0	Coiled-coil domain-containing protein 115 OS=Homo sapiens GN=CCDC115 PE=1 SV=1 - [CC115_HUMAN]	3.89	1	1	1	7	0.60	0.32	0.71	0.38	0.73	0.48	0.82	0.63	180	19.7	6.95
Q5T0U0	Coiled-coil domain-containing protein 122 OS=Homo sapiens GN=CCDC122 PE=1 SV=1 - [CC122_HUMAN]	4.76	1	1	1	1					0.71	0.77	0.82	0.89	273	32.2	7.01
Q86Z20	Coiled-coil domain-containing protein 125 OS=Homo sapiens GN=CCDC125 PE=2 SV=2 - [CC125_HUMAN]	1.96	1	1	1	1					0.64	0.42	0.73	0.47	511	58.6	7.24
Q6ZRS4	Coiled-coil domain-containing protein 129 OS=Homo sapiens GN=CCDC129 PE=2 SV=2 - [CC129_HUMAN]	0.57	1	1	1	1	1.02	1.12	1.02	1.13					1044	115.3	5.31
Q8IYE1	Coiled-coil domain-containing protein 13 OS=Homo sapiens GN=CCDC13 PE=2 SV=2 - [CCD13_HUMAN]	3.08	1	2	2	2					1.20	1.23	1.18	1.21	715	80.8	8.84
Q96JN2	Coiled-coil domain-containing protein 136 OS=Homo sapiens GN=CCDC136 PE=2 SV=3 - [CC136_HUMAN]	1.13	1	1	1	1					0.90	1.00	0.95	1.06	1154	134.0	4.65
Q96M89	Coiled-coil domain-containing protein 138 OS=Homo sapiens GN=CCDC138 PE=1 SV=1 - [CC138_HUMAN]	2.71	1	1	1	3	0.68	0.84	0.92	1.15					665	76.2	8.53
Q6ZP82	Coiled-coil domain-containing protein 141 OS=Homo sapiens GN=CCDC141 PE=1 SV=1 - [CC141_HUMAN]	1.14	1	1	1	1	0.90	1.03	1.02	1.18					875	100.5	5.63
Q8IYE0	Coiled-coil domain-containing protein 146 OS=Homo sapiens GN=CCDC146 PE=2 SV=2 - [CC146_HUMAN]	1.05	1	1	1	1					1.53	0.91	1.22	0.72	955	112.7	8.48
Q5T655	Coiled-coil domain-containing protein 147 OS=Homo sapiens GN=CCDC147 PE=2 SV=1 - [CC147_HUMAN]	2.98	1	1	2	2					0.73	0.81	0.67	0.74	872	103.4	8.41
A6NI56	Coiled-coil domain-containing protein 154 OS=Homo sapiens GN=CCDC154 PE=2 SV=4 - [CC154_HUMAN]	1.04	1	1	1	2					0.54	0.83	0.62	0.95	674	76.0	8.38
Q8N6L0	Coiled-coil domain-containing protein 155 OS=Homo sapiens GN=CCDC155 PE=2 SV=2 - [CC155_HUMAN]	3.91	1	2	2	2					0.70	1.05	0.71	1.07	562	62.7	4.68
Q569K6	Coiled-coil domain-containing protein 157 OS=Homo sapiens GN=CCDC157 PE=2 SV=3 - [CC157_HUMAN]	1.86	1	1	1	3	0.44	1.01	0.47	1.09					752	83.9	7.15
Q5M9N0	Coiled-coil domain-containing protein 158 OS=Homo sapiens GN=CCDC158 PE=2 SV=2 - [CD158_HUMAN]	3.23	1	2	3	4					4.07	9.08	6.56	####	1113	127.1	6.46
A2VCL2	Coiled-coil domain-containing protein 162 OS=Homo sapiens GN=CCDC162P PE=2 SV=3 - [CC162_HUMAN]	2.98	1	1	1	1					1.14	0.99	1.33	1.14	907	103.8	7.90
Q9Y4B5	Coiled-coil domain-containing protein 165 OS=Homo sapiens GN=CCDC165 PE=1 SV=5 - [CC165_HUMAN]	1.57	1	2	2	3	0.52	0.63	0.21	0.26	0.45	0.46	0.46	0.47	1905	209.4	6.43
Q9P0B6	Coiled-coil domain-containing protein 167 OS=Homo sapiens GN=CCDC167 PE=2 SV=2 - [CC167_HUMAN]	15.46	1	1	1	1	1.41	1.79	1.08	1.38					97	11.5	9.50
Q8NDH2	Coiled-coil domain-containing protein 168 OS=Homo sapiens GN=CCDC168 PE=2 SV=2 - [CC168_HUMAN]	0.49	1	1	1	1	1.07	1.65	0.92	1.43					2452	277.8	9.31
Q6TFL3	Coiled-coil domain-containing protein 171 OS=Homo sapiens GN=CCDC171 PE=2 SV=1 - [CC171_HUMAN]	2.34	1	2	2	3	1.03	1.10	1.04	1.12					1326	152.7	6.81
Q5T9S5	Coiled-coil domain-containing protein 18 OS=Homo sapiens GN=CCDC18 PE=1 SV=1 - [CCD18_HUMAN]	1.86	1	2	2	4	0.85	0.82	0.91	0.89	0.75	0.86	0.69	0.79	1454	168.9	5.66
Q8N4L8	Coiled-coil domain-containing protein 24 OS=Homo sapiens GN=CCDC24 PE=2 SV=1 - [CCD24_HUMAN]	5.54	1	1	1	9					1.83	2.07	1.73	1.98	307	34.3	7.02
Q8IWP9	Coiled-coil domain-containing protein 28A OS=Homo sapiens GN=CCDC28A PE=1 SV=1 - [CC28A_HUMAN]	5.11	1	1	1	2	1.14	1.23	1.21	1.31					274	30.3	9.25
Q9BUN5	Coiled-coil domain-containing protein 28B OS=Homo sapiens GN=CCDC28B PE=1 SV=2 - [CC28B_HUMAN]	7.50	1	1	1	13	0.73	0.88	0.75	0.91	0.80	0.64	0.87	0.59	200	22.0	5.36
Q8N5R6	Coiled-coil domain-containing protein 33 OS=Homo sapiens GN=CCDC33 PE=1 SV=3 - [CCD33_HUMAN]	2.30	1	2	2	2	0.88	0.37	0.93	0.40	0.93	0.89	0.80	0.76	958	107.1	6.77
Q494V2	Coiled-coil domain-containing protein 37 OS=Homo sapiens GN=CCDC37 PE=1 SV=1 - [CCD37_HUMAN]	5.07	1	3	3	5	1.80	1.74	1.74	1.58	0.27	0.25	0.23	0.22	611	71.1	7.11

Q502W7	Coiled-coil domain-containing protein 38 OS=Homo sapiens GN=CCDC38 PE=2 SV=1 - [CCD38_HUMAN]	1.60	1	1	1	1					1.13	0.99	1.00	0.87	563	65.3	8.75
Q9UFE4	Coiled-coil domain-containing protein 39 OS=Homo sapiens GN=CCDC39 PE=2 SV=3 - [CCD39_HUMAN]	1.17	1	1	1	2					0.90	0.95	1.00	1.06	941	109.8	6.44
Q4G0X9	Coiled-coil domain-containing protein 40 OS=Homo sapiens GN=CCDC40 PE=2 SV=2 - [CCD40_HUMAN]	2.01	1	2	2	2	0.84	0.83	0.79	0.78	0.65	0.44	0.75	0.50	1142	130.0	5.29
Q8NEL0	Coiled-coil domain-containing protein 54 OS=Homo sapiens GN=CCDC54 PE=1 SV=2 - [CCD54_HUMAN]	2.44	1	1	1	2					0.99	0.93	1.00	0.94	328	37.9	8.37
Q2TAC2	Coiled-coil domain-containing protein 57 OS=Homo sapiens GN=CCDC57 PE=2 SV=2 - [CCD57_HUMAN]	2.07	1	1	2	6	1.36	1.32	1.14	1.11					916	103.1	6.55
A2RUB6	Coiled-coil domain-containing protein 66 OS=Homo sapiens GN=CCDC66 PE=1 SV=4 - [CCD66_HUMAN]	1.16	1	1	1	1					1.07	0.97	0.86	0.78	948	109.3	8.25
Q05D60	Coiled-coil domain-containing protein 67 OS=Homo sapiens GN=CCDC67 PE=2 SV=2 - [CCD67_HUMAN]	5.30	1	1	1	1		0.79		0.86					604	70.9	6.21
Q8IV32	Coiled-coil domain-containing protein 71 OS=Homo sapiens GN=CCDC71 PE=2 SV=3 - [CCD71_HUMAN]	2.14	1	1	1	1					0.95	0.17	0.68	0.12	467	49.6	11.80
Q8N9Z2	Coiled-coil domain-containing protein 71L OS=Homo sapiens GN=CCDC71L PE=2 SV=2 - [CC71L_HUMAN]	5.11	1	1	1	1	0.79	0.93	0.82	0.97					235	26.2	11.71
Q9Y2S6	Coiled-coil domain-containing protein 72 OS=Homo sapiens GN=CCDC72 PE=1 SV=1 - [CCD72_HUMAN]	12.50	1	1	1	1					1.85	1.98	1.57	1.68	64	7.1	9.99
Q6ZRK6	Coiled-coil domain-containing protein 73 OS=Homo sapiens GN=CCDC73 PE=1 SV=2 - [CCD73_HUMAN]	1.95	1	1	2	2	0.92	1.02	0.97	1.10					1079	124.1	5.58
Q76M96	Coiled-coil domain-containing protein 80 OS=Homo sapiens GN=CCDC80 PE=1 SV=1 - [CCD80_HUMAN]	2.95	1	1	1	2	1.03	0.85	1.13	1.02					950	108.1	9.72
Q96PX6	Coiled-coil domain-containing protein 85A OS=Homo sapiens GN=CCDC85A PE=2 SV=3 - [CC85A_HUMAN]	2.89	1	1	1	2					1.14	0.78	1.07	0.73	553	59.9	8.79
Q9NVE4	Coiled-coil domain-containing protein 87 OS=Homo sapiens GN=CCDC87 PE=2 SV=2 - [CCD87_HUMAN]	1.77	1	1	1	4	0.46	0.54	1.13	1.34					849	96.3	8.59
A6NC98	Coiled-coil domain-containing protein 88B OS=Homo sapiens GN=CCDC88B PE=1 SV=1 - [CC88B_HUMAN]	1.96	1	2	3	3	1.26	1.43	1.45	1.65					1476	164.7	5.11
Q8N998	Coiled-coil domain-containing protein 89 OS=Homo sapiens GN=CCDC89 PE=2 SV=1 - [CCD89_HUMAN]	5.35	1	2	2	6	1.21	1.20	1.31	1.31					374	43.8	5.36
Q53HC0	Coiled-coil domain-containing protein 92 OS=Homo sapiens GN=CCDC92 PE=2 SV=2 - [CCD92_HUMAN]	2.42	1	1	1	1					0.86	0.43	1.31	0.66	331	36.9	8.90
Q9BW85	Coiled-coil domain-containing protein 94 OS=Homo sapiens GN=CCDC94 PE=1 SV=1 - [CCD94_HUMAN]	4.02	1	1	1	1					0.90	1.43	1.05	1.69	323	37.1	5.92
Q96F63	Coiled-coil domain-containing protein 97 OS=Homo sapiens GN=CCDC97 PE=1 SV=1 - [CCD97_HUMAN]	14.29	1	2	2	2					1.47	1.44	1.32	1.29	343	38.9	4.59
Q8IYT3	Coiled-coil domain-containing protein C6orf97 OS=Homo sapiens GN=C6orf97 PE=2 SV=3 - [CF097_HUMAN]	2.80	1	2	2	5	1.35	1.40	1.47	1.54	1.77	2.09	1.60	1.89	715	82.2	6.64
Q8NCU4	Coiled-coil domain-containing protein KIAA1407 OS=Homo sapiens GN=KIAA1407 PE=2 SV=1 - [K1407_HUMAN]	1.28	1	1	1	1	0.64	0.74	0.70	0.82					936	110.5	9.60
P38432	Coilin OS=Homo sapiens GN=COIL PE=1 SV=1 - [COIL_HUMAN]	2.60	1	1	1	1					0.80	0.28	0.95	0.33	576	62.6	9.07
P02452	Collagen alpha-1(I) chain OS=Homo sapiens GN=COL1A1 PE=1 SV=5 - [CO1A1_HUMAN]	2.66	1	4	4	9	0.92	0.77	0.93	0.78	0.87	0.75	0.98	0.77	1464	138.9	5.80
P02458	Collagen alpha-1(II) chain OS=Homo sapiens GN=COL2A1 PE=1 SV=3 - [CO2A1_HUMAN]	1.82	1	1	1	1	0.88	0.87	0.98	0.98					1487	141.7	6.92
P20849	Collagen alpha-1(IX) chain OS=Homo sapiens GN=COL9A1 PE=1 SV=3 - [CO9A1_HUMAN]	1.63	1	1	1	1	1.10	1.12	1.09	1.12					921	91.8	8.72
P20908	Collagen alpha-1(V) chain OS=Homo sapiens GN=COL5A1 PE=1 SV=3 - [CO5A1_HUMAN]	2.07	1	3	3	6	1.24	1.38	1.37	1.54	0.86	0.84	0.88	0.86	1838	183.4	5.06
P12109	Collagen alpha-1(VI) chain OS=Homo sapiens GN=COL6A1 PE=1 SV=3 - [CO6A1_HUMAN]	4.47	1	3	3	5	1.40	1.30	1.23	1.14					1028	108.5	5.43

Q5TAT6	Collagen alpha-1(XIII) chain OS=Homo sapiens GN=COL13A1 PE=1 SV=1 - [CODA1_HUMAN]	2.37	1	1	1	1	1.19	1.32	1.28	1.42					717	69.9	9.17
Q14993	Collagen alpha-1(XIX) chain OS=Homo sapiens GN=COL19A1 PE=1 SV=3 - [COJA1_HUMAN]	0.70	1	1	1	1	1.13	1.26	1.41	1.59					1142	115.1	8.32
P39059	Collagen alpha-1(XV) chain OS=Homo sapiens GN=COL15A1 PE=1 SV=2 - [COFA1_HUMAN]	1.59	1	2	2	4					0.94	1.03	0.96	1.02	1388	141.6	5.00
Q07092	Collagen alpha-1(XVI) chain OS=Homo sapiens GN=COL16A1 PE=1 SV=2 - [COGA1_HUMAN]	2.62	1	2	2	4					2.01	1.82	1.43	1.29	1604	157.7	7.84
Q9UMD9	Collagen alpha-1(XVII) chain OS=Homo sapiens GN=COL17A1 PE=1 SV=3 - [COHA1_HUMAN]	1.20	1	1	1	1					1.58		2.11		1497	150.3	8.79
P39060	Collagen alpha-1(XVIII) chain OS=Homo sapiens GN=COL18A1 PE=1 SV=5 - [COIA1_HUMAN]	5.42	1	6	6	33	0.81	0.83	0.91	0.93	1.11	1.18	1.06	1.07	1754	178.1	6.01
Q8NFW1	Collagen alpha-1(XXII) chain OS=Homo sapiens GN=COL22A1 PE=1 SV=2 - [COMA1_HUMAN]	1.29	1	1	1	1							0.70		1626	161.0	7.23
Q86Y22	Collagen alpha-1(XXIII) chain OS=Homo sapiens GN=COL23A1 PE=2 SV=1 - [CONA1_HUMAN]	2.78	1	1	1	1					1.01	1.04	1.03	1.07	540	51.9	7.25
Q96A83	Collagen alpha-1(XXVI) chain OS=Homo sapiens GN=EMID2 PE=2 SV=1 - [EMID2_HUMAN]	3.17	1	1	1	1	0.94	1.02	0.95	1.04					441	45.4	7.31
Q2UY09	Collagen alpha-1(XXVIII) chain OS=Homo sapiens GN=COL28A1 PE=2 SV=2 - [COSA1_HUMAN]	2.04	1	1	1	1					1.28	2.20	0.71	1.22	1125	116.6	6.40
P08572	Collagen alpha-2(IV) chain OS=Homo sapiens GN=COL4A2 PE=1 SV=4 - [CO4A2_HUMAN]	1.99	1	1	1	1					0.69	1.15	1.06	1.77	1712	167.4	8.66
P05997	Collagen alpha-2(V) chain OS=Homo sapiens GN=COL5A2 PE=1 SV=3 - [CO5A2_HUMAN]	3.94	1	3	3	4	1.25	1.06	1.40	1.17					1499	144.8	6.46
P13942	Collagen alpha-2(XI) chain OS=Homo sapiens GN=COL11A2 PE=1 SV=5 - [COBA2_HUMAN]	1.50	1	1	1	1	0.57		1.01						1736	171.7	6.21
Q01955	Collagen alpha-3(IV) chain OS=Homo sapiens GN=COL4A3 PE=1 SV=3 - [CO4A3_HUMAN]	3.35	1	2	2	7	1.01	1.00	1.13	1.13	0.99	1.10	0.91	1.01	1670	161.7	9.16
Q14050	Collagen alpha-3(IX) chain OS=Homo sapiens GN=COL9A3 PE=2 SV=2 - [CO9A3_HUMAN]	4.39	1	2	2	2	1.23	1.35	1.35	1.50					684	63.6	7.68
P12111	Collagen alpha-3(VI) chain OS=Homo sapiens GN=COL6A3 PE=1 SV=5 - [CO6A3_HUMAN]	14.07	1	33	33	115	0.94	0.93	0.96	0.93	0.92	0.92	0.96	0.94	3177	343.5	6.68
A8TX70	Collagen alpha-5(VI) chain OS=Homo sapiens GN=COL6A5 PE=1 SV=1 - [CO6A5_HUMAN]	1.22	1	1	1	1					1.84	1.61	3.55	3.10	2615	289.7	6.95
Q14031	Collagen alpha-6(IV) chain OS=Homo sapiens GN=COL4A6 PE=2 SV=3 - [CO4A6_HUMAN]	1.48	1	1	1	1	0.81	0.83	0.80	0.82					1691	163.7	9.20
A6NMZ7	Collagen alpha-6(VI) chain OS=Homo sapiens GN=COL6A6 PE=1 SV=2 - [CO6A6_HUMAN]	2.21	1	3	3	3	0.95	1.13	1.02	1.24	1.57	5.53	3.05	####	2263	247.0	6.89
Q9Y6Z7	Collectin-10 OS=Homo sapiens GN=COLEC10 PE=2 SV=2 - [COL10_HUMAN]	9.39	1	3	3	5	0.97	0.93	0.93	0.90					277	30.7	7.33
Q9BWP8	Collectin-11 OS=Homo sapiens GN=COLEC11 PE=1 SV=1 - [COL11_HUMAN]	11.44	1	3	3	10	0.94	0.98	0.97	1.02	1.08	1.15	1.09	1.17	271	28.6	5.41
Q9Y6G5	COMM domain-containing protein 10 OS=Homo sapiens GN=COMMD10 PE=1 SV=1 - [COMDA_HUMAN]	5.94	1	1	1	1					1.15	0.95	7.22	5.95	202	23.0	6.54
Q9H0A8	COMM domain-containing protein 4 OS=Homo sapiens GN=COMMD4 PE=1 SV=1 - [COMD4_HUMAN]	10.55	1	1	1	1	1.15	1.16	1.21	1.23					199	21.8	7.31
Q9GZQ3	COMM domain-containing protein 5 OS=Homo sapiens GN=COMMD5 PE=1 SV=1 - [COMD5_HUMAN]	3.13	1	1	1	1					1.53	1.34	1.43	1.25	224	24.7	7.02
Q86VX2	COMM domain-containing protein 7 OS=Homo sapiens GN=COMMD7 PE=1 SV=2 - [COMD7_HUMAN]	12.50	1	1	1	2	1.70	1.62	1.72	1.66					200	22.5	5.92
Q9NX08	COMM domain-containing protein 8 OS=Homo sapiens GN=COMMD8 PE=1 SV=1 - [COMD8_HUMAN]	9.84	1	1	1	8	1.24	0.99	1.28	1.02					183	21.1	5.43
P02745	Complement C1q subcomponent subunit A OS=Homo sapiens GN=C1QA PE=1 SV=2 - [C1QA_HUMAN]	31.84	1	7	7	83	1.36	1.35	1.39	1.41	0.85	1.04	0.89	0.97	245	26.0	9.11

P02746	Complement C1q subcomponent subunit B OS=Homo sapiens GN=C1QB PE=1 SV=3 - [C1QB_HUMAN]	28.06	1	8	8	306	1.03	1.08	0.99	1.04	0.90	0.89	0.94	0.94	253	26.7	8.63
P02747	Complement C1q subcomponent subunit C OS=Homo sapiens GN=C1QC PE=1 SV=3 - [C1QC_HUMAN]	26.12	1	6	6	332	1.02	1.03	0.97	1.01	0.93	0.92	0.96	0.93	245	25.8	8.41
Q9BXJ4	Complement C1q tumor necrosis factor-related protein 3 OS=Homo sapiens GN=C1QTNF3 PE=2 SV=1 - [C1QT3_HUMAN]	8.13	1	2	2	4	1.00	1.12	0.89	1.01					246	27.0	6.52
P00736	Complement C1r subcomponent OS=Homo sapiens GN=C1R PE=1 SV=2 - [C1R_HUMAN]	41.42	1	23	25	363	1.05	1.02	1.08	1.05	0.84	0.82	0.88	0.85	705	80.1	6.21
Q9NZP8	Complement C1r subcomponent-like protein OS=Homo sapiens GN=C1RL PE=1 SV=2 - [C1RL_HUMAN]	25.46	1	11	13	108	1.21	1.21	1.24	1.22	0.98	0.93	0.97	0.97	487	53.5	7.20
P09871	Complement C1s subcomponent OS=Homo sapiens GN=C1S PE=1 SV=1 - [C1S_HUMAN]	47.97	1	28	29	566	1.05	1.07	1.08	1.11	0.88	0.90	0.83	0.88	688	76.6	4.96
P06681	Complement C2 OS=Homo sapiens GN=C2 PE=1 SV=2 - [CO2_HUMAN]	50.00	1	35	35	483	1.04	1.05	1.07	1.06	1.00	1.06	1.07	1.06	752	83.2	7.42
P01024	Complement C3 OS=Homo sapiens GN=C3 PE=1 SV=2 - [CO3_HUMAN]	90.14	1	198	200	21930	1.04	1.06	1.05	1.09	1.06	1.13	1.03	1.11	1663	187.0	6.40
P0C0L4	Complement C4-A OS=Homo sapiens GN=C4A PE=1 SV=1 - [CO4A_HUMAN]	64.97	3	1	117	6582	0.90	0.76	0.88	0.78	0.91	0.74	1.06	0.82	1744	192.7	7.08
P0C0L5	Complement C4-B OS=Homo sapiens GN=C4B PE=1 SV=1 - [CO4B_HUMAN]	64.97	3	1	117	6634	0.73	0.72	0.78	0.75	0.68	0.66	0.81	0.80	1744	192.7	7.15
P01031	Complement C5 OS=Homo sapiens GN=C5 PE=1 SV=4 - [CO5_HUMAN]	50.42	1	74	74	805	1.04	1.13	1.04	1.11	1.12	1.10	1.07	1.04	1676	188.2	6.52
Q07021	Complement component 1 Q subcomponent-binding protein, mitochondrial OS=Homo sapiens GN=C1QBP PE=1 SV=1 - [C1QBP_HUMAN]	4.61	1	1	1	1					1.50	1.13	2.08	1.58	282	31.3	4.84
Q9NPY3	Complement component C1q receptor OS=Homo sapiens GN=CD93 PE=1 SV=3 - [C1QR1_HUMAN]	13.80	1	5	5	17	0.97	0.87	0.89	0.93					652	68.5	5.44
P13671	Complement component C6 OS=Homo sapiens GN=C6 PE=1 SV=3 - [CO6_HUMAN]	55.89	4	43	43	578	1.00	0.94	0.99	0.96	0.92	0.89	0.92	0.95	934	104.7	6.76
P10643	Complement component C7 OS=Homo sapiens GN=C7 PE=1 SV=2 - [CO7_HUMAN]	63.82	2	40	40	512	1.22	1.19	1.19	1.18	1.04	1.06	1.01	1.03	843	93.5	6.48
P07357	Complement component C8 alpha chain OS=Homo sapiens GN=C8A PE=1 SV=2 - [CO8A_HUMAN]	39.90	1	18	18	339	0.96	0.93	1.01	0.97	0.85	0.81	0.89	0.87	584	65.1	6.47
P07358	Complement component C8 beta chain OS=Homo sapiens GN=C8B PE=1 SV=3 - [CO8B_HUMAN]	41.29	1	23	24	330	0.98	1.03	1.02	1.04	0.89	0.91	0.82	0.87	591	67.0	8.13
P07360	Complement component C8 gamma chain OS=Homo sapiens GN=C8G PE=1 SV=3 - [CO8G_HUMAN]	66.34	1	11	11	172	1.06	1.12	1.05	1.11	1.01	1.04	0.93	1.04	202	22.3	8.31
P02748	Complement component C9 OS=Homo sapiens GN=C9 PE=1 SV=2 - [CO9_HUMAN]	57.78	2	31	32	535	1.00	1.07	1.08	1.12	0.88	0.90	0.87	0.87	559	63.1	5.59
P08174	Complement decay-accelerating factor OS=Homo sapiens GN=CD55 PE=1 SV=4 - [DAF_HUMAN]	6.04	1	3	3	5	1.09	1.00	1.14	1.04					381	41.4	7.59
P00751	Complement factor B OS=Homo sapiens GN=CFB PE=1 SV=2 - [CFAB_HUMAN]	61.26	1	51	51	1909	1.01	0.98	1.00	0.99	0.98	1.01	0.96	1.00	764	85.5	7.06
P00746	Complement factor D OS=Homo sapiens GN=CFD PE=1 SV=5 - [CFAD_HUMAN]	52.17	1	11	11	91	1.01	0.98	1.05	1.06	0.87	0.84	0.92	0.85	253	27.0	7.71
P08603	Complement factor H OS=Homo sapiens GN=CFH PE=1 SV=4 - [CFAH_HUMAN]	60.93	1	60	67	2820	1.02	0.96	1.02	0.97	1.03	1.02	1.00	1.01	1231	139.0	6.61
Q03591	Complement factor H-related protein 1 OS=Homo sapiens GN=CFHR1 PE=1 SV=2 - [FHR1_HUMAN]	48.18	1	3	15	398	0.97	0.76	0.91	0.66	0.79	0.84	0.79	0.85	330	37.6	7.39
P36980	Complement factor H-related protein 2 OS=Homo sapiens GN=CFHR2 PE=1 SV=1 - [FHR2_HUMAN]	47.04	1	5	11	358	1.25	1.20	1.20	1.09	1.25	1.12	1.20	1.06	270	30.6	6.38
Q02985	Complement factor H-related protein 3 OS=Homo sapiens GN=CFHR3 PE=1 SV=2 - [FHR3_HUMAN]	26.36	1	2	8	96	0.87	0.91	0.91	0.98	0.88	0.77	0.98	0.87	330	37.3	7.55
Q92496	Complement factor H-related protein 4 OS=Homo sapiens GN=CFHR4 PE=1 SV=2 - [FHR4_HUMAN]	20.54	1	1	6	65	1.11	0.99	0.95	0.87	0.81	0.64	0.87	0.73	331	37.3	5.26

Q9BXR6	Complement factor H-related protein 5 OS=Homo sapiens GN=CFHR5 PE=1 SV=1 - [FHR5_HUMAN]	36.03	1	19	20	113	1.16	1.10	1.29	1.23	0.82	0.91	0.76	0.83	569	64.4	7.06
P05156	Complement factor I OS=Homo sapiens GN=CFI PE=1 SV=2 - [CFAI_HUMAN]	46.31	1	28	29	433	1.09	1.01	1.09	1.02	0.90	0.83	0.87	0.81	583	65.7	7.50
P17927	Complement receptor type 1 OS=Homo sapiens GN=CR1 PE=1 SV=3 - [CR1_HUMAN]	1.18	1	1	1	2	1.57	1.28	1.29	1.06					2039	223.5	6.95
Q15021	Condensin complex subunit 1 OS=Homo sapiens GN=NCAPD2 PE=1 SV=3 - [CND1_HUMAN]	3.07	1	2	2	3					0.64	0.56	1.01	0.88	1401	157.1	6.61
Q15003	Condensin complex subunit 2 OS=Homo sapiens GN=NCAPH PE=1 SV=3 - [CND2_HUMAN]	1.08	1	1	1	1					2.38	1.69	1.87	1.33	741	82.5	5.06
Q9BPX3	Condensin complex subunit 3 OS=Homo sapiens GN=NCAPG PE=1 SV=1 - [CND3_HUMAN]	3.45	1	1	1	1	0.89	0.83	0.44	0.41					1015	114.3	5.59
Q86XI2	Condensin-2 complex subunit G2 OS=Homo sapiens GN=NCAPG2 PE=1 SV=1 - [CNDG2_HUMAN]	1.92	1	1	1	1	2.27	0.88	2.74	1.07					1143	130.9	6.87
P51160	Cone cGMP-specific 3',5'-cyclic phosphodiesterase subunit alpha' OS=Homo sapiens GN=PDE6C PE=1 SV=2 - [PDE6C_HUMAN]	1.17	1	1	1	1	0.43	0.85	0.24	0.46					858	99.1	5.72
O43186	Cone-rod homeobox protein OS=Homo sapiens GN=CRX PE=1 SV=1 - [CRX_HUMAN]	1.34	1	1	1	1					0.74	0.81	0.79	0.87	299	32.2	9.16
P29279	Connective tissue growth factor OS=Homo sapiens GN=CTGF PE=1 SV=2 - [CTGF_HUMAN]	12.32	1	4	4	8	0.66	0.63	0.67	0.65	0.93	0.95	1.04	0.98	349	38.1	8.00
Q96JB2	Conserved oligomeric Golgi complex subunit 3 OS=Homo sapiens GN=COG3 PE=1 SV=3 - [COG3_HUMAN]	9.30	1	4	4	8	1.18	1.28	1.11	1.21	0.67	0.88	0.63	0.82	828	94.0	5.57
Q9UP83	Conserved oligomeric Golgi complex subunit 5 OS=Homo sapiens GN=COG5 PE=1 SV=3 - [COG5_HUMAN]	7.63	8	3	4	4	0.69	0.49	0.78	0.71					839	92.7	6.60
Q9Y2V7	Conserved oligomeric Golgi complex subunit 6 OS=Homo sapiens GN=COG6 PE=2 SV=2 - [COG6_HUMAN]	4.26	1	1	2	25	0.67	1.27	0.87	1.64					657	73.2	5.76
Q96MW5	Conserved oligomeric Golgi complex subunit 8 OS=Homo sapiens GN=COG8 PE=1 SV=2 - [COG8_HUMAN]	1.96	1	1	1	2					0.66	0.70	0.86	0.91	612	68.4	5.20
Q9NZB2	Constitutive coactivator of PPAR-gamma-like protein 1 OS=Homo sapiens GN=FAM120A PE=1 SV=2 - [F120A_HUMAN]	1.97	1	2	2	3	0.58	0.64	0.59	0.65	0.78	0.89	1.27	1.46	1118	121.8	8.88
Q9NX05	Constitutive coactivator of PPAR-gamma-like protein 2 OS=Homo sapiens GN=FAM120C PE=2 SV=3 - [F120C_HUMAN]	1.73	1	1	1	1					0.91	1.00	0.97	1.07	1096	120.5	9.03
Q12860	Contactin-1 OS=Homo sapiens GN=CNTN1 PE=1 SV=1 - [CNTN1_HUMAN]	11.39	1	9	9	23	1.09	1.04	1.00	0.97	0.80	0.73	0.97	0.76	1018	113.2	5.90
Q9P232	Contactin-3 OS=Homo sapiens GN=CNTN3 PE=1 SV=3 - [CNTN3_HUMAN]	5.25	1	4	4	6	0.98	1.01	1.04	1.08	0.82	0.64	0.88	0.68	1028	112.8	6.30
Q8IWV2	Contactin-4 OS=Homo sapiens GN=CNTN4 PE=1 SV=1 - [CNTN4_HUMAN]	2.92	1	3	3	5	0.97	0.98	0.96	0.97	0.93	1.23	1.50	1.99	1026	113.4	7.47
O94779	Contactin-5 OS=Homo sapiens GN=CNTN5 PE=2 SV=2 - [CNTN5_HUMAN]	2.64	1	2	2	2	0.72	0.85	0.68	0.79					1100	120.6	6.34
Q9UHC6	Contactin-associated protein-like 2 OS=Homo sapiens GN=CNTNAP2 PE=1 SV=1 - [CNTP2_HUMAN]	1.28	1	1	1	1					1.10	1.56	1.54	2.20	1331	148.1	6.58
Q8WYK1	Contactin-associated protein-like 5 OS=Homo sapiens GN=CNTNAP5 PE=2 SV=1 - [CNTP5_HUMAN]	4.36	1	2	2	2					0.88	0.87	0.96	0.94	1306	145.5	6.29
Q96A23	Copine-4 OS=Homo sapiens GN=CPNE4 PE=2 SV=1 - [CPNE4_HUMAN]	1.62	1	1	1	1	0.31	0.28	0.18	0.17					557	62.4	6.33
Q8IYJ1	Copine-9 OS=Homo sapiens GN=CPNE9 PE=1 SV=3 - [CPNE9_HUMAN]	1.81	1	1	1	2	0.58	0.40	0.63	0.43					553	61.8	5.34
Q04656	Copper-transporting ATPase 1 OS=Homo sapiens GN=ATP7A PE=1 SV=3 - [ATP7A_HUMAN]	1.20	1	1	1	1	1.31	1.25	1.25	1.20					1500	163.3	6.24
P35670	Copper-transporting ATPase 2 OS=Homo sapiens GN=ATP7B PE=1 SV=4 - [ATP7B_HUMAN]	2.87	1	2	2	2	1.10	1.25	1.00	1.14	1.24	1.16	0.83	0.78	1465	157.2	6.70
Q53SF7	Cordon-bleu protein-like 1 OS=Homo sapiens GN=COBLL1 PE=1 SV=2 - [COBL1_HUMAN]	3.99	1	3	3	4	1.16	1.45	1.21	1.52	0.79	0.83	0.83	0.87	1204	131.7	6.67

O75367	Core histone macro-H2A.1 OS=Homo sapiens GN=H2AFY PE=1 SV=4 - [H2AY_HUMAN]	4.03	1	1	1	1	1.27	1.10	1.11	0.98					372	39.6	9.79
Q9UBG3	Cornulin OS=Homo sapiens GN=CRNN PE=1 SV=1 - [CRNN_HUMAN]	6.26	1	1	1	1	0.54	0.58	0.69	0.74					495	53.5	6.10
P31146	Coronin-1A OS=Homo sapiens GN=CORO1A PE=1 SV=4 - [COR1A_HUMAN]	11.28	1	5	5	10	0.86	0.80	0.86	0.81	1.97	1.99	1.93	1.95	461	51.0	6.68
Q9BR76	Coronin-1B OS=Homo sapiens GN=CORO1B PE=1 SV=1 - [COR1B_HUMAN]	7.16	1	2	2	2	0.99	1.16	0.93	1.10		3.12		3.42	489	54.2	5.88
Q9ULV4	Coronin-1C OS=Homo sapiens GN=CORO1C PE=1 SV=1 - [COR1C_HUMAN]	12.45	1	5	5	11	0.54	0.49	0.57	0.51	1.33	1.50	1.37	1.55	474	53.2	7.08
Q8WZ74	Cortactin-binding protein 2 OS=Homo sapiens GN=CTTNBP2 PE=1 SV=1 - [CTTB2_HUMAN]	0.72	1	1	1	1					0.66	0.75	0.69	0.78	1663	180.9	7.93
P06850	Corticoliberin OS=Homo sapiens GN=CRH PE=1 SV=1 - [CRF_HUMAN]	16.33	1	1	1	1	1.19	0.76	0.99	0.63					196	21.4	9.96
P08185	Corticosteroid-binding globulin OS=Homo sapiens GN=SERPINA6 PE=1 SV=1 - [CBG_HUMAN]	56.05	1	14	14	275	1.30	1.33	1.30	1.32	1.13	1.13	1.06	1.06	405	45.1	6.04
P34998	Corticotropin-releasing factor receptor 1 OS=Homo sapiens GN=CRHR1 PE=1 SV=1 - [CRFR1_HUMAN]	1.13	1	1	1	3					1.68	1.46	1.67	1.45	444	50.7	9.33
P24387	Corticotropin-releasing factor-binding protein OS=Homo sapiens GN=CRHBP PE=1 SV=2 - [CRHBP_HUMAN]	7.45	1	1	1	1	1.20	0.84	1.34	0.94					322	36.1	6.52
Q9P1F3	Costars family protein ABRACL OS=Homo sapiens GN=ABRACL PE=1 SV=1 - [ABRAL_HUMAN]	16.05	1	1	1	1					1.71	1.41	1.40	1.15	81	9.1	6.29
P10589	COUP transcription factor 1 OS=Homo sapiens GN=NR2F1 PE=1 SV=1 - [COT1_HUMAN]	7.33	1	1	1	1	0.73	0.85	0.78	0.91					423	46.1	8.25
Q9P0U4	CpG-binding protein OS=Homo sapiens GN=CXXC1 PE=1 SV=2 - [CXXC1_HUMAN]	1.37	1	1	1	1					0.43	0.33	0.31	0.24	656	75.7	8.24
P06732	Creatine kinase M-type OS=Homo sapiens GN=CKM PE=1 SV=2 - [KCRM_HUMAN]	2.62	1	1	1	2	0.82	0.81	0.71	0.71					381	43.1	7.25
Q9NS37	CREB/ATF bZIP transcription factor OS=Homo sapiens GN=CREBZF PE=1 SV=2 - [ZHANG_HUMAN]	5.37	1	1	1	1	0.74	0.77	0.83	0.87					354	37.1	5.24
P46109	Crk-like protein OS=Homo sapiens GN=CRKL PE=1 SV=1 - [CRKL_HUMAN]	27.39	1	7	7	24	0.94	0.48	0.80	0.51	1.09	1.19	1.20	1.20	303	33.8	6.74
Q9BZJ0	Crooked neck-like protein 1 OS=Homo sapiens GN=CRNKL1 PE=1 SV=4 - [CRNL1_HUMAN]	0.94	1	1	1	1	1.49	1.62	1.78	1.96					848	100.4	8.00
Q2NKJ3	CST complex subunit CTC1 OS=Homo sapiens GN=CTC1 PE=1 SV=2 - [CTC1_HUMAN]	1.15	1	1	1	1	1.07	1.03	0.86	0.83					1217	134.5	7.88
Q9P2B4	CTTNBP2 N-terminal-like protein OS=Homo sapiens GN=CTTNBP2NL PE=1 SV=2 - [CT2NL_HUMAN]	2.35	1	1	1	2	0.91	0.78	0.88	0.77					639	70.1	8.06
Q96PZ7	CUB and sushi domain-containing protein 1 OS=Homo sapiens GN=CSMD1 PE=1 SV=2 - [CSMD1_HUMAN]	0.81	1	1	1	3	1.22	1.30	1.17	1.24					3565	388.6	6.07
Q7Z408	CUB and sushi domain-containing protein 2 OS=Homo sapiens GN=CSMD2 PE=1 SV=2 - [CSMD2_HUMAN]	0.66	1	1	1	1					0.34	0.16	0.58	0.27	3487	379.8	6.09
O60494	Cubilin OS=Homo sapiens GN=CUBN PE=1 SV=5 - [CUBN_HUMAN]	0.80	1	1	1	2	1.50	2.01	1.31	1.77					3623	398.5	5.35
Q8N6W0	CUGBP Elav-like family member 5 OS=Homo sapiens GN=CELF5 PE=1 SV=1 - [CELF5_HUMAN]	1.65	2	1	1	2	0.94	1.12	0.91	1.08	1.05	1.07	1.09	1.10	485	52.3	8.32
Q13619	Cullin-4A OS=Homo sapiens GN=CUL4A PE=1 SV=3 - [CUL4A_HUMAN]	2.24	1	2	2	4	1.27	1.31	1.43	1.48	1.02	0.95	0.89	0.83	759	87.6	8.13
Q13620	Cullin-4B OS=Homo sapiens GN=CUL4B PE=1 SV=4 - [CUL4B_HUMAN]	2.96	1	2	2	2	1.23	1.06	1.35	1.16					913	103.9	7.37
Q14999	Cullin-7 OS=Homo sapiens GN=CUL7 PE=1 SV=2 - [CUL7_HUMAN]	0.65	1	1	1	2	0.86	0.75	1.92	1.67					1698	191.0	5.87
Q8IWT3	Cullin-9 OS=Homo sapiens GN=CUL9 PE=1 SV=2 - [CUL9_HUMAN]	2.42	1	3	3	22	1.35	0.91	1.17	0.78	0.93	0.96	1.10	1.12	2517	281.0	5.45

Q86VP6	Cullin-associated NEDD8-dissociated protein 1 OS=Homo sapiens GN=CAND1 PE=1 SV=2 - [CAND1_HUMAN]	1.54	1	1	1	1	1.02	0.89	1.09	0.96					1230	136.3	5.78
O75155	Cullin-associated NEDD8-dissociated protein 2 OS=Homo sapiens GN=CAND2 PE=1 SV=3 - [CAND2_HUMAN]	1.05	1	1	1	1	1.20	1.31	1.28	1.41					1236	135.2	5.68
Q2TBE0	CWF19-like protein 2 OS=Homo sapiens GN=CWF19L2 PE=1 SV=4 - [C19L2_HUMAN]	1.34	1	1	1	2					2.81	2.84	2.52	2.55	894	103.7	8.65
Q14D33	CXXC-type zinc finger protein 11 OS=Homo sapiens GN=CXXC11 PE=2 SV=2 - [CB085_HUMAN]	4.90	1	1	1	1	0.96	1.09	1.03	1.17					572	60.4	6.57
P18846	Cyclic AMP-dependent transcription factor ATF-1 OS=Homo sapiens GN=ATF1 PE=1 SV=2 - [ATF1_HUMAN]	12.55	1	2	2	2	1.17	1.10	1.20	1.14	0.44	0.09	0.79	0.16	271	29.2	8.37
P15336	Cyclic AMP-dependent transcription factor ATF-2 OS=Homo sapiens GN=ATF2 PE=1 SV=4 - [ATF2_HUMAN]	7.52	1	1	1	1	0.44	0.57	0.39	0.51					505	54.5	7.88
Q9Y2D1	Cyclic AMP-dependent transcription factor ATF-5 OS=Homo sapiens GN=ATF5 PE=1 SV=4 - [ATF5_HUMAN]	10.28	1	1	1	1	0.34	0.35	0.54	0.55					282	30.7	4.97
Q68CJ9	Cyclic AMP-responsive element-binding protein 3-like protein 3 OS=Homo sapiens GN=CREB3L3 PE=1 SV=2 - [CR3L3_HUMAN]	9.54	1	2	2	3					0.16	0.40	0.74	1.84	461	49.0	5.15
Q14028	Cyclic nucleotide-gated cation channel beta-1 OS=Homo sapiens GN=CNGB1 PE=1 SV=2 - [CNGB1_HUMAN]	2.00	1	1	1	1	1.25	1.31	1.50	1.58					1251	139.6	4.81
Q9NQW8	Cyclic nucleotide-gated cation channel beta-3 OS=Homo sapiens GN=CNGB3 PE=1 SV=2 - [CNGB3_HUMAN]	1.11	1	1	1	1	0.58	0.41	0.67	0.48					809	92.1	7.93
Q8N815	Cyclin N-terminal domain-containing protein 1 OS=Homo sapiens GN=CNTD1 PE=2 SV=2 - [CNTD1_HUMAN]	3.03	1	1	1	1					0.94	2.05	0.57	1.24	330	36.9	6.93
O95273	Cyclin-D1-binding protein 1 OS=Homo sapiens GN=CCNDBP1 PE=1 SV=2 - [CCDB1_HUMAN]	6.11	1	1	1	1	1.74	1.33	1.00	0.78					360	40.2	4.82
Q9NYV4	Cyclin-dependent kinase 12 OS=Homo sapiens GN=CDK12 PE=1 SV=2 - [CDK12_HUMAN]	2.62	1	1	1	1	0.50	0.80	0.40	0.65					1490	164.1	9.44
P55273	Cyclin-dependent kinase 4 inhibitor D OS=Homo sapiens GN=CDKN2D PE=1 SV=1 - [CDN2D_HUMAN]	6.63	1	1	1	3					2.17	2.21	1.83	1.87	166	17.7	6.13
Q00534	Cyclin-dependent kinase 6 OS=Homo sapiens GN=CDK6 PE=1 SV=1 - [CDK6_HUMAN]	11.66	1	2	2	2					1.74	1.63	1.43	1.33	326	36.9	6.46
P50613	Cyclin-dependent kinase 7 OS=Homo sapiens GN=CDK7 PE=1 SV=1 - [CDK7_HUMAN]	2.60	1	1	1	1	0.87	0.85	0.68	0.67					346	39.0	8.47
Q8IVW4	Cyclin-dependent kinase-like 3 OS=Homo sapiens GN=CDKL3 PE=2 SV=1 - [CDKL3_HUMAN]	6.08	1	1	1	1			0.31	0.57					592	67.5	9.31
O76039	Cyclin-dependent kinase-like 5 OS=Homo sapiens GN=CDKL5 PE=1 SV=1 - [CDKL5_HUMAN]	3.30	1	2	2	2					0.99	1.00	0.92	0.93	1030	115.5	9.54
Q16589	Cyclin-G2 OS=Homo sapiens GN=CCNG2 PE=1 SV=1 - [CCNG2_HUMAN]	4.94	1	1	1	2					1.32	1.17	1.30	1.14	344	38.8	5.48
O60583	Cyclin-T2 OS=Homo sapiens GN=CCNT2 PE=1 SV=2 - [CCNT2_HUMAN]	2.60	1	1	1	5					1.39	1.66	1.78	2.14	730	81.0	8.92
Q8ND76	Cyclin-Y OS=Homo sapiens GN=CCNY PE=1 SV=2 - [CCNY_HUMAN]	5.57	1	1	1	1	1.37	1.53	1.30	1.46					341	39.3	7.20
P01040	Cystatin-A OS=Homo sapiens GN=CSTA PE=1 SV=1 - [CYTA_HUMAN]	15.31	1	2	2	3					1.18	1.09	1.15	1.05	98	11.0	5.50
P04080	Cystatin-B OS=Homo sapiens GN=CSTB PE=1 SV=2 - [CYTB_HUMAN]	12.24	1	1	1	3					1.61	1.83	1.28	1.45	98	11.1	7.56
P01034	Cystatin-C OS=Homo sapiens GN=CST3 PE=1 SV=1 - [CYTC_HUMAN]	34.93	1	5	5	77	0.96	0.92	0.93	0.90	1.04	1.02	0.97	1.01	146	15.8	8.75
O76096	Cystatin-F OS=Homo sapiens GN=CST7 PE=1 SV=1 - [CYTF_HUMAN]	4.83	1	1	1	15	1.12	1.16	1.16	1.22	0.66	0.75	0.60	0.65	145	16.4	8.48
Q15828	Cystatin-M OS=Homo sapiens GN=CST6 PE=1 SV=1 - [CYTM_HUMAN]	28.19	1	3	3	7					1.37	1.53	1.20	1.33	149	16.5	8.09
P21291	Cysteine and glycine-rich protein 1 OS=Homo sapiens GN=CSRP1 PE=1 SV=3 - [CSRP1_HUMAN]	16.58	1	3	3	12					1.27	1.41	1.16	1.29	193	20.6	8.57

Q9UHD1	Cysteine and histidine-rich domain-containing protein 1 OS=Homo sapiens GN=CHORDC1 PE=1 SV=2 - [CHRD1_HUMAN]	4.52	1	1	1	1					1.00	0.96	0.82	0.79	332	37.5	7.87
Q9Y697	Cysteine desulfurase, mitochondrial OS=Homo sapiens GN=NFS1 PE=1 SV=3 - [NFS1_HUMAN]	3.50	1	1	1	4	1.01	1.07	0.98	1.06	0.87	0.78	0.87	0.77	457	50.2	8.31
Q9Y600	Cysteine sulfinic acid decarboxylase OS=Homo sapiens GN=CSAD PE=1 SV=2 - [CSAD_HUMAN]	5.68	1	1	1	1					0.71	0.75	0.89	0.94	493	55.0	6.48
Q5VXU3	Cysteine-rich hydrophobic domain 1 protein OS=Homo sapiens GN=CHIC1 PE=2 SV=2 - [CHIC1_HUMAN]	5.80	1	1	1	1					0.31	0.24	0.72	0.58	224	25.6	4.51
P16562	Cysteine-rich secretory protein 2 OS=Homo sapiens GN=CRISP2 PE=1 SV=1 - [CRIS2_HUMAN]	4.53	1	1	1	1					0.59	0.69	0.70	0.81	243	27.2	6.49
P54108	Cysteine-rich secretory protein 3 OS=Homo sapiens GN=CRISP3 PE=1 SV=1 - [CRIS3_HUMAN]	13.06	1	3	3	15	0.95	1.15	0.98	1.19	1.09	1.02	1.11	1.11	245	27.6	7.80
Q96HD1	Cysteine-rich with EGF-like domain protein 1 OS=Homo sapiens GN=CRELD1 PE=1 SV=3 - [CREL1_HUMAN]	4.05	1	1	1	3	1.27	1.04	1.31	1.08	1.79	1.62	2.11	1.90	420	45.4	4.87
Q8WYN3	Cysteine/serine-rich nuclear protein 3 OS=Homo sapiens GN=CSRNP3 PE=1 SV=1 - [CSRN3_HUMAN]	1.54	1	1	1	5					1.22	0.02	0.86	0.03	585	64.9	4.75
P13569	Cystic fibrosis transmembrane conductance regulator OS=Homo sapiens GN=CFTR PE=1 SV=3 - [CFTR_HUMAN]	0.88	1	1	1	2	4.65		1.06						1480	168.0	8.73
Q53TN4	Cytochrome b reductase 1 OS=Homo sapiens GN=CYBRD1 PE=1 SV=1 - [CYBR1_HUMAN]	4.20	1	1	1	1	0.09	0.47	1.94	####					286	31.6	8.76
P07919	Cytochrome b-c1 complex subunit 6, mitochondrial OS=Homo sapiens GN=UQCRH PE=1 SV=2 - [QCR6_HUMAN]	8.79	1	1	1	1					1.62	1.37	1.68	1.41	91	10.7	4.44
P14927	Cytochrome b-c1 complex subunit 7 OS=Homo sapiens GN=UQCRB PE=1 SV=2 - [QCR7_HUMAN]	6.31	1	1	1	9	1.02	1.49	1.06	1.57					111	13.5	8.78
P99999	Cytochrome c OS=Homo sapiens GN=CYCS PE=1 SV=2 - [CYC_HUMAN]	10.48	1	1	1	5	0.56	0.54	0.55	0.54	0.67	0.63	0.72	0.68	105	11.7	9.57
P15954	Cytochrome c oxidase subunit 7C, mitochondrial OS=Homo sapiens GN=COX7C PE=1 SV=1 - [COX7C_HUMAN]	9.52	1	1	1	2					0.47	1.50	0.68	2.15	63	7.2	10.27
P05177	Cytochrome P450 1A2 OS=Homo sapiens GN=CYP1A2 PE=1 SV=3 - [CP1A2_HUMAN]	6.80	1	1	1	1	0.89	1.33	0.71	1.06					515	58.3	9.06
Q9NR63	Cytochrome P450 26B1 OS=Homo sapiens GN=CYP26B1 PE=2 SV=1 - [CP26B_HUMAN]	4.49	1	1	1	1					1.36	0.04	2.02	0.07	512	57.5	8.46
P33261	Cytochrome P450 2C19 OS=Homo sapiens GN=CYP2C19 PE=1 SV=3 - [CP2CJ_HUMAN]	3.67	1	1	2	2	0.86	0.91	0.78	0.83					490	55.9	7.39
P11712	Cytochrome P450 2C9 OS=Homo sapiens GN=CYP2C9 PE=1 SV=3 - [CP2C9_HUMAN]	5.71	1	2	2	10	1.61	1.21	2.01	1.51	1.19	0.92	0.97	0.80	490	55.6	7.91
P51589	Cytochrome P450 2J2 OS=Homo sapiens GN=CYP2J2 PE=1 SV=2 - [CP2J2_HUMAN]	1.39	1	1	1	2					0.53	0.04	1.09	0.07	502	57.6	8.60
Q7Z449	Cytochrome P450 2U1 OS=Homo sapiens GN=CYP2U1 PE=1 SV=1 - [CP2U1_HUMAN]	5.15	1	1	1	9	1.16	1.00	1.26	1.00					544	61.9	8.40
P08684	Cytochrome P450 3A4 OS=Homo sapiens GN=CYP3A4 PE=1 SV=4 - [CP3A4_HUMAN]	2.78	2	1	1	16					0.92	0.93	1.05	1.03	503	57.3	8.10
P20815	Cytochrome P450 3A5 OS=Homo sapiens GN=CYP3A5 PE=1 SV=1 - [CP3A5_HUMAN]	3.19	1	1	1	1	0.86	1.05	0.92	1.14					502	57.1	8.75
Q9HC73	Cytokine receptor-like factor 2 OS=Homo sapiens GN=CRLF2 PE=1 SV=1 - [CRLF2_HUMAN]	9.97	1	2	2	3	0.86	0.97	0.98	1.12					371	42.0	5.25
P21399	Cytoplasmic aconitate hydratase OS=Homo sapiens GN=ACO1 PE=1 SV=3 - [ACOC_HUMAN]	3.71	1	2	2	3	0.98	0.15	0.93	0.23					889	98.3	6.68
Q14204	Cytoplasmic dynein 1 heavy chain 1 OS=Homo sapiens GN=DYNC1H1 PE=1 SV=5 - [DYHC1_HUMAN]	3.27	1	7	8	14	1.18	1.12	1.23	1.32	0.63	0.85	0.91	1.47	4646	532.1	6.40
O14576	Cytoplasmic dynein 1 intermediate chain 1 OS=Homo sapiens GN=DYNC1I1 PE=1 SV=2 - [DC1I1_HUMAN]	3.72	1	1	1	1	1.22	1.31	0.94	1.01					645	72.9	5.12
Q13409	Cytoplasmic dynein 1 intermediate chain 2 OS=Homo sapiens GN=DYNC1I2 PE=1 SV=3 - [DC1I2_HUMAN]	5.17	1	1	1	2					1.25	1.10	1.06	0.93	638	71.4	5.20

Q8NCM8	Cytoplasmic dynein 2 heavy chain 1 OS=Homo sapiens GN=DYNC2H1 PE=1 SV=4 - [DYHC2_HUMAN]	1.11	1	3	3	3	0.91	0.83	1.02	0.94	1.01	1.00	0.98	0.97	4307	492.3	6.54
Q8TCX1	Cytoplasmic dynein 2 light intermediate chain 1 OS=Homo sapiens GN=DYNC2LI1 PE=2 SV=1 - [DC2L1_HUMAN]	13.39	1	2	2	8					1.15	0.86	1.10	0.90	351	39.6	7.53
Q7L576	Cytoplasmic FMR1-interacting protein 1 OS=Homo sapiens GN=CYFIP1 PE=1 SV=1 - [CYFP1_HUMAN]	1.04	2	1	1	1	1.29	1.38	1.28	1.37					1253	145.1	6.90
Q8NE35	Cytoplasmic polyadenylation element-binding protein 3 OS=Homo sapiens GN=CPEB3 PE=1 SV=2 - [CPEB3_HUMAN]	5.87	3	2	2	2	1.29	1.17	1.14	1.04					698	76.0	7.03
P16333	Cytoplasmic protein NCK1 OS=Homo sapiens GN=NCK1 PE=1 SV=1 - [NCK1_HUMAN]	15.92	1	3	4	5					1.26	1.28	1.27	1.29	377	42.8	6.47
O43639	Cytoplasmic protein NCK2 OS=Homo sapiens GN=NCK2 PE=1 SV=2 - [NCK2_HUMAN]	7.89	1	2	3	8					1.50	1.37	1.39	1.27	380	42.9	6.95
Q14008	Cytoskeleton-associated protein 5 OS=Homo sapiens GN=CKAP5 PE=1 SV=3 - [CKAP5_HUMAN]	3.54	1	4	4	8	0.92	1.19	0.77	1.01	2.31	3.03	2.03	2.57	2032	225.4	7.80
O00154	Cytosolic acyl coenzyme A thioester hydrolase OS=Homo sapiens GN=ACOT7 PE=1 SV=3 - [BACH_HUMAN]	2.37	1	2	2	3	0.69	0.69	0.74	0.74	0.84	0.91	0.69	0.75	380	41.8	8.54
Q6ZUV0	Cytosolic acyl coenzyme A thioester hydrolase-like OS=Homo sapiens GN=ACOT7L PE=1 SV=1 - [BACHL_HUMAN]	7.54	1	1	1	1					0.90	0.80	1.46	1.29	252	28.1	7.15
Q9H227	Cytosolic beta-glucosidase OS=Homo sapiens GN=GBA3 PE=1 SV=2 - [GBA3_HUMAN]	1.71	1	1	1	1					1.30	0.89	0.89	0.61	469	53.7	5.63
Q9UPW5	Cytosolic carboxypeptidase 1 OS=Homo sapiens GN=AGTPBP1 PE=1 SV=3 - [CBPC1_HUMAN]	1.22	1	1	1	1					2.41	1.05	3.14	1.37	1226	138.4	6.15
Q5U5Z8	Cytosolic carboxypeptidase 2 OS=Homo sapiens GN=AGBL2 PE=2 SV=2 - [CBPC2_HUMAN]	4.66	1	2	3	9					0.80	0.90	0.92	1.04	902	104.1	9.00
Q8NEM8	Cytosolic carboxypeptidase 3 OS=Homo sapiens GN=AGBL3 PE=2 SV=2 - [CBPC3_HUMAN]	2.80	1	2	2	4	1.14	1.24	1.14	1.24	0.81	0.67	0.58	0.36	1001	115.9	8.79
Q96MI9	Cytosolic carboxypeptidase 4 OS=Homo sapiens GN=AGBL1 PE=2 SV=2 - [CBPC4_HUMAN]	1.22	1	1	1	2					0.64	0.68	0.71	0.76	1066	120.2	7.24
Q86XP0	Cytosolic phospholipase A2 delta OS=Homo sapiens GN=PLA2G4D PE=2 SV=2 - [PA24D_HUMAN]	5.13	1	3	3	3		0.54		0.73	0.87	0.35	0.70	0.28	818	91.9	5.50
Q3MJ16	Cytosolic phospholipase A2 epsilon OS=Homo sapiens GN=PLA2G4E PE=1 SV=3 - [PA24E_HUMAN]	3.15	1	2	2	2	1.30	1.10	1.37	1.17					856	97.5	5.95
P47712	Cytosolic phospholipase A2 OS=Homo sapiens GN=PLA2G4A PE=1 SV=2 - [PA24A_HUMAN]	1.87	1	1	1	1	0.39	0.29	1.30	0.97					749	85.2	5.38
Q69YQ0	Cytospin-A OS=Homo sapiens GN=SPECC1L PE=1 SV=2 - [CYTSA_HUMAN]	1.52	1	1	1	1	0.36	0.38	0.41	0.43					1117	124.5	5.72
Q5M775	Cytospin-B OS=Homo sapiens GN=SPECC1 PE=1 SV=1 - [CYTSB_HUMAN]	2.06	1	2	2	4					2.39	1.97	2.18	1.80	1068	118.5	6.70
O95727	Cytotoxic and regulatory T-cell molecule OS=Homo sapiens GN=CRTAM PE=1 SV=2 - [CRTAM_HUMAN]	2.29	1	1	1	2					0.74	0.83	0.84	0.94	393	44.6	6.84
P30046	D-dopachrome decarboxylase OS=Homo sapiens GN=DDT PE=1 SV=3 - [DOPD_HUMAN]	16.10	1	2	2	3					1.63	1.58	1.19	1.15	118	12.7	7.30
Q8TEA8	D-tyrosyl-tRNA(Tyr) deacylase 1 OS=Homo sapiens GN=DTD1 PE=1 SV=2 - [DTD1_HUMAN]	7.66	1	1	1	3					1.23	1.33	0.93	0.99	209	23.4	8.24
Q5D0E6	DALR anticodon-binding domain-containing protein 3 OS=Homo sapiens GN=DALRD3 PE=2 SV=2 - [DALD3_HUMAN]	4.24	4	1	2	3	0.74	0.62	0.88	0.74					543	59.3	7.42
Q5SW24	Dapper homolog 2 OS=Homo sapiens GN=DACT2 PE=2 SV=1 - [DACT2_HUMAN]	5.56	1	2	2	3					0.59	1.89	4.47	1.10	774	82.6	8.87
Q96B18	Dapper homolog 3 OS=Homo sapiens GN=DACT3 PE=2 SV=2 - [DACT3_HUMAN]	1.59	1	1	1	1					1.56	1.20	1.68	1.29	629	64.9	10.36
Q9HCK1	DBF4-type zinc finger-containing protein 2 OS=Homo sapiens GN=ZDBF2 PE=1 SV=3 - [ZDBF2_HUMAN]	1.66	1	1	2	4		0.84		0.92					2354	265.5	6.16
Q5T1A1	DC-STAMP domain-containing protein 2 OS=Homo sapiens GN=DCST2 PE=2 SV=2 - [DCST2_HUMAN]	2.33	1	1	1	1	1.77	1.89	1.42	1.54					773	86.2	8.16

Q9UKG1	DCC-interacting protein 13-alpha OS=Homo sapiens GN=APPL1 PE=1 SV=1 - [DP13A_HUMAN]	4.51	2	2	2	4	0.85	0.86	1.06	1.08	1.21	1.37	1.18	1.34	709	79.6	5.41
Q8IWE4	DCN1-like protein 3 OS=Homo sapiens GN=DCUN1D3 PE=2 SV=1 - [DCNL3_HUMAN]	5.26	1	1	1	2					1.57	2.38	1.50	2.28	304	34.3	5.12
Q9H773	dCTP pyrophosphatase 1 OS=Homo sapiens GN=DCTPP1 PE=1 SV=1 - [DCTP1_HUMAN]	11.76	1	1	1	2	1.32	1.35	1.11	1.14	0.53	0.80	0.87	1.32	170	18.7	5.03
Q8TEB1	DDB1- and CUL4-associated factor 11 OS=Homo sapiens GN=DCAF11 PE=1 SV=1 - [DCA11_HUMAN]	3.66	1	1	2	2					0.90	0.78	0.73	0.63	546	61.6	6.40
Q5VU92	DDB1- and CUL4-associated factor 12-like protein 1 OS=Homo sapiens GN=DCAF12L1 PE=2 SV=1 - [DC121_HUMAN]	6.05	1	1	1	1	0.03	0.03	0.04	0.04					463	51.2	8.56
Q66K64	DDB1- and CUL4-associated factor 15 OS=Homo sapiens GN=DCAF15 PE=1 SV=1 - [DCA15_HUMAN]	1.33	1	1	1	1	1.11	1.19	1.05	1.13					600	66.4	6.58
Q8WV16	DDB1- and CUL4-associated factor 4 OS=Homo sapiens GN=DCAF4 PE=1 SV=3 - [DCAF4_HUMAN]	2.63	1	1	1	1	1.93	1.92	2.03	2.02					495	55.7	9.23
Q96JK2	DDB1- and CUL4-associated factor 5 OS=Homo sapiens GN=DCAF5 PE=1 SV=2 - [DCAF5_HUMAN]	1.80	1	1	1	3	0.83	0.92	0.77	0.86					942	103.9	5.76
Q58WW2	DDB1- and CUL4-associated factor 6 OS=Homo sapiens GN=DCAF6 PE=1 SV=1 - [DCAF6_HUMAN]	1.40	1	1	1	1					0.78	0.71	0.86	0.78	860	96.2	5.27
Q9UER7	Death domain-associated protein 6 OS=Homo sapiens GN=DAXX PE=1 SV=2 - [DAXX_HUMAN]	3.24	1	1	1	1	0.75	0.65	0.84	0.73					740	81.3	4.87
Q9BTC0	Death-inducer obliterator 1 OS=Homo sapiens GN=DIDO1 PE=1 SV=5 - [DIDO1_HUMAN]	0.67	1	1	1	2	0.86	1.78	1.09	2.24	0.70	0.82	0.64	0.75	2240	243.7	7.88
P07585	Decorin OS=Homo sapiens GN=DCN PE=1 SV=1 - [PGS2_HUMAN]	4.18	1	1	1	3	1.23	1.45	1.22	1.45					359	39.7	8.54
Q5JSL3	Dedicator of cytokinesis protein 11 OS=Homo sapiens GN=DOCK11 PE=1 SV=2 - [DOC11_HUMAN]	0.48	1	1	1	4					0.46	0.03	0.72	0.04	2073	237.5	7.74
Q92608	Dedicator of cytokinesis protein 2 OS=Homo sapiens GN=DOCK2 PE=1 SV=2 - [DOCK2_HUMAN]	3.55	1	3	4	6	1.15	1.09	1.20	1.15	1.27	1.02	1.39	1.06	1830	211.8	6.87
Q8IZD9	Dedicator of cytokinesis protein 3 OS=Homo sapiens GN=DOCK3 PE=1 SV=1 - [DOCK3_HUMAN]	0.99	1	1	1	1	1.59	1.23	1.71	1.33					2030	233.0	6.98
Q8N1I0	Dedicator of cytokinesis protein 4 OS=Homo sapiens GN=DOCK4 PE=1 SV=3 - [DOCK4_HUMAN]	0.97	1	1	1	1	0.97	1.03	0.85	0.91					1966	225.1	7.65
Q9H7D0	Dedicator of cytokinesis protein 5 OS=Homo sapiens GN=DOCK5 PE=1 SV=3 - [DOCK5_HUMAN]	2.25	1	2	3	16	1.21	1.26	1.32	1.39					1870	215.2	7.96
Q96HP0	Dedicator of cytokinesis protein 6 OS=Homo sapiens GN=DOCK6 PE=1 SV=3 - [DOCK6_HUMAN]	1.47	1	1	1	1					0.89		1.78		2047	229.4	6.74
Q8NF50	Dedicator of cytokinesis protein 8 OS=Homo sapiens GN=DOCK8 PE=1 SV=3 - [DOCK8_HUMAN]	1.62	1	2	2	6	1.02	1.21	0.90	1.07	0.85	0.86	0.78	0.78	2099	238.4	6.87
Q9BZ29	Dedicator of cytokinesis protein 9 OS=Homo sapiens GN=DOCK9 PE=1 SV=2 - [DOCK9_HUMAN]	2.13	1	3	3	4	1.16	0.83	0.99	0.72					2069	236.3	7.49
Q01524	Defensin-6 OS=Homo sapiens GN=DEFA6 PE=1 SV=1 - [DEF6_HUMAN]	14.00	1	1	1	2	1.31	1.48	1.38	1.56					100	11.0	5.38
Q96LJ7	Dehydrogenase/reductase SDR family member 1 OS=Homo sapiens GN=DHRS1 PE=1 SV=1 - [DHRS1_HUMAN]	6.71	1	1	1	2	1.25	0.67	1.88	1.01					313	33.9	7.83
Q6IAN0	Dehydrogenase/reductase SDR family member 7B OS=Homo sapiens GN=DHRS7B PE=1 SV=2 - [DRS7B_HUMAN]	4.00	1	1	1	1	0.82	0.54	0.79	0.53					325	35.1	9.55
Q9UGM3	Deleted in malignant brain tumors 1 protein OS=Homo sapiens GN=DMBT1 PE=1 SV=2 - [DMBT1_HUMAN]	7.83	1	3	3	3	0.86	0.64	0.73	0.54	1.11	1.08	0.88	0.86	2413	260.6	5.44
A4D2P6	Delphilin OS=Homo sapiens GN=GRID2IP PE=2 SV=2 - [GRD2I_HUMAN]	2.56	1	1	1	1	1.64	1.77	1.58	1.73					1211	132.2	7.40
P13716	Delta-aminolevulinic acid dehydratase OS=Homo sapiens GN=ALAD PE=1 SV=1 - [HEM2_HUMAN]	13.33	1	3	3	5	0.59	0.68	0.57	0.67					330	36.3	6.79
O00548	Delta-like protein 1 OS=Homo sapiens GN=DLL1 PE=2 SV=2 - [DLL1_HUMAN]	1.24	1	1	1	1					0.73	0.10	1.06	0.14	723	78.0	6.24

P41143	Delta-type opioid receptor OS=Homo sapiens GN=OPRD1 PE=1 SV=4 - [OPRD_HUMAN]	4.84	1	2	2	11	2.87	1.89	0.94	0.71	1.11	0.88	1.11	0.92	372	40.3	8.92
Q08495	Dematin OS=Homo sapiens GN=EPB49 PE=1 SV=3 - [DEMA_HUMAN]	6.17	1	2	2	7					1.20	1.37	1.36	1.53	405	45.5	8.88
O94850	Dendrin OS=Homo sapiens GN=DDN PE=1 SV=3 - [DEND_HUMAN]	3.66	1	1	1	1	1.33	1.69	1.33	1.70					711	75.9	10.17
Q8TEH3	DENN domain-containing protein 1A OS=Homo sapiens GN=DENND1A PE=1 SV=2 - [DEN1A_HUMAN]	3.17	1	2	2	10	3.53	2.50	1.95	1.52	0.99	1.01	0.92	0.97	1009	110.5	6.96
Q5VZ89	DENN domain-containing protein 4C OS=Homo sapiens GN=DENND4C PE=1 SV=2 - [DEN4C_HUMAN]	1.14	1	1	1	1	0.94	0.95	0.92	0.94					1673	186.7	6.86
Q6IQ26	DENN domain-containing protein 5A OS=Homo sapiens GN=DENND5A PE=1 SV=2 - [DEN5A_HUMAN]	2.10	2	1	2	4	1.25	1.16	1.17	1.09					1287	147.0	6.65
Q13316	Dentin matrix acidic phosphoprotein 1 OS=Homo sapiens GN=DMP1 PE=1 SV=2 - [DMP1_HUMAN]	1.95	1	1	1	1	1.50	1.11	1.25	0.92					513	55.7	4.15
Q8IXL6	Dentin matrix protein 4 OS=Homo sapiens GN=FAM20C PE=1 SV=2 - [DMP4_HUMAN]	12.67	1	5	5	8	1.02	1.00	1.20	1.16	0.89	0.34	1.05	0.40	584	66.2	7.74
P27707	Deoxycytidine kinase OS=Homo sapiens GN=DCK PE=1 SV=1 - [DCK_HUMAN]	3.08	1	1	1	2	0.29		0.62						260	30.5	5.21
Q16854	Deoxyguanosine kinase, mitochondrial OS=Homo sapiens GN=DGUOK PE=1 SV=2 - [DGUOK_HUMAN]	6.50	1	1	1	1	0.79	0.58	0.87	0.65					277	32.0	8.66
P49366	Deoxyhypusine synthase OS=Homo sapiens GN=DHPS PE=1 SV=1 - [DHYS_HUMAN]	5.96	1	1	1	1					0.52	1.56	0.63	1.90	369	40.9	5.36
Q9H147	Deoxynucleotidyltransferase terminal-interacting protein 1 OS=Homo sapiens GN=DNTTIP1 PE=1 SV=2 - [TDIF1_HUMAN]	13.37	1	3	3	7	1.40	1.67	1.18	1.41	0.79	0.33	1.14	0.27	329	37.0	8.97
Q8TB45	DEP domain-containing mTOR-interacting protein OS=Homo sapiens GN=DEPTOR PE=1 SV=2 - [DPTOR_HUMAN]	2.20	1	1	1	1	1.41	1.37	1.11	1.09					409	46.3	8.07
Q5TB30	DEP domain-containing protein 1A OS=Homo sapiens GN=DEPDC1 PE=1 SV=2 - [DEP1A_HUMAN]	1.36	1	1	1	1							0.79		811	92.9	8.68
Q8WUY9	DEP domain-containing protein 1B OS=Homo sapiens GN=DEPDC1B PE=2 SV=2 - [DEP1B_HUMAN]	2.84	1	1	1	1					0.63	0.60	0.82	0.78	529	61.7	8.85
O75140	DEP domain-containing protein 5 OS=Homo sapiens GN=DEPDC5 PE=2 SV=1 - [DEPD5_HUMAN]	0.38	1	1	1	3					1.02	0.92	0.97	0.88	1572	177.8	6.73
Q8WVC6	Dephospho-CoA kinase domain-containing protein OS=Homo sapiens GN=DCAKD PE=1 SV=1 - [DCAKD_HUMAN]	8.23	1	1	1	1	0.95	1.00	0.99	1.05					231	26.5	9.58
Q96Q80	Derlin-3 OS=Homo sapiens GN=DERL3 PE=1 SV=2 - [DERL3_HUMAN]	3.83	1	1	1	3	2.97	4.16	2.73	3.87					235	26.7	8.48
Q9UL01	Dermatan-sulfate epimerase OS=Homo sapiens GN=DSE PE=1 SV=1 - [DSE_HUMAN]	4.18	1	2	2	4					1.27	1.31	1.06	1.08	958	109.7	8.09
Q07507	Dermatopontin OS=Homo sapiens GN=DPT PE=2 SV=2 - [DERM_HUMAN]	13.93	1	2	2	4					0.68	0.92	0.71	0.97	201	24.0	4.82
P81605	Dermcidin OS=Homo sapiens GN=DCD PE=1 SV=2 - [DCD_HUMAN]	38.18	1	5	6	33	0.85	0.74	1.25	0.97	1.62	1.48	1.68	1.53	110	11.3	6.54
Q6E0U4	Dermokine OS=Homo sapiens GN=DMKN PE=1 SV=3 - [DMKN_HUMAN]	4.83	1	2	2	2	0.63	0.81	0.64	0.84					476	47.1	7.28
Q08554	Desmocollin-1 OS=Homo sapiens GN=DSC1 PE=1 SV=2 - [DSC1_HUMAN]	8.28	1	5	5	10	1.32	1.21	1.30	1.20	1.20	1.20	1.10	1.11	894	99.9	5.43
Q14574	Desmocollin-3 OS=Homo sapiens GN=DSC3 PE=1 SV=3 - [DSC3_HUMAN]	2.46	1	3	3	5					1.09	1.36	1.25	1.17	896	99.9	6.10
Q02413	Desmoglein-1 OS=Homo sapiens GN=DSG1 PE=1 SV=2 - [DSG1_HUMAN]	5.53	1	3	3	3	0.69	0.78	0.74	0.85	0.91	1.06	2.46	2.70	1049	113.7	5.03
Q14126	Desmoglein-2 OS=Homo sapiens GN=DSG2 PE=1 SV=2 - [DSG2_HUMAN]	7.51	1	7	7	16	1.04	0.98	1.10	1.02	1.04	0.94	0.95	0.87	1118	122.2	5.24
P15924	Desmoplakin OS=Homo sapiens GN=DSP PE=1 SV=3 - [DESP_HUMAN]	1.22	1	3	3	10	0.92	0.74	0.64	0.52	0.95	0.79	1.10	1.25	2871	331.6	6.81

P60981	Destrin OS=Homo sapiens GN=DSTN PE=1 SV=3 - [DEST_HUMAN]	16.36	1	2	3	5					0.62	0.61	1.03	0.81	165	18.5	7.85
Q01459	Di-N-acetylchitobiase OS=Homo sapiens GN=CTBS PE=1 SV=1 - [DIAC_HUMAN]	17.92	1	6	6	15	1.40	1.27	1.46	1.29	1.10	1.15	0.95	1.14	385	43.7	6.64
Q16760	Diacylglycerol kinase delta OS=Homo sapiens GN=DGKD PE=1 SV=4 - [DGKD_HUMAN]	1.15	1	1	1	2	1.23	1.21	1.47	1.47	1.00	0.93	1.10	1.02	1214	134.4	7.56
Q86XP1	Diacylglycerol kinase eta OS=Homo sapiens GN=DGKH PE=1 SV=1 - [DGKH_HUMAN]	0.90	1	1	1	1					0.91	0.27	1.04	0.31	1220	134.8	6.54
O75912	Diacylglycerol kinase iota OS=Homo sapiens GN=DGKI PE=1 SV=1 - [DGKI_HUMAN]	1.50	1	1	1	1	1.20	1.31	1.10	1.21					1065	116.9	7.77
Q5KSL6	Diacylglycerol kinase kappa OS=Homo sapiens GN=DGKK PE=1 SV=1 - [DGKK_HUMAN]	1.10	1	2	2	3	0.16	0.07	0.15	0.07					1271	141.7	5.53
Q13574	Diacylglycerol kinase zeta OS=Homo sapiens GN=DGKZ PE=1 SV=3 - [DGKZ_HUMAN]	1.34	1	1	1	2					0.84	0.46	0.96	0.53	1117	124.1	9.04
Q6ZPD8	Diacylglycerol O-acyltransferase 2-like protein 6 OS=Homo sapiens GN=DGAT2L6 PE=2 SV=1 - [DG2L6_HUMAN]	4.15	1	1	1	2					1.46	1.34	1.18	1.08	337	38.6	9.86
P21673	Diamine acetyltransferase 1 OS=Homo sapiens GN=SAT1 PE=1 SV=1 - [SAT1_HUMAN]	2.92	1	1	1	1	0.72	1.17	0.62	1.02					171	20.0	5.16
Q9UBP4	Dickkopf-related protein 3 OS=Homo sapiens GN=DKK3 PE=1 SV=2 - [DKK3_HUMAN]	9.43	1	2	2	4	0.95	0.81	0.92	0.80					350	38.4	4.65
Q68CQ4	Digestive organ expansion factor homolog OS=Homo sapiens GN=DIEXF PE=1 SV=2 - [DIEXF_HUMAN]	6.88	1	3	4	9	0.68	0.85	0.84	1.10					756	87.0	5.88
Q14195	Dihydropyrimidinase-related protein 3 OS=Homo sapiens GN=DPYSL3 PE=1 SV=1 - [DPYL3_HUMAN]	2.28	1	1	1	1	0.91	0.99	0.92	1.02					570	61.9	6.49
Q12882	Dihydropyrimidine dehydrogenase [NADP(+)] OS=Homo sapiens GN=DPYD PE=1 SV=2 - [DPYD_HUMAN]	0.98	1	1	1	2	0.86	0.72	0.92	0.76	0.80	0.82	0.83	0.85	1025	111.3	7.05
O15228	Dihydroxyacetone phosphate acyltransferase OS=Homo sapiens GN=GNPAT PE=1 SV=1 - [GNPAT_HUMAN]	7.65	1	3	3	3	1.07	1.23	1.06	1.24	0.88	1.00	0.83	0.94	680	77.1	6.57
P31512	Dimethylaniline monooxygenase [N-oxide-forming] 4 OS=Homo sapiens GN=FMO4 PE=2 SV=3 - [FMO4_HUMAN]	3.41	1	1	1	1					0.45	0.45	0.46	0.47	558	63.3	8.46
Q9UI17	Dimethylglycine dehydrogenase, mitochondrial OS=Homo sapiens GN=DMGDH PE=1 SV=2 - [M2GD_HUMAN]	1.27	1	1	1	1	1.34	1.41	1.20	1.28					866	96.7	7.62
Q9H4A9	Dipeptidase 2 OS=Homo sapiens GN=DPEP2 PE=1 SV=2 - [DPEP2_HUMAN]	13.58	1	5	5	31	1.02	1.01	1.10	1.05	1.23	1.28	1.38	1.34	486	53.3	6.40
P53634	Dipeptidyl peptidase 1 OS=Homo sapiens GN=CTSC PE=1 SV=2 - [CATC_HUMAN]	2.59	1	1	1	8	0.82	0.91	0.75	0.85	1.05	1.01	1.16	1.15	463	51.8	6.99
Q9UHL4	Dipeptidyl peptidase 2 OS=Homo sapiens GN=DPP7 PE=1 SV=3 - [DPP2_HUMAN]	4.07	1	1	1	2	1.02	0.96	1.18	1.03					492	54.3	6.32
Q9NY33	Dipeptidyl peptidase 3 OS=Homo sapiens GN=DPP3 PE=1 SV=2 - [DPP3_HUMAN]	3.26	1	2	2	2	0.98	0.85	0.97	0.85					737	82.5	5.10
P27487	Dipeptidyl peptidase 4 OS=Homo sapiens GN=DPP4 PE=1 SV=2 - [DPP4_HUMAN]	2.87	1	3	3	6	0.87	0.93	0.89	0.96	1.27	0.76	1.53	0.80	766	88.2	6.04
O95989	Diphosphoinositol polyphosphate phosphohydrolase 1 OS=Homo sapiens GN=NUDT3 PE=1 SV=1 - [NUDT3_HUMAN]	8.14	1	1	1	3					1.17	1.08	1.21	1.11	172	19.5	6.34
Q9BZG8	Diphthamide biosynthesis protein 1 OS=Homo sapiens GN=DPH1 PE=1 SV=2 - [DPH1_HUMAN]	1.35	1	1	1	1	1.13	1.13	0.91	0.93					443	48.8	8.18
P98082	Disabled homolog 2 OS=Homo sapiens GN=DAB2 PE=1 SV=3 - [DAB2_HUMAN]	4.03	1	2	2	2							1.19	0.76	770	82.4	5.53
Q9P265	Disco-interacting protein 2 homolog B OS=Homo sapiens GN=DIP2B PE=1 SV=3 - [DIP2B_HUMAN]	1.02	1	1	1	1	1.42	1.18	1.25	1.04					1576	171.4	8.09
Q16832	Discoidin domain-containing receptor 2 OS=Homo sapiens GN=DDR2 PE=1 SV=2 - [DDR2_HUMAN]	2.69	1	1	1	5	0.97	0.92	1.05	1.00	1.39	1.42	1.32	1.35	855	96.7	5.36
Q9Y4D1	Disheveled-associated activator of morphogenesis 1 OS=Homo sapiens GN=DAAM1 PE=1 SV=2 - [DAAM1_HUMAN]	0.83	1	1	1	2	0.89	0.81	0.91	0.84					1078	123.4	7.23

O14672	Disintegrin and metalloproteinase domain-containing protein 10 OS=Homo sapiens GN=ADAM10 PE=1 SV=1 - [ADA10_HUMAN]	1.47	1	1	1	6	0.78	0.83	0.76	0.88					748	84.1	7.77
O43184	Disintegrin and metalloproteinase domain-containing protein 12 OS=Homo sapiens GN=ADAM12 PE=1 SV=3 - [ADA12_HUMAN]	12.54	1	9	9	27	3.05	3.48	3.25	3.61	5.92	5.81	5.36	5.68	909	99.5	8.40
Q9Y3Q7	Disintegrin and metalloproteinase domain-containing protein 18 OS=Homo sapiens GN=ADAM18 PE=2 SV=1 - [ADA18_HUMAN]	2.17	1	1	1	1					0.94	0.79	0.88	0.74	739	82.8	7.28
Q13443	Disintegrin and metalloproteinase domain-containing protein 9 OS=Homo sapiens GN=ADAM9 PE=1 SV=1 - [ADAM9_HUMAN]	3.66	1	2	2	20	0.69	0.60	0.70	0.61	1.20	0.68	1.22	1.18	819	90.5	7.52
Q15700	Disks large homolog 2 OS=Homo sapiens GN=DLG2 PE=1 SV=3 - [DLG2_HUMAN]	1.38	1	1	1	1	1.51	1.25	1.69	1.41					870	97.5	6.47
P78352	Disks large homolog 4 OS=Homo sapiens GN=DLG4 PE=1 SV=3 - [DLG4_HUMAN]	2.21	1	1	1	70	1.19	1.06	1.19	1.08	1.06	0.91	1.03	0.89	724	80.4	5.88
Q8TDM6	Disks large homolog 5 OS=Homo sapiens GN=DLG5 PE=1 SV=4 - [DLG5_HUMAN]	0.57	1	1	1	3	0.72	0.86	0.74	0.68					1919	213.7	7.42
O14490	Disks large-associated protein 1 OS=Homo sapiens GN=DLGAP1 PE=1 SV=1 - [DLGP1_HUMAN]	1.54	1	1	1	3	0.94	0.64	1.14	0.78	2.49	3.43	1.99	2.74	977	108.8	7.08
O95886	Disks large-associated protein 3 OS=Homo sapiens GN=DLGAP3 PE=1 SV=3 - [DLGP3_HUMAN]	1.63	1	1	1	1					0.98	0.92	1.07	1.00	979	106.0	8.76
Q9Y2H0	Disks large-associated protein 4 OS=Homo sapiens GN=DLGAP4 PE=1 SV=3 - [DLGP4_HUMAN]	0.81	1	1	1	2					2.33	2.33	1.87	1.86	992	107.9	7.08
Q969H9	Disrupted in renal carcinoma protein 1 OS=Homo sapiens GN=DIRC1 PE=2 SV=1 - [DIRC1_HUMAN]	7.69	1	1	1	2					0.51	0.92	0.65	0.99	104	11.4	8.37
Q96SL1	Disrupted in renal carcinoma protein 2 OS=Homo sapiens GN=DIRC2 PE=2 SV=1 - [DIRC2_HUMAN]	6.90	1	1	1	2	0.72	0.69	0.75	0.72					478	52.1	8.47
Q9Y485	DmX-like protein 1 OS=Homo sapiens GN=DMXL1 PE=1 SV=3 - [DMXL1_HUMAN]	3.50	1	5	5	7	1.01	1.22	0.86	1.05	0.94	0.81	0.82	0.70	3027	337.6	6.34
Q8TDJ6	DmX-like protein 2 OS=Homo sapiens GN=DMXL2 PE=1 SV=2 - [DMXL2_HUMAN]	1.22	1	2	3	27	0.82	1.15	0.59	0.84					3036	339.4	6.38
P26358	DNA (cytosine-5)-methyltransferase 1 OS=Homo sapiens GN=DNMT1 PE=1 SV=2 - [DNMT1_HUMAN]	1.05	1	1	1	1	0.58	0.35	0.66	0.41					1616	183.0	7.75
Q6PJP8	DNA cross-link repair 1A protein OS=Homo sapiens GN=DCLRE1A PE=1 SV=3 - [DCR1A_HUMAN]	1.63	1	2	2	2	1.31	1.37	1.82	1.92	1.26	1.44	0.71	0.81	1040	116.3	7.97
Q6NTF7	DNA dC->dU-editing enzyme APOBEC-3H OS=Homo sapiens GN=APOBEC3H PE=1 SV=3 - [ABC3H_HUMAN]	7.00	1	1	1	1					0.39	0.28	0.72	0.52	200	23.5	8.73
Q99708	DNA endonuclease RBBP8 OS=Homo sapiens GN=RBBP8 PE=1 SV=2 - [COM1_HUMAN]	0.89	1	1	1	3	0.92	1.00	0.88	0.97					897	101.9	6.30
Q2NKX8	DNA excision repair protein ERCC-6-like OS=Homo sapiens GN=ERCC6L PE=1 SV=1 - [ERC6L_HUMAN]	3.36	1	2	3	3	0.62	0.17	0.88	0.24	2.20	1.95	2.46	2.18	1250	141.0	5.31
O00273	DNA fragmentation factor subunit alpha OS=Homo sapiens GN=DFFA PE=1 SV=1 - [DFFA_HUMAN]	16.31	1	4	4	8					1.08	1.19	1.16	1.04	331	36.5	4.79
Q8NG08	DNA helicase B OS=Homo sapiens GN=HELB PE=1 SV=2 - [HELB_HUMAN]	1.10	1	1	1	1					4.79	3.32	4.47	3.10	1087	123.2	5.87
P40692	DNA mismatch repair protein Mlh1 OS=Homo sapiens GN=MLH1 PE=1 SV=1 - [MLH1_HUMAN]	1.32	1	1	1	1	2.02	2.43	1.98	2.39					756	84.5	5.72
Q9UHC1	DNA mismatch repair protein Mlh3 OS=Homo sapiens GN=MLH3 PE=1 SV=3 - [MLH3_HUMAN]	2.41	1	2	2	7		0.58		1.41	1.04	1.03	1.04	1.01	1453	163.6	6.73
P43246	DNA mismatch repair protein Msh2 OS=Homo sapiens GN=MSH2 PE=1 SV=1 - [MSH2_HUMAN]	3.53	1	2	2	4					1.36	0.85	1.14	0.71	934	104.7	5.77
P20585	DNA mismatch repair protein Msh3 OS=Homo sapiens GN=MSH3 PE=1 SV=4 - [MSH3_HUMAN]	1.50	1	1	1	1					1.19	1.13	1.10	1.05	1137	127.3	8.02
P52701	DNA mismatch repair protein Msh6 OS=Homo sapiens GN=MSH6 PE=1 SV=2 - [MSH6_HUMAN]	1.32	1	1	1	2					0.86	0.81	0.92	0.87	1360	152.7	6.90
P04053	DNA nucleotidylexotransferase OS=Homo sapiens GN=DNTT PE=1 SV=3 - [TDT_HUMAN]	2.95	1	1	1	2					1.06	1.30	0.92	1.12	509	58.5	8.43

Q9Y253	DNA polymerase eta OS=Homo sapiens GN=POLH PE=1 SV=1 - [POLH_HUMAN]	1.96	1	1	1	1	1.16	1.12	1.06	1.03					713	78.4	8.44
Q9UNA4	DNA polymerase iota OS=Homo sapiens GN=POLI PE=1 SV=3 - [POLI_HUMAN]	1.62	1	1	1	1					0.36	0.30	0.47	0.40	740	83.0	6.07
Q9UGP5	DNA polymerase lambda OS=Homo sapiens GN=POLL PE=1 SV=1 - [DPOLL_HUMAN]	4.17	1	2	2	3	1.24	1.14	1.36	1.25	0.80	0.68	0.59	0.50	575	63.4	7.87
Q9UHN1	DNA polymerase subunit gamma-2, mitochondrial OS=Homo sapiens GN=POLG2 PE=1 SV=1 - [DPOG2_HUMAN]	6.60	1	2	2	2					1.66	1.53	1.62	1.49	485	54.9	8.35
O75417	DNA polymerase theta OS=Homo sapiens GN=POLQ PE=1 SV=2 - [DPOLQ_HUMAN]	5.10	1	7	7	13	1.01	1.39	0.99	1.34	0.91	1.13	0.76	0.94	2590	289.4	7.36
O60673	DNA polymerase zeta catalytic subunit OS=Homo sapiens GN=REV3L PE=1 SV=2 - [DPOLZ_HUMAN]	2.30	1	4	4	13	0.15	0.31	0.52	1.11	0.94	0.81	0.93	0.80	3130	352.6	8.47
Q9Y620	DNA repair and recombination protein RAD54B OS=Homo sapiens GN=RAD54B PE=1 SV=1 - [RA54B_HUMAN]	3.41	1	2	2	9	1.26	1.55	1.29	1.49	1.05	1.08	1.03	1.06	910	102.9	8.12
Q92889	DNA repair endonuclease XPF OS=Homo sapiens GN=ERCC4 PE=1 SV=3 - [XPF_HUMAN]	1.97	1	1	1	2							1.12	0.11	916	104.4	6.93
Q92878	DNA repair protein RAD50 OS=Homo sapiens GN=RAD50 PE=1 SV=1 - [RAD50_HUMAN]	1.98	7	1	2	2	1.38	1.26	1.49	1.36					1312	153.8	6.89
O43502	DNA repair protein RAD51 homolog 3 OS=Homo sapiens GN=RAD51C PE=1 SV=1 - [RA51C_HUMAN]	4.52	1	1	1	1					0.88	0.74	0.87	0.73	376	42.2	6.74
O75771	DNA repair protein RAD51 homolog 4 OS=Homo sapiens GN=RAD51D PE=1 SV=1 - [RA51D_HUMAN]	4.88	1	1	1	10	0.97	0.97	0.98	0.98	1.22	1.33	1.18	1.29	328	35.0	6.25
Q1ZZU3	DNA repair protein SWI5 homolog OS=Homo sapiens GN=SWI5 PE=1 SV=1 - [SWI5_HUMAN]	11.49	4	1	2	3	0.39	0.19	0.68	0.33					235	26.7	10.24
O43542	DNA repair protein XRCC3 OS=Homo sapiens GN=XRCC3 PE=1 SV=1 - [XRCC3_HUMAN]	9.83	1	1	1	1			1.25	1.91					346	37.8	8.48
Q9Y248	DNA replication complex GINS protein PSF2 OS=Homo sapiens GN=GINS2 PE=1 SV=1 - [PSF2_HUMAN]	6.49	1	1	1	1	1.00	1.20	0.87	1.04					185	21.4	5.44
Q9H211	DNA replication factor Cdt1 OS=Homo sapiens GN=CDT1 PE=1 SV=3 - [CDT1_HUMAN]	3.48	1	1	1	1	1.60	1.25	1.40	1.10					546	60.4	9.77
P49736	DNA replication licensing factor MCM2 OS=Homo sapiens GN=MCM2 PE=1 SV=4 - [MCM2_HUMAN]	5.86	1	4	4	6	1.06	0.95	1.03	0.93	0.75	0.96	0.77	1.00	904	101.8	5.52
P33992	DNA replication licensing factor MCM5 OS=Homo sapiens GN=MCM5 PE=1 SV=5 - [MCM5_HUMAN]	6.13	1	2	2	4	2.27	5.82	2.17	6.41					734	82.2	8.37
Q14566	DNA replication licensing factor MCM6 OS=Homo sapiens GN=MCM6 PE=1 SV=1 - [MCM6_HUMAN]	1.46	1	1	1	1	1.05	1.11	1.18	1.24					821	92.8	5.41
Q9NXL9	DNA replication licensing factor MCM9 OS=Homo sapiens GN=MCM9 PE=1 SV=4 - [MCM9_HUMAN]	0.87	1	1	1	1	0.21	0.19	0.19	0.18					1143	127.2	7.74
Q02880	DNA topoisomerase 2-beta OS=Homo sapiens GN=TOP2B PE=1 SV=3 - [TOP2B_HUMAN]	1.78	1	1	2	6	1.09	1.26	1.13	1.32					1626	183.2	8.00
Q92547	DNA topoisomerase 2-binding protein 1 OS=Homo sapiens GN=TOPBP1 PE=1 SV=3 - [TOPB1_HUMAN]	1.58	1	2	2	2	1.07	1.09	1.18	1.20					1522	170.6	6.96
P29372	DNA-3-methyladenine glycosylase OS=Homo sapiens GN=MPG PE=1 SV=3 - [3MG_HUMAN]	3.36	1	1	1	1					1.11	1.01	0.94	0.86	298	32.8	9.57
Q8WXF8	DNA-binding death effector domain-containing protein 2 OS=Homo sapiens GN=DEDD2 PE=1 SV=1 - [DEDD2_HUMAN]	3.99	1	1	1	1					####	###	###	####	326	36.2	8.97
P16989	DNA-binding protein A OS=Homo sapiens GN=CSDA PE=1 SV=4 - [DBPA_HUMAN]	3.49	1	1	1	1					0.90	0.76	0.76	0.64	372	40.1	9.77
Q2KHR2	DNA-binding protein RFX7 OS=Homo sapiens GN=RFX7 PE=1 SV=1 - [RFX7_HUMAN]	1.10	1	1	1	1	1.47	1.53	1.48	1.55					1363	146.8	6.76
O14593	DNA-binding protein RFXANK OS=Homo sapiens GN=RFXANK PE=1 SV=2 - [RFXK_HUMAN]	5.38	1	1	1	1	0.84	0.89	0.95	1.01					260	28.1	4.55
P78527	DNA-dependent protein kinase catalytic subunit OS=Homo sapiens GN=PRKDC PE=1 SV=3 - [PRKDC_HUMAN]	1.79	1	3	4	5					0.39	0.43	0.30	0.34	4128	468.8	7.12

O95602	DNA-directed RNA polymerase I subunit RPA1 OS=Homo sapiens GN=POLR1A PE=1 SV=2 - [RPA1_HUMAN]	3.02	1	3	3	4					1.16	1.09	1.12	1.08	1720	194.7	7.03
O15446	DNA-directed RNA polymerase I subunit RPA34 OS=Homo sapiens GN=CD3EAP PE=1 SV=1 - [RPA34_HUMAN]	1.37	1	1	1	1					0.62	0.13	0.63	0.13	510	55.0	8.51
P24928	DNA-directed RNA polymerase II subunit RPB1 OS=Homo sapiens GN=POLR2A PE=1 SV=2 - [RPB1_HUMAN]	2.59	1	3	3	4	0.83	0.84	0.81	0.83	####	5.17	###	5.49	1970	217.0	7.37
P30876	DNA-directed RNA polymerase II subunit RPB2 OS=Homo sapiens GN=POLR2B PE=1 SV=1 - [RPB2_HUMAN]	1.28	1	2	2	2					1.32	1.30	1.28	1.25	1174	133.8	6.87
P62487	DNA-directed RNA polymerase II subunit RPB7 OS=Homo sapiens GN=POLR2G PE=1 SV=1 - [RPB7_HUMAN]	11.05	1	1	1	1					0.48	0.18	1.04	0.40	172	19.3	5.54
O14802	DNA-directed RNA polymerase III subunit RPC1 OS=Homo sapiens GN=POLR3A PE=1 SV=2 - [RPC1_HUMAN]	1.80	1	2	2	2					0.89	0.67	0.93	0.71	1390	155.5	8.48
P31689	DnaJ homolog subfamily A member 1 OS=Homo sapiens GN=DNAJA1 PE=1 SV=2 - [DNJA1_HUMAN]	4.79	1	1	1	2	1.05	0.95	1.55	1.40					397	44.8	7.08
O60884	DnaJ homolog subfamily A member 2 OS=Homo sapiens GN=DNAJA2 PE=1 SV=1 - [DNJA2_HUMAN]	9.71	1	2	2	2	2.25	1.98	1.03	0.91					412	45.7	6.48
Q9UBS4	DnaJ homolog subfamily B member 11 OS=Homo sapiens GN=DNAJB11 PE=1 SV=1 - [DJB11_HUMAN]	9.22	1	2	3	6	1.08	1.01	1.06	1.00	0.99	0.97	1.09	1.07	358	40.5	6.18
P59910	DnaJ homolog subfamily B member 13 OS=Homo sapiens GN=DNAJB13 PE=2 SV=1 - [DJB13_HUMAN]	3.16	1	2	2	2	0.72	0.63	0.94	0.83	0.77	0.75	1.01	0.98	316	36.1	7.87
Q8TBM8	DnaJ homolog subfamily B member 14 OS=Homo sapiens GN=DNAJB14 PE=2 SV=1 - [DJB14_HUMAN]	2.64	1	1	1	1					####	###	1.55	1.65	379	42.5	8.59
O75165	DnaJ homolog subfamily C member 13 OS=Homo sapiens GN=DNAJC13 PE=1 SV=5 - [DJC13_HUMAN]	0.62	1	1	1	1					0.94	1.02	0.98	1.07	2243	254.3	6.74
Q9NVM6	DnaJ homolog subfamily C member 17 OS=Homo sapiens GN=DNAJC17 PE=1 SV=1 - [DJC17_HUMAN]	7.24	1	2	2	3					0.81	0.91	0.79	0.84	304	34.7	8.53
Q99543	DnaJ homolog subfamily C member 2 OS=Homo sapiens GN=DNAJC2 PE=1 SV=4 - [DNJC2_HUMAN]	2.58	1	1	1	1					1.12	1.15	2.01	2.07	621	72.0	8.70
Q9NNZ3	DnaJ homolog subfamily C member 4 OS=Homo sapiens GN=DNAJC4 PE=2 SV=1 - [DNJC4_HUMAN]	4.15	1	1	1	1					0.89	0.93	1.02	1.06	241	27.6	10.55
Q99704	Docking protein 1 OS=Homo sapiens GN=DOK1 PE=1 SV=1 - [DOK1_HUMAN]	4.99	1	3	3	4	0.81	0.86	0.70	0.75	0.71	0.44	0.63	0.38	481	52.4	6.47
O60496	Docking protein 2 OS=Homo sapiens GN=DOK2 PE=1 SV=2 - [DOK2_HUMAN]	8.01	1	2	2	4	0.93	0.87	1.01	0.94	1.26	1.04	0.97	0.80	412	45.4	6.02
Q7L591	Docking protein 3 OS=Homo sapiens GN=DOK3 PE=1 SV=2 - [DOK3_HUMAN]	14.11	1	4	4	13	1.32	1.22	1.59	1.47	0.86	1.05	0.93	1.01	496	53.3	7.77
Q6PKX4	Docking protein 6 OS=Homo sapiens GN=DOK6 PE=1 SV=1 - [DOK6_HUMAN]	3.93	1	1	1	2	1.13	0.97	1.07	0.92					331	38.3	8.48
Q5BKT4	Dol-P-Glc:Glc(2)Man(9)GlcNAc(2)-PP-Dol alpha-1,2-glucosyltransferase OS=Homo sapiens GN=ALG10 PE=1 SV=1 - [AG10A_HUMAN]	4.65	1	1	1	1					0.91				473	55.6	9.29
P46977	Dolichyl-diphosphooligosaccharide--protein glycosyltransferase subunit STT3A OS=Homo sapiens GN=STT3A PE=1 SV=2 - [STT3A_HUMAN]	3.55	1	2	2	3					0.95	1.04	0.94	1.02	705	80.5	8.07
P09172	Dopamine beta-hydroxylase OS=Homo sapiens GN=DBH PE=1 SV=3 - [DOPO_HUMAN]	18.80	1	10	10	48	1.17	1.19	1.13	1.19	1.13	1.30	1.04	1.19	617	69.0	6.42
Q6RFH8	Double homeobox protein 4C OS=Homo sapiens GN=DUX4L9 PE=1 SV=1 - [DUX4C_HUMAN]	2.94	4	1	1	1					1.99	2.04	1.08	1.10	374	39.4	11.11
Q96PT3	Double homeobox protein 5 OS=Homo sapiens GN=DUX5 PE=2 SV=1 - [DUX5_HUMAN]	13.71	1	1	1	1	0.87	0.65	0.97	0.72					197	22.2	10.65
O95793	Double-stranded RNA-binding protein Staufen homolog 1 OS=Homo sapiens GN=STAU1 PE=1 SV=2 - [STAU1_HUMAN]	3.29	1	1	2	2	1.05	1.10	1.02	1.07					577	63.1	9.44
P55265	Double-stranded RNA-specific adenosine deaminase OS=Homo sapiens GN=ADAR PE=1 SV=4 - [DSRAD_HUMAN]	3.51	1	3	3	3	1.53	1.27	1.65	1.37	1.01	0.97	0.91	0.87	1226	136.0	8.65
P78563	Double-stranded RNA-specific editase 1 OS=Homo sapiens GN=ADARB1 PE=1 SV=1 - [RED1_HUMAN]	1.21	1	1	1	23	0.80	0.78	0.84	0.81	1.00	1.06	0.94	1.00	741	80.7	9.01

Q9Y5R6	Doublesex- and mab-3-related transcription factor 1 OS=Homo sapiens GN=DMRT1 PE=1 SV=2 - [DMRT1_HUMAN]	2.95	1	1	1	1					1.45	0.74	1.34	0.68	373	39.4	7.61
Q5VZB9	Doublesex- and mab-3-related transcription factor A1 OS=Homo sapiens GN=DMRTA1 PE=2 SV=1 - [DMRTA_HUMAN]	2.98	1	1	1	1	0.71	0.73	0.77	0.79					504	53.1	8.90
O60469	Down syndrome cell adhesion molecule OS=Homo sapiens GN=DSCAM PE=1 SV=2 - [DSCAM_HUMAN]	1.39	1	1	2	32	1.17	1.01	1.10	0.96					2012	222.1	7.68
Q8TD84	Down syndrome cell adhesion molecule-like protein 1 OS=Homo sapiens GN=DSCAML1 PE=1 SV=2 - [DSCL1_HUMAN]	1.66	1	1	2	32					1.12	1.61	0.98	1.40	2053	224.3	8.16
P59022	Down syndrome critical region protein 10 OS=Homo sapiens GN=DSCR10 PE=2 SV=1 - [DSC10_HUMAN]	17.24	1	1	1	1					1.17	1.15	0.67	0.66	87	9.3	5.60
P56555	Down syndrome critical region protein 4 OS=Homo sapiens GN=DSCR4 PE=2 SV=1 - [DSCR4_HUMAN]	6.78	1	1	1	4					0.84	0.74	0.91	0.92	118	12.9	7.91
Q8NBI3	Draxin OS=Homo sapiens GN=C1orf187 PE=1 SV=2 - [DRAXI_HUMAN]	5.44	1	1	1	2					0.58	0.69	0.75	0.88	349	38.6	7.69
Q16643	Drebrin OS=Homo sapiens GN=DBN1 PE=1 SV=4 - [DREB_HUMAN]	4.16	1	2	2	5					2.75	2.87	2.72	2.84	649	71.4	4.45
Q9UJU6	Drebrin-like protein OS=Homo sapiens GN=DBNL PE=1 SV=1 - [DBNL_HUMAN]	13.02	1	4	4	16	1.00	0.96	1.09	1.05	1.27	1.40	1.25	1.42	430	48.2	5.05
Q8NBA8	DTW domain-containing protein 2 OS=Homo sapiens GN=DTWD2 PE=2 SV=1 - [DTWD2_HUMAN]	11.07	1	1	1	1							0.54		298	33.4	8.68
Q9UN19	Dual adapter for phosphotyrosine and 3-phosphotyrosine and 3-phosphoinositide OS=Homo sapiens GN=DAPP1 PE=1 SV=1 - [DAPP1_HUMAN]	4.29	1	1	1	4	1.37	0.93	0.87	0.60	1.00	1.05	0.86	0.90	280	32.2	7.80
Q9NRD9	Dual oxidase 1 OS=Homo sapiens GN=DUOX1 PE=1 SV=1 - [DUOX1_HUMAN]	1.74	2	2	3	3					0.86	0.16	0.86	0.16	1551	177.1	7.90
Q6XUX3	Dual serine/threonine and tyrosine protein kinase OS=Homo sapiens GN=DSTYK PE=1 SV=2 - [DUSTY_HUMAN]	4.52	1	3	3	4	1.49	1.26	1.46	1.24	0.93	0.95	1.05	1.08	929	105.1	6.74
Q68J44	Dual specificity phosphatase DUPD1 OS=Homo sapiens GN=DUPD1 PE=1 SV=1 - [DUPD1_HUMAN]	5.00	1	1	1	1	1.23	1.16	1.05	1.00					220	25.3	6.04
P33981	Dual specificity protein kinase TTK OS=Homo sapiens GN=TTK PE=1 SV=2 - [TTK_HUMAN]	5.02	2	3	4	8	1.25	0.92	1.48	1.07					857	97.0	8.16
Q9H596	Dual specificity protein phosphatase 21 OS=Homo sapiens GN=DUSP21 PE=1 SV=1 - [DUS21_HUMAN]	3.68	1	1	1	1	2.63	2.68	2.56	2.63					190	21.5	8.82
P51452	Dual specificity protein phosphatase 3 OS=Homo sapiens GN=DUSP3 PE=1 SV=1 - [DUS3_HUMAN]	36.22	1	6	6	10	0.89	0.95	0.97	1.06	1.06	1.17	1.87	0.58	185	20.5	7.80
Q15569	Dual specificity testis-specific protein kinase 1 OS=Homo sapiens GN=TESK1 PE=1 SV=2 - [TESK1_HUMAN]	3.19	1	1	1	2	1.16	1.14	1.09	1.07	1.08	1.19	1.13	1.24	626	67.6	8.10
Q96S53	Dual specificity testis-specific protein kinase 2 OS=Homo sapiens GN=TESK2 PE=2 SV=1 - [TESK2_HUMAN]	2.63	1	1	1	3	0.90	0.74	0.78	0.65	0.89	1.01	0.84	0.96	571	63.6	7.06
Q9NR20	Dual specificity tyrosine-phosphorylation-regulated kinase 4 OS=Homo sapiens GN=DYRK4 PE=1 SV=2 - [DYRK4_HUMAN]	1.35	1	1	1	5	1.02	1.19	0.82	1.33					520	59.6	8.90
Q7RTS9	Dymeclin OS=Homo sapiens GN=DYM PE=1 SV=1 - [DYM_HUMAN]	3.74	1	2	2	2	1.76	2.02	1.82	2.10					669	75.9	5.86
Q14203	Dynactin subunit 1 OS=Homo sapiens GN=DCTN1 PE=1 SV=3 - [DCTN1_HUMAN]	3.29	1	3	3	6	0.96	0.80	0.98	0.83	0.75	1.05	1.10	1.55	1278	141.6	5.81
Q9UJW0	Dynactin subunit 4 OS=Homo sapiens GN=DCTN4 PE=1 SV=1 - [DCTN4_HUMAN]	1.30	1	1	1	1	1.07	0.82	1.11	0.86					460	52.3	7.34
O00429	Dynamin-1-like protein OS=Homo sapiens GN=DNM1L PE=1 SV=2 - [DNM1L_HUMAN]	7.47	1	4	4	7	0.84	0.80	0.73	0.71	2.45	2.27	2.29	2.13	736	81.8	6.81
Q8NEP3	Dynein assembly factor 1, axonemal OS=Homo sapiens GN=DNAAF1 PE=1 SV=5 - [DAAF1_HUMAN]	3.03	1	1	1	3	0.63	0.33	0.91	0.48	0.77	0.79	0.70	0.71	725	80.0	4.67
Q9P2D7	Dynein heavy chain 1, axonemal OS=Homo sapiens GN=DNAH1 PE=1 SV=4 - [DYH1_HUMAN]	1.96	1	4	4	6	1.05	1.06	0.92	0.95	1.29	0.25	1.04	0.22	4330	493.6	5.94
Q8IVF4	Dynein heavy chain 10, axonemal OS=Homo sapiens GN=DNAH10 PE=1 SV=4 - [DYH10_HUMAN]	1.12	8	3	5	5	0.91	0.99	1.13	1.23	0.72	1.42	0.91	1.79	4471	514.5	5.88

Q96DT5	Dynein heavy chain 11, axonemal OS=Homo sapiens GN=DNAH11 PE=1 SV=3 - [DYH11_HUMAN]	0.91	8	2	3	4	1.02	1.03	0.91	0.92		0.98		1.31	4523	520.7	6.44
Q0VDD8	Dynein heavy chain 14, axonemal OS=Homo sapiens GN=DNAH14 PE=2 SV=3 - [DYH14_HUMAN]	0.97	1	1	2	44					0.49	0.34	0.73	0.58	3507	399.6	6.93
Q9UFH2	Dynein heavy chain 17, axonemal OS=Homo sapiens GN=DNAH17 PE=2 SV=2 - [DYH17_HUMAN]	0.45	7	2	3	25	1.16	1.18	1.27	1.32	0.05	0.23	0.43	1.88	4485	511.5	5.77
Q9P225	Dynein heavy chain 2, axonemal OS=Homo sapiens GN=DNAH2 PE=1 SV=3 - [DYH2_HUMAN]	0.32	1	1	1	1	0.90	0.84	0.87	0.82					4427	507.4	6.37
Q8TD57	Dynein heavy chain 3, axonemal OS=Homo sapiens GN=DNAH3 PE=2 SV=1 - [DYH3_HUMAN]	1.72	1	2	3	4	1.24	1.29	0.95	1.06					4116	470.5	6.43
Q8TE73	Dynein heavy chain 5, axonemal OS=Homo sapiens GN=DNAH5 PE=1 SV=3 - [DYH5_HUMAN]	1.23	8	3	5	20	0.90	1.32	1.12	1.65	1.18	1.21	1.24	1.27	4624	528.7	6.10
Q9C0G6	Dynein heavy chain 6, axonemal OS=Homo sapiens GN=DNAH6 PE=1 SV=3 - [DYH6_HUMAN]	0.87	9	3	4	5	2.38	2.28	2.49	2.41	1.24	1.18	1.15	1.09	4158	475.7	6.00
Q8WXX0	Dynein heavy chain 7, axonemal OS=Homo sapiens GN=DNAH7 PE=1 SV=2 - [DYH7_HUMAN]	0.92	1	3	3	5	1.30	1.15	1.40	1.26	1.41	1.16	0.95	0.78	4024	460.9	6.00
Q96JB1	Dynein heavy chain 8, axonemal OS=Homo sapiens GN=DNAH8 PE=1 SV=2 - [DYH8_HUMAN]	2.65	11	6	9	28	2.30	2.05	0.90	0.81	0.94	0.79	1.06	0.94	4490	514.3	6.32
Q9NYC9	Dynein heavy chain 9, axonemal OS=Homo sapiens GN=DNAH9 PE=1 SV=3 - [DYH9_HUMAN]	0.74	1	2	2	2	1.27	1.65	0.97	1.27					4486	511.6	5.91
Q96M86	Dynein heavy chain domain-containing protein 1 OS=Homo sapiens GN=DNHD1 PE=1 SV=2 - [DNHD1_HUMAN]	1.51	1	4	4	18	1.12	1.10	1.16	1.14	1.01	0.88	1.03	0.89	4753	533.3	6.71
Q96FJ2	Dynein light chain 2, cytoplasmic OS=Homo sapiens GN=DYNLL2 PE=1 SV=1 - [DYL2_HUMAN]	24.72	1	1	1	1	1.02	0.71	0.61	0.42					89	10.3	7.37
O75923	Dysferlin OS=Homo sapiens GN=DYSF PE=1 SV=1 - [DYSF_HUMAN]	1.20	1	1	1	1	0.54	0.70	0.70	0.92					2080	237.1	5.64
Q8IZA0	Dyslexia-associated protein KIAA0319-like protein OS=Homo sapiens GN=KIAA0319L PE=1 SV=2 - [K319L_HUMAN]	0.76	1	1	1	3					0.90	1.14	1.09	1.38	1049	115.6	6.05
Q03001	Dystonin OS=Homo sapiens GN=DST PE=1 SV=4 - [DYST_HUMAN]	2.22	7	10	12	23	1.04	0.86	0.97	0.85	1.13	0.99	1.25	1.05	7570	860.1	5.25
Q9Y4J8	Dystrobrevin alpha OS=Homo sapiens GN=DTNA PE=1 SV=2 [DTNA_HUMAN]	2.29	1	1	1	3			1.21	0.77					743	83.8	6.89
Q14118	Dystroglycan OS=Homo sapiens GN=DAG1 PE=1 SV=2 - [DAG1_HUMAN]	16.42	1	10	10	34	1.20	1.13	1.18	1.08	0.95	0.86	0.90	0.83	895	97.4	8.56
P11532	Dystrophin OS=Homo sapiens GN=DMD PE=1 SV=3 - [DMD_HUMAN]	1.00	2	2	3	5	0.95	1.11	1.12	1.34					3685	426.5	5.88
Q96L91	E1A-binding protein p400 OS=Homo sapiens GN=EP400 PE=1 SV=4 - [EP400_HUMAN]	1.17	1	1	1	1					0.91		0.82		3159	343.3	9.19
Q9UII4	E3 ISG15--protein ligase HERC5 OS=Homo sapiens GN=HERC5 PE=1 SV=2 - [HERC5_HUMAN]	1.86	1	1	1	1					1.99	1.98	2.12	2.11	1024	116.8	7.65
P49792	E3 SUMO-protein ligase RanBP2 OS=Homo sapiens GN=RANBP2 PE=1 SV=2 - [RBP2_HUMAN]	1.83	2	2	5	16	1.61	1.37	1.20	1.02	0.60	0.87	0.40	0.58	3224	358.0	6.20
Q6ZNA4	E3 ubiquitin-protein ligase Arkadia OS=Homo sapiens GN=RNF111 PE=1 SV=3 - [RN111_HUMAN]	1.21	1	1	1	1					0.26	0.22	0.52	0.45	994	108.8	7.15
Q5VTR2	E3 ubiquitin-protein ligase BRE1A OS=Homo sapiens GN=RNF20 PE=1 SV=2 - [BRE1A_HUMAN]	0.92	1	1	1	1					1.62	0.66			975	113.6	5.94
O75150	E3 ubiquitin-protein ligase BRE1B OS=Homo sapiens GN=RNF40 PE=1 SV=4 - [BRE1B_HUMAN]	3.90	1	2	2	2	1.42	1.19	1.59	1.33	0.74	0.71	0.90	0.86	1001	113.6	6.23
P22681	E3 ubiquitin-protein ligase CBL OS=Homo sapiens GN=CBL PE=1 SV=2 - [CBL_HUMAN]	1.43	1	1	1	1					0.73	0.73	0.69	0.69	906	99.6	6.54
Q9NW38	E3 ubiquitin-protein ligase FANCL OS=Homo sapiens GN=FANCL PE=1 SV=2 - [FANCL_HUMAN]	8.53	1	2	2	3	1.08	1.13	0.89	0.93	1.51	1.26	1.76	1.47	375	42.9	6.46
Q8IYU2	E3 ubiquitin-protein ligase HACE1 OS=Homo sapiens GN=HACE1 PE=1 SV=2 - [HACE1_HUMAN]	3.74	1	1	1	1					2.30	1.83	2.81	2.24	909	102.3	5.92

Q9ULT8	E3 ubiquitin-protein ligase HECTD1 OS=Homo sapiens GN=HECTD1 PE=1 SV=3 - [HECD1_HUMAN]	2.15	1	3	3	4	0.80	0.89	0.95	1.05	0.85	0.87	0.93	0.95	2610	289.2	5.35
Q76N89	E3 ubiquitin-protein ligase HECW1 OS=Homo sapiens GN=HECW1 PE=1 SV=3 - [HECW1_HUMAN]	2.86	1	2	2	2	0.96	0.92	1.03	0.99					1606	179.4	5.50
O95714	E3 ubiquitin-protein ligase HERC2 OS=Homo sapiens GN=HERC2 PE=1 SV=2 - [HERC2_HUMAN]	0.27	1	1	1	1	0.61	0.61	0.58	0.58					4834	526.9	6.28
Q96J02	E3 ubiquitin-protein ligase Itchy homolog OS=Homo sapiens GN=ITCH PE=1 SV=2 - [ITCH_HUMAN]	1.55	1	1	1	1	1.10	0.96	0.98	0.85					903	102.7	6.30
Q86YJ5	E3 ubiquitin-protein ligase MARCH9 OS=Homo sapiens GN=MARCH9 PE=1 SV=2 - [MARH9_HUMAN]	4.62	1	2	2	3					1.11	1.07	1.14	1.11	346	37.7	9.13
Q96AX9	E3 ubiquitin-protein ligase MIB2 OS=Homo sapiens GN=MIB2 PE=1 SV=3 - [MIB2_HUMAN]	5.13	1	3	3	3					0.38	0.31	0.72	0.59	1013	109.9	8.44
O60260	E3 ubiquitin-protein ligase parkin OS=Homo sapiens GN=PARK2 PE=1 SV=2 - [PRKN2_HUMAN]	2.37	1	1	1	1	0.86		0.82						465	51.6	7.06
Q8N2H9	E3 ubiquitin-protein ligase pellino homolog 3 OS=Homo sapiens GN=PELI3 PE=1 SV=2 - [PELI3_HUMAN]	5.33	1	1	1	3					0.87	0.59	0.69	0.47	469	50.7	7.44
O43164	E3 ubiquitin-protein ligase Praja-2 OS=Homo sapiens GN=PJA2 PE=1 SV=4 - [PJA2_HUMAN]	4.24	1	2	2	21	5.97	7.87	6.38	8.43	0.40	0.26	1.25	0.88	708	78.2	4.39
Q7Z6E9	E3 ubiquitin-protein ligase RBBP6 OS=Homo sapiens GN=RBBP6 PE=1 SV=1 - [RBBP6_HUMAN]	1.56	1	4	4	5					0.98	0.95	0.78	0.74	1792	201.4	9.64
Q99496	E3 ubiquitin-protein ligase RING2 OS=Homo sapiens GN=RNF2 PE=1 SV=1 - [RING2_HUMAN]	4.46	1	1	1	1	1.01	0.90	1.15	1.03					336	37.6	6.84
Q9NVW2	E3 ubiquitin-protein ligase RLIM OS=Homo sapiens GN=RLIM PE=1 SV=3 - [RNF12_HUMAN]	1.92	1	1	1	2					1.12	0.51	0.84	0.38	624	68.5	7.56
Q5XPI4	E3 ubiquitin-protein ligase RNF123 OS=Homo sapiens GN=RNF123 PE=1 SV=1 - [RN123_HUMAN]	1.45	1	2	2	3	1.08	1.14	1.06	1.12	0.91	0.65	0.98	0.70	1314	148.4	6.74
Q96EQ8	E3 ubiquitin-protein ligase RNF125 OS=Homo sapiens GN=RNF125 PE=1 SV=4 - [RN125_HUMAN]	5.17	1	1	1	1					1.28	1.20	1.37	1.28	232	26.4	7.12
O43567	E3 ubiquitin-protein ligase RNF13 OS=Homo sapiens GN=RNF13 PE=1 SV=1 - [RNF13_HUMAN]	2.10	1	1	1	1					1.44	1.36	1.28	1.21	381	42.8	4.98
Q86XS8	E3 ubiquitin-protein ligase RNF130 OS=Homo sapiens GN=RNF130 PE=1 SV=1 - [GOLI_HUMAN]	3.10	1	1	1	2					0.70	0.57	0.81	0.65	419	46.4	8.87
Q8IUD6	E3 ubiquitin-protein ligase RNF135 OS=Homo sapiens GN=RNF135 PE=1 SV=2 - [RN135_HUMAN]	3.47	1	1	1	1	0.88	0.86	0.85	0.84					432	47.9	6.81
Q8WVD3	E3 ubiquitin-protein ligase RNF138 OS=Homo sapiens GN=RNF138 PE=1 SV=1 - [RN138_HUMAN]	4.08	1	1	1	1	0.22	0.44							245	28.2	6.93
Q9P0P0	E3 ubiquitin-protein ligase RNF181 OS=Homo sapiens GN=RNF181 PE=1 SV=1 - [RN181_HUMAN]	6.54	1	1	1	1					0.84	0.52	0.92	0.57	153	17.9	5.06
Q9NV58	E3 ubiquitin-protein ligase RNF19A OS=Homo sapiens GN=RNF19A PE=1 SV=3 - [RN19A_HUMAN]	1.79	1	1	1	1					0.86	0.82	0.87	0.84	838	90.6	7.01
Q96BH1	E3 ubiquitin-protein ligase RNF25 OS=Homo sapiens GN=RNF25 PE=1 SV=1 - [RNF25_HUMAN]	4.79	1	1	1	4					1.35	1.66	1.19	1.46	459	51.2	6.54
Q969K3	E3 ubiquitin-protein ligase RNF34 OS=Homo sapiens GN=RNF34 PE=1 SV=1 - [RNF34_HUMAN]	7.53	1	1	1	1	1.02	1.03	0.98	1.00					372	41.6	4.96
Q149N8	E3 ubiquitin-protein ligase SHPRH OS=Homo sapiens GN=SHPRH PE=1 SV=2 - [SHPRH_HUMAN]	2.97	1	3	3	5	1.10	1.04	0.99	0.94	0.69	0.89	0.58	0.74	1683	193.0	7.47
Q86TM6	E3 ubiquitin-protein ligase synoviolin OS=Homo sapiens GN=SYVN1 PE=1 SV=2 - [SYVN1_HUMAN]	3.40	1	2	2	2	0.78	0.99	0.77	0.98	0.79	1.08	3.50	4.78	617	67.6	6.95
Q9NS56	E3 ubiquitin-protein ligase Topors OS=Homo sapiens GN=TOPORS PE=1 SV=1 - [TOPRS_HUMAN]	2.97	1	1	1	1	0.90	0.93	0.90	0.94					1045	119.1	9.51
P36406	E3 ubiquitin-protein ligase TRIM23 OS=Homo sapiens GN=TRIM23 PE=1 SV=1 - [TRI23_HUMAN]	5.75	2	2	2	5	0.99	1.12	0.92	1.04					574	64.0	6.38
Q13049	E3 ubiquitin-protein ligase TRIM32 OS=Homo sapiens GN=TRIM32 PE=1 SV=2 - [TRI32_HUMAN]	5.67	1	2	2	2					0.92	0.61	1.34	0.89	653	71.9	6.98

Q9UPN9	E3 ubiquitin-protein ligase TRIM33 OS=Homo sapiens GN=TRIM33 PE=1 SV=3 - [TRI33_HUMAN]	0.80	1	1	1	3	0.92	0.47	0.88	0.45					1127	122.5	6.67
Q9NQ86	E3 ubiquitin-protein ligase TRIM36 OS=Homo sapiens GN=TRIM36 PE=2 SV=2 - [TRI36_HUMAN]	2.20	1	1	1	1	0.99	1.17	0.88	1.03					728	83.0	6.11
Q9C026	E3 ubiquitin-protein ligase TRIM9 OS=Homo sapiens GN=TRIM9 PE=1 SV=1 - [TRIM9_HUMAN]	1.69	1	1	1	1	0.54	0.45	0.65	0.54					710	79.1	6.84
Q8IWV7	E3 ubiquitin-protein ligase UBR1 OS=Homo sapiens GN=UBR1 PE=1 SV=1 - [UBR1_HUMAN]	0.69	1	1	1	1	0.82	0.92	0.94	1.07					1749	200.1	6.01
Q6ZT12	E3 ubiquitin-protein ligase UBR3 OS=Homo sapiens GN=UBR3 PE=2 SV=2 - [UBR3_HUMAN]	0.37	1	1	1	1	0.73	1.08	2.91	4.35					1888	212.3	6.10
Q5T4S7	E3 ubiquitin-protein ligase UBR4 OS=Homo sapiens GN=UBR4 PE=1 SV=1 - [UBR4_HUMAN]	1.93	9	2	5	16	1.04	1.07	0.99	1.04					5183	573.5	6.04
Q8ND25	E3 ubiquitin-protein ligase ZNRF1 OS=Homo sapiens GN=ZNRF1 PE=1 SV=2 - [ZNRF1_HUMAN]	2.64	1	1	1	1	0.71		0.79						227	23.8	6.67
Q15075	Early endosome antigen 1 OS=Homo sapiens GN=EEA1 PE=1 SV=2 - [EEA1_HUMAN]	1.20	1	1	1	1	1.52	1.03	1.85	1.25					1411	162.4	5.68
O95834	Echinoderm microtubule-associated protein-like 2 OS=Homo sapiens GN=EML2 PE=2 SV=1 - [EMAL2_HUMAN]	1.54	1	1	1	1					1.18	0.36	0.74	0.23	649	70.6	6.32
Q9HC35	Echinoderm microtubule-associated protein-like 4 OS=Homo sapiens GN=EML4 PE=1 SV=3 - [EMAL4_HUMAN]	2.65	1	1	1	8					0.38	0.16	0.88	0.38	981	108.8	6.40
Q6ZMW3	Echinoderm microtubule-associated protein-like 6 OS=Homo sapiens GN=EML6 PE=2 SV=2 - [EMAL6_HUMAN]	1.43	2	2	2	2	1.13	1.13	1.04	1.04					1958	217.8	7.44
Q13508	Ecto-ADP-ribosyltransferase 3 OS=Homo sapiens GN=ART3 PE=1 SV=2 - [NAR3_HUMAN]	9.77	3	2	3	7	0.98	1.10	1.01	1.15	0.75	0.75	0.88	0.87	389	43.9	6.06
Q93070	Ecto-ADP-ribosyltransferase 4 OS=Homo sapiens GN=ART4 PE=2 SV=2 - [NAR4_HUMAN]	3.18	1	1	1	5	1.01	1.15	1.06	1.22	1.12	1.04	1.02	0.95	314	35.9	9.22
O14682	Ectoderm-neural cortex protein 1 OS=Homo sapiens GN=ENC1 PE=1 SV=2 - [ENC1_HUMAN]	1.70	1	1	1	1					1.32	1.21	0.88	0.80	589	66.1	6.84
O75356	Ectonucleoside triphosphate diphosphohydrolase 5 OS=Homo sapiens GN=ENTPD5 PE=1 SV=1 - [ENTP5_HUMAN]	3.50	1	1	1	1	1.08	1.23	1.09	1.24					428	47.5	6.33
Q13822	Ectonucleotide pyrophosphatase/phosphodiesterase family member 2 OS=Homo sapiens GN=ENPP2 PE=1 SV=3 - [ENPP2_HUMAN]	15.30	1	9	9	30	1.60	1.98	1.37	1.72	1.24	1.13	1.00	1.12	863	98.9	7.37
Q6UWV6	Ectonucleotide pyrophosphatase/phosphodiesterase family member 7 OS=Homo sapiens GN=ENPP7 PE=1 SV=3 - [ENPP7_HUMAN]	3.49	1	1	1	1	1.04	1.23	1.02	1.21					458	51.5	6.89
Q9HCE0	Ectopic P granules protein 5 homolog OS=Homo sapiens GN=EPG5 PE=2 SV=2 - [EPG5_HUMAN]	0.58	1	1	1	1					0.68	0.92	0.67	0.90	2579	292.3	6.43
Q9HAE3	EF-hand calcium-binding domain-containing protein 1 OS=Homo sapiens GN=EFCAB1 PE=2 SV=1 - [EFCB1_HUMAN]	5.21	1	1	1	1	0.81	0.61	0.76	0.58					211	24.5	5.06
Q8N7B9	EF-hand calcium-binding domain-containing protein 3 OS=Homo sapiens GN=EFCAB3 PE=1 SV=1 - [EFCB3_HUMAN]	4.57	1	1	1	1	0.79	0.83	0.55	0.59					438	50.1	9.22
Q9BSW2	EF-hand calcium-binding domain-containing protein 4B OS=Homo sapiens GN=EFCAB4B PE=1 SV=1 - [EFC4B_HUMAN]	2.03	1	1	1	1					1.21	1.23	1.49	1.52	395	45.6	5.01
Q5JST6	EF-hand domain-containing family member C2 OS=Homo sapiens GN=EFHC2 PE=1 SV=2 - [EFHC2_HUMAN]	1.07	1	1	1	1					0.71	0.77	0.77	0.84	749	87.3	7.37
Q96C19	EF-hand domain-containing protein D2 OS=Homo sapiens GN=EFHD2 PE=1 SV=1 - [EFHD2_HUMAN]	2.92	1	1	1	2					1.36	1.36	1.21	1.22	240	26.7	5.20
Q12805	EGF-containing fibulin-like extracellular matrix protein 1 OS=Homo sapiens GN=EFEMP1 PE=1 SV=2 - [FBLN3_HUMAN]	37.93	1	12	12	102	1.02	1.06	0.97	1.03	0.99	0.91	0.95	0.86	493	54.6	5.07
Q9UHX3	EGF-like module-containing mucin-like hormone receptor-like 2 OS=Homo sapiens GN=EMR2 PE=1 SV=2 - [EMR2_HUMAN]	6.44	1	2	2	3	1.08	1.04	1.15	1.11					823	90.4	6.87
Q9GZT9	Egl nine homolog 1 OS=Homo sapiens GN=EGLN1 PE=1 SV=1 - [EGLN1_HUMAN]	2.82	1	1	1	1					0.91	1.12	0.99	1.21	426	46.0	8.53
Q8NDI1	EH domain-binding protein 1 OS=Homo sapiens GN=EHBP1 PE=1 SV=3 - [EHBP1_HUMAN]	3.01	1	2	2	2	0.67	0.61	0.60	0.55	2.92	2.79	2.82	2.70	1231	139.9	5.35

Q8N3D4	EH domain-binding protein 1-like protein 1 OS=Homo sapiens GN=EHBP1L1 PE=1 SV=2 - [EH1L1_HUMAN]	0.72	1	1	1	1	0.72	0.84	0.89	1.04					1523	161.8	4.83
Q9H4M9	EH domain-containing protein 1 OS=Homo sapiens GN=EHD1 PE=1 SV=2 - [EHD1_HUMAN]	9.74	2	1	3	4	0.87	0.53	1.09	0.66					534	60.6	6.83
Q9NZN4	EH domain-containing protein 2 OS=Homo sapiens GN=EHD2 PE=1 SV=2 - [EHD2_HUMAN]	2.76	1	1	1	1					0.71	0.68	0.61	0.59	543	61.1	6.46
Q9NZN3	EH domain-containing protein 3 OS=Homo sapiens GN=EHD3 PE=1 SV=2 - [EHD3_HUMAN]	20.93	2	6	8	26	0.50	0.49	0.55	0.54	1.58	1.56	1.51	1.38	535	60.8	6.57
P19957	Elafin OS=Homo sapiens GN=PI3 PE=1 SV=3 - [ELAF_HUMAN]	7.69	1	1	1	2					1.12	1.04	1.12	1.04	117	12.3	8.82
Q8IUD2	ELKS/Rab6-interacting/CAST family member 1 OS=Homo sapiens GN=ERC1 PE=1 SV=1 - [RB6I2_HUMAN]	1.61	1	1	1	1					1.63	1.19	0.94	0.69	1116	128.0	5.97
P57679	Ellis-van Creveld syndrome protein OS=Homo sapiens GN=EVC PE=1 SV=1 - [EVC_HUMAN]	2.52	1	2	2	2	1.11	1.48	1.18	1.59					992	111.9	6.73
P68104	Elongation factor 1-alpha 1 OS=Homo sapiens GN=EEF1A1 PE=1 SV=1 - [EF1A1_HUMAN]	4.11	3	2	2	6	0.87	1.05	0.83	0.99	1.04	0.97	1.05	0.98	462	50.1	9.01
P29692	Elongation factor 1-delta OS=Homo sapiens GN=EEF1D PE=1 SV=5 - [EF1D_HUMAN]	14.23	2	4	4	8					1.17	1.46	1.31	1.54	281	31.1	5.01
P26641	Elongation factor 1-gamma OS=Homo sapiens GN=EEF1G PE=1 SV=3 - [EF1G_HUMAN]	6.41	1	3	3	6					1.47	1.23	1.41	1.18	437	50.1	6.67
P13639	Elongation factor 2 OS=Homo sapiens GN=EEF2 PE=1 SV=4 - [EF2_HUMAN]	2.68	1	2	2	3	0.97	0.97	1.07	1.08	0.63	0.70	0.57	0.64	858	95.3	6.83
Q7Z2Z2	Elongation factor Tu GTP-binding domain-containing protein 1 OS=Homo sapiens GN=EFTUD1 PE=1 SV=2 - [ETUD1_HUMAN]	1.88	1	1	1	2		1.08		1.18	1.12	0.95	1.12	0.95	1120	125.3	5.91
P49411	Elongation factor Tu, mitochondrial OS=Homo sapiens GN=TUFM PE=1 SV=2 - [EFTU_HUMAN]	7.52	1	1	1	1	0.58	0.38	0.66	0.44					452	49.5	7.61
Q96EB1	Elongator complex protein 4 OS=Homo sapiens GN=ELP4 PE=1 SV=2 - [ELP4_HUMAN]	5.42	1	1	1	1					1.25	1.23	1.21	1.19	424	46.6	8.51
Q6PCB8	Embigin OS=Homo sapiens GN=EMB PE=1 SV=1 - [EMB_HUMAN]	2.75	1	1	1	1					0.97	0.08	1.12	0.10	327	36.9	6.54
Q9Y6C2	EMILIN-1 OS=Homo sapiens GN=EMILIN1 PE=1 SV=2 - [EMIL1_HUMAN]	0.89	1	1	1	3	0.77	0.75	0.73	0.72					1016	106.6	5.15
Q9UI08	Ena/VASP-like protein OS=Homo sapiens GN=EVL PE=1 SV=2 - [EVL_HUMAN]	5.53	1	2	2	7					0.69	0.48	0.83	0.48	416	44.6	8.84
Q9NRM1	Enamelin OS=Homo sapiens GN=ENAM PE=2 SV=3 - [ENAM_HUMAN]	1.84	1	1	1	6	1.12	1.12	1.16	1.16					1142	128.7	6.83
P17813	Endoglin OS=Homo sapiens GN=ENG PE=1 SV=2 - [EGLN_HUMAN]	10.18	1	5	5	12	1.42	1.41	1.40	1.36	0.79	0.83	0.73	0.76	658	70.5	6.61
O94919	Endonuclease domain-containing 1 protein OS=Homo sapiens GN=ENDOD1 PE=1 SV=2 - [ENDD1_HUMAN]	4.80	1	2	2	5	0.87	0.87	0.99	1.01	0.97	0.93	0.99	0.96	500	55.0	5.71
Q7L9B9	Endonuclease/exonuclease/phosphatase family domain-containing protein 1 OS=Homo sapiens GN=EEPD1 PE=1 SV=2 - [EEPD1_HUMAN]	3.87	1	2	2	2					1.54	1.38	1.44	1.30	569	62.4	8.41
Q9NR46	Endophilin-B2 OS=Homo sapiens GN=SH3GLB2 PE=1 SV=1 - [SHLB2_HUMAN]	3.80	1	1	1	2	0.64	0.62	0.57	0.57	1.24	1.31	1.26	1.33	395	43.9	5.99
Q9NZ08	Endoplasmic reticulum aminopeptidase 1 OS=Homo sapiens GN=ERAP1 PE=1 SV=3 - [ERAP1_HUMAN]	1.38	1	1	1	2	0.66	0.69	0.60	0.63					941	107.2	6.46
Q6P179	Endoplasmic reticulum aminopeptidase 2 OS=Homo sapiens GN=ERAP2 PE=1 SV=2 - [ERAP2_HUMAN]	7.40	1	6	6	11	0.95	0.94	0.97	0.91	1.63	1.45	1.46	1.31	960	110.4	6.71
Q9UKM7	Endoplasmic reticulum mannosyl-oligosaccharide 1,2-alpha-mannosidase OS=Homo sapiens GN=MAN1B1 PE=1 SV=2 - [MA1B1_HUMAN]	3.58	1	1	1	1	1.29		1.35						699	79.5	7.72
Q7Z2K6	Endoplasmic reticulum metallopeptidase 1 OS=Homo sapiens GN=ERMP1 PE=1 SV=2 - [ERMP1_HUMAN]	1.11	1	1	1	1	1.03	1.11	1.07	1.15					904	100.2	7.52
P30040	Endoplasmic reticulum resident protein 29 OS=Homo sapiens GN=ERP29 PE=1 SV=4 - [ERP29_HUMAN]	6.51	1	2	2	3	1.04	1.12	0.90	0.96	0.88	1.06	0.89	1.06	261	29.0	7.31

Q9BS26	Endoplasmic reticulum resident protein 44 OS=Homo sapiens GN=ERP44 PE=1 SV=1 - [ERP44_HUMAN]	15.27	1	5	5	11	0.75	0.76	0.83	0.84	1.00	0.97	0.98	0.95	406	46.9	5.26
P14625	Endoplasmin OS=Homo sapiens GN=HSP90B1 PE=1 SV=1 - [ENPL_HUMAN]	13.08	1	7	10	18	0.92	0.90	0.96	0.95	1.09	1.02	1.01	0.79	803	92.4	4.84
Q9UPY3	Endoribonuclease Dicer OS=Homo sapiens GN=DICER1 PE=1 SV=3 - [DICER_HUMAN]	3.43	1	3	3	18	1.65	1.69	2.02	2.08					1922	218.5	5.68
Q9HCU0	Endosialin OS=Homo sapiens GN=CD248 PE=1 SV=1 - [CD248_HUMAN]	1.32	1	1	1	2	1.10	0.90	1.00	0.82					757	80.8	5.35
Q96AP7	Endothelial cell-selective adhesion molecule OS=Homo sapiens GN=ESAM PE=1 SV=1 - [ESAM_HUMAN]	2.82	1	1	1	2					1.04	1.05	1.02	1.02	390	41.2	9.32
Q99814	Endothelial PAS domain-containing protein 1 OS=Homo sapiens GN=EPAS1 PE=1 SV=3 - [EPAS1_HUMAN]	1.95	1	2	2	5	0.54	0.64	0.61	0.75					870	96.4	6.28
O60344	Endothelin-converting enzyme 2 OS=Homo sapiens GN=ECE2 PE=1 SV=4 - [ECE2_HUMAN]	4.98	1	2	2	12	1.72	1.06	1.86	1.16					883	99.7	5.10
Q96JJ3	Engulfment and cell motility protein 2 OS=Homo sapiens GN=ELMO2 PE=1 SV=2 - [ELMO2_HUMAN]	0.83	1	1	1	1	1.16	0.85	1.17	0.86					720	82.6	5.90
Q96BJ8	Engulfment and cell motility protein 3 OS=Homo sapiens GN=ELMO3 PE=1 SV=3 - [ELMO3_HUMAN]	2.36	1	1	1	1	1.10	1.21	1.15	1.27					720	81.4	6.30
Q14511	Enhancer of filamentation 1 OS=Homo sapiens GN=NEDD9 PE=1 SV=1 - [CASL_HUMAN]	0.96	1	1	1	4					0.90	0.97	1.45	1.56	834	92.8	6.70
Q6P2E9	Enhancer of mRNA-decapping protein 4 OS=Homo sapiens GN=EDC4 PE=1 SV=1 - [EDC4_HUMAN]	2.43	1	3	3	4	0.50	0.57	0.49	0.55	0.47	0.93	0.46	0.91	1401	151.6	5.86
Q9H2F5	Enhancer of polycomb homolog 1 OS=Homo sapiens GN=EPC1 PE=1 SV=1 - [EPC1_HUMAN]	3.47	1	1	1	2	1.40	1.06	1.82	1.38					836	93.4	8.59
P30084	Enoyl-CoA hydratase, mitochondrial OS=Homo sapiens GN=ECHS1 PE=1 SV=4 - [ECHM_HUMAN]	7.24	1	1	1	1					1.17	1.01	1.22	1.05	290	31.4	8.07
Q8IYW4	ENTH domain-containing protein 1 OS=Homo sapiens GN=ENTHD1 PE=2 SV=1 - [ENTD1_HUMAN]	1.98	1	1	1	3	1.25	1.11	1.36	1.22					607	67.5	6.62
Q92817	Envoplakin OS=Homo sapiens GN=EVPL PE=1 SV=3 - [EVPL_HUMAN]	0.44	1	1	1	1					0.77	0.85	0.92	1.02	2033	231.5	6.96
P12724	Eosinophil cationic protein OS=Homo sapiens GN=RNASE3 PE=1 SV=2 - [ECP_HUMAN]	17.50	1	2	2	8					1.59	1.49	1.54	1.47	160	18.4	10.02
Q05315	Eosinophil lysophospholipase OS=Homo sapiens GN=CLC PE=1 SV=3 - [LPPL_HUMAN]	13.38	1	2	3	3					1.30	1.50	1.23	1.41	142	16.4	7.37
P11678	Eosinophil peroxidase OS=Homo sapiens GN=EPX PE=1 SV=2 - [PERE_HUMAN]	7.13	1	5	6	10	1.68	1.84	1.64	1.76					715	81.0	10.29
Q8N140	EP300-interacting inhibitor of differentiation 3 OS=Homo sapiens GN=EID3 PE=1 SV=1 - [EID3_HUMAN]	15.32	1	2	2	3	1.27								333	38.1	5.15
Q6ZTU2	EP400 N-terminal-like protein OS=Homo sapiens GN=EP400NL PE=2 SV=2 - [E400N_HUMAN]	3.28	1	1	1	1	1.50	1.33	0.80	0.71					488	51.7	7.27
P21709	Ephrin type-A receptor 1 OS=Homo sapiens GN=EPHA1 PE=1 SV=4 - [EPHA1_HUMAN]	1.84	1	2	2	4	0.71	0.90	0.71	0.91	4.19	4.92	4.09	4.80	976	108.1	6.64
P29317	Ephrin type-A receptor 2 OS=Homo sapiens GN=EPHA2 PE=1 SV=2 - [EPHA2_HUMAN]	4.10	1	2	2	3	1.00	0.99	1.04	1.05	1.65	1.83	1.34	1.49	976	108.2	6.23
P29320	Ephrin type-A receptor 3 OS=Homo sapiens GN=EPHA3 PE=1 SV=2 - [EPHA3_HUMAN]	1.53	1	1	1	2					0.85	0.76	1.37	0.65	983	110.1	6.76
P54764	Ephrin type-A receptor 4 OS=Homo sapiens GN=EPHA4 PE=1 SV=1 - [EPHA4_HUMAN]	4.67	1	2	2	2					0.44	0.20	0.94	0.42	986	109.8	6.61
Q15375	Ephrin type-A receptor 7 OS=Homo sapiens GN=EPHA7 PE=1 SV=3 - [EPHA7_HUMAN]	1.30	1	1	1	5					0.59	0.40	0.64	0.50	998	112.0	5.80
P54760	Ephrin type-B receptor 4 OS=Homo sapiens GN=EPHB4 PE=1 SV=2 - [EPHB4_HUMAN]	2.13	1	1	1	1	0.66	0.98	0.42	0.63					987	108.2	6.90
O15197	Ephrin type-B receptor 6 OS=Homo sapiens GN=EPHB6 PE=1 SV=4 - [EPHB6_HUMAN]	0.69	1	1	1	1					0.17	0.16	0.15	0.14	1021	110.6	6.65

P20827	Ephrin-A1 OS=Homo sapiens GN=EFNA1 PE=1 SV=2 - [EFNA1_HUMAN]	8.29	1	2	2	5					1.13	1.15	1.13	1.15	205	23.8	6.83
P00533	Epidermal growth factor receptor OS=Homo sapiens GN=EGFR PE=1 SV=2 - [EGFR_HUMAN]	6.94	1	6	6	10	1.02	1.00	0.98	0.96					1210	134.2	6.68
P42566	Epidermal growth factor receptor substrate 15 OS=Homo sapiens GN=EPS15 PE=1 SV=2 - [EPS15_HUMAN]	8.48	1	4	4	8	0.77	1.09	0.87	1.23	0.55	0.70	0.71	0.91	896	98.6	4.64
Q9UBC2	Epidermal growth factor receptor substrate 15-like 1 OS=Homo sapiens GN=EPS15L1 PE=1 SV=1 - [EP15R_HUMAN]	6.71	1	4	4	4	1.85	1.56	1.18	1.00	2.46	2.23	2.13	1.93	864	94.2	5.11
Q8N3Y7	Epidermal retinol dehydrogenase 2 OS=Homo sapiens GN=SDR16C5 PE=2 SV=2 - [RDHE2_HUMAN]	3.88	1	1	1	1	1.10	1.38	1.06	1.34					309	34.1	8.46
P61916	Epididymal secretory protein E1 OS=Homo sapiens GN=NPC2 PE=1 SV=1 - [NPC2_HUMAN]	38.41	1	4	4	9					1.33	1.38	1.20	1.25	151	16.6	7.65
Q99645	Epiphycan OS=Homo sapiens GN=EPYC PE=2 SV=3 - [EPYC_HUMAN]	6.52	1	1	1	6	1.18	1.24	1.12	1.18					322	36.6	4.81
P34913	Epoxide hydrolase 2 OS=Homo sapiens GN=EPHX2 PE=1 SV=2 - [HYES_HUMAN]	2.16	1	1	1	1	1.13	1.16	1.15	1.19					555	62.6	6.28
Q9H201	Epsin-3 OS=Homo sapiens GN=EPN3 PE=1 SV=1 - [EPN3_HUMAN]	1.58	1	1	1	1					0.74	0.74	0.69	0.69	632	68.2	5.91
Q7RTT9	Equilibrative nucleoside transporter 4 OS=Homo sapiens GN=SLC29A4 PE=1 SV=1 - [S29A4_HUMAN]	3.77	1	1	1	4	0.75	0.93	0.68	0.85					530	58.0	7.72
Q9BZQ6	ER degradation-enhancing alpha-mannosidase-like 3 OS=Homo sapiens GN=EDEM3 PE=1 SV=2 - [EDEM3_HUMAN]	4.94	1	2	2	12	2.28	3.42	0.39	0.60	0.83	0.47	0.75	0.42	932	104.6	4.93
O15083	ERC protein 2 OS=Homo sapiens GN=ERC2 PE=1 SV=3 - [ERC2_HUMAN]	2.19	1	1	1	10	0.91	0.88	0.92	0.90					957	110.5	6.99
A8K979	ERI1 exoribonuclease 2 OS=Homo sapiens GN=ERI2 PE=2 SV=2 - [ERI2_HUMAN]	4.78	1	1	1	1	1.14	1.16	0.96	0.97					691	77.4	8.90
Q86YB8	ERO1-like protein beta OS=Homo sapiens GN=ERO1LB PE=1 SV=2 - [ERO1B_HUMAN]	4.50	1	1	1	1	0.78	0.74	0.65	0.62					467	53.5	7.99
P27105	Erythrocyte band 7 integral membrane protein OS=Homo sapiens GN=STOM PE=1 SV=3 - [STOM_HUMAN]	15.28	2	5	5	7	0.75	0.82	0.66	0.72					288	31.7	7.88
P15976	Erythroid transcription factor OS=Homo sapiens GN=GATA1 PE=1 SV=1 - [GATA1_HUMAN]	2.42	1	1	1	1	1.78	1.25	1.24	0.88					413	42.7	8.75
P03372	Estrogen receptor OS=Homo sapiens GN=ESR1 PE=1 SV=2 - [ESR1_HUMAN]	1.85	1	1	1	1					1.05	1.15	1.15	1.26	595	66.2	8.06
P62508	Estrogen-related receptor gamma OS=Homo sapiens GN=ESRRG PE=1 SV=1 - [ERR3_HUMAN]	3.06	1	1	1	1	0.82	0.85	0.80	0.84					458	51.3	6.48
O00321	ETS translocation variant 2 OS=Homo sapiens GN=ETV2 PE=2 SV=1 - [ETV2_HUMAN]	6.74	1	1	1	1					1.15		1.69		341	36.5	6.54
P41162	ETS translocation variant 3 OS=Homo sapiens GN=ETV3 PE=1 SV=2 - [ETV3_HUMAN]	2.34	1	1	1	1	1.33	1.16	1.44	1.26					512	57.0	8.57
Q15723	ETS-related transcription factor Elf-2 OS=Homo sapiens GN=ELF2 PE=1 SV=2 - [ELF2_HUMAN]	2.53	1	1	1	1	1.09	1.03	1.13	1.07					593	63.9	6.57
P60842	Eukaryotic initiation factor 4A-I OS=Homo sapiens GN=EIF4A1 PE=1 SV=1 - [IF4A1_HUMAN]	8.13	2	2	2	3	2.48	2.64	2.59	2.79	0.80	0.65	0.86	0.70	406	46.1	5.48
P38919	Eukaryotic initiation factor 4A-III OS=Homo sapiens GN=EIF4A3 PE=1 SV=4 - [IF4A3_HUMAN]	4.62	1	1	1	2	1.68	1.65	1.10	1.08					411	46.8	6.73
P15170	Eukaryotic peptide chain release factor GTP-binding subunit ERF3A OS=Homo sapiens GN=GSPT1 PE=1 SV=1 - [ERF3A_HUMAN]	6.81	1	2	2	4					2.27	2.27	2.27	2.26	499	55.7	5.62
Q8IYD1	Eukaryotic peptide chain release factor GTP-binding subunit ERF3B OS=Homo sapiens GN=GSPT2 PE=1 SV=2 - [ERF3B_HUMAN]	3.66	1	1	1	1					0.91	0.80	0.77	0.68	628	68.8	5.43
P05198	Eukaryotic translation initiation factor 2 subunit 1 OS=Homo sapiens GN=EIF2S1 PE=1 SV=3 - [IF2A_HUMAN]	4.13	1	1	1	1					0.98	1.14	0.91	1.07	315	36.1	5.08
P41091	Eukaryotic translation initiation factor 2 subunit 3 OS=Homo sapiens GN=EIF2S3 PE=1 SV=3 - [IF2G_HUMAN]	3.39	2	1	1	1					1.14	1.09	1.10	1.06	472	51.1	8.40

Q99613	Eukaryotic translation initiation factor 3 subunit C OS=Homo sapiens GN=EIF3C PE=1 SV=1 - [EIF3C_HUMAN]	1.75	1	1	1	2					1.23	1.16	0.94	0.88	913	105.3	5.68
O15371	Eukaryotic translation initiation factor 3 subunit D OS=Homo sapiens GN=EIF3D PE=1 SV=1 - [EIF3D_HUMAN]	6.02	1	2	2	2	1.17	1.23	1.21	1.28	1.00		1.18		548	63.9	6.05
Q9Y262	Eukaryotic translation initiation factor 3 subunit L OS=Homo sapiens GN=EIF3L PE=1 SV=1 - [EIF3L_HUMAN]	0.89	1	1	1	1					0.46	0.73	0.36	0.58	564	66.7	6.34
Q04637	Eukaryotic translation initiation factor 4 gamma 1 OS=Homo sapiens GN=EIF4G1 PE=1 SV=4 - [IF4G1_HUMAN]	0.63	1	1	1	2					1.86	1.84	1.91	1.88	1599	175.4	5.33
O43432	Eukaryotic translation initiation factor 4 gamma 3 OS=Homo sapiens GN=EIF4G3 PE=1 SV=2 - [IF4G3_HUMAN]	0.88	1	1	1	2					0.96	1.12	0.92	1.07	1585	176.5	5.38
P23588	Eukaryotic translation initiation factor 4B OS=Homo sapiens GN=EIF4B PE=1 SV=2 - [IF4B_HUMAN]	1.31	1	1	1	3					1.34	1.30	1.29	1.25	611	69.1	5.73
P06730	Eukaryotic translation initiation factor 4E OS=Homo sapiens GN=EIF4E PE=1 SV=2 - [IF4E_HUMAN]	5.07	1	2	2	3					0.50	0.53	0.46	0.49	217	25.1	6.15
Q13542	Eukaryotic translation initiation factor 4E-binding protein 2 OS=Homo sapiens GN=EIF4EBP2 PE=1 SV=1 - [4EBP2_HUMAN]	36.67	1	2	2	2	1.39	1.76	0.88	1.11					120	12.9	6.67
P55010	Eukaryotic translation initiation factor 5 OS=Homo sapiens GN=EIF5 PE=1 SV=2 - [IF5_HUMAN]	2.32	1	1	1	1	1.47	1.47	1.38	1.40					431	49.2	5.58
P63241	Eukaryotic translation initiation factor 5A-1 OS=Homo sapiens GN=EIF5A PE=1 SV=2 - [IF5A1_HUMAN]	12.99	3	2	2	7	0.75	0.77	0.84	0.86	1.57	1.35	1.40	1.18	154	16.8	5.24
Q96CN4	EVI5-like protein OS=Homo sapiens GN=EVI5L PE=1 SV=1 - [EVI5L_HUMAN]	1.39	1	1	1	2	0.98	0.71	1.26	0.91	1.01	0.75	0.98	0.72	794	91.3	5.34
Q9NV70	Exocyst complex component 1 OS=Homo sapiens GN=EXOC1 PE=1 SV=4 - [EXOC1_HUMAN]	2.46	1	1	1	1					1.05	1.07	0.94	0.95	894	101.9	6.61
Q96KP1	Exocyst complex component 2 OS=Homo sapiens GN=EXOC2 PE=1 SV=1 - [EXOC2_HUMAN]	2.06	1	1	1	1					0.78	0.72	1.40	1.29	924	104.0	6.90
Q17RC7	Exocyst complex component 3-like protein 4 OS=Homo sapiens GN=EXOC3L4 PE=2 SV=2 - [EX3L4_HUMAN]	4.02	1	2	2	2	0.78	0.50	1.28	0.82	0.60	1.04	0.66	1.14	722	79.8	6.32
Q8TAG9	Exocyst complex component 6 OS=Homo sapiens GN=EXOC6 PE=1 SV=3 - [EXOC6_HUMAN]	2.24	1	1	1	1	1.64	1.88	1.31	1.50					804	93.7	6.20
Q9Y2D4	Exocyst complex component 6B OS=Homo sapiens GN=EXOC6B PE=1 SV=3 - [EXC6B_HUMAN]	1.60	1	1	1	7	1.10	0.97	1.03	0.92	0.93	1.03	0.89	0.99	811	94.1	6.46
Q9Y2L1	Exosome complex exonuclease RRP44 OS=Homo sapiens GN=DIS3 PE=1 SV=2 - [RRP44_HUMAN]	5.11	1	3	3	4					1.02	1.05	1.01	1.05	958	108.9	7.14
Q93063	Exostosin-2 OS=Homo sapiens GN=EXT2 PE=1 SV=1 - [EXT2_HUMAN]	1.39	1	1	1	1	1.12	1.10	0.99	0.98					718	82.2	6.55
Q9UBQ6	Exostosin-like 2 OS=Homo sapiens GN=EXTL2 PE=1 SV=1 - [EXTL2_HUMAN]	3.33	1	1	1	1	1.01	1.01	1.05	1.06					330	37.4	8.95
O43909	Exostosin-like 3 OS=Homo sapiens GN=EXTL3 PE=2 SV=1 - [EXTL3_HUMAN]	2.18	1	1	2	2	0.97	1.01	0.81	0.84					919	104.7	6.51
P55060	Exportin-2 OS=Homo sapiens GN=CSE1L PE=1 SV=3 - [XPO2_HUMAN]	1.75	1	1	1	1	0.63	0.74	0.70	0.82					971	110.3	5.77
Q9C0E2	Exportin-4 OS=Homo sapiens GN=XPO4 PE=1 SV=2 - [XPO4_HUMAN]	1.82	1	1	2	2	0.87	1.05	0.72	0.87					1151	130.1	5.05
Q96QU8	Exportin-6 OS=Homo sapiens GN=XPO6 PE=1 SV=1 - [XPO6_HUMAN]	0.71	1	1	1	1					1.95	2.27	1.81	2.10	1125	128.8	6.35
O43592	Exportin-T OS=Homo sapiens GN=XPOT PE=1 SV=2 - [XPOT_HUMAN]	2.81	1	2	2	2					0.85	0.84	0.81	0.80	962	109.9	5.39
Q9BSJ8	Extended synaptotagmin-1 OS=Homo sapiens GN=ESYT1 PE=1 SV=1 - [ESYT1_HUMAN]	2.63	1	2	2	5	0.72	0.73	0.65	0.67	1.60	1.71	1.49	1.59	1104	122.8	5.83
A0FGR9	Extended synaptotagmin-3 OS=Homo sapiens GN=ESYT3 PE=2 SV=1 - [ESYT3_HUMAN]	1.24	1	1	1	1	1.19	0.99	1.22	1.02					886	100.0	8.37
Q16610	Extracellular matrix protein 1 OS=Homo sapiens GN=ECM1 PE=1 SV=2 - [ECM1_HUMAN]	44.63	1	19	19	205	1.05	1.03	1.02	1.02	0.78	0.92	0.82	0.96	540	60.6	6.71

Q86XX4	Extracellular matrix protein FRAS1 OS=Homo sapiens GN=FRAS1 PE=2 SV=1 - [FRAS1_HUMAN]	0.87	4	1	2	3					1.73	1.42	1.28	1.04	4007	442.6	5.59
Q8IWU6	Extracellular sulfatase Sulf-1 OS=Homo sapiens GN=SULF1 PE=1 SV=1 - [SULF1_HUMAN]	2.18	1	1	1	2					0.96	1.00	1.09	1.13	871	101.0	9.09
Q8IWU5	Extracellular sulfatase Sulf-2 OS=Homo sapiens GN=SULF2 PE=1 SV=1 - [SULF2_HUMAN]	1.49	1	1	1	2					1.08	1.16	1.09	1.17	870	100.4	9.17
P08294	Extracellular superoxide dismutase [Cu-Zn] OS=Homo sapiens GN=SOD3 PE=1 SV=2 - [SODE_HUMAN]	20.00	1	4	4	26	0.62	0.65	0.58	0.62	0.75	0.81	0.78	0.85	240	25.8	6.61
P52907	F-actin-capping protein subunit alpha-1 OS=Homo sapiens GN=CAPZA1 PE=1 SV=3 - [CAZA1_HUMAN]	9.09	2	2	2	4	0.64	0.57	0.61	0.54					286	32.9	5.69
P47756	F-actin-capping protein subunit beta OS=Homo sapiens GN=CAPZB PE=1 SV=4 - [CAPZB_HUMAN]	5.05	1	1	1	2	0.69	0.78	0.71	0.80					277	31.3	5.59
Q86XK2	F-box only protein 11 OS=Homo sapiens GN=FBXO11 PE=1 SV=3 - [FBX11_HUMAN]	1.62	1	1	1	1					1.31	1.61	1.14	1.40	927	103.5	6.99
Q96EF6	F-box only protein 17 OS=Homo sapiens GN=FBXO17 PE=1 SV=1 - [FBX17_HUMAN]	8.27	1	1	1	1							0.69	0.83	278	31.5	8.10
Q8TCJ0	F-box only protein 25 OS=Homo sapiens GN=FBXO25 PE=1 SV=3 - [FBX25_HUMAN]	4.09	1	1	1	2	1.01	1.00	0.97	0.96					367	43.3	8.09
Q9NVF7	F-box only protein 28 OS=Homo sapiens GN=FBXO28 PE=1 SV=1 - [FBX28_HUMAN]	5.98	1	1	1	1	0.94	1.03	0.91	1.02					368	41.1	9.55
Q9UK99	F-box only protein 3 OS=Homo sapiens GN=FBXO3 PE=1 SV=3 - [FBX3_HUMAN]	3.61	1	1	1	1	0.12	0.13	0.19	0.20					471	54.5	5.00
Q7Z6M2	F-box only protein 33 OS=Homo sapiens GN=FBXO33 PE=2 SV=1 - [FBX33_HUMAN]	2.34	1	1	1	1	0.54	0.58	1.17	1.28					555	62.6	7.36
Q9NWN3	F-box only protein 34 OS=Homo sapiens GN=FBXO34 PE=1 SV=2 - [FBX34_HUMAN]	0.84	1	1	1	1	1.92	2.21	1.43	1.64					711	78.7	8.00
Q8NEA4	F-box only protein 36 OS=Homo sapiens GN=FBXO36 PE=2 SV=2 - [FBX36_HUMAN]	12.23	1	1	1	1	0.84	0.76	0.82	0.75					188	22.1	8.43
Q9UH90	F-box only protein 40 OS=Homo sapiens GN=FBXO40 PE=1 SV=2 - [FBX40_HUMAN]	2.68	1	1	1	1	1.14	1.01	0.94	0.84					709	79.7	7.11
Q8TF61	F-box only protein 41 OS=Homo sapiens GN=FBXO41 PE=2 SV=5 - [FBX41_HUMAN]	1.60	1	1	1	1					0.13	0.15	0.14	0.16	875	94.4	8.24
Q4G163	F-box only protein 43 OS=Homo sapiens GN=FBXO43 PE=1 SV=3 - [FBX43_HUMAN]	2.54	1	1	1	1	1.19	1.05	1.38	1.23					708	78.4	8.07
Q5MNV8	F-box only protein 47 OS=Homo sapiens GN=FBXO47 PE=2 SV=2 - [FBX47_HUMAN]	4.65	1	1	2	6	0.85	0.83	0.81	0.79	0.86	0.88	0.91	0.93	452	51.9	8.35
Q9NRD1	F-box only protein 6 OS=Homo sapiens GN=FBXO6 PE=1 SV=1 - [FBX6_HUMAN]	2.05	1	1	1	2					0.71	0.44	0.97	0.60	293	33.9	6.09
Q8NEE6	F-box/LRR-repeat protein 13 OS=Homo sapiens GN=FBXL13 PE=2 SV=3 - [FXL13_HUMAN]	1.50	1	1	1	1	2.00	1.27	1.27	0.81					735	83.9	8.72
Q96ME1	F-box/LRR-repeat protein 18 OS=Homo sapiens GN=FBXL18 PE=2 SV=2 - [FXL18_HUMAN]	5.71	1	2	2	2					1.18	1.37	1.15	1.34	805	88.3	8.32
Q6P050	F-box/LRR-repeat protein 22 OS=Homo sapiens GN=FBXL22 PE=2 SV=1 - [FXL22_HUMAN]	4.56	1	1	1	1	0.97	0.99	1.17	1.19					241	26.5	9.91
Q9UKT7	F-box/LRR-repeat protein 3 OS=Homo sapiens GN=FBXL3 PE=1 SV=1 - [FBXL3_HUMAN]	2.80	1	1	1	1					1.37	1.25	1.33	1.21	428	48.7	6.89
Q9Y297	F-box/WD repeat-containing protein 1A OS=Homo sapiens GN=BTRC PE=1 SV=1 - [FBW1A_HUMAN]	4.63	1	1	1	2					1.38	1.71	1.13	1.41	605	68.8	7.96
Q969H0	F-box/WD repeat-containing protein 7 OS=Homo sapiens GN=FBXW7 PE=1 SV=1 - [FBXW7_HUMAN]	1.13	1	1	1	2					1.66	1.78	1.64	1.75	707	79.6	5.80
Q5TGI0	Failed axon connections homolog OS=Homo sapiens GN=FAXC PE=2 SV=2 - [FAXC_HUMAN]	3.91	1	1	1	2					0.88	0.96	1.03	1.13	409	46.8	5.57
O15360	Fanconi anemia group A protein OS=Homo sapiens GN=FANCA PE=1 SV=2 - [FANCA_HUMAN]	1.79	1	2	2	9	1.23	1.59	1.26	1.63	1.72	1.51	1.69	1.48	1455	162.7	6.60

Q9HB96	Fanconi anemia group E protein OS=Homo sapiens GN=FANCE PE=1 SV=1 - [FANCE_HUMAN]	1.49	1	1	1	1	0.75	0.93	0.81	1.01					536	58.7	5.24
Q9NPI8	Fanconi anemia group F protein OS=Homo sapiens GN=FANCF PE=1 SV=1 - [FANCF_HUMAN]	3.48	1	1	1	1					0.09	0.04	0.68	0.35	374	42.2	8.94
Q9BX63	Fanconi anemia group J protein OS=Homo sapiens GN=BRIP1 PE=1 SV=1 - [FANCJ_HUMAN]	5.12	1	4	4	7	0.71	0.59	0.69	0.56	1.42	1.30	1.41	1.29	1249	140.8	6.92
Q8IYD8	Fanconi anemia group M protein OS=Homo sapiens GN=FANCM PE=1 SV=2 - [FANCM_HUMAN]	1.71	1	3	3	3	1.10	4.30	0.63	2.51	1.02	0.79	1.04	0.80	2048	232.0	6.11
Q9UNN5	FAS-associated factor 1 OS=Homo sapiens GN=FAF1 PE=1 SV=2 - [FAF1_HUMAN]	3.54	1	2	2	4					1.07	1.07	1.10	1.10	650	73.9	4.88
Q8TES7	Fas-binding factor 1 OS=Homo sapiens GN=FBF1 PE=1 SV=2 - [FBF1_HUMAN]	2.29	1	1	1	1					0.99	0.77	1.01	0.78	1133	125.4	7.08
Q53R41	FAST kinase domain-containing protein 1 OS=Homo sapiens GN=FASTKD1 PE=1 SV=1 - [FAKD1_HUMAN]	4.49	1	2	2	2	0.74	0.54	1.02	0.75					847	97.3	7.74
Q14CZ7	FAST kinase domain-containing protein 3 OS=Homo sapiens GN=FASTKD3 PE=2 SV=2 - [FAKD3_HUMAN]	2.27	1	1	1	1					0.82	0.94	1.00	1.15	662	75.6	8.29
P49327	Fatty acid synthase OS=Homo sapiens GN=FASN PE=1 SV=3 - [FAS_HUMAN]	1.35	1	2	2	2	1.03	1.05	0.62	0.63	0.52	0.52	0.59	0.58	2511	273.3	6.44
Q01469	Fatty acid-binding protein, epidermal OS=Homo sapiens GN=FABP5 PE=1 SV=3 - [FABP5_HUMAN]	30.37	2	3	3	8					0.93	0.63	1.00	0.92	135	15.2	7.01
P05413	Fatty acid-binding protein, heart OS=Homo sapiens GN=FABP3 PE=1 SV=4 - [FABPH_HUMAN]	6.77	1	1	1	3					1.10	1.11	1.16	1.18	133	14.8	6.80
P12104	Fatty acid-binding protein, intestinal OS=Homo sapiens GN=FABP2 PE=1 SV=2 - [FABPI_HUMAN]	17.42	1	1	1	2	0.70	0.83	0.94	1.13					132	15.2	7.24
P07148	Fatty acid-binding protein, liver OS=Homo sapiens GN=FABP1 PE=1 SV=1 - [FABPL_HUMAN]	7.87	1	1	1	4					1.13	1.06	0.99	0.92	127	14.2	7.18
Q96K12	Fatty acyl-CoA reductase 2 OS=Homo sapiens GN=FAR2 PE=2 SV=1 - [FACR2_HUMAN]	1.55	1	1	1	2	1.39	1.34	1.28	1.24					515	59.4	9.38
Q6GMR7	Fatty-acid amide hydrolase 2 OS=Homo sapiens GN=FAAH2 PE=2 SV=1 - [FAAH2_HUMAN]	3.76	1	1	1	1					1.89	1.85	3.46	3.40	532	58.3	9.07
Q96PJ5	Fc receptor-like protein 4 OS=Homo sapiens GN=FCRL4 PE=1 SV=1 - [FCRL4_HUMAN]	2.91	1	1	1	1	0.96	0.62	1.04	0.66					515	57.2	6.52
Q96RD9	Fc receptor-like protein 5 OS=Homo sapiens GN=FCRL5 PE=1 SV=3 - [FCRL5_HUMAN]	4.30	1	3	3	6	1.15	1.29	1.25	1.40	0.89	0.95	0.95	1.05	977	106.4	7.14
Q86WN1	FCH and double SH3 domains protein 1 OS=Homo sapiens GN=FCHSD1 PE=1 SV=1 - [FCSD1_HUMAN]	5.07	1	1	1	5	2.14	1.59	1.49	1.10					690	76.9	5.34
A9Z1Z3	Fer-1-like protein 4 OS=Homo sapiens GN=FER1L4 PE=2 SV=1 - [FR1L4_HUMAN]	0.72	1	1	1	2	0.70	0.64	0.70	0.65					1794	200.9	6.24
A0AVI2	Fer-1-like protein 5 OS=Homo sapiens GN=FER1L5 PE=2 SV=2 - [FR1L5_HUMAN]	0.76	1	1	1	6	0.56	0.53	0.60	0.57	0.54	0.49	0.54	0.49	2093	241.8	7.96
Q2WGJ9	Fer-1-like protein 6 OS=Homo sapiens GN=FER1L6 PE=2 SV=2 - [FR1L6_HUMAN]	1.51	1	2	2	2					1.27	1.46	1.11	1.27	1857	209.2	6.38
Q5SYB0	FERM and PDZ domain-containing protein 1 OS=Homo sapiens GN=FRMPD1 PE=1 SV=1 - [FRPD1_HUMAN]	0.44	1	1	1	4	1.87	1.80	1.73	1.71					1578	173.3	5.25
Q68DX3	FERM and PDZ domain-containing protein 2 OS=Homo sapiens GN=FRMPD2 PE=1 SV=3 - [FRPD2_HUMAN]	1.45	1	1	1	1	0.92	0.97	0.93	0.98					1309	144.2	6.74
Q5JV73	FERM and PDZ domain-containing protein 3 OS=Homo sapiens GN=FRMPD3 PE=2 SV=2 - [FRPD3_HUMAN]	2.04	1	2	3	17	1.08	0.78	0.84	0.65	1.19	1.27	1.36	1.35	1810	199.1	8.15
Q14CM0	FERM and PDZ domain-containing protein 4 OS=Homo sapiens GN=FRMPD4 PE=1 SV=1 - [FRPD4_HUMAN]	3.86	1	2	2	2	1.19	1.30	1.19	1.30					1322	144.3	5.25
Q9BQL6	Fermitin family homolog 1 OS=Homo sapiens GN=FERMT1 PE=1 SV=1 - [FERM1_HUMAN]	1.92	1	1	1	1					0.48	0.53	0.48	0.53	677	77.4	6.28
Q96AC1	Fermitin family homolog 2 OS=Homo sapiens GN=FERMT2 PE=1 SV=1 - [FERM2_HUMAN]	5.59	1	2	2	3			1.05	1.03	1.27	0.83	1.15	0.76	680	77.8	6.70

Q86UX7	Fermitin family homolog 3 OS=Homo sapiens GN=FERMT3 PE=1 SV=1 - [URP2_HUMAN]	29.39	1	13	13	44	0.67	0.67	0.68	0.68	0.96	1.01	0.87	0.92	667	75.9	6.98
Q6ZNA5	Ferric-chelate reductase 1 OS=Homo sapiens GN=FRRS1 PE=2 SV=2 - [FRRS1_HUMAN]	3.72	1	1	1	1					0.77	0.75	0.64	0.63	592	66.1	7.47
Q9UGM5	Fetuin-B OS=Homo sapiens GN=FETUB PE=1 SV=2 - [FETUB_HUMAN]	48.69	1	13	13	84	1.45	1.46	1.45	1.47	1.16	1.43	1.03	1.25	382	42.0	6.83
Q9Y613	FH1/FH2 domain-containing protein 1 OS=Homo sapiens GN=FHOD1 PE=1 SV=3 - [FHOD1_HUMAN]	1.29	1	1	1	2	0.55	0.54	0.56	0.55					1164	126.5	6.39
Q2V2M9	FH1/FH2 domain-containing protein 3 OS=Homo sapiens GN=FHOD3 PE=1 SV=2 - [FHOD3_HUMAN]	1.05	9	1	2	2	0.97	0.68	1.15	0.81					1422	158.5	5.99
Q9C0D6	FH2 domain-containing protein 1 OS=Homo sapiens GN=FHDC1 PE=2 SV=2 - [FHDC1_HUMAN]	1.57	1	1	1	1					1.13	1.20	0.91	0.96	1143	124.7	9.03
P35555	Fibrillin-1 OS=Homo sapiens GN=FBN1 PE=1 SV=3 - [FBN1_HUMAN]	12.92	1	27	29	60	0.98	0.90	0.91	0.85	1.04	0.83	0.83	0.67	2871	312.0	4.93
P35556	Fibrillin-2 OS=Homo sapiens GN=FBN2 PE=1 SV=3 - [FBN2_HUMAN]	1.65	1	2	4	5	1.12	0.65	0.84	0.57					2912	314.6	4.86
Q75N90	Fibrillin-3 OS=Homo sapiens GN=FBN3 PE=2 SV=3 - [FBN3_HUMAN]	0.64	1	2	2	3	1.15	1.19	1.03	1.07					2809	300.1	5.07
P02671	Fibrinogen alpha chain OS=Homo sapiens GN=FGA PE=1 SV=2 - [FIBA_HUMAN]	48.85	1	42	42	1773	0.68	0.66	0.66	0.65	0.86	0.87	0.91	0.92	866	94.9	6.01
P02675	Fibrinogen beta chain OS=Homo sapiens GN=FGB PE=1 SV=2 - [FIBB_HUMAN]	63.34	1	27	27	591	0.32	0.30	0.30	0.27	0.32	0.36	0.34	0.38	491	55.9	8.27
Q8N539	Fibrinogen C domain-containing protein 1 OS=Homo sapiens GN=FIBCD1 PE=1 SV=2 - [FBCD1_HUMAN]	4.12	1	1	1	2	0.86	0.72	0.97	0.81					461	50.7	6.48
P02679	Fibrinogen gamma chain OS=Homo sapiens GN=FGG PE=1 SV=3 - [FIBG_HUMAN]	60.26	1	26	26	361	0.63	0.57	0.64	0.57	0.27	0.28	0.23	0.25	453	51.5	5.62
O95073	Fibrinogen silencer-binding protein OS=Homo sapiens GN=FSBP PE=1 SV=1 - [FSBP_HUMAN]	3.68	1	1	1	1					0.70	0.50	0.52	0.37	299	34.7	7.12
P61328	Fibroblast growth factor 12 OS=Homo sapiens GN=FGF12 PE=1 SV=1 - [FGF12_HUMAN]	7.82	1	3	3	3					0.98	0.85	0.89	0.79	243	27.4	9.96
O60258	Fibroblast growth factor 17 OS=Homo sapiens GN=FGF17 PE=1 SV=1 - [FGF17_HUMAN]	3.70	1	1	1	1	1.35	0.78	1.06	0.62					216	24.9	10.42
Q9NP95	Fibroblast growth factor 20 OS=Homo sapiens GN=FGF20 PE=1 SV=1 - [FGF20_HUMAN]	2.84	1	1	1	2					1.04	1.37	0.75	0.98	211	23.5	8.76
P11362	Fibroblast growth factor receptor 1 OS=Homo sapiens GN=FGFR1 PE=1 SV=3 - [FGFR1_HUMAN]	3.89	1	3	3	5	0.88	1.01	0.98	1.02					822	91.8	6.21
Q9BYJ0	Fibroblast growth factor-binding protein 2 OS=Homo sapiens GN=FGFBP2 PE=1 SV=1 - [FGFP2_HUMAN]	7.17	1	1	1	3					1.00	1.15	0.89	1.02	223	24.6	8.87
P08F94	Fibrocystin OS=Homo sapiens GN=PKHD1 PE=1 SV=1 - [PKHD1_HUMAN]	0.74	1	1	1	1	1.15	0.44	1.36	0.52					4074	446.4	6.57
Q86WI1	Fibrocystin-L OS=Homo sapiens GN=PKHD1L1 PE=2 SV=2 - [PKHL1_HUMAN]	1.70	1	4	4	6	0.95	0.88	0.94	1.03					4243	465.4	6.11
P02751	Fibronectin OS=Homo sapiens GN=FN1 PE=1 SV=4 - [FINC_HUMAN]	57.84	1	115	115	4463	1.13	1.15	1.14	1.18	0.99	0.99	1.01	1.00	2386	262.5	5.71
Q8TC84	Fibronectin type 3 and ankyrin repeat domains protein 1 OS=Homo sapiens GN=FANK1 PE=2 SV=3 - [FANK1_HUMAN]	3.48	1	1	1	1					0.93	0.89	0.90	0.87	345	38.3	8.73
Q9BTV5	Fibronectin type III and SPRY domain-containing protein 1 OS=Homo sapiens GN=FSD1 PE=1 SV=1 - [FSD1_HUMAN]	2.62	1	2	2	16					1.02	0.94	0.94	0.93	496	55.8	6.99
Q4ZHG4	Fibronectin type III domain-containing protein 1 OS=Homo sapiens GN=FNDC1 PE=1 SV=4 - [FNDC1_HUMAN]	1.43	1	2	2	3	1.13	1.08	1.39	1.34	1.19	1.10	1.10	1.02	1894	205.4	9.32
Q5CZC0	Fibrous sheath-interacting protein 2 OS=Homo sapiens GN=FSIP2 PE=1 SV=4 - [FSIP2_HUMAN]	1.13	1	5	6	6	0.98	0.83	1.05	0.90	0.37	0.64	0.71	1.23	6907	780.1	6.71
P23142	Fibulin-1 OS=Homo sapiens GN=FBLN1 PE=1 SV=4 - [FBLN1_HUMAN]	47.94	1	23	23	190	1.07	1.01	1.06	1.02	0.90	0.89	1.05	0.96	703	77.2	5.22

Q9UBX5	Fibulin-5 OS=Homo sapiens GN=FBLN5 PE=1 SV=1 - [FBLN5_HUMAN]	10.71	1	4	4	10	1.13	1.06	1.17	1.10	1.24	1.27	1.24	1.28	448	50.1	4.73
O00602	Ficolin-1 OS=Homo sapiens GN=FCN1 PE=1 SV=2 - [FCN1_HUMAN]	14.42	1	3	3	8	1.08	1.06	1.13	1.11	1.06	1.20	1.24	1.39	326	35.1	6.86
Q15485	Ficolin-2 OS=Homo sapiens GN=FCN2 PE=1 SV=2 - [FCN2_HUMAN]	14.70	1	3	3	44	1.07	0.94	1.08	0.93	0.88	0.84	0.87	0.85	313	34.0	6.77
O75636	Ficolin-3 OS=Homo sapiens GN=FCN3 PE=1 SV=2 - [FCN3_HUMAN]	56.86	2	12	13	135	1.10	1.15	1.14	1.21	1.00	1.09	0.99	1.09	299	32.9	6.67
Q6PIW4	Fidgetin-like protein 1 OS=Homo sapiens GN=FIGNL1 PE=1 SV=2 - [FIGL1_HUMAN]	1.63	1	1	1	1					1.22	0.94	0.98	0.75	674	74.0	7.85
P20930	Filaggrin OS=Homo sapiens GN=FLG PE=1 SV=3 - [FILA_HUMAN]	3.94	1	2	2	2	0.63	0.77	0.84	1.03	1.07	0.71	1.42	0.94	4061	434.9	9.25
Q5D862	Filaggrin-2 OS=Homo sapiens GN=FLG2 PE=1 SV=1 - [FILA2_HUMAN]	2.05	1	2	2	5	0.72	0.81	0.50	0.59					2391	247.9	8.31
Q4L180	Filamin A-interacting protein 1-like OS=Homo sapiens GN=FILIP1L PE=1 SV=2 - [FIL1L_HUMAN]	1.15	1	1	1	1					0.97	0.42	0.97	0.42	1135	130.3	6.57
P21333	Filamin-A OS=Homo sapiens GN=FLNA PE=1 SV=4 - [FLNA_HUMAN]	38.87	2	72	77	446	0.79	0.78	0.79	0.78	1.08	1.02	1.06	1.06	2647	280.6	6.06
O75369	Filamin-B OS=Homo sapiens GN=FLNB PE=1 SV=2 - [FLNB_HUMAN]	3.42	1	2	7	18	0.92	0.93	1.23	1.24					2602	278.0	5.73
Q9UM11	Fizzy-related protein homolog OS=Homo sapiens GN=FZR1 PE=1 SV=2 - [FZR_HUMAN]	8.06	1	2	2	6	1.19	1.33	1.25	1.40	1.02	0.83	1.14	0.93	496	55.1	9.33
Q9UIM3	FK506-binding protein-like OS=Homo sapiens GN=FKBPL PE=1 SV=1 - [FKBPL_HUMAN]	8.02	1	1	1	2	0.32	0.33	0.43	0.45					349	38.2	5.50
Q17RS7	Flap endonuclease GEN homolog 1 OS=Homo sapiens GN=GEN1 PE=1 SV=2 - [GEN_HUMAN]	1.10	1	1	1	1					0.66	7.72	1.10	####	908	102.8	7.81
P30043	Flavin reductase (NADPH) OS=Homo sapiens GN=BLVRB PE=1 SV=3 - [BLVRB_HUMAN]	30.58	1	5	5	25	0.61	0.59	0.64	0.64	0.60	0.63	0.62	0.65	206	22.1	7.65
Q14254	Flotillin-2 OS=Homo sapiens GN=FLOT2 PE=1 SV=2 - [FLOT2_HUMAN]	1.40	1	1	1	1	2.63	3.08	2.39	2.80					428	47.0	5.25
P41439	Folate receptor gamma OS=Homo sapiens GN=FOLR3 PE=1 SV=1 - [FOLR3_HUMAN]	7.82	1	2	2	4	0.98	1.12	0.90	1.03	1.37	1.39	1.01	1.26	243	27.6	7.83
Q53S99	Folate transporter-like protein C2orf83 OS=Homo sapiens GN=C2orf83 PE=2 SV=1 - [CB083_HUMAN]	15.33	1	1	1	1	1.85	1.43	1.86	1.45					150	16.4	6.67
P23945	Follicle-stimulating hormone receptor OS=Homo sapiens GN=FSHR PE=1 SV=3 - [FSHR_HUMAN]	1.58	1	1	1	2	8.37	4.23	1.89	0.96					695	78.2	7.18
Q9P278	Folliculin-interacting protein 2 OS=Homo sapiens GN=FNIP2 PE=1 SV=2 - [FNIP2_HUMAN]	1.53	1	2	2	4	1.79	1.81	1.78	1.80	0.87	1.05	0.73	1.03	1114	122.0	6.62
Q12841	Follistatin-related protein 1 OS=Homo sapiens GN=FSTL1 PE=1 SV=1 - [FSTL1_HUMAN]	2.60	1	1	1	2	2.28	2.23	1.49	1.47					308	35.0	5.52
Q6MZW2	Follistatin-related protein 4 OS=Homo sapiens GN=FSTL4 PE=2 SV=3 - [FSTL4_HUMAN]	0.83	1	1	1	1	1.88	2.18	0.93	1.08					842	93.0	6.32
Q9UJU5	Forkhead box protein D3 OS=Homo sapiens GN=FOXD3 PE=1 SV=1 - [FOXD3_HUMAN]	2.72	1	1	1	2					0.74	1.04	0.85	1.18	478	47.6	6.46
Q9NU39	Forkhead box protein D4-like 1 OS=Homo sapiens GN=FOXD4L1 PE=2 SV=1 - [FX4L1_HUMAN]	6.86	1	1	1	1	1.10	1.27	1.28	1.48					408	43.6	9.29
Q9UPW0	Forkhead box protein J3 OS=Homo sapiens GN=FOXJ3 PE=1 SV=2 - [FOXJ3_HUMAN]	1.77	1	1	1	1	0.99	0.89	0.98	0.88					622	68.9	7.09
P85037	Forkhead box protein K1 OS=Homo sapiens GN=FOXK1 PE=1 SV=1 - [FOXK1_HUMAN]	3.27	1	1	1	1	0.74	0.67	0.77	0.71					733	75.4	9.32
Q9H334	Forkhead box protein P1 OS=Homo sapiens GN=FOXP1 PE=1 SV=1 - [FOXP1_HUMAN]	1.92	1	1	1	1					1.19	1.22	1.33	1.36	677	75.3	6.67
O43638	Forkhead box protein S1 OS=Homo sapiens GN=FOXS1 PE=2 SV=2 - [FOXS1_HUMAN]	8.18	1	1	1	1					0.83	0.85	1.01	1.03	330	35.4	9.07

Q8N3X1	Formin-binding protein 4 OS=Homo sapiens GN=FNBP4 PE=1 SV=3 - [FNBP4_HUMAN]	0.49	1	1	1	2					####	###	8.60	8.49	1017	110.2	4.74
O95466	Formin-like protein 1 OS=Homo sapiens GN=FMNL1 PE=1 SV=3 - [FMNL_HUMAN]	2.45	1	2	2	2	1.21	1.20	1.07	1.06	1.39	1.55	0.99	1.11	1100	121.8	5.72
Q13642	Four and a half LIM domains protein 1 OS=Homo sapiens GN=FHL1 PE=1 SV=4 - [FHL1_HUMAN]	13.62	1	4	4	10	0.80	0.93	0.85	1.00	1.42	1.40	1.30	1.29	323	36.2	8.97
P78423	Fractalkine OS=Homo sapiens GN=CX3CL1 PE=1 SV=1 - [X3CL1_HUMAN]	12.09	1	2	2	4					1.04	0.24	1.43	0.09	397	42.2	6.52
Q5SZK8	FRAS1-related extracellular matrix protein 2 OS=Homo sapiens GN=FREM2 PE=1 SV=2 - [FREM2_HUMAN]	0.92	1	2	2	2	1.68	1.42	1.97	1.67					3169	350.9	5.03
P0C091	FRAS1-related extracellular matrix protein 3 OS=Homo sapiens GN=FREM3 PE=2 SV=2 - [FREM3_HUMAN]	0.51	1	1	1	6	1.49	0.82	1.75	1.07	1.12	1.23	0.87	0.97	2139	238.0	5.33
Q16595	Frataxin, mitochondrial OS=Homo sapiens GN=FXN PE=1 SV=2 - [FRDA_HUMAN]	9.05	1	1	1	1					0.46	0.49	0.48	0.52	210	23.1	8.69
Q9UP38	Frizzled-1 OS=Homo sapiens GN=FZD1 PE=1 SV=2 - [FZD1_HUMAN]	6.96	1	3	3	3					0.61	0.18	0.81	0.24	647	71.1	7.99
Q9NPG1	Frizzled-3 OS=Homo sapiens GN=FZD3 PE=1 SV=1 - [FZD3_HUMAN]	2.10	1	1	1	1	0.83	1.01	0.73	0.89					666	76.2	7.74
Q13467	Frizzled-5 OS=Homo sapiens GN=FZD5 PE=2 SV=2 - [FZD5_HUMAN]	1.20	1	1	1	2					0.69	0.67	0.73	0.70	585	64.5	8.29
P09467	Fructose-1,6-bisphosphatase 1 OS=Homo sapiens GN=FBP1 PE=1 SV=5 - [F16P1_HUMAN]	7.40	2	2	2	3	0.68	0.66	0.73	0.72	0.72	0.88	0.56	0.69	338	36.8	6.99
P04075	Fructose-bisphosphate aldolase A OS=Homo sapiens GN=ALDOA PE=1 SV=2 - [ALDOA_HUMAN]	40.38	1	11	11	43	0.75	0.75	0.78	0.79	0.77	0.78	0.70	0.71	364	39.4	8.09
P05062	Fructose-bisphosphate aldolase B OS=Homo sapiens GN=ALDOB PE=1 SV=2 - [ALDOB_HUMAN]	20.88	1	6	6	12	0.72	0.74	0.72	0.70	0.72	0.70	0.69	0.67	364	39.4	7.87
P16930	Fumarylacetoacetase OS=Homo sapiens GN=FAH PE=1 SV=2 - [FAAA_HUMAN]	8.59	1	3	3	10	0.89	0.87	0.91	0.90	0.78	0.82	0.76	0.80	419	46.3	6.95
P09958	Furin OS=Homo sapiens GN=FURIN PE=1 SV=2 - [FURIN_HUMAN]	0.63	1	1	1	1					0.14	0.97	0.27	1.88	794	86.6	6.47
Q9H0Q3	FXYD domain-containing ion transport regulator 6 OS=Homo sapiens GN=FXYD6 PE=2 SV=1 - [FXYD6_HUMAN]	24.21	1	1	1	1	0.71	0.59	0.83	0.70					95	10.5	5.10
O15117	FYN-binding protein OS=Homo sapiens GN=FYB PE=1 SV=2 - [FYB_HUMAN]	9.83	1	6	6	13	1.60	1.70	1.61	1.72	1.30	1.27	1.14	1.12	783	85.3	6.48
Q9BQS8	FYVE and coiled-coil domain-containing protein 1 OS=Homo sapiens GN=FYCO1 PE=1 SV=3 - [FYCO1_HUMAN]	1.01	1	1	1	2					0.77	0.71	0.95	0.88	1478	166.9	4.92
Q6ZNL6	FYVE, RhoGEF and PH domain-containing protein 5 OS=Homo sapiens GN=FGD5 PE=1 SV=3 - [FGD5_HUMAN]	2.12	1	1	1	1	1.11	1.13	1.08	1.10					1462	159.8	5.01
Q6ZV73	FYVE, RhoGEF and PH domain-containing protein 6 OS=Homo sapiens GN=FGD6 PE=1 SV=2 - [FGD6_HUMAN]	1.19	1	1	1	1					0.99	0.04	0.77	0.03	1430	160.7	7.03
O75459	G antigen family B member 1 OS=Homo sapiens GN=PAGE1 PE=1 SV=2 - [GAGB1_HUMAN]	23.97	1	2	2	3					1.15	0.89	1.16	0.90	146	16.1	4.22
Q8WTP9	G antigen family D member 4 OS=Homo sapiens GN=XAGE3 PE=2 SV=1 - [GAGD4_HUMAN]	9.91	1	1	1	1						0.50		1.29	111	12.3	4.49
Q9NW75	G patch domain-containing protein 2 OS=Homo sapiens GN=GPATCH2 PE=1 SV=1 - [GPTC2_HUMAN]	2.46	1	1	1	3					1.34	1.47	0.82	1.06	528	58.9	9.09
P48549	G protein-activated inward rectifier potassium channel 1 OS=Homo sapiens GN=KCNJ3 PE=1 SV=1 - [IRK3_HUMAN]	3.19	1	2	2	4	1.18	1.23	1.21	1.27	0.79	0.73	0.76	0.70	501	56.6	8.22
P48051	G protein-activated inward rectifier potassium channel 2 OS=Homo sapiens GN=KCNJ6 PE=1 SV=1 - [IRK6_HUMAN]	3.07	1	1	1	2							0.04	0.06	423	48.4	5.38
P43250	G protein-coupled receptor kinase 6 OS=Homo sapiens GN=GRK6 PE=1 SV=2 - [GRK6_HUMAN]	2.95	1	1	1	1	1.76	1.18	1.07	0.73					576	65.9	8.00
Q8WTQ7	G protein-coupled receptor kinase 7 OS=Homo sapiens GN=GRK7 PE=1 SV=1 - [GRK7_HUMAN]	3.62	1	1	1	2					0.96	1.30	1.13	1.53	553	62.2	6.57

Q7Z2K8	G protein-regulated inducer of neurite outgrowth 1 OS=Homo sapiens GN=GPRIN1 PE=1 SV=2 - [GRIN1_HUMAN]	1.59	1	1	1	1					0.69	1.17	1.17	1.97	1008	102.3	8.06
Q96PE1	G-protein coupled receptor 124 OS=Homo sapiens GN=GPR124 PE=1 SV=2 - [GP124_HUMAN]	2.09	1	1	1	1	0.84	1.12	0.87	1.16					1338	142.6	8.44
Q86SQ4	G-protein coupled receptor 126 OS=Homo sapiens GN=GPR126 PE=1 SV=3 - [GP126_HUMAN]	2.29	1	2	2	4	1.13	1.21	1.27	1.41					1221	136.6	7.87
P46089	G-protein coupled receptor 3 OS=Homo sapiens GN=GPR3 PE=1 SV=1 - [GPR3_HUMAN]	1.52	2	1	1	1					0.74	1.17	0.67	1.06	330	35.0	8.06
P46095	G-protein coupled receptor 6 OS=Homo sapiens GN=GPR6 PE=1 SV=1 - [GPR6_HUMAN]	1.38	1	1	1	1					1.30	0.18	1.29	0.18	362	37.9	7.68
Q8IZP9	G-protein coupled receptor 64 OS=Homo sapiens GN=GPR64 PE=1 SV=2 - [GPR64_HUMAN]	3.15	1	2	2	4	1.08	1.10	1.12	1.15					1017	111.5	7.55
Q8WXG9	G-protein coupled receptor 98 OS=Homo sapiens GN=GPR98 PE=1 SV=2 - [GPR98_HUMAN]	0.76	3	2	3	3	0.85	0.96	0.88	1.00					6306	692.6	4.64
Q9NZD1	G-protein coupled receptor family C group 5 member D OS=Homo sapiens GN=GPRC5D PE=2 SV=1 - [GPC5D_HUMAN]	2.90	1	1	1	2					0.75	0.90	0.79	0.95	345	38.8	5.00
Q5T6X5	G-protein coupled receptor family C group 6 member A OS=Homo sapiens GN=GPRC6A PE=1 SV=1 - [GPC6A_HUMAN]	1.94	1	1	1	2	1.11	1.21	1.15	1.26					926	104.7	7.96
Q5JY77	G-protein coupled receptor-associated sorting protein 1 OS=Homo sapiens GN=GPRASP1 PE=1 SV=3 - [GASP1_HUMAN]	1.86	1	1	1	3	0.60	0.38	0.51	0.33					1395	156.8	4.68
P81274	G-protein-signaling modulator 2 OS=Homo sapiens GN=GPSM2 PE=1 SV=3 - [GPSM2_HUMAN]	6.29	1	2	3	3					1.03	1.18	1.40	1.61	684	76.6	6.39
Q9NYZ3	G2 and S phase-expressed protein 1 OS=Homo sapiens GN=GTSE1 PE=1 SV=3 - [GTSE1_HUMAN]	3.47	1	1	1	1	1.15	1.69	1.42	2.12					720	76.6	9.39
Q8WWL7	G2/mitotic-specific cyclin-B3 OS=Homo sapiens GN=CCNB3 PE=1 SV=2 - [CCNB3_HUMAN]	0.79	1	1	1	3							1.05		1395	157.8	6.68
Q06546	GA-binding protein alpha chain OS=Homo sapiens GN=GABPA PE=1 SV=1 - [GABPA_HUMAN]	5.29	1	1	1	1	1.11	1.27	0.84	0.97					454	51.3	4.97
P51570	Galactokinase OS=Homo sapiens GN=GALK1 PE=1 SV=1 - [GALK1_HUMAN]	2.81	1	1	1	3	0.87	0.79	1.24	1.12	2.48	2.23	2.36	2.12	392	42.2	6.46
Q96RP7	Galactose-3-O-sulfotransferase 4 OS=Homo sapiens GN=GAL3ST4 PE=1 SV=1 - [G3ST4_HUMAN]	6.58	1	2	2	2	0.59	0.17	0.64	0.18	0.56	0.84	0.41	0.62	486	54.1	10.18
O94766	Galactosylgalactosylxylosylprotein 3-beta-glucuronosyltransferase 3 OS=Homo sapiens GN=B3GAT3 PE=1 SV=2 - [B3GA3_HUMAN]	4.78	1	1	2	3					0.50	0.29	1.01	0.59	335	37.1	8.27
P47211	Galanin receptor type 1 OS=Homo sapiens GN=GALR1 PE=2 SV=3 - [GALR1_HUMAN]	3.44	1	1	1	2					0.76	0.61	1.24	1.00	349	38.9	9.47
P09382	Galectin-1 OS=Homo sapiens GN=LGALS1 PE=1 SV=2 - [LEG1_HUMAN]	46.67	1	4	4	13					0.87	0.97	0.88	0.94	135	14.7	5.50
P05162	Galectin-2 OS=Homo sapiens GN=LGALS2 PE=1 SV=3 - [LEG2_HUMAN]	6.82	1	1	1	1	0.70	0.62	0.66	0.59					132	14.6	6.38
P17931	Galectin-3 OS=Homo sapiens GN=LGALS3 PE=1 SV=5 - [LEG3_HUMAN]	19.60	1	5	5	9					1.10	1.07	1.01	0.98	250	26.1	8.56
Q08380	Galectin-3-binding protein OS=Homo sapiens GN=LGALS3BP PE=1 SV=1 - [LG3BP_HUMAN]	43.25	1	20	20	204	1.04	1.07	1.07	1.06	1.08	1.10	1.11	1.18	585	65.3	5.27
O00182	Galectin-9 OS=Homo sapiens GN=LGALS9 PE=1 SV=2 - [LEG9_HUMAN]	6.48	3	3	3	7	0.94	0.93	1.13	1.12	0.52	0.64	0.60	0.69	355	39.5	9.17
Q3ZCW2	Galectin-related protein OS=Homo sapiens GN=LGALSL PE=1 SV=2 - [LEGL_HUMAN]	41.86	1	6	6	26	1.04	1.45	0.89	1.16	1.26	1.20	1.20	1.10	172	19.0	5.35
Q86UU5	Gametogenetin OS=Homo sapiens GN=GGN PE=2 SV=2 - [GGN_HUMAN]	8.74	1	2	2	2	0.98	1.14	1.04	1.21	0.47	0.52	0.76	0.85	652	66.7	10.29
Q9UEY8	Gamma-adducin OS=Homo sapiens GN=ADD3 PE=1 SV=1 - [ADDG_HUMAN]	2.97	1	1	1	1					0.31	0.27	0.85	0.75	706	79.1	6.32
P14867	Gamma-aminobutyric acid receptor subunit alpha-1 OS=Homo sapiens GN=GABRA1 PE=1 SV=3 - [GBRA1_HUMAN]	4.39	1	1	1	1	0.89	0.76	0.47	0.41					456	51.8	9.22

P47869	Gamma-aminobutyric acid receptor subunit alpha-2 OS=Homo sapiens GN=GABRA2 PE=2 SV=2 - [GBRA2_HUMAN]	1.77	1	1	1	1					0.69	0.73	0.77	0.81	451	51.3	9.11
Q16445	Gamma-aminobutyric acid receptor subunit alpha-6 OS=Homo sapiens GN=GABRA6 PE=2 SV=2 - [GBRA6_HUMAN]	9.05	1	3	3	4	0.70	0.68	0.93	0.91	0.75	0.94	0.88	1.19	453	51.0	8.22
P28472	Gamma-aminobutyric acid receptor subunit beta-3 OS=Homo sapiens GN=GABRB3 PE=1 SV=1 - [GBRB3_HUMAN]	8.88	1	2	2	2	0.71	0.58	0.98	0.80	1.17	1.27	1.02	1.11	473	54.1	9.11
O00591	Gamma-aminobutyric acid receptor subunit pi OS=Homo sapiens GN=GABRP PE=1 SV=1 - [GBRP_HUMAN]	4.32	1	1	1	5					1.90	1.33	1.91	1.34	440	50.6	6.99
P24046	Gamma-aminobutyric acid receptor subunit rho-1 OS=Homo sapiens GN=GABRR1 PE=2 SV=2 - [GBRR1_HUMAN]	3.34	1	1	1	1					1.10	1.24	1.14	1.29	479	55.8	8.60
Q9UBS5	Gamma-aminobutyric acid type B receptor subunit 1 OS=Homo sapiens GN=GABBR1 PE=1 SV=1 - [GABR1_HUMAN]	2.08	1	2	2	2	0.94	1.11	0.66	0.78	0.80	0.69	1.11	0.96	961	108.3	8.21
O75899	Gamma-aminobutyric acid type B receptor subunit 2 OS=Homo sapiens GN=GABBR2 PE=1 SV=1 - [GABR2_HUMAN]	1.38	1	1	1	1	1.06	1.03	1.13	1.10					941	105.8	8.66
Q92820	Gamma-glutamyl hydrolase OS=Homo sapiens GN=GGH PE=1 SV=2 - [GGH_HUMAN]	23.58	1	6	6	28	1.09	1.15	1.09	1.20	0.89	1.01	0.85	0.99	318	35.9	7.11
O75223	Gamma-glutamylcyclotransferase OS=Homo sapiens GN=GGCT PE=1 SV=1 - [GGCT_HUMAN]	5.32	1	1	1	8	0.98	0.76	1.04	0.81	0.96	0.96	0.92	0.91	188	21.0	5.14
P36269	Gamma-glutamyltransferase 5 OS=Homo sapiens GN=GGT5 PE=1 SV=2 - [GGT5_HUMAN]	7.68	1	3	3	5	1.10	1.05	1.03	0.98	1.03	0.88	1.26	1.08	586	62.2	7.55
Q9UJ14	Gamma-glutamyltransferase 7 OS=Homo sapiens GN=GGT7 PE=1 SV=2 - [GGT7_HUMAN]	1.96	1	1	1	1	1.38	1.16	1.33	1.11					662	70.4	5.10
P13284	Gamma-interferon-inducible lysosomal thiol reductase OS=Homo sapiens GN=IFI30 PE=1 SV=3 - [GILT_HUMAN]	9.60	1	2	2	11	0.89	0.99	0.90	1.00	0.91	0.92	0.90	0.91	250	27.9	4.88
Q9BSJ2	Gamma-tubulin complex component 2 OS=Homo sapiens GN=TUBGCP2 PE=1 SV=2 - [GCP2_HUMAN]	2.55	1	1	1	4	0.72	0.84	0.66	0.78					902	102.5	6.84
Q96CW5	Gamma-tubulin complex component 3 OS=Homo sapiens GN=TUBGCP3 PE=1 SV=2 - [GCP3_HUMAN]	0.77	1	1	1	2					1.00	0.62	0.82	0.51	907	103.5	8.12
Q96RT8	Gamma-tubulin complex component 5 OS=Homo sapiens GN=TUBGCP5 PE=1 SV=1 - [GCP5_HUMAN]	0.88	1	1	1	1	1.29	1.11	1.15	0.99					1024	118.2	5.90
Q96RT7	Gamma-tubulin complex component 6 OS=Homo sapiens GN=TUBGCP6 PE=1 SV=3 - [GCP6_HUMAN]	1.10	1	1	1	1					1.46	1.29	1.08	0.95	1819	200.4	6.32
P17900	Ganglioside GM2 activator OS=Homo sapiens GN=GM2A PE=1 SV=4 - [SAP3_HUMAN]	14.51	1	2	2	4					0.95	0.93	0.87	0.85	193	20.8	5.31
Q9NXN4	Ganglioside-induced differentiation-associated protein 2 OS=Homo sapiens GN=GDAP2 PE=2 SV=1 - [GDAP2_HUMAN]	3.62	1	1	1	4	1.19	2.06	0.73	1.27					497	56.2	5.74
P36382	Gap junction alpha-5 protein OS=Homo sapiens GN=GJA5 PE=1 SV=3 - [CXA5_HUMAN]	1.68	1	1	1	1					1.00	0.20	1.00	0.20	358	40.4	8.50
P48165	Gap junction alpha-8 protein OS=Homo sapiens GN=GJA8 PE=1 SV=3 - [CXA8_HUMAN]	4.85	1	1	1	1	1.13	1.34	1.14	1.36					433	48.2	5.27
P57773	Gap junction alpha-9 protein OS=Homo sapiens GN=GJA9 PE=2 SV=2 - [CXA9_HUMAN]	10.49	1	3	3	4	1.44	0.88	1.76	1.08					515	58.8	8.37
Q99501	GAS2-like protein 1 OS=Homo sapiens GN=GAS2L1 PE=1 SV=2 - [GA2L1_HUMAN]	2.79	1	1	1	1					0.75	0.88	0.71	0.84	681	72.7	10.05
Q96QA5	Gasdermin-A OS=Homo sapiens GN=GSDMA PE=2 SV=4 - [GSDMA_HUMAN]	4.27	1	1	1	3	1.30	1.53	1.28	1.51					445	49.3	5.29
Q9BYG8	Gasdermin-C OS=Homo sapiens GN=GSDMC PE=2 SV=3 - [GSDMC_HUMAN]	2.95	1	1	1	1	0.44	0.66	0.38	0.57					508	57.7	5.78
P09681	Gastric inhibitory polypeptide OS=Homo sapiens GN=GIP PE=1 SV=1 - [GIP_HUMAN]	9.15	1	1	1	4					1.49	1.73	1.41	1.63	153	17.1	8.00
P27352	Gastric intrinsic factor OS=Homo sapiens GN=GIF PE=1 SV=2 - [IF_HUMAN]	7.19	1	1	1	1					0.95	2.01			417	45.4	6.14
P20142	Gastricsin OS=Homo sapiens GN=PGC PE=1 SV=1 - [PEPC_HUMAN]	1.80	1	1	1	8	0.86	0.86	0.69	0.69	0.62	0.56	0.92	0.83	388	42.4	4.46

Q9NS71	Gastrokine-1 OS=Homo sapiens GN=GKN1 PE=2 SV=3 - [GKN1_HUMAN]	10.55	1	2	2	2					1.03	0.89	1.06	0.92	199	22.0	6.32
Q9Y5B6	GC-rich sequence DNA-binding factor 1 OS=Homo sapiens GN=GCFC1 PE=1 SV=2 - [GCFC1_HUMAN]	4.58	1	4	4	7	1.13	1.36	0.80	1.00	0.76	0.83	0.81	0.89	917	104.7	5.68
O95479	GDH/6PGL endoplasmic bifunctional protein OS=Homo sapiens GN=H6PD PE=1 SV=2 - [G6PE_HUMAN]	7.84	1	5	5	14	0.77	0.81	0.68	0.67	1.01	1.02	1.11	1.02	791	88.8	7.30
P56159	GDNF family receptor alpha-1 OS=Homo sapiens GN=GFRA1 PE=2 SV=2 - [GFRA1_HUMAN]	2.80	1	1	1	1					2.06	2.30	1.53	1.71	465	51.4	7.91
P06396	Gelsolin OS=Homo sapiens GN=GSN PE=1 SV=1 - [GELS_HUMAN]	60.49	1	42	42	819	0.93	0.87	0.94	0.88	0.92	0.98	0.88	0.96	782	85.6	6.28
Q12789	General transcription factor 3C polypeptide 1 OS=Homo sapiens GN=GTF3C1 PE=1 SV=4 - [TF3C1_HUMAN]	0.57	1	1	1	1	0.84	0.91	0.82	0.90					2109	238.7	7.30
Q8WUA4	General transcription factor 3C polypeptide 2 OS=Homo sapiens GN=GTF3C2 PE=1 SV=2 - [TF3C2_HUMAN]	2.63	1	2	2	4	0.70	0.73	0.84	0.88	0.73	0.78	0.68	0.73	911	100.6	7.31
Q9Y5Q9	General transcription factor 3C polypeptide 3 OS=Homo sapiens GN=GTF3C3 PE=1 SV=1 - [TF3C3_HUMAN]	2.37	1	2	2	2	0.77	0.81	0.76	0.80	1.65	1.46	1.38	1.22	886	101.2	5.07
P78347	General transcription factor II-I OS=Homo sapiens GN=GTF2I PE=1 SV=2 - [GTF2I_HUMAN]	0.60	1	1	1	1					1.01	1.33	1.06	1.38	998	112.3	6.39
Q9UHL9	General transcription factor II-I repeat domain-containing protein 1 OS=Homo sapiens GN=GTF2IRD1 PE=1 SV=1 - [GT2D1_HUMAN]	5.11	1	3	3	3	1.22	1.06	1.22	0.69	1.03	0.91	1.08	0.95	959	106.0	6.87
Q6EKJ0	General transcription factor II-I repeat domain-containing protein 2B OS=Homo sapiens GN=GTF2IRD2B PE=1 SV=1 - [GTD2B_HUMAN]	1.26	2	1	1	1					0.79	1.00	0.54	0.68	949	107.2	5.85
P32780	General transcription factor IIH subunit 1 OS=Homo sapiens GN=GTF2H1 PE=1 SV=1 - [TF2H1_HUMAN]	3.10	1	1	1	1					3.06	2.91	3.07	2.93	548	62.0	8.66
Q13888	General transcription factor IIH subunit 2 OS=Homo sapiens GN=GTF2H2 PE=1 SV=1 - [TF2H2_HUMAN]	1.77	2	1	1	1					0.58	0.90	0.58	0.89	395	44.4	6.76
O60763	General vesicular transport factor p115 OS=Homo sapiens GN=USO1 PE=1 SV=2 - [USO1_HUMAN]	1.77	1	1	1	5	1.33	1.41	1.53	1.63					962	107.8	4.91
Q14687	Genetic suppressor element 1 OS=Homo sapiens GN=GSE1 PE=1 SV=3 - [GSE1_HUMAN]	0.74	1	1	1	1	0.94	1.52	0.79	1.28					1217	136.1	7.74
Q92696	Geranylgeranyl transferase type-2 subunit alpha OS=Homo sapiens GN=RABGGTA PE=1 SV=2 - [PGTA_HUMAN]	5.29	1	1	1	2					1.17	1.07	1.16	1.06	567	65.0	5.67
Q96IK5	Germ cell-less protein-like 1 OS=Homo sapiens GN=GMCL1 PE=1 SV=1 - [GMCL1_HUMAN]	2.72	1	1	1	2					0.98	0.99	0.91	0.93	515	58.6	7.44
Q8N6F7	Germinal center B-cell-expressed transcript 2 protein OS=Homo sapiens GN=GCET2 PE=1 SV=1 - [GCET2_HUMAN]	11.80	1	1	1	6	1.09	0.99	1.15	1.05	1.54	1.50	1.93	1.88	178	21.0	6.52
Q8N2G8	GH3 domain-containing protein OS=Homo sapiens GN=GHDC PE=1 SV=2 - [GHDC_HUMAN]	3.77	1	1	1	1					1.10	1.07	0.81	0.79	530	57.5	7.88
Q3V6T2	Girdin OS=Homo sapiens GN=CCDC88A PE=1 SV=2 - [GRDN_HUMAN]	1.87	4	2	4	99	0.59	0.73	0.79	0.90					1871	215.9	6.21
P60983	Glia maturation factor beta OS=Homo sapiens GN=GMFB PE=1 SV=2 - [GMFB_HUMAN]	38.03	1	3	4	14					1.18	1.14	1.08	1.13	142	16.7	5.29
O60234	Glia maturation factor gamma OS=Homo sapiens GN=GMFG PE=1 SV=1 - [GMFG_HUMAN]	15.49	1	1	2	9					1.33	1.42	1.24	1.32	142	16.8	5.26
P07093	Glia-derived nexin OS=Homo sapiens GN=SERPINE2 PE=1 SV=1 - [GDN_HUMAN]	14.32	1	5	5	13	1.35	1.38	1.35	1.43	1.10	1.14	1.19	1.24	398	44.0	9.29
Q6ZMI3	Gliomedin OS=Homo sapiens GN=GLDN PE=2 SV=1 - [GLDN_HUMAN]	4.36	1	1	1	4	1.08	1.33	0.89	0.84					551	58.9	8.02
Q92990	Glomulin OS=Homo sapiens GN=GLMN PE=1 SV=2 - [GLMN_HUMAN]	1.68	1	1	1	2					1.10	1.11	1.12	1.13	594	68.2	5.33
Q06210	Glucosamine--fructose-6-phosphate aminotransferase [isomerizing] 1 OS=Homo sapiens GN=GFPT1 PE=1 SV=3 - [GFPT1_HUMAN]	2.43	1	1	2	2					####	###	###	####	699	78.8	7.11
Q8TDQ7	Glucosamine-6-phosphate isomerase 2 OS=Homo sapiens GN=GNPDA2 PE=1 SV=1 - [GNPI2_HUMAN]	10.87	1	1	1	1	0.78	1.01	0.64	0.84					276	31.1	6.95

Q6PCE3	Glucose 1,6-bisphosphate synthase OS=Homo sapiens GN=PGM2L1 PE=1 SV=3 - [PGM2L_HUMAN]	3.22	1	1	1	1	0.68	0.43	0.77	0.50					622	70.4	7.15
Q9NQR9	Glucose-6-phosphatase 2 OS=Homo sapiens GN=G6PC2 PE=1 SV=1 - [G6PC2_HUMAN]	6.20	1	1	1	1					0.82	0.96	0.80	0.94	355	40.6	8.32
P11413	Glucose-6-phosphate 1-dehydrogenase OS=Homo sapiens GN=G6PD PE=1 SV=4 - [G6PD_HUMAN]	7.57	1	2	2	2	0.72	0.82	0.62	0.71	0.66	0.68	0.83	0.86	515	59.2	6.84
P06744	Glucose-6-phosphate isomerase OS=Homo sapiens GN=GPI PE=1 SV=4 - [G6PI_HUMAN]	9.86	1	4	4	11	0.93	0.86	0.87	0.82					558	63.1	8.32
P14314	Glucosidase 2 subunit beta OS=Homo sapiens GN=PRKCSH PE=1 SV=2 - [GLU2B_HUMAN]	12.31	1	4	5	10	0.78	0.70	0.71	0.64	1.32	1.21	1.29	1.18	528	59.4	4.41
Q4G148	Glucoside xylosyltransferase 1 OS=Homo sapiens GN=GXYLT1 PE=1 SV=2 - [GXLT1_HUMAN]	3.64	1	1	1	2	0.99	1.04	1.02	1.08					440	50.5	8.65
P04062	Glucosylceramidase OS=Homo sapiens GN=GBA PE=1 SV=3 - [GLCM_HUMAN]	1.12	1	1	1	1	1.20	1.21	1.27	1.28					536	59.7	7.61
Q8TCU5	Glutamate [NMDA] receptor subunit 3A OS=Homo sapiens GN=GRIN3A PE=1 SV=2 - [NMD3A_HUMAN]	2.60	1	1	1	1					1.16	0.87	1.13	0.85	1115	125.4	7.59
Q12879	Glutamate [NMDA] receptor subunit epsilon-1 OS=Homo sapiens GN=GRIN2A PE=1 SV=1 - [NMDE1_HUMAN]	1.84	1	3	3	3					0.50	0.48	0.94	0.91	1464	165.2	7.11
Q13224	Glutamate [NMDA] receptor subunit epsilon-2 OS=Homo sapiens GN=GRIN2B PE=1 SV=3 - [NMDE2_HUMAN]	1.21	1	1	1	6	0.78	0.77	0.99	0.96	1.12	1.16	1.18	1.22	1484	166.3	6.93
O15399	Glutamate [NMDA] receptor subunit epsilon-4 OS=Homo sapiens GN=GRIN2D PE=2 SV=2 - [NMDE4_HUMAN]	0.90	1	1	1	2					1.21	1.10	1.07	0.97	1336	143.7	8.35
Q05586	Glutamate [NMDA] receptor subunit zeta-1 OS=Homo sapiens GN=GRIN1 PE=1 SV=1 - [NMDZ1_HUMAN]	1.39	1	1	1	1					2.12	1.59	0.93	0.69	938	105.3	8.88
Q04609	Glutamate carboxypeptidase 2 OS=Homo sapiens GN=FOLH1 PE=1 SV=1 - [FOLH1_HUMAN]	6.40	2	2	2	2	0.43	0.22	0.89	0.46	1.28	1.72	1.25	1.67	750	84.3	6.98
Q05329	Glutamate decarboxylase 2 OS=Homo sapiens GN=GAD2 PE=1 SV=1 - [DCE2_HUMAN]	2.39	1	1	1	2	0.81	0.86	0.84	0.90					585	65.4	6.90
P00367	Glutamate dehydrogenase 1, mitochondrial OS=Homo sapiens GN=GLUD1 PE=1 SV=2 - [DHE3_HUMAN]	2.87	1	1	1	1	0.95	0.98	0.99	1.03					558	61.4	7.80
P49448	Glutamate dehydrogenase 2, mitochondrial OS=Homo sapiens GN=GLUD2 PE=1 SV=2 - [DHE4_HUMAN]	5.56	1	2	2	2					0.89	1.10	0.57	0.77	558	61.4	8.46
P42262	Glutamate receptor 2 OS=Homo sapiens GN=GRIA2 PE=1 SV=3 - [GRIA2_HUMAN]	3.96	1	2	2	4					1.14	1.35	0.91	1.07	883	98.8	7.58
P48058	Glutamate receptor 4 OS=Homo sapiens GN=GRIA4 PE=2 SV=2 - [GRIA4_HUMAN]	2.88	1	2	2	3	1.70		0.82		1.79	1.18	1.06	0.70	902	100.8	8.10
O43424	Glutamate receptor delta-2 subunit OS=Homo sapiens GN=GRID2 PE=2 SV=2 - [GRID2_HUMAN]	1.69	1	1	1	1	0.55	0.64	0.64	0.75					1007	113.3	6.07
Q9Y3R0	Glutamate receptor-interacting protein 1 OS=Homo sapiens GN=GRIP1 PE=1 SV=3 - [GRIP1_HUMAN]	1.86	1	1	1	1	0.31	0.32	0.27	0.28					1128	122.3	6.47
Q9C0E4	Glutamate receptor-interacting protein 2 OS=Homo sapiens GN=GRIP2 PE=1 SV=3 - [GRIP2_HUMAN]	1.82	1	1	1	3	0.93	0.83	0.78	0.76					1043	112.4	6.49
P39086	Glutamate receptor, ionotropic kainate 1 OS=Homo sapiens GN=GRIK1 PE=1 SV=1 - [GRIK1_HUMAN]	0.87	1	2	2	2					1.44	1.25	1.30	0.94	918	103.9	7.08
Q16099	Glutamate receptor, ionotropic kainate 4 OS=Homo sapiens GN=GRIK4 PE=2 SV=2 - [GRIK4_HUMAN]	1.67	1	1	1	1					0.37	0.58	0.64	1.00	956	107.2	6.73
Q9UI32	Glutaminase liver isoform, mitochondrial OS=Homo sapiens GN=GLS2 PE=1 SV=2 - [GLSL_HUMAN]	2.16	1	2	2	27					1.47	1.15	0.57	0.45	602	66.3	7.30
Q2TAL8	Glutamine-rich protein 1 OS=Homo sapiens GN=QRICH1 PE=1 SV=1 - [QRIC1_HUMAN]	1.42	1	1	1	1					1.16	1.17	1.18	1.20	776	86.4	5.87
Q9H0J4	Glutamine-rich protein 2 OS=Homo sapiens GN=QRICH2 PE=2 SV=1 - [QRIC2_HUMAN]	1.62	1	1	1	1						0.93		0.52	1663	180.7	6.73
Q16769	Glutaminyl-peptide cyclotransferase OS=Homo sapiens GN=QPCT PE=1 SV=1 - [QPCT_HUMAN]	3.88	1	1	1	2	0.86	0.94	0.80	0.89					361	40.9	6.61

Q07075	Glutamyl aminopeptidase OS=Homo sapiens GN=ENPEP PE=1 SV=3 - [AMPE_HUMAN]	2.61	1	2	2	3	1.13	1.39	0.96	1.17					957	109.2	5.47
A8MXD5	Glutaredoxin domain-containing cysteine-rich protein 1 OS=Homo sapiens GN=GRXCR1 PE=1 SV=1 - [GRCR1_HUMAN]	4.48	1	1	1	1	1.09	1.23	1.19	1.34					290	32.3	7.52
P35754	Glutaredoxin-1 OS=Homo sapiens GN=GLRX PE=1 SV=2 - [GLRX1_HUMAN]	30.19	1	2	2	6					1.00	0.95	0.93	0.89	106	11.8	8.09
P22352	Glutathione peroxidase 3 OS=Homo sapiens GN=GPX3 PE=1 SV=2 - [GPX3_HUMAN]	37.61	1	8	8	160	1.05	1.04	1.03	1.01	1.14	1.20	1.09	1.16	226	25.5	8.13
Q96SL4	Glutathione peroxidase 7 OS=Homo sapiens GN=GPX7 PE=1 SV=1 - [GPX7_HUMAN]	6.42	9	1	2	3					0.89	1.04	0.87	1.02	187	21.0	8.27
P00390	Glutathione reductase, mitochondrial OS=Homo sapiens GN=GSR PE=1 SV=2 - [GSHR_HUMAN]	2.49	1	1	1	2	0.88	0.87	0.87	0.85					522	56.2	8.50
P28161	Glutathione S-transferase Mu 2 OS=Homo sapiens GN=GSTM2 PE=1 SV=2 - [GSTM2_HUMAN]	7.80	1	1	1	3					1.18	1.16	1.01	1.00	218	25.7	6.37
P78417	Glutathione S-transferase omega-1 OS=Homo sapiens GN=GSTO1 PE=1 SV=2 - [GSTO1_HUMAN]	15.77	1	4	4	15	0.63	0.66	0.64	0.68	0.79	0.92	0.88	1.01	241	27.5	6.60
P09211	Glutathione S-transferase P OS=Homo sapiens GN=GSTP1 PE=1 SV=2 - [GSTP1_HUMAN]	38.57	1	5	5	19	0.56	0.68	0.47	0.57	1.02	1.08	0.92	0.93	210	23.3	5.64
P48637	Glutathione synthetase OS=Homo sapiens GN=GSS PE=1 SV=1 - [GSHB_HUMAN]	3.16	1	2	2	5	0.90	0.89	0.90	0.89					474	52.4	5.92
P04406	Glyceraldehyde-3-phosphate dehydrogenase OS=Homo sapiens GN=GAPDH PE=1 SV=3 - [G3P_HUMAN]	37.61	2	11	11	50	0.43	0.43	0.45	0.44	0.69	0.77	0.72	0.78	335	36.0	8.46
Q6NUI2	Glycerol-3-phosphate acyltransferase 2, mitochondrial OS=Homo sapiens GN=GPAT2 PE=2 SV=2 - [GPAT2_HUMAN]	0.88	1	1	1	1	1.89	1.60	1.90	1.61					795	87.8	7.61
P23378	Glycine dehydrogenase [decarboxylating], mitochondrial OS=Homo sapiens GN=GLDC PE=1 SV=2 - [GCSP_HUMAN]	0.69	1	1	1	1	1.03	0.97	1.08	1.01					1020	112.7	7.11
Q14749	Glycine N-methyltransferase OS=Homo sapiens GN=GNMT PE=1 SV=3 - [GNMT_HUMAN]	9.15	1	1	1	1	0.82	0.94	0.84	0.97					295	32.7	7.02
P23415	Glycine receptor subunit alpha-1 OS=Homo sapiens GN=GLRA1 PE=1 SV=2 - [GLRA1_HUMAN]	7.00	1	2	2	5	1.33	1.11	1.19	1.00	0.75	0.93	0.92	1.12	457	52.6	8.79
Q5JXX5	Glycine receptor subunit alpha-4 OS=Homo sapiens GN=GLRA4 PE=2 SV=3 - [GLRA4_HUMAN]	4.08	1	1	1	2	1.10	1.04	1.24	1.17					417	47.7	8.24
P54840	Glycogen [starch] synthase, liver OS=Homo sapiens GN=GYS2 PE=1 SV=2 - [GYS2_HUMAN]	5.55	1	2	2	4	1.37	1.39	1.11	1.12	1.22	1.83	1.55	2.33	703	80.9	6.83
P06737	Glycogen phosphorylase, liver form OS=Homo sapiens GN=PYGL PE=1 SV=4 - [PYGL_HUMAN]	8.26	1	5	5	8	0.88	0.87	0.92	0.89	0.98	1.00	1.05	1.08	847	97.1	7.17
P46976	Glycogenin-1 OS=Homo sapiens GN=GYG1 PE=1 SV=4 - [GLYG_HUMAN]	10.00	1	2	2	2	1.73	1.33	2.82	2.18	0.77	0.75	0.77	0.75	350	39.4	5.53
O15488	Glycogenin-2 OS=Homo sapiens GN=GYG2 PE=1 SV=2 - [GLYG2_HUMAN]	4.59	1	1	1	2	0.68	0.70	0.47	0.48	1.07	0.98	1.14	1.05	501	55.1	5.10
Q9NZD2	Glycolipid transfer protein OS=Homo sapiens GN=GLTP PE=1 SV=3 - [GLTP_HUMAN]	5.74	1	1	1	2	0.80	0.63	0.86	0.67					209	23.8	7.39
P01215	Glycoprotein hormones alpha chain OS=Homo sapiens GN=CGA PE=1 SV=1 - [GLHA_HUMAN]	12.07	1	2	2	2					2.92	2.98	2.77	2.82	116	13.1	8.19
Q99445	Glycosyl-phosphatidylinositol-anchored molecule-like protein OS=Homo sapiens GN=GML PE=2 SV=1 - [GML_HUMAN]	9.49	1	1	1	1	0.35	0.28	0.41	0.32					158	17.7	6.48
Q96MS3	Glycosyltransferase 1 domain-containing protein 1 OS=Homo sapiens GN=GLT1D1 PE=2 SV=2 - [GL1D1_HUMAN]	2.89	1	1	1	1	0.99	1.17	1.00	1.19					346	38.5	6.47
Q9HC38	Glyoxalase domain-containing protein 4 OS=Homo sapiens GN=GLOD4 PE=1 SV=1 - [GLOD4_HUMAN]	3.19	1	1	1	1					1.09	1.40	0.90	1.16	313	34.8	5.60
Q8N158	Glypican-2 OS=Homo sapiens GN=GPC2 PE=1 SV=1 - [GPC2_HUMAN]	2.42	1	1	1	1					0.88	0.93	0.84	0.89	579	62.8	8.00
O75487	Glypican-4 OS=Homo sapiens GN=GPC4 PE=1 SV=4 - [GPC4_HUMAN]	4.32	1	2	2	4		1.26		0.73	1.18	1.49	1.22	1.55	556	62.4	6.68

Q92896	Golgi apparatus protein 1 OS=Homo sapiens GN=GLG1 PE=1 SV=2 - [GSLG1_HUMAN]	6.36	1	2	3	17	1.04	0.83	1.14	0.92	0.95	1.31	1.04	1.19	1179	134.5	6.90
Q8NBJ4	Golgi membrane protein 1 OS=Homo sapiens GN=GOLM1 PE=1 SV=1 - [GOLM1_HUMAN]	4.74	1	2	2	3	0.75	0.78	0.84	0.87	1.00	0.76	0.95	0.73	401	45.3	4.97
Q9H4A6	Golgi phosphoprotein 3 OS=Homo sapiens GN=GOLPH3 PE=1 SV=1 - [GOLP3_HUMAN]	2.01	1	1	1	1					0.79	0.75	0.86	0.83	298	33.8	6.44
Q9BQQ3	Golgi reassembly-stacking protein 1 OS=Homo sapiens GN=GORASP1 PE=1 SV=3 - [GORS1_HUMAN]	6.14	1	1	1	1					0.82	0.92	0.92	1.02	440	46.5	4.50
Q9H4G4	Golgi-associated plant pathogenesis-related protein 1 OS=Homo sapiens GN=GLIPR2 PE=1 SV=3 - [GAPR1_HUMAN]	16.88	1	2	2	8	0.89	0.73	0.75	0.61	1.29	1.35	1.23	1.29	154	17.2	9.41
Q92805	Golgin subfamily A member 1 OS=Homo sapiens GN=GOLGA1 PE=1 SV=3 - [GOGA1_HUMAN]	4.95	1	2	2	2	0.80	0.85	0.91	0.98	1.07	0.86	1.18	0.94	767	88.1	5.27
Q08379	Golgin subfamily A member 2 OS=Homo sapiens GN=GOLGA2 PE=1 SV=3 - [GOGA2_HUMAN]	1.70	1	1	1	1	0.76	0.82	1.00	1.08					1002	113.0	5.02
Q8N8X6	Golgin subfamily A member 2-like protein 4 OS=Homo sapiens PE=2 SV=1 - [GG2L4_HUMAN]	9.76	1	1	1	1	0.93	0.97	0.81	0.85					164	19.8	8.84
Q13439	Golgin subfamily A member 4 OS=Homo sapiens GN=GOLGA4 PE=1 SV=1 - [GOGA4_HUMAN]	2.74	1	2	3	43	1.45	1.39	1.37	1.27	1.11	1.18	1.00	1.11	2230	261.0	5.39
Q8N7Z2	Golgin subfamily A member 6-like protein 1 OS=Homo sapiens GN=GOLGA6L1 PE=2 SV=2 - [GG6L1_HUMAN]	4.83	1	1	2	4	0.69	0.23	0.48	0.16					621	77.0	5.36
Q14789	Golgin subfamily B member 1 OS=Homo sapiens GN=GOLGB1 PE=1 SV=2 - [GOGB1_HUMAN]	2.73	3	4	6	8	2.91	3.37	1.23	1.42	0.62	0.68	0.78	0.86	3259	375.8	5.00
Q3T8J9	GON-4-like protein OS=Homo sapiens GN=GON4L PE=1 SV=1 - [GON4L_HUMAN]	2.19	1	2	2	2			0.78		1.09	0.13	0.90	0.11	2241	248.5	5.01
Q75T13	GPI inositol-deacylase OS=Homo sapiens GN=PGAP1 PE=2 SV=1 - [PGAP1_HUMAN]	1.95	1	1	1	7	0.90	1.13	0.80	1.02	1.13	1.00	0.97	1.00	922	105.3	9.01
Q9H3S5	GPI mannosyltransferase 1 OS=Homo sapiens GN=PIGM PE=1 SV=1 - [PIGM_HUMAN]	5.44	9	1	2	3	1.94	2.20	1.65	1.88	0.95	0.99	0.73	0.77	423	49.4	9.11
Q92521	GPI mannosyltransferase 3 OS=Homo sapiens GN=PIGB PE=2 SV=1 - [PIGB_HUMAN]	1.99	1	1	1	1	0.89	0.93	0.82	0.87					554	65.0	9.29
Q86VD9	GPI mannosyltransferase 4 OS=Homo sapiens GN=PIGZ PE=2 SV=4 - [PIGZ_HUMAN]	2.42	1	1	1	1					0.84	1.09	1.67	2.15	579	63.4	8.34
Q969N2	GPI transamidase component PIG-T OS=Homo sapiens GN=PIGT PE=1 SV=1 - [PIGT_HUMAN]	2.08	1	1	1	1					1.05	1.06	0.95	0.97	578	65.7	8.38
Q6ISB3	Grainyhead-like protein 2 homolog OS=Homo sapiens GN=GRHL2 PE=1 SV=1 - [GRHL2_HUMAN]	3.36	1	2	2	2					1.09	1.03	1.24	1.17	625	71.1	6.44
Q96CP6	GRAM domain-containing protein 1A OS=Homo sapiens GN=GRAMD1A PE=1 SV=2 - [GRM1A_HUMAN]	2.07	1	1	1	1	1.71	1.23	1.49	1.07					724	80.6	6.74
P28676	Grancalcin OS=Homo sapiens GN=GCA PE=1 SV=2 - [GRAN_HUMAN]	4.15	1	1	1	1					1.05	1.08	1.08	1.10	217	24.0	5.21
P28799	Granulins OS=Homo sapiens GN=GRN PE=1 SV=2 - [GRN_HUMAN]	24.62	1	9	9	28	1.05	1.06	1.18	1.20	1.15	1.16	1.16	1.15	593	63.5	6.83
P15509	Granulocyte-macrophage colony-stimulating factor receptor subunit alpha OS=Homo sapiens GN=CSF2RA PE=1 SV=1 - [CSF2R_HUMAN]	8.50	1	2	2	2	1.16	1.21	1.17	1.23	1.05	1.09	1.03	1.07	400	46.2	7.75
P51124	Granzyme M OS=Homo sapiens GN=GZMM PE=1 SV=2 - [GRAM_HUMAN]	5.45	1	1	1	1					0.85	0.87	0.69	0.70	257	27.5	10.13
O75791	GRB2-related adapter protein 2 OS=Homo sapiens GN=GRAP2 PE=1 SV=1 - [GRAP2_HUMAN]	19.09	3	4	5	9	0.86	0.50	0.73	0.42	1.26	1.29	1.22	1.21	330	37.9	6.95
Q13588	GRB2-related adapter protein OS=Homo sapiens GN=GRAP PE=1 SV=1 - [GRAP_HUMAN]	5.99	3	1	2	3	1.01	0.99	0.99	0.98					217	25.3	7.03
Q8IWJ2	GRIP and coiled-coil domain-containing protein 2 OS=Homo sapiens GN=GCC2 PE=1 SV=4 - [GCC2_HUMAN]	4.04	4	4	6	6	0.93	0.98	0.86	0.91	1.35	1.43	1.23	1.30	1684	195.8	5.14
P53816	Group XVI phospholipase A1/A2 OS=Homo sapiens GN=PLA2G16 PE=1 SV=2 - [PAG16_HUMAN]	18.52	1	1	1	1	0.81	0.66	0.65	0.53					162	17.9	7.99

P62993	Growth factor receptor-bound protein 2 OS=Homo sapiens GN=GRB2 PE=1 SV=1 - [GRB2_HUMAN]	13.36	1	3	3	7					0.85	0.86	0.88	0.90	217	25.2	6.32
Q92847	Growth hormone secretagogue receptor type 1 OS=Homo sapiens GN=GHSR PE=1 SV=1 - [GHSR_HUMAN]	3.83	1	1	1	2					0.22	0.22	0.69	0.67	366	41.3	8.31
Q9UK05	Growth/differentiation factor 2 OS=Homo sapiens GN=GDF2 PE=1 SV=1 - [GDF2_HUMAN]	3.96	1	1	1	1	0.60	0.81	0.68	0.93					429	47.3	6.48
O14793	Growth/differentiation factor 8 OS=Homo sapiens GN=MSTN PE=1 SV=1 - [GDF8_HUMAN]	8.53	7	2	3	12					0.80	0.79	0.89	0.88	375	42.7	6.76
Q9H4S2	GS homeobox 1 OS=Homo sapiens GN=GSX1 PE=2 SV=1 - [GSX1_HUMAN]	6.82	1	1	1	1					1.12	1.24	1.10	1.21	264	27.9	8.92
P62826	GTP-binding nuclear protein Ran OS=Homo sapiens GN=RAN PE=1 SV=3 - [RAN_HUMAN]	10.65	1	2	2	3	0.87	0.84	0.88	0.86	0.83	1.02	1.10	1.37	216	24.4	7.49
Q15382	GTP-binding protein Rheb OS=Homo sapiens GN=RHEB PE=1 SV=1 - [RHEB_HUMAN]	5.43	1	1	1	2	0.96	0.93	0.97	0.94					184	20.5	5.92
Q9Y6B6	GTP-binding protein SAR1b OS=Homo sapiens GN=SAR1B PE=1 SV=1 - [SAR1B_HUMAN]	8.08	1	1	1	1	1.24	1.51	0.98	1.19					198	22.4	6.11
Q9UIJ7	GTP:AMP phosphotransferase, mitochondrial OS=Homo sapiens GN=AK3 PE=1 SV=4 - [KAD3_HUMAN]	3.52	1	1	1	1					1.33	1.54	0.97	1.12	227	25.5	9.16
Q14C86	GTPase-activating protein and VPS9 domain-containing protein 1 OS=Homo sapiens GN=GAPVD1 PE=1 SV=2 - [GAPD1_HUMAN]	1.89	1	1	1	1	2.27	1.05	2.48	1.15					1478	164.9	5.22
Q9Y2T3	Guanine deaminase OS=Homo sapiens GN=GDA PE=1 SV=1 - [GUAD_HUMAN]	6.39	1	3	3	6	0.97	1.13	1.06	1.25	0.88	0.11	0.87	0.08	454	51.0	5.68
P04899	Guanine nucleotide-binding protein G(i) subunit alpha-2 OS=Homo sapiens GN=GNAI2 PE=1 SV=3 - [GNAI2_HUMAN]	3.66	1	1	1	2	0.78	0.88	0.80	0.91					355	40.4	5.54
P62873	Guanine nucleotide-binding protein G(I)/G(S)/G(T) subunit beta-1 OS=Homo sapiens GN=GNB1 PE=1 SV=3 - [GBB1_HUMAN]	5.88	3	2	2	5	0.79	0.93	0.79	0.98	0.76	0.83	0.75	0.83	340	37.4	6.00
P50148	Guanine nucleotide-binding protein G(q) subunit alpha OS=Homo sapiens GN=GNAQ PE=1 SV=4 - [GNAQ_HUMAN]	5.29	1	1	1	1					0.78	0.88	0.98	1.11	359	42.1	5.68
P19086	Guanine nucleotide-binding protein G(z) subunit alpha OS=Homo sapiens GN=GNAZ PE=2 SV=3 - [GNAZ_HUMAN]	3.38	1	1	1	1					0.81	0.80	0.82	0.81	355	40.9	7.61
Q03113	Guanine nucleotide-binding protein subunit alpha-12 OS=Homo sapiens GN=GNA12 PE=1 SV=4 - [GNA12_HUMAN]	5.77	1	2	2	2	0.76	0.89	0.78	0.92	0.84	1.05	0.87	1.08	381	44.3	9.83
Q14344	Guanine nucleotide-binding protein subunit alpha-13 OS=Homo sapiens GN=GNA13 PE=1 SV=2 - [GNA13_HUMAN]	3.98	1	1	1	1					0.71	0.69	0.88	0.85	377	44.0	8.00
P30679	Guanine nucleotide-binding protein subunit alpha-15 OS=Homo sapiens GN=GNA15 PE=2 SV=2 - [GNA15_HUMAN]	2.67	1	1	1	1	0.65	0.64	0.70	0.69					374	43.5	8.51
Q9HAV0	Guanine nucleotide-binding protein subunit beta-4 OS=Homo sapiens GN=GNB4 PE=1 SV=3 - [GBB4_HUMAN]	6.76	1	1	1	1					0.97	0.93	1.48	1.43	340	37.5	6.00
P36915	Guanine nucleotide-binding protein-like 1 OS=Homo sapiens GN=GNL1 PE=1 SV=2 - [GNL1_HUMAN]	2.80	1	1	1	2	3.04	4.21	3.14	4.36					607	68.6	5.80
Q9BVP2	Guanine nucleotide-binding protein-like 3 OS=Homo sapiens GN=GNL3 PE=1 SV=2 - [GNL3_HUMAN]	2.73	1	1	1	1					0.39	0.15	1.33	0.52	549	62.0	9.16
Q9NVN8	Guanine nucleotide-binding protein-like 3-like protein OS=Homo sapiens GN=GNL3L PE=1 SV=1 - [GNL3L_HUMAN]	2.92	1	1	1	2	0.80	0.91	0.70	0.80	0.81	1.25	0.56	0.87	582	65.5	8.44
Q02108	Guanylate cyclase soluble subunit alpha-3 OS=Homo sapiens GN=GUCY1A3 PE=1 SV=2 - [GCYA3_HUMAN]	7.25	1	2	2	2	0.30	0.32	0.34	0.36	0.77	0.74	0.86	0.83	690	77.4	7.11
Q16774	Guanylate kinase OS=Homo sapiens GN=GUK1 PE=1 SV=2 - [KGUA_HUMAN]	3.05	1	1	1	1					0.75	0.07	1.51	0.14	197	21.7	6.55
Q96PP9	Guanylate-binding protein 4 OS=Homo sapiens GN=GBP4 PE=1 SV=2 - [GBP4_HUMAN]	1.09	1	1	1	1					0.63	1.01	1.56	2.51	640	73.1	6.02
Q96PP8	Guanylate-binding protein 5 OS=Homo sapiens GN=GBP5 PE=2 SV=1 - [GBP5_HUMAN]	2.39	1	1	1	1					0.92	0.89	0.87	0.84	586	66.6	5.55
Q02747	Guanylin OS=Homo sapiens GN=GUCA2A PE=1 SV=2 - [GUC2A_HUMAN]	9.57	1	1	1	2					0.75	0.74	0.85	0.83	115	12.4	4.59

Q9NXP7	Gypsy retrotransposon integrase-like protein 1 OS=Homo sapiens GN=GIN1 PE=2 SV=3 - [GIN1_HUMAN]	8.81	1	2	2	12	0.71	1.08	0.64	0.98	0.82	0.74	0.99	0.90	522	59.8	7.97
Q92574	Hamartin OS=Homo sapiens GN=TSC1 PE=1 SV=2 - [TSC1_HUMAN]	0.52	1	1	1	1	0.34	0.38	1.25	1.42					1164	129.7	6.47
P00738	Haptoglobin OS=Homo sapiens GN=HP PE=1 SV=1 - [HPT_HUMAN]	69.70	3	23	43	6211	1.19	1.18	1.20	1.18	1.03	1.05	0.99	1.03	406	45.2	6.58
P00739	Haptoglobin-related protein OS=Homo sapiens GN=HPR PE=1 SV=2 - [HPTR_HUMAN]	68.68	3	10	31	3042	1.04	1.07	1.07	1.12	1.12	1.12	1.01	1.03	348	39.0	7.09
Q68CZ6	HAUS augmin-like complex subunit 3 OS=Homo sapiens GN=HAUS3 PE=1 SV=1 - [HAUS3_HUMAN]	1.66	1	1	1	2					1.24	0.78	1.17	0.73	603	69.6	5.55
Q7Z4H7	HAUS augmin-like complex subunit 6 OS=Homo sapiens GN=HAUS6 PE=1 SV=2 - [HAUS6_HUMAN]	1.47	1	1	1	1					0.89	1.09	0.89	1.09	955	108.6	6.47
Q9BT25	HAUS augmin-like complex subunit 8 OS=Homo sapiens GN=HAUS8 PE=1 SV=3 - [HAUS8_HUMAN]	7.32	1	1	2	3					0.79	0.69	0.94	0.82	410	44.8	7.06
Q9Y450	HBS1-like protein OS=Homo sapiens GN=HBS1L PE=1 SV=1 - [HBS1L_HUMAN]	1.61	1	1	1	1					0.91	0.95	0.88	0.91	684	75.4	6.61
O00165	HCLS1-associated protein X-1 OS=Homo sapiens GN=HAX1 PE=1 SV=2 - [HAX1_HUMAN]	11.47	1	1	1	1	0.58	0.31	0.89	0.49					279	31.6	4.92
Q53T59	HCLS1-binding protein 3 OS=Homo sapiens GN=HS1BP3 PE=1 SV=1 - [H1BP3_HUMAN]	4.85	1	1	1	1					1.10	1.26	1.57	1.79	392	42.8	5.01
Q9H583	HEAT repeat-containing protein 1 OS=Homo sapiens GN=HEATR1 PE=1 SV=3 - [HEAT1_HUMAN]	1.96	1	2	2	2	1.03	0.80	1.05	0.82					2144	242.2	6.54
Q86Y56	HEAT repeat-containing protein 2 OS=Homo sapiens GN=HEATR2 PE=1 SV=4 - [HEAT2_HUMAN]	1.29	1	1	1	1					0.69	0.66	0.84	0.80	855	93.5	6.42
Q86WZ0	HEAT repeat-containing protein 4 OS=Homo sapiens GN=HEATR4 PE=1 SV=2 - [HEAT4_HUMAN]	4.09	1	2	2	2					1.04	0.91	0.75	0.66	1026	117.1	9.11
Q86XA9	HEAT repeat-containing protein 5A OS=Homo sapiens GN=HEATR5A PE=1 SV=2 - [HTR5A_HUMAN]	1.91	1	2	2	2	1.51	1.46	1.21	1.17	1.05	1.04	1.09	1.07	2040	221.9	6.58
Q8NDA8	HEAT repeat-containing protein 7A OS=Homo sapiens GN=HEATR7A PE=1 SV=3 - [HTR7A_HUMAN]	0.61	1	1	1	1					4.11	2.82	4.12	2.82	1641	181.1	6.89
A6NES4	HEAT repeat-containing protein 7B1 OS=Homo sapiens GN=HEATR7B1 PE=4 SV=3 - [HTRB1_HUMAN]	0.64	1	1	1	2	0.98	0.71	1.03	0.75					1706	193.1	6.68
Q7Z745	HEAT repeat-containing protein 7B2 OS=Homo sapiens GN=HEATR7B2 PE=2 SV=3 - [HTRB2_HUMAN]	1.83	4	1	2	4	0.63	0.99	0.61	0.96					1585	180.7	6.28
Q68CQ1	HEAT repeat-containing protein 8 OS=Homo sapiens GN=HEATR8 PE=2 SV=4 - [HEAT8_HUMAN]	0.91	1	1	1	2	0.69	0.86	0.84	1.05					1323	145.6	6.96
P34931	Heat shock 70 kDa protein 1-like OS=Homo sapiens GN=HSPA1L PE=1 SV=2 - [HS71L_HUMAN]	12.79	1	2	8	21	1.07	1.04	0.93	0.91					641	70.3	6.02
P08107	Heat shock 70 kDa protein 1A/1B OS=Homo sapiens GN=HSPA1A PE=1 SV=5 - [HSP71_HUMAN]	17.94	1	3	11	30	0.96	0.97	0.90	0.91	0.71	0.77	0.75	0.82	641	70.0	5.66
P17066	Heat shock 70 kDa protein 6 OS=Homo sapiens GN=HSPA6 PE=1 SV=2 - [HSP76_HUMAN]	11.04	1	1	6	19					0.58	0.51	1.04	0.92	643	71.0	6.14
P11142	Heat shock cognate 71 kDa protein OS=Homo sapiens GN=HSPA8 PE=1 SV=1 - [HSP7C_HUMAN]	19.97	1	5	12	30	0.71	0.68	0.73	0.71	1.10	1.06	1.11	1.06	646	70.9	5.52
Q92598	Heat shock protein 105 kDa OS=Homo sapiens GN=HSPH1 PE=1 SV=1 - [HS105_HUMAN]	1.98	1	1	1	1					1.07	1.05	1.10	1.08	858	96.8	5.39
Q12931	Heat shock protein 75 kDa, mitochondrial OS=Homo sapiens GN=TRAP1 PE=1 SV=3 - [TRAP1_HUMAN]	5.40	2	2	3	28	0.97	1.02	0.83	0.90					704	80.1	8.21
P04792	Heat shock protein beta-1 OS=Homo sapiens GN=HSPB1 PE=1 SV=2 - [HSPB1_HUMAN]	31.22	1	6	6	21	0.67	0.60	0.60	0.52	1.15	1.23	1.21	1.25	205	22.8	6.40
Q9Y547	Heat shock protein beta-11 OS=Homo sapiens GN=HSPB11 PE=1 SV=1 - [HSB11_HUMAN]	7.64	1	1	1	3					0.91	1.01	0.96	1.07	144	16.3	5.03
Q9BQS6	Heat shock protein beta-9 OS=Homo sapiens GN=HSPB9 PE=1 SV=1 - [HSPB9_HUMAN]	26.42	1	1	1	1	1.63	1.07	1.40	0.93					159	17.5	8.87

P54652	Heat shock-related 70 kDa protein 2 OS=Homo sapiens GN=HSPA2 PE=1 SV=1 - [HSP72_HUMAN]	11.74	1	1	7	18					0.33	0.26	0.81	0.65	639	70.0	5.74
Q96QV1	Hedgehog-interacting protein OS=Homo sapiens GN=HHIP PE=1 SV=3 - [HHIP_HUMAN]	2.43	1	1	2	60	0.68	0.80	0.87	1.02	1.85	2.21	1.74	2.03	700	78.8	7.90
Q9H910	Hematological and neurological expressed 1-like protein OS=Homo sapiens GN=HN1L PE=1 SV=1 - [HN1L_HUMAN]	8.95	1	1	1	1	0.74	1.03	0.82	1.15					190	20.1	9.26
P09601	Heme oxygenase 1 OS=Homo sapiens GN=HMOX1 PE=1 SV=1 - [HMOX1_HUMAN]	7.99	1	2	2	2	0.70	0.98	0.76	1.18					288	32.8	8.25
Q9Y5Z4	Heme-binding protein 2 OS=Homo sapiens GN=HEBP2 PE=1 SV=1 - [HEBP2_HUMAN]	16.59	1	2	2	4					0.94	1.01	0.92	1.00	205	22.9	4.63
Q96RW7	Hemicentin-1 OS=Homo sapiens GN=HMCN1 PE=1 SV=2 - [HMCN1_HUMAN]	0.82	1	2	2	3					0.79	0.68	0.89	0.77	5635	613.0	6.49
Q8NDA2	Hemicentin-2 OS=Homo sapiens GN=HMCN2 PE=2 SV=2 - [HMCN2_HUMAN]	1.11	5	3	4	7	1.24	0.77	1.05	0.66	0.88	0.76	0.96	0.79	5065	542.3	5.83
P69905	Hemoglobin subunit alpha OS=Homo sapiens GN=HBA1 PE=1 SV=2 - [HBA_HUMAN]	70.42	2	9	9	747	0.64	0.58	0.58	0.57	0.71	0.70	0.68	0.69	142	15.2	8.68
P68871	Hemoglobin subunit beta OS=Homo sapiens GN=HBB PE=1 SV=2 - [HBB_HUMAN]	89.80	2	8	14	1707	0.57	0.54	0.51	0.48	0.70	0.73	0.69	0.69	147	16.0	7.28
P02042	Hemoglobin subunit delta OS=Homo sapiens GN=HBD PE=1 SV=2 - [HBD_HUMAN]	89.80	2	8	14	761	0.56	0.57	0.52	0.52	0.85	0.84	0.86	0.84	147	16.0	8.05
P69891	Hemoglobin subunit gamma-1 OS=Homo sapiens GN=HBG1 PE=1 SV=2 - [HBG1_HUMAN]	50.34	2	1	7	75					1.15	1.01	1.33	1.16	147	16.1	7.20
P69892	Hemoglobin subunit gamma-2 OS=Homo sapiens GN=HBG2 PE=1 SV=2 - [HBG2_HUMAN]	50.34	2	1	7	81					0.59	0.61	0.69	0.66	147	16.1	7.20
P02790	Hemopexin OS=Homo sapiens GN=HPX PE=1 SV=2 - [HEMO_HUMAN]	81.39	2	44	44	5743	1.21	1.18	1.18	1.15	1.01	1.00	0.99	1.00	462	51.6	7.02
Q8IZT8	Heparan sulfate glucosamine 3-O-sulfotransferase 5 OS=Homo sapiens GN=HS3ST5 PE=1 SV=1 - [HS3S5_HUMAN]	5.20	1	2	2	3	1.09	1.07	1.10	1.09	1.00	0.62	1.16	0.72	346	40.4	9.79
Q9Y251	Heparanase OS=Homo sapiens GN=HPSE PE=1 SV=2 - [HPSE_HUMAN]	6.63	1	3	3	17	1.22	1.28	1.23	1.29	1.97	1.82	1.78	1.64	543	61.1	9.28
P05546	Heparin cofactor 2 OS=Homo sapiens GN=SERPIND1 PE=1 SV=3 - [HEP2_HUMAN]	65.93	1	29	29	643	1.12	1.17	1.12	1.14	1.05	1.06	1.01	1.01	499	57.0	6.90
P11150	Hepatic triacylglycerol lipase OS=Homo sapiens GN=LIPC PE=1 SV=3 - [LIPC_HUMAN]	3.41	1	1	1	2	0.86	1.18	0.89	1.22					499	55.9	9.04
Q04756	Hepatocyte growth factor activator OS=Homo sapiens GN=HGFAC PE=1 SV=1 - [HGFA_HUMAN]	31.45	1	16	16	123	1.23	1.20	1.21	1.21	1.12	1.09	1.09	1.09	655	70.6	7.24
P08581	Hepatocyte growth factor receptor OS=Homo sapiens GN=MET PE=1 SV=4 - [MET_HUMAN]	3.38	1	3	3	4					1.01	1.11	1.04	1.11	1390	155.4	7.33
P26927	Hepatocyte growth factor-like protein OS=Homo sapiens GN=MST1 PE=1 SV=2 - [HGFL_HUMAN]	47.96	2	26	26	214	1.18	1.18	1.14	1.14	1.05	1.09	1.04	1.07	711	80.3	7.68
O14964	Hepatocyte growth factor-regulated tyrosine kinase substrate OS=Homo sapiens GN=HGS PE=1 SV=1 - [HGS_HUMAN]	1.67	1	1	2	9					1.20	1.21	1.22	1.30	777	86.1	6.16
P55317	Hepatocyte nuclear factor 3-alpha OS=Homo sapiens GN=FOXA1 PE=1 SV=2 - [FOXA1_HUMAN]	3.81	1	1	1	15	1.01	0.89	1.09	0.95	1.41	1.23	1.44	1.25	472	49.1	8.82
P51858	Hepatoma-derived growth factor OS=Homo sapiens GN=HDGF PE=1 SV=1 - [HDGF_HUMAN]	3.33	1	1	1	3					1.54	1.51	1.40	1.36	240	26.8	4.73
P81172	Hepcidin OS=Homo sapiens GN=HAMP PE=1 SV=2 - [HEPC_HUMAN]	7.14	1	1	1	1					0.89	1.04	0.98	1.15	84	9.4	8.81
Q9BQS7	Hephaestin OS=Homo sapiens GN=HEPH PE=2 SV=3 - [HEPH_HUMAN]	0.78	1	1	1	1	0.82	1.08	0.92	1.23					1158	130.4	5.99
Q9UPZ3	Hermansky-Pudlak syndrome 5 protein OS=Homo sapiens GN=HPS5 PE=1 SV=2 - [HPS5_HUMAN]	0.97	1	1	1	1					0.98	1.16	0.79	0.93	1129	127.4	5.54
P63135	HERV-K_1q22 provirus ancestral Pol protein OS=Homo sapiens PE=3 SV=1 - [POK12_HUMAN]	5.48	14	1	3	3	1.14	1.08	1.02	0.98					1459	165.1	8.88

O42043	HERV-K_1q23.3 provirus ancestral Env polyprotein OS=Homo sapiens PE=1 SV=2 - [ENK7_HUMAN]	7.50	4	2	3	3	0.70	1.45	0.74	1.53	1.03	0.72	1.38	0.98	560	63.6	7.71
Q99729	Heterogeneous nuclear ribonucleoprotein A/B OS=Homo sapiens GN=HNRNPAB PE=1 SV=2 - [ROAA_HUMAN]	9.94	1	1	1	1	0.88	0.83	1.04	0.97					332	36.2	8.21
P31943	Heterogeneous nuclear ribonucleoprotein H OS=Homo sapiens GN=HNRNPH1 PE=1 SV=4 - [HNRH1_HUMAN]	8.02	3	2	2	4					1.92	1.17	1.52	1.17	449	49.2	6.30
P61978	Heterogeneous nuclear ribonucleoprotein K OS=Homo sapiens GN=HNRNPK PE=1 SV=1 - [HNRPK_HUMAN]	4.32	1	2	2	5					1.38	1.33	1.30	1.25	463	50.9	5.54
O60506	Heterogeneous nuclear ribonucleoprotein Q OS=Homo sapiens GN=SYNCRIP PE=1 SV=2 - [HNRPQ_HUMAN]	2.09	1	1	1	2					1.03	0.97	1.17	1.09	623	69.6	8.59
O43390	Heterogeneous nuclear ribonucleoprotein R OS=Homo sapiens GN=HNRNPR PE=1 SV=1 - [HNRPR_HUMAN]	2.05	1	1	1	3					0.82	0.70	0.98	0.83	633	70.9	8.13
Q00839	Heterogeneous nuclear ribonucleoprotein U OS=Homo sapiens GN=HNRNPU PE=1 SV=6 - [HNRPU_HUMAN]	1.09	1	1	1	1	0.91	0.76	0.95	0.80					825	90.5	6.00
P22626	Heterogeneous nuclear ribonucleoproteins A2/B1 OS=Homo sapiens GN=HNRNPA2B1 PE=1 SV=2 - [ROA2_HUMAN]	4.25	1	1	1	2					1.25	1.18	1.31	1.23	353	37.4	8.95
Q9NZJ6	Hexaprenyldihydroxybenzoate methyltransferase, mitochondrial OS=Homo sapiens GN=COQ3 PE=1 SV=3 - [COQ3_HUMAN]	2.71	1	1	1	1					0.25	0.28	0.82	0.93	369	41.0	7.42
Q8WVB3	Hexosaminidase D OS=Homo sapiens GN=HEXDC PE=2 SV=3 - [HEXDC_HUMAN]	3.50	1	1	1	1					0.51	0.42	0.55	0.45	486	53.8	5.97
Q96JK4	HHIP-like protein 1 OS=Homo sapiens GN=HHIPL1 PE=2 SV=2 - [HIPL1_HUMAN]	4.09	1	2	2	2	1.10	1.07	1.11	1.10					782	86.7	7.58
O95263	High affinity cAMP-specific and IBMX-insensitive 3',5'-cyclic phosphodiesterase 8B OS=Homo sapiens GN=PDE8B PE=1 SV=2 - [PDE8B_HUMAN]	2.03	2	1	2	4	1.34	1.10	1.39	1.16					885	98.9	6.83
P82970	High mobility group nucleosome-binding domain-containing protein 5 OS=Homo sapiens GN=HMGN5 PE=1 SV=1 - [HMGN5_HUMAN]	2.48	1	1	1	1	0.55	0.33	0.77	0.46					282	31.5	4.55
Q96MC6	Hippocampus abundant transcript 1 protein OS=Homo sapiens GN=HIAT1 PE=2 SV=2 - [HIAT1_HUMAN]	1.84	1	1	1	1					0.77	0.76	2.82	2.75	490	53.0	8.40
Q9BW71	HIRA-interacting protein 3 OS=Homo sapiens GN=HIRIP3 PE=1 SV=3 - [HIRP3_HUMAN]	3.42	1	1	2	4							0.94	0.72	556	61.9	8.54
O95568	Histidine protein methyltransferase 1 homolog OS=Homo sapiens GN=METTL18 PE=1 SV=1 - [MET18_HUMAN]	4.84	1	1	2	23	0.94	1.31	1.01	1.42					372	42.1	6.76
P49773	Histidine triad nucleotide-binding protein 1 OS=Homo sapiens GN=HINT1 PE=1 SV=2 - [HINT1_HUMAN]	21.43	1	2	2	5					0.97	0.89	1.09	0.85	126	13.8	6.95
Q9BX68	Histidine triad nucleotide-binding protein 2, mitochondrial OS=Homo sapiens GN=HINT2 PE=1 SV=1 - [HINT2_HUMAN]	10.43	1	1	1	1	1.09	1.24	1.07	1.22					163	17.2	9.16
P12081	Histidine--tRNA ligase, cytoplasmic OS=Homo sapiens GN=HARS PE=1 SV=2 - [SYHC_HUMAN]	11.39	1	2	2	3	1.41	1.24	1.48	1.30					509	57.4	5.88
P04196	Histidine-rich glycoprotein OS=Homo sapiens GN=HRG PE=1 SV=1 - [HRG_HUMAN]	52.76	1	25	25	1106	1.28	1.23	1.29	1.23	1.03	0.99	1.04	1.03	525	59.5	7.50
Q92993	Histone acetyltransferase KAT5 OS=Homo sapiens GN=KAT5 PE=1 SV=2 - [KAT5_HUMAN]	2.53	1	1	1	1	0.94	1.16	0.98	1.22					513	58.5	8.48
Q92769	Histone deacetylase 2 OS=Homo sapiens GN=HDAC2 PE=1 SV=2 - [HDAC2_HUMAN]	1.43	1	1	1	1	1.01	1.08	0.91	0.98					488	55.3	5.91
P56524	Histone deacetylase 4 OS=Homo sapiens GN=HDAC4 PE=1 SV=3 - [HDAC4_HUMAN]	2.03	1	1	1	9	1.02	0.96	0.98	0.93	1.11	1.34	1.06	1.29	1084	119.0	6.96
Q9UBN7	Histone deacetylase 6 OS=Homo sapiens GN=HDAC6 PE=1 SV=2 - [HDAC6_HUMAN]	0.99	1	1	1	1					0.69	0.87	0.64	0.80	1215	131.3	5.30
Q9H0E3	Histone deacetylase complex subunit SAP130 OS=Homo sapiens GN=SAP130 PE=1 SV=1 - [SP130_HUMAN]	3.44	1	1	1	6	0.86	0.83	0.83	0.82					1048	110.3	9.83
O14607	Histone demethylase UTY OS=Homo sapiens GN=UTY PE=1 SV=2 - [UTY_HUMAN]	0.89	1	1	1	1	0.88	0.98	0.90	1.00					1347	149.5	7.75
P16403	Histone H1.2 OS=Homo sapiens GN=HIST1H1C PE=1 SV=2 - [H12_HUMAN]	9.86	5	1	2	5					1.89	1.81	1.78	1.71	213	21.4	10.93

P16401	Histone H1.5 OS=Homo sapiens GN=HIST1H1B PE=1 SV=3 - [H15_HUMAN]	8.85	3	1	2	5					1.75	1.47	1.63	1.38	226	22.6	10.92
Q5VVJ2	Histone H2A deubiquitinase MYSM1 OS=Homo sapiens GN=MYSM1 PE=1 SV=1 - [MYSM1_HUMAN]	3.62	1	2	2	7	1.01	1.19	0.97	1.14	1.32	1.37	1.08	1.20	828	95.0	5.53
Q96KK5	Histone H2A type 1-H OS=Homo sapiens GN=HIST1H2AH PE=1 SV=3 - [H2A1H_HUMAN]	27.34	13	1	3	19					2.46	2.21	2.10	1.88	128	13.9	10.89
P0C5Y9	Histone H2A-Bbd type 1 OS=Homo sapiens GN=H2AFB1 PE=2 SV=1 - [H2AB1_HUMAN]	10.43	2	1	1	3	0.47	0.43	0.50	0.46	0.94	0.83	0.96	0.86	115	12.7	10.67
P0C0S5	Histone H2A.Z OS=Homo sapiens GN=H2AFZ PE=1 SV=2 - [H2AZ_HUMAN]	20.31	5	1	3	19					1.63	1.59	1.36	1.33	128	13.5	10.58
O60814	Histone H2B type 1-K OS=Homo sapiens GN=HIST1H2BK PE=1 SV=3 - [H2B1K_HUMAN]	19.05	15	2	2	26	1.00	0.85	1.06	0.91	1.71	1.71	1.69	1.69	126	13.9	10.32
P49450	Histone H3-like centromeric protein A OS=Homo sapiens GN=CENPA PE=1 SV=1 - [CENPA_HUMAN]	8.57	1	1	1	1	1.14	1.35	1.10	1.31					140	16.0	11.71
P68431	Histone H3.1 OS=Homo sapiens GN=HIST1H3A PE=1 SV=2 - [H31_HUMAN]	16.91	5	4	4	8					1.44	1.46	1.39	1.41	136	15.4	11.12
P62805	Histone H4 OS=Homo sapiens GN=HIST1H4A PE=1 SV=2 - [H4_HUMAN]	19.42	1	2	2	7	0.93	0.89	0.87	0.84	1.57	1.36	1.45	1.27	103	11.4	11.36
Q9BQA5	Histone H4 transcription factor OS=Homo sapiens GN=HINFP PE=1 SV=2 - [HINFP_HUMAN]	2.51	1	1	1	1					0.43	0.30	1.51	1.06	517	59.6	6.19
Q9UPP1	Histone lysine demethylase PHF8 OS=Homo sapiens GN=PHF8 PE=1 SV=3 - [PHF8_HUMAN]	2.08	1	1	1	1	5.15	3.54	1.46	1.01					1060	117.8	8.72
Q09028	Histone-binding protein RBBP4 OS=Homo sapiens GN=RBBP4 PE=1 SV=3 - [RBBP4_HUMAN]	8.00	1	1	1	2	0.90	1.08	0.95	1.15					425	47.6	4.89
Q9NR48	Histone-lysine N-methyltransferase ASH1L OS=Homo sapiens GN=ASH1L PE=1 SV=2 - [ASH1L_HUMAN]	3.74	11	5	8	11	1.11	1.18	1.34	1.53					2969	332.6	9.39
Q15910	Histone-lysine N-methyltransferase EZH2 OS=Homo sapiens GN=EZH2 PE=1 SV=2 - [EZH2_HUMAN]	0.67	1	1	1	1					0.74	0.78	0.68	0.71	746	85.3	7.02
Q03164	Histone-lysine N-methyltransferase MLL OS=Homo sapiens GN=MLL PE=1 SV=5 - [MLL1_HUMAN]	0.96	1	2	2	2	0.85	0.89	0.78	0.82	0.98	1.02	0.87	0.91	3969	431.5	9.09
O14686	Histone-lysine N-methyltransferase MLL2 OS=Homo sapiens GN=MLL2 PE=1 SV=2 - [MLL2_HUMAN]	0.52	1	1	1	1			0.84						5537	593.0	5.58
Q8NEZ4	Histone-lysine N-methyltransferase MLL3 OS=Homo sapiens GN=MLL3 PE=1 SV=3 - [MLL3_HUMAN]	0.96	1	3	3	7					1.52	1.43	1.39	1.31	4911	541.0	6.49
Q9UMN6	Histone-lysine N-methyltransferase MLL4 OS=Homo sapiens GN=WBP7 PE=1 SV=1 - [MLL4_HUMAN]	0.55	1	2	2	2	0.37	0.47	0.41	0.54	0.73	0.90	0.82	1.01	2715	293.3	8.22
Q9BZ95	Histone-lysine N-methyltransferase NSD3 OS=Homo sapiens GN=WHSC1L1 PE=1 SV=1 - [NSD3_HUMAN]	2.02	1	1	1	2	0.90	0.79	0.95	0.84	1.20	1.10	1.19	1.10	1437	161.5	8.21
Q9NQV7	Histone-lysine N-methyltransferase PRDM9 OS=Homo sapiens GN=PRDM9 PE=2 SV=2 - [PRDM9_HUMAN]	6.38	2	3	3	6	1.18	1.21	0.67	0.70	1.05	0.81	1.43	1.09	894	103.3	9.19
O15047	Histone-lysine N-methyltransferase SETD1A OS=Homo sapiens GN=SETD1A PE=1 SV=3 - [SET1A_HUMAN]	1.17	1	1	1	1	1.71		2.40						1707	185.9	5.14
Q86TU7	Histone-lysine N-methyltransferase setd3 OS=Homo sapiens GN=SETD3 PE=1 SV=1 - [SETD3_HUMAN]	1.52	1	1	1	1	0.90	1.01	0.88	1.00					594	67.2	5.96
Q96T68	Histone-lysine N-methyltransferase SETDB2 OS=Homo sapiens GN=SETDB2 PE=1 SV=2 - [SETB2_HUMAN]	1.39	1	1	1	1	1.26	1.01	1.21	0.97					719	81.8	7.50
Q96L73	Histone-lysine N-methyltransferase, H3 lysine-36 and H4 lysine-20 specific OS=Homo sapiens GN=NSD1 PE=1 SV=1 - [NSD1_HUMAN]	2.08	1	3	3	9	0.75	0.68	0.78	0.82	1.40	1.19	1.42	1.20	2696	296.5	8.03
P05534	HLA class I histocompatibility antigen, A-24 alpha chain OS=Homo sapiens GN=HLA-A PE=1 SV=2 - [1A24_HUMAN]	13.42	9	1	3	11	2.40	2.27	2.26	2.16					365	40.7	6.34
P16188	HLA class I histocompatibility antigen, A-30 alpha chain OS=Homo sapiens GN=HLA-A PE=1 SV=2 - [1A30_HUMAN]	15.89	23	2	4	15	0.97	0.85	0.95	0.84	1.04	0.93	1.00	0.89	365	40.9	6.10
P01889	HLA class I histocompatibility antigen, B-7 alpha chain OS=Homo sapiens GN=HLA-B PE=1 SV=3 - [1B07_HUMAN]	12.98	41	1	3	12					0.84	0.83	0.99	0.98	362	40.4	5.85

Q31612	HLA class I histocompatibility antigen, B-73 alpha chain OS=Homo sapiens GN=HLA-B PE=1 SV=1 - [1B73_HUMAN]	14.05	5	1	3	19					1.01	0.85	1.02	0.85	363	40.4	6.24
P05538	HLA class II histocompatibility antigen, DQ beta 2 chain OS=Homo sapiens GN=HLA-DQB2 PE=3 SV=2 - [DQB2_HUMAN]	10.82	1	1	1	1	1.16	2.48	1.03	2.21					268	30.4	6.80
Q30154	HLA class II histocompatibility antigen, DR beta 5 chain OS=Homo sapiens GN=HLA-DRB5 PE=1 SV=1 - [DRB5_HUMAN]	4.51	1	1	1	1	0.68	0.74	0.69	0.75					266	30.0	6.93
Q8WY36	HMG box transcription factor BBX OS=Homo sapiens GN=BBX PE=1 SV=1 - [BBX_HUMAN]	1.49	1	1	1	3	0.85	0.94	0.89	0.99	1.26	1.16	1.18	1.09	941	105.1	8.79
Q12766	HMG domain-containing protein 3 OS=Homo sapiens GN=HMGXB3 PE=2 SV=2 - [HMGX3_HUMAN]	3.19	1	3	3	3					0.72	0.75	2.38	2.42	1538	168.2	8.05
Q03828	Homeobox even-skipped homolog protein 2 OS=Homo sapiens GN=EVX2 PE=2 SV=2 - [EVX2_HUMAN]	9.87	1	1	1	1	2.10	1.86	1.41	1.26					476	47.8	8.92
Q6ZNG2	Homeobox protein DBX2 OS=Homo sapiens GN=DBX2 PE=2 SV=2 - [DBX2_HUMAN]	9.14	1	1	1	4					0.73	0.78	0.75	0.81	339	36.5	9.35
P19622	Homeobox protein engrailed-2 OS=Homo sapiens GN=EN2 PE=1 SV=3 - [HME2_HUMAN]	13.21	1	2	2	2	0.99	2.69	0.28	0.78					333	34.2	9.44
Q14549	Homeobox protein GBX-1 OS=Homo sapiens GN=GBX1 PE=2 SV=2 - [GBX1_HUMAN]	3.31	1	1	1	3					1.24	0.91	1.07	0.79	363	37.6	7.53
Q9NP08	Homeobox protein HMX1 OS=Homo sapiens GN=HMX1 PE=2 SV=2 - [HMX1_HUMAN]	2.01	1	1	1	3					0.46	0.08	0.83	0.14	348	36.1	6.65
A6NHT5	Homeobox protein HMX3 OS=Homo sapiens GN=HMX3 PE=2 SV=1 - [HMX3_HUMAN]	3.64	1	1	1	1					1.06	1.11	1.03	1.07	357	37.8	8.12
P31268	Homeobox protein Hox-A7 OS=Homo sapiens GN=HOXA7 PE=2 SV=3 - [HXA7_HUMAN]	6.52	1	1	1	2					0.84	0.76	0.81	0.71	230	25.3	5.36
P09067	Homeobox protein Hox-B5 OS=Homo sapiens GN=HOXB5 PE=2 SV=3 - [HXB5_HUMAN]	9.29	1	1	1	1	1.22	1.17	1.15	1.12					269	29.4	8.98
P17482	Homeobox protein Hox-B9 OS=Homo sapiens GN=HOXB9 PE=1 SV=2 - [HXB9_HUMAN]	20.00	1	2	2	5	1.58	1.84	1.72	2.03	1.39	1.29	1.30	1.20	250	28.0	8.90
Q9NYD6	Homeobox protein Hox-C10 OS=Homo sapiens GN=HOXC10 PE=1 SV=2 - [HXC10_HUMAN]	6.73	1	2	2	2	0.84	0.67	0.99	0.80	0.83	1.16	0.77	1.07	342	38.0	8.18
P09017	Homeobox protein Hox-C4 OS=Homo sapiens GN=HOXC4 PE=2 SV=2 - [HXC4_HUMAN]	4.55	1	1	1	1					0.14	0.30	0.40	0.84	264	29.8	9.13
P52952	Homeobox protein Nkx-2.5 OS=Homo sapiens GN=NKX2-5 PE=1 SV=1 - [NKX25_HUMAN]	4.32	2	1	1	1	0.28	0.50	1.18	2.11					324	34.9	9.38
A6NCS4	Homeobox protein Nkx-2.6 OS=Homo sapiens GN=NKX2-6 PE=1 SV=1 - [NKX26_HUMAN]	8.97	1	2	2	2	0.78	0.60	1.09	0.84	1.16	1.22	1.11	1.17	301	32.1	9.88
Q99801	Homeobox protein Nkx-3.1 OS=Homo sapiens GN=NKX3-1 PE=1 SV=2 - [NKX31_HUMAN]	5.98	1	1	1	2	0.77	1.17	0.74	1.13					234	26.3	9.20
P78426	Homeobox protein Nkx-6.1 OS=Homo sapiens GN=NKX6-1 PE=2 SV=2 - [NKX61_HUMAN]	3.81	1	1	1	1	1.22	1.40	0.96	1.12					367	37.8	9.45
A6NJ46	Homeobox protein Nkx-6.3 OS=Homo sapiens GN=NKX6-3 PE=2 SV=1 - [NKX63_HUMAN]	8.68	1	1	1	3	0.96	1.70	0.81	1.45					265	28.9	9.23
O60393	Homeobox protein NOBOX OS=Homo sapiens GN=NOBOX PE=1 SV=4 - [NOBOX_HUMAN]	0.72	1	1	1	1					0.95	0.76	1.60	1.27	691	73.9	6.13
Q5XKR4	Homeobox protein orthopedia OS=Homo sapiens GN=OTP PE=1 SV=1 - [OTP_HUMAN]	3.69	1	1	1	1					2.61	2.69	0.74	0.76	325	34.1	9.47
O95343	Homeobox protein SIX3 OS=Homo sapiens GN=SIX3 PE=1 SV=1 - [SIX3_HUMAN]	19.58	1	2	2	6					1.46	0.19			332	35.5	8.73
Q9GZN2	Homeobox protein TGIF2 OS=Homo sapiens GN=TGIF2 PE=1 SV=1 - [TGIF2_HUMAN]	6.75	1	1	1	1					0.63	0.72	0.75	0.86	237	25.9	7.99
O95231	Homeobox protein VENTX OS=Homo sapiens GN=VENTX PE=2 SV=1 - [VENTX_HUMAN]	4.65	1	1	1	1					0.71	0.50	0.65	0.46	258	27.5	9.63
P57058	Hormonally up-regulated neu tumor-associated kinase OS=Homo sapiens GN=HUNK PE=1 SV=1 - [HUNK_HUMAN]	1.40	1	1	1	1					1.09	0.99	0.86	0.79	714	79.6	9.13

Q05469	Hormone-sensitive lipase OS=Homo sapiens GN=LIPE PE=1 SV=4 - [LIPS_HUMAN]	4.09	1	2	2	4					1.65	1.50	1.66	1.51	1076	116.5	6.70
Q96KN8	HRAS-like suppressor 5 OS=Homo sapiens GN=HRASLS5 PE=2 SV=2 - [HRSL5_HUMAN]	4.30	1	1	1	5	1.05	0.88	0.99	0.85					279	30.3	8.24
P50502	Hsc70-interacting protein OS=Homo sapiens GN=ST13 PE=1 SV=2 - [F10A1_HUMAN]	15.72	3	5	5	11	0.69	0.67	0.66	0.64	0.95	1.01	0.96	0.97	369	41.3	5.27
Q9NZL4	Hsp70-binding protein 1 OS=Homo sapiens GN=HSPBP1 PE=1 SV=1 - [HPBP1_HUMAN]	2.49	1	1	1	1	0.55	0.61	0.49	0.55					362	39.4	5.21
P42858	Huntingtin OS=Homo sapiens GN=HTT PE=1 SV=2 - [HD_HUMAN]	0.83	1	2	2	2	0.89	0.86	0.91	0.87					3142	347.4	6.20
O00291	Huntingtin-interacting protein 1 OS=Homo sapiens GN=HIP1 PE=1 SV=5 - [HIP1_HUMAN]	2.60	1	1	2	2					0.95	0.92	0.88	0.85	1037	116.1	5.30
Q86UW8	Hyaluronan and proteoglycan link protein 4 OS=Homo sapiens GN=HAPLN4 PE=2 SV=1 - [HPLN4_HUMAN]	2.49	1	1	1	1					1.03	0.17	1.09	0.18	402	42.8	8.85
Q92819	Hyaluronan synthase 2 OS=Homo sapiens GN=HAS2 PE=2 SV=1 - [HAS2_HUMAN]	4.53	1	2	2	2	0.92	0.97	0.88	0.94	0.73	0.09	0.88	0.11	552	63.5	8.60
Q14520	Hyaluronan-binding protein 2 OS=Homo sapiens GN=HABP2 PE=1 SV=1 - [HABP2_HUMAN]	26.61	1	14	14	131	1.08	1.10	1.14	1.17	1.03	1.10	0.97	0.98	560	62.6	6.54
Q12794	Hyaluronidase-1 OS=Homo sapiens GN=HYAL1 PE=1 SV=2 - [HYAL1_HUMAN]	17.93	1	5	5	18	1.12	1.24	1.07	1.19	0.93	0.87	0.88	0.84	435	48.3	6.77
Q4G0P3	Hydrocephalus-inducing protein homolog OS=Homo sapiens GN=HYDIN PE=1 SV=3 - [HYDIN_HUMAN]	2.36	1	8	8	14	0.73	0.74	0.72	0.87	1.19	1.23	1.02	1.05	5121	575.5	6.06
Q9UJM8	Hydroxyacid oxidase 1 OS=Homo sapiens GN=HAO1 PE=1 SV=1 - [HAOX1_HUMAN]	3.78	1	1	1	5	0.90	1.02	1.08	1.34					370	40.9	8.09
Q9NYQ3	Hydroxyacid oxidase 2 OS=Homo sapiens GN=HAO2 PE=2 SV=1 - [HAOX2_HUMAN]	11.68	1	1	1	2	1.03	0.91	0.35	0.31					351	38.8	7.64
Q01581	Hydroxymethylglutaryl-CoA synthase, cytoplasmic OS=Homo sapiens GN=HMGCS1 PE=1 SV=2 - [HMCS1_HUMAN]	4.81	1	2	2	2	1.17	1.15	1.01	1.00	1.15	1.30	1.05	1.18	520	57.3	5.41
Q14526	Hypermethylated in cancer 1 protein OS=Homo sapiens GN=HIC1 PE=1 SV=5 - [HIC1_HUMAN]	1.09	1	1	1	1					0.53	0.34	0.45	0.29	733	76.5	6.83
P00492	Hypoxanthine-guanine phosphoribosyltransferase OS=Homo sapiens GN=HPRT1 PE=1 SV=2 - [HPRT_HUMAN]	5.96	1	1	1	1	0.97	1.04	0.99	1.08					218	24.6	6.68
Q9Y4L1	Hypoxia up-regulated protein 1 OS=Homo sapiens GN=HYOU1 PE=1 SV=1 - [HYOU1_HUMAN]	14.61	1	10	11	33	0.98	1.00	0.96	0.92	1.06	1.11	1.03	1.04	999	111.3	5.22
Q16665	Hypoxia-inducible factor 1-alpha OS=Homo sapiens GN=HIF1A PE=1 SV=1 - [HIF1A_HUMAN]	2.91	1	1	1	1					1.54	1.83	1.31	1.55	826	92.6	5.33
Q9Y2N7	Hypoxia-inducible factor 3-alpha OS=Homo sapiens GN=HIF3A PE=1 SV=2 - [HIF3A_HUMAN]	3.29	1	1	1	1	0.77	0.84	0.90	0.98					669	72.4	6.02
O75144	ICOS ligand OS=Homo sapiens GN=ICOSLG PE=1 SV=2 - [ICOSL_HUMAN]	8.61	2	3	3	21	1.14	1.14	1.15	1.10	0.80	0.83	0.78	0.82	302	33.3	5.31
P01876	Ig alpha-1 chain C region OS=Homo sapiens GN=IGHA1 PE=1 SV=2 - [IGHA1_HUMAN]	66.01	1	8	26	6129	0.97	0.98	0.97	0.99	0.90	0.98	0.86	0.93	353	37.6	6.51
P01877	Ig alpha-2 chain C region OS=Homo sapiens GN=IGHA2 PE=1 SV=3 - [IGHA2_HUMAN]	66.76	1	5	23	4674	1.01	1.01	0.97	0.97	0.83	0.82	0.81	0.80	340	36.5	6.10
P01880	Ig delta chain C region OS=Homo sapiens GN=IGHD PE=1 SV=2 - [IGHD_HUMAN]	49.74	1	15	15	115	0.92	0.91	0.92	0.94	0.91	1.04	0.83	0.96	384	42.2	7.93
P80422	Ig gamma lambda chain V-II region DOT OS=Homo sapiens PE=1 SV=1 - [LV212_HUMAN]	14.41	1	1	1	4	1.21	1.28	1.15	1.22	1.24	1.62	1.20	1.58	111	11.8	4.78
P01857	Ig gamma-1 chain C region OS=Homo sapiens GN=IGHG1 PE=1 SV=1 - [IGHG1_HUMAN]	73.64	1	15	28	13046	1.11	1.11	1.12	1.13	1.04	1.13	1.05	1.14	330	36.1	8.19
P01859	Ig gamma-2 chain C region OS=Homo sapiens GN=IGHG2 PE=1 SV=2 - [IGHG2_HUMAN]	73.01	1	11	23	9123	0.85	0.90	0.85	0.89	0.98	0.92	0.94	0.93	326	35.9	7.59
P01860	Ig gamma-3 chain C region OS=Homo sapiens GN=IGHG3 PE=1 SV=2 - [IGHG3_HUMAN]	63.93	1	13	28	10503	0.90	0.87	0.92	0.89	0.98	1.04	0.98	0.98	377	41.3	7.90

P01861	Ig gamma-4 chain C region OS=Homo sapiens GN=IGHG4 PE=1 SV=1 - [IGHG4_HUMAN]	70.64	1	8	17	7010	0.87	0.88	0.88	0.89	0.80	0.89	0.78	0.88	327	35.9	7.36
P01742	Ig heavy chain V-I region EU OS=Homo sapiens PE=1 SV=1 - [HV101_HUMAN]	16.24	3	2	2	559	2.06	1.55	1.25	1.04	1.34	1.28	1.22	1.22	117	12.5	6.57
P01743	Ig heavy chain V-I region HG3 OS=Homo sapiens PE=4 SV=1 [HV102_HUMAN]	18.80	2	1	2	13					1.46	1.52	1.42	1.48	117	12.9	8.92
P01744	Ig heavy chain V-I region ND (Fragments) OS=Homo sapiens PE=1 SV=2 - [HV104_HUMAN]	4.76	1	1	1	1	0.89	0.94	0.89	0.94					147	16.5	7.39
P23083	Ig heavy chain V-I region V35 OS=Homo sapiens PE=1 SV=1 - [HV103_HUMAN]	38.46	2	3	4	105	1.01	0.94	0.97	0.88	0.94	0.99	0.94	0.96	117	13.0	9.55
P01760	Ig heavy chain V-I region WOL OS=Homo sapiens PE=1 SV=1 - [HV105_HUMAN]	22.58	1	2	3	41	1.18	1.36	1.36	1.41	0.99	1.06	0.95	1.10	124	13.7	8.41
P06331	Ig heavy chain V-II region ARH-77 OS=Homo sapiens PE=4 SV=1 - [HV209_HUMAN]	17.12	2	2	2	38	0.85	0.95	0.83	0.93	0.98	1.09	0.92	1.00	146	16.2	8.28
P01815	Ig heavy chain V-II region COR OS=Homo sapiens PE=1 SV=1 - [HV202_HUMAN]	15.83	2	1	3	15	0.92	1.09	0.77	0.92	0.91	0.80	0.84	0.73	120	13.2	6.57
P01818	Ig heavy chain V-II region HE OS=Homo sapiens PE=1 SV=1 - [HV205_HUMAN]	5.79	1	1	1	1					0.96	1.07	0.94	1.05	121	13.5	8.50
P04438	Ig heavy chain V-II region SESS OS=Homo sapiens PE=2 SV=1 - [HV208_HUMAN]	19.73	3	2	4	58	0.81	0.77	0.80	0.82	0.84	0.95	0.78	0.89	147	16.3	7.09
P01824	Ig heavy chain V-II region WAH OS=Homo sapiens PE=1 SV=1 - [HV206_HUMAN]	17.05	2	2	3	26	1.02	1.11	0.96	1.01	0.98	1.05	0.96	1.02	129	14.1	8.46
P01766	Ig heavy chain V-III region BRO OS=Homo sapiens PE=1 SV=1 - [HV305_HUMAN]	41.67	1	2	4	636	0.99	0.96	1.03	1.00	0.96	1.08	0.95	1.04	120	13.2	6.57
P01773	Ig heavy chain V-III region BUR OS=Homo sapiens PE=1 SV=1 - [HV312_HUMAN]	36.13	1	2	3	15	1.14	1.42	1.07	1.34	1.15	1.23	1.18	1.24	119	13.0	9.60
P01767	Ig heavy chain V-III region BUT OS=Homo sapiens PE=1 SV=1 - [HV306_HUMAN]	30.43	1	1	5	166	0.89	0.85	0.89	0.91	1.01	1.08	1.00	1.05	115	12.4	9.25
P01781	Ig heavy chain V-III region GAL OS=Homo sapiens PE=1 SV=1 - [HV320_HUMAN]	54.31	2	7	8	208	0.90	1.01	0.86	0.98	0.97	1.06	1.03	1.06	116	12.7	8.48
P80419	Ig heavy chain V-III region GAR OS=Homo sapiens PE=1 SV=1 - [HV322_HUMAN]	20.34	1	1	2	7	0.79	0.77	0.91	0.89	0.58	0.62	0.53	0.56	118	13.1	4.81
P01780	Ig heavy chain V-III region JON OS=Homo sapiens PE=1 SV=1 - [HV319_HUMAN]	43.48	1	2	4	29	0.97	1.02	1.11	1.09	0.84	1.08	0.86	1.02	115	12.6	9.29
P01772	Ig heavy chain V-III region KOL OS=Homo sapiens PE=1 SV=1 - [HV311_HUMAN]	26.19	2	2	4	45	0.98	0.93	1.06	0.96	1.00	1.04	1.03	1.03	126	13.7	5.87
P01775	Ig heavy chain V-III region LAY OS=Homo sapiens PE=1 SV=1 - [HV314_HUMAN]	27.73	1	1	3	36	0.84	0.90	0.81	0.84	0.72	0.79	0.72	0.77	119	12.8	8.48
P01770	Ig heavy chain V-III region NIE OS=Homo sapiens PE=1 SV=1 - [HV309_HUMAN]	41.18	1	2	5	32	1.05	1.11	1.01	1.10	0.97	0.84	1.02	0.88	119	13.2	9.72
P01765	Ig heavy chain V-III region TIL OS=Homo sapiens PE=1 SV=1 - [HV304_HUMAN]	54.78	1	1	5	576	0.90	0.97	0.96	0.99	1.01	0.87	1.03	0.91	115	12.3	9.13
P01762	Ig heavy chain V-III region TRO OS=Homo sapiens PE=1 SV=1 - [HV301_HUMAN]	32.79	1	4	4	43	1.18	1.20	1.26	1.19	1.04	1.06	1.02	1.13	122	13.5	9.72
P01764	Ig heavy chain V-III region VH26 OS=Homo sapiens PE=1 SV=1 - [HV303_HUMAN]	30.77	2	1	4	67	0.91	1.05	0.86	1.02	1.01	1.00	1.00	0.98	117	12.6	8.28
P01776	Ig heavy chain V-III region WAS OS=Homo sapiens PE=1 SV=1 - [HV315_HUMAN]	68.38	2	3	7	493	0.86	1.04	0.92	1.14	1.06	1.00	1.11	1.06	117	13.1	7.99
P01763	Ig heavy chain V-III region WEA OS=Homo sapiens PE=1 SV=1 - [HV302_HUMAN]	40.35	1	2	4	118	1.00	0.98	1.02	1.00	0.97	1.04	0.99	1.12	114	12.2	8.50
P01778	Ig heavy chain V-III region ZAP OS=Homo sapiens PE=1 SV=1 - [HV317_HUMAN]	44.83	1	2	4	33	1.38	1.42	1.28	1.41	1.03	1.14	0.94	1.02	116	12.6	8.51
P01834	Ig kappa chain C region OS=Homo sapiens GN=IGKC PE=1 SV=1 - [IGKC_HUMAN]	92.45	1	12	12	9500	0.93	0.96	0.93	0.95	0.94	0.99	0.94	0.97	106	11.6	5.87

P01593	Ig kappa chain V-I region AG OS=Homo sapiens PE=1 SV=1 - [KV101_HUMAN]	42.59	2	2	5	877	1.84	1.54	1.54	1.30	1.39	1.53	1.25	1.37	108	12.0	5.99
P01594	Ig kappa chain V-I region AU OS=Homo sapiens PE=1 SV=1 - [KV102_HUMAN]	31.48	2	1	2	843					2.27	2.26	2.35	2.34	108	11.9	5.33
P04430	Ig kappa chain V-I region BAN OS=Homo sapiens PE=1 SV=1 [KV122_HUMAN]	22.22	3	1	2	241	0.78	0.74	0.85	0.76	0.92	0.93	0.96	0.89	108	11.8	8.44
P01596	Ig kappa chain V-I region CAR OS=Homo sapiens PE=1 SV=1 - [KV104_HUMAN]	49.53	1	3	4	360	1.07	1.14	1.02	1.08	1.10	1.14	1.10	1.09	107	11.7	9.41
P01597	Ig kappa chain V-I region DEE OS=Homo sapiens PE=1 SV=1 [KV105_HUMAN]	32.41	3	3	5	215	1.06	1.14	1.03	1.08	0.95	1.04	0.99	1.11	108	11.7	9.36
P01598	Ig kappa chain V-I region EU OS=Homo sapiens PE=1 SV=1 - [KV106_HUMAN]	38.89	3	3	5	531	1.09	1.06	1.06	1.07	1.12	1.22	1.08	1.18	108	11.8	8.44
P01599	Ig kappa chain V-I region Gal OS=Homo sapiens PE=1 SV=1 - [KV107_HUMAN]	33.33	2	3	4	852	0.87	0.92	0.83	0.89	1.35	1.32	0.95	0.93	108	11.8	8.88
P01600	Ig kappa chain V-I region Hau OS=Homo sapiens PE=1 SV=1 - [KV108_HUMAN]	38.89	5	1	4	962	1.30	1.15	1.58	1.41	1.40	1.79	1.38	1.76	108	11.7	8.48
P01601	Ig kappa chain V-I region HK101 (Fragment) OS=Homo sapiens PE=4 SV=1 - [KV109_HUMAN]	18.80	3	1	2	117	0.96	1.05	0.92	1.01	1.09	1.03	1.09	1.02	117	12.8	8.16
P01602	Ig kappa chain V-I region HK102 (Fragment) OS=Homo sapiens GN=IGKV1-5 PE=4 SV=1 - [KV110_HUMAN]	34.19	3	2	3	118	1.33	1.46	1.25	1.39	1.85	1.58	1.59	1.37	117	12.8	6.51
P01603	Ig kappa chain V-I region Ka OS=Homo sapiens PE=1 SV=1 - [KV111_HUMAN]	37.96	1	2	3	29	3.35	3.64	3.67	3.95	0.87	0.88	0.76	0.80	108	11.9	8.85
P01604	Ig kappa chain V-I region Kue OS=Homo sapiens PE=1 SV=1 - [KV112_HUMAN]	32.41	3	2	3	118	0.75	0.73	0.76	0.75	0.86	0.86	0.80	0.79	108	12.1	8.81
P01605	Ig kappa chain V-I region Lay OS=Homo sapiens PE=1 SV=1 - [KV113_HUMAN]	31.48	1	2	3	151	1.12	1.17	1.17	1.19	1.36	1.44	1.39	1.45	108	11.8	7.96
P01612	Ig kappa chain V-I region Mev OS=Homo sapiens PE=1 SV=1 - [KV120_HUMAN]	36.70	1	3	3	290	0.91	0.87	0.92	0.88	0.90	0.96	0.87	0.95	109	11.9	6.57
P01613	Ig kappa chain V-I region Ni OS=Homo sapiens PE=1 SV=1 - [KV121_HUMAN]	30.36	1	1	2	177	0.81	0.85	0.73	0.79	0.88	0.90	0.86	0.83	112	12.2	5.36
P01606	Ig kappa chain V-I region OU OS=Homo sapiens PE=1 SV=1 - [KV114_HUMAN]	22.22	3	1	5	186	0.81	0.58	0.88	0.63	1.02	1.07	0.92	0.97	108	11.8	9.91
P01608	Ig kappa chain V-I region Roy OS=Homo sapiens PE=1 SV=1 - [KV116_HUMAN]	24.07	2	1	2	845					1.04	0.76	0.89	0.66	108	11.8	5.36
P01609	Ig kappa chain V-I region Scw OS=Homo sapiens PE=1 SV=1 - [KV117_HUMAN]	27.78	2	1	2	879	0.83	0.79	0.85	0.83	0.79	0.76	0.87	0.81	108	11.8	6.00
P01610	Ig kappa chain V-I region WEA OS=Homo sapiens PE=1 SV=1 - [KV118_HUMAN]	55.56	5	3	6	971	0.86	0.94	0.82	0.91	1.02	0.92	1.07	0.97	108	11.8	8.91
P01611	Ig kappa chain V-I region Wes OS=Homo sapiens PE=1 SV=1 - [KV119_HUMAN]	37.04	3	2	3	311	1.04	1.00	1.06	0.96	1.02	1.08	0.99	1.06	108	11.6	7.28
P01614	Ig kappa chain V-II region Cum OS=Homo sapiens PE=1 SV=1 - [KV201_HUMAN]	41.74	2	1	5	175	1.36	1.11	1.38	1.12	0.99	1.25	0.96	1.19	115	12.7	5.40
P01615	Ig kappa chain V-II region FR OS=Homo sapiens PE=1 SV=1 - [KV202_HUMAN]	24.78	1	2	3	19	0.87	0.91	0.91	0.96	0.77	0.89	0.85	1.17	113	12.7	7.94
P01616	Ig kappa chain V-II region MIL OS=Homo sapiens PE=1 SV=1 - [KV203_HUMAN]	27.68	1	1	3	53	1.21	0.95	1.21	1.10	0.96	1.02	0.86	1.00	112	12.0	9.29
P06310	Ig kappa chain V-II region RPMI 6410 OS=Homo sapiens PE=4 SV=1 - [KV206_HUMAN]	20.30	1	1	4	142	1.29	0.98	1.38	1.06					133	14.7	9.25
P01617	Ig kappa chain V-II region TEW OS=Homo sapiens PE=1 SV=1 - [KV204_HUMAN]	41.59	2	1	5	396	1.09	0.95	1.09	0.97	0.96	1.04	0.92	1.03	113	12.3	6.00
P01619	Ig kappa chain V-III region B6 OS=Homo sapiens PE=1 SV=1 - [KV301_HUMAN]	26.85	1	4	5	87	0.91	0.96	0.87	0.92	0.90	0.85	0.98	0.90	108	11.6	9.25
P04207	Ig kappa chain V-III region CLL OS=Homo sapiens PE=1 SV=2 - [KV308_HUMAN]	45.74	1	2	5	114	0.91	0.94	0.86	0.90	0.92	0.86	0.98	0.93	129	14.3	8.51

P18135	Ig kappa chain V-III region HAH OS=Homo sapiens PE=2 SV=1 - [KV312_HUMAN]	55.81	2	2	9	546	1.34	1.08	1.08	0.86	1.17	1.27	1.03	1.16	129	14.1	7.96
P06311	Ig kappa chain V-III region IARC/BL41 OS=Homo sapiens PE=1 SV=1 - [KV311_HUMAN]	41.41	1	5	5	52	1.05	1.05	1.01	1.01	1.29	1.11	1.24	1.06	128	14.1	6.61
P01621	Ig kappa chain V-III region NG9 (Fragment) OS=Homo sapiens PE=1 SV=1 - [KV303_HUMAN]	60.00	1	2	5	88	0.92	0.90	0.71	0.75	0.82	0.79	0.93	0.91	100	10.7	6.52
P01624	Ig kappa chain V-III region POM OS=Homo sapiens PE=1 SV=1 - [KV306_HUMAN]	36.70	1	2	5	78	1.01	1.05	0.96	0.97	1.04	1.14	0.79	1.07	109	11.9	8.94
P01620	Ig kappa chain V-III region SIE OS=Homo sapiens PE=1 SV=1 - [KV302_HUMAN]	71.56	1	1	8	1308	1.02	1.09	1.08	1.17	1.15	1.10	1.10	1.05	109	11.8	8.48
P01622	Ig kappa chain V-III region Ti OS=Homo sapiens PE=1 SV=1 - [KV304_HUMAN]	71.56	1	2	8	1301	0.62	0.61	0.64	0.62	1.07	1.22	1.07	1.22	109	11.8	8.50
P04433	Ig kappa chain V-III region VG (Fragment) OS=Homo sapiens PE=1 SV=1 - [KV309_HUMAN]	63.48	1	3	5	69	1.00	0.98	0.96	1.00	1.04	1.00	1.04	1.00	115	12.6	4.96
P04434	Ig kappa chain V-III region VH (Fragment) OS=Homo sapiens PE=4 SV=1 - [KV310_HUMAN]	28.45	1	2	3	73	0.94	0.86	0.96	0.90	0.93	1.02	0.86	0.94	116	12.7	5.94
P01625	Ig kappa chain V-IV region Len OS=Homo sapiens PE=1 SV=2 - [KV402_HUMAN]	48.25	2	4	5	557	0.97	0.92	0.98	0.94	1.01	1.07	0.94	1.02	114	12.6	7.93
P83593	Ig kappa chain V-IV region STH (Fragment) OS=Homo sapiens PE=1 SV=1 - [KV405_HUMAN]	42.20	1	3	4	90	0.97	1.13	0.94	1.10	1.18	1.17	1.12	1.14	109	12.1	7.94
P04211	Ig lambda chain V region 4A OS=Homo sapiens PE=4 SV=1 - [LV001_HUMAN]	21.37	1	3	3	60	1.09	1.07	1.03	1.01	1.02	1.10	1.02	1.09	117	12.4	7.03
P06888	Ig lambda chain V-I region EPS OS=Homo sapiens PE=1 SV=1 - [LV109_HUMAN]	19.27	1	1	3	61	1.15	1.26	0.96	1.06	1.18		0.68		109	11.4	9.29
P01700	Ig lambda chain V-I region HA OS=Homo sapiens PE=1 SV=1 - [LV102_HUMAN]	23.21	3	1	4	376	1.03	0.74	0.97	0.70	1.11	1.70	1.04	1.34	112	11.9	8.91
P06887	Ig lambda chain V-I region MEM OS=Homo sapiens PE=1 SV=1 - [LV108_HUMAN]	14.29	1	1	1	3					0.70	0.77	0.78	0.86	112	11.8	5.25
P01701	Ig lambda chain V-I region NEW OS=Homo sapiens PE=1 SV=1 - [LV103_HUMAN]	18.92	1	1	2	114	0.79	0.85	0.79	0.83	0.86	0.83	0.95	0.92	111	11.4	8.00
P01703	Ig lambda chain V-I region NEWM OS=Homo sapiens PE=1 SV=1 - [LV105_HUMAN]	36.89	1	4	4	37	1.04	1.06	1.00	0.97	1.13	1.02	1.11	1.02	103	10.9	9.29
P01702	Ig lambda chain V-I region NIG-64 OS=Homo sapiens PE=1 SV=1 - [LV104_HUMAN]	18.92	2	2	4	73	1.12	1.02	1.22	1.15	1.35	1.31	1.10	1.07	111	11.4	4.89
P01699	Ig lambda chain V-I region VOR OS=Homo sapiens PE=1 SV=1 - [LV101_HUMAN]	29.73	1	2	3	17	1.20	1.13	1.11	1.05	0.87	0.93	0.85	0.90	111	11.5	5.29
P04208	Ig lambda chain V-I region WAH OS=Homo sapiens PE=1 SV=1 - [LV106_HUMAN]	42.20	1	1	4	348	1.00	0.93	0.97	0.95	0.89	0.91	1.03	1.05	109	11.7	6.54
P01710	Ig lambda chain V-II region BO OS=Homo sapiens PE=1 SV=1 - [LV207_HUMAN]	26.13	1	2	2	20	1.28	1.18	1.21	1.11	1.49	1.56	1.64	1.57	111	11.8	6.00
P01708	Ig lambda chain V-II region BUR OS=Homo sapiens PE=1 SV=1 - [LV205_HUMAN]	10.09	1	1	1	19	1.14	1.22	1.10	1.17	1.18	1.21	1.17	1.19	109	11.5	7.87
P01705	Ig lambda chain V-II region NEI OS=Homo sapiens PE=1 SV=1 - [LV202_HUMAN]	7.21	1	1	1	31	1.07	1.08	1.05	1.02	1.18	1.23	1.26	1.31	111	11.6	8.63
P04209	Ig lambda chain V-II region NIG-84 OS=Homo sapiens PE=1 SV=1 - [LV211_HUMAN]	21.43	1	2	2	21	0.99	0.98	0.97	0.99	1.03	1.00	1.05	1.03	112	11.6	7.12
P01707	Ig lambda chain V-II region TRO OS=Homo sapiens PE=1 SV=1 - [LV204_HUMAN]	26.13	1	2	3	69	0.95	0.95	0.91	0.90	0.95	0.98	0.91	0.89	111	11.6	8.29
P01711	Ig lambda chain V-II region VIL OS=Homo sapiens PE=1 SV=1 - [LV208_HUMAN]	6.31	1	1	1	1	0.96	1.00	0.99	1.04					111	11.4	6.51
P80748	Ig lambda chain V-III region LOI OS=Homo sapiens PE=1 SV=1 - [LV302_HUMAN]	45.95	1	4	4	778	0.98	1.00	0.95	0.95	0.96	0.92	1.06	1.02	111	11.9	5.08
P01714	Ig lambda chain V-III region SH OS=Homo sapiens PE=1 SV=1 - [LV301_HUMAN]	42.59	1	4	4	100	0.98	0.94	0.93	0.89	0.97	1.01	0.95	0.99	108	11.4	6.52

P01715	Ig lambda chain V-IV region Bau OS=Homo sapiens PE=1 SV=1 - [LV401_HUMAN]	7.55	1	1	1	8	1.23	0.96	1.28	1.02	0.86	0.76	1.08	0.96	106	11.3	5.24
P01717	Ig lambda chain V-IV region Hil OS=Homo sapiens PE=1 SV=1 - [LV403_HUMAN]	17.76	1	1	1	90	1.02	1.01	1.02	1.05	1.12	1.08	1.12	1.08	107	11.5	6.51
P06889	Ig lambda chain V-IV region MOL OS=Homo sapiens PE=1 SV=1 - [LV405_HUMAN]	23.58	1	1	1	14	1.05	1.09	1.05	1.10	1.27	1.28	1.11	1.13	106	11.3	4.42
P01716	Ig lambda chain V-IV region X OS=Homo sapiens PE=1 SV=1 - [LV402_HUMAN]	6.60	1	1	1	1	0.67	0.70	0.65	0.68					106	11.3	4.59
P01719	Ig lambda chain V-V region DEL OS=Homo sapiens PE=1 SV=1 - [LV501_HUMAN]	59.26	1	3	3	202	1.11	1.17	1.09	1.15	1.13	1.03	1.20	1.11	108	11.3	5.36
P01722	Ig lambda chain V-VI region NIG-48 OS=Homo sapiens PE=1 SV=1 - [LV602_HUMAN]	21.43	1	2	2	7	1.06	1.17	0.98	1.09	1.11	1.10	1.61	1.58	112	12.1	6.51
P06317	Ig lambda chain V-VI region SUT OS=Homo sapiens PE=1 SV=1 - [LV603_HUMAN]	36.94	2	4	4	33	0.77	0.72	0.74	0.70	0.92	0.85	0.99	0.89	111	12.2	5.12
P06318	Ig lambda chain V-VI region WLT OS=Homo sapiens PE=1 SV=1 - [LV604_HUMAN]	18.02	2	1	1	29	1.02	1.08	1.01	1.06	1.23	1.30	1.04	1.12	111	12.0	4.78
P0CG05	Ig lambda-2 chain C regions OS=Homo sapiens GN=IGLC2 PE=1 SV=1 - [LAC2_HUMAN]	96.23	1	2	11	3396	1.11	1.05	1.12	1.06	1.22	1.28	1.08	1.16	106	11.3	7.24
P0CG06	Ig lambda-3 chain C regions OS=Homo sapiens GN=IGLC3 PE=1 SV=1 - [LAC3_HUMAN]	89.62	1	1	9	3117	0.82	0.99	0.88	1.07	1.16	1.23	1.25	1.32	106	11.2	7.24
P0CF74	Ig lambda-6 chain C region OS=Homo sapiens GN=IGLC6 PE=4 SV=1 - [LAC6_HUMAN]	72.64	1	1	6	2708					5.48	4.91	4.99	4.48	106	11.3	7.24
A0M8Q6	Ig lambda-7 chain C region OS=Homo sapiens GN=IGLC7 PE=1 SV=2 - [LAC7_HUMAN]	88.68	1	5	9	2178	1.34	1.32	1.31	1.31	1.59	1.74	1.36	1.58	106	11.3	8.28
P01871	Ig mu chain C region OS=Homo sapiens GN=IGHM PE=1 SV=3 - [IGHM_HUMAN]	64.60	1	18	36	4279	1.24	1.30	1.27	1.34	1.12	1.11	1.02	1.07	452	49.3	6.77
P04220	Ig mu heavy chain disease protein OS=Homo sapiens PE=1 SV=1 - [MUCB_HUMAN]	61.89	1	2	22	2217	0.88	0.83	0.89	0.92					391	43.0	5.24
Q9Y6R7	IgGFc-binding protein OS=Homo sapiens GN=FCGBP PE=1 SV=3 - [FCGBP_HUMAN]	13.62	1	39	39	124	0.86	0.84	0.83	0.79	0.62	0.71	0.65	0.74	5405	571.6	5.34
Q5T953	Immediate early response gene 5-like protein OS=Homo sapiens GN=IER5L PE=2 SV=1 - [IER5L_HUMAN]	2.23	1	1	1	1	1.37	1.32	1.17	1.13					404	42.1	6.93
P01591	Immunoglobulin J chain OS=Homo sapiens GN=IGJ PE=1 SV=4 - [IGJ_HUMAN]	72.33	1	9	9	290	1.17	1.11	1.29	1.23	1.14	1.09	1.15	1.07	159	18.1	5.24
P15814	Immunoglobulin lambda-like polypeptide 1 OS=Homo sapiens GN=IGLL1 PE=1 SV=1 - [IGLL1_HUMAN]	20.19	1	2	3	42	0.99	0.96	1.01	0.99	0.72	0.89	0.72	0.94	213	22.9	10.07
B9A064	Immunoglobulin lambda-like polypeptide 5 OS=Homo sapiens GN=IGLL5 PE=2 SV=2 - [IGLL5_HUMAN]	51.40	2	5	12	2093	0.97	0.94	0.99	0.95	1.05	1.14	0.97	1.10	214	23.0	8.84
O14498	Immunoglobulin superfamily containing leucine-rich repeat protein OS=Homo sapiens GN=ISLR PE=1 SV=1 - [ISLR_HUMAN]	10.05	1	3	3	22	0.99	0.95	1.03	0.96	0.76	0.82	0.72	0.76	428	46.0	5.15
Q6WRI0	Immunoglobulin superfamily member 10 OS=Homo sapiens GN=IGSF10 PE=1 SV=1 - [IGS10_HUMAN]	1.14	1	2	2	3	0.30	0.33	0.35	0.39					2623	290.7	9.13
O75054	Immunoglobulin superfamily member 3 OS=Homo sapiens GN=IGSF3 PE=1 SV=3 - [IGSF3_HUMAN]	1.76	1	1	1	2	0.67	0.55	0.67	0.56					1194	135.1	6.07
Q9NSI5	Immunoglobulin superfamily member 5 OS=Homo sapiens GN=IGSF5 PE=2 SV=2 - [IGSF5_HUMAN]	1.72	1	1	1	1					0.66	0.65	0.78	0.78	407	44.6	7.80
Q86VF2	Immunoglobulin-like and fibronectin type III domain-containing protein 1 OS=Homo sapiens GN=IGFN1 PE=1 SV=2 - [IGFN1_HUMAN]	3.36	1	2	2	27	1.18	1.16	1.05	1.10	1.02	1.05	0.99	1.01	1251	137.7	7.56
Q71H61	Immunoglobulin-like domain-containing receptor 2 OS=Homo sapiens GN=ILDR2 PE=2 SV=1 - [ILDR2_HUMAN]	1.88	1	2	2	3					0.68	0.81	0.93	0.83	639	71.2	8.12
A9QM74	Importin subunit alpha-8 OS=Homo sapiens GN=KPNA7 PE=1 SV=1 - [IMA8_HUMAN]	2.13	2	1	1	1					0.77	0.80	0.79	0.81	516	56.9	6.04
Q14974	Importin subunit beta-1 OS=Homo sapiens GN=KPNB1 PE=1 SV=2 - [IMB1_HUMAN]	1.03	1	1	1	1	0.56	0.66	0.66	0.78					876	97.1	4.78

Q9UI26	Importin-11 OS=Homo sapiens GN=IPO11 PE=1 SV=1 - [IPO11_HUMAN]	1.44	1	1	1	1	0.91	0.36	0.90	0.36					975	112.5	5.25
Q96P70	Importin-9 OS=Homo sapiens GN=IPO9 PE=1 SV=3 - [IPO9_HUMAN]	1.54	1	1	1	7					1.09	1.12	1.08	1.11	1041	115.9	4.81
Q6UXS9	Inactive caspase-12 OS=Homo sapiens GN=CASP12 PE=2 SV=1 - [CASPC_HUMAN]	3.81	1	1	1	1					0.60	0.96	0.57	0.90	341	38.9	6.02
Q8N608	Inactive dipeptidyl peptidase 10 OS=Homo sapiens GN=DPP10 PE=1 SV=2 - [DPP10_HUMAN]	1.51	1	1	1	1	0.88	0.76	1.00	0.86					796	90.8	6.57
Q9UPR0	Inactive phospholipase C-like protein 2 OS=Homo sapiens GN=PLCL2 PE=1 SV=2 - [PLCL2_HUMAN]	3.37	1	2	2	2					4.53	4.92	0.82	0.92	1127	125.8	6.90
Q8N7P1	Inactive phospholipase D5 OS=Homo sapiens GN=PLD5 PE=2 SV=2 - [PLD5_HUMAN]	0.93	1	1	1	1					1.11	0.92	1.06	0.88	536	61.3	8.72
Q6ZVT0	Inactive polyglycylase TTLL10 OS=Homo sapiens GN=TTLL10 PE=1 SV=2 - [TTL10_HUMAN]	1.63	1	1	1	1					0.69	0.50	0.56	0.41	673	75.0	9.23
Q8IWB6	Inactive serine/threonine-protein kinase TEX14 OS=Homo sapiens GN=TEX14 PE=1 SV=2 - [TEX14_HUMAN]	1.87	1	2	2	2	1.56	1.10	1.21	0.86	1.13	0.94	1.07	0.89	1497	167.8	5.15
Q8IV63	Inactive serine/threonine-protein kinase VRK3 OS=Homo sapiens GN=VRK3 PE=1 SV=2 - [VRK3_HUMAN]	1.27	1	1	1	1	0.89	1.06	0.81	0.97					474	52.8	9.04
Q13308	Inactive tyrosine-protein kinase 7 OS=Homo sapiens GN=PTK7 PE=1 SV=2 - [PTK7_HUMAN]	1.12	1	1	1	2	1.00	0.99	1.10	1.10					1070	118.3	7.09
A6NCW7	Inactive ubiquitin carboxyl-terminal hydrolase 17-like protein 4 OS=Homo sapiens GN=USP17L4 PE=3 SV=3 - [U17L4_HUMAN]	1.32	3	1	1	1					2.71	1.57	1.15	0.67	530	59.6	7.42
Q70EL3	Inactive ubiquitin carboxyl-terminal hydrolase 50 OS=Homo sapiens GN=USP50 PE=2 SV=1 - [UBP50_HUMAN]	4.13	1	1	1	2	1.63	1.63	1.80	1.82	0.86	0.88	0.90	0.92	339	38.9	6.74
Q70EL1	Inactive ubiquitin carboxyl-terminal hydrolase 54 OS=Homo sapiens GN=USP54 PE=1 SV=4 - [UBP54_HUMAN]	2.08	1	1	1	1	0.55	0.12	0.38	0.08					1684	187.3	7.28
Q8NI35	InaD-like protein OS=Homo sapiens GN=INADL PE=1 SV=3 - [INADL_HUMAN]	1.55	1	2	2	4	0.66	0.68	0.64	0.67	0.46	0.43	0.47	0.45	1801	196.2	4.94
Q14623	Indian hedgehog protein OS=Homo sapiens GN=IHH PE=1 SV=4 - [IHH_HUMAN]	5.60	1	2	2	6	1.08	1.05	1.07	1.04	1.11	1.25	1.10	1.24	411	45.2	8.76
P14902	Indoleamine 2,3-dioxygenase 1 OS=Homo sapiens GN=IDO1 PE=1 SV=1 - [I23O1_HUMAN]	9.68	1	1	1	1	1.19	1.08	0.85	0.78					403	45.3	7.30
Q9Y6Y0	Influenza virus NS1A-binding protein OS=Homo sapiens GN=IVNS1ABP PE=1 SV=3 - [NS1BP_HUMAN]	1.40	1	1	2	2					3.86	2.09	2.14	1.15	642	71.7	5.53
P55103	Inhibin beta C chain OS=Homo sapiens GN=INHBC PE=1 SV=1 - [INHBC_HUMAN]	22.44	1	5	5	28	0.92	0.93	0.96	0.90	1.07	1.09	0.97	1.04	352	38.2	7.11
P58166	Inhibin beta E chain OS=Homo sapiens GN=INHBE PE=1 SV=1 - [INHBE_HUMAN]	18.00	1	5	5	12	1.09	1.15	1.24	1.31	0.69	1.10	0.80	1.27	350	38.5	9.25
Q9P2D0	Inhibitor of Bruton tyrosine kinase OS=Homo sapiens GN=IBTK PE=1 SV=3 - [IBTK_HUMAN]	2.73	2	2	3	4	####	###	####	11.77	0.67	0.87	0.77	0.99	1353	150.4	7.71
Q9Y2U8	Inner nuclear membrane protein Man1 OS=Homo sapiens GN=LEMD3 PE=1 SV=2 - [MAN1_HUMAN]	4.39	1	2	2	2	0.75	0.88	1.07	1.27					911	99.9	7.55
Q9C086	INO80 complex subunit B OS=Homo sapiens GN=INO80B PE=1 SV=2 - [IN80B_HUMAN]	1.97	1	1	1	1	0.77	0.79	0.85	0.88					356	38.6	9.61
Q53TQ3	INO80 complex subunit D OS=Homo sapiens GN=INO80D PE=1 SV=2 - [IN80D_HUMAN]	3.99	1	2	2	2	1.94	2.15	2.06	2.28	0.88	0.45	0.85	0.43	878	98.1	8.18
Q9H2U2	Inorganic pyrophosphatase 2, mitochondrial OS=Homo sapiens GN=PPA2 PE=1 SV=2 - [IPYR2_HUMAN]	4.79	1	1	1	2	1.62	1.58	1.46	1.43					334	37.9	7.39
Q15181	Inorganic pyrophosphatase OS=Homo sapiens GN=PPA1 PE=1 SV=2 - [IPYR_HUMAN]	7.27	1	1	1	4					0.83	1.00	0.51	0.61	289	32.6	5.86
Q14643	Inositol 1,4,5-trisphosphate receptor type 1 OS=Homo sapiens GN=ITPR1 PE=1 SV=2 - [ITPR1_HUMAN]	0.44	1	1	1	1	1.17	1.05	1.16	1.05					2758	313.7	6.04
Q14571	Inositol 1,4,5-trisphosphate receptor type 2 OS=Homo sapiens GN=ITPR2 PE=1 SV=2 - [ITPR2_HUMAN]	2.11	1	3	3	4	1.24	1.17	1.29	1.24					2701	307.9	6.43

Q14573	Inositol 1,4,5-trisphosphate receptor type 3 OS=Homo sapiens GN=ITPR3 PE=1 SV=2 - [ITPR3_HUMAN]	1.50	1	2	2	2	0.92	1.02	0.86	1.15					2671	303.9	6.48
Q8IWB1	Inositol 1,4,5-trisphosphate receptor-interacting protein OS=Homo sapiens GN=ITPRIP PE=1 SV=1 - [IPRI_HUMAN]	2.56	1	1	1	1	1.09	1.13	0.88	0.91					547	62.0	5.88
P49441	Inositol polyphosphate 1-phosphatase OS=Homo sapiens GN=INPP1 PE=1 SV=1 - [INPP_HUMAN]	7.27	1	1	1	2	1.01	1.18	0.88	1.03			0.55	0.55	399	44.0	5.26
P35568	Insulin receptor substrate 1 OS=Homo sapiens GN=IRS1 PE=1 SV=1 - [IRS1_HUMAN]	1.05	1	1	1	1	0.29	0.19	0.43	0.29					1242	131.5	8.54
Q9NZI8	Insulin-like growth factor 2 mRNA-binding protein 1 OS=Homo sapiens GN=IGF2BP1 PE=1 SV=2 - [IF2B1_HUMAN]	1.04	1	1	1	1	1.19	1.38	1.21	1.40					577	63.4	9.20
P05019	Insulin-like growth factor I OS=Homo sapiens GN=IGF1 PE=1 SV=1 - [IGF1_HUMAN]	21.54	1	4	4	17					1.40	1.34	1.27	1.30	195	21.8	9.72
P01344	Insulin-like growth factor II OS=Homo sapiens GN=IGF2 PE=1 SV=1 - [IGF2_HUMAN]	17.22	3	2	3	16	1.16	1.23	1.16	1.25	1.31	0.98	1.50	1.36	180	20.1	9.32
P08833	Insulin-like growth factor-binding protein 1 OS=Homo sapiens GN=IGFBP1 PE=1 SV=1 - [IBP1_HUMAN]	5.41	1	1	1	3					1.25	1.31	1.25	1.30	259	27.9	5.19
P18065	Insulin-like growth factor-binding protein 2 OS=Homo sapiens GN=IGFBP2 PE=1 SV=2 - [IBP2_HUMAN]	19.69	1	6	6	18	1.09	0.95	0.96	0.85	1.23	1.21	1.23	1.19	325	34.8	7.50
P17936	Insulin-like growth factor-binding protein 3 OS=Homo sapiens GN=IGFBP3 PE=1 SV=2 - [IBP3_HUMAN]	37.80	9	11	12	144	1.01	0.85	0.97	0.87	1.05	0.90	1.04	0.92	291	31.7	8.69
P22692	Insulin-like growth factor-binding protein 4 OS=Homo sapiens GN=IGFBP4 PE=1 SV=2 - [IBP4_HUMAN]	32.17	1	7	7	25	0.90	1.01	0.98	1.11	1.10	1.02	1.07	1.06	258	27.9	7.15
P24593	Insulin-like growth factor-binding protein 5 OS=Homo sapiens GN=IGFBP5 PE=1 SV=1 - [IBP5_HUMAN]	20.22	1	5	5	21					1.03	1.08	1.07	1.02	272	30.6	8.21
P24592	Insulin-like growth factor-binding protein 6 OS=Homo sapiens GN=IGFBP6 PE=1 SV=1 - [IBP6_HUMAN]	37.08	2	6	7	17	0.82	0.89	0.81	0.87	1.29	1.10	1.15	1.06	240	25.3	7.81
Q16270	Insulin-like growth factor-binding protein 7 OS=Homo sapiens GN=IGFBP7 PE=1 SV=1 - [IBP7_HUMAN]	10.64	1	2	2	5					1.47	1.33	1.29	1.16	282	29.1	7.90
P35858	Insulin-like growth factor-binding protein complex acid labile subunit OS=Homo sapiens GN=IGFALS PE=1 SV=1 - [ALS_HUMAN]	49.59	1	23	23	438	1.09	1.09	1.05	1.08	0.97	0.96	0.98	0.98	605	66.0	6.79
Q9Y287	Integral membrane protein 2B OS=Homo sapiens GN=ITM2B PE=1 SV=1 - [ITM2B_HUMAN]	8.65	1	2	2	6					1.86	1.63	1.65	1.45	266	30.3	5.14
P98153	Integral membrane protein DGCR2/IDD OS=Homo sapiens GN=DGCR2 PE=1 SV=1 - [IDD_HUMAN]	6.73	1	1	1	2	0.61	0.49			1.60	1.38	0.83	0.72	550	60.8	5.30
Q8N201	Integrator complex subunit 1 OS=Homo sapiens GN=INTS1 PE=1 SV=2 - [INT1_HUMAN]	0.41	1	1	1	1	1.02	1.53	0.85	1.26					2190	244.1	6.13
Q9NVR2	Integrator complex subunit 10 OS=Homo sapiens GN=INTS10 PE=1 SV=2 - [INT10_HUMAN]	2.82	1	2	2	3	0.84	0.87	0.85	0.89	1.25	1.22	1.10	1.08	710	82.2	7.44
Q5TA45	Integrator complex subunit 11 OS=Homo sapiens GN=CPSF3L PE=1 SV=2 - [INT11_HUMAN]	2.00	1	1	1	2					0.77	0.76	0.81	0.80	600	67.6	8.06
Q9H0H0	Integrator complex subunit 2 OS=Homo sapiens GN=INTS2 PE=1 SV=2 - [INT2_HUMAN]	1.08	1	1	1	1					0.43	0.37	0.41	0.35	1204	134.2	6.05
Q96HW7	Integrator complex subunit 4 OS=Homo sapiens GN=INTS4 PE=1 SV=2 - [INT4_HUMAN]	1.87	1	1	1	1					0.69	0.62	0.66	0.60	963	108.1	6.44
Q9UKX5	Integrin alpha-11 OS=Homo sapiens GN=ITGA11 PE=1 SV=2 - [ITA11_HUMAN]	0.76	1	1	1	1					1.40	0.93	1.49	0.99	1188	133.4	6.70
P17301	Integrin alpha-2 OS=Homo sapiens GN=ITGA2 PE=1 SV=1 - [ITA2_HUMAN]	5.59	1	6	6	9	0.88	0.98	0.92	1.00	1.28	1.02	1.14	0.91	1181	129.2	5.31
P26006	Integrin alpha-3 OS=Homo sapiens GN=ITGA3 PE=1 SV=5 - [ITA3_HUMAN]	1.43	1	1	1	6	1.03	1.02	1.08	1.07					1051	116.5	6.77
P13612	Integrin alpha-4 OS=Homo sapiens GN=ITGA4 PE=1 SV=3 - [ITA4_HUMAN]	3.39	1	2	2	2	0.87	1.12	0.97	1.25	1.06	0.69	1.24	0.81	1032	114.8	6.48
P23229	Integrin alpha-6 OS=Homo sapiens GN=ITGA6 PE=1 SV=4 - [ITA6_HUMAN]	0.71	1	1	1	2	0.76	0.81	0.67	0.72					1130	126.6	6.80

Q13683	Integrin alpha-7 OS=Homo sapiens GN=ITGA7 PE=1 SV=3 - [ITA7_HUMAN]	0.51	1	1	1	1					1.03	0.94	1.00	0.90	1181	128.9	5.73
Q13349	Integrin alpha-D OS=Homo sapiens GN=ITGAD PE=1 SV=2 - [ITAD_HUMAN]	1.21	1	1	1	1					0.92	0.72	0.94	0.74	1161	126.7	5.77
P38570	Integrin alpha-E OS=Homo sapiens GN=ITGAE PE=1 SV=3 - [ITAE_HUMAN]	1.27	1	1	1	1	0.84	0.82	0.79	0.78					1179	130.1	5.74
P08514	Integrin alpha-IIb OS=Homo sapiens GN=ITGA2B PE=1 SV=3 - [ITA2B_HUMAN]	20.89	1	15	15	31	0.78	0.81	0.84	0.81					1039	113.3	5.38
P11215	Integrin alpha-M OS=Homo sapiens GN=ITGAM PE=1 SV=2 - [ITAM_HUMAN]	6.86	1	6	6	13	1.17	1.26	1.12	1.27					1152	127.1	7.23
P06756	Integrin alpha-V OS=Homo sapiens GN=ITGAV PE=1 SV=2 - [ITAV_HUMAN]	4.29	1	2	2	2					1.03	0.79	0.76	0.65	1048	116.0	5.68
P20702	Integrin alpha-X OS=Homo sapiens GN=ITGAX PE=1 SV=3 - [ITAX_HUMAN]	2.67	1	2	2	3	1.09	1.06	0.97	0.95	0.72	0.74	0.69	0.70	1163	127.7	6.64
P05556	Integrin beta-1 OS=Homo sapiens GN=ITGB1 PE=1 SV=2 - [ITB1_HUMAN]	12.28	1	6	6	16	1.08	1.02	1.10	1.04					798	88.4	5.39
Q9UKP3	Integrin beta-1-binding protein 2 OS=Homo sapiens GN=ITGB1BP2 PE=2 SV=1 - [ITBP2_HUMAN]	6.63	1	1	1	1					1.06	1.92	0.55	1.00	347	38.4	5.16
P05107	Integrin beta-2 OS=Homo sapiens GN=ITGB2 PE=1 SV=2 - [ITB2_HUMAN]	2.47	1	2	2	4	1.26	1.31	1.14	1.19					769	84.7	6.95
P05106	Integrin beta-3 OS=Homo sapiens GN=ITGB3 PE=1 SV=2 - [ITB3_HUMAN]	7.11	1	4	4	8	1.03	0.83	0.97	0.77					788	87.0	5.24
P18564	Integrin beta-6 OS=Homo sapiens GN=ITGB6 PE=1 SV=2 - [ITB6_HUMAN]	1.14	1	1	1	9	0.71	0.75	0.69	0.73	0.59	0.75	0.59	0.69	788	85.9	5.55
Q9H0C8	Integrin-linked kinase-associated serine/threonine phosphatase 2C OS=Homo sapiens GN=ILKAP PE=1 SV=1 - [ILKAP_HUMAN]	9.69	1	2	2	3	1.12	1.06	1.06	1.00					392	42.9	7.09
Q13418	Integrin-linked protein kinase OS=Homo sapiens GN=ILK PE=1 SV=2 - [ILK_HUMAN]	10.62	1	5	5	17	0.75	0.73	0.69	0.78	2.08	2.14	1.87	1.95	452	51.4	8.07
Q8WWA0	Intelectin-1 OS=Homo sapiens GN=ITLN1 PE=1 SV=1 - [ITLN1_HUMAN]	12.78	2	4	4	9	1.01	0.98	1.04	1.00					313	34.9	6.01
P19827	Inter-alpha-trypsin inhibitor heavy chain H1 OS=Homo sapiens GN=ITIH1 PE=1 SV=3 - [ITIH1_HUMAN]	55.10	1	41	41	2870	1.09	1.10	1.11	1.10	1.14	1.23	1.14	1.26	911	101.3	6.79
P19823	Inter-alpha-trypsin inhibitor heavy chain H2 OS=Homo sapiens GN=ITIH2 PE=1 SV=2 - [ITIH2_HUMAN]	56.13	1	51	51	2045	1.12	1.14	1.19	1.20	1.23	1.23	1.24	1.25	946	106.4	6.86
Q06033	Inter-alpha-trypsin inhibitor heavy chain H3 OS=Homo sapiens GN=ITIH3 PE=1 SV=2 - [ITIH3_HUMAN]	39.21	1	28	28	532	0.97	0.92	0.96	0.95	1.04	1.05	1.04	1.07	890	99.8	5.74
Q14624	Inter-alpha-trypsin inhibitor heavy chain H4 OS=Homo sapiens GN=ITIH4 PE=1 SV=4 - [ITIH4_HUMAN]	67.10	2	63	63	2797	1.27	1.30	1.26	1.28	1.29	1.34	1.29	1.37	930	103.3	6.98
P05362	Intercellular adhesion molecule 1 OS=Homo sapiens GN=ICAM1 PE=1 SV=2 - [ICAM1_HUMAN]	21.99	1	10	10	23	1.15	1.08	1.09	1.02	0.76	0.77	0.75	0.75	532	57.8	7.99
P13598	Intercellular adhesion molecule 2 OS=Homo sapiens GN=ICAM2 PE=1 SV=2 - [ICAM2_HUMAN]	23.27	1	6	6	42	1.43	1.35	1.43	1.33	1.12	1.04	0.90	0.90	275	30.6	7.43
P32942	Intercellular adhesion molecule 3 OS=Homo sapiens GN=ICAM3 PE=1 SV=2 - [ICAM3_HUMAN]	6.76	1	3	3	5	1.00	0.91	0.95	0.87					547	59.5	5.43
Q9UMF0	Intercellular adhesion molecule 5 OS=Homo sapiens GN=ICAM5 PE=1 SV=3 - [ICAM5_HUMAN]	2.49	1	1	1	1	0.82	0.66	0.71	0.57					924	97.1	5.95
Q96BM0	Interferon alpha-inducible protein 27-like protein 1 OS=Homo sapiens GN=IFI27L1 PE=2 SV=1 - [I27L1_HUMAN]	33.65	1	1	1	2	1.00	0.84	1.04	0.88					104	9.5	9.72
Q86WN2	Interferon epsilon OS=Homo sapiens GN=IFNE PE=2 SV=1 - [IFNE_HUMAN]	7.21	1	1	1	19	1.57	1.63	1.81	1.81					208	24.4	8.57
P38484	Interferon gamma receptor 2 OS=Homo sapiens GN=IFNGR2 PE=1 SV=2 - [INGR2_HUMAN]	3.86	1	1	1	1	1.09	1.03	1.13	1.07					337	37.8	5.47
Q14653	Interferon regulatory factor 3 OS=Homo sapiens GN=IRF3 PE=1 SV=1 - [IRF3_HUMAN]	4.92	1	1	1	1					0.78	0.82	0.77	0.80	427	47.2	5.34

Q02556	Interferon regulatory factor 8 OS=Homo sapiens GN=IRF8 PE=1 SV=2 - [IRF8_HUMAN]	2.82	1	1	1	1					1.12	1.13	1.07	1.09	426	48.3	6.77
Q00978	Interferon regulatory factor 9 OS=Homo sapiens GN=IRF9 PE=1 SV=1 - [IRF9_HUMAN]	2.80	1	1	1	1					1.04	1.01	1.01	0.99	393	43.7	5.77
P20591	Interferon-induced GTP-binding protein Mx1 OS=Homo sapiens GN=MX1 PE=1 SV=4 - [MX1_HUMAN]	0.91	1	1	1	1					1.19	0.81	1.08	0.74	662	75.5	5.83
Q7Z2Y8	Interferon-induced very large GTPase 1 OS=Homo sapiens GN=GVINP1 PE=2 SV=2 - [GVIN1_HUMAN]	1.82	1	3	3	5	0.95	0.95	0.89	0.89					2422	278.9	6.55
Q12905	Interleukin enhancer-binding factor 2 OS=Homo sapiens GN=ILF2 PE=1 SV=2 - [ILF2_HUMAN]	2.82	1	1	1	1					2.21	1.88	2.25	1.92	390	43.0	5.26
Q12906	Interleukin enhancer-binding factor 3 OS=Homo sapiens GN=ILF3 PE=1 SV=3 - [ILF3_HUMAN]	3.47	1	1	1	1	1.20	1.39	1.10	1.28					894	95.3	8.76
Q9NPH3	Interleukin-1 receptor accessory protein OS=Homo sapiens GN=IL1RAP PE=1 SV=2 - [IL1AP_HUMAN]	14.56	1	10	10	59	1.31	1.27	1.23	1.21	1.03	0.97	0.95	0.95	570	65.4	8.12
Q9NZN1	Interleukin-1 receptor accessory protein-like 1 OS=Homo sapiens GN=IL1RAPL1 PE=1 SV=2 - [IRPL1_HUMAN]	1.72	1	1	1	2	0.95	1.02	0.96	1.04					696	79.9	6.23
P27930	Interleukin-1 receptor type 2 OS=Homo sapiens GN=IL1R2 PE=1 SV=1 - [IL1R2_HUMAN]	2.26	1	1	1	2	1.22	1.32	1.21	1.33					398	45.4	7.90
Q9HB29	Interleukin-1 receptor-like 2 OS=Homo sapiens GN=IL1RL2 PE=2 SV=2 - [ILRL2_HUMAN]	2.78	2	2	2	2	0.99	1.11	1.13	1.28					575	65.4	7.46
P20809	Interleukin-11 OS=Homo sapiens GN=IL11 PE=1 SV=1 - [IL11_HUMAN]	16.58	1	1	1	1					0.51	0.69	0.64	0.87	199	21.4	10.62
Q99665	Interleukin-12 receptor subunit beta-2 OS=Homo sapiens GN=IL12RB2 PE=1 SV=1 - [I12R2_HUMAN]	1.74	1	1	1	1					0.88	1.05	0.77	0.92	862	97.1	7.75
P78552	Interleukin-13 receptor subunit alpha-1 OS=Homo sapiens GN=IL13RA1 PE=1 SV=1 - [I13R1_HUMAN]	5.85	1	1	1	1	1.24	1.43	0.94	1.08					427	48.7	6.01
Q14627	Interleukin-13 receptor subunit alpha-2 OS=Homo sapiens GN=IL13RA2 PE=1 SV=1 - [I13R2_HUMAN]	2.37	1	1	1	3					0.94	0.97	0.92	0.91	380	44.1	4.97
P40933	Interleukin-15 OS=Homo sapiens GN=IL15 PE=1 SV=1 - [IL15_HUMAN]	22.22	1	1	1	1					0.95	0.71	1.05	0.78	162	18.1	5.27
Q8NFR9	Interleukin-17 receptor E OS=Homo sapiens GN=IL17RE PE=1 SV=1 - [I17RE_HUMAN]	3.45	1	1	1	1	0.81	0.81	0.83	0.84					667	74.8	9.22
Q8TAD2	Interleukin-17D OS=Homo sapiens GN=IL17D PE=2 SV=1 - [IL17D_HUMAN]	7.92	1	1	1	1					0.78	0.94	0.93	1.13	202	21.9	8.98
O95998	Interleukin-18-binding protein OS=Homo sapiens GN=IL18BP PE=1 SV=2 - [I18BP_HUMAN]	14.95	1	3	3	7	1.46	1.45	1.37	1.37	0.80	0.62	0.98	0.77	194	21.1	7.39
P60568	Interleukin-2 OS=Homo sapiens GN=IL2 PE=1 SV=1 - [IL2_HUMAN]	8.50	1	1	1	1					1.88	1.38	1.61	1.18	153	17.6	7.80
P14784	Interleukin-2 receptor subunit beta OS=Homo sapiens GN=IL2RB PE=1 SV=1 - [IL2RB_HUMAN]	3.09	1	1	1	1	0.65	0.59	0.71	0.65					551	61.1	5.05
Q9UHF4	Interleukin-20 receptor subunit alpha OS=Homo sapiens GN=IL20RA PE=1 SV=2 - [I20RA_HUMAN]	2.71	1	1	1	2					1.27	1.04	1.37	1.12	553	62.4	4.84
Q13007	Interleukin-24 OS=Homo sapiens GN=IL24 PE=1 SV=1 - [IL24_HUMAN]	13.59	1	1	1	1					0.69	0.61	2.66	2.35	206	23.8	8.72
Q14213	Interleukin-27 subunit beta OS=Homo sapiens GN=EBI3 PE=1 SV=2 - [IL27B_HUMAN]	4.80	1	1	1	3	3.21	3.47	3.32	3.63	4.34	5.11	4.11	4.84	229	25.4	9.25
Q9UHA7	Interleukin-36 alpha OS=Homo sapiens GN=IL36A PE=1 SV=1 - [IL36A_HUMAN]	7.59	1	1	1	1					0.56	0.59	0.70	0.75	158	17.7	6.30
P05231	Interleukin-6 OS=Homo sapiens GN=IL6 PE=1 SV=1 - [IL6_HUMAN]	8.49	1	1	1	1	0.87	0.54	1.02	0.64					212	23.7	6.57
P08887	Interleukin-6 receptor subunit alpha OS=Homo sapiens GN=IL6R PE=1 SV=1 - [IL6RA_HUMAN]	20.73	1	5	5	11	0.98	1.00	1.02	1.05	1.09	1.22	0.98	1.10	468	51.5	8.22
P40189	Interleukin-6 receptor subunit beta OS=Homo sapiens GN=IL6ST PE=1 SV=2 - [IL6RB_HUMAN]	2.18	1	1	1	5					1.09	1.09	0.93	0.93	918	103.5	5.95

P10145	Interleukin-8 OS=Homo sapiens GN=IL8 PE=1 SV=1 - [IL8_HUMAN]	9.09	1	1	1	2					1.86	1.98	1.77	1.88	99	11.1	8.84
Q9BZV3	Interphotoreceptor matrix proteoglycan 2 OS=Homo sapiens GN=IMPG2 PE=1 SV=3 - [IMPG2_HUMAN]	0.56	1	1	1	1					1.09	0.79	0.87	0.63	1241	138.5	4.61
Q15811	Intersectin-1 OS=Homo sapiens GN=ITSN1 PE=1 SV=3 - [ITSN1_HUMAN]	0.46	1	1	1	5	1.27	1.19	1.33	1.25	0.89	1.08	0.97	1.20	1721	195.3	7.77
P03956	Interstitial collagenase OS=Homo sapiens GN=MMP1 PE=1 SV=3 - [MMP1_HUMAN]	3.20	1	1	1	1					1.00	0.84	0.92	0.77	469	54.0	6.96
Q2M1V0	Intestine-specific homeobox OS=Homo sapiens GN=ISX PE=2 SV=2 - [ISX_HUMAN]	8.98	1	2	2	3	1.04	1.16	1.01	1.14	2.01	2.41	2.09	2.50	245	27.0	8.56
Q9HBG6	Intraflagellar transport protein 122 homolog OS=Homo sapiens GN=IFT122 PE=1 SV=2 - [IF122_HUMAN]	3.30	1	2	2	3	0.86	0.82	0.80	0.76					1241	141.7	6.49
Q96RY7	Intraflagellar transport protein 140 homolog OS=Homo sapiens GN=IFT140 PE=1 SV=1 - [IF140_HUMAN]	1.30	1	1	1	1	1.97	1.24	1.62	1.03					1462	165.1	6.04
Q9UG01	Intraflagellar transport protein 172 homolog OS=Homo sapiens GN=IFT172 PE=1 SV=2 - [IF172_HUMAN]	1.37	1	2	2	2					1.10	1.26	0.97	1.11	1749	197.5	6.13
Q13099	Intraflagellar transport protein 88 homolog OS=Homo sapiens GN=IFT88 PE=2 SV=2 - [IFT88_HUMAN]	5.16	1	2	2	2	1.59	1.63	1.63	1.69					833	94.2	6.54
O60306	Intron-binding protein aquarius OS=Homo sapiens GN=AQR PE=1 SV=4 - [AQR_HUMAN]	1.55	1	1	1	1					0.85	0.84	0.79	0.78	1485	171.2	6.37
Q27J81	Inverted formin-2 OS=Homo sapiens GN=INF2 PE=1 SV=2 - [INF2_HUMAN]	3.44	1	3	3	5	0.78	1.00	0.71	0.91					1249	135.5	5.38
Q9NPI9	Inward rectifier potassium channel 16 OS=Homo sapiens GN=KCNJ16 PE=2 SV=1 - [IRK16_HUMAN]	1.91	1	1	1	1	0.88	0.91	1.08	1.12					418	47.9	7.87
Q8N2Y8	Iporin OS=Homo sapiens GN=RUSC2 PE=1 SV=3 - [RUSC2_HUMAN]	1.65	1	1	1	5	0.93	0.96	0.83	0.88	2.08	1.19	1.73	0.99	1516	161.1	6.62
Q8NA54	IQ and ubiquitin-like domain-containing protein OS=Homo sapiens GN=IQUB PE=1 SV=2 - [IQUB_HUMAN]	2.02	1	1	1	1					0.90	0.72	1.55	1.24	791	92.5	6.67
Q15051	IQ calmodulin-binding motif-containing protein 1 OS=Homo sapiens GN=IQCB1 PE=1 SV=1 - [IQCB1_HUMAN]	5.18	1	2	2	14					0.65	0.85	0.63	0.77	598	68.9	9.10
A8MTL0	IQ domain-containing protein F5 OS=Homo sapiens GN=IQCF5 PE=2 SV=2 - [IQCF5_HUMAN]	4.05	1	1	1	3					6.36	1.25		####	148	18.0	10.01
Q9H095	IQ domain-containing protein G OS=Homo sapiens GN=IQCG PE=2 SV=1 - [IQCG_HUMAN]	5.19	1	2	2	2					1.36	1.37	1.28	1.29	443	51.9	6.52
Q5JU85	IQ motif and SEC7 domain-containing protein 2 OS=Homo sapiens GN=IQSEC2 PE=1 SV=1 - [IQEC2_HUMAN]	1.62	1	2	2	3					0.59	0.65	0.68	0.79	1478	161.6	8.56
Q8IWL3	Iron-sulfur cluster co-chaperone protein HscB, mitochondrial OS=Homo sapiens GN=HSCB PE=1 SV=3 - [HSC20_HUMAN]	7.66	1	2	2	3	0.82	0.78	0.86	0.83					235	27.4	7.69
Q8TB37	Iron-sulfur protein NUBPL OS=Homo sapiens GN=NUBPL PE=1 SV=3 - [NUBPL_HUMAN]	4.08	1	1	1	2	0.77	0.94	0.74	0.90					319	34.1	9.04
P78411	Iroquois-class homeodomain protein IRX-5 OS=Homo sapiens GN=IRX5 PE=2 SV=3 - [IRX5_HUMAN]	3.93	6	2	2	2	1.21	1.00	1.23	1.02	0.08	0.09	0.38	0.41	483	50.3	6.47
Q9UKU7	Isobutyryl-CoA dehydrogenase, mitochondrial OS=Homo sapiens GN=ACAD8 PE=1 SV=1 - [ACAD8_HUMAN]	4.34	1	1	1	2					0.73	0.74	0.73	0.75	415	45.0	7.85
P50213	Isocitrate dehydrogenase [NAD] subunit alpha, mitochondrial OS=Homo sapiens GN=IDH3A PE=1 SV=1 - [IDH3A_HUMAN]	2.46	1	1	1	1					0.77	0.26	0.90	0.30	366	39.6	6.92
P26440	Isovaleryl-CoA dehydrogenase, mitochondrial OS=Homo sapiens GN=IVD PE=1 SV=1 - [IVD_HUMAN]	3.07	1	1	1	1	1.33	1.25	1.43	1.34					423	46.3	8.19
Q8IYV9	Izumo sperm-egg fusion protein 1 OS=Homo sapiens GN=IZUMO1 PE=1 SV=2 - [IZUM1_HUMAN]	6.57	1	1	1	1	1.44	1.30	0.87	0.79					350	38.9	6.54
Q96N16	Janus kinase and microtubule-interacting protein 1 OS=Homo sapiens GN=JAKMIP1 PE=1 SV=1 - [JKIP1_HUMAN]	1.92	1	1	1	3	0.42	1.25	0.52	1.57					626	73.2	6.10
Q9H9V9	JmjC domain-containing protein 4 OS=Homo sapiens GN=JMJD4 PE=2 SV=2 - [JMJD4_HUMAN]	2.81	1	1	1	1					0.82	0.79	0.74	0.72	463	52.5	6.21

Q8N9B5	Junction-mediating and -regulatory protein OS=Homo sapiens GN=JMY PE=1 SV=2 - [JMY_HUMAN]	0.71	1	1	1	1	1.42	1.39	1.42	1.41					988	111.4	6.18
Q9Y624	Junctional adhesion molecule A OS=Homo sapiens GN=F11R PE=1 SV=1 - [JAM1_HUMAN]	2.34	1	1	1	1					0.07	0.97	0.33	4.62	299	32.6	7.90
Q9P266	Junctional protein associated with coronary artery disease OS=Homo sapiens GN=KIAA1462 PE=1 SV=3 - [JCAD_HUMAN]	1.18	1	1	1	1	0.35	0.30	0.45	0.38					1359	148.3	7.09
Q9BR39	Junctophilin-2 OS=Homo sapiens GN=JPH2 PE=1 SV=2 - [JPH2_HUMAN]	2.01	1	1	1	1	0.73	0.29	0.44	0.18					696	74.2	8.72
O60229	Kalirin OS=Homo sapiens GN=KALRN PE=1 SV=2 - [KALRN_HUMAN]	1.71	1	3	3	5	0.72	0.04	0.87	0.05	1.19	1.25	1.23	1.28	2985	340.0	6.07
Q92876	Kallikrein-6 OS=Homo sapiens GN=KLK6 PE=1 SV=1 - [KLK6_HUMAN]	4.92	1	1	1	3					1.12	1.09	1.12	1.09	244	26.8	7.44
P49862	Kallikrein-7 OS=Homo sapiens GN=KLK7 PE=1 SV=1 - [KLK7_HUMAN]	9.88	1	1	1	3	1.15	2.07	0.69	1.24	1.20		1.54		253	27.5	8.47
P29622	Kallistatin OS=Homo sapiens GN=SERPINA4 PE=1 SV=3 - [KAIN_HUMAN]	52.22	1	21	21	318	1.07	1.11	1.11	1.14	1.00	1.14	0.98	1.10	427	48.5	7.75
Q7Z3B3	KAT8 regulatory NSL complex subunit 1 OS=Homo sapiens GN=KANSL1 PE=1 SV=2 - [KANL1_HUMAN]	1.54	1	1	1	1	0.77	0.82	0.79	0.84					1105	121.0	8.81
A0AUZ9	KAT8 regulatory NSL complex subunit 1-like protein OS=Homo sapiens GN=KANSL1L PE=1 SV=2 - [KAL1L_HUMAN]	0.61	1	1	1	1					0.77	1.04	0.74	1.02	987	112.2	8.44
Q9P2N6	KAT8 regulatory NSL complex subunit 3 OS=Homo sapiens GN=KANSL3 PE=1 SV=2 - [KANL3_HUMAN]	3.65	1	1	1	10	0.83	0.48	0.95	0.55					904	95.9	9.39
Q8IYD2	Kelch domain-containing protein 8A OS=Homo sapiens GN=KLHDC8A PE=2 SV=2 - [KLD8A_HUMAN]	4.29	1	1	1	2	1.00	1.08	1.22	1.34					350	38.8	9.26
Q8IXV7	Kelch domain-containing protein 8B OS=Homo sapiens GN=KLHDC8B PE=2 SV=1 - [KLD8B_HUMAN]	5.08	1	1	1	1	0.56	0.50	0.62	0.54					354	37.7	8.27
Q8NAB2	Kelch repeat and BTB domain-containing protein 3 OS=Homo sapiens GN=KBTBD3 PE=2 SV=2 - [KBTB3_HUMAN]	2.14	1	1	1	1					1.05	1.26	1.09	1.31	608	69.3	5.39
Q8WVZ9	Kelch repeat and BTB domain-containing protein 7 OS=Homo sapiens GN=KBTBD7 PE=1 SV=1 - [KBTB7_HUMAN]	9.65	2	3	3	7	0.90	0.86	1.00	0.97					684	77.1	5.57
Q8NFY9	Kelch repeat and BTB domain-containing protein 8 OS=Homo sapiens GN=KBTBD8 PE=2 SV=2 - [KBTB8_HUMAN]	2.16	1	1	1	2	0.69	0.59	0.71	0.61					601	68.8	6.29
Q53G59	Kelch-like protein 12 OS=Homo sapiens GN=KLHL12 PE=1 SV=2 - [KLH12_HUMAN]	1.76	1	1	1	1					1.12	0.64	1.30	0.74	568	63.2	5.44
Q9P2G3	Kelch-like protein 14 OS=Homo sapiens GN=KLHL14 PE=1 SV=2 - [KLH14_HUMAN]	2.87	1	1	1	2	1.13	0.68	0.93	0.56					628	70.7	6.68
O95198	Kelch-like protein 2 OS=Homo sapiens GN=KLHL2 PE=1 SV=2 - [KLHL2_HUMAN]	1.69	1	1	1	1					1.12	0.75	0.81	0.54	593	65.9	6.74
Q9UJP4	Kelch-like protein 21 OS=Homo sapiens GN=KLHL21 PE=1 SV=4 - [KLH21_HUMAN]	1.68	1	1	1	1					3.00	7.04	2.10	4.95	597	66.6	5.36
Q8NBE8	Kelch-like protein 23 OS=Homo sapiens GN=KLHL23 PE=2 SV=1 - [KLH23_HUMAN]	1.79	1	1	1	1					0.57	0.90	1.19	1.88	558	63.9	5.62
Q6TFL4	Kelch-like protein 24 OS=Homo sapiens GN=KLHL24 PE=2 SV=1 - [KLH24_HUMAN]	2.33	1	1	1	1	1.05	1.04	1.03	1.02					600	68.3	6.39
Q9H511	Kelch-like protein 31 OS=Homo sapiens GN=KLHL31 PE=2 SV=1 - [KLH31_HUMAN]	4.57	1	1	1	4	1.09	1.48	1.06	1.46	1.55	1.42	1.28	1.17	634	70.2	6.73
Q8N239	Kelch-like protein 34 OS=Homo sapiens GN=KLHL34 PE=2 SV=1 - [KLH34_HUMAN]	6.52	1	1	1	1					1.11	1.35	1.09	1.33	644	70.6	5.60
A6NCN2	Keratin-81-like protein OS=Homo sapiens PE=2 SV=3 - [KT81L_HUMAN]	8.02	1	1	3	7					7.98	7.39	1.78	1.65	486	53.4	6.35
Q8N1A0	Keratin-like protein KRT222 OS=Homo sapiens GN=KRT222 PE=2 SV=1 - [KT222_HUMAN]	9.83	1	1	1	1	1.05	1.06	1.06	1.07					295	34.1	5.87
Q14525	Keratin, type I cuticular Ha3-II OS=Homo sapiens GN=KRT33B PE=2 SV=3 - [KT33B_HUMAN]	3.71	9	1	2	11					6.16	4.64	1.70	1.28	404	46.2	4.84

Q92764	Keratin, type I cuticular Ha5 OS=Homo sapiens GN=KRT35 PE=2 SV=5 - [KRT35_HUMAN]	5.27	4	1	2	11					0.57	1.09	0.68	1.30	455	50.3	4.91
P13645	Keratin, type I cytoskeletal 10 OS=Homo sapiens GN=KRT10 PE=1 SV=6 - [K1C10_HUMAN]	45.55	9	19	22	235	0.79	0.75	0.84	0.77	0.98	0.92	1.44	1.29	584	58.8	5.21
P02533	Keratin, type I cytoskeletal 14 OS=Homo sapiens GN=KRT14 PE=1 SV=4 - [K1C14_HUMAN]	29.03	6	2	15	92	0.81	0.71	0.70	0.61	1.29	0.73	2.19	1.20	472	51.5	5.16
P08779	Keratin, type I cytoskeletal 16 OS=Homo sapiens GN=KRT16 PE=1 SV=4 - [K1C16_HUMAN]	34.04	6	5	16	93	0.87	0.76	1.17	1.09	1.71	0.35	2.05	0.47	473	51.2	5.05
Q04695	Keratin, type I cytoskeletal 17 OS=Homo sapiens GN=KRT17 PE=1 SV=2 - [K1C17_HUMAN]	19.21	5	3	9	29	1.00	0.90	0.71	0.64	1.37	0.84	1.84	1.13	432	48.1	5.02
Q7Z3Y9	Keratin, type I cytoskeletal 26 OS=Homo sapiens GN=KRT26 PE=1 SV=2 - [K1C26_HUMAN]	5.34	1	1	2	27	1.47	1.96	1.09	1.46					468	51.9	4.92
P35527	Keratin, type I cytoskeletal 9 OS=Homo sapiens GN=KRT9 PE=1 SV=3 - [K1C9_HUMAN]	46.23	1	20	21	195	0.80	1.07	0.85	1.15	0.87	0.88	1.10	1.14	623	62.0	5.24
O43790	Keratin, type II cuticular Hb6 OS=Homo sapiens GN=KRT86 PE=1 SV=1 - [KRT86_HUMAN]	8.44	4	2	5	21	2.07	1.76	1.92	1.64	3.52	3.30	1.69	1.57	486	53.5	5.66
P04264	Keratin, type II cytoskeletal 1 OS=Homo sapiens GN=KRT1 PE=1 SV=6 - [K2C1_HUMAN]	46.58	2	23	30	390	0.75	0.81	0.87	0.97	0.94	0.84	1.29	1.15	644	66.0	8.12
Q7Z794	Keratin, type II cytoskeletal 1b OS=Homo sapiens GN=KRT77 PE=1 SV=3 - [K2C1B_HUMAN]	9.00	1	2	6	40	1.07		1.75		0.95	0.61	0.91	0.58	578	61.9	5.99
P35908	Keratin, type II cytoskeletal 2 epidermal OS=Homo sapiens GN=KRT2 PE=1 SV=2 - [K22E_HUMAN]	38.03	2	13	21	200	0.88	0.95	0.83	0.84	0.91	0.93	1.12	1.22	639	65.4	8.00
P13647	Keratin, type II cytoskeletal 5 OS=Homo sapiens GN=KRT5 PE=1 SV=3 - [K2C5_HUMAN]	26.10	6	6	19	85	0.76	0.81	0.92	0.98	1.12	0.99	1.73	1.29	590	62.3	7.74
P02538	Keratin, type II cytoskeletal 6A OS=Homo sapiens GN=KRT6A PE=1 SV=3 - [K2C6A_HUMAN]	26.77	6	2	18	75	0.69	0.50	0.47	0.34					564	60.0	8.00
P04259	Keratin, type II cytoskeletal 6B OS=Homo sapiens GN=KRT6B PE=1 SV=5 - [K2C6B_HUMAN]	25.18	5	1	17	74					2.42	2.92	2.18	2.63	564	60.0	8.00
P08729	Keratin, type II cytoskeletal 7 OS=Homo sapiens GN=KRT7 PE=1 SV=5 - [K2C7_HUMAN]	12.37	3	1	5	23					1.29	1.01	1.09	0.85	469	51.4	5.48
Q8N1N4	Keratin, type II cytoskeletal 78 OS=Homo sapiens GN=KRT78 PE=2 SV=2 - [K2C78_HUMAN]	11.35	1	1	4	14	0.25		0.52						520	56.8	6.02
P05787	Keratin, type II cytoskeletal 8 OS=Homo sapiens GN=KRT8 PE=1 SV=7 - [K2C8_HUMAN]	18.01	3	3	11	45					1.90	1.39	2.07	1.51	483	53.7	5.59
P60985	Keratinocyte differentiation-associated protein OS=Homo sapiens GN=KRTDAP PE=1 SV=1 - [KTDAP_HUMAN]	27.27	1	2	2	11					2.58	2.57	2.47	2.45	99	11.0	7.42
Q53RY4	Keratinocyte-associated protein 3 OS=Homo sapiens GN=KRTCAP3 PE=2 SV=1 - [KCP3_HUMAN]	2.92	1	1	1	1					0.72	0.59	0.70	0.57	240	25.6	7.88
Q5SVS4	Kidney mitochondrial carrier protein 1 OS=Homo sapiens GN=SLC25A30 PE=2 SV=1 - [KMCP1_HUMAN]	8.93	1	1	1	2	0.87	1.03	0.84	1.02					291	32.5	9.44
Q6ZWJ8	Kielin/chordin-like protein OS=Homo sapiens GN=KCP PE=2 SV=2 - [KCP_HUMAN]	2.20	1	2	2	3					0.89	0.84	0.79	0.75	1503	159.8	5.74
Q96EK5	KIF1-binding protein OS=Homo sapiens GN=KIAA1279 PE=1 SV=1 - [KBP_HUMAN]	0.97	1	1	1	1					1.52	1.49	1.60	1.58	621	71.8	5.49
P43626	Killer cell immunoglobulin-like receptor 2DL1 OS=Homo sapiens GN=KIR2DL1 PE=1 SV=1 - [KI2L1_HUMAN]	1.44	3	1	1	2					0.64	0.62	0.39	1.70	348	38.5	6.83
Q8N109	Killer cell immunoglobulin-like receptor 2DL5A OS=Homo sapiens GN=KIR2DL5A PE=2 SV=1 - [KI2LA_HUMAN]	2.93	2	1	1	2	1.34	0.71	1.31	0.70					375	40.7	8.10
D3W0D1	Killer cell lectin-like receptor subfamily F member 2 OS=Homo sapiens GN=KLRF2 PE=1 SV=1 - [KLRF2_HUMAN]	5.80	1	1	1	4					0.62	0.48	0.72	0.71	207	24.0	6.27
Q8IZU9	Kin of IRRE-like protein 3 OS=Homo sapiens GN=KIRREL3 PE=1 SV=1 - [KIRR3_HUMAN]	0.77	1	1	1	2					0.93	0.15	0.88	0.14	778	85.2	6.90
Q9ULH0	Kinase D-interacting substrate of 220 kDa OS=Homo sapiens GN=KIDINS220 PE=1 SV=3 - [KDIS_HUMAN]	1.64	1	1	1	1	0.78	0.63	1.10	0.88					1771	196.4	6.62

Q12840	Kinesin heavy chain isoform 5A OS=Homo sapiens GN=KIF5A PE=1 SV=2 - [KIF5A_HUMAN]	3.00	1	3	3	4					1.71	1.68	1.56	1.53	1032	117.3	5.90
O60282	Kinesin heavy chain isoform 5C OS=Homo sapiens GN=KIF5C PE=1 SV=1 - [KIF5C_HUMAN]	0.94	1	1	1	1					1.07	1.03	1.02	0.98	957	109.4	6.19
Q07866	Kinesin light chain 1 OS=Homo sapiens GN=KLC1 PE=1 SV=2 - [KLC1_HUMAN]	5.06	1	1	1	1	0.84	0.93	0.88	0.97					573	65.3	6.20
Q96FN5	Kinesin-like protein KIF12 OS=Homo sapiens GN=KIF12 PE=2 SV=3 - [KIF12_HUMAN]	1.70	1	1	1	5					0.82	0.72	0.85	0.70	646	70.6	8.85
Q9NQT8	Kinesin-like protein KIF13B OS=Homo sapiens GN=KIF13B PE=1 SV=1 - [KI13B_HUMAN]	2.14	1	2	2	2					1.33	1.43	1.36	1.45	1826	202.5	5.78
Q15058	Kinesin-like protein KIF14 OS=Homo sapiens GN=KIF14 PE=1 SV=1 - [KIF14_HUMAN]	0.97	1	1	1	2	1.55	2.00	1.63	2.12	1.38	1.21	1.14	1.00	1648	186.4	7.91
Q9NS87	Kinesin-like protein KIF15 OS=Homo sapiens GN=KIF15 PE=1 SV=1 - [KIF15_HUMAN]	3.39	1	3	3	3	1.08	1.19	0.96	1.07	0.64	0.49	0.72	0.54	1388	160.1	6.00
Q96L93	Kinesin-like protein KIF16B OS=Homo sapiens GN=KIF16B PE=1 SV=2 - [KI16B_HUMAN]	4.48	1	4	4	6	1.08	0.95	1.05	0.87	1.43	1.64	1.22	1.40	1317	151.9	6.16
Q8NI77	Kinesin-like protein KIF18A OS=Homo sapiens GN=KIF18A PE=1 SV=2 - [KI18A_HUMAN]	1.56	1	1	1	4					0.99	0.95	1.08	0.99	898	102.2	8.91
Q2TAC6	Kinesin-like protein KIF19 OS=Homo sapiens GN=KIF19 PE=2 SV=2 - [KIF19_HUMAN]	2.61	1	1	1	1	1.12	1.63	1.29	1.88					998	111.3	8.69
O95235	Kinesin-like protein KIF20A OS=Homo sapiens GN=KIF20A PE=1 SV=1 - [KI20A_HUMAN]	1.80	1	1	1	2	0.74	1.01	0.82	1.13					890	100.2	6.92
Q96Q89	Kinesin-like protein KIF20B OS=Homo sapiens GN=KIF20B PE=1 SV=3 - [KI20B_HUMAN]	1.70	7	1	4	142	0.98	0.83	1.05	0.89					1820	210.5	5.67
Q7Z4S6	Kinesin-like protein KIF21A OS=Homo sapiens GN=KIF21A PE=1 SV=2 - [KI21A_HUMAN]	0.60	1	1	1	2	0.98	1.10	0.99	1.11					1674	187.1	6.42
O75037	Kinesin-like protein KIF21B OS=Homo sapiens GN=KIF21B PE=1 SV=2 - [KI21B_HUMAN]	1.28	2	2	4	6					0.86	1.45	0.78	1.31	1637	182.5	7.08
Q5T7B8	Kinesin-like protein KIF24 OS=Homo sapiens GN=KIF24 PE=1 SV=2 - [KIF24_HUMAN]	1.90	1	2	2	3					0.74	0.32	0.64	0.28	1368	151.8	7.14
Q9UIL4	Kinesin-like protein KIF25 OS=Homo sapiens GN=KIF25 PE=2 SV=2 - [KIF25_HUMAN]	17.71	1	3	3	3	0.71	0.83	0.97	1.14	0.71	0.62	0.67	0.58	384	40.7	8.31
Q9ULI4	Kinesin-like protein KIF26A OS=Homo sapiens GN=KIF26A PE=1 SV=3 - [KI26A_HUMAN]	1.43	1	1	1	1					1.06	0.54	0.61	0.32	1882	194.5	8.81
Q86VH2	Kinesin-like protein KIF27 OS=Homo sapiens GN=KIF27 PE=2 SV=1 - [KIF27_HUMAN]	2.36	1	3	3	9	0.96	0.83	1.10	0.95	1.16	1.32	1.03	1.18	1401	160.2	7.28
O00139	Kinesin-like protein KIF2A OS=Homo sapiens GN=KIF2A PE=1 SV=3 - [KIF2A_HUMAN]	2.69	1	2	2	6					####	9.38	###	8.93	706	79.9	6.68
Q8N4N8	Kinesin-like protein KIF2B OS=Homo sapiens GN=KIF2B PE=1 SV=3 - [KIF2B_HUMAN]	2.97	1	1	1	1					####	###	###	####	673	76.2	8.65
O14782	Kinesin-like protein KIF3C OS=Homo sapiens GN=KIF3C PE=1 SV=3 - [KIF3C_HUMAN]	9.71	1	4	4	4	1.05	1.16	1.12	1.24	1.03	0.84	1.25	1.00	793	89.4	8.22
Q6ZMV9	Kinesin-like protein KIF6 OS=Homo sapiens GN=KIF6 PE=1 SV=3 - [KIF6_HUMAN]	1.47	1	1	1	2	9.37	8.09	0.87	0.75					814	92.5	6.93
Q9BW19	Kinesin-like protein KIFC1 OS=Homo sapiens GN=KIFC1 PE=1 SV=2 - [KIFC1_HUMAN]	5.20	1	1	2	2	1.15	1.01	1.55	1.36					673	73.7	8.98
Q9BZD4	Kinetochore protein Nuf2 OS=Homo sapiens GN=NUF2 PE=1 SV=2 - [NUF2_HUMAN]	7.97	1	4	4	5	0.88	0.83	0.68	0.64	0.70	0.53	0.80	0.60	464	54.3	8.27
Q8NBT2	Kinetochore protein Spc24 OS=Homo sapiens GN=SPC24 PE=1 SV=1 - [SPC24_HUMAN]	13.20	1	1	1	1					0.81	0.88	1.01	1.09	197	22.4	4.70
P01042	Kininogen-1 OS=Homo sapiens GN=KNG1 PE=1 SV=2 - [KNG1_HUMAN]	51.09	1	41	41	1256	1.25	1.22	1.23	1.20	1.00	0.97	1.00	1.00	644	71.9	6.81
Q14678	KN motif and ankyrin repeat domain-containing protein 1 OS=Homo sapiens GN=KANK1 PE=1 SV=3 - [KANK1_HUMAN]	5.55	1	2	2	5					0.84	0.89	0.91	0.96	1352	147.2	5.30

Q63ZY3	KN motif and ankyrin repeat domain-containing protein 2 OS=Homo sapiens GN=KANK2 PE=1 SV=1 - [KANK2_HUMAN]	1.41	1	1	1	1					1.04	1.08	1.00	1.04	851	91.1	5.63
Q96MU8	Kremen protein 1 OS=Homo sapiens GN=KREMEN1 PE=2 SV=3 - [KREM1_HUMAN]	1.69	1	1	1	1	0.73	0.78	0.68	0.73					473	51.7	7.11
Q13601	KRR1 small subunit processome component homolog OS=Homo sapiens GN=KRR1 PE=1 SV=4 - [KRR1_HUMAN]	2.36	1	1	1	2					1.33	1.28	1.49	1.43	381	43.6	9.77
Q9Y2Y9	Krueppel-like factor 13 OS=Homo sapiens GN=KLF13 PE=1 SV=1 - [KLF13_HUMAN]	4.86	1	1	1	4					1.25	0.78	1.19	0.75	288	31.2	9.54
O43278	Kunitz-type protease inhibitor 1 OS=Homo sapiens GN=SPINT1 PE=1 SV=2 - [SPIT1_HUMAN]	1.32	1	1	1	1	1.87	1.76	2.10	2.00					529	58.4	6.29
O43291	Kunitz-type protease inhibitor 2 OS=Homo sapiens GN=SPINT2 PE=1 SV=2 - [SPIT2_HUMAN]	1.98	1	1	1	1	0.95	1.39	0.96	1.42					252	28.2	8.29
Q9NZI2	Kv channel-interacting protein 1 OS=Homo sapiens GN=KCNIP1 PE=1 SV=2 - [KCIP1_HUMAN]	6.17	1	1	1	2	0.95	1.03	4.49	4.95					227	26.8	5.26
Q9NS61	Kv channel-interacting protein 2 OS=Homo sapiens GN=KCNIP2 PE=1 SV=3 - [KCIP2_HUMAN]	3.33	1	1	1	1	1.17	1.55	0.88	1.18					270	30.9	5.11
Q6PIL6	Kv channel-interacting protein 4 OS=Homo sapiens GN=KCNIP4 PE=1 SV=1 - [KCIP4_HUMAN]	3.60	1	1	1	1	0.66	1.36	0.75	1.54					250	28.7	5.21
Q8N0W3	L-fucose kinase OS=Homo sapiens GN=FUK PE=2 SV=2 - [FUK_HUMAN]	0.92	1	1	1	7					0.80	0.84	0.91	0.94	1084	117.5	6.25
P00338	L-lactate dehydrogenase A chain OS=Homo sapiens GN=LDHA PE=1 SV=2 - [LDHA_HUMAN]	17.17	3	5	6	16	1.05	1.11	1.06	1.13	0.59	0.58	0.86	0.75	332	36.7	8.27
P07195	L-lactate dehydrogenase B chain OS=Homo sapiens GN=LDHB PE=1 SV=2 - [LDHB_HUMAN]	33.53	3	10	11	40	0.94	0.95	0.98	0.96	0.99	0.85	1.11	0.95	334	36.6	6.05
P14151	L-selectin OS=Homo sapiens GN=SELL PE=1 SV=2 - [LYAM1_HUMAN]	12.10	1	5	5	31	1.29	1.21	1.15	1.09	0.99	1.06	0.95	0.98	372	42.2	6.60
Q8IV42	L-seryl-tRNA(Sec) kinase OS=Homo sapiens GN=PSTK PE=2 SV=2 - [PSTK_HUMAN]	2.59	1	1	1	2					0.61	0.68	0.61	0.68	348	39.5	8.28
Q6PKG0	La-related protein 1 OS=Homo sapiens GN=LARP1 PE=1 SV=2 - [LARP1_HUMAN]	1.09	1	1	1	7	1.12	0.90	1.23	0.99	1.01	1.05	1.09	1.14	1096	123.4	8.82
Q659C4	La-related protein 1B OS=Homo sapiens GN=LARP1B PE=1 SV=2 - [LAR1B_HUMAN]	2.74	1	1	1	2	0.92	0.55	0.92	0.55					914	105.3	7.61
Q4G0J3	La-related protein 7 OS=Homo sapiens GN=LARP7 PE=1 SV=1 - [LARP7_HUMAN]	3.09	1	1	1	2	1.15	1.35	1.23	1.45					582	66.9	9.55
Q8WV93	Lactation elevated protein 1 OS=Homo sapiens GN=LACE1 PE=2 SV=2 - [LACE1_HUMAN]	2.49	1	1	1	1	1.47	1.79	1.38	1.69					481	54.8	7.27
P22079	Lactoperoxidase OS=Homo sapiens GN=LPO PE=1 SV=2 - [PERL_HUMAN]	1.54	1	1	1	1					0.26	0.62	2.88	6.90	712	80.2	8.62
P02788	Lactotransferrin OS=Homo sapiens GN=LTF PE=1 SV=6 - [TRFL_HUMAN]	58.31	4	39	40	292	1.26	1.24	1.27	1.23	1.12	1.24	1.05	1.09	710	78.1	8.12
O00515	Ladinin-1 OS=Homo sapiens GN=LAD1 PE=1 SV=2 - [LAD1_HUMAN]	1.74	1	1	1	6					0.62	0.63	1.26	1.28	517	57.1	9.67
P24043	Laminin subunit alpha-2 OS=Homo sapiens GN=LAMA2 PE=1 SV=4 - [LAMA2_HUMAN]	5.61	1	8	8	11	0.87	0.85	0.75	0.72	0.50	0.62	0.57	0.71	3122	343.7	6.40
Q16787	Laminin subunit alpha-3 OS=Homo sapiens GN=LAMA3 PE=1 SV=2 - [LAMA3_HUMAN]	1.59	1	2	3	3	0.88	1.03	0.86	1.02	0.80	0.51	1.11	0.71	3333	366.4	7.24
Q16363	Laminin subunit alpha-4 OS=Homo sapiens GN=LAMA4 PE=1 SV=4 - [LAMA4_HUMAN]	0.88	1	1	1	4	1.07	0.95	1.22	1.10	0.67	0.37	0.79	0.44	1823	202.4	6.28
O15230	Laminin subunit alpha-5 OS=Homo sapiens GN=LAMA5 PE=1 SV=8 - [LAMA5_HUMAN]	0.43	1	1	1	6					0.96	0.75	1.01	0.79	3695	399.5	7.02
P07942	Laminin subunit beta-1 OS=Homo sapiens GN=LAMB1 PE=1 SV=2 - [LAMB1_HUMAN]	9.57	1	12	12	29	1.05	1.07	1.10	1.06					1786	197.9	4.94
P55268	Laminin subunit beta-2 OS=Homo sapiens GN=LAMB2 PE=1 SV=2 - [LAMB2_HUMAN]	0.95	1	2	2	2	1.01	0.86	1.01	0.87	0.99	0.90	1.07	0.97	1798	195.9	6.52

Q13751	Laminin subunit beta-3 OS=Homo sapiens GN=LAMB3 PE=1 SV=1 - [LAMB3_HUMAN]	1.02	1	1	1	1					0.28	4.47	0.42	6.59	1172	129.5	7.21
P11047	Laminin subunit gamma-1 OS=Homo sapiens GN=LAMC1 PE=1 SV=3 - [LAMC1_HUMAN]	2.73	1	3	3	4	0.97	0.94	1.02	0.99					1609	177.5	5.12
Q9Y6N6	Laminin subunit gamma-3 OS=Homo sapiens GN=LAMC3 PE=1 SV=3 - [LAMC3_HUMAN]	1.33	1	1	1	1	0.94	1.08	0.92	1.06					1575	171.1	6.58
Q01650	Large neutral amino acids transporter small subunit 1 OS=Homo sapiens GN=SLC7A5 PE=1 SV=2 - [LAT1_HUMAN]	1.78	1	1	1	2	0.88	0.82	1.02	0.95	0.87	0.64	0.82	0.61	507	55.0	7.72
P46379	Large proline-rich protein BAG6 OS=Homo sapiens GN=BAG6 PE=1 SV=2 - [BAG6_HUMAN]	1.59	1	2	2	3	2.20	2.49	2.23	2.55	1.62	1.70	2.56	2.68	1132	119.3	5.60
Q14766	Latent-transforming growth factor beta-binding protein 1 OS=Homo sapiens GN=LTBP1 PE=1 SV=4 - [LTBP1_HUMAN]	14.41	1	18	19	53	1.02	0.95	1.02	0.97	1.03	0.95	1.12	0.99	1721	186.7	5.96
Q9NS15	Latent-transforming growth factor beta-binding protein 3 OS=Homo sapiens GN=LTBP3 PE=1 SV=4 - [LTBP3_HUMAN]	4.60	1	2	3	4					1.37	1.06	1.22	0.95	1303	139.3	6.07
Q9HCC9	Lateral signaling target protein 2 homolog OS=Homo sapiens GN=ZFYVE28 PE=1 SV=3 - [LST2_HUMAN]	5.86	1	2	2	2					1.60		1.67		887	96.4	5.11
Q9BS40	Latexin OS=Homo sapiens GN=LXN PE=1 SV=2 - [LXN_HUMAN]	18.92	1	2	2	3	1.04	1.08	1.28	1.34					222	25.7	5.78
O95490	Latrophilin-2 OS=Homo sapiens GN=LPHN2 PE=1 SV=2 - [LPHN2_HUMAN]	2.74	1	2	2	2					0.98	0.65	1.22	0.81	1459	163.2	6.43
Q9HAR2	Latrophilin-3 OS=Homo sapiens GN=LPHN3 PE=1 SV=2 - [LPHN3_HUMAN]	1.31	1	1	1	5	0.97	0.87	0.98	0.89					1447	161.7	6.44
Q9H400	Lck-interacting transmembrane adapter 1 OS=Homo sapiens GN=LIME1 PE=1 SV=1 - [LIME1_HUMAN]	5.76	1	1	1	1	0.86	0.81	0.97	0.91					295	31.3	9.58
Q14696	LDLR chaperone MESD OS=Homo sapiens GN=MESDC2 PE=1 SV=2 - [MESD_HUMAN]	12.82	1	3	3	10					1.18	1.31	1.39	1.34	234	26.1	7.78
Q86VQ0	Lebercilin OS=Homo sapiens GN=LCA5 PE=1 SV=2 - [LCA5_HUMAN]	4.88	1	2	2	2	1.39	1.35	1.50	1.45	1.73	1.30	1.56	1.17	697	80.5	7.68
O95447	Lebercilin-like protein OS=Homo sapiens GN=LCA5L PE=2 SV=1 - [LCA5L_HUMAN]	3.28	1	2	2	2					1.37	1.43	3.48	4.08	670	76.5	9.48
Q96KR4	Leishmanolysin-like peptidase OS=Homo sapiens GN=LMLN PE=2 SV=2 - [LMLN_HUMAN]	1.53	1	1	1	1					1.00	1.12	0.86	0.97	655	73.5	6.93
P41159	Leptin OS=Homo sapiens GN=LEP PE=1 SV=1 - [LEP_HUMAN]	24.55	1	3	3	8					0.93	0.81	0.99	0.86	167	18.6	6.37
P48357	Leptin receptor OS=Homo sapiens GN=LEPR PE=1 SV=2 - [LEPR_HUMAN]	2.75	1	2	2	3					1.38	0.86	1.98	1.22	1165	132.4	6.52
Q9Y468	Lethal(3)malignant brain tumor-like protein 1 OS=Homo sapiens GN=L3MBTL1 PE=1 SV=3 - [LMBL1_HUMAN]	1.20	1	1	1	1					0.25	0.24	1.24	1.18	752	83.8	5.95
Q96JM7	Lethal(3)malignant brain tumor-like protein 3 OS=Homo sapiens GN=L3MBTL3 PE=1 SV=2 - [LMBL3_HUMAN]	3.33	1	1	2	2	1.56	1.35	1.72	1.49					780	88.3	6.46
Q8NA19	Lethal(3)malignant brain tumor-like protein 4 OS=Homo sapiens GN=L3MBTL4 PE=2 SV=2 - [LMBL4_HUMAN]	3.85	1	1	1	1	1.16	1.25	1.23	1.33					623	71.1	7.43
O95202	LETM1 and EF-hand domain-containing protein 1, mitochondrial OS=Homo sapiens GN=LETM1 PE=1 SV=1 - [LETM1_HUMAN]	7.04	1	3	3	3	1.31	1.46	1.12	1.22					739	83.3	6.70
Q2VYF4	LETM1 domain-containing protein LETM2, mitochondrial OS=Homo sapiens GN=LETM2 PE=2 SV=2 - [LETM2_HUMAN]	2.85	1	1	1	1	1.08	0.99	0.94	0.87					491	55.9	9.23
O60294	Leucine carboxyl methyltransferase 2 OS=Homo sapiens GN=LCMT2 PE=1 SV=3 - [LCMT2_HUMAN]	2.19	1	1	1	2	1.12	1.41	1.12	1.41					686	75.6	6.73
Q86V48	Leucine zipper protein 1 OS=Homo sapiens GN=LUZP1 PE=1 SV=2 - [LUZP1_HUMAN]	3.81	1	2	2	2	1.14	1.06	1.46	1.35					1076	120.2	8.50
Q9P127	Leucine zipper protein 4 OS=Homo sapiens GN=LUZP4 PE=2 SV=1 - [LUZP4_HUMAN]	3.19	1	1	1	1					0.72	0.72	0.64	0.64	313	35.9	9.45
P02750	Leucine-rich alpha-2-glycoprotein OS=Homo sapiens GN=LRG1 PE=1 SV=2 - [A2GL_HUMAN]	57.06	2	16	17	421	1.08	1.05	1.07	1.00	0.81	0.83	0.83	0.84	347	38.2	6.95

Q9P244	Leucine-rich repeat and fibronectin type III domain-containing protein 1 OS=Homo sapiens GN=LRFN1 PE=1 SV=2 - [LRFN1_HUMAN]	3.24	1	1	1	1	0.84	0.85	0.81	0.82					771	82.3	7.05
Q9ULH4	Leucine-rich repeat and fibronectin type-III domain-containing protein 2 OS=Homo sapiens GN=LRFN2 PE=1 SV=2 - [LRFN2_HUMAN]	3.93	1	2	2	2	1.27	0.98	1.15	0.88	0.78	0.65	0.84	0.71	789	84.7	6.74
Q6PJG9	Leucine-rich repeat and fibronectin type-III domain-containing protein 4 OS=Homo sapiens GN=LRFN4 PE=1 SV=1 - [LRFN4_HUMAN]	0.94	1	1	1	1					2.25	1.55	0.93	0.64	635	66.8	6.92
Q96M69	Leucine-rich repeat and guanylate kinase domain-containing protein OS=Homo sapiens GN=LRGUK PE=1 SV=1 - [LRGUK_HUMAN]	2.06	1	2	2	3	0.95	0.88	0.88	0.82	0.67	0.86	0.87	1.12	825	93.6	6.43
Q96JM4	Leucine-rich repeat and IQ domain-containing protein 1 OS=Homo sapiens GN=LRRIQ1 PE=2 SV=3 - [LRIQ1_HUMAN]	0.99	9	1	2	2					1.39	1.38	1.44	1.43	1722	199.2	6.19
Q9HBL6	Leucine-rich repeat and transmembrane domain-containing protein 1 OS=Homo sapiens GN=LRTM1 PE=1 SV=1 - [LRTM1_HUMAN]	3.19	9	1	2	2					0.56	0.47	1.14	0.95	345	38.1	6.74
Q9ULI1	Leucine-rich repeat and WD repeat-containing protein KIAA1239 OS=Homo sapiens GN=KIAA1239 PE=2 SV=3 - [K1239_HUMAN]	0.98	1	1	1	1	4.81	5.30	4.54	5.06					1742	197.3	6.24
Q32MZ4	Leucine-rich repeat flightless-interacting protein 1 OS=Homo sapiens GN=LRRFIP1 PE=1 SV=2 - [LRRF1_HUMAN]	8.79	1	3	5	9					1.00	0.99	1.08	1.02	808	89.2	4.65
Q9Y608	Leucine-rich repeat flightless-interacting protein 2 OS=Homo sapiens GN=LRRFIP2 PE=1 SV=1 - [LRRF2_HUMAN]	3.61	1	1	2	4					1.44	1.49	1.41	1.46	721	82.1	6.95
Q6UXK5	Leucine-rich repeat neuronal protein 1 OS=Homo sapiens GN=LRRN1 PE=1 SV=1 - [LRRN1_HUMAN]	2.93	1	1	1	1					0.70	0.58	0.99	0.82	716	80.7	6.15
Q9H3W5	Leucine-rich repeat neuronal protein 3 OS=Homo sapiens GN=LRRN3 PE=2 SV=1 - [LRRN3_HUMAN]	3.25	1	2	2	4	0.97	0.92	0.97	0.92					708	79.4	7.83
Q8WUT4	Leucine-rich repeat neuronal protein 4 OS=Homo sapiens GN=LRRN4 PE=1 SV=3 - [LRRN4_HUMAN]	4.59	1	1	1	2	0.97	1.56	1.19	1.91					740	78.8	7.20
Q96L50	Leucine-rich repeat protein 1 OS=Homo sapiens GN=LRR1 PE=1 SV=2 - [LLR1_HUMAN]	1.45	1	1	1	1	0.72	1.04	0.63	0.91					414	46.7	9.09
Q38SD2	Leucine-rich repeat serine/threonine-protein kinase 1 OS=Homo sapiens GN=LRRK1 PE=1 SV=3 - [LRRK1_HUMAN]	1.74	1	2	2	3	0.77	1.01	0.72	0.96	2.25	1.94	2.14	1.84	2015	225.2	6.68
Q86VH5	Leucine-rich repeat transmembrane neuronal protein 3 OS=Homo sapiens GN=LRRTM3 PE=2 SV=2 - [LRRT3_HUMAN]	6.88	1	2	2	2	0.49	0.53	0.41	0.44					581	65.9	9.13
Q9NZU1	Leucine-rich repeat transmembrane protein FLRT1 OS=Homo sapiens GN=FLRT1 PE=1 SV=3 - [FLRT1_HUMAN]	1.86	1	1	1	2						1.73		1.83	646	71.3	6.38
Q9HBX8	Leucine-rich repeat-containing G-protein coupled receptor 6 OS=Homo sapiens GN=LGR6 PE=1 SV=3 - [LGR6_HUMAN]	2.90	1	1	1	1					0.52	0.36	0.95	0.66	967	104.2	5.72
Q9BTT6	Leucine-rich repeat-containing protein 1 OS=Homo sapiens GN=LRRC1 PE=1 SV=1 - [LRRC1_HUMAN]	6.11	8	2	3	4					2.01	0.13			524	59.2	5.02
Q5BKY1	Leucine-rich repeat-containing protein 10 OS=Homo sapiens GN=LRRC10 PE=2 SV=1 - [LRC10_HUMAN]	9.03	1	1	1	1	0.65	0.79	0.71	0.87					277	31.6	8.47
Q5VZK9	Leucine-rich repeat-containing protein 16A OS=Homo sapiens GN=LRRC16A PE=1 SV=1 - [LR16A_HUMAN]	1.31	1	1	1	1					1.32	1.06	1.22	0.98	1371	151.5	7.85
Q6F5E8	Leucine-rich repeat-containing protein 16C OS=Homo sapiens GN=RLTPR PE=1 SV=2 - [LR16C_HUMAN]	1.11	1	2	2	4					1.22	0.95	1.29	1.00	1435	154.6	6.76
Q9C0I9	Leucine-rich repeat-containing protein 27 OS=Homo sapiens GN=LRRC27 PE=2 SV=2 - [LRC27_HUMAN]	3.77	1	1	1	1	0.69	0.85	0.69	0.86					530	60.1	9.60
A6NM36	Leucine-rich repeat-containing protein 30 OS=Homo sapiens GN=LRRC30 PE=2 SV=1 - [LRC30_HUMAN]	6.31	1	1	1	48	1.00	0.93	1.09	1.00	0.95	1.14	0.99	1.19	301	34.0	9.33
Q86YC3	Leucine-rich repeat-containing protein 33 OS=Homo sapiens GN=LRRC33 PE=2 SV=1 - [LRC33_HUMAN]	3.32	1	1	1	1	0.98	0.87	1.01	0.90					692	76.3	6.16
O60309	Leucine-rich repeat-containing protein 37A3 OS=Homo sapiens GN=LRRC37A3 PE=2 SV=2 - [L37A3_HUMAN]	2.51	1	2	2	2	0.77	0.90	0.84	0.98		0.69		0.62	1634	180.5	5.31
Q15345	Leucine-rich repeat-containing protein 41 OS=Homo sapiens GN=LRRC41 PE=1 SV=3 - [LRC41_HUMAN]	2.09	1	1	1	1					1.11	1.04	1.11	1.03	812	88.6	8.38
Q9Y546	Leucine-rich repeat-containing protein 42 OS=Homo sapiens GN=LRRC42 PE=1 SV=1 - [LRC42_HUMAN]	3.04	1	1	1	1	0.87	0.86	0.81	0.81					428	48.5	7.46

Q8N309	Leucine-rich repeat-containing protein 43 OS=Homo sapiens GN=LRRC43 PE=2 SV=2 - [LRC43_HUMAN]	2.74	1	1	1	1					0.85	1.02	0.98	1.19	656	73.0	5.24
Q8N1G4	Leucine-rich repeat-containing protein 47 OS=Homo sapiens GN=LRRC47 PE=1 SV=1 - [LRC47_HUMAN]	3.26	1	1	1	1	0.94	0.64	0.84	0.57					583	63.4	8.28
Q9NT99	Leucine-rich repeat-containing protein 4B OS=Homo sapiens GN=LRRC4B PE=1 SV=3 - [LRC4B_HUMAN]	1.68	1	1	1	3	1.02	1.09	0.87	0.94					713	76.4	7.23
Q8N9N7	Leucine-rich repeat-containing protein 57 OS=Homo sapiens GN=LRRC57 PE=1 SV=1 - [LRC57_HUMAN]	14.23	1	1	1	3	1.04	1.26	0.97	1.18					239	26.7	8.43
Q68CR7	Leucine-rich repeat-containing protein 66 OS=Homo sapiens GN=LRRC66 PE=2 SV=1 - [LRC66_HUMAN]	6.59	1	2	2	13					0.86	0.81	0.93	0.88	880	97.7	6.46
Q6ZNQ3	Leucine-rich repeat-containing protein 69 OS=Homo sapiens GN=LRRC69 PE=2 SV=2 - [LRC69_HUMAN]	15.56	1	2	2	29	0.80	0.91	0.90	1.04	1.27	0.96	1.01	0.81	347	39.6	8.57
Q96NW7	Leucine-rich repeat-containing protein 7 OS=Homo sapiens GN=LRRC7 PE=1 SV=1 - [LRRC7_HUMAN]	1.95	1	2	2	2	0.97	1.10	0.99	1.12					1537	172.5	6.81
Q7Z2Q7	Leucine-rich repeat-containing protein 70 OS=Homo sapiens GN=LRRC70 PE=2 SV=1 - [LRR70_HUMAN]	4.50	1	2	2	2	0.88	0.87	0.96	0.96	0.99	1.10	3.20	3.55	622	70.3	8.76
Q8TDW0	Leucine-rich repeat-containing protein 8C OS=Homo sapiens GN=LRRC8C PE=1 SV=2 - [LRC8C_HUMAN]	2.24	1	1	1	1					0.67	0.28	1.20	0.51	803	92.4	7.62
Q7L1W4	Leucine-rich repeat-containing protein 8D OS=Homo sapiens GN=LRRC8D PE=1 SV=1 - [LRC8D_HUMAN]	2.68	2	2	2	2	0.98	1.05	0.80	0.86					858	98.1	7.72
Q6NSJ5	Leucine-rich repeat-containing protein 8E OS=Homo sapiens GN=LRRC8E PE=2 SV=2 - [LRC8E_HUMAN]	1.88	1	1	1	2	0.91	0.72	1.01	0.81	1.37	0.95	1.05	0.73	796	90.2	6.96
Q6ZRR7	Leucine-rich repeat-containing protein 9 OS=Homo sapiens GN=LRRC9 PE=2 SV=2 - [LRRC9_HUMAN]	1.51	1	2	2	2					0.40	0.44	0.89	0.97	1453	166.8	7.65
O94898	Leucine-rich repeats and immunoglobulin-like domains protein 2 OS=Homo sapiens GN=LRIG2 PE=1 SV=3 - [LRIG2_HUMAN]	3.19	1	2	2	3	0.70	0.74	0.67	0.71					1065	118.9	5.55
Q6UXM1	Leucine-rich repeats and immunoglobulin-like domains protein 3 OS=Homo sapiens GN=LRIG3 PE=2 SV=1 - [LRIG3_HUMAN]	3.93	1	3	3	4					0.89	0.91	0.98	0.70	1119	123.4	6.19
Q8N653	Leucine-zipper-like transcriptional regulator 1 OS=Homo sapiens GN=LZTR1 PE=2 SV=2 - [LZTR1_HUMAN]	5.83	1	3	3	3					1.14	1.28	0.96	1.07	840	94.7	6.57
Q9UIQ6	Leucyl-cystinyl aminopeptidase OS=Homo sapiens GN=LNPEP PE=1 SV=3 - [LCAP_HUMAN]	0.88	1	1	1	1	4.27	3.38	4.15	3.30					1025	117.3	5.73
O75829	Leukocyte cell-derived chemotaxin 1 OS=Homo sapiens GN=LECT1 PE=2 SV=1 - [LECT1_HUMAN]	5.99	1	2	2	4					1.64	2.04	0.99	1.43	334	37.1	7.56
O14960	Leukocyte cell-derived chemotaxin-2 OS=Homo sapiens GN=LECT2 PE=1 SV=2 - [LECT2_HUMAN]	5.96	1	1	1	4					1.05	1.21	1.10	1.26	151	16.4	9.39
P30740	Leukocyte elastase inhibitor OS=Homo sapiens GN=SERPINB1 PE=1 SV=1 - [ILEU_HUMAN]	30.61	1	10	10	23	0.96	0.97	0.99	1.00	0.92	0.98	0.92	1.02	379	42.7	6.28
Q8N149	Leukocyte immunoglobulin-like receptor subfamily A member 2 OS=Homo sapiens GN=LILRA2 PE=1 SV=2 - [LIRA2_HUMAN]	2.28	1	1	1	1	1.13	1.02	1.13	1.03					483	53.0	6.92
Q8N6C8	Leukocyte immunoglobulin-like receptor subfamily A member 3 OS=Homo sapiens GN=LILRA3 PE=1 SV=3 - [LIRA3_HUMAN]	8.20	2	3	3	7	0.83	0.76	0.82	0.78	0.42	0.45	0.41	0.44	439	47.4	8.09
A6NI73	Leukocyte immunoglobulin-like receptor subfamily A member 5 OS=Homo sapiens GN=LILRA5 PE=1 SV=1 - [LIRA5_HUMAN]	10.37	1	1	1	3					1.09	1.32	1.13	1.46	299	32.7	6.99
O75023	Leukocyte immunoglobulin-like receptor subfamily B member 5 OS=Homo sapiens GN=LILRB5 PE=1 SV=1 - [LIRB5_HUMAN]	3.90	1	2	2	4	0.87	0.92	0.92	0.99					590	64.1	7.30
Q08722	Leukocyte surface antigen CD47 OS=Homo sapiens GN=CD47 PE=1 SV=1 - [CD47_HUMAN]	2.79	1	1	1	1					0.77	0.15	0.96	0.19	323	35.2	7.21
P29376	Leukocyte tyrosine kinase receptor OS=Homo sapiens GN=LTK PE=1 SV=3 - [LTK_HUMAN]	1.85	1	1	1	1					0.47	1.02	0.56	1.22	864	91.6	6.49
Q6ISS4	Leukocyte-associated immunoglobulin-like receptor 2 OS=Homo sapiens GN=LAIR2 PE=2 SV=1 - [LAIR2_HUMAN]	20.39	1	1	1	1	0.91	0.88	0.98	0.95					152	16.3	5.27
P09960	Leukotriene A-4 hydrolase OS=Homo sapiens GN=LTA4H PE=1 SV=2 - [LKHA4_HUMAN]	8.02	1	4	4	8	1.57	1.56	1.54	1.52					611	69.2	6.18

P48059	LIM and senescent cell antigen-like-containing domain protein 1 OS=Homo sapiens GN=LIMS1 PE=1 SV=4 - [LIMS1_HUMAN]	2.77	2	1	1	4	0.74	0.74	0.70	0.71	0.67	0.74	0.70	0.77	325	37.2	8.05
Q14847	LIM and SH3 domain protein 1 OS=Homo sapiens GN=LASP1 PE=1 SV=2 - [LASP1_HUMAN]	21.46	1	4	4	16					1.22	1.05	1.24	1.09	261	29.7	7.05
Q9UHB6	LIM domain and actin-binding protein 1 OS=Homo sapiens GN=LIMA1 PE=1 SV=1 - [LIMA1_HUMAN]	1.45	1	1	1	2	1.03	1.08	1.04	1.10					759	85.2	6.84
P53667	LIM domain kinase 1 OS=Homo sapiens GN=LIMK1 PE=1 SV=3 - [LIMK1_HUMAN]	2.32	1	1	1	1	1.84	2.70	0.81	1.20					647	72.5	6.98
Q86U70	LIM domain-binding protein 1 OS=Homo sapiens GN=LDB1 PE=1 SV=2 - [LDB1_HUMAN]	5.11	1	1	1	1					0.59	0.57	0.69	0.68	411	46.5	6.96
O75112	LIM domain-binding protein 3 OS=Homo sapiens GN=LDB3 PE=1 SV=2 - [LDB3_HUMAN]	3.30	1	2	2	3	1.31	1.23	1.15	1.22					727	77.1	8.13
Q13449	Limbic system-associated membrane protein OS=Homo sapiens GN=LSAMP PE=1 SV=2 - [LSAMP_HUMAN]	3.25	1	1	1	4	0.95	0.72	1.08	0.82					338	37.4	6.98
Q8WWY8	Lipase member H OS=Homo sapiens GN=LIPH PE=1 SV=1 - [LIPH_HUMAN]	7.10	1	2	2	3	0.91	0.92	0.89	0.92	1.50	1.41	1.54	1.45	451	50.8	7.42
Q32ZL2	Lipid phosphate phosphatase-related protein type 5 OS=Homo sapiens GN=LPPR5 PE=2 SV=2 - [LPPR5_HUMAN]	4.67	1	1	1	2	3.02	3.45	2.99	3.43					321	35.4	7.02
Q93052	Lipoma-preferred partner OS=Homo sapiens GN=LPP PE=1 SV=1 - [LPP_HUMAN]	3.76	1	1	1	2					1.23	1.10	1.38	1.23	612	65.7	7.37
P18428	Lipopolysaccharide-binding protein OS=Homo sapiens GN=LBP PE=1 SV=3 - [LBP_HUMAN]	25.16	1	11	11	94	1.05	1.18	1.06	1.12	1.16	1.14	1.02	1.11	481	53.3	6.70
P50851	Lipopolysaccharide-responsive and beige-like anchor protein OS=Homo sapiens GN=LRBA PE=1 SV=4 - [LRBA_HUMAN]	2.65	2	5	5	12	0.77	0.75	0.81	0.80	0.79	0.65	0.72	0.59	2863	318.9	5.60
P06858	Lipoprotein lipase OS=Homo sapiens GN=LPL PE=1 SV=1 - [LIPL_HUMAN]	3.58	1	1	1	3					0.92	0.98	0.85	0.90	475	53.1	8.15
Q8IVV2	Lipoxygenase homology domain-containing protein 1 OS=Homo sapiens GN=LOXHD1 PE=2 SV=3 - [LOXH1_HUMAN]	2.88	1	3	3	3					0.83	0.69	0.87	0.73	1947	221.8	5.38
Q13136	Liprin-alpha-1 OS=Homo sapiens GN=PPFIA1 PE=1 SV=1 - [LIPA1_HUMAN]	0.58	1	1	1	2					1.05	1.14	1.12	1.21	1202	135.7	6.29
O75334	Liprin-alpha-2 OS=Homo sapiens GN=PPFIA2 PE=1 SV=1 - [LIPA2_HUMAN]	3.42	1	2	2	4					1.12	1.08	0.59	0.57	1257	143.1	5.96
O75145	Liprin-alpha-3 OS=Homo sapiens GN=PPFIA3 PE=1 SV=3 - [LIPA3_HUMAN]	1.26	1	1	1	1	0.91	0.76	1.00	0.84					1194	133.4	5.68
Q86W92	Liprin-beta-1 OS=Homo sapiens GN=PPFIBP1 PE=1 SV=2 - [LIPB1_HUMAN]	1.88	1	2	2	4					0.89	1.17	0.97	1.28	1011	114.0	5.55
Q8ND30	Liprin-beta-2 OS=Homo sapiens GN=PPFIBP2 PE=1 SV=3 - [LIPB2_HUMAN]	6.39	1	2	3	8	1.73	1.14	1.58	1.03					876	98.5	6.27
Q9P260	LisH domain and HEAT repeat-containing protein KIAA1468 OS=Homo sapiens GN=KIAA1468 PE=1 SV=2 - [K1468_HUMAN]	1.07	1	1	1	1					1.10	1.23	0.99	1.11	1216	134.5	5.45
P05451	Lithostathine-1-alpha OS=Homo sapiens GN=REG1A PE=1 SV=3 - [REG1A_HUMAN]	10.84	2	2	2	4					1.59	1.51	1.44	1.37	166	18.7	5.94
P23141	Liver carboxylesterase 1 OS=Homo sapiens GN=CES1 PE=1 SV=2 - [EST1_HUMAN]	7.58	2	3	3	7	1.04	1.00	1.15	1.11	1.16	1.81	0.93	1.46	567	62.5	6.60
Q86WA8	Lon protease homolog 2, peroxisomal OS=Homo sapiens GN=LONP2 PE=1 SV=1 - [LONP2_HUMAN]	2.11	1	1	1	1	0.93	0.82	1.07	0.95					852	94.6	7.30
P36776	Lon protease homolog, mitochondrial OS=Homo sapiens GN=LONP1 PE=1 SV=2 - [LONM_HUMAN]	1.36	1	1	1	1	1.21	1.27	1.24	1.30					959	106.4	6.39
Q5K4L6	Long-chain fatty acid transport protein 3 OS=Homo sapiens GN=SLC27A3 PE=2 SV=3 - [S27A3_HUMAN]	1.92	1	1	1	1					0.68	0.63	1.19	1.10	730	78.6	7.49
Q9ULC5	Long-chain-fatty-acid--CoA ligase 5 OS=Homo sapiens GN=ACSL5 PE=1 SV=1 - [ACSL5_HUMAN]	3.51	1	1	1	1	0.82	0.89	0.87	0.95					683	75.9	6.92
Q96GR2	Long-chain-fatty-acid--CoA ligase ACSBG1 OS=Homo sapiens GN=ACSBG1 PE=1 SV=2 - [ACBG1_HUMAN]	1.52	1	1	1	1	1.05	1.00	0.98	0.94					724	81.2	6.02

P04000	Long-wave-sensitive opsin 1 OS=Homo sapiens GN=OPN1LW PE=1 SV=1 - [OPSR_HUMAN]	3.30	2	1	1	1					1.14	1.20	1.03	1.08	364	40.5	8.60
Q969J3	Loss of heterozygosity 12 chromosomal region 1 protein OS=Homo sapiens GN=LOH12CR1 PE=1 SV=1 - [L12R1_HUMAN]	15.82	1	1	2	2					0.72	0.64	0.93	0.83	196	22.2	6.35
P12318	Low affinity immunoglobulin gamma Fc region receptor II-a OS=Homo sapiens GN=FCGR2A PE=1 SV=4 - [FCG2A_HUMAN]	20.19	3	4	4	11	1.07	1.00	0.80	0.94	0.93	0.99	0.88	0.93	317	35.0	6.68
P08637	Low affinity immunoglobulin gamma Fc region receptor III-A OS=Homo sapiens GN=FCGR3A PE=1 SV=2 - [FCG3A_HUMAN]	17.72	1	1	5	17	1.09	1.07	1.09	1.09					254	29.1	8.07
O75015	Low affinity immunoglobulin gamma Fc region receptor III-B OS=Homo sapiens GN=FCGR3B PE=1 SV=2 - [FCG3B_HUMAN]	15.45	1	1	5	15					1.03	1.05	1.04	1.07	233	26.2	6.71
Q9NY91	Low affinity sodium-glucose cotransporter OS=Homo sapiens GN=SLC5A4 PE=2 SV=1 - [SC5A4_HUMAN]	1.52	1	1	1	1					0.63	0.65	0.67	0.70	659	72.4	6.15
P24666	Low molecular weight phosphotyrosine protein phosphatase OS=Homo sapiens GN=ACP1 PE=1 SV=3 - [PPAC_HUMAN]	13.29	1	2	2	6					1.27	1.46	1.10	1.27	158	18.0	6.74
Q9NZR2	Low-density lipoprotein receptor-related protein 1B OS=Homo sapiens GN=LRP1B PE=1 SV=2 - [LRP1B_HUMAN]	1.26	1	3	3	19	0.90	0.88	0.92	0.90	0.79	0.87	0.83	0.98	4599	515.2	5.30
O75096	Low-density lipoprotein receptor-related protein 4 OS=Homo sapiens GN=LRP4 PE=1 SV=4 - [LRP4_HUMAN]	1.47	1	2	2	3	0.97	0.54	0.99	0.55	0.50	0.64	0.77	0.98	1905	211.9	5.27
O75197	Low-density lipoprotein receptor-related protein 5 OS=Homo sapiens GN=LRP5 PE=1 SV=2 - [LRP5_HUMAN]	2.72	1	3	3	4	1.34	1.49	0.86	0.96	0.75	0.47	0.70	0.44	1615	179.0	5.34
O75581	Low-density lipoprotein receptor-related protein 6 OS=Homo sapiens GN=LRP6 PE=1 SV=2 - [LRP6_HUMAN]	0.62	1	1	1	1					0.88	0.88	1.05	1.05	1613	180.3	5.31
Q14114	Low-density lipoprotein receptor-related protein 8 OS=Homo sapiens GN=LRP8 PE=1 SV=4 - [LRP8_HUMAN]	1.45	1	1	1	1	1.00	0.60	1.09	0.65					963	105.6	5.05
P51884	Lumican OS=Homo sapiens GN=LUM PE=1 SV=2 - [LUM_HUMAN]	43.20	1	13	13	145	0.85	0.83	0.86	0.83	0.65	0.61	0.69	0.63	338	38.4	6.61
Q8IYD9	Lung adenoma susceptibility protein 2 OS=Homo sapiens GN=LAS2 PE=2 SV=1 - [LAS2_HUMAN]	4.57	1	1	1	1	0.51	0.47	0.51	0.48					372	41.8	8.19
P22888	Lutropin-choriogonadotropic hormone receptor OS=Homo sapiens GN=LHCGR PE=1 SV=4 - [LSHR_HUMAN]	1.29	1	1	1	1	1.24	1.63	1.21	1.59					699	78.6	8.50
Q8N2G4	Ly6/PLAUR domain-containing protein 1 OS=Homo sapiens GN=LYPD1 PE=1 SV=2 - [LYPD1_HUMAN]	5.67	1	1	1	1					1.92	2.12	1.23	1.36	141	15.2	8.02
O95274	Ly6/PLAUR domain-containing protein 3 OS=Homo sapiens GN=LYPD3 PE=1 SV=2 - [LYPD3_HUMAN]	13.58	1	3	3	5	1.72	1.57	1.73	1.59					346	35.9	7.75
Q9Y5Y7	Lymphatic vessel endothelial hyaluronic acid receptor 1 OS=Homo sapiens GN=LYVE1 PE=1 SV=2 - [LYVE1_HUMAN]	31.99	1	8	8	49	0.85	0.91	0.85	0.92	0.92	0.86	0.81	0.78	322	35.2	8.28
Q5SQ64	Lymphocyte antigen 6 complex locus protein G6f OS=Homo sapiens GN=LY6G6F PE=1 SV=2 - [LY66F_HUMAN]	14.14	1	4	4	12	0.96	0.97	0.90	0.92	1.22	1.22	1.17	1.23	297	32.4	8.56
O95711	Lymphocyte antigen 86 OS=Homo sapiens GN=LY86 PE=1 SV=1 - [LY86_HUMAN]	9.26	1	1	1	2					1.04	1.15	2.93	3.22	162	17.9	6.07
Q13094	Lymphocyte cytosolic protein 2 OS=Homo sapiens GN=LCP2 PE=1 SV=1 - [LCP2_HUMAN]	2.06	1	2	2	3	1.08	1.35	1.01	1.26	1.11	1.16	1.10	1.15	533	60.2	6.27
Q15046	Lysine--tRNA ligase OS=Homo sapiens GN=KARS PE=1 SV=3 - [SYK_HUMAN]	2.01	1	1	1	1	0.91	1.01	1.07	1.18					597	68.0	6.35
Q8NHM5	Lysine-specific demethylase 2B OS=Homo sapiens GN=KDM2B PE=1 SV=1 - [KDM2B_HUMAN]	0.60	1	1	1	2	0.87	0.85	1.00	0.97					1336	152.5	8.56
Q9Y4C1	Lysine-specific demethylase 3A OS=Homo sapiens GN=KDM3A PE=1 SV=4 - [KDM3A_HUMAN]	2.57	1	2	2	15	0.87	0.70	0.74	0.60	1.40	0.96	1.36	0.93	1321	147.2	8.07
Q7LBC6	Lysine-specific demethylase 3B OS=Homo sapiens GN=KDM3B PE=1 SV=2 - [KDM3B_HUMAN]	1.59	1	2	2	3	1.02	0.78	0.90	0.70	1.61	2.32	0.69	0.99	1761	191.5	7.18
Q6B0I6	Lysine-specific demethylase 4D OS=Homo sapiens GN=KDM4D PE=1 SV=3 - [KDM4D_HUMAN]	1.91	1	1	1	2					1.04	0.95	0.84	0.77	523	58.6	9.16
Q9UGL1	Lysine-specific demethylase 5B OS=Homo sapiens GN=KDM5B PE=1 SV=3 - [KDM5B_HUMAN]	1.62	1	1	1	2	1.27		1.00						1544	175.5	6.70

Q9BY66	Lysine-specific demethylase 5D OS=Homo sapiens GN=KDM5D PE=1 SV=2 - [KDM5D_HUMAN]	2.08	2	3	3	5	1.48	1.60	1.53	1.65					1539	174.0	5.85
O15054	Lysine-specific demethylase 6B OS=Homo sapiens GN=KDM6B PE=1 SV=4 - [KDM6B_HUMAN]	1.22	1	1	1	1	0.28	0.74	0.22	0.58					1643	176.5	8.54
O60341	Lysine-specific histone demethylase 1A OS=Homo sapiens GN=KDM1A PE=1 SV=2 - [KDM1A_HUMAN]	1.29	1	1	1	1					0.88	0.85	0.92	0.90	852	92.8	6.52
Q8NB78	Lysine-specific histone demethylase 1B OS=Homo sapiens GN=KDM1B PE=1 SV=3 - [KDM1B_HUMAN]	1.46	1	1	1	1					6.91	6.70	6.33	6.14	822	92.0	7.87
Q92633	Lysophosphatidic acid receptor 1 OS=Homo sapiens GN=LPAR1 PE=1 SV=3 - [LPAR1_HUMAN]	5.77	1	1	1	1					0.52	0.66	0.60	0.77	364	41.1	8.53
P43657	Lysophosphatidic acid receptor 6 OS=Homo sapiens GN=LPAR6 PE=1 SV=3 - [LPAR6_HUMAN]	2.91	1	2	2	3					0.98	0.95	1.06	1.03	344	39.4	8.98
Q6ZWT7	Lysophospholipid acyltransferase 2 OS=Homo sapiens GN=MBOAT2 PE=2 SV=2 - [MBOA2_HUMAN]	2.12	1	1	1	2					2.24	2.17	2.12	2.05	520	59.5	8.03
P10253	Lysosomal alpha-glucosidase OS=Homo sapiens GN=GAA PE=1 SV=4 - [LYAG_HUMAN]	1.05	1	1	1	2	1.11	1.01	1.21	1.10					952	105.3	6.00
P42785	Lysosomal Pro-X carboxypeptidase OS=Homo sapiens GN=PRCP PE=1 SV=1 - [PCP_HUMAN]	10.48	1	3	3	7	0.99	1.03	1.02	1.09					496	55.8	7.21
P10619	Lysosomal protective protein OS=Homo sapiens GN=CTSA PE=1 SV=2 - [PPGB_HUMAN]	13.96	1	6	6	17	1.32	1.28	1.29	1.31	1.03	1.08	0.93	0.91	480	54.4	6.61
Q13571	Lysosomal-associated transmembrane protein 5 OS=Homo sapiens GN=LAPTM5 PE=1 SV=1 - [LAPM5_HUMAN]	8.02	1	1	1	1	0.61	0.67	0.81	0.89					262	29.9	8.68
Q99698	Lysosomal-trafficking regulator OS=Homo sapiens GN=LYST PE=1 SV=3 - [LYST_HUMAN]	0.37	1	1	1	1	0.84	0.84	0.89	0.89					3801	428.9	6.61
P11279	Lysosome-associated membrane glycoprotein 1 OS=Homo sapiens GN=LAMP1 PE=1 SV=3 - [LAMP1_HUMAN]	3.60	1	2	2	3	1.18	1.22	1.24	1.29					417	44.9	8.75
P13473	Lysosome-associated membrane glycoprotein 2 OS=Homo sapiens GN=LAMP2 PE=1 SV=2 - [LAMP2_HUMAN]	7.07	1	3	3	17	1.07	1.15	1.01	1.09	0.86	0.81	0.91	0.85	410	44.9	5.63
Q9UQV4	Lysosome-associated membrane glycoprotein 3 OS=Homo sapiens GN=LAMP3 PE=1 SV=3 - [LAMP3_HUMAN]	5.05	1	1	1	2					0.31	0.22	1.22	0.88	416	44.3	8.41
P61626	Lysozyme C OS=Homo sapiens GN=LYZ PE=1 SV=1 - [LYSC_HUMAN]	56.08	1	8	8	50	0.79	0.66	0.83	0.70	1.14	1.20	1.12	1.22	148	16.5	9.16
Q96KX0	Lysozyme-like protein 4 OS=Homo sapiens GN=LYZL4 PE=2 SV=1 - [LYZL4_HUMAN]	4.79	1	1	1	1					1.43	1.32	1.40	1.30	146	16.4	8.28
Q08397	Lysyl oxidase homolog 1 OS=Homo sapiens GN=LOXL1 PE=2 SV=2 - [LOXL1_HUMAN]	1.39	1	1	1	1					0.95	0.83	1.21	1.04	574	63.1	7.52
P30307	M-phase inducer phosphatase 3 OS=Homo sapiens GN=CDC25C PE=1 SV=2 - [MPIP3_HUMAN]	5.07	1	2	2	19	1.29	1.28	1.39	1.30					473	53.3	6.79
Q99547	M-phase phosphoprotein 6 OS=Homo sapiens GN=MPHOSPH6 PE=1 SV=2 - [MPH6_HUMAN]	7.50	1	1	1	14	0.94	0.86	0.96	0.86	0.90	0.70	0.88	0.67	160	19.0	5.20
Q99549	M-phase phosphoprotein 8 OS=Homo sapiens GN=MPHOSPH8 PE=1 SV=2 - [MPP8_HUMAN]	1.63	1	1	1	1					1.11	1.00	1.12	1.00	860	97.1	6.06
Q99550	M-phase phosphoprotein 9 OS=Homo sapiens GN=MPHOSPH9 PE=1 SV=3 - [MPP9_HUMAN]	2.91	1	2	2	2	1.54	1.20	1.54	1.42					1031	116.1	6.38
Q8N5G2	Macoilin OS=Homo sapiens GN=TMEM57 PE=1 SV=1 - [MACOI_HUMAN]	3.77	1	2	3	14	0.88	0.68	0.78	0.53	1.27	1.05	1.42	1.11	664	76.1	9.07
P09603	Macrophage colony-stimulating factor 1 OS=Homo sapiens GN=CSF1 PE=1 SV=2 - [CSF1_HUMAN]	2.53	1	1	1	1	1.25	0.52	1.20	0.50					554	60.1	5.29
P07333	Macrophage colony-stimulating factor 1 receptor OS=Homo sapiens GN=CSF1R PE=1 SV=2 - [CSF1R_HUMAN]	5.97	1	6	6	18	1.20	1.22	1.22	1.24					972	107.9	6.37
P22897	Macrophage mannose receptor 1 OS=Homo sapiens GN=MRC1 PE=1 SV=1 - [MRC1_HUMAN]	2.82	1	4	4	7	1.00	1.03	1.03	1.06	1.15	1.10	1.07	1.07	1456	165.9	6.54
P39900	Macrophage metalloelastase OS=Homo sapiens GN=MMP12 PE=1 SV=1 - [MMP12_HUMAN]	1.70	1	1	1	1					1.64	1.60	1.96	1.90	470	54.0	8.66

P14174	Macrophage migration inhibitory factor OS=Homo sapiens GN=MIF PE=1 SV=4 - [MIF_HUMAN]	17.39	1	2	2	5					0.86	0.84	1.02	1.01	115	12.5	7.88
Q9UEW3	Macrophage receptor MARCO OS=Homo sapiens GN=MARCO PE=1 SV=1 - [MARCO_HUMAN]	3.08	1	1	1	2	0.81	0.74	0.90	0.84					520	52.6	8.78
Q8N3R9	MAGUK p55 subfamily member 5 OS=Homo sapiens GN=MPP5 PE=1 SV=3 - [MPP5_HUMAN]	1.04	1	1	1	4	1.78	1.81	1.75	1.84					675	77.2	6.14
Q14728	Major facilitator superfamily domain-containing protein 10 OS=Homo sapiens GN=MFSD10 PE=2 SV=1 - [MFS10_HUMAN]	7.47	1	1	1	1					0.78	0.64	0.81	0.66	455	48.3	9.60
Q8NA29	Major facilitator superfamily domain-containing protein 2A OS=Homo sapiens GN=MFSD2A PE=1 SV=1 - [MFS2A_HUMAN]	5.16	1	1	1	1					1.93	1.29	1.64	1.09	543	60.1	6.95
P40925	Malate dehydrogenase, cytoplasmic OS=Homo sapiens GN=MDH1 PE=1 SV=4 - [MDHC_HUMAN]	10.78	1	2	2	3	0.94	1.04	0.90	0.75					334	36.4	7.36
Q68DK7	Male-specific lethal 1 homolog OS=Homo sapiens GN=MSL1 PE=1 SV=3 - [MSL1_HUMAN]	3.26	1	1	1	1					0.87	###	0.44	7.19	614	67.1	8.92
Q9Y4C4	Malignant fibrous histiocytoma-amplified sequence 1 OS=Homo sapiens GN=MFHAS1 PE=1 SV=2 - [MFHA1_HUMAN]	1.90	1	2	2	2					4.59	3.38	3.71	2.73	1052	116.9	7.88
P48740	Mannan-binding lectin serine protease 1 OS=Homo sapiens GN=MASP1 PE=1 SV=3 - [MASP1_HUMAN]	31.76	1	16	16	97	1.16	1.14	1.17	1.17	1.05	1.04	0.98	0.99	699	79.2	5.49
O00187	Mannan-binding lectin serine protease 2 OS=Homo sapiens GN=MASP2 PE=1 SV=4 - [MASP2_HUMAN]	21.87	1	11	11	75	1.24	1.23	1.27	1.28	1.35	1.34	1.42	1.38	686	75.7	5.63
P11226	Mannose-binding protein C OS=Homo sapiens GN=MBL2 PE=1 SV=2 - [MBL2_HUMAN]	52.82	1	11	11	102	1.10	1.21	1.11	1.26	1.16	1.13	1.13	1.08	248	26.1	5.49
P33908	Mannosyl-oligosaccharide 1,2-alpha-mannosidase IA OS=Homo sapiens GN=MAN1A1 PE=1 SV=3 - [MA1A1_HUMAN]	16.69	1	7	10	41	1.02	1.02	1.08	1.05	1.26	1.30	1.10	1.16	653	72.9	6.47
O60476	Mannosyl-oligosaccharide 1,2-alpha-mannosidase IB OS=Homo sapiens GN=MAN1A2 PE=2 SV=1 - [MA1A2_HUMAN]	2.18	1	1	2	3					1.46	1.34	1.16	1.06	641	73.0	7.61
Q9NR34	Mannosyl-oligosaccharide 1,2-alpha-mannosidase IC OS=Homo sapiens GN=MAN1C1 PE=1 SV=1 - [MA1C1_HUMAN]	2.38	1	2	2	5	1.72	1.75	1.62	1.65	1.22	1.60	1.10	1.45	630	70.9	7.46
Q13724	Mannosyl-oligosaccharide glucosidase OS=Homo sapiens GN=MOGS PE=1 SV=5 - [MOGS_HUMAN]	4.06	1	2	2	3	1.13	1.11	1.19	1.18	2.11	2.20	1.94	2.02	837	91.9	8.90
Q9H8J5	MANSC domain-containing protein 1 OS=Homo sapiens GN=MANSC1 PE=2 SV=1 - [MANS1_HUMAN]	9.28	1	2	2	4	2.01		1.28		0.94	0.88	1.15	1.07	431	46.8	6.54
Q16644	MAP kinase-activated protein kinase 3 OS=Homo sapiens GN=MAPKAPK3 PE=1 SV=1 - [MAPK3_HUMAN]	4.45	1	1	1	1	1.01	0.87	1.04	0.91					382	43.0	7.28
Q9H9H5	MAP6 domain-containing protein 1 OS=Homo sapiens GN=MAP6D1 PE=1 SV=1 - [MA6D1_HUMAN]	9.55	1	1	1	1					1.00	1.10	0.84	0.92	199	21.0	10.07
Q3KQU3	MAP7 domain-containing protein 1 OS=Homo sapiens GN=MAP7D1 PE=1 SV=1 - [MA7D1_HUMAN]	5.47	1	2	2	2	0.73	0.77	0.66	0.70					841	92.8	10.11
Q96T17	MAP7 domain-containing protein 2 OS=Homo sapiens GN=MAP7D2 PE=1 SV=2 - [MA7D2_HUMAN]	5.19	1	2	2	2					1.19	0.86	1.10	0.79	732	81.9	8.84
Q9UQ07	MAPK/MAK/MRK overlapping kinase OS=Homo sapiens GN=MOK PE=2 SV=1 - [MOK_HUMAN]	5.01	1	1	1	1					1.08	1.62	0.96	1.43	419	48.0	9.60
Q86SM8	Mas-related G-protein coupled receptor member E OS=Homo sapiens GN=MRGPRE PE=2 SV=2 - [MRGRE_HUMAN]	2.25	1	1	1	1					0.91	0.62	0.94	0.63	311	34.1	8.09
P35410	Mas-related G-protein coupled receptor MRG OS=Homo sapiens GN=MAS1L PE=2 SV=1 - [MAS1L_HUMAN]	2.12	1	1	1	4					0.67	0.41	0.70	0.33	378	42.4	8.32
P10721	Mast/stem cell growth factor receptor Kit OS=Homo sapiens GN=KIT PE=1 SV=1 - [KIT_HUMAN]	6.56	1	6	6	14	1.11	1.02	1.04	0.98	1.20	0.78	1.08	0.74	976	109.8	6.98
Q13495	Mastermind-like domain-containing protein 1 OS=Homo sapiens GN=MAMLD1 PE=1 SV=2 - [MAMD1_HUMAN]	1.42	1	2	2	2					0.89	0.88	0.90	0.89	774	83.2	8.29
Q8IZL2	Mastermind-like protein 2 OS=Homo sapiens GN=MAML2 PE=1 SV=2 - [MAML2_HUMAN]	1.56	1	1	1	1	0.63	0.62	0.69	0.69					1156	125.1	9.42
Q14680	Maternal embryonic leucine zipper kinase OS=Homo sapiens GN=MELK PE=1 SV=3 - [MELK_HUMAN]	4.30	1	2	2	3					0.86	0.99	0.92	1.06	651	74.6	8.72

P43243	Matrin-3 OS=Homo sapiens GN=MATR3 PE=1 SV=2 - [MATR3_HUMAN]	3.78	1	1	1	2	1.84	1.65	1.72	1.54					847	94.6	6.25
P08493	Matrix Gla protein OS=Homo sapiens GN=MGP PE=1 SV=2 - [MGP_HUMAN]	10.68	1	1	1	9					1.05	1.06	1.26	1.26	103	12.3	9.67
P50281	Matrix metalloproteinase-14 OS=Homo sapiens GN=MMP14 PE=1 SV=3 - [MMP14_HUMAN]	1.55	1	1	1	3					1.29	1.26	1.31	1.27	582	65.9	7.77
P51512	Matrix metalloproteinase-16 OS=Homo sapiens GN=MMP16 PE=1 SV=2 - [MMP16_HUMAN]	2.64	1	2	2	5					0.99	0.74	0.80	1.02	607	69.5	8.54
O60882	Matrix metalloproteinase-20 OS=Homo sapiens GN=MMP20 PE=1 SV=3 - [MMP20_HUMAN]	2.07	1	1	1	1					0.70	0.81	0.64	0.75	483	54.4	8.87
Q9NRE1	Matrix metalloproteinase-26 OS=Homo sapiens GN=MMP26 PE=2 SV=2 - [MMP26_HUMAN]	3.45	1	1	1	1	1.23	1.00	1.06	0.86					261	29.7	6.47
P14780	Matrix metalloproteinase-9 OS=Homo sapiens GN=MMP9 PE=1 SV=3 - [MMP9_HUMAN]	27.44	1	16	16	75	1.29	1.31	1.34	1.37	1.32	1.33	1.31	1.36	707	78.4	6.06
Q9NR99	Matrix-remodeling-associated protein 5 OS=Homo sapiens GN=MXRA5 PE=2 SV=3 - [MXRA5_HUMAN]	2.09	1	2	2	3					1.02	0.80	1.00	0.79	2828	312.0	8.32
Q8IWI9	MAX gene-associated protein OS=Homo sapiens GN=MGA PE=1 SV=2 - [MGAP_HUMAN]	0.50	1	1	1	1	0.71	0.79	0.64	0.71					3026	331.6	6.79
Q9NPJ1	McKusick-Kaufman/Bardet-Biedl syndromes putative chaperonin OS=Homo sapiens GN=MKKS PE=1 SV=1 - [MKKS_HUMAN]	2.46	1	1	1	1	0.81	0.65	0.83	0.67					570	62.3	7.08
Q96DY7	Mdm2-binding protein OS=Homo sapiens GN=MTBP PE=1 SV=1 - [MTBP_HUMAN]	2.77	1	2	2	2	1.29	0.99	0.88	0.69	1.62	1.55	1.52	1.45	904	102.1	7.77
Q03112	MDS1 and EVI1 complex locus protein EVI1 OS=Homo sapiens GN=MECOM PE=1 SV=2 - [EVI1_HUMAN]	3.71	1	2	2	5	0.38	0.21	0.40	0.30	0.83	0.69	0.70	0.59	1051	118.2	6.74
Q5HYA8	Meckelin OS=Homo sapiens GN=TMEM67 PE=1 SV=2 - [MKS3_HUMAN]	1.81	1	1	1	2	0.86	0.68	1.02	0.82	1.19	0.99	1.03	0.86	995	111.7	6.77
Q14676	Mediator of DNA damage checkpoint protein 1 OS=Homo sapiens GN=MDC1 PE=1 SV=3 - [MDC1_HUMAN]	1.39	1	1	1	1	1.21	1.09	1.04	0.95					2089	226.5	5.47
Q15648	Mediator of RNA polymerase II transcription subunit 1 OS=Homo sapiens GN=MED1 PE=1 SV=4 - [MED1_HUMAN]	0.89	1	1	1	1					2.37	2.28	2.14	2.06	1581	168.4	8.73
Q86YW9	Mediator of RNA polymerase II transcription subunit 12-like protein OS=Homo sapiens GN=MED12L PE=1 SV=2 - [MD12L_HUMAN]	2.19	1	3	3	4	0.73	0.72	0.74	0.74	1.12	1.05	1.25	1.18	2145	240.0	7.77
Q9UHV7	Mediator of RNA polymerase II transcription subunit 13 OS=Homo sapiens GN=MED13 PE=1 SV=3 - [MED13_HUMAN]	3.59	1	5	6	6	1.06	1.16	1.21	1.34	0.36	0.64	0.71	1.28	2174	239.1	5.64
Q71F56	Mediator of RNA polymerase II transcription subunit 13-like OS=Homo sapiens GN=MED13L PE=1 SV=1 - [MD13L_HUMAN]	2.13	1	2	2	6	1.06	0.82	1.17	0.92					2210	242.4	6.04
Q96RN5	Mediator of RNA polymerase II transcription subunit 15 OS=Homo sapiens GN=MED15 PE=1 SV=2 - [MED15_HUMAN]	4.31	1	2	2	4					0.82	0.95	0.89	1.03	788	86.7	9.42
Q9Y2X0	Mediator of RNA polymerase II transcription subunit 16 OS=Homo sapiens GN=MED16 PE=1 SV=2 - [MED16_HUMAN]	1.60	1	1	1	1					1.06	1.12	1.11	1.17	877	96.7	7.37
Q96HR3	Mediator of RNA polymerase II transcription subunit 30 OS=Homo sapiens GN=MED30 PE=1 SV=1 - [MED30_HUMAN]	8.43	1	2	2	12	1.29	1.25	1.08	1.24	0.84	0.80	1.05	0.96	178	20.3	8.27
Q9Y4F3	Meiosis arrest female protein 1 OS=Homo sapiens GN=KIAA0430 PE=1 SV=6 - [MARF1_HUMAN]	1.55	1	1	1	1	0.85	0.61	0.97	0.70					1742	192.7	7.87
Q5TIA1	Meiosis inhibitor protein 1 OS=Homo sapiens GN=MEI1 PE=2 SV=2 - [MEI1_HUMAN]	0.47	1	1	1	1	0.61	0.71	0.46	0.55					1274	141.1	6.70
A8MW99	Meiosis-specific protein MEI4-like OS=Homo sapiens GN=MEI4 PE=3 SV=1 - [MEI4L_HUMAN]	2.33	1	1	1	3	0.69	0.66	0.79	0.76	0.85	0.81	0.73	0.70	386	44.2	6.16
Q9BWT6	Meiotic nuclear division protein 1 homolog OS=Homo sapiens GN=MND1 PE=1 SV=1 - [MND1_HUMAN]	6.83	1	1	1	3					1.24	1.06	1.47	1.25	205	23.7	8.19
Q14565	Meiotic recombination protein DMC1/LIM15 homolog OS=Homo sapiens GN=DMC1 PE=1 SV=2 - [DMC1_HUMAN]	7.65	1	2	2	2	0.89	1.01	0.91	1.05					340	37.7	5.88
Q7Z4M0	Meiotic recombination protein REC114-like OS=Homo sapiens GN=C15orf60 PE=2 SV=2 - [R114L_HUMAN]	6.39	1	1	1	3					1.15	1.13	1.26	1.35	266	29.1	5.66

Q9Y5K1	Meiotic recombination protein SPO11 OS=Homo sapiens GN=SPO11 PE=2 SV=1 - [SPO11_HUMAN]	2.53	1	1	1	1	1.16	1.29	1.16	1.30					396	44.5	8.87
Q99705	Melanin-concentrating hormone receptor 1 OS=Homo sapiens GN=MCHR1 PE=1 SV=2 - [MCHR1_HUMAN]	1.42	1	1	1	1					0.62	0.67	0.61	0.66	422	45.9	8.98
P43360	Melanoma-associated antigen 6 OS=Homo sapiens GN=MAGEA6 PE=1 SV=1 - [MAGA6_HUMAN]	4.78	1	1	1	3					2.38	1.23	1.89	0.97	314	34.9	4.59
A8MXT2	Melanoma-associated antigen B17 OS=Homo sapiens GN=MAGEB17 PE=4 SV=2 - [MAGBH_HUMAN]	6.35	1	2	3	4	0.70	0.64	0.69	0.64	1.13	1.23	0.81	1.16	457	51.1	9.00
Q9UBF1	Melanoma-associated antigen C2 OS=Homo sapiens GN=MAGEC2 PE=1 SV=1 - [MAGC2_HUMAN]	4.83	1	1	1	2					0.64	1.24	0.45	0.88	373	41.1	4.31
Q9Y5V3	Melanoma-associated antigen D1 OS=Homo sapiens GN=MAGED1 PE=1 SV=3 - [MAGD1_HUMAN]	6.68	1	2	2	3					####	###	###	####	778	86.1	5.83
Q8TD90	Melanoma-associated antigen E2 OS=Homo sapiens GN=MAGEE2 PE=2 SV=1 - [MAGE2_HUMAN]	2.29	1	1	1	1					1.33	1.35	1.26	1.28	523	60.3	5.10
P15529	Membrane cofactor protein OS=Homo sapiens GN=CD46 PE=1 SV=3 - [MCP_HUMAN]	1.79	1	1	1	1					1.24	0.95	1.17	0.90	392	43.7	6.74
Q495T6	Membrane metallo-endopeptidase-like 1 OS=Homo sapiens GN=MMEL1 PE=1 SV=2 - [MMEL1_HUMAN]	2.05	1	1	1	2					1.23	1.23	1.25	1.25	779	89.3	5.87
Q16853	Membrane primary amine oxidase OS=Homo sapiens GN=AOC3 PE=1 SV=3 - [AOC3_HUMAN]	17.96	2	10	10	26	0.58	0.62	0.67	0.68					763	84.6	6.52
Q5TCQ9	Membrane-associated guanylate kinase, WW and PDZ domain-containing protein 3 OS=Homo sapiens GN=MAGI3 PE=1 SV=2 - [MAGI3_HUMAN]	1.33	1	1	1	1					1.65	1.66	1.29	1.30	1506	165.5	8.02
Q9BZ71	Membrane-associated phosphatidylinositol transfer protein 3 OS=Homo sapiens GN=PITPNM3 PE=1 SV=2 - [PITM3_HUMAN]	2.46	1	1	1	1					1.01				974	106.7	7.14
Q99640	Membrane-associated tyrosine- and threonine-specific cdc2-inhibitory kinase OS=Homo sapiens GN=PKMYT1 PE=1 SV=1 - [PMYT1_HUMAN]	2.40	1	1	1	1					1.13	1.40	1.00	1.24	499	54.5	6.93
Q96JA4	Membrane-spanning 4-domains subfamily A member 14 OS=Homo sapiens GN=MS4A14 PE=2 SV=2 - [M4A14_HUMAN]	4.86	1	2	2	11	0.46	0.32	0.63	0.45	0.63	0.44	0.61	0.43	679	76.5	5.60
Q16820	Meprin A subunit beta OS=Homo sapiens GN=MEP1B PE=1 SV=3 - [MEP1B_HUMAN]	5.42	1	2	2	2					0.98	1.16	0.57	0.68	701	79.5	5.74
P55145	Mesencephalic astrocyte-derived neurotrophic factor OS=Homo sapiens GN=MANF PE=1 SV=3 - [MANF_HUMAN]	24.73	1	5	5	14					1.23	1.34	1.06	1.16	182	20.7	8.69
Q7Z3K6	Mesoderm induction early response protein 3 OS=Homo sapiens GN=MIER3 PE=1 SV=2 - [MIER3_HUMAN]	6.00	1	1	1	1	1.54	0.95	0.93	0.58					550	61.4	4.51
Q9BRJ9	Mesoderm posterior protein 1 OS=Homo sapiens GN=MESP1 PE=2 SV=1 - [MESP1_HUMAN]	13.06	1	1	1	1				0.71					268	28.5	8.90
O15303	Metabotropic glutamate receptor 6 OS=Homo sapiens GN=GRM6 PE=1 SV=2 - [GRM6_HUMAN]	1.03	1	1	1	1					0.17	0.27	0.76	1.17	877	95.4	8.02
Q9NRU3	Metal transporter CNNM1 OS=Homo sapiens GN=CNNM1 PE=2 SV=3 - [CNNM1_HUMAN]	1.37	1	1	1	4					0.92	1.12	0.83	0.96	951	104.3	6.28
Q8NE01	Metal transporter CNNM3 OS=Homo sapiens GN=CNNM3 PE=1 SV=1 - [CNNM3_HUMAN]	3.68	1	1	1	2	1.57	1.49	1.63	1.57					707	76.1	6.09
Q6P4Q7	Metal transporter CNNM4 OS=Homo sapiens GN=CNNM4 PE=1 SV=3 - [CNNM4_HUMAN]	2.19	2	2	2	9	1.06	1.31	1.05	1.00	0.75	0.70	0.82	0.77	775	86.6	6.07
P01033	Metalloproteinase inhibitor 1 OS=Homo sapiens GN=TIMP1 PE=1 SV=1 - [TIMP1_HUMAN]	18.84	1	4	4	19	1.23	1.22	1.17	1.18	1.21	1.37	1.21	1.39	207	23.2	8.10
P16035	Metalloproteinase inhibitor 2 OS=Homo sapiens GN=TIMP2 PE=1 SV=2 - [TIMP2_HUMAN]	16.36	1	5	5	6					0.97	1.10	1.00	1.13	220	24.4	7.49
P35625	Metalloproteinase inhibitor 3 OS=Homo sapiens GN=TIMP3 PE=1 SV=2 - [TIMP3_HUMAN]	10.90	1	3	3	7					1.44	1.44	1.39	1.41	211	24.1	8.72
Q6ZN28	Metastasis-associated in colon cancer protein 1 OS=Homo sapiens GN=MACC1 PE=1 SV=2 - [MACC1_HUMAN]	1.17	1	1	1	1					0.43	0.39	0.88	0.81	852	96.6	6.90
Q15726	Metastasis-suppressor KiSS-1 OS=Homo sapiens GN=KISS1 PE=1 SV=4 - [KISS1_HUMAN]	9.42	1	1	1	3					5.92	5.28	5.83	5.20	138	14.7	10.14

Q5HYI7	Metaxin-3 OS=Homo sapiens GN=MTX3 PE=1 SV=2 - [MTX3_HUMAN]	5.13	1	1	1	3					0.73	0.68	0.76	0.71	312	35.1	7.80
P56192	Methionine--tRNA ligase, cytoplasmic OS=Homo sapiens GN=MARS PE=1 SV=2 - [SYMC_HUMAN]	0.78	1	1	1	1	0.74	0.74	0.66	0.67					900	101.1	6.16
Q96DP5	Methionyl-tRNA formyltransferase, mitochondrial OS=Homo sapiens GN=MTFMT PE=2 SV=2 - [FMT_HUMAN]	4.11	1	1	1	1	1.06	1.40	0.88	1.16					389	43.8	9.66
Q9UBB5	Methyl-CpG-binding domain protein 2 OS=Homo sapiens GN=MBD2 PE=1 SV=1 - [MBD2_HUMAN]	9.98	1	1	1	1	0.96	0.66							411	43.2	10.04
O95243	Methyl-CpG-binding domain protein 4 OS=Homo sapiens GN=MBD4 PE=1 SV=1 - [MBD4_HUMAN]	1.03	1	1	1	2	0.77	0.63	0.84	0.70	0.86	0.90	1.06	1.11	580	66.0	8.82
P51608	Methyl-CpG-binding protein 2 OS=Homo sapiens GN=MECP2 PE=1 SV=1 - [MECP2_HUMAN]	5.14	1	1	2	2	1.06	1.09	1.04	1.06					486	52.4	9.95
Q8NFU7	Methylcytosine dioxygenase TET1 OS=Homo sapiens GN=TET1 PE=1 SV=2 - [TET1_HUMAN]	0.42	1	1	1	1		1.16		1.10					2136	235.2	8.24
Q6N021	Methylcytosine dioxygenase TET2 OS=Homo sapiens GN=TET2 PE=1 SV=3 - [TET2_HUMAN]	1.35	1	2	2	4	1.76	2.58	1.05	1.57					2002	223.7	7.99
Q8N6R0	Methyltransferase-like protein 13 OS=Homo sapiens GN=METTL13 PE=1 SV=1 - [MET13_HUMAN]	1.00	1	1	1	1	4.64	5.68	3.49	4.28					699	78.7	6.73
Q9BUU2	Methyltransferase-like protein 22 OS=Homo sapiens GN=METTL22 PE=2 SV=2 - [MET22_HUMAN]	4.70	1	1	1	1					0.87	1.10	0.68	0.87	404	44.5	5.07
Q5JXM2	Methyltransferase-like protein 24 OS=Homo sapiens GN=METTL24 PE=2 SV=1 - [MET24_HUMAN]	12.84	1	2	2	2	0.85	1.22	0.70	1.01	1.15	1.39	1.09	1.31	366	41.3	9.26
Q8N3J2	Methyltransferase-like protein 4 OS=Homo sapiens GN=METTL4 PE=2 SV=3 - [METL4_HUMAN]	3.81	1	1	1	1					1.09	1.33	1.11	1.35	472	54.0	6.86
P22670	MHC class II regulatory factor RFX1 OS=Homo sapiens GN=RFX1 PE=1 SV=2 - [RFX1_HUMAN]	2.35	1	1	1	1	1.03	1.19	1.59	1.83					979	104.7	6.29
Q6ZW33	MICAL C-terminal-like protein OS=Homo sapiens GN=MICALCL PE=2 SV=3 - [MICLK_HUMAN]	2.73	1	1	1	2	0.70	0.70	0.69	0.69					695	77.2	8.50
Q8NEM0	Microcephalin OS=Homo sapiens GN=MCPH1 PE=1 SV=3 - [MCPH1_HUMAN]	1.32	1	1	1	1					0.86	1.04	0.85	1.03	835	92.8	8.25
Q13361	Microfibrillar-associated protein 5 OS=Homo sapiens GN=MFAP5 PE=1 SV=1 - [MFAP5_HUMAN]	5.20	1	1	1	1					1.31	1.48	1.21	1.36	173	19.6	5.94
O75030	Microphthalmia-associated transcription factor OS=Homo sapiens GN=MITF PE=1 SV=2 - [MITF_HUMAN]	1.71	1	1	1	1	0.62	0.64	0.61	0.64					526	58.8	6.33
Q8WYQ5	Microprocessor complex subunit DGCR8 OS=Homo sapiens GN=DGCR8 PE=1 SV=1 - [DGCR8_HUMAN]	2.46	1	1	1	1					1.05	0.79	1.01	0.77	773	86.0	5.95
Q9UPN3	Microtubule-actin cross-linking factor 1, isoforms 1/2/3/5 OS=Homo sapiens GN=MACF1 PE=1 SV=4 - [MACF1_HUMAN]	2.40	5	13	15	98	1.00	0.98	1.07	1.00	0.91	0.89	0.94	0.96	7388	837.8	5.39
P46821	Microtubule-associated protein 1B OS=Homo sapiens GN=MAP1B PE=1 SV=2 - [MAP1B_HUMAN]	2.27	1	3	3	4	1.09	0.98	0.81	0.73	1.25	1.11	1.24	1.10	2468	270.5	4.81
P11137	Microtubule-associated protein 2 OS=Homo sapiens GN=MAP2 PE=1 SV=4 - [MAP2_HUMAN]	1.70	1	2	2	2	1.15	1.21	1.17	1.23	0.39	0.88	0.49	1.12	1827	199.4	4.91
P27816	Microtubule-associated protein 4 OS=Homo sapiens GN=MAP4 PE=1 SV=3 - [MAP4_HUMAN]	1.30	1	1	1	3	0.87	1.07	0.87	1.07					1152	120.9	5.43
Q96JE9	Microtubule-associated protein 6 OS=Homo sapiens GN=MAP6 PE=1 SV=2 - [MAP6_HUMAN]	1.35	1	1	1	1	0.90	1.45	0.88	1.43					813	86.5	9.16
Q49MG5	Microtubule-associated protein 9 OS=Homo sapiens GN=MAP9 PE=1 SV=3 - [MAP9_HUMAN]	3.55	1	1	1	1	0.68	0.63	0.90	0.83					647	74.2	7.80
Q15691	Microtubule-associated protein RP/EB family member 1 OS=Homo sapiens GN=MAPRE1 PE=1 SV=3 - [MARE1_HUMAN]	23.51	1	5	5	16	0.69	0.61	0.73	0.65	1.00	0.98	1.16	1.15	268	30.0	5.14
Q15555	Microtubule-associated protein RP/EB family member 2 OS=Homo sapiens GN=MAPRE2 PE=1 SV=1 - [MARE2_HUMAN]	11.62	1	3	3	14	0.88	0.74	0.88	0.74	1.82	1.68	1.66	1.44	327	37.0	5.57
Q9Y2H9	Microtubule-associated serine/threonine-protein kinase 1 OS=Homo sapiens GN=MAST1 PE=1 SV=2 - [MAST1_HUMAN]	0.64	1	1	1	1					1.90	2.97	1.18	1.84	1570	170.6	8.44

Q6P0Q8	Microtubule-associated serine/threonine-protein kinase 2 OS=Homo sapiens GN=MAST2 PE=1 SV=2 - [MAST2_HUMAN]	1.39	1	2	2	4	1.07	1.11	1.08	1.13					1798	196.3	8.16
O15021	Microtubule-associated serine/threonine-protein kinase 4 OS=Homo sapiens GN=MAST4 PE=1 SV=3 - [MAST4_HUMAN]	0.76	1	1	1	1					0.72	0.66	0.87	0.80	2626	284.2	8.62
Q9NU22	Midasin OS=Homo sapiens GN=MDN1 PE=1 SV=2 - [MDN1_HUMAN]	0.57	2	3	3	3	1.42	1.63	1.40	1.61	1.67	0.90	1.93	1.04	5596	632.4	5.68
P21741	Midkine OS=Homo sapiens GN=MDK PE=1 SV=1 - [MK_HUMAN]	16.08	1	2	2	6	1.29	1.36	1.06	1.14	1.74	1.58	1.64	1.49	143	15.6	9.79
P20774	Mimecan OS=Homo sapiens GN=OGN PE=1 SV=1 - [MIME_HUMAN]	11.74	1	2	2	8	0.94	0.91	0.93	0.92	0.82	0.83	0.72	0.72	298	33.9	5.63
Q9BTE3	Mini-chromosome maintenance complex-binding protein OS=Homo sapiens GN=MCMBP PE=1 SV=2 - [MCMBP_HUMAN]	1.25	1	1	1	3					3.18	2.95	3.09	2.86	642	72.9	5.87
Q8TD10	Mirror-image polydactyly gene 1 protein OS=Homo sapiens GN=MIPOL1 PE=2 SV=1 - [MIPO1_HUMAN]	2.04	1	1	1	5	1.35	1.25	1.12	1.06					442	51.5	5.72
P54278	Mismatch repair endonuclease PMS2 OS=Homo sapiens GN=PMS2 PE=1 SV=2 - [PMS2_HUMAN]	2.67	1	1	1	1	0.22	0.17	0.57	0.44					862	95.7	6.86
Q8WV92	MIT domain-containing protein 1 OS=Homo sapiens GN=MITD1 PE=1 SV=1 - [MITD1_HUMAN]	5.22	1	1	1	1	0.99	0.94	0.91	0.87					249	29.3	8.00
Q7Z434	Mitochondrial antiviral-signaling protein OS=Homo sapiens GN=MAVS PE=1 SV=2 - [MAVS_HUMAN]	1.67	1	1	1	2					1.28	1.26	1.23	1.21	540	56.5	5.52
P25874	Mitochondrial brown fat uncoupling protein 1 OS=Homo sapiens GN=UCP1 PE=1 SV=3 - [UCP1_HUMAN]	2.28	1	1	1	1	0.38	0.37	0.50	0.49					307	33.0	9.09
Q8N2A8	Mitochondrial cardiolipin hydrolase OS=Homo sapiens GN=PLD6 PE=1 SV=1 - [PLD6_HUMAN]	11.51	1	3	3	8	1.05	1.15	1.06	1.08	0.83	0.85	0.99	0.98	252	28.3	9.35
Q7L5Y1	Mitochondrial enolase superfamily member 1 OS=Homo sapiens GN=ENOSF1 PE=1 SV=1 - [ENOF1_HUMAN]	2.71	1	1	1	1					1.68	1.35	1.44	1.15	443	49.8	6.48
Q9GZY8	Mitochondrial fission factor OS=Homo sapiens GN=MFF PE=1 SV=1 - [MFF_HUMAN]	6.14	1	1	1	1					0.26	0.18	0.38	0.26	342	38.4	8.95
Q9H1K4	Mitochondrial glutamate carrier 2 OS=Homo sapiens GN=SLC25A18 PE=2 SV=1 - [GHC2_HUMAN]	8.89	1	1	1	1								0.53	315	33.8	9.25
Q9Y3D7	Mitochondrial import inner membrane translocase subunit TIM16 OS=Homo sapiens GN=PAM16 PE=1 SV=2 - [TIM16_HUMAN]	6.40	1	1	1	1					0.66	0.73	0.65	0.73	125	13.8	9.70
Q99595	Mitochondrial import inner membrane translocase subunit Tim17-A OS=Homo sapiens GN=TIMM17A PE=1 SV=1 - [TI17A_HUMAN]	16.96	1	1	1	1					1.44	1.48	1.13	1.17	171	18.0	7.87
O60220	Mitochondrial import inner membrane translocase subunit Tim8 A OS=Homo sapiens GN=TIMM8A PE=1 SV=1 - [TIM8A_HUMAN]	17.53	1	1	1	1					0.37	0.72	0.67	1.31	97	11.0	5.16
Q969M1	Mitochondrial import receptor subunit TOM40B OS=Homo sapiens GN=TOMM40L PE=2 SV=1 - [TM40L_HUMAN]	5.19	1	1	1	2	1.06	1.01	1.02	0.99					308	33.9	7.24
O94826	Mitochondrial import receptor subunit TOM70 OS=Homo sapiens GN=TOMM70A PE=1 SV=1 - [TOM70_HUMAN]	4.77	1	1	1	166	1.23	1.23	1.21	1.26	0.99	1.20	0.68	0.83	608	67.4	7.12
Q10713	Mitochondrial-processing peptidase subunit alpha OS=Homo sapiens GN=PMPCA PE=1 SV=2 - [MPPA_HUMAN]	3.24	1	1	1	1	0.94	0.95	1.07	1.10					525	58.2	6.92
Q8IWA4	Mitofusin-1 OS=Homo sapiens GN=MFN1 PE=1 SV=2 - [MFN1_HUMAN]	1.62	1	1	1	1	1.48	0.13	1.60	0.15					741	84.0	6.25
O95140	Mitofusin-2 OS=Homo sapiens GN=MFN2 PE=1 SV=3 - [MFN2_HUMAN]	1.72	1	1	1	1					1.24	1.14	1.11	1.02	757	86.3	6.98
O15264	Mitogen-activated protein kinase 13 OS=Homo sapiens GN=MAPK13 PE=1 SV=1 - [MK13_HUMAN]	3.29	1	1	1	28	0.62	0.62	0.65	0.66	0.40	0.80	0.88	1.77	365	42.1	8.38
P27361	Mitogen-activated protein kinase 3 OS=Homo sapiens GN=MAPK3 PE=1 SV=4 - [MK03_HUMAN]	2.37	1	1	1	2	0.63	0.59	0.67	0.62					379	43.1	6.74
P45983	Mitogen-activated protein kinase 8 OS=Homo sapiens GN=MAPK8 PE=1 SV=2 - [MK08_HUMAN]	3.04	2	1	1	1					1.14	1.91			427	48.3	6.89
P45984	Mitogen-activated protein kinase 9 OS=Homo sapiens GN=MAPK9 PE=1 SV=2 - [MK09_HUMAN]	3.77	1	1	1	1					1.54	1.30	1.39	1.17	424	48.1	5.63

Q99558	Mitogen-activated protein kinase kinase kinase 14 OS=Homo sapiens GN=MAP3K14 PE=1 SV=2 - [M3K14_HUMAN]	3.91	1	2	2	3	1.07	1.15	1.07	1.16					947	104.0	7.72
Q6ZN16	Mitogen-activated protein kinase kinase kinase 15 OS=Homo sapiens GN=MAP3K15 PE=1 SV=2 - [M3K15_HUMAN]	2.82	1	1	1	1					0.83	0.53	1.14	0.73	1313	147.3	5.63
Q99759	Mitogen-activated protein kinase kinase kinase 3 OS=Homo sapiens GN=MAP3K3 PE=1 SV=2 - [M3K3_HUMAN]	2.08	1	1	1	1	1.01	1.27	1.02	1.30					626	70.9	8.84
Q9Y6R4	Mitogen-activated protein kinase kinase kinase 4 OS=Homo sapiens GN=MAP3K4 PE=1 SV=2 - [M3K4_HUMAN]	1.31	1	1	1	2	1.24	2.81	6.13	####					1608	181.6	6.32
Q99683	Mitogen-activated protein kinase kinase kinase 5 OS=Homo sapiens GN=MAP3K5 PE=1 SV=1 - [M3K5_HUMAN]	1.82	1	1	1	1	2.58	1.22	1.79	0.85					1374	154.4	5.78
O95382	Mitogen-activated protein kinase kinase kinase 6 OS=Homo sapiens GN=MAP3K6 PE=1 SV=3 - [M3K6_HUMAN]	1.86	1	1	1	1	0.95	0.90	1.00	0.95					1288	142.5	7.11
O43318	Mitogen-activated protein kinase kinase kinase 7 OS=Homo sapiens GN=MAP3K7 PE=1 SV=1 - [M3K7_HUMAN]	1.98	1	1	1	2					1.35	1.11	1.36	1.12	606	67.2	7.11
P41279	Mitogen-activated protein kinase kinase kinase 8 OS=Homo sapiens GN=MAP3K8 PE=1 SV=2 - [M3K8_HUMAN]	3.43	1	1	1	1					2.56	2.19	2.63	2.25	467	52.9	5.81
Q12851	Mitogen-activated protein kinase kinase kinase kinase 2 OS=Homo sapiens GN=MAP4K2 PE=1 SV=2 - [M4K2_HUMAN]	5.24	1	2	2	2	1.31	1.00	1.40	1.08	0.93	1.13	1.10	1.34	820	91.5	6.34
Q5TCX8	Mitogen-activated protein kinase kinase kinase MLK4 OS=Homo sapiens GN=MLK4 PE=1 SV=1 - [M3KL4_HUMAN]	4.44	2	2	2	2	0.55	0.17	0.75	0.24					1036	113.9	8.73
Q9Y6D9	Mitotic spindle assembly checkpoint protein MAD1 OS=Homo sapiens GN=MAD1L1 PE=1 SV=2 - [MD1L1_HUMAN]	1.67	1	1	1	1	1.18	1.61	1.13	1.55					718	83.0	5.92
Q6NZ67	Mitotic-spindle organizing protein 2B OS=Homo sapiens GN=MZT2B PE=1 SV=1 - [MZT2B_HUMAN]	4.43	1	1	1	2					0.44	0.68	0.81	1.27	158	16.2	10.15
Q9ULH7	MKL/myocardin-like protein 2 OS=Homo sapiens GN=MKL2 PE=1 SV=3 - [MKL2_HUMAN]	1.10	1	1	1	1					1.50	1.66	1.47	1.62	1088	118.1	6.28
Q96T76	MMS19 nucleotide excision repair protein homolog OS=Homo sapiens GN=MMS19 PE=1 SV=2 - [MMS19_HUMAN]	1.17	1	1	1	1	1.25	1.56	1.22	1.54					1030	113.2	6.35
Q7L9L4	MOB kinase activator 1B OS=Homo sapiens GN=MOB1B PE=1 SV=3 - [MOB1B_HUMAN]	5.09	2	1	1	1	1.13	1.25	1.15	1.28					216	25.1	6.73
P26038	Moesin OS=Homo sapiens GN=MSN PE=1 SV=3 - [MOES_HUMAN]	15.25	3	10	10	38	0.78	0.75	0.79	0.79	1.09	1.16	1.06	1.06	577	67.8	6.40
Q9NZB8	Molybdenum cofactor biosynthesis protein 1 OS=Homo sapiens GN=MOCS1 PE=1 SV=3 - [MOCS1_HUMAN]	2.36	1	1	1	1					0.81	2.07	0.82	2.10	636	70.1	9.26
Q9BV23	Monoacylglycerol lipase ABHD6 OS=Homo sapiens GN=ABHD6 PE=2 SV=1 - [ABHD6_HUMAN]	7.72	1	2	2	5	1.22	1.39	1.33	1.53	3.64	4.16	3.00	3.42	337	38.3	8.41
P53985	Monocarboxylate transporter 1 OS=Homo sapiens GN=SLC16A1 PE=1 SV=3 - [MOT1_HUMAN]	4.20	1	1	1	1					0.88	0.54	1.57	0.97	500	53.9	8.66
O60669	Monocarboxylate transporter 2 OS=Homo sapiens GN=SLC16A7 PE=2 SV=2 - [MOT2_HUMAN]	3.14	1	1	1	1	0.94	0.83	0.91	0.81					478	52.2	9.31
O95907	Monocarboxylate transporter 3 OS=Homo sapiens GN=SLC16A8 PE=2 SV=1 - [MOT3_HUMAN]	2.38	1	1	2	10					0.55	0.34	2.21	1.36	504	52.3	5.67
O15375	Monocarboxylate transporter 6 OS=Homo sapiens GN=SLC16A5 PE=2 SV=1 - [MOT6_HUMAN]	1.98	1	1	1	1					0.95	1.01	0.82	0.87	505	55.0	8.22
O15403	Monocarboxylate transporter 7 OS=Homo sapiens GN=SLC16A6 PE=1 SV=2 - [MOT7_HUMAN]	2.29	1	2	2	6					1.07	0.82	0.58	0.43	523	57.4	7.81
P08571	Monocyte differentiation antigen CD14 OS=Homo sapiens GN=CD14 PE=1 SV=2 - [CD14_HUMAN]	43.20	1	13	13	139	1.07	1.12	1.03	1.08	0.93	1.10	0.98	0.97	375	40.1	6.23
Q99685	Monoglyceride lipase OS=Homo sapiens GN=MGLL PE=1 SV=2 - [MGLL_HUMAN]	4.62	1	1	1	2	0.43	0.47	0.44	0.48					303	33.2	6.99
Q9UBU8	Mortality factor 4-like protein 1 OS=Homo sapiens GN=MORF4L1 PE=1 SV=2 - [MO4L1_HUMAN]	2.21	1	1	1	1					0.53	0.61	0.50	0.58	362	41.4	9.28
Q969Z3	MOSC domain-containing protein 2, mitochondrial OS=Homo sapiens GN=MARC2 PE=1 SV=1 - [MOSC2_HUMAN]	4.18	1	1	1	2					1.03	0.32	1.08	0.33	335	38.0	9.16

Q15797	Mothers against decapentaplegic homolog 1 OS=Homo sapiens GN=SMAD1 PE=1 SV=1 - [SMAD1_HUMAN]	3.23	1	1	1	10					0.91	0.92	0.93	0.99	465	52.2	7.31
Q13485	Mothers against decapentaplegic homolog 4 OS=Homo sapiens GN=SMAD4 PE=1 SV=1 - [SMAD4_HUMAN]	3.44	1	1	1	1	1.03	1.22	1.38	1.65					552	60.4	6.99
O43541	Mothers against decapentaplegic homolog 6 OS=Homo sapiens GN=SMAD6 PE=1 SV=2 - [SMAD6_HUMAN]	1.61	1	1	1	1	1.03	1.30	0.96	1.22					496	53.5	8.10
Q8N594	MPN domain-containing protein OS=Homo sapiens GN=MPND PE=1 SV=1 - [MPND_HUMAN]	3.40	1	1	1	1	0.96	0.94	0.89	0.87					471	50.6	5.72
Q9NPI6	mRNA-decapping enzyme 1A OS=Homo sapiens GN=DCP1A PE=1 SV=2 - [DCP1A_HUMAN]	4.47	1	1	1	1	0.67	0.41	0.65	0.40					582	63.3	6.25
Q96T58	Msx2-interacting protein OS=Homo sapiens GN=SPEN PE=1 SV=1 - [MINT_HUMAN]	1.86	16	3	6	14	0.72	0.60	0.70	0.60	0.61	0.42	0.72	0.50	3664	402.0	7.64
Q765P7	MTSS1-like protein OS=Homo sapiens GN=MTSS1L PE=1 SV=1 - [MTSSL_HUMAN]	3.35	9	1	2	2					2.08	2.27	1.83	1.99	747	79.9	7.47
Q9UKN1	Mucin-12 OS=Homo sapiens GN=MUC12 PE=1 SV=2 - [MUC12_HUMAN]	0.75	1	1	1	1	0.81	0.87	0.92	0.99					5478	557.8	5.55
Q8WXI7	Mucin-16 OS=Homo sapiens GN=MUC16 PE=1 SV=2 - [MUC16_HUMAN]	1.00	1	9	9	31	1.17	1.05	1.18	1.08	1.03	1.02	1.12	1.10	22152	#####	6.00
Q685J3	Mucin-17 OS=Homo sapiens GN=MUC17 PE=1 SV=2 - [MUC17_HUMAN]	0.45	1	1	1	2	0.94	1.67	1.08	1.93					4493	451.5	4.11
Q7Z5P9	Mucin-19 OS=Homo sapiens GN=MUC19 PE=1 SV=2 - [MUC19_HUMAN]	0.14	1	1	1	1					1.01	1.06	1.02	1.08	6254	597.8	5.00
Q02817	Mucin-2 OS=Homo sapiens GN=MUC2 PE=1 SV=2 - [MUC2_HUMAN]	0.21	1	1	1	1					0.91	1.26	1.06	1.46	5179	540.0	5.80
Q5SSG8	Mucin-21 OS=Homo sapiens GN=MUC21 PE=1 SV=2 - [MUC21_HUMAN]	3.00	1	1	1	1					0.90	0.40	0.82	0.36	566	54.2	4.81
E2RYF6	Mucin-22 OS=Homo sapiens GN=MUC22 PE=1 SV=1 - [MUC22_HUMAN]	4.29	1	2	2	3	0.66	0.81	0.75	0.97					1773	173.4	3.90
Q9HC84	Mucin-5B OS=Homo sapiens GN=MUC5B PE=1 SV=3 - [MUC5B_HUMAN]	0.33	1	1	1	1					0.92	0.81	1.05	0.92	5762	596.0	6.64
Q6W4X9	Mucin-6 OS=Homo sapiens GN=MUC6 PE=1 SV=3 - [MUC6_HUMAN]	0.62	1	1	1	7					1.08	1.39	1.32	1.73	2439	256.9	7.39
Q13477	Mucosal addressin cell adhesion molecule 1 OS=Homo sapiens GN=MADCAM1 PE=1 SV=2 - [MADCA_HUMAN]	9.42	1	3	3	5	1.42	1.33	1.40	1.31	1.37	1.51	1.36	1.49	382	40.1	5.12
P21439	Multidrug resistance protein 3 OS=Homo sapiens GN=ABCB4 PE=1 SV=2 - [MDR3_HUMAN]	4.35	2	2	3	25					1.19	1.07	1.08	1.00	1286	141.4	8.48
P33527	Multidrug resistance-associated protein 1 OS=Homo sapiens GN=ABCC1 PE=1 SV=3 - [MRP1_HUMAN]	0.98	1	1	1	1	1.23	0.84	0.99	0.69					1531	171.5	7.11
O95255	Multidrug resistance-associated protein 6 OS=Homo sapiens GN=ABCC6 PE=1 SV=2 - [MRP6_HUMAN]	0.60	1	1	1	1					1.26	1.19	1.35	1.28	1503	164.8	8.50
Q13201	Multimerin-1 OS=Homo sapiens GN=MMRN1 PE=1 SV=3 - [MMRN1_HUMAN]	26.14	1	23	24	63	1.25	1.15	1.29	1.17	1.04	1.07	0.94	0.98	1228	138.0	7.93
Q9H8L6	Multimerin-2 OS=Homo sapiens GN=MMRN2 PE=1 SV=2 - [MMRN2_HUMAN]	7.59	1	5	5	26	1.23	1.14	1.44	1.37	1.03	1.05	0.98	1.01	949	104.3	5.86
Q7Z7M0	Multiple epidermal growth factor-like domains protein 8 OS=Homo sapiens GN=MEGF8 PE=1 SV=2 - [MEGF8_HUMAN]	6.82	1	15	15	21	1.08	1.15	0.98	1.05	0.71	0.84	0.91	1.08	2845	302.9	6.87
Q9H1U4	Multiple epidermal growth factor-like domains protein 9 OS=Homo sapiens GN=MEGF9 PE=2 SV=3 - [MEGF9_HUMAN]	1.83	1	1	1	1					0.83	0.83	0.77	0.78	602	62.9	5.67
Q9UNW1	Multiple inositol polyphosphate phosphatase 1 OS=Homo sapiens GN=MINPP1 PE=1 SV=1 - [MINP1_HUMAN]	14.58	1	6	6	34	0.97	0.92	0.98	0.94	1.09	1.03	1.05	0.98	487	55.0	7.81
Q9H7P6	Multivesicular body subunit 12B OS=Homo sapiens GN=FAM125B PE=1 SV=2 - [F125B_HUMAN]	2.19	1	1	1	1	0.98	0.99	0.77	0.78					319	35.6	8.15
P11229	Muscarinic acetylcholine receptor M1 OS=Homo sapiens GN=CHRM1 PE=1 SV=2 - [ACM1_HUMAN]	3.26	1	1	1	2					2.56	2.66	2.39	2.47	460	51.4	9.32

P08912	Muscarinic acetylcholine receptor M5 OS=Homo sapiens GN=CHRM5 PE=2 SV=2 - [ACM5_HUMAN]	2.26	1	1	1	1					1.16	1.17	1.27	1.29	532	60.0	9.28
O15146	Muscle, skeletal receptor tyrosine-protein kinase OS=Homo sapiens GN=MUSK PE=1 SV=1 - [MUSK_HUMAN]	6.67	1	3	3	106	0.91	0.95	0.76	0.83	0.77	0.16	0.97	0.40	869	97.0	7.23
Q9NR56	Muscleblind-like protein 1 OS=Homo sapiens GN=MBNL1 PE=1 SV=2 - [MBNL1_HUMAN]	4.64	3	2	2	5					1.56	1.47	1.36	1.28	388	41.8	8.90
O60682	Musculin OS=Homo sapiens GN=MSC PE=1 SV=2 - [MUSC_HUMAN]	9.22	1	1	1	1	0.89	0.93	0.95	1.00					206	22.1	9.09
Q9UL63	Muskelin OS=Homo sapiens GN=MKLN1 PE=1 SV=2 - [MKLN1_HUMAN]	3.67	1	2	2	2	0.25	0.24	0.23	0.23					735	84.7	6.34
O15457	MutS protein homolog 4 OS=Homo sapiens GN=MSH4 PE=2 SV=2 - [MSH4_HUMAN]	2.24	1	1	1	2					1.38	3.06	1.10	2.44	936	104.7	7.37
O43196	MutS protein homolog 5 OS=Homo sapiens GN=MSH5 PE=1 SV=1 - [MSH5_HUMAN]	3.60	1	2	2	4					0.30	0.20	0.62	0.40	834	92.8	6.37
Q9BQG0	Myb-binding protein 1A OS=Homo sapiens GN=MYBBP1A PE=1 SV=2 - [MBB1A_HUMAN]	2.41	2	3	3	10	1.30	1.26	1.46	1.42	1.88	1.07	2.33	1.32	1328	148.8	9.28
Q6ZTZ1	Myb/SANT-like DNA-binding domain-containing protein 1 OS=Homo sapiens GN=MSANTD1 PE=2 SV=2 - [MSD1_HUMAN]	2.52	1	1	1	2					1.15	0.94	1.09	0.89	278	31.6	8.82
Q6P1R3	Myb/SANT-like DNA-binding domain-containing protein 2 OS=Homo sapiens GN=MSANTD2 PE=1 SV=1 - [MSD2_HUMAN]	2.50	1	1	1	1					0.92	0.91	0.89	0.88	559	61.3	6.19
P01106	Myc proto-oncogene protein OS=Homo sapiens GN=MYC PE=1 SV=1 - [MYC_HUMAN]	6.15	1	1	2	3	1.02	0.89	0.78	0.69					439	48.8	5.49
Q8N699	Myc target protein 1 OS=Homo sapiens GN=MYCT1 PE=2 SV=1 - [MYCT1_HUMAN]	5.11	1	1	1	2	0.57	0.55	0.60	0.57					235	26.6	9.83
Q9Y2G1	Myelin gene regulatory factor OS=Homo sapiens GN=MRF PE=1 SV=3 - [MRF_HUMAN]	3.30	1	2	2	5	1.23	1.14	1.29	1.21					1151	124.3	7.44
P25189	Myelin protein P0 OS=Homo sapiens GN=MPZ PE=1 SV=1 - [MYP0_HUMAN]	2.82	1	1	1	1	0.76	0.85	0.73	0.81					248	27.5	9.54
Q6UWV2	Myelin protein zero-like protein 3 OS=Homo sapiens GN=MPZL3 PE=1 SV=1 - [MPZL3_HUMAN]	5.53	1	1	1	1					0.85	0.82	0.71	0.69	235	26.0	7.94
Q9UL68	Myelin transcription factor 1-like protein OS=Homo sapiens GN=MYT1L PE=2 SV=3 - [MYT1L_HUMAN]	3.71	1	2	2	4	0.85	0.88	0.81	0.85					1186	133.0	4.97
P24158	Myeloblastin OS=Homo sapiens GN=PRTN3 PE=1 SV=3 - [PRTN3_HUMAN]	7.81	1	2	2	13	1.95	2.61	1.96	2.67	0.97	0.82	0.89	0.75	256	27.8	8.35
P41218	Myeloid cell nuclear differentiation antigen OS=Homo sapiens GN=MNDA PE=1 SV=1 - [MNDA_HUMAN]	2.95	9	1	2	4					1.17	1.12	1.46	1.39	407	45.8	9.76
P20138	Myeloid cell surface antigen CD33 OS=Homo sapiens GN=CD33 PE=1 SV=2 - [CD33_HUMAN]	3.57	1	1	1	2	0.79	0.80	0.75	0.77					364	39.8	8.38
Q99836	Myeloid differentiation primary response protein MyD88 OS=Homo sapiens GN=MYD88 PE=1 SV=1 - [MYD88_HUMAN]	1.69	1	1	1	1	1.91	2.35	1.45	1.78					296	33.2	6.15
P05164	Myeloperoxidase OS=Homo sapiens GN=MPO PE=1 SV=1 - [PERM_HUMAN]	25.37	1	18	19	77	1.05	1.04	1.02	1.00	1.42	1.45	1.42	1.47	745	83.8	8.97
P0CAP1	Myocardial zonula adherens protein OS=Homo sapiens GN=MYZAP PE=1 SV=1 - [MYZAP_HUMAN]	3.65	1	1	1	1	1.27	0.74	0.97	0.57					466	54.2	6.18
Q99972	Myocilin OS=Homo sapiens GN=MYOC PE=1 SV=2 - [MYOC_HUMAN]	4.76	1	2	2	4					1.23	0.77	1.12	0.70	504	56.9	5.54
Q06413	Myocyte-specific enhancer factor 2C OS=Homo sapiens GN=MEF2C PE=1 SV=1 - [MEF2C_HUMAN]	1.48	1	1	1	1					1.10	0.41	1.79	0.67	473	51.2	8.03
Q9NZM1	Myoferlin OS=Homo sapiens GN=MYOF PE=1 SV=1 - [MYOF_HUMAN]	1.26	1	2	2	5					0.83	0.79	0.76	0.72	2061	234.6	6.18
Q5VU43	Myomegalin OS=Homo sapiens GN=PDE4DIP PE=2 SV=1 - [MYOME_HUMAN]	2.22	1	3	4	4	1.34	1.16	1.28	1.12					2346	264.9	5.44
P52179	Myomesin-1 OS=Homo sapiens GN=MYOM1 PE=1 SV=2 - [MYOM1_HUMAN]	1.36	1	1	1	2					0.86	0.89	0.77	0.79	1685	187.5	6.93

Q9H1R3	Myosin light chain kinase 2, skeletal/cardiac muscle OS=Homo sapiens GN=MYLK2 PE=1 SV=3 - [MYLK2_HUMAN]	2.01	1	1	1	2					0.69	0.57	1.01	0.82	596	64.6	7.02
Q15746	Myosin light chain kinase, smooth muscle OS=Homo sapiens GN=MYLK PE=1 SV=4 - [MYLK_HUMAN]	1.31	1	2	2	8					1.42	1.47	1.33	1.38	1914	210.6	6.15
P60660	Myosin light polypeptide 6 OS=Homo sapiens GN=MYL6 PE=1 SV=2 - [MYL6_HUMAN]	39.74	4	6	6	31	0.54	0.49	0.51	0.47	0.44	0.40	0.48	0.44	151	16.9	4.65
Q6WCQ1	Myosin phosphatase Rho-interacting protein OS=Homo sapiens GN=MPRIP PE=1 SV=3 - [MPRIP_HUMAN]	0.98	1	1	1	2					1.11	1.07	0.84	0.81	1025	116.5	6.21
P24844	Myosin regulatory light polypeptide 9 OS=Homo sapiens GN=MYL9 PE=1 SV=4 - [MYL9_HUMAN]	44.77	3	5	5	25	0.39	0.47	0.64	0.64	0.44	0.42	0.48	0.53	172	19.8	4.92
P12882	Myosin-1 OS=Homo sapiens GN=MYH1 PE=1 SV=3 - [MYH1_HUMAN]	2.53	2	1	4	13	1.21	1.34	1.25	1.39					1939	223.0	5.74
P35749	Myosin-11 OS=Homo sapiens GN=MYH11 PE=1 SV=3 - [MYH11_HUMAN]	4.97	1	2	8	23	0.67	0.80	0.71	0.86	1.19	1.20	1.44	1.45	1972	227.2	5.50
Q9UKX3	Myosin-13 OS=Homo sapiens GN=MYH13 PE=1 SV=2 - [MYH13_HUMAN]	2.48	1	3	4	5					1.35	1.03	1.89	1.09	1938	223.5	5.68
Q7Z406	Myosin-14 OS=Homo sapiens GN=MYH14 PE=1 SV=2 - [MYH14_HUMAN]	4.86	1	2	9	19	0.55	0.50	0.59	0.54	0.90	0.80	0.86	0.76	1995	227.7	5.60
Q9Y2K3	Myosin-15 OS=Homo sapiens GN=MYH15 PE=1 SV=5 - [MYH15_HUMAN]	1.85	1	2	2	2					0.77	0.88	0.89	1.02	1946	224.5	5.85
Q9Y623	Myosin-4 OS=Homo sapiens GN=MYH4 PE=1 SV=2 - [MYH4_HUMAN]	2.53	2	1	4	13					0.88	0.85	1.01	0.98	1939	222.9	5.85
P12883	Myosin-7 OS=Homo sapiens GN=MYH7 PE=1 SV=5 - [MYH7_HUMAN]	1.34	3	2	3	3	0.41	0.84	1.66	3.40	1.15	0.09	1.11	0.08	1935	223.0	5.80
A7E2Y1	Myosin-7B OS=Homo sapiens GN=MYH7B PE=2 SV=3 - [MYH7B_HUMAN]	1.70	5	2	3	3					1.47	0.99	1.27	0.85	1941	221.3	6.01
P13535	Myosin-8 OS=Homo sapiens GN=MYH8 PE=1 SV=3 - [MYH8_HUMAN]	3.20	2	1	4	13	0.91	1.16	0.82	1.05					1937	222.6	5.74
P35579	Myosin-9 OS=Homo sapiens GN=MYH9 PE=1 SV=4 - [MYH9_HUMAN]	33.98	2	47	58	387	0.51	0.43	0.65	0.50	1.67	1.90	1.27	1.42	1960	226.4	5.60
Q13203	Myosin-binding protein H OS=Homo sapiens GN=MYBPH PE=2 SV=4 - [MYBPH_HUMAN]	2.10	1	1	1	1					0.63	0.49	0.70	0.54	477	52.0	6.73
Q09013	Myotonin-protein kinase OS=Homo sapiens GN=DMPK PE=1 SV=3 - [DMPK_HUMAN]	1.43	1	1	1	1	1.10	0.92	1.32	1.12					629	69.3	5.02
P58546	Myotrophin OS=Homo sapiens GN=MTPN PE=1 SV=2 - [MTPN_HUMAN]	38.14	1	3	3	9					1.27	1.23	1.19	0.99	118	12.9	5.52
Q13613	Myotubularin-related protein 1 OS=Homo sapiens GN=MTMR1 PE=1 SV=4 - [MTMR1_HUMAN]	2.26	1	1	1	4					1.63	0.76	1.58	0.74	665	74.6	7.14
Q9C0I1	Myotubularin-related protein 12 OS=Homo sapiens GN=MTMR12 PE=1 SV=2 - [MTMRC_HUMAN]	6.69	1	2	2	2	0.81	0.85	0.75	0.79	0.18	0.19	0.21	0.22	747	86.1	6.62
Q8NCE2	Myotubularin-related protein 14 OS=Homo sapiens GN=MTMR14 PE=1 SV=2 - [MTMRE_HUMAN]	6.15	1	2	2	3	0.81	0.73	0.73	0.66					650	72.2	6.24
Q13614	Myotubularin-related protein 2 OS=Homo sapiens GN=MTMR2 PE=1 SV=4 - [MTMR2_HUMAN]	6.69	1	3	3	14	0.92	0.85	0.99	0.92	0.61	0.54	1.28	0.99	643	73.3	7.40
Q96QG7	Myotubularin-related protein 9 OS=Homo sapiens GN=MTMR9 PE=1 SV=1 - [MTMR9_HUMAN]	2.91	1	1	1	1					1.22	3.01	0.39	0.97	549	63.4	6.39
Q9NPC6	Myozenin-2 OS=Homo sapiens GN=MYOZ2 PE=1 SV=1 - [MYOZ2_HUMAN]	3.03	1	1	1	1					0.72	0.88	0.91	1.10	264	29.9	8.25
P29966	Myristoylated alanine-rich C-kinase substrate OS=Homo sapiens GN=MARCKS PE=1 SV=4 - [MARCS_HUMAN]	15.66	1	3	3	4					1.04	1.00	1.02	0.98	332	31.5	4.45
Q9UQQ1	N-acetylated-alpha-linked acidic dipeptidase-like protein OS=Homo sapiens GN=NAALADL1 PE=2 SV=2 - [NALDL_HUMAN]	4.59	1	2	2	3		0.69		1.06	0.63	0.57	1.01	0.90	740	80.5	5.36
Q86SF2	N-acetylgalactosaminyltransferase 7 OS=Homo sapiens GN=GALNT7 PE=1 SV=1 - [GALT7_HUMAN]	4.11	1	2	2	3	0.85	0.92	0.79	0.86	1.09	1.21	1.28	1.42	657	75.3	7.11

Q9UK23	N-acetylglucosamine-1-phosphodiester alpha-N-acetylglucosaminidase OS=Homo sapiens GN=NAGPA PE=2 SV=2 - [NAGPA_HUMAN]	4.27	1	2	2	7	1.20	1.09	1.18	1.09	0.85	0.89	0.88	0.91	515	56.0	6.60
Q9UJJ9	N-acetylglucosamine-1-phosphotransferase subunit gamma OS=Homo sapiens GN=GNPTG PE=1 SV=1 - [GNPTG_HUMAN]	21.97	1	3	3	15	1.17	0.96	1.18	1.02	1.00	0.92	0.98	0.93	305	34.0	6.95
Q8N159	N-acetylglutamate synthase, mitochondrial OS=Homo sapiens GN=NAGS PE=1 SV=1 - [NAGS_HUMAN]	2.06	1	1	1	1	1.01	0.85	1.10	0.93					534	58.1	8.90
Q8NFS9	N-acetyllactosaminide beta-1,6-N-acetylglucosaminyl-transferase, isoform C OS=Homo sapiens GN=GCNT2 PE=2 SV=2 - [GNT2C_HUMAN]	4.48	1	1	1	1	1.29	1.41	1.23	1.35					402	46.5	8.16
Q96PD5	N-acetylmuramoyl-L-alanine amidase OS=Homo sapiens GN=PGLYRP2 PE=1 SV=1 - [PGRP2_HUMAN]	47.22	1	18	18	427	1.06	1.05	1.02	1.04	0.83	0.83	0.86	0.83	576	62.2	7.55
O95671	N-acetylserotonin O-methyltransferase-like protein OS=Homo sapiens GN=ASMTL PE=1 SV=3 - [ASML_HUMAN]	1.45	1	1	1	1					1.00	1.00	0.94	0.94	621	68.8	6.07
Q9H0A0	N-acetyltransferase 10 OS=Homo sapiens GN=NAT10 PE=1 SV=2 - [NAT10_HUMAN]	1.76	1	1	1	1					0.98	1.31	0.68	0.90	1025	115.7	8.27
Q5FWF5	N-acetyltransferase ESCO1 OS=Homo sapiens GN=ESCO1 PE=1 SV=3 - [ESCO1_HUMAN]	2.38	1	1	1	2	0.86	0.37	0.71	0.31					840	94.9	9.16
Q14CX7	N-alpha-acetyltransferase 25, NatB auxiliary subunit OS=Homo sapiens GN=NAA25 PE=1 SV=1 - [NAA25_HUMAN]	1.95	11	1	3	3					1.13	1.41	1.11	1.38	972	112.2	6.64
Q86UY6	N-alpha-acetyltransferase 40 OS=Homo sapiens GN=NAA40 PE=2 SV=1 - [NAA40_HUMAN]	8.44	1	1	1	1	0.72	0.69	0.70	0.67					237	27.2	7.39
P15882	N-chimaerin OS=Homo sapiens GN=CHN1 PE=1 SV=3 - [CHIN_HUMAN]	5.88	1	2	2	2					1.16		1.52		459	53.1	6.98
Q7Z6G3	N-terminal EF-hand calcium-binding protein 2 OS=Homo sapiens GN=NECAB2 PE=1 SV=1 - [NECA2_HUMAN]	9.84	1	1	2	2	1.43	1.32	1.38	1.28					386	43.2	5.50
P20933	N(4)-(beta-N-acetylglucosaminyl)-L-asparaginase OS=Homo sapiens GN=AGA PE=1 SV=2 - [ASPG_HUMAN]	7.80	1	3	3	6	1.06	1.07	1.03	1.06	1.02	0.91	1.11	0.98	346	37.2	6.28
O14745	Na(+)/H(+) exchange regulatory cofactor NHE-RF1 OS=Homo sapiens GN=SLC9A3R1 PE=1 SV=4 - [NHRF1_HUMAN]	16.48	1	6	6	19	0.88	0.81	1.00	0.92	1.36	1.21	1.38	1.27	358	38.8	5.77
Q15599	Na(+)/H(+) exchange regulatory cofactor NHE-RF2 OS=Homo sapiens GN=SLC9A3R2 PE=1 SV=2 - [NHRF2_HUMAN]	8.01	1	2	2	2					0.77	0.73	1.04	0.98	337	37.4	7.93
Q149M9	NACHT and WD repeat domain-containing protein 1 OS=Homo sapiens GN=NWD1 PE=2 SV=3 - [NWD1_HUMAN]	1.92	1	2	2	2	0.74	1.01	0.77	1.05					1564	174.4	6.74
Q9C000	NACHT, LRR and PYD domains-containing protein 1 OS=Homo sapiens GN=NLRP1 PE=1 SV=1 - [NALP1_HUMAN]	1.43	1	1	1	1	0.42	0.29	0.45	0.31					1473	165.8	6.84
Q86W24	NACHT, LRR and PYD domains-containing protein 14 OS=Homo sapiens GN=NLRP14 PE=2 SV=1 - [NAL14_HUMAN]	5.03	1	3	3	5	0.73	0.79	0.79	0.87	0.07	0.11	0.61	0.59	1093	124.7	6.61
Q96P20	NACHT, LRR and PYD domains-containing protein 3 OS=Homo sapiens GN=NLRP3 PE=1 SV=3 - [NALP3_HUMAN]	5.41	1	2	2	2					1.53	1.32	1.32	1.14	1036	118.1	6.65
Q96MN2	NACHT, LRR and PYD domains-containing protein 4 OS=Homo sapiens GN=NLRP4 PE=2 SV=3 - [NALP4_HUMAN]	0.91	1	1	1	1	1.96	2.34	2.26	2.70					994	113.3	5.80
Q7RTR0	NACHT, LRR and PYD domains-containing protein 9 OS=Homo sapiens GN=NLRP9 PE=2 SV=1 - [NALP9_HUMAN]	2.83	1	1	2	2					0.58	0.59	0.82	0.84	991	113.2	6.51
P23368	NAD-dependent malic enzyme, mitochondrial OS=Homo sapiens GN=ME2 PE=1 SV=1 - [MAOM_HUMAN]	2.74	1	1	1	1					0.80	0.83	0.70	0.73	584	65.4	7.61
O95299	NADH dehydrogenase [ubiquinone] 1 alpha subcomplex subunit 10, mitochondrial OS=Homo sapiens GN=NDUFA10 PE=1 SV=1 - [NDUAA_HUMAN]	2.82	1	1	1	1					0.89	0.84	0.93	0.88	355	40.7	8.48
Q9P0J0	NADH dehydrogenase [ubiquinone] 1 alpha subcomplex subunit 13 OS=Homo sapiens GN=NDUFA13 PE=1 SV=3 - [NDUAD_HUMAN]	7.64	1	1	1	6	1.51	1.46	1.61	1.54					144	16.7	8.43
O95139	NADH dehydrogenase [ubiquinone] 1 beta subcomplex subunit 6 OS=Homo sapiens GN=NDUFB6 PE=1 SV=3 - [NDUB6_HUMAN]	7.03	1	1	1	5	0.87	0.83	0.93	0.88	1.19	0.88	1.25	1.04	128	15.5	9.63
P49821	NADH dehydrogenase [ubiquinone] flavoprotein 1, mitochondrial OS=Homo sapiens GN=NDUFV1 PE=1 SV=4 - [NDUV1_HUMAN]	4.31	1	1	1	1	0.96	1.28	1.10	1.47					464	50.8	8.21
O75251	NADH dehydrogenase [ubiquinone] iron-sulfur protein 7, mitochondrial OS=Homo sapiens GN=NDUFS7 PE=1 SV=3 - [NDUS7_HUMAN]	6.57	1	1	1	1	0.94	1.12	1.16	1.38					213	23.5	9.99

Q16798	NADP-dependent malic enzyme, mitochondrial OS=Homo sapiens GN=ME3 PE=2 SV=2 - [MAON_HUMAN]	1.32	1	1	1	1					0.22	0.28	1.19	1.53	604	67.0	7.97
P22570	NADPH:adrenodoxin oxidoreductase, mitochondrial OS=Homo sapiens GN=FDXR PE=1 SV=3 - [ADRO_HUMAN]	2.24	1	1	1	1					####	8.61	###	7.56	491	53.8	8.44
Q6T4R5	Nance-Horan syndrome protein OS=Homo sapiens GN=NHS PE=1 SV=1 - [NHS_HUMAN]	2.15	9	2	3	6	1.17	0.93	1.13	0.91	0.67	1.09	1.07	1.74	1630	176.6	6.79
P60323	Nanos homolog 3 OS=Homo sapiens GN=NANOS3 PE=1 SV=1 - [NANO3_HUMAN]	7.51	1	1	1	6	1.17	1.01	0.98	0.85	0.49	0.29	0.63	0.37	173	18.8	8.91
Q9H009	Nascent polypeptide-associated complex subunit alpha-2 OS=Homo sapiens GN=NACA2 PE=1 SV=1 - [NACA2_HUMAN]	9.77	1	2	2	5					0.43	0.86	0.36	0.71	215	23.2	4.73
P01160	Natriuretic peptides A OS=Homo sapiens GN=NPPA PE=1 SV=1 - [ANF_HUMAN]	5.88	1	1	1	2	1.05	1.06	1.04	1.07	1.82	1.78	1.61	1.58	153	16.7	7.15
Q9Y2A7	Nck-associated protein 1 OS=Homo sapiens GN=NCKAP1 PE=1 SV=1 - [NCKP1_HUMAN]	1.33	1	1	1	1	0.87	0.86	0.90	0.90					1128	128.7	6.62
O14513	Nck-associated protein 5 OS=Homo sapiens GN=NCKAP5 PE=2 SV=2 - [NCKP5_HUMAN]	0.63	1	1	1	1	1.13	0.88	0.91	0.71					1909	208.4	8.07
Q9HCH0	Nck-associated protein 5-like OS=Homo sapiens GN=NCKAP5L PE=1 SV=2 - [NCK5L_HUMAN]	6.69	1	3	3	3	0.70	0.87	0.88	1.11					1330	138.9	8.13
Q9NZQ3	NCK-interacting protein with SH3 domain OS=Homo sapiens GN=NCKIPSD PE=1 SV=1 - [SPN90_HUMAN]	2.08	9	1	2	3	0.77	0.77	0.72	0.71					722	78.9	6.38
O76041	Nebulette OS=Homo sapiens GN=NEBL PE=2 SV=1 - [NEBL_HUMAN]	1.38	1	1	1	1	0.67	0.77	0.94	1.09					1014	116.4	7.99
P20929	Nebulin OS=Homo sapiens GN=NEB PE=1 SV=4 - [NEBU_HUMAN]	0.70	1	3	3	4	0.94	1.01	0.95	1.03	1.04	1.03	0.91	0.90	6669	772.4	9.07
Q86VF7	Nebulin-related-anchoring protein OS=Homo sapiens GN=NRAP PE=2 SV=2 - [NRAP_HUMAN]	2.77	1	2	2	2					1.53	1.39	1.30	1.18	1730	197.0	9.20
Q86UW6	NEDD4-binding protein 2 OS=Homo sapiens GN=N4BP2 PE=1 SV=2 - [N4BP2_HUMAN]	1.02	1	1	2	186	1.07	1.16	0.92	1.00					1770	198.7	5.21
O15049	NEDD4-binding protein 3 OS=Homo sapiens GN=N4BP3 PE=1 SV=3 - [N4BP3_HUMAN]	2.57	1	1	1	1					0.80	0.74	0.84	0.77	544	60.4	8.10
Q15843	NEDD8 OS=Homo sapiens GN=NEDD8 PE=1 SV=1 - [NEDD8_HUMAN]	13.58	1	1	1	3					1.75	1.61	1.44	1.32	81	9.1	8.43
P18615	Negative elongation factor E OS=Homo sapiens GN=RDBP PE=1 SV=3 - [NELFE_HUMAN]	6.05	1	1	1	1					1.00	0.93	0.96	0.90	380	43.2	9.33
Q92859	Neogenin OS=Homo sapiens GN=NEO1 PE=1 SV=2 - [NEO1_HUMAN]	3.08	1	3	3	11	1.03	0.94	1.07	0.99	1.00	1.12	0.91	1.02	1461	159.9	6.54
Q7Z494	Nephrocystin-3 OS=Homo sapiens GN=NPHP3 PE=1 SV=1 - [NPHP3_HUMAN]	2.93	1	2	2	3	0.74	0.71	0.80	0.77	1.16	1.01	1.09	0.95	1330	150.8	6.76
Q6UXI9	Nephronectin OS=Homo sapiens GN=NPNT PE=2 SV=3 - [NPNT_HUMAN]	1.24	1	1	1	2					1.20	1.08	1.57	1.40	565	61.9	8.34
Q8NF91	Nesprin-1 OS=Homo sapiens GN=SYNE1 PE=1 SV=3 - [SYNE1_HUMAN]	1.46	5	9	10	31	0.92	0.88	0.88	0.90	1.00	0.98	0.90	0.86	8797	#####	5.53
Q8WXH0	Nesprin-2 OS=Homo sapiens GN=SYNE2 PE=1 SV=3 - [SYNE2_HUMAN]	1.26	1	7	7	8	0.89	0.72	1.01	0.80	2.09	2.22	1.80	1.95	6885	795.9	5.36
Q6ZMZ3	Nesprin-3 OS=Homo sapiens GN=C14orf49 PE=1 SV=2 - [SYNE3_HUMAN]	3.59	1	2	2	17					1.82	1.65	1.67	1.53	975	112.1	6.23
Q8IZJ1	Netrin receptor UNC5B OS=Homo sapiens GN=UNC5B PE=1 SV=2 - [UNC5B_HUMAN]	0.85	1	1	1	1	1.54	1.31	1.41	1.22					945	103.6	6.02
O95185	Netrin receptor UNC5C OS=Homo sapiens GN=UNC5C PE=1 SV=2 - [UNC5C_HUMAN]	2.15	1	1	1	1	0.83	0.91	0.75	0.83					931	103.1	6.10
O00634	Netrin-3 OS=Homo sapiens GN=NTN3 PE=2 SV=1 - [NET3_HUMAN]	1.03	1	1	1	1					1.39	1.39	1.07	1.07	580	61.4	8.79
Q8WTR8	Netrin-5 OS=Homo sapiens GN=NTN5 PE=2 SV=2 - [NET5_HUMAN]	4.70	1	1	1	1	0.57	0.54	0.55	0.53					489	53.1	9.29

Q9UMX5	Neudesin OS=Homo sapiens GN=NENF PE=1 SV=1 - [NENF_HUMAN]	17.44	1	3	3	11					1.07	1.04	0.97	1.02	172	18.8	5.69
Q96SB3	Neurabin-2 OS=Homo sapiens GN=PPP1R9B PE=1 SV=2 - [NEB2_HUMAN]	3.93	1	2	2	4	1.51	0.87	1.78	1.00	1.05	0.68	1.27	0.83	815	89.1	4.97
P13591	Neural cell adhesion molecule 1 OS=Homo sapiens GN=NCAM1 PE=1 SV=3 - [NCAM1_HUMAN]	10.37	1	8	8	21	1.00	0.97	0.96	0.85	0.93	0.88	0.98	0.95	858	94.5	4.87
O15394	Neural cell adhesion molecule 2 OS=Homo sapiens GN=NCAM2 PE=1 SV=2 - [NCAM2_HUMAN]	2.03	1	1	2	3					0.94	1.02	1.18	1.28	837	93.0	5.60
P32004	Neural cell adhesion molecule L1 OS=Homo sapiens GN=L1CAM PE=1 SV=2 - [L1CAM_HUMAN]	1.83	1	2	2	2	1.76	1.50	1.25	1.13					1257	139.9	6.24
O00533	Neural cell adhesion molecule L1-like protein OS=Homo sapiens GN=CHL1 PE=1 SV=4 - [CHL1_HUMAN]	17.63	1	18	18	36	0.94	1.17	0.80	0.87	0.90	0.82	0.96	0.87	1208	135.0	5.76
O76050	Neuralized-like protein 1A OS=Homo sapiens GN=NEURL PE=2 SV=1 - [NEU1A_HUMAN]	9.58	1	2	2	3	1.06	0.80	1.01	0.77					574	61.8	8.51
Q9ULB1	Neurexin-1-alpha OS=Homo sapiens GN=NRXN1 PE=2 SV=1 - [NRX1A_HUMAN]	1.62	1	1	1	2					1.14	0.85	1.43	1.07	1477	161.8	5.90
P58417	Neurexophilin-1 OS=Homo sapiens GN=NXPH1 PE=2 SV=1 - [NXPH1_HUMAN]	9.59	1	2	2	2	0.81	0.95	0.80	0.94	1.07	1.00	1.18	1.10	271	31.1	8.63
Q6ZS30	Neurobeachin-like protein 1 OS=Homo sapiens GN=NBEAL1 PE=2 SV=3 - [NBEL1_HUMAN]	1.37	1	2	2	2					1.25	1.18	1.29	1.22	2694	307.0	6.44
Q6ZNJ1	Neurobeachin-like protein 2 OS=Homo sapiens GN=NBEAL2 PE=1 SV=2 - [NBEL2_HUMAN]	0.47	1	1	1	2	0.91	0.93	0.84	0.86	0.63	0.64	0.73	0.73	2754	302.3	6.38
Q09666	Neuroblast differentiation-associated protein AHNAK OS=Homo sapiens GN=AHNAK PE=1 SV=2 - [AHNK_HUMAN]	0.95	1	4	4	4	0.84	0.73	0.77	0.68	1.22	0.96	1.55	1.23	5890	628.7	6.15
Q3BBV0	Neuroblastoma breakpoint family member 1 OS=Homo sapiens GN=NBPF1 PE=2 SV=1 - [NBPF1_HUMAN]	1.81	1	1	1	1					1.08	1.09	0.94	0.95	1214	139.3	4.78
Q9H094	Neuroblastoma breakpoint family member 3 OS=Homo sapiens GN=NBPF3 PE=2 SV=1 - [NBPF3_HUMAN]	2.37	1	1	1	1	0.60	0.67	0.68	0.76					633	72.9	4.50
P41271	Neuroblastoma suppressor of tumorigenicity 1 OS=Homo sapiens GN=NBL1 PE=1 SV=1 - [NBL1_HUMAN]	7.78	1	2	2	3					1.05	1.09	0.81	0.85	180	19.3	5.29
A2RRP1	Neuroblastoma-amplified sequence OS=Homo sapiens GN=NBAS PE=1 SV=2 - [NBAS_HUMAN]	0.76	8	1	2	2					0.69	0.06	1.07	0.10	2371	268.4	5.96
Q7Z628	Neuroepithelial cell-transforming gene 1 protein OS=Homo sapiens GN=NET1 PE=1 SV=1 - [ARHG8_HUMAN]	5.54	1	1	1	2	1.08	0.94	1.17	1.01					596	67.7	9.23
P21359	Neurofibromin OS=Homo sapiens GN=NF1 PE=1 SV=2 - [NF1_HUMAN]	0.56	1	1	1	2	0.89	0.85	0.79	0.76					2839	319.2	7.39
P07197	Neurofilament medium polypeptide OS=Homo sapiens GN=NEFM PE=1 SV=3 - [NFM_HUMAN]	1.42	1	1	1	1					1.19	0.24	0.83	0.17	916	102.4	4.91
Q13562	Neurogenic differentiation factor 1 OS=Homo sapiens GN=NEUROD1 PE=1 SV=3 - [NDF1_HUMAN]	4.21	1	1	1	1	0.67	0.59	0.68	0.61					356	39.9	5.34
P46531	Neurogenic locus notch homolog protein 1 OS=Homo sapiens GN=NOTCH1 PE=1 SV=4 - [NOTC1_HUMAN]	2.00	1	3	3	3	0.92	0.88	0.85	0.81					2555	272.3	5.12
Q04721	Neurogenic locus notch homolog protein 2 OS=Homo sapiens GN=NOTCH2 PE=1 SV=3 - [NOTC2_HUMAN]	1.98	1	3	4	10	1.15	1.04	0.99	0.90					2471	265.2	5.14
Q9UM47	Neurogenic locus notch homolog protein 3 OS=Homo sapiens GN=NOTCH3 PE=1 SV=2 - [NOTC3_HUMAN]	5.51	1	10	11	21	0.97	0.94	0.95	0.94	1.00	0.97	0.99	0.96	2321	243.5	5.39
Q8NFZ3	Neuroligin-4, Y-linked OS=Homo sapiens GN=NLGN4Y PE=2 SV=1 - [NLGNY_HUMAN]	3.68	1	1	1	1					1.08	1.12	1.23	1.27	816	92.0	6.20
Q9BYT8	Neurolysin, mitochondrial OS=Homo sapiens GN=NLN PE=1 SV=1 - [NEUL_HUMAN]	1.56	1	1	1	2	1.27	1.05	1.19	0.99	0.99	1.07	0.95	1.02	704	80.6	6.64
Q8IVL1	Neuron navigator 2 OS=Homo sapiens GN=NAV2 PE=1 SV=3 - [NAV2_HUMAN]	3.01	1	6	6	6	0.90	0.86	0.98	0.97	1.54	1.62	1.44	1.51	2488	268.0	9.03
Q8IVL0	Neuron navigator 3 OS=Homo sapiens GN=NAV3 PE=1 SV=3 - [NAV3_HUMAN]	2.39	1	3	3	6	0.87	1.01	0.99	1.09	0.77	0.65	0.92	0.77	2385	255.5	8.76

Q92823	Neuronal cell adhesion molecule OS=Homo sapiens GN=NRCAM PE=1 SV=3 - [NRCAM_HUMAN]	3.60	1	3	3	5	0.96	0.66	0.73	0.50	1.14	1.36	1.19	1.42	1304	143.8	5.66
Q7Z3B1	Neuronal growth regulator 1 OS=Homo sapiens GN=NEGR1 PE=1 SV=3 - [NEGR1_HUMAN]	3.67	1	1	1	1	0.99	0.96	1.13	1.10					354	38.7	6.21
P51674	Neuronal membrane glycoprotein M6-a OS=Homo sapiens GN=GPM6A PE=1 SV=2 - [GPM6A_HUMAN]	3.96	1	1	1	1	0.84	0.87	0.75	0.79					278	31.2	5.27
Q9P242	Neuronal tyrosine-phosphorylated phosphoinositide-3-kinase adapter 2 OS=Homo sapiens GN=NYAP2 PE=1 SV=3 - [NYAP2_HUMAN]	1.38	1	1	1	1					1.23	1.27	1.25	1.30	653	70.5	8.48
Q8IY17	Neuropathy target esterase OS=Homo sapiens GN=PNPLA6 PE=1 SV=2 - [PLPL6_HUMAN]	2.34	1	2	2	2	1.19	1.05	1.02	0.90					1366	149.9	7.81
Q8NG41	Neuropeptide B OS=Homo sapiens GN=NPB PE=1 SV=1 - [NPB_HUMAN]	12.00	1	2	2	4					0.81	0.82	0.88	0.85	125	13.1	9.91
P25929	Neuropeptide Y receptor type 1 OS=Homo sapiens GN=NPY1R PE=1 SV=1 - [NPY1R_HUMAN]	5.47	1	1	1	3	0.60	0.48	0.59	0.48					384	44.4	7.80
P49146	Neuropeptide Y receptor type 2 OS=Homo sapiens GN=NPY2R PE=2 SV=1 - [NPY2R_HUMAN]	3.67	1	1	1	7					0.71	0.81	0.69	0.79	381	42.7	7.23
P50391	Neuropeptide Y receptor type 4 OS=Homo sapiens GN=PPYR1 PE=2 SV=1 - [NPY4R_HUMAN]	6.40	1	1	1	11	0.91	0.86	0.99	0.93	1.39	1.25	1.44	1.38	375	42.2	8.13
P48145	Neuropeptides B/W receptor type 1 OS=Homo sapiens GN=NPBWR1 PE=1 SV=2 - [NPBW1_HUMAN]	3.66	1	2	2	3					0.84	0.85	0.71	0.74	328	36.1	8.73
P48146	Neuropeptides B/W receptor type 2 OS=Homo sapiens GN=NPBWR2 PE=1 SV=2 - [NPBW2_HUMAN]	2.40	1	1	1	1					0.83	1.13	0.77	1.04	333	36.8	9.17
O14786	Neuropilin-1 OS=Homo sapiens GN=NRP1 PE=1 SV=3 - [NRP1_HUMAN]	12.78	1	7	7	20	1.15	1.23	1.07	1.22	1.13	1.23	1.17	1.25	923	103.1	5.88
O60462	Neuropilin-2 OS=Homo sapiens GN=NRP2 PE=1 SV=2 - [NRP2_HUMAN]	0.86	1	1	1	1					0.84	0.41	0.88	0.43	931	104.8	5.17
O15240	Neurosecretory protein VGF OS=Homo sapiens GN=VGF PE=1 SV=2 - [VGF_HUMAN]	2.28	1	1	1	1	0.93	0.93	1.01	1.01					615	67.2	4.78
Q9P121	Neurotrimin OS=Homo sapiens GN=NTM PE=1 SV=1 - [NTRI_HUMAN]	2.91	1	1	1	2	0.85	0.74	0.85	0.75					344	37.9	7.81
Q96GC6	Neurotrophin receptor-interacting factor homolog OS=Homo sapiens GN=ZNF274 PE=1 SV=2 - [ZN274_HUMAN]	3.06	1	2	2	3	0.78	0.63	0.70	0.57					653	74.1	6.74
P56730	Neurotrypsin OS=Homo sapiens GN=PRSS12 PE=2 SV=2 - [NETR_HUMAN]	2.74	1	1	1	1					0.78	0.79	0.95	0.96	875	97.0	8.03
Q14697	Neutral alpha-glucosidase AB OS=Homo sapiens GN=GANAB PE=1 SV=3 - [GANAB_HUMAN]	7.73	1	7	7	13	1.08	1.09	1.10	1.13	0.77	0.86	0.76	0.85	944	106.8	6.14
P43007	Neutral amino acid transporter A OS=Homo sapiens GN=SLC1A4 PE=1 SV=1 - [SATT_HUMAN]	5.08	1	1	1	10					0.91	1.12	1.02	1.24	532	55.7	6.25
Q9NR71	Neutral ceramidase OS=Homo sapiens GN=ASAH2 PE=1 SV=2 - [ASAH2_HUMAN]	3.08	1	1	1	3	0.69	0.71	0.64	0.66					780	85.5	7.23
P22894	Neutrophil collagenase OS=Homo sapiens GN=MMP8 PE=1 SV=1 - [MMP8_HUMAN]	8.57	1	3	3	8	1.21	1.28	1.31	1.37	1.10	1.31	1.04	1.24	467	53.4	6.87
Q15080	Neutrophil cytosol factor 4 OS=Homo sapiens GN=NCF4 PE=1 SV=2 - [NCF4_HUMAN]	5.31	1	2	2	2	0.92	0.81	1.14	1.00					339	39.0	6.83
P59665	Neutrophil defensin 1 OS=Homo sapiens GN=DEFA1 PE=1 SV=1 - [DEF1_HUMAN]	35.11	1	1	5	20					0.83	1.03	0.96	1.18	94	10.2	6.99
P59666	Neutrophil defensin 3 OS=Homo sapiens GN=DEFA3 PE=1 SV=1 - [DEF3_HUMAN]	35.11	1	1	5	20					1.48	1.51	1.46	1.48	94	10.2	5.99
P08246	Neutrophil elastase OS=Homo sapiens GN=ELANE PE=1 SV=1 - [ELNE_HUMAN]	29.96	1	5	5	42	1.35	1.33	1.37	1.42	0.50	0.54	0.47	0.50	267	28.5	9.35
P80188	Neutrophil gelatinase-associated lipocalin OS=Homo sapiens GN=LCN2 PE=1 SV=2 - [NGAL_HUMAN]	29.80	1	4	4	32	1.03	1.04	1.15	1.15	1.33	1.41	1.32	1.37	198	22.6	8.91
Q0ZGT2	Nexilin OS=Homo sapiens GN=NEXN PE=1 SV=1 - [NEXN_HUMAN]	8.15	1	6	6	18					2.24	2.17	2.18	2.01	675	80.6	5.33

O15453	Next to BRCA1 gene 2 protein OS=Homo sapiens GN=NBR2 PE=2 SV=1 - [NBR2_HUMAN]	21.43	1	1	1	3	0.99	0.85	1.40	1.22	0.84	0.83	0.91	0.89	112	12.3	7.83
Q15653	NF-kappa-B inhibitor beta OS=Homo sapiens GN=NFKBIB PE=1 SV=2 - [IKBB_HUMAN]	5.34	1	1	1	1					1.36	1.10	1.32	1.06	356	37.7	4.84
O00221	NF-kappa-B inhibitor epsilon OS=Homo sapiens GN=NFKBIE PE=1 SV=3 - [IKBE_HUMAN]	5.00	1	1	1	2					0.49	0.49	0.44	0.43	500	52.8	6.68
Q9BYH8	NF-kappa-B inhibitor zeta OS=Homo sapiens GN=NFKBIZ PE=1 SV=1 - [IKBZ_HUMAN]	2.09	1	1	1	4	0.82	0.86	0.78	0.82					718	78.0	6.62
Q9UMS0	NFU1 iron-sulfur cluster scaffold homolog, mitochondrial OS=Homo sapiens GN=NFU1 PE=1 SV=2 - [NFU1_HUMAN]	1.97	1	1	1	2					0.97	1.17	0.65	0.79	254	28.4	5.07
Q9P2E3	NFX1-type zinc finger-containing protein 1 OS=Homo sapiens GN=ZNFX1 PE=1 SV=2 - [ZNFX1_HUMAN]	0.83	1	1	1	1					1.10	1.11	1.16	1.17	1918	220.1	7.30
Q5SYE7	NHS-like protein 1 OS=Homo sapiens GN=NHSL1 PE=1 SV=2 - [NHSL1_HUMAN]	3.48	1	2	2	2	0.99	0.29	1.22	0.36					1610	170.6	6.96
O60934	Nibrin OS=Homo sapiens GN=NBN PE=1 SV=1 - [NBN_HUMAN]	0.93	1	1	1	2	1.30	1.10	1.34	1.15	1.05	1.38	0.97	1.29	754	84.9	6.90
Q969V3	Nicalin OS=Homo sapiens GN=NCLN PE=1 SV=2 - [NCLN_HUMAN]	5.51	1	1	2	3					1.09	1.05	5.44	5.23	563	62.9	6.89
Q92542	Nicastrin OS=Homo sapiens GN=NCSTN PE=1 SV=2 - [NICA_HUMAN]	7.05	2	3	4	22	0.66	0.70	0.72	0.77	0.62	0.54	0.86	0.75	709	78.4	5.99
P43490	Nicotinamide phosphoribosyltransferase OS=Homo sapiens GN=NAMPT PE=1 SV=1 - [NAMPT_HUMAN]	3.67	1	1	1	1					0.98	0.83	0.95	0.80	491	55.5	7.15
P14543	Nidogen-1 OS=Homo sapiens GN=NID1 PE=1 SV=3 - [NID1_HUMAN]	11.47	1	12	12	23	1.18	1.19	1.17	1.18	0.96	0.94	0.97	0.95	1247	136.3	5.29
Q14112	Nidogen-2 OS=Homo sapiens GN=NID2 PE=1 SV=3 - [NID2_HUMAN]	1.45	1	1	1	1					0.70	0.74	0.78	0.82	1375	151.2	5.29
Q7Z2Y5	Nik-related protein kinase OS=Homo sapiens GN=NRK PE=2 SV=2 - [NRK_HUMAN]	0.82	1	1	1	1	1.54		1.27						1582	178.4	6.33
Q9Y2I6	Ninein-like protein OS=Homo sapiens GN=NINL PE=1 SV=2 - [NINL_HUMAN]	1.74	1	2	2	2	1.18	1.23	1.14	1.19	1.39	1.10	1.32	1.04	1382	156.2	5.06
Q9H841	NIPA-like protein 2 OS=Homo sapiens GN=NIPAL2 PE=2 SV=1 - [NPAL2_HUMAN]	3.26	1	1	1	3					1.27	1.26	1.30	1.29	368	40.8	8.92
Q6KC79	Nipped-B-like protein OS=Homo sapiens GN=NIPBL PE=1 SV=2 - [NIPBL_HUMAN]	0.96	1	2	2	5					0.93	0.84	0.92	0.83	2804	315.9	7.91
Q9Y2I1	Nischarin OS=Homo sapiens GN=NISCH PE=1 SV=3 - [NISCH_HUMAN]	0.60	1	1	1	1	0.71	0.44	0.67	0.42					1504	166.5	5.14
P29475	Nitric oxide synthase, brain OS=Homo sapiens GN=NOS1 PE=1 SV=2 - [NOS1_HUMAN]	2.16	1	2	2	2	1.46	1.06	1.26	0.92					1434	160.9	7.42
P29474	Nitric oxide synthase, endothelial OS=Homo sapiens GN=NOS3 PE=1 SV=3 - [NOS3_HUMAN]	0.50	1	1	1	1		0.83		0.99					1203	133.2	7.27
P26718	NKG2-D type II integral membrane protein OS=Homo sapiens GN=KLRK1 PE=1 SV=1 - [NKG2D_HUMAN]	8.80	1	1	1	6					0.61	0.45	1.03	0.76	216	25.3	8.21
Q5JPE7	Nodal modulator 2 OS=Homo sapiens GN=NOMO2 PE=1 SV=1 - [NOMO2_HUMAN]	5.37	3	2	2	3	1.20	1.21	1.30	1.32					1267	139.4	5.76
Q99784	Noelin OS=Homo sapiens GN=OLFM1 PE=1 SV=4 - [NOE1_HUMAN]	12.99	1	6	6	13	0.92	0.96	0.92	0.96	0.90	0.99	0.82	0.90	485	55.3	6.95
O95897	Noelin-2 OS=Homo sapiens GN=OLFM2 PE=2 SV=2 - [NOE2_HUMAN]	2.42	1	1	1	2	0.99	1.05	0.95	1.00					454	51.4	7.94
Q13253	Noggin OS=Homo sapiens GN=NOG PE=1 SV=1 - [NOGG_HUMAN]	6.47	1	1	1	1					1.09	1.04	1.03	0.99	232	25.8	8.85
P29597	Non-receptor tyrosine-protein kinase TYK2 OS=Homo sapiens GN=TYK2 PE=1 SV=3 - [TYK2_HUMAN]	1.85	1	1	1	2	1.00	0.98	1.05	1.04					1187	133.6	7.15
P10153	Non-secretory ribonuclease OS=Homo sapiens GN=RNASE2 PE=1 SV=2 - [RNAS2_HUMAN]	8.70	1	1	1	3					0.78	0.83	0.97	1.04	161	18.3	8.73

P22307	Non-specific lipid-transfer protein OS=Homo sapiens GN=SCP2 PE=1 SV=2 - [NLTP_HUMAN]	3.47	1	1	1	1	1.78	1.64	1.54	1.43					547	59.0	6.89
O60443	Non-syndromic hearing impairment protein 5 OS=Homo sapiens GN=DFNA5 PE=1 SV=2 - [DFNA5_HUMAN]	3.83	1	1	1	1	0.73	0.73	0.76	0.76					496	54.5	5.17
Q8IVI9	Nostrin OS=Homo sapiens GN=NOSTRIN PE=1 SV=2 - [NOSTN_HUMAN]	5.73	1	2	2	3	0.35	0.69	1.71	3.40	0.61	0.63	0.69	0.71	506	57.6	8.97
Q9UNZ2	NSFL1 cofactor p47 OS=Homo sapiens GN=NSFL1C PE=1 SV=2 - [NSF1C_HUMAN]	3.24	1	1	1	3					1.63	1.72	1.84	1.94	370	40.5	5.10
Q16288	NT-3 growth factor receptor OS=Homo sapiens GN=NTRK3 PE=1 SV=2 - [NTRK3_HUMAN]	2.62	1	1	1	1					1.49	1.37	1.45	1.33	839	94.4	6.58
Q9H093	NUAK family SNF1-like kinase 2 OS=Homo sapiens GN=NUAK2 PE=1 SV=1 - [NUAK2_HUMAN]	1.59	1	1	1	1					1.18	1.46	0.91	1.13	628	69.6	8.87
P08651	Nuclear factor 1 C-type OS=Homo sapiens GN=NFIC PE=1 SV=2 - [NFIC_HUMAN]	2.56	1	1	1	4	0.78	0.81	0.71	0.75					508	55.6	8.38
Q14494	Nuclear factor erythroid 2-related factor 1 OS=Homo sapiens GN=NFE2L1 PE=1 SV=1 - [NF2L1_HUMAN]	1.30	1	1	1	1					0.15	0.50	0.22	0.75	772	84.7	4.64
Q00653	Nuclear factor NF-kappa-B p100 subunit OS=Homo sapiens GN=NFKB2 PE=1 SV=4 - [NFKB2_HUMAN]	1.78	1	1	1	1	1.18	1.81	1.10	1.70					900	96.7	6.25
Q6P4R8	Nuclear factor related to kappa-B-binding protein OS=Homo sapiens GN=NFRKB PE=1 SV=2 - [NFRKB_HUMAN]	0.46	1	1	1	1					1.26	1.39	0.96	1.06	1299	138.9	9.25
Q9UHK0	Nuclear fragile X mental retardation-interacting protein 1 OS=Homo sapiens GN=NUFIP1 PE=1 SV=2 - [NUFP1_HUMAN]	3.03	2	2	2	2	0.95	0.96	0.89	0.91	0.78	1.02	0.69	0.91	495	56.3	9.13
Q8WUM0	Nuclear pore complex protein Nup133 OS=Homo sapiens GN=NUP133 PE=1 SV=2 - [NU133_HUMAN]	2.60	1	2	2	2		0.77		0.52	1.34	1.06	###	####	1156	128.9	5.10
Q92621	Nuclear pore complex protein Nup205 OS=Homo sapiens GN=NUP205 PE=1 SV=3 - [NU205_HUMAN]	2.24	1	1	3	3					1.75	2.03	0.86	1.00	2012	227.8	6.19
Q99567	Nuclear pore complex protein Nup88 OS=Homo sapiens GN=NUP88 PE=1 SV=2 - [NUP88_HUMAN]	4.05	1	2	2	4	1.13	1.13	1.49	1.49	1.60	1.67	1.33	1.40	741	83.5	5.69
Q8N1F7	Nuclear pore complex protein Nup93 OS=Homo sapiens GN=NUP93 PE=1 SV=2 - [NUP93_HUMAN]	1.47	1	1	1	1	1.28	1.24	1.32	1.30					819	93.4	5.72
P52948	Nuclear pore complex protein Nup98-Nup96 OS=Homo sapiens GN=NUP98 PE=1 SV=4 - [NUP98_HUMAN]	1.65	1	1	1	1					1.35	0.99	1.25	0.91	1817	197.5	6.40
Q9UND3	Nuclear pore complex-interacting protein OS=Homo sapiens GN=NPIP PE=2 SV=3 - [NPIP_HUMAN]	6.29	2	1	1	2	0.99	0.78	1.23	0.97					350	40.1	9.36
P37198	Nuclear pore glycoprotein p62 OS=Homo sapiens GN=NUP62 PE=1 SV=3 - [NUP62_HUMAN]	1.15	1	1	1	1	0.68	0.64	1.05	0.99					522	53.2	5.31
Q5VU65	Nuclear pore membrane glycoprotein 210-like OS=Homo sapiens GN=NUP210L PE=2 SV=1 - [P210L_HUMAN]	2.01	1	2	2	2	0.88	1.21	0.97	1.37					1888	210.5	7.50
Q15596	Nuclear receptor coactivator 2 OS=Homo sapiens GN=NCOA2 PE=1 SV=2 - [NCOA2_HUMAN]	1.23	1	1	1	1	0.87	1.22	0.77	1.08					1464	159.1	6.64
Q13772	Nuclear receptor coactivator 4 OS=Homo sapiens GN=NCOA4 PE=1 SV=1 - [NCOA4_HUMAN]	1.30	1	1	1	1					2.57	2.10	2.14	1.75	614	69.7	6.01
Q8NI08	Nuclear receptor coactivator 7 OS=Homo sapiens GN=NCOA7 PE=1 SV=2 - [NCOA7_HUMAN]	2.65	1	1	1	2	1.34	1.09	1.22	1.00					942	106.1	5.59
O75376	Nuclear receptor corepressor 1 OS=Homo sapiens GN=NCOR1 PE=1 SV=2 - [NCOR1_HUMAN]	2.42	1	2	2	2	1.15	1.08	1.16	1.10	0.87	0.75	0.69	0.59	2440	270.0	7.11
Q9Y618	Nuclear receptor corepressor 2 OS=Homo sapiens GN=NCOR2 PE=1 SV=2 - [NCOR2_HUMAN]	1.07	1	2	2	5					1.03	1.43	1.03	1.42	2525	274.6	7.59
O00482	Nuclear receptor subfamily 5 group A member 2 OS=Homo sapiens GN=NR5A2 PE=1 SV=2 - [NR5A2_HUMAN]	2.96	1	1	2	2					0.91	0.62	0.86	0.59	541	61.3	7.88
Q9H0G5	Nuclear speckle splicing regulatory protein 1 OS=Homo sapiens GN=NSRP1 PE=1 SV=1 - [NSRP1_HUMAN]	2.87	1	2	2	2	0.95	0.87	0.83	0.76					558	66.4	8.84
O15381	Nuclear valosin-containing protein-like OS=Homo sapiens GN=NVL PE=1 SV=1 - [NVL_HUMAN]	6.19	1	3	3	3	1.21	1.19	1.18	1.30					856	95.0	6.48

Q86WB0	Nuclear-interacting partner of ALK OS=Homo sapiens GN=ZC3HC1 PE=1 SV=1 - [NIPA_HUMAN]	3.59	1	1	1	1	1.30	1.16	1.29	1.16					502	55.2	5.62
P67809	Nuclease-sensitive element-binding protein 1 OS=Homo sapiens GN=YBX1 PE=1 SV=3 - [YBOX1_HUMAN]	5.86	1	1	1	1	1.00	1.06	1.17	1.25					324	35.9	9.88
Q02818	Nucleobindin-1 OS=Homo sapiens GN=NUCB1 PE=1 SV=4 - [NUCB1_HUMAN]	7.81	1	3	3	6					1.13	1.16	1.27	1.31	461	53.8	5.25
P80303	Nucleobindin-2 OS=Homo sapiens GN=NUCB2 PE=1 SV=2 - [NUCB2_HUMAN]	2.14	1	1	1	1	2.04	1.26	1.63	1.01					420	50.2	5.12
Q14978	Nucleolar and coiled-body phosphoprotein 1 OS=Homo sapiens GN=NOLC1 PE=1 SV=2 - [NOLC1_HUMAN]	2.00	1	1	1	1	0.70	0.79	0.76	0.87					699	73.6	9.47
Q9Y3T9	Nucleolar complex protein 2 homolog OS=Homo sapiens GN=NOC2L PE=1 SV=4 - [NOC2L_HUMAN]	1.60	1	1	1	2							1.58	0.11	749	84.9	5.62
O60287	Nucleolar pre-ribosomal-associated protein 1 OS=Homo sapiens GN=URB1 PE=1 SV=4 - [NPA1P_HUMAN]	1.45	1	2	2	4	1.14	1.22	1.33	1.44	0.48	1.34	0.60	1.69	2271	254.2	6.47
Q9BSC4	Nucleolar protein 10 OS=Homo sapiens GN=NOL10 PE=1 SV=1 - [NOL10_HUMAN]	1.45	1	1	1	1	0.87	1.51	0.70	1.20					688	80.3	8.46
O00567	Nucleolar protein 56 OS=Homo sapiens GN=NOP56 PE=1 SV=4 - [NOP56_HUMAN]	10.77	1	3	3	5	1.00	1.34	1.07	1.44	0.38	0.34	0.48	0.43	594	66.0	9.19
Q9Y2X3	Nucleolar protein 58 OS=Homo sapiens GN=NOP58 PE=1 SV=1 - [NOP58_HUMAN]	2.84	1	1	1	1					1.21	0.69	1.43	0.82	529	59.5	8.92
Q9H6R4	Nucleolar protein 6 OS=Homo sapiens GN=NOL6 PE=1 SV=2 [NOL6_HUMAN]	3.23	1	1	1	2							1.42	0.31	1146	127.5	7.64
O75607	Nucleoplasmin-3 OS=Homo sapiens GN=NPM3 PE=1 SV=3 - [NPM3_HUMAN]	2.81	1	1	1	1					0.23	0.30	0.50	0.65	178	19.3	4.63
Q5SRE5	Nucleoporin NUP188 homolog OS=Homo sapiens GN=NUP188 PE=1 SV=1 - [NU188_HUMAN]	0.69	1	1	1	1	0.53	0.68	0.52	0.66					1749	195.9	6.73
Q7Z3B4	Nucleoporin p54 OS=Homo sapiens GN=NUP54 PE=1 SV=2 - [NUP54_HUMAN]	3.16	1	1	1	2					1.39	1.05	1.12	0.85	507	55.4	7.02
O15504	Nucleoporin-like protein 2 OS=Homo sapiens GN=NUPL2 PE=1 SV=1 - [NUPL2_HUMAN]	4.73	1	1	1	1	0.85	0.86	0.78	0.80					423	44.8	9.19
P15531	Nucleoside diphosphate kinase A OS=Homo sapiens GN=NME1 PE=1 SV=1 - [NDKA_HUMAN]	23.03	3	3	3	9					0.85	0.95	0.84	0.91	152	17.1	6.19
A8MXV4	Nucleoside diphosphate-linked moiety X motif 19, mitochondrial OS=Homo sapiens GN=NUDT19 PE=1 SV=1 - [NUD19_HUMAN]	3.73	1	1	1	1	0.70	1.22	0.82	1.43					375	42.2	7.64
Q9BRQ3	Nucleoside diphosphate-linked moiety X motif 22 OS=Homo sapiens GN=NUDT22 PE=2 SV=3 - [NUD22_HUMAN]	4.95	1	1	1	2	0.97	0.66	1.15	0.79					303	32.6	5.11
Q99733	Nucleosome assembly protein 1-like 4 OS=Homo sapiens GN=NAP1L4 PE=1 SV=1 - [NP1L4_HUMAN]	5.07	1	1	1	1					1.00	0.92	0.88	0.81	375	42.8	4.69
Q9Y239	Nucleotide-binding oligomerization domain-containing protein 1 OS=Homo sapiens GN=NOD1 PE=1 SV=1 - [NOD1_HUMAN]	0.84	1	1	1	1	1.48	2.15	1.28	1.85					953	107.6	7.11
Q96RS6	NudC domain-containing protein 1 OS=Homo sapiens GN=NUDCD1 PE=1 SV=2 - [NUDC1_HUMAN]	2.57	1	1	1	1					1.12	1.21	1.18	1.28	583	66.7	5.11
Q5VST9	Obscurin OS=Homo sapiens GN=OBSCN PE=1 SV=3 - [OBSCN_HUMAN]	1.03	1	3	4	407	1.26	1.88	1.39	2.08	2.14	2.13	1.83	1.82	7968	867.9	5.99
O75147	Obscurin-like protein 1 OS=Homo sapiens GN=OBSL1 PE=1 SV=4 - [OBSL1_HUMAN]	0.95	1	1	1	2	1.52	1.45	1.33	1.27					1896	206.8	5.63
Q6UX06	Olfactomedin-4 OS=Homo sapiens GN=OLFM4 PE=1 SV=1 - [OLFM4_HUMAN]	5.49	1	1	2	3	1.12	0.93	0.66	0.55					510	57.2	5.69
Q6IF99	Olfactory receptor 10K2 OS=Homo sapiens GN=OR10K2 PE=2 SV=1 - [O10K2_HUMAN]	1.92	1	1	1	2	1.15	1.15	1.25	1.27					312	35.0	8.57
Q8NGE3	Olfactory receptor 10P1 OS=Homo sapiens GN=OR10P1 PE=2 SV=1 - [O10P1_HUMAN]	1.92	1	1	1	1					1.10	1.25	1.09	1.23	313	34.7	9.70
Q8NGX3	Olfactory receptor 10T2 OS=Homo sapiens GN=OR10T2 PE=2 SV=1 - [O10T2_HUMAN]	9.24	1	1	1	1	1.16	1.30	1.12	1.27					314	35.0	9.03

B2RN74	Olfactory receptor 11H12 OS=Homo sapiens GN=OR11H12 PE=2 SV=1 - [O11HC_HUMAN]	10.43	3	2	2	6	0.96	1.04	0.95	1.04					326	36.5	7.84
Q8NHC7	Olfactory receptor 14C36 OS=Homo sapiens GN=OR14C36 PE=2 SV=1 - [O14CZ_HUMAN]	12.18	1	2	2	2	1.12	1.28	0.88	1.01	0.87	1.05	1.08	1.30	312	34.8	8.35
P47884	Olfactory receptor 1D4 OS=Homo sapiens GN=OR1D4 PE=2 SV=3 - [OR1D4_HUMAN]	10.61	2	2	2	7	1.09	1.02	1.06	1.00					311	35.2	8.02
Q6IF42	Olfactory receptor 2A2 OS=Homo sapiens GN=OR2A2 PE=2 SV=2 - [OR2A2_HUMAN]	5.35	1	1	1	2	1.10	0.80	0.88	0.64					318	35.8	8.37
Q9H210	Olfactory receptor 2D2 OS=Homo sapiens GN=OR2D2 PE=2 SV=4 - [OR2D2_HUMAN]	2.27	1	1	1	1					0.86	1.06	0.62	0.77	308	34.1	9.13
Q9GZK4	Olfactory receptor 2H1 OS=Homo sapiens GN=OR2H1 PE=2 SV=1 - [OR2H1_HUMAN]	1.90	1	1	1	1	1.13	0.88	0.96	0.76					316	35.3	8.72
Q6IEZ7	Olfactory receptor 2T5 OS=Homo sapiens GN=OR2T5 PE=2 SV=1 - [OR2T5_HUMAN]	2.54	3	1	1	2					1.28	1.09	1.34	1.14	315	35.6	8.40
Q8NHC8	Olfactory receptor 2T6 OS=Homo sapiens GN=OR2T6 PE=2 SV=2 - [OR2T6_HUMAN]	3.90	1	1	1	1	1.80	2.00	1.98	2.20					308	34.7	8.06
Q6IF82	Olfactory receptor 4A47 OS=Homo sapiens GN=OR4A47 PE=2 SV=2 - [O4A47_HUMAN]	2.27	1	1	1	4					0.77	0.77	0.81	0.89	309	34.7	8.46
A6NHA9	Olfactory receptor 4C46 OS=Homo sapiens GN=OR4C46 PE=2 SV=1 - [O4C46_HUMAN]	16.18	1	2	2	7	1.61	1.83	1.87	2.15	1.07	1.31	0.96	1.18	309	34.5	7.91
Q8NGC6	Olfactory receptor 4K17 OS=Homo sapiens GN=OR4K17 PE=2 SV=3 - [OR4KH_HUMAN]	3.81	1	2	2	5					0.87	0.68	0.67	0.53	315	35.3	8.72
Q8TCB6	Olfactory receptor 51E1 OS=Homo sapiens GN=OR51E1 PE=2 SV=1 - [O51E1_HUMAN]	3.47	1	1	1	1					0.87	1.05	0.88	1.06	317	35.2	8.40
A6NGY5	Olfactory receptor 51F1 OS=Homo sapiens GN=OR51F1 PE=2 SV=1 - [O51F1_HUMAN]	5.33	1	1	1	2	1.14	1.29	1.01	1.15					319	35.8	8.03
Q8NGF1	Olfactory receptor 52R1 OS=Homo sapiens GN=OR52R1 PE=2 SV=2 - [O52R1_HUMAN]	3.17	1	1	1	1					0.60	0.73	0.83	1.01	315	34.9	8.16
Q9NZP5	Olfactory receptor 5AC2 OS=Homo sapiens GN=OR5AC2 PE=2 SV=2 - [O5AC2_HUMAN]	2.59	1	1	1	1	0.37	0.48	0.44	0.58					309	35.3	8.94
Q8NH48	Olfactory receptor 5B3 OS=Homo sapiens GN=OR5B3 PE=2 SV=1 - [OR5B3_HUMAN]	2.23	1	1	1	1					0.90	0.85	1.08	1.02	314	35.2	6.96
Q8NGR4	Olfactory receptor 5C1 OS=Homo sapiens GN=OR5C1 PE=2 SV=1 - [OR5C1_HUMAN]	8.13	1	1	1	1	1.12	1.01	1.15	1.05					320	35.0	8.53
Q8NGL1	Olfactory receptor 5D18 OS=Homo sapiens GN=OR5D18 PE=2 SV=1 - [OR5DI_HUMAN]	5.43	1	1	1	1	1.06	1.03	1.04	1.02					313	35.3	8.34
Q8NGV7	Olfactory receptor 5H2 OS=Homo sapiens GN=OR5H2 PE=2 SV=3 - [OR5H2_HUMAN]	1.91	1	1	1	1					0.76	0.12	0.93	0.15	314	35.9	7.65
Q8WZ92	Olfactory receptor 5P2 OS=Homo sapiens GN=OR5P2 PE=2 SV=1 - [OR5P2_HUMAN]	5.59	1	1	1	1					0.45		0.83		322	35.8	7.84
O95007	Olfactory receptor 6B1 OS=Homo sapiens GN=OR6B1 PE=2 SV=1 - [OR6B1_HUMAN]	4.18	1	1	1	1	0.62	0.99	1.01	1.61					311	35.3	8.46
Q9NZP2	Olfactory receptor 6C2 OS=Homo sapiens GN=OR6C2 PE=2 SV=2 - [OR6C2_HUMAN]	5.13	1	1	1	2					1.00	1.25	0.96	1.11	312	35.2	8.66
Q8NGQ2	Olfactory receptor 6Q1 OS=Homo sapiens GN=OR6Q1 PE=2 SV=2 - [OR6Q1_HUMAN]	2.52	1	1	1	1	0.20	0.21	0.18	0.19					317	35.7	8.06
Q8NGN1	Olfactory receptor 6T1 OS=Homo sapiens GN=OR6T1 PE=2 SV=1 - [OR6T1_HUMAN]	5.26	1	1	1	1					0.52	0.90	2.12	3.69	323	36.3	9.22
O60412	Olfactory receptor 7C2 OS=Homo sapiens GN=OR7C2 PE=2 SV=4 - [OR7C2_HUMAN]	2.82	1	1	1	2	0.29	0.33	0.26	0.29					319	35.3	7.80
Q96RA2	Olfactory receptor 7D2 OS=Homo sapiens GN=OR7D2 PE=2 SV=2 - [OR7D2_HUMAN]	1.60	1	1	1	1					0.80	1.07	0.62	0.83	312	34.7	7.05
Q8WWZ8	Oncoprotein-induced transcript 3 protein OS=Homo sapiens GN=OIT3 PE=1 SV=2 - [OIT3_HUMAN]	12.29	1	5	5	10	1.11	1.15	1.10	1.13	0.58	0.65	0.85	0.95	545	60.0	5.58

Q99650	Oncostatin-M-specific receptor subunit beta OS=Homo sapiens GN=OSMR PE=1 SV=1 - [OSMR_HUMAN]	3.17	1	2	2	3	0.79	0.83	0.79	0.83					979	110.4	5.82
O75665	Oral-facial-digital syndrome 1 protein OS=Homo sapiens GN=OFD1 PE=1 SV=1 - [OFD1_HUMAN]	4.05	1	2	2	2	1.03	1.05	0.98	1.02					1012	116.6	6.10
Q9UBD5	Origin recognition complex subunit 3 OS=Homo sapiens GN=ORC3 PE=1 SV=1 - [ORC3_HUMAN]	2.81	1	1	1	1	1.01	0.90	0.93	0.83					711	82.2	7.61
O43929	Origin recognition complex subunit 4 OS=Homo sapiens GN=ORC4 PE=1 SV=2 - [ORC4_HUMAN]	3.44	1	1	1	1					1.73	1.34	1.53	1.19	436	50.3	8.00
P00480	Ornithine carbamoyltransferase, mitochondrial OS=Homo sapiens GN=OTC PE=1 SV=3 - [OTC_HUMAN]	2.54	1	1	1	4					1.45	1.51	1.27	1.32	354	39.9	8.63
P11926	Ornithine decarboxylase OS=Homo sapiens GN=ODC1 PE=1 SV=2 - [DCOR_HUMAN]	2.17	1	1	1	1					0.86	1.15	0.62	0.83	461	51.1	5.26
Q8IYS5	Osteoclast-associated immunoglobulin-like receptor OS=Homo sapiens GN=OSCAR PE=2 SV=3 - [OSCAR_HUMAN]	5.32	1	1	1	1	0.84	1.21	0.75	1.09					282	30.5	6.52
Q99983	Osteomodulin OS=Homo sapiens GN=OMD PE=1 SV=1 - [OMD_HUMAN]	6.65	1	1	1	2					1.69	1.27	1.50	1.12	421	49.5	5.59
P10451	Osteopontin OS=Homo sapiens GN=SPP1 PE=1 SV=1 - [OSTP_HUMAN]	5.41	1	1	1	3					1.15	1.04	1.18	1.08	314	35.4	4.58
Q02509	Otoconin-90 OS=Homo sapiens GN=OC90 PE=2 SV=3 - [OC90_HUMAN]	5.07	1	1	1	1	1.25	1.24	0.83	0.83					493	53.4	4.94
Q6ZRI0	Otogelin OS=Homo sapiens GN=OTOG PE=2 SV=3 - [OTOG_HUMAN]	1.47	1	2	2	4	1.17	0.70	1.19	0.66	0.82	0.96	0.66	0.77	2925	314.6	5.91
A6NHN0	Otolin-1 OS=Homo sapiens GN=OTOL1 PE=3 SV=1 - [OTOL1_HUMAN]	5.87	1	2	2	2	0.22	0.20	0.21	0.19	0.87	0.91	0.84	0.89	477	49.4	8.34
Q5T2D3	OTU domain-containing protein 3 OS=Homo sapiens GN=OTUD3 PE=1 SV=1 - [OTUD3_HUMAN]	2.26	1	1	1	1					0.56	0.67	1.09	1.29	398	45.1	8.29
Q8TE49	OTU domain-containing protein 7A OS=Homo sapiens GN=OTUD7A PE=1 SV=1 - [OTU7A_HUMAN]	1.30	1	1	1	1	0.46	0.44	0.43	0.42					926	100.6	8.40
Q86UD1	Out at first protein homolog OS=Homo sapiens GN=OAF PE=2 SV=1 - [OAF_HUMAN]	23.44	1	4	4	19	0.90	0.91	0.93	0.94	1.13	1.12	1.02	1.02	273	30.7	6.84
Q9ULJ1	Outer dense fiber protein 2-like OS=Homo sapiens GN=ODF2L PE=2 SV=2 - [ODF2L_HUMAN]	3.77	1	2	2	3	0.86	0.76	0.95	0.85	0.90	0.90	1.06	1.10	636	73.7	6.48
Q8IXM7	Outer dense fiber protein 3-like protein 1 OS=Homo sapiens GN=ODF3L1 PE=2 SV=1 - [OD3L1_HUMAN]	5.84	1	1	1	1					0.80	1.00	0.64	0.80	274	31.0	9.54
Q12889	Oviduct-specific glycoprotein OS=Homo sapiens GN=OVGP1 PE=2 SV=1 - [OVGP1_HUMAN]	2.06	1	1	1	1	1.54	2.17	1.55	2.18					678	75.4	8.78
Q7RTY7	Ovochymase-1 OS=Homo sapiens GN=OVCH1 PE=2 SV=2 - [OVCH1_HUMAN]	5.20	1	4	4	8	1.50	1.54	1.44	1.49	1.02	0.95	0.99	0.92	1134	125.0	8.32
Q7RTZ1	Ovochymase-2 OS=Homo sapiens GN=OVCH2 PE=1 SV=2 - [OVCH2_HUMAN]	3.37	1	2	2	4	3.31	2.11	1.28	0.93	1.17	1.43	1.29	1.66	564	62.6	7.43
Q6IE37	Ovostatin homolog 1 OS=Homo sapiens GN=OVOS1 PE=2 SV=2 - [OVOS1_HUMAN]	1.18	1	1	2	4					0.57	0.98	0.52	0.91	1185	134.4	5.29
Q6IE36	Ovostatin homolog 2 OS=Homo sapiens GN=OVOS2 PE=2 SV=2 - [OVOS2_HUMAN]	2.58	1	1	1	1					0.74	0.88	0.83	0.99	1432	161.1	5.27
Q8N573	Oxidation resistance protein 1 OS=Homo sapiens GN=OXR1 PE=1 SV=2 - [OXR1_HUMAN]	4.00	1	2	2	3	0.91	0.60	0.82	0.54	1.31	1.37	1.23	1.28	874	97.9	5.47
P56715	Oxygen-regulated protein 1 OS=Homo sapiens GN=RP1 PE=1 SV=1 - [RP1_HUMAN]	0.37	1	1	1	1					0.86		0.51		2156	240.5	5.80
Q9BXW6	Oxysterol-binding protein-related protein 1 OS=Homo sapiens GN=OSBPL1A PE=1 SV=2 - [OSBL1_HUMAN]	3.16	1	1	1	1	1.19	1.00	1.31	1.11					950	108.4	6.38
Q9H1P3	Oxysterol-binding protein-related protein 2 OS=Homo sapiens GN=OSBPL2 PE=1 SV=1 - [OSBL2_HUMAN]	10.00	1	2	2	3	0.14	0.32	1.38	3.12	0.85	1.21	0.75	1.08	480	55.2	6.35
Q9H4L5	Oxysterol-binding protein-related protein 3 OS=Homo sapiens GN=OSBPL3 PE=1 SV=1 - [OSBL3_HUMAN]	1.24	1	1	1	1	0.82	0.95	1.03	1.20					887	101.2	6.87

Q9BZF1	Oxysterol-binding protein-related protein 8 OS=Homo sapiens GN=OSBPL8 PE=1 SV=3 - [OSBL8_HUMAN]	1.80	1	1	1	1					1.04	0.96	0.85	0.78	889	101.1	6.96
P16109	P-selectin OS=Homo sapiens GN=SELP PE=1 SV=3 - [LYAM3_HUMAN]	15.54	1	10	10	24	1.06	1.10	1.08	1.13	1.11	0.94	1.09	0.96	830	90.8	6.60
Q9UBL9	P2X purinoceptor 2 OS=Homo sapiens GN=P2RX2 PE=2 SV=1 - [P2RX2_HUMAN]	3.61	1	1	1	1					0.74	0.80	0.79	0.85	471	51.7	8.09
O15547	P2X purinoceptor 6 OS=Homo sapiens GN=P2RX6 PE=1 SV=2 - [P2RX6_HUMAN]	8.16	1	1	1	2					0.97	0.31	1.59	0.51	441	48.8	7.58
Q9BPV8	P2Y purinoceptor 13 OS=Homo sapiens GN=P2RY13 PE=2 SV=3 - [P2Y13_HUMAN]	3.39	1	1	1	1	1.30	0.94	1.08	0.79					354	40.8	9.92
Q15391	P2Y purinoceptor 14 OS=Homo sapiens GN=P2RY14 PE=1 SV=1 - [P2Y14_HUMAN]	3.25	1	1	1	1	0.88	0.86	0.84	0.83					338	38.9	9.47
Q96ST3	Paired amphipathic helix protein Sin3a OS=Homo sapiens GN=SIN3A PE=1 SV=2 - [SIN3A_HUMAN]	2.91	1	1	1	3	1.38	1.26	0.97	0.89					1273	145.1	7.25
P23760	Paired box protein Pax-3 OS=Homo sapiens GN=PAX3 PE=1 SV=2 - [PAX3_HUMAN]	8.14	1	2	2	2					0.76	0.64	1.00	0.77	479	52.9	8.72
P23759	Paired box protein Pax-7 OS=Homo sapiens GN=PAX7 PE=2 SV=3 - [PAX7_HUMAN]	6.15	1	2	2	2					0.79	0.73	0.79	0.72	520	56.9	8.73
P54821	Paired mesoderm homeobox protein 1 OS=Homo sapiens GN=PRRX1 PE=1 SV=2 - [PRRX1_HUMAN]	5.31	2	1	1	1					0.29	0.77	0.35	0.93	245	27.3	9.47
O14813	Paired mesoderm homeobox protein 2A OS=Homo sapiens GN=PHOX2A PE=1 SV=2 - [PHX2A_HUMAN]	2.46	1	1	1	1					1.13	1.20	0.95	1.01	284	29.6	8.76
Q9UIJ5	Palmitoyltransferase ZDHHC2 OS=Homo sapiens GN=ZDHHC2 PE=2 SV=1 - [ZDHC2_HUMAN]	3.81	1	1	1	13	0.40	0.39	0.96	0.94	0.86	0.87	0.85	0.95	367	42.0	8.35
P04746	Pancreatic alpha-amylase OS=Homo sapiens GN=AMY2A PE=1 SV=2 - [AMYP_HUMAN]	2.15	1	1	1	2	1.42	1.48	1.63	1.72					511	57.7	7.05
Q17RR3	Pancreatic lipase-related protein 3 OS=Homo sapiens GN=PNLIPRP3 PE=2 SV=2 - [LIPR3_HUMAN]	3.85	1	1	1	1	1.17	1.23	0.93	0.98					467	52.2	8.27
P00995	Pancreatic secretory trypsin inhibitor OS=Homo sapiens GN=SPINK1 PE=1 SV=2 - [ISK1_HUMAN]	10.13	1	1	1	3					1.23	1.41	1.38	1.58	79	8.5	7.61
O95497	Pantetheinase OS=Homo sapiens GN=VNN1 PE=1 SV=2 - [VNN1_HUMAN]	11.89	3	4	4	9	1.12	1.16	1.14	1.17	1.17	0.90	1.22	0.93	513	57.0	5.55
Q9NVE7	Pantothenate kinase 4 OS=Homo sapiens GN=PANK4 PE=1 SV=1 - [PANK4_HUMAN]	1.42	1	1	1	1	1.17	1.33	1.33	1.53					773	85.9	6.28
O95428	Papilin OS=Homo sapiens GN=PAPLN PE=2 SV=4 - [PPN_HUMAN]	1.17	1	1	1	2	1.08	0.93	1.16	1.01					1278	137.6	6.89
Q13219	Pappalysin-1 OS=Homo sapiens GN=PAPPA PE=1 SV=3 - [PAPP1_HUMAN]	14.63	1	19	19	38	2.65	3.92	2.98	3.97					1627	180.9	6.18
Q9UQ90	Paraplegin OS=Homo sapiens GN=SPG7 PE=1 SV=2 - [SPG7_HUMAN]	2.01	2	1	2	2					1.49	1.22	1.14	0.93	795	88.2	8.69
Q9BRP8	Partner of Y14 and mago OS=Homo sapiens GN=WIBG PE=1 SV=1 - [WIBG_HUMAN]	15.69	1	1	2	2					1.27	1.27	1.13	1.13	204	22.6	9.45
Q96RG2	PAS domain-containing serine/threonine-protein kinase OS=Homo sapiens GN=PASK PE=1 SV=3 - [PASK_HUMAN]	2.12	1	1	1	1	0.60	0.76	1.03	1.32					1323	142.8	4.86
Q96NR3	Patched domain-containing protein 1 OS=Homo sapiens GN=PTCHD1 PE=2 SV=2 - [PTHD1_HUMAN]	2.59	1	1	1	1					0.22	0.51	0.18	0.41	888	101.3	8.27
Q9GZU2	Paternally-expressed gene 3 protein OS=Homo sapiens GN=PEG3 PE=2 SV=1 - [PEG3_HUMAN]	3.27	1	3	3	6	1.22	0.81	1.14	0.76	0.79	0.66	0.84	0.70	1588	180.7	5.48
Q9Y365	PCTP-like protein OS=Homo sapiens GN=STARD10 PE=2 SV=2 - [PCTL_HUMAN]	5.50	1	1	1	2					0.95	1.00	0.98	1.04	291	33.0	7.12
O00151	PDZ and LIM domain protein 1 OS=Homo sapiens GN=PDLIM1 PE=1 SV=4 - [PDLI1_HUMAN]	38.91	1	9	9	56	0.30	0.27	0.29	0.27	1.17	1.18	1.19	1.20	329	36.0	7.02
Q96HC4	PDZ and LIM domain protein 5 OS=Homo sapiens GN=PDLIM5 PE=1 SV=5 - [PDLI5_HUMAN]	10.23	1	5	5	15					1.20	1.14	1.18	1.12	596	63.9	8.21

Q9NR12	PDZ and LIM domain protein 7 OS=Homo sapiens GN=PDLIM7 PE=1 SV=1 - [PDLI7_HUMAN]	19.47	1	7	7	29	0.91	0.88	0.96	0.93	1.30	1.23	1.23	1.22	457	49.8	8.41
O15018	PDZ domain-containing protein 2 OS=Homo sapiens GN=PDZD2 PE=1 SV=4 - [PDZD2_HUMAN]	0.56	1	1	2	2	1.37	1.34	1.30	1.28					2839	301.5	7.43
Q76G19	PDZ domain-containing protein 4 OS=Homo sapiens GN=PDZD4 PE=2 SV=1 - [PDZD4_HUMAN]	2.21	1	2	2	3					0.68	0.86	0.70	0.91	769	86.1	6.13
Q9H6Y5	PDZ domain-containing protein MAGIX OS=Homo sapiens GN=MAGIX PE=1 SV=3 - [MAGIX_HUMAN]	7.78	1	1	2	2					1.00	0.98	1.21	1.19	334	35.2	10.81
Q96RV3	Pecanex-like protein 1 OS=Homo sapiens GN=PCNX PE=2 SV=2 - [PCX1_HUMAN]	0.81	1	1	1	1					1.06	1.18	1.30	1.43	2341	258.5	7.21
A6NKB5	Pecanex-like protein 2 OS=Homo sapiens GN=PCNXL2 PE=1 SV=3 - [PCX2_HUMAN]	0.33	1	1	1	1	0.83	0.96	0.84	0.97					2137	237.1	6.76
Q63HM2	Pecanex-like protein C14orf135 OS=Homo sapiens GN=C14orf135 PE=2 SV=4 - [CN135_HUMAN]	1.45	1	1	1	3	0.79	1.36	1.30	2.24					1172	132.6	6.29
O75127	Pentatricopeptide repeat-containing protein 1 OS=Homo sapiens GN=PTCD1 PE=1 SV=2 - [PTCD1_HUMAN]	7.00	1	2	2	3	1.34	1.58	1.19	1.42					700	78.8	8.59
Q8WV60	Pentatricopeptide repeat-containing protein 2 OS=Homo sapiens GN=PTCD2 PE=2 SV=3 - [PTCD2_HUMAN]	4.38	1	1	1	4	0.94	0.98	0.85	0.90	0.82	0.93	0.71	0.80	388	43.9	9.23
P26022	Pentraxin-related protein PTX3 OS=Homo sapiens GN=PTX3 PE=1 SV=3 - [PTX3_HUMAN]	17.06	1	4	4	6	1.00	1.14	1.00	1.07					381	41.9	5.01
Q6UXB8	Peptidase inhibitor 16 OS=Homo sapiens GN=PI16 PE=1 SV=1 - [PI16_HUMAN]	27.86	1	10	10	77	1.07	0.95	0.94	0.85	1.04	1.08	0.76	0.75	463	49.4	5.39
Q9UGC7	Peptide chain release factor 1-like, mitochondrial OS=Homo sapiens GN=MTRF1L PE=1 SV=1 - [RF1ML_HUMAN]	3.68	1	1	1	1	1.27	1.49	1.27	1.50					380	43.6	8.34
Q9HBH1	Peptide deformylase, mitochondrial OS=Homo sapiens GN=PDF PE=1 SV=1 - [DEFM_HUMAN]	15.64	1	2	2	2					0.86	1.01	0.67	0.79	243	27.0	9.16
Q96IV0	Peptide-N(4)-(N-acetyl-beta-glucosaminyl)asparagine amidase OS=Homo sapiens GN=NGLY1 PE=1 SV=1 - [NGLY1_HUMAN]	1.68	1	1	1	3	1.40	1.28	1.35	1.25	1.59	1.71	1.43	1.54	654	74.3	6.89
O75594	Peptidoglycan recognition protein 1 OS=Homo sapiens GN=PGLYRP1 PE=1 SV=1 - [PGRP1_HUMAN]	15.82	1	2	2	7	1.23	1.22	1.37	1.38	1.19	1.30	1.22	1.30	196	21.7	8.59
P19021	Peptidyl-glycine alpha-amidating monooxygenase OS=Homo sapiens GN=PAM PE=1 SV=2 - [AMD_HUMAN]	6.37	1	6	6	24	1.24	1.37	1.33	1.43	1.11	0.73	1.00	0.81	973	108.3	6.42
P62937	Peptidyl-prolyl cis-trans isomerase A OS=Homo sapiens GN=PPIA PE=1 SV=2 - [PPIA_HUMAN]	67.88	3	8	10	113	0.94	0.88	0.89	0.88	0.96	1.04	1.00	1.03	165	18.0	7.81
A2BFH1	Peptidyl-prolyl cis-trans isomerase A-like 4G OS=Homo sapiens GN=PPIAL4G PE=2 SV=1 - [PAL4G_HUMAN]	20.73	1	1	2	8	1.09	1.31	1.21	1.46					164	18.2	9.06
P23284	Peptidyl-prolyl cis-trans isomerase B OS=Homo sapiens GN=PPIB PE=1 SV=2 - [PPIB_HUMAN]	42.59	2	9	10	35	0.95	0.93	0.99	0.90	0.99	1.11	0.98	1.07	216	23.7	9.41
P45877	Peptidyl-prolyl cis-trans isomerase C OS=Homo sapiens GN=PPIC PE=1 SV=1 - [PPIC_HUMAN]	6.60	2	1	2	2					1.64	1.84	1.39	1.56	212	22.7	8.40
Q6UX04	Peptidyl-prolyl cis-trans isomerase CWC27 homolog OS=Homo sapiens GN=CWC27 PE=1 SV=1 - [CWC27_HUMAN]	2.75	1	1	1	3	0.96	1.01	1.01	1.06					472	53.8	5.80
Q08752	Peptidyl-prolyl cis-trans isomerase D OS=Homo sapiens GN=PPID PE=1 SV=3 - [PPID_HUMAN]	14.86	2	2	3	3					0.74	0.97	0.75	0.88	370	40.7	7.21
Q9UNP9	Peptidyl-prolyl cis-trans isomerase E OS=Homo sapiens GN=PPIE PE=1 SV=1 - [PPIE_HUMAN]	3.99	1	1	1	2					1.05	0.17	1.18	0.19	301	33.4	5.60
Q96AY3	Peptidyl-prolyl cis-trans isomerase FKBP10 OS=Homo sapiens GN=FKBP10 PE=1 SV=1 - [FKB10_HUMAN]	2.41	1	1	1	1					0.78	0.89	0.84	0.95	582	64.2	5.62
P62942	Peptidyl-prolyl cis-trans isomerase FKBP1A OS=Homo sapiens GN=FKBP1A PE=1 SV=2 - [FKB1A_HUMAN]	25.00	1	2	2	14	0.84	0.78	0.77	0.72	1.15	1.06	1.07	1.01	108	11.9	8.16
P26885	Peptidyl-prolyl cis-trans isomerase FKBP2 OS=Homo sapiens GN=FKBP2 PE=1 SV=2 - [FKBP2_HUMAN]	49.30	1	5	5	14					1.50	1.56	1.39	1.34	142	15.6	9.13
Q00688	Peptidyl-prolyl cis-trans isomerase FKBP3 OS=Homo sapiens GN=FKBP3 PE=1 SV=1 - [FKBP3_HUMAN]	3.57	1	1	1	3					1.09	0.82	1.00	0.74	224	25.2	9.28

Q13451	Peptidyl-prolyl cis-trans isomerase FKBP5 OS=Homo sapiens GN=FKBP5 PE=1 SV=2 - [FKBP5_HUMAN]	3.06	1	1	1	1	0.74	0.71	0.75	0.73					457	51.2	5.90
Q9Y680	Peptidyl-prolyl cis-trans isomerase FKBP7 OS=Homo sapiens GN=FKBP7 PE=1 SV=1 - [FKBP7_HUMAN]	4.63	1	1	1	1					0.95	0.98	1.11	1.14	259	30.0	6.54
Q13526	Peptidyl-prolyl cis-trans isomerase NIMA-interacting 1 OS=Homo sapiens GN=PIN1 PE=1 SV=1 - [PIN1_HUMAN]	9.20	1	1	1	3					1.33	1.39	1.07	1.13	163	18.2	8.82
Q9Y3C6	Peptidyl-prolyl cis-trans isomerase-like 1 OS=Homo sapiens GN=PPIL1 PE=1 SV=1 - [PPIL1_HUMAN]	6.63	1	1	1	1					2.10	1.74	1.27	1.05	166	18.2	7.99
Q13356	Peptidyl-prolyl cis-trans isomerase-like 2 OS=Homo sapiens GN=PPIL2 PE=1 SV=1 - [PPIL2_HUMAN]	3.27	1	1	1	1					1.07	1.26	0.94	1.10	520	58.8	8.78
Q8IXY8	Peptidyl-prolyl cis-trans isomerase-like 6 OS=Homo sapiens GN=PPIL6 PE=2 SV=1 - [PPIL6_HUMAN]	1.93	1	1	1	1					0.46	0.84	0.67	1.21	311	35.2	7.02
Q96BP3	Peptidylprolyl isomerase domain and WD repeat-containing protein 1 OS=Homo sapiens GN=PPWD1 PE=1 SV=1 - [PPWD1_HUMAN]	1.39	1	1	1	1					1.01	1.03	0.98	0.99	646	73.5	7.15
P55201	Peregrin OS=Homo sapiens GN=BRPF1 PE=1 SV=2 - [BRPF1_HUMAN]	2.22	1	2	2	2					4.09	4.36	1.18	1.25	1214	137.4	7.93
Q9BXM0	Periaxin OS=Homo sapiens GN=PRX PE=1 SV=2 - [PRAX_HUMAN]	1.10	1	2	2	2	1.00	0.94	1.29	1.22	0.96	1.07	0.94	1.04	1461	154.8	7.50
O95613	Pericentrin OS=Homo sapiens GN=PCNT PE=1 SV=4 - [PCNT_HUMAN]	0.78	1	2	2	3					0.29	0.23	1.18	0.93	3336	377.8	5.55
Q15154	Pericentriolar material 1 protein OS=Homo sapiens GN=PCM1 PE=1 SV=4 - [PCM1_HUMAN]	3.11	4	2	3	98					0.92	0.88	0.67	0.88	2024	228.4	5.02
O60664	Perilipin-3 OS=Homo sapiens GN=PLIN3 PE=1 SV=3 - [PLIN3_HUMAN]	4.38	1	1	1	1					2.01	1.81	1.84	1.66	434	47.0	5.44
P56645	Period circadian protein homolog 3 OS=Homo sapiens GN=PER3 PE=2 SV=4 - [PER3_HUMAN]	1.58	1	1	1	1	0.90	0.91	0.93	0.95					1201	131.8	6.89
Q15063	Periostin OS=Homo sapiens GN=POSTN PE=1 SV=2 - [POSTN_HUMAN]	24.28	1	17	17	55	0.87	0.83	0.85	0.84	0.61	0.72	0.64	0.73	836	93.3	7.53
O95153	Peripheral-type benzodiazepine receptor-associated protein 1 OS=Homo sapiens GN=BZRAP1 PE=1 SV=2 - [RIMB1_HUMAN]	1.08	1	1	1	1	0.66	0.83	0.80	1.01					1857	199.9	5.11
P23942	Peripherin-2 OS=Homo sapiens GN=PRPH2 PE=1 SV=1 - [PRPH2_HUMAN]	2.02	1	1	1	1	5.02		1.51						346	39.2	8.18
O60437	Periplakin OS=Homo sapiens GN=PPL PE=1 SV=4 - [PEPL_HUMAN]	1.88	1	3	3	4	0.65	0.70	0.67	0.72	0.68	0.75	0.74	0.81	1756	204.6	5.60
Q92626	Peroxidasin homolog OS=Homo sapiens GN=PXDN PE=1 SV=2 - [PXDN_HUMAN]	1.49	1	2	2	3	1.71	4.04	1.45	1.54					1479	165.2	7.17
A1KZ92	Peroxidasin-like protein OS=Homo sapiens GN=PXDNL PE=2 SV=3 - [PXDNL_HUMAN]	0.96	1	1	1	1	1.06	0.94	1.08	0.97					1463	163.6	7.43
Q06830	Peroxiredoxin-1 OS=Homo sapiens GN=PRDX1 PE=1 SV=1 - [PRDX1_HUMAN]	13.57	1	1	2	6					0.63	0.90	0.90	1.30	199	22.1	8.13
P32119	Peroxiredoxin-2 OS=Homo sapiens GN=PRDX2 PE=1 SV=5 - [PRDX2_HUMAN]	40.91	1	6	7	29	0.66	0.62	0.61	0.59	0.71	0.74	0.63	0.65	198	21.9	5.97
P30044	Peroxiredoxin-5, mitochondrial OS=Homo sapiens GN=PRDX5 PE=1 SV=4 - [PRDX5_HUMAN]	35.98	1	5	5	24	0.75	0.72	0.81	0.78	0.94	1.05	0.87	1.01	214	22.1	8.70
P30041	Peroxiredoxin-6 OS=Homo sapiens GN=PRDX6 PE=1 SV=3 - [PRDX6_HUMAN]	46.88	1	8	8	25	0.77	0.67	0.79	0.73	0.95	0.93	0.91	0.94	224	25.0	6.38
O15254	Peroxisomal acyl-coenzyme A oxidase 3 OS=Homo sapiens GN=ACOX3 PE=1 SV=2 - [ACOX3_HUMAN]	3.00	1	1	1	2					2.59	1.60	2.28	1.34	700	77.6	7.25
Q08426	Peroxisomal bifunctional enzyme OS=Homo sapiens GN=EHHADH PE=1 SV=3 - [ECHP_HUMAN]	3.32	1	1	1	2					1.08	0.88	0.62	0.50	723	79.4	9.14
P40855	Peroxisomal biogenesis factor 19 OS=Homo sapiens GN=PEX19 PE=1 SV=1 - [PEX19_HUMAN]	3.34	1	1	1	1	1.24	1.27	1.27	1.32					299	32.8	4.34
Q9UKG9	Peroxisomal carnitine O-octanoyltransferase OS=Homo sapiens GN=CROT PE=1 SV=2 - [OCTC_HUMAN]	1.31	1	1	1	2	0.96	0.98	0.79	0.81					612	70.1	7.08

Q92968	Peroxisomal membrane protein PEX13 OS=Homo sapiens GN=PEX13 PE=1 SV=2 - [PEX13_HUMAN]	1.74	1	1	1	1					0.86	0.64	0.77	0.58	403	44.1	8.05
O43808	Peroxisomal membrane protein PMP34 OS=Homo sapiens GN=SLC25A17 PE=1 SV=1 - [PM34_HUMAN]	3.91	1	1	1	1	0.27	0.19	0.30	0.21					307	34.5	10.08
P51659	Peroxisomal multifunctional enzyme type 2 OS=Homo sapiens GN=HSD17B4 PE=1 SV=3 - [DHB4_HUMAN]	1.90	1	1	1	1	0.97	1.13	1.03	1.20					736	79.6	8.84
Q9BQG2	Peroxisomal NADH pyrophosphatase NUDT12 OS=Homo sapiens GN=NUDT12 PE=1 SV=1 - [NUD12_HUMAN]	3.46	1	1	1	1	0.55		0.77						462	52.0	6.83
Q9P0Z9	Peroxisomal sarcosine oxidase OS=Homo sapiens GN=PIPOX PE=2 SV=2 - [SOX_HUMAN]	3.08	1	1	1	4	1.10	0.96	1.28	1.12					390	44.0	8.41
O43933	Peroxisome biogenesis factor 1 OS=Homo sapiens GN=PEX1 PE=1 SV=1 - [PEX1_HUMAN]	3.12	1	2	2	3	0.88	1.02	0.90	1.05					1283	142.8	6.33
O60683	Peroxisome biogenesis factor 10 OS=Homo sapiens GN=PEX10 PE=1 SV=1 - [PEX10_HUMAN]	8.59	1	1	1	3	1.14	0.82	1.12	0.82					326	37.0	9.99
Q07869	Peroxisome proliferator-activated receptor alpha OS=Homo sapiens GN=PPARA PE=1 SV=2 - [PPARA_HUMAN]	4.06	1	1	1	1	1.01	1.11	1.20	1.32					468	52.2	6.25
Q5VV67	Peroxisome proliferator-activated receptor gamma coactivator-related protein 1 OS=Homo sapiens GN=PPRC1 PE=1 SV=1 - [PPRC1_HUMAN]	2.34	1	2	2	3	0.96	1.14	0.89	1.06	0.86	0.74	0.95	0.82	1664	177.4	6.51
P37231	Peroxisome proliferator-activated receptor gamma OS=Homo sapiens GN=PPARG PE=1 SV=3 - [PPARG_HUMAN]	3.76	1	1	1	1					0.83	1.22	0.96	1.40	505	57.6	5.94
O00541	Pescadillo homolog OS=Homo sapiens GN=PES1 PE=1 SV=1 - [PESC_HUMAN]	2.72	1	1	2	2	0.80	0.82	0.82	0.85					588	68.0	7.33
Q9BQI7	PH and SEC7 domain-containing protein 2 OS=Homo sapiens GN=PSD2 PE=2 SV=3 - [PSD2_HUMAN]	6.10	1	2	2	2	0.71	0.52			1.79	1.40	1.84	1.44	771	84.6	5.19
Q9NYI0	PH and SEC7 domain-containing protein 3 OS=Homo sapiens GN=PSD3 PE=1 SV=2 - [PSD3_HUMAN]	2.10	1	1	1	8					1.43	1.46	1.03	1.05	1048	116.0	5.99
Q8NDX1	PH and SEC7 domain-containing protein 4 OS=Homo sapiens GN=PSD4 PE=1 SV=2 - [PSD4_HUMAN]	2.75	1	1	1	1	1.22	0.78	1.29	0.83					1056	116.2	5.48
Q6ZVD8	PH domain leucine-rich repeat-containing protein phosphatase 2 OS=Homo sapiens GN=PHLPP2 PE=1 SV=3 - [PHLP2_HUMAN]	1.13	1	1	1	1	0.82	0.82	0.86	0.86					1323	146.7	5.71
Q8WWQ0	PH-interacting protein OS=Homo sapiens GN=PHIP PE=1 SV=2 - [PHIP_HUMAN]	1.37	1	1	1	1	0.74	0.62	0.85	0.72					1821	206.6	8.85
Q8WUB8	PHD finger protein 10 OS=Homo sapiens GN=PHF10 PE=1 SV=3 - [PHF10_HUMAN]	12.25	1	2	2	3					1.21	0.99	0.80	0.66	498	56.0	6.62
Q9BVI0	PHD finger protein 20 OS=Homo sapiens GN=PHF20 PE=1 SV=2 - [PHF20_HUMAN]	0.99	1	1	1	1					0.62	0.67	0.72	0.78	1012	115.3	6.99
A8MW92	PHD finger protein 20-like protein 1 OS=Homo sapiens GN=PHF20L1 PE=1 SV=2 - [P20L1_HUMAN]	4.62	1	2	2	2	1.68	1.45	2.01	1.76	0.73	0.79	0.52	0.56	1017	114.9	6.83
Q8IWS0	PHD finger protein 6 OS=Homo sapiens GN=PHF6 PE=1 SV=1 - [PHF6_HUMAN]	5.21	1	1	1	1						0.67		1.16	365	41.3	8.68
O95363	Phenylalanine--tRNA ligase, mitochondrial OS=Homo sapiens GN=FARS2 PE=1 SV=1 - [SYFM_HUMAN]	2.22	1	1	1	1	0.86	0.93	0.67	0.73					451	52.3	7.46
Q13794	Phorbol-12-myristate-13-acetate-induced protein 1 OS=Homo sapiens GN=PMAIP1 PE=1 SV=1 - [APR_HUMAN]	24.07	1	1	1	1					1.15	1.38	1.03	1.23	54	6.0	10.29
P78562	Phosphate-regulating neutral endopeptidase OS=Homo sapiens GN=PHEX PE=1 SV=1 - [PHEX_HUMAN]	1.47	1	1	1	1	1.48	1.31	1.24	1.10					749	86.4	8.76
Q92539	Phosphatidate phosphatase LPIN2 OS=Homo sapiens GN=LPIN2 PE=1 SV=1 - [LPIN2_HUMAN]	1.34	1	1	1	3	0.91	1.14	0.59	0.74					896	99.3	5.33
Q5VZY2	Phosphatidate phosphatase PPAPDC1A OS=Homo sapiens GN=PPAPDC1A PE=1 SV=2 - [PPC1A_HUMAN]	8.12	1	1	1	1							0.27	0.51	271	30.4	8.18
P04180	Phosphatidylcholine-sterol acyltransferase OS=Homo sapiens GN=LCAT PE=1 SV=1 - [LCAT_HUMAN]	8.41	1	3	3	45	1.04	1.07	1.03	1.06	0.87	0.79	0.88	0.82	440	49.5	6.11
Q86VZ5	Phosphatidylcholine:ceramide cholinephosphotransferase 1 OS=Homo sapiens GN=SGMS1 PE=1 SV=2 - [SMS1_HUMAN]	1.91	1	1	1	2					1.14	1.36	0.91	1.03	419	49.2	8.40

Q8NHU3	Phosphatidylcholine:ceramide cholinephosphotransferase 2 OS=Homo sapiens GN=SGMS2 PE=1 SV=1 - [SMS2_HUMAN]	2.47	1	1	1	1					0.85	0.83	0.92	0.89	365	42.3	8.81
P30086	Phosphatidylethanolamine-binding protein 1 OS=Homo sapiens GN=PEBP1 PE=1 SV=3 - [PEBP1_HUMAN]	18.18	1	3	3	5	0.93	0.86	0.89	0.83	0.95	0.87	0.89	0.82	187	21.0	7.53
Q96S96	Phosphatidylethanolamine-binding protein 4 OS=Homo sapiens GN=PEBP4 PE=1 SV=3 - [PEBP4_HUMAN]	7.93	1	2	2	7	0.90	0.94	0.86	0.91	0.93	0.97	0.87	0.88	227	25.7	6.54
O00459	Phosphatidylinositol 3-kinase regulatory subunit beta OS=Homo sapiens GN=PIK3R2 PE=1 SV=2 - [P85B_HUMAN]	0.96	1	1	1	1					0.83	0.95	0.74	0.86	728	81.5	6.43
Q6XPS3	Phosphatidylinositol 3,4,5-trisphosphate 3-phosphatase TPTE2 OS=Homo sapiens GN=TPTE2 PE=1 SV=2 - [TPTE2_HUMAN]	2.49	2	1	1	1					0.98	0.95	0.97	0.94	522	61.1	8.63
Q92835	Phosphatidylinositol 3,4,5-trisphosphate 5-phosphatase 1 OS=Homo sapiens GN=INPP5D PE=1 SV=2 - [SHIP1_HUMAN]	4.46	1	2	3	6	1.24	1.35	1.27	1.38	1.14	0.26	0.69	0.15	1189	133.2	7.59
Q8TCU6	Phosphatidylinositol 3,4,5-trisphosphate-dependent Rac exchanger 1 protein OS=Homo sapiens GN=PREX1 PE=1 SV=3 - [PREX1_HUMAN]	0.42	1	1	1	1	0.64	0.58	0.69	0.64					1659	186.1	6.44
P42356	Phosphatidylinositol 4-kinase alpha OS=Homo sapiens GN=PI4KA PE=1 SV=3 - [PI4KA_HUMAN]	0.64	1	1	1	1	1.69	1.75	1.95	2.04					2044	231.2	6.87
O00443	Phosphatidylinositol 4-phosphate 3-kinase C2 domain-containing subunit alpha OS=Homo sapiens GN=PIK3C2A PE=1 SV=2 - [P3C2A_HUMAN]	1.01	1	1	1	3	0.89	0.84	0.48	0.45	0.88	0.92	0.82	0.86	1686	190.6	8.02
O75747	Phosphatidylinositol 4-phosphate 3-kinase C2 domain-containing subunit gamma OS=Homo sapiens GN=PIK3C2G PE=1 SV=3 - [P3C2G_HUMAN]	0.62	1	1	1	2					1.07	0.50	0.88	0.41	1445	165.6	6.93
O14986	Phosphatidylinositol 4-phosphate 5-kinase type-1 beta OS=Homo sapiens GN=PIP5K1B PE=1 SV=2 - [PI51B_HUMAN]	3.33	4	1	2	5	3.00	2.21	0.98	0.72					540	61.0	6.86
P42338	Phosphatidylinositol 4,5-bisphosphate 3-kinase catalytic subunit beta isoform OS=Homo sapiens GN=PIK3CB PE=1 SV=1 - [PK3CB_HUMAN]	1.87	1	2	2	4	0.91	1.48	1.02	1.54					1070	122.7	7.09
Q15735	Phosphatidylinositol 4,5-bisphosphate 5-phosphatase A OS=Homo sapiens GN=INPP5J PE=1 SV=3 - [PI5PA_HUMAN]	3.78	1	1	1	1	0.85	1.26	0.93	1.38					1006	107.1	9.11
P78356	Phosphatidylinositol 5-phosphate 4-kinase type-2 beta OS=Homo sapiens GN=PIP4K2B PE=1 SV=1 - [PI42B_HUMAN]	2.64	1	1	1	2					0.60	0.57	0.82	0.78	416	47.3	7.33
Q8TBX8	Phosphatidylinositol 5-phosphate 4-kinase type-2 gamma OS=Homo sapiens GN=PIP4K2C PE=1 SV=3 - [PI42C_HUMAN]	6.41	1	1	1	3					1.89	1.45	1.47	1.13	421	47.3	6.84
Q13492	Phosphatidylinositol-binding clathrin assembly protein OS=Homo sapiens GN=PICALM PE=1 SV=2 - [PICAL_HUMAN]	5.06	1	1	1	1	0.98	1.40	1.26	1.81					652	70.7	7.90
P80108	Phosphatidylinositol-glycan-specific phospholipase D OS=Homo sapiens GN=GPLD1 PE=1 SV=3 - [PHLD_HUMAN]	26.31	1	18	18	134	1.21	1.25	1.27	1.26	1.28	1.37	1.19	1.27	840	92.3	6.37
P36871	Phosphoglucomutase-1 OS=Homo sapiens GN=PGM1 PE=1 SV=3 - [PGM1_HUMAN]	2.85	1	1	1	3	1.30	1.36	0.96	1.07					562	61.4	6.76
P00558	Phosphoglycerate kinase 1 OS=Homo sapiens GN=PGK1 PE=1 SV=3 - [PGK1_HUMAN]	43.17	2	12	12	27	0.89	0.77	0.93	0.83	0.83	0.95	0.94	1.07	417	44.6	8.10
P15259	Phosphoglycerate mutase 2 OS=Homo sapiens GN=PGAM2 PE=1 SV=3 - [PGAM2_HUMAN]	4.35	2	1	1	5	0.85	0.97	0.87	1.00	0.57	0.60	0.65	0.69	253	28.7	8.88
A6NDG6	Phosphoglycolate phosphatase OS=Homo sapiens GN=PGP PE=1 SV=1 - [PGP_HUMAN]	4.98	1	1	1	1	0.66	0.75	0.55	0.62					321	34.0	6.14
Q8WYR1	Phosphoinositide 3-kinase regulatory subunit 5 OS=Homo sapiens GN=PIK3R5 PE=1 SV=1 - [PI3R5_HUMAN]	1.48	1	1	1	1	0.93	0.99	0.89	0.95					880	97.3	6.74
Q96FE7	Phosphoinositide-3-kinase-interacting protein 1 OS=Homo sapiens GN=PIK3IP1 PE=1 SV=2 - [P3IP1_HUMAN]	2.66	1	1	1	1	0.85	0.95	0.99	1.13					263	28.2	5.01
Q9Y263	Phospholipase A-2-activating protein OS=Homo sapiens GN=PLAA PE=1 SV=2 - [PLAP_HUMAN]	5.16	1	2	2	2					0.72	0.63	0.75	0.66	795	87.1	6.37
Q53H76	Phospholipase A1 member A OS=Homo sapiens GN=PLA1A PE=2 SV=2 - [PLA1A_HUMAN]	4.82	1	1	1	4	1.01	1.01	0.99	0.99					456	49.7	7.37
Q6P1J6	Phospholipase B1, membrane-associated OS=Homo sapiens GN=PLB1 PE=1 SV=3 - [PLB1_HUMAN]	0.96	1	1	1	1					1.22	1.31	1.26	1.34	1458	163.0	5.88
Q13393	Phospholipase D1 OS=Homo sapiens GN=PLD1 PE=1 SV=1 - [PLD1_HUMAN]	0.74	1	1	1	6	0.76	0.49	0.64	0.42					1074	124.1	8.78

P55058	Phospholipid transfer protein OS=Homo sapiens GN=PLTP PE=1 SV=1 - [PLTP_HUMAN]	41.38	1	16	16	113	1.06	1.03	0.88	0.89	0.79	0.74	0.97	0.94	493	54.7	7.01
Q96CD2	Phosphopantothenoylcysteine decarboxylase OS=Homo sapiens GN=PPCDC PE=1 SV=2 - [COAC_HUMAN]	9.31	1	1	1	1					1.28	1.08	1.35	1.13	204	22.4	6.10
Q14558	Phosphoribosyl pyrophosphate synthase-associated protein 1 OS=Homo sapiens GN=PRPSAP1 PE=1 SV=2 - [KPRA_HUMAN]	3.93	1	1	1	2	0.96	0.93	0.85	0.83					356	39.4	7.20
O15067	Phosphoribosylformylglycinamidine synthase OS=Homo sapiens GN=PFAS PE=1 SV=4 - [PUR4_HUMAN]	3.29	2	2	3	4	1.16	1.19	1.11	1.16					1338	144.6	5.76
Q93100	Phosphorylase b kinase regulatory subunit beta OS=Homo sapiens GN=PHKB PE=1 SV=3 - [KPBB_HUMAN]	3.39	1	3	3	10	0.80	0.90	0.81	0.78					1093	124.8	6.95
Q9H4Z3	Phosphorylated CTD-interacting factor 1 OS=Homo sapiens GN=PCIF1 PE=1 SV=1 - [PCIF1_HUMAN]	2.13	1	2	2	4					0.76	0.82	1.05	1.05	704	80.6	7.42
Q9UMZ3	Phosphotidylinositol phosphatase PTPRQ OS=Homo sapiens GN=PTPRQ PE=1 SV=2 - [PTPRQ_HUMAN]	0.69	1	1	1	1	1.10	1.07	0.95	0.92					2332	260.8	5.81
Q9Y5X4	Photoreceptor-specific nuclear receptor OS=Homo sapiens GN=NR2E3 PE=1 SV=1 - [NR2E3_HUMAN]	4.39	1	1	1	1					0.77	0.81	0.66	0.69	410	44.7	7.90
Q0VAA5	PI-PLC X domain-containing protein 2 OS=Homo sapiens GN=PLCXD2 PE=1 SV=1 - [PLCX2_HUMAN]	4.26	1	1	1	1					0.30	1.26	0.60	2.53	305	34.8	9.44
Q63HM9	PI-PLC X domain-containing protein 3 OS=Homo sapiens GN=PLCXD3 PE=2 SV=2 - [PLCX3_HUMAN]	3.74	1	1	1	1					0.69	0.97	0.69	0.98	321	36.3	6.29
Q8N414	PiggyBac transposable element-derived protein 5 OS=Homo sapiens GN=PGBD5 PE=2 SV=3 - [PGBD5_HUMAN]	14.29	1	3	3	3	1.26	1.06	1.52	1.14	0.71	0.62	1.04	0.91	455	51.6	9.31
P36955	Pigment epithelium-derived factor OS=Homo sapiens GN=SERPINF1 PE=1 SV=4 - [PEDF_HUMAN]	54.07	1	21	21	209	0.94	0.91	1.02	0.96	0.84	0.89	0.84	0.89	418	46.3	6.38
Q9NWS0	PIH1 domain-containing protein 1 OS=Homo sapiens GN=PIH1D1 PE=1 SV=1 - [PIHD1_HUMAN]	6.21	1	1	1	2	1.05	1.05	1.01	1.02					290	32.3	5.14
Q8WWB5	PIH1 domain-containing protein 2 OS=Homo sapiens GN=PIH1D2 PE=2 SV=1 - [PIHD2_HUMAN]	3.17	1	1	1	3					0.29	0.97	0.38	1.89	315	35.9	6.37
P41586	Pituitary adenylate cyclase-activating polypeptide type I receptor OS=Homo sapiens GN=ADCYAP1R1 PE=1 SV=1 - [PACR_HUMAN]	2.35	1	1	1	1					0.82	0.89	0.87	0.95	468	53.3	5.78
Q96J94	Piwi-like protein 1 OS=Homo sapiens GN=PIWIL1 PE=1 SV=1 - [PIWL1_HUMAN]	3.25	1	2	2	3					0.74	0.76	0.65	0.66	861	98.5	9.44
Q7Z3Z4	Piwi-like protein 4 OS=Homo sapiens GN=PIWIL4 PE=2 SV=2 - [PIWL4_HUMAN]	2.35	1	1	1	1	0.93	1.01	1.11	1.22					852	96.5	8.88
Q6PK18	PKHD domain-containing transmembrane protein C17orf101 OS=Homo sapiens GN=C17orf101 PE=2 SV=2 - [CQ101_HUMAN]	4.08	1	1	1	1					0.76	0.28	0.95	0.35	319	35.6	8.18
A1L4L8	PLAC8-like protein 1 OS=Homo sapiens GN=PLAC8L1 PE=2 SV=1 - [PL8L1_HUMAN]	10.17	1	1	1	1							0.45	0.73	177	19.9	7.71
Q5JTB6	Placenta-specific protein 9 OS=Homo sapiens GN=PLAC9 PE=2 SV=1 - [PLAC9_HUMAN]	17.53	1	1	1	1					0.93	1.25	0.93	1.24	97	10.3	4.92
Q13835	Plakophilin-1 OS=Homo sapiens GN=PKP1 PE=1 SV=2 - [PKP1_HUMAN]	4.28	1	2	2	4	1.05	1.07	0.89	0.91					747	82.8	9.13
Q9Y446	Plakophilin-3 OS=Homo sapiens GN=PKP3 PE=1 SV=1 - [PKP3_HUMAN]	5.02	1	2	2	2	8.87	5.31	2.23	1.34	0.91	0.95	1.10	1.16	797	87.0	9.32
Q99569	Plakophilin-4 OS=Homo sapiens GN=PKP4 PE=1 SV=2 - [PKP4_HUMAN]	0.76	2	1	1	1					1.04	1.05	0.97	0.98	1192	131.8	8.94
Q9BTY2	Plasma alpha-L-fucosidase OS=Homo sapiens GN=FUCA2 PE=1 SV=2 - [FUCO2_HUMAN]	3.85	1	2	2	3	1.30	1.17	1.27	1.15					467	54.0	6.25
P03952	Plasma kallikrein OS=Homo sapiens GN=KLKB1 PE=1 SV=1 - [KLKB1_HUMAN]	65.36	2	41	41	536	1.43	1.43	1.35	1.36	1.03	1.01	1.04	1.04	638	71.3	8.22
P20020	Plasma membrane calcium-transporting ATPase 1 OS=Homo sapiens GN=ATP2B1 PE=1 SV=3 - [AT2B1_HUMAN]	5.33	3	2	3	5					1.00	1.04	0.78	0.80	1258	138.7	6.04
Q16720	Plasma membrane calcium-transporting ATPase 3 OS=Homo sapiens GN=ATP2B3 PE=1 SV=3 - [AT2B3_HUMAN]	2.46	1	1	2	3	0.88	1.07	0.92	1.11					1220	134.1	5.62

P05155	Plasma protease C1 inhibitor OS=Homo sapiens GN=SERPING1 PE=1 SV=2 - [IC1_HUMAN]	49.20	1	27	27	1009	0.95	0.94	0.94	0.93	0.85	0.88	0.82	0.88	500	55.1	6.55
P05154	Plasma serine protease inhibitor OS=Homo sapiens GN=SERPINA5 PE=1 SV=3 - [IPSP_HUMAN]	43.35	3	16	16	177	0.96	1.00	0.94	0.97	0.95	1.03	0.89	0.96	406	45.6	9.26
P05121	Plasminogen activator inhibitor 1 OS=Homo sapiens GN=SERPINE1 PE=1 SV=1 - [PAI1_HUMAN]	15.92	1	7	7	17	1.40	1.33	1.33	1.33	1.38	1.24	1.29	1.16	402	45.0	7.20
P05120	Plasminogen activator inhibitor 2 OS=Homo sapiens GN=SERPINB2 PE=1 SV=2 - [PAI2_HUMAN]	10.36	1	1	1	1	0.88	0.78	0.77	0.69					415	46.6	5.63
P00747	Plasminogen OS=Homo sapiens GN=PLG PE=1 SV=2 - [PLMN_HUMAN]	72.47	2	59	65	2192	1.11	1.09	1.11	1.10	0.89	0.91	0.90	0.92	810	90.5	7.24
Q02325	Plasminogen-related protein B OS=Homo sapiens GN=PLGLB1 PE=1 SV=1 - [PLGB_HUMAN]	36.46	1	1	6	74					1.28	1.30	1.33	1.35	96	11.0	6.52
P13796	Plastin-2 OS=Homo sapiens GN=LCP1 PE=1 SV=6 - [PLSL_HUMAN]	43.22	3	22	22	95	0.87	0.86	0.91	0.92	1.03	1.01	1.01	0.95	627	70.2	5.43
P02775	Platelet basic protein OS=Homo sapiens GN=PPBP PE=1 SV=3 - [CXCL7_HUMAN]	53.91	1	9	9	395	1.26	1.35	1.15	1.24	1.24	1.06	1.22	1.06	128	13.9	8.79
Q5VY43	Platelet endothelial aggregation receptor 1 OS=Homo sapiens GN=PEAR1 PE=1 SV=1 - [PEAR1_HUMAN]	6.17	1	4	4	8	1.01	1.07	1.00	0.96					1037	110.6	6.81
P16284	Platelet endothelial cell adhesion molecule OS=Homo sapiens GN=PECAM1 PE=1 SV=1 - [PECA1_HUMAN]	1.49	1	1	1	3					0.57	0.59	1.10	0.85	738	82.5	6.99
P02776	Platelet factor 4 OS=Homo sapiens GN=PF4 PE=1 SV=2 - [PLF4_HUMAN]	45.54	1	1	5	178					1.47	1.32	1.31	1.19	101	10.8	8.62
P10720	Platelet factor 4 variant OS=Homo sapiens GN=PF4V1 PE=1 SV=1 - [PF4V_HUMAN]	58.65	1	2	6	196					1.27	1.24	1.21	1.06	104	11.5	9.10
P07359	Platelet glycoprotein Ib alpha chain OS=Homo sapiens GN=GP1BA PE=1 SV=1 - [GP1BA_HUMAN]	14.06	1	9	9	39	1.05	1.00	1.04	1.01	1.13	1.09	1.18	1.14	626	68.9	6.68
P13224	Platelet glycoprotein Ib beta chain OS=Homo sapiens GN=GP1BB PE=1 SV=1 - [GP1BB_HUMAN]	14.56	1	3	3	8	0.73	0.74	0.74	0.75	0.52	0.93	0.94	1.70	206	21.7	9.31
P40197	Platelet glycoprotein V OS=Homo sapiens GN=GP5 PE=1 SV=1 - [GPV_HUMAN]	44.82	1	17	17	83	1.22	1.19	1.20	1.18	1.24	1.16	1.28	1.25	560	60.9	9.63
Q9HCN6	Platelet glycoprotein VI OS=Homo sapiens GN=GP6 PE=1 SV=4 - [GPVI_HUMAN]	3.24	1	1	1	1	1.05	1.27	0.95	1.16					339	36.8	9.20
Q99487	Platelet-activating factor acetylhydrolase 2, cytoplasmic OS=Homo sapiens GN=PAFAH2 PE=1 SV=1 - [PAFA2_HUMAN]	2.81	1	1	1	2					1.13	1.31	1.07	1.23	392	44.0	6.89
P43034	Platelet-activating factor acetylhydrolase IB subunit alpha OS=Homo sapiens GN=PAFAH1B1 PE=1 SV=2 - [LIS1_HUMAN]	4.88	1	1	1	1					1.28	1.44	1.40	1.58	410	46.6	7.37
Q13093	Platelet-activating factor acetylhydrolase OS=Homo sapiens GN=PLA2G7 PE=1 SV=1 - [PAFA_HUMAN]	18.37	1	6	6	10	1.15	1.21	1.15	1.28					441	50.0	7.56
Q9NRA1	Platelet-derived growth factor C OS=Homo sapiens GN=PDGFC PE=1 SV=2 - [PDGFC_HUMAN]	3.19	1	1	1	2					1.83	2.03	1.68	1.86	345	39.0	6.14
Q9GZP0	Platelet-derived growth factor D OS=Homo sapiens GN=PDGFD PE=1 SV=1 - [PDGFD_HUMAN]	3.78	1	1	1	1					0.67	0.17	0.71	0.18	370	42.8	7.97
P09619	Platelet-derived growth factor receptor beta OS=Homo sapiens GN=PDGFRB PE=1 SV=1 - [PGFRB_HUMAN]	0.99	1	1	1	3	1.37	1.33	1.37	1.33	0.65	0.82	1.04	1.32	1106	123.9	4.98
Q15198	Platelet-derived growth factor receptor-like protein OS=Homo sapiens GN=PDGFRL PE=1 SV=1 - [PGFRL_HUMAN]	11.47	1	2	2	2					0.88	0.72	0.88	0.71	375	41.8	8.50
P04085	Platelet-derived growth factor subunit A OS=Homo sapiens GN=PDGFA PE=1 SV=1 - [PDGFA_HUMAN]	13.74	1	2	2	5					1.87	1.82	1.89	1.84	211	24.0	9.39
P01127	Platelet-derived growth factor subunit B OS=Homo sapiens GN=PDGFB PE=1 SV=1 - [PDGFB_HUMAN]	2.49	1	1	1	1					1.76	1.72	1.83	1.79	241	27.3	9.16
Q9Y2H5	Pleckstrin homology domain-containing family A member 6 OS=Homo sapiens GN=PLEKHA6 PE=1 SV=4 - [PKHA6_HUMAN]	7.63	1	3	3	3	0.66		0.37		1.21	1.17	1.39	1.34	1048	117.1	9.10
Q6IQ23	Pleckstrin homology domain-containing family A member 7 OS=Homo sapiens GN=PLEKHA7 PE=1 SV=2 - [PKHA7_HUMAN]	3.93	1	3	3	6	0.96	0.96	0.86	0.86	0.99	0.96	0.94	0.91	1121	127.1	9.35

Q96JA3	Pleckstrin homology domain-containing family A member 8 OS=Homo sapiens GN=PLEKHA8 PE=1 SV=3 - [PKHA8_HUMAN]	7.71	2	2	3	3	0.88	0.44	0.84	0.43	0.64	1.37	0.74	1.57	519	58.2	5.11
Q9H7P9	Pleckstrin homology domain-containing family G member 2 OS=Homo sapiens GN=PLEKHG2 PE=1 SV=3 - [PKHG2_HUMAN]	2.74	1	2	2	2	1.46	1.33	1.32	1.22					1386	147.9	5.86
Q96PX9	Pleckstrin homology domain-containing family G member 4B OS=Homo sapiens GN=PLEKHG4B PE=2 SV=4 - [PKH4B_HUMAN]	2.83	1	2	2	3	0.67	0.65	0.63	0.61	0.54	0.73	0.70	0.95	1271	139.6	6.79
Q3KR16	Pleckstrin homology domain-containing family G member 6 OS=Homo sapiens GN=PLEKHG6 PE=1 SV=3 - [PKHG6_HUMAN]	7.22	1	3	3	3					0.70	0.77	0.70	0.79	790	88.9	7.09
Q9ULM0	Pleckstrin homology domain-containing family H member 1 OS=Homo sapiens GN=PLEKHH1 PE=2 SV=2 - [PKHH1_HUMAN]	1.10	1	1	1	1	0.90	1.04	0.88	1.02					1364	151.1	7.94
Q8IWE5	Pleckstrin homology domain-containing family M member 2 OS=Homo sapiens GN=PLEKHM2 PE=1 SV=2 - [PKHM2_HUMAN]	1.47	1	1	1	1					0.47	1.69	1.11	3.98	1019	112.7	4.93
Q6ZWE6	Pleckstrin homology domain-containing family M member 3 OS=Homo sapiens GN=PLEKHM3 PE=2 SV=2 - [PKHM3_HUMAN]	1.71	1	1	1	1	1.22	1.15	1.26	1.18					761	87.1	7.09
Q53GL0	Pleckstrin homology domain-containing family O member 1 OS=Homo sapiens GN=PLEKHO1 PE=1 SV=2 - [PKHO1_HUMAN]	4.40	1	1	1	2	1.11	1.07	1.25	1.22					409	46.2	8.82
Q8TD55	Pleckstrin homology domain-containing family O member 2 OS=Homo sapiens GN=PLEKHO2 PE=1 SV=1 - [PKHO2_HUMAN]	7.76	3	1	2	3	0.81		1.22						490	53.3	5.43
Q6NSJ2	Pleckstrin homology-like domain family B member 3 OS=Homo sapiens GN=PHLDB3 PE=2 SV=3 - [PHLB3_HUMAN]	8.28	1	2	2	5	1.39	1.44	1.43	1.49					640	71.9	6.57
P08567	Pleckstrin OS=Homo sapiens GN=PLEK PE=1 SV=3 - [PLEK_HUMAN]	22.57	1	6	6	31	0.89	0.80	1.00	0.92	0.85	0.92	0.91	1.01	350	40.1	8.28
Q15149	Plectin OS=Homo sapiens GN=PLEC PE=1 SV=3 - [PLEC_HUMAN]	1.17	5	4	5	9	1.85	1.81	1.65	1.61	2.31	1.84	2.30	1.82	4684	531.5	5.96
O43660	Pleiotropic regulator 1 OS=Homo sapiens GN=PLRG1 PE=1 SV=1 - [PLRG1_HUMAN]	7.98	1	2	2	4	0.67	0.63	0.86	0.82					514	57.2	9.17
Q8IUK5	Plexin domain-containing protein 1 OS=Homo sapiens GN=PLXDC1 PE=1 SV=2 - [PLDX1_HUMAN]	6.60	1	2	3	5	0.94	0.80	0.99	0.82					500	55.7	5.85
Q6UX71	Plexin domain-containing protein 2 OS=Homo sapiens GN=PLXDC2 PE=1 SV=1 - [PXDC2_HUMAN]	12.85	1	6	7	28	1.05	1.02	0.99	1.03	1.06	1.00	1.05	1.02	529	59.5	6.46
Q9UIW2	Plexin-A1 OS=Homo sapiens GN=PLXNA1 PE=1 SV=3 - [PLXA1_HUMAN]	1.05	1	1	1	1					2.16	1.67	2.07	1.60	1896	210.9	6.92
O75051	Plexin-A2 OS=Homo sapiens GN=PLXNA2 PE=1 SV=4 - [PLXA2_HUMAN]	1.95	1	2	2	2	0.92	1.07	0.90	1.05	1.15	1.28	1.08	1.20	1894	211.0	6.48
Q9HCM2	Plexin-A4 OS=Homo sapiens GN=PLXNA4 PE=1 SV=4 - [PLXA4_HUMAN]	1.00	1	1	1	1					0.21	1.26	0.47	2.81	1894	212.3	6.86
O43157	Plexin-B1 OS=Homo sapiens GN=PLXNB1 PE=1 SV=3 - [PLXB1_HUMAN]	1.59	1	3	3	4	0.93	0.94	0.89	0.90	1.08	0.87	1.14	0.92	2135	232.2	5.49
O15031	Plexin-B2 OS=Homo sapiens GN=PLXNB2 PE=1 SV=3 - [PLXB2_HUMAN]	2.77	1	4	4	5	1.59	1.26	0.95	0.77	0.97	1.00	1.46	1.28	1838	205.0	6.24
O60486	Plexin-C1 OS=Homo sapiens GN=PLXNC1 PE=1 SV=1 - [PLXC1_HUMAN]	3.25	4	2	3	4	0.84	0.68	0.84	0.69	2.25	3.19	1.95	2.76	1568	175.6	7.61
P54277	PMS1 protein homolog 1 OS=Homo sapiens GN=PMS1 PE=1 SV=1 - [PMS1_HUMAN]	1.72	1	1	1	1					1.00	1.27	0.92	1.18	932	105.8	6.67
Q86V59	PNMA-like protein 1 OS=Homo sapiens GN=PNMAL1 PE=2 SV=2 - [PNML1_HUMAN]	7.97	1	1	1	1					2.06	1.70	1.50	1.24	439	48.1	8.97
O00592	Podocalyxin OS=Homo sapiens GN=PODXL PE=1 SV=2 - [PODXL_HUMAN]	5.20	1	3	3	7	0.74	0.67	0.70	0.68	0.95	0.90	1.03	0.98	558	58.6	5.49
P15151	Poliovirus receptor OS=Homo sapiens GN=PVR PE=1 SV=2 - [PVR_HUMAN]	13.19	1	6	6	43	1.41	1.30	1.37	1.33	0.94	1.03	1.05	1.17	417	45.3	6.52
Q15223	Poliovirus receptor-related protein 1 OS=Homo sapiens GN=PVRL1 PE=1 SV=3 - [PVRL1_HUMAN]	2.71	1	1	1	5	1.23	1.17	1.22	1.18	0.61	0.52	0.75	0.63	517	57.1	6.10
Q92692	Poliovirus receptor-related protein 2 OS=Homo sapiens GN=PVRL2 PE=1 SV=1 - [PVRL2_HUMAN]	0.74	1	1	1	1							0.96	1.16	538	57.7	4.82

P09874	Poly [ADP-ribose] polymerase 1 OS=Homo sapiens GN=PARP1 PE=1 SV=4 - [PARP1_HUMAN]	3.75	1	3	3	3	1.06	0.99	1.09	1.06					1014	113.0	8.88
Q53GL7	Poly [ADP-ribose] polymerase 10 OS=Homo sapiens GN=PARP10 PE=1 SV=2 - [PAR10_HUMAN]	2.93	1	1	1	3					1.01	1.13	0.98	1.10	1025	109.9	4.97
Q9UKK3	Poly [ADP-ribose] polymerase 4 OS=Homo sapiens GN=PARP4 PE=1 SV=3 - [PARP4_HUMAN]	2.78	1	3	3	5	1.11	1.18	1.13	1.20					1724	192.5	5.66
Q8IXQ6	Poly [ADP-ribose] polymerase 9 OS=Homo sapiens GN=PARP9 PE=1 SV=2 - [PARP9_HUMAN]	2.34	1	1	1	4					1.19	1.51	1.04	1.32	854	96.3	7.91
Q9NVV4	Poly(A) RNA polymerase, mitochondrial OS=Homo sapiens GN=MTPAP PE=1 SV=1 - [PAPD1_HUMAN]	1.03	1	1	1	1					1.22	1.99	0.81	1.32	582	66.1	9.04
O95453	Poly(A)-specific ribonuclease PARN OS=Homo sapiens GN=PARN PE=1 SV=1 - [PARN_HUMAN]	1.25	1	1	1	2	1.02	0.22	1.03	0.22	1.02	2.05	1.17	2.35	639	73.4	6.20
Q9NX46	Poly(ADP-ribose) glycohydrolase ARH3 OS=Homo sapiens GN=ADPRHL2 PE=1 SV=1 - [ARHL2_HUMAN]	4.41	1	1	1	3	0.95	0.60	0.88	0.56					363	38.9	5.07
Q15365	Poly(rC)-binding protein 1 OS=Homo sapiens GN=PCBP1 PE=1 SV=2 - [PCBP1_HUMAN]	8.15	1	2	2	4	0.96	0.86	0.93	0.84	0.76	0.96	0.70	0.88	356	37.5	7.09
Q15366	Poly(rC)-binding protein 2 OS=Homo sapiens GN=PCBP2 PE=1 SV=1 - [PCBP2_HUMAN]	9.32	4	2	3	104	1.12	1.03	1.10	1.02	1.34	1.25	1.00	0.94	365	38.6	6.79
P57723	Poly(rC)-binding protein 4 OS=Homo sapiens GN=PCBP4 PE=2 SV=1 - [PCBP4_HUMAN]	3.47	1	1	1	2	0.77	0.76	0.79	0.78					403	41.5	8.16
P11940	Polyadenylate-binding protein 1 OS=Homo sapiens GN=PABPC1 PE=1 SV=2 - [PABP1_HUMAN]	7.39	1	1	3	4					0.40	0.07	0.83	0.15	636	70.6	9.50
Q9H361	Polyadenylate-binding protein 3 OS=Homo sapiens GN=PABPC3 PE=1 SV=2 - [PABP3_HUMAN]	6.50	1	1	3	4	0.71	0.56							631	70.0	9.67
Q8TBY8	Polyamine-modulated factor 1-binding protein 1 OS=Homo sapiens GN=PMFBP1 PE=2 SV=1 - [PMFBP_HUMAN]	1.47	1	1	1	1	1.13	0.77	2.13	1.46					1022	119.0	6.29
P35227	Polycomb group RING finger protein 2 OS=Homo sapiens GN=PCGF2 PE=1 SV=1 - [PCGF2_HUMAN]	3.78	1	2	2	3					1.31	1.26	1.07	1.03	344	37.8	7.96
Q96GD3	Polycomb protein SCMH1 OS=Homo sapiens GN=SCMH1 PE=1 SV=1 - [SCMH1_HUMAN]	3.03	1	1	1	1	1.06	0.77	1.04	0.76					660	73.3	9.25
Q9P0L9	Polycystic kidney disease 2-like 1 protein OS=Homo sapiens GN=PKD2L1 PE=1 SV=1 - [PK2L1_HUMAN]	1.74	1	1	1	1	0.85	1.05	0.98	1.23					805	91.9	5.48
Q9NZM6	Polycystic kidney disease 2-like 2 protein OS=Homo sapiens GN=PKD2L2 PE=2 SV=2 - [PK2L2_HUMAN]	1.28	1	1	1	1	1.00	0.82	0.79	0.65					624	73.7	8.88
Q8TDX9	Polycystic kidney disease protein 1-like 1 OS=Homo sapiens GN=PKD1L1 PE=2 SV=1 - [PK1L1_HUMAN]	0.56	1	1	1	1					0.95	0.99	0.95	0.98	2849	315.2	7.06
Q7Z442	Polycystic kidney disease protein 1-like 2 OS=Homo sapiens GN=PKD1L2 PE=1 SV=4 - [PK1L2_HUMAN]	1.46	4	2	3	4	0.69	0.67	0.78	0.77					2459	272.4	5.82
P98161	Polycystin-1 OS=Homo sapiens GN=PKD1 PE=1 SV=3 - [PKD1_HUMAN]	1.70	1	3	3	4	0.93	1.10	0.91	1.08					4303	462.2	6.73
Q13563	Polycystin-2 OS=Homo sapiens GN=PKD2 PE=1 SV=3 - [PKD2_HUMAN]	4.34	1	2	2	29	0.57	0.73	0.76	0.97	0.60	0.41	0.66	0.38	968	109.6	5.69
Q8NDX5	Polyhomeotic-like protein 3 OS=Homo sapiens GN=PHC3 PE=1 SV=1 - [PHC3_HUMAN]	1.42	1	1	1	2	0.83	0.77	1.02	0.95					983	106.1	6.62
Q9BY77	Polymerase delta-interacting protein 3 OS=Homo sapiens GN=POLDIP3 PE=1 SV=2 - [PDIP3_HUMAN]	3.33	1	1	1	1	1.11	0.94	1.11	0.95					421	46.1	9.99
P01833	Polymeric immunoglobulin receptor OS=Homo sapiens GN=PIGR PE=1 SV=4 - [PIGR_HUMAN]	26.05	1	18	18	88	1.51	1.50	1.47	1.52	1.70	1.33	1.29	0.96	764	83.2	5.74
Q5SY16	Polynucleotide 5'-hydroxyl-kinase NOL9 OS=Homo sapiens GN=NOL9 PE=1 SV=1 - [NOL9_HUMAN]	2.56	1	1	1	1	1.03	0.95	1.37	1.26					702	79.3	9.13
Q86SR1	Polypeptide N-acetylgalactosaminyltransferase 10 OS=Homo sapiens GN=GALNT10 PE=1 SV=2 - [GLT10_HUMAN]	3.65	1	1	1	2	2.05	1.92	1.50	1.41	1.15	1.18	1.31	1.35	603	68.9	8.59
Q8NCW6	Polypeptide N-acetylgalactosaminyltransferase 11 OS=Homo sapiens GN=GALNT11 PE=2 SV=2 - [GLT11_HUMAN]	5.76	1	2	2	2	0.96	1.27	1.13	1.49					608	68.9	8.19

Q8IUC8	Polypeptide N-acetylgalactosaminyltransferase 13 OS=Homo sapiens GN=GALNT13 PE=2 SV=2 - [GLT13_HUMAN]	5.22	1	2	2	4	1.17	1.30	0.88	0.99	1.26	1.18	1.26	1.23	556	64.0	6.83
Q10471	Polypeptide N-acetylgalactosaminyltransferase 2 OS=Homo sapiens GN=GALNT2 PE=1 SV=1 - [GALT2_HUMAN]	1.58	1	1	1	2	1.06	1.03	1.04	1.02					571	64.7	8.35
Q14435	Polypeptide N-acetylgalactosaminyltransferase 3 OS=Homo sapiens GN=GALNT3 PE=2 SV=2 - [GALT3_HUMAN]	3.63	1	1	1	1					0.95	1.00	0.84	0.88	633	72.6	7.99
Q7Z7M9	Polypeptide N-acetylgalactosaminyltransferase 5 OS=Homo sapiens GN=GALNT5 PE=1 SV=1 - [GALT5_HUMAN]	1.28	1	1	1	1					0.91	1.13	0.95	1.19	940	106.2	9.47
Q8TCS8	Polyribonucleotide nucleotidyltransferase 1, mitochondrial OS=Homo sapiens GN=PNPT1 PE=1 SV=2 - [PNPT1_HUMAN]	2.43	1	1	1	1	1.42	1.64	1.91	2.21					783	85.9	7.77
Q5K4E3	Polyserase-2 OS=Homo sapiens GN=PRSS36 PE=1 SV=2 - [POLS2_HUMAN]	2.11	1	1	1	1					1.04	0.84	1.13	0.91	855	91.9	5.66
P0CG47	Polyubiquitin-B OS=Homo sapiens GN=UBB PE=1 SV=1 - [UBB_HUMAN]	77.29	4	6	6	18	0.99	1.12	0.87	0.98	1.08	1.08	1.08	1.11	229	25.7	7.43
Q96KW2	POM121-like protein 2 OS=Homo sapiens GN=POM121L2 PE=1 SV=1 - [P12L2_HUMAN]	1.13	1	1	1	2	0.81	0.81	0.89	0.91					971	103.0	10.10
Q6ICG8	Postacrosomal sheath WW domain-binding protein OS=Homo sapiens GN=WBP2NL PE=2 SV=1 - [WBP2L_HUMAN]	11.33	1	1	1	3	0.89	0.81	0.94	0.73					309	31.9	7.84
Q8N5I3	Potassium channel regulatory protein OS=Homo sapiens GN=KCNRG PE=1 SV=1 - [KCNRG_HUMAN]	3.68	1	1	1	1	1.39	1.29	1.37	1.28					272	31.0	7.40
O95279	Potassium channel subfamily K member 5 OS=Homo sapiens GN=KCNK5 PE=1 SV=1 - [KCNK5_HUMAN]	1.40	1	1	1	1					1.34	1.22	0.76	0.69	499	55.1	6.74
Q5JUK3	Potassium channel subfamily T member 1 OS=Homo sapiens GN=KCNT1 PE=2 SV=2 - [KCNT1_HUMAN]	3.58	1	2	2	8					1.03	1.15	0.97	1.07	1230	138.3	7.52
A8MYU2	Potassium channel subfamily U member 1 OS=Homo sapiens GN=KCNU1 PE=2 SV=2 - [KCNU1_HUMAN]	1.39	1	1	1	1					1.29	1.14	0.80	0.70	1149	129.5	7.83
P22459	Potassium voltage-gated channel subfamily A member 4 OS=Homo sapiens GN=KCNA4 PE=1 SV=2 - [KCNA4_HUMAN]	0.92	1	1	1	1					0.49	0.72			653	73.2	5.17
Q14721	Potassium voltage-gated channel subfamily B member 1 OS=Homo sapiens GN=KCNB1 PE=1 SV=2 - [KCNB1_HUMAN]	1.28	1	1	1	3	0.84		1.23		1.04	1.37	1.11	1.46	858	95.8	8.07
Q96PR1	Potassium voltage-gated channel subfamily C member 2 OS=Homo sapiens GN=KCNC2 PE=1 SV=1 - [KCNC2_HUMAN]	1.41	1	1	1	6	0.90	1.45	1.03	1.82					638	70.2	7.96
Q9UJ90	Potassium voltage-gated channel subfamily E member 1-like protein OS=Homo sapiens GN=KCNE1L PE=2 SV=1 - [KCE1L_HUMAN]	11.97	1	1	1	2	####	###	####	####	####	###	###	####	142	15.0	6.30
Q9UIX4	Potassium voltage-gated channel subfamily G member 1 OS=Homo sapiens GN=KCNG1 PE=2 SV=1 - [KCNG1_HUMAN]	2.73	1	1	1	5	1.16	1.22	1.11	1.22					513	57.9	6.29
O95259	Potassium voltage-gated channel subfamily H member 1 OS=Homo sapiens GN=KCNH1 PE=1 SV=1 - [KCNH1_HUMAN]	0.61	1	1	1	2	0.03	0.05	0.16	0.22					989	111.4	7.58
Q9UQ05	Potassium voltage-gated channel subfamily H member 4 OS=Homo sapiens GN=KCNH4 PE=2 SV=1 - [KCNH4_HUMAN]	1.87	1	1	1	2	0.73	0.62	0.96	0.82					1017	111.6	8.54
Q9NS40	Potassium voltage-gated channel subfamily H member 7 OS=Homo sapiens GN=KCNH7 PE=1 SV=2 - [KCNH7_HUMAN]	1.25	1	1	1	1					0.82	0.80	0.69	0.67	1196	134.9	7.65
Q96L42	Potassium voltage-gated channel subfamily H member 8 OS=Homo sapiens GN=KCNH8 PE=2 SV=2 - [KCNH8_HUMAN]	3.52	1	1	1	1	0.88	1.16	0.93	1.22					1107	123.7	6.92
P20648	Potassium-transporting ATPase alpha chain 1 OS=Homo sapiens GN=ATP4A PE=2 SV=5 - [ATP4A_HUMAN]	2.80	1	2	2	2	0.97	0.92	0.91	0.88	1.03	1.06	0.95	0.97	1035	114.0	5.81
O60741	Potassium/sodium hyperpolarization-activated cyclic nucleotide-gated channel 1 OS=Homo sapiens GN=HCN1 PE=2 SV=3 - [HCN1_HUMAN]	5.39	2	1	3	5	1.22	0.71	1.71	0.99	1.74	2.37	1.25	1.70	890	98.7	8.40
Q9Y3Q4	Potassium/sodium hyperpolarization-activated cyclic nucleotide-gated channel 4 OS=Homo sapiens GN=HCN4 PE=1 SV=1 - [HCN4_HUMAN]	2.08	2	1	2	2					1.09	0.82	1.36	1.02	1203	129.0	8.91
A5A3E0	POTE ankyrin domain family member F OS=Homo sapiens GN=POTEF PE=1 SV=2 - [POTEF_HUMAN]	12.84	5	3	11	59	1.18	1.30	1.23	1.36	0.86	0.76	0.90	0.77	1075	121.4	6.20
Q9UKI9	POU domain, class 2, transcription factor 3 OS=Homo sapiens GN=POU2F3 PE=2 SV=3 - [PO2F3_HUMAN]	5.28	1	1	1	1	0.94	0.95	0.96	0.97					436	47.4	8.43

Q01851	POU domain, class 4, transcription factor 1 OS=Homo sapiens GN=POU4F1 PE=2 SV=4 - [PO4F1_HUMAN]	3.82	1	1	1	2	1.03	0.81	1.04	0.82					419	42.7	9.01
O75626	PR domain zinc finger protein 1 OS=Homo sapiens GN=PRDM1 PE=1 SV=2 - [PRDM1_HUMAN]	3.27	1	1	3	27					0.52	0.69	0.56	0.74	825	91.7	8.48
Q9NQV6	PR domain zinc finger protein 10 OS=Homo sapiens GN=PRDM10 PE=1 SV=2 - [PRD10_HUMAN]	1.22	1	1	1	1	0.97	1.12	0.99	1.16					1147	130.0	6.74
P57071	PR domain zinc finger protein 15 OS=Homo sapiens GN=PRDM15 PE=2 SV=4 - [PRD15_HUMAN]	2.39	1	2	2	3	0.76	0.81	0.80	0.86	0.97		0.69		1507	169.2	8.24
Q13029	PR domain zinc finger protein 2 OS=Homo sapiens GN=PRDM2 PE=1 SV=3 - [PRDM2_HUMAN]	0.64	1	1	1	1					1.83	1.27	1.37	0.95	1718	188.8	7.31
Q9NQV8	PR domain zinc finger protein 8 OS=Homo sapiens GN=PRDM8 PE=2 SV=3 - [PRDM8_HUMAN]	5.95	1	1	1	2	0.85	0.82	0.72	0.70					689	71.6	7.84
O60809	PRAME family member 10 OS=Homo sapiens GN=PRAMEF10 PE=1 SV=3 - [PRA10_HUMAN]	5.49	1	1	1	1	1.16		1.28						474	55.0	8.10
Q5SWL8	PRAME family member 19 OS=Homo sapiens GN=PRAMEF19 PE=2 SV=1 - [PRA19_HUMAN]	4.39	2	1	2	10	0.99	1.06	1.09	1.17					410	47.4	7.90
P40424	Pre-B-cell leukemia transcription factor 1 OS=Homo sapiens GN=PBX1 PE=1 SV=1 - [PBX1_HUMAN]	3.49	2	1	1	1					1.38	1.49	1.29	1.39	430	46.6	7.03
Q9C0J8	pre-mRNA 3' end processing protein WDR33 OS=Homo sapiens GN=WDR33 PE=1 SV=2 - [WDR33_HUMAN]	3.67	1	2	2	3					0.20	0.27	0.23	0.30	1336	145.8	9.17
O94913	Pre-mRNA cleavage complex 2 protein Pcf11 OS=Homo sapiens GN=PCF11 PE=1 SV=3 - [PCF11_HUMAN]	0.84	1	1	1	1	0.84	0.74	0.98	0.87					1555	172.9	8.48
Q9UMS4	Pre-mRNA-processing factor 19 OS=Homo sapiens GN=PRPF19 PE=1 SV=1 - [PRP19_HUMAN]	1.79	1	1	1	6	1.43	1.42	1.38	1.40					504	55.1	6.61
O75400	Pre-mRNA-processing factor 40 homolog A OS=Homo sapiens GN=PRPF40A PE=1 SV=2 - [PR40A_HUMAN]	1.78	1	1	1	2	0.81	0.88	0.72	0.80					957	108.7	7.56
O94906	Pre-mRNA-processing factor 6 OS=Homo sapiens GN=PRPF6 PE=1 SV=1 - [PRP6_HUMAN]	1.38	1	1	1	1	1.08	0.96	0.97	0.87					941	106.9	8.25
Q6P2Q9	Pre-mRNA-processing-splicing factor 8 OS=Homo sapiens GN=PRPF8 PE=1 SV=2 - [PRP8_HUMAN]	0.90	1	1	1	1	0.26	0.46	0.13	0.23					2335	273.4	8.84
Q99633	Pre-mRNA-splicing factor 18 OS=Homo sapiens GN=PRPF18 PE=1 SV=1 - [PRP18_HUMAN]	3.22	1	1	1	1	1.45	1.43	1.38	1.36					342	39.8	8.15
Q92620	Pre-mRNA-splicing factor ATP-dependent RNA helicase PRP16 OS=Homo sapiens GN=DHX38 PE=1 SV=2 - [PRP16_HUMAN]	1.30	1	1	1	1	1.72	1.23	1.38	0.99					1227	140.4	6.54
Q9ULR0	Pre-mRNA-splicing factor ISY1 homolog OS=Homo sapiens GN=ISY1 PE=1 SV=3 - [ISY1_HUMAN]	14.39	1	2	2	3					1.18	0.72	1.17	0.72	285	33.0	5.17
O95391	Pre-mRNA-splicing factor SLU7 OS=Homo sapiens GN=SLU7 PE=1 SV=2 - [SLU7_HUMAN]	2.22	1	1	1	1	0.83	0.87	0.92	0.97					586	68.3	7.14
O75934	Pre-mRNA-splicing factor SPF27 OS=Homo sapiens GN=BCAS2 PE=1 SV=1 - [SPF27_HUMAN]	19.56	1	3	3	3					1.08	1.11	0.96	0.99	225	26.1	5.66
P20742	Pregnancy zone protein OS=Homo sapiens GN=PZP PE=1 SV=4 - [PZP_HUMAN]	45.07	2	49	64	4032	1.72	1.67	1.69	1.70	1.76	1.84	1.57	1.72	1482	163.8	6.38
P11464	Pregnancy-specific beta-1-glycoprotein 1 OS=Homo sapiens GN=PSG1 PE=2 SV=1 - [PSG1_HUMAN]	34.61	1	4	10	131	4.65	6.41	4.65	5.82	6.56	7.05	6.48	6.95	419	47.2	8.12
Q9UQ72	Pregnancy-specific beta-1-glycoprotein 11 OS=Homo sapiens GN=PSG11 PE=2 SV=3 - [PSG11_HUMAN]	20.30	1	2	6	60	6.41	8.90	6.29	8.86	6.96	6.89	6.77	6.69	335	37.1	7.14
P11465	Pregnancy-specific beta-1-glycoprotein 2 OS=Homo sapiens GN=PSG2 PE=2 SV=2 - [PSG2_HUMAN]	23.28	1	2	6	72	0.95	1.00	1.07	1.14	####	###	###	####	335	37.2	7.80
Q16557	Pregnancy-specific beta-1-glycoprotein 3 OS=Homo sapiens GN=PSG3 PE=2 SV=2 - [PSG3_HUMAN]	22.90	1	2	10	136	4.38	5.29	5.07	6.19	1.22	0.92	0.94	0.71	428	47.9	8.54
Q00888	Pregnancy-specific beta-1-glycoprotein 4 OS=Homo sapiens GN=PSG4 PE=2 SV=3 - [PSG4_HUMAN]	24.34	1	3	8	102	2.48	3.58	2.32	3.15	5.22	4.36	5.01	4.18	419	47.1	9.26
Q15238	Pregnancy-specific beta-1-glycoprotein 5 OS=Homo sapiens GN=PSG5 PE=1 SV=3 - [PSG5_HUMAN]	23.58	1	3	9	69	3.44	4.41	3.46	4.28	3.25	3.21	3.15	3.11	335	37.7	8.87

Q00887	Pregnancy-specific beta-1-glycoprotein 9 OS=Homo sapiens GN=PSG9 PE=2 SV=2 - [PSG9_HUMAN]	15.02	1	2	7	68	2.36	2.78	2.35	2.80	3.65	3.43	3.20	3.01	426	48.2	8.07
Q9UHG3	Prenylcysteine oxidase 1 OS=Homo sapiens GN=PCYOX1 PE=1 SV=3 - [PCYOX_HUMAN]	25.74	1	11	11	39	1.12	1.28	1.01	1.18	1.21	1.31	1.12	1.21	505	56.6	6.18
Q8NBM8	Prenylcysteine oxidase-like OS=Homo sapiens GN=PCYOX1L PE=1 SV=2 - [PCYXL_HUMAN]	5.67	1	1	1	1	0.96	0.77	1.36	1.11					494	54.6	7.31
Q13519	Prepronociceptin OS=Homo sapiens GN=PNOC PE=1 SV=1 - [PNOC_HUMAN]	5.68	1	1	1	1					1.39	2.03	1.46	2.12	176	20.3	8.40
P49768	Presenilin-1 OS=Homo sapiens GN=PSEN1 PE=1 SV=1 - [PSN1_HUMAN]	3.85	1	1	1	1					1.20	1.54	1.39	1.78	467	52.6	5.31
Q96MT3	Prickle-like protein 1 OS=Homo sapiens GN=PRICKLE1 PE=1 SV=2 - [PRIC1_HUMAN]	2.41	1	1	1	1	1.24	1.70	1.15	1.57					831	94.2	6.24
Q9UKY0	Prion-like protein doppel OS=Homo sapiens GN=PRND PE=1 SV=2 - [PRND_HUMAN]	13.64	1	1	1	1					1.22	1.28	1.14	1.19	176	20.3	9.03
Q9NRD5	PRKCA-binding protein OS=Homo sapiens GN=PICK1 PE=1 SV=2 - [PICK1_HUMAN]	1.69	1	1	1	1							0.77	0.78	415	46.6	5.30
Q9H875	PRKR-interacting protein 1 OS=Homo sapiens GN=PRKRIP1 PE=1 SV=1 - [PKRI1_HUMAN]	14.13	1	1	1	1	0.38	0.30	0.61	0.48					184	21.0	9.79
Q14005	Pro-interleukin-16 OS=Homo sapiens GN=IL16 PE=1 SV=4 - [IL16_HUMAN]	0.83	1	1	1	1		0.38		0.56					1332	141.7	8.06
Q02297	Pro-neuregulin-1, membrane-bound isoform OS=Homo sapiens GN=NRG1 PE=1 SV=3 - [NRG1_HUMAN]	2.03	1	1	1	4					0.36	0.33	0.77	0.72	640	70.3	8.79
P07602	Proactivator polypeptide OS=Homo sapiens GN=PSAP PE=1 SV=2 - [SAP_HUMAN]	13.55	1	6	6	10	1.07	0.89	1.11	0.95	1.57	1.03	1.45	1.10	524	58.1	5.17
Q96HY7	Probable 2-oxoglutarate dehydrogenase E1 component DHKTD1, mitochondrial OS=Homo sapiens GN=DHTKD1 PE=2 SV=2 - [DHTK1_HUMAN]	0.87	1	1	1	1					1.16	1.34	1.19	1.38	919	103.0	6.93
Q8N6M5	Probable allantoicase OS=Homo sapiens GN=ALLC PE=2 SV=3 - [ALLC_HUMAN]	4.15	1	1	1	1	1.28	1.18	1.14	1.05					410	45.7	6.18
Q8NDH3	Probable aminopeptidase NPEPL1 OS=Homo sapiens GN=NPEPL1 PE=1 SV=3 - [PEPL1_HUMAN]	3.82	1	1	1	2					1.44	1.46	1.15	1.17	523	55.8	6.87
Q96I59	Probable asparagine--tRNA ligase, mitochondrial OS=Homo sapiens GN=NARS2 PE=1 SV=3 - [SYNM_HUMAN]	2.94	1	1	1	1	1.17	0.95	1.08	0.88					477	54.1	7.24
Q13206	Probable ATP-dependent RNA helicase DDX10 OS=Homo sapiens GN=DDX10 PE=1 SV=2 - [DDX10_HUMAN]	1.03	1	1	1	2					0.48	3.77	0.38	2.99	875	100.8	8.63
Q9UHI6	Probable ATP-dependent RNA helicase DDX20 OS=Homo sapiens GN=DDX20 PE=1 SV=2 - [DDX20_HUMAN]	2.18	1	1	1	1					0.73	0.61	1.11	0.92	824	92.2	6.95
Q9NUL7	Probable ATP-dependent RNA helicase DDX28 OS=Homo sapiens GN=DDX28 PE=2 SV=2 - [DDX28_HUMAN]	4.26	1	2	2	2	1.11	0.86	1.16	0.90	0.99	0.57	0.76	0.44	540	59.5	10.42
Q9H8H2	Probable ATP-dependent RNA helicase DDX31 OS=Homo sapiens GN=DDX31 PE=1 SV=2 - [DDX31_HUMAN]	1.76	1	1	1	1	1.57	1.24	1.70	1.34					851	94.0	9.99
Q9NXZ2	Probable ATP-dependent RNA helicase DDX43 OS=Homo sapiens GN=DDX43 PE=1 SV=2 - [DDX43_HUMAN]	5.56	1	2	2	2					0.73	0.78	0.69	0.74	648	72.8	8.75
Q7L014	Probable ATP-dependent RNA helicase DDX46 OS=Homo sapiens GN=DDX46 PE=1 SV=2 - [DDX46_HUMAN]	0.97	1	1	1	2					1.04	0.97	0.90	0.84	1031	117.3	9.29
Q9Y6V7	Probable ATP-dependent RNA helicase DDX49 OS=Homo sapiens GN=DDX49 PE=1 SV=1 - [DDX49_HUMAN]	3.31	1	1	1	5					0.71	0.15	0.75	0.16	483	54.2	9.06
Q86TM3	Probable ATP-dependent RNA helicase DDX53 OS=Homo sapiens GN=DDX53 PE=1 SV=3 - [DDX53_HUMAN]	5.86	1	2	2	3	0.84	1.07	0.77	0.98					631	71.1	9.07
Q8IY21	Probable ATP-dependent RNA helicase DDX60 OS=Homo sapiens GN=DDX60 PE=2 SV=3 - [DDX60_HUMAN]	3.62	1	4	4	7	0.95	0.95	0.96	0.96	1.11	1.07	1.60	1.55	1712	197.7	7.59
Q9H2U1	Probable ATP-dependent RNA helicase DHX36 OS=Homo sapiens GN=DHX36 PE=1 SV=2 - [DHX36_HUMAN]	2.38	1	1	2	2	0.85	0.89	0.91	0.95					1008	114.7	7.68
Q8IY37	Probable ATP-dependent RNA helicase DHX37 OS=Homo sapiens GN=DHX37 PE=1 SV=1 - [DHX37_HUMAN]	2.51	1	2	2	7	0.77	0.97	0.74	0.82	1.00		0.94		1157	129.5	8.10

Q9H6S0	Probable ATP-dependent RNA helicase YTHDC2 OS=Homo sapiens GN=YTHDC2 PE=1 SV=2 - [YTDC2_HUMAN]	1.47	2	1	2	14	1.10	1.00	1.09	1.00	0.47	0.53	0.45	0.50	1430	160.1	8.40
Q9NQ11	Probable cation-transporting ATPase 13A2 OS=Homo sapiens GN=ATP13A2 PE=1 SV=2 - [AT132_HUMAN]	2.63	1	2	2	2	0.90	0.78	1.38	1.21					1180	128.7	8.12
Q9H7F0	Probable cation-transporting ATPase 13A3 OS=Homo sapiens GN=ATP13A3 PE=1 SV=4 - [AT133_HUMAN]	2.20	1	2	2	2	1.06	1.23	0.93	1.08	0.89	0.75	0.94	0.78	1226	138.0	6.64
Q4VNC1	Probable cation-transporting ATPase 13A4 OS=Homo sapiens GN=ATP13A4 PE=2 SV=3 - [AT134_HUMAN]	2.01	1	1	1	4	0.82		1.25						1196	133.9	6.70
Q4VNC0	Probable cation-transporting ATPase 13A5 OS=Homo sapiens GN=ATP13A5 PE=2 SV=1 - [AT135_HUMAN]	1.64	1	1	1	5	0.84	1.27	0.85	1.28	1.06		1.18		1218	137.2	7.90
Q15751	Probable E3 ubiquitin-protein ligase HERC1 OS=Homo sapiens GN=HERC1 PE=1 SV=2 - [HERC1_HUMAN]	0.37	1	1	2	48	0.98	0.95	0.95	0.93					4861	531.9	6.04
Q5GLZ8	Probable E3 ubiquitin-protein ligase HERC4 OS=Homo sapiens GN=HERC4 PE=1 SV=1 - [HERC4_HUMAN]	1.32	1	1	1	2	1.07	1.20	0.71	0.80					1057	118.5	6.19
Q8IVU3	Probable E3 ubiquitin-protein ligase HERC6 OS=Homo sapiens GN=HERC6 PE=2 SV=2 - [HERC6_HUMAN]	1.17	1	1	1	1					0.80	0.80	0.76	0.76	1022	115.1	7.96
Q8NA82	Probable E3 ubiquitin-protein ligase MARCH10 OS=Homo sapiens GN=MARCH10 PE=1 SV=3 - [MARHA_HUMAN]	1.49	1	1	1	1					0.94	0.95	0.88	0.88	808	90.5	6.71
O75592	Probable E3 ubiquitin-protein ligase MYCBP2 OS=Homo sapiens GN=MYCBP2 PE=1 SV=3 - [MYCB2_HUMAN]	1.85	1	3	3	5	0.89	0.98	0.87	0.96	0.74	0.80	0.77	0.83	4640	509.8	7.03
P50876	Probable E3 ubiquitin-protein ligase RNF144A OS=Homo sapiens GN=RNF144A PE=1 SV=2 - [R144A_HUMAN]	3.77	1	1	1	1					0.78	0.95	1.03	1.26	292	32.9	6.28
Q9NX53	Probable exonuclease mut-7 homolog, isoform 5 OS=Homo sapiens GN=EXD3 PE=1 SV=2 - [MUT7B_HUMAN]	7.54	1	1	1	1	1.12	1.05	0.97	0.91					252	26.3	9.47
Q5T601	Probable G-protein coupled receptor 110 OS=Homo sapiens GN=GPR110 PE=2 SV=2 - [GP110_HUMAN]	3.30	1	2	2	2	0.73	0.72	0.71	0.71		0.55			910	101.3	8.62
Q8IZF7	Probable G-protein coupled receptor 111 OS=Homo sapiens GN=GPR111 PE=2 SV=1 - [GP111_HUMAN]	2.12	1	1	1	1	1.02	0.95	0.92	0.86					708	78.5	8.10
Q8IZF3	Probable G-protein coupled receptor 115 OS=Homo sapiens GN=GPR115 PE=2 SV=3 - [GP115_HUMAN]	2.01	1	1	1	1					0.58	1.01	0.62	1.06	695	77.7	9.01
Q8IZF2	Probable G-protein coupled receptor 116 OS=Homo sapiens GN=GPR116 PE=1 SV=3 - [GP116_HUMAN]	4.16	1	4	4	9	1.02	0.95	1.02	0.93	1.01	1.18	1.01	1.18	1346	149.4	6.65
Q8IWK6	Probable G-protein coupled receptor 125 OS=Homo sapiens GN=GPR125 PE=1 SV=2 - [GP125_HUMAN]	1.29	1	2	2	2	1.52	2.46	0.61	0.99	0.93	1.05	1.10	1.25	1321	146.1	8.48
Q6QNK2	Probable G-protein coupled receptor 133 OS=Homo sapiens GN=GPR133 PE=2 SV=1 - [GP133_HUMAN]	1.37	1	1	1	1	0.99	0.95	1.04	1.01					874	96.5	7.88
Q7Z7M1	Probable G-protein coupled receptor 144 OS=Homo sapiens GN=GPR144 PE=2 SV=1 - [GP144_HUMAN]	3.32	1	1	1	3					0.88	0.93	0.67	0.85	963	104.0	8.02
Q8NFN8	Probable G-protein coupled receptor 156 OS=Homo sapiens GN=GPR156 PE=2 SV=2 - [GP156_HUMAN]	1.97	1	1	1	1	1.85	3.03	1.47	2.40					814	89.0	7.64
Q5T848	Probable G-protein coupled receptor 158 OS=Homo sapiens GN=GPR158 PE=1 SV=1 - [GP158_HUMAN]	0.82	1	1	1	2					1.42	1.19	1.07	0.90	1215	135.4	8.28
Q9UJ42	Probable G-protein coupled receptor 160 OS=Homo sapiens GN=GPR160 PE=2 SV=1 - [GP160_HUMAN]	4.73	2	2	2	5	0.94	0.99	0.98	1.03	2.03	2.68	1.83	2.41	338	39.8	8.51
Q9NS66	Probable G-protein coupled receptor 173 OS=Homo sapiens GN=GPR173 PE=2 SV=1 - [GP173_HUMAN]	1.88	1	1	1	7	0.97	1.08	0.99	1.11					373	41.5	9.16
Q14439	Probable G-protein coupled receptor 176 OS=Homo sapiens GN=GPR176 PE=2 SV=1 - [GP176_HUMAN]	7.96	1	1	1	1					0.99	1.20	0.58	0.71	515	57.0	8.51
Q6PRD1	Probable G-protein coupled receptor 179 OS=Homo sapiens GN=GPR179 PE=2 SV=2 - [GP179_HUMAN]	0.46	1	1	1	2	0.73	0.94	0.70	0.92					2367	257.2	5.71
Q15760	Probable G-protein coupled receptor 19 OS=Homo sapiens GN=GPR19 PE=2 SV=2 - [GPR19_HUMAN]	3.37	1	1	1	1					0.64	0.09	1.03	0.15	415	47.7	9.55
Q99679	Probable G-protein coupled receptor 21 OS=Homo sapiens GN=GPR21 PE=2 SV=1 - [GPR21_HUMAN]	7.16	1	1	1	1					0.72				349	39.5	7.71

Q9UPC5	Probable G-protein coupled receptor 34 OS=Homo sapiens GN=GPR34 PE=1 SV=2 - [GPR34_HUMAN]	2.10	1	1	1	3					2.25	1.80	1.82	1.75	381	43.8	9.86
O15354	Probable G-protein coupled receptor 37 OS=Homo sapiens GN=GPR37 PE=1 SV=2 - [GPR37_HUMAN]	1.96	1	1	1	1					0.88	1.02	0.96	1.10	613	67.1	8.27
Q9Y5Y3	Probable G-protein coupled receptor 45 OS=Homo sapiens GN=GPR45 PE=2 SV=2 - [GPR45_HUMAN]	4.30	1	1	1	1					0.54	0.18	0.78	0.26	372	41.9	9.25
Q9BZJ6	Probable G-protein coupled receptor 63 OS=Homo sapiens GN=GPR63 PE=2 SV=2 - [GPR63_HUMAN]	3.58	1	1	1	1	1.28	0.83	1.60	1.04					419	47.5	9.52
P51531	Probable global transcription activator SNF2L2 OS=Homo sapiens GN=SMARCA2 PE=1 SV=2 - [SMCA2_HUMAN]	1.76	1	2	2	2	1.07	1.03	1.22	1.17	0.93	0.86	0.94	0.87	1590	181.2	7.20
Q86YR7	Probable guanine nucleotide exchange factor MCF2L2 OS=Homo sapiens GN=MCF2L2 PE=2 SV=3 - [MF2L2_HUMAN]	1.80	1	1	1	2	1.23	1.78	1.14	1.65					1114	126.9	6.42
P42694	Probable helicase with zinc finger domain OS=Homo sapiens GN=HELZ PE=1 SV=2 - [HELZ_HUMAN]	1.03	1	1	1	55	0.77	0.94	0.81	0.93	1.04	1.11	1.01	1.01	1942	218.8	7.42
Q15652	Probable JmjC domain-containing histone demethylation protein 2C OS=Homo sapiens GN=JMJD1C PE=1 SV=2 - [JHD2C_HUMAN]	1.42	1	3	3	4	0.55	0.54	0.62	0.65					2540	284.3	7.87
Q5TEU4	Probable methyltransferase C20orf7, mitochondrial OS=Homo sapiens GN=C20orf7 PE=1 SV=1 - [CT007_HUMAN]	5.80	1	2	2	2	1.14	1.19	1.09	1.15	1.02	0.89	1.12	0.98	345	38.9	6.57
Q13395	Probable methyltransferase TARBP1 OS=Homo sapiens GN=TARBP1 PE=1 SV=1 - [TARB1_HUMAN]	1.91	1	1	1	1	0.89	1.20	0.64	0.86					1621	181.6	7.05
Q969W1	Probable palmitoyltransferase ZDHHC16 OS=Homo sapiens GN=ZDHHC16 PE=2 SV=1 - [ZDH16_HUMAN]	2.12	1	1	1	1					0.56	0.67	0.69	0.82	377	43.6	9.63
Q9ULC8	Probable palmitoyltransferase ZDHHC8 OS=Homo sapiens GN=ZDHHC8 PE=1 SV=3 - [ZDHC8_HUMAN]	4.44	1	1	1	1	1.09	1.19	1.42	1.55					765	81.4	9.17
Q86Y79	Probable peptidyl-tRNA hydrolase OS=Homo sapiens GN=PTRH1 PE=1 SV=1 - [PTH_HUMAN]	3.74	1	1	1	1	0.90	0.89	0.98	0.97					214	22.9	10.56
Q9NTI2	Probable phospholipid-transporting ATPase IB OS=Homo sapiens GN=ATP8A2 PE=2 SV=2 - [AT8A2_HUMAN]	1.05	1	1	1	1	1.11	1.10	1.02	1.02					1148	129.2	7.78
Q9Y2G3	Probable phospholipid-transporting ATPase IF OS=Homo sapiens GN=ATP11B PE=1 SV=2 - [AT11B_HUMAN]	1.44	1	1	1	1	0.73	1.01	0.67	0.92					1177	134.1	6.96
Q8NB49	Probable phospholipid-transporting ATPase IG OS=Homo sapiens GN=ATP11C PE=1 SV=3 - [AT11C_HUMAN]	1.15	1	1	1	14	0.85	0.83	0.87	0.86	0.94	0.94	0.88	0.92	1132	129.4	6.67
O43861	Probable phospholipid-transporting ATPase IIB OS=Homo sapiens GN=ATP9B PE=2 SV=4 - [ATP9B_HUMAN]	5.06	1	4	4	7		0.83		0.58	1.94	3.58	2.92	2.70	1147	129.2	7.61
O60423	Probable phospholipid-transporting ATPase IK OS=Homo sapiens GN=ATP8B3 PE=2 SV=4 - [AT8B3_HUMAN]	1.92	1	2	2	2	1.00		0.92		1.02	1.07	0.89	0.93	1300	146.7	7.90
Q8TF62	Probable phospholipid-transporting ATPase IM OS=Homo sapiens GN=ATP8B4 PE=2 SV=3 - [AT8B4_HUMAN]	1.51	1	1	1	27	0.96	1.17	1.10	1.10					1192	135.8	6.99
O60312	Probable phospholipid-transporting ATPase VA OS=Homo sapiens GN=ATP10A PE=2 SV=2 - [AT10A_HUMAN]	0.47	1	1	1	1	0.86	0.94	0.78	0.87					1499	167.6	8.37
O94823	Probable phospholipid-transporting ATPase VB OS=Homo sapiens GN=ATP10B PE=2 SV=2 - [AT10B_HUMAN]	0.89	1	1	1	1					0.79	0.80	0.85	0.86	1461	165.3	6.89
Q9P241	Probable phospholipid-transporting ATPase VD OS=Homo sapiens GN=ATP10D PE=2 SV=3 - [AT10D_HUMAN]	4.14	1	2	2	2	1.43	1.14	1.46	1.17					1426	160.2	7.15
Q9NY28	Probable polypeptide N-acetylgalactosaminyltransferase 8 OS=Homo sapiens GN=GALNT8 PE=2 SV=1 - [GALT8_HUMAN]	5.34	1	1	1	1					0.76	0.54	0.66	0.47	637	72.8	8.87
Q7L3T8	Probable proline--tRNA ligase, mitochondrial OS=Homo sapiens GN=PARS2 PE=1 SV=1 - [SYPM_HUMAN]	5.47	1	2	2	2					1.22	1.35	1.15	1.29	475	53.2	8.10
Q5HYM0	Probable ribonuclease ZC3H12B OS=Homo sapiens GN=ZC3H12B PE=2 SV=2 - [ZC12B_HUMAN]	1.94	1	1	1	1					0.19	0.26	0.66	0.92	825	93.0	7.80
A2A288	Probable ribonuclease ZC3H12D OS=Homo sapiens GN=ZC3H12D PE=2 SV=3 - [ZC12D_HUMAN]	2.09	1	1	1	1					0.69	0.58	0.69	0.58	527	58.0	8.68
Q96H35	Probable RNA-binding protein 18 OS=Homo sapiens GN=RBM18 PE=2 SV=1 - [RBM18_HUMAN]	10.53	1	1	1	1	1.10	1.00	1.11	1.02					190	21.6	9.44

Q9Y4C8	Probable RNA-binding protein 19 OS=Homo sapiens GN=RBM19 PE=1 SV=3 - [RBM19_HUMAN]	2.50	1	2	2	2	0.85	1.07	0.94	1.24					960	107.3	6.54
Q8IYL2	Probable tRNA (uracil-O(2)-)-methyltransferase OS=Homo sapiens GN=METTL19 PE=2 SV=2 - [TRM44_HUMAN]	1.72	1	1	1	1					0.99	1.52	0.75	1.16	757	84.6	7.27
Q10571	Probable tumor suppressor protein MN1 OS=Homo sapiens GN=MN1 PE=1 SV=3 - [MN1_HUMAN]	1.36	1	1	1	1					0.99	0.89	1.01	0.92	1320	135.9	6.71
Q15113	Procollagen C-endopeptidase enhancer 1 OS=Homo sapiens GN=PCOLCE PE=1 SV=2 - [PCOC1_HUMAN]	18.26	1	5	5	21	1.00	1.04	1.01	1.03	0.71	0.81	0.72	0.80	449	47.9	7.43
Q9UKZ9	Procollagen C-endopeptidase enhancer 2 OS=Homo sapiens GN=PCOLCE2 PE=1 SV=1 - [PCOC2_HUMAN]	2.89	1	1	1	1	0.58	0.51	0.66	0.58					415	45.7	8.47
Q8NBJ5	Procollagen galactosyltransferase 1 OS=Homo sapiens GN=GLT25D1 PE=1 SV=1 - [GT251_HUMAN]	4.82	1	1	1	3	0.51	0.57	0.91	1.01					622	71.6	7.31
Q8IYK4	Procollagen galactosyltransferase 2 OS=Homo sapiens GN=GLT25D2 PE=1 SV=1 - [GT252_HUMAN]	6.39	1	2	2	3	3.32	4.16	3.49	4.35					626	72.9	6.20
Q02809	Procollagen-lysine,2-oxoglutarate 5-dioxygenase 1 OS=Homo sapiens GN=PLOD1 PE=1 SV=2 - [PLOD1_HUMAN]	3.30	1	2	2	5	1.10	1.11	1.14	1.16	0.62	0.14	0.66	0.14	727	83.5	6.95
O00469	Procollagen-lysine,2-oxoglutarate 5-dioxygenase 2 OS=Homo sapiens GN=PLOD2 PE=1 SV=2 - [PLOD2_HUMAN]	3.93	1	2	2	2					0.47	0.73	0.77	1.20	737	84.6	6.71
P07737	Profilin-1 OS=Homo sapiens GN=PFN1 PE=1 SV=2 - [PROF1_HUMAN]	70.71	1	9	9	133	0.89	0.91	0.88	0.92	0.98	0.95	0.94	0.93	140	15.0	8.27
Q9NZQ7	Programmed cell death 1 ligand 1 OS=Homo sapiens GN=CD274 PE=1 SV=1 - [PD1L1_HUMAN]	3.45	1	1	1	1					0.98	0.23	0.71	0.16	290	33.3	7.23
Q9BUL8	Programmed cell death protein 10 OS=Homo sapiens GN=PDCD10 PE=1 SV=1 - [PDC10_HUMAN]	6.13	1	1	1	3	1.21	1.11	1.04	0.74					212	24.7	8.19
O75340	Programmed cell death protein 6 OS=Homo sapiens GN=PDCD6 PE=1 SV=1 - [PDCD6_HUMAN]	3.14	1	1	1	1	1.15	1.26	1.16	1.28					191	21.9	5.40
P35232	Prohibitin OS=Homo sapiens GN=PHB PE=1 SV=1 - [PHB_HUMAN]	4.41	1	1	1	1	0.85	0.98	0.78	0.90					272	29.8	5.76
P01236	Prolactin OS=Homo sapiens GN=PRL PE=1 SV=1 - [PRL_HUMAN]	5.73	1	1	1	1	1.45	1.33	1.02	0.95					227	25.9	6.98
Q9UQ80	Proliferation-associated protein 2G4 OS=Homo sapiens GN=PA2G4 PE=1 SV=3 - [PA2G4_HUMAN]	11.17	1	2	2	2	0.92	1.54	7.83	####					394	43.8	6.55
O43272	Proline dehydrogenase 1, mitochondrial OS=Homo sapiens GN=PRODH PE=1 SV=3 - [PROD_HUMAN]	5.33	1	2	2	2	1.35	1.22	1.56	1.42	0.17	0.12	0.44	0.31	600	68.0	7.87
Q8IZL8	Proline-, glutamic acid- and leucine-rich protein 1 OS=Homo sapiens GN=PELP1 PE=1 SV=2 - [PELP1_HUMAN]	1.24	1	1	1	2	1.12	1.17	0.92	0.96					1130	119.6	4.34
Q96NZ9	Proline-rich acidic protein 1 OS=Homo sapiens GN=PRAP1 PE=2 SV=2 - [PRAP1_HUMAN]	28.48	1	3	3	9					1.27	1.21	1.18	1.09	151	17.2	5.36
Q9ULL5	Proline-rich protein 12 OS=Homo sapiens GN=PRR12 PE=1 SV=2 - [PRR12_HUMAN]	1.65	1	1	1	1					0.90	1.36	1.24	1.88	1215	129.9	8.00
Q16378	Proline-rich protein 4 OS=Homo sapiens GN=PRR4 PE=1 SV=3 - [PROL4_HUMAN]	11.19	1	1	1	1	0.63	0.72	0.64	0.74					134	15.1	7.06
P85299	Proline-rich protein 5 OS=Homo sapiens GN=PRR5 PE=1 SV=1 - [PRR5_HUMAN]	4.38	1	1	1	1					0.46	0.78	0.76	1.27	388	42.7	8.18
Q92733	Proline-rich protein PRCC OS=Homo sapiens GN=PRCC PE=1 SV=1 - [PRCC_HUMAN]	4.07	1	1	1	1	1.01	0.98	1.03	1.00					491	52.4	5.10
Q7Z6L0	Proline-rich transmembrane protein 2 OS=Homo sapiens GN=PRRT2 PE=1 SV=1 - [PRRT2_HUMAN]	3.82	1	1	1	1	0.63	0.66	0.62	0.64					340	34.9	4.70
Q5FWE3	Proline-rich transmembrane protein 3 OS=Homo sapiens GN=PRRT3 PE=1 SV=3 - [PRRT3_HUMAN]	3.77	1	2	2	5	0.85	0.94	0.90	1.01					981	102.1	7.78
Q9H939	Proline-serine-threonine phosphatase-interacting protein 2 OS=Homo sapiens GN=PSTPIP2 PE=1 SV=4 - [PPIP2_HUMAN]	2.40	1	1	1	2					2.50	2.50	2.42	2.42	334	38.8	8.48
Q07954	Prolow-density lipoprotein receptor-related protein 1 OS=Homo sapiens GN=LRP1 PE=1 SV=2 - [LRP1_HUMAN]	8.21	1	27	27	46	1.02	1.07	1.00	1.03	1.01	1.02	1.16	1.18	4544	504.3	5.39

Q8IVL5	Prolyl 3-hydroxylase 2 OS=Homo sapiens GN=LEPREL1 PE=1 SV=1 - [P3H2_HUMAN]	6.92	1	2	2	2	0.83	0.80	0.82	0.80					708	80.9	5.71
Q8IVL6	Prolyl 3-hydroxylase 3 OS=Homo sapiens GN=LEPREL2 PE=2 SV=1 - [P3H3_HUMAN]	3.26	1	2	2	2					2.38	2.47	2.57	2.66	736	81.8	6.32
O15460	Prolyl 4-hydroxylase subunit alpha-2 OS=Homo sapiens GN=P4HA2 PE=1 SV=1 - [P4HA2_HUMAN]	2.62	1	1	1	1	0.59	0.57	0.47	0.46					535	60.9	5.71
Q7Z4N8	Prolyl 4-hydroxylase subunit alpha-3 OS=Homo sapiens GN=P4HA3 PE=1 SV=1 - [P4HA3_HUMAN]	2.76	1	1	1	1	1.34	1.57	1.53	1.81					544	61.1	6.49
P48147	Prolyl endopeptidase OS=Homo sapiens GN=PREP PE=1 SV=2 - [PPCE_HUMAN]	3.52	1	1	1	1					1.00	0.64	1.34	0.85	710	80.6	5.86
Q8N271	Prominin-2 OS=Homo sapiens GN=PROM2 PE=1 SV=1 - [PROM2_HUMAN]	0.84	1	1	1	3	0.99	0.67	0.98	0.67	0.75	0.72	0.69	0.67	834	91.8	6.15
P27918	Properdin OS=Homo sapiens GN=CFP PE=1 SV=2 - [PROP_HUMAN]	38.17	1	14	15	139	1.33	1.15	1.15	1.13	0.79	0.86	0.92	0.92	469	51.2	7.90
P05165	Propionyl-CoA carboxylase alpha chain, mitochondrial OS=Homo sapiens GN=PCCA PE=1 SV=4 - [PCCA_HUMAN]	4.67	1	2	2	6	1.04	1.05	0.82	0.84					728	80.0	7.52
Q8NBP7	Proprotein convertase subtilisin/kexin type 9 OS=Homo sapiens GN=PCSK9 PE=1 SV=3 - [PCSK9_HUMAN]	13.58	1	8	8	25	1.14	1.08	1.06	1.00	0.89	0.94	0.86	0.96	692	74.2	6.61
Q92786	Prospero homeobox protein 1 OS=Homo sapiens GN=PROX1 PE=1 SV=2 - [PROX1_HUMAN]	3.12	1	1	2	2	1.02	0.66	0.78	0.50					737	83.2	7.18
P34995	Prostaglandin E2 receptor EP1 subtype OS=Homo sapiens GN=PTGER1 PE=2 SV=3 - [PE2R1_HUMAN]	1.74	1	1	1	1					1.22	1.10	1.53	1.38	402	41.8	11.72
P23219	Prostaglandin G/H synthase 1 OS=Homo sapiens GN=PTGS1 PE=1 SV=2 - [PGH1_HUMAN]	1.50	2	1	1	3	1.17	1.39	1.09	1.30					599	68.6	7.23
P41222	Prostaglandin-H2 D-isomerase OS=Homo sapiens GN=PTGDS PE=1 SV=1 - [PTGDS_HUMAN]	23.16	1	5	5	18	0.89	0.84	0.83	0.79	1.04	0.95	0.98	0.90	190	21.0	7.80
Q6UY27	Prostate and testis expressed protein 2 OS=Homo sapiens GN=PATE2 PE=2 SV=1 - [PATE2_HUMAN]	16.81	1	1	1	1					0.23	0.19	1.61	1.33	113	13.0	7.25
Q86YD1	Prostate tumor-overexpressed gene 1 protein OS=Homo sapiens GN=PTOV1 PE=1 SV=1 - [PTOV1_HUMAN]	4.57	1	1	1	3	1.37	1.05	1.35	1.04					416	46.8	10.54
P07288	Prostate-specific antigen OS=Homo sapiens GN=KLK3 PE=1 SV=2 - [KLK3_HUMAN]	2.68	1	1	1	1					1.03	0.81	0.86	0.68	261	28.7	7.68
P25786	Proteasome subunit alpha type-1 OS=Homo sapiens GN=PSMA1 PE=1 SV=1 - [PSA1_HUMAN]	8.37	1	2	2	2	0.88	0.90	0.93	0.96					263	29.5	6.61
P28066	Proteasome subunit alpha type-5 OS=Homo sapiens GN=PSMA5 PE=1 SV=3 - [PSA5_HUMAN]	4.15	1	1	1	2	0.96	0.89	0.99	0.93					241	26.4	4.79
P60900	Proteasome subunit alpha type-6 OS=Homo sapiens GN=PSMA6 PE=1 SV=1 - [PSA6_HUMAN]	5.28	1	1	1	3					0.81	0.88	0.87	0.94	246	27.4	6.76
P49721	Proteasome subunit beta type-2 OS=Homo sapiens GN=PSMB2 PE=1 SV=1 - [PSB2_HUMAN]	5.47	1	1	1	2	0.62	0.66	0.59	0.63					201	22.8	7.02
P28062	Proteasome subunit beta type-8 OS=Homo sapiens GN=PSMB8 PE=1 SV=3 - [PSB8_HUMAN]	8.33	1	2	2	5	0.81	0.84	0.89	0.92	0.87	0.90	0.97	1.00	276	30.3	7.43
Q9NUX5	Protection of telomeres protein 1 OS=Homo sapiens GN=POT1 PE=1 SV=1 - [POTE1_HUMAN]	1.10	1	1	1	1					0.16	0.51	0.05	0.15	634	71.4	6.73
P11171	Protein 4.1 OS=Homo sapiens GN=EPB41 PE=1 SV=4 - [41_HUMAN]	0.93	1	1	1	2	1.21	1.01	1.09	0.92	1.63	2.02	1.29	1.59	864	97.0	5.58
P55198	Protein AF-17 OS=Homo sapiens GN=MLLT6 PE=1 SV=2 - [AF17_HUMAN]	2.65	1	1	1	1	0.75	0.41	0.77	0.42					1093	112.0	8.62
Q8IVF2	Protein AHNAK2 OS=Homo sapiens GN=AHNAK2 PE=1 SV=2 - [AHNK2_HUMAN]	0.72	1	2	2	2	1.39	1.26	1.04	0.96	1.28	1.15	1.40	1.26	5795	616.2	5.36
Q5T1N1	Protein AKNAD1 OS=Homo sapiens GN=AKNAD1 PE=2 SV=3 - [AKND1_HUMAN]	1.56	1	1	1	1					0.62	0.62	1.06	1.07	836	92.8	6.81
P84996	Protein ALEX OS=Homo sapiens GN=GNAS PE=1 SV=1 - [ALEX_HUMAN]	1.76	1	1	1	4					0.75	0.09	0.92	0.13	626	67.9	11.55

P02760	Protein AMBP OS=Homo sapiens GN=AMBP PE=1 SV=1 - [AMBP_HUMAN]	57.39	1	20	20	1146	1.07	1.06	1.06	1.04	0.94	0.95	0.93	0.97	352	39.0	6.25
Q8IY45	Protein AMN1 homolog OS=Homo sapiens GN=AMN1 PE=2 SV=4 - [AMN1_HUMAN]	5.81	1	1	1	1					1.42	1.42	1.36	1.36	258	28.4	7.85
Q5VTE6	Protein angel homolog 2 OS=Homo sapiens GN=ANGEL2 PE=2 SV=1 - [ANGE2_HUMAN]	0.92	1	1	1	1	1.06	0.83	1.02	0.80					544	62.3	7.81
Q8J025	Protein APCDD1 OS=Homo sapiens GN=APCDD1 PE=1 SV=1 - [APCD1_HUMAN]	2.14	1	1	1	1	1.73	2.54	0.88	1.29					514	58.8	8.15
Q96LA8	Protein arginine N-methyltransferase 6 OS=Homo sapiens GN=PRMT6 PE=1 SV=1 - [ANM6_HUMAN]	3.73	1	1	1	2					0.89	0.83	6.00	5.57	375	41.9	5.44
Q9UKV8	Protein argonaute-2 OS=Homo sapiens GN=EIF2C2 PE=1 SV=3 - [AGO2_HUMAN]	1.40	1	1	1	1	0.87	0.93	0.93	1.00					859	97.1	9.19
Q9HCK5	Protein argonaute-4 OS=Homo sapiens GN=EIF2C4 PE=1 SV=2 - [AGO4_HUMAN]	2.09	1	1	2	2					1.20	0.99	1.43	1.18	861	97.0	9.06
Q96SD1	Protein artemis OS=Homo sapiens GN=DCLRE1C PE=1 SV=2 - [DCR1C_HUMAN]	1.01	1	1	1	1					1.48	1.29	1.06	0.92	692	78.4	6.02
Q8N100	Protein atonal homolog 7 OS=Homo sapiens GN=ATOH7 PE=2 SV=1 - [ATOH7_HUMAN]	8.55	1	1	1	4					0.58	0.56	0.48	0.47	152	16.9	9.51
Q6PGQ7	Protein aurora borealis OS=Homo sapiens GN=BORA PE=1 SV=2 - [BORA_HUMAN]	2.50	1	2	2	2					0.69	0.68	0.69	0.69	559	61.2	4.94
Q6PI77	Protein BHLHb9 OS=Homo sapiens GN=BHLHB9 PE=2 SV=1 - [BHLH9_HUMAN]	2.56	1	1	1	2					0.95	0.84	1.04	0.91	547	60.3	7.58
Q96G01	Protein bicaudal D homolog 1 OS=Homo sapiens GN=BICD1 PE=1 SV=3 - [BICD1_HUMAN]	2.36	1	2	2	3					0.88	0.92	0.69	0.73	975	110.7	5.81
Q8WUW1	Protein BRICK1 OS=Homo sapiens GN=BRK1 PE=1 SV=1 - [BRK1_HUMAN]	6.67	1	1	1	1	0.75	0.79	0.72	0.75					75	8.7	5.45
Q96NH3	Protein broad-minded OS=Homo sapiens GN=BROMI PE=2 SV=4 - [BROMI_HUMAN]	1.03	1	1	1	2	0.98	0.98	1.17	1.17					1257	144.7	6.74
P15036	Protein C-ets-2 OS=Homo sapiens GN=ETS2 PE=1 SV=1 - [ETS2_HUMAN]	5.33	1	1	1	1	1.11	1.15	1.04	1.08					469	53.0	5.03
Q9Y2B0	Protein canopy homolog 2 OS=Homo sapiens GN=CNPY2 PE=1 SV=1 - [CNPY2_HUMAN]	4.40	1	1	1	3					0.99	0.92	1.05	0.98	182	20.6	4.92
Q9BT09	Protein canopy homolog 3 OS=Homo sapiens GN=CNPY3 PE=1 SV=1 - [CNPY3_HUMAN]	3.60	1	1	1	2	0.85	0.93	1.06	1.17					278	30.7	5.49
Q8NG31	Protein CASC5 OS=Homo sapiens GN=CASC5 PE=1 SV=3 - [CASC5_HUMAN]	1.67	1	3	3	3	1.26	1.48	1.26	1.50	0.78	0.82	0.79	0.83	2342	265.2	5.47
Q13948	Protein CASP OS=Homo sapiens GN=CUX1 PE=1 SV=2 - [CASP_HUMAN]	1.62	2	1	1	4	1.65	1.60	1.64	1.60	1.63	2.29	1.55	2.17	678	77.4	5.44
Q9UKY7	Protein CDV3 homolog OS=Homo sapiens GN=CDV3 PE=1 SV=1 - [CDV3_HUMAN]	22.87	1	2	2	2					1.14	1.51	1.35	1.40	258	27.3	6.40
Q8WUH1	Protein Churchill OS=Homo sapiens GN=CHURC1 PE=1 SV=1 - [CHUR_HUMAN]	5.36	1	1	1	1							1.03	1.08	112	12.9	4.84
O75629	Protein CREG1 OS=Homo sapiens GN=CREG1 PE=1 SV=1 - [CREG1_HUMAN]	2.73	1	1	1	1	0.81	0.51	0.79	0.50					220	24.1	7.59
Q96RT6	Protein cTAGE-2 OS=Homo sapiens GN=CTAGE1 PE=1 SV=2 - [CTGE2_HUMAN]	0.81	3	1	1	1					1.12	0.73	0.99	0.64	745	85.2	6.16
O60888	Protein CutA OS=Homo sapiens GN=CUTA PE=1 SV=2 - [CUTA_HUMAN]	23.46	1	3	3	6	1.14	1.16	1.58	1.64	0.94	1.03	0.94	1.02	179	19.1	5.50
Q9P219	Protein Daple OS=Homo sapiens GN=CCDC88C PE=1 SV=3 - [DAPLE_HUMAN]	0.79	1	1	2	7	0.80	1.03	0.86	1.10					2028	228.1	6.23
Q9UBU7	Protein DBF4 homolog A OS=Homo sapiens GN=DBF4 PE=1 SV=1 - [DBF4A_HUMAN]	1.19	1	1	1	1					3.55	3.53	3.17	3.16	674	76.8	7.90
Q86Y01	Protein deltex-1 OS=Homo sapiens GN=DTX1 PE=1 SV=1 - [DTX1_HUMAN]	4.03	1	1	1	1	1.04	1.02	1.15	1.13					620	67.3	9.60

O60610	Protein diaphanous homolog 1 OS=Homo sapiens GN=DIAPH1 PE=1 SV=2 - [DIAP1_HUMAN]	2.99	1	3	3	9	0.68	0.67	0.69	0.69	1.89	1.75	1.72	1.59	1272	141.3	5.41
Q9NSV4	Protein diaphanous homolog 3 OS=Homo sapiens GN=DIAPH3 PE=1 SV=4 - [DIAP3_HUMAN]	5.11	1	4	4	4	0.89	0.81	1.05	0.96	0.98	0.80	1.06	0.86	1193	136.8	7.03
P30101	Protein disulfide-isomerase A3 OS=Homo sapiens GN=PDIA3 PE=1 SV=4 - [PDIA3_HUMAN]	8.32	1	4	4	10	0.59	0.58	0.57	0.56	0.82	0.83	0.80	0.81	505	56.7	6.35
P13667	Protein disulfide-isomerase A4 OS=Homo sapiens GN=PDIA4 PE=1 SV=2 - [PDIA4_HUMAN]	4.65	1	2	2	3	1.25	1.56	0.73	0.92	0.48	0.46	0.56	0.53	645	72.9	5.07
Q15084	Protein disulfide-isomerase A6 OS=Homo sapiens GN=PDIA6 PE=1 SV=1 - [PDIA6_HUMAN]	10.68	1	3	3	7					1.06	1.16	1.14	1.25	440	48.1	5.08
P07237	Protein disulfide-isomerase OS=Homo sapiens GN=P4HB PE=1 SV=3 - [PDIA1_HUMAN]	14.37	1	6	6	14	1.33	1.41	1.18	1.22	0.67	0.69	0.70	0.72	508	57.1	4.87
Q99497	Protein DJ-1 OS=Homo sapiens GN=PARK7 PE=1 SV=2 - [PARK7_HUMAN]	41.80	1	8	8	107	0.91	0.84	0.81	0.80	1.12	1.08	1.01	1.01	189	19.9	6.79
Q18PE1	Protein Dok-7 OS=Homo sapiens GN=DOK7 PE=1 SV=1 - [DOK7_HUMAN]	1.59	1	1	1	1	0.89	0.98	0.87	0.96					504	53.1	6.89
Q9Y3R5	Protein dopey-2 OS=Homo sapiens GN=DOPEY2 PE=1 SV=5 - [DOP2_HUMAN]	0.57	1	1	1	1	1.51	1.48	1.47	1.45					2298	258.1	6.29
Q8N350	Protein Dos OS=Homo sapiens GN=DOS PE=1 SV=2 - [DOS_HUMAN]	2.48	1	1	1	4					1.13	1.23	1.15	1.25	725	75.8	6.61
Q9NYP3	Protein downstream neighbor of Son OS=Homo sapiens GN=DONSON PE=1 SV=2 - [DONS_HUMAN]	2.83	1	1	1	1					0.72	0.24	0.72	0.24	566	62.7	8.66
Q6NUT2	Protein dpy-19 homolog 2 OS=Homo sapiens GN=DPY19L2 PE=1 SV=2 - [D19L2_HUMAN]	1.19	1	1	1	1					1.06	1.03	0.91	0.89	758	87.3	9.10
Q8WYP5	Protein ELYS OS=Homo sapiens GN=AHCTF1 PE=1 SV=3 - [ELYS_HUMAN]	2.96	1	4	4	4	1.16	1.15	1.06	1.06	0.85	0.86	0.88	0.89	2266	252.3	6.60
Q03111	Protein ENL OS=Homo sapiens GN=MLLT1 PE=1 SV=2 - [ENL_HUMAN]	1.97	1	2	2	4	1.01	0.97	1.39	1.34	0.86	0.80	1.03	0.60	559	62.0	8.59
P49257	Protein ERGIC-53 OS=Homo sapiens GN=LMAN1 PE=1 SV=2 - [LMAN1_HUMAN]	9.61	1	3	3	6	0.93	0.89	0.94	0.91	1.01	0.90	0.87	0.77	510	57.5	6.77
Q9HAT1	Protein ERGIC-53-like OS=Homo sapiens GN=LMAN1L PE=2 SV=2 - [LMA1L_HUMAN]	2.85	1	1	1	1					0.63	0.65	0.55	0.56	526	57.1	7.83
Q9NYK6	Protein EURL homolog OS=Homo sapiens GN=EURL PE=2 SV=3 - [EURL_HUMAN]	5.72	1	1	1	1					1.23	2.38	0.90	1.75	297	33.9	6.57
Q13158	Protein FADD OS=Homo sapiens GN=FADD PE=1 SV=1 - [FADD_HUMAN]	9.13	1	1	1	1					1.44	0.23	1.19	0.19	208	23.3	5.69
Q5XKR9	Protein FAM104B OS=Homo sapiens GN=FAM104B PE=2 SV=1 - [F104B_HUMAN]	10.43	1	1	1	1	1.08	1.26	0.92	1.09					115	13.1	9.61
Q96BN8	Protein FAM105B OS=Homo sapiens GN=FAM105B PE=1 SV=3 - [F105B_HUMAN]	1.99	1	1	1	3					1.61	1.37	1.39	1.18	352	40.2	5.47
Q8TC76	Protein FAM110B OS=Homo sapiens GN=FAM110B PE=1 SV=1 - [F110B_HUMAN]	2.97	1	1	1	1					0.98	1.15	1.14	1.35	370	40.7	9.39
Q8TAY7	Protein FAM110D OS=Homo sapiens GN=FAM110D PE=2 SV=1 - [F110D_HUMAN]	3.69	1	1	1	1					0.93	0.82	0.89	0.79	271	28.7	10.04
Q9NRY5	Protein FAM114A2 OS=Homo sapiens GN=FAM114A2 PE=1 SV=4 - [F1142_HUMAN]	3.56	1	1	1	1	1.85	1.36	1.45	1.09					505	55.4	4.88
Q9C073	Protein FAM117A OS=Homo sapiens GN=FAM117A PE=2 SV=1 - [F117A_HUMAN]	3.53	1	1	1	1					0.45	0.37	1.57	1.31	453	48.3	8.31
Q9BPY3	Protein FAM118B OS=Homo sapiens GN=FAM118B PE=1 SV=1 - [F118B_HUMAN]	10.54	1	2	2	2					0.78	0.86	1.00	1.10	351	39.5	5.99
Q8IXS8	Protein FAM126B OS=Homo sapiens GN=FAM126B PE=1 SV=1 - [F126B_HUMAN]	2.26	1	1	1	4					0.83	0.41	0.94	0.48	530	58.6	7.90
Q4G0M1	Protein FAM132B OS=Homo sapiens GN=FAM132B PE=2 SV=2 - [F132B_HUMAN]	2.26	1	1	1	1					0.96	0.71	0.93	0.69	354	37.3	9.83

Q49AJ0	Protein FAM135B OS=Homo sapiens GN=FAM135B PE=2 SV=2 - [F135B_HUMAN]	1.07	1	1	1	1					2.14	2.12	0.93	0.92	1406	155.7	5.86
Q6VEP3	Protein FAM138A/B/C/F OS=Homo sapiens GN=FAM138A PE=2 SV=1 - [F138A_HUMAN]	15.29	1	1	1	3					0.77	0.94	0.60	0.91	85	9.2	7.39
Q8NE31	Protein FAM13C OS=Homo sapiens GN=FAM13C PE=2 SV=2 - [FA13C_HUMAN]	2.22	1	1	1	2					0.91	0.73	0.78	0.62	585	65.7	5.73
Q6UXT8	Protein FAM150A OS=Homo sapiens GN=FAM150A PE=2 SV=1 - [F150A_HUMAN]	13.18	1	2	2	2					0.68	0.56	0.90	0.74	129	14.3	10.58
Q6UX46	Protein FAM150B OS=Homo sapiens GN=FAM150B PE=2 SV=2 - [F150B_HUMAN]	4.61	1	1	1	1					0.85	1.01	0.71	0.85	152	16.9	9.69
Q5W0V3	Protein FAM160B1 OS=Homo sapiens GN=FAM160B1 PE=2 SV=1 - [F16B1_HUMAN]	3.27	1	1	2	2	1.46		1.34						765	86.5	5.29
Q86V87	Protein FAM160B2 OS=Homo sapiens GN=FAM160B2 PE=2 SV=2 - [F16B2_HUMAN]	6.19	1	2	2	2					0.89	0.13	0.85	0.12	743	82.3	5.47
Q96MY7	Protein FAM161B OS=Homo sapiens GN=FAM161B PE=2 SV=2 - [F161B_HUMAN]	6.18	1	2	2	2		1.30		0.88	0.88	0.74	0.93	0.78	647	73.6	9.41
Q96GL9	Protein FAM163A OS=Homo sapiens GN=FAM163A PE=2 SV=1 - [F163A_HUMAN]	7.78	1	1	1	1					0.94	1.04	1.08	1.21	167	17.6	5.82
P0C2L3	Protein FAM163B OS=Homo sapiens GN=FAM163B PE=2 SV=1 - [F163B_HUMAN]	4.82	1	1	1	1					1.35	1.33	0.86	0.86	166	18.2	4.92
Q96KS9	Protein FAM167A OS=Homo sapiens GN=FAM167A PE=2 SV=1 - [F167A_HUMAN]	10.75	1	1	1	1	0.87	1.25	0.87	1.26					214	24.2	6.32
Q5VUB5	Protein FAM171A1 OS=Homo sapiens GN=FAM171A1 PE=1 SV=1 - [F1711_HUMAN]	1.69	1	1	1	1	0.66	0.69	0.70	0.73					890	97.8	6.58
Q6UWF9	Protein FAM180A OS=Homo sapiens GN=FAM180A PE=2 SV=1 - [F180A_HUMAN]	4.62	1	1	1	1					0.31	0.68	0.42	0.91	173	19.7	8.47
Q8NB25	Protein FAM184A OS=Homo sapiens GN=FAM184A PE=2 SV=3 - [F184A_HUMAN]	3.60	1	2	3	8	0.94	1.30	0.79	1.16	0.52	0.65	0.76	0.96	1140	132.9	5.83
Q9ULE4	Protein FAM184B OS=Homo sapiens GN=FAM184B PE=2 SV=3 - [F184B_HUMAN]	1.89	1	1	1	1	0.80	0.65	0.71	0.59					1060	121.0	6.18
Q8IYM0	Protein FAM186B OS=Homo sapiens GN=FAM186B PE=2 SV=2 - [F186B_HUMAN]	3.70	1	2	2	3					1.16	1.10	1.11	1.06	893	103.7	9.01
A8MYZ0	Protein FAM188B2 OS=Homo sapiens GN=FAM188B2 PE=3 SV=2 - [F1882_HUMAN]	5.56	1	1	1	2	1.26	1.67	0.91	1.21					360	41.0	7.91
Q96ET8	Protein FAM18B2 OS=Homo sapiens GN=FAM18B2 PE=1 SV=3 - [F18B2_HUMAN]	6.16	1	1	1	1	0.92	1.58	1.18	2.06					276	31.1	9.29
Q9C0I3	Protein FAM190A OS=Homo sapiens GN=FAM190A PE=2 SV=2 - [F190A_HUMAN]	1.67	1	1	1	1					1.03				900	99.4	7.83
P78312	Protein FAM193A OS=Homo sapiens GN=FAM193A PE=1 SV=2 - [F193A_HUMAN]	2.92	1	2	2	5	0.43	0.36	0.53	0.56					1265	139.9	6.48
Q6ZU69	Protein FAM205A OS=Homo sapiens GN=FAM205A PE=2 SV=4 - [F205A_HUMAN]	0.82	1	1	1	1					0.66	0.69	1.04	1.10	1335	148.0	8.29
Q9UK61	Protein FAM208A OS=Homo sapiens GN=FAM208A PE=1 SV=3 - [F208A_HUMAN]	2.34	1	3	3	7	1.09	0.95	1.17	1.02	1.42	1.19	1.24	1.04	1670	188.9	5.80
Q96MK3	Protein FAM20A OS=Homo sapiens GN=FAM20A PE=1 SV=4 [FA20A_HUMAN]	6.47	1	2	2	2	1.05	1.12	1.02	1.09					541	61.4	7.99
Q32MH5	Protein FAM214A OS=Homo sapiens GN=FAM214A PE=2 SV=2 - [F214A_HUMAN]	4.83	1	2	2	5	3.11	1.97	2.30	1.46	1.12	0.99	1.08	1.04	1076	121.6	7.90
Q96MZ4	Protein FAM218A OS=Homo sapiens GN=FAM218A PE=2 SV=1 - [F218A_HUMAN]	5.10	1	1	1	1					1.17	1.13	1.05	1.01	157	17.0	11.81
Q5SNX5	Protein FAM27E2 OS=Homo sapiens GN=FAM27E2 PE=2 SV=1 - [F27E2_HUMAN]	16.80	3	2	2	3	1.55	1.43	1.03	0.96	0.20	0.41	0.24	0.51	125	14.7	11.84
Q92520	Protein FAM3C OS=Homo sapiens GN=FAM3C PE=1 SV=1 - [FAM3C_HUMAN]	21.59	1	4	4	7	1.01	0.89	1.03	0.92	0.89	0.90	1.03	1.03	227	24.7	8.29

Q9ULQ0	Protein FAM40B OS=Homo sapiens GN=FAM40B PE=2 SV=2 - [FA40B_HUMAN]	2.04	1	1	1	1					1.24	1.16	1.18	1.10	834	95.3	5.91
Q3ZLR7	Protein FAM48B1 OS=Homo sapiens GN=FAM48B1 PE=2 SV=2 - [F48B1_HUMAN]	1.94	1	1	1	2	0.96	0.67	0.86	0.60					823	89.4	8.78
Q9NYF3	Protein FAM53C OS=Homo sapiens GN=FAM53C PE=1 SV=1 - [FA53C_HUMAN]	7.65	1	1	1	2	0.96	0.99	1.00	1.04					392	43.1	8.65
Q75VX8	Protein FAM59B OS=Homo sapiens GN=FAM59B PE=2 SV=3 - [FA59B_HUMAN]	1.60	1	1	1	1					0.98		1.60		874	92.8	6.90
Q6ZS17	Protein FAM65A OS=Homo sapiens GN=FAM65A PE=1 SV=1 [FA65A_HUMAN]	1.23	1	1	1	3					1.12	1.01	1.02	0.93	1223	132.2	6.28
Q9Y4F9	Protein FAM65B OS=Homo sapiens GN=FAM65B PE=1 SV=4 - [FA65B_HUMAN]	1.31	1	1	1	2	0.67	0.62	0.66	0.61	0.95	1.36	0.57	0.82	1068	118.4	5.41
Q96MK2	Protein FAM65C OS=Homo sapiens GN=FAM65C PE=2 SV=4 - [FA65C_HUMAN]	1.37	1	1	1	2	1.84	1.75	1.97	1.87					946	105.2	7.43
Q5T7M9	Protein FAM69A OS=Homo sapiens GN=FAM69A PE=2 SV=1 [FA69A_HUMAN]	2.10	1	1	1	1					0.87	0.77	0.96	0.85	428	49.0	7.17
Q8IYT1	Protein FAM71A OS=Homo sapiens GN=FAM71A PE=2 SV=2 [FA71A_HUMAN]	2.86	1	1	1	1					0.95	0.86	0.93	0.85	594	63.1	9.64
Q8NAN2	Protein FAM73A OS=Homo sapiens GN=FAM73A PE=2 SV=1 [FA73A_HUMAN]	3.32	1	1	1	1					0.77	1.08	0.66	0.92	632	71.0	5.63
Q5RGS2	Protein FAM75A2 OS=Homo sapiens GN=FAM75A2 PE=3 SV=1 - [F75A2_HUMAN]	2.30	6	1	2	2	1.11	0.91	1.22	1.00					1347	148.6	8.85
Q6ZQQ2	Protein FAM75D1 OS=Homo sapiens GN=FAM75D1 PE=1 SV=1 - [F75D1_HUMAN]	2.03	3	2	3	3	1.00	1.23	1.00	1.23					1576	175.5	8.85
Q6ZUB1	Protein FAM75E1 OS=Homo sapiens GN=FAM75E1 PE=2 SV=2 - [F75E1_HUMAN]	0.42	1	1	1	1					1.13	1.10	1.12	1.09	1445	157.0	9.10
Q96LP2	Protein FAM81B OS=Homo sapiens GN=FAM81B PE=2 SV=3 - [FA81B_HUMAN]	2.65	1	1	1	1					1.17	1.01	1.00	0.86	452	52.0	9.10
Q9BQN1	Protein FAM83C OS=Homo sapiens GN=FAM83C PE=1 SV=3 - [FA83C_HUMAN]	5.89	1	2	2	4	0.78	0.60	0.93	0.67					747	81.0	8.07
Q96KN4	Protein FAM84A OS=Homo sapiens GN=FAM84A PE=1 SV=2 [FA84A_HUMAN]	6.85	1	1	1	2					0.91	0.66	0.84	0.61	292	32.5	5.72
Q9NVL1	Protein FAM86C1 OS=Homo sapiens GN=FAM86C1 PE=2 SV=3 - [FA86C_HUMAN]	18.18	1	1	1	1				1.17					165	18.4	8.19
Q658Y4	Protein FAM91A1 OS=Homo sapiens GN=FAM91A1 PE=1 SV=3 - [F91A1_HUMAN]	2.15	3	1	3	26	0.85	1.13	0.90	1.20					838	93.8	6.39
Q68CZ1	Protein fantom OS=Homo sapiens GN=RPGRIP1L PE=1 SV=2 - [FTM_HUMAN]	1.37	1	1	1	4	0.81	1.08	0.79	1.07	0.28	0.32	0.81	0.92	1315	151.1	5.35
Q96JP0	Protein fem-1 homolog C OS=Homo sapiens GN=FEM1C PE=2 SV=1 - [FEM1C_HUMAN]	3.57	1	1	2	2	1.04	1.91	1.23	2.28					617	68.6	7.68
Q13045	Protein flightless-1 homolog OS=Homo sapiens GN=FLII PE=1 SV=2 - [FLII_HUMAN]	1.10	1	1	1	7	1.24	1.32	1.23	1.31	0.83	1.01	0.82	1.05	1269	144.7	6.05
Q5TBA9	Protein furry homolog OS=Homo sapiens GN=FRY PE=1 SV=1 - [FRY_HUMAN]	0.66	5	1	3	3					0.80	0.74	0.76	0.70	3013	338.7	5.99
O94915	Protein furry homolog-like OS=Homo sapiens GN=FRYL PE=1 SV=2 - [FRYL_HUMAN]	2.36	2	4	5	11	0.57	0.62	0.55	0.61	2.09	1.68	1.14	0.92	3013	339.4	5.58
O95866	Protein G6b OS=Homo sapiens GN=G6B PE=1 SV=1 - [G6B_HUMAN]	19.92	1	5	5	17					1.15	1.17	1.14	1.13	241	26.1	9.61
Q8N292	Protein GAPT OS=Homo sapiens GN=GAPT PE=1 SV=1 - [GAPT_HUMAN]	13.38	1	1	1	1	0.95	1.22	1.10	1.43					157	17.9	8.44
Q5U4N7	Protein GDF5OS, mitochondrial OS=Homo sapiens GN=GDF5OS PE=1 SV=2 - [GDF5O_HUMAN]	3.60	1	1	1	1					0.91	0.85	1.14	1.06	250	28.2	12.03
Q5VW38	Protein GPR107 OS=Homo sapiens GN=GPR107 PE=2 SV=1 - [GP107_HUMAN]	2.67	1	1	1	2					0.68	0.66	3.56	3.46	600	66.9	7.24

Q4ZG55	Protein GREB1 OS=Homo sapiens GN=GREB1 PE=2 SV=1 - [GREB1_HUMAN]	3.13	1	2	2	3	0.74	0.74	1.14	0.77					1949	216.3	6.95
Q9ULI3	Protein HEG homolog 1 OS=Homo sapiens GN=HEG1 PE=1 SV=3 - [HEG1_HUMAN]	5.50	1	5	5	16	1.44	1.56	1.33	1.48	1.01	0.83	1.13	0.95	1381	147.4	6.18
Q9UJC3	Protein Hook homolog 1 OS=Homo sapiens GN=HOOK1 PE=1 SV=2 - [HOOK1_HUMAN]	3.30	1	1	1	1					1.65	0.80	1.85	0.89	728	84.6	5.15
Q96ED9	Protein Hook homolog 2 OS=Homo sapiens GN=HOOK2 PE=1 SV=3 - [HOOK2_HUMAN]	5.15	1	2	2	2	0.79	0.86	0.94	1.03					719	83.2	5.47
Q86VS8	Protein Hook homolog 3 OS=Homo sapiens GN=HOOK3 PE=1 SV=2 - [HOOK3_HUMAN]	5.43	1	2	2	6	0.81	0.62	0.76	0.59					718	83.1	5.17
Q1MX18	Protein inscuteable homolog OS=Homo sapiens GN=INSC PE=1 SV=1 - [INSC_HUMAN]	4.84	1	1	1	1					0.44	0.11	0.54	0.14	579	63.4	5.52
Q9ULD6	Protein inturned OS=Homo sapiens GN=INTU PE=2 SV=2 - [INTU_HUMAN]	3.18	1	1	2	3	0.64	0.61	0.75	0.72					942	105.6	5.90
P78504	Protein jagged-1 OS=Homo sapiens GN=JAG1 PE=1 SV=3 - [JAG1_HUMAN]	1.56	1	2	2	2					0.44	0.10	0.67	0.83	1218	133.7	6.06
Q92833	Protein Jumonji OS=Homo sapiens GN=JARID2 PE=1 SV=2 - [JARD2_HUMAN]	2.57	1	2	3	4	0.94	0.58	0.71	0.44					1246	138.6	9.38
O15033	Protein KIAA0317 OS=Homo sapiens GN=KIAA0317 PE=2 SV=3 - [K0317_HUMAN]	1.94	1	1	1	1	1.34	1.11	1.07	0.89					823	94.2	7.25
Q8WUJ3	Protein KIAA1199 OS=Homo sapiens GN=KIAA1199 PE=1 SV=2 - [K1199_HUMAN]	2.72	1	3	3	4	0.98	0.88	0.92	0.82	0.77	0.90	1.05	1.22	1361	152.9	7.85
Q8IX03	Protein KIBRA OS=Homo sapiens GN=WWC1 PE=1 SV=1 - [KIBRA_HUMAN]	0.99	1	1	1	2					1.40	1.16	1.21	1.00	1113	125.2	5.85
Q9UKS6	Protein kinase C and casein kinase substrate in neurons protein 3 OS=Homo sapiens GN=PACSIN3 PE=1 SV=2 - [PACN3_HUMAN]	2.12	1	1	1	1					0.64	0.86	0.52	0.71	424	48.5	6.18
Q02156	Protein kinase C epsilon type OS=Homo sapiens GN=PRKCE PE=1 SV=1 - [KPCE_HUMAN]	4.75	1	2	2	2					0.47		0.49		737	83.6	7.12
P05129	Protein kinase C gamma type OS=Homo sapiens GN=PRKCG PE=1 SV=3 - [KPCG_HUMAN]	0.86	1	1	1	1	1.16	1.59	0.96	1.32					697	78.4	7.46
Q04759	Protein kinase C theta type OS=Homo sapiens GN=PRKCQ PE=1 SV=3 - [KPCT_HUMAN]	3.68	1	1	2	2					1.26	1.23	1.41	1.39	706	81.8	7.61
Q9ULU4	Protein kinase C-binding protein 1 OS=Homo sapiens GN=ZMYND8 PE=1 SV=2 - [PKCB1_HUMAN]	2.95	1	1	1	1	0.99	0.73	1.04	0.78					1186	131.6	7.20
A5PL33	Protein KRBA1 OS=Homo sapiens GN=KRBA1 PE=2 SV=2 - [KRBA1_HUMAN]	3.30	1	2	2	5	0.85	0.69	0.93	0.77	1.08	1.28	0.61	0.73	1030	107.4	7.83
Q8N9T8	Protein KRI1 homolog OS=Homo sapiens GN=KRI1 PE=1 SV=2 - [KRI1_HUMAN]	2.96	1	2	2	5	1.00	0.99	1.09	1.09	0.30	6.36	1.01	####	709	83.2	5.17
Q96RT1	Protein LAP2 OS=Homo sapiens GN=ERBB2IP PE=1 SV=2 - [LAP2_HUMAN]	1.27	1	1	1	1					1.25	0.93	1.35	1.00	1412	158.2	5.50
Q6ICC9	Protein LDOC1L OS=Homo sapiens GN=LDOC1L PE=2 SV=1 - [LDOCL_HUMAN]	10.46	1	1	1	1	0.82	1.07	0.98	1.27					239	26.1	11.18
Q8NG48	Protein Lines homolog OS=Homo sapiens GN=LINS PE=2 SV=2 - [LINES_HUMAN]	2.77	2	3	3	3	0.93	1.08	0.96	1.17					757	85.8	6.52
Q96GA3	Protein LTV1 homolog OS=Homo sapiens GN=LTV1 PE=1 SV=1 - [LTV1_HUMAN]	7.16	1	1	1	1					0.88	1.03	0.77	0.90	475	54.8	4.91
Q86UE4	Protein LYRIC OS=Homo sapiens GN=MTDH PE=1 SV=2 - [LYRIC_HUMAN]	1.89	1	1	1	3					0.94	1.10	0.95	1.11	582	63.8	9.32
Q8WZA0	Protein LZIC OS=Homo sapiens GN=LZIC PE=1 SV=1 - [LZIC_HUMAN]	4.21	1	1	1	3					4.52	3.87	4.50	3.84	190	21.5	4.94
Q8N8X9	Protein mab-21-like 3 OS=Homo sapiens GN=MAB21L3 PE=2 SV=2 - [MB213_HUMAN]	7.46	1	1	1	1	1.83	1.60	1.90	1.68					362	42.3	8.54
Q7L590	Protein MCM10 homolog OS=Homo sapiens GN=MCM10 PE=1 SV=2 - [MCM10_HUMAN]	1.26	1	1	1	1	0.74	0.87	1.04	1.24					875	98.1	8.75

Q7L592	Protein midA homolog, mitochondrial OS=Homo sapiens GN=C2orf56 PE=1 SV=1 - [MIDA_HUMAN]	2.95	1	1	1	1	1.11	1.08	1.09	1.07					441	49.2	8.34
Q6ZRQ5	Protein MMS22-like OS=Homo sapiens GN=MMS22L PE=1 SV=3 - [MMS22_HUMAN]	0.48	1	1	1	2	0.81	0.87	0.95	1.03					1243	142.2	7.12
Q8NHV4	Protein NEDD1 OS=Homo sapiens GN=NEDD1 PE=1 SV=1 - [NEDD1_HUMAN]	5.91	1	1	1	1	0.98	0.83	1.38	1.16					660	71.9	7.97
Q9BZQ8	Protein Niban OS=Homo sapiens GN=FAM129A PE=1 SV=1 - [NIBAN_HUMAN]	3.34	1	2	2	4	1.09	1.15	1.27	1.34	1.18	0.97	1.13	0.90	928	103.1	4.78
Q86WI3	Protein NLRC5 OS=Homo sapiens GN=NLRC5 PE=1 SV=3 - [NLRC5_HUMAN]	0.96	1	1	1	1	0.78	0.75	0.85	0.81					1866	204.5	6.44
Q6P988	Protein notum homolog OS=Homo sapiens GN=NOTUM PE=2 SV=2 - [NOTUM_HUMAN]	3.02	1	1	1	1					0.83	0.90	1.72	1.86	496	55.7	7.55
Q8WZA1	Protein O-linked-mannose beta-1,2-N-acetylglucosaminyltransferase 1 OS=Homo sapiens GN=POMGNT1 PE=1 SV=2 - [PMGT1_HUMAN]	5.45	1	2	2	3	1.12	1.09	1.03	1.01					660	75.2	6.83
Q13438	Protein OS-9 OS=Homo sapiens GN=OS9 PE=1 SV=1 - [OS9_HUMAN]	2.40	1	1	1	1					0.98	0.91	0.83	0.77	667	75.5	4.87
Q9BRX2	Protein pelota homolog OS=Homo sapiens GN=PELO PE=1 SV=2 - [PELO_HUMAN]	4.68	1	1	1	1	0.63	0.49	0.81	0.64					385	43.3	6.34
Q96T49	Protein phosphatase 1 regulatory inhibitor subunit 16B OS=Homo sapiens GN=PPP1R16B PE=1 SV=1 - [PP16B_HUMAN]	1.76	1	1	1	1	1.02	0.71	1.12	0.79					567	63.5	6.49
O14974	Protein phosphatase 1 regulatory subunit 12A OS=Homo sapiens GN=PPP1R12A PE=1 SV=1 - [MYPT1_HUMAN]	4.56	1	3	3	4	0.70	0.74	0.73	0.77	2.18	2.47	2.25	2.55	1030	115.2	5.40
Q96A00	Protein phosphatase 1 regulatory subunit 14A OS=Homo sapiens GN=PPP1R14A PE=1 SV=1 - [PP14A_HUMAN]	27.21	1	2	2	10					1.23	1.15	1.14	1.04	147	16.7	9.38
Q5R3F8	Protein phosphatase 1 regulatory subunit 29 OS=Homo sapiens GN=ELFN2 PE=1 SV=1 - [PPR29_HUMAN]	3.05	1	2	2	2	0.51	0.52	0.48	0.49			0.13	0.84	820	89.6	7.62
Q96LQ0	Protein phosphatase 1 regulatory subunit 36 OS=Homo sapiens GN=PPP1R36 PE=1 SV=1 - [PPR36_HUMAN]	4.03	1	1	1	1	4.20	3.44	4.41	3.61					422	49.4	9.36
Q16821	Protein phosphatase 1 regulatory subunit 3A OS=Homo sapiens GN=PPP1R3A PE=1 SV=3 - [PPR3A_HUMAN]	0.98	1	1	1	3	0.50	0.74	0.36	0.54					1122	125.7	5.00
P35813	Protein phosphatase 1A OS=Homo sapiens GN=PPM1A PE=1 SV=1 - [PPM1A_HUMAN]	10.73	2	2	2	3	0.69	0.70	0.68	0.69	0.68				382	42.4	5.36
Q5JR12	Protein phosphatase 1J OS=Homo sapiens GN=PPM1J PE=1 SV=1 - [PPM1J_HUMAN]	6.14	2	2	2	2	1.14	1.22	0.96	1.03					505	54.8	7.46
Q9Y6V0	Protein piccolo OS=Homo sapiens GN=PCLO PE=1 SV=4 - [PCLO_HUMAN]	1.05	1	4	4	6	0.89	1.29	0.87	1.27	0.60	0.33	0.70	0.42	5065	552.9	6.51
Q5THK1	Protein PRR14L OS=Homo sapiens GN=PRR14L PE=1 SV=1 - [PR14L_HUMAN]	0.51	1	1	1	1					0.34	0.39	0.34	0.39	2151	237.2	6.33
P48634	Protein PRRC2A OS=Homo sapiens GN=PRRC2A PE=1 SV=3 - [PRC2A_HUMAN]	0.93	1	1	1	1					2.15	1.96	1.99	1.81	2157	228.7	9.45
Q9Y520	Protein PRRC2C OS=Homo sapiens GN=PRRC2C PE=1 SV=4 - [PRC2C_HUMAN]	1.35	1	3	3	4	1.14	1.13	1.24	1.21	0.72	0.84	0.72	0.84	2896	316.7	9.13
Q8WUY3	Protein prune homolog 2 OS=Homo sapiens GN=PRUNE2 PE=1 SV=3 - [PRUN2_HUMAN]	1.39	1	2	2	3	0.79	0.62	1.13	0.89					3088	340.4	4.45
Q86TP1	Protein prune homolog OS=Homo sapiens GN=PRUNE PE=1 SV=2 - [PRUNE_HUMAN]	12.14	1	2	2	3					0.32	0.40	0.84	1.05	453	50.2	5.50
Q7Z5B4	Protein RIC-3 OS=Homo sapiens GN=RIC3 PE=1 SV=1 - [RIC3_HUMAN]	5.42	1	1	1	1	0.47	0.40	0.52	0.45					369	41.1	5.10
Q9H871	Protein RMD5 homolog A OS=Homo sapiens GN=RMND5A PE=1 SV=1 - [RMD5A_HUMAN]	11.51	1	2	2	4	0.82	0.79	0.92	0.91					391	44.0	6.06
P31949	Protein S100-A11 OS=Homo sapiens GN=S100A11 PE=1 SV=2 - [S10AB_HUMAN]	34.29	1	3	3	14	0.88	0.87	0.81	0.80	1.21	1.11	1.33	1.17	105	11.7	7.12
P80511	Protein S100-A12 OS=Homo sapiens GN=S100A12 PE=1 SV=2 - [S10AC_HUMAN]	39.13	1	4	4	21					2.34	2.32	2.13	2.08	92	10.6	6.25

P26447	Protein S100-A4 OS=Homo sapiens GN=S100A4 PE=1 SV=1 - [S10A4_HUMAN]	16.83	1	2	2	17	0.75	0.82	0.78	0.87	0.85	0.96	0.75	0.82	101	11.7	6.11
P06703	Protein S100-A6 OS=Homo sapiens GN=S100A6 PE=1 SV=1 - [S10A6_HUMAN]	24.44	1	3	3	14	0.60	1.43	0.84	2.00	1.01	1.08	0.92	0.98	90	10.2	5.48
P31151	Protein S100-A7 OS=Homo sapiens GN=S100A7 PE=1 SV=4 - [S10A7_HUMAN]	42.57	2	3	3	23	0.64	0.64	0.75	0.82	1.58	1.43	1.53	1.41	101	11.5	6.77
P05109	Protein S100-A8 OS=Homo sapiens GN=S100A8 PE=1 SV=1 - [S10A8_HUMAN]	80.65	1	10	10	107	0.76	0.65	0.82	0.72	1.47	1.46	1.36	1.37	93	10.8	7.03
P06702	Protein S100-A9 OS=Homo sapiens GN=S100A9 PE=1 SV=1 - [S10A9_HUMAN]	86.84	1	9	9	275	0.65	0.59	0.67	0.57	1.11	1.02	1.12	1.09	114	13.2	6.13
P04271	Protein S100-B OS=Homo sapiens GN=S100B PE=1 SV=2 - [S100B_HUMAN]	16.30	1	1	1	1					1.42	2.66	1.56	2.93	92	10.7	4.59
Q99590	Protein SCAF11 OS=Homo sapiens GN=SCAF11 PE=1 SV=2 - [SCAFB_HUMAN]	2.60	2	2	3	3					0.75	0.73	3.34	3.05	1463	164.6	8.41
Q8N9R8	Protein SCAI OS=Homo sapiens GN=SCAI PE=1 SV=2 - [SCAI_HUMAN]	4.13	1	2	2	3					0.95	0.55	0.93	0.53	606	70.4	8.60
O43819	Protein SCO2 homolog, mitochondrial OS=Homo sapiens GN=SCO2 PE=1 SV=3 - [SCO2_HUMAN]	17.29	1	2	2	2					0.78	0.86	0.85	0.94	266	29.8	8.85
Q5TEA6	Protein sel-1 homolog 2 OS=Homo sapiens GN=SEL1L2 PE=1 SV=2 - [SE1L2_HUMAN]	3.05	1	1	1	7	1.41	1.42	1.32	1.30					688	77.9	9.23
Q68CR1	Protein sel-1 homolog 3 OS=Homo sapiens GN=SEL1L3 PE=1 SV=2 - [SE1L3_HUMAN]	0.80	1	1	1	1	1.24	1.27	1.02	1.05					1132	128.5	7.28
A0PJX4	Protein shisa-3 homolog OS=Homo sapiens GN=SHISA3 PE=2 SV=1 - [SHSA3_HUMAN]	8.40	1	1	1	3	1.14	1.04	1.29	1.19					238	25.8	6.92
A6NL88	Protein shisa-7 OS=Homo sapiens GN=SHISA7 PE=2 SV=3 - [SHSA7_HUMAN]	6.32	1	1	1	2	0.95	0.93	0.94	0.92					538	56.2	10.02
Q8TF72	Protein Shroom3 OS=Homo sapiens GN=SHROOM3 PE=1 SV=2 - [SHRM3_HUMAN]	0.75	1	1	1	1	0.66	0.63	0.60	0.57					1996	216.7	7.80
Q9ULL8	Protein Shroom4 OS=Homo sapiens GN=SHROOM4 PE=1 SV=3 - [SHRM4_HUMAN]	4.02	1	2	2	3	0.71	0.90	0.94	1.22					1493	164.8	6.65
Q58EX2	Protein sidekick-2 OS=Homo sapiens GN=SDK2 PE=1 SV=3 - [SDK2_HUMAN]	2.76	1	3	3	3	1.04	1.06	1.09	1.12					2172	239.2	7.06
Q9Y3B1	Protein slowmo homolog 2 OS=Homo sapiens GN=SLMO2 PE=1 SV=2 - [SLMO2_HUMAN]	13.92	1	1	1	1					1.78	2.22	1.41	1.76	194	21.5	6.73
Q9UPU9	Protein Smaug homolog 1 OS=Homo sapiens GN=SAMD4A PE=1 SV=3 - [SMAG1_HUMAN]	3.06	1	1	1	1					0.57	0.70	0.60	0.74	718	79.4	8.32
Q8ND04	Protein SMG8 OS=Homo sapiens GN=SMG8 PE=1 SV=1 - [SMG8_HUMAN]	6.66	1	2	2	2	0.87	0.93	1.08	1.16					991	109.6	7.68
Q9H0W8	Protein SMG9 OS=Homo sapiens GN=SMG9 PE=1 SV=1 - [SMG9_HUMAN]	4.42	1	1	1	1					1.38	0.83	1.61	0.97	520	57.6	7.01
P18583	Protein SON OS=Homo sapiens GN=SON PE=1 SV=4 - [SON_HUMAN]	0.91	1	1	1	1	0.79	0.66	0.62	0.52					2426	263.7	5.64
Q8IVW8	Protein spinster homolog 2 OS=Homo sapiens GN=SPNS2 PE=1 SV=2 - [SPNS2_HUMAN]	4.01	1	1	1	1	0.93	0.94	1.00	1.01					549	58.0	9.25
Q6ZMD2	Protein spinster homolog 3 OS=Homo sapiens GN=SPNS3 PE=2 SV=2 - [SPNS3_HUMAN]	2.73	1	1	1	1					1.27	0.66	1.24	0.64	512	54.7	6.52
O43609	Protein sprouty homolog 1 OS=Homo sapiens GN=SPRY1 PE=1 SV=2 - [SPY1_HUMAN]	4.70	1	1	1	1					1.12	1.15	0.96	0.99	319	35.1	8.24
O43610	Protein sprouty homolog 3 OS=Homo sapiens GN=SPRY3 PE=1 SV=2 - [SPY3_HUMAN]	6.60	1	1	1	2	0.38	0.53	0.64	0.89					288	31.2	7.40
Q68D10	Protein SPT2 homolog OS=Homo sapiens GN=SPTY2D1 PE=1 SV=3 - [SPT2_HUMAN]	5.99	1	2	2	5	0.93	1.09	1.06	1.25	0.96	1.28	1.04	1.16	685	75.6	9.79
O60225	Protein SSX5 OS=Homo sapiens GN=SSX5 PE=2 SV=3 - [SSX5_HUMAN]	7.45	1	1	1	1					1.18	1.05	1.13	0.99	188	21.6	9.36

A3KN83	Protein strawberry notch homolog 1 OS=Homo sapiens GN=SBNO1 PE=1 SV=1 - [SBNO1_HUMAN]	1.08	1	1	1	2	1.00	0.88	0.87	0.77					1393	154.2	7.88
Q9Y2G9	Protein strawberry notch homolog 2 OS=Homo sapiens GN=SBNO2 PE=2 SV=3 - [SBNO2_HUMAN]	1.32	1	1	1	1	0.99	0.99	0.88	0.89					1366	150.2	6.52
Q5T011	Protein SZT2 OS=Homo sapiens GN=SZT2 PE=2 SV=3 - [SZT2_HUMAN]	0.64	1	1	2	49	0.92	1.62	0.92	1.62					3432	377.8	6.27
Q9C0D5	Protein TANC1 OS=Homo sapiens GN=TANC1 PE=1 SV=3 - [TANC1_HUMAN]	1.24	1	2	2	3	0.85	0.99	0.79	0.93	1.12	1.00	0.72	0.64	1861	202.1	8.32
Q9HCD6	Protein TANC2 OS=Homo sapiens GN=TANC2 PE=1 SV=3 - [TANC2_HUMAN]	1.31	1	1	1	3	0.84	0.73	0.96	0.84					1990	219.5	8.07
Q969Z0	Protein TBRG4 OS=Homo sapiens GN=TBRG4 PE=1 SV=1 - [TBRG4_HUMAN]	4.91	1	1	1	1					0.95	0.92	1.25	1.23	631	70.7	7.42
Q8N1K5	Protein THEMIS OS=Homo sapiens GN=THEMIS PE=1 SV=3 [THMS1_HUMAN]	1.40	1	1	1	1	1.08	1.25	1.42	1.67					641	73.4	5.90
Q86X45	Protein TILB homolog OS=Homo sapiens GN=LRRC6 PE=2 SV=3 - [TILB_HUMAN]	2.79	1	1	1	1	1.17	1.29	1.19	1.32					466	54.2	6.51
Q9UNS1	Protein timeless homolog OS=Homo sapiens GN=TIMELESS PE=1 SV=2 - [TIM_HUMAN]	1.32	1	1	1	2	0.62	0.53	0.71	0.61					1208	138.6	5.40
Q4W5P6	Protein TMEM155 OS=Homo sapiens GN=TMEM155 PE=2 SV=3 - [TM155_HUMAN]	9.23	1	1	1	1	0.89		1.15						130	14.2	10.26
Q15436	Protein transport protein Sec23A OS=Homo sapiens GN=SEC23A PE=1 SV=2 - [SC23A_HUMAN]	1.70	3	3	3	9	1.60	1.53	1.21	1.16	0.90	0.20	1.26	0.43	765	86.1	7.08
O94855	Protein transport protein Sec24D OS=Homo sapiens GN=SEC24D PE=1 SV=2 - [SC24D_HUMAN]	1.74	1	1	1	1					1.06	1.17	1.11	1.23	1032	112.9	7.25
O94979	Protein transport protein Sec31A OS=Homo sapiens GN=SEC31A PE=1 SV=3 - [SC31A_HUMAN]	2.62	1	2	3	6					0.91	0.81	0.95	0.84	1220	132.9	6.89
Q9H9S3	Protein transport protein Sec61 subunit alpha isoform 2 OS=Homo sapiens GN=SEC61A2 PE=2 SV=3 - [S61A2_HUMAN]	2.73	1	1	1	1					0.37	0.54	1.01	1.49	476	52.2	8.06
Q8WU66	Protein TSPEAR OS=Homo sapiens GN=TSPEAR PE=2 SV=2 - [TSEAR_HUMAN]	2.69	1	1	1	1					0.69	0.66	0.65	0.61	669	74.9	6.44
A2A3K4	Protein tyrosine phosphatase domain-containing protein 1 OS=Homo sapiens GN=PTPDC1 PE=2 SV=1 - [PTPC1_HUMAN]	1.59	1	1	1	2					0.95	1.06	3.50	3.90	754	84.4	7.55
Q12974	Protein tyrosine phosphatase type IVA 2 OS=Homo sapiens GN=PTP4A2 PE=1 SV=1 - [TP4A2_HUMAN]	8.38	2	1	1	2					1.30	1.44	1.17	1.29	167	19.1	8.37
Q9UPW8	Protein unc-13 homolog A OS=Homo sapiens GN=UNC13A PE=2 SV=4 - [UN13A_HUMAN]	0.88	1	1	1	1					1.25	1.14	1.47	1.35	1703	192.9	5.35
Q8NB66	Protein unc-13 homolog C OS=Homo sapiens GN=UNC13C PE=1 SV=3 - [UN13C_HUMAN]	0.59	1	1	1	1					0.22	0.68	0.29	0.88	2214	250.8	5.92
Q70J99	Protein unc-13 homolog D OS=Homo sapiens GN=UNC13D PE=1 SV=1 - [UN13D_HUMAN]	2.84	1	2	2	7	0.81	0.68	1.12	0.95	1.02	0.87	0.86	0.74	1090	123.2	6.65
Q9H3U1	Protein unc-45 homolog A OS=Homo sapiens GN=UNC45A PE=1 SV=1 - [UN45A_HUMAN]	2.65	1	1	1	2					1.12	0.81	1.09	0.79	944	103.0	6.07
Q9P2D8	Protein unc-79 homolog OS=Homo sapiens GN=UNC79 PE=2 SV=3 - [UNC79_HUMAN]	2.24	1	3	3	4	0.95	0.56	1.24	1.05					2635	295.1	6.32
Q8N2C7	Protein unc-80 homolog OS=Homo sapiens GN=UNC80 PE=2 SV=2 - [UNC80_HUMAN]	0.74	1	2	2	12	2.37	2.60	2.30	2.52					3258	363.2	6.86
Q76NI1	Protein very KIND OS=Homo sapiens GN=KNDC1 PE=2 SV=2 - [VKIND_HUMAN]	2.06	1	2	2	3	0.86	0.71	1.06	0.88	0.13	0.12	0.37	0.35	1749	191.3	6.16
Q69YN4	Protein virilizer homolog OS=Homo sapiens GN=KIAA1429 PE=1 SV=2 - [VIR_HUMAN]	0.77	1	1	1	1			0.51						1812	201.9	5.01
Q8NEX5	Protein WFDC9 OS=Homo sapiens GN=WFDC9 PE=2 SV=1 - [WFDC9_HUMAN]	5.62	1	1	1	4					1.69	1.77	0.99	2.08	89	10.5	7.55
O95785	Protein Wiz OS=Homo sapiens GN=WIZ PE=1 SV=2 - [WIZ_HUMAN]	0.42	1	1	1	2					1.07	1.10	1.05	1.07	1651	178.6	6.86

P56705	Protein Wnt-4 OS=Homo sapiens GN=WNT4 PE=1 SV=4 - [WNT4_HUMAN]	3.99	1	1	1	1					0.82	0.72	0.81	0.72	351	39.0	8.51
Q5T9L3	Protein wntless homolog OS=Homo sapiens GN=WLS PE=1 SV=2 - [WLS_HUMAN]	9.06	1	2	2	3	0.59	0.38	0.49	0.32	0.91	0.87	0.82	0.78	541	62.2	7.36
Q9ULE0	Protein WWC3 OS=Homo sapiens GN=WWC3 PE=2 SV=3 - [WWC3_HUMAN]	1.65	2	1	2	3					0.49	0.83	0.90	1.19	1092	122.6	6.37
O75695	Protein XRP2 OS=Homo sapiens GN=RP2 PE=1 SV=4 - [XRP2_HUMAN]	5.71	1	1	1	1							1.18	0.13	350	39.6	5.12
Q9GZM5	Protein YIPF3 OS=Homo sapiens GN=YIPF3 PE=1 SV=1 - [YIPF3_HUMAN]	2.86	1	1	1	3					1.30	1.21	1.24	1.15	350	38.2	5.76
Q9UK55	Protein Z-dependent protease inhibitor OS=Homo sapiens GN=SERPINA10 PE=1 SV=1 - [ZPI_HUMAN]	54.05	1	20	20	89	1.07	1.10	1.05	1.05	1.05	1.05	1.02	1.00	444	50.7	8.27
Q75MW2	Protein ZNF767 OS=Homo sapiens GN=ZNF767 PE=2 SV=1 - [ZN767_HUMAN]	7.10	1	1	1	1	1.43	1.18	1.16	0.96					155	17.2	9.64
Q9C0D3	Protein zyg-11 homolog B OS=Homo sapiens GN=ZYG11B PE=1 SV=2 - [ZY11B_HUMAN]	1.88	1	1	1	2	0.99	0.90	1.03	0.94					744	83.9	6.86
Q9Y2J8	Protein-arginine deiminase type-2 OS=Homo sapiens GN=PADI2 PE=1 SV=2 - [PADI2_HUMAN]	2.11	1	1	1	7	1.16	1.04	0.99	1.15					665	75.5	5.59
Q9ULW8	Protein-arginine deiminase type-3 OS=Homo sapiens GN=PADI3 PE=1 SV=2 - [PADI3_HUMAN]	2.11	1	1	1	1	0.97	0.98	1.01	1.03					664	74.7	5.54
Q9UM07	Protein-arginine deiminase type-4 OS=Homo sapiens GN=PADI4 PE=1 SV=2 - [PADI4_HUMAN]	2.56	1	1	1	1	1.07	1.05	1.09	1.07					663	74.0	6.58
P49221	Protein-glutamine gamma-glutamyltransferase 4 OS=Homo sapiens GN=TGM4 PE=1 SV=2 - [TGM4_HUMAN]	1.02	1	1	1	6	0.80	0.77	0.84	0.85	0.78	0.83	0.80	0.85	684	77.1	6.76
Q08188	Protein-glutamine gamma-glutamyltransferase E OS=Homo sapiens GN=TGM3 PE=1 SV=4 - [TGM3_HUMAN]	3.90	1	1	1	1	1.55		0.57						693	76.6	5.86
Q96PF1	Protein-glutamine gamma-glutamyltransferase Z OS=Homo sapiens GN=TGM7 PE=2 SV=1 - [TGM7_HUMAN]	5.21	1	1	1	1					1.30	1.55	1.42	1.69	710	79.9	7.01
P28300	Protein-lysine 6-oxidase OS=Homo sapiens GN=LOX PE=1 SV=2 - [LYOX_HUMAN]	10.07	1	2	2	5					0.86	0.77	0.98	0.88	417	46.9	8.09
Q8TDZ2	Protein-methionine sulfoxide oxidase MICAL1 OS=Homo sapiens GN=MICAL1 PE=1 SV=2 - [MICA1_HUMAN]	2.25	1	2	2	5	0.56	0.56	0.45	0.46	0.86	0.73	0.92	0.78	1067	117.8	6.40
O94851	Protein-methionine sulfoxide oxidase MICAL2 OS=Homo sapiens GN=MICAL2 PE=1 SV=1 - [MICA2_HUMAN]	3.56	2	4	4	9	3.45	1.80	1.17	0.89	0.75	1.33	0.89	1.58	1124	126.6	8.65
Q9Y2Y8	Proteoglycan 3 OS=Homo sapiens GN=PRG3 PE=1 SV=2 - [PRG3_HUMAN]	3.56	1	1	1	6	1.24	1.12	1.60	1.43	1.07	1.18	0.83	0.92	225	25.4	4.81
Q92954	Proteoglycan 4 OS=Homo sapiens GN=PRG4 PE=1 SV=2 - [PRG4_HUMAN]	17.45	1	26	26	140	1.15	1.18	1.14	1.22	1.19	1.08	1.15	1.03	1404	151.0	9.50
Q04941	Proteolipid protein 2 OS=Homo sapiens GN=PLP2 PE=1 SV=1 - [PLP2_HUMAN]	10.53	1	1	1	1					1.66	1.46	1.90	1.68	152	16.7	7.24
P00734	Prothrombin OS=Homo sapiens GN=F2 PE=1 SV=2 - [THRB_HUMAN]	62.54	1	41	41	2372	1.10	1.07	1.09	1.07	1.17	1.18	1.17	1.18	622	70.0	5.90
Q04864	Proto-oncogene c-Rel OS=Homo sapiens GN=REL PE=1 SV=1 - [REL_HUMAN]	2.26	1	1	1	1					1.06	0.99	1.33	1.25	619	68.5	5.86
P10911	Proto-oncogene DBL OS=Homo sapiens GN=MCF2 PE=1 SV=3 - [MCF2_HUMAN]	5.84	1	3	3	3	1.10	1.00	1.07	0.98	1.18	1.03	0.90	0.79	925	107.6	6.01
P00540	Proto-oncogene serine/threonine-protein kinase mos OS=Homo sapiens GN=MOS PE=2 SV=1 - [MOS_HUMAN]	2.60	1	1	1	2	1.21	1.14	1.21	1.14					346	37.8	8.91
P07949	Proto-oncogene tyrosine-protein kinase receptor Ret OS=Homo sapiens GN=RET PE=1 SV=3 - [RET_HUMAN]	0.90	1	1	1	1					0.21	0.87	0.65	2.63	1114	124.2	6.60
P12931	Proto-oncogene tyrosine-protein kinase Src OS=Homo sapiens GN=SRC PE=1 SV=3 - [SRC_HUMAN]	6.34	1	2	2	2	1.17	1.45	1.21	1.52	0.97	1.09	0.94	1.05	536	59.8	7.42
Q9Y5I2	Protocadherin alpha-10 OS=Homo sapiens GN=PCDHA10 PE=2 SV=1 - [PCDAA_HUMAN]	1.16	1	1	1	1					1.66	1.42	1.44	1.23	948	102.8	5.17

Q9Y5I1	Protocadherin alpha-11 OS=Homo sapiens GN=PCDHA11 PE=2 SV=1 - [PCDAB_HUMAN]	1.69	1	1	1	1	0.67	0.75	1.13	1.26					949	103.2	5.36
Q9Y5H7	Protocadherin alpha-5 OS=Homo sapiens GN=PCDHA5 PE=2 SV=1 - [PCDA5_HUMAN]	4.91	1	2	2	4					1.09	0.95	0.91	0.79	936	102.0	5.43
Q9H158	Protocadherin alpha-C1 OS=Homo sapiens GN=PCDHAC1 PE=2 SV=2 - [PCDC1_HUMAN]	2.39	1	1	1	1					1.04	0.89	0.76	0.65	963	103.9	5.21
Q9Y5F1	Protocadherin beta-12 OS=Homo sapiens GN=PCDHB12 PE=2 SV=1 - [PCDBC_HUMAN]	2.77	1	1	1	3	1.02	0.78	0.97	0.76					795	86.7	4.84
Q9NRJ7	Protocadherin beta-16 OS=Homo sapiens GN=PCDHB16 PE=1 SV=3 - [PCDBG_HUMAN]	1.42	1	1	1	1	1.41	1.36	1.53	1.49					776	84.9	5.11
Q9Y5E7	Protocadherin beta-2 OS=Homo sapiens GN=PCDHB2 PE=1 SV=1 - [PCDB2_HUMAN]	2.51	1	2	2	5	0.85	0.84	0.85	0.84	0.81	0.82	0.85	0.87	798	87.2	4.89
Q14517	Protocadherin Fat 1 OS=Homo sapiens GN=FAT1 PE=1 SV=2 - [FAT1_HUMAN]	1.24	1	2	2	5	1.10	1.01	1.14	1.05					4588	506.0	5.00
Q9NYQ8	Protocadherin Fat 2 OS=Homo sapiens GN=FAT2 PE=1 SV=2 - [FAT2_HUMAN]	0.99	1	2	2	5	1.44	1.47	1.38	1.41	4.25	3.89	4.22	3.86	4349	479.0	5.16
Q8TDW7	Protocadherin Fat 3 OS=Homo sapiens GN=FAT3 PE=2 SV=2 - [FAT3_HUMAN]	2.83	1	6	6	14	1.39	1.39	1.45	1.45	1.67	1.52	1.52	1.37	4589	505.2	4.87
Q6V0I7	Protocadherin Fat 4 OS=Homo sapiens GN=FAT4 PE=1 SV=2 - [FAT4_HUMAN]	0.44	3	1	2	4	0.59	0.85	0.64	0.93					4981	542.4	4.94
Q9Y5H4	Protocadherin gamma-A1 OS=Homo sapiens GN=PCDHGA1 PE=2 SV=1 - [PCDG1_HUMAN]	1.18	1	1	1	1					0.96	0.99	0.95	0.97	931	101.2	5.03
Q9Y5H1	Protocadherin gamma-A2 OS=Homo sapiens GN=PCDHGA2 PE=2 SV=1 - [PCDG2_HUMAN]	6.55	1	3	3	3	0.10	0.07	0.29	0.21	0.07	0.11	0.86	1.40	932	101.4	5.01
Q9Y5H0	Protocadherin gamma-A3 OS=Homo sapiens GN=PCDHGA3 PE=2 SV=2 - [PCDG3_HUMAN]	2.04	1	1	1	1	0.78	0.91	0.91	1.07					932	100.9	5.00
Q9BZA7	Protocadherin-11 X-linked OS=Homo sapiens GN=PCDH11X PE=2 SV=1 - [PC11X_HUMAN]	2.30	2	2	2	3					0.93	1.02	0.95	1.03	1347	147.5	5.21
Q96QU1	Protocadherin-15 OS=Homo sapiens GN=PCDH15 PE=1 SV=2 - [PCD15_HUMAN]	0.66	1	1	1	1					1.27	1.19	1.29	1.21	1955	215.9	5.07
O14917	Protocadherin-17 OS=Homo sapiens GN=PCDH17 PE=2 SV=2 - [PCD17_HUMAN]	1.98	1	1	1	2	2.12	2.91	1.91	2.63					1159	126.2	5.19
Q9HCL0	Protocadherin-18 OS=Homo sapiens GN=PCDH18 PE=2 SV=3 - [PCD18_HUMAN]	3.70	1	3	3	3	1.49	1.11	1.40	1.05	0.68	0.76	0.70	0.80	1135	126.1	5.15
Q8N6Y1	Protocadherin-20 OS=Homo sapiens GN=PCDH20 PE=2 SV=1 - [PCD20_HUMAN]	2.71	1	1	1	1	0.78	1.03	0.88	1.16					924	101.9	4.94
O60245	Protocadherin-7 OS=Homo sapiens GN=PCDH7 PE=1 SV=2 - [PCDH7_HUMAN]	4.77	1	2	3	3	0.64	0.29	0.69	0.31					1069	116.0	5.19
O95206	Protocadherin-8 OS=Homo sapiens GN=PCDH8 PE=2 SV=2 - [PCDH8_HUMAN]	2.24	1	1	1	1	1.20	1.30	1.27	1.39					1070	112.9	5.54
Q9HC56	Protocadherin-9 OS=Homo sapiens GN=PCDH9 PE=1 SV=2 - [PCDH9_HUMAN]	0.73	1	1	1	1	0.84	0.94	0.84	0.94					1237	136.0	5.47
Q2VWP7	Protogenin OS=Homo sapiens GN=PRTG PE=2 SV=1 - [PRTG_HUMAN]	2.43	1	1	1	1					0.78	0.82	0.80	0.84	1150	127.0	7.59
Q7Z2H8	Proton-coupled amino acid transporter 1 OS=Homo sapiens GN=SLC36A1 PE=1 SV=1 - [S36A1_HUMAN]	5.88	1	1	1	5	0.96	1.00	0.93	0.97					476	53.0	6.99
Q96NT5	Proton-coupled folate transporter OS=Homo sapiens GN=SLC46A1 PE=1 SV=1 - [PCFT_HUMAN]	3.05	1	1	1	3					0.67	0.74	0.53	0.58	459	49.7	8.81
Q8IWL1	Pulmonary surfactant-associated protein A2 OS=Homo sapiens GN=SFTPA2 PE=1 SV=1 - [SFPA2_HUMAN]	5.24	2	1	1	2	1.40	1.27	1.37	1.25					248	26.2	5.17
P07988	Pulmonary surfactant-associated protein B OS=Homo sapiens GN=SFTPB PE=1 SV=3 - [PSPB_HUMAN]	18.90	1	5	5	12	1.82	1.80	1.57	1.39	0.82	0.95	0.93	1.07	381	42.1	5.49
Q86U38	Pumilio domain-containing protein C14orf21 OS=Homo sapiens GN=C14orf21 PE=1 SV=1 - [CN021_HUMAN]	3.14	1	1	2	2	1.45	1.51	1.38	1.44					636	69.4	7.28

Q8TB72	Pumilio homolog 2 OS=Homo sapiens GN=PUM2 PE=1 SV=2 [PUM2_HUMAN]	2.06	1	1	1	1	3.24	5.19	1.38	2.21					1066	114.1	7.08
P00491	Purine nucleoside phosphorylase OS=Homo sapiens GN=PNP PE=1 SV=2 - [PNPH_HUMAN]	3.11	1	1	1	2	0.52	0.58	0.58	0.66					289	32.1	6.95
P55786	Puromycin-sensitive aminopeptidase OS=Homo sapiens GN=NPEPPS PE=1 SV=2 - [PSA_HUMAN]	3.48	1	2	2	9	0.83	0.69	0.85	0.71	1.26	1.46	0.98	1.26	919	103.2	5.72
Q6A1A2	Putative 3-phosphoinositide-dependent protein kinase 2 OS=Homo sapiens GN=PDPK2 PE=5 SV=1 - [PDPK2_HUMAN]	4.04	2	1	1	1					1.11	1.14	1.04	1.07	396	44.7	8.37
P20848	Putative alpha-1-antitrypsin-related protein OS=Homo sapiens GN=SERPINA2 PE=5 SV=1 - [A1ATR_HUMAN]	3.33	1	1	2	147	1.75	1.56	1.70	1.50	0.79	0.70	0.82	0.72	420	47.9	7.90
Q6NSI1	Putative ankyrin repeat domain-containing protein 26-like protein OS=Homo sapiens GN=ANKRD26P1 PE=5 SV=2 - [AR26L_HUMAN]	5.92	1	1	1	1	0.75	0.24	0.69	0.22					321	35.4	5.39
A6NMY6	Putative annexin A2-like protein OS=Homo sapiens GN=ANXA2P2 PE=5 SV=2 - [AXA2L_HUMAN]	14.75	2	4	4	7	0.70	0.73	0.53	0.56	1.20	1.16	1.22	1.19	339	38.6	6.95
A6NL99	Putative aquaporin-7-like protein 3 OS=Homo sapiens GN=AQP7P3 PE=5 SV=2 - [AQP73_HUMAN]	7.31	1	1	1	1	0.95	0.78	0.85	0.70					342	37.3	8.00
Q16740	Putative ATP-dependent Clp protease proteolytic subunit, mitochondrial OS=Homo sapiens GN=CLPP PE=1 SV=1 - [CLPP_HUMAN]	10.11	1	1	2	3							0.92	0.55	277	30.2	8.09
Q7L2E3	Putative ATP-dependent RNA helicase DHX30 OS=Homo sapiens GN=DHX30 PE=1 SV=1 - [DHX30_HUMAN]	3.02	1	2	3	7	0.97	0.82	1.20	1.02					1194	133.9	8.78
Q9H6R0	Putative ATP-dependent RNA helicase DHX33 OS=Homo sapiens GN=DHX33 PE=1 SV=2 - [DHX33_HUMAN]	6.08	1	2	2	9					0.87	1.54	0.73	1.30	707	78.8	8.91
Q8NDG6	Putative ATP-dependent RNA helicase TDRD9 OS=Homo sapiens GN=TDRD9 PE=2 SV=3 - [TDRD9_HUMAN]	1.66	1	2	2	4					1.13	1.03	1.16	1.13	1382	155.6	7.05
Q15486	Putative beta-glucuronidase-like protein SMA3 OS=Homo sapiens GN=GUSBP1 PE=5 SV=3 - [GUSP1_HUMAN]	9.29	1	1	1	1					0.13	0.65	0.33	1.60	140	15.4	6.14
Q5H9B9	Putative BMP-2-inducible kinase-like protein OS=Homo sapiens GN=BMP2KL PE=5 SV=2 - [BM2KL_HUMAN]	3.65	1	1	1	2	1.41	0.82	1.50	0.87					411	46.1	5.63
Q6WQI6	Putative cancer susceptibility gene HEPN1 protein OS=Homo sapiens GN=HEPN1 PE=5 SV=2 - [HEPN1_HUMAN]	23.86	1	1	1	1	0.87	0.96	0.95	1.06					88	10.3	8.28
A6NM15	Putative COBW domain-containing protein 7 OS=Homo sapiens GN=CBWD7 PE=5 SV=3 - [CBWD7_HUMAN]	7.29	6	2	2	2	0.77	0.96	0.75	0.93					247	28.2	5.52
Q6ZSU1	Putative cytochrome P450 2G1-like protein OS=Homo sapiens PE=5 SV=1 - [C2G1L_HUMAN]	13.70	1	1	1	1					0.98	0.99	1.34	1.36	146	16.7	6.77
P0C838	Putative DAP-2 like protein C8orf68 OS=Homo sapiens GN=C8orf68 PE=5 SV=1 - [CH068_HUMAN]	21.43	1	1	1	1					0.78	0.76	0.85	0.83	84	9.2	8.95
Q6P1N9	Putative deoxyribonuclease TATDN1 OS=Homo sapiens GN=TATDN1 PE=1 SV=2 - [TATD1_HUMAN]	10.10	1	2	3	5	1.15	1.14	1.05	1.04	0.97	0.99	0.82	0.83	297	33.6	6.96
Q6NVV9	Putative disintegrin and metalloproteinase domain-containing protein 5 OS=Homo sapiens GN=ADAM5P PE=5 SV=2 - [ADAM5_HUMAN]	1.70	1	1	1	1	0.63	0.59	0.61	0.58					412	47.2	6.80
Q5T890	Putative DNA repair and recombination protein RAD26-like OS=Homo sapiens GN=RAD26L PE=1 SV=1 - [RAD26_HUMAN]	1.83	1	1	1	1					0.05	0.02	0.26	0.13	712	81.0	9.01
Q9H9P5	Putative E3 ubiquitin-protein ligase UNKL OS=Homo sapiens GN=UNKL PE=1 SV=3 - [UNKL_HUMAN]	6.03	1	1	1	1	1.10	0.85	1.48	1.14					680	73.8	8.06
Q58FF3	Putative endoplasmin-like protein OS=Homo sapiens GN=HSP90B2P PE=5 SV=1 - [ENPLL_HUMAN]	4.01	1	1	2	3	1.50	1.16	1.52	1.19					399	45.8	5.26
A0PJM3	Putative exonuclease GOR-like protein OS=Homo sapiens GN=REXO1L2P PE=5 SV=2 - [GORL_HUMAN]	1.72	2	1	1	1					1.15	0.66	0.87	0.50	583	64.2	9.23
Q8NGA4	Putative G-protein coupled receptor GPCR39 OS=Homo sapiens PE=5 SV=2 - [GPC39_HUMAN]	5.88	1	1	1	16					1.14	0.97	1.10	1.03	272	30.3	9.26
A6NGU5	Putative gamma-glutamyltranspeptidase 3 OS=Homo sapiens GN=GGT3P PE=5 SV=2 - [GGT3_HUMAN]	1.41	2	1	1	1	0.98	0.54	0.89	0.50					568	61.5	7.14
Q8WZA8	Putative gastric cancer-related gene 224 protein OS=Homo sapiens GN=GCRG224 PE=5 SV=1 - [GC224_HUMAN]	45.71	1	1	1	1	0.67	1.16	0.86	1.49					35	3.8	8.22

A8MS94	Putative golgin subfamily A member 2-like protein 5 OS=Homo sapiens PE=5 SV=2 - [GG2L5_HUMAN]	8.18	1	1	1	1	2.09	2.13	1.90	1.94					110	12.0	5.68
A6NFL8	Putative golgin subfamily A member 6-like protein 5 OS=Homo sapiens PE=5 SV=3 - [GG6L5_HUMAN]	3.99	1	1	1	1	1.12	1.12	1.49	1.49					401	47.6	4.96
A6NDN3	Putative golgin subfamily A member 6B OS=Homo sapiens GN=GOLGA6B PE=5 SV=3 - [GOG6B_HUMAN]	3.32	2	1	1	1					0.88	0.79	0.80	0.72	693	79.9	5.50
A6NN73	Putative golgin subfamily A member 8C OS=Homo sapiens GN=GOLGA8C PE=5 SV=2 - [GOG8C_HUMAN]	4.19	1	1	1	1	1.07	0.42	1.22	0.48					597	67.1	8.54
O43824	Putative GTP-binding protein 6 OS=Homo sapiens GN=GTPBP6 PE=2 SV=3 - [GTPB6_HUMAN]	2.91	1	1	1	1	1.04	1.52	0.83	1.21					516	56.8	9.42
Q14568	Putative heat shock protein HSP 90-alpha A2 OS=Homo sapiens GN=HSP90AA2 PE=1 SV=2 - [HS902_HUMAN]	7.87	3	3	4	4	0.86	1.03	0.94	1.14					343	39.3	4.65
Q58FG1	Putative heat shock protein HSP 90-alpha A4 OS=Homo sapiens GN=HSP90AA4P PE=5 SV=1 - [HS904_HUMAN]	4.31	1	1	1	1	1.37	1.41	1.42	1.47					418	47.7	5.19
Q9HCE1	Putative helicase MOV-10 OS=Homo sapiens GN=MOV10 PE=1 SV=2 - [MOV10_HUMAN]	1.50	1	1	1	2					0.71	0.83	0.75	0.87	1003	113.6	8.82
Q9BXT6	Putative helicase Mov10l1 OS=Homo sapiens GN=MOV10L1 PE=1 SV=1 - [M10L1_HUMAN]	3.14	1	2	2	2					2.09	3.21	0.77	1.19	1211	135.2	6.46
Q8N3S3	Putative homeodomain transcription factor 2 OS=Homo sapiens GN=PHTF2 PE=2 SV=2 - [PHTF2_HUMAN]	3.69	1	2	2	2	0.82	1.07	0.79	1.04	0.78	0.72	0.98	0.90	785	88.7	8.90
Q2TVT3	Putative keratinocyte growth factor-like protein 2 OS=Homo sapiens GN=KGFLP2 PE=5 SV=1 - [KGFL2_HUMAN]	13.40	3	2	2	3	1.24	1.16	1.16	1.08	1.04	0.97	1.15	1.08	97	11.0	8.60
Q5VSP4	Putative lipocalin 1-like protein 1 OS=Homo sapiens GN=LCN1P1 PE=5 SV=1 - [LC1L1_HUMAN]	6.79	2	1	1	3					1.46	0.87	1.02	0.61	162	17.9	5.00
Q8NE18	Putative methyltransferase NSUN7 OS=Homo sapiens GN=NSUN7 PE=2 SV=4 - [NSUN7_HUMAN]	2.37	1	1	1	1					1.20	1.24	1.09	1.13	718	81.0	8.75
A8MVU1	Putative neutrophil cytosol factor 1C OS=Homo sapiens GN=NCF1C PE=5 SV=1 - [NCF1C_HUMAN]	4.10	3	1	1	1	1.17	1.07	1.30	1.20					366	41.8	8.84
Q3KNT7	Putative NOL1/NOP2/Sun domain family member 5B OS=Homo sapiens GN=NSUN5P1 PE=5 SV=1 - [NSN5B_HUMAN]	11.66	1	1	1	1					0.42	4.71	0.41	4.58	163	17.7	5.73
Q8NGY7	Putative olfactory receptor 10J6 OS=Homo sapiens GN=OR10J6P PE=5 SV=1 - [O10J6_HUMAN]	5.07	1	1	1	2					0.87	0.85	0.61	0.59	276	31.0	8.53
Q8NHC6	Putative olfactory receptor 14L1 OS=Homo sapiens GN=OR14L1P PE=5 SV=1 - [O14L1_HUMAN]	5.19	1	1	1	1					0.18	0.27	0.98	1.46	308	34.7	8.40
Q8NGU4	Putative olfactory receptor 2I1 OS=Homo sapiens GN=OR2I1P PE=5 SV=1 - [OR2I1_HUMAN]	8.86	1	1	1	6			0.62	0.21					316	34.1	9.44
Q8NGA2	Putative olfactory receptor 7A2 OS=Homo sapiens GN=OR7A2P PE=5 SV=1 - [OR7A2_HUMAN]	5.81	1	1	1	1	1.05	1.12	0.93	1.00					310	34.4	6.89
Q49A26	Putative oxidoreductase GLYR1 OS=Homo sapiens GN=GLYR1 PE=1 SV=3 - [GLYR1_HUMAN]	5.97	7	1	2	4	0.83	0.51	1.35	0.83					553	60.5	9.17
A8MUH7	Putative PDZ domain-containing protein 1P OS=Homo sapiens GN=PDZK1P1 PE=5 SV=2 - [PDZ1P_HUMAN]	2.99	2	1	1	1	0.89	1.01	0.87	0.99					402	44.0	5.63
Q6GMV3	Putative peptidyl-tRNA hydrolase PTRHD1 OS=Homo sapiens GN=PTRHD1 PE=1 SV=1 - [PTRD1_HUMAN]	21.43	1	1	1	1	3.00	2.74	1.04	0.96					140	15.8	9.10
A2A3N6	Putative PIP5K1A and PSMD4-like protein OS=Homo sapiens GN=PIPSL PE=5 SV=1 - [PIPSL_HUMAN]	3.02	1	1	1	1					1.17	1.22	1.23	1.28	862	95.0	5.71
Q69YJ1	Putative pleckstrin homology domain-containing family M member 1P OS=Homo sapiens GN=PLEKHM1P PE=5 SV=1 - [PKHMP_HUMAN]	2.88	2	1	1	2	0.96	0.64	0.91	0.61	0.31	0.35	0.33	0.38	520	58.9	6.33
Q8IXJ9	Putative Polycomb group protein ASXL1 OS=Homo sapiens GN=ASXL1 PE=1 SV=3 - [ASXL1_HUMAN]	2.53	1	1	1	2	1.22	1.28	1.19	1.25					1541	165.3	6.21
Q9C0F0	Putative Polycomb group protein ASXL3 OS=Homo sapiens GN=ASXL3 PE=2 SV=3 - [ASXL3_HUMAN]	2.05	1	2	2	3					1.58	1.45	1.38	1.29	2248	241.8	6.14
Q6IS24	Putative polypeptide N-acetylgalactosaminyltransferase-like protein 3 OS=Homo sapiens GN=WBSCR17 PE=2 SV=2 - [GLTL3_HUMAN]	5.18	1	2	2	3	1.10	1.32	1.17	1.42					598	67.7	8.90

A6NI47	Putative POTE ankyrin domain family member M OS=Homo sapiens GN=POTEM PE=3 SV=2 - [POTEM_HUMAN]	9.45	2	2	2	6	1.12	1.34	1.01	1.20	1.22	1.14	1.18	1.10	508	57.0	7.06
A6NMC2	Putative PRAME family member 24 OS=Homo sapiens GN=PRAMEF24 PE=3 SV=1 - [PRA24_HUMAN]	2.74	3	1	1	1	0.82	0.97	0.82	0.98					474	53.6	6.52
Q7L7V1	Putative pre-mRNA-splicing factor ATP-dependent RNA helicase DHX32 OS=Homo sapiens GN=DHX32 PE=1 SV=1 - [DHX32_HUMAN]	2.02	1	1	1	1					1.05	0.81	1.00	0.77	743	84.4	4.97
Q13046	Putative pregnancy-specific beta-1-glycoprotein 7 OS=Homo sapiens GN=PSG7 PE=5 SV=2 - [PSG7_HUMAN]	30.31	1	4	11	124	3.77	4.88	3.57	5.06	6.49	5.60	5.54	4.78	419	47.0	8.44
A6NC97	Putative protein FAM172B OS=Homo sapiens GN=FAM172BP PE=5 SV=3 - [F172B_HUMAN]	2.49	1	1	1	1					1.37		3.51		362	41.9	8.43
A8MTS5	Putative protein FAM177A2 OS=Homo sapiens GN=FAM177A2 PE=5 SV=1 - [F1772_HUMAN]	5.88	1	1	1	2	0.98	1.08	0.90	1.00					136	15.3	4.53
A6NL58	Putative protein FAM86A-like 2 OS=Homo sapiens PE=5 SV=3 - [F86A2_HUMAN]	5.47	2	1	1	1	0.75	0.55	0.86	0.63					329	36.6	6.58
A6NK25	Putative protein FAM86A-like 3 OS=Homo sapiens PE=5 SV=2 - [F86A3_HUMAN]	10.53	1	3	3	4	1.15	0.84	1.28	0.94	1.20	0.12	1.29	0.13	285	31.6	6.37
A6NIG7	Putative protein FAM86B-like 1 OS=Homo sapiens PE=3 SV=3 - [F86B3_HUMAN]	5.42	1	1	1	2	0.76	0.73	0.91	0.89					295	32.8	6.32
B8ZZ34	Putative protein shisa-8 OS=Homo sapiens GN=SHISA8 PE=5 SV=2 - [SHSA8_HUMAN]	1.83	1	1	1	2					0.76	0.68	0.66	0.59	492	51.4	10.83
Q8N8H1	Putative protein ZNF321 OS=Homo sapiens GN=ZNF321P PE=5 SV=3 - [ZN321_HUMAN]	7.93	1	1	1	1					2.45	2.54	2.19	2.27	164	19.0	7.01
A8MT19	Putative rhophilin-2-like protein OS=Homo sapiens PE=5 SV=2 - [RHN2L_HUMAN]	2.74	1	1	1	1	1.03	0.96	0.99	0.93					583	65.9	6.98
Q96IC2	Putative RNA exonuclease NEF-sp OS=Homo sapiens GN=44M2.3 PE=2 SV=1 - [REXON_HUMAN]	4.39	1	2	2	3	0.94	1.02	0.93	1.01	0.86	0.94	1.07	1.17	774	86.8	8.32
A6NG73	Putative RNA polymerase II subunit A C-terminal domain phosphatase SSU72-like protein 3 OS=Homo sapiens PE=3 SV=2 - [S72L3_HUMAN]	12.89	3	2	2	2	0.67	0.71	0.72	0.77	0.98	0.78	0.89	0.70	194	22.5	5.60
Q8IXW5	Putative RNA polymerase II subunit B1 CTD phosphatase RPAP2 OS=Homo sapiens GN=RPAP2 PE=1 SV=1 - [RPAP2_HUMAN]	4.90	1	2	2	3	1.29	0.85	1.13	0.88					612	69.5	7.78
Q96T37	Putative RNA-binding protein 15 OS=Homo sapiens GN=RBM15 PE=1 SV=2 - [RBM15_HUMAN]	1.23	1	1	1	1					0.94	1.06	0.83	0.94	977	107.1	10.08
Q8NDT2	Putative RNA-binding protein 15B OS=Homo sapiens GN=RBM15B PE=1 SV=3 - [RB15B_HUMAN]	2.25	1	1	1	1	1.32	1.21	1.84	1.69					890	97.1	9.85
Q9Y383	Putative RNA-binding protein Luc7-like 2 OS=Homo sapiens GN=LUC7L2 PE=1 SV=2 - [LC7L2_HUMAN]	4.59	1	1	1	1					1.63	1.72	1.57	1.65	392	46.5	10.01
A8MVG2	Putative selection and upkeep of intraepithelial T-cells protein 1 homolog OS=Homo sapiens GN=SKINT1 PE=5 SV=2 - [SKIT1_HUMAN]	5.05	1	1	1	6	1.48	1.12	1.36	1.04	0.49	0.78	0.42	0.66	218	25.4	5.87
Q6UXR4	Putative serpin A13 OS=Homo sapiens GN=SERPINA13 PE=5 SV=1 - [SPA13_HUMAN]	12.70	1	3	3	3					0.82	0.96	0.81	0.95	307	34.8	6.40
P0C7V7	Putative signal peptidase complex catalytic subunit SEC11B OS=Homo sapiens GN=SEC11B PE=5 SV=1 - [SC11B_HUMAN]	4.82	1	1	1	1	1.04	0.95	1.03	0.94					166	19.1	9.66
Q9BZL3	Putative small membrane protein NID67 OS=Homo sapiens GN=NID67 PE=2 SV=1 - [NID67_HUMAN]	18.33	1	1	1	2					0.99	0.92	1.39	1.30	60	6.6	7.34
Q6ZMV5	Putative SMEK homolog 3 OS=Homo sapiens GN=SMEK3P PE=5 SV=2 - [SMEK3_HUMAN]	1.56	1	1	1	1					0.74	0.81	0.78	0.85	832	95.7	4.82
Q9HBR0	Putative sodium-coupled neutral amino acid transporter 10 OS=Homo sapiens GN=SLC38A10 PE=1 SV=2 - [S38AA_HUMAN]	3.04	1	2	2	3	1.12	1.18	1.13	1.19	0.74	0.54	0.83	0.61	1119	119.7	5.73
Q495Y7	Putative speedy protein E7 OS=Homo sapiens GN=SPDYE7P PE=5 SV=1 - [SPDE7_HUMAN]	4.81	7	1	1	1					1.44	1.64	1.25	1.42	208	25.2	8.44
P0C672	Putative tetraspanin-19 OS=Homo sapiens GN=TSPAN19 PE=5 SV=1 - [TSN19_HUMAN]	4.84	1	1	1	1					0.74	0.75	0.64	0.65	248	28.4	7.64
Q9UF72	Putative TP73 antisense gene protein 1 OS=Homo sapiens GN=TP73-AS1 PE=5 SV=1 - [T73AS_HUMAN]	10.45	1	1	1	5	1.42	1.24	1.43	1.29					201	20.6	6.42

A6NLI5	Putative tripartite motif-containing protein 64C OS=Homo sapiens GN=TRIM64C PE=5 SV=3 - [TR64C_HUMAN]	4.70	1	1	1	2	0.43	0.50	0.54	0.62					447	51.3	5.52
A6NL28	Putative tropomyosin alpha-3 chain-like protein OS=Homo sapiens PE=5 SV=2 - [TPM3L_HUMAN]	10.31	1	1	2	10					3.03	2.28	2.97	2.24	223	26.3	4.51
Q8NHM4	Putative trypsin-6 OS=Homo sapiens GN=TRY6 PE=5 SV=1 - [TRY6_HUMAN]	20.24	1	1	2	45					0.83	1.09	0.76	0.99	247	26.5	6.27
O14990	Putative type-1 protein phosphatase inhibitor 4 OS=Homo sapiens GN=PPP1R2P9 PE=5 SV=1 - [IPP4_HUMAN]	14.85	1	2	2	2	0.91	1.08	0.92	1.09					202	22.6	5.12
O75061	Putative tyrosine-protein phosphatase auxilin OS=Homo sapiens GN=DNAJC6 PE=1 SV=3 - [AUXI_HUMAN]	2.19	1	1	1	1	1.03	1.14	0.94	1.06					913	99.9	7.25
Q6UWK7	Putative uncharacterized protein C10orf99 OS=Homo sapiens GN=C10orf99 PE=2 SV=1 - [CJ099_HUMAN]	13.58	1	1	1	2					1.79	1.75	0.80	0.79	81	9.2	10.49
Q8WZ69	Putative uncharacterized protein C11orf40 OS=Homo sapiens GN=C11orf40 PE=2 SV=1 - [CK040_HUMAN]	11.52	1	1	1	1			1.08						217	24.6	7.69
Q52M58	Putative uncharacterized protein C14orf177 OS=Homo sapiens GN=C14orf177 PE=2 SV=1 - [CN177_HUMAN]	15.20	1	1	1	1	1.27	1.26	1.23	1.23					125	13.9	8.91
Q8NBB2	Putative uncharacterized protein C15orf37 OS=Homo sapiens GN=C15orf37 PE=2 SV=1 - [CO037_HUMAN]	23.08	1	1	1	1					0.52		0.87		130	13.9	11.82
Q8N910	Putative uncharacterized protein C15orf56 OS=Homo sapiens GN=C15orf56 PE=2 SV=1 - [CO056_HUMAN]	14.29	1	1	1	1	1.19	1.29	0.95	1.03					161	17.0	11.88
Q6ZSH3	Putative uncharacterized protein C16orf85 OS=Homo sapiens GN=C16orf85 PE=2 SV=1 - [CP085_HUMAN]	4.76	1	1	1	1					0.99	0.79	0.74	0.59	147	15.9	8.66
Q5JT78	Putative uncharacterized protein C1orf137 OS=Homo sapiens GN=C1orf137 PE=2 SV=1 - [CA137_HUMAN]	6.12	1	1	1	2					0.77	0.81	0.74	0.77	98	11.0	7.72
Q5T0J3	Putative uncharacterized protein C1orf220 OS=Homo sapiens GN=C1orf220 PE=2 SV=1 - [CA220_HUMAN]	6.72	1	1	1	1					0.74	0.79	0.73	0.78	134	15.1	9.86
Q9NV39	Putative uncharacterized protein C22orf26 OS=Homo sapiens GN=C22orf26 PE=2 SV=1 - [CV026_HUMAN]	10.14	1	1	1	1	1.29	1.28	1.31	1.31					138	14.7	12.41
Q96BT1	Putative uncharacterized protein C3orf49 OS=Homo sapiens GN=C3orf49 PE=2 SV=1 - [CC049_HUMAN]	2.05	1	1	1	1	1.13	1.14	1.17	1.20					292	33.4	10.18
Q5JQF7	Putative uncharacterized protein C6orf100 OS=Homo sapiens GN=C6orf100 PE=4 SV=2 - [CF100_HUMAN]	32.26	1	1	1	1					0.32	0.71	0.49	1.08	62	7.3	11.22
Q5TEZ4	Putative uncharacterized protein C6orf164 OS=Homo sapiens GN=C6orf164 PE=2 SV=2 - [CF164_HUMAN]	7.89	1	1	1	2	0.76	0.54	0.76	0.54					76	8.4	8.60
Q5T699	Putative uncharacterized protein C6orf183 OS=Homo sapiens GN=C6orf183 PE=5 SV=2 - [CF183_HUMAN]	2.53	1	1	1	1					0.26	0.24	0.51	0.47	396	45.7	5.62
Q9BWJ2	Putative uncharacterized protein C8orf51 OS=Homo sapiens GN=C8orf51 PE=5 SV=1 - [CH051_HUMAN]	10.17	1	1	1	1					0.71	0.88	0.84	1.05	59	6.7	6.51
Q9HA58	Putative uncharacterized protein C8orf60 OS=Homo sapiens GN=C8orf60 PE=5 SV=1 - [CH060_HUMAN]	21.25	1	2	2	2	0.93	0.40	0.92	0.39					160	16.9	8.22
Q0IIN9	Putative uncharacterized protein C8orf77 OS=Homo sapiens GN=C8orf77 PE=5 SV=1 - [CH077_HUMAN]	5.21	1	1	1	3					1.00	0.77	0.92	0.73	211	22.5	8.37
A6NHY6	Putative uncharacterized protein C9orf118 OS=Homo sapiens GN=C9orf118 PE=4 SV=2 - [CI118_HUMAN]	37.68	1	1	1	3	0.98		0.94						69	7.9	8.18
A6PW82	Putative uncharacterized protein CXorf30 OS=Homo sapiens GN=CXorf30 PE=2 SV=2 - [CX030_HUMAN]	1.11	1	1	1	1					0.56	1.02	0.51	0.93	633	72.0	6.09
Q8ND99	Putative uncharacterized protein DKFZp434G1729 OS=Homo sapiens PE=5 SV=2 - [YS038_HUMAN]	2.82	1	1	1	1					0.58	0.69	0.80	0.95	567	60.1	9.72
Q6XXX2	Putative uncharacterized protein encoded by LINC00114 OS=Homo sapiens GN=LINC00114 PE=5 SV=1 - [CU024_HUMAN]	2.86	1	1	1	1					0.71	0.35	0.48	0.24	140	15.7	9.61
Q8N2A0	Putative uncharacterized protein encoded by LINC00269 OS=Homo sapiens GN=LINC00269 PE=5 SV=1 - [CX062_HUMAN]	8.05	1	1	1	2					1.40	1.25	0.93	0.83	174	18.3	7.23
Q8N616	Putative uncharacterized protein encoded by LINC00311 OS=Homo sapiens GN=LINC00311 PE=5 SV=1 - [TM148_HUMAN]	4.20	1	1	1	2					0.73	0.85	0.95	1.11	119	12.9	7.09

Q8IVM7	Putative uncharacterized protein encoded by LINC00346 OS=Homo sapiens GN=LINC00346 PE=5 SV=1 - [CM029_HUMAN]	12.80	1	2	2	6	1.24	1.01	1.26	1.04	1.02	1.13	1.00	1.10	164	18.1	9.00
Q8WZB0	Putative uncharacterized protein encoded by LINC00476 OS=Homo sapiens GN=LINC00476 PE=5 SV=1 - [CI130_HUMAN]	11.76	1	1	1	1	0.80	0.62	0.70	0.55					136	14.7	11.93
Q8N0U6	Putative uncharacterized protein encoded by LINC00518 OS=Homo sapiens GN=LINC00518 PE=2 SV=1 - [CF218_HUMAN]	22.03	1	1	1	1					1.15	0.81			118	13.4	5.10
Q96LP4	Putative uncharacterized protein FLJ25328 OS=Homo sapiens PE=2 SV=1 - [YS017_HUMAN]	4.39	1	1	1	1	0.97	1.35	0.85	1.18					205	22.0	8.50
Q8NA96	Putative uncharacterized protein FLJ35723 OS=Homo sapiens PE=5 SV=2 - [YE027_HUMAN]	30.00	1	2	2	2	1.07	1.17	1.09	1.20					180	19.6	8.59
A4D1N5	Putative uncharacterized protein FLJ40288 OS=Homo sapiens PE=2 SV=1 - [YG018_HUMAN]	4.67	1	1	1	1					0.57	1.42	0.59	1.46	150	16.7	8.06
Q6ZW54	Putative uncharacterized protein FLJ41591 OS=Homo sapiens PE=2 SV=1 - [YQ046_HUMAN]	6.08	1	1	1	1					0.57	0.28	0.72	0.36	148	15.9	9.61
Q6ZVN7	Putative uncharacterized protein FLJ42280 OS=Homo sapiens PE=2 SV=2 - [YG008_HUMAN]	19.53	1	1	1	1	1.10	0.83	1.16	0.88					128	14.1	9.55
Q6ZSV7	Putative uncharacterized protein FLJ45177 OS=Homo sapiens PE=2 SV=1 - [YF010_HUMAN]	19.63	1	1	1	1	1.01	0.92	1.00	0.93					163	16.9	8.41
Q6ZSR3	Putative uncharacterized protein FLJ45275, mitochondrial OS=Homo sapiens PE=5 SV=1 - [YO027_HUMAN]	14.88	1	1	1	1	0.49	0.60	0.56	0.70					168	17.9	8.41
Q6ZRU5	Putative uncharacterized protein FLJ46089 OS=Homo sapiens PE=5 SV=2 - [YQ032_HUMAN]	6.76	1	1	1	1					0.50	0.44	0.65	0.57	148	16.9	9.55
Q5TG08	Putative uncharacterized protein LOC388900 OS=Homo sapiens PE=1 SV=2 - [YV009_HUMAN]	1.70	1	1	1	1					1.16	1.44	0.58	0.72	646	74.9	9.25
Q0VD67	Putative uncharacterized protein LOC440356 OS=Homo sapiens PE=5 SV=1 - [YP032_HUMAN]	7.23	1	1	1	1					1.45	1.36	1.54	1.43	83	9.0	8.31
Q9UI72	Putative uncharacterized protein PRO0255 OS=Homo sapiens GN=PRO0255 PE=5 SV=1 - [YE014_HUMAN]	34.78	1	2	2	2	0.68	0.74	0.73	0.79					69	8.2	9.14
Q8N0V1	Putative uncharacterized protein ZNF295-AS1 OS=Homo sapiens GN=ZNF295-AS1 PE=5 SV=2 - [ZNAS1_HUMAN]	13.14	1	1	1	4	0.10	0.22	0.14	0.21					137	15.0	8.68
A8MVJ9	Putative UPF0609 protein C4orf27-like OS=Homo sapiens PE=1 SV=1 - [YI028_HUMAN]	2.59	1	2	2	14	0.73	0.63	0.71	0.62	1.11	0.99	1.01	1.00	347	39.7	8.10
A6NJ16	Putative V-set and immunoglobulin domain-containing-like protein IGHV4OR15-8 OS=Homo sapiens GN=IGHV4OR15-8 PE=5 SV=2 - [IV4F8_HUMAN]	16.26	2	3	4	17	1.03	1.09	1.03	1.08	1.45	1.50	1.21	1.25	123	13.6	9.60
P0CG00	Putative zinc finger and SCAN domain-containing protein 5D OS=Homo sapiens GN=ZSCAN5D PE=5 SV=1 - [ZSA5D_HUMAN]	5.84	1	1	1	1					0.98	1.06	1.01	1.10	497	56.2	8.63
B1APH4	Putative zinc finger protein 487 OS=Homo sapiens GN=ZNF487P PE=5 SV=3 - [ZN487_HUMAN]	2.23	1	1	1	1					1.36	1.93	1.19	1.69	448	51.6	7.85
A8MUZ8	Putative zinc finger protein 705G OS=Homo sapiens GN=ZNF705G PE=2 SV=2 - [Z705G_HUMAN]	7.33	1	1	1	2	0.66	0.65	0.58	0.57					300	34.7	9.22
A8MSQ6	Putative zinc finger protein 725 OS=Homo sapiens GN=ZNF725 PE=4 SV=2 - [ZN725_HUMAN]	7.25	1	1	1	2	1.24	1.09	1.40	1.23					138	15.8	8.88
Q15940	Putative zinc finger protein 726P1 OS=Homo sapiens GN=ZNF726P1 PE=5 SV=2 - [ZNF67_HUMAN]	3.11	1	1	1	2					1.06	0.85	1.07	0.86	193	23.0	9.80
P0C7V5	Putative zinc finger protein 812 OS=Homo sapiens GN=ZNF812 PE=5 SV=1 - [ZN812_HUMAN]	3.74	1	1	1	4	0.54	0.82	0.57	0.54					454	51.5	8.38
Q96N64	PWWP domain-containing protein 2A OS=Homo sapiens GN=PWWP2A PE=1 SV=2 - [PWP2A_HUMAN]	1.06	1	1	1	5					0.80	1.12	0.84	1.17	755	81.9	9.00
Q6NUJ5	PWWP domain-containing protein 2B OS=Homo sapiens GN=PWWP2B PE=1 SV=3 - [PWP2B_HUMAN]	1.36	1	1	1	1					1.57	1.66	1.04	1.10	590	63.9	8.21
Q5H9M0	PWWP domain-containing protein MUM1L1 OS=Homo sapiens GN=MUM1L1 PE=2 SV=1 - [MUML1_HUMAN]	1.58	1	1	1	1					0.85	0.98	0.80	0.92	696	79.0	4.97
Q8TCD6	Pyridoxal phosphate phosphatase PHOSPHO2 OS=Homo sapiens GN=PHOSPHO2 PE=1 SV=1 - [PHOP2_HUMAN]	10.79	1	1	1	5	1.25	0.67	1.25	0.67	0.73	0.97	0.66	0.83	241	27.8	6.79

Q8WXC3	Pyrin domain-containing protein 1 OS=Homo sapiens GN=PYDC1 PE=1 SV=1 - [PYDC1_HUMAN]	5.62	1	1	1	1					1.11	1.71	1.10	1.70	89	10.1	6.79
O15553	Pyrin OS=Homo sapiens GN=MEFV PE=1 SV=1 - [MEFV_HUMAN]	2.18	1	1	1	1					1.46	1.51	0.73	0.76	781	86.4	8.03
Q9NXJ5	Pyroglutamyl-peptidase 1 OS=Homo sapiens GN=PGPEP1 PE=1 SV=1 - [PGPI_HUMAN]	7.66	1	1	1	1	0.85	0.71	0.86	0.72					209	23.1	5.90
Q96C36	Pyrroline-5-carboxylate reductase 2 OS=Homo sapiens GN=PYCR2 PE=1 SV=1 - [P5CR2_HUMAN]	5.31	1	1	1	1	1.39	1.15	1.36	1.14					320	33.6	7.77
O00330	Pyruvate dehydrogenase protein X component, mitochondrial OS=Homo sapiens GN=PDHX PE=1 SV=3 - [ODPX_HUMAN]	2.79	1	1	1	1	1.18	1.19	1.01	1.04					501	54.1	8.66
P14618	Pyruvate kinase isozymes M1/M2 OS=Homo sapiens GN=PKM2 PE=1 SV=4 - [KPYM_HUMAN]	27.50	1	10	11	48	0.81	0.85	0.80	0.82	0.93	0.92	1.05	1.02	531	57.9	7.84
P30613	Pyruvate kinase isozymes R/L OS=Homo sapiens GN=PKLR PE=1 SV=2 - [KPYR_HUMAN]	6.27	1	1	2	5	0.99	0.84	0.90	0.78					574	61.8	7.74
Q2I0M5	R-spondin-4 OS=Homo sapiens GN=RSPO4 PE=1 SV=2 - [RSPO4_HUMAN]	2.56	1	1	1	4	0.39	0.57	0.47	0.72	0.85	1.34	0.68	1.06	234	26.2	9.07
P50395	Rab GDP dissociation inhibitor beta OS=Homo sapiens GN=GDI2 PE=1 SV=2 - [GDIB_HUMAN]	2.47	1	1	1	1	0.88	0.93	0.73	0.77					445	50.6	6.47
Q5R372	Rab GTPase-activating protein 1-like OS=Homo sapiens GN=RABGAP1L PE=1 SV=1 - [RBG1L_HUMAN]	1.35	1	1	1	4	0.90	0.98	0.86	0.94	1.20	1.22	1.09	1.11	815	92.5	5.31
Q15276	Rab GTPase-binding effector protein 1 OS=Homo sapiens GN=RABEP1 PE=1 SV=2 - [RABE1_HUMAN]	4.18	2	3	4	20	0.88	1.19	0.84	1.14	1.23	1.31	1.15	1.23	862	99.2	5.01
Q3YEC7	Rab-like protein 1 OS=Homo sapiens GN=PARF PE=1 SV=2 - [RBEL1_HUMAN]	5.21	1	1	1	2	0.91	0.99	1.02	1.13					729	79.5	5.22
Q6WKZ4	Rab11 family-interacting protein 1 OS=Homo sapiens GN=RAB11FIP1 PE=1 SV=2 - [RFIP1_HUMAN]	2.96	2	3	3	3	0.37	0.35	0.58	0.55					1283	137.1	5.47
Q86YS3	Rab11 family-interacting protein 4 OS=Homo sapiens GN=RAB11FIP4 PE=1 SV=1 - [RFIP4_HUMAN]	1.57	1	1	1	1	1.80	1.71	2.09	1.99					637	71.9	4.87
P31751	RAC-beta serine/threonine-protein kinase OS=Homo sapiens GN=AKT2 PE=1 SV=2 - [AKT2_HUMAN]	4.78	1	2	2	2	0.56	0.91	0.60	0.99					481	55.7	6.37
Q6NUQ1	RAD50-interacting protein 1 OS=Homo sapiens GN=RINT1 PE=1 SV=1 - [RINT1_HUMAN]	2.02	1	1	1	2	0.67	0.67	0.62	0.63					792	90.6	5.45
Q52LD8	Raftlin-2 OS=Homo sapiens GN=RFTN2 PE=1 SV=3 - [RFTN2_HUMAN]	4.19	1	1	1	2	1.68	1.71	1.60	1.64					501	55.9	5.83
Q2PPJ7	Ral GTPase-activating protein subunit alpha-2 OS=Homo sapiens GN=RALGAPA2 PE=1 SV=2 - [RGPA2_HUMAN]	2.51	1	3	3	5	0.62	0.58	0.63	0.60	0.81	0.64	0.89	0.63	1873	210.6	6.07
Q12967	Ral guanine nucleotide dissociation stimulator OS=Homo sapiens GN=RALGDS PE=1 SV=2 - [GNDS_HUMAN]	1.75	1	1	1	1					1.01	0.82	1.08	0.88	914	100.5	5.72
Q96D71	RalBP1-associated Eps domain-containing protein 1 OS=Homo sapiens GN=REPS1 PE=1 SV=3 - [REPS1_HUMAN]	2.01	1	1	1	1	0.20	0.07	0.84	0.28					796	86.6	5.69
P46060	Ran GTPase-activating protein 1 OS=Homo sapiens GN=RANGAP1 PE=1 SV=1 - [RAGP1_HUMAN]	2.73	1	1	1	2	1.07	0.65	0.74	0.45					587	63.5	4.68
Q9H2T7	Ran-binding protein 17 OS=Homo sapiens GN=RANBP17 PE=2 SV=1 - [RBP17_HUMAN]	1.01	1	1	1	3	1.29	1.20	1.38	1.35					1088	124.3	6.44
O60518	Ran-binding protein 6 OS=Homo sapiens GN=RANBP6 PE=1 SV=2 - [RNBP6_HUMAN]	2.17	1	1	1	3	0.54	0.33	0.67	0.42					1105	124.6	5.01
P43487	Ran-specific GTPase-activating protein OS=Homo sapiens GN=RANBP1 PE=1 SV=1 - [RANG_HUMAN]	5.47	1	1	1	2					1.90	1.60	1.97	1.65	201	23.3	5.29
A6NKT7	RanBP2-like and GRIP domain-containing protein 3 OS=Homo sapiens GN=RGPD3 PE=2 SV=2 - [RGPD3_HUMAN]	1.76	6	1	3	3					0.82	0.67	0.84	0.69	1758	197.4	6.33
Q13905	Rap guanine nucleotide exchange factor 1 OS=Homo sapiens GN=RAPGEF1 PE=1 SV=3 - [RPGF1_HUMAN]	0.93	1	1	1	2					0.95	1.17	0.67	0.83	1077	120.5	5.92
O95398	Rap guanine nucleotide exchange factor 3 OS=Homo sapiens GN=RAPGEF3 PE=1 SV=6 - [RPGF3_HUMAN]	3.03	1	1	1	1	0.58	0.66	0.60	0.68					923	103.7	7.56

Q8TEU7	Rap guanine nucleotide exchange factor 6 OS=Homo sapiens GN=RAPGEF6 PE=1 SV=2 - [RPGF6_HUMAN]	3.87	2	4	5	12					1.00	0.74	1.01	0.71	1601	179.3	6.40
Q6R327	Rapamycin-insensitive companion of mTOR OS=Homo sapiens GN=RICTOR PE=1 SV=1 - [RICTR_HUMAN]	3.40	1	3	3	3	0.91	0.84	0.88	0.82					1708	192.1	7.47
Q13671	Ras and Rab interactor 1 OS=Homo sapiens GN=RIN1 PE=1 SV=4 - [RIN1_HUMAN]	2.55	1	1	1	1	1.31	1.30	0.82	0.82					783	84.0	8.02
P50749	Ras association domain-containing protein 2 OS=Homo sapiens GN=RASSF2 PE=1 SV=1 - [RASF2_HUMAN]	3.99	1	1	1	3	1.18	1.49	1.02	1.28					326	37.8	8.84
Q6ZTQ3	Ras association domain-containing protein 6 OS=Homo sapiens GN=RASSF6 PE=1 SV=1 - [RASF6_HUMAN]	2.17	1	1	1	1					0.98	0.28	0.94	0.26	369	43.4	8.78
Q02833	Ras association domain-containing protein 7 OS=Homo sapiens GN=RASSF7 PE=2 SV=1 - [RASF7_HUMAN]	2.68	1	1	1	2					0.99	1.21	0.88	1.08	373	39.9	5.62
P20936	Ras GTPase-activating protein 1 OS=Homo sapiens GN=RASA1 PE=1 SV=1 - [RASA1_HUMAN]	0.76	1	1	1	1					1.65	1.27	1.50	1.16	1047	116.3	6.54
Q14644	Ras GTPase-activating protein 3 OS=Homo sapiens GN=RASA3 PE=1 SV=3 - [RASA3_HUMAN]	2.04	1	1	1	1					1.60	1.50	1.51	1.41	834	95.6	7.15
Q9UJF2	Ras GTPase-activating protein nGAP OS=Homo sapiens GN=RASAL2 PE=1 SV=2 - [NGAP_HUMAN]	1.23	1	1	1	1					0.20	1.87	0.46	4.35	1139	128.5	8.24
Q13576	Ras GTPase-activating-like protein IQGAP2 OS=Homo sapiens GN=IQGAP2 PE=1 SV=4 - [IQGA2_HUMAN]	2.41	1	3	3	3	0.39	0.39	0.51	0.50	0.46	0.40	1.05	0.78	1575	180.5	5.64
Q86VI3	Ras GTPase-activating-like protein IQGAP3 OS=Homo sapiens GN=IQGAP3 PE=1 SV=2 - [IQGA3_HUMAN]	3.19	1	3	4	23	0.73	0.84	0.75	0.83	0.62	0.70	0.56	0.63	1631	184.6	7.65
Q7LDG7	RAS guanyl-releasing protein 2 OS=Homo sapiens GN=RASGRP2 PE=1 SV=1 - [GRP2_HUMAN]	9.03	1	3	3	8	0.72	0.38	0.74	0.39	1.61	1.59	1.64	1.42	609	69.2	7.80
Q86YV0	RAS protein activator like-3 OS=Homo sapiens GN=RASAL3 PE=1 SV=2 - [RASL3_HUMAN]	0.89	1	1	1	2	1.41	0.91	1.44	0.93	1.24	1.08	1.21	1.06	1011	111.8	8.75
Q15404	Ras suppressor protein 1 OS=Homo sapiens GN=RSU1 PE=1 SV=3 - [RSU1_HUMAN]	29.60	1	5	5	9	0.60	0.54	0.62	0.60					277	31.5	8.65
Q96JH8	Ras-associating and dilute domain-containing protein OS=Homo sapiens GN=RADIL PE=1 SV=5 - [RADIL_HUMAN]	3.07	1	1	1	1	1.22	1.17	1.48	1.42					1075	117.4	7.09
P15153	Ras-related C3 botulinum toxin substrate 2 OS=Homo sapiens GN=RAC2 PE=1 SV=1 - [RAC2_HUMAN]	20.83	6	3	4	7	1.12	0.84	1.08	0.88	0.96	0.96	0.93	0.93	192	21.4	7.61
Q9HB90	Ras-related GTP-binding protein C OS=Homo sapiens GN=RRAGC PE=1 SV=1 - [RRAGC_HUMAN]	3.51	2	1	1	1					1.41	1.16	1.42	1.17	399	44.2	5.10
P61026	Ras-related protein Rab-10 OS=Homo sapiens GN=RAB10 PE=1 SV=1 - [RAB10_HUMAN]	14.00	2	1	3	6	0.88	0.94	0.78	0.85					200	22.5	8.38
P62491	Ras-related protein Rab-11A OS=Homo sapiens GN=RAB11A PE=1 SV=3 - [RB11A_HUMAN]	20.37	2	4	4	13	0.99	0.90	0.92	0.85	1.52	1.51	1.50	1.41	216	24.4	6.57
P51153	Ras-related protein Rab-13 OS=Homo sapiens GN=RAB13 PE=1 SV=1 - [RAB13_HUMAN]	17.73	2	2	3	6					0.91	0.86	0.93	1.00	203	22.8	9.19
P61106	Ras-related protein Rab-14 OS=Homo sapiens GN=RAB14 PE=1 SV=4 - [RAB14_HUMAN]	20.93	1	3	3	19	0.84	0.85	0.86	0.88	1.46	1.47	1.40	1.54	215	23.9	6.21
Q9NP72	Ras-related protein Rab-18 OS=Homo sapiens GN=RAB18 PE=1 SV=1 - [RAB18_HUMAN]	6.80	1	1	2	4					5.08	5.01	4.72	4.65	206	23.0	5.24
A4D1S5	Ras-related protein Rab-19 OS=Homo sapiens GN=RAB19 PE=2 SV=2 - [RAB19_HUMAN]	5.07	1	1	1	1	1.23	1.13	1.19	1.10					217	24.4	6.52
Q9H0U4	Ras-related protein Rab-1B OS=Homo sapiens GN=RAB1B PE=1 SV=1 - [RAB1B_HUMAN]	33.33	4	4	5	16	0.90	0.74	0.84	0.75	1.14	1.05	1.27	1.10	201	22.2	5.73
Q9NX57	Ras-related protein Rab-20 OS=Homo sapiens GN=RAB20 PE=1 SV=1 - [RAB20_HUMAN]	5.13	1	1	1	1	0.57	0.61	0.90	0.98					234	26.3	6.55
Q9UL25	Ras-related protein Rab-21 OS=Homo sapiens GN=RAB21 PE=1 SV=3 - [RAB21_HUMAN]	4.89	1	1	1	6	0.78	0.82	0.93	0.98	1.00	0.95	1.16	1.10	225	24.3	7.94
O00194	Ras-related protein Rab-27B OS=Homo sapiens GN=RAB27B PE=1 SV=4 - [RB27B_HUMAN]	27.06	2	5	5	60	0.83	0.93	0.60	0.62	1.77	1.65	1.48	1.45	218	24.6	5.52

P61019	Ras-related protein Rab-2A OS=Homo sapiens GN=RAB2A PE=1 SV=1 - [RAB2A_HUMAN]	7.55	1	1	1	1					1.17	1.17	1.08	1.08	212	23.5	6.54
Q9BZG1	Ras-related protein Rab-34 OS=Homo sapiens GN=RAB34 PE=1 SV=1 - [RAB34_HUMAN]	2.70	1	1	1	1					0.75	0.67	0.88	0.78	259	29.0	7.88
Q15286	Ras-related protein Rab-35 OS=Homo sapiens GN=RAB35 PE=1 SV=1 - [RAB35_HUMAN]	18.91	2	1	3	6					0.83	0.86	0.97	1.01	201	23.0	8.29
P20336	Ras-related protein Rab-3A OS=Homo sapiens GN=RAB3A PE=1 SV=1 - [RAB3A_HUMAN]	15.91	2	2	2	2	0.93	0.84	1.01	0.91					220	25.0	5.03
Q7Z6P3	Ras-related protein Rab-44 OS=Homo sapiens GN=RAB44 PE=3 SV=3 - [RAB44_HUMAN]	2.35	1	1	1	1					0.70	1.05	0.72	1.08	723	77.6	4.92
P20339	Ras-related protein Rab-5A OS=Homo sapiens GN=RAB5A PE=1 SV=2 - [RAB5A_HUMAN]	11.63	2	1	2	16	1.00	1.18	0.88	1.06	0.83	0.90	0.74	0.81	215	23.6	8.15
P61020	Ras-related protein Rab-5B OS=Homo sapiens GN=RAB5B PE=1 SV=1 - [RAB5B_HUMAN]	12.09	2	1	2	5							0.91		215	23.7	8.13
P51148	Ras-related protein Rab-5C OS=Homo sapiens GN=RAB5C PE=1 SV=2 - [RAB5C_HUMAN]	14.35	1	2	2	3	0.86	0.72	0.93	0.78					216	23.5	8.41
P51149	Ras-related protein Rab-7a OS=Homo sapiens GN=RAB7A PE=1 SV=1 - [RAB7A_HUMAN]	35.27	1	6	6	14	0.94	0.93	0.93	0.93	1.09	1.11	1.10	1.09	207	23.5	6.70
P61006	Ras-related protein Rab-8A OS=Homo sapiens GN=RAB8A PE=1 SV=1 - [RAB8A_HUMAN]	14.98	2	1	3	10	0.68	0.63	0.74	0.70	1.06	1.19	0.97	1.09	207	23.7	9.07
Q92930	Ras-related protein Rab-8B OS=Homo sapiens GN=RAB8B PE=1 SV=2 - [RAB8B_HUMAN]	21.74	2	2	4	7	0.79	0.86	0.81	0.88	1.16	0.85	1.15	0.84	207	23.6	9.07
Q9NP90	Ras-related protein Rab-9B OS=Homo sapiens GN=RAB9B PE=1 SV=1 - [RAB9B_HUMAN]	12.44	1	1	1	2	1.02	1.00	1.03	1.02	0.73	0.85	0.62	0.72	201	22.7	4.87
P61224	Ras-related protein Rap-1b OS=Homo sapiens GN=RAP1B PE=1 SV=1 - [RAP1B_HUMAN]	63.59	3	8	8	46	0.82	0.71	0.93	0.78	0.86	0.87	1.00	0.86	184	20.8	5.78
P10114	Ras-related protein Rap-2a OS=Homo sapiens GN=RAP2A PE=1 SV=1 - [RAP2A_HUMAN]	8.74	1	1	1	1					0.54	0.81	0.48	0.71	183	20.6	4.82
Q86X27	Ras-specific guanine nucleotide-releasing factor RalGPS2 OS=Homo sapiens GN=RALGPS2 PE=1 SV=1 - [RGPS2_HUMAN]	3.43	1	1	1	1					0.68	0.35	0.59	0.31	583	65.1	8.73
O95294	RasGAP-activating-like protein 1 OS=Homo sapiens GN=RASAL1 PE=1 SV=3 - [RASL1_HUMAN]	1.24	1	1	1	2					0.59	0.77	0.62	0.81	804	90.0	6.51
Q8TDY2	RB1-inducible coiled-coil protein 1 OS=Homo sapiens GN=RB1CC1 PE=1 SV=3 - [RBCC1_HUMAN]	3.26	4	3	4	6	1.37	1.00	1.56	1.15	0.95	0.93	0.96	0.94	1594	183.0	5.41
Q9BRK0	Receptor expression-enhancing protein 2 OS=Homo sapiens GN=REEP2 PE=2 SV=2 - [REEP2_HUMAN]	8.33	1	1	1	1					1.05	1.15	0.96	1.05	252	28.2	9.54
Q00765	Receptor expression-enhancing protein 5 OS=Homo sapiens GN=REEP5 PE=1 SV=3 - [REEP5_HUMAN]	11.11	1	1	1	1					0.91	0.61	1.33	0.89	189	21.5	8.10
P21860	Receptor tyrosine-protein kinase erbB-3 OS=Homo sapiens GN=ERBB3 PE=1 SV=1 - [ERBB3_HUMAN]	1.64	1	1	1	1	0.78	1.08	0.94	1.30					1342	148.0	6.55
Q15303	Receptor tyrosine-protein kinase erbB-4 OS=Homo sapiens GN=ERBB4 PE=1 SV=1 - [ERBB4_HUMAN]	1.30	1	2	2	2					0.98	0.70	0.93	0.66	1308	146.7	6.39
Q13546	Receptor-interacting serine/threonine-protein kinase 1 OS=Homo sapiens GN=RIPK1 PE=1 SV=3 - [RIPK1_HUMAN]	2.24	1	1	1	3	1.05	0.83	1.10	0.69					671	75.9	6.33
P57078	Receptor-interacting serine/threonine-protein kinase 4 OS=Homo sapiens GN=RIPK4 PE=1 SV=1 - [RIPK4_HUMAN]	2.76	1	1	1	1					1.42	1.52	1.04	1.11	832	91.6	7.17
P23467	Receptor-type tyrosine-protein phosphatase beta OS=Homo sapiens GN=PTPRB PE=1 SV=3 - [PTPRB_HUMAN]	1.30	1	1	1	1					1.55	1.06	1.86	1.26	1997	224.2	7.61
P23468	Receptor-type tyrosine-protein phosphatase delta OS=Homo sapiens GN=PTPRD PE=1 SV=2 - [PTPRD_HUMAN]	1.31	1	2	2	3	1.10	1.04	1.22	1.16	0.74	0.80	0.73	0.78	1912	214.6	6.57
P23469	Receptor-type tyrosine-protein phosphatase epsilon OS=Homo sapiens GN=PTPRE PE=1 SV=1 - [PTPRE_HUMAN]	1.57	1	1	1	1	0.89	0.94	0.94	1.00					700	80.6	7.02
Q12913	Receptor-type tyrosine-protein phosphatase eta OS=Homo sapiens GN=PTPRJ PE=1 SV=3 - [PTPRJ_HUMAN]	8.45	1	9	9	29	0.95	0.98	0.98	0.92	0.95	0.94	0.98	0.97	1337	145.9	5.58

P10586	Receptor-type tyrosine-protein phosphatase F OS=Homo sapiens GN=PTPRF PE=1 SV=2 - [PTPRF_HUMAN]	5.09	1	8	8	16	0.98	1.06	0.93	1.01	0.99	1.03	0.95	1.00	1907	212.7	6.30
P23470	Receptor-type tyrosine-protein phosphatase gamma OS=Homo sapiens GN=PTPRG PE=1 SV=4 - [PTPRG_HUMAN]	4.71	1	5	5	11	0.88	0.88	0.78	0.80	1.29	1.27	1.35	1.34	1445	161.9	6.42
P28827	Receptor-type tyrosine-protein phosphatase mu OS=Homo sapiens GN=PTPRM PE=1 SV=2 - [PTPRM_HUMAN]	1.10	1	2	2	3	0.87	0.92	0.88	0.93	0.93	0.97	1.01	1.04	1452	163.6	6.65
Q92932	Receptor-type tyrosine-protein phosphatase N2 OS=Homo sapiens GN=PTPRN2 PE=1 SV=2 - [PTPR2_HUMAN]	6.40	1	2	2	6	0.95	0.95	0.67	0.71					1015	111.2	5.83
Q13332	Receptor-type tyrosine-protein phosphatase S OS=Homo sapiens GN=PTPRS PE=1 SV=3 - [PTPRS_HUMAN]	5.34	1	6	6	13	1.20	1.15	1.13	1.11					1948	216.9	6.46
O14522	Receptor-type tyrosine-protein phosphatase T OS=Homo sapiens GN=PTPRT PE=1 SV=6 - [PTPRT_HUMAN]	1.60	1	1	1	3					0.98	1.31	1.28	1.29	1441	162.0	6.89
Q92729	Receptor-type tyrosine-protein phosphatase U OS=Homo sapiens GN=PTPRU PE=1 SV=2 - [PTPRU_HUMAN]	1.73	1	1	1	1					0.84	1.06	0.42	0.53	1446	162.3	6.92
Q9UBG7	Recombining binding protein suppressor of hairless-like protein OS=Homo sapiens GN=RBPJL PE=1 SV=3 - [RBPJL_HUMAN]	1.74	1	1	1	2					1.18	0.99	0.98	0.82	517	56.7	7.72
Q9BRX8	Redox-regulatory protein FAM213A OS=Homo sapiens GN=FAM213A PE=1 SV=3 - [F213A_HUMAN]	3.06	1	1	1	2	0.18	0.33	0.22	0.41					229	25.7	8.84
P78509	Reelin OS=Homo sapiens GN=RELN PE=1 SV=3 - [RELN_HUMAN]	2.54	1	5	6	9	0.99	0.94	1.12	0.99					3460	388.1	5.88
Q86UR5	Regulating synaptic membrane exocytosis protein 1 OS=Homo sapiens GN=RIMS1 PE=1 SV=1 - [RIMS1_HUMAN]	2.78	1	3	3	5	0.85	0.98	0.63	0.80	3.48	3.67	3.25	3.42	1692	189.0	9.66
Q9NQG5	Regulation of nuclear pre-mRNA domain-containing protein 1B OS=Homo sapiens GN=RPRD1B PE=1 SV=1 - [RPR1B_HUMAN]	2.45	1	1	1	1	0.77	0.82	0.76	0.82					326	36.9	5.97
O43665	Regulator of G-protein signaling 10 OS=Homo sapiens GN=RGS10 PE=1 SV=2 - [RGS10_HUMAN]	26.01	1	3	4	8					1.06	1.12	1.05	1.11	173	20.2	5.49
O14924	Regulator of G-protein signaling 12 OS=Homo sapiens GN=RGS12 PE=1 SV=1 - [RGS12_HUMAN]	2.63	1	3	3	3					0.94	0.70	0.84	0.62	1447	156.3	7.44
P41220	Regulator of G-protein signaling 2 OS=Homo sapiens GN=RGS2 PE=1 SV=1 - [RGS2_HUMAN]	5.21	1	1	1	2					1.28	1.22	1.93	1.84	211	24.4	8.91
Q8NE09	Regulator of G-protein signaling 22 OS=Homo sapiens GN=RGS22 PE=2 SV=3 - [RGS22_HUMAN]	0.95	1	1	1	2					1.39	1.21	1.55	1.35	1264	147.1	7.90
P49796	Regulator of G-protein signaling 3 OS=Homo sapiens GN=RGS3 PE=1 SV=2 - [RGS3_HUMAN]	1.84	1	2	2	2	1.33	1.37	1.25	1.29	0.86	0.97	0.98	1.10	1198	132.3	6.28
P49798	Regulator of G-protein signaling 4 OS=Homo sapiens GN=RGS4 PE=1 SV=1 - [RGS4_HUMAN]	7.80	1	1	1	1					1.05	0.93	1.21	1.06	205	23.2	8.37
P49802	Regulator of G-protein signaling 7 OS=Homo sapiens GN=RGS7 PE=1 SV=3 - [RGS7_HUMAN]	2.02	1	1	1	1	0.57	0.69	0.63	0.76					495	57.6	8.21
Q6MZT1	Regulator of G-protein signaling 7-binding protein OS=Homo sapiens GN=RGS7BP PE=2 SV=3 - [R7BP_HUMAN]	7.00	1	1	1	1					0.77	0.88	0.99	1.12	257	28.9	8.46
A5PLK6	Regulator of G-protein signaling protein-like OS=Homo sapiens GN=RGSL1 PE=2 SV=1 - [RGSL_HUMAN]	4.37	1	3	3	6	1.05	0.96	1.16	1.17	0.93	0.98	1.19	1.26	1076	125.6	9.01
Q92900	Regulator of nonsense transcripts 1 OS=Homo sapiens GN=UPF1 PE=1 SV=2 - [RENT1_HUMAN]	3.54	1	2	2	2	4.16	2.23	0.97	0.52	1.33	1.21	1.31	1.19	1129	124.3	6.61
Q92681	Regulatory solute carrier protein family 1 member 1 OS=Homo sapiens GN=RSC1A1 PE=2 SV=1 - [RSCA1_HUMAN]	0.97	1	1	1	1	1.22	0.77	1.37	0.86					617	66.7	4.87
P35250	Replication factor C subunit 2 OS=Homo sapiens GN=RFC2 PE=1 SV=3 - [RFC2_HUMAN]	7.34	1	1	1	1					0.58	0.51	1.00	0.88	354	39.1	6.44
P40938	Replication factor C subunit 3 OS=Homo sapiens GN=RFC3 PE=1 SV=2 - [RFC3_HUMAN]	3.09	1	1	1	1					0.69	0.88	0.66	0.84	356	40.5	8.34
Q13156	Replication protein A 30 kDa subunit OS=Homo sapiens GN=RPA4 PE=1 SV=2 - [RFA4_HUMAN]	9.58	1	1	1	1	0.71	0.76	0.66	0.72					261	28.8	6.52
Q9HD89	Resistin OS=Homo sapiens GN=RETN PE=2 SV=1 - [RETN_HUMAN]	48.15	1	4	4	12	1.08	1.00	1.07	1.01	1.27	1.18	1.28	1.18	108	11.4	6.86

Q9P2K3	REST corepressor 3 OS=Homo sapiens GN=RCOR3 PE=1 SV=2 - [RCOR3_HUMAN]	10.71	1	3	3	3	2.05	1.36	0.97	0.78	1.32	1.49	1.12	1.27	495	55.5	8.27
Q16799	Reticulon-1 OS=Homo sapiens GN=RTN1 PE=1 SV=1 - [RTN1_HUMAN]	4.77	1	1	2	2		1.29		1.10					776	83.6	4.69
Q9NQC3	Reticulon-4 OS=Homo sapiens GN=RTN4 PE=1 SV=2 - [RTN4_HUMAN]	1.09	1	1	1	3					1.70	1.77	1.62	1.69	1192	129.9	4.50
Q86UN3	Reticulon-4 receptor-like 2 OS=Homo sapiens GN=RTN4RL2 PE=1 SV=1 - [R4RL2_HUMAN]	1.67	1	1	1	3	0.74	0.83	0.75	0.85					420	46.1	7.62
P00352	Retinal dehydrogenase 1 OS=Homo sapiens GN=ALDH1A1 PE=1 SV=2 - [AL1A1_HUMAN]	5.79	1	2	3	5	0.35	0.42	0.37	0.45					501	54.8	6.73
Q02846	Retinal guanylyl cyclase 1 OS=Homo sapiens GN=GUCY2D PE=1 SV=2 - [GUC2D_HUMAN]	2.72	1	2	2	5	1.13	0.98	0.85	0.79					1103	120.0	7.44
P51841	Retinal guanylyl cyclase 2 OS=Homo sapiens GN=GUCY2F PE=2 SV=2 - [GUC2F_HUMAN]	1.62	1	1	1	27	1.13	1.36	1.10	1.38					1108	124.8	7.27
P78363	Retinal-specific ATP-binding cassette transporter OS=Homo sapiens GN=ABCA4 PE=1 SV=3 - [ABCA4_HUMAN]	0.92	1	2	2	2	1.19	1.71	1.23	1.79	0.31	0.28	0.29	0.26	2273	255.8	6.29
P12271	Retinaldehyde-binding protein 1 OS=Homo sapiens GN=RLBP1 PE=1 SV=2 - [RLBP1_HUMAN]	7.26	1	1	1	2	2.04	1.83	1.19	1.04					317	36.5	5.05
Q8IWN7	Retinitis pigmentosa 1-like 1 protein OS=Homo sapiens GN=RP1L1 PE=1 SV=4 - [RP1L1_HUMAN]	0.48	9	1	2	2	1.59	1.38	1.41	1.24					2480	261.0	4.41
P06400	Retinoblastoma-associated protein OS=Homo sapiens GN=RB1 PE=1 SV=2 - [RB_HUMAN]	0.86	1	1	1	1	0.86	0.80	0.97	0.91					928	106.1	7.94
P28749	Retinoblastoma-like protein 1 OS=Homo sapiens GN=RBL1 PE=1 SV=3 - [RBL1_HUMAN]	1.12	1	1	1	1	0.65	0.34	0.72	0.38					1068	120.8	7.50
Q99969	Retinoic acid receptor responder protein 2 OS=Homo sapiens GN=RARRES2 PE=1 SV=1 - [RARR2_HUMAN]	39.26	1	5	5	31	0.80	0.77	0.80	0.77	1.09	1.10	1.10	1.09	163	18.6	9.09
Q8IZV5	Retinol dehydrogenase 10 OS=Homo sapiens GN=RDH10 PE=1 SV=1 - [RDH10_HUMAN]	3.52	1	1	1	1	0.82	0.91	1.08	1.20					341	38.1	7.40
Q96NR8	Retinol dehydrogenase 12 OS=Homo sapiens GN=RDH12 PE=1 SV=3 - [RDH12_HUMAN]	3.48	1	1	1	1					1.32	1.37	1.29	1.34	316	35.1	9.69
P09455	Retinol-binding protein 1 OS=Homo sapiens GN=RBP1 PE=1 SV=2 - [RET1_HUMAN]	8.89	1	1	1	2	1.43	1.04	1.21	0.88					135	15.8	5.11
P02753	Retinol-binding protein 4 OS=Homo sapiens GN=RBP4 PE=1 SV=3 - [RET4_HUMAN]	82.59	1	19	19	1530	0.96	0.91	0.98	0.93	1.14	1.13	1.15	1.15	201	23.0	6.07
Q8NET4	Retrotransposon gag domain-containing protein 1 OS=Homo sapiens GN=RGAG1 PE=1 SV=1 - [RGAG1_HUMAN]	3.60	1	2	2	6	0.95	0.97	1.18	1.17					1388	144.2	6.16
A6NKG5	Retrotransposon-like protein 1 OS=Homo sapiens GN=RTL1 PE=2 SV=2 - [RTL1_HUMAN]	1.77	1	1	1	1	1.22	1.35	1.18	1.30					1359	155.1	5.20
O95980	Reversion-inducing cysteine-rich protein with Kazal motifs OS=Homo sapiens GN=RECK PE=1 SV=1 - [RECK_HUMAN]	1.24	1	1	1	1					1.47	1.29	1.32	1.16	971	106.4	6.74
Q6NW40	RGM domain family member B OS=Homo sapiens GN=RGMB PE=2 SV=3 - [RGMB_HUMAN]	3.66	1	1	1	1	0.05	0.02	0.13	0.06					437	47.5	6.38
P52565	Rho GDP-dissociation inhibitor 1 OS=Homo sapiens GN=ARHGDIA PE=1 SV=3 - [GDIR1_HUMAN]	11.27	1	2	2	5					0.87	0.88	0.86	0.85	204	23.2	5.11
P52566	Rho GDP-dissociation inhibitor 2 OS=Homo sapiens GN=ARHGDIB PE=1 SV=3 - [GDIR2_HUMAN]	25.87	1	4	4	12					2.65	2.20	2.50	2.12	201	23.0	5.21
Q6P4F7	Rho GTPase-activating protein 11A OS=Homo sapiens GN=ARHGAP11A PE=1 SV=2 - [RHGBA_HUMAN]	1.56	1	1	1	1	1.21	0.98	1.19	0.98					1023	113.8	9.07
Q68EM7	Rho GTPase-activating protein 17 OS=Homo sapiens GN=ARHGAP17 PE=1 SV=1 - [RHG17_HUMAN]	1.36	1	1	1	2					1.03	1.15	0.98	1.09	881	95.4	7.62
Q8N392	Rho GTPase-activating protein 18 OS=Homo sapiens GN=ARHGAP18 PE=1 SV=3 - [RHG18_HUMAN]	1.96	1	1	1	6					1.77	1.64	1.81	1.67	663	74.9	6.44
Q5T5U3	Rho GTPase-activating protein 21 OS=Homo sapiens GN=ARHGAP21 PE=1 SV=1 - [RHG21_HUMAN]	0.97	1	1	1	1	0.87	1.13	0.69	0.91					1957	217.2	7.80

Q8N264	Rho GTPase-activating protein 24 OS=Homo sapiens GN=ARHGAP24 PE=1 SV=2 - [RHG24_HUMAN]	2.14	1	1	2	3					0.76	0.27	6.55	2.32	748	84.2	6.67
Q9UNA1	Rho GTPase-activating protein 26 OS=Homo sapiens GN=ARHGAP26 PE=1 SV=1 - [RHG26_HUMAN]	1.97	1	1	1	1					1.54	1.35	1.31	1.14	814	92.2	6.64
Q7Z6I6	Rho GTPase-activating protein 30 OS=Homo sapiens GN=ARHGAP30 PE=1 SV=3 - [RHG30_HUMAN]	2.00	1	1	1	1	1.30	1.37	1.34	1.41					1101	118.5	4.81
A7KAX9	Rho GTPase-activating protein 32 OS=Homo sapiens GN=ARHGAP32 PE=1 SV=1 - [RHG32_HUMAN]	3.55	1	3	3	52	0.68	0.84	0.67	0.79	0.91	0.78	0.80	0.70	2087	230.4	6.74
Q9NRY4	Rho GTPase-activating protein 35 OS=Homo sapiens GN=ARHGAP35 PE=1 SV=2 - [RHG35_HUMAN]	2.97	1	2	4	8	1.07	1.23	1.00	1.15	1.59	1.52	1.13	1.09	1513	172.1	6.79
Q6ZRI8	Rho GTPase-activating protein 36 OS=Homo sapiens GN=ARHGAP36 PE=2 SV=1 - [RHG36_HUMAN]	2.19	1	1	1	1	0.64	0.78	0.58	0.70					547	61.6	9.42
Q9C0H5	Rho GTPase-activating protein 39 OS=Homo sapiens GN=ARHGAP39 PE=1 SV=2 - [RHG39_HUMAN]	1.39	1	1	1	2	1.73	1.97	1.44	1.65					1083	121.2	7.50
Q5TG30	Rho GTPase-activating protein 40 OS=Homo sapiens GN=ARHGAP40 PE=2 SV=3 - [RHG40_HUMAN]	3.05	1	1	1	1	1.08	1.13	1.16	1.23					622	68.9	7.84
Q96QB1	Rho GTPase-activating protein 7 OS=Homo sapiens GN=DLC1 PE=1 SV=4 - [RHG07_HUMAN]	6.02	4	3	4	10	0.78	0.57	0.46	0.25					1528	170.5	6.40
P85298	Rho GTPase-activating protein 8 OS=Homo sapiens GN=ARHGAP8 PE=1 SV=1 - [RHG08_HUMAN]	3.23	1	1	1	1					3.87	4.83	0.73	0.91	464	53.5	9.41
Q5VT97	Rho GTPase-activating protein SYDE2 OS=Homo sapiens GN=SYDE2 PE=1 SV=2 - [SYDE2_HUMAN]	2.43	1	2	2	3	0.84	0.93	0.78	0.87					1194	133.1	8.57
Q92888	Rho guanine nucleotide exchange factor 1 OS=Homo sapiens GN=ARHGEF1 PE=1 SV=2 - [ARHG1_HUMAN]	0.88	1	1	1	1	0.90	1.74	0.90	1.74					912	102.4	5.66
O15013	Rho guanine nucleotide exchange factor 10 OS=Homo sapiens GN=ARHGEF10 PE=1 SV=4 - [ARHGA_HUMAN]	0.80	1	1	1	1	1.39	1.51	1.32	1.44					1369	151.5	5.68
Q9NZN5	Rho guanine nucleotide exchange factor 12 OS=Homo sapiens GN=ARHGEF12 PE=1 SV=1 - [ARHGC_HUMAN]	2.78	9	2	3	3	1.28	1.82	1.42	2.02	1.42	1.87	1.35	1.77	1544	173.1	5.74
O94989	Rho guanine nucleotide exchange factor 15 OS=Homo sapiens GN=ARHGEF15 PE=1 SV=4 - [ARHGF_HUMAN]	7.02	1	2	2	3	0.71	1.12	0.79	1.25					841	91.9	8.25
Q6ZSZ5	Rho guanine nucleotide exchange factor 18 OS=Homo sapiens GN=ARHGEF18 PE=1 SV=3 - [ARHGI_HUMAN]	5.54	1	4	4	8	0.82	1.08	0.91	1.22	0.80	0.86	0.87	0.94	1173	130.7	7.08
Q92974	Rho guanine nucleotide exchange factor 2 OS=Homo sapiens GN=ARHGEF2 PE=1 SV=4 - [ARHG2_HUMAN]	1.12	1	1	1	1	0.17	0.08	0.21	0.10					986	111.5	7.27
Q96DR7	Rho guanine nucleotide exchange factor 26 OS=Homo sapiens GN=ARHGEF26 PE=1 SV=4 - [ARHGQ_HUMAN]	2.53	1	2	2	2	0.92	0.92	0.95	0.96					871	97.3	8.95
A5YM69	Rho guanine nucleotide exchange factor 35 OS=Homo sapiens GN=ARHGEF35 PE=1 SV=1 - [ARG35_HUMAN]	5.99	1	1	1	1					0.60	0.92	0.75	1.16	484	53.3	4.31
Q12774	Rho guanine nucleotide exchange factor 5 OS=Homo sapiens GN=ARHGEF5 PE=1 SV=3 - [ARHG5_HUMAN]	0.88	1	1	1	1					0.84	1.05	1.35	1.68	1597	176.7	5.53
Q15052	Rho guanine nucleotide exchange factor 6 OS=Homo sapiens GN=ARHGEF6 PE=1 SV=2 - [ARHG6_HUMAN]	2.45	2	2	2	6	1.00	1.05	1.17	1.22	1.39	1.17	1.31	1.15	776	87.4	6.05
O43307	Rho guanine nucleotide exchange factor 9 OS=Homo sapiens GN=ARHGEF9 PE=1 SV=3 - [ARHG9_HUMAN]	2.91	1	1	1	7	0.88	1.03	0.93	1.04	0.66	0.66	0.69	0.69	516	60.9	5.72
Q13464	Rho-associated protein kinase 1 OS=Homo sapiens GN=ROCK1 PE=1 SV=1 - [ROCK1_HUMAN]	0.89	1	1	1	2	1.60	1.49	1.35	1.28					1354	158.1	5.90
Q9BYZ6	Rho-related BTB domain-containing protein 2 OS=Homo sapiens GN=RHOBTB2 PE=2 SV=2 - [RHBT2_HUMAN]	2.48	1	1	1	1	1.83	2.23	1.91	2.34					727	82.6	6.74
P08134	Rho-related GTP-binding protein RhoC OS=Homo sapiens GN=RHOC PE=1 SV=1 - [RHOC_HUMAN]	21.76	3	4	4	11	0.87	0.92	0.86	0.91	0.87	0.90	0.80	0.85	193	22.0	6.58
O00212	Rho-related GTP-binding protein RhoD OS=Homo sapiens GN=RHOD PE=1 SV=2 - [RHOD_HUMAN]	4.29	1	1	1	1					0.79	0.74	0.94	0.87	210	23.4	7.96
P58872	Rhomboid-related protein 3 OS=Homo sapiens GN=RHBDL3 PE=2 SV=1 - [RHBL3_HUMAN]	1.24	1	1	1	1					2.14	1.99	1.89	1.75	404	45.2	7.61

Q8IUC4	Rhophilin-2 OS=Homo sapiens GN=RHPN2 PE=1 SV=1 - [RHPN2_HUMAN]	2.04	1	1	1	1	1.09	0.90	1.52	1.25					686	76.9	6.80
Q9BST9	Rhotekin OS=Homo sapiens GN=RTKN PE=1 SV=2 - [RTKN_HUMAN]	1.24	1	1	1	1					0.85	1.04	0.78	0.96	563	62.6	7.44
Q8IZC4	Rhotekin-2 OS=Homo sapiens GN=RTKN2 PE=2 SV=1 - [RTKN2_HUMAN]	2.13	1	1	1	1					0.27	0.47	0.31	0.53	609	69.3	7.83
Q8NHV9	Rhox homeobox family member 1 OS=Homo sapiens GN=RHOXF1 PE=2 SV=1 - [RHXF1_HUMAN]	4.89	1	1	1	1	0.65	0.60	0.69	0.65					184	20.5	6.11
P34096	Ribonuclease 4 OS=Homo sapiens GN=RNASE4 PE=1 SV=3 - [RNAS4_HUMAN]	40.82	1	8	8	26					1.22	1.08	1.13	1.15	147	16.8	9.03
O75792	Ribonuclease H2 subunit A OS=Homo sapiens GN=RNASEH2A PE=1 SV=2 - [RNH2A_HUMAN]	8.70	1	1	1	1	0.39	0.30	0.56	0.43					299	33.4	5.25
P13489	Ribonuclease inhibitor OS=Homo sapiens GN=RNH1 PE=1 SV=2 - [RINI_HUMAN]	10.20	1	2	2	2					1.27	1.49	0.77	0.89	461	49.9	4.82
Q93091	Ribonuclease K6 OS=Homo sapiens GN=RNASE6 PE=1 SV=2 - [RNAS6_HUMAN]	12.00	1	1	1	2					0.70	0.71	0.80	0.80	150	17.2	8.76
P07998	Ribonuclease pancreatic OS=Homo sapiens GN=RNASE1 PE=1 SV=4 - [RNAS1_HUMAN]	19.23	1	2	2	4	1.02	0.98	1.15	1.11	0.96	0.94	0.99	0.98	156	17.6	8.79
P52758	Ribonuclease UK114 OS=Homo sapiens GN=HRSP12 PE=1 SV=1 - [UK114_HUMAN]	12.41	1	1	1	1	1.22	1.39	0.97	1.11					137	14.5	8.68
Q8IY67	Ribonucleoprotein PTB-binding 1 OS=Homo sapiens GN=RAVER1 PE=1 SV=1 - [RAVR1_HUMAN]	2.64	1	1	1	2	####	###	####	####	####	7.61	###	7.70	606	63.8	8.48
Q9HCJ3	Ribonucleoprotein PTB-binding 2 OS=Homo sapiens GN=RAVER2 PE=1 SV=2 - [RAVR2_HUMAN]	9.41	1	2	2	3	1.17	0.98	0.91	0.76	3.01	2.66	2.36	2.09	691	74.3	7.47
Q15418	Ribosomal protein S6 kinase alpha-1 OS=Homo sapiens GN=RPS6KA1 PE=1 SV=2 - [KS6A1_HUMAN]	2.18	1	1	1	6	0.81	0.78	0.80	0.73					735	82.7	7.83
Q9Y6S9	Ribosomal protein S6 kinase-like 1 OS=Homo sapiens GN=RPS6KL1 PE=2 SV=1 - [RPKL1_HUMAN]	3.28	1	1	1	1					11.05	9.56	###	8.81	549	60.0	7.43
Q14692	Ribosome biogenesis protein BMS1 homolog OS=Homo sapiens GN=BMS1 PE=1 SV=1 - [BMS1_HUMAN]	1.95	1	2	2	6	0.86	0.98	1.07	1.22	1.36	1.13	1.03	0.76	1282	145.7	6.44
Q8TDN6	Ribosome biogenesis protein BRX1 homolog OS=Homo sapiens GN=BRIX1 PE=1 SV=2 - [BRX1_HUMAN]	5.10	1	1	1	1	0.94	1.04	0.86	0.95					353	41.4	9.92
Q9H7B2	Ribosome production factor 2 homolog OS=Homo sapiens GN=RPF2 PE=1 SV=2 - [RPF2_HUMAN]	3.27	1	1	1	1	0.14	0.14	0.13	0.13					306	35.6	9.99
Q9P2E9	Ribosome-binding protein 1 OS=Homo sapiens GN=RRBP1 PE=1 SV=4 - [RRBP1_HUMAN]	3.55	1	4	4	12	0.80	1.03	0.92	1.19	0.63	0.65	0.72	0.75	1410	152.4	8.60
Q96AT9	Ribulose-phosphate 3-epimerase OS=Homo sapiens GN=RPE PE=1 SV=1 - [RPE_HUMAN]	3.07	1	1	1	2					0.81	0.77	0.80	0.75	228	24.9	5.58
Q969X0	RILP-like protein 2 OS=Homo sapiens GN=RILPL2 PE=1 SV=1 - [RIPL2_HUMAN]	2.37	1	1	1	1					1.32	0.69	1.46	0.76	211	24.0	4.98
A6NJZ7	RIMS-binding protein 3C OS=Homo sapiens GN=RIMBP3C PE=1 SV=3 - [RIM3C_HUMAN]	3.17	5	3	3	3				0.38	0.13	0.10	0.38	0.28	1639	180.8	6.77
Q9HBD1	RING finger and CCCH-type zinc finger domain-containing protein 2 OS=Homo sapiens GN=RC3H2 PE=1 SV=2 - [RC3H2_HUMAN]	1.09	1	1	1	1	0.72	0.85	0.78	0.93					1191	131.6	6.89
Q96DX4	RING finger and SPRY domain-containing protein 1 OS=Homo sapiens GN=RSPRY1 PE=2 SV=1 - [RSPRY_HUMAN]	3.65	1	2	2	13	0.67	0.75	0.76	0.87	1.30	0.91	1.06	0.99	576	64.1	5.57
Q5M7Z0	RING finger and transmembrane domain-containing protein 1 OS=Homo sapiens GN=RNFT1 PE=2 SV=2 - [RNFT1_HUMAN]	2.07	1	1	1	1	0.46	0.48	0.45	0.47					435	49.7	8.53
Q9BV68	RING finger protein 126 OS=Homo sapiens GN=RNF126 PE=1 SV=1 - [RN126_HUMAN]	8.59	1	1	1	1	1.62		1.15						326	35.6	5.72
Q8WVD5	RING finger protein 141 OS=Homo sapiens GN=RNF141 PE=1 SV=1 - [RN141_HUMAN]	7.83	1	1	1	1					0.84	1.09	1.23	1.60	230	25.5	5.20
Q8NCN4	RING finger protein 169 OS=Homo sapiens GN=RNF169 PE=1 SV=2 - [RN169_HUMAN]	7.20	1	3	3	4	1.02	0.67	0.54	0.36	1.23	0.91	1.06	0.79	708	77.1	9.10

Q9BXT8	RING finger protein 17 OS=Homo sapiens GN=RNF17 PE=1 SV=3 - [RNF17_HUMAN]	0.43	1	1	1	1							1.01	0.71	1623	184.5	5.40
Q96D59	RING finger protein 183 OS=Homo sapiens GN=RNF183 PE=2 SV=1 - [RN183_HUMAN]	21.35	1	1	1	1							1.35	0.71	192	21.7	7.85
Q6ZRF8	RING finger protein 207 OS=Homo sapiens GN=RNF207 PE=1 SV=2 - [RN207_HUMAN]	3.63	1	2	2	2	1.12	1.16	1.15	1.21	0.80	1.07	0.94	1.26	634	70.8	6.68
Q5W0B1	RING finger protein 219 OS=Homo sapiens GN=RNF219 PE=1 SV=1 - [RN219_HUMAN]	0.96	1	1	1	1					0.73	0.76	1.19	1.23	726	81.1	5.72
Q9Y225	RING finger protein 24 OS=Homo sapiens GN=RNF24 PE=1 SV=1 - [RNF24_HUMAN]	9.46	1	1	1	1					0.58	0.67	0.73	0.85	148	17.2	8.54
Q8TBZ6	RNA (guanine-9-)-methyltransferase domain-containing protein 2 OS=Homo sapiens GN=RG9MTD2 PE=2 SV=1 - [RG9D2_HUMAN]	4.42	1	1	1	1					1.09	1.81	1.15	1.91	339	39.7	7.65
Q9Y2P8	RNA 3'-terminal phosphate cyclase-like protein OS=Homo sapiens GN=RCL1 PE=1 SV=3 - [RCL1_HUMAN]	2.14	1	1	1	1	0.96	1.09	0.80	0.92					373	40.8	9.26
O00472	RNA polymerase II elongation factor ELL2 OS=Homo sapiens GN=ELL2 PE=1 SV=2 - [ELL2_HUMAN]	2.03	1	1	1	1					1.30	1.21	1.19	1.10	640	72.3	9.00
Q9HB65	RNA polymerase II elongation factor ELL3 OS=Homo sapiens GN=ELL3 PE=2 SV=2 - [ELL3_HUMAN]	3.78	1	1	1	10					1.08	0.93	1.14	0.97	397	45.3	6.06
Q8N7H5	RNA polymerase II-associated factor 1 homolog OS=Homo sapiens GN=PAF1 PE=1 SV=2 - [PAF1_HUMAN]	3.95	1	1	1	3					1.05	1.19	1.17	1.32	531	59.9	4.63
Q9BWH6	RNA polymerase II-associated protein 1 OS=Homo sapiens GN=RPAP1 PE=1 SV=3 - [RPAP1_HUMAN]	3.73	1	3	3	8	0.61		0.62		0.80	0.96	0.87	0.87	1393	152.7	6.38
Q9H6T3	RNA polymerase II-associated protein 3 OS=Homo sapiens GN=RPAP3 PE=1 SV=2 - [RPAP3_HUMAN]	1.35	1	1	1	2					1.05	0.99	0.87	0.82	665	75.7	6.84
Q8IZ73	RNA pseudouridylate synthase domain-containing protein 2 OS=Homo sapiens GN=RPUSD2 PE=1 SV=2 - [RUSD2_HUMAN]	3.85	1	1	1	1					0.56	0.46	1.07	0.88	545	61.3	7.17
P38159	RNA-binding motif protein, X chromosome OS=Homo sapiens GN=RBMX PE=1 SV=3 - [RBMX_HUMAN]	3.58	1	1	1	1					1.10	1.09	0.93	0.92	391	42.3	10.05
Q8N7X1	RNA-binding motif protein, X-linked-like-3 OS=Homo sapiens GN=RBMXL3 PE=2 SV=2 - [RMXL3_HUMAN]	1.50	1	1	1	1						2.82		2.56	1067	114.9	9.10
Q6ZP01	RNA-binding protein 44 OS=Homo sapiens GN=RBM44 PE=2 SV=2 - [RBM44_HUMAN]	5.23	1	4	4	8	0.75	0.66	0.87	0.77	1.33	1.20	1.17	1.06	1051	117.9	5.72
Q8IUH3	RNA-binding protein 45 OS=Homo sapiens GN=RBM45 PE=1 SV=1 - [RBM45_HUMAN]	2.73	1	1	1	2					0.94	0.91	1.03	0.99	476	53.5	7.17
Q5RL73	RNA-binding protein 48 OS=Homo sapiens GN=RBM48 PE=2 SV=1 - [RBM48_HUMAN]	2.72	1	1	1	1					1.01	0.75	1.08	0.80	367	41.8	8.72
P78332	RNA-binding protein 6 OS=Homo sapiens GN=RBM6 PE=1 SV=5 - [RBM6_HUMAN]	1.25	1	1	1	1					1.07	1.11	1.01	1.05	1123	128.6	6.32
Q01844	RNA-binding protein EWS OS=Homo sapiens GN=EWSR1 PE=1 SV=1 - [EWS_HUMAN]	5.18	1	1	1	1					0.39	0.42	0.55	0.59	656	68.4	9.33
A1L020	RNA-binding protein MEX3A OS=Homo sapiens GN=MEX3A PE=1 SV=1 - [MEX3A_HUMAN]	2.31	1	1	1	1	0.76	0.93	0.89	1.09					520	54.1	7.27
Q6ZRY4	RNA-binding protein with multiple splicing 2 OS=Homo sapiens GN=RBPMS2 PE=2 SV=1 - [RBPS2_HUMAN]	5.26	1	1	1	3					1.14	1.19	1.14	1.18	209	22.5	8.60
Q5TZA2	Rootletin OS=Homo sapiens GN=CROCC PE=1 SV=1 - [CROCC_HUMAN]	1.29	2	1	3	3	1.14	1.02	1.21	1.08					2017	228.4	5.50
Q96C74	Ropporin-1-like protein OS=Homo sapiens GN=ROPN1L PE=1 SV=2 - [ROP1L_HUMAN]	9.13	1	1	1	1	1.29	1.23	1.39	1.34					230	26.1	7.72
Q86VV8	Rotatin OS=Homo sapiens GN=RTTN PE=1 SV=3 - [RTTN_HUMAN]	1.57	1	2	2	2	0.71	0.67	0.76	0.72					2226	248.5	6.70
Q6PCB5	Round spermatid basic protein 1-like protein OS=Homo sapiens GN=RSBN1L PE=1 SV=2 - [RSBNL_HUMAN]	2.01	2	1	2	26	1.00	1.14	1.01	1.15					846	94.8	8.78
Q9Y6N7	Roundabout homolog 1 OS=Homo sapiens GN=ROBO1 PE=1 SV=1 - [ROBO1_HUMAN]	1.21	1	1	1	3	0.94	0.80	0.98	0.77					1651	180.8	6.04

Q9HCK4	Roundabout homolog 2 OS=Homo sapiens GN=ROBO2 PE=1 SV=2 - [ROBO2_HUMAN]	1.16	1	1	1	1					1.12	1.16	1.05	1.09	1378	151.1	6.33
Q96MS0	Roundabout homolog 3 OS=Homo sapiens GN=ROBO3 PE=1 SV=2 - [ROBO3_HUMAN]	2.45	1	2	2	2	1.13	1.18	0.98	1.02					1386	148.1	7.14
Q8WZ75	Roundabout homolog 4 OS=Homo sapiens GN=ROBO4 PE=1 SV=1 - [ROBO4_HUMAN]	4.37	1	4	4	14	1.03	0.95	1.00	0.94	0.94	0.88	0.97	0.92	1007	107.4	6.64
Q9BRU9	rRNA-processing protein UTP23 homolog OS=Homo sapiens GN=UTP23 PE=2 SV=2 - [UTP23_HUMAN]	5.22	1	1	1	1					0.81	0.76	0.83	0.79	249	28.4	10.07
Q8WXA3	RUN and FYVE domain-containing protein 2 OS=Homo sapiens GN=RUFY2 PE=1 SV=2 - [RUFY2_HUMAN]	4.73	1	3	3	9	1.25	1.42	1.21	1.38	2.61	2.85	2.43	2.65	655	75.0	6.51
Q92622	Run domain Beclin-1 interacting and cystein-rich containing protein OS=Homo sapiens GN=KIAA0226 PE=1 SV=4 - [RUBIC_HUMAN]	4.32	1	2	2	2	1.30	1.34	1.35	1.39	1.03	0.48	1.08	0.51	972	108.6	6.25
Q96C34	RUN domain-containing protein 1 OS=Homo sapiens GN=RUNDC1 PE=1 SV=3 - [RUND1_HUMAN]	14.03	1	4	4	6	0.77	0.56	0.66	0.48	0.99	0.76	1.00	0.77	613	67.6	6.19
Q9Y265	RuvB-like 1 OS=Homo sapiens GN=RUVBL1 PE=1 SV=1 - [RUVB1_HUMAN]	3.07	1	1	1	2			0.41						456	50.2	6.42
Q92736	Ryanodine receptor 2 OS=Homo sapiens GN=RYR2 PE=1 SV=3 - [RYR2_HUMAN]	1.75	1	4	4	10	1.00	0.88	0.82	0.72	0.44	0.28	1.08	0.69	4967	564.2	6.07
Q15413	Ryanodine receptor 3 OS=Homo sapiens GN=RYR3 PE=1 SV=3 - [RYR3_HUMAN]	0.62	1	1	1	2					0.89	0.70	0.68	0.53	4870	551.7	5.68
Q9BY12	S phase cyclin A-associated protein in the endoplasmic reticulum OS=Homo sapiens GN=SCAPER PE=1 SV=1 - [SCAPE_HUMAN]	1.07	1	1	1	1					0.33	1.08	0.59	1.91	1399	158.1	7.44
Q70HW3	S-adenosylmethionine mitochondrial carrier protein OS=Homo sapiens GN=SLC25A26 PE=2 SV=1 - [SAMC_HUMAN]	6.93	5	1	2	2	0.96	1.05	1.00	1.10					274	29.4	9.35
Q00266	S-adenosylmethionine synthase isoform type-1 OS=Homo sapiens GN=MAT1A PE=1 SV=2 - [METK1_HUMAN]	4.56	1	1	1	4					1.04	1.09	1.10	1.14	395	43.6	6.29
P10523	S-arrestin OS=Homo sapiens GN=SAG PE=2 SV=3 - [ARRS_HUMAN]	5.43	1	1	1	1	1.22	0.87	1.34	0.95					405	45.1	6.57
P10768	S-formylglutathione hydrolase OS=Homo sapiens GN=ESD PE=1 SV=2 - [ESTD_HUMAN]	5.67	1	2	2	3	0.63	0.63	0.65	0.66	0.99	1.02	1.03	1.06	282	31.4	7.02
Q8N5C6	S1 RNA-binding domain-containing protein 1 OS=Homo sapiens GN=SRBD1 PE=1 SV=2 - [SRBD1_HUMAN]	0.90	1	1	1	1	1.23	1.46	1.16	1.40					995	111.7	8.72
Q8NBX0	Saccharopine dehydrogenase-like oxidoreductase OS=Homo sapiens GN=SCCPDH PE=1 SV=1 - [SCPDL_HUMAN]	3.03	1	1	1	1					0.97	0.97	0.93	0.94	429	47.1	9.14
Q9NZJ4	Sacsin OS=Homo sapiens GN=SACS PE=1 SV=2 - [SACS_HUMAN]	1.68	1	5	5	36	0.95	0.88	0.97	0.98	1.17	1.27	0.99	1.37	4579	520.8	7.05
Q9NWH9	SAFB-like transcription modulator OS=Homo sapiens GN=SLTM PE=1 SV=2 - [SLTM_HUMAN]	0.87	1	1	1	1	0.96	0.91	1.12	1.06					1034	117.1	7.87
Q9Y467	Sal-like protein 2 OS=Homo sapiens GN=SALL2 PE=1 SV=4 - [SALL2_HUMAN]	3.48	1	2	2	2	1.19	1.43	1.20	1.46					1007	105.2	6.30
Q9BXA9	Sal-like protein 3 OS=Homo sapiens GN=SALL3 PE=2 SV=2 - [SALL3_HUMAN]	2.23	1	1	1	1	1.04	1.07	0.96	0.99					1300	135.3	6.99
P02810	Salivary acidic proline-rich phosphoprotein 1/2 OS=Homo sapiens GN=PRH1 PE=1 SV=2 - [PRPC_HUMAN]	26.51	1	1	1	13	0.86	0.59	0.61	0.42					166	17.0	4.84
O75995	SAM and SH3 domain-containing protein 3 OS=Homo sapiens GN=SASH3 PE=1 SV=2 - [SASH3_HUMAN]	5.53	1	1	1	1	0.36	0.25	0.79	0.57					380	41.6	5.27
Q9Y3Z3	SAM domain and HD domain-containing protein 1 OS=Homo sapiens GN=SAMHD1 PE=1 SV=2 - [SAMH1_HUMAN]	2.56	1	1	1	1					1.04	0.89	1.24	1.06	626	72.2	7.14
Q86TD4	Sarcalumenin OS=Homo sapiens GN=SRL PE=2 SV=2 - [SRCA_HUMAN]	0.97	1	1	1	1					5.89	3.84	4.79	3.12	932	100.7	4.40
Q14BN4	Sarcolemmal membrane-associated protein OS=Homo sapiens GN=SLMAP PE=1 SV=1 - [SLMAP_HUMAN]	1.81	1	1	2	11	0.82	0.74	0.91	0.81					828	95.1	5.47
Q9NXZ1	Sarcoma antigen 1 OS=Homo sapiens GN=SAGE1 PE=2 SV=2 - [SAGE1_HUMAN]	4.54	1	2	2	2					0.75	0.75	0.69	0.70	904	99.2	6.48

O14983	Sarcoplasmic/endoplasmic reticulum calcium ATPase 1 OS=Homo sapiens GN=ATP2A1 PE=1 SV=1 - [AT2A1_HUMAN]	1.60	1	1	1	4	1.25	1.21	1.60	1.55					1001	110.2	5.16
Q6R2W3	SCAN domain-containing protein 3 OS=Homo sapiens GN=SCAND3 PE=2 SV=1 - [SCND3_HUMAN]	1.36	1	1	1	10	0.82	0.67	0.77	0.64	1.17	1.18	1.18	1.21	1325	151.6	6.73
Q96C86	Scavenger mRNA-decapping enzyme DcpS OS=Homo sapiens GN=DCPS PE=1 SV=2 - [DCPS_HUMAN]	5.64	1	1	1	1	1.09	0.90	0.92	0.76					337	38.6	6.38
Q6AZY7	Scavenger receptor class A member 3 OS=Homo sapiens GN=SCARA3 PE=2 SV=1 - [SCAR3_HUMAN]	6.93	1	2	2	3	0.76	0.66	0.81	0.71			0.83		606	65.1	6.54
Q6ZMJ2	Scavenger receptor class A member 5 OS=Homo sapiens GN=SCARA5 PE=2 SV=1 - [SCAR5_HUMAN]	4.65	1	1	1	2					1.14	1.00	1.06	0.93	495	54.0	7.09
Q14162	Scavenger receptor class F member 1 OS=Homo sapiens GN=SCARF1 PE=1 SV=3 - [SREC_HUMAN]	2.41	1	1	1	1	0.80	1.08	0.85	1.17					830	87.3	6.37
Q96GP6	Scavenger receptor class F member 2 OS=Homo sapiens GN=SCARF2 PE=1 SV=4 - [SREC2_HUMAN]	3.91	1	2	2	2	1.72	1.01	1.97	1.70					870	92.4	8.44
Q86VB7	Scavenger receptor cysteine-rich type 1 protein M130 OS=Homo sapiens GN=CD163 PE=1 SV=2 - [C163A_HUMAN]	16.18	1	16	16	41	0.90	0.89	0.82	0.80	0.93	0.82	0.93	0.93	1156	125.4	5.95
Q8IYM2	Schlafen family member 12 OS=Homo sapiens GN=SLFN12 PE=1 SV=2 - [SLN12_HUMAN]	1.04	1	1	1	1					0.84	0.34	0.92	0.37	578	66.9	8.59
Q6IEE8	Schlafen family member 12-like OS=Homo sapiens GN=SLFN12L PE=2 SV=3 - [SN12L_HUMAN]	1.94	1	1	1	1					0.94	0.95	0.92	0.93	620	70.5	8.02
Q15468	SCL-interrupting locus protein OS=Homo sapiens GN=STIL PE=1 SV=2 - [STIL_HUMAN]	1.17	1	1	1	16	1.05	1.10	1.07	1.17	0.52	0.77	0.90	1.34	1287	142.9	6.46
Q9UHJ3	Scm-like with four MBT domains protein 1 OS=Homo sapiens GN=SFMBT1 PE=1 SV=2 - [SMBT1_HUMAN]	1.27	1	1	1	1					11.37	8.60	###	8.22	866	98.1	6.21
Q6P3W7	SCY1-like protein 2 OS=Homo sapiens GN=SCYL2 PE=1 SV=1 - [SCYL2_HUMAN]	0.65	1	1	1	1	0.95	0.81	1.10	0.95					929	103.6	8.22
Q8WU76	Sec1 family domain-containing protein 2 OS=Homo sapiens GN=SCFD2 PE=1 SV=2 - [SCFD2_HUMAN]	1.02	1	1	1	1					1.20	1.29	1.24	1.33	684	75.1	6.68
Q86VW0	SEC14 domain and spectrin repeat-containing protein 1 OS=Homo sapiens GN=SESTD1 PE=1 SV=2 - [SESD1_HUMAN]	3.02	1	2	2	6	1.10	1.15	1.09	1.14	0.81	0.78	0.71	0.69	696	79.3	5.10
Q96FV2	Secernin-2 OS=Homo sapiens GN=SCRN2 PE=2 SV=3 - [SCRN2_HUMAN]	6.35	1	2	2	2	1.16	1.02	1.14	1.01			0.64	0.52	425	46.6	5.67
Q8WVN6	Secreted and transmembrane protein 1 OS=Homo sapiens GN=SECTM1 PE=1 SV=2 - [SCTM1_HUMAN]	6.45	1	1	1	3					0.95	0.95	0.96	0.96	248	27.0	7.43
Q8N474	Secreted frizzled-related protein 1 OS=Homo sapiens GN=SFRP1 PE=1 SV=1 - [SFRP1_HUMAN]	5.10	1	1	1	1					0.80	0.79	0.83	0.82	314	35.4	8.85
Q13103	Secreted phosphoprotein 24 OS=Homo sapiens GN=SPP2 PE=1 SV=1 - [SPP24_HUMAN]	26.54	1	6	6	14	0.85	1.03	0.88	1.08	0.93	0.90	1.05	0.97	211	24.3	8.32
Q96PL1	Secretoglobin family 3A member 2 OS=Homo sapiens GN=SCGB3A2 PE=1 SV=1 - [SG3A2_HUMAN]	33.33	1	2	2	3	1.53	1.64	1.47	1.58					93	10.2	7.28
P05060	Secretogranin-1 OS=Homo sapiens GN=CHGB PE=1 SV=2 - [SCG1_HUMAN]	1.92	1	1	1	1					1.11	1.32	1.01	1.20	677	78.2	5.07
Q8WXD2	Secretogranin-3 OS=Homo sapiens GN=SCG3 PE=1 SV=3 - [SCG3_HUMAN]	1.50	1	1	1	1	1.18	1.39	1.20	1.43					468	53.0	5.03
O15126	Secretory carrier-associated membrane protein 1 OS=Homo sapiens GN=SCAMP1 PE=1 SV=2 - [SCAM1_HUMAN]	3.25	1	1	1	1					0.65	0.59	0.61	0.56	338	37.9	7.42
Q9NZH5	Securin-2 OS=Homo sapiens GN=PTTG2 PE=1 SV=2 - [PTTG2_HUMAN]	20.30	1	2	2	11	0.79	0.93	0.85	0.93	0.99	1.01	1.00	0.99	202	22.3	6.25
Q9UHJ6	Sedoheptulokinase OS=Homo sapiens GN=SHPK PE=1 SV=3 - [SHPK_HUMAN]	3.77	1	1	1	1	1.28	1.43	1.15	1.29					478	51.5	6.83
P54792	Segment polarity protein dishevelled homolog DVL-1-like OS=Homo sapiens GN=DVL1L1 PE=1 SV=1 - [DVL1L_HUMAN]	2.09	2	1	1	1					1.30	1.65	1.25	1.59	670	73.2	6.46
Q6UXD5	Seizure 6-like protein 2 OS=Homo sapiens GN=SEZ6L2 PE=1 SV=2 - [SE6L2_HUMAN]	1.21	1	1	1	2	1.14	1.28	1.15	1.30					910	97.5	4.89

Q9BYH1	Seizure 6-like protein OS=Homo sapiens GN=SEZ6L PE=1 SV=1 - [SE6L1_HUMAN]	0.78	1	1	1	1					4.44	3.34	4.45	3.35	1024	111.7	4.78
Q13228	Selenium-binding protein 1 OS=Homo sapiens GN=SELENBP1 PE=1 SV=2 - [SBP1_HUMAN]	6.14	1	2	2	6	0.90	0.76	0.92	0.79	0.49	0.60	0.58	0.71	472	52.4	6.37
Q96T21	Selenocysteine insertion sequence-binding protein 2 OS=Homo sapiens GN=SECISBP2 PE=1 SV=2 - [SEBP2_HUMAN]	1.05	1	1	1	2					1.15	1.07	0.97	0.90	854	95.4	8.12
Q96I15	Selenocysteine lyase OS=Homo sapiens GN=SCLY PE=1 SV=2 - [SCLY_HUMAN]	11.69	1	2	2	2	1.00	0.87	0.98	0.85					445	48.1	7.12
P57772	Selenocysteine-specific elongation factor OS=Homo sapiens GN=EEFSEC PE=1 SV=4 - [SELB_HUMAN]	1.01	1	1	1	1					0.91	1.16	0.72	0.92	596	65.3	8.35
Q9NZV5	Selenoprotein N OS=Homo sapiens GN=SEPN1 PE=1 SV=5 - [SELN_HUMAN]	2.54	1	3	3	4	1.02	1.32	1.10	1.42	0.81	0.77	0.41	0.52	590	65.8	5.48
P49908	Selenoprotein P OS=Homo sapiens GN=SEPP1 PE=1 SV=3 - [SEPP1_HUMAN]	19.16	1	10	10	96	0.95	0.96	0.93	0.94	0.86	0.85	0.86	0.85	381	43.2	7.87
P63302	Selenoprotein W OS=Homo sapiens GN=SEPW1 PE=1 SV=3 - [SELW_HUMAN]	12.64	1	1	1	2					1.02	0.90	1.17	1.04	87	9.4	9.23
Q9NS98	Semaphorin-3G OS=Homo sapiens GN=SEMA3G PE=1 SV=1 - [SEM3G_HUMAN]	3.71	1	2	2	4	0.85	0.83	0.97	0.95					782	86.6	7.78
Q9NPR2	Semaphorin-4B OS=Homo sapiens GN=SEMA4B PE=1 SV=3 [SEM4B_HUMAN]	4.09	1	2	2	2	0.92	0.95	0.98	1.03	1.29	1.35	1.59	1.68	832	92.1	6.95
Q92854	Semaphorin-4D OS=Homo sapiens GN=SEMA4D PE=1 SV=1 - [SEM4D_HUMAN]	1.97	1	1	1	3	1.06	0.83	1.21	0.95	1.14	1.02	1.15	1.04	862	96.1	7.96
Q13591	Semaphorin-5A OS=Homo sapiens GN=SEMA5A PE=1 SV=3 - [SEM5A_HUMAN]	3.54	1	2	2	3					0.08	0.04	0.42	0.39	1074	120.5	7.21
Q9H3T3	Semaphorin-6B OS=Homo sapiens GN=SEMA6B PE=1 SV=4 [SEM6B_HUMAN]	2.25	1	2	2	2	0.85	0.68	0.86	0.69	0.75	0.93	0.72	0.90	888	95.2	8.48
Q9BQF6	Sentrin-specific protease 7 OS=Homo sapiens GN=SENP7 PE=1 SV=4 - [SENP7_HUMAN]	2.00	1	1	1	1	0.28	0.45	0.21	0.34					1050	119.6	6.61
Q14674	Separin OS=Homo sapiens GN=ESPL1 PE=1 SV=3 - [ESPL1_HUMAN]	0.75	1	1	1	1	1.27	0.64	1.15	0.59					2120	233.0	7.55
Q9NVA2	Septin-11 OS=Homo sapiens GN=SEPT11 PE=1 SV=3 - [SEP11_HUMAN]	6.06	1	3	3	7					1.12	1.06	1.05	0.99	429	49.4	6.81
Q14141	Septin-6 OS=Homo sapiens GN=SEPT6 PE=1 SV=4 - [SEPT6_HUMAN]	2.30	1	1	1	3					1.91	1.66	1.63	1.42	434	49.7	6.67
Q16181	Septin-7 OS=Homo sapiens GN=SEPT7 PE=1 SV=2 - [SEPT7_HUMAN]	6.18	1	2	2	3	1.19	1.49	1.04	1.32	1.51	1.53	1.45	1.47	437	50.6	8.63
P10124	Serglycin OS=Homo sapiens GN=SRGN PE=1 SV=3 - [SRGN_HUMAN]	20.89	1	3	3	36	0.92	0.89	0.92	0.75	2.23	1.89	2.22	1.99	158	17.6	4.96
O15269	Serine palmitoyltransferase 1 OS=Homo sapiens GN=SPTLC1 PE=1 SV=1 - [SPTC1_HUMAN]	4.65	1	1	1	1	0.70	0.86	0.68	0.83					473	52.7	6.01
Q9NUV7	Serine palmitoyltransferase 3 OS=Homo sapiens GN=SPTLC3 PE=1 SV=3 - [SPTC3_HUMAN]	1.99	1	1	1	1	0.68		0.60						552	62.0	8.78
Q6UWY2	Serine protease 57 OS=Homo sapiens GN=PRSS57 PE=2 SV=2 - [PRS57_HUMAN]	6.71	1	1	1	1					0.43	0.21	0.36	0.17	283	30.3	9.32
Q92743	Serine protease HTRA1 OS=Homo sapiens GN=HTRA1 PE=1 SV=1 - [HTRA1_HUMAN]	7.71	1	3	3	5	1.20	1.13	1.25	1.18					480	51.3	7.83
Q9NQ38	Serine protease inhibitor Kazal-type 5 OS=Homo sapiens GN=SPINK5 PE=1 SV=2 - [ISK5_HUMAN]	6.58	1	5	5	11					1.44	0.91	1.40	0.92	1064	120.6	8.06
Q9NP81	Serine--tRNA ligase, mitochondrial OS=Homo sapiens GN=SARS2 PE=1 SV=1 - [SYSM_HUMAN]	4.25	3	1	2	2	1.24	1.14	1.22	1.12					518	58.2	8.13
Q9Y3F4	Serine-threonine kinase receptor-associated protein OS=Homo sapiens GN=STRAP PE=1 SV=1 - [STRAP_HUMAN]	2.86	1	1	1	4	0.76	0.62	0.82	0.68	0.84	0.92	0.77	0.84	350	38.4	5.12
Q9UQ35	Serine/arginine repetitive matrix protein 2 OS=Homo sapiens GN=SRRM2 PE=1 SV=2 - [SRRM2_HUMAN]	0.62	1	1	1	2	1.08	1.05	1.14	1.11	0.99	1.08	0.86	0.94	2752	299.4	12.06

Q96IZ7	Serine/Arginine-related protein 53 OS=Homo sapiens GN=RSRC1 PE=1 SV=1 - [RSRC1_HUMAN]	3.59	1	1	1	3	0.88	0.56	0.79	0.50	0.81	0.63	0.89	0.69	334	38.7	11.08
O94804	Serine/threonine-protein kinase 10 OS=Homo sapiens GN=STK10 PE=1 SV=1 - [STK10_HUMAN]	2.89	1	2	2	2					1.04	1.12	0.95	1.03	968	112.1	6.95
Q9BXU1	Serine/threonine-protein kinase 31 OS=Homo sapiens GN=STK31 PE=2 SV=2 - [STK31_HUMAN]	2.65	1	1	2	2	0.06	0.03	0.04	0.02					1019	115.6	5.14
Q9BYT3	Serine/threonine-protein kinase 33 OS=Homo sapiens GN=STK33 PE=1 SV=1 - [STK33_HUMAN]	7.20	1	1	1	2	0.91	0.73	1.10	0.89					514	57.8	7.03
Q8TDR2	Serine/threonine-protein kinase 35 OS=Homo sapiens GN=STK35 PE=1 SV=2 - [STK35_HUMAN]	7.87	1	2	3	10	1.06	1.17	0.84	0.93	1.06	0.79	1.22	0.86	534	58.0	9.74
Q15208	Serine/threonine-protein kinase 38 OS=Homo sapiens GN=STK38 PE=1 SV=1 - [STK38_HUMAN]	2.80	1	1	1	1	0.80	0.76	0.97	0.92					465	54.2	7.15
Q13535	Serine/threonine-protein kinase ATR OS=Homo sapiens GN=ATR PE=1 SV=3 - [ATR_HUMAN]	2.19	1	3	3	3					0.96	0.94	0.84	0.94	2644	301.2	7.43
O14757	Serine/threonine-protein kinase Chk1 OS=Homo sapiens GN=CHEK1 PE=1 SV=2 - [CHK1_HUMAN]	1.26	1	1	1	1					0.92	0.54	0.74	0.44	476	54.4	8.25
Q96GX5	Serine/threonine-protein kinase greatwall OS=Homo sapiens GN=MASTL PE=1 SV=1 - [GWL_HUMAN]	3.07	1	2	2	2	0.78	0.84	0.80	0.87	0.84	1.06	0.49	0.62	879	97.3	5.99
Q96QS6	Serine/threonine-protein kinase H2 OS=Homo sapiens GN=PSKH2 PE=2 SV=1 - [KPSH2_HUMAN]	2.60	1	1	1	1					0.86	0.61	1.07	0.76	385	43.0	9.63
Q9UPZ9	Serine/threonine-protein kinase ICK OS=Homo sapiens GN=ICK PE=1 SV=1 - [ICK_HUMAN]	1.42	1	1	1	1	1.32	1.41	1.34	1.44					632	71.4	9.77
O95835	Serine/threonine-protein kinase LATS1 OS=Homo sapiens GN=LATS1 PE=1 SV=1 - [LATS1_HUMAN]	2.92	1	1	1	1					0.49	0.63	0.59	0.75	1130	126.8	8.70
Q96Q04	Serine/threonine-protein kinase LMTK3 OS=Homo sapiens GN=LMTK3 PE=2 SV=2 - [LMTK3_HUMAN]	2.26	1	1	1	1	1.21	1.07	1.21	1.08					1460	153.6	4.86
Q5VT25	Serine/threonine-protein kinase MRCK alpha OS=Homo sapiens GN=CDC42BPA PE=1 SV=1 - [MRCKA_HUMAN]	1.15	1	2	2	2	0.56	0.67	0.42	0.50	1.68	1.64	1.58	1.55	1732	197.2	6.58
Q9Y5S2	Serine/threonine-protein kinase MRCK beta OS=Homo sapiens GN=CDC42BPB PE=1 SV=2 - [MRCKB_HUMAN]	1.52	1	2	2	6	0.97	0.73	1.31	0.98	1.32	1.50	1.35	1.54	1711	194.2	6.37
Q9P289	Serine/threonine-protein kinase MST4 OS=Homo sapiens GN=MST4 PE=1 SV=2 - [MST4_HUMAN]	2.40	1	1	1	1					0.91	0.86	0.66	0.62	416	46.5	5.29
Q16512	Serine/threonine-protein kinase N1 OS=Homo sapiens GN=PKN1 PE=1 SV=2 - [PKN1_HUMAN]	1.91	3	1	2	3	1.51	1.93	1.42	1.82					942	103.9	6.37
Q96PY6	Serine/threonine-protein kinase Nek1 OS=Homo sapiens GN=NEK1 PE=1 SV=2 - [NEK1_HUMAN]	1.75	1	1	2	4	0.93	0.81	1.12	0.98					1258	142.7	5.94
P51956	Serine/threonine-protein kinase Nek3 OS=Homo sapiens GN=NEK3 PE=1 SV=2 - [NEK3_HUMAN]	6.92	1	2	3	5	0.43	0.40	0.66	0.62	0.88	0.92	0.88	0.93	506	57.7	7.17
Q9HC98	Serine/threonine-protein kinase Nek6 OS=Homo sapiens GN=NEK6 PE=1 SV=2 - [NEK6_HUMAN]	5.75	2	2	2	2	0.70	0.46	0.84	0.53					313	35.7	8.03
Q9UBE8	Serine/threonine-protein kinase NLK OS=Homo sapiens GN=NLK PE=1 SV=2 - [NLK_HUMAN]	2.28	1	1	1	1			0.28	0.23					527	58.2	8.13
O95747	Serine/threonine-protein kinase OSR1 OS=Homo sapiens GN=OXSR1 PE=1 SV=1 - [OXSR1_HUMAN]	7.40	1	2	2	5					1.06	1.28	1.02	1.23	527	58.0	6.43
O75914	Serine/threonine-protein kinase PAK 3 OS=Homo sapiens GN=PAK3 PE=1 SV=2 - [PAK3_HUMAN]	4.47	1	1	1	1					0.65		0.75		559	62.3	5.45
P53350	Serine/threonine-protein kinase PLK1 OS=Homo sapiens GN=PLK1 PE=1 SV=1 - [PLK1_HUMAN]	3.98	1	2	2	2	1.01	1.02	0.98	1.00					603	68.2	8.91
O14730	Serine/threonine-protein kinase RIO3 OS=Homo sapiens GN=RIOK3 PE=1 SV=2 - [RIOK3_HUMAN]	4.43	1	1	1	1	4.89	1.74	1.41	0.51					519	59.1	5.76
Q9Y2K2	Serine/threonine-protein kinase SIK3 OS=Homo sapiens GN=SIK3 PE=1 SV=3 - [SIK3_HUMAN]	2.85	1	2	2	3	0.85	0.95	1.18	1.10					1263	139.9	6.70
Q96Q15	Serine/threonine-protein kinase SMG1 OS=Homo sapiens GN=SMG1 PE=1 SV=3 - [SMG1_HUMAN]	0.68	3	1	2	2	0.98	0.91	0.91	0.86					3661	410.2	6.46

Q7L7X3	Serine/threonine-protein kinase TAO1 OS=Homo sapiens GN=TAOK1 PE=1 SV=1 - [TAOK1_HUMAN]	2.40	1	1	1	1							0.43	0.24	1001	116.0	7.65
Q9UL54	Serine/threonine-protein kinase TAO2 OS=Homo sapiens GN=TAOK2 PE=1 SV=2 - [TAOK2_HUMAN]	1.94	1	1	1	1	1.20	1.17	1.34	1.31					1235	138.2	7.27
O75385	Serine/threonine-protein kinase ULK1 OS=Homo sapiens GN=ULK1 PE=1 SV=2 - [ULK1_HUMAN]	4.19	1	2	2	3	0.79	0.90	0.79	0.90	0.84	0.87	0.76	0.78	1050	112.6	8.79
Q8IYT8	Serine/threonine-protein kinase ULK2 OS=Homo sapiens GN=ULK2 PE=1 SV=3 - [ULK2_HUMAN]	5.98	1	4	4	17	0.97	0.91	1.03	0.95	1.10	1.30	0.85	1.00	1036	112.6	8.53
Q96C45	Serine/threonine-protein kinase ULK4 OS=Homo sapiens GN=ULK4 PE=2 SV=2 - [ULK4_HUMAN]	0.78	1	1	1	2	1.07	1.35	1.05	1.33					1275	142.4	6.29
Q9H4A3	Serine/threonine-protein kinase WNK1 OS=Homo sapiens GN=WNK1 PE=1 SV=2 - [WNK1_HUMAN]	1.64	1	2	3	5					1.82	1.16	1.40	1.22	2382	250.6	6.34
Q9BYP7	Serine/threonine-protein kinase WNK3 OS=Homo sapiens GN=WNK3 PE=1 SV=3 - [WNK3_HUMAN]	1.33	1	2	2	14	1.26	1.17	1.32	1.23	0.47	0.77	0.75	1.23	1800	198.3	6.07
Q14738	Serine/threonine-protein phosphatase 2A 56 kDa regulatory subunit delta isoform OS=Homo sapiens GN=PPP2R5D PE=1 SV=1 - [2A5D_HUMAN]	2.16	1	1	1	1	1.14	1.14	0.85	0.86					602	69.9	8.13
Q16537	Serine/threonine-protein phosphatase 2A 56 kDa regulatory subunit epsilon isoform OS=Homo sapiens GN=PPP2R5E PE=1 SV=1 - [2A5E_HUMAN]	3.00	1	1	1	1	0.95	0.89	0.77	0.73					467	54.7	6.95
Q13362	Serine/threonine-protein phosphatase 2A 56 kDa regulatory subunit gamma isoform OS=Homo sapiens GN=PPP2R5C PE=1 SV=3 - [2A5G_HUMAN]	1.72	1	1	1	1	1.45	1.62	1.45	1.63					524	61.0	6.87
P30153	Serine/threonine-protein phosphatase 2A 65 kDa regulatory subunit A alpha isoform OS=Homo sapiens GN=PPP2R1A PE=1 SV=4 - [2AAA_HUMAN]	8.15	2	4	4	5	0.71	0.82	0.66	0.76					589	65.3	5.11
Q06190	Serine/threonine-protein phosphatase 2A regulatory subunit B'' subunit alpha OS=Homo sapiens GN=PPP2R3A PE=1 SV=1 - [P2R3A_HUMAN]	4.26	1	2	2	5					0.66	0.30	0.99	0.45	1150	130.2	5.21
Q9NY27	Serine/threonine-protein phosphatase 4 regulatory subunit 2 OS=Homo sapiens GN=PPP4R2 PE=1 SV=3 - [PP4R2_HUMAN]	4.32	1	1	1	1	1.10	1.06	1.15	1.10					417	46.9	4.54
Q6IN85	Serine/threonine-protein phosphatase 4 regulatory subunit 3A OS=Homo sapiens GN=SMEK1 PE=1 SV=1 - [P4R3A_HUMAN]	1.92	1	1	1	1					0.71	0.75	0.84	0.88	833	95.3	4.94
O15084	Serine/threonine-protein phosphatase 6 regulatory ankyrin repeat subunit A OS=Homo sapiens GN=ANKRD28 PE=1 SV=5 - [ANR28_HUMAN]	4.56	1	1	2	6	1.21	0.70	1.00	0.64					1053	112.9	6.25
Q8NB46	Serine/threonine-protein phosphatase 6 regulatory ankyrin repeat subunit C OS=Homo sapiens GN=ANKRD52 PE=1 SV=3 - [ANR52_HUMAN]	1.12	1	1	1	1					1.72	1.18	1.91	1.31	1076	115.0	6.48
Q9UPN7	Serine/threonine-protein phosphatase 6 regulatory subunit 1 OS=Homo sapiens GN=PPP6R1 PE=1 SV=5 - [PP6R1_HUMAN]	2.04	1	1	1	1					0.84	1.20	0.77	1.09	881	96.7	4.55
O75170	Serine/threonine-protein phosphatase 6 regulatory subunit 2 OS=Homo sapiens GN=PPP6R2 PE=1 SV=2 - [PP6R2_HUMAN]	1.97	1	1	1	1	1.09	0.85	1.12	0.87					966	104.9	4.87
Q5H9R7	Serine/threonine-protein phosphatase 6 regulatory subunit 3 OS=Homo sapiens GN=PPP6R3 PE=1 SV=2 - [PP6R3_HUMAN]	1.83	1	1	1	1					0.25	0.12	0.73	0.36	873	97.6	4.60
P36873	Serine/threonine-protein phosphatase PP1-gamma catalytic subunit OS=Homo sapiens GN=PPP1CC PE=1 SV=1 - [PP1G_HUMAN]	4.33	3	1	1	3	1.21	1.62	2.41	3.23	1.24	1.11	1.26	1.12	323	37.0	6.54
Q86SQ7	Serologically defined colon cancer antigen 8 OS=Homo sapiens GN=SDCCAG8 PE=1 SV=1 - [SDCG8_HUMAN]	1.40	1	1	1	2	0.71	0.63	0.73	0.65	0.69	0.62	0.70	0.64	713	82.6	5.81
P02787	Serotransferrin OS=Homo sapiens GN=TF PE=1 SV=3 - [TRFE_HUMAN]	83.09	4	95	99	17041	1.24	1.19	1.24	1.19	1.03	1.02	1.01	1.03	698	77.0	7.12
Q86U17	Serpin A11 OS=Homo sapiens GN=SERPINA11 PE=2 SV=2 - [SPA11_HUMAN]	20.85	1	8	8	22	1.10	1.03	1.11	1.04	0.90	0.72	0.92	0.73	422	47.0	7.68
P48595	Serpin B10 OS=Homo sapiens GN=SERPINB10 PE=1 SV=1 - [SPB10_HUMAN]	11.08	1	3	3	3	1.00	1.00	1.09	1.10	0.51	0.45	1.04	0.84	397	45.4	6.16
Q96P63	Serpin B12 OS=Homo sapiens GN=SERPINB12 PE=1 SV=1 - [SPB12_HUMAN]	8.64	1	1	2	8	1.03	0.86	0.78	0.65	1.00	0.83	1.36	1.44	405	46.2	5.53
Q9UIV8	Serpin B13 OS=Homo sapiens GN=SERPINB13 PE=2 SV=2 - [SPB13_HUMAN]	5.88	1	2	2	3	0.59	0.61	0.54	0.56	0.63	0.87	0.58	0.80	391	44.2	5.71
P36952	Serpin B5 OS=Homo sapiens GN=SERPINB5 PE=1 SV=2 - [SPB5_HUMAN]	4.53	1	1	1	1					1.27	1.16	1.03	0.94	375	42.1	6.05

P35237	Serpin B6 OS=Homo sapiens GN=SERPINB6 PE=1 SV=3 - [SPB6_HUMAN]	11.44	1	3	3	6					0.76	0.84	0.80	0.73	376	42.6	5.27
P50452	Serpin B8 OS=Homo sapiens GN=SERPINB8 PE=1 SV=2 - [SPB8_HUMAN]	3.48	1	1	1	3					0.90	0.74	0.78	0.61	374	42.7	5.57
O75830	Serpin I2 OS=Homo sapiens GN=SERPINI2 PE=2 SV=1 - [SPI2_HUMAN]	2.22	1	1	1	3	0.84	0.70	0.78	0.66					405	46.1	5.16
P02768	Serum albumin OS=Homo sapiens GN=ALB PE=1 SV=2 - [ALBU_HUMAN]	96.06	10	126	127	99215	0.96	0.93	0.98	0.95	1.00	1.01	0.99	1.01	609	69.3	6.28
P02735	Serum amyloid A protein OS=Homo sapiens GN=SAA1 PE=1 SV=2 - [SAA_HUMAN]	62.30	2	9	9	233	0.89	0.89	0.73	0.77	0.75	0.70	0.78	0.72	122	13.5	6.79
P35542	Serum amyloid A-4 protein OS=Homo sapiens GN=SAA4 PE=1 SV=2 - [SAA4_HUMAN]	56.92	1	10	10	431	0.73	0.72	0.73	0.72	0.89	0.91	0.88	0.89	130	14.7	9.07
P02743	Serum amyloid P-component OS=Homo sapiens GN=APCS PE=1 SV=2 - [SAMP_HUMAN]	47.53	1	10	10	166	1.12	1.10	1.09	1.08	0.94	0.99	0.93	0.98	223	25.4	6.54
O95810	Serum deprivation-response protein OS=Homo sapiens GN=SDPR PE=1 SV=3 - [SDPR_HUMAN]	12.47	1	5	5	17	0.69	0.63	0.64	0.59	1.32	1.25	1.43	1.35	425	47.1	5.21
P27169	Serum paraoxonase/arylesterase 1 OS=Homo sapiens GN=PON1 PE=1 SV=3 - [PON1_HUMAN]	64.23	2	14	17	992	1.16	1.14	1.15	1.14	0.99	1.08	0.92	1.04	355	39.7	5.22
Q15165	Serum paraoxonase/arylesterase 2 OS=Homo sapiens GN=PON2 PE=1 SV=3 - [PON2_HUMAN]	5.37	1	1	1	3					0.97	1.31	0.82	1.12	354	39.4	5.60
Q15166	Serum paraoxonase/lactonase 3 OS=Homo sapiens GN=PON3 PE=1 SV=3 - [PON3_HUMAN]	38.14	1	7	9	107	1.12	1.15	1.08	1.05	0.88	0.83	1.02	1.04	354	39.6	5.41
Q8NEF9	Serum response factor-binding protein 1 OS=Homo sapiens GN=SRFBP1 PE=1 SV=1 - [SRFB1_HUMAN]	3.50	1	1	1	4					0.90	0.92	0.77	0.82	429	48.6	9.58
Q9NVD3	SET domain-containing protein 4 OS=Homo sapiens GN=SETD4 PE=2 SV=1 - [SETD4_HUMAN]	11.82	8	2	3	5					1.17	1.19	1.13	1.15	440	50.4	8.22
Q9UBL3	Set1/Ash2 histone methyltransferase complex subunit ASH2 OS=Homo sapiens GN=ASH2L PE=1 SV=1 - [ASH2L_HUMAN]	0.96	1	1	1	1					0.54	0.74	0.63	0.87	628	68.7	5.69
Q9UN30	Sex comb on midleg-like protein 1 OS=Homo sapiens GN=SCML1 PE=1 SV=2 - [SCML1_HUMAN]	6.08	1	1	1	1	0.50	1.16	0.79	1.84					329	37.4	6.89
Q9UQR0	Sex comb on midleg-like protein 2 OS=Homo sapiens GN=SCML2 PE=1 SV=1 - [SCML2_HUMAN]	6.71	1	3	3	3	0.98	1.03	1.02	1.07	1.09	0.82	0.96	0.72	700	77.2	8.54
P04278	Sex hormone-binding globulin OS=Homo sapiens GN=SHBG PE=1 SV=2 - [SHBG_HUMAN]	60.45	1	17	17	228	1.83	1.90	1.72	1.80	1.38	1.50	1.36	1.48	402	43.8	6.71
Q9BRG2	SH2 domain-containing protein 3A OS=Homo sapiens GN=SH2D3A PE=1 SV=1 - [SH23A_HUMAN]	3.30	1	1	1	1					1.07	0.64	1.52	0.91	576	63.1	7.42
Q8N5H7	SH2 domain-containing protein 3C OS=Homo sapiens GN=SH2D3C PE=1 SV=1 - [SH2D3_HUMAN]	5.12	1	2	2	2	0.56	0.43	0.49	0.38					860	94.4	8.07
Q9UQQ2	SH2B adapter protein 3 OS=Homo sapiens GN=SH2B3 PE=2 SV=2 - [SH2B3_HUMAN]	4.52	1	1	1	2							0.51		575	63.2	7.20
Q9Y566	SH3 and multiple ankyrin repeat domains protein 1 OS=Homo sapiens GN=SHANK1 PE=1 SV=2 - [SHAN1_HUMAN]	0.83	1	1	1	46	1.12	1.18	1.14	1.23					2161	224.8	8.15
Q9UPX8	SH3 and multiple ankyrin repeat domains protein 2 OS=Homo sapiens GN=SHANK2 PE=1 SV=3 - [SHAN2_HUMAN]	1.84	1	1	1	1	2.38	1.30	1.46	0.80					1470	158.7	6.90
Q9BYB0	SH3 and multiple ankyrin repeat domains protein 3 OS=Homo sapiens GN=SHANK3 PE=1 SV=2 - [SHAN3_HUMAN]	3.39	3	2	3	3	0.75	0.62	0.91	0.76	0.87	1.07	0.81	1.00	1741	186.2	9.03
Q8TE82	SH3 domain and tetratricopeptide repeat-containing protein 1 OS=Homo sapiens GN=SH3TC1 PE=1 SV=3 - [S3TC1_HUMAN]	0.75	1	1	1	1	1.30	0.93	1.24	0.89					1336	146.9	6.25
Q9UJC5	SH3 domain-binding glutamic acid-rich-like protein 2 OS=Homo sapiens GN=SH3BGRL2 PE=1 SV=2 - [SH3L2_HUMAN]	55.14	1	4	4	27					1.16	1.13	1.17	1.10	107	12.3	6.71
Q9H299	SH3 domain-binding glutamic acid-rich-like protein 3 OS=Homo sapiens GN=SH3BGRL3 PE=1 SV=1 - [SH3L3_HUMAN]	31.18	1	3	3	24	0.85	0.84	0.87	0.87	1.17	1.11	1.36	1.30	93	10.4	4.93
O75368	SH3 domain-binding glutamic acid-rich-like protein OS=Homo sapiens GN=SH3BGRL PE=1 SV=1 - [SH3L1_HUMAN]	30.70	1	3	3	11					1.18	1.23	1.15	1.12	114	12.8	5.25

Q9Y3L3	SH3 domain-binding protein 1 OS=Homo sapiens GN=SH3BP1 PE=1 SV=3 - [3BP1_HUMAN]	3.85	1	2	2	2	0.86	0.99	0.99	1.15					701	75.7	6.77
P78314	SH3 domain-binding protein 2 OS=Homo sapiens GN=SH3BP2 PE=1 SV=2 - [3BP2_HUMAN]	1.07	1	1	1	3	1.63	1.61	1.69	1.69	1.12	1.42	1.07	1.36	561	62.2	7.78
Q9P0V3	SH3 domain-binding protein 4 OS=Homo sapiens GN=SH3BP4 PE=1 SV=1 - [SH3B4_HUMAN]	1.45	1	1	1	1					0.86	0.62	0.73	0.52	963	107.4	7.71
Q96B97	SH3 domain-containing kinase-binding protein 1 OS=Homo sapiens GN=SH3KBP1 PE=1 SV=2 - [SH3K1_HUMAN]	2.11	1	1	1	1					1.21	1.61	0.85	1.13	665	73.1	6.62
Q8TBC3	SH3KBP1-binding protein 1 OS=Homo sapiens GN=SHKBP1 PE=1 SV=2 - [SHKB1_HUMAN]	1.13	1	1	1	3					0.70	0.45	1.02	0.66	707	76.3	8.28
Q8NEM2	SHC SH2 domain-binding protein 1 OS=Homo sapiens GN=SHCBP1 PE=1 SV=3 - [SHCBP_HUMAN]	2.98	1	1	1	1	1.10	1.09	1.32	1.32					672	75.6	4.75
Q92529	SHC-transforming protein 3 OS=Homo sapiens GN=SHC3 PE=1 SV=1 - [SHC3_HUMAN]	3.20	1	1	1	1	1.58	1.65	1.73	1.80					594	64.0	8.32
Q6S5L8	SHC-transforming protein 4 OS=Homo sapiens GN=SHC4 PE=1 SV=1 - [SHC4_HUMAN]	5.71	1	2	2	13	1.16	1.12	1.03	1.00	1.43	1.38	1.46	1.41	630	68.7	7.84
A0MZ66	Shootin-1 OS=Homo sapiens GN=KIAA1598 PE=1 SV=4 - [SHOT1_HUMAN]	1.27	1	1	1	1	1.07	1.27	1.08	1.29					631	71.6	5.33
O60902	Short stature homeobox protein 2 OS=Homo sapiens GN=SHOX2 PE=2 SV=4 - [SHOX2_HUMAN]	11.48	1	1	1	1	0.23	0.46	0.21	0.41					331	34.9	8.82
P48995	Short transient receptor potential channel 1 OS=Homo sapiens GN=TRPC1 PE=1 SV=1 - [TRPC1_HUMAN]	1.77	1	1	1	1					1.01	0.99	0.99	0.97	793	91.2	8.05
O75911	Short-chain dehydrogenase/reductase 3 OS=Homo sapiens GN=DHRS3 PE=1 SV=2 - [DHRS3_HUMAN]	4.30	1	1	1	2					0.62	0.68	0.79	0.87	302	33.5	8.84
Q562F6	Shugoshin-like 2 OS=Homo sapiens GN=SGOL2 PE=1 SV=2 - [SGOL2_HUMAN]	2.13	1	1	1	3	1.20	1.39	1.13	1.37					1265	144.6	7.91
Q9HAT2	Sialate O-acetylesterase OS=Homo sapiens GN=SIAE PE=1 SV=1 - [SIAE_HUMAN]	2.87	1	1	1	1	1.05	1.14	0.93	1.02					523	58.3	7.33
Q08ET2	Sialic acid-binding Ig-like lectin 14 OS=Homo sapiens GN=SIGLEC14 PE=1 SV=1 - [SIG14_HUMAN]	7.32	2	2	2	6	1.87	1.61	1.73	1.51	1.15	1.46	1.22	1.54	396	43.9	7.96
A6NMB1	Sialic acid-binding Ig-like lectin 16 OS=Homo sapiens GN=SIGLEC16 PE=2 SV=3 - [SIG16_HUMAN]	1.46	1	1	1	1					0.78	0.69	0.68	0.60	481	53.0	9.03
Q9NYZ4	Sialic acid-binding Ig-like lectin 8 OS=Homo sapiens GN=SIGLEC8 PE=1 SV=2 - [SIGL8_HUMAN]	7.01	1	1	1	1	0.16	0.17	0.26	0.27					499	54.0	8.09
Q5T5P2	Sickle tail protein homolog OS=Homo sapiens GN=SKT PE=1 SV=2 - [SKT_HUMAN]	1.18	1	1	1	2	0.38	0.27	0.58	0.41					1943	214.0	7.06
Q9NXL6	SID1 transmembrane family member 1 OS=Homo sapiens GN=SIDT1 PE=2 SV=2 - [SIDT1_HUMAN]	2.06	1	1	1	1					0.67	0.79	0.57	0.68	827	93.8	7.30
Q8NBJ9	SID1 transmembrane family member 2 OS=Homo sapiens GN=SIDT2 PE=1 SV=2 - [SIDT2_HUMAN]	1.80	1	1	1	1					0.85	0.60	0.79	0.56	832	94.4	6.96
Q8IUH8	Signal peptide peptidase-like 2C OS=Homo sapiens GN=SPPL2C PE=1 SV=3 - [SPP2C_HUMAN]	2.19	1	1	1	1	0.52	0.55	0.50	0.54					684	74.5	6.76
Q9NQ36	Signal peptide, CUB and EGF-like domain-containing protein 2 OS=Homo sapiens GN=SCUBE2 PE=2 SV=2 - [SCUB2_HUMAN]	1.80	1	1	1	1	1.25	1.67	1.33	1.79					999	109.9	6.65
Q9UHB9	Signal recognition particle 68 kDa protein OS=Homo sapiens GN=SRP68 PE=1 SV=2 - [SRP68_HUMAN]	1.91	1	1	1	1					3.24	4.42	0.83	1.13	627	70.7	8.56
O76094	Signal recognition particle 72 kDa protein OS=Homo sapiens GN=SRP72 PE=1 SV=3 - [SRP72_HUMAN]	2.53	1	1	1	1					0.84	0.09	0.83	0.09	671	74.6	9.26
P08240	Signal recognition particle receptor subunit alpha OS=Homo sapiens GN=SRPR PE=1 SV=2 - [SRPR_HUMAN]	1.57	1	1	1	2	1.33	1.06	1.36	1.08					638	69.8	8.95
P42224	Signal transducer and activator of transcription 1-alpha/beta OS=Homo sapiens GN=STAT1 PE=1 SV=2 - [STAT1_HUMAN]	3.20	1	1	2	2	0.98	0.93	1.01	0.97					750	87.3	6.05
P52630	Signal transducer and activator of transcription 2 OS=Homo sapiens GN=STAT2 PE=1 SV=1 - [STAT2_HUMAN]	2.70	2	2	2	2	0.56	0.34	0.51	0.43					851	97.9	5.49

Q14765	Signal transducer and activator of transcription 4 OS=Homo sapiens GN=STAT4 PE=1 SV=1 - [STAT4_HUMAN]	2.94	1	1	1	1					0.63	0.74	0.63	0.74	748	85.9	6.65
P51692	Signal transducer and activator of transcription 5B OS=Homo sapiens GN=STAT5B PE=1 SV=2 - [STA5B_HUMAN]	4.45	1	2	2	4					1.97	2.05	1.65	1.71	787	89.8	6.05
Q92783	Signal transducing adapter molecule 1 OS=Homo sapiens GN=STAM PE=1 SV=3 - [STAM1_HUMAN]	8.52	2	3	3	6					1.32	1.06	1.50	1.20	540	59.1	4.82
O43166	Signal-induced proliferation-associated 1-like protein 1 OS=Homo sapiens GN=SIPA1L1 PE=1 SV=4 - [SI1L1_HUMAN]	1.22	1	1	1	1	1.07	1.14	1.07	1.14					1804	199.9	8.19
Q9P2F8	Signal-induced proliferation-associated 1-like protein 2 OS=Homo sapiens GN=SIPA1L2 PE=1 SV=2 - [SI1L2_HUMAN]	7.72	1	5	5	10	0.91	0.86	0.72	0.69	1.05	0.90	1.49	1.27	1722	190.3	6.77
Q5TFQ8	Signal-regulatory protein beta-1 isoform 3 OS=Homo sapiens GN=SIRPB1 PE=1 SV=1 - [SIRBL_HUMAN]	5.03	2	2	2	8	1.19	1.17	1.21	1.19	0.81	0.86	0.72	0.77	398	43.3	7.83
Q9UGK3	Signal-transducing adaptor protein 2 OS=Homo sapiens GN=STAP2 PE=1 SV=2 - [STAP2_HUMAN]	2.23	1	1	1	1	0.63	0.74	0.55	0.64					403	44.9	8.16
P81133	Single-minded homolog 1 OS=Homo sapiens GN=SIM1 PE=2 SV=2 - [SIM1_HUMAN]	7.96	1	2	2	12	0.73	0.72	0.83	0.82					766	85.5	7.43
P81877	Single-stranded DNA-binding protein 2 OS=Homo sapiens GN=SSBP2 PE=1 SV=2 - [SSBP2_HUMAN]	11.36	1	1	1	2	0.52	0.89	0.78	1.36					361	37.8	6.64
Q9BVC3	Sister chromatid cohesion protein DCC1 OS=Homo sapiens GN=DSCC1 PE=1 SV=2 - [DCC1_HUMAN]	3.56	1	1	1	1	1.31	1.04	1.41	1.12					393	44.8	5.17
Q29RF7	Sister chromatid cohesion protein PDS5 homolog A OS=Homo sapiens GN=PDS5A PE=1 SV=1 - [PDS5A_HUMAN]	3.52	1	2	2	2	0.61	0.69	0.58	0.66	1.00	0.82	1.22	0.99	1337	150.7	7.91
P84550	SKI family transcriptional corepressor 1 OS=Homo sapiens GN=SKOR1 PE=1 SV=1 - [SKOR1_HUMAN]	2.18	1	1	1	6	0.60	0.62	1.23	1.29	0.92	0.96	0.89	0.93	965	99.8	6.54
Q2VWA4	SKI family transcriptional corepressor 2 OS=Homo sapiens GN=SKOR2 PE=1 SV=2 - [SKOR2_HUMAN]	3.00	1	2	2	3					0.91	0.47	1.03	0.45	1001	104.2	6.48
P12755	Ski oncogene OS=Homo sapiens GN=SKI PE=1 SV=1 - [SKI_HUMAN]	2.20	1	1	1	1					0.70	0.47	2.48	1.66	728	80.0	7.62
P12757	Ski-like protein OS=Homo sapiens GN=SKIL PE=1 SV=2 - [SKIL_HUMAN]	1.90	1	1	1	1					1.03	1.04	1.08	1.09	684	76.9	7.11
Q5T750	Skin-specific protein 32 OS=Homo sapiens GN=XP32 PE=1 SV=1 - [XP32_HUMAN]	3.20	1	1	1	3					1.38	1.16	1.83	1.53	250	26.2	7.97
Q9P270	SLAIN motif-containing protein 2 OS=Homo sapiens GN=SLAIN2 PE=1 SV=2 - [SLAI2_HUMAN]	4.30	1	2	2	3	0.67	0.36	0.71	0.39	0.98	0.90	0.87	0.79	581	62.5	9.45
Q9UIB8	SLAM family member 5 OS=Homo sapiens GN=CD84 PE=1 SV=1 - [SLAF5_HUMAN]	6.67	1	2	2	6	1.12	1.22	1.12	1.23	1.03	1.02	1.01	1.00	345	38.8	7.06
Q96A28	SLAM family member 9 OS=Homo sapiens GN=SLAMF9 PE=2 SV=2 - [SLAF9_HUMAN]	10.03	1	1	1	1			0.45						289	32.4	7.65
Q96PX8	SLIT and NTRK-like protein 1 OS=Homo sapiens GN=SLITRK1 PE=1 SV=2 - [SLIK1_HUMAN]	1.44	1	1	1	3	0.69	0.70	0.66	0.68					696	77.7	6.44
Q8IW52	SLIT and NTRK-like protein 4 OS=Homo sapiens GN=SLITRK4 PE=2 SV=1 - [SLIK4_HUMAN]	5.50	1	3	3	3	0.88	0.91	0.89	0.94	0.75	0.16	0.86	0.18	837	94.3	7.80
O75093	Slit homolog 1 protein OS=Homo sapiens GN=SLIT1 PE=2 SV=4 - [SLIT1_HUMAN]	1.50	1	1	2	2	1.78	1.00	2.06	1.16					1534	167.8	6.57
O75094	Slit homolog 3 protein OS=Homo sapiens GN=SLIT3 PE=2 SV=3 - [SLIT3_HUMAN]	3.74	1	3	3	3	0.84	0.70	0.90	0.72					1523	167.6	7.65
Q7Z6B7	SLIT-ROBO Rho GTPase-activating protein 1 OS=Homo sapiens GN=SRGAP1 PE=1 SV=1 - [SRGP1_HUMAN]	7.00	1	4	4	4	1.18	0.99	1.16	0.98	0.69	0.54	0.63	0.51	1085	124.2	6.83
O75044	SLIT-ROBO Rho GTPase-activating protein 2 OS=Homo sapiens GN=SRGAP2 PE=1 SV=2 - [FNBP2_HUMAN]	2.05	1	1	1	1	0.71	0.68	0.61	0.59					1071	120.8	6.70
O43147	Small G protein signaling modulator 2 OS=Homo sapiens GN=SGSM2 PE=1 SV=4 - [SGSM2_HUMAN]	1.29	1	1	1	20	0.88	0.97	0.90	1.01	0.94	0.97	0.95	0.98	1006	113.2	6.67
P62314	Small nuclear ribonucleoprotein Sm D1 OS=Homo sapiens GN=SNRPD1 PE=1 SV=1 - [SMD1_HUMAN]	12.61	1	1	1	1					0.59	0.66	1.32	1.49	119	13.3	11.56

Q96PI1	Small proline-rich protein 4 OS=Homo sapiens GN=SPRR4 PE=2 SV=1 - [SPRR4_HUMAN]	21.52	1	1	1	1	0.79	0.69	0.74	0.65					79	8.8	9.66
Q5T8I9	Small RNA 2'-O-methyltransferase OS=Homo sapiens GN=HENMT1 PE=2 SV=1 - [HENMT_HUMAN]	3.31	1	1	1	1	1.02	0.94	1.11	1.04					393	44.5	5.29
O75691	Small subunit processome component 20 homolog OS=Homo sapiens GN=UTP20 PE=1 SV=3 - [UTP20_HUMAN]	2.98	1	4	4	5	0.58	0.47	0.63	0.51					2785	318.2	7.39
P61956	Small ubiquitin-related modifier 2 OS=Homo sapiens GN=SUMO2 PE=1 SV=3 - [SUMO2_HUMAN]	12.63	3	1	1	4					0.94	1.03	1.04	1.14	95	10.9	5.50
P53814	Smoothelin OS=Homo sapiens GN=SMTN PE=1 SV=7 - [SMTN_HUMAN]	1.74	1	1	1	1					0.85	0.95	0.80	0.89	917	99.0	9.07
Q99835	Smoothened homolog OS=Homo sapiens GN=SMO PE=1 SV=1 - [SMO_HUMAN]	3.18	1	2	2	3	1.63	1.47	1.04	0.95	0.78	0.57	0.83	0.61	787	86.3	8.34
Q8NCG7	Sn1-specific diacylglycerol lipase beta OS=Homo sapiens GN=DAGLB PE=1 SV=2 - [DGLB_HUMAN]	2.53	1	1	1	1	1.72	1.20	1.29	0.90					672	73.7	6.55
Q8NBS3	Sodium bicarbonate transporter-like protein 11 OS=Homo sapiens GN=SLC4A11 PE=1 SV=2 - [S4A11_HUMAN]	1.23	1	1	1	1					0.93	0.94	0.92	0.93	891	99.5	7.68
Q96NL6	Sodium channel and clathrin linker 1 OS=Homo sapiens GN=SCLT1 PE=1 SV=2 - [SCLT1_HUMAN]	3.34	1	2	2	4	0.76	0.75	0.71	0.71					688	80.9	6.07
Q9NY46	Sodium channel protein type 3 subunit alpha OS=Homo sapiens GN=SCN3A PE=1 SV=2 - [SCN3A_HUMAN]	0.75	1	1	1	1					1.08	1.36	0.89	1.12	2000	226.1	5.80
P35499	Sodium channel protein type 4 subunit alpha OS=Homo sapiens GN=SCN4A PE=1 SV=4 - [SCN4A_HUMAN]	0.76	1	1	1	1	1.37	1.10	1.61	1.30					1836	207.9	5.10
Q9UQD0	Sodium channel protein type 8 subunit alpha OS=Homo sapiens GN=SCN8A PE=1 SV=1 - [SCN8A_HUMAN]	0.71	1	1	1	4	0.96	0.90	1.11	1.05					1980	225.1	6.28
Q15858	Sodium channel protein type 9 subunit alpha OS=Homo sapiens GN=SCN9A PE=1 SV=3 - [SCN9A_HUMAN]	0.35	1	1	1	1					0.62	0.89	0.60	0.86	1988	226.2	6.93
Q8IWT1	Sodium channel subunit beta-4 OS=Homo sapiens GN=SCN4B PE=1 SV=1 - [SCN4B_HUMAN]	7.89	1	1	1	1	1.41	1.57	1.14	1.28					228	25.0	7.36
Q8IZF0	Sodium leak channel non-selective protein OS=Homo sapiens GN=NALCN PE=1 SV=1 - [NALCN_HUMAN]	0.92	1	1	2	26					0.97	1.03	0.96	1.02	1738	200.2	8.68
P48029	Sodium- and chloride-dependent creatine transporter 1 OS=Homo sapiens GN=SLC6A8 PE=1 SV=1 - [SC6A8_HUMAN]	4.09	3	1	2	4	1.02	1.18	1.06	1.23					635	70.5	6.42
P48067	Sodium- and chloride-dependent glycine transporter 1 OS=Homo sapiens GN=SLC6A9 PE=2 SV=3 - [SC6A9_HUMAN]	3.97	1	1	1	1					0.43	0.43	0.78	0.78	706	78.2	7.93
Q6U841	Sodium-driven chloride bicarbonate exchanger OS=Homo sapiens GN=SLC4A10 PE=1 SV=1 - [S4A10_HUMAN]	2.06	1	1	1	3	1.50	1.92	1.33	1.71					1118	125.9	6.49
Q5PT55	Sodium/bile acid cotransporter 5 OS=Homo sapiens GN=SLC10A5 PE=1 SV=1 - [NTCP5_HUMAN]	2.51	1	1	1	1					0.99	1.07	1.04	1.13	438	48.8	8.78
P32418	Sodium/calcium exchanger 1 OS=Homo sapiens GN=SLC8A1 PE=1 SV=3 - [NAC1_HUMAN]	1.34	1	1	1	1					0.26	0.98			973	108.5	5.00
Q2M3M2	Sodium/glucose cotransporter 4 OS=Homo sapiens GN=SLC5A9 PE=2 SV=2 - [SC5A9_HUMAN]	3.67	1	1	1	1	1.63	0.75	1.68	0.78					681	74.0	7.09
Q5TAH2	Sodium/hydrogen exchanger 11 OS=Homo sapiens GN=SLC9A11 PE=2 SV=1 - [S9A11_HUMAN]	1.51	9	1	2	2					1.09	1.07	1.02	1.01	1124	129.0	6.92
Q96T83	Sodium/hydrogen exchanger 7 OS=Homo sapiens GN=SLC9A7 PE=1 SV=1 - [SL9A7_HUMAN]	3.72	1	1	1	2					1.12	0.78	1.28	0.90	725	80.1	6.42
Q8WWX8	Sodium/myo-inositol cotransporter 2 OS=Homo sapiens GN=SLC5A11 PE=2 SV=1 - [SC5AB_HUMAN]	2.22	1	1	1	1	1.38	1.46	1.33	1.40					675	74.0	6.77
P50993	Sodium/potassium-transporting ATPase subunit alpha-2 OS=Homo sapiens GN=ATP1A2 PE=1 SV=1 - [AT1A2_HUMAN]	2.16	1	1	1	2					1.24	1.43	1.15	1.32	1020	112.2	5.66
O60721	Sodium/potassium/calcium exchanger 1 OS=Homo sapiens GN=SLC24A1 PE=1 SV=1 - [NCKX1_HUMAN]	0.73	1	1	1	1					0.42	0.48	0.74	0.85	1099	121.3	4.94
Q6J4K2	Sodium/potassium/calcium exchanger 6 OS=Homo sapiens GN=SLC24A6 PE=2 SV=2 - [NCKX6_HUMAN]	4.62	1	1	1	2	0.84	0.66	0.83	0.66					584	64.2	8.02

A1L4H1	Soluble scavenger receptor cysteine-rich domain-containing protein SSC5D OS=Homo sapiens GN=SSC5D PE=2 SV=3 - [SRCRL_HUMAN]	10.81	1	11	11	23	0.90	0.83	0.82	0.84					1573	165.6	6.13
Q13621	Solute carrier family 12 member 1 OS=Homo sapiens GN=SLC12A1 PE=1 SV=2 - [S12A1_HUMAN]	3.28	1	1	1	1					1.22	1.92	1.44	2.27	1099	121.4	7.39
P55011	Solute carrier family 12 member 2 OS=Homo sapiens GN=SLC12A2 PE=1 SV=1 - [S12A2_HUMAN]	1.65	1	1	1	1	1.21	1.36	1.27	1.44					1212	131.4	6.40
Q9UP95	Solute carrier family 12 member 4 OS=Homo sapiens GN=SLC12A4 PE=1 SV=2 - [S12A4_HUMAN]	1.20	1	1	1	4	0.95	0.93	0.94	0.93					1085	120.6	6.44
Q9H2X9	Solute carrier family 12 member 5 OS=Homo sapiens GN=SLC12A5 PE=2 SV=3 - [S12A5_HUMAN]	1.05	1	1	1	1	0.67	0.52	0.87	0.69					1139	126.1	6.73
Q9Y666	Solute carrier family 12 member 7 OS=Homo sapiens GN=SLC12A7 PE=1 SV=3 - [S12A7_HUMAN]	0.83	1	1	1	1	0.73	0.81	0.68	0.75					1083	119.0	6.71
Q9BXP2	Solute carrier family 12 member 9 OS=Homo sapiens GN=SLC12A9 PE=1 SV=1 - [S12A9_HUMAN]	1.75	1	1	1	2					0.86	0.88	0.84	0.85	914	96.0	8.07
P11166	Solute carrier family 2, facilitated glucose transporter member 1 OS=Homo sapiens GN=SLC2A1 PE=1 SV=2 - [GTR1_HUMAN]	2.64	1	1	1	1	0.93	1.00	0.98	1.05					492	54.0	8.72
Q8TD20	Solute carrier family 2, facilitated glucose transporter member 12 OS=Homo sapiens GN=SLC2A12 PE=2 SV=1 - [GTR12_HUMAN]	2.27	1	2	2	4					0.43	0.99	0.43	0.70	617	66.9	8.37
P11169	Solute carrier family 2, facilitated glucose transporter member 3 OS=Homo sapiens GN=SLC2A3 PE=1 SV=1 - [GTR3_HUMAN]	2.62	1	1	1	1					0.86	0.82	0.88	0.83	496	53.9	7.20
O15245	Solute carrier family 22 member 1 OS=Homo sapiens GN=SLC22A1 PE=1 SV=2 - [S22A1_HUMAN]	1.62	1	1	1	1					0.67	0.76	0.90	1.02	554	61.1	6.83
O15244	Solute carrier family 22 member 2 OS=Homo sapiens GN=SLC22A2 PE=1 SV=2 - [S22A2_HUMAN]	5.23	1	1	1	1	1.03	1.37	0.64	0.86					555	62.5	8.37
Q9H015	Solute carrier family 22 member 4 OS=Homo sapiens GN=SLC22A4 PE=1 SV=3 - [S22A4_HUMAN]	1.09	1	1	1	1					0.76	0.89	0.71	0.84	551	62.1	7.23
Q8IVM8	Solute carrier family 22 member 9 OS=Homo sapiens GN=SLC22A9 PE=2 SV=1 - [S22A9_HUMAN]	1.99	1	1	1	1					1.03	1.09	1.09	1.16	553	62.1	8.10
Q6PIV7	Solute carrier family 25 member 34 OS=Homo sapiens GN=SLC25A34 PE=2 SV=1 - [S2534_HUMAN]	3.29	1	2	2	3					0.82	0.88	0.83	0.89	304	32.2	9.13
Q8NG04	Solute carrier family 26 member 10 OS=Homo sapiens GN=SLC26A10 PE=2 SV=1 - [S2610_HUMAN]	2.84	1	1	1	9					0.70	0.79	0.71	0.79	563	60.0	8.27
Q9NQQ7	Solute carrier family 35 member C2 OS=Homo sapiens GN=SLC35C2 PE=1 SV=2 - [S35C2_HUMAN]	10.41	1	1	1	2					1.09	0.91	1.08	0.90	365	40.4	8.18
P0CK96	Solute carrier family 35 member E2B OS=Homo sapiens GN=SLC35E2B PE=2 SV=1 - [S352B_HUMAN]	8.89	1	1	1	2	0.82	0.80	0.70	0.68					405	43.7	8.40
Q8IXU6	Solute carrier family 35 member F2 OS=Homo sapiens GN=SLC35F2 PE=1 SV=1 - [S35F2_HUMAN]	3.48	1	1	1	1					0.51	0.49	0.51	0.49	374	41.2	6.21
P0C7Q5	Solute carrier family 35 member G4 OS=Homo sapiens GN=SLC35G4 PE=3 SV=1 - [S35G4_HUMAN]	14.20	1	2	2	2	3.92	5.63	4.54	6.61					338	35.4	7.17
Q9NP59	Solute carrier family 40 member 1 OS=Homo sapiens GN=SLC40A1 PE=1 SV=1 - [S40A1_HUMAN]	2.28	1	1	1	1	1.24	1.02	1.26	1.04					571	62.5	6.52
Q8NBI5	Solute carrier family 43 member 3 OS=Homo sapiens GN=SLC43A3 PE=1 SV=2 - [S43A3_HUMAN]	5.09	1	1	1	1					0.79	0.92	0.74	0.87	491	54.5	8.53
Q5BKX6	Solute carrier family 45 member 4 OS=Homo sapiens GN=SLC45A4 PE=1 SV=2 - [S45A4_HUMAN]	1.69	1	1	1	1					0.34	0.10	2.16	0.64	768	83.8	5.53
Q96BD0	Solute carrier organic anion transporter family member 4A1 OS=Homo sapiens GN=SLCO4A1 PE=1 SV=2 - [SO4A1_HUMAN]	3.74	1	1	1	3					0.85	0.96	0.85	0.96	722	77.1	7.83
Q6ZQN7	Solute carrier organic anion transporter family member 4C1 OS=Homo sapiens GN=SLCO4C1 PE=1 SV=1 - [SO4C1_HUMAN]	4.56	1	2	2	3	1.53	1.55	1.41	1.43					724	78.9	7.53
P32745	Somatostatin receptor type 3 OS=Homo sapiens GN=SSTR3 PE=1 SV=1 - [SSR3_HUMAN]	3.35	1	1	1	1	0.37	0.45	0.75	0.92					418	45.8	8.59
P01241	Somatotropin OS=Homo sapiens GN=GH1 PE=1 SV=2 - [SOMA_HUMAN]	14.75	3	1	4	7					0.85	1.34	0.63	1.00	217	24.8	5.43

Q07890	Son of sevenless homolog 2 OS=Homo sapiens GN=SOS2 PE=1 SV=2 - [SOS2_HUMAN]	1.80	1	2	2	2	1.27	1.37	1.28	1.39					1332	152.9	6.84
Q15465	Sonic hedgehog protein OS=Homo sapiens GN=SHH PE=1 SV=1 - [SHH_HUMAN]	4.11	1	1	1	1					1.01	1.21	0.89	1.06	462	49.6	7.99
Q9BX66	Sorbin and SH3 domain-containing protein 1 OS=Homo sapiens GN=SORBS1 PE=1 SV=3 - [SRBS1_HUMAN]	2.63	1	2	2	4					1.11	1.22	1.04	1.15	1292	142.4	6.84
Q9Y512	Sorting and assembly machinery component 50 homolog OS=Homo sapiens GN=SAMM50 PE=1 SV=3 - [SAM50_HUMAN]	4.48	1	1	1	2					1.05	1.15	0.72	0.80	469	51.9	6.90
Q9Y5W8	Sorting nexin-13 OS=Homo sapiens GN=SNX13 PE=1 SV=4 - [SNX13_HUMAN]	3.31	1	1	2	2	1.22	1.83	1.39	2.10					968	112.1	6.58
Q96RF0	Sorting nexin-18 OS=Homo sapiens GN=SNX18 PE=1 SV=2 - [SNX18_HUMAN]	1.91	1	1	1	1					0.47	0.92	0.90	1.77	628	68.9	5.68
Q92543	Sorting nexin-19 OS=Homo sapiens GN=SNX19 PE=1 SV=2 - [SNX19_HUMAN]	1.71	1	1	1	1					0.47	0.83	0.44	0.77	992	108.5	5.03
Q969T3	Sorting nexin-21 OS=Homo sapiens GN=SNX21 PE=2 SV=1 - [SNX21_HUMAN]	3.22	1	1	1	2					0.82	0.49	0.96	0.58	373	41.3	5.24
Q96L92	Sorting nexin-27 OS=Homo sapiens GN=SNX27 PE=1 SV=2 - [SNX27_HUMAN]	4.07	1	2	2	8	1.03	0.93	1.09	0.99	0.65	0.78	0.69	0.73	541	61.2	6.49
Q86XE0	Sorting nexin-32 OS=Homo sapiens GN=SNX32 PE=2 SV=1 - [SNX32_HUMAN]	2.48	1	1	1	1	1.13	1.34	1.16	1.39					403	46.4	7.09
Q9UNH7	Sorting nexin-6 OS=Homo sapiens GN=SNX6 PE=1 SV=1 - [SNX6_HUMAN]	5.91	1	1	2	5					0.34	0.30	1.33	1.16	406	46.6	6.16
Q96AH0	SOSS complex subunit B2 OS=Homo sapiens GN=OBFC2A PE=1 SV=1 - [SOSB2_HUMAN]	2.94	1	1	1	1	0.96	1.77	1.13	2.09					204	22.4	9.58
P09486	SPARC OS=Homo sapiens GN=SPARC PE=1 SV=1 - [SPRC_HUMAN]	43.56	1	11	11	60	1.40	1.33	1.32	1.29	1.12	1.10	1.11	1.08	303	34.6	4.84
Q14515	SPARC-like protein 1 OS=Homo sapiens GN=SPARCL1 PE=1 SV=2 - [SPRL1_HUMAN]	13.55	1	6	6	23	1.06	1.01	1.01	1.08	0.85	0.85	0.91	0.86	664	75.2	4.81
Q8N0X7	Spartin OS=Homo sapiens GN=SPG20 PE=1 SV=1 - [SPG20_HUMAN]	1.20	1	1	1	3					1.28	1.38	1.26	1.35	666	72.8	5.91
Q96JI7	Spatacsin OS=Homo sapiens GN=SPG11 PE=1 SV=3 - [SPTCS_HUMAN]	2.09	1	2	3	5	1.17	0.81	0.76	0.92	0.77	0.79	0.53	0.54	2443	278.7	5.97
Q9BW04	Specifically androgen-regulated gene protein OS=Homo sapiens GN=SARG PE=1 SV=2 - [SARG_HUMAN]	1.66	1	1	1	2					0.75	0.76	0.85	0.86	601	63.9	8.62
O43791	Speckle-type POZ protein OS=Homo sapiens GN=SPOP PE=1 SV=1 - [SPOP_HUMAN]	1.87	2	1	1	3	0.30	0.36	0.32	0.39					374	42.1	5.80
Q13813	Spectrin alpha chain, brain OS=Homo sapiens GN=SPTAN1 PE=1 SV=3 - [SPTA2_HUMAN]	3.56	2	5	6	6	1.25	1.25	1.26	1.26	1.15	1.18	1.00	1.03	2472	284.4	5.35
P02549	Spectrin alpha chain, erythrocyte OS=Homo sapiens GN=SPTA1 PE=1 SV=5 - [SPTA1_HUMAN]	2.15	1	3	4	5					0.66	0.67	0.69	0.69	2419	279.8	5.05
Q01082	Spectrin beta chain, brain 1 OS=Homo sapiens GN=SPTBN1 PE=1 SV=2 - [SPTB2_HUMAN]	4.36	2	6	8	13	0.86	1.18	0.74	0.98	1.13	1.39	1.52	1.87	2364	274.4	5.57
Q9H254	Spectrin beta chain, brain 3 OS=Homo sapiens GN=SPTBN4 PE=1 SV=2 - [SPTN4_HUMAN]	2.11	1	1	4	9					0.98	0.02	0.97	0.02	2564	288.8	6.01
P11277	Spectrin beta chain, erythrocyte OS=Homo sapiens GN=SPTB PE=1 SV=5 - [SPTB1_HUMAN]	4.91	1	5	7	10	1.09	1.22	1.06	1.28					2137	246.3	5.27
Q9C093	Sperm flagellar protein 2 OS=Homo sapiens GN=SPEF2 PE=1 SV=2 - [SPEF2_HUMAN]	1.54	13	1	2	2	1.58	1.30	0.76	0.63					1822	209.7	5.54
Q08648	Sperm-associated antigen 11B OS=Homo sapiens GN=SPAG11B PE=2 SV=4 - [SG11B_HUMAN]	18.45	2	1	1	1	0.58	0.47	0.63	0.52					103	11.4	11.49
Q8N0X2	Sperm-associated antigen 16 protein OS=Homo sapiens GN=SPAG16 PE=2 SV=2 - [SPG16_HUMAN]	1.74	1	1	1	2	1.68	1.33	1.80	1.43	1.04	0.89	1.13	0.97	631	70.8	6.33
Q96R06	Sperm-associated antigen 5 OS=Homo sapiens GN=SPAG5 PE=1 SV=2 - [SPAG5_HUMAN]	3.77	1	3	3	12	0.71	0.76	0.94	1.02					1193	134.3	5.00

O75391	Sperm-associated antigen 7 OS=Homo sapiens GN=SPAG7 PE=1 SV=2 - [SPAG7_HUMAN]	3.96	1	1	1	1	0.67	0.88	0.83	1.10					227	26.0	7.91
Q96SI9	Spermatid perinuclear RNA-binding protein OS=Homo sapiens GN=STRBP PE=1 SV=1 - [STRBP_HUMAN]	3.42	1	1	1	1					1.25	1.67	0.88	1.17	672	73.6	8.72
Q9BXB7	Spermatogenesis-associated protein 16 OS=Homo sapiens GN=SPATA16 PE=1 SV=3 - [SPT16_HUMAN]	3.34	1	1	1	2	0.76	0.96	0.63	0.81					569	65.2	9.14
Q8TB22	Spermatogenesis-associated protein 20 OS=Homo sapiens GN=SPATA20 PE=2 SV=3 - [SPT20_HUMAN]	5.47	1	2	2	2					0.78	0.72	1.63	1.51	786	87.8	7.43
Q86W54	Spermatogenesis-associated protein 24 OS=Homo sapiens GN=SPATA24 PE=2 SV=2 - [SPA24_HUMAN]	6.34	1	1	1	3	0.93	0.86	1.03	0.88					205	23.6	7.99
Q9BVQ7	Spermatogenesis-associated protein 5-like protein 1 OS=Homo sapiens GN=SPATA5L1 PE=1 SV=2 - [SPA5L_HUMAN]	1.99	8	1	2	2	1.14	1.19	1.01	1.07					753	80.7	8.09
Q86XZ4	Spermatogenesis-associated serine-rich protein 2 OS=Homo sapiens GN=SPATS2 PE=1 SV=1 - [SPAS2_HUMAN]	7.16	1	2	2	2	0.97	0.98	0.93	0.95					545	59.5	8.90
Q9H9C1	Spermatogenesis-defective protein 39 homolog OS=Homo sapiens GN=SPE39 PE=1 SV=1 - [SPE39_HUMAN]	7.30	2	2	3	3	0.56	0.66	0.59	0.70	0.26	0.16	0.84	0.51	493	57.0	7.40
Q86VE3	Spermidine/spermine N(1)-acetyltransferase-like protein 1 OS=Homo sapiens GN=SATL1 PE=2 SV=3 - [SATL1_HUMAN]	10.43	1	2	2	2	0.68	0.66	0.71	0.70					508	55.8	5.31
Q9NWM0	Spermine oxidase OS=Homo sapiens GN=SMOX PE=1 SV=1 - [SMOX_HUMAN]	1.26	1	1	1	1					0.43	0.45	0.44	0.47	555	61.8	5.45
P17405	Sphingomyelin phosphodiesterase OS=Homo sapiens GN=SMPD1 PE=1 SV=4 - [ASM_HUMAN]	1.75	1	1	1	1	1.07	1.21	1.15	1.30					629	69.7	7.28
O95136	Sphingosine 1-phosphate receptor 2 OS=Homo sapiens GN=S1PR2 PE=1 SV=2 - [S1PR2_HUMAN]	6.80	1	1	1	1					1.99	0.19	1.07	0.10	353	38.8	8.97
O95470	Sphingosine-1-phosphate lyase 1 OS=Homo sapiens GN=SGPL1 PE=1 SV=3 - [SGPL1_HUMAN]	1.76	1	1	1	5	1.89		1.98						568	63.5	9.16
Q8IWX5	Sphingosine-1-phosphate phosphatase 2 OS=Homo sapiens GN=SGPP2 PE=2 SV=1 - [SGPP2_HUMAN]	1.25	1	1	1	1	1.42	1.48	0.85	0.88					399	44.7	9.04
Q8N0Z3	Spindle and centriole-associated protein 1 OS=Homo sapiens GN=SPICE1 PE=1 SV=1 - [SPICE_HUMAN]	1.87	1	1	1	1					1.08	1.18	0.92	1.01	855	96.2	7.44
Q8IX90	Spindle and kinetochore-associated protein 3 OS=Homo sapiens GN=SKA3 PE=1 SV=2 - [SKA3_HUMAN]	5.34	1	1	1	3	1.26	1.45	1.17	1.35					412	46.3	5.11
Q6UVJ0	Spindle assembly abnormal protein 6 homolog OS=Homo sapiens GN=SASS6 PE=1 SV=1 - [SAS6_HUMAN]	2.44	1	1	1	1					1.17	0.93	1.21	0.97	657	74.4	7.55
Q99865	Spindlin-2A OS=Homo sapiens GN=SPIN2A PE=1 SV=3 - [SPI2A_HUMAN]	12.40	2	2	2	2	0.86	0.87	1.00	1.02					258	29.2	7.23
O75533	Splicing factor 3B subunit 1 OS=Homo sapiens GN=SF3B1 PE=1 SV=3 - [SF3B1_HUMAN]	0.46	1	1	1	1					2.75	2.57	2.32	2.16	1304	145.7	7.09
Q13435	Splicing factor 3B subunit 2 OS=Homo sapiens GN=SF3B2 PE=1 SV=2 - [SF3B2_HUMAN]	1.56	5	1	2	2	1.41	1.32	0.82	0.77					895	100.2	5.67
Q15393	Splicing factor 3B subunit 3 OS=Homo sapiens GN=SF3B3 PE=1 SV=4 - [SF3B3_HUMAN]	1.23	1	1	1	1	0.98	1.08	0.95	1.06					1217	135.5	5.26
Q9H7N4	Splicing factor, arginine/serine-rich 19 OS=Homo sapiens GN=SCAF1 PE=1 SV=3 - [SFR19_HUMAN]	0.76	1	1	1	1	0.86	0.73	0.53	0.46					1312	139.2	9.25
Q12872	Splicing factor, suppressor of white-apricot homolog OS=Homo sapiens GN=SFSWAP PE=1 SV=3 - [SFSWA_HUMAN]	0.95	1	1	1	2					1.30	1.23	1.24	1.17	951	104.8	8.05
Q9HCB6	Spondin-1 OS=Homo sapiens GN=SPON1 PE=1 SV=2 - [SPON1_HUMAN]	4.21	1	3	3	6					0.90	0.90	1.03	1.03	807	90.9	6.11
Q9BUD6	Spondin-2 OS=Homo sapiens GN=SPON2 PE=1 SV=3 - [SPON2_HUMAN]	3.63	1	1	1	3					1.33	1.15	1.28	1.11	331	35.8	5.52
Q7Z698	Sprouty-related, EVH1 domain-containing protein 2 OS=Homo sapiens GN=SPRED2 PE=1 SV=2 - [SPRE2_HUMAN]	11.24	1	1	2	4	2.11	2.36	1.38	1.56					418	47.5	6.79
Q5W111	SPRY domain-containing protein 7 OS=Homo sapiens GN=SPRYD7 PE=1 SV=2 - [SPRY7_HUMAN]	4.08	1	1	1	1					0.84	0.98	0.77	0.91	196	21.7	6.70

Q96A44	SPRY domain-containing SOCS box protein 4 OS=Homo sapiens GN=SPSB4 PE=1 SV=1 - [SPSB4_HUMAN]	6.23	1	1	1	2	1.26	1.19	1.29	1.23	1.33	1.57	1.47	1.74	273	30.2	9.35
Q56UN5	SPS1/STE20-related protein kinase YSK4 OS=Homo sapiens GN=YSK4 PE=1 SV=1 - [YSK4_HUMAN]	4.52	1	2	2	2	0.88	0.92	1.20	1.24					1328	150.4	7.05
Q15020	Squamous cell carcinoma antigen recognized by T-cells 3 OS=Homo sapiens GN=SART3 PE=1 SV=1 - [SART3_HUMAN]	3.32	1	1	1	1	0.83	0.72	1.75	1.52					963	109.9	5.57
O75563	Src kinase-associated phosphoprotein 2 OS=Homo sapiens GN=SKAP2 PE=1 SV=1 - [SKAP2_HUMAN]	10.86	1	4	4	8	0.95	1.04	0.81	0.90	1.68	1.74	1.52	1.57	359	41.2	4.69
Q14247	Src substrate cortactin OS=Homo sapiens GN=CTTN PE=1 SV=2 - [SRC8_HUMAN]	6.00	1	3	3	12					1.59	1.23	1.41	1.22	550	61.5	5.40
Q96SB4	SRSF protein kinase 1 OS=Homo sapiens GN=SRPK1 PE=1 SV=2 - [SRPK1_HUMAN]	3.82	2	2	2	2					0.70	1.09	0.44	0.68	655	74.3	6.16
Q9UPE1	SRSF protein kinase 3 OS=Homo sapiens GN=SRPK3 PE=2 SV=2 - [SRPK3_HUMAN]	2.65	1	1	1	1					0.91	0.85	1.09	1.03	567	62.0	7.25
Q9NY15	Stabilin-1 OS=Homo sapiens GN=STAB1 PE=1 SV=3 - [STAB1_HUMAN]	4.55	1	7	8	13	1.55	1.26	1.17	1.07	0.28	0.25	0.81	0.66	2570	275.3	6.49
Q8WWQ8	Stabilin-2 OS=Homo sapiens GN=STAB2 PE=1 SV=3 - [STAB2_HUMAN]	1.22	1	2	3	6	1.18	1.16	1.29	1.29	1.31	1.30	1.64	1.62	2551	276.8	6.40
Q7KZF4	Staphylococcal nuclease domain-containing protein 1 OS=Homo sapiens GN=SND1 PE=1 SV=1 - [SND1_HUMAN]	2.09	1	1	1	4	0.45	0.61	0.39	0.52					910	101.9	7.17
Q9Y3M8	StAR-related lipid transfer protein 13 OS=Homo sapiens GN=STARD13 PE=1 SV=2 - [STA13_HUMAN]	1.44	1	1	1	1	1.06		1.18						1113	124.9	7.02
Q9P2P6	StAR-related lipid transfer protein 9 OS=Homo sapiens GN=STARD9 PE=1 SV=3 - [STAR9_HUMAN]	1.02	1	2	2	2					0.95	1.22	0.77	1.00	4700	516.0	6.32
O95210	Starch-binding domain-containing protein 1 OS=Homo sapiens GN=STBD1 PE=1 SV=1 - [STBD1_HUMAN]	5.03	1	1	1	4					0.46	0.37	0.54	0.44	358	39.0	5.73
P16949	Stathmin OS=Homo sapiens GN=STMN1 PE=1 SV=3 - [STMN1_HUMAN]	6.04	2	1	1	1					2.29	2.46	1.87	2.00	149	17.3	5.97
Q9H2G2	STE20-like serine/threonine-protein kinase OS=Homo sapiens GN=SLK PE=1 SV=1 - [SLK_HUMAN]	5.10	1	4	4	4	1.30	1.07	1.74	1.44	4.24	3.93	0.79	0.62	1235	142.6	5.15
Q9C0K7	STE20-related kinase adapter protein beta OS=Homo sapiens GN=STRADB PE=1 SV=1 - [STRAB_HUMAN]	1.44	1	1	1	1					1.20	1.07	1.44	1.29	418	47.0	7.01
Q86SK9	Stearoyl-CoA desaturase 5 OS=Homo sapiens GN=SCD5 PE=2 SV=2 - [SCD5_HUMAN]	7.88	1	1	1	1	0.59		0.71						330	37.6	9.61
Q96NU1	Sterile alpha motif domain-containing protein 11 OS=Homo sapiens GN=SAMD11 PE=2 SV=3 - [SAM11_HUMAN]	4.55	1	1	1	1					1.57	1.52	1.41	1.37	681	72.7	7.59
Q8IVG5	Sterile alpha motif domain-containing protein 9-like OS=Homo sapiens GN=SAMD9L PE=1 SV=2 - [SAM9L_HUMAN]	0.95	1	1	1	3	1.46	1.42	1.76	1.73					1584	184.4	8.02
P05093	Steroid 17-alpha-hydroxylase/17,20 lyase OS=Homo sapiens GN=CYP17A1 PE=1 SV=1 - [CP17A_HUMAN]	4.53	1	3	3	10	0.81	1.23	0.66	1.00	0.76	0.76	0.74	0.80	508	57.3	8.57
O95718	Steroid hormone receptor ERR2 OS=Homo sapiens GN=ESRRB PE=1 SV=2 - [ERR2_HUMAN]	4.33	1	1	1	1					0.90	1.22	1.26	1.71	508	56.2	8.28
Q13285	Steroidogenic factor 1 OS=Homo sapiens GN=NR5A1 PE=1 SV=2 - [STF1_HUMAN]	4.34	1	1	2	2					1.17	1.20	1.06	1.08	461	51.6	7.68
P36956	Sterol regulatory element-binding protein 1 OS=Homo sapiens GN=SREBF1 PE=1 SV=2 - [SRBP1_HUMAN]	1.66	7	1	2	2					0.97	0.89	0.94	0.86	1147	121.6	8.13
Q12770	Sterol regulatory element-binding protein cleavage-activating protein OS=Homo sapiens GN=SCAP PE=1 SV=4 - [SCAP_HUMAN]	0.86	1	1	1	1	0.79	0.80	0.86	0.88					1279	139.6	6.86
Q8WXE9	Stonin-2 OS=Homo sapiens GN=STON2 PE=1 SV=1 - [STON2_HUMAN]	0.99	1	1	1	1	0.63	0.59	0.81	0.77					905	101.1	5.39
Q6ZVD7	Storkhead-box protein 1 OS=Homo sapiens GN=STOX1 PE=1 SV=2 - [STOX1_HUMAN]	8.90	1	3	3	18	0.32	0.32	0.58	0.59	0.63	0.25	1.12	0.44	989	110.9	7.78
P31948	Stress-induced-phosphoprotein 1 OS=Homo sapiens GN=STIP1 PE=1 SV=1 - [STIP1_HUMAN]	1.29	1	1	1	3					1.17	1.26	1.10	1.19	543	62.6	6.80

Q13033	Striatin-3 OS=Homo sapiens GN=STRN3 PE=1 SV=3 - [STRN3_HUMAN]	4.14	1	2	2	2	1.39	1.25	1.12	1.02	1.22	1.01	0.73	0.61	797	87.2	5.36
Q9NRL3	Striatin-4 OS=Homo sapiens GN=STRN4 PE=1 SV=2 - [STRN4_HUMAN]	5.44	1	1	1	1	0.85	0.94	0.94	1.04					753	80.5	5.40
P48061	Stromal cell-derived factor 1 OS=Homo sapiens GN=CXCL12 PE=1 SV=1 - [SDF1_HUMAN]	8.60	1	1	1	6					1.48	1.49	1.39	1.40	93	10.7	9.88
Q13586	Stromal interaction molecule 1 OS=Homo sapiens GN=STIM1 PE=1 SV=3 - [STIM1_HUMAN]	1.46	1	1	1	2					0.84	0.77	0.74	0.68	685	77.4	6.67
P08254	Stromelysin-1 OS=Homo sapiens GN=MMP3 PE=1 SV=2 - [MMP3_HUMAN]	1.89	1	1	1	2					5.47	5.97	4.55	4.96	477	53.9	6.16
A6NHR9	Structural maintenance of chromosomes flexible hinge domain-containing protein 1 OS=Homo sapiens GN=SMCHD1 PE=1 SV=2 - [SMHD1_HUMAN]	0.35	1	1	1	1							0.96		2005	226.2	7.30
Q14683	Structural maintenance of chromosomes protein 1A OS=Homo sapiens GN=SMC1A PE=1 SV=2 - [SMC1A_HUMAN]	0.89	1	1	1	1					1.03	1.30	0.89	1.12	1233	143.1	7.64
Q8NDV3	Structural maintenance of chromosomes protein 1B OS=Homo sapiens GN=SMC1B PE=1 SV=2 - [SMC1B_HUMAN]	0.89	1	1	1	1	0.20	0.35	0.71	1.24					1235	143.8	7.66
Q9NTJ3	Structural maintenance of chromosomes protein 4 OS=Homo sapiens GN=SMC4 PE=1 SV=2 - [SMC4_HUMAN]	2.17	1	2	2	3	1.14	1.24	1.06	1.17	0.59	0.59	0.66	0.66	1288	147.1	6.79
Q8IY18	Structural maintenance of chromosomes protein 5 OS=Homo sapiens GN=SMC5 PE=1 SV=2 - [SMC5_HUMAN]	6.18	1	2	3	4	0.91	0.76	0.73	0.61					1101	128.7	8.38
Q8IY92	Structure-specific endonuclease subunit SLX4 OS=Homo sapiens GN=SLX4 PE=1 SV=3 - [SLX4_HUMAN]	0.71	1	1	1	1	1.00	1.12	1.20	1.35					1834	199.9	6.06
A6NFY7	Succinate dehydrogenase assembly factor 1, mitochondrial OS=Homo sapiens GN=SDHAF1 PE=1 SV=1 - [SDHF1_HUMAN]	13.91	1	1	1	1	1.60	1.38	1.56	1.36					115	12.8	11.39
Q9NX18	Succinate dehydrogenase assembly factor 2, mitochondrial OS=Homo sapiens GN=SDHAF2 PE=1 SV=1 - [SDHF2_HUMAN]	9.04	1	1	1	1	0.77	0.92	0.85	1.03					166	19.6	6.80
P53597	Succinyl-CoA ligase [ADP/GDP-forming] subunit alpha, mitochondrial OS=Homo sapiens GN=SUCLG1 PE=1 SV=4 - [SUCA_HUMAN]	7.23	1	1	1	1					0.86	0.86	0.48	0.48	346	36.2	8.79
Q8TED4	Sugar phosphate exchanger 2 OS=Homo sapiens GN=SLC37A2 PE=2 SV=2 - [SPX2_HUMAN]	6.79	1	2	2	3	0.55	0.58	1.01	1.06	0.88	0.66	0.79	0.58	501	54.4	6.81
Q9H2B4	Sulfate anion transporter 1 OS=Homo sapiens GN=SLC26A1 PE=2 SV=2 - [S26A1_HUMAN]	3.14	1	1	1	1	0.45		0.75						701	75.0	8.10
O00391	Sulfhydryl oxidase 1 OS=Homo sapiens GN=QSOX1 PE=1 SV=3 - [QSOX1_HUMAN]	29.59	2	22	22	91	1.13	1.14	1.08	1.15	1.28	1.36	1.18	1.32	747	82.5	8.92
Q6ZRP7	Sulfhydryl oxidase 2 OS=Homo sapiens GN=QSOX2 PE=1 SV=3 - [QSOX2_HUMAN]	0.86	1	1	1	1					0.60	0.39	1.14	0.75	698	77.5	7.72
P50224	Sulfotransferase 1A3/1A4 OS=Homo sapiens GN=SULT1A3 PE=1 SV=1 - [ST1A3_HUMAN]	8.14	3	2	2	4		0.82		0.75	0.98	0.92	1.09	1.03	295	34.2	6.01
O75897	Sulfotransferase 1C4 OS=Homo sapiens GN=SULT1C4 PE=1 SV=2 - [ST1C4_HUMAN]	9.27	1	2	2	3	0.27	0.26	0.33	0.32	0.52	0.36	0.53	0.36	302	35.5	8.19
O00204	Sulfotransferase family cytosolic 2B member 1 OS=Homo sapiens GN=SULT2B1 PE=1 SV=2 - [ST2B1_HUMAN]	4.93	1	1	1	2					0.75	0.82	0.75	0.81	365	41.3	5.40
Q9UBE0	SUMO-activating enzyme subunit 1 OS=Homo sapiens GN=SAE1 PE=1 SV=1 - [SAE1_HUMAN]	2.31	1	1	1	6	0.74	0.67	0.73	0.67	1.21	1.06	1.02	0.89	346	38.4	5.30
Q9UH99	SUN domain-containing protein 2 OS=Homo sapiens GN=SUN2 PE=1 SV=3 - [SUN2_HUMAN]	2.09	1	1	1	1					0.74	0.61	0.95	0.79	717	80.3	6.73
Q8TAQ9	SUN domain-containing protein 3 OS=Homo sapiens GN=SUN3 PE=2 SV=4 - [SUN3_HUMAN]	9.80	1	2	2	6	1.84		1.20						357	40.5	7.83
P00441	Superoxide dismutase [Cu-Zn] OS=Homo sapiens GN=SOD1 PE=1 SV=2 - [SODC_HUMAN]	43.51	1	5	5	18	0.87	0.83	0.92	0.85	1.14	1.08	1.05	1.01	154	15.9	6.13
P04179	Superoxide dismutase [Mn], mitochondrial OS=Homo sapiens GN=SOD2 PE=1 SV=2 - [SODM_HUMAN]	13.51	1	3	3	11	0.59	0.58	0.54	0.54	0.79	0.82	0.84	0.86	222	24.7	8.25
O60284	Suppression of tumorigenicity 18 protein OS=Homo sapiens GN=ST18 PE=1 SV=1 - [ST18_HUMAN]	1.91	1	1	1	1	1.20	0.96	1.04	0.85					1047	115.1	6.06

Q5SSQ6	Suppressor APC domain-containing protein 1 OS=Homo sapiens GN=SAPCD1 PE=2 SV=2 - [SAPC1_HUMAN]	5.41	1	1	1	1					0.96	0.99	0.66	0.69	148	16.6	8.06
Q9Y2Z0	Suppressor of G2 allele of SKP1 homolog OS=Homo sapiens GN=SUGT1 PE=1 SV=3 - [SUGT1_HUMAN]	14.52	1	3	3	11	1.08	0.88	0.99	0.81	0.73	1.21	0.42	0.90	365	41.0	5.16
Q9BRV8	Suppressor of IKBKE 1 OS=Homo sapiens GN=SIKE1 PE=1 SV=1 - [SIKE1_HUMAN]	5.80	1	1	1	1					0.92	1.30	0.83	1.18	207	23.7	5.21
Q9NQ55	Suppressor of SWI4 1 homolog OS=Homo sapiens GN=PPAN PE=1 SV=1 - [SSF1_HUMAN]	3.59	1	1	1	15	1.13	1.18	1.10	1.09					473	53.2	10.13
Q6UWP8	Suprabasin OS=Homo sapiens GN=SBSN PE=2 SV=1 - [SBSN_HUMAN]	25.51	1	5	5	28	0.97	1.00	1.03	1.05	0.99	1.03	1.01	1.06	247	25.3	7.08
Q6UW10	Surfactant-associated protein 2 OS=Homo sapiens GN=SFTA2 PE=1 SV=1 - [SFTA2_HUMAN]	11.54	1	1	1	3	0.58	0.40	1.10	0.78	1.07	0.92	1.23	1.05	78	8.4	7.56
Q8IWZ8	SURP and G-patch domain-containing protein 1 OS=Homo sapiens GN=SUGP1 PE=1 SV=2 - [SUGP1_HUMAN]	4.19	1	1	1	2	1.08		1.07						645	72.4	7.61
Q9UH64	Susceptibility protein NSG-x OS=Homo sapiens GN=C9orf53 PE=1 SV=1 - [NSGX_HUMAN]	18.99	1	1	1	1	0.37	0.36	1.23	1.18					79	8.8	11.58
Q6UWL2	Sushi domain-containing protein 1 OS=Homo sapiens GN=SUSD1 PE=1 SV=1 - [SUSD1_HUMAN]	3.48	1	2	2	5	1.04	1.00	1.09	1.06	0.98	0.96	0.84	0.82	747	82.7	6.43
O60279	Sushi domain-containing protein 5 OS=Homo sapiens GN=SUSD5 PE=1 SV=3 - [SUSD5_HUMAN]	1.27	1	1	1	2	1.09	1.09	1.07	1.08					629	68.0	4.91
P78539	Sushi repeat-containing protein SRPX OS=Homo sapiens GN=SRPX PE=2 SV=1 - [SRPX_HUMAN]	3.23	1	1	1	2					0.50	0.42	0.58	0.49	464	51.5	8.66
O60687	Sushi repeat-containing protein SRPX2 OS=Homo sapiens GN=SRPX2 PE=1 SV=1 - [SRPX2_HUMAN]	2.80	1	1	1	3	0.97	1.08	1.06	1.17	0.51	1.03	1.07	2.17	465	52.9	7.25
Q8TER0	Sushi, nidogen and EGF-like domain-containing protein 1 OS=Homo sapiens GN=SNED1 PE=1 SV=2 - [SNED1_HUMAN]	3.75	1	4	4	5	0.56	0.87	0.62	0.85	1.16	1.00	1.15	0.99	1413	152.1	6.98
Q4LDE5	Sushi, von Willebrand factor type A, EGF and pentraxin domain-containing protein 1 OS=Homo sapiens GN=SVEP1 PE=1 SV=3 - [SVEP1_HUMAN]	5.49	1	16	16	37	1.69	1.65	1.61	1.60	1.79	2.20	1.85	2.27	3571	389.9	5.50
Q92922	SWI/SNF complex subunit SMARCC1 OS=Homo sapiens GN=SMARCC1 PE=1 SV=3 - [SMRC1_HUMAN]	1.36	1	1	1	1					1.00	1.19	0.89	1.05	1105	122.8	5.76
Q9H4L7	SWI/SNF-related matrix-associated actin-dependent regulator of chromatin subfamily A containing DEAD/H box 1 OS=Homo sapiens GN=SMARCAD1 PE=1 SV=2 - [SMRCD_HUMAN]	2.34	1	1	1	1	0.43	0.28	0.39	0.26					1026	117.3	5.55
O60264	SWI/SNF-related matrix-associated actin-dependent regulator of chromatin subfamily A member 5 OS=Homo sapiens GN=SMARCA5 PE=1 SV=1 - [SMCA5_HUMAN]	2.28	1	2	2	3	1.40	1.16	1.61	1.35					1052	121.8	8.09
Q9NZC9	SWI/SNF-related matrix-associated actin-dependent regulator of chromatin subfamily A-like protein 1 OS=Homo sapiens GN=SMARCAL1 PE=1 SV=1 - [SMAL1_HUMAN]	1.47	1	1	1	1	0.75	0.78	0.57	0.60					954	105.9	9.06
Q12824	SWI/SNF-related matrix-associated actin-dependent regulator of chromatin subfamily B member 1 OS=Homo sapiens GN=SMARCB1 PE=1 SV=2 - [SNF5_HUMAN]	5.45	1	1	1	30	1.03	0.99	1.13	1.11	1.11	1.18	1.31	1.36	385	44.1	6.23
Q96GM5	SWI/SNF-related matrix-associated actin-dependent regulator of chromatin subfamily D member 1 OS=Homo sapiens GN=SMARCD1 PE=1 SV=2 - [SMRD1_HUMAN]	7.57	1	1	1	1	1.38	1.82	1.55	2.05					515	58.2	9.25
Q9P0W2	SWI/SNF-related matrix-associated actin-dependent regulator of chromatin subfamily E member 1-related OS=Homo sapiens GN=HMG20B PE=1 SV=1 - [HM20B_HUMAN]	3.47	1	1	1	1	0.42	0.67	0.87	1.41					317	35.8	9.35
Q86XK3	Swi5-dependent recombination DNA repair protein 1 homolog OS=Homo sapiens GN=SFR1 PE=1 SV=2 - [SFR1_HUMAN]	5.71	1	1	1	1					0.71	0.59	0.92	0.76	245	28.2	6.39
Q92797	Symplekin OS=Homo sapiens GN=SYMPK PE=1 SV=2 - [SYMPK_HUMAN]	1.18	4	1	2	98	1.15	1.31	1.10	1.18	1.05	0.93	0.93	0.83	1274	141.1	6.13
Q8N4V2	Synaptic vesicle 2-related protein OS=Homo sapiens GN=SVOP PE=2 SV=1 - [SVOP_HUMAN]	2.55	1	1	1	1					0.92	0.83	1.13	1.02	548	60.7	5.78
Q7L1I2	Synaptic vesicle glycoprotein 2B OS=Homo sapiens GN=SV2B PE=2 SV=1 - [SV2B_HUMAN]	1.76	1	1	1	1	1.07	0.33	0.68	0.21					683	77.4	5.44
Q99536	Synaptic vesicle membrane protein VAT-1 homolog OS=Homo sapiens GN=VAT1 PE=1 SV=2 - [VAT1_HUMAN]	5.60	2	2	2	2	0.90	0.82	0.98	0.90					393	41.9	6.29
O15498	Synaptobrevin homolog YKT6 OS=Homo sapiens GN=YKT6 PE=1 SV=1 - [YKT6_HUMAN]	6.57	1	1	1	1					0.87	0.85	0.89	0.86	198	22.4	6.92

O43759	Synaptogyrin-1 OS=Homo sapiens GN=SYNGR1 PE=1 SV=2 - [SNG1_HUMAN]	9.87	1	1	1	1	1.36	1.21	1.31	1.17					233	25.4	4.68
O95473	Synaptogyrin-4 OS=Homo sapiens GN=SYNGR4 PE=2 SV=2 - [SNG4_HUMAN]	6.41	1	1	1	1	1.10	1.11	1.24	1.24					234	25.8	7.75
O43426	Synaptojanin-1 OS=Homo sapiens GN=SYNJ1 PE=1 SV=2 - [SYNJ1_HUMAN]	5.66	1	5	5	9	1.01	1.08	0.89	0.95	0.58	1.01	0.61	1.19	1573	173.0	7.42
Q9BX26	Synaptonemal complex protein 2 OS=Homo sapiens GN=SYCP2 PE=2 SV=2 - [SYCP2_HUMAN]	0.98	1	1	1	2					0.29	0.15	0.81	0.42	1530	175.5	8.85
Q8IZU3	Synaptonemal complex protein 3 OS=Homo sapiens GN=SYCP3 PE=1 SV=1 - [SYCP3_HUMAN]	2.97	1	1	1	1					0.51	1.45	0.29	0.82	236	27.7	9.23
Q8N3V7	Synaptopodin OS=Homo sapiens GN=SYNPO PE=1 SV=2 - [SYNPO_HUMAN]	3.34	1	1	1	2					0.70	0.68	0.79	0.78	929	99.4	8.72
O00161	Synaptosomal-associated protein 23 OS=Homo sapiens GN=SNAP23 PE=1 SV=1 - [SNP23_HUMAN]	4.74	1	1	1	3					3.71	2.97	3.45	2.75	211	23.3	5.01
P60880	Synaptosomal-associated protein 25 OS=Homo sapiens GN=SNAP25 PE=1 SV=1 - [SNP25_HUMAN]	3.40	1	1	1	2	0.79	0.76	0.76	0.73					206	23.3	4.77
O95721	Synaptosomal-associated protein 29 OS=Homo sapiens GN=SNAP29 PE=1 SV=1 - [SNP29_HUMAN]	4.65	1	1	1	3					1.82	2.17	1.68	1.99	258	29.0	5.81
Q9BT88	Synaptotagmin-11 OS=Homo sapiens GN=SYT11 PE=1 SV=2 [SYT11_HUMAN]	3.94	1	1	1	1					1.07	1.04	1.00	0.97	431	48.3	9.04
Q9BQS2	Synaptotagmin-15 OS=Homo sapiens GN=SYT15 PE=1 SV=3 - [SYT15_HUMAN]	6.65	1	1	1	6			0.92						421	47.3	7.40
Q9BSW7	Synaptotagmin-17 OS=Homo sapiens GN=SYT17 PE=1 SV=1 - [SYT17_HUMAN]	2.95	1	1	1	1					0.93	0.69	0.77	0.57	474	53.8	7.50
Q96C24	Synaptotagmin-like protein 4 OS=Homo sapiens GN=SYTL4 PE=1 SV=2 - [SYTL4_HUMAN]	6.41	1	2	2	4					1.67	1.44	1.20	1.03	671	76.0	8.98
Q8TDW5	Synaptotagmin-like protein 5 OS=Homo sapiens GN=SYTL5 PE=1 SV=1 - [SYTL5_HUMAN]	2.88	1	1	1	1					1.86	1.53	1.47	1.21	730	81.5	8.78
P31431	Syndecan-4 OS=Homo sapiens GN=SDC4 PE=1 SV=2 - [SDC4_HUMAN]	7.07	1	1	1	7	1.65	1.40	1.60	1.37	1.31	1.15	1.31	1.15	198	21.6	4.50
Q9UMZ2	Synergin gamma OS=Homo sapiens GN=SYNRG PE=1 SV=2 - [SYNRG_HUMAN]	4.11	1	4	4	4	0.91	0.93	0.79	0.80	0.90	1.08	0.84	1.00	1314	140.6	5.03
Q9Y6H5	Synphilin-1 OS=Homo sapiens GN=SNCAIP PE=1 SV=2 - [SNCAP_HUMAN]	1.63	1	2	2	2	1.06	1.04	1.13	1.11	0.76	0.91	0.76	0.90	919	100.3	6.34
O75558	Syntaxin-11 OS=Homo sapiens GN=STX11 PE=2 SV=1 - [STX11_HUMAN]	3.14	1	1	1	3					6.98	8.02	6.26	7.18	287	33.2	6.55
O14662	Syntaxin-16 OS=Homo sapiens GN=STX16 PE=1 SV=3 - [STX16_HUMAN]	9.23	1	1	1	2	0.79	0.65	0.96	0.80					325	37.0	6.11
P56962	Syntaxin-17 OS=Homo sapiens GN=STX17 PE=1 SV=2 - [STX17_HUMAN]	6.62	1	1	1	1					1.10	1.26	0.81	0.92	302	33.4	6.57
Q9P2W9	Syntaxin-18 OS=Homo sapiens GN=STX18 PE=1 SV=1 - [STX18_HUMAN]	2.39	1	1	1	1	0.50	0.63	0.93	1.16					335	38.6	5.49
O15400	Syntaxin-7 OS=Homo sapiens GN=STX7 PE=1 SV=4 - [STX7_HUMAN]	23.37	1	5	5	12					1.06	1.05	1.03	1.02	261	29.8	5.55
P61764	Syntaxin-binding protein 1 OS=Homo sapiens GN=STXBP1 PE=1 SV=1 - [STXB1_HUMAN]	5.05	1	2	2	13	0.88	0.81	0.67	0.64	0.97	0.84	1.10	0.95	594	67.5	6.96
Q15833	Syntaxin-binding protein 2 OS=Homo sapiens GN=STXBP2 PE=1 SV=2 - [STXB2_HUMAN]	2.87	1	1	1	4	1.11	1.13	1.09	1.11	0.70	0.81	0.58	0.67	593	66.4	6.55
O00560	Syntenin-1 OS=Homo sapiens GN=SDCBP PE=1 SV=1 - [SDCB1_HUMAN]	3.36	1	1	1	1					0.94	0.86	1.01	0.93	298	32.4	7.53
Q16650	T-box brain protein 1 OS=Homo sapiens GN=TBR1 PE=1 SV=1 - [TBR1_HUMAN]	1.32	2	1	1	1	0.77	1.14	0.84	1.26					682	74.0	7.33
Q9UMR3	T-box transcription factor TBX20 OS=Homo sapiens GN=TBX20 PE=1 SV=4 - [TBX20_HUMAN]	5.82	1	2	2	3	0.84	0.71	0.86	0.73	0.93	0.84	0.79	0.71	447	49.2	7.71

O15119	T-box transcription factor TBX3 OS=Homo sapiens GN=TBX3 PE=1 SV=4 - [TBX3_HUMAN]	4.17	1	1	1	1	1.14	1.49	1.04	1.36					743	79.3	8.16
Q8N103	T-cell activation Rho GTPase-activating protein OS=Homo sapiens GN=TAGAP PE=2 SV=1 - [TAGAP_HUMAN]	1.23	7	1	2	6					1.20	0.57	0.81	0.38	731	80.7	6.47
Q96H15	T-cell immunoglobulin and mucin domain-containing protein 4 OS=Homo sapiens GN=TIMD4 PE=1 SV=2 - [TIMD4_HUMAN]	2.38	1	1	1	1	1.05	0.85	1.02	0.82					378	41.6	6.10
P56279	T-cell leukemia/lymphoma protein 1A OS=Homo sapiens GN=TCL1A PE=1 SV=1 - [TCL1A_HUMAN]	5.26	1	1	1	1					1.60	1.35	###	11.55	114	13.5	5.11
P04436	T-cell receptor alpha chain V region HPB-MLT (Fragment) OS=Homo sapiens PE=2 SV=1 - [TVA1_HUMAN]	14.50	1	1	1	3	0.77	0.65	0.75	0.64	1.23	1.01	1.34	1.09	131	14.8	5.48
P17987	T-complex protein 1 subunit alpha OS=Homo sapiens GN=TCP1 PE=1 SV=1 - [TCPA_HUMAN]	11.87	4	3	4	100	1.37	1.53	1.40	1.58	1.33	0.95	1.07	0.77	556	60.3	6.11
P48643	T-complex protein 1 subunit epsilon OS=Homo sapiens GN=CCT5 PE=1 SV=1 - [TCPE_HUMAN]	4.62	1	1	1	1	1.09	1.09	1.10	1.11					541	59.6	5.66
Q99832	T-complex protein 1 subunit eta OS=Homo sapiens GN=CCT7 PE=1 SV=2 - [TCPH_HUMAN]	2.03	1	1	1	1					0.68	0.50	0.99	0.72	543	59.3	7.65
P49368	T-complex protein 1 subunit gamma OS=Homo sapiens GN=CCT3 PE=1 SV=4 - [TCPG_HUMAN]	3.49	1	2	2	3					0.76	0.63	0.72	0.60	545	60.5	6.49
Q8TDR4	T-complex protein 10A homolog 2 OS=Homo sapiens GN=TCP10L PE=1 SV=1 - [TCP1L_HUMAN]	6.05	1	1	1	1					0.82	0.81	0.81	0.81	215	23.8	9.67
Q9NUJ3	T-complex protein 11-like protein 1 OS=Homo sapiens GN=TCP11L1 PE=1 SV=1 - [T11L1_HUMAN]	4.52	1	1	1	2					1.29	1.36	1.17	1.23	509	57.0	5.59
Q96NA8	t-SNARE domain-containing protein 1 OS=Homo sapiens GN=TSNARE1 PE=2 SV=2 - [TSNA1_HUMAN]	1.95	1	1	1	1					1.23	1.21	1.24	1.22	513	55.9	9.01
Q9Y6J9	TAF6-like RNA polymerase II p300/CBP-associated factor-associated factor 65 kDa subunit 6L OS=Homo sapiens GN=TAF6L PE=1 SV=1 - [TAF6L_HUMAN]	2.41	1	1	1	1	1.85	1.85	2.05	2.05					622	67.8	8.97
Q9Y490	Talin-1 OS=Homo sapiens GN=TLN1 PE=1 SV=3 - [TLN1_HUMAN]	54.82	4	104	104	851	0.62	0.57	0.59	0.56	1.05	1.02	0.99	0.99	2541	269.6	6.07
O95271	Tankyrase-1 OS=Homo sapiens GN=TNKS PE=1 SV=2 - [TNKS1_HUMAN]	0.68	1	1	1	1	1.05	1.21	1.05	1.21					1327	142.0	7.05
Q7Z7G0	Target of Nesh-SH3 OS=Homo sapiens GN=ABI3BP PE=1 SV=1 - [TARSH_HUMAN]	4.00	1	4	4	11	0.98	1.04	1.02	1.07	0.85	0.88	0.84	0.87	1075	118.6	9.44
Q9BPZ7	Target of rapamycin complex 2 subunit MAPKAP1 OS=Homo sapiens GN=MAPKAP1 PE=1 SV=2 - [SIN1_HUMAN]	2.87	1	1	1	1					1.28	1.32	1.25	1.28	522	59.1	7.55
P59539	Taste receptor type 2 member 45 OS=Homo sapiens GN=TAS2R45 PE=2 SV=1 - [T2R45_HUMAN]	3.68	2	1	1	2					1.22	1.34	1.13	1.24	299	34.3	10.04
P59540	Taste receptor type 2 member 46 OS=Homo sapiens GN=TAS2R46 PE=2 SV=2 - [T2R46_HUMAN]	11.33	1	1	1	1					1.04	0.79	1.13	0.86	309	35.5	9.76
Q9NYW2	Taste receptor type 2 member 8 OS=Homo sapiens GN=TAS2R8 PE=1 SV=1 - [TA2R8_HUMAN]	1.62	1	1	1	1	0.88	0.99	0.84	0.95					309	35.9	9.61
P82094	TATA element modulatory factor OS=Homo sapiens GN=TMF1 PE=1 SV=2 - [TMF1_HUMAN]	1.10	1	1	1	1					1.39	1.05	1.37	1.04	1093	122.8	4.92
O14981	TATA-binding protein-associated factor 172 OS=Homo sapiens GN=BTAF1 PE=1 SV=2 - [BTAF1_HUMAN]	0.43	1	1	1	2	0.83	0.88	0.81	0.87					1849	206.8	6.52
Q5TCY1	Tau-tubulin kinase 1 OS=Homo sapiens GN=TTBK1 PE=1 SV=2 - [TTBK1_HUMAN]	3.41	1	2	2	2	0.61	0.57	0.72	0.67					1321	142.6	5.60
Q9BXI6	TBC1 domain family member 10A OS=Homo sapiens GN=TBC1D10A PE=1 SV=1 - [TB10A_HUMAN]	6.69	1	1	1	1					0.70	0.61	0.47	0.40	508	57.1	8.44
Q4KMP7	TBC1 domain family member 10B OS=Homo sapiens GN=TBC1D10B PE=1 SV=3 - [TB10B_HUMAN]	2.60	1	1	1	3					1.03	1.15	1.18	1.32	808	87.1	9.19
Q96BZ9	TBC1 domain family member 20 OS=Homo sapiens GN=TBC1D20 PE=1 SV=1 - [TBC20_HUMAN]	1.74	1	1	1	1							0.71	2.09	403	45.8	6.86
Q9BYX2	TBC1 domain family member 2A OS=Homo sapiens GN=TBC1D2 PE=1 SV=3 - [TBD2A_HUMAN]	0.86	1	1	1	2	1.33	1.55	1.30	1.52	1.57	1.55	1.27	1.26	928	105.3	6.58

Q92609	TBC1 domain family member 5 OS=Homo sapiens GN=TBC1D5 PE=1 SV=1 - [TBCD5_HUMAN]	1.64	1	1	1	1	1.67	1.89	1.64	1.87					795	88.9	6.54
O95759	TBC1 domain family member 8 OS=Homo sapiens GN=TBC1D8 PE=1 SV=3 - [TBCD8_HUMAN]	1.67	1	1	1	3	0.15	0.12	0.23	0.20	0.95	0.94	0.72	0.72	1140	130.8	5.52
Q0IIM8	TBC1 domain family member 8B OS=Homo sapiens GN=TBC1D8B PE=2 SV=2 - [TBC8B_HUMAN]	1.52	1	1	1	1					2.00	2.85	1.96	2.79	1120	128.6	5.95
Q66K14	TBC1 domain family member 9B OS=Homo sapiens GN=TBC1D9B PE=1 SV=3 - [TBC9B_HUMAN]	0.72	1	1	1	1	1.12	1.01	1.11	1.01					1250	140.4	5.25
P0C1Z6	TCF3 fusion partner OS=Homo sapiens GN=TFPT PE=1 SV=1 - [TFPT_HUMAN]	7.51	1	1	1	1	0.99	1.14	0.89	1.03					253	28.3	5.21
Q6ZSZ6	Teashirt homolog 1 OS=Homo sapiens GN=TSHZ1 PE=2 SV=2 - [TSH1_HUMAN]	3.99	1	2	2	6					0.65	1.25	0.52	0.99	1077	117.8	7.06
O15040	Tectonin beta-propeller repeat-containing protein 2 OS=Homo sapiens GN=TECPR2 PE=1 SV=4 - [TCPR2_HUMAN]	0.64	1	1	1	2					0.75	0.67	0.72	0.64	1411	153.8	5.55
Q96M29	Tektin-5 OS=Homo sapiens GN=TEKT5 PE=2 SV=1 - [TEKT5_HUMAN]	2.68	1	1	1	1	0.57	0.51	0.77	0.69					485	56.3	7.14
Q99973	Telomerase protein component 1 OS=Homo sapiens GN=TEP1 PE=1 SV=2 - [TEP1_HUMAN]	1.29	1	2	2	2					0.86	0.79	0.94	0.68	2627	290.3	7.97
Q9Y4R8	Telomere length regulation protein TEL2 homolog OS=Homo sapiens GN=TELO2 PE=1 SV=2 - [TELO2_HUMAN]	0.84	1	1	1	2					0.38	0.42	0.36	0.39	837	91.7	5.76
Q5UIP0	Telomere-associated protein RIF1 OS=Homo sapiens GN=RIF1 PE=1 SV=2 - [RIF1_HUMAN]	1.38	1	2	2	3	1.32	4.98	1.08	4.10					2472	274.3	5.52
P24821	Tenascin OS=Homo sapiens GN=TNC PE=1 SV=3 - [TENA_HUMAN]	9.86	1	16	16	38	0.92	0.95	0.89	0.89					2201	240.7	4.89
Q9UQP3	Tenascin-N OS=Homo sapiens GN=TNN PE=1 SV=2 - [TENN_HUMAN]	7.16	1	6	6	12	0.95	0.87	0.96	0.90	0.55	0.66	0.54	0.65	1299	143.9	5.63
Q92752	Tenascin-R OS=Homo sapiens GN=TNR PE=1 SV=3 - [TENR_HUMAN]	4.79	1	3	4	6					1.19	1.05	0.95	0.83	1358	149.5	4.82
P22105	Tenascin-X OS=Homo sapiens GN=TNXB PE=1 SV=3 - [TENX_HUMAN]	15.27	2	36	36	114	0.98	0.99	0.97	1.00	0.91	0.97	0.87	0.91	4289	464.0	5.34
Q9NT68	Teneurin-2 OS=Homo sapiens GN=ODZ2 PE=1 SV=3 - [TEN2_HUMAN]	1.66	1	2	2	3					2.64	2.94	2.21	2.46	2774	307.6	6.68
Q9P273	Teneurin-3 OS=Homo sapiens GN=ODZ3 PE=2 SV=3 - [TEN3_HUMAN]	2.70	1	3	4	5					0.80	0.47	0.58	0.42	2699	300.8	6.42
Q9H2S6	Tenomodulin OS=Homo sapiens GN=TNMD PE=2 SV=1 - [TNMD_HUMAN]	4.42	1	1	1	1					1.02	1.16	1.02	1.16	317	37.1	5.19
Q9HBL0	Tensin-1 OS=Homo sapiens GN=TNS1 PE=1 SV=2 - [TENS1_HUMAN]	1.21	1	1	1	1	0.97	0.97	0.97	0.98					1735	185.6	7.75
Q8IZW8	Tensin-4 OS=Homo sapiens GN=TNS4 PE=1 SV=3 - [TENS4_HUMAN]	1.82	1	1	1	1	0.86	0.79	1.10	1.01					715	76.7	7.34
Q5TAX3	Terminal uridylyltransferase 4 OS=Homo sapiens GN=ZCCHC11 PE=1 SV=3 - [TUT4_HUMAN]	1.40	1	1	1	2	1.03	1.15	1.43	1.60	0.67	0.95	0.58	0.82	1644	185.0	7.97
Q8N9V7	Testis- and ovary-specific PAZ domain-containing protein 1 OS=Homo sapiens GN=TOPAZ1 PE=2 SV=3 - [TOPZ1_HUMAN]	2.36	1	2	2	2	1.52	1.10	0.74	0.55	2.92	3.19	3.18	3.47	1692	190.8	7.87
Q9NXF1	Testis-expressed sequence 10 protein OS=Homo sapiens GN=TEX10 PE=1 SV=2 - [TEX10_HUMAN]	1.40	1	1	1	2	0.72	0.71	0.67	0.66					929	105.6	9.36
Q9BXU3	Testis-expressed sequence 13A protein OS=Homo sapiens GN=TEX13A PE=2 SV=1 - [TX13A_HUMAN]	2.69	1	1	1	1					1.31	1.30	1.08	1.08	409	45.6	5.53
Q9BXT5	Testis-expressed sequence 15 protein OS=Homo sapiens GN=TEX15 PE=1 SV=2 - [TEX15_HUMAN]	2.33	5	3	5	7	1.29	0.89	0.81	0.79					2789	315.1	6.19
Q8IWB9	Testis-expressed sequence 2 protein OS=Homo sapiens GN=TEX2 PE=1 SV=2 - [TEX2_HUMAN]	7.19	1	2	2	2	0.47	0.17	0.86	0.31					1127	125.2	6.01
Q8N6V9	Testis-expressed sequence 9 protein OS=Homo sapiens GN=TEX9 PE=2 SV=1 - [TEX9_HUMAN]	4.60	1	1	1	2	1.01	0.95	0.88	0.83					391	44.8	6.60

Q9Y6F8	Testis-specific chromodomain protein Y 1 OS=Homo sapiens GN=CDY1 PE=1 SV=1 - [CDY1_HUMAN]	4.63	1	1	1	1	0.61	0.53	0.96	0.83					540	60.4	9.10
Q9BZW7	Testis-specific gene 10 protein OS=Homo sapiens GN=TSGA10 PE=1 SV=1 - [TSG10_HUMAN]	3.01	1	2	2	5					0.95	1.04	1.10	1.13	698	81.4	5.97
Q3SY00	Testis-specific protein 10-interacting protein OS=Homo sapiens GN=TSGA10IP PE=2 SV=1 - [T10IP_HUMAN]	2.70	1	1	1	2	0.75	0.82	0.78	0.85					556	62.3	10.05
Q96PF2	Testis-specific serine/threonine-protein kinase 2 OS=Homo sapiens GN=TSSK2 PE=1 SV=2 - [TSSK2_HUMAN]	3.91	1	1	1	1					0.50	0.81	0.83	1.32	358	40.9	8.84
Q6SA08	Testis-specific serine/threonine-protein kinase 4 OS=Homo sapiens GN=TSSK4 PE=1 SV=1 - [TSSK4_HUMAN]	2.74	1	1	1	2					1.18	1.44	0.93	1.10	328	37.4	8.57
A2RUG3	Testis-specific XK-related protein, Y-linked 2 OS=Homo sapiens GN=XKRY2 PE=2 SV=2 - [XKRY2_HUMAN]	24.79	2	1	1	1	0.63	0.85	0.85	1.15					117	13.0	8.68
Q9H0U9	Testis-specific Y-encoded-like protein 1 OS=Homo sapiens GN=TSPYL1 PE=1 SV=3 - [TSYL1_HUMAN]	5.03	1	2	2	2	1.15	0.60	0.95	0.50	0.89	0.79	0.96	0.85	437	49.2	5.45
Q8N831	Testis-specific Y-encoded-like protein 6 OS=Homo sapiens GN=TSPYL6 PE=2 SV=1 - [TSYL6_HUMAN]	2.44	1	1	1	1					1.12	0.75	1.02	0.68	410	45.8	5.68
P05452	Tetranectin OS=Homo sapiens GN=CLEC3B PE=1 SV=3 - [TETN_HUMAN]	77.23	1	13	13	180	0.81	0.82	0.82	0.83	1.16	0.94	1.14	0.98	202	22.5	5.67
O60636	Tetraspanin-2 OS=Homo sapiens GN=TSPAN2 PE=2 SV=2 - [TSN2_HUMAN]	2.71	1	1	1	1	0.98	1.03	0.92	0.96					221	24.1	7.77
Q96N46	Tetratricopeptide repeat protein 14 OS=Homo sapiens GN=TTC14 PE=1 SV=1 - [TTC14_HUMAN]	2.47	1	2	2	3	0.74	0.60	0.84	0.68					770	88.3	8.63
Q8NEE8	Tetratricopeptide repeat protein 16 OS=Homo sapiens GN=TTC16 PE=2 SV=2 - [TTC16_HUMAN]	3.67	1	2	2	2	0.76	0.74	0.86	0.83					873	98.2	9.01
Q96AE7	Tetratricopeptide repeat protein 17 OS=Homo sapiens GN=TTC17 PE=1 SV=1 - [TTC17_HUMAN]	1.58	2	1	2	3	0.12	0.06	0.23	0.12					1141	129.5	6.58
Q8NDW8	Tetratricopeptide repeat protein 21A OS=Homo sapiens GN=TTC21A PE=2 SV=3 - [TT21A_HUMAN]	1.59	1	2	2	2	0.61	0.64	0.68	0.72	1.12	1.52	0.82	1.12	1320	150.8	7.24
Q7Z4L5	Tetratricopeptide repeat protein 21B OS=Homo sapiens GN=TTC21B PE=1 SV=2 - [TT21B_HUMAN]	0.53	1	1	1	1					0.59	0.72	0.55	0.67	1316	150.8	6.96
Q6PF05	Tetratricopeptide repeat protein 23-like OS=Homo sapiens GN=TTC23L PE=2 SV=2 - [TT23L_HUMAN]	4.99	1	1	1	1	0.84	0.83	0.99	0.97					361	40.8	7.24
A2A3L6	Tetratricopeptide repeat protein 24 OS=Homo sapiens GN=TTC24 PE=2 SV=1 - [TTC24_HUMAN]	1.89	1	1	1	1					1.85	0.33			582	63.4	9.25
Q96AY4	Tetratricopeptide repeat protein 28 OS=Homo sapiens GN=TTC28 PE=1 SV=4 - [TTC28_HUMAN]	1.65	1	2	2	2	1.18	1.04	1.15	1.05					2481	270.7	6.89
Q6PID6	Tetratricopeptide repeat protein 33 OS=Homo sapiens GN=TTC33 PE=1 SV=2 - [TTC33_HUMAN]	6.49	1	1	1	1					1.39	2.18	0.80	1.25	262	29.4	5.44
A8MYJ7	Tetratricopeptide repeat protein 34 OS=Homo sapiens GN=TTC34 PE=2 SV=2 - [TTC34_HUMAN]	4.42	1	1	1	2	1.98	1.22	0.96	0.73					566	60.9	7.33
A6NLP5	Tetratricopeptide repeat protein 36 OS=Homo sapiens GN=TTC36 PE=2 SV=1 - [TTC36_HUMAN]	7.41	1	1	1	1					1.22	1.86	1.42	2.16	189	20.9	5.12
Q6PGP7	Tetratricopeptide repeat protein 37 OS=Homo sapiens GN=TTC37 PE=1 SV=1 - [TTC37_HUMAN]	1.79	1	1	2	2	0.69	0.74	0.85	0.92					1564	175.4	7.53
Q5R3I4	Tetratricopeptide repeat protein 38 OS=Homo sapiens GN=TTC38 PE=1 SV=1 - [TTC38_HUMAN]	4.26	1	1	1	1					1.06	0.76	1.21	0.86	469	52.8	5.99
Q8IYW2	Tetratricopeptide repeat protein 40 OS=Homo sapiens GN=TTC40 PE=2 SV=3 - [TTC40_HUMAN]	2.98	5	4	4	9	1.23	1.39	1.26	1.54	1.02	1.09	0.87	0.93	2715	303.3	7.36
Q8N0Z6	Tetratricopeptide repeat protein 5 OS=Homo sapiens GN=TTC5 PE=1 SV=2 - [TTC5_HUMAN]	3.41	1	1	1	1	1.45	1.21	1.16	0.98					440	48.9	6.48
Q9ULT0	Tetratricopeptide repeat protein 7A OS=Homo sapiens GN=TTC7A PE=1 SV=3 - [TTC7A_HUMAN]	2.33	1	2	2	2	1.09	1.29	1.15	1.38					858	96.1	6.46
Q6P2S7	Tetratricopeptide repeat protein GNN OS=Homo sapiens GN=GNN PE=2 SV=3 - [GNN_HUMAN]	1.67	1	1	1	5					0.99	0.72	1.16	1.00	1318	151.6	8.25

Q8N5C8	TGF-beta-activated kinase 1 and MAP3K7-binding protein 3 OS=Homo sapiens GN=TAB3 PE=1 SV=2 - [TAB3_HUMAN]	1.40	1	1	1	3					1.23	1.06	1.18	1.06	712	78.6	8.46
Q8WY91	THAP domain-containing protein 4 OS=Homo sapiens GN=THAP4 PE=1 SV=2 - [THAP4_HUMAN]	1.91	6	1	2	96	0.87	1.25	0.71	1.02					577	62.9	9.28
Q7Z6K1	THAP domain-containing protein 5 OS=Homo sapiens GN=THAP5 PE=1 SV=2 - [THAP5_HUMAN]	4.05	1	1	1	1			0.27	0.13					395	45.4	6.71
Q5T1C6	Thioesterase superfamily member 4 OS=Homo sapiens GN=THEM4 PE=1 SV=1 - [THEM4_HUMAN]	3.33	1	1	1	1					0.93	0.89	0.89	0.85	240	27.1	8.28
Q14894	Thiomorpholine-carboxylate dehydrogenase OS=Homo sapiens GN=CRYM PE=1 SV=1 - [CRYM_HUMAN]	4.14	1	1	1	10	1.44	1.54	1.46	1.57	1.53	1.64	1.32	1.47	314	33.8	5.14
Q6PKC3	Thioredoxin domain-containing protein 11 OS=Homo sapiens GN=TXNDC11 PE=1 SV=2 - [TXD11_HUMAN]	2.74	1	1	1	1	1.16	1.34	1.23	1.43					985	110.5	6.55
O95881	Thioredoxin domain-containing protein 12 OS=Homo sapiens GN=TXNDC12 PE=1 SV=1 - [TXD12_HUMAN]	5.23	1	1	1	1						0.68		0.73	172	19.2	5.40
Q9P2K2	Thioredoxin domain-containing protein 16 OS=Homo sapiens GN=TXNDC16 PE=2 SV=4 - [TXD16_HUMAN]	2.91	1	1	1	1					0.76	0.95	0.80	1.00	825	93.5	5.21
Q9BRA2	Thioredoxin domain-containing protein 17 OS=Homo sapiens GN=TXNDC17 PE=1 SV=1 - [TXD17_HUMAN]	11.38	1	1	1	2					1.28	1.14	1.30	1.16	123	13.9	5.52
Q8NBS9	Thioredoxin domain-containing protein 5 OS=Homo sapiens GN=TXNDC5 PE=1 SV=2 - [TXND5_HUMAN]	12.96	1	2	2	2					0.97	1.17	0.75	1.21	432	47.6	5.97
P10599	Thioredoxin OS=Homo sapiens GN=TXN PE=1 SV=3 - [THIO_HUMAN]	49.52	1	5	5	34	1.11	0.99	0.95	0.84	1.03	0.93	0.99	0.95	105	11.7	4.92
P30048	Thioredoxin-dependent peroxide reductase, mitochondrial OS=Homo sapiens GN=PRDX3 PE=1 SV=3 - [PRDX3_HUMAN]	6.64	1	1	1	1					1.14	0.45	1.11	0.44	256	27.7	7.78
Q9H1E5	Thioredoxin-related transmembrane protein 4 OS=Homo sapiens GN=TMX4 PE=1 SV=1 - [TMX4_HUMAN]	4.87	1	1	1	1	0.87	0.81	1.03	0.98					349	38.9	4.37
Q8NI27	THO complex subunit 2 OS=Homo sapiens GN=THOC2 PE=1 SV=2 - [THOC2_HUMAN]	1.57	1	1	1	1	1.59	1.07	1.24	0.83					1593	182.7	8.44
P26639	Threonine--tRNA ligase, cytoplasmic OS=Homo sapiens GN=TARS PE=1 SV=3 - [SYTC_HUMAN]	2.21	2	1	1	5	1.21	1.10	1.22	1.12	0.78	0.80	0.79	0.82	723	83.4	6.67
P07996	Thrombospondin-1 OS=Homo sapiens GN=THBS1 PE=1 SV=2 - [TSP1_HUMAN]	43.76	1	38	41	582	1.13	1.15	1.15	1.14	0.97	1.02	1.02	1.02	1170	129.3	4.94
P35442	Thrombospondin-2 OS=Homo sapiens GN=THBS2 PE=1 SV=2 - [TSP2_HUMAN]	4.27	1	2	4	48	0.65	0.61	0.67	0.63	0.85	0.93	0.89	0.97	1172	129.9	4.83
P49746	Thrombospondin-3 OS=Homo sapiens GN=THBS3 PE=1 SV=1 - [TSP3_HUMAN]	2.72	1	1	3	14					1.21	0.77	1.67	1.06	956	104.1	4.65
P35443	Thrombospondin-4 OS=Homo sapiens GN=THBS4 PE=1 SV=2 - [TSP4_HUMAN]	14.98	1	6	8	44	0.63	0.59	0.64	0.61	0.90	0.94	0.99	0.97	961	105.8	4.68
P24557	Thromboxane-A synthase OS=Homo sapiens GN=TBXAS1 PE=1 SV=3 - [THAS_HUMAN]	3.75	1	1	1	8	0.95	0.95	0.94	0.89					533	60.5	7.62
Q9NXG2	THUMP domain-containing protein 1 OS=Homo sapiens GN=THUMPD1 PE=1 SV=2 - [THUM1_HUMAN]	4.25	1	1	1	1	1.26	1.12	1.24	1.11					353	39.3	7.88
P04216	Thy-1 membrane glycoprotein OS=Homo sapiens GN=THY1 PE=1 SV=2 - [THY1_HUMAN]	19.88	1	2	2	3					1.02	1.01	2.11	2.08	161	17.9	8.73
P19971	Thymidine phosphorylase OS=Homo sapiens GN=TYMP PE=1 SV=2 - [TYPH_HUMAN]	3.53	1	1	1	2					0.58	0.74	0.50	0.64	482	49.9	5.53
P62328	Thymosin beta-4 OS=Homo sapiens GN=TMSB4X PE=1 SV=2 - [TYB4_HUMAN]	70.45	2	6	6	47					2.03	1.99	1.93	1.95	44	5.0	5.06
Q6YHU6	Thyroid adenoma-associated protein OS=Homo sapiens GN=THADA PE=1 SV=1 - [THADA_HUMAN]	1.69	1	2	2	2	0.62	0.79	0.69	0.89	0.68	1.03	0.46	0.70	1953	219.5	6.06
P10827	Thyroid hormone receptor alpha OS=Homo sapiens GN=THRA PE=1 SV=1 - [THA_HUMAN]	4.08	1	1	1	2					0.61	1.02	0.64	1.08	490	54.8	6.86
Q15643	Thyroid receptor-interacting protein 11 OS=Homo sapiens GN=TRIP11 PE=1 SV=3 - [TRIPB_HUMAN]	4.35	1	5	6	10	1.80	2.43	1.15	1.56	1.29	1.30	1.27	1.24	1979	227.4	5.26

Q10587	Thyrotroph embryonic factor OS=Homo sapiens GN=TEF PE=2 SV=3 - [TEF_HUMAN]	10.89	1	1	1	2	1.14	1.21	1.02	1.09					303	33.2	6.05
P01222	Thyrotropin subunit beta OS=Homo sapiens GN=TSHB PE=1 SV=2 - [TSHB_HUMAN]	29.71	1	2	2	2					0.65	0.09	0.52	0.07	138	15.6	7.69
Q9UKU6	Thyrotropin-releasing hormone-degrading ectoenzyme OS=Homo sapiens GN=TRHDE PE=2 SV=1 - [TRHDE_HUMAN]	1.27	1	1	1	2					0.89	0.76	0.90	0.32	1024	116.9	6.99
P05543	Thyroxine-binding globulin OS=Homo sapiens GN=SERPINA7 PE=1 SV=2 - [THBG_HUMAN]	54.22	1	23	23	354	1.25	1.30	1.23	1.30	1.04	1.05	0.95	0.99	415	46.3	6.30
Q6NT04	Tigger transposable element-derived protein 7 OS=Homo sapiens GN=TIGD7 PE=2 SV=1 - [TIGD7_HUMAN]	1.46	1	1	1	3					1.60	1.55	1.49	1.44	549	63.2	8.75
Q07157	Tight junction protein ZO-1 OS=Homo sapiens GN=TJP1 PE=1 SV=3 - [ZO1_HUMAN]	1.37	1	1	1	1	0.76	0.79	0.77	0.81					1748	195.3	6.70
Q9UDY2	Tight junction protein ZO-2 OS=Homo sapiens GN=TJP2 PE=1 SV=2 - [ZO2_HUMAN]	3.28	1	3	3	14					1.45	1.45	1.35	1.35	1190	133.9	7.40
Q86XR7	TIR domain-containing adapter molecule 2 OS=Homo sapiens GN=TICAM2 PE=1 SV=1 - [TCAM2_HUMAN]	9.36	1	1	1	1	1.80	0.90	1.64	0.82					235	26.9	5.07
P04066	Tissue alpha-L-fucosidase OS=Homo sapiens GN=FUCA1 PE=1 SV=4 - [FUCO_HUMAN]	3.65	1	1	2	9	1.16	1.04	1.08	0.97	1.71	1.91	1.46	1.63	466	53.7	6.84
P10646	Tissue factor pathway inhibitor OS=Homo sapiens GN=TFPI PE=1 SV=1 - [TFPI1_HUMAN]	23.03	1	4	4	12	1.77	1.72	1.36	1.35	1.23	1.04	1.18	1.07	304	35.0	8.25
Q8WZ42	Titin OS=Homo sapiens GN=TTN PE=1 SV=4 - [TITIN_HUMAN]	0.54	2	10	12	26	0.83	0.76	0.79	0.74	0.84	1.04	0.96	1.71	34350	#####	6.35
Q96CP7	TLC domain-containing protein 1 OS=Homo sapiens GN=TLCD1 PE=2 SV=1 - [TLCD1_HUMAN]	4.45	1	1	1	1					1.16	1.15	1.13	1.11	247	28.5	9.47
A6NGC4	TLC domain-containing protein 2 OS=Homo sapiens GN=TLCD2 PE=2 SV=3 - [TLCD2_HUMAN]	7.58	1	1	1	4					1.05	0.95	1.05	0.95	264	28.7	10.10
Q9BX74	TM2 domain-containing protein 1 OS=Homo sapiens GN=TM2D1 PE=1 SV=1 - [TM2D1_HUMAN]	2.90	1	1	1	1					1.09	0.97	0.98	0.88	207	22.3	6.51
Q13077	TNF receptor-associated factor 1 OS=Homo sapiens GN=TRAF1 PE=1 SV=1 - [TRAF1_HUMAN]	5.77	1	2	2	3					0.72	0.77	0.50	0.54	416	46.1	6.11
Q13114	TNF receptor-associated factor 3 OS=Homo sapiens GN=TRAF3 PE=1 SV=2 - [TRAF3_HUMAN]	2.99	1	1	1	1	2.67	2.68	2.93	2.96					568	64.4	7.91
O00463	TNF receptor-associated factor 5 OS=Homo sapiens GN=TRAF5 PE=1 SV=2 - [TRAF5_HUMAN]	1.97	1	1	1	1	0.85	1.21	0.78	1.12					557	64.4	7.43
Q9H0E2	Toll-interacting protein OS=Homo sapiens GN=TOLLIP PE=1 SV=1 - [TOLIP_HUMAN]	7.66	1	2	2	2					1.05	1.13	1.21	1.95	274	30.3	5.97
Q15399	Toll-like receptor 1 OS=Homo sapiens GN=TLR1 PE=1 SV=3 - [TLR1_HUMAN]	2.16	2	2	2	2	0.24	0.22	0.27	0.25					786	90.2	7.08
O60603	Toll-like receptor 2 OS=Homo sapiens GN=TLR2 PE=1 SV=1 - [TLR2_HUMAN]	3.57	4	1	2	3	1.33	1.51	1.38	1.58	1.79	1.45	1.47	1.19	784	89.8	6.61
O15455	Toll-like receptor 3 OS=Homo sapiens GN=TLR3 PE=1 SV=1 - [TLR3_HUMAN]	3.32	4	2	3	7	0.69	0.19	0.74	0.20	2.53	2.41	1.01	0.96	904	103.8	7.18
O60602	Toll-like receptor 5 OS=Homo sapiens GN=TLR5 PE=1 SV=4 - [TLR5_HUMAN]	1.63	1	1	1	4	0.87	0.92	0.81	0.86					858	97.8	6.68
Q6ZVM7	TOM1-like protein 2 OS=Homo sapiens GN=TOM1L2 PE=1 SV=1 - [TM1L2_HUMAN]	1.38	1	1	1	1					1.38	1.31	1.44	1.36	507	55.5	4.79
Q8IYR6	Tomoregulin-1 OS=Homo sapiens GN=TMEFF1 PE=1 SV=1 - [TEFF1_HUMAN]	3.95	1	1	1	2					0.81	0.17	0.85	0.33	380	40.9	6.68
Q8NFQ8	Torsin-1A-interacting protein 2 OS=Homo sapiens GN=TOR1AIP2 PE=1 SV=1 - [TOIP2_HUMAN]	3.62	1	1	1	1					0.66	0.75	1.01	1.14	470	51.2	4.96
Q9Y3C4	TP53RK-binding protein OS=Homo sapiens GN=TPRKB PE=1 SV=1 - [TPRKB_HUMAN]	16.00	1	1	1	1			0.58	0.59					175	19.6	6.79
O15050	TPR and ankyrin repeat-containing protein 1 OS=Homo sapiens GN=TRANK1 PE=2 SV=4 - [TRNK1_HUMAN]	0.55	1	1	1	10	1.06	1.05	0.93	0.93					2925	336.0	6.76

Q969N4	Trace amine-associated receptor 8 OS=Homo sapiens GN=TAAR8 PE=2 SV=1 - [TAAR8_HUMAN]	2.63	1	1	1	1	0.93	1.05	0.89	1.02					342	38.0	6.81
Q92844	TRAF family member-associated NF-kappa-B activator OS=Homo sapiens GN=TANK PE=1 SV=2 - [TANK_HUMAN]	3.53	1	2	2	2					0.98	0.72	0.76	0.56	425	47.8	5.68
Q9BWF2	TRAF-interacting protein OS=Homo sapiens GN=TRAIP PE=1 SV=1 - [TRAIP_HUMAN]	5.54	1	1	2	2	1.33	1.29	1.36	1.34					469	53.3	8.48
Q9UKE5	TRAF2 and NCK-interacting protein kinase OS=Homo sapiens GN=TNIK PE=1 SV=1 - [TNIK_HUMAN]	4.34	1	2	2	3	0.72	0.65	1.10	1.01					1360	154.8	7.17
Q9Y228	TRAF3-interacting JNK-activating modulator OS=Homo sapiens GN=TRAF3IP3 PE=1 SV=2 - [T3JAM_HUMAN]	0.91	1	1	1	1	0.43	0.57	0.41	0.55					551	63.6	8.51
Q8TDR0	TRAF3-interacting protein 1 OS=Homo sapiens GN=TRAF3IP1 PE=1 SV=1 - [MIPT3_HUMAN]	1.30	1	1	1	1	0.33	0.32	0.30	0.29					691	78.6	7.93
O60296	Trafficking kinesin-binding protein 2 OS=Homo sapiens GN=TRAK2 PE=1 SV=2 - [TRAK2_HUMAN]	2.19	1	2	2	8	0.80	0.93	0.85	1.00	0.57	0.63	0.53	0.58	914	101.4	5.24
Q9Y5R8	Trafficking protein particle complex subunit 1 OS=Homo sapiens GN=TRAPPC1 PE=1 SV=1 - [TPPC1_HUMAN]	11.03	1	1	1	2	1.23	1.00	1.10	0.89					145	16.8	9.16
Q7Z392	Trafficking protein particle complex subunit 11 OS=Homo sapiens GN=TRAPPC11 PE=1 SV=2 - [TPC11_HUMAN]	2.29	1	2	2	5	0.80	0.87	0.71	0.78	1.09	0.96	1.59	1.40	1133	128.8	7.14
Q9UL33	Trafficking protein particle complex subunit 2-like protein OS=Homo sapiens GN=TRAPPC2L PE=1 SV=1 - [TPC2L_HUMAN]	12.14	1	1	1	1					0.66	1.05	0.94	1.50	140	16.1	6.77
Q9Y2L5	Trafficking protein particle complex subunit 8 OS=Homo sapiens GN=TRAPPC8 PE=1 SV=2 - [TPPC8_HUMAN]	0.98	1	1	1	1	0.95	0.43	0.75	0.34					1435	160.9	6.87
Q9NZ01	Trans-2,3-enoyl-CoA reductase OS=Homo sapiens GN=TECR PE=1 SV=1 - [TECR_HUMAN]	3.25	1	1	1	1	1.17	1.01	1.02	0.89					308	36.0	9.45
P23771	Trans-acting T-cell-specific transcription factor GATA-3 OS=Homo sapiens GN=GATA3 PE=1 SV=1 - [GATA3_HUMAN]	8.58	1	2	2	3					1.00	0.87	0.95	0.82	443	47.9	9.42
O43493	Trans-Golgi network integral membrane protein 2 OS=Homo sapiens GN=TGOLN2 PE=1 SV=2 - [TGON2_HUMAN]	5.83	1	2	2	7	1.20	1.00	1.17	1.02					480	51.1	5.73
P37837	Transaldolase OS=Homo sapiens GN=TALDO1 PE=1 SV=2 - [TALDO_HUMAN]	10.68	1	4	4	7	0.83	0.91	0.93	1.01					337	37.5	6.81
P20061	Transcobalamin-1 OS=Homo sapiens GN=TCN1 PE=1 SV=2 - [TCO1_HUMAN]	1.85	1	1	1	2	1.40	1.42	1.45	1.48					433	48.2	5.03
P51532	Transcription activator BRG1 OS=Homo sapiens GN=SMARCA4 PE=1 SV=2 - [SMCA4_HUMAN]	0.91	1	1	1	3	1.13	1.01	1.11	1.00	0.95	1.48	0.76	1.19	1647	184.5	7.88
Q99990	Transcription cofactor vestigial-like protein 1 OS=Homo sapiens GN=VGLL1 PE=1 SV=1 - [VGLL1_HUMAN]	6.59	1	1	1	1					0.87	0.92	0.89	0.94	258	28.7	9.17
A8MV65	Transcription cofactor vestigial-like protein 3 OS=Homo sapiens GN=VGLL3 PE=2 SV=1 - [VGLL3_HUMAN]	4.91	1	1	1	2					1.72	1.65	1.66	1.59	326	36.0	6.38
O75764	Transcription elongation factor A protein 3 OS=Homo sapiens GN=TCEA3 PE=2 SV=2 - [TCEA3_HUMAN]	5.46	1	1	1	1					1.29	1.19	1.77	1.64	348	38.9	9.19
Q7KZ85	Transcription elongation factor SPT6 OS=Homo sapiens GN=SUPT6H PE=1 SV=2 - [SPT6H_HUMAN]	4.98	1	5	5	14	0.75	0.75	0.81	0.83	0.99	0.83	0.97	0.84	1726	198.9	4.91
Q9Y242	Transcription factor 19 OS=Homo sapiens GN=TCF19 PE=1 SV=2 - [TCF19_HUMAN]	4.64	1	1	1	1					0.81	0.97	0.82	0.99	345	37.2	8.32
Q9UGU0	Transcription factor 20 OS=Homo sapiens GN=TCF20 PE=1 SV=3 - [TCF20_HUMAN]	0.36	1	1	1	1					1.93	1.49	2.14	1.65	1960	211.6	9.04
Q6VUC0	Transcription factor AP-2-epsilon OS=Homo sapiens GN=TFAP2E PE=2 SV=1 - [AP2E_HUMAN]	1.58	1	1	1	2					0.90	1.02	0.78	0.87	442	46.2	8.37
Q01664	Transcription factor AP-4 OS=Homo sapiens GN=TFAP4 PE=1 SV=2 - [TFAP4_HUMAN]	3.25	1	1	1	1	0.49	0.22	1.01	0.44					338	38.7	5.87
Q14209	Transcription factor E2F2 OS=Homo sapiens GN=E2F2 PE=1 SV=1 - [E2F2_HUMAN]	1.37	1	1	1	1					0.90	0.42	1.12	0.53	437	47.5	4.87
Q16254	Transcription factor E2F4 OS=Homo sapiens GN=E2F4 PE=1 SV=2 - [E2F4_HUMAN]	5.81	7	1	2	2	1.62	1.66	1.44	1.48					413	43.9	4.75

P19484	Transcription factor EB OS=Homo sapiens GN=TFEB PE=1 SV=3 - [TFEB_HUMAN]	4.83	1	1	1	1					1.39	1.76	1.02	1.30	476	52.8	6.29
Q9Y603	Transcription factor ETV7 OS=Homo sapiens GN=ETV7 PE=1 SV=1 - [ETV7_HUMAN]	6.45	1	1	1	1	1.34	1.10	1.28	1.06					341	39.0	8.03
Q14469	Transcription factor HES-1 OS=Homo sapiens GN=HES1 PE=1 SV=1 - [HES1_HUMAN]	5.71	1	1	1	3	1.41	1.22	1.34	1.17	1.50	1.91	1.21	1.55	280	29.5	9.61
P17535	Transcription factor jun-D OS=Homo sapiens GN=JUND PE=1 SV=3 - [JUND_HUMAN]	4.90	1	1	1	2	1.24	1.35	1.20	1.31					347	35.2	7.37
P52954	Transcription factor LBX1 OS=Homo sapiens GN=LBX1 PE=2 SV=2 - [LBX1_HUMAN]	11.39	1	1	1	1	0.27	0.25	0.14	0.13					281	30.2	6.93
Q33E94	Transcription factor RFX4 OS=Homo sapiens GN=RFX4 PE=1 SV=2 - [RFX4_HUMAN]	1.90	1	1	1	1	0.77	0.80	0.71	0.74					735	83.3	6.86
Q9UN79	Transcription factor SOX-13 OS=Homo sapiens GN=SOX13 PE=1 SV=3 - [SOX13_HUMAN]	1.29	1	1	1	1					1.05	1.07	0.89	0.90	622	69.2	6.71
Q06945	Transcription factor SOX-4 OS=Homo sapiens GN=SOX4 PE=1 SV=1 - [SOX4_HUMAN]	4.01	1	1	1	1					1.66	1.43	1.67	1.44	474	47.2	7.36
P08047	Transcription factor Sp1 OS=Homo sapiens GN=SP1 PE=1 SV=3 - [SP1_HUMAN]	3.57	1	1	1	1	1.14	1.21	0.80	0.86					785	80.6	7.34
Q02446	Transcription factor Sp4 OS=Homo sapiens GN=SP4 PE=1 SV=2 - [SP4_HUMAN]	3.57	1	1	1	1					2.05	2.00	1.63	1.59	784	81.9	7.05
Q3SY56	Transcription factor Sp6 OS=Homo sapiens GN=SP6 PE=1 SV=1 - [SP6_HUMAN]	6.12	1	1	1	1					1.85	2.02	1.97	2.13	376	39.8	7.25
A6H8Y1	Transcription factor TFIIIB component B'' homolog OS=Homo sapiens GN=BDP1 PE=1 SV=3 - [BDP1_HUMAN]	1.64	1	2	2	2		0.67		0.61					2624	293.7	5.11
O15391	Transcription factor YY2 OS=Homo sapiens GN=YY2 PE=2 SV=1 - [TYY2_HUMAN]	1.34	1	1	1	2					1.26	0.90	1.46	1.04	372	41.3	6.18
Q00403	Transcription initiation factor IIB OS=Homo sapiens GN=GTF2B PE=1 SV=1 - [TF2B_HUMAN]	4.75	1	1	1	1							3.27	1.65	316	34.8	8.35
P21675	Transcription initiation factor TFIID subunit 1 OS=Homo sapiens GN=TAF1 PE=1 SV=2 - [TAF1_HUMAN]	1.23	1	1	1	1	0.86	0.88	0.83	0.86					1872	212.5	5.07
Q8IZX4	Transcription initiation factor TFIID subunit 1-like OS=Homo sapiens GN=TAF1L PE=1 SV=1 - [TAF1L_HUMAN]	1.42	1	1	1	1	1.38	1.61	1.54	1.80					1826	207.2	5.40
Q6P1X5	Transcription initiation factor TFIID subunit 2 OS=Homo sapiens GN=TAF2 PE=1 SV=3 - [TAF2_HUMAN]	3.50	1	2	2	6	0.79	0.85	0.84	0.92					1199	136.9	8.19
Q92750	Transcription initiation factor TFIID subunit 4B OS=Homo sapiens GN=TAF4B PE=1 SV=2 - [TAF4B_HUMAN]	1.97	1	1	1	2	1.02	1.03	1.05	1.06					862	91.0	9.54
Q15545	Transcription initiation factor TFIID subunit 7 OS=Homo sapiens GN=TAF7 PE=1 SV=1 - [TAF7_HUMAN]	4.58	1	1	1	1	0.76	0.65	0.74	0.64					349	40.2	5.20
Q13263	Transcription intermediary factor 1-beta OS=Homo sapiens GN=TRIM28 PE=1 SV=5 - [TIF1B_HUMAN]	4.55	1	2	2	2	0.59	0.53	0.66	0.52					835	88.5	5.77
O14867	Transcription regulator protein BACH1 OS=Homo sapiens GN=BACH1 PE=1 SV=2 - [BACH1_HUMAN]	5.16	1	3	3	8	1.69	1.33	1.77	1.40	1.33	0.97	1.01	0.72	736	81.9	5.05
Q15361	Transcription termination factor 1 OS=Homo sapiens GN=TTF1 PE=1 SV=3 - [TTF1_HUMAN]	0.77	1	1	1	1					1.00	1.20	1.79	2.13	905	103.0	9.38
Q9UNY4	Transcription termination factor 2 OS=Homo sapiens GN=TTF2 PE=1 SV=2 - [TTF2_HUMAN]	0.69	1	1	1	1					1.00	0.67	0.98	0.66	1162	129.5	8.37
P10071	Transcriptional activator GLI3 OS=Homo sapiens GN=GLI3 PE=1 SV=6 - [GLI3_HUMAN]	1.33	1	1	1	1					1.37	1.13	1.35	1.11	1580	169.8	7.40
Q5T5J6	Transcriptional protein SWT1 OS=Homo sapiens GN=SWT1 PE=2 SV=1 - [SWT1_HUMAN]	4.00	1	2	2	6	1.34	1.49	1.27	1.46	0.81	0.71	0.97	0.85	900	103.2	8.81
P46100	Transcriptional regulator ATRX OS=Homo sapiens GN=ATRX PE=1 SV=5 - [ATRX_HUMAN]	2.41	2	3	3	3	0.90	0.79	0.93	0.82	3.14	2.88	3.65	3.34	2492	282.4	6.58
P11308	Transcriptional regulator ERG OS=Homo sapiens GN=ERG PE=1 SV=2 - [ERG_HUMAN]	2.67	1	1	1	1						1.25		1.04	486	54.6	7.47

Q86T24	Transcriptional regulator Kaiso OS=Homo sapiens GN=ZBTB33 PE=1 SV=2 - [KAISO_HUMAN]	3.13	1	1	1	4	0.88	0.79	0.91	0.83					672	74.4	5.12
Q86YP4	Transcriptional repressor p66-alpha OS=Homo sapiens GN=GATAD2A PE=1 SV=1 - [P66A_HUMAN]	2.37	1	1	1	1	0.76	0.78	0.85	0.88					633	68.0	9.94
Q8WXI9	Transcriptional repressor p66-beta OS=Homo sapiens GN=GATAD2B PE=1 SV=1 - [P66B_HUMAN]	3.04	1	1	1	1	0.79	0.80	0.65	0.67					593	65.2	9.70
P02786	Transferrin receptor protein 1 OS=Homo sapiens GN=TFRC PE=1 SV=2 - [TFR1_HUMAN]	17.24	1	11	12	40	0.95	0.95	0.94	0.94	0.82	0.83	0.82	0.83	760	84.8	6.61
Q9UP52	Transferrin receptor protein 2 OS=Homo sapiens GN=TFR2 PE=1 SV=1 - [TFR2_HUMAN]	2.87	1	1	2	5	1.12	0.94	1.19	0.99					801	88.7	6.11
Q9Y4A5	Transformation/transcription domain-associated protein OS=Homo sapiens GN=TRRAP PE=1 SV=3 - [TRRAP_HUMAN]	1.89	1	4	4	4					0.77	0.86	0.91	0.99	3859	437.3	8.19
O75410	Transforming acidic coiled-coil-containing protein 1 OS=Homo sapiens GN=TACC1 PE=1 SV=2 - [TACC1_HUMAN]	4.22	1	2	2	8					1.58	1.56	1.40	1.37	805	87.7	4.88
O95359	Transforming acidic coiled-coil-containing protein 2 OS=Homo sapiens GN=TACC2 PE=1 SV=3 - [TACC2_HUMAN]	3.83	7	6	7	17	1.12	0.88	1.06	0.84	1.11	1.00	1.14	1.07	2948	309.2	4.79
Q03167	Transforming growth factor beta receptor type 3 OS=Homo sapiens GN=TGFBR3 PE=1 SV=3 - [TGBR3_HUMAN]	5.17	1	2	2	3	0.94	0.94	1.07	1.12					851	93.4	5.71
P01137	Transforming growth factor beta-1 OS=Homo sapiens GN=TGFB1 PE=1 SV=2 - [TGFB1_HUMAN]	11.54	1	3	3	15	1.18	1.11	1.22	1.11	1.18	1.13	1.28	1.26	390	44.3	8.53
O43294	Transforming growth factor beta-1-induced transcript 1 protein OS=Homo sapiens GN=TGFB1I1 PE=1 SV=2 - [TGFI1_HUMAN]	3.04	1	1	1	3					1.31	1.29	1.20	1.18	461	49.8	7.03
Q8WUH2	Transforming growth factor-beta receptor-associated protein 1 OS=Homo sapiens GN=TGFBRAP1 PE=1 SV=1 - [TGFA1_HUMAN]	6.74	1	3	3	5	1.42	1.45	1.65	1.69	1.07	1.13	1.08	1.14	860	97.1	6.55
Q15582	Transforming growth factor-beta-induced protein ig-h3 OS=Homo sapiens GN=TGFBI PE=1 SV=1 - [BGH3_HUMAN]	41.58	1	22	22	142	1.23	1.22	1.22	1.26	1.14	1.25	1.05	1.14	683	74.6	7.71
P37802	Transgelin-2 OS=Homo sapiens GN=TAGLN2 PE=1 SV=3 - [TAGL2_HUMAN]	71.36	2	12	12	199	0.73	0.70	0.72	0.69	1.00	0.90	0.98	0.92	199	22.4	8.25
O75762	Transient receptor potential cation channel subfamily A member 1 OS=Homo sapiens GN=TRPA1 PE=2 SV=3 - [TRPA1_HUMAN]	1.61	4	1	2	8	0.62		0.45						1119	127.4	7.12
O94759	Transient receptor potential cation channel subfamily M member 2 OS=Homo sapiens GN=TRPM2 PE=1 SV=2 - [TRPM2_HUMAN]	2.06	1	2	2	2	0.09	0.62	0.05	0.74					1503	171.1	7.50
Q9HCF6	Transient receptor potential cation channel subfamily M member 3 OS=Homo sapiens GN=TRPM3 PE=2 SV=4 - [TRPM3_HUMAN]	1.27	1	2	2	2	1.10	1.18	1.11	1.20	1.28	1.33	1.03	1.07	1732	197.4	7.15
Q9NZQ8	Transient receptor potential cation channel subfamily M member 5 OS=Homo sapiens GN=TRPM5 PE=1 SV=1 - [TRPM5_HUMAN]	1.29	1	1	1	2	0.91	0.67	0.83	0.61					1165	131.4	6.77
Q9BX84	Transient receptor potential cation channel subfamily M member 6 OS=Homo sapiens GN=TRPM6 PE=1 SV=2 - [TRPM6_HUMAN]	2.37	1	4	4	4	0.72	0.98	0.80	1.12	0.95	0.78	0.73	0.60	2022	231.6	7.77
Q96QT4	Transient receptor potential cation channel subfamily M member 7 OS=Homo sapiens GN=TRPM7 PE=1 SV=1 - [TRPM7_HUMAN]	0.64	1	1	1	2	0.88	0.87	0.82	0.82					1865	212.6	7.88
Q9HBA0	Transient receptor potential cation channel subfamily V member 4 OS=Homo sapiens GN=TRPV4 PE=1 SV=2 - [TRPV4_HUMAN]	2.76	1	1	1	3	0.89	0.70	0.83	0.66	0.96	1.00	0.99	1.03	871	98.2	7.77
Q9H1D0	Transient receptor potential cation channel subfamily V member 6 OS=Homo sapiens GN=TRPV6 PE=1 SV=2 - [TRPV6_HUMAN]	1.38	1	1	1	1					0.89	0.99	0.74	0.82	725	83.2	7.62
P55072	Transitional endoplasmic reticulum ATPase OS=Homo sapiens GN=VCP PE=1 SV=4 - [TERA_HUMAN]	9.06	1	5	5	11	0.69	0.69	0.67	0.65	0.52	0.48	0.81	0.75	806	89.3	5.26
P29401	Transketolase OS=Homo sapiens GN=TKT PE=1 SV=3 - [TKT_HUMAN]	5.78	4	2	3	11	1.22	1.12	1.26	1.17	0.72	0.79	0.71	0.78	623	67.8	7.66
Q9NR50	Translation initiation factor eIF-2B subunit gamma OS=Homo sapiens GN=EIF2B3 PE=1 SV=1 - [EI2BG_HUMAN]	7.30	1	1	1	1	0.76	0.86	0.92	1.05					452	50.2	6.47
P46199	Translation initiation factor IF-2, mitochondrial OS=Homo sapiens GN=MTIF2 PE=1 SV=2 - [IF2M_HUMAN]	4.26	1	1	1	1					0.73	1.20	0.44	0.73	727	81.3	7.15
Q9H2K0	Translation initiation factor IF-3, mitochondrial OS=Homo sapiens GN=MTIF3 PE=1 SV=4 - [IF3M_HUMAN]	4.68	1	1	1	1					0.95	0.92	0.87	0.84	278	31.7	9.69

Q92616	Translational activator GCN1 OS=Homo sapiens GN=GCN1L1 PE=1 SV=6 - [GCN1L_HUMAN]	2.06	1	5	5	7	0.97	1.06	0.88	0.97	0.67	0.47	0.79	0.62	2671	292.6	7.47
Q9UGP8	Translocation protein SEC63 homolog OS=Homo sapiens GN=SEC63 PE=1 SV=2 - [SEC63_HUMAN]	3.03	1	2	2	3		1.34		0.75	0.98	1.16	1.22	1.44	760	87.9	5.31
P43307	Translocon-associated protein subunit alpha OS=Homo sapiens GN=SSR1 PE=1 SV=3 - [SSRA_HUMAN]	5.24	1	1	1	1					0.22	0.27	0.22	0.27	286	32.2	4.49
Q9H295	Transmembrane 7 superfamily member 4 OS=Homo sapiens GN=TM7SF4 PE=2 SV=1 - [TM7S4_HUMAN]	2.55	1	1	1	2	1.22	1.33	1.03	1.12					470	53.4	9.29
Q92544	Transmembrane 9 superfamily member 4 OS=Homo sapiens GN=TM9SF4 PE=1 SV=2 - [TM9S4_HUMAN]	3.74	1	1	1	1	0.86	0.95	1.01	1.13					642	74.5	6.54
Q5TGY1	Transmembrane and coiled-coil domain-containing protein 4 OS=Homo sapiens GN=TMCO4 PE=2 SV=1 - [TMCO4_HUMAN]	3.47	1	1	1	1					1.42	1.07	1.67	1.26	634	67.9	5.85
Q96DC7	Transmembrane and coiled-coil domain-containing protein 6 OS=Homo sapiens GN=TMCO6 PE=2 SV=2 - [TMCO6_HUMAN]	6.29	1	2	2	2	0.45	0.56	0.40	0.50	0.85	0.84	0.81	0.80	493	54.4	5.81
Q9C0B7	Transmembrane and coiled-coil domain-containing protein 7 OS=Homo sapiens GN=TMCO7 PE=2 SV=2 - [TMCO7_HUMAN]	1.37	1	2	2	2	0.94	0.56	0.90	0.53	0.14	1.73	0.37	4.70	1094	120.7	6.11
O75069	Transmembrane and coiled-coil domains protein 2 OS=Homo sapiens GN=TMCC2 PE=1 SV=3 - [TMCC2_HUMAN]	3.10	1	1	1	1	0.77	0.57	0.88	0.66					709	77.4	6.81
Q9ULS5	Transmembrane and coiled-coil domains protein 3 OS=Homo sapiens GN=TMCC3 PE=2 SV=3 - [TMCC3_HUMAN]	7.13	1	1	1	1					0.23	0.13	0.65	0.39	477	53.8	8.54
Q6UXZ0	Transmembrane and immunoglobulin domain-containing protein 1 OS=Homo sapiens GN=TMIGD1 PE=2 SV=1 - [TMIG1_HUMAN]	12.60	1	1	1	1	0.79	0.88	0.76	0.86					262	29.2	7.88
Q8IUR5	Transmembrane and TPR repeat-containing protein 1 OS=Homo sapiens GN=TMTC1 PE=2 SV=3 - [TMTC1_HUMAN]	1.13	1	1	1	1					0.67	1.20	1.09	1.94	882	98.8	8.84
Q8N394	Transmembrane and TPR repeat-containing protein 2 OS=Homo sapiens GN=TMTC2 PE=2 SV=1 - [TMTC2_HUMAN]	1.91	1	1	1	2	1.26	0.86	1.30	0.89					836	94.1	8.78
Q7Z5M5	Transmembrane channel-like protein 3 OS=Homo sapiens GN=TMC3 PE=1 SV=3 - [TMC3_HUMAN]	2.73	1	1	2	2					1.19	1.13	0.86	0.81	1100	125.6	9.19
Q13445	Transmembrane emp24 domain-containing protein 1 OS=Homo sapiens GN=TMED1 PE=1 SV=1 - [TMED1_HUMAN]	2.20	1	1	1	1					0.99	1.08	0.84	0.92	227	25.2	4.48
Q9BVK6	Transmembrane emp24 domain-containing protein 9 OS=Homo sapiens GN=TMED9 PE=1 SV=2 - [TMED9_HUMAN]	4.68	1	1	1	1					0.64	0.57	0.71	0.63	235	27.3	8.02
O14668	Transmembrane gamma-carboxyglutamic acid protein 1 OS=Homo sapiens GN=PRRG1 PE=2 SV=1 - [TMG1_HUMAN]	6.88	1	1	1	1					1.38	1.42	1.35	1.38	218	24.9	5.19
Q14956	Transmembrane glycoprotein NMB OS=Homo sapiens GN=GPNMB PE=1 SV=2 - [GPNMB_HUMAN]	1.75	1	1	1	1	1.73	1.73	1.00	1.01					572	63.9	6.64
Q6ZMR5	Transmembrane protease serine 11A OS=Homo sapiens GN=TMPRSS11A PE=1 SV=1 - [TM11A_HUMAN]	2.14	1	1	1	3					0.97	1.38	1.08	1.53	421	47.5	9.22
Q86WS5	Transmembrane protease serine 12 OS=Homo sapiens GN=TMPRSS12 PE=2 SV=2 - [TMPSC_HUMAN]	4.60	1	1	1	1	1.23	0.36	1.01	0.30					348	38.6	8.62
Q9H3S3	Transmembrane protease serine 5 OS=Homo sapiens GN=TMPRSS5 PE=1 SV=2 - [TMPS5_HUMAN]	2.19	1	1	1	1	0.90	0.93	0.97	1.00					457	49.5	6.71
Q7RTY8	Transmembrane protease serine 7 OS=Homo sapiens GN=TMPRSS7 PE=2 SV=3 - [TMPS7_HUMAN]	3.91	1	1	1	4	0.64	0.90	0.61	0.86					843	94.4	8.48
Q86TL2	Transmembrane protein 110 OS=Homo sapiens GN=TMEM110 PE=2 SV=1 - [TM110_HUMAN]	5.10	1	1	1	1	1.00	0.95	1.04	0.99					294	33.2	8.15
Q9BTD3	Transmembrane protein 121 OS=Homo sapiens GN=TMEM121 PE=2 SV=1 - [TM121_HUMAN]	7.21	1	1	1	1	1.09	0.87	1.37	1.09					319	35.8	9.26
Q9H061	Transmembrane protein 126A OS=Homo sapiens GN=TMEM126A PE=1 SV=1 - [T126A_HUMAN]	17.95	1	1	1	1	1.16	1.33	1.08	1.23					195	21.5	9.26
A0AVI4	Transmembrane protein 129 OS=Homo sapiens GN=TMEM129 PE=2 SV=1 - [TM129_HUMAN]	2.76	1	1	1	1					1.61	1.11	1.38	0.95	362	40.4	7.66
Q92545	Transmembrane protein 131 OS=Homo sapiens GN=TMEM131 PE=1 SV=3 - [TM131_HUMAN]	2.18	1	2	2	4	1.42	1.40	1.64	1.64					1883	205.0	8.59

A2VDJ0	Transmembrane protein 131-like OS=Homo sapiens GN=KIAA0922 PE=1 SV=2 - [T131L_HUMAN]	2.24	1	2	2	3					1.34	1.26	1.13	1.06	1609	179.2	6.86
Q6IEE7	Transmembrane protein 132E OS=Homo sapiens GN=TMEM132E PE=2 SV=1 - [T132E_HUMAN]	1.52	1	1	1	1					1.03	1.05	0.98	1.00	984	107.0	5.90
Q6P9G4	Transmembrane protein 154 OS=Homo sapiens GN=TMEM154 PE=2 SV=2 - [TM154_HUMAN]	6.01	1	1	1	1						1.50			183	20.5	4.53
Q8NDZ6	Transmembrane protein 161B OS=Homo sapiens GN=TMEM161B PE=2 SV=1 - [T161B_HUMAN]	2.26	1	1	1	2					0.63	0.10	1.19	0.20	487	55.4	8.37
Q8NBL3	Transmembrane protein 178 OS=Homo sapiens GN=TMEM178 PE=2 SV=1 - [TM178_HUMAN]	2.36	1	1	1	1					0.86	0.95	0.70	0.78	297	33.0	8.47
Q14CX5	Transmembrane protein 180 OS=Homo sapiens GN=TMEM180 PE=2 SV=1 - [TM180_HUMAN]	2.71	1	1	1	3					0.24	0.14	0.78	0.44	517	57.3	7.97
Q9Y519	Transmembrane protein 184B OS=Homo sapiens GN=TMEM184B PE=1 SV=2 - [T184B_HUMAN]	5.90	1	1	1	6	0.87	1.39	0.79	1.17	1.17	1.51	1.26	1.37	407	45.5	6.89
Q8IY95	Transmembrane protein 192 OS=Homo sapiens GN=TMEM192 PE=1 SV=1 - [TM192_HUMAN]	5.90	1	1	1	1					0.51	0.64	0.49	0.61	271	30.9	7.99
Q68D42	Transmembrane protein 215 OS=Homo sapiens GN=TMEM215 PE=2 SV=1 - [TM215_HUMAN]	2.98	1	1	1	1					0.92	0.53	1.14	0.66	235	25.8	5.34
Q24JQ0	Transmembrane protein 241 OS=Homo sapiens GN=TMEM241 PE=2 SV=1 - [TM241_HUMAN]	5.41	1	1	1	1	0.96	1.02	0.91	0.98					296	32.6	8.66
Q86YD3	Transmembrane protein 25 OS=Homo sapiens GN=TMEM25 PE=1 SV=1 - [TMM25_HUMAN]	1.91	1	1	1	1	1.22	1.06	1.21	1.07					366	39.3	7.50
P57088	Transmembrane protein 33 OS=Homo sapiens GN=TMEM33 PE=1 SV=2 - [TMM33_HUMAN]	2.43	1	1	1	1	0.73	0.66	0.77	0.71					247	28.0	9.70
Q8WWA1	Transmembrane protein 40 OS=Homo sapiens GN=TMEM40 PE=1 SV=2 - [TMM40_HUMAN]	9.44	1	1	1	1					0.94	0.50	1.42	0.75	233	25.5	5.59
Q96HV5	Transmembrane protein 41A OS=Homo sapiens GN=TMEM41A PE=2 SV=1 - [TM41A_HUMAN]	3.79	1	1	1	1					0.56	0.57	0.67	0.68	264	29.6	9.03
O94886	Transmembrane protein 63A OS=Homo sapiens GN=TMEM63A PE=1 SV=3 - [TM63A_HUMAN]	2.60	1	1	1	2					2.25	1.54	1.62	1.11	807	92.1	7.27
Q9BSE2	Transmembrane protein 79 OS=Homo sapiens GN=TMEM79 PE=1 SV=1 - [TMM79_HUMAN]	2.79	1	1	1	1	1.09	1.23	1.13	1.30					394	43.5	4.93
A6NKF7	Transmembrane protein 88B OS=Homo sapiens GN=TMEM88B PE=3 SV=1 - [TM88B_HUMAN]	12.27	1	1	1	1					1.13	1.16	1.20	1.23	163	17.1	8.24
Q9HCN3	Transmembrane protein 8A OS=Homo sapiens GN=TMEM8A PE=1 SV=3 - [TMM8A_HUMAN]	0.91	1	1	1	1					1.13	0.83	1.04	0.77	771	84.7	7.53
A6NI61	Transmembrane protein 8C OS=Homo sapiens GN=TMEM8C PE=3 SV=1 - [TMM8C_HUMAN]	4.98	1	1	1	1					0.74	0.13	0.81	0.14	221	24.7	8.72
Q6UXU6	Transmembrane protein 92 OS=Homo sapiens GN=TMEM92 PE=2 SV=1 - [TMM92_HUMAN]	4.40	1	1	1	1					1.09				159	17.2	5.54
Q9NQ34	Transmembrane protein 9B OS=Homo sapiens GN=TMEM9B PE=1 SV=1 - [TMM9B_HUMAN]	2.53	1	1	1	1	0.10	0.21	0.99	2.13					198	22.5	8.06
Q2M3C6	Transmembrane protein C15orf27 OS=Homo sapiens GN=C15orf27 PE=2 SV=2 - [CO027_HUMAN]	4.90	1	1	1	2					0.74	1.08	0.62	0.92	531	58.4	4.72
Q6UWD8	Transmembrane protein C16orf54 OS=Homo sapiens GN=C16orf54 PE=1 SV=1 - [CP054_HUMAN]	12.50	1	1	1	2	1.22	1.10	1.34	1.21					224	24.3	6.19
Q5T6L9	Transmembrane protein C6orf70 OS=Homo sapiens GN=C6orf70 PE=2 SV=1 - [CF070_HUMAN]	4.87	1	2	2	13	1.10	1.09	0.84	1.09	1.05	1.17	0.93	1.03	678	77.7	6.73
A8MUB7	Transmembrane protein ENSP00000382582 OS=Homo sapiens PE=4 SV=2 - [YE031_HUMAN]	1.00	1	1	1	1	1.32	1.19	1.13	1.02					1105	125.8	8.40
Q92973	Transportin-1 OS=Homo sapiens GN=TNPO1 PE=1 SV=2 - [TNPO1_HUMAN]	3.56	1	2	2	2	1.93	2.14	1.88	2.09					898	102.3	4.98
O14787	Transportin-2 OS=Homo sapiens GN=TNPO2 PE=1 SV=3 - [TNPO2_HUMAN]	2.01	1	1	1	1					1.21	0.98	1.32	1.07	897	101.3	5.01

Q8IZ13	Transposon-derived Buster3 transposase-like protein OS=Homo sapiens GN=C5orf54 PE=2 SV=1 - [CE054_HUMAN]	3.87	1	1	2	3	1.09	1.42	1.13	1.47					594	68.3	6.42
P02766	Transthyretin OS=Homo sapiens GN=TTR PE=1 SV=1 - [TTHY_HUMAN]	76.87	2	14	14	2419	0.87	0.84	0.87	0.85	1.04	1.11	1.05	1.14	147	15.9	5.76
Q13428	Treacle protein OS=Homo sapiens GN=TCOF1 PE=1 SV=3 - [TCOF_HUMAN]	0.81	1	2	3	4					0.69	0.89	0.78	1.02	1488	152.0	9.04
Q03403	Trefoil factor 2 OS=Homo sapiens GN=TFF2 PE=1 SV=2 - [TFF2_HUMAN]	10.08	1	1	1	1							0.98	1.11	129	14.3	5.81
Q07654	Trefoil factor 3 OS=Homo sapiens GN=TFF3 PE=1 SV=1 - [TFF3_HUMAN]	31.25	1	2	2	3					2.18	2.39	2.08	2.28	80	8.6	5.92
O43280	Trehalase OS=Homo sapiens GN=TREH PE=1 SV=2 - [TREA_HUMAN]	4.29	1	2	2	4	1.28	1.28	1.06	1.07	1.10	0.77	1.11	0.78	583	66.5	5.68
Q86YW5	Trem-like transcript 1 protein OS=Homo sapiens GN=TREML1 PE=1 SV=2 - [TRML1_HUMAN]	27.65	1	4	4	32	0.87	0.75	0.89	0.77	1.52	1.35	1.43	1.39	311	32.7	6.05
Q7Z2Z1	Treslin OS=Homo sapiens GN=TICRR PE=1 SV=2 - [TICRR_HUMAN]	3.66	1	4	4	5	1.10	1.23	0.99	1.13	5.76	6.31	5.81	6.38	1910	210.7	8.78
Q96RU8	Tribbles homolog 1 OS=Homo sapiens GN=TRIB1 PE=1 SV=2 - [TRIB1_HUMAN]	5.38	1	1	1	2	0.96	0.79	1.20	1.00					372	41.0	7.28
Q5QJ38	Trichohyalin-like protein 1 OS=Homo sapiens GN=TCHHL1 PE=2 SV=1 - [TCHL1_HUMAN]	1.22	1	1	1	1					0.99	1.31	0.97	1.29	904	99.2	4.69
P40939	Trifunctional enzyme subunit alpha, mitochondrial OS=Homo sapiens GN=HADHA PE=1 SV=2 - [ECHA_HUMAN]	2.62	1	1	1	1	0.85	0.76	0.69	0.62					763	82.9	9.04
P22102	Trifunctional purine biosynthetic protein adenosine-3 OS=Homo sapiens GN=GART PE=1 SV=1 - [PUR2_HUMAN]	7.23	1	3	3	3	0.62	1.00	0.53	0.84	1.04	1.09	0.89	0.94	1010	107.7	6.70
Q96RS0	Trimethylguanosine synthase OS=Homo sapiens GN=TGS1 PE=1 SV=3 - [TGS1_HUMAN]	3.40	1	1	1	2					1.01	0.45	0.99	0.44	853	96.6	4.94
O15417	Trinucleotide repeat-containing gene 18 protein OS=Homo sapiens GN=TNRC18 PE=1 SV=3 - [TNC18_HUMAN]	1.28	1	3	3	279	0.90	0.86	0.92	0.90	0.91	0.99	0.91	0.93	2968	314.3	8.70
Q8NDV7	Trinucleotide repeat-containing gene 6A protein OS=Homo sapiens GN=TNRC6A PE=1 SV=2 - [TNR6A_HUMAN]	4.28	1	3	3	8	0.80	0.73	0.99	0.93					1962	210.2	7.01
Q9UPQ9	Trinucleotide repeat-containing gene 6B protein OS=Homo sapiens GN=TNRC6B PE=1 SV=4 - [TNR6B_HUMAN]	2.13	1	2	2	5	1.36	1.40	1.38	1.42	1.80	3.19	2.39	4.24	1833	193.9	6.76
Q9HCJ0	Trinucleotide repeat-containing gene 6C protein OS=Homo sapiens GN=TNRC6C PE=1 SV=3 - [TNR6C_HUMAN]	6.45	1	4	4	5	0.84	0.58	0.84	0.58	0.65	0.70	0.73	0.79	1690	175.9	6.96
Q9H2D6	TRIO and F-actin-binding protein OS=Homo sapiens GN=TRIOBP PE=1 SV=3 - [TARA_HUMAN]	0.97	1	2	2	3					0.76	0.98	0.80	1.03	2365	261.2	8.48
P60174	Triosephosphate isomerase OS=Homo sapiens GN=TPI1 PE=1 SV=3 - [TPIS_HUMAN]	37.41	1	7	7	22	0.92	0.83	0.96	0.84	0.85	0.86	0.86	0.86	286	30.8	5.92
O75382	Tripartite motif-containing protein 3 OS=Homo sapiens GN=TRIM3 PE=1 SV=2 - [TRIM3_HUMAN]	2.55	1	1	1	1	1.28	1.27	1.14	1.13					744	80.8	7.83
Q9C037	Tripartite motif-containing protein 4 OS=Homo sapiens GN=TRIM4 PE=2 SV=2 - [TRIM4_HUMAN]	4.20	1	1	1	1	1.62	2.37	1.59	2.33					500	57.4	8.10
Q8IWZ5	Tripartite motif-containing protein 42 OS=Homo sapiens GN=TRIM42 PE=2 SV=2 - [TRI42_HUMAN]	4.43	1	2	3	5	0.92	0.72	0.96	0.76					723	82.7	7.93
Q96DX7	Tripartite motif-containing protein 44 OS=Homo sapiens GN=TRIM44 PE=1 SV=1 - [TRI44_HUMAN]	4.94	1	1	1	2					1.04	1.18	1.22	1.38	344	38.4	4.21
Q9C035	Tripartite motif-containing protein 5 OS=Homo sapiens GN=TRIM5 PE=1 SV=1 - [TRIM5_HUMAN]	4.26	1	1	1	1	0.62	0.58	0.61	0.57					493	56.3	6.05
O15016	Tripartite motif-containing protein 66 OS=Homo sapiens GN=TRIM66 PE=2 SV=4 - [TRI66_HUMAN]	0.99	1	1	1	1					0.80	0.55	1.17	0.82	1216	134.6	6.98
Q6ZTA4	Tripartite motif-containing protein 67 OS=Homo sapiens GN=TRIM67 PE=2 SV=3 - [TRI67_HUMAN]	2.55	1	1	1	1					0.67	0.36	0.75	0.40	783	83.8	7.33
Q9C029	Tripartite motif-containing protein 7 OS=Homo sapiens GN=TRIM7 PE=1 SV=2 - [TRIM7_HUMAN]	1.57	1	1	1	4	1.25	0.99	1.33	1.06	1.53	1.97	1.21	1.56	511	56.6	7.65

A6NK02	Tripartite motif-containing protein 75 OS=Homo sapiens GN=TRIM75 PE=2 SV=2 - [TRI75_HUMAN]	4.27	1	1	1	1					1.13	1.18	1.06	1.10	468	53.6	7.66
A6NDQ2	Tripartite motif-containing protein ENSP00000309378 OS=Homo sapiens PE=3 SV=2 - [TRIX3_HUMAN]	5.62	1	2	2	2	1.15	1.21	1.14	1.21	1.24	1.24	0.90	0.90	463	53.7	6.87
O14773	Tripeptidyl-peptidase 1 OS=Homo sapiens GN=TPP1 PE=1 SV=2 - [TPP1_HUMAN]	10.30	1	4	4	6	0.94	1.10	0.94	1.11	0.72	0.85	0.77	0.91	563	61.2	6.48
P29144	Tripeptidyl-peptidase 2 OS=Homo sapiens GN=TPP2 PE=1 SV=4 - [TPP2_HUMAN]	2.08	1	2	2	14	3.36	1.69	1.07	0.71	1.08	1.20	0.99	1.19	1249	138.3	6.32
O75962	Triple functional domain protein OS=Homo sapiens GN=TRIO PE=1 SV=2 - [TRIO_HUMAN]	2.29	1	2	2	2	1.47	1.68	1.64	1.88					3097	346.7	6.37
Q7Z2T5	TRMT1-like protein OS=Homo sapiens GN=TRMT1L PE=1 SV=2 - [TRM1L_HUMAN]	4.64	1	2	2	4	1.06	0.83	1.01	0.80	0.94	0.99	0.94	0.98	733	81.7	7.88
Q32P41	tRNA (guanine(37)-N1)-methyltransferase OS=Homo sapiens GN=TRMT5 PE=1 SV=2 - [TRM5_HUMAN]	4.91	1	2	2	2	1.07	1.49	0.95	1.32					509	58.2	8.62
Q9H3H1	tRNA dimethylallyltransferase, mitochondrial OS=Homo sapiens GN=TRIT1 PE=1 SV=1 - [MOD5_HUMAN]	3.00	1	1	1	1					0.02	0.05	0.50	1.17	467	52.7	8.21
O95620	tRNA-dihydrouridine(20a/20b) synthase [NAD(P)+]-like OS=Homo sapiens GN=DUS4L PE=2 SV=2 - [DUS4L_HUMAN]	2.52	1	1	1	7	1.16	1.25	1.23	1.33	0.98	1.23	1.16	1.46	317	35.8	7.33
Q9BUB4	tRNA-specific adenosine deaminase 1 OS=Homo sapiens GN=ADAT1 PE=2 SV=1 - [ADAT1_HUMAN]	2.39	1	1	1	1	0.19	0.20	0.28	0.30					502	55.4	9.01
Q96EY9	tRNA-specific adenosine deaminase-like protein 3 OS=Homo sapiens GN=ADAT3 PE=1 SV=1 - [ADAT3_HUMAN]	3.99	1	1	1	1					0.85	0.79	4.74	4.38	351	38.0	7.88
Q9Y3I0	tRNA-splicing ligase RtcB homolog OS=Homo sapiens GN=C22orf28 PE=1 SV=1 - [RTCB_HUMAN]	2.38	1	1	1	1					0.75	0.84	0.80	0.89	505	55.2	7.23
Q9NYL9	Tropomodulin-3 OS=Homo sapiens GN=TMOD3 PE=1 SV=1 - [TMOD3_HUMAN]	13.92	2	4	4	5					1.04	1.02	1.05	1.02	352	39.6	5.19
P09493	Tropomyosin alpha-1 chain OS=Homo sapiens GN=TPM1 PE=1 SV=2 - [TPM1_HUMAN]	26.41	2	3	9	101	0.27	0.61	0.43	0.97	1.51	1.33	1.40	1.23	284	32.7	4.74
P06753	Tropomyosin alpha-3 chain OS=Homo sapiens GN=TPM3 PE=1 SV=1 - [TPM3_HUMAN]	23.59	2	4	10	106	1.19	1.00	1.74	1.48	2.05	2.12	1.89	1.96	284	32.8	4.72
P67936	Tropomyosin alpha-4 chain OS=Homo sapiens GN=TPM4 PE=1 SV=3 - [TPM4_HUMAN]	41.53	2	10	15	170	0.58	0.53	0.57	0.52	1.11	1.09	1.14	1.05	248	28.5	4.69
P07477	Trypsin-1 OS=Homo sapiens GN=PRSS1 PE=1 SV=1 - [TRY1_HUMAN]	15.38	2	2	3	121	0.84	1.08	0.84	1.08	1.40	1.12	1.14	1.01	247	26.5	6.51
P07478	Trypsin-2 OS=Homo sapiens GN=PRSS2 PE=1 SV=1 - [TRY2_HUMAN]	15.38	1	1	3	49	0.74	0.92	0.71	0.87	0.78	0.93	0.88	1.04	247	26.5	4.92
P35030	Trypsin-3 OS=Homo sapiens GN=PRSS3 PE=1 SV=2 - [TRY3_HUMAN]	6.91	1	1	2	9	0.71	0.92	0.68	0.97	0.80	0.70	0.92	0.81	304	32.5	7.49
P17752	Tryptophan 5-hydroxylase 1 OS=Homo sapiens GN=TPH1 PE=1 SV=4 - [TPH1_HUMAN]	1.35	1	1	1	1					2.66	3.32	2.50	3.13	444	51.0	7.21
Q8IWU9	Tryptophan 5-hydroxylase 2 OS=Homo sapiens GN=TPH2 PE=1 SV=1 - [TPH2_HUMAN]	3.88	1	1	1	1	1.84	2.85	1.68	2.61					490	56.0	6.42
P23381	Tryptophan--tRNA ligase, cytoplasmic OS=Homo sapiens GN=WARS PE=1 SV=2 - [SYWC_HUMAN]	3.40	1	1	1	2	0.78	1.18	0.62	0.94					471	53.1	6.23
Q9UGM6	Tryptophan--tRNA ligase, mitochondrial OS=Homo sapiens GN=WARS2 PE=1 SV=1 - [SYWM_HUMAN]	1.39	1	1	1	1			0.31	0.30					360	40.1	9.28
Q8WUA8	Tsukushin OS=Homo sapiens GN=TSKU PE=2 SV=3 - [TSK_HUMAN]	19.26	1	5	5	12	1.54	1.37	1.43	1.17	1.12	1.14	1.20	1.21	353	37.8	6.87
P49815	Tuberin OS=Homo sapiens GN=TSC2 PE=1 SV=2 - [TSC2_HUMAN]	1.72	7	1	2	12	0.81		0.32		0.97	0.59	0.70	0.88	1807	200.5	7.31
A6NHL2	Tubulin alpha chain-like 3 OS=Homo sapiens GN=TUBAL3 PE=1 SV=2 - [TBAL3_HUMAN]	3.14	1	1	1	2					1.00	0.92	1.03	0.94	446	49.9	6.05
Q9BQE3	Tubulin alpha-1C chain OS=Homo sapiens GN=TUBA1C PE=1 SV=1 - [TBA1C_HUMAN]	19.38	5	3	6	29	0.72	0.73	0.67	0.70	1.26	1.28	1.17	1.17	449	49.9	5.10

P68366	Tubulin alpha-4A chain OS=Homo sapiens GN=TUBA4A PE=1 SV=1 - [TBA4A_HUMAN]	21.65	2	5	7	29	0.59	0.48	0.40	0.42	0.86	0.95	0.85	0.94	448	49.9	5.06
Q9NY65	Tubulin alpha-8 chain OS=Homo sapiens GN=TUBA8 PE=1 SV=1 - [TBA8_HUMAN]	13.59	2	1	4	12					1.44	1.15	1.18	0.94	449	50.1	5.06
Q9H4B7	Tubulin beta-1 chain OS=Homo sapiens GN=TUBB1 PE=1 SV=1 - [TBB1_HUMAN]	21.51	1	6	7	26	0.38	0.38	0.41	0.41	0.87	0.99	1.05	1.01	451	50.3	5.17
Q13885	Tubulin beta-2A chain OS=Homo sapiens GN=TUBB2A PE=1 SV=1 - [TBB2A_HUMAN]	26.97	4	2	8	23	0.50	0.55	0.47	0.52	1.15	1.04	1.43	1.29	445	49.9	4.89
Q13509	Tubulin beta-3 chain OS=Homo sapiens GN=TUBB3 PE=1 SV=2 - [TBB3_HUMAN]	17.33	1	1	6	17	0.75	0.73	0.77	0.76	0.31	0.49	0.83	1.28	450	50.4	4.93
Q3ZCM7	Tubulin beta-8 chain OS=Homo sapiens GN=TUBB8 PE=1 SV=2 - [TBB8_HUMAN]	13.51	4	2	5	10	0.62	0.65	0.54	0.58					444	49.7	4.89
P23258	Tubulin gamma-1 chain OS=Homo sapiens GN=TUBG1 PE=1 SV=2 - [TBG1_HUMAN]	3.99	1	1	1	1	0.85	0.87	0.81	0.83					451	51.1	6.14
A6NNM8	Tubulin polyglutamylase TTLL13 OS=Homo sapiens GN=TTLL13 PE=1 SV=2 - [TTL13_HUMAN]	3.44	1	1	1	1	1.20	1.02	1.20	1.02					815	93.6	8.91
Q14679	Tubulin polyglutamylase TTLL4 OS=Homo sapiens GN=TTLL4 PE=1 SV=2 - [TTLL4_HUMAN]	3.00	1	2	2	2	0.10	0.05	0.24	0.11	0.15	0.96	0.18	1.12	1199	133.3	8.85
Q6EMB2	Tubulin polyglutamylase TTLL5 OS=Homo sapiens GN=TTLL5 PE=1 SV=3 - [TTLL5_HUMAN]	3.67	1	3	3	4	0.93	1.18	0.92	1.16	0.81	0.89	0.82	0.90	1281	143.5	8.63
Q9BTW9	Tubulin-specific chaperone D OS=Homo sapiens GN=TBCD PE=1 SV=2 - [TBCD_HUMAN]	3.78	1	2	2	4					0.69	0.74	0.62	0.67	1192	132.5	6.19
Q9GZM7	Tubulointerstitial nephritis antigen-like OS=Homo sapiens GN=TINAGL1 PE=1 SV=1 - [TINAL_HUMAN]	5.78	1	1	1	1					0.59	0.89	1.87	2.82	467	52.4	6.99
Q9H7E2	Tudor domain-containing protein 3 OS=Homo sapiens GN=TDRD3 PE=1 SV=1 - [TDRD3_HUMAN]	5.22	1	2	2	2	0.75	0.73	0.86	0.85	1.09	1.10	0.90	0.91	651	73.1	9.23
O60522	Tudor domain-containing protein 6 OS=Homo sapiens GN=TDRD6 PE=2 SV=2 - [TDRD6_HUMAN]	1.38	1	1	1	1								0.86	2096	236.4	5.25
Q8NHU6	Tudor domain-containing protein 7 OS=Homo sapiens GN=TDRD7 PE=1 SV=2 - [TDRD7_HUMAN]	1.00	1	1	1	1					0.77	0.97	0.83	1.05	1098	123.5	7.21
Q9NNX1	Tuftelin OS=Homo sapiens GN=TUFT1 PE=2 SV=1 - [TUFT1_HUMAN]	5.13	1	1	2	2	1.00	1.01	1.00	1.02					390	44.2	6.00
Q9UBB9	Tuftelin-interacting protein 11 OS=Homo sapiens GN=TFIP11 PE=1 SV=1 - [TFP11_HUMAN]	3.35	1	2	2	3	0.77	0.74	0.71	0.68					837	96.8	5.67
Q03169	Tumor necrosis factor alpha-induced protein 2 OS=Homo sapiens GN=TNFAIP2 PE=1 SV=2 - [TNAP2_HUMAN]	5.50	1	2	2	3	0.91	0.96	0.93	0.98	0.69				654	72.6	6.46
O14788	Tumor necrosis factor ligand superfamily member 11 OS=Homo sapiens GN=TNFSF11 PE=1 SV=1 - [TNF11_HUMAN]	10.41	1	1	1	1	1.00	1.09	0.90	0.98					317	35.5	7.65
O43508	Tumor necrosis factor ligand superfamily member 12 OS=Homo sapiens GN=TNFSF12 PE=1 SV=1 - [TNF12_HUMAN]	3.21	1	1	1	1	1.31	1.41	1.35	1.47					249	27.2	9.41
O75888	Tumor necrosis factor ligand superfamily member 13 OS=Homo sapiens GN=TNFSF13 PE=1 SV=1 - [TNF13_HUMAN]	8.40	1	1	2	4					1.15	1.24	0.95	1.02	250	27.4	9.63
Q9Y275	Tumor necrosis factor ligand superfamily member 13B OS=Homo sapiens GN=TNFSF13B PE=1 SV=1 - [TN13B_HUMAN]	14.04	1	2	2	4					1.49	1.52	1.43	1.46	285	31.2	6.25
O43557	Tumor necrosis factor ligand superfamily member 14 OS=Homo sapiens GN=TNFSF14 PE=1 SV=2 - [TNF14_HUMAN]	5.42	1	1	1	1	1.02	0.90	0.74	0.65					240	26.3	8.92
O00220	Tumor necrosis factor receptor superfamily member 10A OS=Homo sapiens GN=TNFRSF10A PE=1 SV=3 - [TR10A_HUMAN]	3.42	1	1	1	1					1.97	2.21	1.83	2.06	468	50.1	7.03
O14798	Tumor necrosis factor receptor superfamily member 10C OS=Homo sapiens GN=TNFRSF10C PE=1 SV=3 - [TR10C_HUMAN]	3.47	1	1	1	2	1.13	1.10	1.08	1.07					259	27.4	4.82
Q9NP84	Tumor necrosis factor receptor superfamily member 12A OS=Homo sapiens GN=TNFRSF12A PE=1 SV=1 - [TNR12_HUMAN]	7.75	1	1	1	3					3.43	3.02	3.06	2.69	129	13.9	8.95
Q02223	Tumor necrosis factor receptor superfamily member 17 OS=Homo sapiens GN=TNFRSF17 PE=1 SV=2 - [TNR17_HUMAN]	6.52	1	1	1	2					0.79	0.74	0.93	0.88	184	20.2	5.35

Q969Z4	Tumor necrosis factor receptor superfamily member 19L OS=Homo sapiens GN=RELT PE=1 SV=1 - [TR19L_HUMAN]	1.86	1	1	1	1	1.49	1.59	1.35	1.45					430	46.1	8.35
Q15628	Tumor necrosis factor receptor type 1-associated DEATH domain protein OS=Homo sapiens GN=TRADD PE=1 SV=2 - [TRADD_HUMAN]	13.14	1	2	2	2					1.11	1.08	0.94	0.91	312	34.2	6.27
P98066	Tumor necrosis factor-inducible gene 6 protein OS=Homo sapiens GN=TNFAIP6 PE=1 SV=2 - [TSG6_HUMAN]	3.61	1	1	1	1	1.27	1.01	1.22	0.98					277	31.2	6.79
P55327	Tumor protein D52 OS=Homo sapiens GN=TPD52 PE=1 SV=2 - [TPD52_HUMAN]	6.70	1	1	1	1					2.16	1.55	2.07	1.49	224	24.3	4.83
O43399	Tumor protein D54 OS=Homo sapiens GN=TPD52L2 PE=1 SV=2 - [TPD54_HUMAN]	4.37	1	1	1	3					2.45	2.17	2.38	2.11	206	22.2	5.36
Q96J77	Tumor protein D55 OS=Homo sapiens GN=TPD52L3 PE=1 SV=2 - [TPD55_HUMAN]	10.71	1	1	1	1	1.21	1.06	1.38	1.21					140	15.5	9.95
Q6ZUI0	Tumor protein p63-regulated gene 1 protein OS=Homo sapiens GN=TPRG1 PE=2 SV=1 - [TPRG1_HUMAN]	5.82	1	1	1	1					1.28	1.86	1.26	1.84	275	31.2	7.09
O15350	Tumor protein p73 OS=Homo sapiens GN=TP73 PE=1 SV=1 - [P73_HUMAN]	2.99	2	1	2	2	1.16	1.24	1.06	1.14					636	69.6	6.95
Q6IBS0	Twinfilin-2 OS=Homo sapiens GN=TWF2 PE=1 SV=2 - [TWF2_HUMAN]	19.48	2	5	5	15					1.22	1.23	1.21	1.27	349	39.5	6.84
Q96RR1	Twinkle protein, mitochondrial OS=Homo sapiens GN=PEO1 PE=1 SV=1 - [PEO1_HUMAN]	6.14	1	2	2	4					0.51	0.46	0.82	0.74	684	77.1	8.98
Q9ULQ1	Two pore calcium channel protein 1 OS=Homo sapiens GN=TPCN1 PE=1 SV=3 - [TPC1_HUMAN]	0.86	1	2	2	4	1.14	1.25	1.08	1.18	1.02	0.92	0.90	0.92	816	94.1	8.27
Q9Y274	Type 2 lactosamine alpha-2,3-sialyltransferase OS=Homo sapiens GN=ST3GAL6 PE=1 SV=1 - [SIA10_HUMAN]	2.42	1	1	1	1	1.40	1.20	1.41	1.23					331	38.2	9.00
P32019	Type II inositol 1,4,5-trisphosphate 5-phosphatase OS=Homo sapiens GN=INPP5B PE=1 SV=4 - [I5P2_HUMAN]	1.61	1	1	1	3					1.26	1.20	1.34	1.27	993	112.8	5.57
P55073	Type III iodothyronine deiodinase OS=Homo sapiens GN=DIO3 PE=1 SV=3 - [IOD3_HUMAN]	10.79	1	1	1	1	1.16	0.76	1.21	0.80					278	31.4	6.71
P17735	Tyrosine aminotransferase OS=Homo sapiens GN=TAT PE=1 SV=1 - [ATTY_HUMAN]	3.52	1	1	1	1					1.20	1.29	1.41	1.51	454	50.4	6.30
P54577	Tyrosine--tRNA ligase, cytoplasmic OS=Homo sapiens GN=YARS PE=1 SV=4 - [SYYC_HUMAN]	5.68	1	2	2	70	1.05	0.98	0.97	0.95	1.55	1.90	1.49	1.93	528	59.1	7.05
Q9UIG0	Tyrosine-protein kinase BAZ1B OS=Homo sapiens GN=BAZ1B PE=1 SV=2 - [BAZ1B_HUMAN]	1.48	1	1	1	6	1.61	1.22	1.49	1.14					1483	170.8	8.48
P16591	Tyrosine-protein kinase Fer OS=Homo sapiens GN=FER PE=1 SV=2 - [FER_HUMAN]	3.04	2	2	3	8	2.32	1.24	2.27	1.23					822	94.6	7.14
P42685	Tyrosine-protein kinase FRK OS=Homo sapiens GN=FRK PE=1 SV=1 - [FRK_HUMAN]	9.11	1	2	2	8	1.52	1.46	1.63	1.58	1.55	1.38	1.56	1.39	505	58.2	6.67
O60674	Tyrosine-protein kinase JAK2 OS=Homo sapiens GN=JAK2 PE=1 SV=2 - [JAK2_HUMAN]	1.68	1	1	1	3					1.76	1.73	1.75	1.72	1132	130.6	7.21
Q06418	Tyrosine-protein kinase receptor TYRO3 OS=Homo sapiens GN=TYRO3 PE=1 SV=1 - [TYRO3_HUMAN]	2.81	1	2	2	3	0.66	0.59	0.70	0.64	0.53	0.64	0.52	0.63	890	96.8	5.67
P30530	Tyrosine-protein kinase receptor UFO OS=Homo sapiens GN=AXL PE=1 SV=3 - [UFO_HUMAN]	1.34	1	1	1	2	0.88	0.91	0.89	0.93					894	98.3	5.43
Q9H3Y6	Tyrosine-protein kinase Srms OS=Homo sapiens GN=SRMS PE=2 SV=1 - [SRMS_HUMAN]	2.66	1	1	1	1	1.28	1.50	1.52	1.79					488	54.5	8.21
P43405	Tyrosine-protein kinase SYK OS=Homo sapiens GN=SYK PE=1 SV=1 - [KSYK_HUMAN]	2.99	1	2	2	9	0.77	0.73	0.80	0.76	1.59	1.77	1.36	1.49	635	72.0	8.25
P42681	Tyrosine-protein kinase TXK OS=Homo sapiens GN=TXK PE=1 SV=3 - [TXK_HUMAN]	3.23	1	1	1	5	0.78	0.91	0.91	1.06	0.95	0.88	1.03	0.95	527	61.2	7.97
P18031	Tyrosine-protein phosphatase non-receptor type 1 OS=Homo sapiens GN=PTPN1 PE=1 SV=1 - [PTN1_HUMAN]	1.61	1	1	1	1					0.66	0.74	0.47	0.53	435	49.9	6.27
Q05209	Tyrosine-protein phosphatase non-receptor type 12 OS=Homo sapiens GN=PTPN12 PE=1 SV=3 - [PTN12_HUMAN]	8.85	1	4	4	6	1.49	1.61	1.45	1.58	1.14	1.23	1.16	1.27	780	88.1	5.62

Q12923	Tyrosine-protein phosphatase non-receptor type 13 OS=Homo sapiens GN=PTPN13 PE=1 SV=2 - [PTN13_HUMAN]	3.46	1	4	4	7					1.30	1.01	1.31	1.03	2485	276.7	6.42
Q15678	Tyrosine-protein phosphatase non-receptor type 14 OS=Homo sapiens GN=PTPN14 PE=1 SV=2 - [PTN14_HUMAN]	1.68	1	1	1	1	1.28	1.27	1.29	1.29					1187	135.2	8.31
Q4JDL3	Tyrosine-protein phosphatase non-receptor type 20 OS=Homo sapiens GN=PTPN20A PE=1 SV=1 - [PTN20_HUMAN]	6.43	1	1	1	1	0.23	0.16	0.19	0.13					420	48.4	5.77
Q9Y2R2	Tyrosine-protein phosphatase non-receptor type 22 OS=Homo sapiens GN=PTPN22 PE=1 SV=2 - [PTN22_HUMAN]	2.73	1	2	2	2	1.26	1.28	1.19	1.21					807	91.6	7.59
P26045	Tyrosine-protein phosphatase non-receptor type 3 OS=Homo sapiens GN=PTPN3 PE=1 SV=2 - [PTN3_HUMAN]	3.83	1	2	2	3	0.78	1.48	1.00	1.89	1.40	1.49	1.40	1.48	913	103.9	7.03
P29350	Tyrosine-protein phosphatase non-receptor type 6 OS=Homo sapiens GN=PTPN6 PE=1 SV=1 - [PTN6_HUMAN]	6.72	1	3	3	6	0.03	0.02	0.06	0.04	1.02	1.10	1.11	1.19	595	67.5	7.78
Q6IEG0	U11/U12 small nuclear ribonucleoprotein 48 kDa protein OS=Homo sapiens GN=SNRNP48 PE=1 SV=2 - [SNR48_HUMAN]	5.60	1	1	1	1	1.22	1.27	1.13	1.19					339	39.9	6.95
O15042	U2 snRNP-associated SURP motif-containing protein OS=Homo sapiens GN=U2SURP PE=1 SV=2 - [SR140_HUMAN]	1.17	1	1	1	1					1.65	0.71	1.88	0.81	1029	118.2	8.47
O43290	U4/U6.U5 tri-snRNP-associated protein 1 OS=Homo sapiens GN=SART1 PE=1 SV=1 - [SNUT1_HUMAN]	3.00	1	1	1	1					1.41	1.13	1.21	0.96	800	90.2	6.13
Q53GS9	U4/U6.U5 tri-snRNP-associated protein 2 OS=Homo sapiens GN=USP39 PE=1 SV=2 - [SNUT2_HUMAN]	5.66	1	2	2	2	0.78	0.82	0.85	0.90	0.77	0.73	0.93	0.88	565	65.3	8.91
O75643	U5 small nuclear ribonucleoprotein 200 kDa helicase OS=Homo sapiens GN=SNRNP200 PE=1 SV=2 - [U520_HUMAN]	0.47	1	1	1	1	0.71	0.78	0.84	0.94					2136	244.4	6.06
Q96DE0	U8 snoRNA-decapping enzyme OS=Homo sapiens GN=NUDT16 PE=1 SV=2 - [NUD16_HUMAN]	12.82	1	1	1	1					0.64	0.35	0.90	0.49	195	21.3	6.89
Q9UMX0	Ubiquilin-1 OS=Homo sapiens GN=UBQLN1 PE=1 SV=2 - [UBQL1_HUMAN]	14.43	3	4	4	5	0.95	1.01	1.02	1.10	0.90	1.04	0.91	1.05	589	62.5	5.11
Q9Y2Z9	Ubiquinone biosynthesis monooxygenase COQ6 OS=Homo sapiens GN=COQ6 PE=1 SV=2 - [COQ6_HUMAN]	5.34	1	1	1	1					0.40	0.36	0.68	0.60	468	50.8	7.30
P51784	Ubiquitin carboxyl-terminal hydrolase 11 OS=Homo sapiens GN=USP11 PE=1 SV=3 - [UBP11_HUMAN]	4.67	1	2	2	2	1.01	0.86	1.12	0.97	3.91	6.01	5.71	8.78	963	109.7	5.45
O94966	Ubiquitin carboxyl-terminal hydrolase 19 OS=Homo sapiens GN=USP19 PE=1 SV=2 - [UBP19_HUMAN]	1.97	1	1	1	1	0.85	0.99	0.80	0.94					1318	145.6	6.28
Q9Y2K6	Ubiquitin carboxyl-terminal hydrolase 20 OS=Homo sapiens GN=USP20 PE=1 SV=2 - [UBP20_HUMAN]	4.05	1	2	3	5	0.87	0.67	0.74	0.66					914	101.9	6.11
Q9UPU5	Ubiquitin carboxyl-terminal hydrolase 24 OS=Homo sapiens GN=USP24 PE=1 SV=3 - [UBP24_HUMAN]	0.69	1	1	1	1					0.84	0.88	0.71	0.74	2620	294.2	6.14
Q9BXU7	Ubiquitin carboxyl-terminal hydrolase 26 OS=Homo sapiens GN=USP26 PE=1 SV=1 - [UBP26_HUMAN]	3.29	1	2	2	3	0.71	0.31	1.06	0.47	0.95	1.09	0.87	0.99	913	104.0	8.70
A6NNY8	Ubiquitin carboxyl-terminal hydrolase 27 OS=Homo sapiens GN=USP27X PE=2 SV=3 - [UBP27_HUMAN]	2.28	1	1	1	2	1.09	1.09	1.04	1.04					438	49.6	7.18
Q96RU2	Ubiquitin carboxyl-terminal hydrolase 28 OS=Homo sapiens GN=USP28 PE=1 SV=1 - [UBP28_HUMAN]	6.87	1	3	3	3	1.19	1.21	1.14	1.18					1077	122.4	5.20
Q9HBJ7	Ubiquitin carboxyl-terminal hydrolase 29 OS=Homo sapiens GN=USP29 PE=2 SV=1 - [UBP29_HUMAN]	3.58	1	2	2	3	2.13	2.10	2.30	2.27					922	104.1	5.91
Q70CQ4	Ubiquitin carboxyl-terminal hydrolase 31 OS=Homo sapiens GN=USP31 PE=1 SV=2 - [UBP31_HUMAN]	2.07	1	1	1	1	1.22	0.77	1.23	0.78					1352	146.6	9.22
Q70CQ2	Ubiquitin carboxyl-terminal hydrolase 34 OS=Homo sapiens GN=USP34 PE=1 SV=2 - [UBP34_HUMAN]	0.62	1	1	1	1	1.79		1.56						3546	404.0	5.82
Q9P275	Ubiquitin carboxyl-terminal hydrolase 36 OS=Homo sapiens GN=USP36 PE=1 SV=3 - [UBP36_HUMAN]	5.00	1	2	2	9	1.18	1.17	1.23	1.23					1121	122.6	9.67
Q70EL4	Ubiquitin carboxyl-terminal hydrolase 43 OS=Homo sapiens GN=USP43 PE=1 SV=2 - [UBP43_HUMAN]	4.36	1	2	2	2					1.35	1.31	1.29	1.26	1123	122.7	9.19
Q9H0E7	Ubiquitin carboxyl-terminal hydrolase 44 OS=Homo sapiens GN=USP44 PE=1 SV=2 - [UBP44_HUMAN]	1.97	1	1	1	1	1.20	0.74	1.18	0.73					712	81.1	7.90

Q96K76	Ubiquitin carboxyl-terminal hydrolase 47 OS=Homo sapiens GN=USP47 PE=1 SV=3 - [UBP47_HUMAN]	2.04	1	1	1	1					1.22		2.15		1375	157.2	5.08
Q93009	Ubiquitin carboxyl-terminal hydrolase 7 OS=Homo sapiens GN=USP7 PE=1 SV=2 - [UBP7_HUMAN]	2.90	1	1	1	1	1.16	0.88	1.33	1.01					1102	128.2	5.55
P15374	Ubiquitin carboxyl-terminal hydrolase isozyme L3 OS=Homo sapiens GN=UCHL3 PE=1 SV=1 - [UCHL3_HUMAN]	6.52	1	2	2	4	1.24	0.78	1.08	0.65	0.95	0.79	0.96	0.80	230	26.2	4.92
Q9Y5K5	Ubiquitin carboxyl-terminal hydrolase isozyme L5 OS=Homo sapiens GN=UCHL5 PE=1 SV=3 - [UCHL5_HUMAN]	6.99	1	1	1	1					1.00	0.71	0.95	0.67	329	37.6	5.33
Q14139	Ubiquitin conjugation factor E4 A OS=Homo sapiens GN=UBE4A PE=1 SV=2 - [UBE4A_HUMAN]	1.78	1	1	1	4					0.10	0.05	1.01	0.48	1066	122.5	5.24
Q92890	Ubiquitin fusion degradation protein 1 homolog OS=Homo sapiens GN=UFD1L PE=1 SV=3 - [UFD1_HUMAN]	4.23	1	1	1	3					1.19	1.13	1.14	1.09	307	34.5	6.70
P57075	Ubiquitin-associated and SH3 domain-containing protein A OS=Homo sapiens GN=UBASH3A PE=1 SV=1 - [UBS3A_HUMAN]	2.12	1	1	1	1	1.14	1.40	1.17	1.46					661	74.1	7.66
Q9BSL1	Ubiquitin-associated domain-containing protein 1 OS=Homo sapiens GN=UBAC1 PE=1 SV=1 - [UBAC1_HUMAN]	1.98	1	1	1	3					1.65	1.21	1.54	1.39	405	45.3	4.92
Q14157	Ubiquitin-associated protein 2-like OS=Homo sapiens GN=UBAP2L PE=1 SV=2 - [UBP2L_HUMAN]	2.30	1	1	1	1					0.83	0.86	0.80	0.83	1087	114.5	7.11
P68036	Ubiquitin-conjugating enzyme E2 L3 OS=Homo sapiens GN=UBE2L3 PE=1 SV=1 - [UB2L3_HUMAN]	31.82	1	3	3	13	0.87	0.86	0.87	0.86	1.07	0.99	0.95	0.84	154	17.9	8.51
P61088	Ubiquitin-conjugating enzyme E2 N OS=Homo sapiens GN=UBE2N PE=1 SV=1 - [UBE2N_HUMAN]	17.76	2	3	3	8	0.89	1.01	0.75	0.87	1.16	1.05	1.09	0.99	152	17.1	6.57
Q9C0C9	Ubiquitin-conjugating enzyme E2 O OS=Homo sapiens GN=UBE2O PE=1 SV=3 - [UBE2O_HUMAN]	2.01	1	2	2	7	0.68		0.90		2.61	2.41	2.37	2.18	1292	141.2	5.12
Q13404	Ubiquitin-conjugating enzyme E2 variant 1 OS=Homo sapiens GN=UBE2V1 PE=1 SV=2 - [UB2V1_HUMAN]	30.61	2	4	4	23	0.84	0.83	0.88	0.87	1.15	1.10	1.13	1.06	147	16.5	7.93
Q96B02	Ubiquitin-conjugating enzyme E2 W OS=Homo sapiens GN=UBE2W PE=1 SV=1 - [UBE2W_HUMAN]	16.56	1	1	1	2	1.07	0.73	1.13	0.78					151	17.3	7.75
A1L167	Ubiquitin-conjugating enzyme E2Q-like protein 1 OS=Homo sapiens GN=UBE2QL1 PE=2 SV=2 - [U2QL1_HUMAN]	8.07	1	1	1	1	0.90	0.90	0.93	0.93					161	18.3	7.96
P61960	Ubiquitin-fold modifier 1 OS=Homo sapiens GN=UFM1 PE=1 SV=1 - [UFM1_HUMAN]	32.94	1	1	1	1					0.83	1.00	0.96	1.16	85	9.1	9.31
Q9Y3C8	Ubiquitin-fold modifier-conjugating enzyme 1 OS=Homo sapiens GN=UFC1 PE=1 SV=3 - [UFC1_HUMAN]	7.78	1	1	1	1					0.81	0.65	0.69	0.56	167	19.4	7.40
P22314	Ubiquitin-like modifier-activating enzyme 1 OS=Homo sapiens GN=UBA1 PE=1 SV=3 - [UBA1_HUMAN]	6.05	1	5	5	8	0.69	0.65	0.71	0.61					1058	117.8	5.76
P41226	Ubiquitin-like modifier-activating enzyme 7 OS=Homo sapiens GN=UBA7 PE=1 SV=2 - [UBA7_HUMAN]	1.58	1	1	1	3					1.21	1.29	1.20	1.28	1012	111.6	6.04
Q9BZL1	Ubiquitin-like protein 5 OS=Homo sapiens GN=UBL5 PE=1 SV=1 - [UBL5_HUMAN]	12.33	1	1	1	2					1.13	1.10	1.12	1.08	73	8.5	8.44
Q05086	Ubiquitin-protein ligase E3A OS=Homo sapiens GN=UBE3A PE=1 SV=4 - [UBE3A_HUMAN]	2.86	1	2	2	2	0.71	0.44	0.85	0.52	0.11	0.12	0.12	0.13	875	100.6	5.22
Q7Z3V4	Ubiquitin-protein ligase E3B OS=Homo sapiens GN=UBE3B PE=1 SV=3 - [UBE3B_HUMAN]	4.03	3	2	3	3	0.76	0.68	0.31	0.28	0.63	0.71	0.58	0.65	1068	123.0	8.19
Q92575	UBX domain-containing protein 4 OS=Homo sapiens GN=UBXN4 PE=1 SV=2 - [UBXN4_HUMAN]	5.31	1	1	1	1	0.38		0.40						508	56.7	6.38
Q9NY97	UDP-GlcNAc:betaGal beta-1,3-N-acetylglucosaminyltransferase 2 OS=Homo sapiens GN=B3GNT2 PE=1 SV=2 - [B3GN2_HUMAN]	5.79	1	2	2	4	1.14	1.04	1.19	1.10	0.86	0.78	0.91	0.83	397	46.0	8.54
Q9C0J1	UDP-GlcNAc:betaGal beta-1,3-N-acetylglucosaminyltransferase 4 OS=Homo sapiens GN=B3GNT4 PE=2 SV=1 - [B3GN4_HUMAN]	5.82	1	1	1	1	1.06	1.01	1.10	1.05					378	42.3	9.29
Q7Z7M8	UDP-GlcNAc:betaGal beta-1,3-N-acetylglucosaminyltransferase 8 OS=Homo sapiens GN=B3GNT8 PE=1 SV=1 - [B3GN8_HUMAN]	5.04	1	1	1	2	0.97	0.93	0.80	0.78					397	43.4	8.32
Q14376	UDP-glucose 4-epimerase OS=Homo sapiens GN=GALE PE=1 SV=2 - [GALE_HUMAN]	2.87	1	1	1	1					0.76	0.87	0.78	0.89	348	38.3	6.73

Q9NYU2	UDP-glucose:glycoprotein glucosyltransferase 1 OS=Homo sapiens GN=UGGT1 PE=1 SV=3 - [UGGG1_HUMAN]	2.44	1	2	2	2	0.75	0.81	0.85	0.91					1555	177.1	5.63
Q9NYU1	UDP-glucose:glycoprotein glucosyltransferase 2 OS=Homo sapiens GN=UGGT2 PE=1 SV=4 - [UGGG2_HUMAN]	1.12	1	1	2	2							0.88	0.87	1516	174.6	6.89
P35504	UDP-glucuronosyltransferase 1-5 OS=Homo sapiens GN=UGT1A5 PE=2 SV=1 - [UD15_HUMAN]	5.06	2	1	2	4	1.15	1.38	1.06	1.31					534	60.0	8.06
O60656	UDP-glucuronosyltransferase 1-9 OS=Homo sapiens GN=UGT1A9 PE=1 SV=1 - [UD19_HUMAN]	2.26	3	1	1	9	0.97	1.04	0.95	1.02	1.22	0.90	1.27	0.94	530	59.9	7.90
O75310	UDP-glucuronosyltransferase 2B11 OS=Homo sapiens GN=UGT2B11 PE=2 SV=1 - [UDB11_HUMAN]	4.73	1	2	2	3	0.76	0.89	0.86	1.01	0.14	0.15	0.14	0.15	529	61.0	8.92
Q9H3H5	UDP-N-acetylglucosamine--dolichyl-phosphate N-acetylglucosaminephosphotransferase OS=Homo sapiens GN=DPAGT1 PE=1 SV=2 - [GPT_HUMAN]	1.23	1	1	1	1					0.63	0.80	0.76	0.98	408	46.1	8.00
Q6BDS2	UHRF1-binding protein 1 OS=Homo sapiens GN=UHRF1BP1 PE=1 SV=1 - [URFB1_HUMAN]	0.83	1	1	1	11					0.78	0.90	0.80	0.96	1440	159.4	6.14
Q5EBM0	UMP-CMP kinase 2, mitochondrial OS=Homo sapiens GN=CMPK2 PE=1 SV=3 - [CMPK2_HUMAN]	2.23	1	1	1	2	0.72	0.68	0.66	0.61					449	49.4	7.01
P30085	UMP-CMP kinase OS=Homo sapiens GN=CMPK1 PE=1 SV=3 - [KCY_HUMAN]	13.78	1	2	2	13					1.12	1.23	1.02	1.22	196	22.2	5.57
Q96GV9	UNC119-binding protein C5orf30 OS=Homo sapiens GN=C5orf30 PE=1 SV=1 - [CE030_HUMAN]	4.85	1	1	1	1					0.71	0.83	0.61	0.71	206	23.1	9.45
Q69YW2	Uncharacterized membrane protein C1orf95 OS=Homo sapiens GN=C1orf95 PE=2 SV=1 - [CA095_HUMAN]	5.67	1	1	1	1	0.88	0.86	0.77	0.75					141	15.0	7.21
Q6PEX7	Uncharacterized protein ATPAF1-AS1 OS=Homo sapiens GN=ATPAF1-AS1 PE=2 SV=3 - [ATAS1_HUMAN]	9.71	1	1	1	1	1.10	1.04	1.10	1.05					206	23.3	6.27
Q8TEF2	Uncharacterized protein C10orf105 OS=Homo sapiens GN=C10orf105 PE=2 SV=3 - [CJ105_HUMAN]	7.52	1	1	1	1	1.14	1.16	0.82	0.85					133	14.5	8.41
Q8N655	Uncharacterized protein C10orf12 OS=Homo sapiens GN=C10orf12 PE=1 SV=1 - [CJ012_HUMAN]	4.89	1	3	3	5	1.08	1.09	1.09	1.11	1.09	1.34	1.17	1.44	1247	137.1	8.24
A6NCD4	Uncharacterized protein C10orf131 OS=Homo sapiens GN=C10orf131 PE=4 SV=3 - [CJ131_HUMAN]	3.13	1	1	1	2					0.57	0.51	1.45	1.31	256	29.6	6.19
Q5SWW7	Uncharacterized protein C10orf55 OS=Homo sapiens GN=C10orf55 PE=2 SV=1 - [CJ055_HUMAN]	3.31	1	1	1	1					1.10	1.06	1.05	1.01	151	16.0	7.43
Q711Q0	Uncharacterized protein C10orf71 OS=Homo sapiens GN=C10orf71 PE=2 SV=2 - [CJ071_HUMAN]	1.81	1	1	1	1					0.87	1.35	1.31	2.03	1435	156.4	6.38
Q96A22	Uncharacterized protein C11orf52 OS=Homo sapiens GN=C11orf52 PE=1 SV=2 - [CK052_HUMAN]	4.07	1	1	1	1	0.90	0.69	1.20	0.92					123	13.9	9.41
Q53FT3	Uncharacterized protein C11orf73 OS=Homo sapiens GN=C11orf73 PE=1 SV=2 - [CK073_HUMAN]	5.08	1	1	1	1					0.89	0.81	0.95	0.86	197	21.6	5.45
Q3KP22	Uncharacterized protein C11orf85 OS=Homo sapiens GN=C11orf85 PE=2 SV=1 - [CK085_HUMAN]	4.63	1	1	1	1					0.61	1.14	0.53	0.99	216	24.8	9.33
Q8TC90	Uncharacterized protein C12orf12 OS=Homo sapiens GN=C12orf12 PE=2 SV=1 - [CL012_HUMAN]	3.45	1	1	1	20					0.79	0.81	0.76	0.74	406	46.5	4.64
Q9HCM1	Uncharacterized protein C12orf35 OS=Homo sapiens GN=C12orf35 PE=1 SV=3 - [CL035_HUMAN]	2.35	1	2	2	2					2.99	2.72	2.01	1.74	1747	194.7	8.78
Q96N23	Uncharacterized protein C12orf55 OS=Homo sapiens GN=C12orf55 PE=2 SV=2 - [CL055_HUMAN]	5.42	6	3	4	102	0.79	0.75	0.87	0.83					812	92.3	7.71
Q5U649	Uncharacterized protein C12orf60 OS=Homo sapiens GN=C12orf60 PE=2 SV=2 - [CL060_HUMAN]	4.49	1	1	1	2	1.15	1.02	1.30	1.15					245	27.6	7.96
A6NFE2	Uncharacterized protein C12orf70 OS=Homo sapiens GN=C12orf70 PE=2 SV=2 - [CL070_HUMAN]	6.71	1	2	2	3	0.71	0.57	1.32	1.06	1.32	1.03	1.18	0.92	343	39.5	4.86
Q9NWQ4	Uncharacterized protein C14orf118 OS=Homo sapiens GN=C14orf118 PE=1 SV=3 - [CN118_HUMAN]	3.53	1	1	1	1					0.73	0.88	0.65	0.78	482	54.2	8.44
Q9H367	Uncharacterized protein C14orf57 OS=Homo sapiens GN=C14orf57 PE=2 SV=1 - [CN057_HUMAN]	11.81	1	1	1	1					0.99	0.36	0.79	0.29	127	14.7	9.25

Q9Y2V0	Uncharacterized protein C15orf41 OS=Homo sapiens GN=C15orf41 PE=2 SV=2 - [CO041_HUMAN]	13.52	1	2	3	6	1.14	1.03	1.19	1.14					281	32.2	6.62
Q9H0I2	Uncharacterized protein C16orf48 OS=Homo sapiens GN=C16orf48 PE=2 SV=1 - [CP048_HUMAN]	3.47	1	1	1	1					1.09	1.19	1.10	1.19	346	38.7	9.80
Q8WTQ4	Uncharacterized protein C16orf78 OS=Homo sapiens GN=C16orf78 PE=2 SV=1 - [CP078_HUMAN]	10.57	1	2	2	2	1.38	1.41	1.34	1.38					265	30.8	9.79
Q6ZW13	Uncharacterized protein C16orf86 OS=Homo sapiens GN=C16orf86 PE=2 SV=2 - [CP086_HUMAN]	3.79	1	1	1	3	1.02	1.07	1.03	1.08					317	33.5	5.38
Q8NEP4	Uncharacterized protein C17orf47 OS=Homo sapiens GN=C17orf47 PE=1 SV=3 - [CQ047_HUMAN]	5.44	1	2	2	6	0.86	0.88	0.84	0.91	1.27	1.31	1.37	1.41	570	63.1	9.20
A2RTY3	Uncharacterized protein C17orf66 OS=Homo sapiens GN=C17orf66 PE=2 SV=2 - [CQ066_HUMAN]	2.81	1	1	1	1					1.51	1.79	1.47	1.74	570	65.6	9.20
P0C7W0	Uncharacterized protein C17orf72 OS=Homo sapiens GN=C17orf72 PE=2 SV=1 - [CQ072_HUMAN]	11.11	1	1	1	3	0.79	0.53	0.80	0.55					189	20.6	4.79
Q9BSJ5	Uncharacterized protein C17orf80 OS=Homo sapiens GN=C17orf80 PE=1 SV=2 - [CQ080_HUMAN]	2.79	1	1	1	1	1.25	1.20	1.24	1.20					609	67.3	9.16
Q5BJE1	Uncharacterized protein C18orf34 OS=Homo sapiens GN=C18orf34 PE=1 SV=3 - [CR034_HUMAN]	2.42	1	2	2	2	0.95	0.96	1.01	1.02					867	101.9	6.77
A6NGS2	Uncharacterized protein C19orf69 OS=Homo sapiens GN=C19orf69 PE=2 SV=2 - [CS069_HUMAN]	26.92	1	1	1	2					1.23	1.64	1.03	1.37	130	14.5	4.28
Q3KP66	Uncharacterized protein C1orf106 OS=Homo sapiens GN=C1orf106 PE=2 SV=2 - [CA106_HUMAN]	4.68	1	2	2	3					0.85	0.80	1.31	1.54	663	72.9	9.31
Q5SNV9	Uncharacterized protein C1orf167 OS=Homo sapiens GN=C1orf167 PE=2 SV=2 - [CA167_HUMAN]	1.29	1	1	1	1					0.48	0.12	0.52	0.13	1468	162.3	10.70
Q5VWT5	Uncharacterized protein C1orf168 OS=Homo sapiens GN=C1orf168 PE=2 SV=1 - [CA168_HUMAN]	3.71	1	2	2	3	1.41	1.24	1.44	1.28	1.10	0.84	1.32	1.00	728	82.0	8.38
Q8NAX2	Uncharacterized protein C1orf172 OS=Homo sapiens GN=C1orf172 PE=2 SV=2 - [CA172_HUMAN]	11.06	1	1	1	1					1.63	1.96	1.72	2.07	398	43.6	6.54
Q9H425	Uncharacterized protein C1orf198 OS=Homo sapiens GN=C1orf198 PE=1 SV=1 - [CA198_HUMAN]	3.67	1	1	1	2					0.93	0.85	1.05	0.97	327	36.3	5.72
Q9H246	Uncharacterized protein C1orf21 OS=Homo sapiens GN=C1orf21 PE=1 SV=1 - [CA021_HUMAN]	7.44	1	1	1	1					0.85	0.71	0.84	0.70	121	13.9	5.22
Q9BUN1	Uncharacterized protein C1orf56 OS=Homo sapiens GN=C1orf56 PE=1 SV=1 - [CA056_HUMAN]	14.08	1	4	4	27	0.94	0.88	0.89	0.81	0.61	0.59	0.76	0.78	341	36.7	8.59
Q9H579	Uncharacterized protein C20orf132 OS=Homo sapiens GN=C20orf132 PE=2 SV=2 - [CT132_HUMAN]	5.59	1	2	2	3	0.84	0.75	0.74	0.66	0.51	0.98	0.66	1.08	483	54.8	6.40
Q9BQM9	Uncharacterized protein C20orf144 OS=Homo sapiens GN=C20orf144 PE=2 SV=1 - [CT144_HUMAN]	5.23	1	1	1	1					0.75	0.50	0.96	0.65	153	17.2	11.47
Q96M20	Uncharacterized protein C20orf152 OS=Homo sapiens GN=C20orf152 PE=2 SV=2 - [CT152_HUMAN]	6.60	1	3	3	3	0.66	0.60	0.89	0.81	0.77	0.88	0.97	1.16	576	67.5	9.26
Q9H1L0	Uncharacterized protein C20orf166 OS=Homo sapiens GN=C20orf166 PE=2 SV=1 - [CT166_HUMAN]	25.64	1	1	1	1	1.06	1.01	1.17	1.12					117	12.4	6.87
A1L168	Uncharacterized protein C20orf202 OS=Homo sapiens GN=C20orf202 PE=2 SV=2 - [CT202_HUMAN]	4.10	1	1	1	2					1.11	1.07	0.75	0.72	122	13.6	8.97
Q8N2C9	Uncharacterized protein C21orf128 OS=Homo sapiens GN=C21orf128 PE=2 SV=3 - [CU128_HUMAN]	8.64	1	1	1	1					0.59	0.35	0.85	0.51	162	18.0	7.62
Q6PGQ1	Uncharacterized protein C22orf43 OS=Homo sapiens GN=C22orf43 PE=2 SV=1 - [CV043_HUMAN]	7.86	1	1	1	1	0.85	0.75	1.03	0.92					229	25.0	4.11
P86434	Uncharacterized protein C22orf45 OS=Homo sapiens GN=C22orf45 PE=2 SV=1 - [CV045_HUMAN]	3.14	1	1	1	1					0.96	1.37	0.94	1.35	159	17.2	6.65
A6NCX1	Uncharacterized protein C2orf14-like 1 OS=Homo sapiens PE=4 SV=1 - [CB141_HUMAN]	16.10	3	1	1	1	0.71	1.03	0.63	0.93					118	12.4	9.26
Q8WU43	Uncharacterized protein C2orf15 OS=Homo sapiens GN=C2orf15 PE=2 SV=1 - [CB015_HUMAN]	16.00	1	1	1	3	1.11		0.95						125	13.7	6.30

Q68DN1	Uncharacterized protein C2orf16 OS=Homo sapiens GN=C2orf16 PE=1 SV=3 - [CB016_HUMAN]	2.72	1	2	2	4	1.26	1.29	1.06	1.10					1984	224.2	10.08
Q9NWW7	Uncharacterized protein C2orf42 OS=Homo sapiens GN=C2orf42 PE=2 SV=1 - [CB042_HUMAN]	2.26	1	1	1	1	0.12	0.11	0.13	0.12					574	64.0	8.92
Q8WWC4	Uncharacterized protein C2orf47, mitochondrial OS=Homo sapiens GN=C2orf47 PE=1 SV=1 - [CB047_HUMAN]	3.44	1	1	1	2	0.96	1.07	0.95	1.08	0.78	1.02	0.45	0.58	291	32.5	9.17
Q6NV74	Uncharacterized protein C2orf55 OS=Homo sapiens GN=C2orf55 PE=1 SV=3 - [CB055_HUMAN]	3.95	1	2	2	2					1.13	0.93	1.22	1.00	962	102.1	7.96
Q7Z7H3	Uncharacterized protein C2orf62 OS=Homo sapiens GN=C2orf62 PE=2 SV=1 - [CB062_HUMAN]	3.36	1	1	1	2	1.19	1.68	1.25	1.75	1.00	0.61	1.41	0.86	387	43.9	5.41
Q8NHS4	Uncharacterized protein C2orf63 OS=Homo sapiens GN=C2orf63 PE=2 SV=3 - [CB063_HUMAN]	1.19	1	1	1	1	0.97	1.03	1.10	1.17					586	67.2	6.49
Q8TC57	Uncharacterized protein C2orf65 OS=Homo sapiens GN=C2orf65 PE=2 SV=1 - [CB065_HUMAN]	0.94	1	1	1	1					0.22	1.32	0.25	1.51	530	59.3	6.87
A6NGG8	Uncharacterized protein C2orf71 OS=Homo sapiens GN=C2orf71 PE=1 SV=1 - [CB071_HUMAN]	1.01	1	1	1	1	0.80	0.81	0.82	0.83					1288	139.6	8.07
Q9UK00	Uncharacterized protein C3orf18 OS=Homo sapiens GN=C3orf18 PE=2 SV=2 - [CC018_HUMAN]	10.49	1	1	1	2	0.22	0.11	0.62	0.32					162	17.5	5.54
Q8ND61	Uncharacterized protein C3orf20 OS=Homo sapiens GN=C3orf20 PE=1 SV=2 - [CC020_HUMAN]	3.43	1	2	2	5	1.00	0.93	1.20	1.11	0.80	0.75	0.84	0.79	904	101.2	9.13
Q3SXR2	Uncharacterized protein C3orf36 OS=Homo sapiens GN=C3orf36 PE=2 SV=1 - [CC036_HUMAN]	22.42	1	1	1	1	0.54	0.47							165	16.9	8.29
Q86YA3	Uncharacterized protein C4orf21 OS=Homo sapiens GN=C4orf21 PE=1 SV=2 - [CD021_HUMAN]	2.17	1	2	2	4	0.87	0.52	0.85	0.51	0.32	0.37	0.30	0.36	1062	119.3	5.02
Q8N412	Uncharacterized protein C4orf37 OS=Homo sapiens GN=C4orf37 PE=2 SV=1 - [CD037_HUMAN]	2.83	1	1	1	2	0.75	0.66	0.69	0.61					459	50.6	9.57
Q8NDZ2	Uncharacterized protein C5orf25 OS=Homo sapiens GN=C5orf25 PE=1 SV=2 - [CE025_HUMAN]	3.56	4	1	3	11	0.96	0.99	0.96	1.00					872	96.8	6.62
Q96MH7	Uncharacterized protein C5orf34 OS=Homo sapiens GN=C5orf34 PE=2 SV=2 - [CE034_HUMAN]	3.76	1	2	2	4					1.33	1.27	1.11	1.07	638	72.9	7.93
A4QMS7	Uncharacterized protein C5orf49 OS=Homo sapiens GN=C5orf49 PE=2 SV=1 - [CE049_HUMAN]	5.44	1	1	1	1	2.28	2.36	2.47	2.58					147	17.0	7.59
Q5SRN2	Uncharacterized protein C6orf10 OS=Homo sapiens GN=C6orf10 PE=1 SV=3 - [CF010_HUMAN]	2.66	1	1	1	1	1.45	0.79	1.37	0.75					563	61.6	9.25
Q5T0Z8	Uncharacterized protein C6orf132 OS=Homo sapiens GN=C6orf132 PE=1 SV=4 - [CF132_HUMAN]	2.02	1	2	2	6	1.33	1.46	1.14	1.26					1188	124.0	9.45
Q5TEZ5	Uncharacterized protein C6orf163 OS=Homo sapiens GN=C6orf163 PE=1 SV=2 - [CF163_HUMAN]	5.17	1	1	2	5	0.90	0.94	1.09	1.15					329	38.5	6.96
Q5TF21	Uncharacterized protein C6orf174 OS=Homo sapiens GN=C6orf174 PE=2 SV=1 - [CF174_HUMAN]	1.06	1	1	1	5	1.08	1.08	1.21	1.40					947	103.1	6.06
Q96N11	Uncharacterized protein C7orf26 OS=Homo sapiens GN=C7orf26 PE=2 SV=1 - [CG026_HUMAN]	4.01	1	1	1	1	1.05	0.59	1.08	0.61					449	50.0	7.69
Q8WU49	Uncharacterized protein C7orf33 OS=Homo sapiens GN=C7orf33 PE=2 SV=1 - [CG033_HUMAN]	7.91	1	1	1	1	0.91	0.97	0.98	1.04					177	19.5	10.24
A4D0V7	Uncharacterized protein C7orf58 OS=Homo sapiens GN=C7orf58 PE=2 SV=1 - [CG058_HUMAN]	1.07	1	1	1	1	1.09	1.02	1.34	1.25					1026	117.4	8.09
A5D8W1	Uncharacterized protein C7orf63 OS=Homo sapiens GN=C7orf63 PE=2 SV=3 - [CG063_HUMAN]	3.29	1	2	2	3					2.00	2.44	1.50	1.83	941	105.8	7.17
Q49A92	Uncharacterized protein C8orf34 OS=Homo sapiens GN=C8orf34 PE=1 SV=2 - [CH034_HUMAN]	1.77	1	1	1	1	0.58	0.64	0.69	0.77					452	50.5	5.05
Q96LL4	Uncharacterized protein C8orf48 OS=Homo sapiens GN=C8orf48 PE=1 SV=2 - [CH048_HUMAN]	4.39	1	1	2	3	0.84	0.71	0.67	0.57					319	36.8	8.66
C9J069	Uncharacterized protein C9orf172 OS=Homo sapiens GN=C9orf172 PE=3 SV=1 - [CI172_HUMAN]	2.77	1	1	1	1					0.77	0.82	0.83	0.88	976	106.6	9.03

Q8N7X2	Uncharacterized protein C9orf173 OS=Homo sapiens GN=C9orf173 PE=2 SV=1 - [CI173_HUMAN]	10.80	1	2	2	6	1.00	0.97	1.02	0.99	1.03	1.28	0.95	1.18	389	42.8	9.99
Q9P1Z9	Uncharacterized protein C9orf174 OS=Homo sapiens GN=C9orf174 PE=1 SV=2 - [CI174_HUMAN]	1.76	1	2	2	3	1.24	1.09	1.00	0.91			1.11	0.22	1646	191.0	6.04
Q9P0K9	Uncharacterized protein C9orf4 OS=Homo sapiens GN=C9orf4 PE=2 SV=2 - [CI004_HUMAN]	3.49	1	1	1	1	1.06	1.11	1.05	1.12					344	37.2	7.62
Q6ZRZ4	Uncharacterized protein C9orf47 OS=Homo sapiens GN=C9orf47 PE=1 SV=1 - [CI047_HUMAN]	14.85	1	1	1	1	1.09	1.61	0.83	1.24					202	22.3	10.07
Q6ZTR5	Uncharacterized protein CXorf22 OS=Homo sapiens GN=CXorf22 PE=2 SV=3 - [CX022_HUMAN]	3.07	1	2	2	2	0.82	0.88	1.09	1.16					976	110.3	8.16
A2AJT9	Uncharacterized protein CXorf23 OS=Homo sapiens GN=CXorf23 PE=1 SV=1 - [CX023_HUMAN]	1.69	1	1	1	1					4.60	3.40	4.37	3.23	711	83.8	9.70
Q8TB03	Uncharacterized protein CXorf38 OS=Homo sapiens GN=CXorf38 PE=1 SV=1 - [CX038_HUMAN]	6.58	1	1	1	3	1.17	1.34	1.17	1.36					319	36.6	6.30
Q9NQM4	Uncharacterized protein CXorf41 OS=Homo sapiens GN=CXorf41 PE=2 SV=1 - [CX041_HUMAN]	7.01	1	1	1	1	0.71	0.75	0.73	0.77					214	24.1	4.08
Q8N7P7	Uncharacterized protein FLJ40521 OS=Homo sapiens PE=1 SV=1 - [YH007_HUMAN]	3.98	1	1	1	1					1.11	0.94	1.11	0.94	452	48.3	9.94
Q6ZUG5	Uncharacterized protein FLJ43738 OS=Homo sapiens PE=2 SV=1 - [YC006_HUMAN]	9.27	1	3	3	5	1.67	1.30	1.37	1.07	0.58	0.59	0.74	0.75	572	66.0	7.30
Q6ZU11	Uncharacterized protein FLJ44066 OS=Homo sapiens PE=1 SV=1 - [YD002_HUMAN]	1.30	1	1	1	1	1.15	1.39	1.22	1.49					926	104.4	8.88
Q15053	Uncharacterized protein KIAA0040 OS=Homo sapiens GN=KIAA0040 PE=2 SV=1 - [K0040_HUMAN]	23.53	1	1	1	1	0.72	1.00	0.74	1.03					153	17.0	9.51
Q9H714	Uncharacterized protein KIAA0226-like OS=Homo sapiens GN=KIAA0226L PE=1 SV=3 - [K226L_HUMAN]	4.83	1	3	3	4	1.28	1.06	1.18	0.92	0.93	0.89	0.85	0.81	662	73.4	6.04
A3KMH1	Uncharacterized protein KIAA0564 OS=Homo sapiens GN=KIAA0564 PE=1 SV=2 - [K0564_HUMAN]	2.36	1	3	3	10	1.21	1.28	1.12	1.19	0.49	0.20	0.77	0.31	1905	214.7	7.40
Q9BVV6	Uncharacterized protein KIAA0586 OS=Homo sapiens GN=KIAA0586 PE=1 SV=4 - [K0586_HUMAN]	1.17	1	1	1	1	0.21	0.32	0.40	0.62					1533	169.2	5.54
A2RU30	Uncharacterized protein KIAA0748 OS=Homo sapiens GN=KIAA0748 PE=1 SV=2 - [K0748_HUMAN]	2.50	1	1	1	1					0.43	0.40	0.43	0.40	521	59.2	5.71
Q8IV33	Uncharacterized protein KIAA0825 OS=Homo sapiens GN=KIAA0825 PE=2 SV=2 - [K0825_HUMAN]	5.18	1	4	5	7	3.96	3.62	3.91	3.57	1.26	1.13	1.02	1.05	1275	147.7	6.61
O94964	Uncharacterized protein KIAA0889 OS=Homo sapiens GN=KIAA0889 PE=1 SV=2 - [K0889_HUMAN]	3.16	1	2	2	3			0.90	0.33	0.63	0.66	0.73	0.77	1423	159.7	6.46
Q68EN5	Uncharacterized protein KIAA0895-like OS=Homo sapiens GN=KIAA0895L PE=2 SV=1 - [K895L_HUMAN]	3.82	1	1	1	1	1.08	0.71	1.07	0.71					471	53.4	9.17
Q9UPP5	Uncharacterized protein KIAA1107 OS=Homo sapiens GN=KIAA1107 PE=1 SV=2 - [K1107_HUMAN]	3.34	1	3	3	3	0.79	0.90	0.80	0.92	1.61	1.12	1.22	0.85	1409	155.6	6.19
Q2LD37	Uncharacterized protein KIAA1109 OS=Homo sapiens GN=KIAA1109 PE=1 SV=2 - [K1109_HUMAN]	1.36	1	5	5	5	1.32	1.16	1.42	1.24	0.83	0.86	0.92	0.96	5005	555.1	6.58
Q96AT1	Uncharacterized protein KIAA1143 OS=Homo sapiens GN=KIAA1143 PE=1 SV=2 - [K1143_HUMAN]	9.74	1	1	1	2	0.98	1.05	0.98	1.05					154	17.5	6.11
Q6ZU35	Uncharacterized protein KIAA1211 OS=Homo sapiens GN=KIAA1211 PE=1 SV=3 - [K1211_HUMAN]	2.03	1	2	2	3					1.77	1.85	1.69	1.76	1233	136.7	5.60
Q86T90	Uncharacterized protein KIAA1328 OS=Homo sapiens GN=KIAA1328 PE=1 SV=2 - [K1328_HUMAN]	2.77	1	1	1	1	1.46	1.51	1.22	1.27					577	65.3	8.09
Q9P2H0	Uncharacterized protein KIAA1377 OS=Homo sapiens GN=KIAA1377 PE=1 SV=3 - [K1377_HUMAN]	1.79	1	1	1	1					0.94	0.56	1.21	0.73	1117	125.8	8.76
Q9P2G4	Uncharacterized protein KIAA1383 OS=Homo sapiens GN=KIAA1383 PE=1 SV=2 - [K1383_HUMAN]	1.77	1	1	1	2	1.10	1.52	0.79	1.09					905	100.3	7.17
Q9HCI6	Uncharacterized protein KIAA1586 OS=Homo sapiens GN=KIAA1586 PE=2 SV=2 - [K1586_HUMAN]	4.19	1	2	2	2	1.13	1.57	0.84	1.18					787	89.6	7.74

Q5VZ46	Uncharacterized protein KIAA1614 OS=Homo sapiens GN=KIAA1614 PE=2 SV=3 - [K1614_HUMAN]	1.26	1	1	1	8	2.22	2.39	2.13	2.31	1.70	1.62	1.69	1.61	1190	126.5	8.82
Q5JYT7	Uncharacterized protein KIAA1755 OS=Homo sapiens GN=KIAA1755 PE=2 SV=2 - [K1755_HUMAN]	4.33	1	3	3	6	1.03	0.91	0.91	0.82	1.21	1.15	1.18	1.11	1200	130.8	6.37
Q5VW36	Uncharacterized protein KIAA1797 OS=Homo sapiens GN=KIAA1797 PE=1 SV=1 - [K1797_HUMAN]	3.16	1	3	3	3	1.57	1.90	1.49	1.81	0.72	0.71	0.90	0.89	1801	199.9	6.62
Q96PW8	Uncharacterized protein KIAA1920 OS=Homo sapiens GN=CSPG4P5 PE=5 SV=2 - [K1920_HUMAN]	2.48	1	1	1	1					1.04	0.71	5.54	3.79	444	48.6	4.77
Q8N8K9	Uncharacterized protein KIAA1958 OS=Homo sapiens GN=KIAA1958 PE=1 SV=1 - [K1958_HUMAN]	2.93	1	2	2	2	1.05	1.20	1.26	1.45					716	79.2	6.83
Q0VF49	Uncharacterized protein KIAA2012 OS=Homo sapiens GN=KIAA2012 PE=2 SV=1 - [K2012_HUMAN]	2.35	1	1	1	3	1.50	1.50	1.42	1.44					553	65.1	6.42
Q8IYS2	Uncharacterized protein KIAA2013 OS=Homo sapiens GN=KIAA2013 PE=2 SV=1 - [K2013_HUMAN]	1.10	1	1	1	2					0.51	0.78	0.61	0.92	634	69.1	8.19
Q5QGS0	Uncharacterized protein KIAA2022 OS=Homo sapiens GN=KIAA2022 PE=2 SV=1 - [K2022_HUMAN]	1.65	1	1	1	1	0.87	0.71	0.74	0.61					1516	167.4	6.40
Q6UX82	Uncharacterized protein UNQ511/PRO1026 OS=Homo sapiens GN=UNQ511/PRO1026 PE=2 SV=1 - [YA003_HUMAN]	10.13	1	1	1	3	0.44	0.19	0.62	0.27					237	25.3	5.76
O43795	Unconventional myosin-Ib OS=Homo sapiens GN=MYO1B PE=2 SV=3 - [MYO1B_HUMAN]	2.38	1	2	2	2					0.84	0.88	0.86	0.90	1136	131.9	9.38
O00160	Unconventional myosin-If OS=Homo sapiens GN=MYO1F PE=1 SV=3 - [MYO1F_HUMAN]	3.46	1	3	3	3	0.84	0.53	0.93	0.61					1098	124.8	9.11
B0I1T2	Unconventional myosin-Ig OS=Homo sapiens GN=MYO1G PE=1 SV=2 - [MYO1G_HUMAN]	2.95	1	1	1	1	0.12	0.22	0.54	1.05					1018	116.4	8.73
B2RTY4	Unconventional myosin-IXa OS=Homo sapiens GN=MYO9A PE=1 SV=2 - [MYO9A_HUMAN]	1.37	1	2	2	2	0.41	0.41			0.80	0.82	0.79	0.81	2548	292.5	8.88
Q13459	Unconventional myosin-IXb OS=Homo sapiens GN=MYO9B PE=1 SV=3 - [MYO9B_HUMAN]	1.21	1	1	1	1					1.31	1.01	1.25	0.96	2157	243.2	8.75
Q9ULV0	Unconventional myosin-Vb OS=Homo sapiens GN=MYO5B PE=1 SV=3 - [MYO5B_HUMAN]	2.22	1	5	5	71	1.23	1.06	0.82	0.78	2.90		2.15		1848	213.5	7.20
Q9NQX4	Unconventional myosin-Vc OS=Homo sapiens GN=MYO5C PE=1 SV=2 - [MYO5C_HUMAN]	1.89	1	2	2	2	0.92	0.97	0.95	0.99	1.13	0.64	4.45	2.53	1742	202.7	7.71
Q9UM54	Unconventional myosin-VI OS=Homo sapiens GN=MYO6 PE=1 SV=4 - [MYO6_HUMAN]	1.16	1	1	1	1	1.04	0.83	1.27	1.03					1294	149.6	8.53
Q13402	Unconventional myosin-VIIa OS=Homo sapiens GN=MYO7A PE=1 SV=1 - [MYO7A_HUMAN]	0.68	1	1	1	1					1.38	1.30	1.34	1.26	2215	254.2	8.56
Q9UKN7	Unconventional myosin-XV OS=Homo sapiens GN=MYO15A PE=1 SV=2 - [MYO15_HUMAN]	1.70	1	4	4	8	1.16	0.89	0.86	0.70	0.66	0.63	0.88	0.70	3530	395.0	9.17
Q92614	Unconventional myosin-XVIIIa OS=Homo sapiens GN=MYO18A PE=1 SV=3 - [MY18A_HUMAN]	1.61	1	2	2	3					0.97	0.87	1.25	1.11	2054	233.0	6.30
Q8IUG5	Unconventional myosin-XVIIIb OS=Homo sapiens GN=MYO18B PE=1 SV=1 - [MY18B_HUMAN]	0.51	1	1	1	2	1.05		1.04		0.80	0.77	1.02	0.99	2567	285.0	6.90
Q9HD67	Unconventionnal myosin-X OS=Homo sapiens GN=MYO10 PE=1 SV=3 - [MYO10_HUMAN]	0.87	1	2	2	2	2.42	2.79	2.69	3.15					2058	237.2	6.21
Q9H7C9	UPF0366 protein C11orf67 OS=Homo sapiens GN=C11orf67 PE=1 SV=1 - [CK067_HUMAN]	20.49	1	2	2	4					1.61	1.75	1.61	1.75	122	13.3	8.46
Q14667	UPF0378 protein KIAA0100 OS=Homo sapiens GN=KIAA0100 PE=1 SV=3 - [K0100_HUMAN]	2.64	1	3	3	5	0.99	0.93	0.97	0.76					2235	253.5	7.14
Q7Z6I8	UPF0461 protein C5orf24 OS=Homo sapiens GN=C5orf24 PE=1 SV=1 - [CE024_HUMAN]	7.45	1	1	1	2					1.66	2.00	1.72	2.07	188	20.1	9.85
Q9H7E9	UPF0488 protein C8orf33 OS=Homo sapiens GN=C8orf33 PE=1 SV=1 - [CH033_HUMAN]	5.68	1	1	1	2	0.93	0.79	0.71	0.77					229	25.0	9.95
Q96A73	UPF0498 protein KIAA1191 OS=Homo sapiens GN=KIAA1191 PE=1 SV=1 - [K1191_HUMAN]	2.95	1	1	1	1					1.27	1.04	0.85	0.70	305	33.2	9.39

Q8TAB5	UPF0500 protein C1orf216 OS=Homo sapiens GN=C1orf216 PE=2 SV=1 - [CA216_HUMAN]	5.68	1	1	1	1	1.07	1.03	1.04	1.02					229	25.0	5.30
Q9NUL5	UPF0515 protein C19orf66 OS=Homo sapiens GN=C19orf66 PE=2 SV=2 - [CS066_HUMAN]	2.41	1	1	1	7	0.77	0.92	0.58	0.71	0.99	0.96	0.99	0.96	291	33.1	7.25
Q969H8	UPF0556 protein C19orf10 OS=Homo sapiens GN=C19orf10 PE=1 SV=1 - [CS010_HUMAN]	6.36	1	1	1	2	1.01	1.21	1.06	1.29					173	18.8	6.68
A6NJV1	UPF0573 protein C2orf70 OS=Homo sapiens GN=C2orf70 PE=2 SV=1 - [CB070_HUMAN]	15.42	1	2	2	6	0.95	0.87	0.88	0.86	1.06	1.27	1.23	1.48	201	23.4	9.99
A8MWY0	UPF0577 protein KIAA1324-like OS=Homo sapiens GN=KIAA1324L PE=2 SV=2 - [K132L_HUMAN]	2.92	1	1	1	1	1.00	0.68	1.03	0.70					1029	113.8	6.07
Q96S19	UPF0585 protein C16orf13 OS=Homo sapiens GN=C16orf13 PE=1 SV=2 - [CP013_HUMAN]	4.41	1	1	1	2	1.11	1.07	1.12	1.09					204	22.6	7.83
Q6NVV7	UPF0595 protein C22orf40 OS=Homo sapiens GN=C22orf40 PE=2 SV=1 - [CV040_HUMAN]	8.13	1	2	2	2					0.59	0.86	0.69	1.00	123	13.9	8.00
A7E2U8	UPF0602 protein C4orf47 OS=Homo sapiens GN=C4orf47 PE=2 SV=1 - [CD047_HUMAN]	4.21	1	1	1	1	0.80	0.96	0.82	0.98					309	34.4	9.64
Q9HCM3	UPF0606 protein KIAA1549 OS=Homo sapiens GN=KIAA1549 PE=1 SV=4 - [K1549_HUMAN]	3.28	1	2	2	8	0.78	0.58	0.72	0.65	0.76	0.86	0.74	0.84	1950	210.6	6.11
Q9NWY4	UPF0609 protein C4orf27 OS=Homo sapiens GN=C4orf27 PE=1 SV=2 - [CD027_HUMAN]	2.89	1	1	1	3	0.76	0.84	0.87	0.97	1.05	0.77	0.85	0.62	346	39.4	6.80
Q9H7Z3	UPF0614 protein C14orf102 OS=Homo sapiens GN=C14orf102 PE=1 SV=3 - [CN102_HUMAN]	1.98	1	2	2	3					0.94	1.03	0.99	1.08	1164	132.6	7.68
Q6IQ49	UPF0667 protein C1orf55 OS=Homo sapiens GN=C1orf55 PE=1 SV=1 - [CA055_HUMAN]	3.33	1	1	1	2	0.94	0.95	0.95	0.97					451	49.7	6.05
Q9NX78	UPF0679 protein C14orf101 OS=Homo sapiens GN=C14orf101 PE=1 SV=3 - [CN101_HUMAN]	2.69	1	1	1	1					0.87	0.87	0.94	0.94	707	79.5	8.40
Q8N6I4	UPF0694 transmembrane protein C14orf109 OS=Homo sapiens GN=C14orf109 PE=2 SV=4 - [CN109_HUMAN]	3.68	1	1	1	1					1.42	1.23	1.14	0.99	163	18.7	8.19
Q9H3H3	UPF0696 protein C11orf68 OS=Homo sapiens GN=C11orf68 PE=1 SV=2 - [CK068_HUMAN]	5.58	1	2	2	19	####	5.32	1.01	0.40	0.82	0.78	0.85	0.84	251	27.3	5.45
Q6UW78	UPF0723 protein C11orf83 OS=Homo sapiens GN=C11orf83 PE=1 SV=2 - [CK083_HUMAN]	16.13	1	1	1	1						0.39		0.95	93	10.1	9.41
Q6UX73	UPF0764 protein C16orf89 OS=Homo sapiens GN=C16orf89 PE=2 SV=2 - [CP089_HUMAN]	9.95	1	2	2	3	1.08	1.14	1.20	1.27					402	45.4	6.19
Q15849	Urea transporter 2 OS=Homo sapiens GN=SLC14A2 PE=1 SV=3 - [UT2_HUMAN]	0.65	1	1	1	2	2.04	2.16	1.88	2.02	2.31	2.67	2.01	2.33	920	101.1	6.95
Q16831	Uridine phosphorylase 1 OS=Homo sapiens GN=UPP1 PE=1 SV=1 - [UPP1_HUMAN]	3.87	1	1	1	1	1.05	1.14	1.06	1.15					310	33.9	7.88
P07911	Uromodulin OS=Homo sapiens GN=UMOD PE=1 SV=1 - [UROM_HUMAN]	1.41	1	1	1	2	1.01	0.92	1.06	0.97					640	69.7	5.24
O00526	Uroplakin-2 OS=Homo sapiens GN=UPK2 PE=2 SV=2 - [UPK2_HUMAN]	8.70	1	1	1	1	4.75	2.87	0.96	0.58					184	19.4	10.40
O75631	Uroplakin-3a OS=Homo sapiens GN=UPK3A PE=1 SV=3 - [UPK3A_HUMAN]	4.88	1	1	1	2	0.77	0.53	0.68	0.47					287	30.7	4.83
B0FP48	Uroplakin-3b-like protein OS=Homo sapiens GN=UPK3BL PE=2 SV=1 - [UPK3L_HUMAN]	10.65	1	2	2	2	0.80	0.78	0.93	0.91	1.04	2.45	1.02	2.39	263	28.4	8.21
O75445	Usherin OS=Homo sapiens GN=USH2A PE=1 SV=3 - [USH2A_HUMAN]	0.83	1	1	2	2					0.93	1.03	1.16	1.29	5202	575.2	6.83
Q92738	USP6 N-terminal-like protein OS=Homo sapiens GN=USP6NL PE=1 SV=3 - [US6NL_HUMAN]	1.33	1	1	1	1	1.05	1.08	1.26	1.29					828	94.0	9.07
P11684	Uteroglobin OS=Homo sapiens GN=SCGB1A1 PE=1 SV=1 - [UTER_HUMAN]	12.09	1	1	1	2					2.14	1.96	2.01	1.85	91	10.0	5.06
Q16851	UTP--glucose-1-phosphate uridylyltransferase OS=Homo sapiens GN=UGP2 PE=1 SV=5 - [UGPA_HUMAN]	1.38	1	1	1	2	0.62	0.58	0.56	0.53					508	56.9	8.15

P46939	Utrophin OS=Homo sapiens GN=UTRN PE=1 SV=2 - [UTRO_HUMAN]	0.84	1	2	3	3	1.13	1.04	0.90	0.84					3433	394.2	5.33
P54727	UV excision repair protein RAD23 homolog B OS=Homo sapiens GN=RAD23B PE=1 SV=1 - [RD23B_HUMAN]	21.03	2	7	7	17					1.45	1.34	1.35	1.28	409	43.1	4.84
Q86XK7	V-set and immunoglobulin domain-containing protein 1 OS=Homo sapiens GN=VSIG1 PE=1 SV=1 - [VSIG1_HUMAN]	3.10	1	1	1	2					1.06	0.42	0.97	0.38	387	41.8	4.65
Q86VR7	V-set and immunoglobulin domain-containing protein 10-like OS=Homo sapiens GN=VSIG10L PE=2 SV=2 - [VS10L_HUMAN]	2.19	1	1	1	1	1.25	1.27	1.33	1.36					867	91.6	7.78
Q5VU13	V-set and immunoglobulin domain-containing protein 8 OS=Homo sapiens GN=VSIG8 PE=1 SV=1 - [VSIG8_HUMAN]	5.80	1	1	1	2	1.41	1.35	1.32	1.27					414	43.9	7.20
Q8TAG5	V-set and transmembrane domain-containing protein 2A OS=Homo sapiens GN=VSTM2A PE=2 SV=3 - [VTM2A_HUMAN]	7.20	1	1	1	1					0.82	0.94	0.75	0.86	236	25.8	8.27
Q8IW00	V-set and transmembrane domain-containing protein 4 OS=Homo sapiens GN=VSTM4 PE=2 SV=3 - [VSTM4_HUMAN]	4.38	1	1	1	1	0.68	0.72	0.49	0.52					320	36.1	9.86
P38606	V-type proton ATPase catalytic subunit A OS=Homo sapiens GN=ATP6V1A PE=1 SV=2 - [VATA_HUMAN]	4.38	1	2	2	6	0.57	0.61	0.58	0.63	1.06	1.01	0.70	0.67	617	68.3	5.52
P21281	V-type proton ATPase subunit B, brain isoform OS=Homo sapiens GN=ATP6V1B2 PE=1 SV=3 - [VATB2_HUMAN]	2.94	1	1	1	3	1.22	1.33	1.25	1.34					511	56.5	5.81
P86790	Vacuolar fusion protein CCZ1 homolog B OS=Homo sapiens GN=CCZ1B PE=1 SV=1 - [CCZ1B_HUMAN]	4.77	1	1	1	4					0.99	0.94	0.88	0.84	482	55.8	6.48
Q9H270	Vacuolar protein sorting-associated protein 11 homolog OS=Homo sapiens GN=VPS11 PE=1 SV=1 - [VPS11_HUMAN]	1.17	1	1	1	1	0.90	0.94	1.07	1.12					941	107.8	7.05
Q96RL7	Vacuolar protein sorting-associated protein 13A OS=Homo sapiens GN=VPS13A PE=1 SV=2 - [VP13A_HUMAN]	1.45	1	3	3	7	0.83	1.12	0.81	1.11	1.21	1.06	0.92	1.16	3174	360.0	6.33
Q5THJ4	Vacuolar protein sorting-associated protein 13D OS=Homo sapiens GN=VPS13D PE=1 SV=1 - [VP13D_HUMAN]	1.32	2	4	4	4					0.97	1.00	1.06	1.09	4387	491.5	6.58
Q8NEZ2	Vacuolar protein sorting-associated protein 37A OS=Homo sapiens GN=VPS37A PE=1 SV=1 - [VP37A_HUMAN]	3.27	1	1	1	1					1.06	1.05	1.10	1.10	397	44.3	5.57
Q9NRW7	Vacuolar protein sorting-associated protein 45 OS=Homo sapiens GN=VPS45 PE=1 SV=1 - [VPS45_HUMAN]	4.91	1	1	1	1	0.97	1.16	0.87	1.04					570	65.0	8.24
O75351	Vacuolar protein sorting-associated protein 4B OS=Homo sapiens GN=VPS4B PE=1 SV=2 - [VPS4B_HUMAN]	7.88	1	1	1	1	1.71	1.08	2.40	1.53					444	49.3	7.23
Q8N1B4	Vacuolar protein sorting-associated protein 52 homolog OS=Homo sapiens GN=VPS52 PE=1 SV=1 - [VPS52_HUMAN]	2.07	1	1	2	2	0.97	1.09	1.13	1.27					723	82.2	5.99
Q5ST30	Valine--tRNA ligase, mitochondrial OS=Homo sapiens GN=VARS2 PE=1 SV=2 - [SYVM_HUMAN]	1.60	1	1	1	1	0.62		0.66						1063	118.4	7.02
Q96JC1	Vam6/Vps39-like protein OS=Homo sapiens GN=VPS39 PE=1 SV=2 - [VPS39_HUMAN]	3.50	2	2	3	9					0.66	0.71	0.65	0.63	886	101.7	6.99
Q9ULK5	Vang-like protein 2 OS=Homo sapiens GN=VANGL2 PE=1 SV=2 - [VANG2_HUMAN]	4.03	2	2	2	2					1.40	1.30	1.37	1.27	521	59.7	9.22
P19320	Vascular cell adhesion protein 1 OS=Homo sapiens GN=VCAM1 PE=1 SV=1 - [VCAM1_HUMAN]	31.12	1	19	20	94	1.05	1.04	1.09	1.00	1.05	1.01	1.09	1.06	739	81.2	5.22
P17948	Vascular endothelial growth factor receptor 1 OS=Homo sapiens GN=FLT1 PE=1 SV=2 - [VGFR1_HUMAN]	2.91	1	2	2	2	0.92	1.03	0.87	0.99	0.80	0.84	0.90	0.94	1338	150.7	8.38
P35968	Vascular endothelial growth factor receptor 2 OS=Homo sapiens GN=KDR PE=1 SV=2 - [VGFR2_HUMAN]	1.40	1	1	1	2					0.43	0.20	0.59	0.27	1356	151.4	5.85
P35916	Vascular endothelial growth factor receptor 3 OS=Homo sapiens GN=FLT4 PE=1 SV=3 - [VGFR3_HUMAN]	11.15	1	8	8	13	0.86	0.72	0.96	0.79	1.20	1.17	1.09	1.11	1363	152.7	6.30
Q86WP2	Vasculin OS=Homo sapiens GN=GPBP1 PE=1 SV=1 - [GPBP1_HUMAN]	4.65	1	1	1	1						0.73			473	53.3	7.05
Q9HC44	Vasculin-like protein 1 OS=Homo sapiens GN=GPBP1L1 PE=1 SV=1 - [GPBL1_HUMAN]	5.91	1	1	1	1					1.23	1.16	1.16	1.09	474	52.3	6.84
P50552	Vasodilator-stimulated phosphoprotein OS=Homo sapiens GN=VASP PE=1 SV=3 - [VASP_HUMAN]	36.32	1	14	14	85	0.79	0.73	0.77	0.71	1.44	1.25	1.21	1.19	380	39.8	8.94

Q7L8A9	Vasohibin-1 OS=Homo sapiens GN=VASH1 PE=1 SV=1 - [VASH1_HUMAN]	4.93	1	2	2	2	0.81	1.26	0.72	1.13	1.03	0.73	1.22	0.86	365	40.9	9.48
Q86V25	Vasohibin-2 OS=Homo sapiens GN=VASH2 PE=2 SV=2 - [VASH2_HUMAN]	3.38	1	1	1	11	0.75	0.74	0.77	0.75	0.84	0.87	0.79	0.82	355	40.4	10.01
Q6EMK4	Vasorin OS=Homo sapiens GN=VASN PE=1 SV=1 - [VASN_HUMAN]	14.86	7	6	7	37	1.37	1.32	1.45	1.42	0.91	0.85	0.96	0.91	673	71.7	7.39
Q6UXB2	VEGF co-regulated chemokine 1 OS=Homo sapiens GN=CXCL17 PE=1 SV=1 - [VCC1_HUMAN]	15.13	1	2	2	2	0.64	0.72	0.62	0.70					119	13.8	10.96
P13611	Versican core protein OS=Homo sapiens GN=VCAN PE=1 SV=3 - [CSPG2_HUMAN]	1.33	1	2	2	2	1.07	0.68	0.96	0.59					3396	372.6	4.51
Q9H8Y1	Vertnin OS=Homo sapiens GN=VRTN PE=2 SV=1 - [VRTN_HUMAN]	1.57	1	1	1	1	0.88	0.94	0.89	0.97					702	78.2	9.01
Q6ZVE7	Vesicle transport protein GOT1A OS=Homo sapiens GN=GOLT1A PE=2 SV=1 - [GOT1A_HUMAN]	6.82	1	2	2	2					1.50	1.07	1.14	0.81	132	14.9	10.37
Q9Y3E0	Vesicle transport protein GOT1B OS=Homo sapiens GN=GOLT1B PE=1 SV=1 - [GOT1B_HUMAN]	5.80	1	1	1	2					0.61	0.56	0.85	0.78	138	15.4	10.36
Q12981	Vesicle transport protein SEC20 OS=Homo sapiens GN=BNIP1 PE=1 SV=3 - [SEC20_HUMAN]	2.63	1	1	1	1	0.82	0.77	0.72	0.68					228	26.1	8.95
Q9UEU0	Vesicle transport through interaction with t-SNAREs homolog 1B OS=Homo sapiens GN=VTI1B PE=1 SV=3 - [VTI1B_HUMAN]	12.07	1	1	1	1	0.32	0.26	0.45	0.37					232	26.7	9.04
P51809	Vesicle-associated membrane protein 7 OS=Homo sapiens GN=VAMP7 PE=1 SV=3 - [VAMP7_HUMAN]	7.73	1	2	2	5					1.02	1.11	1.10	1.19	220	24.9	8.60
Q9BV40	Vesicle-associated membrane protein 8 OS=Homo sapiens GN=VAMP8 PE=1 SV=1 - [VAMP8_HUMAN]	10.00	1	1	1	2					2.38	2.47	2.15	2.22	100	11.4	7.34
Q9P0L0	Vesicle-associated membrane protein-associated protein A OS=Homo sapiens GN=VAPA PE=1 SV=3 - [VAPA_HUMAN]	4.42	1	1	1	2					1.50	1.48	1.35	1.33	249	27.9	8.62
P46459	Vesicle-fusing ATPase OS=Homo sapiens GN=NSF PE=1 SV=3 - [NSF_HUMAN]	2.02	1	1	1	5	1.06	1.34	1.15	1.20					744	82.5	6.95
O75396	Vesicle-trafficking protein SEC22b OS=Homo sapiens GN=SEC22B PE=1 SV=4 - [SC22B_HUMAN]	4.65	1	1	1	1					0.72	0.83	0.69	0.80	215	24.6	6.92
Q9BRL7	Vesicle-trafficking protein SEC22c OS=Homo sapiens GN=SEC22C PE=2 SV=1 - [SC22C_HUMAN]	13.20	1	2	2	2					1.07	1.11	0.97	1.00	303	34.2	6.35
Q12907	Vesicular integral-membrane protein VIP36 OS=Homo sapiens GN=LMAN2 PE=1 SV=1 - [LMAN2_HUMAN]	6.74	1	2	2	6	1.05	1.13	0.98	1.06	1.04	1.01	1.23	1.19	356	40.2	6.95
Q00341	Vigilin OS=Homo sapiens GN=HDLBP PE=1 SV=2 - [VIGLN_HUMAN]	2.76	1	2	3	5	0.90	0.83	1.18	1.10	1.13	1.06	1.27	1.19	1268	141.4	6.87
O15195	Villin-like protein OS=Homo sapiens GN=VILL PE=2 SV=3 - [VILL_HUMAN]	0.82	1	1	1	1	1.11	1.24	1.10	1.24					856	95.8	7.02
P18206	Vinculin OS=Homo sapiens GN=VCL PE=1 SV=4 - [VINC_HUMAN]	43.12	1	39	39	122	0.59	0.59	0.58	0.57	0.89	0.93	0.82	0.86	1134	123.7	5.66
Q9H0V9	VIP36-like protein OS=Homo sapiens GN=LMAN2L PE=1 SV=1 - [LMA2L_HUMAN]	4.60	1	1	1	1	1.03	0.60	1.42	0.82					348	39.7	8.38
P02774	Vitamin D-binding protein OS=Homo sapiens GN=GC PE=1 SV=1 - [VTDB_HUMAN]	80.59	1	37	40	1692	1.03	0.98	1.04	0.98	1.06	1.06	1.01	1.07	474	52.9	5.54
P04070	Vitamin K-dependent protein C OS=Homo sapiens GN=PROC PE=1 SV=1 - [PROC_HUMAN]	26.68	1	9	9	81	1.24	1.16	1.26	1.16	0.90	0.88	0.94	0.89	461	52.0	6.28
P07225	Vitamin K-dependent protein S OS=Homo sapiens GN=PROS1 PE=1 SV=1 - [PROS_HUMAN]	53.25	2	32	32	401	1.05	1.04	1.06	1.05	0.82	0.82	0.82	0.81	676	75.1	5.67
P22891	Vitamin K-dependent protein Z OS=Homo sapiens GN=PROZ PE=1 SV=2 - [PROZ_HUMAN]	39.75	1	14	14	67	1.22	1.31	1.22	1.33	0.87	0.90	0.89	0.92	400	44.7	5.97
Q6UXI7	Vitrin OS=Homo sapiens GN=VIT PE=2 SV=1 - [VITRN_HUMAN]	1.62	1	1	1	1					1.74	1.99	1.53	1.75	678	73.9	9.20
P04004	Vitronectin OS=Homo sapiens GN=VTN PE=1 SV=1 - [VTNC_HUMAN]	41.84	1	22	22	1057	1.11	1.12	1.11	1.10	1.09	1.12	1.10	1.13	478	54.3	5.80

P45880	Voltage-dependent anion-selective channel protein 2 OS=Homo sapiens GN=VDAC2 PE=1 SV=2 - [VDAC2_HUMAN]	4.08	1	1	1	1	1.01	1.04	1.20	1.24					294	31.5	7.56
Q9UBN1	Voltage-dependent calcium channel gamma-4 subunit OS=Homo sapiens GN=CACNG4 PE=2 SV=1 - [CCG4_HUMAN]	4.59	1	1	1	1					0.10	0.54	0.11	0.59	327	36.6	9.10
Q9BXT2	Voltage-dependent calcium channel gamma-6 subunit OS=Homo sapiens GN=CACNG6 PE=2 SV=1 - [CCG6_HUMAN]	2.31	1	1	1	1					1.00	1.15	1.18	1.35	260	28.1	9.17
P54289	Voltage-dependent calcium channel subunit alpha-2/delta-1 OS=Homo sapiens GN=CACNA2D1 PE=1 SV=3 - [CA2D1_HUMAN]	5.71	1	4	4	9	1.22	1.12	1.11	1.03	1.10	0.84	1.12	0.81	1103	124.5	5.27
Q7Z3S7	Voltage-dependent calcium channel subunit alpha-2/delta-4 OS=Homo sapiens GN=CACNA2D4 PE=1 SV=2 - [CA2D4_HUMAN]	2.37	1	2	2	5	1.83	1.51	1.75	1.45					1137	127.9	5.35
Q13936	Voltage-dependent L-type calcium channel subunit alpha-1C OS=Homo sapiens GN=CACNA1C PE=1 SV=4 - [CAC1C_HUMAN]	0.81	1	1	2	4	0.78	0.76	0.78	0.75	0.93	0.96	0.79	0.82	2221	248.8	6.76
O00555	Voltage-dependent P/Q-type calcium channel subunit alpha-1A OS=Homo sapiens GN=CACNA1A PE=1 SV=2 - [CAC1A_HUMAN]	1.92	1	2	2	2	1.67		0.68		1.27	0.76	0.87	0.52	2505	282.2	8.84
Q5GFL6	von Willebrand factor A domain-containing protein 2 OS=Homo sapiens GN=VWA2 PE=1 SV=1 - [VWA2_HUMAN]	1.72	1	1	1	1	0.77	1.07	0.69	0.96					755	82.0	8.02
A6NCI4	von Willebrand factor A domain-containing protein 3A OS=Homo sapiens GN=VWA3A PE=2 SV=3 - [VWA3A_HUMAN]	0.84	1	1	1	3	0.46	0.49	0.50	0.54	0.80	0.89	0.70	0.79	1184	133.9	8.46
Q502W6	von Willebrand factor A domain-containing protein 3B OS=Homo sapiens GN=VWA3B PE=2 SV=3 - [VWA3B_HUMAN]	1.08	1	1	1	1	1.57	1.40	1.98	1.77					1294	145.7	7.33
O00534	von Willebrand factor A domain-containing protein 5A OS=Homo sapiens GN=VWA5A PE=2 SV=2 - [VMA5A_HUMAN]	1.78	1	1	1	1	1.00	1.30	1.05	1.36					786	86.4	6.58
Q5TIE3	von Willebrand factor A domain-containing protein 5B1 OS=Homo sapiens GN=VWA5B1 PE=1 SV=2 - [VW5B1_HUMAN]	2.70	1	2	2	3	1.09	0.88	1.30	1.05	1.40	1.54	1.24	1.37	1220	133.7	8.15
Q8N398	von Willebrand factor A domain-containing protein 5B2 OS=Homo sapiens GN=VWA5B2 PE=2 SV=2 - [VW5B2_HUMAN]	1.84	1	1	1	1					0.70	0.74	0.67	0.71	1253	132.7	6.46
B2RUY7	von Willebrand factor C domain-containing protein 2-like OS=Homo sapiens GN=VWC2L PE=2 SV=1 - [VWC2L_HUMAN]	4.05	1	1	1	2					0.58	0.35	0.96	0.58	222	24.6	5.20
P04275	von Willebrand factor OS=Homo sapiens GN=VWF PE=1 SV=4 - [VWF_HUMAN]	39.03	1	87	87	421	1.16	1.19	1.14	1.18	0.87	0.75	0.89	0.76	2813	309.1	5.48
Q9UPU3	VPS10 domain-containing receptor SorCS3 OS=Homo sapiens GN=SORCS3 PE=2 SV=2 - [SORC3_HUMAN]	1.39	1	1	1	1	1.32	1.45	1.46	1.62					1222	135.7	6.60
O43516	WAS/WASL-interacting protein family member 1 OS=Homo sapiens GN=WIPF1 PE=1 SV=3 - [WIPF1_HUMAN]	6.36	1	3	3	7					1.50	1.42	1.33	1.26	503	51.2	11.47
A6NGB9	WAS/WASL-interacting protein family member 3 OS=Homo sapiens GN=WIPF3 PE=2 SV=3 - [WIPF3_HUMAN]	4.97	1	1	1	1	1.03	0.91	1.33	1.18					483	49.3	9.99
O75717	WD repeat and HMG-box DNA-binding protein 1 OS=Homo sapiens GN=WDHD1 PE=1 SV=1 - [WDHD1_HUMAN]	2.13	1	1	2	2					0.60	0.52	0.84	0.72	1129	125.9	5.62
Q9BRX9	WD repeat domain-containing protein 83 OS=Homo sapiens GN=WDR83 PE=1 SV=1 - [WDR83_HUMAN]	4.76	1	1	1	1					0.97	1.21	1.08	1.35	315	34.3	5.58
Q6ZS81	WD repeat- and FYVE domain-containing protein 4 OS=Homo sapiens GN=WDFY4 PE=1 SV=3 - [WDFY4_HUMAN]	0.91	1	1	1	1					1.61	1.17	1.44	1.05	3184	353.4	6.32
O75083	WD repeat-containing protein 1 OS=Homo sapiens GN=WDR1 PE=1 SV=4 - [WDR1_HUMAN]	1.32	1	1	1	2	0.45	0.42	0.45	0.42					606	66.2	6.65
Q9BZH6	WD repeat-containing protein 11 OS=Homo sapiens GN=WDR11 PE=1 SV=1 - [WDR11_HUMAN]	0.98	1	1	1	4	0.55	0.58	0.51	0.53	1.29	0.94	0.88	0.64	1224	136.6	6.92
Q8NEZ3	WD repeat-containing protein 19 OS=Homo sapiens GN=WDR19 PE=1 SV=2 - [WDR19_HUMAN]	0.37	1	1	1	2	1.13	1.00	0.99	0.80					1342	151.5	6.35
Q8TBZ3	WD repeat-containing protein 20 OS=Homo sapiens GN=WDR20 PE=1 SV=2 - [WDR20_HUMAN]	2.11	1	1	1	2	1.47	1.22	1.48	1.22	0.68	0.85	0.84	1.06	569	62.9	8.00
Q9P2L0	WD repeat-containing protein 35 OS=Homo sapiens GN=WDR35 PE=1 SV=3 - [WDR35_HUMAN]	0.68	1	1	1	1	1.08	1.13	1.11	1.18					1181	133.5	6.38
Q5JTN6	WD repeat-containing protein 38 OS=Homo sapiens GN=WDR38 PE=2 SV=1 - [WDR38_HUMAN]	2.23	1	1	1	2	0.87	0.74	0.77	0.66	1.38	0.92	1.25	0.83	314	34.3	8.54

Q5JSH3	WD repeat-containing protein 44 OS=Homo sapiens GN=WDR44 PE=1 SV=1 - [WDR44_HUMAN]	8.54	1	6	6	8	0.51	0.42	0.89	0.73	1.60	1.62	1.45	1.47	913	101.3	5.45
O15213	WD repeat-containing protein 46 OS=Homo sapiens GN=WDR46 PE=1 SV=3 - [WDR46_HUMAN]	0.98	1	1	1	1					1.50	2.49	1.33	2.22	610	68.0	9.67
Q8IV35	WD repeat-containing protein 49 OS=Homo sapiens GN=WDR49 PE=1 SV=1 - [WDR49_HUMAN]	1.15	1	1	1	1					1.08	1.20	1.22	1.35	697	79.2	8.47
Q9H977	WD repeat-containing protein 54 OS=Homo sapiens GN=WDR54 PE=1 SV=1 - [WDR54_HUMAN]	5.39	1	1	1	1	0.80	0.78	0.76	0.74					334	35.9	6.20
Q86VZ2	WD repeat-containing protein 5B OS=Homo sapiens GN=WDR5B PE=2 SV=1 - [WDR5B_HUMAN]	9.39	1	1	1	1	0.81	0.45	0.76	0.42					330	36.3	7.61
Q9NNW5	WD repeat-containing protein 6 OS=Homo sapiens GN=WDR6 PE=1 SV=1 - [WDR6_HUMAN]	2.14	1	1	1	1	0.87	0.74	0.92	0.79					1121	121.6	6.87
O43379	WD repeat-containing protein 62 OS=Homo sapiens GN=WDR62 PE=1 SV=4 - [WDR62_HUMAN]	0.99	1	1	1	1	1.72	0.88	1.73	0.89					1518	165.8	5.91
Q96DN5	WD repeat-containing protein 67 OS=Homo sapiens GN=WDR67 PE=2 SV=2 - [WDR67_HUMAN]	0.84	1	1	1	1	0.93	0.54	1.20	0.71					1066	124.1	8.62
Q9Y4E6	WD repeat-containing protein 7 OS=Homo sapiens GN=WDR7 PE=2 SV=2 - [WDR7_HUMAN]	1.07	1	2	2	3	0.77	0.87	0.77	0.87	0.56	0.04	0.80	0.06	1490	163.7	6.92
Q5VTH9	WD repeat-containing protein 78 OS=Homo sapiens GN=WDR78 PE=2 SV=1 - [WDR78_HUMAN]	1.53	1	1	1	1	0.52	0.18	0.76	0.26					848	94.5	5.76
Q562E7	WD repeat-containing protein 81 OS=Homo sapiens GN=WDR81 PE=2 SV=1 - [WDR81_HUMAN]	3.15	1	1	1	1	1.02	1.05	1.16	1.19					890	95.6	5.11
Q6ZQQ6	WD repeat-containing protein 87 OS=Homo sapiens GN=WDR87 PE=2 SV=3 - [WDR87_HUMAN]	1.15	1	2	3	5	1.30	1.20	1.10	1.02					2873	333.0	7.28
A4D1P6	WD repeat-containing protein 91 OS=Homo sapiens GN=WDR91 PE=1 SV=2 - [WDR91_HUMAN]	1.61	1	1	1	1	1.26	1.51	1.19	1.46					747	83.3	6.58
Q8NDM7	WD repeat-containing protein 96 OS=Homo sapiens GN=WDR96 PE=2 SV=3 - [WDR96_HUMAN]	0.72	1	1	1	1	0.70	0.57	0.85	0.70					1665	191.9	5.99
Q9P202	Whirlin OS=Homo sapiens GN=WHRN PE=1 SV=3 - [WHRN_HUMAN]	1.65	1	1	2	4					1.21	1.16	1.20	1.14	907	96.5	8.75
P19544	Wilms tumor protein OS=Homo sapiens GN=WT1 PE=1 SV=2 [WT1_HUMAN]	6.68	1	1	1	5	1.39	1.56	1.37	1.55	1.72	1.90	1.58	1.75	449	49.2	9.00
Q7Z5K2	Wings apart-like protein homolog OS=Homo sapiens GN=WAPAL PE=1 SV=1 - [WAPL_HUMAN]	3.03	1	2	2	3	1.39	1.55	1.40	1.58	0.71	0.69	0.81	0.79	1190	132.9	5.44
P42768	Wiskott-Aldrich syndrome protein OS=Homo sapiens GN=WAS PE=1 SV=4 - [WASP_HUMAN]	4.18	1	1	1	3					0.62	0.92	1.11	1.64	502	52.9	6.64
O76076	WNT1-inducible-signaling pathway protein 2 OS=Homo sapiens GN=WISP2 PE=1 SV=1 - [WISP2_HUMAN]	10.40	1	2	2	3					0.90	0.68	1.20	0.90	250	26.8	7.88
O76024	Wolframin OS=Homo sapiens GN=WFS1 PE=1 SV=2 - [WFS1_HUMAN]	3.93	1	2	2	4	0.73	0.80	0.70	0.77	1.06	0.54	0.87	0.42	890	100.2	8.05
Q969T9	WW domain-binding protein 2 OS=Homo sapiens GN=WBP2 PE=1 SV=1 - [WBP2_HUMAN]	9.58	1	2	2	7	0.82	0.84	0.92	0.92	1.18	1.13	1.26	1.21	261	28.1	5.91
Q9GZV5	WW domain-containing transcription regulator protein 1 OS=Homo sapiens GN=WWTR1 PE=1 SV=1 - [WWTR1_HUMAN]	6.50	1	1	1	2					1.04	0.71	0.95	0.64	400	44.1	5.82
P17861	X-box-binding protein 1 OS=Homo sapiens GN=XBP1 PE=1 SV=2 - [XBP1_HUMAN]	6.13	1	1	1	1					1.52	1.25	1.40	1.15	261	28.7	9.67
Q9NP60	X-linked interleukin-1 receptor accessory protein-like 2 OS=Homo sapiens GN=IL1RAPL2 PE=2 SV=1 - [IRPL2_HUMAN]	1.90	1	1	1	1					1.06	1.22	1.05	1.21	686	78.6	6.46
Q92834	X-linked retinitis pigmentosa GTPase regulator OS=Homo sapiens GN=RPGR PE=1 SV=2 - [RPGR_HUMAN]	3.24	1	1	1	1					1.04	0.65	1.66	1.03	1020	113.3	4.86
Q6P2D8	X-ray radiation resistance-associated protein 1 OS=Homo sapiens GN=XRRA1 PE=2 SV=2 - [XRRA1_HUMAN]	0.76	1	1	1	1					0.34	0.54	0.66	1.04	792	89.8	8.92
O43895	Xaa-Pro aminopeptidase 2 OS=Homo sapiens GN=XPNPEP2 PE=1 SV=3 - [XPP2_HUMAN]	2.97	1	1	1	2					0.83	1.02	0.72	0.89	674	75.6	6.04

P12955	Xaa-Pro dipeptidase OS=Homo sapiens GN=PEPD PE=1 SV=3 - [PEPD_HUMAN]	19.07	1	8	8	36	1.01	0.95	1.08	1.01	1.03	1.02	0.94	0.99	493	54.5	6.00
A4UGR9	Xin actin-binding repeat-containing protein 2 OS=Homo sapiens GN=XIRP2 PE=1 SV=2 - [XIRP2_HUMAN]	2.52	1	4	4	5	1.54	1.73	0.79	0.89	1.02	1.11	1.06	1.15	3374	382.1	6.38
Q6PP77	XK-related protein 2 OS=Homo sapiens GN=XKRX PE=2 SV=2 - [XKR2_HUMAN]	3.79	1	1	1	2					0.66	0.30	0.95	0.43	449	52.0	8.21
Q5GH76	XK-related protein 4 OS=Homo sapiens GN=XKR4 PE=1 SV=1 - [XKR4_HUMAN]	4.92	1	2	2	2	1.05	1.23	1.29	1.52	1.58	1.33	1.26	1.05	650	71.5	7.78
Q6UX68	XK-related protein 5 OS=Homo sapiens GN=XKR5 PE=2 SV=1 - [XKR5_HUMAN]	1.60	1	1	1	1	0.78	0.84	0.77	0.84					686	74.9	6.68
Q5GH73	XK-related protein 6 OS=Homo sapiens GN=XKR6 PE=2 SV=1 - [XKR6_HUMAN]	3.28	1	1	1	2	1.22	1.03	1.19	1.01					641	71.6	7.90
Q86Y38	Xylosyltransferase 1 OS=Homo sapiens GN=XYLT1 PE=1 SV=1 - [XYLT1_HUMAN]	1.88	1	1	1	1	1.54	1.05	1.22	0.84					959	107.5	9.22
Q9H1B5	Xylosyltransferase 2 OS=Homo sapiens GN=XYLT2 PE=2 SV=2 - [XYLT2_HUMAN]	2.08	1	2	2	4	1.16	1.14	1.13	1.12					865	96.7	8.22
Q9ULM3	YEATS domain-containing protein 2 OS=Homo sapiens GN=YEATS2 PE=1 SV=2 - [YETS2_HUMAN]	2.39	1	2	2	2					0.50	0.60	0.69	0.97	1422	150.7	8.98
A6XGL0	YjeF N-terminal domain-containing protein 3 OS=Homo sapiens GN=YJEFN3 PE=1 SV=1 - [YJEN3_HUMAN]	5.69	1	1	1	5					1.05	1.20	1.10	1.26	299	32.6	6.09
Q9BYJ9	YTH domain family protein 1 OS=Homo sapiens GN=YTHDF1 PE=1 SV=1 - [YTHD1_HUMAN]	2.86	1	1	1	1	1.30	1.29	1.51	1.51					559	60.8	8.79
Q9Y5A9	YTH domain family protein 2 OS=Homo sapiens GN=YTHDF2 PE=1 SV=2 - [YTHD2_HUMAN]	5.87	1	1	1	1		0.28		0.64					579	62.3	8.79
Q9H171	Z-DNA-binding protein 1 OS=Homo sapiens GN=ZBP1 PE=1 SV=2 - [ZBP1_HUMAN]	3.03	1	1	1	1	0.94	1.03	0.93	1.03					429	46.3	6.77
Q9Y2K1	Zinc finger and BTB domain-containing protein 1 OS=Homo sapiens GN=ZBTB1 PE=1 SV=3 - [ZBTB1_HUMAN]	2.24	1	1	1	6	0.85	0.92	1.03	1.17	1.16	0.96	0.85	0.71	713	82.0	6.39
O95625	Zinc finger and BTB domain-containing protein 11 OS=Homo sapiens GN=ZBTB11 PE=1 SV=2 - [ZBT11_HUMAN]	2.18	1	1	1	2					1.00	1.28	1.09	1.40	1053	119.3	8.66
Q5SVQ8	Zinc finger and BTB domain-containing protein 41 OS=Homo sapiens GN=ZBTB41 PE=2 SV=1 - [ZBT41_HUMAN]	1.87	1	1	1	1					0.92	0.83	0.80	0.72	909	105.1	7.93
Q96K62	Zinc finger and BTB domain-containing protein 45 OS=Homo sapiens GN=ZBTB45 PE=2 SV=1 - [ZBT45_HUMAN]	1.76	1	1	1	1					0.58	0.66	0.55	0.62	511	54.0	6.92
Q96C00	Zinc finger and BTB domain-containing protein 9 OS=Homo sapiens GN=ZBTB9 PE=1 SV=1 - [ZBTB9_HUMAN]	3.17	1	1	1	1	0.63	0.68	0.86	0.94					473	50.6	6.76
Q8NBB4	Zinc finger and SCAN domain-containing protein 1 OS=Homo sapiens GN=ZSCAN1 PE=2 SV=2 - [ZSCA1_HUMAN]	3.68	1	1	1	1					4.42	3.59	3.97	3.23	408	45.3	8.54
Q7Z7L9	Zinc finger and SCAN domain-containing protein 2 OS=Homo sapiens GN=ZSCAN2 PE=2 SV=2 - [ZSCA2_HUMAN]	6.03	1	1	1	1					0.91	1.30	1.14	1.62	614	69.5	7.34
Q3MJ62	Zinc finger and SCAN domain-containing protein 23 OS=Homo sapiens GN=ZSCAN23 PE=2 SV=1 - [ZSC23_HUMAN]	3.86	1	1	1	1	1.00	1.09	0.97	1.05					389	44.9	6.32
Q86W11	Zinc finger and SCAN domain-containing protein 30 OS=Homo sapiens GN=ZSCAN30 PE=1 SV=1 - [ZSC30_HUMAN]	3.64	1	1	2	2					0.83	0.80	0.85	0.82	494	56.3	6.70
Q9BUG6	Zinc finger and SCAN domain-containing protein 5A OS=Homo sapiens GN=ZSCAN5A PE=2 SV=1 - [ZSA5A_HUMAN]	5.85	1	1	1	1	0.98	1.20	1.12	1.37					496	55.8	8.37
A6NGD5	Zinc finger and SCAN domain-containing protein 5C OS=Homo sapiens GN=ZSCAN5C PE=3 SV=1 - [ZSA5C_HUMAN]	1.01	1	1	1	1	0.53	0.60	0.71	0.80					496	55.7	8.02
O60293	Zinc finger C3H1 domain-containing protein OS=Homo sapiens GN=ZFC3H1 PE=1 SV=3 - [ZC3H1_HUMAN]	1.51	1	1	1	2	0.88	0.50	0.70	0.41					1989	226.2	8.13
Q5T200	Zinc finger CCCH domain-containing protein 13 OS=Homo sapiens GN=ZC3H13 PE=1 SV=1 - [ZC3HD_HUMAN]	1.92	1	2	2	2	1.43	1.27	1.48	1.33	0.76	0.90	0.65	0.76	1668	196.5	9.42
Q6PJT7	Zinc finger CCCH domain-containing protein 14 OS=Homo sapiens GN=ZC3H14 PE=1 SV=1 - [ZC3HE_HUMAN]	1.49	1	1	1	1	0.58	0.94	0.76	1.23					736	82.8	7.31

Q86VM9	Zinc finger CCCH domain-containing protein 18 OS=Homo sapiens GN=ZC3H18 PE=1 SV=2 - [ZCH18_HUMAN]	7.66	1	3	3	4					1.17	1.05	1.27	1.15	953	106.3	8.32
Q8IXZ2	Zinc finger CCCH domain-containing protein 3 OS=Homo sapiens GN=ZC3H3 PE=1 SV=3 - [ZC3H3_HUMAN]	0.84	1	1	1	2	1.29	1.27	1.36	1.25					948	101.9	10.95
Q8IWR0	Zinc finger CCCH domain-containing protein 7A OS=Homo sapiens GN=ZC3H7A PE=1 SV=1 - [Z3H7A_HUMAN]	1.44	1	1	1	10	1.02	0.92	1.06	0.96	1.06	0.95	1.06	0.92	971	110.5	7.30
Q9UGR2	Zinc finger CCCH domain-containing protein 7B OS=Homo sapiens GN=ZC3H7B PE=1 SV=1 - [Z3H7B_HUMAN]	2.01	2	1	2	3	1.06	0.68	1.24	0.80					993	111.5	7.17
Q8TBK6	Zinc finger CCHC domain-containing protein 10 OS=Homo sapiens GN=ZCCHC10 PE=2 SV=1 - [ZCH10_HUMAN]	7.29	1	1	1	1					3.21	2.27	3.12	2.20	192	21.0	8.53
Q6ZR62	Zinc finger CCHC domain-containing protein 16 OS=Homo sapiens GN=ZCCHC16 PE=2 SV=2 - [ZCH16_HUMAN]	10.65	1	1	1	1	1.39	1.78	1.79	2.32					310	34.7	5.80
Q9C0B9	Zinc finger CCHC domain-containing protein 2 OS=Homo sapiens GN=ZCCHC2 PE=1 SV=6 - [ZCHC2_HUMAN]	2.04	1	2	2	3	0.83	0.62	0.76	0.58	1.14	1.03	1.10	1.00	1178	125.9	7.01
Q8N567	Zinc finger CCHC domain-containing protein 9 OS=Homo sapiens GN=ZCCHC9 PE=2 SV=2 - [ZCHC9_HUMAN]	4.43	1	1	1	1					0.75	1.01	0.80	1.08	271	30.5	9.17
P37275	Zinc finger E-box-binding homeobox 1 OS=Homo sapiens GN=ZEB1 PE=1 SV=2 - [ZEB1_HUMAN]	2.31	1	2	2	3	1.09	1.46	0.87	1.17	1.42	1.50	1.15	1.21	1124	124.0	4.94
Q9BQ24	Zinc finger FYVE domain-containing protein 21 OS=Homo sapiens GN=ZFYVE21 PE=1 SV=1 - [ZFY21_HUMAN]	4.70	1	1	1	1					1.06	1.09	0.92	0.95	234	26.5	8.41
Q68DK2	Zinc finger FYVE domain-containing protein 26 OS=Homo sapiens GN=ZFYVE26 PE=1 SV=3 - [ZFY26_HUMAN]	1.77	3	2	3	4	1.30	1.17	1.03	0.93	1.06	1.14	0.86	0.93	2539	284.4	6.39
O95405	Zinc finger FYVE domain-containing protein 9 OS=Homo sapiens GN=ZFYVE9 PE=1 SV=2 - [ZFYV9_HUMAN]	4.84	1	3	3	13	0.83	0.78	0.92	0.97	1.02	0.62	1.24	0.83	1425	156.3	5.01
Q86UP3	Zinc finger homeobox protein 4 OS=Homo sapiens GN=ZFHX4 PE=1 SV=1 - [ZFHX4_HUMAN]	2.64	3	6	6	24	1.32	1.30	1.24	1.39	1.18	0.64	1.10	0.67	3567	393.5	6.37
Q9ULJ6	Zinc finger MIZ domain-containing protein 1 OS=Homo sapiens GN=ZMIZ1 PE=1 SV=3 - [ZMIZ1_HUMAN]	2.25	8	1	2	2						1.23			1067	115.4	7.46
Q5SVZ6	Zinc finger MYM-type protein 1 OS=Homo sapiens GN=ZMYM1 PE=1 SV=1 - [ZMYM1_HUMAN]	2.19	1	2	2	3					0.97	1.01	0.99	1.03	1142	128.6	7.44
Q5VZL5	Zinc finger MYM-type protein 4 OS=Homo sapiens GN=ZMYM4 PE=1 SV=1 - [ZMYM4_HUMAN]	1.23	3	1	2	12					0.78	0.78	0.96	0.92	1548	172.7	6.84
O95789	Zinc finger MYM-type protein 6 OS=Homo sapiens GN=ZMYM6 PE=1 SV=2 - [ZMYM6_HUMAN]	3.02	1	2	2	3					1.25	1.64	0.92	0.95	1325	148.0	8.22
Q9H0C1	Zinc finger MYND domain-containing protein 12 OS=Homo sapiens GN=ZMYND12 PE=2 SV=3 - [ZMY12_HUMAN]	3.84	1	1	1	3	2.18	2.49	2.32	2.68					365	41.8	6.23
P21506	Zinc finger protein 10 OS=Homo sapiens GN=ZNF10 PE=2 SV=3 - [ZNF10_HUMAN]	1.92	1	1	1	1	0.25	0.29	0.25	0.29					573	66.4	8.32
Q8IZC7	Zinc finger protein 101 OS=Homo sapiens GN=ZNF101 PE=1 SV=1 - [ZN101_HUMAN]	2.06	1	1	1	1					0.58	0.55	1.03	0.96	436	50.3	9.57
Q9UJU3	Zinc finger protein 112 homolog OS=Homo sapiens GN=ZFP112 PE=2 SV=2 - [ZF112_HUMAN]	2.19	1	1	2	3					0.54				913	105.8	8.27
P52740	Zinc finger protein 132 OS=Homo sapiens GN=ZNF132 PE=2 SV=2 - [ZN132_HUMAN]	1.84	1	1	1	4	0.77	0.76	0.75	0.75	0.85	0.81	0.83	0.79	706	80.6	8.40
Q9HCL3	Zinc finger protein 14 homolog OS=Homo sapiens GN=ZFP14 PE=2 SV=2 - [ZFP14_HUMAN]	2.81	1	1	1	1	1.01		1.01						533	63.4	8.62
Q9HCG1	Zinc finger protein 160 OS=Homo sapiens GN=ZNF160 PE=1 SV=3 - [ZN160_HUMAN]	1.96	1	1	1	1					1.23	1.14	1.27	1.18	818	94.1	9.28
O43829	Zinc finger protein 161 homolog OS=Homo sapiens GN=ZFP161 PE=2 SV=2 - [ZF161_HUMAN]	3.56	1	1	1	6	2.15		0.80		0.82	0.87	0.98	1.09	449	50.9	6.04
Q13360	Zinc finger protein 177 OS=Homo sapiens GN=ZNF177 PE=2 SV=3 - [ZN177_HUMAN]	7.17	1	2	2	2					2.03	1.38	1.93	1.31	321	36.4	8.37
P17022	Zinc finger protein 18 OS=Homo sapiens GN=ZNF18 PE=2 SV=2 - [ZNF18_HUMAN]	1.64	1	1	1	1					0.83	0.96	0.91	1.04	549	62.2	5.97

P17025	Zinc finger protein 182 OS=Homo sapiens GN=ZNF182 PE=1 SV=2 - [ZN182_HUMAN]	2.03	1	1	1	53	0.84	0.90	0.86	0.87	1.13	1.22	1.10	1.06	639	73.6	8.68
Q99676	Zinc finger protein 184 OS=Homo sapiens GN=ZNF184 PE=1 SV=4 - [ZN184_HUMAN]	2.00	1	1	1	1					1.08	1.60	1.03	1.52	751	86.1	8.07
O15231	Zinc finger protein 185 OS=Homo sapiens GN=ZNF185 PE=1 SV=3 - [ZN185_HUMAN]	7.55	1	2	2	6					1.36	1.50	1.34	1.47	689	73.5	7.01
O75820	Zinc finger protein 189 OS=Homo sapiens GN=ZNF189 PE=2 SV=2 - [ZN189_HUMAN]	5.91	1	2	2	2					0.72	0.84	0.74	0.87	626	72.9	8.50
O15535	Zinc finger protein 193 OS=Homo sapiens GN=ZNF193 PE=2 SV=1 - [ZN193_HUMAN]	2.28	1	1	1	2					1.09	1.03	0.94	0.89	394	45.9	7.31
O14628	Zinc finger protein 195 OS=Homo sapiens GN=ZNF195 PE=1 SV=2 - [ZN195_HUMAN]	2.54	1	1	1	6					0.30	0.26	0.79	0.67	629	72.3	8.09
O14709	Zinc finger protein 197 OS=Homo sapiens GN=ZNF197 PE=2 SV=1 - [ZN197_HUMAN]	1.26	1	1	1	1					1.30	1.26	1.39	1.35	1029	118.8	8.59
Q6ZN57	Zinc finger protein 2 homolog OS=Homo sapiens GN=ZFP2 PE=2 SV=1 - [ZFP2_HUMAN]	7.38	1	2	2	2	0.70	0.55	0.52	0.42					461	52.7	8.56
O14771	Zinc finger protein 213 OS=Homo sapiens GN=ZNF213 PE=2 SV=2 - [ZN213_HUMAN]	6.32	1	1	1	1					0.71	0.78	0.76	0.83	459	51.2	6.95
Q9UL58	Zinc finger protein 215 OS=Homo sapiens GN=ZNF215 PE=2 SV=2 - [ZN215_HUMAN]	8.70	1	3	3	4	0.75	0.57	0.73	0.58	0.32	0.56	0.61	1.07	517	60.0	8.72
Q9P2Y4	Zinc finger protein 219 OS=Homo sapiens GN=ZNF219 PE=1 SV=2 - [ZN219_HUMAN]	3.74	1	1	1	1					0.73		0.82		722	76.8	9.39
Q9NZL3	Zinc finger protein 224 OS=Homo sapiens GN=ZNF224 PE=1 SV=3 - [ZN224_HUMAN]	1.70	1	1	1	1	0.97	0.96	0.99	0.97					707	82.2	8.69
Q9UK10	Zinc finger protein 225 OS=Homo sapiens GN=ZNF225 PE=1 SV=2 - [ZN225_HUMAN]	2.12	1	2	2	2					0.86	0.59	1.00	0.68	706	82.4	8.91
A6NK53	Zinc finger protein 233 OS=Homo sapiens GN=ZNF233 PE=2 SV=3 - [ZN233_HUMAN]	1.94	1	1	1	1					0.91	0.95	0.88	0.92	670	76.8	8.25
Q8WUN3	Zinc finger protein 252 OS=Homo sapiens GN=ZNF252 PE=5 SV=2 - [ZN252_HUMAN]	4.25	1	2	2	2	1.21	1.21	1.26	1.28	0.78	0.89	0.96	1.11	682	78.7	8.46
Q9Y2P7	Zinc finger protein 256 OS=Homo sapiens GN=ZNF256 PE=1 SV=2 - [ZN256_HUMAN]	3.19	1	1	2	2	0.74	1.05	0.68	0.98					627	71.8	8.81
O14978	Zinc finger protein 263 OS=Homo sapiens GN=ZNF263 PE=2 SV=2 - [ZN263_HUMAN]	1.61	1	1	1	7	1.06	1.20	1.05	1.20	1.26	1.23	1.16	1.15	683	77.2	6.99
Q14587	Zinc finger protein 268 OS=Homo sapiens GN=ZNF268 PE=1 SV=2 - [ZN268_HUMAN]	1.06	1	1	1	6					0.60	0.50	0.91	0.74	947	108.3	8.87
P17035	Zinc finger protein 28 OS=Homo sapiens GN=ZNF28 PE=2 SV=5 - [ZNF28_HUMAN]	1.95	1	1	1	1					1.10	1.23	0.85	0.95	718	83.6	9.03
Q8ND82	Zinc finger protein 280C OS=Homo sapiens GN=ZNF280C PE=1 SV=1 - [Z280C_HUMAN]	1.90	1	1	1	1					0.91	0.88	0.88	0.84	737	83.0	9.11
Q6N043	Zinc finger protein 280D OS=Homo sapiens GN=ZNF280D PE=1 SV=3 - [Z280D_HUMAN]	2.96	1	1	1	1					0.72	0.86	0.73	0.87	979	109.2	8.19
P17039	Zinc finger protein 30 OS=Homo sapiens GN=ZNF30 PE=2 SV=5 - [ZNF30_HUMAN]	0.96	1	1	1	3					0.90	0.97	0.64	0.69	623	71.4	9.07
Q96RE9	Zinc finger protein 300 OS=Homo sapiens GN=ZNF300 PE=2 SV=1 - [ZN300_HUMAN]	2.15	1	1	1	2	1.01	1.04	1.13	1.18	1.04	1.02	1.03	1.02	604	68.7	8.70
Q9HCX3	Zinc finger protein 304 OS=Homo sapiens GN=ZNF304 PE=2 SV=2 - [ZN304_HUMAN]	4.40	1	2	2	2					0.74	0.40	0.81	0.43	659	75.0	8.35
Q96PQ6	Zinc finger protein 317 OS=Homo sapiens GN=ZNF317 PE=2 SV=2 - [ZN317_HUMAN]	2.52	1	1	1	1					0.69	0.86	1.12	1.40	595	67.9	8.98
A2RRD8	Zinc finger protein 320 OS=Homo sapiens GN=ZNF320 PE=2 SV=1 - [ZN320_HUMAN]	1.96	1	1	1	1					1.10	1.01	1.41	1.29	509	59.3	8.91
O75467	Zinc finger protein 324A OS=Homo sapiens GN=ZNF324 PE=1 SV=1 - [Z324A_HUMAN]	6.15	1	2	2	3	0.66	0.78	0.66	0.79	0.91	0.72	0.64	0.51	553	61.1	9.57

Q6AW86	Zinc finger protein 324B OS=Homo sapiens GN=ZNF324B PE=2 SV=1 - [Z324B_HUMAN]	2.76	1	1	1	1	4.85	5.19	4.62	4.99					544	60.6	9.69
Q86UD4	Zinc finger protein 329 OS=Homo sapiens GN=ZNF329 PE=2 SV=2 - [ZN329_HUMAN]	2.40	1	2	2	6					0.76	0.25	0.71	0.50	541	61.7	8.60
Q9UL40	Zinc finger protein 346 OS=Homo sapiens GN=ZNF346 PE=1 SV=1 - [ZN346_HUMAN]	2.38	1	1	1	1					1.03	1.14	0.97	1.06	294	32.9	9.09
Q96LW1	Zinc finger protein 354B OS=Homo sapiens GN=ZNF354B PE=2 SV=1 - [Z354B_HUMAN]	2.29	1	1	1	1	1.13	1.56	1.00	1.38					612	70.5	9.50
Q86Y25	Zinc finger protein 354C OS=Homo sapiens GN=ZNF354C PE=2 SV=1 - [Z354C_HUMAN]	3.97	1	2	2	4	1.17	1.45	1.36	1.69	0.30	0.36	0.30	0.37	554	64.8	8.05
Q96SR6	Zinc finger protein 382 OS=Homo sapiens GN=ZNF382 PE=2 SV=3 - [ZN382_HUMAN]	2.36	1	1	1	1					0.72	0.88			550	64.0	9.31
Q9H8N7	Zinc finger protein 395 OS=Homo sapiens GN=ZNF395 PE=1 SV=2 - [ZN395_HUMAN]	4.29	1	1	1	1	1.33	1.16	1.28	1.12					513	54.9	7.43
Q8NF99	Zinc finger protein 397 OS=Homo sapiens GN=ZNF397 PE=1 SV=2 - [ZN397_HUMAN]	1.87	1	1	1	1	0.50		1.15						534	61.1	7.27
Q9C0G0	Zinc finger protein 407 OS=Homo sapiens GN=ZNF407 PE=2 SV=2 - [ZN407_HUMAN]	0.89	1	1	1	1	0.93	0.93	0.84	0.84					2248	247.2	6.49
Q2M1K9	Zinc finger protein 423 OS=Homo sapiens GN=ZNF423 PE=1 SV=1 - [ZN423_HUMAN]	4.21	1	2	2	4	1.99	2.16	2.14	2.32	1.02	0.99	1.06	1.03	1284	144.5	6.89
Q6IV72	Zinc finger protein 425 OS=Homo sapiens GN=ZNF425 PE=2 SV=1 - [ZN425_HUMAN]	1.99	1	1	1	12	0.75	0.85	0.69	0.75	0.51	0.46	0.54	0.50	752	87.7	9.25
Q7Z4V0	Zinc finger protein 438 OS=Homo sapiens GN=ZNF438 PE=2 SV=1 - [ZN438_HUMAN]	3.26	1	1	1	1	0.82	0.88	0.95	1.02					828	91.8	9.39
Q9Y4E5	Zinc finger protein 451 OS=Homo sapiens GN=ZNF451 PE=2 SV=2 - [ZN451_HUMAN]	1.98	1	1	1	1					1.00				1061	121.4	6.77
Q8TAF7	Zinc finger protein 461 OS=Homo sapiens GN=ZNF461 PE=2 SV=2 - [ZN461_HUMAN]	1.60	1	1	1	1	0.92	1.03	0.79	0.90					563	66.2	8.25
Q96JM2	Zinc finger protein 462 OS=Homo sapiens GN=ZNF462 PE=1 SV=3 - [ZN462_HUMAN]	1.96	1	1	2	5					0.89	1.70	1.06	2.01	2506	284.5	7.58
Q96JG9	Zinc finger protein 469 OS=Homo sapiens GN=ZNF469 PE=1 SV=3 - [ZN469_HUMAN]	0.36	1	1	1	1					1.02	0.86	1.04	0.88	3925	409.9	7.72
Q6ECI4	Zinc finger protein 470 OS=Homo sapiens GN=ZNF470 PE=1 SV=3 - [ZN470_HUMAN]	1.53	1	1	1	1	1.12	1.28	1.15	1.32					717	82.6	8.57
Q96MN9	Zinc finger protein 488 OS=Homo sapiens GN=ZNF488 PE=1 SV=1 - [ZN488_HUMAN]	4.41	1	1	1	1	1.12	0.92	1.28	1.06					340	36.9	8.95
Q96F45	Zinc finger protein 503 OS=Homo sapiens GN=ZNF503 PE=1 SV=1 - [ZN503_HUMAN]	3.41	1	1	1	1	0.65	0.64	0.59	0.59					646	62.5	8.70
Q8TCN5	Zinc finger protein 507 OS=Homo sapiens GN=ZNF507 PE=1 SV=2 - [ZN507_HUMAN]	1.57	1	1	1	7	1.28	1.17	1.46	1.29					953	105.7	5.72
Q6AHZ1	Zinc finger protein 518A OS=Homo sapiens GN=ZNF518A PE=1 SV=2 - [Z518A_HUMAN]	2.56	1	3	3	4	0.75	0.84	0.73	0.82	1.02	0.93	1.04	0.95	1483	166.7	9.28
Q96K83	Zinc finger protein 521 OS=Homo sapiens GN=ZNF521 PE=1 SV=1 - [ZN521_HUMAN]	2.75	1	1	2	2	1.78		1.49						1311	147.8	7.01
Q9HCE3	Zinc finger protein 532 OS=Homo sapiens GN=ZNF532 PE=1 SV=2 - [ZN532_HUMAN]	1.38	1	1	1	2	0.26	0.24	0.18	0.17					1301	141.6	8.65
Q76KX8	Zinc finger protein 534 OS=Homo sapiens GN=ZNF534 PE=2 SV=1 - [ZN534_HUMAN]	7.12	1	2	3	5	0.59	1.20	0.64	0.86	1.33	0.88	1.41	0.93	674	77.1	9.25
Q7Z398	Zinc finger protein 550 OS=Homo sapiens GN=ZNF550 PE=1 SV=2 - [ZN550_HUMAN]	2.61	1	1	1	1					1.29	1.33	1.01	1.04	422	48.4	8.63
Q7Z340	Zinc finger protein 551 OS=Homo sapiens GN=ZNF551 PE=1 SV=3 - [ZN551_HUMAN]	1.04	1	1	1	1	1.03	1.19	1.04	1.22					670	77.5	8.35
Q86TJ5	Zinc finger protein 554 OS=Homo sapiens GN=ZNF554 PE=2 SV=1 - [ZN554_HUMAN]	2.79	1	1	1	1					0.82	0.75	0.98	0.90	538	60.5	7.61

Q8NEP9	Zinc finger protein 555 OS=Homo sapiens GN=ZNF555 PE=2 SV=4 - [ZN555_HUMAN]	1.43	1	1	1	1	0.77	1.03	0.68	0.91					628	73.0	9.04
Q9BR84	Zinc finger protein 559 OS=Homo sapiens GN=ZNF559 PE=2 SV=1 - [ZN559_HUMAN]	2.97	1	1	1	3					0.17	0.20	0.33	0.43	538	62.3	8.56
Q8N587	Zinc finger protein 561 OS=Homo sapiens GN=ZNF561 PE=2 SV=2 - [ZN561_HUMAN]	4.32	2	2	2	2					0.34	0.39	0.68	0.78	486	55.2	8.82
Q8TA94	Zinc finger protein 563 OS=Homo sapiens GN=ZNF563 PE=2 SV=1 - [ZN563_HUMAN]	5.46	1	1	1	1	0.86	0.87	0.77	0.78					476	55.4	9.14
Q8TBZ8	Zinc finger protein 564 OS=Homo sapiens GN=ZNF564 PE=2 SV=1 - [ZN564_HUMAN]	6.51	1	2	2	2	0.85	0.47	1.01	0.57	0.79	2.03	0.83	2.14	553	63.7	8.68
Q8NAF0	Zinc finger protein 579 OS=Homo sapiens GN=ZNF579 PE=1 SV=2 - [ZN579_HUMAN]	1.25	1	1	1	1					0.10	0.14	0.12	0.17	562	60.5	8.69
Q86UQ0	Zinc finger protein 589 OS=Homo sapiens GN=ZNF589 PE=1 SV=1 - [ZN589_HUMAN]	3.02	1	1	1	1					0.99	0.96	0.95	0.92	364	41.2	8.27
Q92610	Zinc finger protein 592 OS=Homo sapiens GN=ZNF592 PE=1 SV=2 - [ZN592_HUMAN]	1.34	1	1	1	1					0.26	0.24	0.28	0.26	1267	137.4	7.84
O00488	Zinc finger protein 593 OS=Homo sapiens GN=ZNF593 PE=1 SV=2 - [ZN593_HUMAN]	14.18	1	1	1	1					1.29	1.34	1.15	1.20	134	15.2	9.82
Q86UK7	Zinc finger protein 598 OS=Homo sapiens GN=ZNF598 PE=1 SV=1 - [ZN598_HUMAN]	1.66	1	1	1	1					0.86	1.52	7.65	####	904	98.6	8.40
Q8WXB4	Zinc finger protein 606 OS=Homo sapiens GN=ZNF606 PE=2 SV=1 - [ZN606_HUMAN]	1.01	1	1	1	1					0.72				792	91.8	7.88
Q96SK3	Zinc finger protein 607 OS=Homo sapiens GN=ZNF607 PE=2 SV=3 - [ZN607_HUMAN]	0.86	1	1	1	1					1.03	0.59	0.89	0.51	696	80.5	8.60
Q8N2I2	Zinc finger protein 619 OS=Homo sapiens GN=ZNF619 PE=2 SV=1 - [ZN619_HUMAN]	5.36	1	2	2	6	0.83	0.90	0.97	1.06	0.91	0.85	1.02	0.97	560	63.3	8.35
Q9P2J8	Zinc finger protein 624 OS=Homo sapiens GN=ZNF624 PE=2 SV=3 - [ZN624_HUMAN]	1.50	1	1	1	1					3.35	2.04	3.37	2.06	865	99.9	8.88
Q14966	Zinc finger protein 638 OS=Homo sapiens GN=ZNF638 PE=1 SV=2 - [ZN638_HUMAN]	2.28	1	2	2	4	1.36	0.99	1.06	0.78					1978	220.5	6.38
Q9NPA5	Zinc finger protein 64 homolog, isoforms 1 and 2 OS=Homo sapiens GN=ZFP64 PE=1 SV=3 - [ZF64A_HUMAN]	3.23	4	1	2	2	1.14	1.21	1.21	1.28					681	74.6	8.40
Q9UJL9	Zinc finger protein 643 OS=Homo sapiens GN=ZNF643 PE=2 SV=2 - [ZN643_HUMAN]	2.06	1	2	2	3					1.15	1.38	1.22	1.46	534	61.4	9.01
Q9Y2D9	Zinc finger protein 652 OS=Homo sapiens GN=ZNF652 PE=1 SV=3 - [ZN652_HUMAN]	4.79	1	1	1	1	0.16	0.29							606	69.7	8.65
Q96CK0	Zinc finger protein 653 OS=Homo sapiens GN=ZNF653 PE=1 SV=1 - [ZN653_HUMAN]	2.11	1	1	1	1	1.45	1.28	1.77	1.57					615	67.2	6.98
Q6ZS27	Zinc finger protein 662 OS=Homo sapiens GN=ZNF662 PE=2 SV=1 - [ZN662_HUMAN]	15.49	1	3	3	8					0.71	0.98	0.82	0.83	426	48.5	7.66
Q2M3X9	Zinc finger protein 674 OS=Homo sapiens GN=ZNF674 PE=1 SV=1 - [ZN674_HUMAN]	1.89	1	1	1	2							0.41	0.23	581	67.2	9.16
Q8TD23	Zinc finger protein 675 OS=Homo sapiens GN=ZNF675 PE=1 SV=3 - [ZN675_HUMAN]	2.99	1	1	1	2	1.12	1.12	1.15	1.17					568	66.3	8.87
O95780	Zinc finger protein 682 OS=Homo sapiens GN=ZNF682 PE=2 SV=1 - [ZN682_HUMAN]	2.61	1	1	1	1						1.24			498	58.3	9.00
Q5TEC3	Zinc finger protein 697 OS=Homo sapiens GN=ZNF697 PE=2 SV=2 - [ZN697_HUMAN]	2.20	1	1	1	33	0.88	1.03	0.84	0.97	1.09	0.83	1.12	0.85	545	60.4	7.28
Q9UC06	Zinc finger protein 70 OS=Homo sapiens GN=ZNF70 PE=2 SV=2 - [ZNF70_HUMAN]	4.71	1	1	1	1	1.00	0.96	0.98	0.94					446	50.8	8.31
Q96C28	Zinc finger protein 707 OS=Homo sapiens GN=ZNF707 PE=2 SV=2 - [ZN707_HUMAN]	1.89	1	1	1	1					1.12	0.25	0.86	0.19	371	43.1	9.61
Q8N859	Zinc finger protein 713 OS=Homo sapiens GN=ZNF713 PE=2 SV=1 - [ZN713_HUMAN]	1.86	1	1	1	1					1.05	1.02	0.83	0.81	430	50.1	6.98

Q96N38	Zinc finger protein 714 OS=Homo sapiens GN=ZNF714 PE=2 SV=2 - [ZN714_HUMAN]	2.16	1	1	1	1	1.28	1.33	1.07	1.11					555	64.0	9.10
O43830	Zinc finger protein 73 OS=Homo sapiens GN=ZNF73 PE=3 SV=1 - [ZNF73_HUMAN]	6.75	1	1	1	1	0.94	0.93	0.91	0.91					326	38.2	9.17
O43361	Zinc finger protein 749 OS=Homo sapiens GN=ZNF749 PE=1 SV=2 - [ZN749_HUMAN]	2.44	1	1	1	1	1.51	1.56	1.42	1.48					778	90.3	8.70
Q96N20	Zinc finger protein 75A OS=Homo sapiens GN=ZNF75A PE=2 SV=1 - [ZN75A_HUMAN]	6.76	1	1	1	1	0.95	1.02	0.69	0.74					296	34.7	9.48
Q86XN6	Zinc finger protein 761 OS=Homo sapiens GN=ZNF761 PE=2 SV=2 - [ZN761_HUMAN]	1.21	1	1	1	2	1.18	1.00	1.06	0.90					746	87.7	9.00
Q0D2J5	Zinc finger protein 763 OS=Homo sapiens GN=ZNF763 PE=2 SV=2 - [ZN763_HUMAN]	3.81	1	1	1	1					0.48	0.35	0.81	0.59	394	46.1	9.13
Q68DY9	Zinc finger protein 772 OS=Homo sapiens GN=ZNF772 PE=2 SV=2 - [ZN772_HUMAN]	3.89	1	2	2	3	1.10	1.04	1.10	1.05	0.45	0.59	0.80	1.03	489	55.3	7.71
Q96BV0	Zinc finger protein 775 OS=Homo sapiens GN=ZNF775 PE=1 SV=2 - [ZN775_HUMAN]	1.68	1	1	1	1					0.80	0.73	0.99	0.89	537	59.7	10.17
Q8NCA9	Zinc finger protein 784 OS=Homo sapiens GN=ZNF784 PE=2 SV=1 - [ZN784_HUMAN]	9.91	1	2	2	2	0.83	0.74	0.85	0.76					323	34.2	7.80
Q8N393	Zinc finger protein 786 OS=Homo sapiens GN=ZNF786 PE=2 SV=2 - [ZN786_HUMAN]	1.66	1	1	1	1					2.04	2.30	1.17	1.32	782	89.8	9.13
Q5FWF6	Zinc finger protein 789 OS=Homo sapiens GN=ZNF789 PE=2 SV=3 - [ZN789_HUMAN]	1.18	1	1	1	2					0.88	0.82	1.03	0.95	425	50.0	9.10
Q6PG37	Zinc finger protein 790 OS=Homo sapiens GN=ZNF790 PE=2 SV=2 - [ZN790_HUMAN]	2.36	1	1	1	2					1.82	1.97	1.27	1.37	636	74.5	8.47
Q3KP31	Zinc finger protein 791 OS=Homo sapiens GN=ZNF791 PE=2 SV=1 - [ZN791_HUMAN]	1.74	1	1	1	1					0.63	0.80	0.66	0.84	576	66.8	9.22
Q5CZA5	Zinc finger protein 805 OS=Homo sapiens GN=ZNF805 PE=2 SV=3 - [ZN805_HUMAN]	1.91	1	1	1	1	1.87	1.80	1.73	1.67					627	71.1	7.69
Q8N4W9	Zinc finger protein 808 OS=Homo sapiens GN=ZNF808 PE=2 SV=2 - [ZN808_HUMAN]	1.44	1	1	1	1					0.72	0.71	0.66	0.66	903	104.8	9.48
P51508	Zinc finger protein 81 OS=Homo sapiens GN=ZNF81 PE=1 SV=3 - [ZNF81_HUMAN]	2.12	1	1	1	1	0.96	0.95	1.04	1.03					661	75.9	8.68
Q6ZN06	Zinc finger protein 813 OS=Homo sapiens GN=ZNF813 PE=2 SV=2 - [ZN813_HUMAN]	2.92	1	2	2	2	1.33	1.44	1.06	1.16					617	71.7	9.36
Q8N141	Zinc finger protein 82 homolog OS=Homo sapiens GN=ZFP82 PE=2 SV=1 - [ZFP82_HUMAN]	1.88	1	1	1	1	0.38	1.10	0.26	0.77					532	62.5	8.98
A8K0R7	Zinc finger protein 839 OS=Homo sapiens GN=ZNF839 PE=2 SV=1 - [ZN839_HUMAN]	10.36	1	4	4	4	1.00	1.23	1.13	1.15	1.41	1.69	1.03	1.24	811	87.4	6.54
P51523	Zinc finger protein 84 OS=Homo sapiens GN=ZNF84 PE=2 SV=2 - [ZNF84_HUMAN]	3.25	1	1	1	1					1.01	1.11	0.92	1.02	738	85.4	8.78
O60290	Zinc finger protein 862 OS=Homo sapiens GN=ZNF862 PE=2 SV=2 - [ZN862_HUMAN]	1.45	1	1	1	1	0.84	0.73	0.83	0.72					1169	131.6	7.23
C9JN71	Zinc finger protein 878 OS=Homo sapiens GN=ZNF878 PE=3 SV=2 - [ZN878_HUMAN]	5.84	1	2	2	2	0.78	0.82			1.19	1.13	1.10	1.05	531	61.5	9.45
P0CJ79	Zinc finger protein 888 OS=Homo sapiens GN=ZNF888 PE=3 SV=1 - [ZN888_HUMAN]	1.40	1	1	1	1	1.06	1.25	1.16	1.39					500	58.7	9.09
Q03938	Zinc finger protein 90 OS=Homo sapiens GN=ZNF90 PE=2 SV=3 - [ZNF90_HUMAN]	2.66	1	1	1	1	0.38	0.19	0.57	0.28					601	69.0	9.22
A8MXY4	Zinc finger protein 99 OS=Homo sapiens GN=ZNF99 PE=2 SV=2 - [ZNF99_HUMAN]	0.87	1	1	1	5	1.03	0.98	0.97	0.98	0.81	0.81	0.93	0.93	1036	120.0	9.31
Q6ZN30	Zinc finger protein basonuclin-2 OS=Homo sapiens GN=BNC2 PE=2 SV=1 - [BNC2_HUMAN]	1.00	1	1	1	5	1.19	1.02	1.09	0.95	1.04	1.13	0.95	1.06	1099	122.3	6.52
Q86V15	Zinc finger protein castor homolog 1 OS=Homo sapiens GN=CASZ1 PE=2 SV=4 - [CASZ1_HUMAN]	0.74	1	1	1	1					0.86	1.04	0.89	1.08	1759	189.9	7.03

Q5VTD9	Zinc finger protein Gfi-1b OS=Homo sapiens GN=GFI1B PE=1 SV=1 - [GFI1B_HUMAN]	7.27	1	1	1	1	1.10	1.27	1.07	1.25					330	37.5	9.04
P10070	Zinc finger protein GLI2 OS=Homo sapiens GN=GLI2 PE=1 SV=4 - [GLI2_HUMAN]	1.32	1	1	1	4	1.00	0.88	1.25	1.11					1586	167.7	7.33
P10075	Zinc finger protein GLI4 OS=Homo sapiens GN=GLI4 PE=1 SV=2 - [GLI4_HUMAN]	4.52	1	1	1	2	1.14	1.36	1.19	1.42					376	41.1	8.81
Q6DJT9	Zinc finger protein PLAG1 OS=Homo sapiens GN=PLAG1 PE=1 SV=1 - [PLAG1_HUMAN]	3.40	1	1	1	3	1.19	1.14	1.24	1.18					500	55.9	8.56
Q63HK3	Zinc finger protein with KRAB and SCAN domains 2 OS=Homo sapiens GN=ZKSCAN2 PE=1 SV=2 - [ZKSC2_HUMAN]	4.65	1	2	2	9	0.89	0.90	0.99	0.94	1.31	1.36	1.25	1.31	967	110.9	8.35
Q9BRR0	Zinc finger protein with KRAB and SCAN domains 3 OS=Homo sapiens GN=ZKSCAN3 PE=1 SV=2 - [ZKSC3_HUMAN]	1.67	1	1	1	1	0.71	0.83	0.72	0.84					538	60.6	6.40
Q9Y2L8	Zinc finger protein with KRAB and SCAN domains 5 OS=Homo sapiens GN=ZKSCAN5 PE=1 SV=1 - [ZKSC5_HUMAN]	1.67	1	1	1	1					0.95	0.83	1.13	1.00	839	96.8	7.56
Q8WW38	Zinc finger protein ZFPM2 OS=Homo sapiens GN=ZFPM2 PE=1 SV=3 - [FOG2_HUMAN]	1.48	1	1	1	2	1.16	1.08	1.16	1.08					1151	128.1	6.47
Q9H040	Zinc finger RAD18 domain-containing protein C1orf124 OS=Homo sapiens GN=C1orf124 PE=1 SV=2 - [CA124_HUMAN]	4.70	1	1	1	3					1.15	1.23	1.11	1.19	489	55.1	8.15
Q5FWF4	Zinc finger Ran-binding domain-containing protein 3 OS=Homo sapiens GN=ZRANB3 PE=2 SV=2 - [ZRAB3_HUMAN]	1.39	1	1	1	2	1.76	1.38	1.26	1.00					1079	123.2	8.44
Q9UPR6	Zinc finger RNA-binding protein 2 OS=Homo sapiens GN=ZFR2 PE=2 SV=3 - [ZFR2_HUMAN]	1.49	1	1	1	1					0.95	0.76	0.96	0.78	939	101.3	8.44
Q96MP5	Zinc finger SWIM domain-containing protein 3 OS=Homo sapiens GN=ZSWIM3 PE=1 SV=2 - [ZSWM3_HUMAN]	1.44	1	1	1	2					0.91	0.61	1.01	0.67	696	79.4	7.47
Q19AV6	Zinc finger SWIM domain-containing protein 7 OS=Homo sapiens GN=ZSWIM7 PE=1 SV=1 - [ZSWM7_HUMAN]	8.57	1	1	1	1	0.06	0.05	0.13	0.12					140	15.4	7.15
A7E2V4	Zinc finger SWIM domain-containing protein KIAA0913 OS=Homo sapiens GN=KIAA0913 PE=1 SV=1 - [K0913_HUMAN]	5.44	1	4	4	7					0.69	0.67	0.64	0.78	1837	197.2	6.80
Q9UHF7	Zinc finger transcription factor Trps1 OS=Homo sapiens GN=TRPS1 PE=1 SV=2 - [TRPS1_HUMAN]	2.42	1	2	2	4	0.80	0.85	0.72	0.77	0.83		0.60		1281	141.4	7.58
P17010	Zinc finger X-chromosomal protein OS=Homo sapiens GN=ZFX PE=1 SV=2 - [ZFX_HUMAN]	1.61	1	1	1	1	1.27	1.37	1.40	1.51					805	90.5	6.14
P08048	Zinc finger Y-chromosomal protein OS=Homo sapiens GN=ZFY PE=1 SV=3 - [ZFY_HUMAN]	1.62	1	1	1	4					1.06	1.22	0.96	1.14	801	90.4	6.06
O43149	Zinc finger ZZ-type and EF-hand domain-containing protein 1 OS=Homo sapiens GN=ZZEF1 PE=1 SV=6 - [ZZEF1_HUMAN]	0.44	1	2	2	3					1.43	1.37	1.28	1.23	2961	330.9	5.95
Q9Y6X8	Zinc fingers and homeoboxes protein 2 OS=Homo sapiens GN=ZHX2 PE=1 SV=1 - [ZHX2_HUMAN]	1.43	1	1	1	1	0.32	0.24	0.25	0.19					837	92.2	6.86
O14863	Zinc transporter 4 OS=Homo sapiens GN=SLC30A4 PE=2 SV=2 - [ZNT4_HUMAN]	9.09	1	1	2	2					0.41	1.04	0.40	1.01	429	47.5	6.58
Q6NXT4	Zinc transporter 6 OS=Homo sapiens GN=SLC30A6 PE=1 SV=2 - [ZNT6_HUMAN]	1.30	1	1	1	3					1.20	1.39	0.97	1.12	461	51.1	9.16
Q8NEW0	Zinc transporter 7 OS=Homo sapiens GN=SLC30A7 PE=1 SV=1 - [ZNT7_HUMAN]	4.26	1	1	1	1	1.04	1.17	1.07	1.20					376	41.6	6.95
Q9C0K1	Zinc transporter ZIP8 OS=Homo sapiens GN=SLC39A8 PE=1 SV=1 - [S39A8_HUMAN]	4.35	1	1	1	1					1.10	0.83	1.40	1.05	460	49.6	6.09
P25311	Zinc-alpha-2-glycoprotein OS=Homo sapiens GN=AZGP1 PE=1 SV=2 - [ZA2G_HUMAN]	57.38	2	19	19	995	1.09	0.98	1.09	0.96	0.91	0.92	0.89	0.94	298	34.2	6.05
Q12836	Zona pellucida sperm-binding protein 4 OS=Homo sapiens GN=ZP4 PE=2 SV=1 - [ZP4_HUMAN]	2.22	1	1	1	2					0.75	0.99	0.77	1.02	540	59.4	6.68
Q9Y493	Zonadhesin OS=Homo sapiens GN=ZAN PE=2 SV=4 - [ZAN_HUMAN]	0.60	1	1	1	4	1.34		1.09						2812	305.5	6.11
O60844	Zymogen granule membrane protein 16 OS=Homo sapiens GN=ZG16 PE=1 SV=1 - [ZG16_HUMAN]	10.78	1	1	1	3					0.92	1.07	0.97	1.13	167	18.2	9.36

Q96DA0	Zymogen granule protein 16 homolog B OS=Homo sapiens GN=ZG16B PE=1 SV=3 - [ZG16B_HUMAN]	4.33	1	1	1	3					2.22	2.18	0.79	0.77	208	22.7	7.39
Q15942	Zyxin OS=Homo sapiens GN=ZYX PE=1 SV=1 - [ZYX_HUMAN]	31.99	1	14	14	115	0.40	0.33	0.48	0.39	1.06	1.03	0.91	0.89	572	61.2	6.67
