# Supplementary material for: Increased circulating resistin levels in early-onset breast cancer patients of normal body mass index correlate with lymph node negative involvement and longer disease free survival: a multi-center POSH cohort serum proteomics study
Source: Breast Cancer Res. 2018 Mar 22;20:19. doi: 10.1186/s13058-018-0938-6 (PMC5863447; doi:10.1186/s13058-018-0938-6)
Supplement: Supplementary file 4 — Section 4 presenting differentially expressed proteins in good vs poor outcome groups. (PDF 489 kb) [file 13058_2018_938_MOESM4_ESM.pdf]

Supplementary Section 4. Differentially expressed proteins

Accession	Description	Log2 [117/119]	Log2 [118/119]	Log2 [117/121]	Log2 [118/121]	Log2 [114/116]	Log2 [114/115]	Log2 [113/116]	Log2 [113/115]	Mean iTRAQ log2ratio (good vs. poor outcome)	ttest
Q9Y5V3	Melanoma-associated antigen D1 OS=Homo sapiens GN=MAGED1 PE=1 SV=3 - [MAGD1_HUMAN]					3.42	3.90	3.49	3.98	3.7	0.00
O00139	Kinesin-like protein KIF2A OS=Homo sapiens GN=KIF2A PE=1 SV=3 - [KIF2A_HUMAN]					3.65	3.23	3.58	3.16	3.4	0.00
Q5M9N0	Coiled-coil domain-containing protein 158 OS=Homo sapiens GN=CCDC158 PE=2 SV=2 - [CD158_HUMAN]					2.03	3.18	2.71	3.87	2.9	0.00
Q9UQ72	Pregnancy-specific beta-1-glycoprotein 11 OS=Homo sapiens GN=PSG11 PE=2 SV=3 - [PSG11_HUMAN]	2.68	3.15	2.65	3.15	2.80	2.78	2.76	2.74	2.8	0.00
P11464	Pregnancy-specific beta-1-glycoprotein 1 OS=Homo sapiens GN=PSG1 PE=2 SV=1 - [PSG1_HUMAN]	2.22	2.68	2.22	2.54	2.71	2.82	2.70	2.80	2.6	0.00
P00709	Alpha-lactalbumin OS=Homo sapiens GN=LALBA PE=1 SV=1 - [LALBA_HUMAN]					3.03	2.30	2.58	1.84	2.4	0.00

P0C7Q5	Solute carrier family 35 member G4 OS=Homo sapiens GN=SLC35G4 PE=3 SV=1 - [S35G4_HUMAN]	1.97	2.49	2.18	2.72					2.3	0.00
Q13046	Putative pregnancy-specific beta-1-glycoprotein 7 OS=Homo sapiens GN=PSG7 PE=5 SV=2 - [PSG7_HUMAN]	1.92	2.29	1.83	2.34	2.70	2.49	2.47	2.26	2.3	0.00
Q5U623	Activating transcription factor 7-interacting protein 2 OS=Homo sapiens GN=ATF7IP2 PE=1 SV=2 - [MCAF2_HUMAN]					2.47	2.04	2.36	1.92	2.2	0.00
Q8N2N9	Ankyrin repeat domain-containing protein 36B OS=Homo sapiens GN=ANKRD36B PE=2 SV=4 - [AN36B_HUMAN]	2.65	2.50	2.57	2.44	0.70				2.2	0.00
O43184	Disintegrin and metalloproteinase domain-containing protein 12 OS=Homo sapiens GN=ADAM12 PE=1 SV=3 - [ADA12_HUMAN]	1.61	1.80	1.70	1.85	2.57	2.54	2.42	2.51	2.1	0.00

P11465	Pregnancy-specific beta-1-glycoprotein 2 OS=Homo sapiens GN=PSG2 PE=2 SV=2 - [PSG2_HUMAN]	-0.08	0.00	0.10	0.19	3.95	3.78	3.95	3.77	2.0	0.03
Q8IYK4	Procollagen galactosyltransferase 2 OS=Homo sapiens GN=GLT25D2 PE=1 SV=1 - [GT252_HUMAN]	1.73	2.06	1.80	2.12					1.9	0.00
P33992	DNA replication licensing factor MCM5 OS=Homo sapiens GN=MCM5 PE=1 SV=5 - [MCM5_HUMAN]	1.18	2.54	1.12	2.68					1.9	0.02
Q00888	Pregnancy-specific beta-1-glycoprotein 4 OS=Homo sapiens GN=PSG4 PE=2 SV=3 - [PSG4_HUMAN]	1.31	1.84	1.22	1.66	2.38	2.12	2.33	2.06	1.9	0.00
Q9Y4C4	Malignant fibrous histiocytoma-amplified sequence 1 OS=Homo sapiens GN=MFHAS1 PE=1 SV=2 - [MFHA1_HUMAN]					2.20	1.76	1.89	1.45	1.8	0.00
Q15238	Pregnancy-specific beta-1-glycoprotein 5 OS=Homo sapiens GN=PSG5 PE=1 SV=3 - [PSG5_HUMAN]	1.78	2.14	1.79	2.10	1.70	1.68	1.66	1.63	1.8	0.00

Q13219	Pappalysin-1 OS=Homo sapiens GN=PAPPA PE=1 SV=3 - [PAPP1_HUMAN]	1.41	1.97	1.57	1.99					1.7	0.00
Q00887	Pregnancy-specific beta-1-glycoprotein 9 OS=Homo sapiens GN=PSG9 PE=2 SV=2 - [PSG9_HUMAN]	1.24	1.48	1.23	1.48	1.87	1.78	1.68	1.59	1.5	0.00
P01215	Glycoprotein hormones alpha chain OS=Homo sapiens GN=CGA PE=1 SV=1 - [GLHA_HUMAN]					1.55	1.58	1.47	1.49	1.5	0.00
P01243	Chorionic somatomammotropin hormone OS=Homo sapiens GN=CSH1 PE=1 SV=2 - [CSH_HUMAN]					1.11	1.78	1.22	1.85	1.5	0.00
Q16643	Drebrin OS=Homo sapiens GN=DBN1 PE=1 SV=4 - [DREB_HUMAN]					1.46	1.52	1.45	1.50	1.5	0.00
Q9HD67	Unconventional myosin-X OS=Homo sapiens GN=MYO10 PE=1 SV=3 - [MYO10_HUMAN]	1.27	1.48	1.43	1.65					1.5	0.00
Q7Z2Z1	Treslin OS=Homo sapiens GN=TICRR PE=1 SV=2 - [TICRR_HUMAN]	0.13	0.30	-0.01	0.17	2.53	2.66	2.54	2.67	1.4	0.02
Q9NT68	Teneurin-2 OS=Homo sapiens GN=ODZ2 PE=1 SV=3 - [TEN2_HUMAN]					1.40	1.56	1.15	1.30	1.4	0.00

P60985	Keratinocyte differentiation-associated protein OS=Homo sapiens GN=KRTDAP PE=1 SV=1 - [KTDAP_HUMAN]					1.37	1.36	1.30	1.29	1.3	0.00
Q8IVL6	Prolyl 3-hydroxylase 3 OS=Homo sapiens GN=LEPREL2 PE=2 SV=1 - [P3H3_HUMAN]					1.25	1.30	1.36	1.41	1.3	0.00
Q8N2C7	Protein unc-80 homolog OS=Homo sapiens GN=UNC80 PE=2 SV=2 - [UNC80_HUMAN]	1.25	1.38	1.20	1.33					1.3	0.00
P51784	Ubiquitin carboxyl-terminal hydrolase 11 OS=Homo sapiens GN=USP11 PE=1 SV=3 - [UBP11_HUMAN]	0.01	-0.21	0.17	-0.04	1.97	2.59	2.51	3.13	1.3	0.04
Q9NYQ8	Protocadherin Fat 2 OS=Homo sapiens GN=FAT2 PE=1 SV=2 - [FAT2_HUMAN]	0.53	0.56	0.47	0.50	2.09	1.96	2.08	1.95	1.3	0.00
Q96BM9	ADP-ribosylation factor-like protein 8A OS=Homo sapiens GN=ARL8A PE=1 SV=1 - [ARL8A_HUMAN]					1.46	1.07	1.41	1.03	1.2	0.00
P52566	Rho GDP-dissociation inhibitor 2 OS=Homo sapiens GN=ARHGDIB PE=1 SV=3 - [GDIR2_HUMAN]					1.40	1.14	1.32	1.08	1.2	0.00

Q9HCM1	Uncharacterized protein C12orf35 OS=Homo sapiens GN=C12orf35 PE=1 SV=3 - [CL035_HUMAN]					1.58	1.45	1.01	0.80	1.2	0.01
P15170	Eukaryotic peptide chain release factor GTP-binding subunit ERF3A OS=Homo sapiens GN=GSPT1 PE=1 SV=1 - [ERF3A_HUMAN]					1.18	1.18	1.18	1.18	1.2	0.00
Q07654	Trefoil factor 3 OS=Homo sapiens GN=TFF3 PE=1 SV=1 - [TFF3_HUMAN]					1.13	1.26	1.06	1.19	1.2	0.00
P46379	Large proline-rich protein BAG6 OS=Homo sapiens GN=BAG6 PE=1 SV=2 - [BAG6_HUMAN]	1.14	1.32	1.16	1.35	0.70	0.77	1.36	1.42	1.2	0.00
P80511	Protein S100-A12 OS=Homo sapiens GN=S100A12 PE=1 SV=2 - [S10AC_HUMAN]					1.22	1.22	1.09	1.05	1.1	0.00
Q9HBJ7	Ubiquitin carboxyl-terminal hydrolase 29 OS=Homo sapiens GN=USP29 PE=2 SV=1 - [UBP29_HUMAN]	1.09	1.07	1.20	1.18					1.1	0.00

Q16557	Pregnancy-specific beta-1-glycoprotein 3 OS=Homo sapiens GN=PSG3 PE=2 SV=2 - [PSG3_HUMAN]	2.13	2.40	2.34	2.63	0.28	-0.12	-0.09	-0.50	1.1	0.05
Q9BV23	Monoacylglycerol lipase ABHD6 OS=Homo sapiens GN=ABHD6 PE=2 SV=1 - [ABHD6_HUMAN]	0.28	0.48	0.41	0.61	1.87	2.06	1.59	1.77	1.1	0.00
Q0ZGT2	Nexilin OS=Homo sapiens GN=NEXN PE=1 SV=1 - [NEXN_HUMAN]					1.16	1.12	1.12	1.01	1.1	0.00
A6NMZ7	Collagen alpha-6(VI) chain OS=Homo sapiens GN=COL6A6 PE=1 SV=2 - [CO6A6_HUMAN]	-0.08	0.18	0.03	0.31	0.65	2.47	1.61	3.42	1.1	0.05
O43790	Keratin, type II cuticular Hb6 OS=Homo sapiens GN=KRT86 PE=1 SV=1 - [KRT86_HUMAN]	1.05	0.82	0.94	0.72	1.81	1.72	0.75	0.65	1.1	0.00
Q5M775	Cytospin-B OS=Homo sapiens GN=SPECC1 PE=1 SV=1 - [CYTSB_HUMAN]					1.26	0.98	1.12	0.85	1.1	0.00
P13727	Bone marrow proteoglycan OS=Homo sapiens GN=PRG2 PE=1 SV=2 - [PRG2_HUMAN]	1.20	1.32	1.21	1.35	0.59	0.91	0.73	0.99	1.0	0.00

Q8IV33	Uncharacterized protein KIAA0825 OS=Homo sapiens GN=KIAA0825 PE=2 SV=2 - [K0825_HUMAN]	1.99	1.86	1.97	1.84	0.34	0.17	0.03	0.08	1.0	0.02
Q92973	Transportin-1 OS=Homo sapiens GN=TNPO1 PE=1 SV=2 - [TNPO1_HUMAN]	0.95	1.10	0.91	1.06					1.0	0.00
P62328	Thymosin beta-4 OS=Homo sapiens GN=TMSB4X PE=1 SV=2 - [TYB4_HUMAN]					1.02	0.99	0.95	0.96	1.0	0.00
Q9UPQ9	Trinucleotide repeat-containing gene 6B protein OS=Homo sapiens GN=TNRC6B PE=1 SV=4 - [TNR6B_HUMAN]	0.45	0.49	0.46	0.51	0.85	1.67	1.26	2.09	1.0	0.00
Q5SQ80	Ankyrin repeat domain-containing protein 20A2 OS=Homo sapiens GN=ANKRD20A2 PE=2 SV=1 - [A20A2_HUMAN]					1.11	0.95	0.93	0.77	0.9	0.00
Q7RTS9	Dymeclin OS=Homo sapiens GN=DYM PE=1 SV=1 - [DYM_HUMAN]	0.81	1.02	0.86	1.07					0.9	0.00
A5D8W1	Uncharacterized protein C7orf63 OS=Homo sapiens GN=C7orf63 PE=2 SV=3 - [CG063_HUMAN]					1.00	1.29	0.59	0.87	0.9	0.01

Q15149	Plectin OS=Homo sapiens GN=PLEC PE=1 SV=3 - [PLEC_HUMAN]	0.89	0.86	0.72	0.69	1.21	0.88	1.20	0.87	0.9	0.00
Q13085	Acetyl-CoA carboxylase 1 OS=Homo sapiens GN=ACACA PE=1 SV=2 - [ACACA_HUMAN]	0.09	-0.14	0.05	-0.15	1.99	1.71	2.00	1.69	0.9	0.04
Q8WXA3	RUN and FYVE domain containing protein 2 OS=Homo sapiens GN=RUFY2 PE=1 SV=2 - [RUFY2_HUMAN]	0.32	0.50	0.28	0.47	1.38	1.51	1.28	1.41	0.9	0.00
P04085	Platelet-derived growth factor subunit A OS=Homo sapiens GN=PDGFA PE=1 SV=1 - [PDGFA_HUMAN]					0.91	0.87	0.92	0.88	0.9	0.00
Q9UPY3	Endoribonuclease Dicer OS=Homo sapiens GN=DICER1 PE=1 SV=3 - [DICER_HUMAN]	0.72	0.76	1.01	1.05					0.9	0.00
P51692	Signal transducer and activator of transcription 5B OS=Homo sapiens GN=STAT5B PE=1 SV=2 - [STA5B_HUMAN]					0.98	1.04	0.72	0.78	0.9	0.00

Q4LDE5	Sushi, von Willebrand factor type A, EGF and pentraxin domain-containing protein 1 OS=Homo sapiens GN=SVEP1 PE=1 SV=3 - [SVEP1_HUMAN]	0.75	0.72	0.69	0.68	0.84	1.14	0.89	1.18	0.9	0.00
Q5VST9	Obscurin OS=Homo sapiens GN=OBSCN PE=1 SV=3 - [OBSCN_HUMAN]	0.33	0.91	0.47	1.06	1.10	1.09	0.87	0.86	0.8	0.00
O00462	Beta-mannosidase OS=Homo sapiens GN=MANBA PE=1 SV=3 - [MANBA_HUMAN]	0.54	0.63	0.62	0.72	1.04	1.07	1.00	1.03	0.8	0.00
Q05682	Caldesmon OS=Homo sapiens GN=CALD1 PE=1 SV=3 - [CALD1_HUMAN]					0.85	0.79	0.85	0.81	0.8	0.00
Q6ZU35	Uncharacterized protein KIAA1211 OS=Homo sapiens GN=KIAA1211 PE=1 SV=3 - [K1211_HUMAN]					0.82	0.89	0.75	0.82	0.8	0.00
P29973	cGMP-gated cation channel alpha-1 OS=Homo sapiens GN=CNGA1 PE=1 SV=3 - [CNGA1_HUMAN]	0.71	0.79	0.83	0.86					0.8	0.00
P11678	Eosinophil peroxidase OS=Homo sapiens GN=EPX PE=1 SV=2 - [PERE_HUMAN]	0.75	0.88	0.71	0.82					0.8	0.00

Q9UBC2	Epidermal growth factor receptor substrate 15-like 1 OS=Homo sapiens GN=EPS15L1 PE=1 SV=1 - [EP15R_HUMAN]	0.89	0.64	0.24	0.00	1.30	1.16	1.09	0.95	0.8	0.00
P20742	Pregnancy zone protein OS=Homo sapiens GN=PZP PE=1 SV=4 - [PZP_HUMAN]	0.78	0.74	0.76	0.77	0.82	0.88	0.65	0.78	0.8	0.00
P05787	Keratin, type II cytoskeletal 8 OS=Homo sapiens GN=KRT8 PE=1 SV=7 - [K2C8_HUMAN]					0.93	0.47	1.05	0.59	0.8	0.01
Q9H7C9	UPF0366 protein C11orf67 OS=Homo sapiens GN=C11orf67 PE=1 SV=1 - [CK067_HUMAN]					0.69	0.81	0.68	0.80	0.7	0.00
Q5SZK8	FRAS1-related extracellular matrix protein 2 OS=Homo sapiens GN=FREM2 PE=1 SV=2 - [FREM2_HUMAN]	0.75	0.50	0.98	0.74					0.7	0.00
Q9C0G6	Dynein heavy chain 6, axonemal OS=Homo sapiens GN=DNAH6 PE=1 SV=3 - [DYH6_HUMAN]	1.25	1.19	1.31	1.27	0.31	0.24	0.20	0.13	0.7	0.01
Q6ZMZ3	Nesprin-3 OS=Homo sapiens GN=C14orf49 PE=1 SV=2 - [SYNE3_HUMAN]					0.86	0.72	0.74	0.61	0.7	0.00

O75962	Triple functional domain protein OS=Homo sapiens GN=TRIO PE=1 SV=2 - [TRIO_HUMAN]	0.56	0.75	0.72	0.91					0.7	0.00
O95274	Ly6/PLAUR domain-containing protein 3 OS=Homo sapiens GN=LYPD3 PE=1 SV=2 - [LYPD3_HUMAN]	0.78	0.65	0.79	0.67					0.7	0.00
Q86UQ4	ATP-binding cassette sub-family A member 13 OS=Homo sapiens GN=ABCA13 PE=2 SV=3 - [ABCAD_HUMAN]					0.60	1.02	0.42	0.83	0.7	0.01
Q9Y287	Integral membrane protein 2B OS=Homo sapiens GN=ITM2B PE=1 SV=1 - [ITM2B_HUMAN]					0.89	0.70	0.72	0.53	0.7	0.00
Q13099	Intraflagellar transport protein 88 homolog OS=Homo sapiens GN=IFT88 PE=2 SV=2 - [IFT88_HUMAN]	0.67	0.70	0.71	0.75					0.7	0.00
Q9HCC9	Lateral signaling target protein 2 homolog OS=Homo sapiens GN=ZFYVE28 PE=1 SV=3 - [LST2_HUMAN]					0.68		0.74		0.7	0.03

Q13360	Zinc finger protein 177 OS=Homo sapiens GN=ZNF177 PE=2 SV=3 - [ZN177_HUMAN]					1.02	0.46	0.95	0.39	0.7	0.02
Q7Z3S7	Voltage-dependent calcium channel subunit alpha-2/delta-4 OS=Homo sapiens GN=CACNA2D4 PE=1 SV=2 - [CA2D4_HUMAN]	0.87	0.60	0.81	0.54					0.7	0.00
P06753	Tropomyosin alpha-3 chain OS=Homo sapiens GN=TPM3 PE=1 SV=1 - [TPM3_HUMAN]	0.25	0.00	0.80	0.56	1.03	1.09	0.91	0.97	0.7	0.00
Q8IYT3	Coiled-coil domain-containing protein C6orf97 OS=Homo sapiens GN=C6orf97 PE=2 SV=3 - [CF097_HUMAN]	0.43	0.49	0.56	0.62	0.83	1.07	0.68	0.92	0.7	0.00
Q12840	Kinesin heavy chain isoform 5A OS=Homo sapiens GN=KIF5A PE=1 SV=2 - [KIF5A_HUMAN]					0.78	0.75	0.65	0.61	0.7	0.00
Q07092	Collagen alpha-1(XVI) chain OS=Homo sapiens GN=COL16A1 PE=1 SV=2 - [COGA1_HUMAN]					1.01	0.86	0.52	0.37	0.7	0.02

Q13111	Chromatin assembly factor 1 subunit A OS=Homo sapiens GN=CHAF1A PE=1 SV=2 - [CAF1A_HUMAN]	0.57	0.82	0.55	0.80					0.7	0.00
P04278	Sex hormone-binding globulin OS=Homo sapiens GN=SHBG PE=1 SV=2 - [SHBG_HUMAN]	0.87	0.92	0.78	0.85	0.47	0.59	0.44	0.56	0.7	0.00
Q9NZN5	Rho guanine nucleotide exchange factor 12 OS=Homo sapiens GN=ARHGEF12 PE=1 SV=1 - [ARHGC_HUMAN]	0.36	0.86	0.50	1.01	0.51	0.91	0.43	0.83	0.7	0.00
Q05469	Hormone-sensitive lipase OS=Homo sapiens GN=LIPE PE=1 SV=4 - [LIPS_HUMAN]					0.73	0.59	0.73	0.59	0.7	0.00
Q9UHN1	DNA polymerase subunit gamma-2, mitochondrial OS=Homo sapiens GN=POLG2 PE=1 SV=1 - [DPOG2_HUMAN]					0.73	0.62	0.69	0.58	0.7	0.00
Q9BY66	Lysine-specific demethylase 5D OS=Homo sapiens GN=KDM5D PE=1 SV=2 - [KDM5D_HUMAN]	0.57	0.68	0.61	0.72					0.6	0.00

Q2M1V0	Intestine-specific homeobox OS=Homo sapiens GN=ISX PE=2 SV=2 - [ISX_HUMAN]	0.06	0.22	0.01	0.19	1.00	1.27	1.06	1.32	0.6	0.02
Q96PL1	Secretoglobin family 3A member 2 OS=Homo sapiens GN=SCGB3A2 PE=1 SV=1 - [SG3A2_HUMAN]	0.61	0.72	0.55	0.66					0.6	0.00
P09960	Leukotriene A-4 hydrolase OS=Homo sapiens GN=LTA4H PE=1 SV=2 - [LKHA4_HUMAN]	0.65	0.64	0.62	0.61					0.6	0.00
Q13724	Mannosyl-oligosaccharide glucosidase OS=Homo sapiens GN=MOGS PE=1 SV=5 - [MOGS_HUMAN]	0.17	0.16	0.25	0.24	1.08	1.14	0.96	1.02	0.6	0.01
P55056	Apolipoprotein C-IV OS=Homo sapiens GN=APOC4 PE=1 SV=1 - [APOC4_HUMAN]	0.14	0.16	0.26	0.29	1.11	1.08	0.92	1.02	0.6	0.01
Q14008	Cytoskeleton-associated protein 5 OS=Homo sapiens GN=CKAP5 PE=1 SV=3 - [CKAP5_HUMAN]	-0.11	0.26	-0.37	0.02	1.20	1.60	1.02	1.36	0.6	0.05
P02730	Band 3 anion transport protein OS=Homo sapiens GN=SLC4A1 PE=1 SV=3 - [B3AT_HUMAN]	0.67	0.73	0.48	0.58					0.6	0.00

Q7RTZ1	Ovochymase-2 OS=Homo sapiens GN=OVCH2 PE=1 SV=2 - [OVCH2_HUMAN]	1.73	1.08	0.36	-0.10	0.23	0.52	0.37	0.73	0.6	0.02
P12724	Eosinophil cationic protein OS=Homo sapiens GN=RNASE3 PE=1 SV=2 - [ECP_HUMAN]					0.67	0.58	0.63	0.55	0.6	0.00
Q00534	Cyclin-dependent kinase 6 OS=Homo sapiens GN=CDK6 PE=1 SV=1 - [CDK6_HUMAN]					0.80	0.70	0.51	0.41	0.6	0.01
Q92545	Transmembrane protein 131 OS=Homo sapiens GN=TMEM131 PE=1 SV=3 - [TM131_HUMAN]	0.50	0.49	0.71	0.71					0.6	0.00
P17482	Homeobox protein Hox-B9 OS=Homo sapiens GN=HOXB9 PE=1 SV=2 - [HXB9_HUMAN]	0.66	0.88	0.78	1.02	0.48	0.36	0.38	0.26	0.6	0.00
P42685	Tyrosine-protein kinase FRK OS=Homo sapiens GN=FRK PE=1 SV=1 - [FRK_HUMAN]	0.60	0.54	0.70	0.66	0.63	0.47	0.64	0.47	0.6	0.00
O15360	Fanconi anemia group A protein OS=Homo sapiens GN=FANCA PE=1 SV=2 - [FANCA_HUMAN]	0.30	0.67	0.33	0.71	0.78	0.59	0.76	0.57	0.6	0.00

Q9Y251	Heparanase OS=Homo sapiens GN=HPSE PE=1 SV=2 - [HPSE_HUMAN]	0.28	0.36	0.30	0.37	0.98	0.86	0.83	0.71	0.6	0.00
Q32MH5	Protein FAM214A OS=Homo sapiens GN=FAM214A PE=2 SV=2 - [F214A_HUMAN]	1.64	0.98	1.20	0.55	0.17	-0.01	0.11	0.06	0.6	0.03
Q9NR34	Mannosyl-oligosaccharide 1,2-alpha-mannosidase IC OS=Homo sapiens GN=MAN1C1 PE=1 SV=1 - [MA1C1_HUMAN]	0.78	0.81	0.69	0.72	0.29	0.68	0.14	0.53	0.6	0.00
Q7Z460	CLIP-associating protein 1 OS=Homo sapiens GN=CLASP1 PE=1 SV=1 - [CLAP1_HUMAN]	0.91	0.46	0.69	0.26					0.6	0.03
Q08ET2	Sialic acid-binding Ig-like lectin 14 OS=Homo sapiens GN=SIGLEC14 PE=1 SV=1 - [SIG14_HUMAN]	0.90	0.69	0.79	0.59	0.21	0.54	0.29	0.62	0.6	0.00
Q2M1K9	Zinc finger protein 423 OS=Homo sapiens GN=ZNF423 PE=1 SV=1 - [ZN423_HUMAN]	1.00	1.11	1.09	1.21	0.03	-0.01	0.09	0.04	0.6	0.03

Q6ZQN7	Solute carrier organic anion transporter family member 4C1 OS=Homo sapiens GN=SLCO4C1 PE=1 SV=1 - [SO4C1_HUMAN]	0.61	0.63	0.50	0.52					0.6	0.00
Q9UJ42	Probable G-protein coupled receptor 160 OS=Homo sapiens GN=GPR160 PE=2 SV=1 - [GP160_HUMAN]	-0.09	-0.02	-0.03	0.05	1.02	1.42	0.87	1.27	0.6	0.04
P05451	Lithostathine-1-alpha OS=Homo sapiens GN=REG1A PE=1 SV=3 - [REG1A_HUMAN]					0.67	0.60	0.53	0.45	0.6	0.00
Q9Y275	Tumor necrosis factor ligand superfamily member 13B OS=Homo sapiens GN=TNFSF13B PE=1 SV=1 - [TN13B_HUMAN]					0.57	0.61	0.52	0.54	0.6	0.00
O75410	Transforming acidic coiled-coil-containing protein 1 OS=Homo sapiens GN=TACC1 PE=1 SV=2 - [TACC1_HUMAN]					0.66	0.64	0.48	0.46	0.6	0.00

P01602	Ig kappa chain V-I region HK102 (Fragment) OS=Homo sapiens GN=IGKV1-5 PE=4 SV=1 - [KV110_HUMAN]	0.42	0.55	0.33	0.47	0.88	0.66	0.67	0.45	0.6	0.00
Q8TDW7	Protocadherin Fat 3 OS=Homo sapiens GN=FAT3 PE=2 SV=2 - [FAT3_HUMAN]	0.47	0.48	0.53	0.54	0.74	0.60	0.60	0.46	0.6	0.00
Q9BQG0	Myb-binding protein 1A OS=Homo sapiens GN=MYBBP1A PE=1 SV=2 - [MBB1A_HUMAN]	0.37	0.33	0.54	0.50	0.91	0.09	1.22	0.40	0.5	0.00
Q15643	Thyroid receptor-interacting protein 11 OS=Homo sapiens GN=TRIP11 PE=1 SV=3 - [TRIPB_HUMAN]	0.85	1.28	0.20	0.64	0.37	0.37	0.34	0.31	0.5	0.00
P01593	Ig kappa chain V-I region AG OS=Homo sapiens PE=1 SV=1 - [KV101_HUMAN]	0.88	0.63	0.62	0.37	0.47	0.61	0.32	0.46	0.5	0.00
P54840	Glycogen [starch] synthase, liver OS=Homo sapiens GN=GYS2 PE=1 SV=2 - [GYS2_HUMAN]	0.46	0.47	0.15	0.16	0.29	0.87	0.64	1.22	0.5	0.00
A6NHA9	Olfactory receptor 4C46 OS=Homo sapiens GN=OR4C46 PE=2 SV=1 - [O4C46_HUMAN]	0.69	0.87	0.90	1.10	0.09	0.39	-0.06	0.24	0.5	0.01

P26885	Peptidyl-prolyl cis-trans isomerase FKBP2 OS=Homo sapiens GN=FKBP2 PE=1 SV=2 - [FKBP2_HUMAN]					0.58	0.64	0.47	0.43	0.5	0.00
A6NC98	Coiled-coil domain-containing protein 88B OS=Homo sapiens GN=CCDC88B PE=1 SV=1 - [CC88B_HUMAN]	0.33	0.52	0.53	0.72					0.5	0.01
Q6NSJ2	Pleckstrin homology-like domain family B member 3 OS=Homo sapiens GN=PHLDB3 PE=2 SV=3 - [PHLB3_HUMAN]	0.47	0.52	0.52	0.57					0.5	0.00
A0M8Q6	Ig lambda-7 chain C region OS=Homo sapiens GN=IGLC7 PE=1 SV=2 - [LAC7_HUMAN]	0.42	0.40	0.39	0.39	0.67	0.80	0.45	0.66	0.5	0.00
P68431	Histone H3.1 OS=Homo sapiens GN=HIST1H3A PE=1 SV=2 - [H31_HUMAN]					0.53	0.55	0.48	0.49	0.5	0.00
Q86VQ0	Lebercilin OS=Homo sapiens GN=LCA5 PE=1 SV=2 - [LCA5_HUMAN]	0.48	0.43	0.58	0.53	0.79	0.38	0.64	0.23	0.5	0.00

Q9C0F0	Putative Polycomb group protein ASXL3 OS=Homo sapiens GN=ASXL3 PE=2 SV=3 - [ASXL3_HUMAN]					0.66	0.54	0.46	0.36	0.5	0.00
P35625	Metalloproteinase inhibitor 3 OS=Homo sapiens GN=TIMP3 PE=1 SV=2 - [TIMP3_HUMAN]					0.52	0.52	0.48	0.49	0.5	0.00
Q99550	M-phase phosphoprotein 9 OS=Homo sapiens GN=MPHOSPH9 PE=1 SV=3 - [MPP9_HUMAN]	0.62	0.26	0.62	0.50					0.5	0.01
O15231	Zinc finger protein 185 OS=Homo sapiens GN=ZNF185 PE=1 SV=3 - [ZN185_HUMAN]					0.45	0.58	0.42	0.55	0.5	0.00
Q9NR56	Muscleblind-like protein 1 OS=Homo sapiens GN=MBNL1 PE=1 SV=2 - [MBNL1_HUMAN]					0.64	0.56	0.44	0.36	0.5	0.00
O15117	FYN-binding protein OS=Homo sapiens GN=FYB PE=1 SV=2 - [FYB_HUMAN]	0.68	0.77	0.68	0.78	0.37	0.35	0.19	0.17	0.5	0.00
Q7L9B9	Endonuclease/exonuclease/phosphatase family domain-containing protein 1 OS=Homo sapiens GN=EEPD1 PE=1 SV=2 - [EEPD1_HUMAN]					0.62	0.47	0.53	0.37	0.5	0.00

Q9NU22	Midasin OS=Homo sapiens GN=MDN1 PE=1 SV=2 - [MDN1_HUMAN]	0.50	0.70	0.48	0.68	0.74	-0.15	0.95	0.06	0.5	0.01
Q96GW7	Brevican core protein OS=Homo sapiens GN=BCAN PE=1 SV=2 - [PGCB_HUMAN]					0.68	0.47	0.52	0.30	0.5	0.01
Q8NEZ4	Histone-lysine N-methyltransferase MLL3 OS=Homo sapiens GN=MLL3 PE=1 SV=3 - [MLL3_HUMAN]					0.60	0.51	0.47	0.39	0.5	0.00
Q15746	Myosin light chain kinase, smooth muscle OS=Homo sapiens GN=MYLK PE=1 SV=4 - [MYLK_HUMAN]					0.50	0.56	0.41	0.47	0.5	0.00
P07108	Acyl-CoA-binding protein OS=Homo sapiens GN=DBI PE=1 SV=2 - [ACBP_HUMAN]					0.59	0.48	0.47	0.40	0.5	0.00
Q9UDY2	Tight junction protein ZO-2 OS=Homo sapiens GN=TJP2 PE=1 SV=2 - [ZO2_HUMAN]					0.53	0.53	0.44	0.44	0.5	0.00
Q13477	Mucosal addressin cell adhesion molecule 1 OS=Homo sapiens GN=MADCAM1 PE=1 SV=2 - [MADCA_HUMAN]	0.50	0.41	0.49	0.39	0.46	0.59	0.44	0.58	0.5	0.00

P21741	Midkine OS=Homo sapiens GN=MDK PE=1 SV=1 - [MK_HUMAN]	0.37	0.45	0.09	0.19	0.80	0.66	0.72	0.58	0.5	0.00
P01833	Polymeric immunoglobulin receptor OS=Homo sapiens GN=PIGR PE=1 SV=4 - [PIGR_HUMAN]	0.59	0.58	0.56	0.61	0.77	0.42	0.37	-0.06	0.5	0.00
Q99567	Nuclear pore complex protein Nup88 OS=Homo sapiens GN=NUP88 PE=1 SV=2 - [NUP88_HUMAN]	0.18	0.17	0.58	0.57	0.68	0.74	0.42	0.48	0.5	0.00
P32004	Neural cell adhesion molecule L1 OS=Homo sapiens GN=L1CAM PE=1 SV=2 - [L1CAM_HUMAN]	0.82	0.59	0.32	0.18					0.5	0.04
Q9NQT8	Kinesin-like protein KIF13B OS=Homo sapiens GN=KIF13B PE=1 SV=1 - [KI13B_HUMAN]					0.41	0.51	0.44	0.54	0.5	0.00
P14868	Aspartate--tRNA ligase, cytoplasmic OS=Homo sapiens GN=DARS PE=1 SV=2 - [SYDC_HUMAN]	0.53	0.50	0.50	0.47	0.38	0.48	0.40	0.50	0.5	0.00
O43639	Cytoplasmic protein NCK2 OS=Homo sapiens GN=NCK2 PE=1 SV=2 - [NCK2_HUMAN]					0.58	0.45	0.48	0.34	0.5	0.00

Q96F63	Coiled-coil domain-containing protein 97 OS=Homo sapiens GN=CCDC97 PE=1 SV=1 - [CCD97_HUMAN]					0.55	0.53	0.40	0.37	0.5	0.00
Q9H4A3	Serine/threonine-protein kinase WNK1 OS=Homo sapiens GN=WNK1 PE=1 SV=2 - [WNK1_HUMAN]					0.87	0.21	0.48	0.29	0.5	0.05
O75127	Pentatricopeptide repeat-containing protein 1 OS=Homo sapiens GN=PTCD1 PE=1 SV=2 - [PTCD1_HUMAN]	0.42	0.66	0.25	0.50					0.5	0.01
Q8WTQ4	Uncharacterized protein C16orf78 OS=Homo sapiens GN=C16orf78 PE=2 SV=1 - [CP078_HUMAN]	0.46	0.49	0.42	0.47					0.5	0.00
O43516	WAS/WASL-interacting protein family member 1 OS=Homo sapiens GN=WIPF1 PE=1 SV=3 - [WIPF1_HUMAN]					0.58	0.51	0.41	0.33	0.5	0.00

O60264	SWI/SNF-related matrix-associated actin-dependent regulator of chromatin subfamily A member 5 OS=Homo sapiens GN=SMARCA5 PE=1 SV=1 - [SMCA5_HUMAN]	0.49	0.22	0.69	0.44					0.5	0.02
P30046	D-dopachrome decarboxylase OS=Homo sapiens GN=DDT PE=1 SV=3 - [DOPD_HUMAN]					0.71	0.66	0.25	0.20	0.5	0.04
P01710	Ig lambda chain V-II region BO OS=Homo sapiens PE=1 SV=1 - [LV207_HUMAN]	0.36	0.24	0.27	0.16	0.58	0.65	0.71	0.65	0.5	0.00
P21810	Biglycan OS=Homo sapiens GN=BGN PE=1 SV=2 - [PGS1_HUMAN]	0.24	0.16	0.17	0.09	0.80	0.71	0.77	0.68	0.5	0.00
P02741	C-reactive protein OS=Homo sapiens GN=CRP PE=1 SV=1 - [CRP_HUMAN]	0.32	0.47	0.35	0.61	0.39	0.51	0.42	0.54	0.5	0.00
Q16181	Septin-7 OS=Homo sapiens GN=SEPT7 PE=1 SV=2 - [SEPT7_HUMAN]	0.25	0.57	0.06	0.40	0.59	0.61	0.54	0.56	0.4	0.00
P29692	Elongation factor 1-delta OS=Homo sapiens GN=EEF1D PE=1 SV=5 - [EF1D_HUMAN]					0.22	0.54	0.39	0.62	0.4	0.02

P16152	Carbonyl reductase [NADPH] 1 OS=Homo sapiens GN=CBR1 PE=1 SV=3 - [CBR1_HUMAN]	-0.02	-0.05	0.05	0.03	0.97	1.01	0.76	0.80	0.4	0.03
Q13822	Ectonucleotide pyrophosphatase/phosphodiesterase family member 2 OS=Homo sapiens GN=ENPP2 PE=1 SV=3 - [ENPP2_HUMAN]	0.68	0.99	0.45	0.78	0.31	0.17	0.00	0.17	0.4	0.01
Q08495	Dematin OS=Homo sapiens GN=EPB49 PE=1 SV=3 - [DEMA_HUMAN]					0.26	0.45	0.44	0.61	0.4	0.01
P54727	UV excision repair protein RAD23 homolog B OS=Homo sapiens GN=RAD23B PE=1 SV=1 - [RD23B_HUMAN]					0.54	0.43	0.44	0.36	0.4	0.00
Q05315	Eosinophil lysophospholipase OS=Homo sapiens GN=CLC PE=1 SV=3 - [LPPL_HUMAN]					0.38	0.58	0.29	0.50	0.4	0.01
Q05209	Tyrosine-protein phosphatase non-receptor type 12 OS=Homo sapiens GN=PTPN12 PE=1 SV=3 - [PTN12_HUMAN]	0.58	0.69	0.53	0.66	0.19	0.30	0.21	0.35	0.4	0.00

Q14247	Src substrate cortactin OS=Homo sapiens GN=CTTN PE=1 SV=2 - [SRC8_HUMAN]					0.67	0.30	0.49	0.29	0.4	0.02
Q14050	Collagen alpha-3(IX) chain OS=Homo sapiens GN=COL9A3 PE=2 SV=2 - [CO9A3_HUMAN]	0.30	0.43	0.44	0.58					0.4	0.00
P12081	Histidine--tRNA ligase, cytoplasmic OS=Homo sapiens GN=HARS PE=1 SV=2 - [SYHC_HUMAN]	0.49	0.30	0.56	0.38					0.4	0.00
Q15828	Cystatin-M OS=Homo sapiens GN=CST6 PE=1 SV=1 - [CYTM_HUMAN]					0.45	0.61	0.26	0.42	0.4	0.01
Q9UBR2	Cathepsin Z OS=Homo sapiens GN=CTSZ PE=1 SV=1 - [CATZ_HUMAN]					0.43	0.55	0.31	0.43	0.4	0.00
Q9UKY7	Protein CDV3 homolog OS=Homo sapiens GN=CDV3 PE=1 SV=1 - [CDV3_HUMAN]					0.19	0.60	0.43	0.49	0.4	0.02
Q86VF7	Nebulin-related-anchoring protein OS=Homo sapiens GN=NRAP PE=2 SV=2 - [NRAP_HUMAN]					0.61	0.48	0.37	0.24	0.4	0.01
P01742	Ig heavy chain V-I region EU OS=Homo sapiens PE=1 SV=1 - [HV101_HUMAN]	1.04	0.64	0.33	0.05	0.42	0.35	0.28	0.29	0.4	0.00

Q9BW91	ADP-ribose pyrophosphatase, mitochondrial OS=Homo sapiens GN=NUDT9 PE=1 SV=1 - [NUDT9_HUMAN]	0.20	0.43	0.40	0.66					0.4	0.02
Q8WWQ8	Stabilin-2 OS=Homo sapiens GN=STAB2 PE=1 SV=3 - [STAB2_HUMAN]	0.24	0.21	0.37	0.36	0.39	0.38	0.71	0.70	0.4	0.00
Q9ULK5	Vang-like protein 2 OS=Homo sapiens GN=VANGL2 PE=1 SV=2 - [VANG2_HUMAN]					0.49	0.38	0.46	0.34	0.4	0.00
Q9H7P9	Pleckstrin homology domain-containing family G member 2 OS=Homo sapiens GN=PLEKHG2 PE=1 SV=3 - [PKHG2_HUMAN]	0.55	0.41	0.40	0.29					0.4	0.00
P05019	Insulin-like growth factor I OS=Homo sapiens GN=IGF1 PE=1 SV=1 - [IGF1_HUMAN]					0.48	0.42	0.35	0.38	0.4	0.00
P14780	Matrix metalloproteinase-9 OS=Homo sapiens GN=MMP9 PE=1 SV=3 - [MMP9_HUMAN]	0.36	0.39	0.42	0.45	0.40	0.41	0.39	0.44	0.4	0.00
Q07890	Son of sevenless homolog 2 OS=Homo sapiens GN=SOS2 PE=1 SV=2 - [SOS2_HUMAN]	0.35	0.45	0.36	0.48					0.4	0.00

Q9UGM5	Fetuin-B OS=Homo sapiens GN=FETUB PE=1 SV=2 - [FETUB_HUMAN]	0.54	0.54	0.54	0.55	0.21	0.51	0.05	0.32	0.4	0.00
P57679	Ellis-van Creveld syndrome protein OS=Homo sapiens GN=EVC PE=1 SV=1 - [EVC_HUMAN]	0.15	0.56	0.24	0.67					0.4	0.05
P26045	Tyrosine-protein phosphatase non-receptor type 3 OS=Homo sapiens GN=PTPN3 PE=1 SV=2 - [PTN3_HUMAN]	-0.35	0.56	0.00	0.92	0.49	0.57	0.49	0.57	0.4	0.02
O43149	Zinc finger ZZ-type and EF-hand domain-containing protein 1 OS=Homo sapiens GN=ZZEF1 PE=1 SV=6 - [ZZEF1_HUMAN]					0.52	0.45	0.36	0.29	0.4	0.00
Q96P20	NACHT, LRR and PYD domains-containing protein 3 OS=Homo sapiens GN=NLRP3 PE=1 SV=3 - [NALP3_HUMAN]					0.62	0.40	0.40	0.19	0.4	0.02
Q9H095	IQ domain-containing protein G OS=Homo sapiens GN=IQCG PE=2 SV=1 - [IQCG_HUMAN]					0.44	0.45	0.35	0.36	0.4	0.00

P26641	Elongation factor 1-gamma OS=Homo sapiens GN=EEF1G PE=1 SV=3 - [EF1G_HUMAN]					0.55	0.30	0.49	0.24	0.4	0.01
P61978	Heterogeneous nuclear ribonucleoprotein K OS=Homo sapiens GN=HNRNPK PE=1 SV=1 - [HNRPK_HUMAN]					0.47	0.41	0.38	0.32	0.4	0.00
P10646	Tissue factor pathway inhibitor OS=Homo sapiens GN=TFPI PE=1 SV=1 - [TFPI1_HUMAN]	0.82	0.78	0.44	0.43	0.29	0.06	0.23	0.10	0.4	0.01
P30307	M-phase inducer phosphatase 3 OS=Homo sapiens GN=CDC25C PE=1 SV=2 - [MPIP3_HUMAN]	0.37	0.35	0.47	0.38					0.4	0.00
Q9Y467	Sal-like protein 2 OS=Homo sapiens GN=SALL2 PE=1 SV=4 - [SALL2_HUMAN]	0.24	0.52	0.27	0.54					0.4	0.02
O00187	Mannan-binding lectin serine protease 2 OS=Homo sapiens GN=MASP2 PE=1 SV=4 - [MASP2_HUMAN]	0.31	0.30	0.34	0.36	0.43	0.42	0.51	0.46	0.4	0.00

Q16270	Insulin-like growth factor-binding protein 7 OS=Homo sapiens GN=IGFBP7 PE=1 SV=1 - [IBP7_HUMAN]					0.56	0.41	0.37	0.22	0.4	0.01
Q12955	Ankyrin-3 OS=Homo sapiens GN=ANK3 PE=1 SV=3 - [ANK3_HUMAN]	0.46	0.40	0.25	0.20	0.49	0.50	0.39	0.40	0.4	0.00
Q8WUH2	Transforming growth factor-beta receptor-associated protein 1 OS=Homo sapiens GN=TGFBRAP1 PE=1 SV=1 - [TGFA1_HUMAN]	0.50	0.54	0.72	0.76	0.10	0.17	0.12	0.19	0.4	0.01
Q70EL4	Ubiquitin carboxyl-terminal hydrolase 43 OS=Homo sapiens GN=USP43 PE=1 SV=2 - [UBP43_HUMAN]					0.44	0.39	0.37	0.33	0.4	0.00
P05121	Plasminogen activator inhibitor 1 OS=Homo sapiens GN=SERPINE1 PE=1 SV=1 - [PAI1_HUMAN]	0.48	0.41	0.41	0.41	0.47	0.31	0.37	0.21	0.4	0.00
Q14696	LDLR chaperone MESD OS=Homo sapiens GN=MESDC2 PE=1 SV=2 - [MESD_HUMAN]					0.23	0.38	0.48	0.42	0.4	0.01

Q14624	Inter-alpha-trypsin inhibitor heavy chain H4 OS=Homo sapiens GN=ITIH4 PE=1 SV=4 - [ITIH4_HUMAN]	0.35	0.38	0.34	0.36	0.36	0.43	0.36	0.46	0.4	0.00
Q9UJL9	Zinc finger protein 643 OS=Homo sapiens GN=ZNF643 PE=2 SV=2 - [ZN643_HUMAN]					0.20	0.46	0.29	0.55	0.4	0.02
P54577	Tyrosine--tRNA ligase, cytoplasmic OS=Homo sapiens GN=YARS PE=1 SV=4 - [SYYC_HUMAN]	0.06	-0.03	-0.05	-0.07	0.63	0.92	0.58	0.95	0.4	0.05
Q9H7D0	Dedicator of cytokinesis protein 5 OS=Homo sapiens GN=DOCK5 PE=1 SV=3 - [DOCK5_HUMAN]	0.27	0.34	0.40	0.48					0.4	0.00
Q9P241	Probable phospholipid-transporting ATPase VD OS=Homo sapiens GN=ATP10D PE=2 SV=3 - [AT10D_HUMAN]	0.52	0.19	0.55	0.22					0.4	0.03
Q5T0Z8	Uncharacterized protein C6orf132 OS=Homo sapiens GN=C6orf132 PE=1 SV=4 - [CF132_HUMAN]	0.41	0.54	0.18	0.33					0.4	0.02

P30876	DNA-directed RNA polymerase II subunit RPB2 OS=Homo sapiens GN=POLR2B PE=1 SV=1 - [RPB2_HUMAN]					0.40	0.38	0.35	0.33	0.4	0.00
P61916	Epididymal secretory protein E1 OS=Homo sapiens GN=NPC2 PE=1 SV=1 - [NPC2_HUMAN]					0.41	0.47	0.26	0.32	0.4	0.00
Q8IV38	Ankyrin repeat and MYND domain-containing protein 2 OS=Homo sapiens GN=ANKMY2 PE=1 SV=1 - [ANKY2_HUMAN]	0.41	0.34	0.29	0.40					0.4	0.00
P01605	Ig kappa chain V-I region Lay OS=Homo sapiens PE=1 SV=1 - [KV113_HUMAN]	0.17	0.23	0.22	0.25	0.44	0.53	0.47	0.54	0.4	0.00
Q9NR48	Histone-lysine N-methyltransferase ASH1L OS=Homo sapiens GN=ASH1L PE=1 SV=2 - [ASH1L_HUMAN]	0.15	0.24	0.42	0.61					0.4	0.04
P21291	Cysteine and glycine-rich protein 1 OS=Homo sapiens GN=CSRP1 PE=1 SV=3 - [CSRP1_HUMAN]					0.34	0.49	0.21	0.36	0.4	0.01

Q5GH76	XK-related protein 4 OS=Homo sapiens GN=XKR4 PE=1 SV=1 - [XKR4_HUMAN]	0.06	0.30	0.37	0.60	0.66	0.41	0.33	0.08	0.4	0.00
O75594	Peptidoglycan recognition protein 1 OS=Homo sapiens GN=PGLYRP1 PE=1 SV=1 - [PGRP1_HUMAN]	0.30	0.29	0.45	0.46	0.25	0.37	0.29	0.38	0.3	0.00
Q7Z591	AT-hook-containing transcription factor OS=Homo sapiens GN=AKNA PE=1 SV=2 - [AKNA_HUMAN]	0.37	0.33	0.37	0.32					0.3	0.00
P16333	Cytoplasmic protein NCK1 OS=Homo sapiens GN=NCK1 PE=1 SV=1 - [NCK1_HUMAN]					0.33	0.35	0.34	0.37	0.3	0.00
Q9BWD1	Acetyl-CoA acetyltransferase, cytosolic OS=Homo sapiens GN=ACAT2 PE=1 SV=2 - [THIC_HUMAN]	0.28	0.35	0.34	0.43					0.3	0.00
Q2WGJ9	Fer-1-like protein 6 OS=Homo sapiens GN=FER1L6 PE=2 SV=2 - [FR1L6_HUMAN]					0.35	0.55	0.14	0.35	0.3	0.02

O95202	LETM1 and EF-hand domain-containing protein 1, mitochondrial OS=Homo sapiens GN=LETM1 PE=1 SV=1 - [LETM1_HUMAN]	0.39	0.54	0.16	0.29					0.3	0.02
P24666	Low molecular weight phosphotyrosine protein phosphatase OS=Homo sapiens GN=ACP1 PE=1 SV=3 - [PPAC_HUMAN]					0.35	0.55	0.14	0.34	0.3	0.02
P01019	Angiotensinogen OS=Homo sapiens GN=AGT PE=1 SV=1 - [ANGT_HUMAN]	0.33	0.46	0.36	0.47	0.27	0.39	0.17	0.32	0.3	0.00
O14647	Chromodomain-helicase-DNA-binding protein 2 OS=Homo sapiens GN=CHD2 PE=1 SV=2 - [CHD2_HUMAN]	0.47	0.38	0.43	0.36	0.37	0.40	0.15	0.17	0.3	0.00
P07093	Glia-derived nexin OS=Homo sapiens GN=SERPINE2 PE=1 SV=1 - [GDN_HUMAN]	0.43	0.46	0.43	0.52	0.13	0.19	0.25	0.31	0.3	0.00
Q8WUA8	Tsukushin OS=Homo sapiens GN=TSKU PE=2 SV=3 - [TSK_HUMAN]	0.63	0.45	0.52	0.23	0.17	0.18	0.26	0.28	0.3	0.00

P12109	Collagen alpha-1(VI) chain OS=Homo sapiens GN=COL6A1 PE=1 SV=3 - [CO6A1_HUMAN]	0.49	0.37	0.30	0.19					0.3	0.01
P80108	Phosphatidylinositol-glycan-specific phospholipase D OS=Homo sapiens GN=GPLD1 PE=1 SV=3 - [PHLD_HUMAN]	0.28	0.32	0.35	0.34	0.36	0.46	0.25	0.35	0.3	0.00
P29317	Ephrin type-A receptor 2 OS=Homo sapiens GN=EPHA2 PE=1 SV=2 - [EPHA2_HUMAN]	0.00	-0.02	0.06	0.07	0.72	0.88	0.42	0.57	0.3	0.03
Q92783	Signal transducing adapter molecule 1 OS=Homo sapiens GN=STAM PE=1 SV=3 - [STAM1_HUMAN]					0.40	0.08	0.58	0.26	0.3	0.05
O75443	Alpha-tectorin OS=Homo sapiens GN=TECTA PE=1 SV=3 - [TECTA_HUMAN]	0.09	0.18	0.31	0.44	0.40	0.49	0.33	0.42	0.3	0.00
Q96ME1	F-box/LRR-repeat protein 18 OS=Homo sapiens GN=FBXL18 PE=2 SV=2 - [FXL18_HUMAN]					0.24	0.45	0.21	0.42	0.3	0.01
Q8N998	Coiled-coil domain-containing protein 89 OS=Homo sapiens GN=CCDC89 PE=2 SV=1 - [CCD89_HUMAN]	0.28	0.27	0.39	0.39					0.3	0.00

Q96KN2	Beta-Ala-His dipeptidase OS=Homo sapiens GN=CNDP1 PE=1 SV=4 - [CNDP1_HUMAN]	0.27	0.25	0.22	0.26	0.33	0.51	0.32	0.46	0.3	0.00
Q5JPE7	Nodal modulator 2 OS=Homo sapiens GN=NOMO2 PE=1 SV=1 - [NOMO2_HUMAN]	0.26	0.28	0.37	0.40					0.3	0.00
Q58F21	Bromodomain testis-specific protein OS=Homo sapiens GN=BRDT PE=1 SV=4 - [BRDT_HUMAN]	0.40	0.42	0.23	0.25					0.3	0.01
Q86SQ4	G-protein coupled receptor 126 OS=Homo sapiens GN=GPR126 PE=1 SV=3 - [GP126_HUMAN]	0.17	0.28	0.34	0.50					0.3	0.02
Q7L3T8	Probable proline--tRNA ligase, mitochondrial OS=Homo sapiens GN=PARS2 PE=1 SV=1 - [SYPM_HUMAN]					0.28	0.43	0.20	0.36	0.3	0.01
Q6IS24	Putative polypeptide N-acetylgalactosaminyltransferase-like protein 3 OS=Homo sapiens GN=WBSCR17 PE=2 SV=2 - [GLTL3_HUMAN]	0.14	0.40	0.23	0.50					0.3	0.03

P01033	Metalloproteinase inhibitor 1 OS=Homo sapiens GN=TIMP1 PE=1 SV=1 - [TIMP1_HUMAN]	0.30	0.29	0.23	0.24	0.28	0.45	0.27	0.47	0.3	0.00
Q14CM0	FERM and PDZ domain-containing protein 4 OS=Homo sapiens GN=FRMPD4 PE=1 SV=1 - [FRPD4_HUMAN]	0.26	0.38	0.25	0.37					0.3	0.00
Q6ZN06	Zinc finger protein 813 OS=Homo sapiens GN=ZNF813 PE=2 SV=2 - [ZN813_HUMAN]	0.41	0.53	0.09	0.22					0.3	0.05
Q7L2Z9	Centromere protein Q OS=Homo sapiens GN=CENPQ PE=1 SV=1 - [CENPQ_HUMAN]	0.19	0.25	0.00	0.08	0.42	0.52	0.46	0.55	0.3	0.00
P01344	Insulin-like growth factor II OS=Homo sapiens GN=IGF2 PE=1 SV=1 - [IGF2_HUMAN]	0.22	0.30	0.22	0.32	0.39	-0.03	0.58	0.45	0.3	0.00
Q6ZS30	Neurobeachin-like protein 1 OS=Homo sapiens GN=NBEAL1 PE=2 SV=3 - [NBEL1_HUMAN]					0.32	0.24	0.37	0.29	0.3	0.00
Q6S5L8	SHC-transforming protein 4 OS=Homo sapiens GN=SHC4 PE=1 SV=1 - [SHC4_HUMAN]	0.21	0.16	0.05	0.00	0.51	0.46	0.54	0.49	0.3	0.01

Q6IBS0	Twinfilin-2 OS=Homo sapiens GN=TWF2 PE=1 SV=2 - [TWF2_HUMAN]					0.29	0.30	0.27	0.35	0.3	0.00
Q9Y2R2	Tyrosine-protein phosphatase non-receptor type 22 OS=Homo sapiens GN=PTPN22 PE=1 SV=2 - [PTN22_HUMAN]	0.34	0.35	0.25	0.27					0.3	0.00
Q13740	CD166 antigen OS=Homo sapiens GN=ALCAM PE=1 SV=2 - [CD166_HUMAN]	0.35	0.28	0.30	0.32	0.32	0.15	0.43	0.26	0.3	0.00
Q14571	Inositol 1,4,5-trisphosphate receptor type 2 OS=Homo sapiens GN=ITPR2 PE=1 SV=2 - [ITPR2_HUMAN]	0.31	0.23	0.37	0.31					0.3	0.00
Q8N8K9	Uncharacterized protein KIAA1958 OS=Homo sapiens GN=KIAA1958 PE=1 SV=1 - [K1958_HUMAN]	0.07	0.27	0.33	0.53					0.3	0.05
P11279	Lysosome-associated membrane glycoprotein 1 OS=Homo sapiens GN=LAMP1 PE=1 SV=3 - [LAMP1_HUMAN]	0.24	0.28	0.31	0.37					0.3	0.00

Q3MJ16	Cytosolic phospholipase A2 epsilon OS=Homo sapiens GN=PLA2G4E PE=1 SV=3 - [PA24E_HUMAN]	0.37	0.14	0.45	0.22					0.3	0.02
P22894	Neutrophil collagenase OS=Homo sapiens GN=MMP8 PE=1 SV=1 - [MMP8_HUMAN]	0.27	0.35	0.39	0.45	0.14	0.39	0.05	0.31	0.3	0.00
P48729	Casein kinase I isoform alpha OS=Homo sapiens GN=CSNK1A1 PE=1 SV=2 - [KC1A_HUMAN]	0.59	0.24	0.61	0.28	0.33	0.04	0.27	-0.02	0.3	0.01
Q13439	Golgin subfamily A member 4 OS=Homo sapiens GN=GOLGA4 PE=1 SV=1 - [GOGA4_HUMAN]	0.54	0.48	0.45	0.34	0.15	0.24	0.00	0.15	0.3	0.00
O75112	LIM domain-binding protein 3 OS=Homo sapiens GN=LDB3 PE=1 SV=2 - [LDB3_HUMAN]	0.38	0.30	0.20	0.28					0.3	0.00
Q9ULT0	Tetratricopeptide repeat protein 7A OS=Homo sapiens GN=TTC7A PE=1 SV=3 - [TTC7A_HUMAN]	0.13	0.37	0.20	0.46					0.3	0.03
Q9Y5K6	CD2-associated protein OS=Homo sapiens GN=CD2AP PE=1 SV=1 - [CD2AP_HUMAN]					0.18	0.41	0.17	0.39	0.3	0.02

P05107	Integrin beta-2 OS=Homo sapiens GN=ITGB2 PE=1 SV=2 - [ITB2_HUMAN]	0.33	0.39	0.19	0.25					0.3	0.01
Q5VU43	Myomegalin OS=Homo sapiens GN=PDE4DIP PE=2 SV=1 - [MYOME_HUMAN]	0.42	0.21	0.36	0.16					0.3	0.02
Q9NW38	E3 ubiquitin-protein ligase FANCL OS=Homo sapiens GN=FANCL PE=1 SV=2 - [FANCL_HUMAN]	0.11	0.17	-0.18	-0.11	0.60	0.34	0.82	0.56	0.3	0.05
O96005	Cleft lip and palate transmembrane protein 1 OS=Homo sapiens GN=CLPTM1 PE=1 SV=1 - [CLPT1_HUMAN]	0.29	0.39	0.17	0.29					0.3	0.01
Q9BTY2	Plasma alpha-L-fucosidase OS=Homo sapiens GN=FUCA2 PE=1 SV=2 - [FUCO2_HUMAN]	0.38	0.23	0.34	0.20					0.3	0.01
P07333	Macrophage colony-stimulating factor 1 receptor OS=Homo sapiens GN=CSF1R PE=1 SV=2 - [CSF1R_HUMAN]	0.26	0.28	0.29	0.31					0.3	0.00
O15381	Nuclear valosin-containing protein-like OS=Homo sapiens GN=NVL PE=1 SV=1 - [NVL_HUMAN]	0.27	0.25	0.24	0.38					0.3	0.00

Q8TE73	Dynein heavy chain 5, axonemal OS=Homo sapiens GN=DNAH5 PE=1 SV=3 - [DYH5_HUMAN]	-0.16	0.40	0.16	0.73	0.23	0.28	0.31	0.35	0.3	0.01
A8K0R7	Zinc finger protein 839 OS=Homo sapiens GN=ZNF839 PE=2 SV=1 - [ZN839_HUMAN]	0.01	0.30	0.18	0.20	0.49	0.76	0.04	0.31	0.3	0.01
P37275	Zinc finger E-box-binding homeobox 1 OS=Homo sapiens GN=ZEB1 PE=1 SV=2 - [ZEB1_HUMAN]	0.13	0.55	-0.20	0.23	0.51	0.58	0.20	0.28	0.3	0.02
P80188	Neutrophil gelatinase-associated lipocalin OS=Homo sapiens GN=LCN2 PE=1 SV=2 - [NGAL_HUMAN]	0.04	0.06	0.20	0.21	0.41	0.49	0.40	0.46	0.3	0.00
P01773	Ig heavy chain V-III region BUR OS=Homo sapiens PE=1 SV=1 - [HV312_HUMAN]	0.19	0.51	0.10	0.42	0.21	0.30	0.24	0.31	0.3	0.00
P05164	Myeloperoxidase OS=Homo sapiens GN=MPO PE=1 SV=1 - [PERM_HUMAN]	0.08	0.06	0.03	0.00	0.51	0.53	0.51	0.56	0.3	0.02
Q9Y2G1	Myelin gene regulatory factor OS=Homo sapiens GN=MRF PE=1 SV=3 - [MRF_HUMAN]	0.30	0.19	0.37	0.27					0.3	0.01

P03973	Antileukoproteinase OS=Homo sapiens GN=SLPI PE=1 SV=2 - [SLPI_HUMAN]					0.23	0.22	0.34	0.33	0.3	0.00
Q02818	Nucleobindin-1 OS=Homo sapiens GN=NUCB1 PE=1 SV=4 - [NUCB1_HUMAN]					0.17	0.21	0.35	0.39	0.3	0.01
P40197	Platelet glycoprotein V OS=Homo sapiens GN=GP5 PE=1 SV=1 - [GPV_HUMAN]	0.29	0.25	0.26	0.23	0.31	0.22	0.35	0.33	0.3	0.00
Q5TIE3	von Willebrand factor A domain-containing protein 5B1 OS=Homo sapiens GN=VWA5B1 PE=1 SV=2 - [VW5B1_HUMAN]	0.12	-0.19	0.37	0.07	0.48	0.62	0.32	0.45	0.3	0.02
P09486	SPARC OS=Homo sapiens GN=SPARC PE=1 SV=1 - [SPRC_HUMAN]	0.49	0.41	0.40	0.37	0.16	0.14	0.15	0.12	0.3	0.00
Q13023	A-kinase anchor protein 6 OS=Homo sapiens GN=AKAP6 PE=1 SV=3 - [AKAP6_HUMAN]	0.48	0.73	0.12	0.38	0.13	0.14	0.13	0.13	0.3	0.01
Q14574	Desmocollin-3 OS=Homo sapiens GN=DSC3 PE=1 SV=3 - [DSC3_HUMAN]					0.12	0.44	0.32	0.23	0.3	0.03

P05997	Collagen alpha-2(V) chain OS=Homo sapiens GN=COL5A2 PE=1 SV=3 - [CO5A2_HUMAN]	0.32	0.08	0.48	0.23					0.3	0.05
Q9Y620	DNA repair and recombination protein RAD54B OS=Homo sapiens GN=RAD54B PE=1 SV=1 - [RA54B_HUMAN]	0.33	0.63	0.37	0.57	0.07	0.11	0.05	0.08	0.3	0.01
Q8IVF2	Protein AHNAK2 OS=Homo sapiens GN=AHNAK2 PE=1 SV=2 - [AHNK2_HUMAN]	0.47	0.34	0.06	-0.06	0.36	0.20	0.49	0.33	0.3	0.01
Q14019	Coactosin-like protein OS=Homo sapiens GN=COTL1 PE=1 SV=3 - [COTL1_HUMAN]	-0.04	0.13	-0.08	0.14	0.64	0.57	0.39	0.42	0.3	0.03
Q9NRY4	Rho GTPase-activating protein 35 OS=Homo sapiens GN=ARHGAP35 PE=1 SV=2 - [RHG35_HUMAN]	0.10	0.30	0.00	0.20	0.67	0.61	0.18	0.12	0.3	0.01
Q01459	Di-N-acetylchitobiase OS=Homo sapiens GN=CTBS PE=1 SV=1 - [DIAC_HUMAN]	0.48	0.34	0.54	0.36	0.14	0.20	-0.07	0.18	0.3	0.01

Q8IYE1	Coiled-coil domain-containing protein 13 OS=Homo sapiens GN=CCDC13 PE=2 SV=2 - [CCD13_HUMAN]					0.27	0.30	0.24	0.27	0.3	0.00
P05067	Amyloid beta A4 protein OS=Homo sapiens GN=APP PE=1 SV=3 - [A4_HUMAN]	0.36	0.42	0.35	0.43	0.17	0.09	0.24	0.08	0.3	0.00
Q8N6Q3	CD177 antigen OS=Homo sapiens GN=CD177 PE=1 SV=2 - [CD177_HUMAN]	0.44	0.46	0.51	0.55	-0.06	0.02	0.06	0.15	0.3	0.02
Q08554	Desmocollin-1 OS=Homo sapiens GN=DSC1 PE=1 SV=2 - [DSC1_HUMAN]	0.40	0.28	0.38	0.26	0.27	0.26	0.14	0.15	0.3	0.00
Q9P275	Ubiquitin carboxyl-terminal hydrolase 36 OS=Homo sapiens GN=USP36 PE=1 SV=3 - [UBP36_HUMAN]	0.23	0.23	0.30	0.30					0.3	0.00
P11215	Integrin alpha-M OS=Homo sapiens GN=ITGAM PE=1 SV=2 - [ITAM_HUMAN]	0.22	0.33	0.17	0.34					0.3	0.01
P20807	Calpain-3 OS=Homo sapiens GN=CAPN3 PE=1 SV=2 - [CAN3_HUMAN]	0.07	0.19	0.33	0.47					0.3	0.05
O00391	Sulfhydryl oxidase 1 OS=Homo sapiens GN=QSOX1 PE=1 SV=3 - [QSOX1_HUMAN]	0.18	0.19	0.11	0.20	0.36	0.44	0.24	0.40	0.3	0.00

Q92625	Ankyrin repeat and SAM domain-containing protein 1A OS=Homo sapiens GN=ANKS1A PE=1 SV=4 - [ANS1A_HUMAN]					0.15	0.28	0.27	0.36	0.3	0.01
P19823	Inter-alpha-trypsin inhibitor heavy chain H2 OS=Homo sapiens GN=ITIH2 PE=1 SV=2 - [ITIH2_HUMAN]	0.16	0.19	0.25	0.26	0.29	0.30	0.32	0.32	0.3	0.00
P08185	Corticosteroid-binding globulin OS=Homo sapiens GN=SERPINA6 PE=1 SV=1 - [CBG_HUMAN]	0.38	0.42	0.37	0.41	0.17	0.17	0.08	0.09	0.3	0.00
Q13620	Cullin-4B OS=Homo sapiens GN=CUL4B PE=1 SV=4 - [CUL4B_HUMAN]	0.30	0.09	0.43	0.22					0.3	0.04
P03952	Plasma kallikrein OS=Homo sapiens GN=KLKB1 PE=1 SV=1 - [KLKB1_HUMAN]	0.51	0.52	0.44	0.44	0.05	0.01	0.05	0.05	0.3	0.02
Q13093	Platelet-activating factor acetylhydrolase OS=Homo sapiens GN=PLA2G7 PE=1 SV=1 - [PAFA_HUMAN]	0.21	0.27	0.21	0.35					0.3	0.00

P02775	Platelet basic protein OS=Homo sapiens GN=PPBP PE=1 SV=3 - [CXCL7_HUMAN]	0.33	0.44	0.20	0.31	0.31	0.09	0.29	0.09	0.3	0.00
P55145	Mesencephalic astrocyte-derived neurotrophic factor OS=Homo sapiens GN=MANF PE=1 SV=3 - [MANF_HUMAN]					0.30	0.43	0.08	0.22	0.3	0.04
P01722	Ig lambda chain V-VI region NIG-48 OS=Homo sapiens PE=1 SV=1 - [LV602_HUMAN]	0.09	0.23	-0.02	0.12	0.15	0.13	0.68	0.66	0.3	0.03
Q9Y2I6	Ninein-like protein OS=Homo sapiens GN=NINL PE=1 SV=2 - [NINL_HUMAN]	0.24	0.30	0.19	0.25	0.47	0.13	0.40	0.06	0.3	0.00
A6NJ16	Putative V-set and immunoglobulin domain-containing-like protein IGHV4OR15-8 OS=Homo sapiens GN=IGHV4OR15-8 PE=5 SV=2 - [IV4F8_HUMAN]	0.04	0.13	0.04	0.11	0.53	0.59	0.27	0.32	0.3	0.01
P01023	Alpha-2-macroglobulin OS=Homo sapiens GN=A2M PE=1 SV=3 - [A2MG_HUMAN]	0.25	0.32	0.27	0.33	0.21	0.25	0.17	0.23	0.3	0.00

Q92743	Serine protease HTRA1 OS=Homo sapiens GN=HTRA1 PE=1 SV=1 - [HTRA1_HUMAN]	0.27	0.18	0.32	0.24					0.3	0.00
P07339	Cathepsin D OS=Homo sapiens GN=CTSD PE=1 SV=1 - [CATD_HUMAN]	0.32	0.26	0.28	0.26	0.25	0.39	0.06	0.19	0.3	0.00
A2VDJ0	Transmembrane protein 131-like OS=Homo sapiens GN=KIAA0922 PE=1 SV=2 - [T131L_HUMAN]					0.42	0.33	0.17	0.08	0.3	0.04
P15151	Poliovirus receptor OS=Homo sapiens GN=PVR PE=1 SV=2 - [PVR_HUMAN]	0.49	0.38	0.46	0.41	-0.09	0.04	0.08	0.23	0.3	0.01
Q86XA9	HEAT repeat-containing protein 5A OS=Homo sapiens GN=HEATR5A PE=1 SV=2 - [HTR5A_HUMAN]	0.60	0.54	0.28	0.23	0.07	0.05	0.12	0.10	0.3	0.01
P10720	Platelet factor 4 variant OS=Homo sapiens GN=PF4V1 PE=1 SV=1 - [PF4V_HUMAN]					0.34	0.31	0.27	0.08	0.3	0.02
P01778	Ig heavy chain V-III region ZAP OS=Homo sapiens PE=1 SV=1 - [HV317_HUMAN]	0.47	0.50	0.36	0.49	0.04	0.19	-0.09	0.03	0.2	0.02

Q96MH7	Uncharacterized protein C5orf34 OS=Homo sapiens GN=C5orf34 PE=2 SV=2 - [CE034_HUMAN]					0.41	0.34	0.16	0.09	0.2	0.04
Q15582	Transforming growth factor-beta-induced protein ig-h3 OS=Homo sapiens GN=TGFBI PE=1 SV=1 - [BGH3_HUMAN]	0.30	0.29	0.29	0.34	0.18	0.32	0.08	0.19	0.2	0.00
O15123	Angiopoietin-2 OS=Homo sapiens GN=ANGPT2 PE=1 SV=1 - [ANGP2_HUMAN]	0.24	0.24	0.25	0.26					0.2	0.00
Q96NZ9	Proline-rich acidic protein 1 OS=Homo sapiens GN=PRAP1 PE=2 SV=2 - [PRAP1_HUMAN]					0.34	0.28	0.24	0.12	0.2	0.01
P06276	Cholinesterase OS=Homo sapiens GN=BCHE PE=1 SV=1 - [CHLE_HUMAN]	0.15	0.18	0.15	0.18	0.33	0.42	0.25	0.31	0.2	0.00
P02788	Lactotransferrin OS=Homo sapiens GN=LTF PE=1 SV=6 - [TRFL_HUMAN]	0.33	0.31	0.35	0.30	0.16	0.31	0.07	0.13	0.2	0.00
Q5VWT5	Uncharacterized protein C1orf168 OS=Homo sapiens GN=C1orf168 PE=2 SV=1 - [CA168_HUMAN]	0.49	0.31	0.52	0.35	0.14	-0.26	0.40	0.00	0.2	0.04

O95466	Formin-like protein 1 OS=Homo sapiens GN=FMNL1 PE=1 SV=3 - [FMNL_HUMAN]	0.27	0.26	0.09	0.08	0.48	0.63	-0.01	0.15	0.2	0.01
O75122	CLIP-associating protein 2 OS=Homo sapiens GN=CLASP2 PE=1 SV=2 - [CLAP2_HUMAN]	0.23	0.22	0.26	0.27					0.2	0.00
Q9Y5C1	Angiopoietin-related protein 3 OS=Homo sapiens GN=ANGPTL3 PE=1 SV=1 - [ANGL3_HUMAN]	0.40	0.56	0.36	0.40	0.05	0.01	0.11	0.05	0.2	0.01
Q9UBX5	Fibulin-5 OS=Homo sapiens GN=FBLN5 PE=1 SV=1 - [FBLN5_HUMAN]	0.18	0.08	0.22	0.14	0.31	0.35	0.31	0.35	0.2	0.00
P01137	Transforming growth factor beta-1 OS=Homo sapiens GN=TGFB1 PE=1 SV=2 - [TGFB1_HUMAN]	0.23	0.15	0.29	0.16	0.23	0.18	0.36	0.34	0.2	0.00
Q96RU2	Ubiquitin carboxyl-terminal hydrolase 28 OS=Homo sapiens GN=USP28 PE=1 SV=1 - [UBP28_HUMAN]	0.25	0.28	0.19	0.24					0.2	0.00
O14786	Neuropilin-1 OS=Homo sapiens GN=NRP1 PE=1 SV=3 - [NRP1_HUMAN]	0.21	0.29	0.09	0.29	0.17	0.30	0.22	0.32	0.2	0.00

Q15052	Rho guanine nucleotide exchange factor 6 OS=Homo sapiens GN=ARHGEF6 PE=1 SV=2 - [ARHG6_HUMAN]	0.01	0.07	0.23	0.29	0.48	0.23	0.39	0.20	0.2	0.00
P01871	Ig mu chain C region OS=Homo sapiens GN=IGHM PE=1 SV=3 - [IGHM_HUMAN]	0.31	0.38	0.34	0.42	0.16	0.15	0.03	0.09	0.2	0.00
Q9UHG3	Prenylcysteine oxidase 1 OS=Homo sapiens GN=PCYOX1 PE=1 SV=3 - [PCYOX_HUMAN]	0.16	0.36	0.01	0.24	0.27	0.39	0.17	0.28	0.2	0.00
Q96GE4	Centrosomal protein of 95 kDa OS=Homo sapiens GN=CEP95 PE=1 SV=1 - [CEP95_HUMAN]					0.30	0.28	0.18	0.16	0.2	0.01
O15371	Eukaryotic translation initiation factor 3 subunit D OS=Homo sapiens GN=EIF3D PE=1 SV=1 - [EIF3D_HUMAN]	0.22	0.30	0.28	0.36	0.00		0.24		0.2	0.01
P48444	Coatomer subunit delta OS=Homo sapiens GN=ARCN1 PE=1 SV=1 - [COPD_HUMAN]					0.23	0.18	0.28	0.23	0.2	0.00
Q68DN1	Uncharacterized protein C2orf16 OS=Homo sapiens GN=C2orf16 PE=1 SV=3 - [CB016_HUMAN]	0.33	0.37	0.08	0.13					0.2	0.05

P36980	Complement factor H-related protein 2 OS=Homo sapiens GN=CFHR2 PE=1 SV=1 - [FHR2_HUMAN]	0.32	0.26	0.27	0.12	0.32	0.16	0.27	0.09	0.2	0.00
O75368	SH3 domain-binding glutamic acid-rich-like protein OS=Homo sapiens GN=SH3BGRL PE=1 SV=1 - [SH3L1_HUMAN]					0.23	0.30	0.20	0.17	0.2	0.00
Q6UX73	UPF0764 protein C16orf89 OS=Homo sapiens GN=C16orf89 PE=2 SV=2 - [CP089_HUMAN]	0.11	0.19	0.26	0.34					0.2	0.02
Q8N655	Uncharacterized protein C10orf12 OS=Homo sapiens GN=C10orf12 PE=1 SV=1 - [CJ012_HUMAN]	0.11	0.13	0.12	0.15	0.12	0.42	0.23	0.52	0.2	0.01
Q13813	Spectrin alpha chain, brain OS=Homo sapiens GN=SPTAN1 PE=1 SV=3 - [SPTA2_HUMAN]	0.32	0.32	0.33	0.33	0.20	0.24	0.00	0.05	0.2	0.00
Q9UJU6	Drebrin-like protein OS=Homo sapiens GN=DBNL PE=1 SV=1 - [DBNL_HUMAN]	0.00	-0.06	0.12	0.08	0.35	0.48	0.32	0.51	0.2	0.02
Q9H4A9	Dipeptidase 2 OS=Homo sapiens GN=DPEP2 PE=1 SV=2 - [DPEP2_HUMAN]	0.03	0.01	0.14	0.07	0.30	0.35	0.46	0.43	0.2	0.01

Q6XUX3	Dual serine/threonine and tyrosine protein kinase OS=Homo sapiens GN=DSTYK PE=1 SV=2 - [DUSTY_HUMAN]	0.58	0.33	0.55	0.32	-0.10	-0.07	0.07	0.11	0.2	0.05
P09172	Dopamine beta-hydroxylase OS=Homo sapiens GN=DBH PE=1 SV=3 - [DOPO_HUMAN]	0.23	0.25	0.18	0.25	0.18	0.37	0.06	0.25	0.2	0.00
P01760	Ig heavy chain V-I region WOL OS=Homo sapiens PE=1 SV=1 - [HV105_HUMAN]	0.24	0.44	0.44	0.49	-0.01	0.08	-0.08	0.14	0.2	0.03
P01702	Ig lambda chain V-I region NIG-64 OS=Homo sapiens PE=1 SV=1 - [LV104_HUMAN]	0.17	0.02	0.28	0.20	0.44	0.39	0.14	0.10	0.2	0.00
Q4ZHG4	Fibronectin type III domain-containing protein 1 OS=Homo sapiens GN=FNDC1 PE=1 SV=4 - [FNDC1_HUMAN]	0.18	0.11	0.47	0.43	0.25	0.13	0.14	0.02	0.2	0.01
P00748	Coagulation factor XII OS=Homo sapiens GN=F12 PE=1 SV=3 - [FA12_HUMAN]	0.40	0.35	0.41	0.40	0.07	-0.03	0.11	0.03	0.2	0.01
Q9NVD3	SET domain-containing protein 4 OS=Homo sapiens GN=SETD4 PE=2 SV=1 - [SETD4_HUMAN]					0.23	0.26	0.18	0.20	0.2	0.00

A6NI47	Putative POTE ankyrin domain family member M OS=Homo sapiens GN=POTEM PE=3 SV=2 - [POTEM_HUMAN]	0.17	0.42	0.01	0.27	0.29	0.19	0.24	0.14	0.2	0.00
P51178	1-phosphatidylinositol 4,5-bisphosphate phosphodiesterase delta-1 OS=Homo sapiens GN=PLCD1 PE=1 SV=2 - [PLCD1_HUMAN]	0.23	0.21	0.21	0.21					0.2	0.00
P54802	Alpha-N-acetylglucosaminidase OS=Homo sapiens GN=NAGLU PE=1 SV=2 - [ANAG_HUMAN]	0.12	0.11	0.16	0.17	0.25	0.23	0.43	0.25	0.2	0.00
Q92608	Dedicator of cytokinesis protein 2 OS=Homo sapiens GN=DOCK2 PE=1 SV=2 - [DOCK2_HUMAN]	0.21	0.12	0.26	0.20	0.34	0.03	0.48	0.08	0.2	0.00
P11277	Spectrin beta chain, erythrocyte OS=Homo sapiens GN=SPTB PE=1 SV=5 - [SPTB1_HUMAN]	0.12	0.29	0.08	0.36					0.2	0.05
Q96HC4	PDZ and LIM domain protein 5 OS=Homo sapiens GN=PDLIM5 PE=1 SV=5 - [PDLI5_HUMAN]					0.26	0.19	0.24	0.16	0.2	0.00

Q9HCF6	Transient receptor potential cation channel subfamily M member 3 OS=Homo sapiens GN=TRPM3 PE=2 SV=4 - [TRPM3_HUMAN]	0.13	0.24	0.15	0.27	0.35	0.41	0.04	0.10	0.2	0.00
P12931	Proto-oncogene tyrosine-protein kinase Src OS=Homo sapiens GN=SRC PE=1 SV=3 - [SRC_HUMAN]	0.22	0.53	0.28	0.60	-0.04	0.12	-0.09	0.08	0.2	0.05
Q86VM9	Zinc finger CCCH domain-containing protein 18 OS=Homo sapiens GN=ZC3H18 PE=1 SV=2 - [ZCH18_HUMAN]					0.22	0.07	0.35	0.20	0.2	0.03
P00450	Ceruloplasmin OS=Homo sapiens GN=CP PE=1 SV=1 - [CERU_HUMAN]	0.17	0.21	0.17	0.22	0.26	0.28	0.15	0.24	0.2	0.00
Q8NE35	Cytoplasmic polyadenylation element-binding protein 3 OS=Homo sapiens GN=CPEB3 PE=1 SV=2 - [CPEB3_HUMAN]	0.37	0.23	0.19	0.05					0.2	0.05
O15067	Phosphoribosylformylglycinamidine synthase OS=Homo sapiens GN=PFAS PE=1 SV=4 - [PUR4_HUMAN]	0.22	0.25	0.15	0.22					0.2	0.00

Q9UKK3	Poly [ADP-ribose] polymerase 4 OS=Homo sapiens GN=PARP4 PE=1 SV=3 - [PARP4_HUMAN]	0.15	0.24	0.18	0.27					0.2	0.00
Q15848	Adiponectin OS=Homo sapiens GN=ADIPOQ PE=1 SV=1 - [ADIPO_HUMAN]	0.27	0.35	0.34	0.37	0.09	0.18	-0.02	0.08	0.2	0.01
P01591	Immunoglobulin J chain OS=Homo sapiens GN=IGJ PE=1 SV=4 - [IGJ_HUMAN]	0.23	0.15	0.37	0.30	0.19	0.12	0.20	0.10	0.2	0.00
Q5VVJ2	Histone H2A deubiquitinase MYSM1 OS=Homo sapiens GN=MYSM1 PE=1 SV=1 - [MYSM1_HUMAN]	0.02	0.25	-0.04	0.19	0.40	0.46	0.11	0.27	0.2	0.01
O00602	Ficolin-1 OS=Homo sapiens GN=FCN1 PE=1 SV=2 - [FCN1_HUMAN]	0.12	0.08	0.18	0.14	0.09	0.26	0.31	0.48	0.2	0.00
P05090	Apolipoprotein D OS=Homo sapiens GN=APOD PE=1 SV=1 - [APOD_HUMAN]	0.22	0.11	0.20	0.10	0.25	0.31	0.20	0.25	0.2	0.00
Q3ZCW2	Galectin-related protein OS=Homo sapiens GN=LGALSL PE=1 SV=2 - [LEGL_HUMAN]	0.06	0.54	-0.16	0.21	0.33	0.26	0.27	0.13	0.2	0.03

Q04756	Hepatocyte growth factor activator OS=Homo sapiens GN=HGFAC PE=1 SV=1 - [HGFA_HUMAN]	0.29	0.26	0.27	0.27	0.16	0.12	0.12	0.13	0.2	0.00
P19652	Alpha-1-acid glycoprotein 2 OS=Homo sapiens GN=ORM2 PE=1 SV=2 - [A1AG2_HUMAN]	0.18	0.12	0.23	0.14	0.31	0.15	0.32	0.18	0.2	0.00
Q6ZWB6	BTB/POZ domain-containing protein KCTD8 OS=Homo sapiens GN=KCTD8 PE=2 SV=1 - [KCTD8_HUMAN]	0.35	0.36	0.31	0.32	0.09	-0.24	0.38	0.05	0.2	0.03
Q15084	Protein disulfide-isomerase A6 OS=Homo sapiens GN=PDIA6 PE=1 SV=1 - [PDIA6_HUMAN]					0.08	0.22	0.19	0.33	0.2	0.03
Q8IUC8	Polypeptide N-acetylgalactosaminyltransferase 13 OS=Homo sapiens GN=GALNT13 PE=2 SV=2 - [GLT13_HUMAN]	0.23	0.38	-0.18	-0.02	0.34	0.24	0.33	0.30	0.2	0.02
Q12851	Mitogen-activated protein kinase kinase kinase kinase 2 OS=Homo sapiens GN=MAP4K2 PE=1 SV=2 - [M4K2_HUMAN]	0.39	0.00	0.48	0.11	-0.10	0.17	0.14	0.42	0.2	0.03

Q13094	Lymphocyte cytosolic protein 2 OS=Homo sapiens GN=LCP2 PE=1 SV=1 - [LCP2_HUMAN]	0.11	0.43	0.02	0.34	0.15	0.22	0.14	0.20	0.2	0.00
P11226	Mannose-binding protein C OS=Homo sapiens GN=MBL2 PE=1 SV=2 - [MBL2_HUMAN]	0.14	0.28	0.16	0.33	0.22	0.18	0.18	0.12	0.2	0.00
Q14847	LIM and SH3 domain protein 1 OS=Homo sapiens GN=LASP1 PE=1 SV=2 - [LASP1_HUMAN]					0.29	0.07	0.31	0.13	0.2	0.05
O95866	Protein G6b OS=Homo sapiens GN=G6B PE=1 SV=1 - [G6B_HUMAN]					0.20	0.23	0.19	0.18	0.2	0.00
P51816	AF4/FMR2 family member 2 OS=Homo sapiens GN=AFF2 PE=1 SV=4 - [AFF2_HUMAN]	-0.05	0.43	-0.08	0.42	0.27	0.19	0.12	0.28	0.2	0.02
Q13332	Receptor-type tyrosine-protein phosphatase S OS=Homo sapiens GN=PTPRS PE=1 SV=3 - [PTPRS_HUMAN]	0.26	0.20	0.17	0.16					0.2	0.00
P19827	Inter-alpha-trypsin inhibitor heavy chain H1 OS=Homo sapiens GN=ITIH1 PE=1 SV=3 - [ITIH1_HUMAN]	0.12	0.14	0.14	0.14	0.19	0.30	0.19	0.33	0.2	0.00

Q9H8L6	Multimerin-2 OS=Homo sapiens GN=MMRN2 PE=1 SV=2 - [MMRN2_HUMAN]	0.29	0.18	0.53	0.46	0.05	0.07	-0.03	0.02	0.2	0.03
P34096	Ribonuclease 4 OS=Homo sapiens GN=RNASE4 PE=1 SV=3 - [RNAS4_HUMAN]					0.29	0.11	0.18	0.20	0.2	0.01
Q76LX8	A disintegrin and metalloproteinase with thrombospondin motifs 13 OS=Homo sapiens GN=ADAMTS13 PE=1 SV=1 - [ATS13_HUMAN]	0.26	0.31	0.18	0.21	0.29	0.01	0.23	0.05	0.2	0.00
P20827	Ephrin-A1 OS=Homo sapiens GN=EFNA1 PE=1 SV=2 - [EFNA1_HUMAN]					0.17	0.20	0.18	0.21	0.2	0.00
Q92954	Proteoglycan 4 OS=Homo sapiens GN=PRG4 PE=1 SV=2 - [PRG4_HUMAN]	0.20	0.24	0.19	0.28	0.25	0.12	0.20	0.05	0.2	0.00
Q9UJC5	SH3 domain-binding glutamic acid-rich-like protein 2 OS=Homo sapiens GN=SH3BGRL2 PE=1 SV=2 - [SH3L2_HUMAN]					0.22	0.18	0.23	0.13	0.2	0.00
Q9H1B5	Xylosyltransferase 2 OS=Homo sapiens GN=XYLT2 PE=2 SV=2 - [XYLT2_HUMAN]	0.22	0.19	0.18	0.16					0.2	0.00

P28799	Granulins OS=Homo sapiens GN=GRN PE=1 SV=2 - [GRN_HUMAN]	0.07	0.09	0.24	0.26	0.20	0.22	0.21	0.21	0.2	0.00
Q96A00	Protein phosphatase 1 regulatory subunit 14A OS=Homo sapiens GN=PPP1R14A PE=1 SV=1 - [PP14A_HUMAN]					0.30	0.20	0.19	0.05	0.2	0.04
Q92547	DNA topoisomerase 2-binding protein 1 OS=Homo sapiens GN=TOPBP1 PE=1 SV=3 - [TOPB1_HUMAN]	0.10	0.13	0.24	0.27					0.2	0.02
P04196	Histidine-rich glycoprotein OS=Homo sapiens GN=HRG PE=1 SV=1 - [HRG_HUMAN]	0.36	0.29	0.37	0.30	0.05	-0.02	0.06	0.04	0.2	0.02
Q9UK61	Protein FAM208A OS=Homo sapiens GN=FAM208A PE=1 SV=3 - [F208A_HUMAN]	0.13	-0.08	0.23	0.02	0.51	0.25	0.31	0.05	0.2	0.03
Q8NA96	Putative uncharacterized protein FLJ35723 OS=Homo sapiens PE=5 SV=2 - [YE027_HUMAN]	0.10	0.23	0.13	0.26					0.2	0.02
P0CG05	Ig lambda-2 chain C regions OS=Homo sapiens GN=IGLC2 PE=1 SV=1 - [LAC2_HUMAN]	0.15	0.06	0.17	0.08	0.29	0.36	0.11	0.22	0.2	0.00

P05543	Thyroxine-binding globulin OS=Homo sapiens GN=SERPINA7 PE=1 SV=2 - [THBG_HUMAN]	0.33	0.38	0.30	0.37	0.06	0.07	-0.07	-0.01	0.2	0.03
P01762	Ig heavy chain V-III region TRO OS=Homo sapiens PE=1 SV=1 - [HV301_HUMAN]	0.23	0.27	0.33	0.25	0.06	0.08	0.03	0.17	0.2	0.00
Q9BX66	Sorbin and SH3 domain-containing protein 1 OS=Homo sapiens GN=SORBS1 PE=1 SV=3 - [SRBS1_HUMAN]					0.15	0.29	0.06	0.20	0.2	0.04
Q6ISB3	Grainyhead-like protein 2 homolog OS=Homo sapiens GN=GRHL2 PE=1 SV=1 - [GRHL2_HUMAN]					0.13	0.05	0.31	0.22	0.2	0.05
P60983	Glia maturation factor beta OS=Homo sapiens GN=GMFB PE=1 SV=2 - [GMFB_HUMAN]					0.24	0.18	0.11	0.17	0.2	0.01
Q9HD89	Resistin OS=Homo sapiens GN=RETN PE=2 SV=1 - [RETN_HUMAN]	0.11	0.01	0.10	0.01	0.34	0.23	0.35	0.24	0.2	0.01
P00734	Prothrombin OS=Homo sapiens GN=F2 PE=1 SV=2 - [THRB_HUMAN]	0.14	0.09	0.12	0.09	0.23	0.24	0.23	0.24	0.2	0.00
O95445	Apolipoprotein M OS=Homo sapiens GN=APOM PE=1 SV=2 - [APOM_HUMAN]	0.04	0.01	0.07	0.08	0.33	0.29	0.28	0.28	0.2	0.01

P01009	Alpha-1-antitrypsin OS=Homo sapiens GN=SERPINA1 PE=1 SV=3 - [A1AT_HUMAN]	0.28	0.25	0.29	0.25	0.09	0.07	0.06	0.08	0.2	0.00
Q9Y2V0	Uncharacterized protein C15orf41 OS=Homo sapiens GN=C15orf41 PE=2 SV=2 - [CO041_HUMAN]	0.19	0.04	0.25	0.19					0.2	0.04
P15509	Granulocyte-macrophage colony-stimulating factor receptor subunit alpha OS=Homo sapiens GN=CSF2RA PE=1 SV=1 - [CSF2R_HUMAN]	0.21	0.27	0.23	0.30	0.07	0.12	0.04	0.09	0.2	0.00
Q01581	Hydroxymethylglutaryl-CoA synthase, cytoplasmic OS=Homo sapiens GN=HMGCS1 PE=1 SV=2 - [HMCS1_HUMAN]	0.22	0.21	0.01	0.00	0.21	0.37	0.07	0.24	0.2	0.01
Q9BZQ8	Protein Niban OS=Homo sapiens GN=FAM129A PE=1 SV=1 - [NIBAN_HUMAN]	0.13	0.21	0.35	0.43	0.24	-0.04	0.17	-0.15	0.2	0.04
P01719	Ig lambda chain V-V region DEL OS=Homo sapiens PE=1 SV=1 - [LV501_HUMAN]	0.15	0.23	0.12	0.20	0.18	0.04	0.26	0.15	0.2	0.00

P33908	Mannosyl-oligosaccharide 1,2-alpha-mannosidase IA OS=Homo sapiens GN=MAN1A1 PE=1 SV=3 - [MA1A1_HUMAN]	0.03	0.03	0.11	0.07	0.33	0.38	0.14	0.21	0.2	0.01
P01040	Cystatin-A OS=Homo sapiens GN=CSTA PE=1 SV=1 - [CYTA_HUMAN]					0.24	0.12	0.20	0.07	0.2	0.03
O43866	CD5 antigen-like OS=Homo sapiens GN=CD5L PE=1 SV=1 - [CD5L_HUMAN]	0.12	0.09	0.16	0.12	0.20	0.23	0.20	0.14	0.2	0.00
Q8IZP9	G-protein coupled receptor 64 OS=Homo sapiens GN=GPR64 PE=1 SV=2 - [GPR64_HUMAN]	0.12	0.14	0.17	0.20					0.2	0.00
Q14623	Indian hedgehog protein OS=Homo sapiens GN=IHH PE=1 SV=4 - [IHH_HUMAN]	0.11	0.07	0.10	0.05	0.15	0.32	0.14	0.31	0.2	0.00
O00273	DNA fragmentation factor subunit alpha OS=Homo sapiens GN=DFFA PE=1 SV=1 - [DFFA_HUMAN]					0.11	0.25	0.21	0.06	0.2	0.04

O95602	DNA-directed RNA polymerase I subunit RPA1 OS=Homo sapiens GN=POLR1A PE=1 SV=2 - [RPA1_HUMAN]					0.22	0.13	0.16	0.11	0.2	0.01
P02787	Serotransferrin OS=Homo sapiens GN=TF PE=1 SV=3 - [TRFE_HUMAN]	0.31	0.25	0.31	0.25	0.04	0.03	0.01	0.04	0.2	0.01
Q99558	Mitogen-activated protein kinase kinase kinase 14 OS=Homo sapiens GN=MAP3K14 PE=1 SV=2 - [M3K14_HUMAN]	0.10	0.21	0.10	0.21					0.2	0.02
P04114	Apolipoprotein B-100 OS=Homo sapiens GN=APOB PE=1 SV=2 - [APOB_HUMAN]	0.23	0.27	0.22	0.28	0.06	0.14	-0.02	0.04	0.2	0.01
P10643	Complement component C7 OS=Homo sapiens GN=C7 PE=1 SV=2 - [CO7_HUMAN]	0.28	0.25	0.25	0.23	0.06	0.09	0.01	0.05	0.2	0.01
P02749	Beta-2-glycoprotein 1 OS=Homo sapiens GN=APOH PE=1 SV=3 - [APOH_HUMAN]	0.21	0.13	0.20	0.13	0.12	0.15	0.16	0.12	0.2	0.00

Q8NDG6	Putative ATP-dependent RNA helicase TDRD9 OS=Homo sapiens GN=TDRD9 PE=2 SV=3 - [TDRD9_HUMAN]					0.18	0.04	0.22	0.17	0.2	0.03
P04004	Vitronectin OS=Homo sapiens GN=VTN PE=1 SV=1 - [VTNC_HUMAN]	0.15	0.16	0.15	0.14	0.13	0.16	0.14	0.18	0.2	0.00
Q14678	KN motif and ankyrin repeat domain-containing protein 1 OS=Homo sapiens GN=KANK1 PE=1 SV=3 - [KANK1_HUMAN]					-0.25	-0.17	-0.13	-0.05	-0.2	0.04
P49908	Selenoprotein P OS=Homo sapiens GN=SEPP1 PE=1 SV=3 - [SEPP1_HUMAN]	-0.08	-0.05	-0.10	-0.08	-0.21	-0.24	-0.22	-0.23	-0.2	0.00
P49257	Protein ERGIC-53 OS=Homo sapiens GN=LMAN1 PE=1 SV=2 - [LMAN1_HUMAN]	-0.11	-0.16	-0.08	-0.14	0.01	-0.15	-0.21	-0.37	-0.2	0.01
P41222	Prostaglandin-H2 D-isomerase OS=Homo sapiens GN=PTGDS PE=1 SV=1 - [PTGDS_HUMAN]	-0.17	-0.25	-0.27	-0.33	0.06	-0.07	-0.02	-0.14	-0.2	0.01
P28062	Proteasome subunit beta type-8 OS=Homo sapiens GN=PSMB8 PE=1 SV=3 - [PSB8_HUMAN]	-0.30	-0.25	-0.18	-0.12	-0.20	-0.15	-0.05	0.00	-0.2	0.00

P01877	Ig alpha-2 chain C region OS=Homo sapiens GN=IGHA2 PE=1 SV=3 - [IGHA2_HUMAN]	0.01	0.01	-0.04	-0.04	-0.27	-0.29	-0.31	-0.32	-0.2	0.02
P05155	Plasma protease C1 inhibitor OS=Homo sapiens GN=SERPING1 PE=1 SV=2 - [IC1_HUMAN]	-0.08	-0.09	-0.09	-0.11	-0.23	-0.18	-0.29	-0.18	-0.2	0.00
P17900	Ganglioside GM2 activator OS=Homo sapiens GN=GM2A PE=1 SV=4 - [SAP3_HUMAN]					-0.08	-0.11	-0.21	-0.24	-0.2	0.03
P00558	Phosphoglycerate kinase 1 OS=Homo sapiens GN=PGK1 PE=1 SV=3 - [PGK1_HUMAN]	-0.16	-0.38	-0.11	-0.27	-0.27	-0.08	-0.09	0.10	-0.2	0.02
P14618	Pyruvate kinase isozymes M1/M2 OS=Homo sapiens GN=PKM2 PE=1 SV=4 - [KPYM_HUMAN]	-0.31	-0.24	-0.32	-0.28	-0.10	-0.11	0.07	0.03	-0.2	0.02
P48637	Glutathione synthetase OS=Homo sapiens GN=GSS PE=1 SV=1 - [GSHB_HUMAN]	-0.15	-0.16	-0.16	-0.17					-0.2	0.00
Q02985	Complement factor H-related protein 3 OS=Homo sapiens GN=CFHR3 PE=1 SV=2 - [FHR3_HUMAN]	-0.20	-0.14	-0.14	-0.03	-0.19	-0.38	-0.03	-0.20	-0.2	0.00

Q03113	Guanine nucleotide-binding protein subunit alpha-12 OS=Homo sapiens GN=GNA12 PE=1 SV=4 - [GNA12_HUMAN]	-0.39	-0.17	-0.35	-0.12	-0.25	0.07	-0.21	0.11	-0.2	0.04
Q99439	Calponin-2 OS=Homo sapiens GN=CNN2 PE=1 SV=4 - [CNN2_HUMAN]	-0.33	-0.35	-0.34	-0.35	0.10	0.08	-0.01	-0.12	-0.2	0.05
Q13495	Mastermind-like domain-containing protein 1 OS=Homo sapiens GN=MAMLD1 PE=1 SV=2 - [MAMD1_HUMAN]					-0.16	-0.18	-0.15	-0.17	-0.2	0.00
P15531	Nucleoside diphosphate kinase A OS=Homo sapiens GN=NME1 PE=1 SV=1 - [NDKA_HUMAN]					-0.23	-0.07	-0.25	-0.13	-0.2	0.03
Q03164	Histone-lysine N-methyltransferase MLL OS=Homo sapiens GN=MLL PE=1 SV=5 - [MLL1_HUMAN]	-0.23	-0.17	-0.35	-0.29	-0.03	0.03	-0.19	-0.13	-0.2	0.01
P02647	Apolipoprotein A-I OS=Homo sapiens GN=APOA1 PE=1 SV=1 - [APOA1_HUMAN]	-0.25	-0.35	-0.24	-0.34	-0.03	-0.07	-0.02	-0.06	-0.2	0.01

Q96TA2	ATP-dependent zinc metalloprotease YME1L1 OS=Homo sapiens GN=YME1L1 PE=1 SV=2 - [YMEL1_HUMAN]	-0.11	-0.13	-0.22	-0.24					-0.2	0.01
O76031	ATP-dependent Clp protease ATP-binding subunit clpX-like, mitochondrial OS=Homo sapiens GN=CLPX PE=1 SV=2 - [CLPX_HUMAN]	-0.24	-0.14	-0.23	-0.11					-0.2	0.01
P02786	Transferrin receptor protein 1 OS=Homo sapiens GN=TFRC PE=1 SV=2 - [TFR1_HUMAN]	-0.08	-0.07	-0.09	-0.09	-0.28	-0.26	-0.29	-0.27	-0.2	0.00
P20073	Annexin A7 OS=Homo sapiens GN=ANXA7 PE=1 SV=3 - [ANXA7_HUMAN]	-0.21	-0.19	-0.17	-0.14					-0.2	0.00
Q9UHK0	Nuclear fragile X mental retardation-interacting protein 1 OS=Homo sapiens GN=NUFIP1 PE=1 SV=2 - [NUFP1_HUMAN]	-0.08	-0.06	-0.16	-0.13	-0.36	0.03	-0.53	-0.14	-0.2	0.03
P60953	Cell division control protein 42 homolog OS=Homo sapiens GN=CDC42 PE=1 SV=2 - [CDC42_HUMAN]	-0.42	-0.38	-0.40	-0.35	0.10	0.01	0.03	-0.06	-0.2	0.05

Q9NZR2	Low-density lipoprotein receptor-related protein 1B OS=Homo sapiens GN=LRP1B PE=1 SV=2 - [LRP1B_HUMAN]	-0.16	-0.19	-0.13	-0.15	-0.33	-0.21	-0.27	-0.03	-0.2	0.00
P42858	Huntingtin OS=Homo sapiens GN=HTT PE=1 SV=2 - [HD_HUMAN]	-0.17	-0.22	-0.14	-0.19					-0.2	0.00
Q9BS26	Endoplasmic reticulum resident protein 44 OS=Homo sapiens GN=ERP44 PE=1 SV=1 - [ERP44_HUMAN]	-0.41	-0.40	-0.26	-0.25	0.01	-0.05	-0.03	-0.08	-0.2	0.02
Q6AHZ1	Zinc finger protein 518A OS=Homo sapiens GN=ZNF518A PE=1 SV=2 - [Z518A_HUMAN]	-0.41	-0.25	-0.45	-0.28	0.03	-0.10	0.06	-0.07	-0.2	0.03
P04220	Ig mu heavy chain disease protein OS=Homo sapiens PE=1 SV=1 - [MUCB_HUMAN]	-0.19	-0.27	-0.16	-0.12					-0.2	0.01
Q6KC79	Nipped-B-like protein OS=Homo sapiens GN=NIPBL PE=1 SV=2 - [NIPBL_HUMAN]					-0.11	-0.26	-0.12	-0.27	-0.2	0.02
P30086	Phosphatidylethanolamine-binding protein 1 OS=Homo sapiens GN=PEBP1 PE=1 SV=3 - [PEBP1_HUMAN]	-0.10	-0.22	-0.17	-0.27	-0.08	-0.20	-0.16	-0.28	-0.2	0.00

Q8N3S3	Putative homeodomain transcription factor 2 OS=Homo sapiens GN=PHTF2 PE=2 SV=2 - [PHTF2_HUMAN]	-0.29	0.10	-0.34	0.06	-0.36	-0.48	-0.03	-0.16	-0.2	0.04
O94979	Protein transport protein Sec31A OS=Homo sapiens GN=SEC31A PE=1 SV=3 - [SC31A_HUMAN]					-0.14	-0.30	-0.07	-0.25	-0.2	0.03
Q86VB7	Scavenger receptor cysteine-rich type 1 protein M130 OS=Homo sapiens GN=CD163 PE=1 SV=2 - [C163A_HUMAN]	-0.15	-0.16	-0.28	-0.32	-0.10	-0.28	-0.10	-0.10	-0.2	0.00
P55290	Cadherin-13 OS=Homo sapiens GN=CDH13 PE=1 SV=1 - [CAD13_HUMAN]	-0.10	-0.28	-0.15	-0.33	-0.21	-0.28	-0.04	-0.13	-0.2	0.00
Q96M69	Leucine-rich repeat and guanylate kinase domain-containing protein OS=Homo sapiens GN=LRGUK PE=1 SV=1 - [LRGUK_HUMAN]	-0.08	-0.18	-0.18	-0.28	-0.58	-0.22	-0.19	0.17	-0.2	0.03
P61158	Actin-related protein 3 OS=Homo sapiens GN=ACTR3 PE=1 SV=3 - [ARP3_HUMAN]	-0.33	-0.46	-0.07	-0.12	0.11	-0.38	0.09	-0.40	-0.2	0.04

P62993	Growth factor receptor-bound protein 2 OS=Homo sapiens GN=GRB2 PE=1 SV=1 - [GRB2_HUMAN]					-0.24	-0.21	-0.19	-0.15	-0.2	0.00
P60174	Triosephosphate isomerase OS=Homo sapiens GN=TPI1 PE=1 SV=3 - [TPIS_HUMAN]	-0.12	-0.27	-0.06	-0.25	-0.23	-0.22	-0.22	-0.21	-0.2	0.00
Q68CR7	Leucine-rich repeat-containing protein 66 OS=Homo sapiens GN=LRRC66 PE=2 SV=1 - [LRC66_HUMAN]					-0.21	-0.30	-0.10	-0.18	-0.2	0.02
P08134	Rho-related GTP-binding protein RhoC OS=Homo sapiens GN=RHOC PE=1 SV=1 - [RHOC_HUMAN]	-0.20	-0.12	-0.21	-0.13	-0.20	-0.16	-0.33	-0.24	-0.2	0.00
Q70J99	Protein unc-13 homolog D OS=Homo sapiens GN=UNC13D PE=1 SV=1 - [UN13D_HUMAN]	-0.30	-0.55	0.16	-0.08	0.02	-0.20	-0.21	-0.44	-0.2	0.05
P35555	Fibrillin-1 OS=Homo sapiens GN=FBN1 PE=1 SV=3 - [FBN1_HUMAN]	-0.03	-0.16	-0.13	-0.24	0.05	-0.27	-0.26	-0.57	-0.2	0.02
O43795	Unconventional myosin-Ib OS=Homo sapiens GN=MYO1B PE=2 SV=3 - [MYO1B_HUMAN]					-0.25	-0.18	-0.22	-0.16	-0.2	0.00

Q8IY37	Probable ATP-dependent RNA helicase DHX37 OS=Homo sapiens GN=DHX37 PE=1 SV=1 - [DHX37_HUMAN]	-0.38	-0.04	-0.43	-0.28	0.00		-0.10		-0.2	0.04
Q9UBP4	Dickkopf-related protein 3 OS=Homo sapiens GN=DKK3 PE=1 SV=2 - [DKK3_HUMAN]	-0.08	-0.30	-0.12	-0.33					-0.2	0.05
P06744	Glucose-6-phosphate isomerase OS=Homo sapiens GN=GPI PE=1 SV=4 - [G6PI_HUMAN]	-0.11	-0.22	-0.20	-0.29					-0.2	0.01
O14498	Immunoglobulin superfamily containing leucine-rich repeat protein OS=Homo sapiens GN=ISLR PE=1 SV=1 - [ISLR_HUMAN]	-0.02	-0.08	0.05	-0.06	-0.40	-0.28	-0.47	-0.39	-0.2	0.02
P00568	Adenylate kinase isoenzyme 1 OS=Homo sapiens GN=AK1 PE=1 SV=3 - [KAD1_HUMAN]	-0.29	-0.56	-0.25	-0.51	0.02	0.08	-0.11	-0.04	-0.2	0.04
P46531	Neurogenic locus notch homolog protein 1 OS=Homo sapiens GN=NOTCH1 PE=1 SV=4 - [NOTC1_HUMAN]	-0.12	-0.18	-0.24	-0.30					-0.2	0.01

P52565	Rho GDP-dissociation inhibitor 1 OS=Homo sapiens GN=ARHGDIA PE=1 SV=3 - [GDIR1_HUMAN]					-0.21	-0.19	-0.22	-0.23	-0.2	0.00
Q6R327	Rapamycin-insensitive companion of mTOR OS=Homo sapiens GN=RICTOR PE=1 SV=1 - [RICTR_HUMAN]	-0.14	-0.26	-0.18	-0.28					-0.2	0.01
P00451	Coagulation factor VIII OS=Homo sapiens GN=F8 PE=1 SV=1 - [FA8_HUMAN]	-0.08	-0.20	-0.03	-0.13	-0.45	-0.64	0.00	-0.18	-0.2	0.03
Q9Y5Y7	Lymphatic vessel endothelial hyaluronic acid receptor 1 OS=Homo sapiens GN=LYVE1 PE=1 SV=2 - [LYVE1_HUMAN]	-0.23	-0.13	-0.24	-0.11	-0.12	-0.22	-0.30	-0.36	-0.2	0.00
Q16445	Gamma-aminobutyric acid receptor subunit alpha-6 OS=Homo sapiens GN=GABRA6 PE=2 SV=2 - [GBRA6_HUMAN]	-0.51	-0.55	-0.10	-0.14	-0.41	-0.09	-0.19	0.25	-0.2	0.05
P01861	Ig gamma-4 chain C region OS=Homo sapiens GN=IGHG4 PE=1 SV=1 - [IGHG4_HUMAN]	-0.20	-0.18	-0.18	-0.17	-0.32	-0.16	-0.35	-0.19	-0.2	0.00

P30044	Peroxiredoxin-5, mitochondrial OS=Homo sapiens GN=PRDX5 PE=1 SV=4 - [PRDX5_HUMAN]	-0.41	-0.48	-0.30	-0.36	-0.09	0.06	-0.21	0.02	-0.2	0.02
Q15365	Poly(rC)-binding protein 1 OS=Homo sapiens GN=PCBP1 PE=1 SV=2 - [PCBP1_HUMAN]	-0.05	-0.22	-0.11	-0.26	-0.39	-0.06	-0.51	-0.18	-0.2	0.01
P47895	Aldehyde dehydrogenase family 1 member A3 OS=Homo sapiens GN=ALDH1A3 PE=1 SV=2 - [AL1A3_HUMAN]	-0.21	-0.35	-0.32	-0.44	0.07	-0.18	-0.05	-0.30	-0.2	0.01
Q9H871	Protein RMD5 homolog A OS=Homo sapiens GN=RMND5A PE=1 SV=1 - [RMD5A_HUMAN]	-0.29	-0.35	-0.13	-0.14					-0.2	0.03
O43819	Protein SCO2 homolog, mitochondrial OS=Homo sapiens GN=SCO2 PE=1 SV=3 - [SCO2_HUMAN]					-0.36	-0.22	-0.24	-0.09	-0.2	0.03
Q6PIV7	Solute carrier family 25 member 34 OS=Homo sapiens GN=SLC25A34 PE=2 SV=1 - [S2534_HUMAN]					-0.29	-0.18	-0.28	-0.17	-0.2	0.01

O00299	Chloride intracellular channel protein 1 OS=Homo sapiens GN=CLIC1 PE=1 SV=4 - [CLIC1_HUMAN]	-0.26	-0.23	-0.34	-0.30	-0.20	-0.15	-0.17	-0.18	-0.2	0.00
P25054	Adenomatous polyposis coli protein OS=Homo sapiens GN=APC PE=1 SV=2 - [APC_HUMAN]					-0.25	-0.06	-0.39	-0.21	-0.2	0.04
O75592	Probable E3 ubiquitin-protein ligase MYCBP2 OS=Homo sapiens GN=MYCBP2 PE=1 SV=3 - [MYCB2_HUMAN]	-0.17	-0.03	-0.20	-0.05	-0.43	-0.33	-0.37	-0.27	-0.2	0.00
P20702	Integrin alpha-X OS=Homo sapiens GN=ITGAX PE=1 SV=3 - [ITAX_HUMAN]	0.12	0.08	-0.05	-0.07	-0.46	-0.44	-0.53	-0.51	-0.2	0.05
Q5VT97	Rho GTPase-activating protein SYDE2 OS=Homo sapiens GN=SYDE2 PE=1 SV=2 - [SYDE2_HUMAN]	-0.26	-0.10	-0.37	-0.21					-0.2	0.02
A1L4H1	Soluble scavenger receptor cysteine-rich domain-containing protein SSC5D OS=Homo sapiens GN=SSC5D PE=2 SV=3 - [SRCRL_HUMAN]	-0.15	-0.26	-0.28	-0.24					-0.2	0.00

Q9H0G5	Nuclear speckle splicing regulatory protein 1 OS=Homo sapiens GN=NSRP1 PE=1 SV=1 - [NSRP1_HUMAN]	-0.07	-0.21	-0.28	-0.40					-0.2	0.04
Q6P1X5	Transcription initiation factor TFIID subunit 2 OS=Homo sapiens GN=TAF2 PE=1 SV=3 - [TAF2_HUMAN]	-0.34	-0.23	-0.25	-0.13					-0.2	0.01
P61224	Ras-related protein Rap-1b OS=Homo sapiens GN=RAP1B PE=1 SV=1 - [RAP1B_HUMAN]	-0.29	-0.50	-0.11	-0.36	-0.22	-0.21	0.00	-0.22	-0.2	0.00
Q96L92	Sorting nexin-27 OS=Homo sapiens GN=SNX27 PE=1 SV=2 - [SNX27_HUMAN]	0.05	-0.11	0.12	-0.01	-0.61	-0.35	-0.53	-0.46	-0.2	0.05
Q9UL68	Myelin transcription factor 1-like protein OS=Homo sapiens GN=MYT1L PE=2 SV=3 - [MYT1L_HUMAN]	-0.24	-0.18	-0.31	-0.23					-0.2	0.00
P61764	Syntaxin-binding protein 1 OS=Homo sapiens GN=STXBP1 PE=1 SV=1 - [STXB1_HUMAN]	-0.18	-0.31	-0.57	-0.64	-0.04	-0.24	0.13	-0.07	-0.2	0.04

Q6P1N0	Coiled-coil and C2 domain-containing protein 1A OS=Homo sapiens GN=CC2D1A PE=1 SV=1 - [C2D1A_HUMAN]	-0.17	-0.16	-0.06	-0.04	-0.39	-0.38	-0.36	-0.36	-0.2	0.00
Q9NXP7	Gypsy retrotransposon integrase-like protein 1 OS=Homo sapiens GN=GIN1 PE=2 SV=3 - [GIN1_HUMAN]	-0.49	0.11	-0.64	-0.03	-0.28	-0.43	-0.02	-0.16	-0.2	0.03
P20774	Mimecan OS=Homo sapiens GN=OGN PE=1 SV=1 - [MIME_HUMAN]	-0.09	-0.13	-0.10	-0.13	-0.28	-0.28	-0.48	-0.48	-0.2	0.00
P08107	Heat shock 70 kDa protein 1A/1B OS=Homo sapiens GN=HSPA1A PE=1 SV=5 - [HSP71_HUMAN]	-0.06	-0.05	-0.15	-0.14	-0.50	-0.37	-0.42	-0.29	-0.2	0.00
Q7KZ85	Transcription elongation factor SPT6 OS=Homo sapiens GN=SUPT6H PE=1 SV=2 - [SPT6H_HUMAN]	-0.42	-0.41	-0.30	-0.27	-0.02	-0.27	-0.04	-0.26	-0.2	0.00
Q9Y5E7	Protocadherin beta-2 OS=Homo sapiens GN=PCDHB2 PE=1 SV=1 - [PCDB2_HUMAN]	-0.24	-0.24	-0.24	-0.24	-0.31	-0.28	-0.23	-0.21	-0.2	0.00

P02452	Collagen alpha-1(I) chain OS=Homo sapiens GN=COL1A1 PE=1 SV=5 - [CO1A1_HUMAN]	-0.12	-0.38	-0.11	-0.35	-0.21	-0.42	-0.03	-0.37	-0.2	0.00
Q8NG41	Neuropeptide B OS=Homo sapiens GN=NPB PE=1 SV=1 - [NPB_HUMAN]					-0.31	-0.29	-0.18	-0.23	-0.3	0.00
Q9NVM6	DnaJ homolog subfamily C member 17 OS=Homo sapiens GN=DNAJC17 PE=1 SV=1 - [DJC17_HUMAN]					-0.30	-0.13	-0.33	-0.25	-0.3	0.01
O14793	Growth/differentiation factor 8 OS=Homo sapiens GN=MSTN PE=1 SV=1 - [GDF8_HUMAN]					-0.32	-0.34	-0.17	-0.18	-0.3	0.01
P16930	Fumarylacetoacetase OS=Homo sapiens GN=FAH PE=1 SV=2 - [FAAA_HUMAN]	-0.17	-0.21	-0.14	-0.15	-0.36	-0.29	-0.40	-0.33	-0.3	0.00
P01621	Ig kappa chain V-III region NG9 (Fragment) OS=Homo sapiens PE=1 SV=1 - [KV303_HUMAN]	-0.12	-0.16	-0.49	-0.41	-0.28	-0.35	-0.11	-0.13	-0.3	0.00
Q9BYB0	SH3 and multiple ankyrin repeat domains protein 3 OS=Homo sapiens GN=SHANK3 PE=1 SV=2 - [SHAN3_HUMAN]	-0.41	-0.68	-0.13	-0.40	-0.21	0.10	-0.31	-0.01	-0.3	0.02

P17858	6-phosphofructokinase, liver type OS=Homo sapiens GN=PFKL PE=1 SV=6 - [K6PL_HUMAN]	-0.31	-0.19	-0.36	-0.18					-0.3	0.01
P11021	78 kDa glucose-regulated protein OS=Homo sapiens GN=HSPA5 PE=1 SV=2 - [GRP78_HUMAN]	-0.08	-0.20	-0.07	-0.12	-0.42	-0.47	-0.36	-0.37	-0.3	0.00
P05093	Steroid 17-alpha-hydroxylase/17,20 lyase OS=Homo sapiens GN=CYP17A1 PE=1 SV=1 - [CP17A_HUMAN]	-0.30	0.29	-0.59	0.00	-0.39	-0.39	-0.43	-0.33	-0.3	0.03
Q8NBJ4	Golgi membrane protein 1 OS=Homo sapiens GN=GOLM1 PE=1 SV=1 - [GOLM1_HUMAN]	-0.41	-0.36	-0.25	-0.20	-0.01	-0.39	-0.07	-0.45	-0.3	0.00
P04083	Annexin A1 OS=Homo sapiens GN=ANXA1 PE=1 SV=2 - [ANXA1_HUMAN]					-0.27	-0.27	-0.27	-0.26	-0.3	0.00
P30041	Peroxiredoxin-6 OS=Homo sapiens GN=PRDX6 PE=1 SV=3 - [PRDX6_HUMAN]	-0.37	-0.58	-0.34	-0.45	-0.08	-0.10	-0.14	-0.09	-0.3	0.01
P04438	Ig heavy chain V-II region SESS OS=Homo sapiens PE=2 SV=1 - [HV208_HUMAN]	-0.31	-0.37	-0.33	-0.29	-0.26	-0.08	-0.37	-0.17	-0.3	0.00

Q53GS9	U4/U6.U5 tri-snRNP-associated protein 2 OS=Homo sapiens GN=USP39 PE=1 SV=2 - [SNUT2_HUMAN]	-0.37	-0.29	-0.24	-0.16	-0.38	-0.46	-0.11	-0.19	-0.3	0.00
P62873	Guanine nucleotide-binding protein G(I)/G(S)/G(T) subunit beta-1 OS=Homo sapiens GN=GNB1 PE=1 SV=3 - [GBB1_HUMAN]	-0.35	-0.11	-0.35	-0.03	-0.40	-0.27	-0.41	-0.28	-0.3	0.00
O75385	Serine/threonine-protein kinase ULK1 OS=Homo sapiens GN=ULK1 PE=1 SV=2 - [ULK1_HUMAN]	-0.33	-0.14	-0.34	-0.15	-0.25	-0.20	-0.40	-0.35	-0.3	0.00
Q96M20	Uncharacterized protein C20orf152 OS=Homo sapiens GN=C20orf152 PE=2 SV=2 - [CT152_HUMAN]	-0.61	-0.74	-0.17	-0.30	-0.38	-0.18	-0.05	0.21	-0.3	0.04
Q9NYU2	UDP-glucose:glycoprotein glucosyltransferase 1 OS=Homo sapiens GN=UGGT1 PE=1 SV=3 - [UGGG1_HUMAN]	-0.42	-0.31	-0.24	-0.13					-0.3	0.02
O43592	Exportin-T OS=Homo sapiens GN=XPOT PE=1 SV=2 - [XPOT_HUMAN]					-0.23	-0.25	-0.30	-0.33	-0.3	0.00
P37802	Transgelin-2 OS=Homo sapiens GN=TAGLN2 PE=1 SV=3 - [TAGL2_HUMAN]	-0.45	-0.51	-0.47	-0.53	0.01	-0.15	-0.03	-0.12	-0.3	0.01

P26447	Protein S100-A4 OS=Homo sapiens GN=S100A4 PE=1 SV=1 - [S10A4_HUMAN]	-0.41	-0.29	-0.35	-0.21	-0.23	-0.05	-0.42	-0.28	-0.3	0.00
P10644	cAMP-dependent protein kinase type I-alpha regulatory subunit OS=Homo sapiens GN=PRKAR1A PE=1 SV=1 - [KAP0_HUMAN]					-0.14	-0.42	-0.14	-0.42	-0.3	0.04
Q01518	Adenylyl cyclase-associated protein 1 OS=Homo sapiens GN=CAP1 PE=1 SV=5 - [CAP1_HUMAN]	-0.54	-0.28	-0.42	-0.24	-0.17	-0.14	-0.25	-0.22	-0.3	0.00
Q93100	Phosphorylase b kinase regulatory subunit beta OS=Homo sapiens GN=PHKB PE=1 SV=3 - [KPBB_HUMAN]	-0.32	-0.15	-0.31	-0.36					-0.3	0.01
O00592	Podocalyxin OS=Homo sapiens GN=PODXL PE=1 SV=2 - [PODXL_HUMAN]	-0.44	-0.57	-0.51	-0.55	-0.07	-0.14	0.04	-0.03	-0.3	0.02
Q8IWL3	Iron-sulfur cluster co-chaperone protein HscB, mitochondrial OS=Homo sapiens GN=HSCB PE=1 SV=3 - [HSC20_HUMAN]	-0.29	-0.36	-0.21	-0.27					-0.3	0.00

Q9H1A4	Anaphase-promoting complex subunit 1 OS=Homo sapiens GN=ANAPC1 PE=1 SV=1 - [APC1_HUMAN]	-0.59	-0.33	-0.69	-0.38	0.08	0.30	-0.44	-0.23	-0.3	0.05
Q9BX84	Transient receptor potential cation channel subfamily M member 6 OS=Homo sapiens GN=TRPM6 PE=1 SV=2 - [TRPM6_HUMAN]	-0.47	-0.03	-0.32	0.17	-0.07	-0.36	-0.46	-0.74	-0.3	0.03
P51148	Ras-related protein Rab-5C OS=Homo sapiens GN=RAB5C PE=1 SV=2 - [RAB5C_HUMAN]	-0.22	-0.48	-0.10	-0.35					-0.3	0.04
P59910	DnaJ homolog subfamily B member 13 OS=Homo sapiens GN=DNAJB13 PE=2 SV=1 - [DJB13_HUMAN]	-0.47	-0.67	-0.09	-0.27	-0.37	-0.42	0.02	-0.04	-0.3	0.01
P06317	Ig lambda chain V-VI region SUT OS=Homo sapiens PE=1 SV=1 - [LV603_HUMAN]	-0.37	-0.47	-0.43	-0.50	-0.12	-0.23	-0.01	-0.17	-0.3	0.00
Q9P2E9	Ribosome-binding protein 1 OS=Homo sapiens GN=RRBP1 PE=1 SV=4 - [RRBP1_HUMAN]	-0.33	0.04	-0.13	0.25	-0.66	-0.62	-0.46	-0.42	-0.3	0.04

Q5T9S5	Coiled-coil domain-containing protein 18 OS=Homo sapiens GN=CCDC18 PE=1 SV=1 - [CCD18_HUMAN]	-0.23	-0.28	-0.14	-0.17	-0.41	-0.21	-0.54	-0.35	-0.3	0.00
Q9UGM3	Deleted in malignant brain tumors 1 protein OS=Homo sapiens GN=DMBT1 PE=1 SV=2 - [DMBT1_HUMAN]	-0.22	-0.65	-0.46	-0.88	0.15	0.11	-0.19	-0.22	-0.3	0.05
Q6ZWJ8	Kielin/chordin-like protein OS=Homo sapiens GN=KCP PE=2 SV=2 - [KCP_HUMAN]					-0.17	-0.25	-0.34	-0.42	-0.3	0.01
Q03591	Complement factor H-related protein 1 OS=Homo sapiens GN=CFHR1 PE=1 SV=2 - [FHR1_HUMAN]	-0.04	-0.39	-0.14	-0.60	-0.35	-0.26	-0.35	-0.23	-0.3	0.00
Q8IVL5	Prolyl 3-hydroxylase 2 OS=Homo sapiens GN=LEPREL1 PE=1 SV=1 - [P3H2_HUMAN]	-0.27	-0.32	-0.28	-0.32					-0.3	0.00
P49747	Cartilage oligomeric matrix protein OS=Homo sapiens GN=COMP PE=1 SV=2 - [COMP_HUMAN]	-0.30	-0.38	-0.27	-0.33	-0.41	-0.25	-0.32	-0.15	-0.3	0.00

Q99650	Oncostatin-M-specific receptor subunit beta OS=Homo sapiens GN=OSMR PE=1 SV=1 - [OSMR_HUMAN]	-0.33	-0.27	-0.34	-0.26					-0.3	0.00
P57071	PR domain zinc finger protein 15 OS=Homo sapiens GN=PRDM15 PE=2 SV=4 - [PRD15_HUMAN]	-0.39	-0.31	-0.31	-0.22	-0.04		-0.54		-0.3	0.01
P08514	Integrin alpha-IIb OS=Homo sapiens GN=ITGA2B PE=1 SV=3 - [ITA2B_HUMAN]	-0.35	-0.31	-0.25	-0.30					-0.3	0.00
Q9Y6D5	Brefeldin A-inhibited guanine nucleotide-exchange protein 2 OS=Homo sapiens GN=ARFGEF2 PE=1 SV=3 - [BIG2_HUMAN]	-0.32	-0.18	-0.32	-0.39					-0.3	0.01
P10768	S-formylglutathione hydrolase OS=Homo sapiens GN=ESD PE=1 SV=2 - [ESTD_HUMAN]	-0.68	-0.67	-0.63	-0.61	-0.02	0.03	0.05	0.09	-0.3	0.05
Q96N23	Uncharacterized protein C12orf55 OS=Homo sapiens GN=C12orf55 PE=2 SV=2 - [CL055_HUMAN]	-0.33	-0.41	-0.21	-0.27					-0.3	0.01

Q71H61	Immunoglobulin-like domain-containing receptor 2 OS=Homo sapiens GN=ILDR2 PE=2 SV=1 - [ILDR2_HUMAN]					-0.57	-0.30	-0.10	-0.26	-0.3	0.05
P17174	Aspartate aminotransferase, cytoplasmic OS=Homo sapiens GN=GOT1 PE=1 SV=3 - [AATC_HUMAN]	-0.27	-0.31	-0.26	-0.40					-0.3	0.00
Q9HBG6	Intraflagellar transport protein 122 homolog OS=Homo sapiens GN=IFT122 PE=1 SV=2 - [IF122_HUMAN]	-0.22	-0.29	-0.33	-0.40					-0.3	0.00
P13639	Elongation factor 2 OS=Homo sapiens GN=EEF2 PE=1 SV=4 - [EF2_HUMAN]	-0.05	-0.04	0.10	0.12	-0.68	-0.51	-0.80	-0.64	-0.3	0.05
Q9Y4W6	AFG3-like protein 2 OS=Homo sapiens GN=AFG3L2 PE=1 SV=2 - [AFG32_HUMAN]	-0.45	-0.64	-0.36	-0.54	-0.06	-0.03	-0.22	-0.20	-0.3	0.01
P06727	Apolipoprotein A-IV OS=Homo sapiens GN=APOA4 PE=1 SV=3 - [APOA4_HUMAN]	-0.23	-0.31	-0.19	-0.26	-0.35	-0.45	-0.32	-0.42	-0.3	0.00
P50502	Hsc70-interacting protein OS=Homo sapiens GN=ST13 PE=1 SV=2 - [F10A1_HUMAN]	-0.54	-0.58	-0.61	-0.63	-0.08	0.01	-0.05	-0.04	-0.3	0.02

P35542	Serum amyloid A-4 protein OS=Homo sapiens GN=SAA4 PE=1 SV=2 - [SAA4_HUMAN]	-0.45	-0.47	-0.46	-0.48	-0.17	-0.13	-0.19	-0.17	-0.3	0.00
Q96P48	Arf-GAP with Rho-GAP domain, ANK repeat and PH domain-containing protein 1 OS=Homo sapiens GN=ARAP1 PE=1 SV=3 - [ARAP1_HUMAN]	-0.13	-0.17	0.01	-0.03	-0.83	-0.88	-0.22	-0.27	-0.3	0.04
P04040	Catalase OS=Homo sapiens GN=CAT PE=1 SV=3 - [CATA_HUMAN]	-0.32	-0.37	-0.53	-0.58	-0.12	-0.19	-0.15	-0.28	-0.3	0.00
Q96G01	Protein bicaudal D homolog 1 OS=Homo sapiens GN=BICD1 PE=1 SV=3 - [BICD1_HUMAN]					-0.18	-0.12	-0.53	-0.46	-0.3	0.05
Q9UMR3	T-box transcription factor TBX20 OS=Homo sapiens GN=TBX20 PE=1 SV=4 - [TBX20_HUMAN]	-0.24	-0.50	-0.21	-0.46	-0.10	-0.25	-0.34	-0.49	-0.3	0.00
P29279	Connective tissue growth factor OS=Homo sapiens GN=CTGF PE=1 SV=2 - [CTGF_HUMAN]	-0.60	-0.66	-0.57	-0.61	-0.10	-0.08	0.05	-0.03	-0.3	0.02
Q8NEE8	Tetratricopeptide repeat protein 16 OS=Homo sapiens GN=TTC16 PE=2 SV=2 - [TTC16_HUMAN]	-0.39	-0.44	-0.22	-0.26					-0.3	0.01

Q15198	Platelet-derived growth factor receptor-like protein OS=Homo sapiens GN=PDGFRL PE=1 SV=1 - [PGFRL_HUMAN]					-0.19	-0.47	-0.18	-0.48	-0.3	0.03
O43707	Alpha-actinin-4 OS=Homo sapiens GN=ACTN4 PE=1 SV=2 - [ACTN4_HUMAN]	-0.22	-0.44	-0.23	-0.43					-0.3	0.01
Q29RF7	Sister chromatid cohesion protein PDS5 homolog A OS=Homo sapiens GN=PDS5A PE=1 SV=1 - [PDS5A_HUMAN]	-0.71	-0.53	-0.79	-0.60	0.00	-0.29	0.28	-0.01	-0.3	0.05
Q9UKN7	Unconventional myosin-XV OS=Homo sapiens GN=MYO15A PE=1 SV=2 - [MYO15_HUMAN]	0.21	-0.17	-0.21	-0.51	-0.60	-0.66	-0.18	-0.51	-0.3	0.02
Q8NCA9	Zinc finger protein 784 OS=Homo sapiens GN=ZNF784 PE=2 SV=1 - [ZN784_HUMAN]	-0.26	-0.43	-0.24	-0.39					-0.3	0.01
Q86UX7	Fermitin family homolog 3 OS=Homo sapiens GN=FERMT3 PE=1 SV=1 - [URP2_HUMAN]	-0.58	-0.58	-0.56	-0.56	-0.06	0.01	-0.20	-0.12	-0.3	0.01

Q562R1	Beta-actin-like protein 2 OS=Homo sapiens GN=ACTBL2 PE=1 SV=2 - [ACTBL_HUMAN]	-0.49	-0.22	-0.44	-0.18					-0.3	0.02
Q9H3T3	Semaphorin-6B OS=Homo sapiens GN=SEMA6B PE=1 SV=4 - [SEM6B_HUMAN]	-0.23	-0.56	-0.22	-0.53	-0.42	-0.10	-0.48	-0.16	-0.3	0.00
P00918	Carbonic anhydrase 2 OS=Homo sapiens GN=CA2 PE=1 SV=2 - [CAH2_HUMAN]	-0.07	-0.31	-0.21	-0.41	-0.40	-0.38	-0.46	-0.44	-0.3	0.00
Q8TE56	A disintegrin and metalloproteinase with thrombospondin motifs 17 OS=Homo sapiens GN=ADAMTS17 PE=2 SV=2 - [ATS17_HUMAN]	-0.51	-0.26	-0.57	-0.32	-0.15	-0.25	-0.30	-0.35	-0.3	0.00
O75820	Zinc finger protein 189 OS=Homo sapiens GN=ZNF189 PE=2 SV=2 - [ZN189_HUMAN]					-0.47	-0.25	-0.43	-0.21	-0.3	0.01
O60309	Leucine-rich repeat-containing protein 37A3 OS=Homo sapiens GN=LRRC37A3 PE=2 SV=2 - [L37A3_HUMAN]	-0.37	-0.16	-0.25	-0.03		-0.54		-0.70	-0.3	0.02

Q8NFD5	AT-rich interactive domain-containing protein 1B OS=Homo sapiens GN=ARID1B PE=1 SV=2 - [ARI1B_HUMAN]	-0.30	-0.24	-0.45	-0.39					-0.3	0.01
P01604	Ig kappa chain V-I region Kue OS=Homo sapiens PE=1 SV=1 - [KV112_HUMAN]	-0.42	-0.45	-0.39	-0.41	-0.21	-0.22	-0.32	-0.34	-0.3	0.00
O75094	Slit homolog 3 protein OS=Homo sapiens GN=SLIT3 PE=2 SV=3 - [SLIT3_HUMAN]	-0.25	-0.51	-0.15	-0.48					-0.3	0.03
P48145	Neuropeptides B/W receptor type 1 OS=Homo sapiens GN=NPBWR1 PE=1 SV=2 - [NPBW1_HUMAN]					-0.25	-0.24	-0.49	-0.44	-0.4	0.01
Q76G19	PDZ domain-containing protein 4 OS=Homo sapiens GN=PDZD4 PE=2 SV=1 - [PDZD4_HUMAN]					-0.56	-0.22	-0.51	-0.13	-0.4	0.04
Q60I27	ALS2 C-terminal-like protein OS=Homo sapiens GN=ALS2CL PE=1 SV=1 - [AL2CL_HUMAN]					-0.45	-0.59	-0.12	-0.27	-0.4	0.04
P35237	Serpin B6 OS=Homo sapiens GN=SERPINB6 PE=1 SV=3 - [SPB6_HUMAN]					-0.40	-0.25	-0.32	-0.46	-0.4	0.00

Q92616	Translational activator GCN1 OS=Homo sapiens GN=GCN1L1 PE=1 SV=6 - [GCN1L_HUMAN]	-0.05	0.09	-0.18	-0.05	-0.57	-1.10	-0.33	-0.69	-0.4	0.04
Q6UVK1	Chondroitin sulfate proteoglycan 4 OS=Homo sapiens GN=CSPG4 PE=1 SV=2 - [CSPG4_HUMAN]	-0.13	-0.08	-0.19	-0.19	-0.50	-0.70	-0.46	-0.66	-0.4	0.00
Q96RW7	Hemicentin-1 OS=Homo sapiens GN=HMCN1 PE=1 SV=2 - [HMCN1_HUMAN]					-0.35	-0.56	-0.17	-0.38	-0.4	0.02
Q8IVV2	Lipoxygenase homology domain-containing protein 1 OS=Homo sapiens GN=LOXHD1 PE=2 SV=3 - [LOXH1_HUMAN]					-0.28	-0.53	-0.19	-0.45	-0.4	0.02
P02735	Serum amyloid A protein OS=Homo sapiens GN=SAA1 PE=1 SV=2 - [SAA_HUMAN]	-0.16	-0.17	-0.45	-0.38	-0.42	-0.52	-0.36	-0.47	-0.4	0.00
Q9BWH6	RNA polymerase II-associated protein 1 OS=Homo sapiens GN=RPAP1 PE=1 SV=3 - [RPAP1_HUMAN]	-0.72		-0.70		-0.33	-0.06	-0.21	-0.20	-0.4	0.02
Q9NZM1	Myoferlin OS=Homo sapiens GN=MYOF PE=1 SV=1 - [MYOF_HUMAN]					-0.26	-0.34	-0.40	-0.48	-0.4	0.00

P81133	Single-minded homolog 1 OS=Homo sapiens GN=SIM1 PE=2 SV=2 - [SIM1_HUMAN]	-0.46	-0.47	-0.27	-0.28					-0.4	0.01
A6NG73	Putative RNA polymerase II subunit A C-terminal domain phosphatase SSU72-like protein 3 OS=Homo sapiens PE=3 SV=2 - [S72L3_HUMAN]	-0.58	-0.50	-0.47	-0.38	-0.02	-0.37	-0.16	-0.51	-0.4	0.00
Q69YH5	Cell division cycle-associated protein 2 OS=Homo sapiens GN=CDCA2 PE=1 SV=2 - [CDCA2_HUMAN]	-0.26	-0.36	-0.40	-0.48					-0.4	0.00
Q9BUN1	Uncharacterized protein C1orf56 OS=Homo sapiens GN=C1orf56 PE=1 SV=1 - [CA056_HUMAN]	-0.09	-0.19	-0.18	-0.30	-0.71	-0.77	-0.40	-0.36	-0.4	0.00
Q96GX5	Serine/threonine-protein kinase greatwall OS=Homo sapiens GN=MASTL PE=1 SV=1 - [GWL_HUMAN]	-0.35	-0.25	-0.32	-0.20	-0.26	0.08	-1.03	-0.69	-0.4	0.02
Q9Y490	Talin-1 OS=Homo sapiens GN=TLN1 PE=1 SV=3 - [TLN1_HUMAN]	-0.70	-0.82	-0.76	-0.83	0.07	0.03	-0.01	-0.01	-0.4	0.04
Q9P2M7	Cingulin OS=Homo sapiens GN=CGN PE=1 SV=2 - [CING_HUMAN]	-0.43	-0.90	-0.45	-0.91	-0.13	0.00	-0.05	-0.16	-0.4	0.02

Q15057	Arf-GAP with coiled-coil, ANK repeat and PH domain-containing protein 2 OS=Homo sapiens GN=ACAP2 PE=1 SV=3 - [ACAP2_HUMAN]	-0.38	-0.44	-0.59	-0.54	-0.38	-0.17	-0.38	-0.17	-0.4	0.00
A7KAX9	Rho GTPase-activating protein 32 OS=Homo sapiens GN=ARHGAP32 PE=1 SV=1 - [RHG32_HUMAN]	-0.55	-0.24	-0.58	-0.35	-0.13	-0.35	-0.32	-0.51	-0.4	0.00
P02671	Fibrinogen alpha chain OS=Homo sapiens GN=FGA PE=1 SV=2 - [FIBA_HUMAN]	-0.57	-0.60	-0.59	-0.62	-0.21	-0.21	-0.13	-0.12	-0.4	0.00
P42684	Abelson tyrosine-protein kinase 2 OS=Homo sapiens GN=ABL2 PE=1 SV=1 - [ABL2_HUMAN]	-0.21	-0.33	-0.43	-0.55					-0.4	0.01
P35443	Thrombospondin-4 OS=Homo sapiens GN=THBS4 PE=1 SV=2 - [TSP4_HUMAN]	-0.68	-0.77	-0.64	-0.70	-0.15	-0.09	-0.02	-0.04	-0.4	0.01
P78417	Glutathione S-transferase omega-1 OS=Homo sapiens GN=GSTO1 PE=1 SV=2 - [GSTO1_HUMAN]	-0.68	-0.60	-0.65	-0.56	-0.33	-0.12	-0.18	0.02	-0.4	0.00
Q96JI7	Spatacsin OS=Homo sapiens GN=SPG11 PE=1 SV=3 - [SPTCS_HUMAN]	0.23	-0.30	-0.39	-0.13	-0.37	-0.33	-0.92	-0.89	-0.4	0.02

Q9HBI1	Beta-parvin OS=Homo sapiens GN=PARVB PE=1 SV=1 - [PARVB_HUMAN]	-0.60	-0.27	-0.42	-0.27					-0.4	0.02
Q8WXI4	Acyl-coenzyme A thioesterase 11 OS=Homo sapiens GN=ACOT11 PE=1 SV=1 - [ACO11_HUMAN]					-0.61	-0.26	-0.52	-0.18	-0.4	0.03
P31944	Caspase-14 OS=Homo sapiens GN=CASP14 PE=1 SV=2 - [CASPE_HUMAN]	-0.33	-0.45	-0.58	-0.70	-0.30	-0.41	-0.12	-0.25	-0.4	0.00
P35442	Thrombospondin-2 OS=Homo sapiens GN=THBS2 PE=1 SV=2 - [TSP2_HUMAN]	-0.62	-0.72	-0.58	-0.67	-0.24	-0.11	-0.18	-0.05	-0.4	0.01
P23759	Paired box protein Pax-7 OS=Homo sapiens GN=PAX7 PE=2 SV=3 - [PAX7_HUMAN]					-0.33	-0.46	-0.34	-0.46	-0.4	0.00
Q9UIL4	Kinesin-like protein KIF25 OS=Homo sapiens GN=KIF25 PE=2 SV=2 - [KIF25_HUMAN]	-0.50	-0.27	-0.04	0.19	-0.49	-0.70	-0.58	-0.79	-0.4	0.01
Q9UHF7	Zinc finger transcription factor Trps1 OS=Homo sapiens GN=TRPS1 PE=1 SV=2 - [TRPS1_HUMAN]	-0.31	-0.24	-0.47	-0.38	-0.27		-0.75		-0.4	0.00

Q9H579	Uncharacterized protein C20orf132 OS=Homo sapiens GN=C20orf132 PE=2 SV=2 - [CT132_HUMAN]	-0.24	-0.42	-0.44	-0.60	-0.98	-0.03	-0.60	0.11	-0.4	0.01
Q8WUA4	General transcription factor 3C polypeptide 2 OS=Homo sapiens GN=GTF3C2 PE=1 SV=2 - [TF3C2_HUMAN]	-0.52	-0.46	-0.25	-0.18	-0.44	-0.35	-0.55	-0.46	-0.4	0.00
Q01813	6-phosphofructokinase type C OS=Homo sapiens GN=PFKP PE=1 SV=2 - [K6PP_HUMAN]	-0.50	-0.32	-0.42	-0.38					-0.4	0.00
O94779	Contactin-5 OS=Homo sapiens GN=CNTN5 PE=2 SV=2 - [CNTN5_HUMAN]	-0.48	-0.24	-0.56	-0.35					-0.4	0.01
Q15063	Periostin OS=Homo sapiens GN=POSTN PE=1 SV=2 - [POSTN_HUMAN]	-0.21	-0.27	-0.24	-0.26	-0.72	-0.48	-0.65	-0.45	-0.4	0.00
O00154	Cytosolic acyl coenzyme A thioester hydrolase OS=Homo sapiens GN=ACOT7 PE=1 SV=3 - [BACH_HUMAN]	-0.53	-0.54	-0.43	-0.44	-0.25	-0.13	-0.53	-0.41	-0.4	0.00
P04075	Fructose-bisphosphate aldolase A OS=Homo sapiens GN=ALDOA PE=1 SV=2 - [ALDOA_HUMAN]	-0.42	-0.42	-0.36	-0.35	-0.37	-0.36	-0.51	-0.49	-0.4	0.00

Q6ZVZ8	Ankyrin repeat and SOCS box protein 18 OS=Homo sapiens GN=ASB18 PE=2 SV=2 - [ASB18_HUMAN]	-0.49	-0.57	-0.26	-0.33					-0.4	0.01
Q9Y6R7	IgGFc-binding protein OS=Homo sapiens GN=FCGBP PE=1 SV=3 - [FCGBP_HUMAN]	-0.22	-0.24	-0.27	-0.33	-0.70	-0.50	-0.62	-0.43	-0.4	0.00
Q6AZY7	Scavenger receptor class A member 3 OS=Homo sapiens GN=SCARA3 PE=2 SV=1 - [SCAR3_HUMAN]	-0.40	-0.60	-0.31	-0.50			-0.27		-0.4	0.00
P09211	Glutathione S-transferase P OS=Homo sapiens GN=GSTP1 PE=1 SV=2 - [GSTP1_HUMAN]	-0.83	-0.56	-1.09	-0.81	0.03	0.12	-0.12	-0.10	-0.4	0.04
Q8IY18	Structural maintenance of chromosomes protein 5 OS=Homo sapiens GN=SMC5 PE=1 SV=2 - [SMC5_HUMAN]	-0.14	-0.40	-0.45	-0.70					-0.4	0.03
Q3V6T2	Girdin OS=Homo sapiens GN=CCDC88A PE=1 SV=2 - [GRDN_HUMAN]	-0.76	-0.45	-0.34	-0.15					-0.4	0.04
Q9UQP3	Tenascin-N OS=Homo sapiens GN=TNN PE=1 SV=2 - [TENN_HUMAN]	-0.07	-0.20	-0.06	-0.15	-0.87	-0.60	-0.89	-0.61	-0.4	0.01

Q8TED4	Sugar phosphate exchanger 2 OS=Homo sapiens GN=SLC37A2 PE=2 SV=2 - [SPX2_HUMAN]	-0.85	-0.79	0.01	0.08	-0.18	-0.61	-0.34	-0.77	-0.4	0.01
O43660	Pleiotropic regulator 1 OS=Homo sapiens GN=PLRG1 PE=1 SV=1 - [PLRG1_HUMAN]	-0.58	-0.66	-0.21	-0.28					-0.4	0.03
P23528	Cofilin-1 OS=Homo sapiens GN=CFL1 PE=1 SV=3 - [COF1_HUMAN]	-0.41	-0.39	-0.38	-0.42	-0.47	-0.41	-0.50	-0.51	-0.4	0.00
P60709	Actin, cytoplasmic 1 OS=Homo sapiens GN=ACTB PE=1 SV=1 - [ACTB_HUMAN]	-0.61	-0.58	-0.54	-0.51	-0.37	-0.30	-0.36	-0.24	-0.4	0.00
Q3KR16	Pleckstrin homology domain-containing family G member 6 OS=Homo sapiens GN=PLEKHG6 PE=1 SV=3 - [PKHG6_HUMAN]					-0.52	-0.37	-0.51	-0.35	-0.4	0.00
Q9BQN1	Protein FAM83C OS=Homo sapiens GN=FAM83C PE=1 SV=3 - [FA83C_HUMAN]	-0.36	-0.73	-0.10	-0.57					-0.4	0.05

P30153	Serine/threonine-protein phosphatase 2A 65 kDa regulatory subunit A alpha isoform OS=Homo sapiens GN=PPP2R1A PE=1 SV=4 - [2AAA_HUMAN]	-0.49	-0.28	-0.60	-0.40					-0.4	0.01
P51884	Lumican OS=Homo sapiens GN=LUM PE=1 SV=2 - [LUM_HUMAN]	-0.23	-0.27	-0.22	-0.27	-0.61	-0.71	-0.54	-0.67	-0.4	0.00
Q9HCM3	UPF0606 protein KIAA1549 OS=Homo sapiens GN=KIAA1549 PE=1 SV=4 - [K1549_HUMAN]	-0.36	-0.80	-0.47	-0.62	-0.40	-0.21	-0.43	-0.25	-0.4	0.00
Q9NXZ2	Probable ATP-dependent RNA helicase DDX43 OS=Homo sapiens GN=DDX43 PE=1 SV=2 - [DDX43_HUMAN]					-0.45	-0.36	-0.53	-0.44	-0.4	0.00
Q9UI72	Putative uncharacterized protein PRO0255 OS=Homo sapiens GN=PRO0255 PE=5 SV=1 - [YE014_HUMAN]	-0.55	-0.44	-0.45	-0.33					-0.4	0.00
P27105	Erythrocyte band 7 integral membrane protein OS=Homo sapiens GN=STOM PE=1 SV=3 - [STOM_HUMAN]	-0.41	-0.29	-0.61	-0.47					-0.4	0.01

Q96NL6	Sodium channel and clathrin linker 1 OS=Homo sapiens GN=SCLT1 PE=1 SV=2 - [SCLT1_HUMAN]	-0.40	-0.41	-0.49	-0.49					-0.4	0.00
P11413	Glucose-6-phosphate 1-dehydrogenase OS=Homo sapiens GN=G6PD PE=1 SV=4 - [G6PD_HUMAN]	-0.48	-0.29	-0.68	-0.49	-0.61	-0.55	-0.28	-0.22	-0.4	0.00
O00571	ATP-dependent RNA helicase DDX3X OS=Homo sapiens GN=DDX3X PE=1 SV=3 - [DDX3X_HUMAN]	-0.46	-0.48	-0.42	-0.43					-0.5	0.00
Q8NCE2	Myotubularin-related protein 14 OS=Homo sapiens GN=MTMR14 PE=1 SV=2 - [MTMRE_HUMAN]	-0.30	-0.46	-0.45	-0.60					-0.5	0.01
P06733	Alpha-enolase OS=Homo sapiens GN=ENO1 PE=1 SV=2 - [ENOA_HUMAN]	-0.49	-0.70	-0.32	-0.58	-0.34	-0.28	-0.49	-0.43	-0.5	0.00
Q9Y2K6	Ubiquitin carboxyl-terminal hydrolase 20 OS=Homo sapiens GN=USP20 PE=1 SV=2 - [UBP20_HUMAN]	-0.21	-0.58	-0.42	-0.61					-0.5	0.02

P50851	Lipopolysaccharide-responsive and beige-like anchor protein OS=Homo sapiens GN=LRBA PE=1 SV=4 - [LRBA_HUMAN]	-0.39	-0.41	-0.31	-0.33	-0.34	-0.62	-0.48	-0.77	-0.5	0.00
O43422	52 kDa repressor of the inhibitor of the protein kinase OS=Homo sapiens GN=PRKRIR PE=2 SV=2 - [P52K_HUMAN]	-0.50	-0.73	-0.17	-0.45					-0.5	0.03
Q96C34	RUN domain-containing protein 1 OS=Homo sapiens GN=RUNDC1 PE=1 SV=3 - [RUND1_HUMAN]	-0.37	-0.85	-0.60	-1.06	-0.02	-0.40	0.00	-0.39	-0.5	0.01
Q7Z442	Polycystic kidney disease protein 1-like 2 OS=Homo sapiens GN=PKD1L2 PE=1 SV=4 - [PK1L2_HUMAN]	-0.54	-0.57	-0.36	-0.38					-0.5	0.00
Q9UBB9	Tuftelin-interacting protein 11 OS=Homo sapiens GN=TFIP11 PE=1 SV=1 - [TFP11_HUMAN]	-0.37	-0.43	-0.50	-0.56					-0.5	0.00
P00915	Carbonic anhydrase 1 OS=Homo sapiens GN=CA1 PE=1 SV=2 - [CAH1_HUMAN]	-0.36	-0.31	-0.36	-0.32	-0.58	-0.52	-0.68	-0.59	-0.5	0.00

Q6E0U4	Dermokine OS=Homo sapiens GN=DMKN PE=1 SV=3 - [DMKN_HUMAN]	-0.67	-0.31	-0.65	-0.24					-0.5	0.02
Q9NXZ1	Sarcoma antigen 1 OS=Homo sapiens GN=SAGE1 PE=2 SV=2 - [SAGE1_HUMAN]					-0.42	-0.41	-0.53	-0.52	-0.5	0.00
Q15051	IQ calmodulin-binding motif-containing protein 1 OS=Homo sapiens GN=IQCB1 PE=1 SV=1 - [IQCB1_HUMAN]					-0.61	-0.24	-0.67	-0.38	-0.5	0.02
O75096	Low-density lipoprotein receptor-related protein 4 OS=Homo sapiens GN=LRP4 PE=1 SV=4 - [LRP4_HUMAN]	-0.05	-0.89	-0.02	-0.85	-0.99	-0.64	-0.37	-0.03	-0.5	0.01
O60296	Trafficking kinesin-binding protein 2 OS=Homo sapiens GN=TRAK2 PE=1 SV=2 - [TRAK2_HUMAN]	-0.32	-0.10	-0.23	0.01	-0.82	-0.67	-0.92	-0.78	-0.5	0.01
P18206	Vinculin OS=Homo sapiens GN=VCL PE=1 SV=4 - [VINC_HUMAN]	-0.75	-0.76	-0.79	-0.80	-0.17	-0.10	-0.28	-0.22	-0.5	0.00
O60437	Periplakin OS=Homo sapiens GN=PPL PE=1 SV=4 - [PEPL_HUMAN]	-0.61	-0.52	-0.57	-0.47	-0.55	-0.41	-0.44	-0.31	-0.5	0.00
Q86VV8	Rotatin OS=Homo sapiens GN=RTTN PE=1 SV=3 - [RTTN_HUMAN]	-0.50	-0.57	-0.40	-0.47					-0.5	0.00

Q3KQU3	MAP7 domain-containing protein 1 OS=Homo sapiens GN=MAP7D1 PE=1 SV=1 - [MA7D1_HUMAN]	-0.46	-0.39	-0.59	-0.52					-0.5	0.00
Q6YHU6	Thyroid adenoma-associated protein OS=Homo sapiens GN=THADA PE=1 SV=1 - [THADA_HUMAN]	-0.70	-0.34	-0.55	-0.17	-0.56	0.05	-1.13	-0.52	-0.5	0.01
P35609	Alpha-actinin-2 OS=Homo sapiens GN=ACTN2 PE=1 SV=1 - [ACTN2_HUMAN]	-0.47	-0.57	-0.41	-0.52					-0.5	0.00
O76024	Wolframin OS=Homo sapiens GN=WFS1 PE=1 SV=2 - [WFS1_HUMAN]	-0.46	-0.33	-0.51	-0.37	0.08	-0.90	-0.20	-1.25	-0.5	0.01
Q96N46	Tetratricopeptide repeat protein 14 OS=Homo sapiens GN=TTC14 PE=1 SV=1 - [TTC14_HUMAN]	-0.43	-0.75	-0.25	-0.56					-0.5	0.02
P05062	Fructose-bisphosphate aldolase B OS=Homo sapiens GN=ALDOB PE=1 SV=2 - [ALDOB_HUMAN]	-0.47	-0.44	-0.48	-0.51	-0.46	-0.52	-0.53	-0.58	-0.5	0.00
Q6UXT8	Protein FAM150A OS=Homo sapiens GN=FAM150A PE=2 SV=1 - [F150A_HUMAN]					-0.56	-0.85	-0.15	-0.44	-0.5	0.04

P38606	V-type proton ATPase catalytic subunit A OS=Homo sapiens GN=ATP6V1A PE=1 SV=2 - [VATA_HUMAN]	-0.82	-0.72	-0.78	-0.67	0.08	0.02	-0.52	-0.58	-0.5	0.01
Q9HCJ0	Trinucleotide repeat-containing gene 6C protein OS=Homo sapiens GN=TNRC6C PE=1 SV=3 - [TNR6C_HUMAN]	-0.26	-0.80	-0.25	-0.78	-0.62	-0.51	-0.44	-0.34	-0.5	0.00
Q8N5R6	Coiled-coil domain-containing protein 33 OS=Homo sapiens GN=CCDC33 PE=1 SV=3 - [CCD33_HUMAN]	-0.19	-1.42	-0.10	-1.31	-0.10	-0.17	-0.32	-0.39	-0.5	0.03
P63104	14-3-3 protein zeta/delta OS=Homo sapiens GN=YWHAZ PE=1 SV=1 - [1433Z_HUMAN]	-0.82	-0.80	-0.69	-0.66	-0.26	-0.33	-0.21	-0.28	-0.5	0.00
O94898	Leucine-rich repeats and immunoglobulin-like domains protein 2 OS=Homo sapiens GN=LRIG2 PE=1 SV=3 - [LRIG2_HUMAN]	-0.51	-0.44	-0.58	-0.50					-0.5	0.00
Q13228	Selenium-binding protein 1 OS=Homo sapiens GN=SELENBP1 PE=1 SV=2 - [SBP1_HUMAN]	-0.16	-0.39	-0.12	-0.34	-1.03	-0.74	-0.79	-0.50	-0.5	0.00

P08294	Extracellular superoxide dismutase [Cu-Zn] OS=Homo sapiens GN=SOD3 PE=1 SV=2 - [SODE_HUMAN]	-0.68	-0.61	-0.78	-0.70	-0.41	-0.31	-0.36	-0.24	-0.5	0.00
Q86VI3	Ras GTPase-activating-like protein IQGAP3 OS=Homo sapiens GN=IQGAP3 PE=1 SV=2 - [IQGA3_HUMAN]	-0.45	-0.25	-0.41	-0.26	-0.68	-0.52	-0.84	-0.67	-0.5	0.00
P35221	Catenin alpha-1 OS=Homo sapiens GN=CTNNA1 PE=1 SV=1 - [CTNA1_HUMAN]	-0.65	-0.69	-0.39	-0.33					-0.5	0.01
P09467	Fructose-1,6-bisphosphatase 1 OS=Homo sapiens GN=FBP1 PE=1 SV=5 - [F16P1_HUMAN]	-0.57	-0.60	-0.45	-0.48	-0.48	-0.19	-0.83	-0.54	-0.5	0.00
Q9BZD4	Kinetochore protein Nuf2 OS=Homo sapiens GN=NUF2 PE=1 SV=2 - [NUF2_HUMAN]	-0.18	-0.27	-0.56	-0.64	-0.51	-0.91	-0.33	-0.73	-0.5	0.00
Q96J94	Piwi-like protein 1 OS=Homo sapiens GN=PIWIL1 PE=1 SV=1 - [PIWL1_HUMAN]					-0.44	-0.39	-0.63	-0.60	-0.5	0.00
O75467	Zinc finger protein 324A OS=Homo sapiens GN=ZNF324 PE=1 SV=1 - [Z324A_HUMAN]	-0.60	-0.36	-0.61	-0.35	-0.14	-0.48	-0.63	-0.98	-0.5	0.00

Q92542	Nicastrin OS=Homo sapiens GN=NCSTN PE=1 SV=2 - [NICA_HUMAN]	-0.60	-0.52	-0.47	-0.37	-0.70	-0.89	-0.22	-0.41	-0.5	0.00
A7E2V4	Zinc finger SWIM domain-containing protein KIAA0913 OS=Homo sapiens GN=KIAA0913 PE=1 SV=1 - [K0913_HUMAN]					-0.53	-0.57	-0.63	-0.37	-0.5	0.00
P35348	Alpha-1A adrenergic receptor OS=Homo sapiens GN=ADRA1A PE=2 SV=2 - [ADA1A_HUMAN]					-0.66	-0.73	-0.33	-0.40	-0.5	0.01
Q9Y263	Phospholipase A-2-activating protein OS=Homo sapiens GN=PLAA PE=1 SV=2 - [PLAP_HUMAN]					-0.48	-0.67	-0.41	-0.60	-0.5	0.00
P35813	Protein phosphatase 1A OS=Homo sapiens GN=PPM1A PE=1 SV=1 - [PPM1A_HUMAN]	-0.54	-0.52	-0.56	-0.53	-0.55				-0.5	0.00
Q86VS8	Protein Hook homolog 3 OS=Homo sapiens GN=HOOK3 PE=1 SV=2 - [HOOK3_HUMAN]	-0.31	-0.68	-0.40	-0.77					-0.5	0.02
Q6PGQ7	Protein aurora borealis OS=Homo sapiens GN=BORA PE=1 SV=2 - [BORA_HUMAN]					-0.54	-0.55	-0.53	-0.55	-0.5	0.00

P24043	Laminin subunit alpha-2 OS=Homo sapiens GN=LAMA2 PE=1 SV=4 - [LAMA2_HUMAN]	-0.20	-0.23	-0.42	-0.47	-1.01	-0.69	-0.81	-0.49	-0.5	0.00
Q86VE3	Spermidine/spermine N(1)-acetyltransferase-like protein 1 OS=Homo sapiens GN=SATL1 PE=2 SV=3 - [SATL1_HUMAN]	-0.56	-0.60	-0.49	-0.52					-0.5	0.00
P30101	Protein disulfide-isomerase A3 OS=Homo sapiens GN=PDIA3 PE=1 SV=4 - [PDIA3_HUMAN]	-0.75	-0.78	-0.82	-0.84	-0.28	-0.28	-0.31	-0.31	-0.5	0.00
Q6P6B7	Ankyrin repeat domain-containing protein 16 OS=Homo sapiens GN=ANKRD16 PE=2 SV=1 - [ANR16_HUMAN]	-0.21	-0.33	-0.16	-0.26	-0.87	-1.04	-0.89	-0.62	-0.5	0.00
P04179	Superoxide dismutase [Mn], mitochondrial OS=Homo sapiens GN=SOD2 PE=1 SV=2 - [SODM_HUMAN]	-0.77	-0.79	-0.88	-0.89	-0.33	-0.29	-0.26	-0.22	-0.6	0.00
P51956	Serine/threonine-protein kinase Nek3 OS=Homo sapiens GN=NEK3 PE=1 SV=2 - [NEK3_HUMAN]	-1.22	-1.33	-0.59	-0.69	-0.19	-0.12	-0.18	-0.11	-0.6	0.02

Q92736	Ryanodine receptor 2 OS=Homo sapiens GN=RYR2 PE=1 SV=3 - [RYR2_HUMAN]	-0.01	-0.19	-0.29	-0.47	-1.20	-1.85	0.11	-0.54	-0.6	0.05
Q4G0X9	Coiled-coil domain-containing protein 40 OS=Homo sapiens GN=CCDC40 PE=2 SV=2 - [CCD40_HUMAN]	-0.25	-0.27	-0.35	-0.36	-0.62	-1.19	-0.42	-1.00	-0.6	0.00
P02042	Hemoglobin subunit delta OS=Homo sapiens GN=HBD PE=1 SV=2 - [HBD_HUMAN]	-0.83	-0.81	-0.93	-0.94	-0.24	-0.26	-0.21	-0.25	-0.6	0.00
Q5T601	Probable G-protein coupled receptor 110 OS=Homo sapiens GN=GPR110 PE=2 SV=2 - [GP110_HUMAN]	-0.46	-0.48	-0.49	-0.50		-0.87			-0.6	0.00
Q96PX9	Pleckstrin homology domain-containing family G member 4B OS=Homo sapiens GN=PLEKHG4B PE=2 SV=4 - [PKH4B_HUMAN]	-0.58	-0.62	-0.66	-0.70	-0.89	-0.45	-0.51	-0.08	-0.6	0.00
Q9BTW9	Tubulin-specific chaperone D OS=Homo sapiens GN=TBCD PE=1 SV=2 - [TBCD_HUMAN]					-0.54	-0.44	-0.68	-0.58	-0.6	0.00

P02549	Spectrin alpha chain, erythrocyte OS=Homo sapiens GN=SPTA1 PE=1 SV=5 - [SPTA1_HUMAN]					-0.59	-0.57	-0.54	-0.54	-0.6	0.00
P49368	T-complex protein 1 subunit gamma OS=Homo sapiens GN=CCT3 PE=1 SV=4 - [TCPG_HUMAN]					-0.40	-0.66	-0.47	-0.73	-0.6	0.01
Q8NGC6	Olfactory receptor 4K17 OS=Homo sapiens GN=OR4K17 PE=2 SV=3 - [OR4KH_HUMAN]					-0.20	-0.55	-0.59	-0.92	-0.6	0.03
Q5JU85	IQ motif and SEC7 domain-containing protein 2 OS=Homo sapiens GN=IQSEC2 PE=1 SV=1 - [IQEC2_HUMAN]					-0.76	-0.61	-0.56	-0.34	-0.6	0.01
P10809	60 kDa heat shock protein, mitochondrial OS=Homo sapiens GN=HSPD1 PE=1 SV=2 - [CH60_HUMAN]					-0.74	-0.55	-0.60	-0.41	-0.6	0.00
Q7Z406	Myosin-14 OS=Homo sapiens GN=MYH14 PE=1 SV=2 - [MYH14_HUMAN]	-0.86	-1.00	-0.76	-0.89	-0.15	-0.32	-0.22	-0.40	-0.6	0.00
Q9P270	SLAIN motif-containing protein 2 OS=Homo sapiens GN=SLAIN2 PE=1 SV=2 - [SLAI2_HUMAN]	-0.57	-1.47	-0.49	-1.37	-0.02	-0.15	-0.20	-0.33	-0.6	0.02

Q6UXB2	VEGF co-regulated chemokine 1 OS=Homo sapiens GN=CXCL17 PE=1 SV=1 - [VCC1_HUMAN]	-0.65	-0.47	-0.70	-0.51					-0.6	0.00
Q2PPJ7	Ral GTPase-activating protein subunit alpha-2 OS=Homo sapiens GN=RALGAPA2 PE=1 SV=2 - [RGPA2_HUMAN]	-0.68	-0.78	-0.67	-0.75	-0.30	-0.64	-0.16	-0.67	-0.6	0.00
P49327	Fatty acid synthase OS=Homo sapiens GN=FASN PE=1 SV=3 - [FAS_HUMAN]	0.04	0.07	-0.70	-0.66	-0.93	-0.95	-0.77	-0.78	-0.6	0.00
P51826	AF4/FMR2 family member 3 OS=Homo sapiens GN=AFF3 PE=1 SV=2 - [AFF3_HUMAN]					-0.61	-0.67	-0.50	-0.58	-0.6	0.00
P22314	Ubiquitin-like modifier-activating enzyme 1 OS=Homo sapiens GN=UBA1 PE=1 SV=3 - [UBA1_HUMAN]	-0.54	-0.62	-0.50	-0.71					-0.6	0.00
Q96GC6	Neurotrophin receptor-interacting factor homolog OS=Homo sapiens GN=ZNF274 PE=1 SV=2 - [ZN274_HUMAN]	-0.37	-0.68	-0.52	-0.82					-0.6	0.01

Q96JC1	Vam6/Vps39-like protein OS=Homo sapiens GN=VPS39 PE=1 SV=2 - [VPS39_HUMAN]					-0.61	-0.49	-0.62	-0.67	-0.6	0.00
Q9UP83	Conserved oligomeric Golgi complex subunit 5 OS=Homo sapiens GN=COG5 PE=1 SV=3 - [COG5_HUMAN]	-0.53	-1.02	-0.36	-0.49					-0.6	0.03
P32119	Peroxiredoxin-2 OS=Homo sapiens GN=PRDX2 PE=1 SV=5 - [PRDX2_HUMAN]	-0.60	-0.70	-0.70	-0.76	-0.49	-0.43	-0.66	-0.61	-0.6	0.00
P55072	Transitional endoplasmic reticulum ATPase OS=Homo sapiens GN=VCP PE=1 SV=4 - [TERA_HUMAN]	-0.54	-0.53	-0.58	-0.62	-0.93	-1.05	-0.31	-0.42	-0.6	0.00
P68366	Tubulin alpha-4A chain OS=Homo sapiens GN=TUBA4A PE=1 SV=1 - [TBA4A_HUMAN]	-0.76	-1.06	-1.31	-1.26	-0.22	-0.08	-0.23	-0.09	-0.6	0.01
Q5D862	Filaggrin-2 OS=Homo sapiens GN=FLG2 PE=1 SV=1 - [FILA2_HUMAN]	-0.48	-0.31	-0.99	-0.76					-0.6	0.02
B2RTY4	Unconventional myosin-IXa OS=Homo sapiens GN=MYO9A PE=1 SV=2 - [MYO9A_HUMAN]	-1.29	-1.28			-0.32	-0.28	-0.33	-0.30	-0.6	0.03

Q9C0C2	182 kDa tankyrase-1-binding protein OS=Homo sapiens GN=TNKS1BP1 PE=1 SV=4 - [TB182_HUMAN]	-0.13	-0.72	-0.24	-0.74	-0.85	-0.82	-0.80	-0.78	-0.6	0.00
Q8TDZ2	Protein-methionine sulfoxide oxidase MICAL1 OS=Homo sapiens GN=MICAL1 PE=1 SV=2 - [MICA1_HUMAN]	-0.84	-0.84	-1.14	-1.14	-0.22	-0.46	-0.12	-0.36	-0.6	0.00
P69905	Hemoglobin subunit alpha OS=Homo sapiens GN=HBA1 PE=1 SV=2 - [HBA_HUMAN]	-0.65	-0.79	-0.78	-0.82	-0.50	-0.51	-0.55	-0.54	-0.6	0.00
O94964	Uncharacterized protein KIAA0889 OS=Homo sapiens GN=KIAA0889 PE=1 SV=2 - [K0889_HUMAN]			-0.15	-1.61	-0.67	-0.60	-0.45	-0.38	-0.6	0.03
O15143	Actin-related protein 2/3 complex subunit 1B OS=Homo sapiens GN=ARPC1B PE=1 SV=3 - [ARC1B_HUMAN]	-0.50	-0.78	-0.52	-0.78					-0.6	0.00
Q5TCY1	Tau-tubulin kinase 1 OS=Homo sapiens GN=TTBK1 PE=1 SV=2 - [TTBK1_HUMAN]	-0.72	-0.81	-0.48	-0.57					-0.6	0.00

Q99704	Docking protein 1 OS=Homo sapiens GN=DOK1 PE=1 SV=1 - [DOK1_HUMAN]	-0.31	-0.22	-0.51	-0.41	-0.49	-1.19	-0.67	-1.38	-0.6	0.00
Q9UIV8	Serpin B13 OS=Homo sapiens GN=SERPINB13 PE=2 SV=2 - [SPB13_HUMAN]	-0.75	-0.71	-0.89	-0.85	-0.68	-0.21	-0.79	-0.33	-0.6	0.00
Q16853	Membrane primary amine oxidase OS=Homo sapiens GN=AOC3 PE=1 SV=3 - [AOC3_HUMAN]	-0.77	-0.69	-0.58	-0.56					-0.7	0.00
Q99814	Endothelial PAS domain-containing protein 1 OS=Homo sapiens GN=EPAS1 PE=1 SV=3 - [EPAS1_HUMAN]	-0.89	-0.65	-0.72	-0.41					-0.7	0.01
Q9UPS8	Ankyrin repeat domain-containing protein 26 OS=Homo sapiens GN=ANKRD26 PE=1 SV=3 - [ANR26_HUMAN]	-0.72	-1.60	-0.03	-0.90	-0.58	-0.90	-0.17	-0.50	-0.7	0.01
Q96DC7	Transmembrane and coiled-coil domain-containing protein 6 OS=Homo sapiens GN=TMCO6 PE=2 SV=2 - [TMCO6_HUMAN]	-1.14	-0.83	-1.32	-0.99	-0.23	-0.25	-0.31	-0.32	-0.7	0.00

P13716	Delta-aminolevulinic acid dehydratase OS=Homo sapiens GN=ALAD PE=1 SV=1 - [HEM2_HUMAN]	-0.77	-0.55	-0.81	-0.58					-0.7	0.00
P30043	Flavin reductase (NADPH) OS=Homo sapiens GN=BLVRB PE=1 SV=3 - [BLVRB_HUMAN]	-0.71	-0.75	-0.64	-0.64	-0.73	-0.67	-0.69	-0.61	-0.7	0.00
Q13077	TNF receptor-associated factor 1 OS=Homo sapiens GN=TRAF1 PE=1 SV=1 - [TRAF1_HUMAN]					-0.47	-0.37	-0.99	-0.89	-0.7	0.02
Q9H4B7	Tubulin beta-1 chain OS=Homo sapiens GN=TUBB1 PE=1 SV=1 - [TBB1_HUMAN]	-1.38	-1.38	-1.30	-1.29	-0.21	-0.02	0.06	0.02	-0.7	0.03
Q15942	Zyxin OS=Homo sapiens GN=ZYX PE=1 SV=1 - [ZYX_HUMAN]	-1.34	-1.59	-1.05	-1.36	0.08	0.04	-0.14	-0.17	-0.7	0.03
Q9UL58	Zinc finger protein 215 OS=Homo sapiens GN=ZNF215 PE=2 SV=2 - [ZN215_HUMAN]	-0.41	-0.82	-0.45	-0.78	-1.64	-0.83	-0.71	0.10	-0.7	0.01
Q9UMN6	Histone-lysine N-methyltransferase MLL4 OS=Homo sapiens GN=WBP7 PE=1 SV=1 - [MLL4_HUMAN]	-1.43	-1.08	-1.27	-0.90	-0.46	-0.15	-0.28	0.02	-0.7	0.01

Q8N8E3	Centrosomal protein of 112 kDa OS=Homo sapiens GN=CEP112 PE=1 SV=2 - [CE112_HUMAN]	-1.24	-2.01	-0.37	-1.14	-0.05	-0.51	0.11	-0.35	-0.7	0.03
Q96RR1	Twinkle protein, mitochondrial OS=Homo sapiens GN=PEO1 PE=1 SV=1 - [PEO1_HUMAN]					-0.96	-1.11	-0.28	-0.43	-0.7	0.04
Q9HC98	Serine/threonine-protein kinase Nek6 OS=Homo sapiens GN=NEK6 PE=1 SV=2 - [NEK6_HUMAN]	-0.51	-1.12	-0.25	-0.91					-0.7	0.04
Q06418	Tyrosine-protein kinase receptor TYRO3 OS=Homo sapiens GN=TYRO3 PE=1 SV=1 - [TYRO3_HUMAN]	-0.61	-0.77	-0.50	-0.65	-0.92	-0.64	-0.94	-0.67	-0.7	0.00
P68871	Hemoglobin subunit beta OS=Homo sapiens GN=HBB PE=1 SV=2 - [HBB_HUMAN]	-0.80	-0.88	-0.96	-1.05	-0.50	-0.46	-0.54	-0.54	-0.7	0.00
Q13563	Polycystin-2 OS=Homo sapiens GN=PKD2 PE=1 SV=3 - [PKD2_HUMAN]	-0.81	-0.46	-0.40	-0.05	-0.74	-1.28	-0.61	-1.39	-0.7	0.00
Q5T9L3	Protein wntless homolog OS=Homo sapiens GN=WLS PE=1 SV=2 - [WLS_HUMAN]	-0.77	-1.39	-1.04	-1.65	-0.14	-0.21	-0.29	-0.36	-0.7	0.01

Q9UI08	Ena/VASP-like protein OS=Homo sapiens GN=EVL PE=1 SV=2 - [EVL_HUMAN]					-0.54	-1.06	-0.27	-1.07	-0.7	0.03
Q96T58	Msx2-interacting protein OS=Homo sapiens GN=SPEN PE=1 SV=1 - [MINT_HUMAN]	-0.48	-0.73	-0.52	-0.75	-0.72	-1.24	-0.48	-1.00	-0.7	0.00
Q3ZCM7	Tubulin beta-8 chain OS=Homo sapiens GN=TUBB8 PE=1 SV=2 - [TBB8_HUMAN]	-0.70	-0.62	-0.89	-0.78					-0.7	0.00
Q01970	1-phosphatidylinositol 4,5-bisphosphate phosphodiesterase beta-3 OS=Homo sapiens GN=PLCB3 PE=1 SV=2 - [PLCB3_HUMAN]	-1.43	-1.54	-1.20	-1.31	-0.16	-0.08	-0.20	-0.12	-0.8	0.01
Q15404	Ras suppressor protein 1 OS=Homo sapiens GN=RSU1 PE=1 SV=3 - [RSU1_HUMAN]	-0.74	-0.89	-0.68	-0.73					-0.8	0.00
P52907	F-actin-capping protein subunit alpha-1 OS=Homo sapiens GN=CAPZA1 PE=1 SV=3 - [CAZA1_HUMAN]	-0.64	-0.82	-0.72	-0.88					-0.8	0.00

Q15652	Probable JmjC domain-containing histone demethylation protein 2C OS=Homo sapiens GN=JMJD1C PE=1 SV=2 - [JHD2C_HUMAN]	-0.87	-0.89	-0.68	-0.63					-0.8	0.00
Q8N6C8	Leukocyte immunoglobulin-like receptor subfamily A member 3 OS=Homo sapiens GN=LILRA3 PE=1 SV=3 - [LIRA3_HUMAN]	-0.28	-0.39	-0.29	-0.36	-1.23	-1.14	-1.27	-1.18	-0.8	0.00
Q6P2E9	Enhancer of mRNA-decapping protein 4 OS=Homo sapiens GN=EDC4 PE=1 SV=1 - [EDC4_HUMAN]	-1.00	-0.81	-1.04	-0.85	-1.08	-0.10	-1.12	-0.14	-0.8	0.00
Q13263	Transcription intermediary factor 1-beta OS=Homo sapiens GN=TRIM28 PE=1 SV=5 - [TIF1B_HUMAN]	-0.77	-0.92	-0.60	-0.93					-0.8	0.00
P04406	Glyceraldehyde-3-phosphate dehydrogenase OS=Homo sapiens GN=GAPDH PE=1 SV=3 - [G3P_HUMAN]	-1.21	-1.21	-1.15	-1.20	-0.53	-0.38	-0.48	-0.35	-0.8	0.00

Q9HCX3	Zinc finger protein 304 OS=Homo sapiens GN=ZNF304 PE=2 SV=2 - [ZN304_HUMAN]					-0.43	-1.32	-0.31	-1.21	-0.8	0.05
Q9P273	Teneurin-3 OS=Homo sapiens GN=ODZ3 PE=2 SV=3 - [TEN3_HUMAN]					-0.32	-1.09	-0.80	-1.23	-0.9	0.02
Q8NI35	InaD-like protein OS=Homo sapiens GN=INADL PE=1 SV=3 - [INADL_HUMAN]	-0.60	-0.56	-0.63	-0.59	-1.12	-1.20	-1.09	-1.17	-0.9	0.00
O75691	Small subunit processome component 20 homolog OS=Homo sapiens GN=UTP20 PE=1 SV=3 - [UTP20_HUMAN]	-0.79	-1.10	-0.66	-0.97					-0.9	0.00
Q7Z3V4	Ubiquitin-protein ligase E3B OS=Homo sapiens GN=UBE3B PE=1 SV=3 - [UBE3B_HUMAN]	-0.39	-0.55	-1.71	-1.85	-0.67	-0.49	-0.79	-0.61	-0.9	0.00
Q6ZN57	Zinc finger protein 2 homolog OS=Homo sapiens GN=ZFP2 PE=2 SV=1 - [ZFP2_HUMAN]	-0.52	-0.86	-0.93	-1.26					-0.9	0.01
Q86XP0	Cytosolic phospholipase A2 delta OS=Homo sapiens GN=PLA2G4D PE=2 SV=2 - [PA24D_HUMAN]		-0.88		-0.45	-0.20	-1.53	-0.51	-1.84	-0.9	0.02

Q9BYE9	Cadherin-related family member 2 OS=Homo sapiens GN=CDHR2 PE=1 SV=2 - [CDHR2_HUMAN]	-0.86	-0.92	-0.90	-0.95					-0.9	0.00
Q13576	Ras GTPase-activating-like protein IQGAP2 OS=Homo sapiens GN=IQGAP2 PE=1 SV=4 - [IQGA2_HUMAN]	-1.35	-1.37	-0.98	-1.00	-1.11	-1.32	0.07	-0.37	-0.9	0.00
P46013	Antigen KI-67 OS=Homo sapiens GN=MKI67 PE=1 SV=2 - [KI67_HUMAN]	-2.20	-1.78	-1.72	-1.38	-0.28	-0.49	0.32	0.10	-0.9	0.03
Q8N587	Zinc finger protein 561 OS=Homo sapiens GN=ZNF561 PE=2 SV=2 - [ZN561_HUMAN]					-1.57	-1.36	-0.57	-0.36	-1.0	0.05
Q02108	Guanylate cyclase soluble subunit alpha-3 OS=Homo sapiens GN=GUCY1A3 PE=1 SV=2 - [GCYA3_HUMAN]	-1.75	-1.66	-1.57	-1.47	-0.38	-0.43	-0.22	-0.27	-1.0	0.01
P24844	Myosin regulatory light polypeptide 9 OS=Homo sapiens GN=MYL9 PE=1 SV=4 - [MYL9_HUMAN]	-1.35	-1.09	-0.65	-0.64	-1.20	-1.25	-1.05	-0.92	-1.0	0.00

P06730	Eukaryotic translation initiation factor 4E OS=Homo sapiens GN=EIF4E PE=1 SV=2 - [IF4E_HUMAN]					-1.01	-0.92	-1.12	-1.03	-1.0	0.00
Q96JA4	Membrane-spanning 4-domains subfamily A member 14 OS=Homo sapiens GN=MS4A14 PE=2 SV=2 - [M4A14_HUMAN]	-1.13	-1.62	-0.67	-1.14	-0.67	-1.20	-0.70	-1.23	-1.0	0.00
P02538	Keratin, type II cytoskeletal 6A OS=Homo sapiens GN=KRT6A PE=1 SV=3 - [K2C6A_HUMAN]	-0.54	-1.00	-1.09	-1.56					-1.0	0.02
Q02156	Protein kinase C epsilon type OS=Homo sapiens GN=PRKCE PE=1 SV=1 - [KPCE_HUMAN]					-1.08		-1.04		-1.1	0.01
Q6ZVD7	Storkhead-box protein 1 OS=Homo sapiens GN=STOX1 PE=1 SV=2 - [STOX1_HUMAN]	-1.64	-1.62	-0.79	-0.77	-0.66	-2.00	0.16	-1.18	-1.1	0.00
Q9H9C1	Spermatogenesis-defective protein 39 homolog OS=Homo sapiens GN=SPE39 PE=1 SV=1 - [SPE39_HUMAN]	-0.85	-0.60	-0.75	-0.50	-1.96	-2.67	-0.25	-0.97	-1.1	0.01

Q96QB1	Rho GTPase-activating protein 7 OS=Homo sapiens GN=DLC1 PE=1 SV=4 - [RHG07_HUMAN]	-0.37	-0.82	-1.12	-1.97					-1.1	0.05
Q96AX9	E3 ubiquitin-protein ligase MIB2 OS=Homo sapiens GN=MIB2 PE=1 SV=3 - [MIB2_HUMAN]					-1.40	-1.68	-0.48	-0.76	-1.1	0.03
Q86YA3	Uncharacterized protein C4orf21 OS=Homo sapiens GN=C4orf21 PE=1 SV=2 - [CD021_HUMAN]	-0.21	-0.95	-0.24	-0.97	-1.67	-1.44	-1.72	-1.49	-1.1	0.00
P60660	Myosin light polypeptide 6 OS=Homo sapiens GN=MYL6 PE=1 SV=2 - [MYL6_HUMAN]	-0.90	-1.02	-0.98	-1.10	-1.17	-1.31	-1.07	-1.19	-1.1	0.00
Q03112	MDS1 and EVI1 complex locus protein EVI1 OS=Homo sapiens GN=MECOM PE=1 SV=2 - [EVI1_HUMAN]	-1.41	-2.23	-1.31	-1.75	-0.27	-0.53	-0.51	-0.77	-1.1	0.00
P62158	Calmodulin OS=Homo sapiens GN=CALM1 PE=1 SV=2 - [CALM_HUMAN]	-0.81	-1.03	-0.81	-1.01	-1.93	-0.81	-1.76	-0.64	-1.1	0.00
Q13702	43 kDa receptor-associated protein of the synapse OS=Homo sapiens GN=RAPSN PE=1 SV=4 - [RAPSN_HUMAN]	-2.34	-2.74	-1.49	-1.88	-0.46	0.25	-0.43	0.28	-1.1	0.03

Q86VH5	Leucine-rich repeat transmembrane neuronal protein 3 OS=Homo sapiens GN=LRRTM3 PE=2 SV=2 - [LRRT3_HUMAN]	-1.03	-0.92	-1.29	-1.18					-1.1	0.00
P78312	Protein FAM193A OS=Homo sapiens GN=FAM193A PE=1 SV=2 - [F193A_HUMAN]	-1.23	-1.46	-0.91	-0.83					-1.1	0.00
Q8N5H7	SH2 domain-containing protein 3C OS=Homo sapiens GN=SH2D3C PE=1 SV=1 - [SH2D3_HUMAN]	-0.84	-1.21	-1.04	-1.41					-1.1	0.00
Q5T7B8	Kinesin-like protein KIF24 OS=Homo sapiens GN=KIF24 PE=1 SV=2 - [KIF24_HUMAN]					-0.44	-1.63	-0.65	-1.84	-1.1	0.05
Q6WKZ4	Rab11 family-interacting protein 1 OS=Homo sapiens GN=RAB11FIP1 PE=1 SV=2 - [RFIP1_HUMAN]	-1.42	-1.50	-0.78	-0.86					-1.1	0.01
P52630	Signal transducer and activator of transcription 2 OS=Homo sapiens GN=STAT2 PE=1 SV=1 - [STAT2_HUMAN]	-0.82	-1.57	-0.97	-1.22					-1.1	0.01

O60245	Protocadherin-7 OS=Homo sapiens GN=PCDH7 PE=1 SV=2 - [PCDH7_HUMAN]	-0.63	-1.78	-0.53	-1.67					-1.2	0.04
Q96RP7	Galactose-3-O-sulfotransferase 4 OS=Homo sapiens GN=GAL3ST4 PE=1 SV=1 - [G3ST4_HUMAN]	-0.77	-2.57	-0.65	-2.44	-0.82	-0.24	-1.27	-0.70	-1.2	0.01
Q5R3F8	Protein phosphatase 1 regulatory subunit 29 OS=Homo sapiens GN=ELFN2 PE=1 SV=1 - [PPR29_HUMAN]	-0.98	-0.93	-1.06	-1.02			-2.95	-0.25	-1.2	0.02
Q9Y2T1	Axin-2 OS=Homo sapiens GN=AXIN2 PE=1 SV=1 - [AXIN2_HUMAN]	-1.47	-1.60	-0.80	-0.93					-1.2	0.01
Q12802	A-kinase anchor protein 13 OS=Homo sapiens GN=AKAP13 PE=1 SV=2 - [AKP13_HUMAN]	-2.25	-3.08	-1.87	-2.78	0.17	-0.24	0.31	0.03	-1.2	0.05
Q76NI1	Protein very KIND OS=Homo sapiens GN=KNDC1 PE=2 SV=2 - [VKIND_HUMAN]	-0.21	-0.49	0.08	-0.18	-2.94	-3.05	-1.42	-1.52	-1.2	0.03
Q86W24	NACHT, LRR and PYD domains-containing protein 14 OS=Homo sapiens GN=NLRP14 PE=2 SV=1 - [NAL14_HUMAN]	-0.46	-0.34	-0.34	-0.21	-3.78	-3.23	-0.72	-0.75	-1.2	0.04

A6NHN0	Otolin-1 OS=Homo sapiens GN=OTOL1 PE=3 SV=1 - [OTOL1_HUMAN]	-2.20	-2.34	-2.25	-2.37	-0.21	-0.13	-0.24	-0.17	-1.2	0.02
Q9Y4B5	Coiled-coil domain-containing protein 165 OS=Homo sapiens GN=CCDC165 PE=1 SV=5 - [CC165_HUMAN]	-0.95	-0.66	-2.25	-1.96	-1.16	-1.13	-1.12	-1.09	-1.3	0.00
Q9C0I1	Myotubularin-related protein 12 OS=Homo sapiens GN=MTMR12 PE=1 SV=2 - [MTMRC_HUMAN]	-0.30	-0.24	-0.41	-0.33	-2.43	-2.36	-2.26	-2.18	-1.3	0.01
P00352	Retinal dehydrogenase 1 OS=Homo sapiens GN=ALDH1A1 PE=1 SV=2 - [AL1A1_HUMAN]	-1.52	-1.26	-1.43	-1.15					-1.3	0.00
P02679	Fibrinogen gamma chain OS=Homo sapiens GN=FGG PE=1 SV=3 - [FIBG_HUMAN]	-0.67	-0.81	-0.64	-0.82	-1.92	-1.86	-2.11	-2.03	-1.4	0.00
P78527	DNA-dependent protein kinase catalytic subunit OS=Homo sapiens GN=PRKDC PE=1 SV=3 - [PRKDC_HUMAN]					-1.37	-1.20	-1.72	-1.54	-1.5	0.00

O75310	UDP-glucuronosyltransferase 2B11 OS=Homo sapiens GN=UGT2B11 PE=2 SV=1 - [UDB11_HUMAN]	-0.40	-0.16	-0.21	0.02	-2.79	-2.73	-2.81	-2.74	-1.5	0.02
O75897	Sulfotransferase 1C4 OS=Homo sapiens GN=SULT1C4 PE=1 SV=2 - [ST1C4_HUMAN]	-1.87	-1.92	-1.61	-1.64	-0.93	-1.48	-0.91	-1.46	-1.5	0.00
O43196	MutS protein homolog 5 OS=Homo sapiens GN=MSH5 PE=1 SV=1 - [MSH5_HUMAN]					-1.72	-2.35	-0.69	-1.31	-1.5	0.02
Q6WRI0	Immunoglobulin superfamily member 10 OS=Homo sapiens GN=IGSF10 PE=1 SV=1 - [IGS10_HUMAN]	-1.73	-1.61	-1.51	-1.38					-1.6	0.00
P02675	Fibrinogen beta chain OS=Homo sapiens GN=FGB PE=1 SV=2 - [FIBB_HUMAN]	-1.65	-1.75	-1.72	-1.90	-1.65	-1.48	-1.56	-1.41	-1.6	0.00
P05141	ADP/ATP translocase 2 OS=Homo sapiens GN=SLC25A5 PE=1 SV=7 - [ADT2_HUMAN]	-1.82	-1.55	-1.78	-1.51					-1.7	0.00
P56377	AP-1 complex subunit sigma 2 OS=Homo sapiens GN=AP1S2 PE=1 SV=1 - [AP1S2_HUMAN]					-1.60	-1.42	-1.97	-1.79	-1.7	0.00

Q05086	Ubiquitin-protein ligase E3A OS=Homo sapiens GN=UBE3A PE=1 SV=4 - [UBE3A_HUMAN]	-0.49	-1.20	-0.24	-0.93	-3.21	-3.05	-3.06	-2.91	-1.9	0.00
Q9H8Y5	Ankyrin repeat and zinc finger domain-containing protein 1 OS=Homo sapiens GN=ANKZF1 PE=1 SV=1 - [ANKZ1_HUMAN]	-2.29	-2.13	-1.88	-1.72					-2.0	0.00
Q9C0J8	pre-mRNA 3' end processing protein WDR33 OS=Homo sapiens GN=WDR33 PE=1 SV=2 - [WDR33_HUMAN]					-2.31	-1.91	-2.13	-1.72	-2.0	0.00
Q9UNU6	7-alpha-hydroxycholest-4-en-3-one 12-alpha-hydroxylase OS=Homo sapiens GN=CYP8B1 PE=2 SV=2 - [CP8B1_HUMAN]	-3.64	-3.60	-4.09	-4.05	-0.23	-0.34	-0.06	-0.17	-2.0	0.02
Q15399	Toll-like receptor 1 OS=Homo sapiens GN=TLR1 PE=1 SV=3 - [TLR1_HUMAN]	-2.07	-2.18	-1.88	-1.97					-2.0	0.00
Q9UL63	Muskelin OS=Homo sapiens GN=MKLN1 PE=1 SV=2 - [MKLN1_HUMAN]	-2.02	-2.07	-2.09	-2.14					-2.1	0.00

A6NJZ7	RIMS-binding protein 3C OS=Homo sapiens GN=RIMBP3C PE=1 SV=3 - [RIM3C_HUMAN]				-1.41	-2.96	-3.38	-1.40	-1.82	-2.2	0.01
Q14679	Tubulin polyglutamylase TTLL4 OS=Homo sapiens GN=TTLL4 PE=1 SV=2 - [TTLL4_HUMAN]	-3.27	-4.36	-2.07	-3.15	-2.70	-0.07	-2.47	0.16	-2.2	0.00
Q9Y5H1	Protocadherin gamma-A2 OS=Homo sapiens GN=PCDHGA2 PE=2 SV=1 - [PCDG2_HUMAN]	-3.39	-3.84	-1.80	-2.24	-3.86	-3.16	-0.22	0.48	-2.3	0.01
P29350	Tyrosine-protein phosphatase non-receptor type 6 OS=Homo sapiens GN=PTPN6 PE=1 SV=1 - [PTN6_HUMAN]	-5.21	-5.70	-4.10	-4.58	0.03	0.14	0.15	0.24	-2.4	0.04
Q13591	Semaphorin-5A OS=Homo sapiens GN=SEMA5A PE=1 SV=3 - [SEM5A_HUMAN]					-3.71	-4.56	-1.25	-1.35	-2.7	0.05
Q5KSL6	Diacylglycerol kinase kappa OS=Homo sapiens GN=DGKK PE=1 SV=1 - [DGKK_HUMAN]	-2.67	-3.74	-2.77	-3.84					-3.3	0.00
