# Supplementary material for: Increased circulating resistin levels in early-onset breast cancer patients of normal body mass index correlate with lymph node negative involvement and longer disease free survival: a multi-center POSH cohort serum proteomics study
Source: Breast Cancer Res. 2018 Mar 22;20:19. doi: 10.1186/s13058-018-0938-6 (PMC5863447; doi:10.1186/s13058-018-0938-6)
Supplement: Supplementary file 5 — Section 5 presenting ELISA measurements for resistin. (PDF 356 kb) [file 13058_2018_938_MOESM5_ESM.pdf]

Supplementary Section 5. ELISA measurements of resistin

Plate 1 (1:25 dilution)

Outcome group	Sample ID	RESISTIN c (ng/mL)	RESISTIN LOG10(c)	OD450	interpolated data	transform c (1:25)	c (ng/mL)
	Standard	10.00	1.00	0.8292			
	Standard	10.00	1.00	0.7675			
	Standard	5.00	0.70	0.4299			
	Standard	5.00	0.70	0.5702			
	Standard	2.50	0.40	0.1552			
	Standard	2.50	0.40	0.2017			
	Standard	1.25	0.10	0.0804			
	Standard	1.25	0.10	0.1033			
	Standard	0.63	-0.20	0.0453			
	Standard	0.63	-0.20	0.0231			
	Standard	0.31	-0.51	0.0231			
	Standard	0.31	-0.51	0.0429			
	Standard	0.16	-0.81	0.0122			
	Standard	0.16	-0.81	0.0219			
Poor	65			0.1322	0.2945	1.97	49.25
Good	85			0.1284	0.2864	1.93	48.34
Good	42			0.1300	0.2899	1.95	48.74
Good	9			0.1322	0.2946	1.97	49.26
Poor	60			0.0847	0.1642	1.46	36.49
Poor	74			0.0681	0.0919	1.24	30.89
Poor	7			0.1347	0.2997	1.99	49.85
Good	65			0.1581	0.3442	2.21	55.23
Poor	49			0.1257	0.2804	1.91	47.68
Good	25			0.1410	0.3125	2.05	51.33
Good	61			0.3712	0.5933	3.92	98.01
Good	39			0.1492	0.3282	2.13	53.23
Good	45			0.0914	0.1877	1.54	38.52
Good	47			0.1624	0.3516	2.25	56.18
Good	48			0.4083	0.6259	4.23	105.65
Poor	38			0.3592	0.5825	3.82	95.59
Poor	86			0.3369	0.5619	3.65	91.17
Good	49			0.3560	0.5795	3.80	94.95
Poor	63			0.1507	0.3309	2.14	53.55
Good	14			0.0528	-0.0064	0.99	24.63
Good	38			0.1155	0.2565	1.80	45.12
Good	73			0.2994	0.5253	3.35	83.79
Good	83			0.1095	0.2412	1.74	43.56
Good	88			0.2626	0.4864	3.06	76.62
Poor	8			0.2004	0.4097	2.57	64.21
Good	3			0.1026	0.2221	1.67	41.69
Poor	2			0.1907	0.3959	2.49	62.20

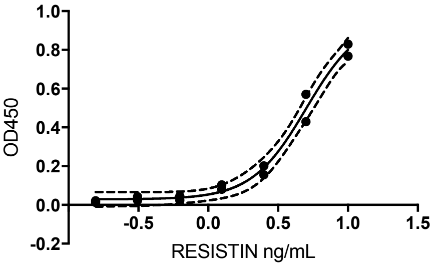

Good	15			0.0380	-0.1957	0.64	15.93
Good	5			0.2440	0.4652	2.92	72.97
Good	77			0.2145	0.4286	2.68	67.08
Poor	79			0.2568	0.4799	3.02	75.47
Poor	16			0.0982	0.2091	1.62	40.47
Good	50			0.1782	0.3772	2.38	59.59
Poor	56			0.1728	0.3687	2.34	58.43
Poor	24			0.2969	0.5227	3.33	83.31
Good	26			0.2373	0.4572	2.87	71.64
Poor	71			0.0920	0.1895	1.55	38.68
Poor	28			0.1730	0.3691	2.34	58.48
Poor	40			0.2841	0.5096	3.23	80.82
Poor	90			0.0935	0.1946	1.57	39.13
Poor	13			0.2968	0.5227	3.33	83.29

Plate 2 (1:25 dilution)

Outcome group	Sample ID	RESISTIN ng/mL	RESISTIN LOG10(c)	OD450	interpolated data	transform c (1:25)	c (ng/mL)
	Standard	10	1.00	0.6690			
	Standard	10	1.00	0.6730			
	Standard	5	0.70	0.4732			
	Standard	5	0.70	0.4600			
	Standard	2.5	0.40	0.1608			
	Standard	2.5	0.40	0.2232			
	Standard	1.25	0.10	0.0848			
	Standard	1.25	0.10	0.0871			
	Standard	0.625	-0.20	0.0364			
	Standard	0.625	-0.20	0.0413			
	Standard	0.312	-0.51	0.0061			
	Standard	0.312	-0.51	0.0291			
	Standard	0.156	-0.81	0.0004			
	Standard	0.156	-0.81	0.0124			
Poor	21			0.0336	-0.1643	0.69	17.1
Poor	81			0.0770	0.1154	1.30	32.6
Good	6			0.1016	0.1950	1.57	39.2
Poor	82			0.0536	0.0050	1.01	25.3
Good	79			0.1776	0.3547	2.26	56.6
Poor	53			0.1710	0.3436	2.21	55.2
Poor	51			0.0598	0.0396	1.10	27.4
Poor	18			0.0610	0.0456	1.11	27.8
Poor	57			0.0597	0.0389	1.09	27.3
Poor	87			0.2453	0.4536	2.84	71.0
Poor	45			0.0646	0.0634	1.16	28.9
Good	56			0.2952	0.5156	3.28	81.95
Poor	29			0.1059	0.2068	1.61	40.2

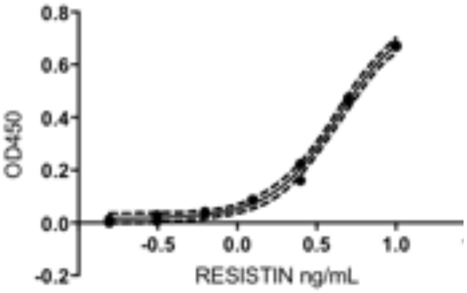

Good	31	0.1017	0.1953	1.57	39.2
Poor	84	0.1148	0.2295	1.70	42.4
Good	75	0.1061	0.2072	1.61	40.3
Poor	54	0.0288	-0.2354	0.58	14.5
Good	66	0.0648	0.0640	1.16	29.0
Poor	39	0.3393	0.5664	3.68	92.1
Good	32	0.1623	0.3284	2.13	53.25
Poor	64	0.0581	0.0309	1.07	26.8
Poor	5	0.3302	0.5561	3.60	90.0
Good	60	0.3060	0.5283	3.38	84.4
Good	37	0.1589	0.3223	2.10	52.5
Good	69	0.1361	0.2779	1.90	47.4
Poor	58	0.0548	0.0121	1.03	25.7
Poor	47	0.2435	0.4513	2.83	70.7
Good	40	0.1447	0.2953	1.97	49.35
Poor	32	0.1165	0.2337	1.71	42.8
Poor	41	0.2228	0.4231	2.65	66.2
Poor	70	0.0753	0.1090	1.29	32.1
Poor	62	0.1458	0.2975	1.98	49.6
Poor	91	0.3035	0.5254	3.35	83.8
Poor	67	0.1781	0.3555	2.27	56.7
Poor	23	0.1310	0.2670	1.85	46.2
Poor	44	0.1591	0.3226	2.10	52.5
Poor	78	0.1702	0.3423	2.20	55.0
Good	68	0.1087	0.2140	1.64	40.9
Good	27	0.1281	0.2605	1.82	45.6
Poor	20	0.1132	0.2257	1.68	42.0
Poor	88	0.1712	0.3439	2.21	55.2
Poor	30	0.4267	0.6625	4.60	114.9
Good	90	0.1799	0.3585	2.28	57.1
Poor	12	0.1717	0.3448	2.21	55.3
Poor	75	0.2405	0.4473	2.80	70.0
Good	16	0.2394	0.4458	2.79	69.8
Good	51	0.1541	0.3135	2.06	51.5
Poor	66	0.0540	0.0075	1.02	25.4
Good	70	0.0683	0.0798	1.20	30.05
Poor	14	0.2672	0.4816	3.03	75.8
Good	43	0.6961	1.0527	11.29	282.3
Poor	36	0.2869	0.5057	3.20	80.1
Poor	59	0.1319	0.2690	1.86	46.4
Poor	6	0.0330	-0.1717	0.67	16.8
Poor	48	0.1839	0.3651	2.32	57.9
Poor	27	0.2692	0.4840	3.05	76.2
Good	13	0.0861	0.1476	1.40	35.1
Poor	26	0.4318	0.6681	4.66	116.4
Good	17	0.1153	0.2308	1.70	42.5

Good	59			0.1461	0.2980	1.99	49.7
Poor	22			0.2143	0.4112	2.58	64.4
Poor	50			0.5765	0.8401	6.92	173.0
Good	35			0.1337	0.2727	1.87	46.8
Good	82			0.1229	0.2489	1.77	44.35
Poor	15			0.1038	0.2012	1.59	39.7
Poor	1			0.1002	0.1911	1.55	38.8
Good	89			0.0862	0.1480	1.41	35.15
Good	21			0.0735	0.1017	1.26	31.6

Plate 3 (1:50 dilution)

Outcome group	Sample ID	RESISTIN ng/mL	RESISTIN LOG10(c)	OD450	interpolated data	transform c (1:50)	c (ng/mL)
	Standard	10.00	1.00	0.5577			
	Standard	10.00	1.00	0.5907			
	Standard	5.00	0.70	0.3197			
	Standard	5.00	0.70	0.3407			
	Standard	2.50	0.40	0.1207			
	Standard	2.50	0.40	0.1507			
	Standard	1.25	0.10	0.0367			
	Standard	1.25	0.10	0.0577			
	Standard	0.63	-0.20	0.0207			
	Standard	0.63	-0.20	0.0247			
	Standard	0.31	-0.51	0.0057			
	Standard	0.31	-0.51	0.0127			
	Standard	0.16	-0.81	0.0047			
	Standard	0.16	-0.81	0.0127			
Good	46			0.0957	0.2953	1.97	98.70
Poor	85			0.1597	0.4479	2.80	140.22

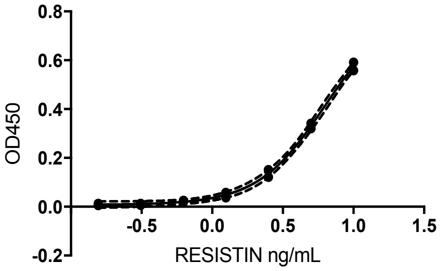

Plate 3 (1:25 dilution)

Outcome group	Sample ID	RESISTIN ng/mL	RESISTIN ng/mL	OD450	interpolated data	transform c (1:25)	c (ng/mL)
	Standard	10.00	1.00	0.5580			
	Standard	10.00	1.00	0.5910			
	Standard	5.00	0.70	0.3200			
	Standard	5.00	0.70	0.3410			
	Standard	2.50	0.40	0.1210			
	Standard	2.50	0.40	0.1510			
	Standard	1.25	0.10	0.0370			
	Standard	1.25	0.10	0.0580			
	Standard	0.63	-0.20	0.0210			
	Standard	0.63	-0.20	0.0250			
	Standard	0.31	-0.51	0.0060			
	Standard	0.31	-0.51	0.0130			

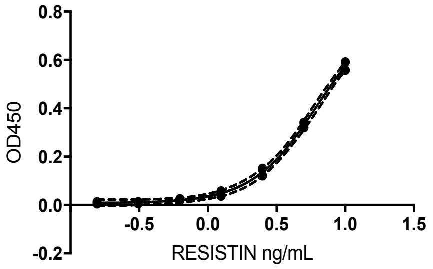

	Standard	0.16	-0.81	0.0050			
	Standard	0.16	-0.81	0.0130			
Good	54			0.3090	0.6722	4.70	235.1
Poor	55			0.0700	0.2034	1.60	79.9
Good	34			0.1420	0.4113	2.58	128.9
Good	44			0.0830	0.2530	1.79	89.5
Good	4			0.0470	0.0856	1.22	60.9
Good	71			0.2320	0.5680	3.70	184.9
Poor	35			0.2490	0.5925	3.91	195.7
Good	81			0.0330	-0.0248	0.94	47.2
Good	87			0.1110	0.3379	2.18	108.9
Poor	17			0.1020	0.3131	2.06	102.8
Good	63			0.1400	0.4071	2.55	127.7
Poor	10			0.0860	0.2633	1.83	91.7
Good	58			0.0950	0.2923	1.96	98.0
Poor	73			0.0950	0.2923	1.96	98.0
Poor	19			0.1730	0.4722	2.97	148.3
Good	11			0.2020	0.5218	3.33	166.3
Good	36			0.5810	1.0092	10.21	510.7
Poor	68			0.1680	0.4630	2.90	145.2
Poor	76			0.3140	0.6785	4.77	238.5
Good	84			0.2760	0.6295	4.26	213.0
Poor	9			0.1850	0.4934	3.11	155.7
Good	10			0.2120	0.5377	3.45	172.5
Good	57			0.0760	0.2273	1.69	84.4
Good	74			0.0450	0.0724	1.18	59.1
Poor	11			0.1950	0.5103	3.24	161.9
Good	64			0.0880	0.2700	1.86	93.1
Good	2			0.1470	0.4219	2.64	132.1
Good	55			0.1850	0.4934	3.11	155.7
Poor	3			0.3540	0.7276	5.34	267.0
Poor	61			0.1300	0.3848	2.43	121.3
Good	19			0.1480	0.4239	2.65	132.7
Poor	72			0.2220	0.5531	3.57	178.7
Good	22			0.4830	0.8813	7.61	380.4
Poor	83			0.2950	0.6544	4.51	225.6
Poor	43			0.1510	0.4301	2.69	134.6
Good	24			0.1390	0.4049	2.54	127.0
Poor	42			0.1070	0.3271	2.12	106.2
Good	8			0.1000	0.3073	2.03	101.45
Good	76			0.6330	1.0897	12.29	614.6
Good	41			0.0350	-0.0058	0.99	49.3
Good	53			0.3440	0.7155	5.19	259.7
Good	20			0.1100	0.3352	2.16	108.2
Poor	46			0.1180	0.3559	2.27	113.5
Poor	89			0.2420	0.5826	3.82	191.2

Poor	25	0.1470	0.4219	2.64	132.1
Good	67	0.0460	0.0791	1.20	60.0
Good	62	0.2240	0.5561	3.60	179.9
Good	86	0.1290	0.3825	2.41	120.6
Poor	37	0.1470	0.4219	2.64	132.1
Good	18	0.2270	0.5606	3.64	181.8
Poor	80	0.1530	0.4341	2.72	135.8
Poor	69	0.0960	0.2953	1.97	98.7
Poor	77	0.1940	0.5087	3.23	161.3
Good	1	0.4700	0.8655	7.34	366.9
Good	52	0.0840	0.2564	1.80	90.2
Good	7	0.0700	0.2034	1.60	79.9
Good	23	0.2400	0.5797	3.80	190.0
Good	80	0.0960	0.2953	1.97	98.7
Poor	33	0.2790	0.6335	4.30	215.0
Poor	31	0.0750	0.2235	1.67	83.6
Poor	4	0.3130	0.6773	4.76	237.8
Good	28	0.1750	0.4758	2.99	149.55
Good	29	0.5850	1.0150	10.35	517.5
Poor	34	0.1770	0.4794	3.02	150.8
Good	33	0.5600	0.9799	9.55	477.4
Good	72	0.0730	0.2156	1.64	82.1
Good	78	0.2090	0.5330	3.41	170.6
Poor	52	0.2080	0.5314	3.40	170.0
Good	30	0.2550	0.6009	3.99	199.5

1.5
