# Supplementary material for: Increased circulating resistin levels in early-onset breast cancer patients of normal body mass index correlate with lymph node negative involvement and longer disease free survival: a multi-center POSH cohort serum proteomics study
Source: Breast Cancer Res. 2018 Mar 22;20:19. doi: 10.1186/s13058-018-0938-6 (PMC5863447; doi:10.1186/s13058-018-0938-6)
Supplement: Supplementary file 6 — Section 6 presenting linear and generalized linear modelling of resistin, ER, PR, LN and HER-2 clinical parameters. (DOCX 108 kb) [file 13058_2018_938_MOESM6_ESM.docx]

**Circulating resistin in early-onset breast cancer patients with normal body mass index correlates with disease-free survival and lymph node involvement:**

**An agnostic quantitative proteomics study from the multi-center POSH^¶^ cohort**

Zeidan^1^, B. et al.

^¶^ The **P**rospective study of **O**utcomes in **S**poradic versus **H**ereditary breast cancer

**Linear and generalized linear modelling**

Both linear and generalized linear modelling techniques were utilized to determine which covariates should be included in the final model that would relate outcome to resistin measurements. Generalized linear modelling was used when the response variable was categorical (Prognostic outcome as a function of Resistin) and linear modelling was used when the response variable was numerical (Resistin as a function of LN status).

Resistin measures were subjected to linear regression modelling with LN as the independent variable. LN was found to be significant (**Table 1**) (*p-value = 0.004*) Based upon these results, it was decided that including LN in modelling would violate the assumptions of independence of variables. Additionally, the effect of resistin is partially captured by the LN variable (**Table 1**). So, to properly assess the effect of resistin, LN was removed as a covariate from outcome modelling.

Resistin was further modelled against all other covariates ER, PR, HER2, and BMI separately. This testing was performed on pairs of other coefficients to determine which were clearly dependent from others and should be removed from the final model due to potential co-linearity issues. HER2 demonstrated a significant association with LN and PR, while PR demonstrated a significant association with LN and ER (**Table 1**).

**Table 1**. Significant results for collinearity testing upon coefficients

| Coefficient | Estimate | Std. Error | p-value | Significance |
| --- | --- | --- | --- | --- |
| **Resistin~LN** | |  |  |  |
| LN Positive | -40.105 | 13.616 | 0.003654 | ** |
|  |  |  |  |  |
| **HER2~LN** |  |  |  |  |
| LN Positive | 1.31079 | 0.4642 | 0.004747 | ** |
|  |  |  |  |  |
| **HER2~PR** |  |  |  |  |
| PR Negative | 1.46283 | 0.53255 | 0.006017 | ** |
| PR High | 1.09861 | 0.61299 | 0.073098 |  |
| PR Low | 1.60944 | 1.11192 | 0.147773 |  |
| PR Medium | 16.77761 | 1021.481 | 0.986896 |  |
|  |  |  |  |  |
| **LN~PR** |  |  |  |  |
| PR Negative | 0.336 | 0.417 | 0.41985 |  |
| PR High | 0.539 | 0.488 | 0.26933 |  |
| PR Low | 0.693 | 0.707 | 0.32696 |  |
| PR Medium | 1.87 | 0.838 | 0.02547 | * |
|  |  |  |  |  |
| **ER~PR** |  |  |  |  |
| PR Negative | -2.62 | 0.498 | 1.35E-07 | *** |
| PR High | 17.5 | 1058 | 0.986829 |  |
| PR Low | -1.66E-15 | 7.82E-01 | 1 |  |
| PR Medium | 1.54 | 1.11 | 0.166229 |  |

**Note**: All pairs of coefficients were tested and only significant results are reported. Significant results are indicated (*, **, and ***, for p-value < 0.05, 0.01, and 0.001 respectively). Formulas used for modelling are highlighted in bold.

Additionally, not all patients had measures for HER2 and PR. Due to these results, HER2, and PR were removed as covariates. The final model treated the outcome as a function of resistin, ER, and BMI. Resistin, high ER and medium ER status showed a significant effect upon outcome while low ER status and BMI did not (**Table 2**).

**Table** . Outcome modelled as a function of resistin, BMI, and ER status, and outcome modelled as a function of the previous covariates and LN status.

|  |  |  |  |  |
| --- | --- | --- | --- | --- |
| Coefficient | Estimate | Std. Error | P_VAL | Significance |
|  |  |  |  |  |
| **Outcome ~ Resistin + BMI +**  **ER + LN** |  |  |  |  |
| Resistin | 2.88E-03 | 2.00E-03 | 1.50E-01 |  |
| BMI | 3.85E-02 | 7.37E-02 | 6.01E-01 |  |
| ER Positive | 1.04E+00 | 3.28E-01 | 1.49E-03 | ** |
| LN Medium | -1.03E+00 | 3.44E-01 | 2.65E-03 | ** |
|  |  |  |  |  |
| **Outcome ~ Resistin + BMI + ER** |  |  |  |  |
| Resistin | 4.11E-03 | 2.03E-03 | 4.28E-02 | * |
| BMI | 1.29E-02 | 7.09E-02 | 8.56E-01 |  |
| ER Positive | 8.77E-01 | 3.11E-01 | 4.83E-03 | ** |

**Note**: Significant results are indicated (*, **, and ***, for p-value < 0.05, 0.01, and 0.001 respectively). Formulas used for modelling are highlighted in bold.
