# Supplementary material for: Increased circulating resistin levels in early-onset breast cancer patients of normal body mass index correlate with lymph node negative involvement and longer disease free survival: a multi-center POSH cohort serum proteomics study
Source: Breast Cancer Res. 2018 Mar 22;20:19. doi: 10.1186/s13058-018-0938-6 (PMC5863447; doi:10.1186/s13058-018-0938-6)
Supplement: Supplementary file 1 — Sections 1A and 1B presenting POSH Serum Procurement SOPs. (ZIP 244 kb) [file 13058_2018_938_MOESM1_ESM.zip › Supplementary Information 1B-POSH SOP 2.doc]

**TITLE:**

**Protocol for Collection, Preparation and Storage of POSH Study Breast Tissue.**

| **SOP Code** | CSD/SOP/127 | | **Issue No** | 4 | **No of pages** | | 10 | **Manual No.** | Q PULSE |
| --- | --- | --- | --- | --- | --- | --- | --- | --- | --- |
|  |  | |  |  |  | |  |  |  |
| **ISSUE No.** | **Revision History** | | | | | | **Author** | | **Effective Date** |
| 01 | New Procedure | | | | | | Louise Shaw | | 24.10.01 |
| 04 | Changes to header and footer to reflect new University logo and re- branding.  4.0 Update of Related Documents  5.1.3 Revision of consent procedure  5.7.6 Block size  6.0 Revision of Release procedure | | | | | | Ryan Jones | |  |
|  |  | |  |  | |  |  |  |  |
|  | | **NAME (print)** | | | | **SIGNATURE** | | | **DATE** |
| **WRITTEN BY:** | | Ryan Jones | | | |  | | |  |
| **APPROVED BY:** | | Diana Eccles | | | |  | | |  |
| **REVIEWED BY:**  **QA:** | | Sarah Moyle | | | |  | | |  |
| **EFFECTIVE DATE:** | | | | | | **REVIEW DATE:** | | | |

**Full Revision History**

| **ISSUE No.** | **Revision History** | **Author** | **Effective Date** |
| --- | --- | --- | --- |
| 01 | New Procedure | Louise Shaw | 24.10.01 |
| 02 | Format update and major rewrite of SOP to update procedures and incorporate Tumour Bank SOPs into Cancer Sciences Division (CSD) Document Control System. Replaces ‘Preparation of Blood and Breast Tissue Samples for POSH Study’ | Myra Armstrong | 18 May 04 |
| 03 | Update to include Full Revision History page 2 and addition of required training status 8.0. Review to include the addition of Related Documents 4.1.5 to 4.1.8. Change to disinfectant used 5.4.1 & 5.7.7. Room changes 5.1.3, 5.6.3, 5.8.4. | Myra Armstrong | 7 Mar 06 |
| 04 | Changes to header and footer to reflect new University logo and re- branding.  4.0 Update of Related Documents  5.1.3 Revision of consent procedure  5.7.6 Block size  6.0 Revision of Release procedure | Ryan Jones |  |

1. **INTRODUCTION.**

This Standard Operating Procedure documents the procedures to follow for the collection, preparation and storage of samples of normal and malignant breast tissue for the Prospective Study of Outcomes in Sporadic versus Hereditary Breast Cancer (POSH) study MREC/00/6/69

The objective of this procedure is to prepare and store material suitable for future analysis.

This procedure describes the preparation and storage of solid tissue.

The aim is to ensure an adequate and controlled protocol with appropriate storage for long term integrity until further analysis commences.

2. **SCOPE.**

2.1 This procedure applies to any patient samples for which consent has

been sought for the POSH Study as part of the Cancer Research-UK

clinical trials within the Medical Oncology Department.

3. **RESPONSIBILITIES.**
   3.1 It is the responsibility of the Principal Investigator to ensure that staff of adequate experience and training perform the preparation outlined in this Standard Operating Procedure.
   3.2 It is the responsibility of the Tumour Bank Technicians to become familiarised with and follow the procedures outlined in this protocol and that all sample preparation is done in a safe and responsible manner.
   3.3 It is the responsibility of all staff members at all times to adhere to Data Protection with regards to patient confidentiality.
   3.4 It is the responsibility of all staff to ensure that any problems with sample collection, analysis, storage or documentation should be reported to the Unit Head immediately.
   3.5 It is the responsibility of all staff members to work safely and respectfully of others at all times and to report anything they believe inappropriate or unsafe to their Section Head/Senior immediately.

4. **RELATED DOCUMENTS.**
   4.1 **DOCUMENTS.**

**Internal**

4.1.1 University of Southampton Cancer Sciences Division HTA licence 12009
4.1.2 CSD/SOP/101	Protocol for Release and Distribution of Bank Material
4.1.3 CSD/SOP/038	UoS CSD Policy & Procedures for Collection Storage and Distribution of Human Tissue
4.1.4 CSD/SOP/039	UoS CSD Policy & Procedures for ‘Future Use’ Human Tissue post HTA 2006
4.1.5 CSD/SOP/098	Research Ethics Related Procedures
4.1.6 CSD/SOP/330	Training of Staff Involved with Tissue Bank and Clinical Trial Endpoints
4.1.7 CSD/SOP/231	Data Protection Procedures
4.1.8 CSD/SOP/220	Written Informed Consent Procedures
4.1.9 CSD/SOP/102	SOP for Unlinking Anonymised Samples
4.1.10 CSD/SOP/400	SOP Safety Precautions for Handling Liquid Nitrogen
4.1.11 CSD/SOP/037	Adverse Events Relating to Tissue Stored under HTA Licence 12009
4.1.12 CSD/SOP/416	SOP for Safe Working Practices
4.1.13 CSD/SOP/310	Class II Safety Cabinets Operation, Cleaning, Preventative Maintenance and Servicing
4.1.14 CSD/FORMS/121 Worksheet
4.1.15 CSD/SOP/125	Operation of POSH Database
4.1.16 CSD/SOP/416	SOP for Safe Working Practices

**External**

4.1.17 2004/23/EC European Union ‘Parent Directive’ on Setting Standards of

Quality and Safety for the Donation, Procurement, Testing and Processing Storage and Distribution of Human Tissue and Cells.

4.1.18 2006/86/EC European Union ‘Commission Directive’ implementing

Directive 2004/23/EC for the Traceability Requirements, Notification of Serious Adverse Reactions and Events and Certain Technical Requirements for the Coding, Processing, Preservation, Storage and Distribution of Human Tissue and Cells

4.1.19 Human Tissue Act 2004
4.1.20 2006/86/EC European Union ‘Commission Directive’ Implementing Technical Requirements of 2004/23/EC for Donation Procurement and Testing of Human Tissue and Cells.
4.1.21 Human Tissue Authority Codes of Practise – Consent
4.1.22 Human Tissue Authority Codes of Practise – Removal, Storage and Disposal of Human Organs and Tissue
4.1.23 HTA Directions given	under the Human Tissue Act 2004: (Check for updates–issued April and October yearly).

*Note many of above documents are written more specifically for transplant type activities; legislation covers research use and so many systems and requirements above may therefore be applicable*

4.1.24 British Medical Association (BMA) Human Tissue Legislation Guidance

from BMAs Ethics Department

4.1.25 2001/20/EC European Union ;Clinical Trials Directive’
4.1.26 The ICH Guidelines for Good Clinical Practise. Jan

1999/CPMP/ICH/135/95.

4.1.27 ICH Harmonised Tripartite Guidelines for Good Clinical Practice.

Jan 1999/CPMP/ICH/135/95.

.

4.2 **CONTACTS**
    4.2.1 POSH administrator	x5171
    4.2.2 Principal Investigator	x5162
    4.2.3 Cancer Sciences Reception	x6184
    4.2.4 SGH – Post room	x4125

5. **PROCEDURE.**
   5.1 **Consent for Tumour Banking.**
       5.1.1 Consent is obtained prior to sample collection via the POSH administrator.
       5.1.2 Various Research Nurses from participating hospitals hold the consent form and will forward a copy along with the samples directly to the Tumour Bank.
       5.1.3 All consent forms received should be photocopied and given to the POSH administrator. The administrator will log the relevant details and update the POSH Database.
   5.2 **EQUIpment / REAGENTS**

| ITEM OF EQUIPMENT | SUPPLIER | CODE | LOCATION |
| --- | --- | --- | --- |
|  |  |  |  |
| SAFETY CABINET | KENDRO | 510191142 | CS/C19 |
| RNAlater | AMBION | 7021 | CS/C19 |
| -80°c FREEZER | BOROLABS | - | CS/B12 |
|  |  |  |  |

5.3 **APPARATUS**

| APPARATUS | QTY | SUPPLIER | CODE | LOCATION |
| --- | --- | --- | --- | --- |
|  |  |  |  |  |
| PETRI DISH | X2 | GREINER | 664160 | CS/C19 |
| STERILE SCALPLE | X1 | - | - | CS/C19 |
| STERILE FORCEPS | X1 | - | - | CS/C19 |
| CRYOVIALS  UNIVERSALS | 1.5ml  - | GREINER  - | 122263  - | CS/C19  CS/C19 |
| PARAFILM | - | - | - | CS/C19 |
|  |  |  |  |  |

5.4 **REAGENT PREPARATION**

5.4.1	TRIGENE: 4% to be made fresh daily. Stored in room CS/C19.

5.5 **HAZARDOUS SUBSTANCE INFORMATION**
    5.5.1 Biological Hazard – All biological samples should be treated as a possible cause of infection, departmental and SUHT safety policy guidelines must be adhered to when handling human material.
    5.5.2 All staff must adhere to CSD/SOP/416 Safe Working Practices.
5.6 **SAMPLE COLLECTION**
    5.6.1 Posh tissue samples will be arranged via the participating hospital and the POSH administrator.
    5.6.2 Once sample collection notification has been made, a tissue collection package is posted to the participating hospital. The package contains two universals each containing 15ml RNAlater and sealed with Parafilm, Additional Parafilm for the return journey, sufficient absorbent material to soak up any spills in transit, information sheet and return postage. RNAlater can be stored at room temperature for six months. Any precipitation may be rectified by heating to 37°c and agitating the solution.
    5.6.3 A fax will be sent directly to the Cancer Sciences Department confirming samples have been posted. This fax should be filed in the POSH fax file located in room CS/B9.
    5.6.4 Time of receipt must be noted on a CSD/FORMS/121 worksheet (see appendix I). Tissue stored in RNAlater must be kept in the prep room refrigerator (located in room CS/C19) at 4°c overnight prior to processing.
    5.6.5 Enter the patient information in to the POSH database (see CS/SOP/125)

where a sample number will be generated. This number must now be used for sample identification to protect patient anonymity.

5.7 **METHOD**
    5.7.1 All users of this SOP must adhere to the SOP for Safe Working Practice CSD/SOP/416.
    5.7.2 All procedures must be performed in sterile conditions in a Class II Safety Cabinet according to CSD/SOP/310.
    5.7.3 Ensure all instruments needed are available and to hand.
    5.7.4 Samples received should be cut to no less than 0.5cm thick in any one dimension (other dimensions are not important) and be in five times its volume in RNAlater. Any deviation from this should be noted on the worksheet CS/FORMS/121.
    5.7.5 Samples must be incubated overnight at 4°c.
    5.7.6 In a safety cabinet, place samples in to a sterile Petri dish using sterile forceps. Cut the sample with a sterile scalple in to small tissue blocks approximately 5 x 5 mm^2^ (if not already done so prior to shipping) and place each block in to a BLUE cryovial labelled with sample type, POSH database generated number and sample date.
    5.7.7 All metal apparatus must be thoroughly cleaned with TriGene, dried and each piece placed in an autoclave bag with contents clearly labelled.
5.8 **SAMPLE STORAGE**
    5.8.1 All staff following this SOP must adhere to CSD/SOP/315 Liquid Nitrogen Tanks and CSD/SOP/416 Safe Working Practice.
    5.8.2 All vials are stored in the POSH Tissue Boxes located in -80°c freezer located in room CS/B11 noting storage location on the relevant CSD/FORMS/121 worksheet.
    5.8.3 Enter all information on to the POSH database including storage location, number of vials stored, sample received date, sample processed date and sample stored date.
    5.8.4 Fully completed worksheets should be filed in the POSH Sample File located in CS/B9.

6.0 **RELEASE OF STORED MATERIAL**
    6.1 Follow SOP/CSD/101 for the release of material stored within the Tissue Bank. A summary of this procedure is described below.

*Researchers who wish to access samples from the tumour bank should liaise with the Tumour Bank Steering Group, who will provide details of stored materials and those materials available for use. The Steering Committee will notify the Clinician responsible for storing the tumour samples. A full proposal will be required for submission to the Cancer Protocol Review Committee and to the POSH Steering Group via the Principal Investigator. If approved a further subsequent submission to the National Research Ethics Services (NRES) will take place.*

*Once the relevant approval has been obtained, access to tumour samples will be through the Tissue Bank Manager (PD). A copy of the ethics approval must be provided to the PD prior to release of samples.*

*The PI of the study must sign departmental policy documents CSD/SOP/038 and CSD/SOP/039 prior to sample release (this needs to be done only once).*

*On collection of the samples, the researcher receiving tissue as agreed with PI above, must sign copies of CSD/FORMS/152 Sample Release Compliance Form to acknowledge receipt of the samples, change of ownership and their obligations under the Human Tissue Act 2004. One copy is to be held within the bank and one copy provided to the researcher for their own records (no patient identifiable information will be disclosed on this form). The Tissue Bank Manager (or approved Senior Bank staff in Managers absence) will arrange for samples to be located and removed from storage as and when required.*

*Full details of sample removal must be logged as per CSD/SOP/101. All Ethics copies/Paperwork must be stored as per CSD/SOP/101. All sample release must be logged on reverse side of work sheets or on historical release sheet as appropriate CSD/FORMS/125 and the database amended accordingly.*

6.2 No samples will be released without full and proper authorisation.

.

7.0 **APPENDED**

Appendix I CSD/FORMS/121 Worksheet

**8.0	LIST OF CURRENT USERS and TRAINING.**

Level of training required for this update.

√

Read. Demonstrate/Briefing. Full Training

The Following people have been trained /read and understood this SOP and are currently using these procedures in the laboratory:

**Name**	**Signature**	**Date**
