# Supplementary material for: Increased circulating resistin levels in early-onset breast cancer patients of normal body mass index correlate with lymph node negative involvement and longer disease free survival: a multi-center POSH cohort serum proteomics study
Source: Breast Cancer Res. 2018 Mar 22;20:19. doi: 10.1186/s13058-018-0938-6 (PMC5863447; doi:10.1186/s13058-018-0938-6)
Supplement: Supplementary file 1 — Sections 1A and 1B presenting POSH Serum Procurement SOPs. (ZIP 244 kb) [file 13058_2018_938_MOESM1_ESM.zip › Supplementary Section 1A-POSH SOP A.doc]

**TITLE:**

**Protocol for Whole Blood, Serum and Lymphocyte Extraction of POSH Bloods.**

| **SOP Code** | CSD/SOP/126 | | **Issue No** | 5 | **No of pages** | | 12 | **Manual No.** | Q PULSE |
| --- | --- | --- | --- | --- | --- | --- | --- | --- | --- |
|  |  | |  |  |  | |  |  |  |
| **ISSUE No.** | **Revision History** | | | | | | **Author** | | **Effective Date** |
| 01 | New Procedure | | | | | | Louise Shaw | | 24.10.01 |
| 05 | Changes to header and footer to reflect new University logo and re- branding.  1. EDTA replacing heparinised blood  4.0 Update of Related Documents  5.1 Revision of consent procedure  5.3 Supplier change and media update  6.0 Revision of Release procedure | | | | | | Ryan Jones | |  |
|  |  | |  |  | |  |  |  |  |
|  | | **NAME (print)** | | | | **SIGNATURE** | | | **DATE** |
| **WRITTEN BY:** | | Ryan Jones | | | |  | | |  |
| **APPROVED BY:** | | Diana Eccles | | | |  | | |  |
| **REVIEWED BY:**  **QA:** | | Sarah Moyle | | | |  | | |  |
| **EFFECTIVE DATE:** | | | | | | **REVIEW DATE:** | | | |

**Full Revision History**

| **ISSUE No.** | **Revision History** | **Author** | **Effective Date** |
| --- | --- | --- | --- |
| 01 | New Procedure | Louise Shaw | 24.10.01 |
| 02 | Format update and major rewrite of SOP to update procedures and incorporate Tumour Bank SOPs into Cancer Sciences Division (CSD) Document Control System. Replaces ‘Preparation Of Blood and Breast Tissue Samples for POSH Study.’ | Myra Armstrong | 18 May 04 |
| 03 | CSD/FORMS/120 POSH Samples Weekly Report added to appendix, 5.7.6 and Related Documents. Room change for filed paperwork. | Myra Armstrong | 29 April 05 |
| 04 | Update to include Full Revision History page 2 and addition of training required status 8.0. Review to include the addition of Related Documents 4.1.11 to 4.1.14. Change to disinfectant used 5.3 & 5.5.3. Removal of Hepes from Reagent Prep 5.5.1. Change to blood sampling requirements 5.8.6 & 5.8.8. | Myra Armstrong | 7 Mar 06 |
| 05 | Changes to header and footer to reflect new University logo and re- branding.  1. EDTA replacing heparinised blood  4.0 Update of Related Documents  5.1 Revision of consent procedure  5.3 Supplier change and media update  6.0 Revision of Release procedure | Ryan Jones |  |

1. **INTRODUCTION.**

This Standard Operating Procedure documents the procedures to follow for the collection, preparation and storage of the Prospective Study of Outcomes in Sporadic versus Hereditary Breast Cancer (POSH) blood samples.

The objective of this procedure is to prepare material suitable for future analysis.

These procedures describe; whole blood, serum extraction from clotted blood, the preparation of a cell suspension from EDTA blood by density gradient centrifugation, cryopreservation, storage and tracking methods for these materials.

The aim is to ensure an adequate and controlled protocol with appropriate storage for long term integrity until further analysis commences.

2. **SCOPE.**
   2.1 This procedure applies to any patient samples for which consent has

been sought for the POSH Studies as part of the Cancer Research-UK Clinical Trials within the Medical Oncology Department.

3. **RESPONSIBILITIES.**
   3.1 It is the responsibility of the Principal Investigator to ensure that staff of adequate experience and training perform the preparation outlined in this Standard Operating Procedure.
   3.2 It is the responsibility of the Tumour Bank Technicians to become familiarised with and follow the procedures outlined in this protocol and that all sample preparation is done in a safe and responsible manner.
   3.3 It is the responsibility of all staff members at all times to adhere to Data Protection with regards to patient confidentiality.
   3.4 It is the responsibility of all staff to ensure that any problems with sample collection, analysis, storage or documentation should be reported to the Unit Head immediately.
   3.5 It is the responsibility of all staff members to work safely and to be respectful of others at all times and to report anything they believe inappropriate or unsafe to their Section Head/Senior immediately.
4. **RELATED DOCUMENTS.**
   4.1 **DOCUMENTS.**

**Internal**

4.1.1 University of Southampton Cancer Sciences Division HTA licence 12009
4.1.2 CSD/SOP/101	Protocol for Release and Distribution of Bank Material
4.1.3 CSD/SOP/038	UoS CSD Policy & Procedures for Collection Storage and Distribution of Human Tissue
4.1.4 CSD/SOP/039	UoS CSD Policy & Procedures for ‘Future Use’ Human Tissue post HTA 2006
4.1.5 CSD/SOP/098	Research Ethics Related Procedures
4.1.6 CSD/SOP/330	Training of Staff Involved with Tissue Bank and Clinical Trial Endpoints
4.1.7 CSD/SOP/231	Data Protection Procedures
4.1.8 CSD/SOP/220	Written Informed Consent Procedures
4.1.9 CSD/SOP/102	SOP for Unlinking Anonymised Samples
4.1.10 CSD/SOP/400	SOP Safety Precautions for Handling Liquid Nitrogen
4.1.11 CSD/SOP/037	Adverse Events Relating to Tissue Stored under HTA Licence 12009
4.1.12 CSD/SOP/416	SOP for Safe Working Practices
4.1.13 CSD/SOP/300	Pipette Operation, Calibration, Preventative Maintenance and Servicing
4.1.14 CSD/SOP/301	Pipette Aids Operation and Preventative Maintenance
4.1.15 CSD/SOP/305	Microscope Operation, Preventative Maintenance and Servicing
4.1.16 CSD/SOP/306	SOP for Cell Counts with Trypan Blue and Improved Neubauer Haemocytometers
4.1.17 CSD/SOP/310	Class II Safety Cabinets Operation, Cleaning, Preventative Maintenance and Servicing
4.1.18 CSD/SOP/311	Bench Top Centrifuge Operation, Cleaning, Preventative Maintenance and Servicing
4.1.19 CSD/SOP/315	SOP for Liquid Nitrogen Tanks
4.1.20 CSD/FORMS/121	POSH Worksheet
4.1.21 CSD/SOP/125	POSH Database
4.1.22 CSD/FORMS/120 POSH Samples Weekly report

**External**

4.1.23 2004/23/EC European Union ‘Parent Directive’ on Setting Standards of

Quality and Safety for the Donation, Procurement, Testing and Processing Storage and Distribution of Human Tissue and Cells.

4.1.24 2006/86/EC European Union ‘Commission Directive’ implementing

Directive 2004/23/EC for the Traceability Requirements, Notification of Serious Adverse Reactions and Events and Certain Technical Requirements for the Coding, Processing, Preservation, Storage and Distribution of Human Tissue and Cells

4.1.25 Human Tissue Act 2004
4.1.26 2006/86/EC European Union ‘Commission Directive’ Implementing Technical Requirements of 2004/23/EC for Donation Procurement and Testing of Human Tissue and Cells.
4.1.27 Human Tissue Authority Codes of Practise – Consent
4.1.28 Human Tissue Authority Codes of Practise – Removal, Storage and Disposal of Human Organs and Tissue
4.1.29 HTA Directions given	under the Human Tissue Act 2004: (Check for updates–issued April and October yearly).

*Note many of above documents are written more specifically for transplant type activities; legislation covers research use and so many systems and requirements above may therefore be applicable*

4.1.30 British Medical Association (BMA) Human Tissue Legislation Guidance

from BMAs Ethics Department

4.1.31 2001/20/EC European Union ;Clinical Trials Directive’
4.1.32 The ICH Guidelines for Good Clinical Practise. Jan

1999/CPMP/ICH/135/95.

4.1.33 ICH Harmonised Tripartite Guidelines for Good Clinical Practice.

Jan 1999/CPMP/ICH/135/95.

.

4.2 **CONTACTS**
    4.2.1 POSH Administrator	x5171
    4.2.2 Principal Investigator	x5162
    4.2.3 Cancer Sciences reception	x6184
    4.2.4 SGH – Post room	x4125

5. **PROCEDURE.**
   5.1 **Consent for Tumour Banking.**
       5.1.1 Consent is obtained prior to sample collection via the POSH Administrator.
       5.1.2 Various Research Nurses from participating hospitals hold the consent form and will forward a photocopy along with the samples directly to the Tumour Bank.
       5.1.3 All consent forms received should be given to the POSH administrator. The administrator will log the necessary details and update the POSH Database.

5.2 **EQUIpment.**

| ITEM OF EQUIPMENT | SUPPLIER | CODE | LOCATION |
| --- | --- | --- | --- |
|  |  |  |  |
| SAFETY CABINET | KENDRO | 510191142 | CS/C19 |
| E200 MICROSCOPE | NIKON | 450-953 | CS/C19 |
| CENTRIFUGE | KENDRO | LEGEND RTE | CS/C19 |
| PIPETTE BOY | FISHER | PMR-100-010C | CS/C19 |
| HAEMOCYTOMETER | NALGENE | - | CS/C19 |
| ADJUSTABLE PIPETTES | GILSON | P1000, P200, P20 | CS/C19 |
| NALGENE FREEZING BOX | FISHER | CRY-120-010T | CS/C19 |
| -80°C FREEZER | BOROLABS | - | CS/B11 |
| LIQUID NITROGEN TANK | STATEBOURNE | BIOSYSTEM 20 | CS/B12 |
|  |  |  |  |

5.3 **REAGENTS / CHEMICALS**

| CHEMICAL-REAGENT | SUPPLIER | CODE | STORE | LOCATION |
| --- | --- | --- | --- | --- |
|  |  |  |  |  |
| RPMI 1640 without L-Glutamine | BIOWHITTAKER | BE12-167F | 4°C | CS/C17 |
| SODIUM PYRUVATE | BIOWHITTAKER | BE13-115E | -20°C | CS/C19 |
| L GLUTAMINE | BIOWHITTAKER | BE17-605E | -20°C | CS/C19 |
| FUNGIZONE | INVITROGEN | 15290-018 | -20°C | CS/C19 |
| DMSO | SIGMA | 472301 | RT | CS/C19 |
| HUMAN AB SERUM | SIGMA | H4522 | -20°C | CS/C19 |
| NON-ESSENTIAL AMINO ACIDS (x100) | BIOWHITTAKER | BE13-114E | 4°C | CS/C17 |
| FICOLL-PAQUE  TRIGENE | AMERSHAM  - | 11140-035  - | 4°C  RT | CS/C19  CS/C19 |
| TRYPAN BLUE | SIGMA | T8154 | RT | CS/C19 |
| PENICILLIN/ STREPTOMYCIN | BIOWHITTAKER | DE17-603E | -20°C | CS/C19 |
|  |  |  |  |  |

5.4 **APPARATUS**

| APPARATUS | QTY | SUPPLIER | CODE | LOCATION |
| --- | --- | --- | --- | --- |
|  |  |  |  |  |
| DISPOSABLE PIPETTE | 10ml x 2 | GREINER | 607180 | CS/C19 |
| DISPOSABLE PIPETTE | 5ml x 1 | GREINER | 606180 | CS/C19 |
| PETRI DISH | X1 | GREINER | 664160 | CS/C19 |
| TUBE | 15ml x 2 | GREINER | 227270 | CS/C19 |
| PIPETTE TIPS 1000µl | - | STARLABS | S1111-2721 | CS/C19 |
| PIPETTE TIPS 200µl | - | STARLABS | S1111-1706 | CS/C19 |
| PIPETTE TIPS 20µl | - | STARLABS | D1011-2413 | CS/C19 |
| TUBE | 50ml x 2 | GREINER | 227270 | CS/C19 |
| BEAKER | X1 | - | - | CS/C19 |
| PASTEUR PIPETTE | X1 | ALPHA LABS | LW4112 | CS/C19 |
| CRYOVIALS | 1.5ml | GREINER | 122263 | CS/C19 |
| MICRO CENTRIFUGE TUBE | 1.5ml x 1 | SARSTEDT | 72.690 | CS/C19 |
|  |  |  |  |  |

5.5 **REAGENT PREPARATION**

5.5.1	PREP MEDIUM – Take 500ml RPMI 1640 without L-Glutamine

Add 5ml L-Glutamine.

Add 6ml Penicillin & Streptomycin

Add 5ml Sodium Pyruvate

Add 5ml Non-Essential Amino Acids

Add 1ml Fungizone

Label bottle with ‘R’, date and initial and expiry date of 1 month from date of preparation. Store in prep room fridge located in room CS/C19.

5.5.2	FREEZING MEDIUM – To be made fresh before use.

By use of an adjustable pipette make up a solution of

Prep Media ‘R’	40%

Human AB Serum	50%

DMSO	10%

5.5.3	TRIGENE: 4% To be made fresh daily. Stored in room CS/C19.

5.6 **HAZARDOUS SUBSTANCE INFORMATION**
    5.6.1 Biological Hazard – All biological samples should be treated as a possible cause of infection, departmental and SUHT safety policy guidelines must be adhered to when handling human material.
    5.6.2 All staff must adhere to CSD/SOP/416 Safe Working Practices.
5.7 **SAMPLE COLLECTION**
    5.7.1 POSH samples may arrive at any time. A fax will be sent directly to the Cancer Sciences Department confirming a sample has been posted. This fax should be filed in the POSH Fax file located in CS/B9.
    5.7.2 Post Room – SGH will phone to inform Tumour Bank of sample arrivals over the weekend. Samples should be collected from the Post Room Reception, Level B, South Block, Pathology Monday morning and processed immediately.

5.7.3 Samples arriving on week days will be delivered to the Cancer Sciences Reception upon postal delivery at 10.30am and 2.30pm.The sample will arrive in a sealed bag containing a copy of the fax and consent forms.
5.7.4 Patient name, date of birth, hospital number, hospital name, date samples sent and date samples received should be noted on the CSD/FORMS/121 worksheet (see appendix I). The fax copy should now be shredded and a photocopy made of the consent form for the attention of the POSH Study Administrator.
5.7.5 Enter the patient information in to the POSH Database (CSD/SOP/125) where a sample number will be generated. This number must now be used for sample identification to protect patient anonymity.
5.7.6 Details of all samples processed should be detailed on CSD/FORMS/120 (see appendix II) POSH Samples Weekly Report. Details must include date sample was sent, received and processed together with notification of problem samples or request for repeat bloods. Fully completed forms should be given to the Study Administrator (see contacts), dated and initialled each week.
5.8 **METHOD**
    5.8.1 All users of this SOP must adhere to the SOP for Safe Working Practice CSD/SOP/416.
    5.8.2 All procedures must be performed in sterile conditions in a Class II Safety Cabinet according to CSD/SOP/310.
    5.8.3 Ensure all instruments needed are available and to hand.
    5.8.4 A clotted blood sample is required for serum and two EDTA bloods for whole blood and lymphocyte suspension. Any deviation from this should be recorded on Worksheet CSD/FORMS/121.
    5.8.5 If a sample received is of low volume or poor quality the POSH Study Administrator must be informed. When only one EDTA sample is received, whole blood (see 5.8.6) must take priority and the POSH Study Administrator informed - additional samples may then be requested.
    5.8.6 WHOLE BLOOD – at least 5ml EDTA should be labelled with the POSH number, date of birth and date of processing and stored in the next available POSH box located in -80°C freezer room CS/B11 making a note of the location on CSD/FORMS/121 worksheet.
    5.8.7 SERUM – Pour the clotted blood in to a 15ml tube and spin at 1500rpm for 10 minutes. During this time prepare the cells.
    5.8.8 CELLS – Transfer 9ml EDTA blood (or the volume of the second tube received) in to a 50ml tube and add an equal volume of prep medium. Mix well. Place 12ml Ficoll-paque in to a second clean 50ml tube and carefully/slowly overlay the diluted blood on to the surface of the Ficoll using a disposable pipette. Transfer to the centrifuge without disturbing the layers and spin immediately at 1500rpm for 20mins with the brake OFF. During this time complete the serum prep.
    5.8.9 SERUM – A Pasteur Pipette is used to draw off the supernatant (straw coloured) in to a clean 15ml tube discarding the clot. Spin again at 1500rpm for 10 minutes. Use an automated pipette to transfer 1ml serum in to BLUE cryovials clearly labelled with Banks generated number, sample type and date. Note numbers of vials prepared on worksheet CSD/FORMS/121 and enter details on to POSH Database.
    5.8.10 CELLS – Carefully remove sample tube from centrifuge without disturbing the layers and collect the interface (cloudy layer with clear layer below and straw layer above) in to a clean 50ml tube being careful not to disturb the Ficoll layer. A maximum of three pipettes should contain all the cells in this layer. Make up to 50ml with prep medium and spin at 1500 for 5 minutes with brake on 60%.
    5.8.11 Decant the supernatant in to a beaker of TriGene being careful not to dislodge the pellet of cells. Re-suspend the cells in 10ml prep media carefully using the pipette action to gently mix the cell suspension.
    5.8.12 Perform a cell count (see CSD/SOP/306) and enter the data on to CSD/FORMS/121 worksheet.
5.9 **SAMPLE STORAGE**
    5.9.1 All staff following this SOP must adhere to the CSD/SOP/315 SOP for Liquid Nitrogen Tanks and CSD/SOP/416 Safe Working Practice.
    5.9.2 Centrifuge the remaining cell suspension at 1500rpm for 5 minutes and aspirate off the supernatant being careful not to dislodge the pellet.
    5.9.3 Make fresh freezing medium (see Reagent Preparation 5.5.2). Cell degradation occurs rapidly upon contact with DMSO and so samples should be frozen immediately on addition of freezing mixture.
    5.9.4 Re-suspend the pellet of cells in 1ml freezing mixture and transfer to a BLUE cryovial labelled with POSH generated number, cell count and date.
    5.9.5 Transfer all vials (cells and serum) to a freeze controlled Nalgene box (Mr Frosty) which is designed to slow freeze the samples at a rate of 1°C per minute using Isopropanol which must be changed after every five uses to protect sample integrity. Place the box in to a -80°C freezer. A minimum of 4 hours (preferably overnight) should be allowed before transfer of samples to liquid Nitrogen storage tanks. Note storage locations on CSD/FORMS/121 POSH worksheet.
    5.9.6 Enter all information on to the POSH database including storage location, number of vials stored, cell concentration and viability, sample received date, sample processed date and sample stored date.
    5.9.7 Fully completed CSD/FORMS/121 worksheets should be filed in the POSH File located in CS/B9.

6.0 **RELEASE OF STORED MATERIAL**
    6.1 Follow SOP/CSD/101 for the release of material stored within the Tissue Bank. A summary of this procedure is described below.

*Researchers who wish to access samples from the tumour bank should liaise with the Tumour Bank Steering Group, who will provide details of stored materials and those materials available for use. The Steering Committee will notify the Clinician responsible for storing the tumour samples. A full proposal will be required for submission to the Cancer Protocol Review Committee and to the POSH Steering Group via the Principal Investigator. If approved a further subsequent submission to the National Research Ethics Services (NRES) will take place.*

*Once the relevant approval has been obtained, access to tumour samples will be through the Tissue Bank Manager (PD). A copy of the ethics approval must be provided to the PD prior to release of samples.*

*The PI of the study must sign departmental policy documents CSD/SOP/038 and CSD/SOP/039 prior to sample release (this needs to be done only once).*

*On collection of the samples, the researcher receiving tissue as agreed with PI above, must sign copies of CSD/FORMS/152 Sample Release Compliance Form to acknowledge receipt of the samples, change of ownership and their obligations under the Human Tissue Act 2004. One copy is to be held within the bank and one copy provided to the researcher for their own records (no patient identifiable information will be disclosed on this form). The Tissue Bank Manager (or approved Senior Bank staff in Managers absence) will arrange for samples to be located and removed from storage as and when required.*

*Full details of sample removal must be logged as per CSD/SOP/101. All Ethics copies/Paperwork must be stored as per CSD/SOP/101. All sample release must be logged on reverse side of work sheets or on historical release sheet as appropriate CSD/FORMS/125 and the database amended accordingly.*

6.2 No samples will be released without full and proper authorisation.

7.0 **APPENDED**

Appendix I CSD/FORMS/121	POSH Worksheet

Appendix II CSD/FORMS/120 POSH Samples Weekly Report

**8.0	LIST OF CURRENT USERS and TRAINING.**

Level of training required for this update.

√

Read. Demonstrate/Briefing. Full Training

The Following people have been trained /read and understood this SOP and are currently using these procedures in the laboratory:

**Name**	**Signature**	**Date**
